# Identification of Genetic Loci Associated With Crude Protein Content and Fiber Composition in Alfalfa (*Medicago sativa* L.) Using QTL Mapping

**DOI:** 10.3389/fpls.2021.608940

**Published:** 2021-02-18

**Authors:** Changfu Yang, Fan Zhang, Xueqian Jiang, Xijiang Yang, Fei He, Zhen Wang, Ruicai Long, Lin Chen, Tianhui Yang, Chuan Wang, Ting Gao, Junmei Kang, Qingchuan Yang

**Affiliations:** ^1^Institute of Animal Science, Chinese Academy of Agricultural Sciences, Beijing, China; ^2^Institute of Animal Science, Ningxia Academy of Agricultural and Forestry Sciences, Yinchuan, China

**Keywords:** crude protein, fiber composition, quality, QTL mapping, *Medicago sativa* L.

## Abstract

Forage quality determined mainly by protein content and fiber composition has a crucial influence on digestibility and nutrition intake for animal feeding. To explore the genetic basis of quality traits, we conducted QTL mapping based on the phenotypic data of crude protein (CP), neutral detergent fiber (NDF), acid detergent fiber (ADF), and lignin of an F_1_ alfalfa population generated by crossing of two alfalfa parents with significant difference in quality. In total, 83 QTLs were identified with contribution to the phenotypic variation (PVE) ranging from 1.45 to 14.35%. Among them, 47 QTLs interacted significantly with environment and 12 QTLs were associated with more than one trait. Epistatic effect was also detected for 73 pairs of QTLs with PVE of 1.08–14.06%. The results suggested that the inheritance of quality-related traits was jointly affected by additive, epistasis and environment. In addition, 83.33% of the co-localized QTLs were shared by ADF and NDF with the same genetic direction, while the additive effect of crude protein-associated QTLs was opposite to that fiber composition on the same locus, suggesting that the loci may antagonistically contribute to protein content and fiber composition. Further analysis of a QTL related to all the three traits of fiber composition (*qNDF1C, qADF1C-2*, and *qlignin1C-2*) showed that five candidate genes were homologs of cellulose synthase-like protein A1 in *Medicago truncatula*, indicating the potential role in fiber synthesis. For the protein-associated loci we identified, *qCP4C-1* was located in the shortest region (chr 4.3 39.3–39.4 Mb), and two of the seven corresponding genes in this region were predicted to be E3 ubiquitin-protein ligase in protein metabolism. Therefore, our results provide some reliable regions significantly associated with alfalfa quality, and identification of the key genes would facilitate marker-assisted selection for favorable alleles in breeding program of alfalfa quality improvement.

## Introduction

Crude protein and fiber components are two key indicators of forage quality, which affects digestibility and nutrition intake for animal feeding. Perennial legume alfalfa (*Medicago sativa* L.) has become an important forage in the diet of ruminants due to its high protein content (15–20%), as well as vitamins and minerals (Annicchiarico et al., 2015). Improvement of alfalfa’s protein content lowers the cost of raising of ruminants. Generally, fiber component of alfalfa is consisted of neutral detergent fiber (NDF), acid detergent fiber (ADF), and lignin. Among them, NDF is negatively correlated with feed intake of animals (Oba and Allen, 1999), and high content of ADF or lignin lowers digestibility (Sarwar et al., 1999). Therefore, breeding alfalfa new varieties with improved quality has been a major focus of the forage breeders worldwide.

Forage quality is affected by both genetic and environmental factors (Griffin et al., 1994; Palmonari et al., 2014; Liu et al., 2018; Yang et al., 2019). A lot of progress has been made in improving alfalfa quality by genetic manipulation and optimization of cultivation strategy. For example, protein content in alfalfa was increased by overexpressing of glutamate synthase or glutamine synthase, two enzymes involving in ammonium assimilation (Vance et al., 1995; Kaur et al., 2019). Reduction of lignin content was achieved by down-regulating lignin synthesis genes such as, hydroxycinnamoyl -CoA:shikimate hydroxycinnamoyl transferase (*HCT*), coumarate 3-hydroxylase (*C3H*), cinnamyl alcohol dehydrogenase (*CAD*), caffeic acid 3-*O*-methyltransferase (*COMT*), and caffeoyl CoA 3-*O*-methyltransferase (*CCoAOMT*) (Baucher et al., 1999; Marita et al., 2003; Nakashima et al., 2008; Tong et al., 2015; Gallego-Giraldo et al., 2016). In addition, suitable field management of seasonal fertilization and irrigation, is beneficial for alfalfa growth and enables a better balance of protein and fiber content (Bacenetti et al., 2018).

Since the proposal of QTL interval mapping (IM) in 1989 (Lander and Botstein, 1989), QTL mapping has become a focus of quantitative genetics research. There have been a number of QTL studies in other crops, such as rice and soybean, for these quality-related traits (Zhang et al., 2008, 2018; Warrington et al., 2015; Bazrkarkhatibani et al., 2019). For example, 47 QTLs related to fiber were identified in a corn RIL population (Li et al., 2017), and 14 QTLs related to grain protein content were mapped in a rice BC_3_F_4_ population, and gluten family genes were identified from *qGPC1.1* (Chattopadhyay et al., 2019).

QTL research in alfalfa is rather slow primarily due to its nature of heterozygosity and autotetraploidy which hampers the construction of genetic maps. It has been documented that the linkage distance between molecular markers could be estimated using single dose alleles (SDA) with a separation ratio of 1:1 in alfalfa (Brouwer and Osborn, 1999). Based on SDA, using this method, a series of alfalfa genetic maps containing eight chromosomes have been constructed (Sledge et al., 2005). In 2014, a saturated genetic linkage map was developed using 3,591 single nucleotide polymorphism (SNP) markers of alfalfa (Li et al., 2014). Some of the linkage maps have been applied to analyze the agriculturally important traits especially yield, fall dormancy and stress tolerance to advert environmental conditions (Ray et al., 2015; Adhikari et al., 2018; Zhang et al., 2019). Used the method described by Zhang et al. (2020), we previously also constructed an alfalfa linkage map containing 3,818 SNP markers obtained by genotyping-by-sequencing (GBS) of an F_1_ population of about 392 individual plants. To identified the key QTLs or candidate genes associated with alfalfa quality, here we conducted QTL mapping based on our 3-year data of crude protein content and fiber composition of the F_1_ population. Our results provide useful information on reliable candidate locus for alfalfa quality improvement.

## Materials and Methods

### Plant Materials and Growth Conditions

Alfalfa materials used in this study were an F_1_ population of 392 individuals generated by crossing of a landrace “Cangzhou” (the paternal parent) with Zhongmu No.1 (the maternal parent) as described previously (Zhang et al., 2020).

The field trials were conducted at the research base of the Chinese Academy of Agricultural Sciences in Langfang, Hebei province. The average temperature annually is 11.9°C, the average temperature in the coldest month (January) is −4.7°C, and in the hottest month (July) is 26.2°C. The annual precipitation is 554.9 mm. The soil is medium loam with 1.69% organic matter and a pH of 7.37.

### Phenotyping

Seedlings including F_1_ population and the parental plants were cultured in greenhouse under conditions of 16 h day/8 h night, 22°C, and 40% relative humidity. Clones were generated *via* stem cuttings and transplanted into field in early April of 2014 with three clones as replicates randomly planted in three adjacent blocks. Row spacing is 100 cm and column spacing 80 cm. Plants were clipped late fall with the ground retention height of 5 cm. No application of fertilizer or irrigation was carried out for the field.

The first cut of 2016, 2019, and 2020 with a height of ground stubble at 5 cm was used for phenotypic data collection. Samples were oven-dried (60°C, 24 h), and ground to pass through a 1-mm sieve. The content of crude protein, NDF, ADF, and lignin was measured using near-infrared reflectance spectroscopy (Foss NIRS 1650) with an analytical model corrected by chemically measured data of 20 alfalfa samples. SAS 9.4 was performed to analyze the mean comparison of the parental phenotypic data and the normal distribution test (variation range, mean, variance, skewness, and kurtosis) and correlation analysis for the crude protein, NDF, ADF, and lignin of F_1_ population. The frequency distribution of the F_1_ population phenotypic data was plotted using SPSS 18.0 (Baarda and van Dijkum, 2019). The broad sense heritability (*H*^2^) of each trait was calculated using the ANOVA function of the software QTL-IciMapping (Meng et al., 2015).

### QTL Analysis

Raw data of Genotyping-by-Sequencing (GBS) were submitted to the NCBI Sequence Read Archive with bioproject ID: PRJNA522887^1^.

The genetic linkage map was constructed as has been described by our previous study (Zhang et al., 2020). Since our population is a pseudo-testcross F_1_ population, we constructed linkage map separately in maternal and paternal parents (Grattapaglia, 1997).

For environmental effect, best linear unbiased prediction (BLUP) was calculated by ANOVA model of IciMapping (ICIM) (Malosetti et al., 2004; Messmer et al., 2009). Additive QTLs were detected using Biparental Populations (BIP) function of ICIM and mapped by ICIM-ADD with Likehood of odd (LOD) threshold of 2.5 for significance. The additive QTL result was graphically represented using MapChart (Voorrips, 2002). Additive QTLs interacting with environment were identified by MET (QTL by Environment Interactions for Multi-Environment Trials) model of ICIM, the threshold was set to 2.5. For epistatic QTLs, BIP-ICIM-EPI model was used, the LOD threshold was set to 5.0 (Li et al., 2007; Meng et al., 2015).

## Results

### Analysis of the Phenotypic Variations

**FIGURE 1:**
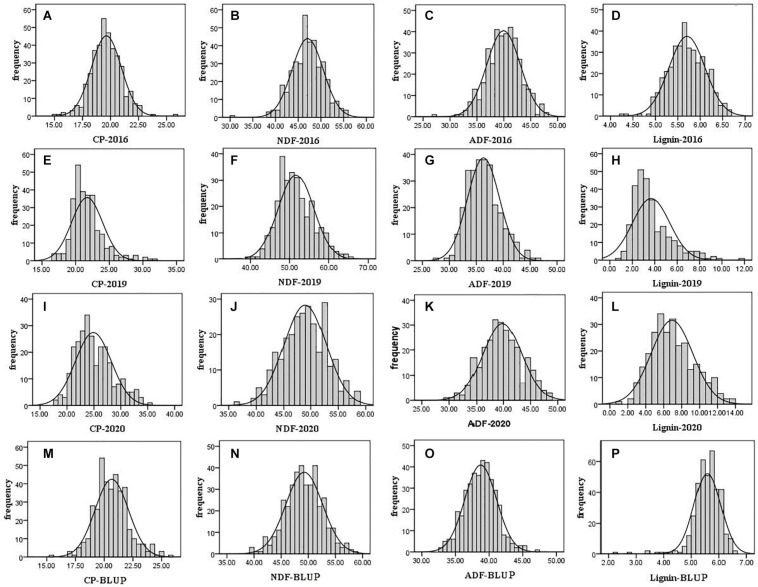
Frequency distribution histogram of F_1_ population quality traits in 2016, 2019, and 2020. The black solid line is the normal distribution curve. The traits are include the following: **(A)** CP in 2016; **(B)** NDF in 2016; **(C)** ADF in 2016; **(D)** lignin in 2016; **(E)** CP in 2019; **(F)** NDF in 2019; **(G)** ADF in 2019; **(H)** lignin in 2019; **(I)** CP in 2020; **(J)** NDF in 2020; **(K)** ADF in 2020; **(L)** lignin in 2020; **(M)** CP of BLUP; **(N)** NDF of BLUP; **(O)** ADF of BLUP; and **(P)** lignin of BLUP.

For evaluation of alfalfa quality, phenotypic data including crude protein and fiber composition of F_1_ population and its two parental plants were measured in 2016, 2019, and 2020. According to the t-test, the crude protein content of the parental parent was significantly higher than that of the maternal parent (21.19 vs. 18.83%; 22.32 vs. 19.27%) in 2016 and 2019 (*P* < 0.01). In contrast, the content of NDF, ADF, and lignin of the paternal parent was significantly lower than that in the maternal parent tested in the 3 years (*P* < 0.01) except lignin in 2020 (Supplementary Table 1). The results demonstrated that the paternal parent was superior to the maternal one in quality. A frequency distribution histogram based on the F_1_ population quality data showed transgressive segregation in the F_1_ population with a continuous distribution (Figure 1), indicating that the quality indexes used here were quantitative traits. The phenotypic variation we observed is consistent with the previous reports (Julier et al., 2000; Wang et al., 2016; Biazzi et al., 2017). The variation over years suggested that these traits are affected by the environment.

### Analysis of Correlation Coefficiency and the Broad-Sense Heritability in F_1_ Population

The correlation coefficient was calculated using the BLUP value. As shown in Table 1, the three indexes for fiber composition were positively correlated and the correlation coefficient ranged from 0.40–0.68 (*P* < 0.01) with the highest between NDF and ADF. Crude protein was negatively correlated with both ADF (−0.49) and Lignin (−0.17) for *P* < 0.01, but not with NDF (*P* > 0.05). The calculation of the broad-sense heritability showed a relatively higher value for NDF (0.56) and lower one for lignin (0.46) (Table 1).

**TABLE 1:** Correlation coefficiency and the broad-sense heritability in the F_1_ population.

**Traits**	**CP**	**NDF**	**ADF**	**Lignin**	***H*^2^**
CP	1				0.55
NDF	−0.05^*ns*^	1			0.56
ADF	−0.49**	0.68**	1		0.55
Lignin	−0.17**	0.40**	0.60**	1	0.46

**means a significant difference for *P* < 0.01 and ^*ns*^means *p* > 0.05; *H*^2^ means the broad-sense heritability.

### Mappping of QTLs Associated With Crude Protein and Fiber Composition

Based on the data of crude protein and fiber composition of the F_1_ population and its parental plants collected in 2016, 2019, and 2020, QTL mapping was performed using the alfalfa linkage map we previously constructed (Zhang et al., 2020). In total, we detected 83 QTLs related to either protein content or fiber composition with 31, 15, and 17 loci from the year of 2016, 2019, and 2020, respectively, and the rest were detected using BLUP values.

For crude protein, a total of 23 QTLs were mapped including seven QTLs identified using BLUP values (Table 2 and Supplementary Table 2). The LOD values ranged from 2.62 to 10.24, the percentage of phenotypic variation explained by QTL (PVE) was 1.45–11.34%, and the additive effect value of −0.87 to 1.32. QTL *qCP7D*, had the highest LOD value (Supplementary Table 2), and QTL located on the chromosome 5B (*qCP5B*) had the highest PVE and additive effect values (Table 2).

**TABLE 2:** Maternal QTL mapping results.

**Year**	**QTL**	**Linkage group**	**Position/cM**	**Left marker**	**Right marker**	**LOD**	**A**	**PVE (A)/%**	**AE**	**PVE (AE)/%**
2016	*qCP4A*	4A	15.5–17.5	TP8410	TP2329	2.69	−0.25	3.00		
	*qCP5A-2	5A	11.5–12.5	TP22206	TP63158	3.28	0.27	2.61	0.12	0.45
	***qCP6C-1***	6C	11.5–12.5	TP41126	TP1505	4.56	0.34	4.13		
	*qCP8C*	8C	33.5–35.5	TP33947	TP71109	2.68	−0.24	2.76		
	*qNDF4B-2*	4B	88.5–90.5	TP46160	TP70472	2.50	0.58	2.18	0.22	0.56
	***qNDF5C***	5C	93.5–96.5	TP94547	TP63743	4.04	0.76	3.49	0.21	0.40
	***qNDF6C***	6C	11.5–12.5	TP41126	TP1505	4.53	−0.81	3.99	−0.55	3.22
	***qNDF6D***	6D	23.5–25.5	TP72418	TP48491	6.28	−0.91	5.00	−0.45	1.75
	***qNDF8C***	8C	81.5–83.5	TP66658	TP40849	2.73	0.60	2.35	0.17	2.07
	***qNDF8D***	8D	73.5–75.5	TP94054	TP60447	5.24	−0.93	5.30	−0.42	1.68
	***qADF6C***	6C	11.5–12.5	TP41126	TP1505	3.37	−0.64	3.65	−0.37	1.65
	***qADF6D***	6D	23.5–25.5	TP72418	TP48491	6.67	−0.87	6.68		
	*qADF7A*	7A	117.5–122.5	TP2379	TP26898	2.58	0.52	2.54		
	***qADF8C***	8C	81.5–83.5	TP66658	TP40849	2.84	0.57	2.92	0.39	1.82
	***qADF8D***	8D	73.5–75.5	TP94054	TP60447	2.56	−0.60	3.37	−0.35	1.52
	*qlignin4A*	4A	31.5–35.5	TP95797	TP49781	3.29	0.08	3.74	0.05	0.45
	***qlignin6D-2***	6D	23.5–25.5	TP72418	TP48491	11.75	−0.15	14.35	−0.14	2.04
2019	*qCP2C*	2C	30.5–32.5	TP91889	TP65244	3.91	0.60	4.43	0.26	1.45
	*qCP3C*	3C	60.5–65.5	TP16675	TP25696	3.10	−0.57	4.10	−0.16	0.68
	***qNDF1C***	1C	86.5–87.5	TP5293	TP97853	2.87	1.02	4.16	0.37	1.37
	*qNDF2D*	2D	0–5.5	TP23158	TP59255	2.65	−1.09	4.99	−0.46	1.92
	*qNDF8B	8B	124.5–127.5	TP6047	TP57365	4.32	−1.20	5.94	−0.48	2.07
	***qADF1C-1***	1C	86.5–87.5	TP5293	TP97853	2.61	0.65	4.12	0.25	0.73
	*qADF8B	8B	124.5–128.5	TP6047	TP57365	3.18	−0.70	4.06	−0.18	0.39
	*qlignin1D*	1D	48.5–51.5	TP24124	TP8248	2.67	−0.35	4.80	0.10	2.87
2020	*qCP4C-1*	4C	92.5–95.5	TP32398	TP76698	4.15	0.85	4.41	0.41	3.84
	*qCP5B*	5B	129.5–130.5	TP58284	TP75694	8.16	1.32	11.34	0.40	3.48
	*qCP5D*	5D	17.5–20.5	TP37244	TP743	2.77	−0.69	3.14	−0.30	2.23
	*qCP6A-2*	6A	145.5–147.5	TP66649	TP95828	2.62	−0.80	4.21	−0.28	1.93
	*qCP6C-3*	6C	41.5–44.5	TP29297	TP13076	4.31	−0.88	4.87	−0.04	1.00
	*qNDF3C*	3C	103.5–105.5	TP40480	TP52516	4.53	−1.25	6.27	0.27	1.02
	*qNDF4B-1*	4B	43.5–45.5	TP100066	TP34030	2.73	0.94	3.78		
	***qNDF5C***	5C	74.5–75.5	TP56843	TP45939	4.38	1.12	5.08		
	*qADF1C-2*	1C	123.5–124.5	TP74914	TP95255	2.96	0.99	5.12	0.28	1.02
	*qADF5B*	5B	107.5–113.5	TP71024	TP46218	2.52	−0.85	3.45	−0.02	1.84
	*qlignin4C*	4C	138.5–140.5	TP15413	TP8148	3.80	0.59	4.78	0.25	5.29
	*qlignin5B*	5B	119.5–122.5	TP62644	TP78353	2.90	0.63	4.78	0.21	3.52
	*qlignin6A*	6A	105.5–106.5	TP27849	TP8131	2.71	−0.51	3.41		
	*qlignin6C*	6C	36.5–38.5	TP97699	TP20847	5.34	−0.72	6.97		
BLUP	*qCP2B*	2B	115.5–117.5	TP44590	TP14817	6.45	−0.39	4.11		
	*qCP4C-2*	4C	111.5–114.5	TP10439	TP87009	4.06	0.30	2.47		
	*qCP5A-1*	5A	0–1.5	TP13068	TP27740	4.85	0.34	3.14		
	*qCP6A-1*	6A	6.5–7.5	TP97202	TP72286	5.16	−0.35	3.48		
	*qCP6C-2*	6C	30.5–33.5	TP24704	TP8104	7.08	−0.40	4.38		
	*qNDF3A*	3A	5.5–6.5	TP86255	TP29176	4.68	−0.80	4.38		
	*qNDF4D*	4D	77.5–79.5	TP52612	TP13026	3.45	0.69	3.43		
	***qNDF5C***	5C	86.5–88.5	TP49480	TP8930	4.38	0.70	3.54		
	***qNDF6D***	6D	24.5–25.5	TP72418	TP48491	6.38	−0.87	5.37		
	*qNDF8B	8B	124.5–127.5	TP65937	TP6047	6.77	−0.92	5.99		
	***qADF1C***	1C	86.5–87.5	TP5293	TP97853	4.64	0.58	5.93		
	***qADF5C***	5C	93.5–95.5	TP94547	TP63743	2.78	0.46	3.76		
	*qADF8B	8B	123.5–128.5	TP6047	TP57365	2.57	−0.41	3.08		
	***qlignin5C***	5C	93.5–95.5	TP25891	TP94547	3.76	0.12	4.22		
	*qlignin6D*	6D	8.5–10.5	TP74220	TP71911	5.26	−0.15	6.24		

QTLs were bold to indicates that this QTL co-located with others. *QTL means it had been identified previously, *qCP5A-2* (Biazzi et al., 2017); *qNDF8B* and *qADF8B* (Sakiroglu and Brummer, 2017). A, additive effects; PVE(A), percentage of phenotypic variation explained by the QTL at the current position; AE, the additive QTL × environment interaction under current year; PVE(AE), percentage of phenotype variance explained by additive QTL × environment.

QTLs associated with fiber composition were separately identified based on our evaluation of NDF, ADF, and lignin content. For NDF-related QTLs, the 29 loci with 12 mapped in the paternal and 17 in the maternal parent were located on 18 linkage groups with PVE varying from 2.18–6.27% (Table 2 and Supplementary Table 2). The number of QTLs we discovered varied in the test years with a double amount mapped in 2016 compared with the 2019 and 2020. Additive effect of half the loci (14 out of 29) was positive and the other half (15 out of 29) negative, suggesting they were inherited from the maternal and paternal parent, respectively.

For QTLs associated with ADF, in total, 18 QTLs locating on 12 different linkage groups were discovered to be associated with ADF (Table 2 and Supplementary Table 2). In 2016, 8 QTLs were mapped, and additive effect of five loci was negative with PVE of 4.06 and 4.12%. Using, BLUP values of ADF, four QTLs were detected with three from the maternal parent.

For lignin-associated QTLs, a total of 13 QTLs were discovered on 10 linkage groups (Table 2 and Supplementary Table 2). Their LOD values ranged from 2.55 to 11.75. PVE of 3.01–14.35%, and additive effect of −0.72 to 0.63. QTL *qlignin6D-2* had the highest LOD and PVE of 11.75 and 14.35, respectively. QTL *qlignin5B* locating on chromosome 5B had the highest additive effect.

### QTLs Associated With Multiple Traits

**FIGURE 2:**
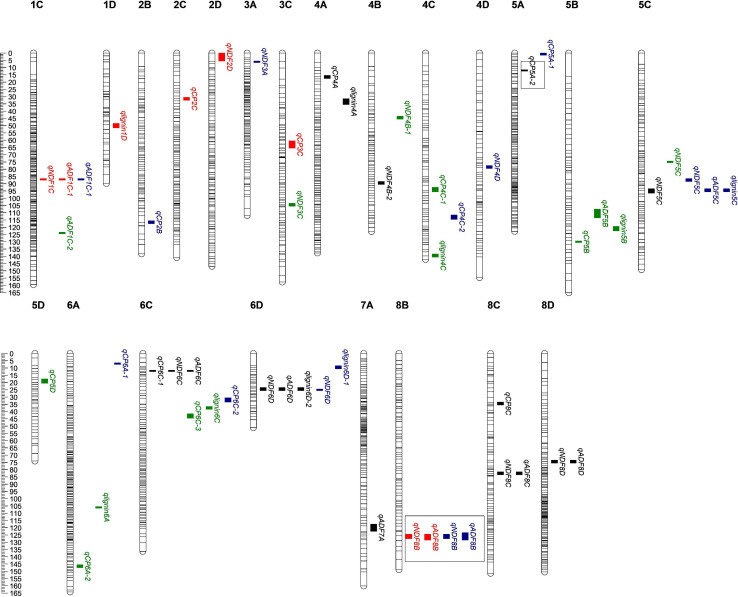
Quality-related QTL across the 32 linkage groups from a genetic map of maternal parent. The ruler on the left side represents the relative position of SNP on the chromosome. The small colored blocks represent QTL intervals. Black, QTLs in 2016; red, QTLs in 2019; green, QTLs in 2020; BLUP, QTLs of BLUP. The QTL in the box means it has been reported, *qCP5A-2* (Biazzi et al., 2017); *qNDF8B* and *qADF8B* (Sakiroglu and Brummer, 2017).

Our results showed that 83 QTLs were detected to be associated with the content of protein or fiber in alfalfa. Among them, 12 QTLs were found to be associated with more than one trait, and seven loci were distributed on the maternal linkage map and five on the paternal one (Figure 2 and Supplementary Figure 1). QTL *qlignin6D-2*, which was associated with both lignin and crude protein, has the biggest LOD and PVE (Table 2), suggesting this QTL may contain key genes that can significantly affect lignin content.

83.33% (10 out of 12) of the QTLs were co-localized by ADF and NDF (Figure 2 and Supplementary Figure 1), indicating that both indexes contribute to fiber connect in alfalfa. Among them, three loci were identified to be associated with ADF, NDF, and lignin simultaneously, suggesting the higher reliability of these loci as candidates for further investigation of alfalfa fiber composition. The rest two co-localized QTLs were related to crude protein and NDF (Figure 2 and Supplementary Figure 1). Interestingly, the values of addition effect for individual QTL co-localized by fiber composition were unanimously negative or positive, suggesting that thy shared the same genetic direction. In contrast, the additive effect for QTLs associated with both crude protein and fiber composition were opposite, suggesting that the loci may antagonistically contribute to the two traits.

### Analysis of Additive QTLs Interacted With the Environment

We also analyzed effect of environment by measuring the interaction effects. Among the 83 quality related QTLs, 47 were identified as interacting with the environment (Table 2 and Supplementary Table 2). The additive QTL × environment interaction effect (AE) of 24 loci was positive (0.05 ∼ 0.49), suggesting an increase of the phenotypic value of the corresponding trait, and the rest 23 were negative (−0.02 ∼ −0.55). PVE of AE was ranged from 0.10 ∼ 3.84%, suggesting these interaction effects have less effect on the phenotype.

### Analysis of Epistatic QTLs

**FIGURE 3:**
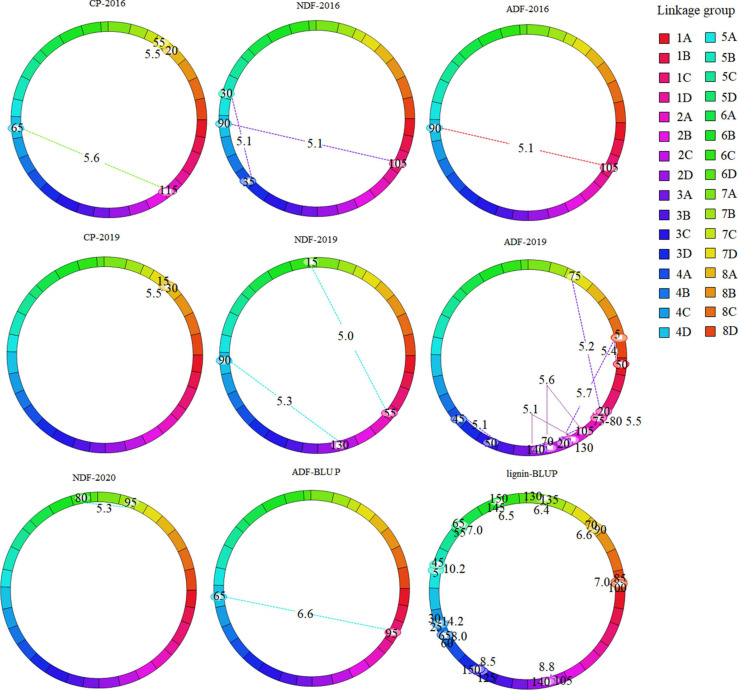
Cyclic diagram of epistatic QTLs for quality-related traits of the maternal parent. The dotted lines indicate the interacting SNP marker pairs mapped on the same or different linkage group with corresponding LOD scores owing to their epistatic effects. Marker position (cM) is mentioned inside the oval located on linkage group.

To explore the interaction between QTLs, we analyzed epistasis effect. In total, 73 epistatic QTL pairs (epQTLs) were detected with PVE ranging from 1.08 to 14.06%, and 31 pairs were identified using the BLUP values. No additive QTLs were found to be affected by other QTLs. 73 epQTLs all independently affected the phenotype, and these loci were distributed on 29 linkage groups except 2B, 3B, and 5A (Supplementary Tables 3, 4, Figure 3, and Supplementary Figure 2). Eleven pairs were associated with crude protein, eight with NDF, 17 with ADF, and 37 with lignin, respectively. A total of 25 epQTLs showed positive effects (0.12 ∼ 1.62), indicated that they could increase the phenotypic value independently from additive QTL, and 37 pairs showed negative effect (−0.11 ∼ −3.06), showed that they could reduce the phenotypic value independently. ep-QTL (*eqNDF1C*-*eqNDF5B*) associated with NDF had the highest PVE of 11.18%. In addition, 43 pairs of epistatic QTLs were identified at similar positions on the same chromosome, suggesting that most quality-related gene regulatory factors exist nearby. Moreover, we also identified the co-localized regions of two epistatic QTLs for different traits, which showed these loci may contribute to the two traits. The co-localized regions: (1) The region between markers TP6403 and TP71156 and the region between markers TP71156 and TP57346 on chromosome 1 (linkage group 1C) were associated with crude protein and lignin. (2) The region (TP49934-TP25688) on chromosome 1 (linkage group 1C) and the region (TP19263-TP27443) on chromosome 4 (linkage group 4D) were associated with NDF and ADF.

### Analysis of Potential Candidate Genes

To search candidate genes, sequences of the markers flanking our additive QTLs were used to perform BLAST against the *Medicago truncatula* genome. Because of the genetic difference between *Medicago truncatula* and *Medicago sativa*, limited markers were matched. Among them, the region of *qNDF1C*, which co-localized with *qADF1C* and *qlignin1C*, contains eight related genes, five annotated as cellulose synthase-like protein A1 (Table 3). Consistently, a NDF-associated SNP was detected on this locus by Li et al. (2011). Genetic manipulation of the candidate genes would help to elucidate their role in alfalfa quality formation.

**TABLE 3:** Annotated genes associated with related traits in the detected region.

**Reference genome**	**QTL**	**Gene**	**Description**
*Medicago truncatula* Mt4.0v1	*qNDF1C*	Medtr1g061270	Cellulose synthase-like protein A1
		Medtr1g061280	Cellulose synthase-like protein A1
		Medtr1g061340	Cellulose synthase-like protein A1
		Medtr1g061510	Cellulose synthase-like protein A1
		Medtr1g061740	UDP-glycosyltransferase, putative
		Medtr1g062380	UDP-glucosyltransferase family protein
		Medtr1g067530	UDP-glucuronic acid decarboxylase
		Medtr1g069605	Cellulose synthase-like protein
*Medicago sativa*	*qCP4C-1*	MS.gene125630.t1	*M. truncatula* DNA sequence from clone MTH2-21G21
		MS.gene004006.t1	PREDICTED: *Medicago truncatula* uncharacterized
		MS.gene004008.t1	PREDICTED: *Medicago truncatula* E3 ubiquitin-protein ligase SDIR1
		MS.gene004009.t1	PREDICTED: *Medicago truncatula* E3 ubiquitin-protein ligase RDUF2
		MS.gene004011.t1	PREDICTED: *Medicago truncatula* laccase-2
		MS.gene004005.t1	PREDICTED: *Medicago truncatula* pumilio homolog 2
		MS.gene004007.t1	*Medicago truncatula* chromosome 8 clone mth2-101n14

We also referenced the alfalfa genome (Chen et al., 2020) using TBtools to do BLAST. Six QTLs were matched on six chromosomal regions and the corresponding genes within these regions were listed (Supplementary Tables 5–10). The narrowest region (qCP4C-1) encoded seven genes, and two were predicted to be E3 ubiquitin-protein ligase SDIR1 and E3 ubiquitin-protein ligase RDUF2 (Table 3), which have been proven to be closely related to the degradation of plant proteins (Holdsworth et al., 2020). More effort are needed to align the QTLs in future.

## Discussion

Alfalfa (*Medicago sativa* L.) known as the “queen of forage” has been cultivated worldwide due to its high nutritional quality as animal feed, and become the fourth most valuable field crop in the United States. Alfalfa quality determined mainly by protein content and fiber composition affects the digestibility and nutrition intake for animal feeding. Improving alfalfa quality by increasing crude protein or reducing fiber content has benefited animal husbandry (Koçer et al., 2018). Using QTL mapping combined with high throughput genotyping technology, we identified 83 regions associated with crude protein, fiber or lignin based on the phenotypic data of an F_1_ population established by crossing two alfalfa parents with significant difference in quality.

It has been documented that analysis of multiple effects including additive effect, epistatic effect, and QTL × environment interaction effect helps to avoid an underestimation of the total genetic impact of a trait (Patil et al., 2013; Chattopadhyay and Behera, 2019). We found that 60.26% (47 out of 83) of additive QTLs interacted with the environment, and 73 pairs regions had epistatic effect, which suggested the complexity of alfalfa quality traits. This phenomenon was also found in the QTL identification of rice grain protein content (Chattopadhyay et al., 2019). Future research of different mapping groups with phenotypic data from multiple temporal and spatial collection would be effective.

Previous study revealed that the QTLs of NDF, ADF, and lignin in alfalfa have co-localizations on chromosomes 1 and 3 (Wang et al., 2016). We identified 12 QTLs associated with multiple traits and they distribute on chromosomes 1, 4, 5, 6, 7, and 8. The poor continuity of QTL mapping has been reported in crop yield studies of rice (Agrama et al., 2007; Zhang et al., 2014), probably due to the difference of populations used for the studies, as well as the number of markers available. Colocalization and pleiotropic associations have been reported to help reveal important genomic regions or genes associated with the target traits (Huang et al., 2015). Although the 12 co-localized regions scattered on six linage groups, the traits for fiber composition, including ADF, NDF, and lignin, were found unanimously sharing either negative or positive values of addition effect, indicating the similar genetic contribution of the loci to these traits. In contrast, the two QTLs co-localized by crude protein and NDF/ADF had the opposite additive effect. For example, the additive effect of a QTL associated with crude protein, NDF, and ADF in linkage group 6C, was 0.3391, −0.8079, and −0.6446, respectively, implying that the QTL antagonistically contributes to protein content and fiber composition. The findings are consistent with the correlation analysis between these traits. Supportively, down-regulation of lignin synthesis gene hydroxycinnamoyl CoA: shikimate hydroxycinnamoyl transferase in transgenic alfalfa could reduce NDF and ADF (Shadle et al., 2007). The colocalized QTLs may be applied in improvement of multiple traits simultaneously.

Four of the 83 QTLs we identified here have been documented to be associated with alfalfa quality (Li et al., 2011; Espinoza and Julier, 2013; Biazzi et al., 2017; Sakiroglu and Brummer, 2017). For example, A SNP associated with NDF in Li’s report shared the same region with qNDF1C, and the QTL was co-localized by qADF1C-2 and qlignin1C-2. The shortest region of a QTL related to protein content has two E3 ubiquitin-protein ligases, which mediate the polyubiquitination of lysine and cysteine residues on target proteins, and have been proven to play an important role as regulators of protein trafficking and degradation. These findings are in supportive of our association analysis of alfalfa quality QTLs, and the loci are worthy of further investigation. Narrow down of the regions covered by our QTLs would specify more candidates potentially contributing to alfalfa quality formation. An increase of sequencing coverage, enrichment of markers and application of higher density linkage maps would facilitate marker-assisted selection for favorable alleles in breeding alfalfa varieties with improved quality.

## Conclusion

The identified QTLs associated with quality-related traits provide important information for understanding the genetic controls of alfalfa quality. The results of this study could be used for molecular marker-assisted selection, dramatically improving the quality of alfalfa by molecular means.

## Data Availability Statement

The original contributions presented in the study are publicly available. This data can be found here: https://www.ncbi.nlm.nih.gov/bioproject/?term=PRJNA522887.

## Author Contributions

JK and QY designed the experiments and developed mapping population. CY, ZW, and JK performed the data analysis, and wrote the manuscript. FZ, LC, and RL performed the genotyping and sequencing, and constructed genetic maps. XY, XJ, FH, ZW, TY, CW, and TG managed field work and investigated phenotypic data. All authors read and approved the final manuscript.

## Conflict of Interest

The authors declare that the research was conducted in the absence of any commercial or financial relationships that could be construed as a potential conflict of interest.

## Footnotes

1 https://www.ncbi.nlm.nih.gov/bioproject/?term=PRJNA522887

## Supplementary Material

Supplementary Figure 1 Quality-related QTLs across the 32 linkage groups from a genetic map of the paternal parent. The ruler on the left side represents the relative position of SNP on the chromosome. The small colored blocks represent QTL intervals. Black, QTLs in 2016; red, QTLs in 2019; green, QTLs in 2020; BLUP, QTLs of BLUP.

Supplementary Figure 2 Cyclic diagram of epistatic QTLs for quality-related traits of the paternal parent. The dotted lines indicate the interacting SNP marker pairs mapped on the same or different linkage group with corresponding LOD scores owing to their epistatic effects. Marker position (cM) is mentioned inside the oval located on linkage group. The QTL in the box means it has been reported, *qNDF1C*, *qADF1C-2*, and *qlignin1C-2* (Li et al., 2011); *qNDF7D* and *qADF7D* (Espinoza and Julier, 2013).
