# Supplementary figures and images for: Identification of Genetic Loci Associated With Crude Protein Content and Fiber Composition in Alfalfa (Medicago sativa L.) Using QTL Mapping
Source: Front Plant Sci. 2021 Feb 18;12:608940. doi: 10.3389/fpls.2021.608940 (PMC7933732; doi:10.3389/fpls.2021.608940)

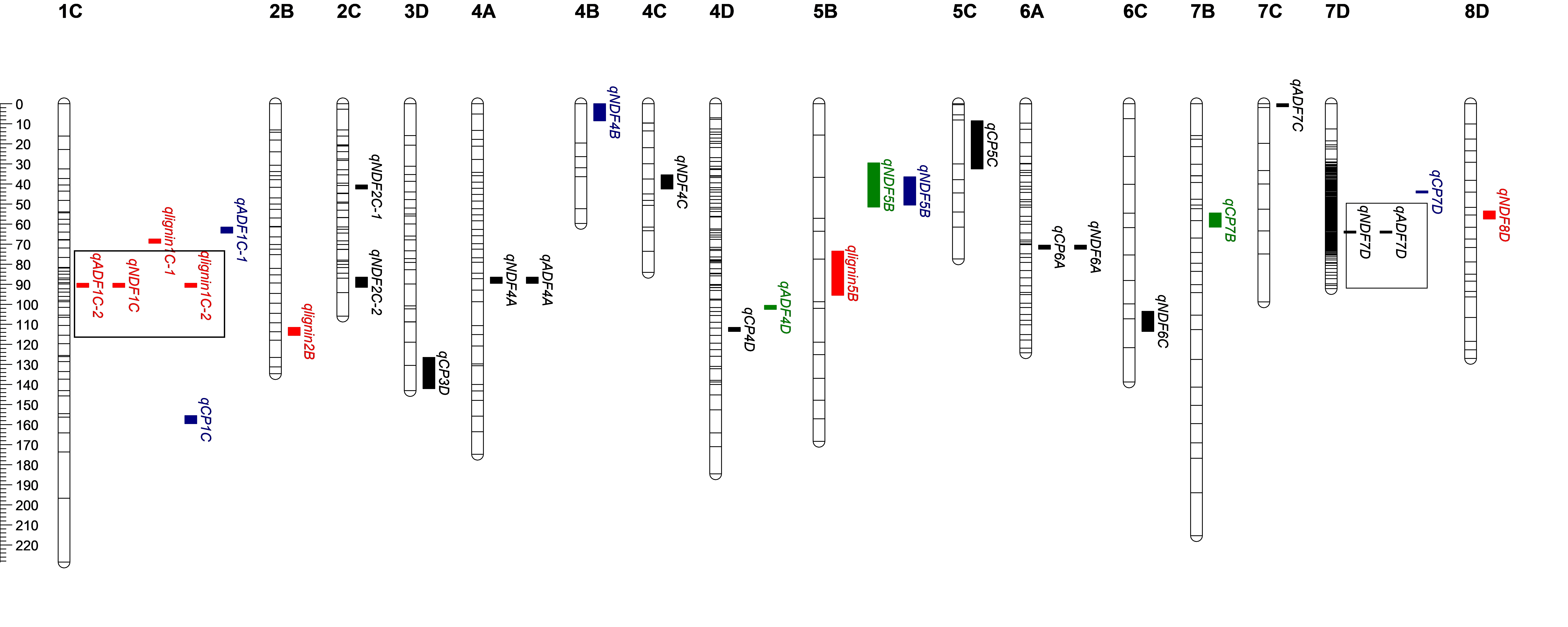

Supplement: Supplementary Figure 1 — Quality-related QTLs across the 32 linkage groups from a genetic map of the paternal parent. The ruler on the left side represents the relative position of SNP on the chromosome. The small colored blocks represent QTL intervals. Black, QTLs in 2016; red, QTLs in 2019; green, QTLs in 2020; BLUP, QTLs of BLUP. [file Image_1.TIF]

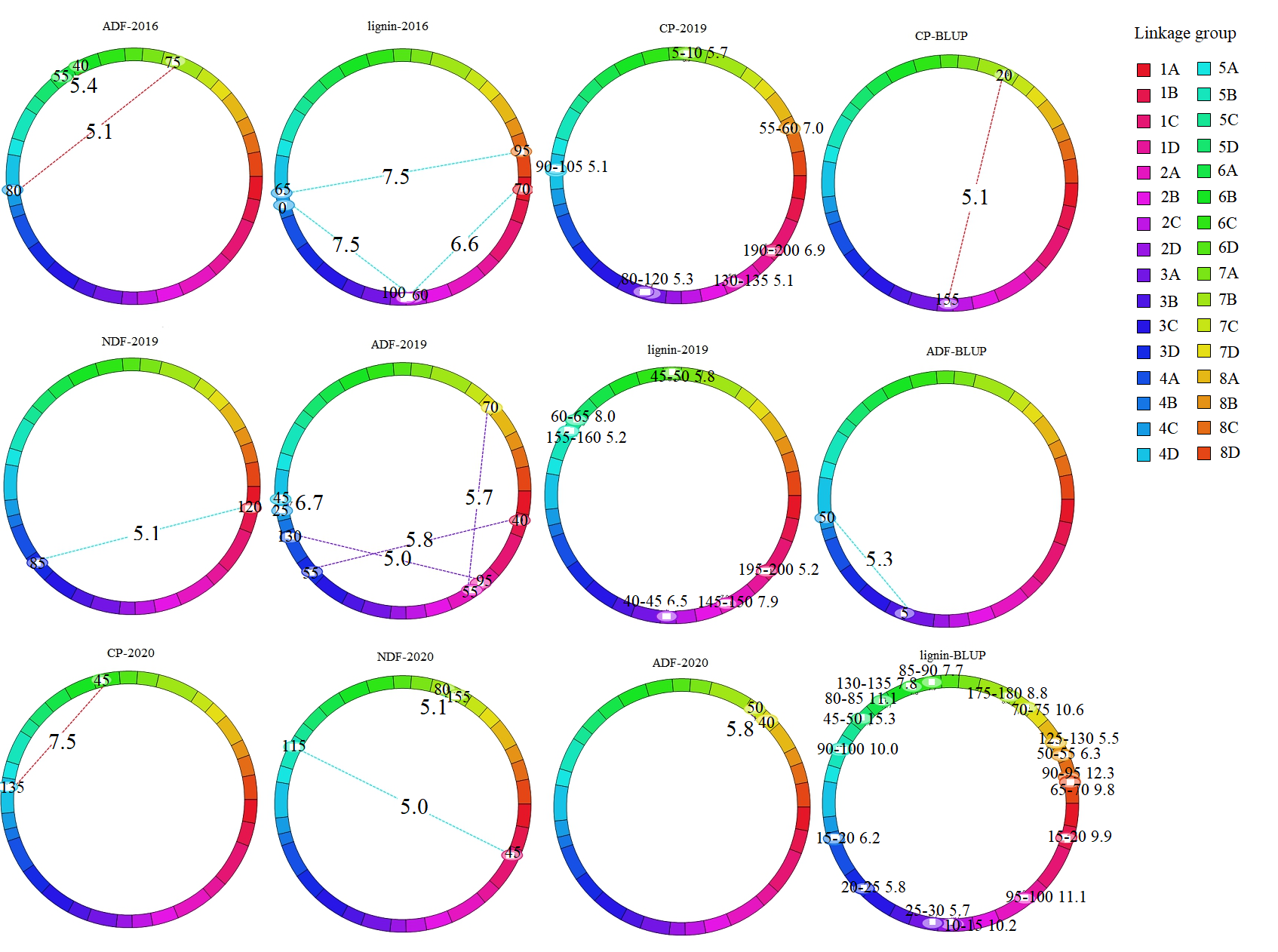

Supplement: Supplementary Figure 2 — Cyclic diagram of epistatic QTLs for quality-related traits of the paternal parent. The dotted lines indicate the interacting SNP marker pairs mapped on the same or different linkage group with corresponding LOD scores owing to their epistatic effects. Marker position (cM) is mentioned inside the oval located on linkage group. The QTL in the box means it has been reported, qNDF1C, qADF1C-2, and qlignin1C-2 (Li et al., 2011); qNDF7D and qADF7D (Espinoza and Julier, 2013). [file Image_2.tif]
