# Supplementary material for: Identification of Genetic Loci Associated With Crude Protein Content and Fiber Composition in Alfalfa (Medicago sativa L.) Using QTL Mapping
Source: Front Plant Sci. 2021 Feb 18;12:608940. doi: 10.3389/fpls.2021.608940 (PMC7933732; doi:10.3389/fpls.2021.608940)
Supplement: Supplementary file 3 [file Table_1.DOCX]

Table S1. Summary statistics of phenotypes for crude protein and fiber in the F1 progeny and parents

(%)

| Items | CP | | | NDF | | | ADF | | | Lignin | | |
| --- | --- | --- | --- | --- | --- | --- | --- | --- | --- | --- | --- | --- |
| Year | 2016 | 2019 | 2020 | 2016 | 2019 | 2020 | 2016 | 2019 | 2020 | 2016 | 2019 | 2020 |
| Mean of  paternal parent | 21.19** | 22.32** | 22.04 | 43.05** | 40.92** | 46.26** | 33.06** | 32.97** | 36.70** | 2.42** | 2.19** | 4.6 |
| Mean of  maternal parent | 18.83 | 19.27 | 20.88 | 51.86 | 48.97 | 50.22 | 38.51 | 35.9 | 40.74 | 3.36 | 2.95 | 5.47 |
| Variation range | 15.16 - 25.75 | 16.79 - 31.52 | 17.94 - 35.03 | 30.92 - 55.87 | 39.80 - 65.33 | 36.53 - 58.91 | 27.28 - 48.11 | 27.60 - 46.41 | 29.01 - 48.55 | 4.28 - 6.65 | 0.76 - 11.57 | 1.22 - 13.92 |
| Mean±σ | 19.62 ± 1.33 | 21.68 ± 2.37 | 25.06 ± 3.46 | 47.10 ± 3.43 | 51.53 ± 4.60 | 48.92 ± 4.08 | 39.98 ± 3.11 | 36.39 ± 3.05 | 39.70 ± 3.74 | 5.71 ± 0.40 | 3.73 ± 1.72 | 7.04 ± 2.43 |
| Skewness | 0.13 | 1.32 | 0.6 | -0.2 | 0.48 | -0.05 | -0.2 | 0.54 | 0.08 | -0.1 | 1.35 | 0.42 |
| Kurtosis | 1.3 | 3 | -0.11 | 0.83 | 0.11 | -0.3 | 0.45 | 0.34 | -0.26 | -0.08 | 2.1 | -0.3 |

Note: ** means a significant difference (P < 0.01)
