# Supplementary material for: Identification of Genetic Loci Associated With Crude Protein Content and Fiber Composition in Alfalfa (Medicago sativa L.) Using QTL Mapping
Source: Front Plant Sci. 2021 Feb 18;12:608940. doi: 10.3389/fpls.2021.608940 (PMC7933732; doi:10.3389/fpls.2021.608940)
Supplement: Supplementary file 4 [file Table_2.DOCX]

Table S2. Paternal QTL mapping results

| Year | QTL | Linkage  Group | Position  /cM | Left  Marker | Right  Marker | LOD | A | PVE(A)/% | AE | PVE(AE)/% |
| --- | --- | --- | --- | --- | --- | --- | --- | --- | --- | --- |
| 2016 | *qCP3D* | 3D | 126.5 - 142 | TP20533 | TP93056 | 2.66 | 0.26 | 3.09 | 0.19 | 0.52 |
|  | *qCP4D* | 4D | 111.5 - 113.5 | TP7700 | TP40032 | 3.92 | -0.29 | 3.84 | -0.11 | 0.32 |
|  | *qCP5C* | 5C | 8.5 - 32.5 | TP41667 | TP24513 | 3.12 | 0.29 | 3.71 | 0.08 | 0.41 |
|  | ***qCP6A*** | 6A | 70.5 - 72.5 | TP38894 | TP40565 | 2.70 | 0.27 | 3.24 | 0.14 | 0.10 |
|  | *qNDF2C-1* | 2C | 40.5 - 42.5 | TP37679 | TP77429 | 6.13 | 0.89 | 4.87 |  |  |
|  | *qNDF2C-2* | 2C | 86.5 - 91.5 | TP76542 | TP97847 | 3.08 | 0.64 | 2.53 | 0.30 | 1.05 |
|  | ***qNDF4A*** | 4A | 86.5 - 89.5 | TP35668 | TP13805 | 6.40 | -0.92 | 5.34 | -0.36 | 1.28 |
|  | *qNDF4C* | 4C | 35.5 - 42.5 | TP22563 | TP58863 | 3.58 | -0.73 | 3.11 |  |  |
|  | ***qNDF6A*** | 6A | 70.5 - 72.5 | TP38894 | TP40565 | 2.55 | -0.61 | 2.29 |  |  |
|  | *qNDF6C* | 6C | 103.5 - 113.5 | TP87419 | TP71529 | 3.29 | 0.64 | 2.39 |  |  |
|  | ****qNDF7D*** | 7D | 63.5 - 64.5 | TP63197 | TP51972 | 7.62 | 1.02 | 6.14 | 0.40 | 1.28 |
|  | ***qADF4A*** | 4A | 86.5 - 89.5 | TP23875 | TP35668 | 3.37 | -0.62 | 3.91 | -0.37 | 1.75 |
|  | *qADF7C* | 7C | 0 - 1.5 | TP49162 | TP31792 | 2.84 | -0.55 | 3.02 | -0.46 | 2.70 |
|  | ****qADF7D*** | 7D | 63.5 - 64.5 | TP63197 | TP51972 | 7.11 | 0.96 | 8.73 | 0.49 | 3.03 |
| 2019 | ****qNDF1C*** | 1C | 89.5 - 91.5 | TP88518 | TP74179 | 3.36 | -1.07 | 4.76 | -0.37 | 1.60 |
|  | *qNDF8D* | 8D | 53.5 - 57.5 | TP12005 | TP30612 | 3.57 | 1.15 | 5.40 | 0.51 | 2.28 |
|  | ****qADF1C-2*** | 1C | 89.5 - 91.5 | TP88518 | TP74179 | 2.86 | -0.67 | 5.08 | -0.27 | 1.08 |
|  | *qlignin1C-1* | 1C | 67.5 - 69.5 | TP78664 | TP65950 | 2.55 | -0.33 | 3.01 |  |  |
|  | ****qlignin1C-2*** | 1C | 89.5 - 91.5 | TP88518 | TP74179 | 2.91 | -0.42 | 4.95 | -0.15 | 1.45 |
|  | *qlignin2B* | 2B | 111.5 - 115.5 | TP72790 | TP72423 | 3.45 | 0.42 | 3.59 |  |  |
|  | *qlignin5B* | 5B | 73.5 - 95.5 | TP19967 | TP73306 | 2.92 | 0.39 | 4.26 | 0.14 | 1.17 |
| 2020 | *qCP7B* | 7B | 54.5 - 61.5 | TP41636 | TP36891 | 2.80 | -0.81 | 4.70 | -0.30 | 1.63 |
|  | ***qNDF5B*** | **5B** | 29.5 - 51.5 | TP64394 | TP54345 | 2.83 | -1.09 | 4.36 | -0.19 | 0.46 |
|  | *qADF4D* | 4D | 100.5 - 102.5 | TP96228 | TP27054 | 2.56 | -0.91 | 4.85 |  |  |
| BLUP | *qCP1C* | 1C | 155.5 - 159.5 | TP83295 | TP86272 | 2.62 | 0.27 | 1.45 |  |  |
|  | *qCP7D* | 7D | 43.5 - 44.5 | TP93438 | TP62238 | 10.24 | 0.54 | 5.52 |  |  |
|  | *qNDF4B* | 4B | 0 - 8.5 | TP65453 | TP58519 | 4.25 | 0.77 | 4.64 |  |  |
|  | ***qNDF5B*** | **5B** | 36.5 - 50.5 | TP64394 | TP54345 | 3.35 | - 0.75 | 4.51 |  |  |
|  | *qADF1C-1* | 1C | 61.5 - 64.5 | TP28232 | TP91134 | 4.82 | - 0.64 | 6.31 |  |  |

Note: QTLs were bold to indicates that this QTL co-located with others. “*QTL” means it had been identified previously. *qNDF1C*, *qADF1C-2* and *qlignin1C-2* (Li et al., 2011*)*; *qNDF7D* and *qADF7D* (Espinoza and Julier, 2013); A: additive effects. PVE(A): percentage of phenotypic variation explained by the QTL at the current position. AE: The additive QTL×environment interaction under current year. PVE(AE): percentage of phenotype variance explained by additive QTL×environment.
