# Supplementary material for: Identification of Genetic Loci Associated With Crude Protein Content and Fiber Composition in Alfalfa (Medicago sativa L.) Using QTL Mapping
Source: Front Plant Sci. 2021 Feb 18;12:608940. doi: 10.3389/fpls.2021.608940 (PMC7933732; doi:10.3389/fpls.2021.608940)
Supplement: Supplementary file 5 [file Table_3.DOCX]

Table S3. Paternal epistatic QTL mapping results

| Year | Trait | Lingkage  group | Position  /cM | Left  Marker | Right  Marker | Lingkage  group | Position  /cM | Left  Marker | Right  Marker | LOD | AA | PVE(AA)/% |
| --- | --- | --- | --- | --- | --- | --- | --- | --- | --- | --- | --- | --- |
| 2016 | ADF | 6A | 55 | TP7725 | TP58364 | 6B | 40 | TP77253 | TP41621 | 5.44 | -1.18 | 6.22 |
|  | ADF | 4C | 80 | TP3709 | TP32979 | 7B | 75 | TP66624 | TP62959 | 5.05 | -0.84 | 3.04 |
|  | Lignin | 1A | 70 | TP10131 | TP7452 | 2C | 60 | TP96830 | TP23662 | 5.50 | 0.12 | 6.61 |
|  | Lignin | 2C | 100 | TP97847 | TP10264 | 4C | 0 | TP47706 | TP77966 | 5.21 | -0.11 | 7.45 |
|  | Lignin | 4C | 65 | TP72000 | TP3709 | 8C | 95 | TP73871 | TP17764 | 5.98 | -0.12 | 7.52 |
| 2019 | CP | 1C | 190 | TP6403 | TP71156 | 1A | 200 | TP71156 | TP57346 | 6.93 | -3.06 | 3.03 |
|  | CP | 2A | 130 | TP7156 | TP67828 | 2A | 135 | TP67828 | TP80161 | 5.07 | -2.15 | 2.27 |
|  | CP | 3A | 80 | TP1452 | TP69867 | 3A | 120 | TP78202 | TP31047 | 5.30 | -1.87 | 1.62 |
|  | CP | 4D | 90 | TP15851 | TP5449 | 4D | 105 | TP40011 | TP65488 | 5.11 | -1.94 | 5.38 |
|  | CP | 7A | 5 | TP47318 | TP83927 | 7A | 10 | TP47318 | TP83927 | 5.69 | -1.62 | 4.56 |
|  | CP | 8B | 55 | TP37910 | TP57964 | 8B | 60 | TP57964 | TP6915 | 7.00 | -2.88 | 3.23 |
|  | ADF | 1B | 40 | TP6474 | TP92786 | 3D | 55 | TP29794 | TP26931 | 5.79 | -1.15 | 6.51 |
|  | ADF | 2A | 95 | TP42093 | TP26965 | 4A | 130 | TP32369 | TP56961 | 5.04 | -1.18 | 6.72 |
|  | ADF | 4A | 45 | TP22563 | TP58863 | 4D | 25 | TP75063 | TP85200 | 6.67 | -1.27 | 7.62 |
|  | ADF | 2A | 55 | TP13658 | TP901 | 7D | 70 | TP84290 | TP18647 | 5.68 | 1.10 | 6.17 |
|  | NDF | 1A | 120 | TP52698 | TP4371 | 3D | 85 | TP65714 | TP2199 | 5.06 | -1.68 | 6.55 |
|  | Lignin | 1C | 195 | TP6403 | TP71156 | 1C | 200 | TP71156 | TP57346 | 5.19 | -2.05 | 1.93 |
|  | Lignin | 2A | 145 | TP80161 | TP46917 | 2A | 150 | TP46917 | TP4820 | 7.92 | -1.75 | 1.73 |
|  | Lignin | 2D | 40 | TP11742 | TP4045 | 2D | 45 | TP4045 | TP49553 | 6.55 | -1.96 | 1.86 |
|  | Lignin | 5B | 155 | TP43755 | TP63177 | 5B | 160 | TP63177 | TP57195 | 5.24 | -1.03 | 1.35 |
|  | Lignin | 5C | 60 | TP6526 | TP23258 | 5C | 65 | TP23258 | TP48964 | 8.04 | -1.96 | 2.10 |
|  | Lignin | 6D | 45 | TP96577 | TP77971 | 6D | 50 | TP96577 | TP77971 | 5.76 | -1.01 | 1.50 |
| 2020 | CP | 4D | 135 | TP48855 | TP29285 | 6C | 45 | TP23040 | TP80223 | 7.48 | -1.81 | 10.27 |
|  | ADF | 7C | 50 | TP86482 | TP21999 | 7D | 40 | TP55016 | TP10879 | 5.78 | -1.44 | 7.75 |
|  | NDF | 1C | 45 | TP88886 | TP71134 | 5B | 115 | TP32287 | TP42044 | 5.06 | -1.56 | 11.18 |
|  | NDF | 7B | 80 | TP62959 | TP28033 | 7B | 155 | TP1657 | TP81082 | 5.12 | 1.62 | 9.01 |
| BLUP | CP | 2D | 20 | TP82897 | TP11742 | 7B | 155 | TP1657 | TP81082 | 5.10 | 0.49 | 7.53 |
|  | ADF | 3B | 5 | TP62361 | TP33868 | 4C | 50 | TP4405 | TP65371 | 5.29 | -0.78 | 6.12 |
|  | Lignin | 1B | 65 | TP76436 | TP80663 | 1B | 70 | TP80663 | TP7556 | 9.77 | 0.40 | 1.16 |
|  | Lignin | 2A | 15 | TP100166 | TP27169 | 2A | 20 | TP27169 | TP69910 | 9.88 | 0.63 | 1.08 |
|  | Lignin | 2C | 95 | TP97847 | TP10264 | 2C | 100 | TP97847 | TP10264 | 11.12 | 0.54 | 0.98 |
|  | Lignin | 2D | 10 | TP22629 | TP82897 | 2D | 15 | TP22629 | TP82897 | 10.17 | 0.58 | 1.00 |
|  | Lignin | 3A | 25 | TP83007 | TP68685 | 3A | 30 | TP68685 | TP72134 | 5.69 | 0.61 | 0.83 |
|  | Lignin | 3D | 20 | TP80109 | TP69882 | 3D | 25 | TP69882 | TP51708 | 5.83 | 0.54 | 1.07 |
|  | Lignin | 4B | 15 | TP65453 | TP58519 | 4B | 20 | TP58519 | TP7159 | 6.23 | 0.45 | 0.72 |
|  | Lignin | 5B | 90 | TP19967 | TP73306 | 5B | 100 | TP73306 | TP32287 | 9.98 | 0.49 | 0.76 |
|  | Lignin | 5D | 45 | TP64261 | TP28239 | 5D | 50 | TP64261 | TP28239 | 15.32 | 0.47 | 1.37 |
|  | Lignin | 6A | 80 | TP38434 | TP98963 | 6A | 85 | TP98963 | TP22713 | 11.13 | 0.64 | 1.08 |
|  | Lignin | 6B | 130 | TP52094 | TP87392 | 6B | 135 | TP87392 | TP70541 | 7.79 | 0.63 | 0.91 |
|  | Lignin | 6C | 85 | TP37735 | TP29900 | 6C | 90 | TP29900 | TP87419 | 7.67 | 0.55 | 0.70 |
|  | Lignin | 7B | 175 | TP48222 | TP64065 | 7B | 180 | TP64065 | TP81770 | 8.80 | 0.58 | 1.04 |
|  | Lignin | 7C | 70 | TP71154 | TP63371 | 7C | 75 | TP63371 | TP21579 | 10.64 | 0.55 | 1.18 |
|  | Lignin | 8A | 125 | TP24723 | TP39434 | 8A | 130 | TP39434 | TP80497 | 5.50 | 0.48 | 0.69 |
|  | Lignin | 8B | 50 | TP37910 | TP57964 | 8B | 55 | TP37910 | TP57964 | 6.33 | 0.55 | 0.88 |
|  | Lignin | 8C | 90 | TP34592 | TP73871 | 8C | 95 | TP73871 | TP17764 | 12.39 | 0.63 | 1.05 |
|  | Lignin | 8D | 15 | TP35711 | TP100630 | 8D | 20 | TP100630 | TP33350 | 5.32 | 0.60 | 0.78 |

Note: QTLs were bold to indicates that this QTL co-located with others.A: additive effects. AA：epistatic effect at its direction, the positive value means parent-type effect is more than recombinant-type effect, and the negative value means parent-type effect is low than recombinant-type effect; PVE(AA): percentage of phenotypic variance explained by a pair of epistatic QTLs .
