# Supplementary material for: Identification of Genetic Loci Associated With Crude Protein Content and Fiber Composition in Alfalfa (Medicago sativa L.) Using QTL Mapping
Source: Front Plant Sci. 2021 Feb 18;12:608940. doi: 10.3389/fpls.2021.608940 (PMC7933732; doi:10.3389/fpls.2021.608940)
Supplement: Supplementary file 6 [file Table_4.docx]

Table 4. Maternal epistatic QTL mapping results

| Year | Trait | Lingkage  group | Position  /cM | Left  Marker | Right  Marker | Lingkage group | Position  /cM | Left  Marker | Right  Marker | LOD | AA | PVE(AA)/% |
| --- | --- | --- | --- | --- | --- | --- | --- | --- | --- | --- | --- | --- |
| 2016 | ADF | 1C | 105 | TP49934 | TP25688 | 4D | 90 | TP19263 | TP27443 | 5.14 | -0.85 | 6.53 |
|  | NDF | 1C | 105 | TP49934 | TP25688 | 4D | 90 | TP19263 | TP27443 | 5.14 | -0.93 | 6.12 |
|  | NDF | 4A | 35 | TP49781 | TP29000 | 5B | 30 | TP84450 | TP8666 | 5.14 | -0.90 | 6.45 |
|  | CP | 2A | 115 | TP61092 | TP24700 | 4D | 65 | TP52539 | TP98872 | 5.59 | 0.36 | 4.73 |
|  | CP | 7D | 55 | TP65979 | TP20321 | 8A | 20 | TP56066 | TP66664 | 5.51 | 0.39 | 5.67 |
| 2019 | CP | 8A | 15 | TP53343 | TP28159 | 8A | 30 | TP57124 | TP80035 | 5.54 | -2.04 | 9.58 |
|  | ADF | 1D | 75 | TP19593 | TP101046 | 1D | 80 | TP76240 | TP28034 | 5.49 | -1.51 | 8.54 |
|  | ADF | 2A | 130 | TP88115 | TP29238 | 2C | 140 | TP9298 | TP92928 | 5.11 | -0.93 | 4.02 |
|  | ADF | 2B | 105 | TP70295 | TP80588 | 2D | 70 | TP77221 | TP62151 | 5.57 | -1.01 | 5.04 |
|  | ADF | 3C | 50 | TP55388 | TP82617 | 4A | 45 | TP12678 | TP63461 | 5.10 | 0.92 | 4.00 |
|  | ADF | 2A | 20 | TP64012 | TP80444 | 7C | 75 | TP34608 | TP65366 | 5.24 | -1.08 | 6.11 |
|  | ADF | 1A | 50 | TP7459 | TP58514 | 8D | 5 | TP36743 | TP92422 | 5.35 | -1.06 | 4.42 |
|  | ADF | 2C | 20 | TP24016 | TP71363 | 8D | 10 | TP92422 | TP83401 | 5.74 | -1.08 | 6.07 |
|  | NDF | 2C | 130 | TP18803 | TP74542 | 4D | 90 | TP19263 | TP27443 | 5.25 | -1.56 | 8.75 |
|  | NDF | 1D | 55 | TP15096 | TP44384 | 6D | 15 | TP2500 | TP88872 | 5.01 | 1.44 | 9.84 |
| 2020 | NDF | 6C | 80 | TP32090 | TP65171 | 7B | 95 | TP82142 | TP71217 | 5.33 | 1.28 | 8.36 |
| BLUP | ADF | 1C | 95 | TP50518 | TP56601 | 4D | 65 | TP52539 | TP98872 | 6.60 | -0.82 | 14.06 |
|  | lignin | 2C | 105 | TP43293 | TP92468 | 2C | 140 | TP9298 | TP92928 | 8.77 | 0.58 | 1.03 |
|  | lignin | 3C | 125 | TP5711 | TP16585 | 3C | 150 | TP18006 | TP58533 | 8.46 | 0.50 | 1.41 |
|  | lignin | 4B | 60 | TP91460 | TP42210 | 4B | 65 | TP30181 | TP50777 | 8.02 | 0.60 | 2.22 |
|  | lignin | 4C | 25 | TP67727 | TP83028 | 4C | 30 | TP83028 | TP73453 | 14.15 | 0.61 | 2.69 |
|  | lignin | 5B | 5 | TP45260 | TP56801 | 5B | 45 | TP18390 | TP56230 | 10.15 | 0.46 | 1.80 |
|  | lignin | 5D | 55 | TP38687 | TP89063 | 5D | 65 | TP23542 | TP100802 | 6.97 | 0.25 | 1.24 |
|  | lignin | 6B | 145 | TP56692 | TP46412 | 6B | 150 | TP46412 | TP27249 | 6.49 | 0.59 | 1.58 |
|  | lignin | 7A | 130 | TP18598 | TP22527 | 7A | 135 | TP83170 | TP64804 | 6.37 | 0.69 | 1.36 |
|  | lignin | 8A | 70 | TP42256 | TP90122 | 8A | 90 | TP89679 | TP77453 | 6.59 | 0.60 | 1.56 |
|  | lignin | 8D | 85 | TP2112 | TP23361 | 8D | 100 | TP56432 | TP26092 | 6.97 | 0.34 | 1.15 |

Note: QTLs were bold to indicates that this QTL co-located with others.A: additive effects. AA：epistatic effect at its direction, the positive value means parent-type effect is more than recombinant-type effect, and the negative value means parent-type effect is low than recombinant-type effect; PVE(AA): percentage of phenotypic variance explained by a pair of epistatic QTLs .
