# Supplementary material for: Identification of Genetic Loci Associated With Crude Protein Content and Fiber Composition in Alfalfa (Medicago sativa L.) Using QTL Mapping
Source: Front Plant Sci. 2021 Feb 18;12:608940. doi: 10.3389/fpls.2021.608940 (PMC7933732; doi:10.3389/fpls.2021.608940)
Supplement: Supplementary file 7 [file Table_5.docx]

### Table S5. Potential Candidate Genes of *qCP4C-1*

>MS.gene004008.t1

ATGGATCCAAAAAAGGTTGAAGATTTGGTTTATGTGCATGTTAATCTTCGACCCTTGTCA

AGGAAAGAAGAAGGGTATAAGAAAGGCGAGACAAAGTTGTGGGACATTAGTGGAGATGCA

CATGACCCGCTTAATGGTGTTGGAATTGAATTAGCTGAGTTGTCTTTAGATGAACCGGAG

TTTGAGGCTACAATGTTCTTTGATGATGGAAACAGAGGAGAGGAGACTGATTCAATCTGT

GTTTCCTCTTAA

>MS.gene004006.t1

ATGGCTGCGAATGTTGTGAAAGAAGCTGAAGCAACGGAGATGGAAGCTGTTTTGACCGCA

AAACGCAGCTGTTGCGGGTTGGTGGAAGAGTGCACACATGAGTACATTGCGCGCGTGAGG

GAGAGATTCGGTGGAAGATGGATATGCGGTTTATGTGCTGAGGCAGTGAAGGAAGAAAGG

ACGAAGTCGGAGTCATCCAGCAGCAACGTACAAGGTCGCGAACGGGATCCTCTTCATCAC

AGATCAACCGAATCGAAGTCTTGA

>MS.gene004010.t1

ATGAGTTTTGCTTTTCGAGGGAGTAGGGGAGATATTGAAAACGGATTTTCAGAATATGTT

CCTGAGAGAACCTCAATGCGGGTTCGTCCATCTCGGCCAGTCCATAGCAATTGTCTGGTT

TTTCTTTTTGCCGTGATTATGATATTCGTGATATTATACTCCCCTCAAATGATATATTAT

TTTTTGCGCTGGATAATACTGTCGGTCTTTGTGATGGTTACAAGTCTGAGAGCGTATGCA

ATTTATCTACATCTTCAAGCACAGGCCAGGGCTCATGCTGCAGCAGCCTCTGGGTTACTT

GGCCATGCTGAATTACGAGTTCACGTGCCACCATCTATAGCATTTGCAACAGGAGGAAGA

TTGCAGGAACTTAGACTCCAGCTTGCACTTCTTGACCGAGATTTTAATGAGATAGATTAT

GACACTTTGAGGGTTCTTGCTTTTGGTACTCGTTCAATGTCTGAGGAGGAGATAAATGCC

TTGCCTATTCACAAACATAAAGTTACTGGCCCAATAGAAGATGGCTCCACCGGTTCGACA

TCCTCTTCTTCAGAGGCAGCTGAGATTAAACAAGATTTCAAAGGAGAAGAGGGAAGTGCC

AATGATCAAGAAGATGGGTTGACATGTGCTATATGCTTGGATGAAGTTCAGAGGGGGGAA

CTTGTGCGTAGCTTGCCATGCTTGCATCAGTTTCATGCCAACTGCATCGATCAATGGCTC

CGGCGAAAACGAACATGTCCTGTATGCAAATTTAAGATGGGGGCGGGATGGCTATGTAAC

AATGCATGCGAGTCGGATGATTCAAATATTGTGTAG

>MS.gene004009.t1

ATGAATTCTGAATCCTCACCCGAAACGCCGTCGTACTGGTGCTACAGTTGCACACGCTTC

ATCAACCTATCAGATCACACCCTCATCGTTTGTCCTCACTGCGATAACGGTTTCGTCGAA

GAGATCGCCGCCGGCGAGTCACCGCATCACCGTCTCAGTCCTTTCCCCGCCGACACCGTT

TCTTCCCGTCGTCAAGGCTTTCGCAGACGCCGTCGTGACGCTGGTAGTCGTTCACCTTTC

AATCCTGTCATCGTTCTTCGTGGAGCCGGTGATGATGGAGTCGGAGAAGACGGCGCCGCT

GCTGGTGGTTCCGCTTTTGAACTTTTCTACGATGACGGTGATGGTTCGGGGTTACGTCCG

CTACCACCGACGGTTTCGGAGTTTTTGCTTGGATCTGGATTTGATCGGTTGTTGGAGCAG

TTTTCTCAGATCGAGATGAATGGTTTTGGACGGCCGGAGAATCCACCGGCGTCTAAAGCG

GCGATTGAATCGATGCCAACGGTTGAGATCTGTGAAGAACACGTTAGTTGTGAATTACAC

TGCGCCGTTTGCAAAGAGGAGTTTGAGCTACACGCGGAGGCGCGTGAGTTACCTTGCAAG

CATTTATATCATTCCGATTGCATTCTTCCATGGCTGACAGTGAGAAACTCTTGCCCGGTG

TGCCGCCATGAGCTTCCTTCTGATCTCAACAATCCTCTTGAAACTAGGGTTTCTGGTCAA

ATTGATGAAGAAGCTATCGGGTTAACGATATGGAGGTTACCCGGTGGTGGATTCGCTGTT

GGGAGATTCTCCGGTGGAAGAAGTGCCGGGGAGAGTCATTTTCCGGTTGTGTATACTGAG

ATGGATGGTGGGCTCAATGCTGCACAAGGATCGGCTCCGAGGAGAATATCTAGAACGGTT

AGAGCTCATAGGGTTAGGGAAAGTCATGGAATTGGAAGAGTTTTTCGGAATTTTGTCTCA

TTCTTTGGGAGAATTGGTTCTAGGTCAAATTCAAATTTGAATTCTGGAACGGAGAATGTT

GCTTCTGTAAGTAGAAGCCGTAGTTTAGTGAATTCAATGTTCAATAGAAACTCGCGGCGA

CGTAGGAGAGCTTTGGAGTTGGAAGATTGA

>MS.gene004011.t1

ATGCATATCAAAATGATTGTCACTGTTCCATTATCAAAGGCTCTTTTTGCAGCTTTTCTC

TTTGTCACCACCTTCCTCTTAACCTCCCCTGGATTTGTTTTGGCTACTTCACAACATGGA

AGTGTTACAAGACACTACAAATTTGATATAAGGTTGGCAAAGGTTACGAGGCTGTGCCAC

ACAAAAAGCATGGTTACCGTGAATGGGAAGTTCCCGGGTCCTCGAATTGTTGTACGAGAA

GGGGACAGATTATTGGTTAAGGTTGTTAATCATGTTCCAAACAATATCAGCCTGCATTGG

CATGGTGTGAGACAACTTCGTAGTGGTTGGTCGGATGGTCCATCATACATAACTCAATGC

CCAATTCAAACAGGTCAGAGTTATGTGTACAACTTCACCATTGTTGGACAAAGAGGAACT

CTCTTCTGGCATGCTCATTTCTCATGGTTAAGAGCTACTGTCTATGGACCTCTCATCCTT

CTCCCAAGGCACAATGAATCTTATCCTTTTCAAAAACCCTACAAGGAAGTCCCCATTCTC

TTTGGTGAGTGGTGGAATGCAGATCCTGAAGCTGTAATTGCACAAGCTCTGCAGACAGGG

GGTGGCCCAAATGTTTCTGATGCCTACACCATTAATGGGTTTCCTGGACCTCTTTACAAT

TGCTCCAAAGATACATACAAATTGAAGGTGAAGCCAGGAAAAACATATCTATTACGTTTG

ATCAACGCTGCACTCAACGACGAACTCTTTTTCAGCATTGCTAATCACACATTAATCCTT

GTCGAAGCAGATGCATCTTACATTAAACCATTCGAGTCCAATACCATCATACTTGGACCA

GGTCAAACCACAAATGTTCTATTAAAAACTAAACCAAATTACCCAAATGCCACATTCTAC

ATGCTAGCTAGACCATATTTCACAGGGCAGGGTACTTTCGACAATTCCACAGTAGCTGGA

ATTTTAGAATACACTAAACCAAATGATCAAACAAATAAAAATCTCCATATTCTAAAACCT

GTGCTTCCAGCCATCAATGATACTAATTTTGTTGCTAATTTTAGTAACAAATTTCTTAGC

TTAAATAGTCCAAAATACCCTGCAAATGTGCCTAAAACTATTGACAAAAACTTTTTCTTT

ACCGTTGGACTAGGAACTAGTCCATGCCCAAAAAACCAAACTTGTCAAGGACCAAATAAT

AGTTCAAATTCAAAATTTGCAGCTTCAATGAACAATGTATCATTTGCTTTACCTTCTATA

GCACTTCTCCAACAACATTTCTTTGGAACTAATAATGGTTTTACAACGGATTTTCCGGTT

GTACCGTTAAGGCCGTTTAACTATACAGGAACTCCTCCAAACAATACTTTGGTTAGTAAT

GGTACAAAGACAGTGGTGATACCATATAACACTAGAGTTCAGGTTATATTGCAGGATACA

AGCATTTTGGGAGCTGAGAGTCATCCTTTGCATCTTCATGGATTTAATTTCTTTGTTGTT

GGTCAAGGTTTTGGAAATTTTAATGCTAGTAGTGACCCTGCAAAGTTTAATCTTGTTGAT

CCTGTTGAGAGGAATACTGTTGCTGTGCCTTCTGGTGGTTGGGTCGCGATACGATTCCTT

GCTGACAATCCAGGTGTTTGGCTGATGCATTGCCACTTTGATGTGCACCTTAGTTGGGGA

TTGAGGATGGCTTGGATTGTTGAGGATGGAAAGTTACCTAATCAAAAATTGCCTCCTCCT

CCTAAGGATCTCCCCAAATGTTGA

>MS.gene004005.t1

ATGTTGTCTGAGTTGGGAAGAAGACCAGTGATTGGCAATAACGAGGGTTGTTTTGGTGAT

GAATTGGAGAAGGAAATAGGAATGTTGCTCCGCGAACAACGGAGGCAAGAAGGCGATGAT

CGCGAAAGAGAGTTGAATATATTTAGGAGCGGATCAGCTCCTCCTACTGTGGAGGGTTCT

TCGAATGCTGTTGGAGGGTTGTTTGGTGGCGGTGGTGGTGGTGGTGTTGCTGGTGGTTTT

TCAGATTTTCCTGGGAATAAAGATGTGAATGGTGTTGTTTCTGAGGAAGAGCTTAGGTCT

GATCCAGCTTATCTTCAATACTATTATTCAAATGTGAATTTGAATCCTAGGCTTCCACCT

CCTTTGTTGTCAAAGGAAGATTGGAGGTTTCAACAAAGGCTTAAAGGTGGGGCCTCAGCT

GTAGGTGGAATTGGAGATAGGAGGAAAGGTAACATGACTGATGATAGTGGTGGTAGGTCA

ATGTTTTCGACGCCACCTGGTTTTAACTTTAGGAAGCAAGAAAGGAGTGAGGTGGAGAAT

GAAAAGACCAGAGGTTCTGCTGAATGGGGTGGTGATGGTTTGATTGGTTTGCCTAGCTTG

GGACTTAGAAGCAAACAAAAGAGCCTTGCTGAAATTTTTCAGGATGATATGGAGCGTAAC

ACCCCCGTCACAGGCCTTCCTTCCCGTCCAGCAAGTCGTAATGCATTCGATGAAAATGTT

GATAAAGTAAATACTTCTGAAGCAGAGTTCGCTCATGTTCAAGGATCATCTGCAACACAA

AATATTGGTCTGCCAGCTTCATATTCTTATGCTGCTGCACTAGGAGGATCTTCATTGTCA

AGAAGCACTACTCCTGATCCACAACATATTGCAAGGGCTCCTAGTCCATGTCTCACACCT

ATTGGCGGAGGGAGAAATGTCTCTTCTGACAAGAGAGGCATTGTCAGTCCAGATTCATTT

AACGGTGTTTCATCTGGCATGAACGAGTCAGCAGATCTTGTGGCTGCTTTATCCGGGATG

AACTTGTCAGCAGAAGATGAAAACCATTTGCCATCACAGGTTGAGTCTGATGTCGATAAT

CATCAGAGATATCTTTTTGGTATGCAGGGTGGTCAAGAGCACGGCAAGCAACATCCATAT

CTAAAAAATTCTGAATCAGGGCATTTGCAAAGTTCAGGTAAGAGCAGGTCAGACCTTAAT

AATCTGTCCCTGCACAGGCAGGCTGAGCTACAAAACTCGACTGCTCCTTTGAATAACTCA

TATTTCAAAGGATCACCTACCTCCCATTTTAGTGGAGGAGGTAATATGCAAGCTCAATAT

CAGTCTATAGATGGTATGAATTCATCATTCACTAATTATGGCTTAAGCGGTTATGGTGGA

AATCCTGCAGCATTGACATCCTTGATGACTAACCAATATGGCACCAGTAATCTGCCACCG

ATGTTTGAAAACGTTGCCGCTGCATCCATGATGGCATCCCCTGGAATGGACTCGAGAATT

CTTGGAGGTGGTTTGGCTTCGGGAGCTGGTTCTCCTTCTGATGCGCATAATCTTGGTCGG

ATGGGAAATCAAATTGCTGGTGGTGCTCTTCAGGCTCCGTTTGTTGATCCGATGTATCTT

CAGTATATGAGGACGTCAGAGTATGCTGCGGCACAACTCGCTGCTCTTAACGACCCCTCT

GTGGACAGGAACTACTTGGGTAATTCGTACATGAATTTACTTGAGCTGCAGAAAGCCTAC

CTCGGGTCTGTTCTCTCGCCTCAAAAGTCACAGTACAATGCACAACTTGGTGGTAAATCA

GGCAACTCCAATCACCATGGTTACTATGGAAATCCGGCATATGGTGTTGGGTTGTCTTAT

CCAGGAAGTCCAATGGCAAACTCTGGTTCTCCAGTTGGATCCGGAAGTCCAATTAGGCAC

AATGATCTGAACAACATGCGTTTTGCTTCTGGAATGAGGAACTTAGCTGGGGTCATGGGA

CCTTGGCATGTAGATTCCGGGAATATGGATGAAAGCTTTGCTTCTTCTCTGTTGGAAGAG

TTTAAAAGCAATAAAGCCAAGTGTTTTGAGCTCTCTGAAATTGCCGGACATGTTGTTGAA

TTCAGTGCCGATCAATATGGAAGCCGATTTATTCAACAGAAGCTTGAAACAGCAACTACA

GAAGAAAAAAACATGGTTTATCAGGAAATCATGCCACATGCTCTTGCTTTGATGACCGAT

GTCTTTGGTAATTATGTGGTTCAAAAGTTTTTTGAGCATGGACTTGCACCCCAGAGAAGA

GAATTAGCAAACAAGCTTATTGGCCATGTTCTTACACTTAGCCTTCAGATGTATGGTTGC

CGGGTTATTCAGAAGGCCATTGAAGTTGTTGATCTGGATCAGAAAATAGAGATGGTTAAA

GAGCTTGATGGTAATATCATGCGTTGTGTTCGTGATCAGAATGGTAATCATGTCATTCAA

AAATGTATTGAATGTGTCCCTGAAGATGCGATCGACTTTATTGTTTCAACATTTTTCGAT

CAAGTTGTGACACTCTCAACCCATCCATACGGTTGTCGTGTGATACAGAGAGTACTGGAG

CACTGTGAAAGCCCTGCAACACAGCAGAAAGTTATGGACGAGATTTTAGGCGCTGTTAGC

ATGCTAGCACAGGATCAGTATGGCAACTACGTTGTTCAGCATGTACTGGAACATGGGAAG

CCTCATGAGCGTTCTACCATAATAAAGGAGTTAGCAGGGAAGATAGTTCAAATGAGTCAA

CAAAAGTTTGCCTCCAATGTTGTGGAGAAGTGTCTGACCTTCAGTGGTCCTGCAGAACGC

CAAATACTAGTCAGTGAGATGCTTGGAACCACGGATGAAAATGAGCCTCTTCAGGCAATG

ATGAAAGATCAATTTGCAAATTATGTTGTCCAAAAGGTGCTGGAAACATGTGACGACCAT

CAACGTGAGTTGATTCTTTCGCGGATCAAGGTTCATTTGAACGCATTGAAAAAGTATACC

TATGGAAAGCACATCGTCGCACGTGTAGAGAAACTTGTTGCTGCTGGAGAGAGAAGAATG

GCTGCTCAGACTCCTCAACCTGCTTAG

>MS.gene004007.t1

ATGAATGTCTTCAACATATTTATACCTCCAGTAAAAGAAAGTGTTACTAGTTCATTTTCT

TCTAAAGAACTCCAAGAGGATAGTAAACCACTTTGGAGATTTGTCACTGTGGTGCAGAAT

AATGATTCTGCAGGTGGAAACAAGCTTTGGAGGTGCAACTTTTGTCAAAAAGAGGTGAAG

AGTTCATACTCTATAGTGAGATTTCACTTATTGAAAATTTCCGGTGAAGGAGTTGTGGTA

TGTCATAAGGTTACTGATATATTGTTAGCTGATTTGAATAGAATACGTGAAGAGCCTGTG

AATAAATTAAAGCTTAAACATGTTCTATTACCTACCCTTAAATCGTCTCAAATCCCACCA

ATAGTGCAACAAAAGAGAAGAAAGGGTAGTATAGAAAATGCCTTTAATGTTGATGATATG

AATCAGCATAGAGAAACTATAGCAAGGATGTTTTATTCTGGTGGCTTACCTTTCCATCTT

TCCAGAAATCTTTACTATGTTAGTTCTCATTCTTTTGCTGCTAATCACAACTTCAGTGGC

TTCCTTCCTCCTAGTTATAATGCTCTAAGAACCACTCTTTTGAAGCAAGAGAGAGCTAAA

TTTGAAAGGTGGCTTCAACCTATAAAATCTCCGTGGACATTGAAGGGAGTTACATTGGTT

TCTGATGCTAAGAACATTGATGGAAATGAAAATGTTTATAGTGAATGCAATTGGATCACT

GAAGTGGTTGATGATGTATCTTTTATCAAGACTTTCATTATGACTCACTTTATGTCATTG

GCTATTTTCAATGAATTTTCTACTTTGAAATTGCTTTCTATTGGTAAAACACGTTTTGCG

TCAATGATAGTGATGCTAAAAAGAATCAAGCTGCTCAAAACTGCTTTGCAAAGGATGGTT

ATTAGTGATCAGTGGAGTACTTACAGGGAAGATAATCAACAGAGCTACACATGTGAAGGA

GCTTCTTTTAAATGA
