# Supplementary material for: Identification of Genetic Loci Associated With Crude Protein Content and Fiber Composition in Alfalfa (Medicago sativa L.) Using QTL Mapping
Source: Front Plant Sci. 2021 Feb 18;12:608940. doi: 10.3389/fpls.2021.608940 (PMC7933732; doi:10.3389/fpls.2021.608940)
Supplement: Supplementary file 8 [file Table_6.docx]

### Table S6. Potential Candidate Genes of *qCP4C-2*

>MS.gene28694.t1

ATGAAAGAGCTTCTTCTTCTTCTTCTTCTTCTTCTGCTGTTGATTCTTTACTACCCTTCT

TGCCACTCACTTGACACCATAACACTGAACCAATCATTGAAAGACAACGATGTTTTGGTC

TCCAACCCTCGAGGAACCTTTGCACTTGGTTTCTTCACCCTACAGAAAGATTCTAAAACA

CGATATCTTGGTATATGGTACAACAAAATTTCAGAACAAACCATTGTTTGGGTTGCAAAC

AAAGACACTCCTTTGTACAACACTTCCGGTGTTGTCTCCATCAATGACGGAAACCTCGTA

CTCCACAACAATAACCTCACCAACAACAAAAACCTTAAACCAATTTGGTCTTCCAATGTT

TCTGTTTCACCTTCATTTGGCAATGTTTCGGCTAAGCTTTTAGACAATGGAAACTTTGTT

TTGACTCATAACAATGGAAAAAACATTGTGTGGCAGAGTTTTGATTTTCCAACGAACACT

CTTCTTCCTTTCATGAAACTTGGGTTGGACCGTAAAACCGGTTTGAACAGGTTCCTGACA

TCTTGGAAGTCTCCCAATGATCCAGGAACCGGGAATTTGACATACCGGATCGATCCAACC

GGGTTTCCTCAACTGTTTCTATACAGACACAATAAAGTTCCAGTATGGAGAACTGGGTCA

TGGACGGGTCAAAGATGGAGCGGGGTACCCGAAATGACTCCGACTTTTATCTTCAATGTT

AGTTTTGTTAACAACGTAGATGAAGTTTTTATAGAGTATGGTGTGAAAGATCCAAGGGTG

ATATCAAGAATGGTTTTAGAAGACTCGGGTCACGTAAGGAGGCTGACGTGGCAGCCAAAC

GAAAATAGATGGTTTCAAATATGGTTCGGCCCGAAAGAAGAATGTGATAATTTCAAGCAA

TGCGGGTTGAACTCGAATTGTGATCCATATAATGCGGAAAAATTTGAGTGTGAGTGTTTA

CCCGGATTTGAACCAAAGTTTGAGAGGGAATGGTATTTGAGAGATGGGTCGGGTGGGTGT

GTGAGGAAGTCGAATGTGTCCACTTGTCGAAATGGGGAAGGGTTTGTGAAAGTGGCACGT

GTGAAGGTGCCGAATACTTCGATGACACGTGTGAATGAGAGTTTGGGATTGAAGGAGTGT

AGAGAGATGTGTTTGGGGGATTGTTCTTGTGTTGCATTTACAAGTGAAAATGAGATGTTG

CAAAGTGGGTGTGTTACTTGGCATGGTGATATGGAGGATACAAGAACTTACACACAAGTA

GGACAAGATTTGTATGTACGTGTGGATAAGCATGAATTGGCTATGTATGCAAAACATCCG

TACGGTTCTCTTGGCAAAAAGGGAATGGTGGCACTTGTAGTCGTTGGTACTTGCTTGATA

TTGTTTATGGGGATCATTTTGGTGTATTGGTTTGTAAAAGCAAGGAAAAAATGGTCGAGA

AGGGATCGCAAGTTTTCCTTTCGTCTTAGCTTTGGTGACTCTGATCAGCAAGAATTTGAC

AGTGCAAACAATTCAAATTTACCATTCTTTGATCTTAGCTCCGTAGCTGCTGCAACTGAC

AGTTTTTCAATTGTTAACAAGCTCGGTGAAGGTGGTTTTGGTTCAGTTTATAAGGGTATA

TTGAGCAACGGAATGGAAATTGCAGTTAAGAGACTGTCAAAGCATTCCGGACAAGGCATA

GAAGAGTTTAAAAATGAGGTTGTTTTGATTTCAAAACTCCAACACAGAAATCTTGTGAGG

ATCTTAGGTTGCTGCGTTCAAGGAGAAGAGAAGATGTTAATCTATGAGTATTTGCCTAAC

AAAAGCTTAGACTTTTTCATTTTTGATAAATCTAAAAGTTCCGAGCTAGACTGGAGAAAG

CGGTTTGATATTATCTGTGGAGTCGCTAGAGGGATCTTATATCTTCATCACGACTCGCGA

TTAAGAATTATTCATAGAGACTTAAAGGCCAGCAATGTCTTGTTGGACATTGCATTGAAC

CCAAAAATTGCAGATTTTGGTATGGCTAGAATGTTTGGTGGAGATCAAGTTGAAGCAATT

ACAAATCGTGTTGTTGGAACCTATGGTTATATGTCTCCGGAGTATGCCATGGAAGGACAA

TTTTCAGTGAAGTCTGATGTATACAGCTTTGGAGTTTTGCTGCTAGAGATTATTACAGGC

AAAAAGAACAGTGGTCAATATGCAGACGATGCATCCACAAATTTAGTTGGACAT

>MS.gene28695.t1

ATGGCGACCAATCTCCCAGAACACTTCAAATGTCCAATCTCACTCGAAATAATGTCCGAC

CCAGTCATCCTCTCTTCCGGTCACACATTCGATCGTCCTTCAATCCAACGTTGGCTTGAC

GAAGGTCATCGTACTTGTCCAATTACCAAACTACCCCTTCCTGATTCTCCTATCCTCATC

CCGAACCACGCGCTTCGTAGTTTAATCTCTAGTTACACACTCCTTCCACCACTTCATCAA

ATCATTTCACAACCCGAAACCCTAATTTCAACCCTAATTTTGAATTCTTCTTCTTCCGAT

TCCAAAATTGACTCACTCCGTCAACTCGCTCGTCTTTCTAAACGTGATGCGAGTTTCCGT

CGCCGTCTTGTTGATTCCGGTGCCGTCTCCGCCGTGCTTTTCTGTCTTGATTCATCTTCC

TCTTCTTCTTCTTCCGGTAACGTTAAGCTGCAGGAGAAGGCTTTGTCTCTTCTTCTTAAT

CTCAGTCTTGATGATGATAGCAAAATTGGTCTTGTTGCAGAAGGAGCAATTGACCGGGTT

GTGAATTTTCTAGTCGGAGTAGCTTCATCAGACTGTCGTGCTTTGGCCGCGACGATAATC

ACGAGCCTTGCCGTGGTGGAGGTGAATAAAGCAACAATCGGAGCATTTCCTGGCGCAATT

GAAGCTCTGGTGATGATTCTTCGCGACGGGAAGGGGAGAGAGAGGAAGGAAGCTGCTACC

GCGCTTTACGCGCTTTGTTGTTTTAGGGATAATCGAAAAAGGGCTGTGGATTGTGGCGCG

GTGCCGATTTTGTTAAGAAATGTTGAATGTGGACTTGAGAGGGGTGTTGAGGTGATTGGT

GTTTTGGCAAAGTGTAAGGAAGGGAGAGAACAATTGGAGAGTTATGGTGGTTGTGTGAAG

ATTTTGGTGAGTGTTTTGAGGAATGGAAGTTCAAGAGGCGTTCAATATGCATTGTTGGCA

CTTACTTTGGTTTGTTTGCATAGCAAGGAAATACTTTTGATGACTCTTCAAGAAGGGGTT

TTGGAAATTTGTCTTGGGTTGGTGGAGGATGATAGTGAGAAAGTTAGGAGAAATGCGTCT

AATTTGATTCGGGTTCTTCGTGGAGGAAACCATCACCGGATCAGTTGA

>MS.gene28698.t1

ATGGAACAAGTAAATAACAATAGACTATGTGTGTCTATGGAAGAGTTGGGTTTCAATGCT

GAAAATGGTAGCAATAGGGGAAACTGGAGAAGGAACAAGCACCGTGATAATGATGGTGTC

CCAAAAGTGTTCACATCCAAGAACCTTGAGACAGAAAGAAAGAGAAGGGAGAAACTCACT

AGCAGACTTTTGGTGCTTAGGTCTTTAGTACCAATCATCACAAATATGAACAAAGCAAGC

ATTATTGAGGATGCTATCACTTATACCATGAAGTTGCAACATGAAGTTGAGAGTCTCACA

ATAGAGCTTGATGGAATGGAACCAAGAAGAGAGAAAAGAGTTGAGCCAAAACAAAGAGAG

TCTTCTGCTGTTGATGAGATGAATAAAATGGGGTTACAGGAAGAGGTGCAGGTAGCAAAA

GTTGATGGAAATAAGCTATGGATTAAGATGCTCATTGAGAAGAAAAGAGGAAGGTTCAAC

AAATTAATACAAGACTTGAATGATTTGAACATTGAAATGGTAGACCTCAGTGTTACTACT

ATGGCAGGAGCATATCTCATTACAGCCTCCTTGCAGGACATGGGTGGTGAACCACTTGAA

CTTTATCGAATTCCAACTATTATCAAGTATTAG

>MS.gene28697.t1

ATGTACGGACATGTTGAGAAGCTAGCTCAAGAGATTTTAAAAGGCGCGGCTTCCGTGGAA

GGAGTAGAAGCAAAGCTATGGCAGGTACCTGAAACTCTGCCTGCGGAGGTCGTTGGGAAG

ATGGGAGGACCTCCAAAGAGTGATGTGCCAATTATTACACCTGATGCGCTACCTGAGGCT

GATGGTTTGTTGCTTGGCTTCCCGACAAGATTTGGAATGATGGCTGCTCAGTTTAAAGCA

TTCCTCGATGCAACTGGTGGCCTATGGCGTACTCAGGCACTTGCAGGAAAGCCTGCAGGA

ATCTTTTACAGCACTGGTTCTCAAGGAGGAGGACAAGAGACTACACCGTTGACATCCATC

ACTCAGCTTGCTCACCATGGAATGATTTTTGTGCCCATTGGTTACACATTTGGTGCTGGT

ATGTTTGAGATGGAGAGCATAAAGGGTGGTTCCCCATATGGTTCGGGAACCTTCGCCGGA

GACGGCACGAGACAGCCTACCGAATTGGAATTGGCTCAAGCTTTCCATCAGGGGAAGTAC

TTTGCTGGCATTGCAAAGAAGCTCAAAGGATCTCAGTGA

>MS.gene28699.t1

ATGTCTCTGACACCTTCGTCTCTTGTTCCCATTTTCCTTTTGGTCGTTGTTTTCATTTTT

CCTCTCACAGGGATAGCTAAAGAATCTTCTCTTGGAGAGATTCTTCGAAACCATGGCCTT

CCAGCTGGTCTCTTTCCTCAGAGTGTGAAATCATTCAAATTGGATCAAATGGGACGTTTA

GAGGTACACTTGGATCGTCCTTGTTTGGCTCAATATGAAACCACTGTGTTCTTTGACACT

GTTGTAAAAGCCAACCTCAGTTTCGGACAACTTAAGGTTTTGGAGGGTATGTCTCGTGAA

GAGCTTTTCTTGTGGCTACCTGTTAAAGATATCATTGTTACTGATCCAAAATCTGGTGTA

ATTGTCATTGATATTGGTTATGCCTTCAAACGTCTCTCTTTCTCTCGTTTTGACGAACCT

CGCATTTGTAGATCTCATCATG

>MS.gene28696.t1

ATGGGAGGACCTCCAAAGAGTGATGTGCCAATTATTACACCTGATGCGCTACCTGAGGCT

GATGGTTTTTTGCTTGGCTTCCCGACAAGATTTGGAATGATGGCCGCTCAATTTAAAGCA

TTCTTGGATGCAACTGGTGGCCTATGGCGTACTCAGGCACTTGCAGGAAAGCCTGCAGGA

ATCTTTTACAGCACTGCTTCTCAAGGAGGAGGACAAGAGAAGAGACTACACCATGGAGAG

TATAAAGGGTGGTTCCCCGTATGGTTCCGGAACCTACGCTGGAGATGGCTCGAGACAGCC

TACCGAGTTGGAATTGGCTCAAGCTTTCCATCAGGGGAAGCATTTTGCTGGCATTGCAAA

GAAGCTCAAAGGATCTCAGTGATTTTTATCCTCTATACATAA

>MS.gene33849.t1

ATGGTTACACAAAGAGTTCATAGATACACTAAACCAGGCAGCTCAATGGGAACCGCAAAC

TTTCTCCGTAAATTATTTGTGGCTCTCAAGGTATCCAATCAGTTTTCTCAACCCGAAGTT

GTCTGGGAAAAGACTTGGGATAATCTCAGTGATGATGTTTTAAGCTGCCAGAGACGTGTT

CTACGCATGGAAGGACTTGGTCATGGATGA

>MS.gene33850.t1

ATGTCGCTGATTCCAAGTTTCTTTGGTGGTCGAAGGAGCAATGTTTTCGATCCATTCTCC

CTTGACGTTTGGGATCCCTTCAAGGATTTTCCATTCTCCAATTCTGCACTTTCTGCATCT

TCATTCCCTCAGGAGAATTCTGCGTTTGTGAGCACCAGGGTGGATTGGAAGGAGACCCCA

GAAGCTCATGTGTTCAAGGCTGATCTTCCAGGAATGAAGAAGGAAGAAGTGAAGGTTGAA

ATTGAAGATGACAGAGTTCTTCAGATTAGTGGAGAGAGGAACGTTGAGAAAGAAGACAAG

AACGATCAATGGCATCGCGTGGAGCGTAGCAGTGGGAAATTTATGAGGAGGTTTAGATTG

CCTGAGAATGCTAAAATGATCAAGTGA

>MS.gene33852.t1

ATGTCGCTGATTCCAAGTTTCTTTGGTGGTCGAAGGAGCAATGTTTTCGATCCATTCTCC

CTTGACGTTTGGGATCCCTTCAAGGATTTTCCATTCTCCAATTCTGCACTTTCTGCTTCT

TCATTCCCTCAGGAGAATTCTGCGTTTGTGAGCACCAGGGTGGATTGGAAGGAGACCCCA

GAAGCTCATGTGTTCAAGGCTGATCTTCCAGGAATGAAGAAGGAAGAAGTGAAGGTTGAA

ATTGAAGATGACAGAGTTCTTCAGATTAGTGGAGAGAGGAACGTTGAGAAAGAAGACAAG

AACGATCAATGGCATCGCGTGGAGCGTAGCAGTGGGAAATTTATGAGGAGGTTTAGATTG

CCTGAGAATGCTAAAATGGATCAAGTGAAAGCTGCAATGGAGAATGGTGTTCTCACTGTT

ACTGTTCCAAAAGAAGAAGTTAAGAAACATGATGTTAAGTCTATTGAGATCTCTGGTTGA

>MS.gene33853.t1

ATGATTAGGAAAGGTTGTCATCCAAATACTTATACTTGCAATACGTTGCTGAATAGCCTG

TGGAAAGAGGGGAGGAAGTCAGAGGCTGAGGAAATGCTGCAAAAGATGAACGAAAAGAGT

TATCAGTTAGATACCGTGACCTGTAATATCGTGGTCAATGGTCTGTGTAGAAATGGAGAA

TTGGAGAAAGCAAGTGAAGTTGTAAGTGAGATGTGGACTGATGGAACAAATTCCCTTGGT

AAAGAAAACCCGGTTGCTGGCCTAGTTAATTCAATCCATAATGTATCGACCAATGTACCT

GATGTGATCACTTATACAACCTTAATTAATGGACTTTGCAAGGTTGGGAAACTAGAGGAA

GCCAAAAAGAAATTTATTGAGATGATGGCGAAAAACTTACACCCTGATTCTGTGACCTAT

GATACGTTTGTATTGAATTTCTGCAAACAAGGGAAGATATCATCGGCTTTACGTGTATTG

AAAGACATGGAGAGAAATGGTTGCAGCAAGACTCTTCAAACTTATAATTCATTGATCTTG

GGGTTAGGAAGTAAAGGGCAAATATTTGAAATGTACGGATTGATGGATGAGATGAGAGAA

AGAGGAATACATCCAGATATTTGCACTTACAATAATATGATCAATTGTCTTTGCGAAGGA

GGAAAAACAAAGGATGCCACTTCTCTTTTACATGAAATGTTGGATAAGGGAGTCGTCTCT

CCCAATGTATCATCCTTCAAAATATTGATTAAAGCATTCTGCAAGTCTGAAGATTTTAAA

GTAGCATGTGAACTTTTTGATGTAGCTCTGAGTGTATGTGGCCACAAAGAAGCCTTGTAC

AGTTTGATGTTCAACGAATTACTTGCTGGGGGAAAACTCTCTGATGCAAAAGAGCTATTT

GAAGCTTCGTTAGAAAGATCTCTCTTATCAAAGAACTTTATGTATGAAGATTTGATTGAC

AAACTTTGCAAGGATGGAAGGTTAGACGATGCTCACGGCCTTCTTCAGAAATTGATTGAT

AAGGGATATTGCTTCGACCATTCATCATTCATACCTGTGATTGATGGCTTAAGTAAAAGG

GGAAACAAACAGCAAGCTGATGAACTAGCAAGGATAATGGAATTGGCTTTAGAAGAAGAT

AAAACTTTTGATAGGACGTACCGAAATGGAAACTCTATCTTTCGTCGAAAATTACACAAG

GATGGTGGAAGTGATTGGCAGGATATAATTAACAGGTATGACCATATGGTAGTGAAGATA

GTCTTTTCATTCCACTTTTGGGTTCTGTTCTTTTATAAAAGAGATGTTTTTAACTAG

>MS.gene33854.t1

AGATGGATCATCGATGATGCAACAGGACAAAGAATGAAACTAACCTGTGCACATTGGGTT

GCACATGCAACCCCAATGCTTGTTGAAGGTCTTGACAAATTGCCTATACAAGACATAGCT

AATCAAATTGCTAAGTCAGGTTTCAATTGTGTCCGTTTGTCTTATGCTACTTACATGTTC

ACACGACATGCAAATGATACAATTCGAGATACACTTTATAGTCTCGATATACCCAAGGAT

GTTGTGTCGGCTATTGAGAAACATAACCCTTTAATGTTGAACATGACTCATGTTCAAGCT

TATGAAGCTGCCATTGATGCTCTTGGTGAAAAAGGTGTTATGGTTCTTATTGATAATCAT

GTTAGTATGCCAGATTGGTGTTGTGATAATAATGATCAGAATGGATTCTTTGGTGATAGG

CATTTTCATCCTGATGAATGGCTTCAAGGTTTGGCTATTATAGCTAAGCACTTCAAGGGA

AAACCCAATGTGATAGCCATGGACTTGCGGAATGAGCTAAGAGGTGGACGTCAAAATTTG

CCCGATTGGTACAAGTATGTAACCCAAGGAGCAAGCACAATTCACAAACACAACCCAGAT

TTATTAATAGTTATTTCAGGGTTTAACTTTGACAATGACCTTTCATTTTTGAAGAAAAAG

ACCCTTGATCTAAACTTCACCAACAAATTAGTGTACGAGGCACACATTTATTCCTTCTCA

GGAAACCAAGATAGGTGGAAGTTGCAACCAATGAATTGGGTTTGTTCCTCTGTCATTGAA

ACCTTAAACGACCAAGCTGGTTTTCTTATTAGTGGTAACAATCCAGTACCTTTATTCATA

AGTGAATTTGGATATGACATGACTGGTGGCAATGCCGTGGACAACAAATTCATGCCATGC

TTTGTGTCTTATGCCGTCTCTAATGACTTGGATTGGAGCTTGTGGTCTTTTGGTGGAAGC

TACTATTTTAGACAAGGCAATGTTGGGGCTGGTGAGACATATGCTGTAATGGACTATGAT

TGGAAAAATTACAGGGACCCAAATTTCCCTCAGAAATTTCAGCTTCTCCAGAAGAAAATT

CAAGATCCAACTTCAAACCTCTCAAAGTCTCATATAATGTTCCACCCATTGACCGGTAAG

TGCGCTCACGTGAATGAGAGTAACAATGAACTTGTATTGGGAGATTGTAAGAGTCATAGC

CAATGGAGTTCTGAGGGAGATGGTTCTCCAATAAGGTTGATGGACTCTGCTAAGTGTCTA

AAGGCAGAGGGTGAAGGGCTTCCTGCAACCCTCTCAGAACATTGCTTGTTGCCGCAAAGT

TCCTGGAAATCTGTTTCAAAGACTGGTCTTCACTTGGCCACCTCTCATGGGAACGGATCC

CTTTTGTGCTTGGAAATGGATTCAGATTCCTCCAAGATAGTGACCAGAAAATGCATCTGC

ATAGATGATAATGATTCTTCATGTTTGGACAACCCCCAAAGCCAGTGGTTCAAACTTATT

TCAACCAATGTTTAG

>MS.gene33851.t1

GGTTAGTTAACAGGTCAAAGTTCGTCCACGTGTCACGTTTTAATTTGAAGAGCTGCTGTT

ACCGCAATAAATCCGCAATTGATTTTTGCTATAAAATCAAATCAGGCAAACCCTTCTTCA

TTCCCAATCCCATCGCTACAAAACCAATTCCTTTCGTTGAAGAGAAAAGAGATTCAATCA

AACCTAGCACCCAATTCCGATTCAAGATGCAAATATTCGTTAAGACCCTCACTGGCAAGA

CCATCACTCTCGAAGTTGAGAGTTCAGATACCATAGACAATGTTAAGGCAAAGATTCAAG

ACAAGGAAGGAATCCCACCAGACCAGCAGCGTCTGATTTTTGCTGGAAAGCAGCTGGAGG

ATGGGCGTACCCTTGCTGATTATAACATCCAAAAGGAGTCCACTCTCCATTTGGTGCTTC

GTCTCCGTGGTGGTATGCAAATATTTGTGAAGACTCTCACTGGAAAGACAATCACCCTTG

AGGTGGAGAGCTCTGATACCATTGACAATGTTAAGGCCAAGATTCAAGACAAAGAAGGTA

TTCCACCAGACCAGCAGAGGCTCATCTTTGCTGGAAAACAGCTTGAAGATGGCAGAACCT

TGGCTGATTACAACATCCAGAAGGAGTCAACTCTCCATCTTGTGTTGCGGCTTCGTGGTG

GTATGCAGATTTTTGTAAAGACCCTTACAGGAAAGACAATTACTTTGGAGGTAGAGAGCT

CGGACACAATTGACAATGTGAAGGCCAAGATACAAGACAAGGAGGGTATCCCCCCAGACC

AGCAAAGGTTGATTTTTGCTGGAAAGCAACTCGAAGATGGTCGAACTCTGGCTGATTATA

ACATCCAGAAAGAGTCGACTCTTCATCTTGTCTTGCGACTTCGTGGTGGTATGCAGATTT

TTGTTAAGACCCTAACAGGCAAAACAATTACTTTGGAGGTGGAGAGCTCAGACACAATTG

ACAATGTAAAGGCTAAGATTCAGGACAAGGAGGGCATTCCCCCAGACCAGCAAAGGTTGA

TTTTTGCTGGAAAGCAGCTTGAAGATGGCAGAACCTTGGCAGATTATAACATCCAGAAGG

AATCCACCCTTCATCTTGTCCTTCGTCTGAGGGGAGGCATGCAGATCTTTGTGAAGACTT

TGACAGGAAAGACCATTACATTGGAGGTGGAAAGCTCTGACACCATCGATAATGTTAAGG

CAAAAATACAGGATAAGGAGGGAATCCCACCAGATCAGCAGAGGTTGATCTTTGCTGGGA

AGCAGCTTGAAGACGGTCGGACTTTGGCTGATTATAATATTCAGAAAGAGTCCACCCTTC

ACCTTGTCCTTCGTCTGAGGGGAGGTATGCAAATCTTTGTGAAGACGTTGACCGGAAAGA

CCATCACCTTAGAGGTGGAAAGTTCAGACACTATTGATAATGTCAAGGCAAAAATCCAGG

ACAAGGAGGGAATCCCCCCAGATCAGCAGAGGTTGATCTTTGCTGGGAAGCAATTGGAGG

ATGGAAGGACCCTTGCTGATTACAACATTCAGAAAGAGTCTACTCTTCACCTTGTTTTGC

GTCTTCGTGGTGGATTTTAA

>MS.gene40088.t1

ATAAGGCATTTGTGCAGAATGCGATGAATATAACTAGGCTCTTGATCAATCCTGACATTC

TACAGGCTCTTCAACTGAAGAATGGGGTGGGCTTTGATGGAGTTGAGACATCCAGGACTG

TTCCCAAACTTGGTCCCCGTGTAAGGTCGTCTTTTGATGAAGTGTTTTTGCGATTGTATG

CAAAGAAAACCATTGCTCAGCTGACTGATATAGCTGACGCTGGAACTTTTATTGTAAGTG

CAACTGTTAATGAGAGTTATGAATATGACTGTGGTTGGGTGGTTGATTGA

>MS.gene40087.t1

ATGGCATCGGCTTTATTAGACAACCGTAACGAACCAAATTGGCTGCAACACAGAGGCGGT

GGAGCTGAATTCATGGGTAAAGCACCTAACCCTAACCCTAAATTTAGTAACAAAAAGAGA

ACTCAATCCCCATCAGATGACGCTTCCTCAATCAATCGACGATCCAACGATAATCATTCA

CAATACGTTACTTTCAACATCGAATCGTACACAAAGACGGAGCTATACGAGCTCAAAAAT

CGACTCGTATCAGAACTCGACCAAATTCGTCAGCTTAAGACTCGAATTGAATCAGGTGAG

TTTAAACCCAGGCTAAACCACAACGGTGGTGGTCCCACTAAGAAATCAGGGAGCAAGAAG

GTTTCCGGCAACAAACGACCTTTTCCTGCGGTGAAGGAATTGAAGAAATCAAAGTCAGAG

ATTGGGGATGCGATGAAGGCTTGTGGTCAGATTTTGACGAAGCTCATGAAAAACAAAAGT

GGTTGGATTTTTAACACTTGGATCTTTAACACTCCTGTAAATGCAACGGCTTTGAATCTT

CATGATTACTTTGATATAATTAAGCATCCTATGGATCTGGGTACTGTCAAGTCAAAACTT

GCTAAGAATGCTTACTCTACGCCGGCGGATTTTGCTGCTGATGTGAAGTTAACGTTCAAC

AATGCATTGACATACAATCCCAAGGGCCATGATGTTAACACTGCGGCGAAGCAGCTTCTT

GAAAAATTCGAGGAGCTTTATCGACCAATACACGAGAAATTTGATGAAAAAAGTTTTGAT

GATGAATTACAGGCTAGCTCGTGGAACCATGTTGAACCAGAGAGGGAAAGGGAGAGGGTG

AAGAAGAAAGATAATCCAATTCCAATCCCTCCACCGGTAGCAAAACGCCAAGAGCCACTG

CCTGAGCCTGCTAGTACTTCTAACCAGCCAAGCACTTCGAATCCCCCATTAGCTCAGTCA

CCAGTTCGCATACCCTCCCCAACTCGAGCACTTCCAGTGAAGCCCTTGAAGCAGCCCAAG

CCAAAGGCCAGGGATCCAAATAAGAGGGAAATGAATGTTGAGGAAAAACATAAGTTGGGA

CTAGGGTTGCAGATTTTGCCACCAGAGAAGATGGAACAAGTAGTACAGATCATAAGGAAG

AGAAATGGGCATTTGGAGCAAGATGGGGATGAGATTGAGCTTGATATGGAGGCTGTTGAC

ACGGAGACTCTCTGGGAACTCGATCGATTGGTTACCAATTGGAAAAAGATGGTGAGCAAG

ATTAAGCGGCAGGCGCTAATGGATAATAATAATGTGCCTTCAAACAAAGGCAATGGGGAA

TTGCCTGATAGGGAGAAGGTTGATGCAACCCCTCCTTCTGAGGGGAAGAAGCAGAAGAAG

ATAGATACTGTTGACGAGGATGTTGACATCGGCGATGATATGCCAGCAAACAACTTCCCA

CCTGTGGAGATTGAGAAAGATAAAGATATGGGTGCTACTGGTGGCGGTGCCAGTAGTAGT

TCCAGTGGCTCCAGCAGTTCTGGTAGTGATTCCTCGTCTAGTGATTCGGATTCAGGGAGT

TCTTCAGGAAGTGACTCTGAAGCGGGCAATGGTCATTTGTAG

>MS.gene40082.t1

ATGTCGAGAATGTCTGAGTCTAACGAAGAAAGCATGATTCTAGAAAAGATGAATAAAAGA

AGAAAATGTGAGAATCAAAGTAATGAAGAAAGTGAAGACAGGCTTAGTGACTTACCCGAC

GGTGTTATCCTTCACATTTTGTCATTTTTGAATACCAAACATGTCGTTCGAACTTGTCTT

TTGTCCAAGAGATGGGAACATCTCTGGAAACGTATTCCAACTCTTATGTTGTATGCATCA

AGATTTTCCACTGTTAAGCAATTTGCCATGTTTGTGTCTAAGATTTTGACTCTTCGTGAT

ACCTCAACTGCGCTGCACGCTCTTGATCTTGACCGTCATGGTGATATTGAGCCTCAACTC

CTTAAAAAGATTTTAAACTATGTTTCATCTCATAATACCCACCTCCATGAATTAGGAATC

ACTGTTAATGGTGATACTAGTCTCATTATGAGCTGTGTTTCTTCATGCCGGGCTCTTACA

TCTCTTAAGCTTGATGTTGCCTCTAGAGGTCGTCATAATATTGGACAAACATTATTTCCA

AAATCTTTGAATTTGCCAACATTGACCAGCTTGGAACTTATATGCATCACCTTTTGCGGC

GGTGAAAGCAGTTGTGCTGAGCCCTTTTCAGCCTTTAACAGGTTGAATAGTTTGGTCATT

TCTTATTGTACAGTTAAGGATGCACAAATCCTCAAGATATCAAGTGAGACACTTGCCAAT

TCAGCTATGCATCATAATTCCTCTAACTTTGCCCAAATCGAGTTATCTGCTCCTAGGCTC

TGTACCTTTACTATTACTGGTATGCCTATACACAAAATATGCGGCAGTGGTCTTTCTTCT

GTTAAACAAATAAATATTGCTGCACAAATTTATTCAAATTCGGAGAAACCTCCTATGGTT

CTATTGAGTTGGCTGCTAGACCTTGCCAATGTAAAATCAATGACAGTCTCTTCAACTACT

CTTCAGATTCTCTCCTTAGTTCCTGATTTATTAGAGGTTAAGATCCATTCTTTGTGTAAC

TTGAAGTCGATGGAAATAAAATTGGAACCACTTGAAGCTGAATTCGGATTACCCTACATA

GTGAAAAATGCCATGTTAAAGAAAGCTGCTGCCAACTCACGCAAAGAAGTTGCTAAGTTA

AGAAAGGCGTTTAAAGCAGGTTTGAAACCGCCTCTCATACCAGATGGAATAGTTGACTTC

TTGCGACAAAACTCACCATCGGTGGAAATTAACATCACAACAAAGCACATGAGTGATTTT

AATATTAAGCAGGTTGTAGAATCTATAAAGGACGCAAATATTATCAACTACAGCTCACAG

TTTACCGCGCCTGCCACTACCGCGCCTGCTTCGGCTGCTGAGTCTGCTTCTGCCACTGCG

CCTGCCACTGCTGTACTTCCAAATCTTCTTCTCTGTCACGCTGAAAAGGACAACAAATCA

TCAATTGAAGATAAGGCGAAGAAGCACTGA

>MS.gene40086.t1

ATGGCCGCCGTCTTGCGCCGTGCTCTCCGCCGCGATCTTGCTTCTTCCTCTGTCTCTGCC

TTTCGATCGTTGACAGGCAGCACTAAGCCATCATATGCAGCTCAAAAGTGGGCAAGTTTG

GCTCGACCTTTCAGTTCTAGGCCTGCTGGTAGTGATGTTATTGGAATTGATTTGGGGACT

ACCAATTCATGTGTTTCTTTAATGGAGGGAAAGAATCCTAAAGTTATTGAGAATTCTGAA

GGAGCTCGGACAACACCATCAGTGGTTGCCTTCAACCAGAAAGGAGAGCTGCTTGTTGGT

ACACCTGCCAAGCGTCAGGCTGTAACCAACCCAACAAATACTCTTTTTGGCACCAAACGT

TTGATTGGTAGGCGCTTTGATGATCCGCAAACACAAAAGGAGATGAAAATGGTACCCTAT

AAGATTGTCAAGGCACCCAATGGAGATGCATGGGTTGAAATCAATAAGCAGCAGTATTCT

CCCAGCCAAATTGGTGCTTTTGTTCTTACTAAGATGAAGGAAACTGCTGAGGCATACCTT

GGAAAAACAATTTCTAAAGCTGTAGTTACTGTTCCAGCTTACTTCAATGATGCTCAGAGG

CAGGCAACAAAAGATGCTGGTAGAATTGCCGGTCTTGAAGTGCTGAGAATTATCAATGAG

CCCACTGCAGCTGCACTTTCATATGGGATGAACAGCAAAGAGGGGCTCATTGCTGTTTTT

GATCTCGGAGGTGGAACATTTGATGTGTCCATTTTAGAAATTTCAAACGGTGTATTTGAG

GTGAAAGCTACCAATGGTGACACTTTCTTGGGAGGAGAAGATTTTGATAATGCTTTGTTG

GACTTTCTAGTAAGTGAATTCAAGAGAACTGATAGTATTGATCTTGCAAAGGATAAGCTT

GCGTTGCAGAGGCTTCGAGAAGCTGCTGAGAAAGCAAAAATAGAACTCTCTTCAACTTCA

CAAACTGAAATAAACCTTCCTTTCATCACTGCTGATGCATCCGGTGCAAAGCATTTGAAT

ATCACGTTGACTAGATCGAAGTTTGAGGCTTTGGTAAATAACCTGATTGAAAGGACAAAG

GCACCCTGTAAGAGCTGCTTGAAAGATGCCAACATATCTATCAAGGATGTTGATGAGGTT

CTTCTCGTCGGTGGGATGACTCGTGTACCTAAAGTGCAGGAGGTGGTTTCAGAGATCTTT

GGAAAGAGTCCTAGCAAAGGAGTAAATCCTGATGAGGCTGTTGCAATGGGAGCAGCTCTT

CAGGGTGGTATTCTCCGTGGTGATGTTAAAGAGCTGCTACTCCTAGATGTCACTCCTCTA

TCCCTGGGTATTGAGACTTTGGGTGGTATCTTTACAAGATTGATCTCCCGCAACACAACA

ATTCCTACAAAGAAGAGTCAGGTGTTTTCGACAGCAGCTGACAATCAGACTCAAGTGGGT

ATCAAGGTGCTCCAAGGGGAGCGTGAAATGGCTGCAGACAACAAGAGTCTTGGAGAATTT

GACCTTGTTGGCATTCCTCCTGCCCCAAGAGGTTTGCCTCAGATTGAAGTCACTTTTGAC

ATAGATGCCAATGGGATTGTTACTGTCTCTGCCAAAGACAAATCCACTGGAAAAGAACAA

CAGATTACCATCCGCTCATCAGGAGGACTCTCAGATGATGAAATTAATAATATGGTCAAA

GAAGCTGAGTTGCATGCTCAGAGAGATCAGGAAAGGAAAGCACTTATCGATATCAAGAAT

AGTGCCGACACATCAATCTACTCGATTGAAAAGAGTTTGAGCGAGTATAGGGAAAAGATT

CCTAGTGAGGTTGCGAAGGAAATTGAAGATGCGGTCTCAGATTTGAGAACGGCTATAGCA

GGAGAAAGTGTTGATGAAATTAAGACAAAACTTGACGCTGCAAACAAAGCCGTGTCAAAG

ATTGGTCAGCATATGTCAGGTGGGTCAAGTGGAGGTTCCTCTGATGGAGGTTCTCCAGGT

GGTGGTGAACAGGCTCCAGAGGCAGAGTATGAAGAGGTCAAGAAGTGA

>MS.gene40084.t1

ATGAAATGGTGTTTGCTTAGAAAATGGGGGACGAAGATCCAATTCTCATTCTCTCCGTGC

GTTGGAATTGTGTATAGGAGCTTACGTTCAGATGCAGCATTGGAAGCCATAGCCAAAGCT

TCAGAAGACAAAGTTCCCAATATAGTTCTCTACAATTACCCTTCATTTTCTGGAGCTTTC

TCTTCTCTCTTCGCTCACCTCTTCCACACTCGTCATAATCTCCCTTCCCTTTCTTTACCT

TTCTCTTCTGTTCCTTCCCTCGCTTTCAGGGTTGAAGATTTGTGCATTGAAAGCCTTCAA

ACATGTTATCTACTCGACTTTCTTCCTCCAAAGGAATTCCTTTTCAAACTTTCCCATCAA

TCAAATTGCAAGATTATTGGGTTTGACCATCGAAAATCGGTGCTTTCACAGATTCCTTCT

GCAAATGAGTGTCCCGGGAATATTATGATTAATGTTAACCATGAAAAGAGTAGTTCAAGA

GCTGTTTATGAGTACTTTACCGACAAACACGAGGATATTAAAACTTCTAATGGTGTGGTT

CCAAGTTTGGTGGACTCAAAAGATAAAGGGCGAGTGGAGCTTATTCTTAAGTATATTGAG

GATGCAGATCTTCGCCGATGGAGCTTGCCTGACATTAAACCCTTTAACATTGGACTGAGT

GAATGGCGATCGAGGTTTAGCTGCATTTCTAACCCATACATGTTTAAGCAGTTGCTGGAA

TTGAGTGTTGAAGAGTTAATTGCTAAAGGAAATTCATCTCTTTCAGCGCGCCGGAATGCT

GCAAGTAAATTGCTGGATAAGGTTTTCAGGGTTCGGCTGGGTAGAGGATTTTATGGAGAG

TGCCTGGGAGTTCGGGCAGATGGGAACTCTAACTTAAGTGATGAAATGGGCATGCTTCTC

AGCGTAAAAAGTGCTGCTATTGGTCTGAGGCCCATAGGAGCTGTGATATTCATGCAACGG

AATAATCTCAAAATGTGCTTGCGCAGCTCTGATAGTGCTACTGATACCTCTGAAGTTGCT

AAGGCATATGGCGGAGGAGGTTCTGCAAGTTCAAGCTCCTTCATAATAAGGATGGATGAG

TATAACCAATGGCTTTCAGCTAATTCATTATGA

>MS.gene40083.t1

ATGAAGAAAAGAAGGCAATGTGAGAATGATGAAAACGAAGACAGACTGAGTGACTTACCT

GATGGTGTTATCCTTCACATTTTGTCATTCTTGAATACCAAACATGCCGTCGGAACTTGT

GTTTTGTCCAAGAGATGGGAACATCTCTGGAGGCGAATTCCAACTCTTATGTTGCATTCT

TCAAGATTTTCCACTATTAAGCAATTTACAACATTTGTGTCTAAGATTTTGACTCTCCAC

GATACTTCAACCGCACTGCACAAACTTGATCTTGACCGCCGTGGTGATATTGAGCCTCAA

CTCCTTAAAAAGATTTTAAACTATGTTACTTCCCATAATACCCACCTCCATGAATTAGGA

ATCTCTGTCCGTGGTGACAGTAGTCTCATTATGAGATGCGTTTCTTCATGTCATGCCCTT

ACATCTCTTAAGCTTTCGCTTTACCCTAGAGGTAGCACTCATATTCATAATCATACAAAA

ACATTATTTCCAAAATCTTTGAATTTCCCTTTCTTGACCACCTTGTATCTAGAAAACTTT

GCCTTTTGCGGTAGTGAAAACGGTTGTGCTGAGCCCTTTTCCGCCTTTACCAAGTTGAAT

AGTTTGGTCATTAGTAGTTGTGAGGTAAAGGATGCTCAAATCCTCAACATATCAAGTGAG

ACACTTGTCAATTTAGCCCTGCACGATAATTTACTTGACTTTGCCAAAATCGAGTTATCT

GCTCCAAGTCTTTGTACCTTTACTTTTACCGGTGGCCTTCTTCAGAAAATATATGGGAGC

AGTCTCTCTTCTATTAAACAAGTAGATATCGATGCACAAGAGGTGTTATATTCGGAGGAT

TCTGCTATGGTTCTATTAAGCTTGCTGCAAGACCTTGCCAATGTAGAATCATTGGCAGTC

ACTTCAACTACTCTTCAGATTCTGTCCTTAGTTCCTGATTTATTGGAGGTTAAATTCTAT

TCTTTATGTAACTTGAAGTCATTGGAAGTAGAGTTGATACCACTTCAGGATGGATATTTA

TCCCAATCAATCAAAAATGTCATGTATAATAAAGCTGCTGCCAAGTCACATGAAGAACTT

CTTAAGTTATGCAAGGCATTTAAAAGACGTATGGAACTACCTGCCATACCTGATGGAATA

GTTGACTTCTTGCGACAAAACGCGCCCTCGGCAGAAGTTAACATCACAACAGATTACTTG

ATTTATTTTAATCTTAAGCAGATTGTAGAATCTATAAAGGGTGCGAAGAGTAACCATTGT

TCACAATATGCCGTGTCTCCTTCCTCCTCTGCCGCACCTGATTTCGCTGCTGAGTCTACT

TCTGCCGCTAGCGCCTGCCACTGCTGCACCTCCCAATCTTTATCTCTGTAG

>MS.gene40085.t1

ATGGAGAACAACAACATAGACACCGAAAAAGGAACCATGAAGGAAGAAGAAGAAGAAGAG

TCACCAATTGAGGAAGTCCGGTTAACGGTGACAAAAACCGACGACCCAACACTTCCAGTA

TGGACCTTCCGCATGTGGTTTCTAGGTCTTCTCTCATGCGCTCTCCTCTCCTTTCTCAAC

CAATTCTTCGCATATCGATCCGAACCACTCATCATAACTCAAATCACCGTTCAAGTCGCC

ACACTCCCCCTCGGCCACCTCATGGCCGCCGTGTTACCTTCAAAGACGTTCAGGATCCCA

GGTTTTGGATCGAAACGGTTTTCGTTTAACCCTGGTCCGTTTAACATGAAGGAACATGTT

CTTATTACCATATTTGCAAATGCTGGGGCTGCGTTTGGGTCTGGTTCATCTTATGCTGTT

GGTATTGTGAATATTATCAAAGCGTTTTATGGAAGGAATATATCGTTTCTTGCTTCATGG

CTTCTGATTATTACGACTCAGGTGTTGGGATATGGATGGGCTGGTTTGCTTAGGAAGTAT

GTTGTTGAACCTGCTCATATGTGGTGGCCTAGTACCCTTGTTCAAGTCTCTCTTTTCCGA

ACTTTGCATGAGAAAGACGATAATCCCCATCAATTTTCAAGGGCAAAGTTTTTCTTCATA

GCACTAGTATGCAGTTTTACATGGTATATAGTCCCAGGATACTTATTCACAACGCTTACA

AGCATATCATGGGTATGTTGGGTATTCTCCAAATCAGTCACAGCTCAACAAATAGGTTCA

GGAATGAATGGTTTAGGACTTGGAGCCTTAACACTTGATTGGTCTGCTGTTGCTTCATTC

TTGTTCAGTCCTCTTATCTCACCTTTCTTTGCCATTGTCAATGTTTTTGTTGGCTATGCA

TTGCTTGTGTATGCTGTGATTCCTATAGCATATTGGGGACTCAATGTGTATGGTGCAAAT

AGGTTTTCTATTTTCTCCTCTGATCTGTACACAGCACAGGGACAACCATATAACATATCT

AATATTGTTAATGACAAATTTGAGATTGATCTTGCAAAGTATCACGAACAAGGTCGAATT

CATTTAAGCACGTTTTTCGCTCTTAGTTATGGATTTGGATTCGCCACCATAGCATCCACC

GTTACACATGTTGCTTGCTTCTATGGAAGGGAGATTATGGAGCGGTATCGTGCTTCGAAA

AACGGAAAAGAAGATATTCACACAAAACTGATGAAAAATTACAAAGACATTCCTTCTTGG

TGGTTTTATTTGTTGCTAGGAGTGACATTTGTTGTTTCTTTAATGATCTGCATCTTTCTA

AATGATCAGATTCAGATGCCATGGTGGGGACTTCTCATTGCTTCAGCATTGGCCTTTATA

TTCACCCTCCCAATTAGTATCATAACTGCTACCACAAACCAGACACCGGGGTTGAATATC

ATCACTGAGTATATCTTCGGTATCATTTACCCTGGAAGACCAATAGCAAATGTATGCTTC

AAAACCTATGGTTACATAAGCATGGCTCAGGCTGTCTCATTCCTTAGCGATTTCAAGCTT

GGACACTACATGAAAATCCCTCCAAGATCAATGTTCTTAGTGCAGTTCATTGGGACAGTG

CTTGCTGGAACTATCAACATTGGTGTAGCATGGTGGTTGCTAGACTCCGTCAAAAACATA

TGTAATAAAGATCTTCTTCCCGAAGGCAGTCCATGGACATGTCCCAATGATCGTGTTTTC

TTCGATGCATCGGTTGTTTGGGGTTTAGTAGGACCTAAGAGGATCTTTGGTTCTCTTGGA

GAATACTCAACATTGAACTGGTTTTTCCTTGGAGGTGCAATAGGGCCAATACTAGTTTGG

CTATTGCACAAGGCATTCCCAAAACAGTCATGGATTCCGTTGATCAATCTTCCAGTACTC

CTGGGAGCAACAGGAATGATGCCACCAGCAACAGCATTGAACTACAATTCATGGATTATA

GTTGGAACCATTTTCAACTTCTTTATCTTCCGTTACAGAAAGAAATGGTGGCAGAGATAC

AACTATGTTTTATCAGCAGCACTTGATGCAGGGGTGGCCTTTATGGCTGTACTGCTTTAC

TTGGCATTAGGCCTGGAAAATGTGAGTCTCAATTGGTGGGGAACTGCTGGGGAGCATTGT

CCTTTGGCAGGTTGCCCAACAGCTAAGGGTATAGCGGTTGATGGTTGCCCTGTCTTCTGA

>MS.gene40090.t1

ATGGCATCGGCTTTATTAGACAACCGTAACGAACCAAATTGGCTGCAACACAGAGGCGGT

GGAGCTGAATTCATGGGTAAAGCACCTAACCCTAACCCTAAATTTAGTAACAAAAAGAGA

ACTCAATCCCCATCAGATGACGCTTCCTCAATCAATCGACGATCCAACGATAATCATTCA

CAATACGTTACTTTCAACATCGAATCGTACACAAAGACGGAGCTATACGAGCTCAAAAAT

CGACTCGTATCAGAACTCGACCAAATTCGTCAGCTTAAGACTCGAATTGAATCAGGTGAG

TTTAAACCCAGGCTAAACCACAACGGTGGTGGTCCCACTAAGAAATCAGGGAGCAAGAAG

GTTTCCGGCAACAAACGACCTTTTCCTGCGGTGAAGGAATTGAAGAAATCAAAGTCAGAG

ATTGGGGATGCGATGAAGGCTTGTGGTCAGATTTTGACGAAGCTCATGAAAAACAAAAGT

GGTTGGATTTTTTAA

>MS.gene40091.t1

GTGGAGCAGTTCCTAAAAGCATTCAAGTTGAAGAATTTTTATTTAGATAAGGCATTTGTG

CAGAATGCGATGAATATAACTAGGCTCTTGATCAATCCTGACATTCTACAGGCTCTTCAA

CTGAAGAATGGGGTGGGCTTTGATGGAGTTGAGACATCCAGGACTGTTCCCAAACTTGGT

CCCCGTGTAAGGTCGTCTTTTGATGAAGTGTTTTTGCGATTGTATGCAAAGAAAACCATT

GCTCAGCTGACTGATATAGCTGACGCTGGAACTTTTATT

>MS.gene40089.t1

ATGGATCTGGGTACTGTCAAGTCAAAACTTGCTAAGAATGCTTACTCTACGCCGGCGGAT

TTTGCTGCTGATGTGAAGTTAACGTTCAACAATGCATTGACATACAATCCCAAGGGCCAT

GATGTTAACACTGCGGCGAAGCAGCTTCTTGAAAAATTCGAGGAGCTTTATCGACCAATA

CACGAGAAATTTGATGAAAAAAGTTTTGATGATGAATTACAGGCTAGCTCGTGGAACCAT

GTTGAACCAGAGAGGGAAAGGGAGAGGGTGAAGAAGAAAGATAATCCAATTCCAATCCCT

CCACCGGTAGCAAAACGCCAAGAGCCACTGCCTGAGCCTGCTAGTACTTCTAACCAGCCA

AGCACTTCGAATCCCCCATTAGCTCAGTCACCAGTTCGCATACCCTCCCCAACTCGAGCA

CTTCCAGTGAAGCCCTTGAAGCAGCCCAAGCCAAAGGCCAGGGATCCAAATAAGAGGGAA

ATGAATGTTGAGGAAAAACATAAGTTGGGACTAGGGTTGCAGATTTTGCCACCAGAGAAG

ATGGAACAAGTAGTACAGATCATAAGGAAGAGAAATGGGCATTTGGAGCAAGATGGGGAT

GAGATTGAGCTTGATATGGAGGCTGTTGACACGGAGACTCTCTGGGAACTCGATCGATTG

GTTACCAATTGGAAAAAGATGGTGAGCAAGATTAAGCGGCAGGCGCTAATGGATAATAAT

AATGTGCCTTCAAACAAAGGCAATGGG

>MS.gene40107.t1

ATGGAGAATATTTCATTTATCAGAGGGTTTCCTGAGCAGGAAATGATGGAGGATCCTTCA

TTTCTTCATCGTCAGTGGCATTTGAGCTCTATCAATGAGTCTAACTCACTGCCAATAGGT

ACTGCTTTTGGAGACACTTCACAACACCATTCATACGTTTATCCAAACTTCAACCCTAGA

ACTTCAATGGAAACTGCTCAGACACTAGAGACTCAATTTGTTCCGTACCCGAATCTTCTT

TCATTTGTCGATTTGAATCAGTTAAATCAGTTGGGATTGGTGAAGCCTAAGGATGAGATG

ATTGGTTCTCAAAACAACAACACAACTTCTGACATGATTTCTCAAGGAACCTTTGAGGCC

AAAAAGGTAGCAACACGTCCTAAGCTCTCTCTTCCTCAAGACCATATAGTAGCTGAAAGA

AAGCGGCGTGAGAAGCTCAGCCAGCGCTTCATTGCTCTATCTGCCCTTGTTCCTGGACTA

CAAAAGATGGACAAAGTTACTGTTCTTGGAGATGCTATCAAGTACTTAAAGAAATTGCAA

GAGAAGGTGAAGGTTCTTGAGGAGGAACAGAACATGAAGAAAAACGTGGAATTTGTGGTG

GTTGTGAAGAAATATCAACTATCCAATGATGTTGAAAACTCTTCTGCAGAATCTGGTGGT

CCCTTTGACGAGGAACTGCCAGAAATTGAAGCAAGATTTTGTGATAGAAATGTCCTCATA

AGAGTTCACTGTGAGAAAATAAAAGGAGTTGTGGAAAAAACAATCCATGAAATCGAGAAA

CTCAATCTAAAAGTCACCAATAGCAGTTTCATGACATTTGGGAGTTGTGCACTTGATATA

ACAATTATTGCACAGATGGATATGGAATTTTGCATGACGGTGAAGGATCTTGTGAGAAAC

CTACGCTCAGTTTTTACGTCTTTCATCTAA

>MS.gene40101.t1

ATGCTAAATGGATATGACCTGACGAAACAAAAGAGTCCCGGTTATTCTCGTGCTGTTTCG

CGGGTTCATACAAGTTCTATGTTCACGCATTATTCAGTTGACAAGCATGTTCCATTTCAA

TGTCAAAGTACTGCTGCATCAGAAAAATTCACAAGAAGGCGGCCTCTGGATGCTCCTTCA

TTTTCTGGTTGGAATTTATCCTATTTCTCCAAGCGTAGGCCACTACGGTGGGATCGGTTA

CAAACAAATGTTTCATATGACGTTGCTAGTGCGGTTGAAGTCATCAATGATTTAGGATTG

GATACTCTTACTTTCTTGGCAGTCACTGTCTTTATTGTCCCCTCATTCAAATTAATTAAA

GCCAGTCCTATACTTGGTTTCTTCTGTGCTGGAGTGGTGCTAAATCAGTTTGGTTTAATT

AGGAACCTTGAAGATGTTAAAGTTTTGTCTGAATGGGGGATCCTTTTCTTGCTGTTTGAG

ATGGGTTTAGAACTTTCCCTAGCACGTCTGAAGGCTCTTGCAAAATATGCCTTTGGAATG

GGACTGACTCAGGTTCTACTATCTACTTTAGCATTCACTGCTTTTGAGCTTCCACCAAAT

GGAGCTGTCGGAACAAAGATATTGCAATTCCTTTTCCACTCAAGGTCTGATCTGGTGAAC

ATCAGGAGTGTCGATGAAGCTGTAGTGATTGGGGCTGCTCTCTCTCTGTCATCCTCTGCA

TTTGTTCTACAGCTTCTTGCAGAGAAAGGCGAGCTTCCTACAAGGCTTGGTTCAGCAACC

CTTGGGATACTTCTCTTGCAGGACATAGCTGTTGTGCCTCTTTTGGTCATACTTCCAGTA

CTTGAGAGCCAGAATATGACCGAGGGAAGTATTTGGCCCATGCTTGCTCAAGAAAGTTTG

AAGGCATTAGGTGGATTGGGCCTGCTTTCTTTTGGAGCAAAGTTTTTTCTCAGAAGAATT

TTTGAGGTTGTAGCAGATACAAGAAGTTCAGAGGCTTTTGTCGCTCTTTGCTTGCTGACT

ATTGCTGGGACTTCACTACTCACACAGAAATTGGGCTTCAGTGATACGCTTGGAGCATTT

TTAGCTGGGGCAATCCTAGCAGAGACAAATTTCAGGACCCAGATTGAAGCTGACATAAGA

CCATTTAGAGGCTTGCTGCTCGGGTTGTTTTTTCTAACTACAGGGACTTCAATTGACATG

CAGGTTCTCTTGAGAGAGTGGCCAAATGTACTTGCGCTCTTGGGGGGTTTAATTACTATC

AAGACATTGATCATAACTGCAATCGGTCCTCGTGTTGGCCTTACTTTACAAGAAAGTGTA

AGAATAGGATTGCTTCTATCCCAAGGAGGAGAGTTTGGATTTGTAGTATTCTCTTTGGCG

AATAGTCTTGGGGTGCTTCCACTTGAGCTGAACAAGCTGCTCATAATTGTCGTTGTATTG

TCAATGGCGTTAACCCCATTTCTGAATGAAGCTGGAAGGAGGGCTGCTAGCTTTATTGAA

GACAAATATGATGTTGATAATAAACAGAAAGATCCTGAGATGGTTAACTTCGAAGTTAAT

GAACCCGTTGTCATCCTTGGATTTGGACAAATGGGCCAGGTCCTTGCCAATTTGCTGTCC

AACCCAATGGCTTCAGGGGGAGATGGTGATGCAATAGGATCGACTTATGTGGCATTCGAT

ATTGACCCCAGGGTTGTAAAGACAGCTAGAAAACTTGGTTTTCCTATTCTATATGGGGAT

GGATCACGTCCAGCTGTTCTTCAATCTGCCGGAATCTCTTCTCCAAAAGCTATCATGATT

ATGCTCACCGAGAAGGAGAAGTCAATTGAAGCTGTTCAGAGGTTACGATTGGCTTTTCCT

GCAGTTCCAATATATGCCAGAGCTCGAGATCTAAAACATCTTTTAGATCTGAAGAAAGCA

GGTGCAACAGATGCTACTTTGGAAAAGGCTGAGACTAGCTTACAACTGGGTTCTAAGATG

CTGAAAGGTCTCGGGATGATGTCCGATGATGTATCATTTTTGAGTCAGCTTGTTAGAGAT

TCTATGGAGCTACAAGCTGAAGAAGCAATCAGCCAATCAGAATATCAGGAATCAAATATT

ATGGAACCATTCCAGGTGAGAGTTGCTGACGTTATGGACGCACGTGTTCCGGTGACAACT

AATACACCAAAATATGAAGTATCAGTACCAAACCAGGAAGATCAAGCTTCATTGAGTAGA

ATTCAGAAGGAAGCAGACCTTGAAGAACAGGATTATGAACTAAATCAAGCTGTTAAATTA

GAAGGAAATGGTGCTCCATGTAGCAAACAAGATATCGGAGAAAGCACGGTGGTTGGATCT

GAAGATGATTTAGGACACTAG

>MS.gene40108.t1

ATGGAAGCACATGCAGGAGCCAACCAAAGGATTGCAAGAATCTCTGCTCATCTTCACCCT

TCCAATTTGCAGGAAGGAGGTGATGTTGTAATTGACAGAGCTAACTGCAGAGCCAAAGGT

GGGGCACCTGGATTCAAAGTAGCAGTATTGGGGGCTGCTGGTGGAATTGGTCAATCCCTT

TCTTTGCTGTTGAAGATGAACCCATTGGTTTCAGTTCTTCATCTTTACGATGTCGTCAAC

ACCCCTGGTGTCACAGCTGATGTTAGTCACATGGACACCGGTGCTGTGGTTCGCGGCTTT

TTGGGGCAACCACAACTTGAGAATGCACTCACAGGCATGGACTTGGTAGTTATACCTGCT

GGTGTGCCAAGGAAACCTGGAATGACCAGGGACGACTTATTTAAGATAAATGCTGGAATT

GTGAGGACTCTTTGTGAAGGAATTGCCAAGTGTTGCCCCAATGCGATTGTCAACTTGATT

AGCAATCCAGTGAATTCCACTGTGCCAATTGCTGCTGAGGTCTTCAAGAAAGCTGGTACA

TATGATCCAAAGCGACTTTTAGGAGTTACAGCCCTTGATGTTGTGAGGGCAAATACTTTT

GTGGCAGAGGTACTTGGTGTTGATCCAAGAGAAGTTGATGTTCCAGTGGTAGGAGGTCAT

GCAGGAGTCACAATATTGCCTCTTTTATCACAGGTTAAGCCTCCTAGTAGCTTCACTGCA

GAAGAAACCGAATACCTGACAAATCGCATTCAAAATGGTGGAACAGAAGTTGTTGAGGCT

AAAGCTGGGGCTGGTTCTGCAACACTATCAATGGCATATGCAGCTGCTAAGTTTGCTAAC

TCATGCCTCCATGGCTTGAAAGGAGAAGCTGGAGTGGTGGAGTGTGCTTATGTTGATTCT

CAGGTCACGGAACTTCCTTTCTTTGCAACCAAGGTTCGTCTTGGTCGTGCTGGAGCAGAA

GAAATATTTCCACTGGGTCCCCTCAATGAGTATGAGAGGATTGGGTTGGAAAAAGCGAAG

AAAGAGTTGGCAGGAAGCATCCAGAAGGGAGTAGAATTCATTAAAAAATAA

>MS.gene40092.t1

ATGAATATAACTAGGCTCTTGATCAATCCTGACATTCTACAGGCTCTTCAACTGAAGAAT

GGGGTGGGCTTTGATGGAGTTGAGACATCCAGGACTGTTCCCAAACTTGGTCCCCGTGTA

AGGTCGTCTTTTGATGAAGTGTTTTTGCGATTGTATGCAAAGAAAACCATTGCTCAGCTG

ACTGATATAGCTGACGCTGGAACTTTTATTGGTAATTTGGAATGGGAAGTGATTGTGTAC

ATCTAA

>MS.gene40111.t1

ATGGAAGACCACAATTTTGTTGTTGGTCAAGAATTCCCTGATGTGAAAGCATTTCGGAAT

GCTATTAAAGAAGCTGCTATTGCACAACATTTTGAGCTTCGTATTATTAAAAGCGACTTG

ATTCGATACTTTGCTAAGTGTGCCTCTGAGGGCTGTCCATGGCGGATTCGTGCTGTCAAA

CTCCCCAATGCTTCAACATTTACCATAAGAAGTCTTGAAGGGACTCATACTTGTGGGAAA

AATGCACTCAATGGACACCACCAGGCTTCTGTTGATTGGATTGTGAGTTTCATTGAAGAA

AGGTTACGTGATAACATCAATTATAAACCAAAAGATATTTTACATGACATCCATAAGCAA

TATGGTATAACTATACCATATAAGCAAGCTTGGCGTGCAAAGGAACGGGGCCTTGCCGCA

ATATATGGCTCTTCTGAAGAAGGATTTTACCTACTTCCTTCATACTGTGAAGAAATAAAG

AAAACAAATCCTGGAAGTGTTGCTGAGGTATTTACCACTGGTGCAGATAGCCGCTTTCAG

AGACTCTTTATTTCCTTTTACGCATCAATTCATGGTTTTGTTAATGGTTGTTTGCCCATT

GTTGCTCTTGGTGGAATCCAGCTGAAAAGTAAATACCTTAGCACATTCCTTTCAGCAACT

TCTTTTGATGCTGATGGTGGGCTGTTTCCTCTTGCTTTCGCTGTTGTCGATGTAGAAAAT

GATGAAAGCTGGACATGGTTCCTGTCTGAGTTGCATAATGCACTAGAGGTGAATACCGAG

TGCATGCCACAGATTATATTTTTATCAGATGGGCAAAAGGGTATTGTGGATGCAATAAGA

AGGAAGTTCACAAAATCTTCTCATGCATTCTGCATGCGCCACTTAAGTGAAAACATTGGC

AAAGAGTTCAAGAACTCTAGGCTTATCCATCTTCTATGGAGTGCTGCATATGCTACTACA

ATTAATGCATTCAGAGAAAAAATGGCTGAAATAGATGAGGTCTCTCCCAATGCCGGCATA

TGGTTACAACATTTTCATCCTTCCCAATGGGCCCTAGTATATTTTGAAGGGACAAGATAT

GGTCATTTATCCTCTAATATTGAGGAGTTCAATAAATGGATTCTTGAAGCCCAGGAGTTG

CCAATTATCCAGGTGATTGAGCGGATTCAAAGCAAACTTAAAACTGAGTTTGATGACAGG

CGTTTGAAAAGCAGTTCATGGTTTTCTGTGCTTACTCCATCTGCAGAGAGACGAATGGTT

GAAGCCATTAATCGTGCATCCACATATCAAGTGCTTAGATCAGATGAGGTAGAGTTTGAG

GTTATTTCCGCTGATCGATCCGATATTGTAAATATTGGCAGCCACAGCTGTTCCTGCCGT

GATTGGCAGCTGTATGGGATACCATGTTCCCACGCTGTTGCAGCCCTTATCTCATCTCGT

AAGGATGTCTACGCATACACTGCAAAGTGTTTTACTGTTGCAAGTTACAGGGATACGTAT

GCAGAGGTGTTACACCCTGTCCCTGGAAAACTCGAATGGAGAACAGATGAATCTGCTTTG

GATAATGATATCGCAGTTGTGCGGCCACCGAAATTTCGCCGACCTCCAGGAAGACCAGAA

AAGAAACGAATTTGTGTGGAGGACCATAATCGCGACAAACATACAGTGCATTGCAGTCGA

TGTAATCAAACTGGACATTACAAGACAACATGCAAAGCAGAGATGATTAGTAGTATAGAA

CAGTTTTAG

>MS.gene40096.t1

ATGGAGACCTATCAATATCCACCGTCATATCCAGATTCCGGTAACTCATCACCACTTTCA

CGAGAAATTGATTTCGAAAATCCATCTTCATGGGAGGAACAACAGAACTACAAAGCAAAA

TTCATGTGCAGCTACGGCGGAAAAATCCAACCACGCAGCCACGACAACCAGCTTTCCTAC

ATCGGCGGCGACACCAAAATACTCGCCGTCGACCGCAGCATCAAGTTTCAAGCCTTTCTC

TCGAAGCTTTCAACTCTCTGCGACGCACTTCAACAAGATATAAGCTTCAAGTATCAGCTT

CCGGGAGAAGAACTCGATGCTCTTATCTCGGTTACTACTGAAGATGATCTTGAACATATG

ATGCATGAGTATGATCGTCTCTATCGTCCTTCTTCAAAACCTGTGAGGATGAGGCTTTTC

ATATTCATCACTCCAAATTCGGGTTCGGTTTCGGGTTCTCAACCTGACCCACTTAAACCT

AATTCTAACGCTGATTTTCTCTTTGGTCTTGAGAATAAAACCATAGCTCCCCCGATTCAA

CCTTCGTATGCTGCTGTGACTGCGAAGTATCACGATCCTGTGCCGGATCTTGTTGCTCCA

CAACCGGAGTATCCGCCACGTGGATCCACCGACGAGAGCGTAGAGATTCAAAGACAGTTA

CAGCGTTTGCAGGTTTCTGAGAGTGAGCAGTCTCTGTATCGGAGAAGTGTAGATGGAACC

ACAGGAGGCTTCGCTGTTGCTCCCAGCGGTGAATATTACATGCAGAAAATGCCAGAGAAT

ATTCCCCGGTCTGATTCACCAGTTACAGTTCATCATCCTGCTAGTTACTGGCCGGATAAA

CAATTTTCCGGCGAAGGTTTTCCGGTGACAGGGATGAACACTTCCGGTGGAGGGGATCAG

CATGTTTACATGATGCCGCCGCCGGGGACATTCTATCAGACAACACAGATGATGCGTCCA

CCGACAGCGCAGGGGTACTACGCAGTGCAAAGAATGGCTTCTGATGGATACCGGGAACAA

CCGGTGTACGGCGGCGTTCCGCCTCAGAACGTTGCGTTTTCGTCGGCGGCGGCTCAGCCA

CTCAAACCTTCAGCTTATCAGGAAGGATACGGCTTAGTTAGGCCAGCTGGTGTAGCGGAA

AATACGGGAGGTGCTGGGTATGCGCAAGTGGCGTATGATAGTGCAAGTGGGAGACAGGTT

TACTATACTACGCCGGGAGGCATGGTCCACGCACCACAGTATCAACAAGGGGTTTCTCCG

GTATTCAGCAATGATATGAGACCGGCTGCGGTTCCTGTGGGCCAGGATCCTAAAGTGGTA

AACAAAGGTTCACATTCACAAGGATTGTGA

>MS.gene40109.t1

ATGGCTCCTAAGAATCCTCAAAAGAGAAAGAAGGGTGAAACCTCTGCACCCCAACCCTCT

AGAAACCAAAACTTCGACTCAACCAGATTTAAAACCCGTTACCATCAGGATAGGTACGTC

GAGTTACTCGACCAAAACATGTGGGCTGAGAGAGTGTTTAACATAAGCCCTAATGGACCT

TACGGGGAAATTGCTAAACTTTTAAAAGATCAAGGGTGGGATAGGCTTTGTAACCCCATC

ACCAACCTAAATGCTGAGCTTGTGCGTGAGTTTTACGCCAATGCTCTTCCTGAAAACCCC

CTCACTGATATCTTTCCATATGAGACTATTGTCCGGGGTCACACCATTAAATTTGATAGG

GCAGCCATCAACAAATACCTTGGCAATCCCTTTGAACTACCCCATGACCATGAGTATGAT

GAATACCACACTAAGAGGAACTTGGGGCATTTTAAGGTTGATGAAACTCATGAAGAAATC

AAACGATTTCTTTTGCTTGAGGGTTTCAACTATGACAAGAGTGAGGCCAGGAGAGAGCAT

CGCTGCCAATACAAATTCATGACTAACCCAGCCAAAATCATCCAGAAATTTATCCTCTAT

AATGCCATGCCTAGTAGCCACATGTCTGATTGTGTTGTGGAAGTCTGCCCCCTCATTTAC

TATATCCTTAATGGCAAACCAGTGGACATAGCTCGAACCATTGCGTGGGAGTTGAGGAAA

GTCGTTTTACAAGTGAAGGGTGAGCCAGCAGCCCGCCTATTCTTCCCCGGTCTCATCATG

GGACTCATTAAGGATACCAGTATGAAGCTTCCTTCTGCCGTTCATGAGAAAATTCGCAAC

CCCATCAATGATATTTTCATCACTCGTTTCATCATGGGTGAATCTAAGAAAGATAAAGGT

AACACAATTCAACAATGCAAGAAACTAAGCTCAATTCAAGGTAAGGAAGGATTAGAGAAG

GATAATAAGAAAAATCAGACTCAACAACAACATCACAGTGTAGTTTCACAACCTAGAAAT

CCATCTAAAAACGATGTTGGGACTCGAGATAATATGGAGGTTTTGAACGCAAGCACTTCA

ATATTACAATCTAAAGTTGCAGTAGCTCATCATAGTGTGGAAATTGGCATTAATTCTGCT

TTGGCGACTAGTACTCGTACCAACACTTTTCAGGTTGATATGGTAGCAGGAAATCCTAGT

GTAGTAGATGTGCCAGATTCAGTCACTGGACATGATTGCAATGAAGGTAATGCTCTTGCT

CTGAATAATTCTTTTGACGTGCTGAATTCTATTAATGATAAGTTAGATGGTGAGGCCTTG

TTTGAGGATTTGGGTAGCAAGGTGGTTTCACCTGACAAGTTGGTAGTGATCAGAGATAAT

CATGCTGTTAATCCTCTAAAGGATGTTGGTTTAGTGTCTGACACAAGGTTAATGGCACCT

GCTAAGGTTGCAAAACCCTCACAACCTTTGTTACATGAGACTACTCAGCCTAGCAATTTG

GTAGGAGTTCAATTTGATCCCAACAATGCTATGCAATGTGATTCTGCTGAGTTGCTTACT

ACTAAATTAGTGGAATCTGTGAGTGCTTTTGCTGAGATGCGTGTTACACATAAATTGGTG

GAACATGTTACTGTTACTAAAGTGTTGGGACCAGACAAAGGAAGAATTTAG

>MS.gene40099.t1

ATGGCGGCTGCAGGTGGCGCAACCTCCTCCACCGCCACTCTTCGCTCCTCCGCCAAATTC

TCCCCTGCTCAATTTCCTAAATCCACATTCCCTCCCCATCGACTCTCTTTTAACACCCCC

AAAATTCCCAAATGCACCCTTCGTTCTGATAATCTCCTTCCTCAAAACGTCATCGTTTCC

AAACCTTCTCCTCTCGAGATCCTTAAAACCTCTTCAGCTGACAGATATACAAAGGAAAAG

AGCAGCATAATTGTGATTGGTTTGAATGTTCACACTGCTCCTGTTGAGATGCGCGAGAAA

CTTGCAATTCCAGAAGCTCAGTGGCCTCAAGTTATTCAGGAGTTATGTGCTCTTAATCAT

ATTGAAGAAGCAGCTGTTCTCAGCACATGTAATCGAATTGAAATATACCTTGTTGCCCTC

TCTCAACACCGCGGTGTTAGAGAAGTCACTGATTGGATCTCCAAGAAAAGTGGGGTTTCC

GTACCTGAGATTTCTAAACATCAGATCTTGTTGTATAATAAGGATGCCACACAACATCTG

TTTGAAGTAGCTGCTGGTCTTGACTCCCTTGTTCTTGGAGAAGGTCAAATTCTTTCTCAA

GTTAAACAAGTTGTTAAATCTGGACAAGGGGTGCCAGGTTTTGATAGGAAAATAAGTGGT

TTGTTCAAGCAGGCTATCTCTGTTGGAAAACGGGTTCGAACAGAGACTAACATTTCGTCT

GGGTCGGTTTCTGTTAGTTCAGCTGCTGTGGAGTTGGCGCTTATGAAGCGTTCAGAGTCT

TCTTTTGGTGATGCTAGGGTGTTGGTAATTGGGGCGGGGAAGATGGGGAAGCTAGTGATT

AAACATTTGGTTGCTAAAGGGTGTCAAAAAATGGTTGTTGTTAATAGAAGTGAAGAGAAA

GTCAATGCCATTAAGAAGGAGTTGAAGGATGTTGACATAGTATATAGACGTCTTTCTGAT

ATGATGGAATGTGCTGCTGAGGCTGATGTGATCTTCACTGGTACTGCATCTGAGTCCCTT

TTGTTTTCAAAAGAGAACGTGGAGATTCTTCCTCCTGTTGGCCAAGGTGTGAGAAGACGG

CTCTTCGTTGATATATCTATTCCAAGGAACGTGGATCCAGGCGTGTCGGAACTTGAGAAT

GCACTAGTGTACAATGTGGATGATCTTCGAGAAGTTGTTGATGCTAACAAGGAGGATAGA

CAACAGAAAGCTATGGAAGCACGGGGAATTATTCAGGAGGAATTGAATACATTTGAGGCG

TGGAAAGACTCTCTTGAAACTGTTCCTACTATTAAGAAATTTAGAGCTTATGTTGAAAGG

ATTAGAGCTTCTGAGATGGAAAAATGTTTGTCAAAGATGCGTGGTGACGTCTCGAAGGAA

CAGAAAGAAGCAATGTATGCCCTTAGTATGGGTATTGTGAATAAGCTACTTCATGGTCCT

ATGCAGCATCTGAGGTGTGATGGGAATGATAATAAATGTCTTGACGAAGTCCTTGAAAAC

ATGCGTGCTCTCAACAGAATGTATGATCTTGAGACGGAAATATCATTGATGGAAGAAAAG

ATCAGAGTCAAGATGGGAAAGGCTAAGAAGTAA

>MS.gene40093.t1

ATGGCATCGGCTTTATTAGACAACCGTAACGAACCAAATTGGCTGCAACACAGAGGCGGT

GGAGCTGAATTCATGGGTAAAGCACCTAACCCTAACCCTAAATTTAGTAACAAAAAGAGA

ACTCAATCCCCATCAGATGACGCTTCCTCAATCAATCGACGATCCAACGATAATCATTCA

CAATACGTTACTTTCAACATCGAATCGTACACAAAGACGGAGCTATACGAGCTCAAAAAT

CGACTCGTATCAGAACTCGACCAAATTCGTCAGCTTAAGACTCGAATTGAATCAGGTGAG

TTTAAACCCAGGCTAAACCACAACGGTGGTGGTCCCACTAAGAAATCAGGGAGCAAGAAG

GTTTCCGGCAACAAACGACCTTTTCCTGCGGTGAAGGAATTGAAGAAATCAAAGTCAGAG

ATTGGGGATGCGATGAAGGCTTGTGGTCAGATTTTGACGAAGCTCATGAAAAACAAAAGT

GGTTGGATTTTTAACACTTGGATCTTTAACACTCCTGTAAATGCAACGGCTTTGAATCTT

CATGATTACTTTGATATAATTAAGCATCCTATGGATCTGGGTACTGTCAAGTCAAAACTT

GCTAAGAATGCTTACTCTACGCCGGCGGATTTTGCTGCTGATGTGAAGTTAACGTTCAAC

AATGCATTGACATACAATCCCAAGGGCCATGATGTTAACACTGCGGCGAAGCAGCTTCTT

GAAAAATTCGAGGAGCTTTATCGACCAATACACGAGAAATTTGATGAAAAAAGTTTTGAT

GATGAATTACAGGCTAGCTCGTGGAACCATGTTGAACCAGAGAGGGAAAGGGAGAGGGTG

AAGAAGAAAGATAATCCAATTCCAATCCCTCCACCGGTAGCAAAACGCCAAGAGCCACTG

CCTGAGCCTGCTAGTACTTCTAACCAGCCAAGCACTTCGAATCCCCCATTAGCTCAGTCA

CCAGTTCGCATACCCTCCCCAACGCGAGCACTTCCAGTGAAGCCCTTGAAGCAGCCCAAG

CCAAAGGCCAGGGATCCAAATAAGAGGGAAATGAATGTTGAGGAAAAACATAAGTTGGGA

CTAGGGTTGCAGATTTTGCCACCAGAGAAGATGGAACAAGTAGTACAGATCATAAGGAAG

AGAAATGGGCATTTGGAGCAAGATGGGGATGAGATTGAGCTTGATATGGAGGCTGTTGAC

ACGGAGACTCTCTGGGAACTCGATCGATTGGTTACCAATTGGAAAAAGATGGTGAGCAAG

ATTAAGCGGCAGGCGCTAATGGATAATAATAATGTGCCTTCAAACAAAGGCAATGGGGAA

TTGCCTGATAGGGAGAAGGTTGATGCAACCCCTCCTTCTGAGGGAAAGAAACAGAAGAAG

ATAGATGCTGTTGATGAGGATGTTGACATCGGGGATGATATGCCAGCAAACGATTTCCCA

CCTGTGGAGATTGAGCAAGATAAAGATATGGGTGGTACTGGTGGCCGTGCCAGTAGTAGT

TCCAGTGGTTCCAGCAGCTCTGGGAGTGATTCCTCGTCGAGTGATTCGGATTCAGGGAGT

TCTTCAGGAAGTGATTCTGAAGCTGGCAATGGTCATTTGTAG

>MS.gene40105.t1

ATGGCTTCATCAGAGATCGAGGTAGTTTCATCAGAATCGCCGAAGCCCCAAGAACAGCAG

CTACCTCCCATACTCGACGTCTTCACTGCTTCCATCCATGGTGACCTTCAAAAATTGAGA

ATTTTCGTTGAACAAGACGGTGCCTCTGTTTCTAACCCCGATTTTAATGGCTATTATGCT

CTTCAATGGGCTTCTCTCAATAACTTTCACCACATTGTTCACTATCTCATTCAGCATGGT

GCCGATGTCAATGCTAAAGACCTCATGTTACAAACGGCTTTACATTGGGCGGCAGTTCGT

GGTGCAATGGCAGCTGCTGATGTGCTCTTGGAGAATGGTGCTCGGGTTGAAGCTGCTGAT

ATAAATGGATACAGGGCAGTTCATGTTGCTGCACAATATGGACAGACAGCTTTCCTAAAT

CACATTGTTGCAAAATACCAAGCTGATTTTGATGTGCCTGATAATGATGGAAGGAGTCCT

CTACACTGGGCTGCATATAAGGGATTTGCTGATACAATAAGATTGCTTCTATTTAGAGAT

GCAAGTCAAGGAAGACAAGATAAAGATGGTGCTACTCCATTGCATTGGGCAGCATTAAGA

GGTCATTCAGAGGCATGTGCTGTGCTTGTGCATGCTGGGACAAAGGAAGAATTGACGGTG

AAGGACAACGCAGGATTCACTCCTGTACAACTTGCATATGATAAAGGTCATCAAAATGTC

GCTCCTTTCCTTTCTAATCAACAACGGGCTCATAGCAACCACTGGAGAGGCAAACTTTGT

TGTGGGTTGGTGACAGATATTGGTTATGCTCCCATTTTATTGTGTATTATAGTCTTTCTC

TCGTTCCTCTTCATCAATTCAGTTGTTGCAGCTCCTAACCTCAAAAAGATTACCGCTGTT

GTTGGATTTTGGTCATGGATAGCTCTCTCTTCATCTGTTGGTTCCTTGATTATGTTTTAT

AGGTGTAGTAGCAGAGATCCAGGGTATATAAAACGGCCAGGAGACTTGGGTACTCTGAAC

GAAACAGAGGATCCGTTGCTGAATATTGAGCTGAATAGTTCATCCGTTTGGACGGGAAAT

TGGTCTCAACTTTGCCCTACCTGCAAGATAATAAGACCTGTCCGATCTAAGCATTGCCCT

ACTTGTAAGCGTTGTGTAGAACAGTTCGACCACCATTGTCCATGGATATCTAATTGTGTT

GGGAAGAGGAACAAAAGGGATTTCTTTATTTTTATCTGCTTGGGAACTATTACTTCTTCT

GTTAGTGCCGTCATTGCAGTTCACAGAATTTGGACATCAATACCAGCATTACCAGCAGGA

GAAACATGGATCCATCAGGTGTTAGTAAGACATCCGGGTCTTGTTGCATTTTTGGTGATG

GATGCTGTTGTCGTTGTTGCAACTACAACTTTGACAGTAACACAGGCTTCCATGATAGCA

CGAAATGTGACCACAAATGAACTAGCGAATTCCACACGGTATGAGTATCTTCGTGGTCCT

GATGGGCGGTTCAGAAACCCATATAATCATGGTTGGTGGAAGAATTGTGCTGATTTTCTT

TTGCTGGGTCACACAGATGACGATGCTATTGCTTGGCCTCCATTACAGCAGGTGGCTTCT

TAG

>MS.gene40112.t1

ATGTCAATAACACAAAACCTCACCATTTCAATCTCTTCTTCTTCATCTTCTTTCTTATCT

CCTTCCAATTTCAATTCCAGGAGTCAGGTTTCATTGCCTGTGAAGAGTGTCAGCATTTGT

AAATGCGTTGCTACACCCCAAGAAGCTGAGACTGCCTACAAGACAAGGGTCTCTCGCAAT

GAAAATTTGGGTAAACTTCAAGCTGGCTATCTCTTTCCTGAGATTGCTAGAAGAAGGTCT

GCACACTTGCTGAAGTACCCTGATGCAAAAATAATAAGCCTTGGGATTGGTGATACTACT

GAACCCATTCCTGAAGTCATAACTTCTGCATTGGCAAAGAAATCACATGCATTGTCAACC

TTAGAAGGATATAGTGGTTATGGAGCTGAACAGGGTGAAAAGCCATTAAGAAGTGCAATT

GCGTCAACATTTTACCCCGATCTTGGCATAGAAGATGATGATATATTTGTCTCAGATGGA

GCAAAGTGTGATATATCTCGTCTCCAGATTGTCTTCGGTTCGAATGTGAAAATGGCTGTG

CAGGATCCATCCTACCCGGCCTATGTCGACTCGAGTGTAATTATGGGCCAGACTGGTCTC

TACCAAAAGGATGTTCAAAAGTTCGCGAACATTGAATACATGAGGTGTAATCCAGAAAAT

GGTTTCTTTCCTGATTTGTCCTCTCTTTCTCGGCCAGATATTATTTTCTTCTGTTCTCCA

AACAATCCTACTGGTGCTGCAGCAACAAGGGAGCAACTGGTCCAACTAGTTCAGTTTGCT

AAGGACAATGGATCTATAATAGTATATGATTCAGCATACGCTATGTATATTTCTGGCGAC

AACCCCCGCTCCATCTTTGAAATTCCCGGAGCCAAAGAGGTTGCCATTGAAACTTCATCA

TTTAGCAAGTATGCTGGGTTCACTGGAGTTCGACTGGGTTGGACTGTGATTCCAAAGCAG

TTACTGTTTTCTGATGGATTTCCCGTGGCCAAGGACTTCAACCGTATTGTATGTACTTGT

TTCAATGGTGCATCAAATATTTCCCAGGCTGGTGGTCTCGCCTGCCTTTCACCAGACGGC

CTTAAGGCTATGCGCGGGGTTATTGGATTCTACAAAGAAAATACTGACATTATAGTGGAA

ACATTTGATTCTCTCGGGTTTAAAGTGTATGGGGGAAAAAGTGCACCATACGTGTGGGTC

CACTTCCCTGGGCAAAATTCATGGGATGTATTCAGTGAGATTCTGGAGAAGACACATGTG

GTTACAACACCTGGGAGTGGTTTTGGACCTGGTGGTGAAGGTTTTGTCAGGGTCAGTGCC

TTTGGTCACCGGGAAAATGTCTTGGAGGCCTGCAGAAGATTCAAGCAGCTATACAAGTGA

>MS.gene40104.t1

ATGAAAGAACTTTTGTCTGATAAGGTTTTCGTCGTCAACGTTCTAGGTTATATAGCATAC

AACTTTGTCATCGGTGCTTACTCGTATTGGGGCCCCAAAGCTGGTTATAGTATATATAAC

ATGACTAATGCAGATATGATATTTGGAGGAATTACAATTGTATGCGGAATATTGGGCACT

TTAGCAGGAGGCCTTGTTCTTGATTACATGACTAACACCCTATCAAATGCATTTAAGCTT

CTCTCATTGACAACATTAGTCGGTGGTGCGTTTTGTTTTGGTGCCTTCACATTCAAAAGC

ATGTATGGCTTCCTAGCTCTTTTTGCTATTGGTGAACTACTTGTTTTTGCCACTCAGGGT

CCCGTGAATTATGTATGTCTCCATTGTGTTAAGCCAAGTTTGAGGCCACTATCAATGGCT

ATGTCTACTGTAGCTATTCATATTTTCGGAGATGTTCCTTCCGCGCCTCTTGTTGGAGTT

GTCCAGGATCATATTAACAACTGGAGAACTACCGCGTTGATTCTAACAACAATATTTTTT

CCTACAGCTGCAATATGGTTTATTGGAATATTTTTGCATAGTGTGGATAAATTTAATGAA

GAGAGTGAGCATCATGTATCAAATGTAGAAGGAACAACCACTGCGCCGTTGCTTGAAGAG

AAGACCGCAGAACCTCAATCCCAAGAAGGTTAA

>MS.gene40110.t1

ATGGATTATCAGTATATTCTAGGAGGGATTTTGGCTTTTAGTTTGGCGTTTGTGTTTGTT

ATGTATAGTTTTGGAGAAAAGAAGAAAACAGGTTCAAGTTCAATGGATGTGAAAAGTAAT

GGATATGCAAAGACATCATCAGAAAATGGAATTTGTTCACAAGAGGTTGTTGGAGAAACA

GATATCATCATTGTAGGTGCCGGAGTTGCTGGTGCTGCTCTTGCTTATACACTTGGAAAG

GATGGACGGCGAGTACATGTGATAGAAAGAGACTTAACTGAGCCTGATAGAATTGTGGGT

GAGTTGCTACAACCTGGTGGCTATTTGAAGTTACTTGAGTTGGGTCTTGAAGATTGTGTG

GATGAAATTGATGCTCAAAGAGTGTTTGGCTATGCACTTTATAAGGATGGGAGAAATACC

AAACTGTCTTATCCCTTGGAAAAGTTTGACTCTGATGTATCTGGAAGAAGTTTTCACAAT

GGTCGTTTCATACAGAGAATGCGAGAAAAGGCTTCAACTATTCCAAATGTTAAATTAGAA

CAAGGAACTGTTACATCATTACTTGAAGAAAATGGAACTATCAAAGGTGTGAACTACAAA

AATAAGAGTGGTCAAGAATTCACAGCAAAGGCTCCTCTCACCATAGTATGTGATGGTTGT

TTTTCCAACTTGAGGCGTTCTCTTTGTAATCCTAAGGTTGAAGTTCCCTCTCATTTTGTT

GGATTGATTTTGGAGAATTGTAATCTTCCACATGCAAATCATGGACATGTTATCTTGGGT

GATCCTTCACCCATTTTGTTTTACCCTATAAGTAGTACCGAGATTCGCTGTTTGGTTGAT

GTGCCTACTGGCCAAAAATTACCTTCTCTTGGTAATGGTGAAATGGCAAATTATTTGAAA

ACTGTGGTTGCTCCCCAGGTTCCTCCAGAACTGCAAGCTTCTTTTATTGCAGCAGTTGAT

AAAGGAAACATAAGAAGCATGCCAAATAGAAGCATGCCTGCATCACCTTATCCCACCCCC

GGGGCTCTTCTGATGGGAGATGCCTTCAATATGCGTCATCCTTTAACAGGAGGAGGAATG

ACCGTGGCTTTGTCTGACATTGTTTTGCTAAGGAATCTACTTAAACCTCTGCGTAATCTG

CATGATGCTTCTGCTCTTTGCAATTACCTTGAGTCGTTCTACACTCTACGCAAGCCAGTG

GCATCTACAATAAACACATTAGCAGGTGCATTGTACAAAGTATTTTGTGCATCTCCTGAT

CCAGCTAGCATTGAAATGCGCCGAGCTTGTTTTGATTATTTGAGCCTTGGAGGTGTTTGC

TCAGACGGACCAATAGCTCTACTCTCCGGTCTGAATCCGCGTCCATTGAGCTTGGTTCTA

CATTTCTTCGCAGTGGCTGTATATGGTGTTGGCCGCCTCTTGATACCGTTTCCTTCTCCA

AAACGAATGTGGATCGGAGCTAGATTGATTTCTGGTGCATCAGGTATCATTTTCCCCATT

ATCAAGGCTGAAGGAGTGAGACAAATGTTTTTTCCAGCAAGTGTGCCAGCATATTACAGA

ATGCCTCCTGTCCATTAA

>MS.gene40098.t1

ATGGAATTGCATCAGAAACCAACGGCAAAATCTGAGCTTGATCAATTCATGGAAATGGGT

CCCACGTGGCTGCGATTTGAATCGGATCGACCCGTTTCGGTTTTTGGGGTTGCTGATGAG

GGTGCACGAAAGTGGAAAAAGAAAGGTGCTTGGAGGGGTGCTTATTTTCCTAAATTTAAT

GTTACTGTTGCTTATCATCGTGCTGTTTTGCTCTCCAAATACAAGTGGCAGCCAAAGCAA

TCTGAATCTGGGATGCAAGATGGATCAGAAGGTATTCATCGAGTTGACGTGGATGTTCCT

GCAGATGAATGGGCTAAAATTGCAGGTTTCTATGATGTCCTGCTGTTTAATACTGGTCAC

TGGTGGAATCATGACAAATTCCCAAAGGAGAAACCTCTTGTCTTTTATAAAGCAGGACAA

CCAATAGTTCCTCCACTCGAGATGTTAGATGGACTTAAAGTTGTTCTTGGCACTTTTATG

GTGGAGATTGGAATCAAAACGGTAGCTGCTTGTTCAACAAGCCACTTGAGGAGAATGAGG

AACAACGGAGTAAACAAGGAAGCAAGACAGATGAATTTTGTGATTGAAAAGGTATTACAG

GGCACAAACATCCATGTGGTTGATTTTACGCATTTAAGTGAGTTTAGAGCCGATGCTCAT

CCAGCCATATGGCTAGGAAGGAAAGACGCAGTTGCAATTTGGGGTCAGGATTGCATGCAT

TGGTGTCTGCCGGGTGTCCCTGACACATGGGTTGATATTTTATCACAACTAATCCTTGAT

TGTTTTGGAAGAACTGGAGCATGCTGCCAAGTGGTGGGACCAATATATGAGAACCATGAG

CTGTACATAGAAATGTCAAAGGTTTGA

>MS.gene40103.t1

ATGGCACAAAAACCTGAACATGAACCGAAACCTTCAACAACAACTTCTTCTTCAACACCT

AACCATGTTCCTTCTTCTGTGGAACCAAACATGGTCCCCAAGTCCACCATGATTCCTGCA

ACTTCTTGGTTTACACCAAAAAGGTTACTGGCTATATTTTGTGTGATTAACATGTTGAAC

TATTTGGACCGAGGAGCAATAGCTAGCAATGGTGTTAATGGACATCGTGGGACTTGCACT

GATGGTATCTGCAAGGGGGATTTTAACTTGAACAATTTTCAAGATGGAGTTCTCTCATCT

GCTTTTATGGTTGGACTTCTTATAGCTTCTCCAATATTTGCATCTCTTTCAAAGAGTGTA

AACCCCTTTAGACTTATTGGAGTTGGTTTGTCAGTTTGGACTGTTGCAACCTTATGCTGT

GGTCTTTCTTTCAATTTCTGGTCCATTACATTATGTCGCATGCTCGTTGGTGTTGGTGAG

GCTTCATTTATAAGTCTTGCAGCACCTTTCATTGATGATAACGCCCCAGCTTCACAGAAA

ACAGTGTGGCTTTCTATATTTTACATGTGTATACCAGGAGGATATGCAATTGGCTATGTA

TATGGTGGAGTGGTTGGAAGTCATTTTGGTTGGCGTTATGCGTTTTGGGTGGAGGCTATC

TTGATGCTTCCATTTGCTATTATGGGATTTGTTATGAAGCCTTTGCAGTTAAAAGGTGCA

CATTTTCATCTTGCATTGATTTATTTATTGTGGCACCTTGATATTGTTTTCGATCTCTAA

>MS.gene40100.t1

ATGACTGTGAATATACTAAGGATAACACCTCTTTCTTCCCTCAACGTCACCAAACCCATC

TCCTCAATTTGTTCTAACCCTACCTTTCTCTTCAACTCCAAATTCACCTTCACCCCACCC

AACTCTCTCCCTACCATCTCCATTCGTCAGCCGAAGCGTGGTTTTCGCGGTGGAATTGTC

GCCATGTCTGCTGCACCTACTCCTGGCTCGGTCCAGAAATCGGAGGAAGAATGGCAAGCT

ATTCTCTCCCCTGAGCAGTTTCGAATTCTGAGGCAGAAAGGCACCGAGTACCCTGGAACA

GGAGAATATGACAAATTCTTTGGGGAGGGAGTCTACAGTTGTGCAGGTTGCGGGACTCCA

CTCTACAAGTCAACAACAAAATTCAATTCCGGTTGTGGCTGGCCAGCCTTCTATGAGGGT

GTTCCTGGAGCTATAAACCGCCACCCGGACCCAGATGGAATGAGGATAGAAATAACATGT

GCTGCTTGTGGGGGACATCTCGGTCATGTATTTAAAGGTGAAGGATTTCCGACACCCACG

AACGAACGCCATTGTGTCAATAGCATTTCACTAAAATTTGCGCCAGCCAATTCCTAG

>MS.gene40095.t1

ATGGTATCCGCCGTATTAGCCAACCGTAATGAACCAAATTGGCCGCAGCACAGAGGCGGT

GGAGCCGGATTCATGGGTAAAGTACCTTACGCTAACACTAACACTAACCCTAACCCTAAA

TTCAAGAGGAATCAATCTCCTTCAGATGACGCTTCTTCAATCAACCGGCGTTCCAACGAT

GTAGTCACGAATCATTCACAATACGTGACTTTCAATGTCTCTTCGTACTCAAAATCAGAG

CTCAACGAGCTTAGAAACCGTTTCGTAACGGAGCTTGAACAAATTCGACAGCTCCAGACA

CGAATTGAATCAGGGGAATTAAAATCCAGGTCAAGCCACAACGGCGGTGGTCCCCCTAAG

AAATCGGCGAACAAGAAGTTTTCCGGCAATAAGCGACCTTTTCCGGCGGATAAGAGATCA

AAGTCAGAGGTTGGAAGCGCGATGAAGGCTTGTGGTCAGATTTTGCAAAAGCTGATGAAA

ACCAAAATAGCTTGGATTTTTAGCCATCCGGTTGACCCGGTTGCTTTGAATCTTCATGAT

TACTTTGATATAATTAAGCATCATATGGATCTGGGTACTGTTAAGTCAAAGCTTGCTAAG

AATGCTTACGCTATGCCGGTGGATTTTGCTGATGATGTGAGGTTAACGTTCAAAAATGCA

TTGACATACAATACCAAGGGCCATGATGTTAACACTGCGGCGACGCAGCTTCTTGGAAAA

TTCGAGGAGCTTTATCGACCAATACATGAGAAATTTGATGAAAAAAGTTTTGATGATGAA

TTACATGCTAGCTCGTGGAACCCTGTTGAAATGGAGAGGGAAAGGAAGAGGGTCAAGAAA

AAAGATAATCCAATTCCAATCCCACCACCGCTAGCAAAACGCCAAGACCCAATGCCTGAG

CCTGCTAGTACTTCTAACCAGCCAAGCACTTCAAATCCCTCATTAGCGCAATCACCAGTT

CGCACACCCTCCCCAACGCGAGCACTTCCAGTGAACCCCTTGAAGCAGCCCAAGCCAAAG

GCCAGGGATCCAAATAAGAGGGAAATGAATCCGCCAGAGAAGATGGAACAAGTAGTACAT

ATCACAAGGAAGAGAAATGGGCATTTGGAGCAAGATGGGGATGAGATTGAGCTTGATATG

GAGGCTATGGTGAGCAAGATTAAGCGGCAGGCGCTAATGGATAATAATAATGTGCCTTCA

AACAAAGGCAATGGGGAATTGCCTGATAGGGAGAAGGTTGATGCAACCCCTCCTTCTGAG

GGAAACAAACAGAAGAAGATAGATGATGTTGATGAGGATGTTGACATCGGGGATGATATG

CCAGCAAACGATTTCCCACCTGTGGAGATTGAGCAAGATAAAGATATGAGTGGTACTGGT

GGCTGTGTCAGTAGTAGTTCCAGTGGTTCCAGCAGCTCTGGGAGTGATTCCTCGTCGAGT

GATTCGGATTCAGGGAGTTCTTCAGGAAGTGATTCTGAAGCTGGCAATGGGGTTGTAGAC

ATTGGTTGTTGGTTTCAGCGTGGGAATGGCTACTTGGATTGGGACTAA

>MS.gene40106.t1

ATGGAGTTACGTGAACTTGGAAGAACTGGACTTAAACTAAGCTCCGTCGGGTTCGGAGCT

TCTCCTCTCGGCAACGTCTTCGGTTCCGTTTCAGAAGAACAAGCAAACTCCTCTGTTCGT

ATCGCTTTTCAATCCGGCATCAACTTCTTCGACACTTCTCCATATTATGGAGGAACACTG

TCTGAGAAAGTGCTCGGAAAGGCGTTGAAAGCGTTAAACGCTCCGAGAAGTGAATACATT

GTAGCAACTAAGTGTGGAAGGTACAAAGAGGGTTTTGATTTTAGTGCAGAGAGAGTCACC

ACAAGTGTAGACGAGAGTTTGGAAAGGTTGCAGCTTGATTATGTTGATATTCTTCAGTGT

CATGATATTGAATTTGGATCGTTAGATCAGATTGTTAATGAAACAATTCCTGCACTCCAG

AAACTGAAGGAAGCAGGGAAGACTCGTTTCATTGGGATTACGGGACTTCCTTTGGAGGTA

TTTACTTATGTTCTTGATAGGGTTCCGCCTGGAACATTGGATGTGATACTTTCCTATTGC

CATCACTCTGTCAATGATTCGACTTTGGAGGATATAGTGCCCTATCTGAAGTCCAAGGGT

GTTGGCATTATCAGTGCTTCTCCGTTGGCGATGGGTCTTCTCACTGAGGCTGGCCCTCCT

GAATGGCATCCTGCTTCACCGGAACTTAAGTCTGCTTGTAGAGCTGCTGCAACTTATTGC

AAAAAAAATGGAAAAAACATTTCAAAGTTAGCAATGCAGTACAGCTTGTTAAATAAGGAA

ATCACATCAGTTCTTGTTGGCATGAGATCTGTCGAACAGGTGGAGGAAAATGTTACTGCC

GCAAGAGAACTTGCAGCTTCTGGAATCGACGAAGCGGCTCTGTCAGAAGTCGGAACCATT

CTAAAGCCTGTCAAAAATCAGTCATGGCCTAGCGGAATCCAGCAGAGTTGA

>MS.gene40097.t1

ATGGATTGCGTTGCCGGCACGTGTTTGTTTCCGTTACATCGTTGCAAAACGATTCACCTT

GTGAGACACGCTCAAGGGATCCACAATGTGGAAGGAGACAAAAACTACAAAGCGTATTTG

AATCCTGATTATTTTGATGCTCATCTCACTCCTCTTGGATGGGAACAGGTTGATAATCTG

CGTAAACATGTTCGTTCCTCTGGATTAATTAACAAGATCGATCTCGTCATCGCTTCTCCT

TTGATGAGGACTTTGCAAACAGCTGTTGGGGTATTTGGTGGGGAGGGATACACTGATGAT

AAAACGGATGTGCTGCCTCTTATGGTGGCAAATGCAGGAAATAGCTTCCGTGGTGCAATT

TCAAGTCATAATTGCCCACCAATTGTTGCTGGCGAACTTTGCCGTGAACATTTGGGAGTT

CATCCCTGTGACAAAAGAAGAAGTGTTAGTGAGTATCAGTTCCTTTTTCCTGCTGTTGAT

TTTTCACTGATAGACAGTGATGAGGATGTTTGGTGGAAGGATAATGTAAGAGAGACAAAG

GAGGAACTGGCAGCTAGGGGGGTGGAGTTCTTAAACTGGTTGTGGACACGAAAAGAGAAA

GAGATAGCGATTGTGACACACAGTGGATTCTTGTTTCACACTCTAACTACATTTGGAAAT

GATTGCCACCCCTTGGTGAAGAAAGAAATATCCAAGCACTTTGCCAATTGTGAGCTTCGC

TCCATGGTCCTTGTGGATAGAAATATGATAGGATCAGAAGCATCAACAACTAACTATCCG

GGCAAGATACCTTCAGGGCTGGACAAGCCTAGTGATGCTGTGGATGAAAATGTCGAGAAA

CAGGGGGTTTAG

>MS.gene40094.t1

ATGATGGGTGAGGGTTCAAACATCTTCGAGAAGATAATTGATGAAGAAACACCAAAGGGA

GTGTGGCAAAGACTCAAGAAGGTGTATGGTGGCGATGTCAAGCTCAAGAAAGCGAAGCTC

CAAGCCTTGTGCCATCACCATGAGATGCTCCAAATGACTAAGCAAGAGTCAATTGCAGAG

TTCTAA

>MS.gene40102.t1

ATGGGGCATTCGGCAGCTGTGTGGGACTTTAGAGCAGCAACTGAATTTACAAAGGACTTG

AATGGAATCGATCAAGTTGTACTTCGGACCCCACAAGGCGCTTCGGCACGGGTAAGCTTA

CATGGAGCGCAGGTCACTTCATGGCGTAATGAGCAGGGGGAAGAACTGCTATTCACAAGC

TGCAAGACACTTTCAAAGGCGCCAAAAGCAATACGAGGAGGAATTCCAATATGTTTTCCA

CAGTTTGGAAACTGTGGATCGCTGGAGCTGCATGGATTTGCAAGAAATAAAATGTGGGCA

ATTGACGAGAATCCTCCTCCTTTGCCTCCAAATGATTCCAATGGAAAATCCTTTGTCGAC

CTGCTACTCAAATTATCTGAAGAAGATGTGAAGTGCTGGCCACATAGTTTTGAGTTCCGC

CTTAGAGTGTCTCTTACGACAGATGGAGACCTAACCCTGATATCACGAGTGAGGAATATC

AATGCCAAGCCATTTAGTTTCTCATTCGCATACCATACATACTTGTTGGTTTCTGACATA

AGTGAGATAAGGGTTGAAGGTCTTGAGACACTTGATTACCTGGACAACCTTTCCCAAAAA

GCACGAGTTACAGAACAAGGAGATGCCATAACATTTGAATCCGAGGTGGATCGTGTTTAT

CTTAGCTCTCCAAACATAATCGCAGTTCTAGATCATGAGAGGAAACGGACATACATTATA

AGAAAGGAAGGTCTCCCTGATGTTGCCGTGTGGAATCCATGGGAGAAGAAATCGAAGTCG

ATGGTGGACTTTGGTGATGAAGAGTACAAACAAATGCTTTGTGTAGATGGTGCAGTTATA

GAGAAACCTGTGAACTTGAAGCCTGGAGAGGAGTGGACAGGGAGGATTCAACTCTCAGTT

GTGCCATCAAGTTTTTGTAGTGACCGACTAGGTCTCGACAGAAGTGGTATTTGA

>MS.gene40115.t1

GTACCTCCTCCAGGGAAAGGGACGCTTTATCTTAGGCCTTTGCTGATAGGAACAGGAGCT

GCATTAGGCTTGGCTCCATCACCTGAGTACACATTTCTCATTTATTGCTCCCCTGTTGGA

AATTACCACGAGGGAGGAAGACTAAACTTAAAAGTGGAGGATAAATTTCATCGATCAATA

GCTGGCAGTGGTGGAACAGGAGGAATCAAGAGTGTTACTAATTATGCCCCGATATATACT

GCAGTAACTGAAGCAAAAGCCAATGGATTTTCTGATGTCTTGTTCTTGGATTCAGCAACT

GGAAACAATATTGAGGAGGCTACTGCATGCAATATATTTGTTGTGAAGGAAAATGATATC

TTCACTCCGGCAATAGATGGATCTATTCTGCCTGGGGTCACACGAAAATCCATCATAGAC

ATCGCCATTGATTTGGGTTATAAGGTCATAGAACGTTCCATATCAGTGGAGGAAATGATG

AGTGCTGATGAAGTGTTCTGCACAGGAACTGCAGTGGTTGTTACCTCTGTTGCATCTGTA

ACATATAAGGAAACAAG

>MS.gene40113.t1

GAGTCAGGTTTCATTGCCTGTGAAGAGTGTCAGCATTTGTAAATGCGTTGCTACACCCCA

AGAAGCTGAGACTGCCTACAAGACAAGGGTCTCTCGCAATGAAAATTTGGGTAAACTTCA

AGCTGGCTATCTCTTTCCTGAGATTGCTAGAAGAAGGTCTGCACACTTGCTGAAGTACCC

TGATGCAAAAATAATAAGCCTTGGGATTGGTGATACTACTGAACCCATTCCTGAAGTCAT

AACTTCTGCATTGGCAAAGAAATCACATGCATTGTCAACCTTAGAAGGATATAGTGGTTA

TGGAGCTGAACAGGGTGAAAAGCCATTAAGAAGTGCAATTGCGTCAACATTTTACCCCGA

TCTTGGCATAGAAGATGATGATATATTTGTCTCAGATGGAGCAAAGTGTGATATATCTCG

TCTCCAGATTGTCTTCGGTTCGAATGTGAAAATGGCTGTGCAGGATCCATCCTACCCGGC

CTATGTCGACTCGAGTGTAATTATGGGCCAGACTGGTCTCTACCAAAAGGATGTTCAAAA

GTTCGCGAACATTGAATACATGAGGTGTAATCCAGAAAATGGTTTCTTTCCTGATTTGTC

CTCTCTTTCTCGGCCAGATATTATTTTCTTCTGTTCTCCAAACAATCCTACTGGTGCTGC

AGCAACAAGGGAGCAACTGGTCCAACTAGTTCAGTTTGCTAAGGACAATGGATCTATAAT

AGTATATGATTCAGCATACGCTATGTATATTTCTGGCGACAACCCCCGCTCCATCTTTGA

AATTCCCGGAGCCAAAGAGGTTGCCATTGAAACTTCATCATTTAGCAAGTATGCTGGGTT

CACTGGAGTTCGACTGGGTTGGACTGTGATTCCAAAGCAGTTACTGTTTTCTGATGGATT

TCCCGTGGCCAAGGACTTCAACCGTATTGTATGTACTTGTTTCAATGGTGCATCAAATAT

TTCCCAGGCTGGTGGTCTCGCCTGCCTTTCACCAGACGGCCTTAAGGCTATGCGCGGGGT

TATTGGATTCTACAAAGAAAATACTGACATTATAGTGGAAACATTTGATTCTCTCGGGTT

TAAAGTGTATGGGGGAAAAAGTGCACCATACGTGTGGGTCCACTTCCCTGGGCAAAATTC

ATGGGATGTATTCAGTGAGATTCTGGAGAAGACACATGTGGTTACAACACCTGGGAGTGG

TTTTGGACCTGGTGGTGAAGGTTTTGTCAGGGTCAGTGCCTTTGGTCACCGGGAAAATGT

CTTGGAGGCCTGCAGAAGATTCAAGCAGCTATACAAGTGA

>MS.gene40114.t1

ATGGCGGTGGAATACACATGCTGCGAATCGGAGTTCTTCATCCACATATTGGTTATCATC

TTCTTGGTGGTCTTCGCCGGTTTAATGTCCGGACTCACCTTAGGTCTCATGTCTCTCAGT

CTCGTTGATCTTGAAGTTCTTGCTAAGTCTGGTACTCCTCACGATCGTAAACACGCCGTG

AAGATATTACCTGTTGTGAGAAATCAGCATTTATTGCTTTGTACTTTACTCATTTGCAAT

GCCGCCGCCATGGAGGCACTTCCTATTTTTCTTGATAGTCTTGTTACTGCTTGGGGTGCT

ATCCTGATTTCAGTCACGTTGATTCTTCTCTTTGGTGAGATTATACCCCAATCAGTTTGT

TCTCGATATGGTTTGGCCATTGGTGCATCAGTCACTCCTTTTGTCCGCGTGCTTGTATGG

ATATGTTATCCAGTTGCTTTTCCAATTAGCAAGTTGTTAGACTATTTGCTGGGTCATCGA

CATGAAGCCCTTTTCCGTAGAGCCGAGTTGAAAACACTAGTAGATTTGCATGGTAATGAG

GCTGGAAAAGGTGGGGAACTGACACATGATGAAACAACAATAATTGCCGGGGCACTTGAA

CTCAGTGAGAAGACAGCCAGTGATGCTATGACTCCTATATCTGAAACATTTGCTATTGAT

ATTAATTCGAAGCTTGATAGGGAACTGATGACTGAAATATTGGAGAAAGGACATAGCAGA

GTCCCAGTCTATTATGAGCAGTCTACTAACATTATTGGATTAATACTGATCAAGAACTTG

TTGACTATTCATCCAGAAGATGAATCGCCTGTAAAGAGTGTAACCATACGCAGGATTCCA

AGGGTTCCAGAAAGTATGCCGCTGTATGACATTTTGAATGAGTTTCAGAAGGGGCATAGT

CACATGGCCGTTGTTGTACGACAATGCGATAAAACCAAGCAACCATCTTCCAAAAATGAT

TCAAATGATTCTGTGAGAGAGGTGAAGGTGGATATTGATGGTGAAAAGCCTCTCCAAGAG

AAAGTCTTGAAACCCAAGATACCAATCCAAAAGTGGAAAAGCTTTCCAAATACGAACAAG

TCGAATAGGGGTTCTCGGAGCAGAAAATGGTCAAAAAATATGTACTCAGATATTTTAGAG

ATAGATGGTAGTCCTCTTCCAAATATCCCTGAAGAAGAAGAAGCTGTTGGAATAATTACA

ATGGAGGATGTTATTGAAGAGCTTTTACAG

>MS.gene40116.t1

TGATATGGGTGGAAATTATGCTGACATCAATTGGGAAGGACTTAGTTTTAATCTGACTCC

AACAGATTACATGCATGTCATGAAATGCACAAAAGGAGAAAAGTTTTCACAAGGATCCCT

CATTCGCTATGGAAACATTGAGATAAGCCCGGCTGCTGGTATCATAAACTATGGACAGGG

AATCTTTGAGGGACTAAAAGCATATAGAACCGAAGATGGGCGAATCCTTCTTTTCCGACC

AGAGGAGAATGCTCTACGCATGAAGATGGGGGCTGATAGGTTGTGTATGCCATCACCATC

AGTTGAGCAGTTTGTTGATGCTGTTAAGCAAACAGTTCTTGCCAATAAACGTTGGGTACC

TCCTCCAGGGAAAGGGACGCTTTATCTTAGGCCTTTGCTGATGGGAACAGGAGCTGCATT

AGGCCCGGCTCCATCACCTGAGTACACATTTCTCATTTATTGCTCCCCTGTTGGAAAGTA

CCACGAGGGCGGAAGACTAAACTTAAAAGTGGAGGATAAATTTCATCGATCAATAGCTGG

CAGTGGTGGAACAGGAGGAATCAAGAGTGTTACTAATTATGCCCCGATATATACTGCAGT

AACTGAAGCAAAAGCCAATGGATTTTCTGATGTCTTGTTCTTGGATTCAGCAACTGGAAA

AAATATTGAGGAGGCTACTGCGTGCAATATATTTGTTGTGAAGGAAAATGATATTTTCAC

TCCGGCAATAGATGGATCTATTCTGCCTGGGGTCACACGAAAATCCATCATAGACATCGC

CATTGATTTGGGTTATAAGGTCATAGAACGTTCCATATCAGTGGAGGAAATTATGAGTGC

TGATGAAGTGTTCTGCACAGGAACTGCAGTGGTTGTTACCTCTGTTGCATCTGTAACATA

TAAGGAAACAAG

>MS.gene40120.t1

ATGAAGCCTTCAAATATTCTCGTAATCTTAACCATTTTGTGCATGTGTTGTTTCCTTTGC

CTGGCAAAAGACTCCCATCAAGAATACGTAGACGCGCACAATAAGGCTCGTGCTGAGGTT

GGTGTTCCTCCACTAAAGTGGAATGACACCGTTGCAGAATATGCCAAGAACTTTGCTAAA

TCGAAGATTGATAGCTTCGGAATTGATCCCTCCGGAGGGCCTTATGGTGAGAACATTGCA

ACATTTGCCTTTGGTAAAGTGAGTCCTAGTGATGTTGTGGATAAGTGGCTCAATGAGAAG

AAGGATTATAATTATACTGCCAACTCGTGTACTCTAGGTTTATACTGCGGCAATTATAAA

CAGATTGTTTGGCGTGATACTGTTCATCTTGGTTGTGCTAAAACAAAATGTAACAATGGT

TGGTATTTTTTCGTTTGTAACTATGACCCACCAGCAAATCTTAAAGGCGGTCGTCCTTAC

TAG

>MS.gene40118.t1

ATGGCAACATCCCATCAATCACCCAACAATGGTAAAGCTTCCAACAGGGAGACTGAAAAA

ATATATGCCAATATGGATTGGGACAAACTTGCATGTGGAGTGATTCCAACTGATTATATG

TACATCATTAAATCCAATGAAGACCGGACCTTTTCAAACGGCACTCTCGTGCCTTTTGGA

ACCATTGATATCAACCCACATTCTGCTGTTATAAATTATGGACAGGGATTATTTGAGGGC

ATGAAGGCTTACAGAACAAAAGACGGCAATGTGCAACTATTCCGACCCGAAGAGAATGCA

TTGCGCATGCAGATGGGAGCAGAGAGGCTGCTGATGCCATCACCTTCTGTTGATCAGTAC

ATTGATGCTGTAAAACAAGTTGTTCAAGCAAATAAACGTTGGGTGCCTCCTTGGGGAAAA

GGAACATTGTACATTAGGCCTTTACTATTTGGAAGTGGACCTGTTCTGGGTATTGGACCA

GCACCTCAATGCACCCTCTTAATATTCACTAATCCAATTAGCAACATTTACAAGGGACAA

AAATCAGCCTTGAATTTGTTGATTAATGAAAACTTTCCTCGTGCATATCCTGGTGGAACT

GGTGGAGTAAAAAGTATTAGTAATTATCCACCTGTTTTTCAAGTTGTAAAAGAAGCAAAA

GCCAAAGGATTTTCCGATGTGCTTTTTCTAGATGCAGTGGAACATAAATACATTGAAGAG

GTATCTTCGTGTAATGCTTTCATTGTGAAGGGTAAGGTTATTTCAACTGCATCTACACTC

GGAACTATTCTTCCTGGAGTCACAAGGAAAAGTGTCATTGAACTTGCACGTGATTTGGGT

TACGAGGTGATGGAACGCAAGGTCTCGGTAGAAGAACTGCTTGAAGCTGATGAGGTTTTC

TGCACTGGAACCGCTGTTGGGATCTCTGCTGTTGGCAGTGTAACATACAAGAATAAAAGT

GTTAACTTCAAAACAGGGGCAGATAATGTGACTAAGAAGTTGTATGATTTGATTACGGGC

ATCCAGACAGGTCTCTTGGAAGATAAGAAAGGATGGGTGGTCAAGATTGATTGA

>MS.gene40117.t1

ATGAAGGCTTACAGAACAAAAGACGGCAATGTGCAACTATTCCGACCGGAAGAGAATGCG

CTGCGAATGCAGATGGGAGCAGAGAGGCTGCTGATGCCATCACCTTCTGTTGAGCAGTAC

ATTGATGCTGTAAAACAAGTTGTTCATGCAAATAAACGTTGGGTGCCTCCTTGGGGAAAA

GGAACATTGTACATTAGGCCTTTACTATTTGGAAGTGGACCTGTTCTGGGTATTGGACCA

GCACCTCAATGCACCCTCTTAATATTCACTAATCCAATTAGCAACATTTACAAGTTCTAT

GAAGGTGAACGGCGGATGTTGTTGATGCTTGAGGCATATCCGAACCAAAAAATTGTTGTG

ATGAGAGTTGTTGAAGAATTTGGATTTGGCTGCTGTGCTTGTGTGAACCAAAAAATTGAA

TTTGGATCCGAACCAAGTGTTGTTCTGTTCCATGTTGTGATTTCTCTGTCGGATGAACCG

GTGAAAAGGAATTTGCCGAAGAGAGTGAGAGAGGAGGTTTTTCAAGTTGTAAAAGAAGCA

AAAGCCAAAGGATTTTCCGATGTGCTTTTTCTAGATGCAGTGGAACATAAATACATTGAA

GAGGTATCTTCGTGTAATGCTTTCATTGTGAAGGTTATTTCAACTGCATCTACACTCGGA

ACTATTCTTCCTGGAGTCACAAGGAAAAGTGTCATTGAACTTGCACGTGATTTGGGTTAC

GAGGTGATGGAACGCAAGGTCTCGGTAGAAGAACTGCTTGAAGCTGATGAGGTTTTCTGC

ACTGGAACCGCTGTTGGGATCTCTGCTGTTGGCAGTGTAACATACAAGAATAAAAGTGTT

AACTTCAAAACAGGGGCAGATAATGTGACTAAGAAGTTGTATGATTTGATTACGGGCATC

CAGACAGGTCTCTTGGAAGATAAGAAAGGATGGGTGGTCAAGATTGATTGA

>MS.gene40122.t1

ATGTGCTGTTTCCTTTGCTTGGCACACAACGCCCCTCAAGACTACCTTGACGTGCACAAC

AAGGCTCGTGCCGAGGTTGGCGTTGGCCCACTCGTGTGGAACGAAACCCTTGCAGCCTAT

GCCATGAACTATGCTAAATCGAAGCACGAAACCTGCGAAATGGTGCACTCCCAAGGGCCT

TATGGCGAGAACCTTGCAGAGGGTTCCGACCCCCAAATGACTGCTGCCGCTGCTGTGAAA

TTATGGGTGGATGAGAAGGCCTTCTATGACTATAGTACCAACGCGTGTGTTAAGATGAGT

GCAGACATTATACACAAGTGGTTTGGAGCAATACCAAGCAGCTTGGTTGTGCTAGAGAAA

GTTGTAAGAATGGATGGACATTTTTCATTTGTAGCTATTACCCACCAGGAAATTATGTCG

GCGATAAACCTTACTAGATTTGAG

>MS.gene40121.t1

ATGGCAACTAATCAGATGCACGAATTGTTACATCCGTGGCTGGCACGCAAAGAAATCAAG

GAGATCTCTGTATTGTTGGGAAGATTGACTTCAACAATATGGTTAGAAGAACTCCGGGAC

ACAACCGACTGTTTTGCTATAGACAATGCTATTGGGGTGGGAAAGATGGGAATGATGTAC

CAGGGAAATCTACCAAATGGTCAGTTACTAGCCGTTAAGAGACTATCTAACTCTCGTCTT

TTTGAAAGGCAGTTCCTTTTAGAAGCAACGATTATGAGTAGGTACAGACACAAAAATATA

GTTCCCATGCTTGGATTCTGCATTGAAGGAAAGGAAAGGCTTTTGGCATATGAATACATG

TCAAATGGAAGGCTTTCCAAATGGTTGCACCCTTTGAAAAGTGAAGTCATCAGACTGAAA

TGGCCTGAGAGGGAAAACATTGCACTTGGAATAGCAAGAGGATTATCATGGCTCCATCAT

AGCTGTGATTTGAGCATAGTGCATTTTAATATATGTTCACAATGCATACTGCTAGATGAG

AATTTTGAACCCAAAATATCCAATTTCGGGCAAGCCAAATTTATGAATCCTAACAATGAG

GATGATCTTGGCAGAATGTTTAAAGTAAACGATGGGAAGAAGGATGTCCATGACTTTGGA

AGTGTGCTCTTTGAACTGATTACAGGGAAAACATATAACGAACTGACTCGTTCATCCACC

ACCACTAATCTCTATGATAACCCTTCAAATTTTTATAATGCCATCGATAAATCTCTGCCC

GGACAAGGATTTGAAAACGGAGTATGTGCTCTCCTAAAGGTTGCTTGTGAGTGTGTTAAA

CCTATAAATCAAAGACCAACAATGCTTGAAGTTTACAACAATTTGAGCAATATAAGTAAG

TCACAATATGTCTCTAGTGATGATTCTAATCCAACCGGTGGATCACAAATTGCTTCTGGT

ATTACTATAGACGAAATTACCGAGTTATAA

>MS.gene40119.t1

ATGGCAAAGTCAGTCAATGGTGGTTACATCACTCAGACAGATAGCAACCACAGTGTTCCT

ACACGTGCACAAATAGCTTCAAAAAGAAAAAAGAAGAACCTTAGACATAAGAAAATAGGA

GAGATGCGTAATCAGAACACCAAACAGGAGAAGATCAAAGAGAAACTAAAAAGGGCCAGA

CAAAAGGACAAAATGTTACCTGTGGAGTTGCTAGATAAAAGAATACAGATCAATAAAGTT

GGACGGTTGGCTTATAAGATGAACTTCACAAAATTCTATCATGCAACAGAATACTTTAGC

AAGGACAATGTCATTGGGGTGGGAGTGACTGGGATAATGTACAAAGCAACACTTTCCAAC

GGTTGTATTTTCGCAGCTAAGAGACTACATGATTCTCAATTTTGTATCAAAAGGTTTGAA

TTAGAAATAATGCTTTTGGGACAATACAGTCACAGAAACATACTTCCCTTGATTGGTTTC

TGCATAGAAGAAGAAAAAAATGAAAGAGTTTTGGTGTATCAATACATGTCAAATGGAAGA

CTTTCTGATTGGTTGAATGATGATACTACAAAATTAGGATGGCCAAGGGTTATTAAAATT

GTTCTTGGGATAGCAAGAGGTTTATGTTGCCTACATTATAGCTTGCATATGGTTCATCTT

AATATAAGCTCCGAGTGTATCTTGCTAGGGGACAATTTTGAGCCGAAAATATCCAATTTT

CGAGGGGCCATGTTTATGCATAAAGATGCGAACAAAAACATAGGATTTGGAAAGAAGGAT

GTTTATGATTTTGGTTGTTTGCTTTTTGAGCTGATTAAGGGGAAGAAATTTGGCCAAACA

AGTGATTGTTTAAGTAATACTAATGTTCCTTTTGCGACGTACACGTATCCTAATCCTATG

AATCTGTTGGAGGACCATTCTGGGTTCTATGATGCAGTGAATGAATCTCTAAACATGATG

GAATTTGAAGATGAGGTATCTGCTCTACTTAGAGTTGCATGTGATTGTGTTCATCCTTTG

TTTGATAAAAGACCTACTATGCTTGAGGTGTACAGAAAGATGGGCAACATATGGGAGAGA

GATGATATTTGTGAAGATCCTGACTTCTTGTCGATCGATTTTGAAATAGAGACCAATATA

CTTATTAATTAA

>MS.gene40127.t1

ATGCATGAATTGCTACATCCACAACTGGTGGAGAAAGGAAGCAAAGAGATCTCTATACTA

ATGGAGGGACTGACTTCAACAATATGGTTGGATGAACTTCGTAACGCAACCGATTGTTTC

GCTGTAGACAATGCTATTAGTGTAGGAAAGATGGGAATGATGTACCAAGGATTTCTGCCT

AATGGAAAAGAAAGGATGTTGGCATACGCATACATGTCAAATGGAAGGCTTTCCAAATGG

TTGCATCCTTTGGAAAGTGAAGTCATAAGATTGAAATGGCATGACAGGGTAAACATTGCA

CTTGGAATAGCAAGAGGGTTATCATGGCTACATCATACATGCAATTTGGGCATAGTGCAT

TTTAATATATGTTCAGAATGCATATTGCTAGATGAGAATTTTGAACCCAAAATATCCAAT

TTTGGAGAGGCCAAATTTATGAATCCCAACATTGAAGATCATTTAGGAACAATGTTTAAA

GTGAATGATGGGAAGAAGGATGTCTACGACTTTGGAAGTGTGCTTTTTGAATTGATGACA

GGGAAAACATATAACGAGTCAGTCCCTCCATTAGTGCTTTAA

>MS.gene40123.t1

ATGGCAACTAATCAGATGCACGAATTGTTACATCCGTGGCTGGCACGCAAAGAAATCAAG

GAGATCTCTGTATTGTTGGGAAGATTGACTTCAACAATATGGTTAGAAGAACTCCGGGAC

ACAACCGACTGTTTTGCTATAGACAATGCTATTGGGGTGGGAAAGATGGGAATGATGTAC

CAGGGAAATCTACCAAATGGTCAGTTACTAGCCGTTAAGAGACTATCTAACTCTCGTCTT

TTTGAAAGGCAGTTCCTTTTAGAAGCAACGATTATGAGTAGGTACAGACACAAAAATATA

GTTCCCATGCTTGGATTCTGCATTGAAGGAAAGGAAAGGCTTTTGGCATATGAATACATG

TCAAATGGAAGGCTTTCCAAATGGTTGCACCCTTTGAAAAGTGAAGTCATCAGACTGAAA

TGGCCTGAGAGGGAAAACATTGCACTTGGAATAGCAAGAGGATTATCATGGCTCCATCAT

AGCTGTGATTTGAGCATAGTGCATTTTAATATATGTTCACAATGCATACTGCTAGATGAG

AATTTTGAACCCAAAATATCCAATTTCGGGCAAGCCAAATTTATGAATCCTAACAATGAG

GATGATCTTGGCAGAATGTTTAAAGTAAACGATGGGAAGAAGGATGTCCATGACTTTGGA

AGTGTGCTCTTTGAACTGATTACAGGGAAAACATATAACGAACTGACTCGTTCATCCACC

ACCACTAATCTCTATGATAACCCTTCAAATTTTTATAATGCCATCGATAAATCTCTGCCC

GGACAAGGATTTGAAAACGGAGTATGTGCTCTCCTAAAGGTTGCTTGTGAGTGTGTTAAA

CCTATAAATCAAAGACCAACAATGCTTGAAGTTTACAACAATTTGAGCAATATAAGTAAG

TCACAATATGTCTCTAGTGATGATTCTAATCCAACCGGTGGATCACAAATTGCTTCTGGT

ATTACTATAGACGAAATTACCGAGTTATAA

>MS.gene40126.t1

ATGGCTTTTCAAATAAAAAGGGATATTCTATTGCAAATACTCTTGTGCCTCACTATGTTA

ATGACTAGTCAAAGTACTGAGACTGATATCTTCTGTTTAAAATCCATTAAGAATTCCTTG

GAAGATCCAAATGGTTACTTACAAAACTGGGACTTCAACAACGGAACAGAAGGTTTCATA

TGTAAATTTACTGGTGTTGAATGCTGGAATCCAGATGAAAATAGAGTACTAAATCTCAAA

CTATCTAACATGGGGTTGAAGGGTCAGTTTCCACGGGGACTTGAAAATTGCTCTTCGTTG

ACGGGCTTAGACCTTTCGCTCAATGACCTCTCGGGATCCATCCCATCTGATATTTCAAGG

ATACTCACATTTGTTACCAACGTTGATCTCTCTAGTAACAAATTCACCGGTGAAATTCCA

ATAGGTTTAGCAAATTGCAGTTACCTGAATAATCTTAAACTTGACCAAAATATGTTAAGC

GGTGAAATTCCACCACAATTTGCCGGACTTACGAGGCTTAAAACAATTTCTTTTTCCAAC

AATCAATTGTATGGGCCAGTGCCAAATTTCAAAACAGGATTAGATGTTGCTAGCTATGCT

AACAATAGTGGACTTTGTGGAGCGCCTTTGAGACCTTGCTCCTTGACACGTGATAAGCAC

GGCAATTTCCATCAATCGTTCAAGAGTGGTCTTATTGTTGGTTATGTTTTTTCACTAATT

TTTTCTGTTACGCTCACTTATATCTTCTACTCCAAATGTGAACATTGGGTGCATCAATCG

AAAAAGAAGAAAAACAATCACCTAAACAAAGCCATAGAATTGGGCAAGCATATTATCTCC

ATCACCAGCAGGAGAACACAGATGGTAGCTGATCAGATGCACCAATTGTTACATTCGTGG

ATTGTACACAAAGAGACCAAAGAGATCTCTATACTAATGGAGAGACTGACTTCAACAATA

TGGTTGGAAGAACTGCGTGACGCGACCGATTGTTTTGCTGTGGACAATGCCATTGGGGTA

GGAAAGATGGGAATGATGTACCAAGGATTTCTGCCTAATGGTCAATTACTAGCTGTTAAG

AGACTATTTGACACTCGCCTCTTTAAAAGGCAGTTCCTTTTAGAAACAGCAATTTTGTGC

AAGTACAGACACAAAAATATAGTTCCCATGCTTGGGTTCTCCATTGAAGGAAAGGAGCAG

GTTTTGGCATATGCATACATGTCAAATGGAAGGCTTTCCAAATGGTTGCATCCTTTAGAA

AGTGAAGTCATAAGATTGACATGGCATCAAAGGGTTAACATTGCACTTGGAATAGCAAGA

GGATTATCATGGCTGCATCATACATGCGATTTGGGCATAGTGCATTTTAGCATATGTTCC

GAATGCATATTGCTAGATGAGAATTTTGAACCAAAAATATCCAATTTTGGAGAGGCCAAA

TTTATGAATCCCAACATTGATGATCATCTAAGCACAATGTTTAAAGTGAATGACGGGAAG

AAGGATGTCTACGACTTTGGAAGTGTGCTTTTTGAATTGATGACAGGGAAAACATATAAT

GAATTGTCGCGTTCATCTGACACTACTAATCTATGTGGTAACCCTTTGAGTTTTTATAAT

GTCATTGATAAATCTTTGACCGAGGAAGGACTTGAAAATGAAGTTTGTACTCTCATCAAG

ATTGCCTGTGAGTGTGTTCACCCTTTTCCAGACCAAAGGCCAACAATGCTTGAAGTTTAC

AACAATATGAGCAACGTTAGGAAGGGACCAAATGGTTCTGGTGATGATTCTGACACATTA

AGGAGATTAGAATATGCTTCTTCTATTCCTATAGATGAAATCGTCGAGTTTTAA

>MS.gene40128.t1

ATGACTAGTCAAAGTGCTAATAATACTGATATCTTCTGTTTAAAATCCATTAAGAATTCA

TTGGAAGACCCAAATAATTTCTTACAAAACTGGGACTTCAACAACAGAACAGAAGGTTTC

ATATGCAAATTTACTGGTGTTGAATGCTGGCATCCAGATGAAAATAGAGTACTAAATCTC

AAACTATCTAACATGGGGTTGAAGGGTCAGTTTCCACGGGGACTTGAAAATTGCTCTTCG

TTGACGGGCTTAGACCTTTCGCTCAATGACCTCTCGGGATCCATCCCATCTGATATTTCA

AGGATACTCACATTTGTTACCAACCTTGATCTCTCTAGTAACAAATTCACCGGTGAAATT

CCCACAACTATAGTAAACTGCACTTACCTTCACACTCTTAAACTTGAGAACAATATGTTA

AGCGGCGAAATTCCAAAAGGACTTGGCCAACTTAGTAGAATTAAAACAATTTCTGTTGCT

AACAATCAATTGTGTGGGCAAGTGCCGGTGTTCGACAACTTCAGTGAAGGAGATTTTAAC

TATGCTAACAATAGTGGAGTTTGTGGCAAGCCCAACGATTTCCTCCAATCATTCAAGAGT

GGTCTTATTGTTGGATATGTTTTTTCACTCACATGTTATGTTATGCTGACATGTATGTTC

TACTCCAAATGTGTGCAACTTACGAAGAAAGAAAACAATCACATAAACAAAGCTAATGAA

TTTGGCAAATGTATTTGCTCCATCGTCAGCAGGAGGTCAAGAGTGGTAGCTAATCATGTG

CACGAATATTTACATCCGTGGCTAACGCACAAAGAAAGCAAAGAGATGCATCCACAAATC

ACTGCATTATTGGAAAGACTGACATCAACAATATGGTTCGAAGAACTGCGCGACGCAACT

GACTGTTTCGCAATGGACAGTGCCATTGGGGTAGGAAAGATGGGAATGATGTACCAGGGA

AGACTACCCAATGGTCAGTTACTAGCTGTTAAGAGACTGTTTGACTCTCAGTTGTTTAAA

ATGCAGTTCCTTTTAGAAACAACGATTATGGGTAGGTACAGACACAAAAATATAGTTCCC

ATGCTTGGATTCTGCATTGAAGGAAAGGAAAGGCTTTTGGCATATGAATACATGTCAAAT

GGAAGGCTTTCCAAATGGCTGCACCCTTTGAAAAGTGAAGTCATCAGACTGAAATGGCCT

GAGAGGGTCAACATTGCACTTGGAATAGCAAGAGGATTATCATGGCTCCATCATAGCTGT

GATTTGGGCATAGCGCATTTTAATATATGTTCAGAATGCATATTGCTAGATGAGAACTTT

GAACCCAAAATATCCAACTTTGGAGAGGCCAAATTTATGAATCCCAACATTGAGGATGAT

CTTGGCATGATGTTTAAAGTAAACGATGGAAAGAAGGATGTCTATGACTTTGGAAGTGTG

CTTTTTGAACTGATTACAGGGAAAACATATAACGAACTGAATCGTTTATTCACCACCATC

ACTAATCTCTATGAAAACCCTTCGAATTTTTACAATACCATTGATAAATCTTTGACTGCT

GAAGGTTTTGAAAACGAAGTATGTACTCTCCTAAAGATTGCTTGTGAGTGTGTTAAACCT

ATATACCAAAGGCCAACAATGCTTGAAGTTTACAATAATTTGAGCAATGTAAGGAAGACA

CAGTATGGCTCTAGTGATGATTCTAACTCAGCCGGTGTATCACAAATTGATTCTGGAATC

ATTGCAGATGAAATTACCGAGTTATAA

>MS.gene40129.t1

ATGAGTTTCAACCACACATTTTCACTTTTTAGCCTCTTCTTGACCATTTTATCCCTTTGG

TTTTCATTCTCCAATGCACAAAACTTACCTCAAGATTTTCTCGACGCCCACAACACAGCT

CGTGCACAAGTTGGCGTTGCAAACATTACATGGGACAACACAGTTGCTACCTATGCTTTG

AACTATGCAAATTCAAGGAAAAGTGATTGCAATATGGTTCACTCAAATGGACCTTACGGT

GAGAATCTTGGCAAAGGCTCAAGTGGTACATTCACTGGAGTTACTGCGGGTGTGCTAGGG

TACAATGTACCAATGGATGGTGGTTTGTCACTTGCAACTATGATCCCCCGGAAAATGAGG

CGCGGATTTATTACAGAATCATGCACGGATGATTCTACCGAAGAAAACACCATTTTGGAA

AAGACGAAGAAGAGAAGGCAGTATACAAATGAAGTAATTGAAGACAGGCTGAGTGACTTA

CCCGACGGTGTTATTCTTCACATTTTGTCATTATTGGATACCAAACAGGATGTGAGAACT

TGTGTTTTGTCAAAGAGATGGGAACATCTCTGGAAACGTATTTCAACCCTTATTTTGCAT

TCTTCTAGATTTTCCTATGTTAAGCAATTTACAACATTCGTGTCTAAGATTTTGACTCTT

CGTGATACCTCAACTGCATTGCGCGTTCTTGATGTTGACCGTAATGGTAATTTCGAACCT

CGACTCCTTAAAAGGATTTTAAACTACATTTCCTCCCATAGTACCCACCTCCAGGAATTA

GGTATCTCTGTCCGTGGTGATAGTTCTCTCATTTTGAGCTGCTTTTCTTCTTGTCGGGCT

CTTACATCTCTTAAGCTTTCAATTTACCCTAGAACTGATTATACCGAAACATTATTTCCA

AAATCGTTGAATTTGCCTTTATTGACCAGCTTAGATCTAACAAACTTCGCCTTCTGTGGT

AGTGAAAGCGGTTGTGCTGAGCCATTTTCCGTCTTTACCATGTTGAATAGTTTGGTCCTT

CGTAGTTGTAAGGTAAAGGATGCTCAAATCCTCACCATATCAAGTGATACACTTGCCAAT

TTAGCTATACATAGTCCTGTTGGAGAGATTAATTTGGCTATACATGATAATTCGTCCAAC

ATGCCCAAAATTGAGCTATCGACCCCAAGTCTCTATACATTTACATACGACGGTAGTCTT

ATTCAGGAAATATGTGGGAGCGGACTTTCTTCTGTTAAACAGGTACATATCGATGATTCA

CGAGAATTTTCAGCTTCAGTGAGGCATGGTTTGCTTCTATTCAACTGGCTGCTAGACTTT

GCCAATGTAGAATCATTAGCACTCTCTTCAACTACTCTTCAGATTCTCTCCTTGGTTCCT

GATTTATTGGAGGTTAAGCTCCCTTCCTTATGTAACTTGAAGTCATTGGAAGTAGAATTG

GTACCATTTATTCACCGCGATGGATTTTTATTCCGATCAATCGAAGCTGCCATGTTAAAG

AAAGCTGCTGCCAAGTCTCGTAAAGAAGTTGCTAAGTTAAGAAAGGCATTCAAAGCACGT

TTGGAACCACGTGCCATACCTGACGGTATGGTTGACTTCTTGCGACAAAACTCGCCGTCG

GCGGAAGTTAACATCACCACAGATTTCTCGCATGTTTTAAATCCTAAGCAGGTTGCAGAA

TCTATAAAGGGCGCGAAGATCATCAGCTATCGTTCGCGATTTTCCAAACGGCTCAGGCCT

GTCCCCTTCCATGCCGCACCTGCTTCTGGTACTGAATATGATTCTGCCGCTGCTCTTGCT

TCTGTCTCCATGACTGCTCCTGACGCGGTGCCTGCCTCTGCTGCACCTCCCAATCTTCAT

CTCTGCCGCGCTGAAAAGGATGATAAATCATCAAATGAAGATGAGGTGGAGAAGCGCCAA

CCTAACACCGACTCTCCACTTCTAGATAATGGCCAGTGA

>MS.gene40125.t1

ATGGCTTTTCGACTAAATAGGGATTTCATATGTAAGTTTGATGGTGTTGAATGCTGGCAA

TCAAGCTATGATAGTAAAGTACAAAATCTCAGACTATCTGGCATGGGGTTGAAGGGCCAA

TTTCCAGGTGCCGTTCAGAACTGCTTTTCACTCGTGAGCCTAGATTTTTCATTCAATGAA

CTCTCAGGACCAATCCCATCTAAAATATCAAGTATACTCAAATATGTTGTTAGCTTTGAT

CTCTCGAATAACAAGTTCACTGGTGAAATTCCAGCATCATTAGCAAGTTATACTTACCTG

AATACCCTTAAACTTAACAACAATATGTTAAGTAGTGAAATTCCAAGACAACTTGGCACG

CTTACCAGGCTTAAAGAAATTTCTTTTGCTAACAACTATTTGTCAGGGCTAGTGCCTGTG

TTCGCAGGAGGAGGGGTAGATTTTAACTATGCTAACAATAGCGAACTTTGTGGAGAATCT

TTGAGACCTTGCTCCTTGAGACATAACAAGCCCAACCATTTCCTTCAATCGTTCAAGAGT

GGTCTTATTGTTGGATATGTTTTTTCACTGACAAGTTCTGTTATGCTGACTTGTATCTTC

TACTCCAAATGTGTGCAATTTAAGAAGAAGGAAAAAAAACAATCACGAATGGCTTTTGAA

ATGGAAAGGGATACTCTATTCGAAATATTCTTGTGCCTCGGTATGTTAATGACTAGTCAG

AGTACCGATACTGATATCTTCTGTTTAAAATCCATTAAGAATTCATTGGAAGACCCAAAT

AATTTCTTAGAAAACTGGAACTTCAACAACAAAACTGAAGGTTTCATATGTGAATTTAAA

GGTGTCAAATGCTGGCATCCAGATGAGAATAAAGTACTAAATCTCAAACTATCTAACATG

GGATTGAAGGGTCAGTTTCCACGTGGTCTTGAAAACTGCTCTTCAATTACAGGCGTAGAC

CTTTCAATCAACAAGCTATCGGGATTCATCCCATCTGATATATCAAGTATACTTTCGTAT

GTTACCAGCATTGATCTCTCTGGTAATAAATTTACTGGTGAAATTCCAATAGATTTTACA

AATTGCACTTACCTGAATACTCTTAAGCTTGATGGTAACATGTTAAGCGGTCATATCCCA

AAAGAATTTGCCATGCTTAACAGGCTTAAAGTGATTACTTTTTCTAATAATAATTTGTCA

GGGCCAGTACCGATGTTCCAAAGAGTAGTAGTTTATAATTATGCTAATAATGATGAACTT

TGTGGAGGAGTGTCTTTGGCCCCTTGCTCCGTTGGCAAGTTCCATCAAGCATTAAAGGGT

GGCCTCATTGTTGGTTTTGCATTATCATTCACTTCTTATATTGTGGTTACTTTTTACATC

TCCTACTCCAAGGGTGTACCTCGCATGCAATCAAAGAAGAAGAGGAATAGAAACAGCCTC

CTAAATAAAGCTAAAGAGTTGGGCAAATATATTTATTCCATCACGAGCAAGACGACACAA

AGGATATCTAATTATATGCACGAACTGCTACATCCACAGTTGGTGGAGAAAGGAAGCAAA

GAGATCTCTATACTAATTGAGAGACTGACTTCAACAATATGGTTGGAAGAACTGCGTGAC

GCAACCGATTGTTTCGCAGTGGACAATGCCATTGGGGTAGGAAAAATGGGAATGATGTAC

CAAGGATTTCTGCCTAATGGTCAATTACTAGCTGTTAAGAGACTATTTGACTCTCGCCTC

TTTAAAAGGCAGTTCCTTTTAGAAACAGCAATTTTGTGCAAGTACAGACACAAAAATATA

GTTCCCCTGCTTGGGTTCTGCATTGAAGGAAATGAAAGGCTCTTGGCATACGCATACATG

TCAAATGGAAGGCTTTCCAAATGGTTGCATCCTTTGGAAAGTGAAGTCATAAGATTGAAA

TGGCATGACAGGGTAAACATTGCACTTGGAATAGCAAGAGGGTTATCATGGCTACATCAT

ACATGCAATTTGGGCATAGTGCATTTTAATATATGTTCAGAATGCATATTGCTAGATGAG

AATTTTGAACCAAAAATATCCAATTTTGGAGAGGCCAAATTTATGAATCCCAACATTGAA

GATCATTTAGGAACAATGTTTAAAGTGAATGACGGGAAGAAGGATGTCTACGACTTTGGA

AGTGTGCTTTTTGAATTGATGACAGGGAAAACATATATTGAATTGTCGCGTTCATCTGAC

ACTACTAATCTATGCGGTAACCCTTTGAATTTTTATAATGTTATTGATAAATCTTTGATC

GGGGAAGGACTTGAAAATGAAGTTTGTACTCTCATCAATATTGCCTGTGAGTGTGTTCAC

CCTTTTCCAGGCCAAAGGCCAACAATGCTTGATGTTTACAACAATATGAGCAACGTAAGG

AAAGGACCAAATGGTTCTAGTGATGATTCTGACGCATTAAGGAGATTAGAATATGCTTCT

TCTATTACTATAGATGAAATTGTTGAGTTTTAA

>MS.gene40124.t1

ATGAAGTCCTCAAATATTCTCGTAATCTTAACCATTTTCTCTATGTGCTGTTTCCTTTGC

TTGGCACACAACGCCCCTCAAGACTACCTTGACGTGCACAACAAGGCTCGTGCCGAGGTT

GGCGTTGGCCCACTCGTGTGGAACGAAACCCTTGCAGCCTATGCCATGAACTATGCTAAA

TCGAAGCACGAAACCTGCGAAATGGTGCACTCCCAAGGGCCTTATGGCGAGAACCTTGCA

GAGGGTTCCGACCCCCAAATGACTGCTGCCGCTGCTGTGAAATTATGGGTGGATGAGAAG

GCCTTCTATGACTATAGTACCAACGCGTGTGTTAAAGATGAGTGCAGACATTATACACAA

GTGGTTTGGAGCAATACCAAGCAGCTTGGTTGTGCTAGAGAAAGTTGTAAGAATGGATGG

ACATTTTTCATTTGTAGCTATTACCCACCAGGAAATTATGTCGGCGATAAACCTTACTAG

>MS.gene40131.t1

ATGAGCTGTGTTTCTTCATGCCGGGCTCTTACATCTCTTAAGCTTGATGTTGCCTCTAGA

GGTCGTCATAATATTGGACAAACATTATTTCCAAAATCTTTGAATTTGCCAACATTAACC

AACTTATATCTAACAAATTTCGACTTTTGCGGTGATGAAGATGGTTGTGCTGAGCCTTTT

TTGGCCTTTCCCAAGTTGACTACTTTGGTCATTCGTAGTTGTAAGGTAAGGGATGCACAA

ATCCTCAGGATATCAAGTGAAACCGTTGTCAATTTTAAAATGCGCAATTATTCAGAAGAC

TTTGCAAAAATTGAGTTATGTGCTCCAAGTCTTTGTACCTTTACTTTTACTGGTACTCCT

GTTCAGAAAATATGTGGGAGTGGTCTTTCTGCTGTCAAACAAGTAAAGATTAAAGCAGAT

ATGTTTTCAAGATGGGATGAGCCTCCTATGATTCTATTCGACTGGCTGCTAAACCTTTCC

AATGTAAAATCATTGATGGTCTCTTCAACTACTCTTCGGATTCTCTCATTAGTTCCTGAT

TTACTAGAGGTTAAGCTGCCTTCTTTGTGTAACTTGAAGTCTATGGAAATAAAACTGGAA

ACCTGTAGTAATCAAATGGCATTACCCAACCTGGTGAAAGAAACCATGTTAAAGAAAGCT

GCTGCCAAGTCACGTAAAGAAGCTGCCAAGTTAAGAAAGGCTTTTAAAGCACGTTTGGAA

CCACCTCCCATACCTGATGGAATAGTTGACTTCTTGCGACAAAACTCGCCATCCGCCGAA

GTTAACATCACAACAAATTACCCGGGTTGTTTTAATCTTAAGCAGGTTGAAGAATCTATA

AAGGGTGCAAAGGTTATCAACTATCATTCGCAATTCGCCGTGCCTGCCTCCTCTGCTGCA

CCTGTTCATGCTGCTGAGTCTGCTTCTGCCACCGTGCCTGCCTCGACATCACCTCCCAAT

CTTCATCTATGTTGCGCTGAAAAGGAACCTGTTTGGCCTCGTTTAAAGAATATTTATAAG

AGGATGGGACAGTATTAA

>MS.gene40130.t1

ATGACACAATTTCCCAGTTGCCGCAACGTTCCAACTTTCAATCCATCGGAGAACTCACAA

CTCGACCACGACATCGAGATATACAAAATCGTTGGAATGGATTGGACGTACCAGATTGAT

TCTCACGAAGAAAACCTTATTCTAGAAAAGTTGAAGAGAAGAAGGCAAAGTGGGAATAAT

GAAGTAACTGATGAAAGTGAAGACAGGCTAAGTGACTTACCTGATTTTCTTCTTCTTTTT

ATTTTATCATTTTTGTACACCAAACACGCTGTGTCCAAGAGATGGAAACATCTCTGGAAA

CGTATTCCAACCCTTACATTGCATTCCTCAAGATTTTCCAATGTGGAGAATTTTGACATG

TTCGTTTCTAAGATTTTGACTCTTCGTGATACCTCAACTGCATTGCAGGCTTTTGATCTT

GACCGTCGAGGTAAGATTGAGCCTCAAGTCCTTAAAAATGTTTTAGACTATGTTTGCTCC

CATAATACCTACCTCCAGGAATTAGGAATCTCTGTCCATGGTGATAGTTCTCTCATTCTG

AGCTGCGTTTCTTCTTGTCGGGCTCTTACATCTCTTAAGCTTTCAGTTCACCCTAGAGAT

AATAATGGTAGTGATACTGGAATATTATTTCCAAAATCTTTGAATTTACCTGCACTGACC

AGCTTAGATCTAACGAATTTTGCCTTTTGCGGTGATGAAAACGGTTGTGCTGAGCCTTTT

TCGGTCTTTACCAAGTTGAATGGTTTGGTCATTCGTAGTTGTAAGGTTAAGGATGCACAA

ATCCTCAATATATCAAGTGAGACACTTGTAGATTTAGCAATGCATGATAACTCGCGTGAC

TTTGCCAAAATCGAGTTATCTACTCCGAGTCTTTGTAACTTCACTTTTTGTGGTAGACCT

GTTCAAAGAATACGTGGTAGCGGTATTTCTTCTGTTAAACATGTAAGTATTGAAGCTGAA

ATGTATTCAGTAAGGGAGAAGTATCCTTGGATTCTATTCAACTGGCTGTTAGGCTTTGCC

AATGTAAAATCATTGAAAGTCACTTCAAGTACTCTTCAGATTCTCTCCTTAGTTCCTAAT

TTATTGAAGTTTAAGATACCTTCTTTGTGTAAGTTGAAGTCACTGGAAATAGAACTGGAA

TCACTTCAAATGGGATTACCCGACATACTGATGGAGTCCATGTTAAGGAAAGCTGCTGCC

AAGTCACTTAAGAAAGCCGCCAAGTTACGAAAAGCTTATGAAGAAGGTTGGAGACCACCG

CTCATACCTAATGGAATGGTTGACTTCTTGCTACAGAACTCGCCTTCGGCGAAACTTGAA

ATCTCGATGCGTTCAAATCCATCGAAAAAATCACTACTCAACGACGACAGCGAGAAAATG

AGGCGCGCGTACGATTCTTCCAAGAAAATCAGGATTCAAGAAAAGATGAAAAAAATAAGG

CAATGTGAGACTGAAAATGAAGGAAGTGAAGAAAATGAAGACAAGCTAAGTGACTTACCT

GAGTGTGTTATCCTTCACATTTTATCATTTTTGGACTCCAAACATGCCGTTCAAACCTGT

GTTTTGTCCACAAGATGGAAGCATCTTTGGAAACGTATTCCTACCCTTATATTACATTCC

TCAAACTTTTCAACTGTCAAGAAATTTGCCATATTCGTGTCTAACATTTTGACTCTTCGC

GATAGCTCAACTTCGCTGCATGCTCTCAATCTTGATCGTCGTGGTGATATTGAACCTCAA

CTCCTTAAAAAGATTTTAAATTATATATGCTCCCATAATACCCACCTCCACGAATTAGGA

ATCTCTCTCCGTCGTGGAAGTGATCTAATTCTGAACTGTGTTTCTTCATGCCACACTCTT

ACATCTCTTAAGCTTTCACTTGACCCTAGAAGTAGTCGTCGTTATTTTATTTCTGAAGAA

ACATTATTTCCAAAATCTTTGAATTTGCCTTTGTTGACCAACTTAGATTTAACAAATTTA

GTCTTTTGCGGTAGTGAAAGCGGTTGTGCTGATCCCTTTTTGGCCTTTCCGAAGTTGAAT

AGTTTGGCCATTCGTTGTTGTAAGATAAAGGATGCTCAAATCCTCAACATATCAAGTGAG

ACACTTGTCAATTTAGCTATACATTATCCTTTGGAAAAAATGAAGTTATCTTACCATGAT

ACTTCATCCAAATTTGCCAAAATAGAGCTATCTACTCCAAGTCTTTGTACATTTACTTTT

ATTGGTAGTCTTATTCAAAAAATATGCGGGAGCGGACTTTCCTCTGTTAAACAAGTAAAT

ATTGATGATTCACATCAGCTTTATGCTTCAGCGGGGAATGGTTTGATTCTACTCAGCTGG

CTGCTAGACTTTGCGAATGTAGAATCATTTAGAGTCACTTCAACTACTCTTCAGATTCTC

TCCATAGTTCCTGATTTATTGAAGGTTAAGCTCCATTCTTTATGTAACTTGAAGTCATTG

GTAATAGAATTGATACCATTTCATGATGGATCTTCATCCCGCTCAATCGTAGATGCCTTG

TTAAAGAAAGATGCTGCCATGTCACGTAAAGAAGTTGCTAAGTTAAGAAAGACATTTAAA

GCTCGTTTGGAACCACCTGCCATACCTAATGGAATAGTGGACTTCTTGCGACAAAACTCA

CCGTCTGCGGAAGTTAACATCTTTACAGATTACCCGGATTGTTTTAATCTTAAGCAGGTT

GTAGAATCTATAAAGGGCGCTAAGATCATCAGCTATCGTTCCAAACTTGCCGCGTCTGCC

TCCTCCTCTGCCCCACCTGCTTCTGCTGCTGAATATGCTCCTCCCACTGCTCCTGCTTCT

GCCTCCGTGCCTGCTTCTGCCGCTGTGCCTACCTCTGCTGCACCTTCCAAACTTCATCTC

TATATCGCTGAAAAGGATGATAAATCATCAACTGAAGATAAGAAGGAGAAGCGCCAATGT

AACACCGACTCTCCACTTCTAGATAATGGGCAGTGA

>MS.gene40136.t1

ATGATTCTTCGAAGTATGAAGAGAAGAAGGATCTATGAGGTTAAAAATGATAAAAAGGCT

GAAAATGGAGAAATTGAAGACAGGCTAAGTAGTTTACCTGATGGCGTTCTCCTTCACATT

TTGTCATTTTTGGACGCCACATATGCAGTTCAAACTTGTGTTTTGTCCACAAGGTGGAAG

TATCTTTGGAAACTTATTCCAACTCTTAATTTGCATTCTGAAAGATTTTATACTGTTAAG

CAATTTTCTAAATTTGCGTCTAAGATTTTGACTCTTCGTCTTCGTAAAAAGTCAGCTGCT

TTGGGTGCCCTTGATCTTGACTGTAGAGGTAATATTGAGCCTCAAATCCTTAAAAAGATT

TTGAATTATGTTTCCCCACATAATACCCACCTCCAGAAGTTAGAAATCTATGGCTATGGG

GATTCTAGTCTCATTATGAGATGCGTTTCTTCATGCCAGGCTCTTACATCTCTTAAACTT

GGTCCTATTAATTTTTCTAGAACAATCTTTCCAAAATCTTTGAATTTGCCAGCATTGACC

AGCTTAAATTTAAACACATTTGTCTTTTGTGGTGGTGATGACAATTGTGTTGAACCCTTT

TCCGCCTTTACCAAGTTGAATAGTTTGGTCATTGATAGATGTGCGATTAGGGATGCACAA

ACCCTCAGGATATCAAGCGAGACACTAGTAAATTTAGCTATGCATGAAAGTTCATATGAA

TTTGACAAAATTCAGTTATTTACTCCGAGTCTTTGTACCTTTACTTATACCGGTCATCCT

GATCAGAGAATATGTGGGAGTGGTCTTTCTTCTGTTAAACAAGTAAATATTGCTGCAGAA

ATATATTTTTGTTGGGAAAAGCCTGCTAGGCTTCTACTTAGTTGGCTGCAAGATCTTGCC

AATGTAAAATCATTGACAGTTTCTTCAACTACTCTTCAGATTCTCTCCTTAATTCCTGAT

TTATTAGAGGTTAAGCTCCCTTCTTTGTGCAACTTAAAGTCTATAGAAATAAAATTGGAA

CCGCTTGCAGACTATACAACTTTATTTGATGAATTGCAACGTGTCATGTTAAAGAAAATT

GCTGCTAAGTCAATGAAACAGCGTATAGAACGTTTGGAACCACCTCCCATACCAGATGGA

ATAGTTGCATTCTTGCTTCAAAACTCACCGTCCGCAAAAGTTGACATCACAACAGAGTAC

CCAAATGCTTTTAAGATTAAGCAGGATGATAGATCATCAAATGAAGATAAGGTGGAGAAT

TGCCAACCTAACGTAGCAACTGCTTGA

>MS.gene40134.t1

ATGCTTCCCAAAAAGGAAATTGGGAATATCTCTGTTTTTCTCGAAGAGAGCAGGATTTGT

CGAAAGACGAAGAAGAAGAAAAGAAGACTCTATGAAGTTGAAAATGATGAAAATGAAGAC

AGACTGAGTGACTTACCGGACGGTGTAATCCTTCACATTTTGTCATTTTTAAATACCAAA

CATGTTGTGAGAACTTGTGTTTTATCTAAGAGATGGGAACATCTCTGGAAACGCATTTCA

ACCCTTATATTGCATTCTTCTAGATTTTCCTCTGTTAAGCAATTTGCCACGTTCGTGTCT

AAGATTTTGACACTTCGTGATACCTCAACTGCATTGCGCACTCTTGATCTTGATCGTACG

GGCAATATTGAACCTCAGCTCCTTAACAAGATTTTAAACTATGTTTATTCTCATAATACC

CACCTCGAGGAATTAGGGATATCTCTACAGGGTGAGACTTGTCTCATTATGCGTTGTGTT

TCTTCATGTCAGGCTCTTACATCTCTTAGACTTTCAATTTCCCCTAGAGGTGGTATATAT

AGTAGTTCTAGAATATTATTTCCAATGTCTTTGAATTTACCGGAATTGACCAACTTATAT

CTATCAAATTTCACCTTTTGTGGTGGTGAAAACGGTTGTGCTGAGCCTTTTTCTGCATTT

ACCAAGTTGAATAGTTTGGTCCTTGGTGGTTGTGAGCTAAAGGATGCTCAAACCCTCAAC

ATATCAAGTGAAACACTTGTCAATTTAGCTATGCGTGGTATTTCATCAAACTTTTCCAAA

ATCGAGCTATTTACTCCAAGTCTTTGTTCCTTTACTTTTACCGGTAGACCTTTTCATAAA

ATATGTGGGAGCGGTCTTTCTTCTGTTAGACAAGTAAATATTGCTGCAGTTATGTATTCA

ATCGGGGATGAAGCTCCTATGGTTCTATTCAATTGGCTGCTAGAATTTACCAATGTAAAA

TCATTGATTGTTTCTTCAACTACTCTGCAGATTCTCTCCTTAGTTCCTGATTTATTGGAG

CTCGAGCTCCCTTCTTTGGGTAACCTGAAGTCAATGGAAATAAAATTGGAACCACCTGAA

GTTCAATTGGGATTGCCCTTCATATTGAAAGATGCCATGTTAAAGAGAGCTATTGCCATA

TCACGTAAAGAAGCTGCAAAGTGTTCCTCCTCTGTATCACCTCACACGATCAGCCCCATT

CCATCCACTCGTGTAATCATGTACTTTTTTATGCTGTTTTCTGTATTGAAGGTTAATGTT

GGAGTAGTGGGTCGAGTCATTCCAGCAACATCAATGCTCTTTCCCATTGTTTTGCATCAC

AACCACTCCCTCTTCTCTCACACTGTACAGAACAAAGTCTCTCTCACGCTTCCCAAGAAA

CTCACCTCCAACGGTACTCTATACGACGACGTTTACGGTGGTCCTCCAAAGTTCACCGGT

TCTTCCATCTCGCCGAGGTTTGAAGACTACGGCGAGATTTTCTCCAGCTTTCATGCCGGA

CGGTCTTCTTCCATTCCGATGTTGGATCTTCCGGCGGTTGACGCCGGTGAGGTTTTGTTT

GACTTCCGGCGACACGCTTTTGACTATACTGAGGTGTTTGGTGGCTCCGGTGGACTCGAT

TTTTGGTCCTCGCATGAAGACTTGTTTCGTGAAGGTGATTCTGACGAAGAAGATGATGAA

GTTTGGAGTCCAGAAGAAACCGATTCGTTCTCGGGTGACTTGGGCAATTTTGTATATAAT

CAAGGTGTATCAAGTGGAGGTGTTTTCCAGTCTGTTGTTGATGGGAATACCGATGTTAAC

ATTTCGTATCATATTGAGTGGTTAAATCTCGATACAGTCTGTGACTTGTATGGTGACCTC

GAGTTTCACCAGAATAGACCAGAAGGTGTTGTTTATGATGAACAGTTGCGAAAATTGATG

TTGGTTTCTGGATGA

>MS.gene40133.t1

ATGGTAGAGTCCGACTCACAAGTGGCCCTTAGTTGGGTTAAGAGGAGTGAGGATTGTCCA

TGGTCAATGCGGTTCTTCAGTGACAAACTGCGTAATTGCTTGGCAGCACTGCATCAGGTA

GAGTTTGTGCATGTAAACAGAGAAATGAACTCATTTGCAGATGCTTTAGCAAAAAGTGGC

GTGAATATGGAGTGGCAGAGAATTTGGCTGCCTGAGGATTGA

>MS.gene40135.t1

ATGTTGAGTACACTTCTATTCCTCATGGTTTTACCGATTGCTGCAGCTTTCCAGTTCAGC

TCACCAAACCAGTCATTAGCAACTGATCAAGAAGAAGAAAATGAGAATAATGAAGAATAT

GAAGATAGGCTGAGCGACTTACCTAACAGTGTTATCCTTCATATTTTGTCATTTTTAAAC

ACCAAAGATGGTGTTCGAACTTGCGTTTTGTCTAGAAGATGGAAAGATATATGGAAACAT

ATTCCTACTCTTGTATTGGATTCCTCAAGATTCGACACTGTTAGGCAATTTGAAATATTC

ATGTCTAAAATCTTGACTCTTCGTGATAACACAATTGCACTGCACTCTCTTGATTTTGAT

CATATAGGCAAAATGGAGCATCAACTACTCCAAAAGATTTTAGATTATGTTTATTCCCAT

AAAACCAAACTCCAACGATTAAGAATTTTTGTTCATAATGATAATGGTCTCATTATGCAA

TGTGTTTCTTCATGCAAGAATCTTACATCTCTGCGACTTTCGATTTACCCTAGAATGAGT

TTTTGTTTGAAGACAATATTTCCTAAATCCTTGAATTTACCAGCATTAGAAACGTTGGAT

CTAAGTAATTTCACCTTTTACGGTGGCGAAAACGGCTATGCTGGGCCCTTTTCGGGCTTT

ACCAAATTGAAGAGTTTGATCATTCGTGGTTGTACGGTAATGGACACACAAATCATCACC

ATATCAAGTGAGACACTTGTCAATTTTGCTACGCATTATAGTTCACCTAAAATTGCCAAA

ATTGAGCTATCCACTCCAAGCCTTTGTACCTTTACTTTTTACGGGATTCCACATCCGAAA

ATATGTGGAAGCAATCTTTCTTCTGTTAAAGAAGTCAAAATTTATGCACACATGGACACA

TCTTTGGAGAAGCTTCCTATGGTTCTATTCAACTGGCTGCAAGAGCTTTCAGGTGTAAAA

TCATTGAAAGTCTCTTCAATTACTCTCCAGATTCTGTCCTTAGTTCCTGATCTATTGGAG

GTTAAGCTCCCTTCTTTATGTAACTTGAAGTCGGTGGAAATAAAGATTCAACCACTTAGC

TTCGCTTTATCTTGCATACTAAAAGATGCCATGTTAATGAAAGCTGCTGAGAAGTCACCC

GAAGAAGCTGCCGATTTACGAAAAACATTCGGAGCTGGTTTGGAACCACCTTCAATACCT

GATGGAATTGTGGACTTTCTACTTCAAAACTCATCAACGACCAAAGTGAACATCATATAA

>MS.gene40137.t1

ATGAAGAAAGCAAAGCACTGTGAAAACGAGAATGAAGATAGATTGAGTGATTTACCCGAT

TGTGTTCTCCTTCACATTCTGTCATTTTTTAATACTAAACATGCCGTTCAAACTTGCATT

TTGTCCACAAGATGGAAGTATCTTTGGAAACGTATTCCCACCCTTACATTGCATTCTTCG

GGATTTTCCACTATGAAGAGTTTTTCCACATTTGTGTCTAAGATTTTGACTCTTCGTGAT

AGCTCAACTTCTCTGCATGCTCTCGATCTTGACTGTAATGGATGTAATGGTAATATCGTC

CCGTCTCAACTCCTCAAAATGATTTTGAA

>MS.gene40132.t1

GAATCACTGTTAATGGTGATACTAGTCTCATTATGAGCTGTGTTTCTTCATGCCGGGCTC

TTACATCTCTTAAGCTTGATGTTGCCTCTAGAGATGGTTGTGCTGAGCCTTTTTTGGCCT

TTCCCAAGTTGACTACTTTGGTCATTCGTAGTAAAATATGTGGGAGTGGTCTTTCTGCTG

TCAAACAAGTAAAGATTAAAGCAGATATGTTTTCAAGATGGGATGAGCCTCCTATGATTC

TATTCGACTGGCTGCTAAACCTTTCCAATGTAAAATCATTGATGGTCTCTTCAACTACTC

TTCGGATTCTCTCATTAGTTCCTGATTTACTAGAGGTTAAGCTGCCTTCTTTGTGTAACT

TGAAGTCTATGGAAATAAAACTGGAAACCTGTAGTAATCAAATGGCATTACCCAACCTGG

TGAAAGAAACCATGTTAAAGAAAGCTGCTGCCAAGTCACGTAAAGAAGCTGCCAAGTTAA

GAAAGGCTTTTAAAGCACGTTTGGAACCACCTCCCATACCTGATGGAATAGTTGACTTCT

TGCGACAAAACTCGCCATCCGCCGAAGTTAACATCACAACAAATTACCCGGGTTGTTTTA

ATCTTAAGCAGGTTGAAGAATCTATAAAGGGTGCAAAGGTTATCAACTATCATTCGCAAT

TCGCCGTGCCTGCCTCCTCTGCTGCACCTGTTCATGCTGCTGAGTCTGCTTCTGCCACCG

TGCCTGCCTCGACATCACCTCCCAATCTTCATCTATGTTGCGCTGAAAAGGAACCTGTTT

GGCCTCGTTTAAAGAATATTTATAAGAGGATGGGACAGTATTAA

>MS.gene40141.t1

ATGCATCGTTCCATTACGCGAGTCGTAGGCAGTTTTCCAATCAACCTCCCTCCCGTGCAG

ACCGATTTTGACTCCAATTTTCAAGTAATCAAACCTCAATCGCCAGGTACTTACGGACTT

ACTCGAATAATGTCGAATTCAGTGGATGAAGTAATGATTCCACCCGATAAGAGAGTAAGG

CGCGGTAATGAAGAGAATCAAGACAGGCTGAGTGACTTACCTGATTGTGTTATCCTTCAC

ATTTTGTCGTTTTTGAAATCAAAGTTTGTTGTGCAAACTTCCATTTTGTCTACAAGATGG

AAACATCTTTGGAAACGCATTCCTACTCTTATATTGCATTCCTCAGACTTTTCCACTAAG

AAGCGTTTTTCCGTATTTGTGTCTCAGATTTTGGCTAATCGTGATAGATCGACTGCACTG

CACTCTCTCGATCTTGTGCGCCATGGCAAGATTGAGACTTACCTCCTCAAAATGATTTTA

AAATATATTTGTTCCCATAACACCCACATCCAACAATTAGGAATCCATGTCACTGCTGAC

AGTTGTCCCATTCTGAGCTGCGTTTCTAAATGTCATGCTCTTACATCTCTTAAGCTTATA

GTTAACCATAGAGGTAATCAAACAGAAACATTATTTCCAAAATCTTTGAATTTGCCTTTA

TTGACCAACTTAGATCTAACACATTTGGCCTTTTGCGGTGGTGAAAACGGTTGTGCTGAG

CCCTTTTCCGCCTTTCCCAAGTTGAATAGTTTGGTTATTTCTGGTTGTAAGGTAAAAGAT

GCTCAAATTCTTAGCATATCAAGTGAGACACTTGTCAATTTTGCGATGCATTATTGTTCT

TCAAGAATTGCCAAAATCGAGTTATCTGCTCCTAGTCTTTGTACCTTTACTTTTTCTGGT

ATGCTTGTTCAGAAAATATGTGGGAGCAGTCTTTTTTCTGTTAAAAAAGTATATATCAAT

CCATTTGTTTCTTTGGCTCCGGAGGATTATGCTTTGGCTATGGAGGATTATTCTTTGGTT

CTATTAAGCTGGCTGCAAGACCTTGCCAATGTAGAATCATTGAGAGTCACCTCAGCTACT

CTTCAGATTCTCTCCTTAGTTCCTGATTTATCGGAGGTTAAGTTCACTTCTTTGTGTAAC

TTGAAGTCATTGGAAGTAGAACTGGTACCAATTTATTATCAATTATTTATTGATTTGATG

GAAGAGACCATGTTAAACAAGGCAGCTACCAAGTCACCTGAAGAAGCTGCTAAGTTAAAC

AAGGCATTTAATGCTGGTTTGCAACCATCTCCCATACCTGATGGAACAGTTGACTTCCTG

CGACAAAACTCGCCTTCGACGACAGTTAACATCAACACAACTTACATGAGAAATTTTAAT

ATTAAGCAGGTTGAAGAATCCATAAAGGGAGCGAAGATGACCAGCTACCATTCACAATTT

TCTGCGTCTGCCCCTTCCTCTGTCGCACCTGCTTCTGCTGAGTCTGCTCCATCTCCTGCC

ACCGCGCCTTCTTCTTCCGCACCTCCCAATCTTCATCCTTGTTTCTCTGAAAAGGTTGAA

GAATCCGTAAAGGGCGTGAAGAACACCAGCTACCATTCGCAATTTGCCACACCTGCCACC

TCCTCTTCCGCACCTGTTTCTACTGCTCCTGCTTCTGCTGCACCTCTCAATTTTCATCTC

TGTTGCGCTGTAAAGGTTGAAGAATCCATACAGGGTGTGAAGAACACCAGCTATCGTTCG

CAATTTGACGCGCCTGCCTCCTCTTCTGCTGCACCTCTTAATCTTCATCTTTGTTGCACT

GAAAAGATGACAACTAATGTTGACAACAGTGATTCAACACTTTGGCCTGAGTTGGTAACT

AAAGCTTTCATCGAAATTATGGTTGATGAAGTTACAAAAGGAAATATGCAAAATGGTGTG

TTTCATACTGGAACCTGGACCTCAATGACTGCTAAGTTGAATTCCACAACTAATTGCTCC

TATAATAAGGAACAACTAAAGGCTAAAATGCATAGGCTAAGAGCCATGTTTTACGAGTTC

TATTCACTTTTGCAAAACACAGAATTTGGGTGGAATGCAGAAACCAACATTGTTACTGCA

AGTGAAGAGGTCTGCCAAAATTATCTTAAGACACATGATAAAGCATCTCAGTTTCAAAAT

AAAGGGTGCGACCATTATAAGTTGTTGGAAATCATATTCAACAAAAATAATGAAACTGAA

GGACTTCATCATTCATCTACCCAAGACCAACAAAATACCGATAAAGAAAATGAGCTCGAT

AATCAATACTTTAACACTGGGAGTGCAAATAATGTATGTGTTGATGATGATAGTTCAAAC

AATGATATTCAAGAAGTGGAGCGCATCACATGCAATGAGAAGCAAATGATTGAAGTAAAA

GATCATATATTCAGGAAAGAGTCTACAACACGTCAGATAGGAGAAGCACTTGTAGAACGG

GGTAAGATTGTGGAGGCTAGTTCTTCACATGTAACAAAAGATTGCTCTCTTACTAAGTGT

GTGGTTGCTCTTGAAGAGATTGAAGACATTTCAGATGACATCTATGGGAAAGCTTTGGAA

AAATTTAAGGATCCTGATTGGAGGGAAATGTTTATGGCCATGTCTAAAGATAGGAAACGT

GGATGGCTATATAGACTTTAA

>MS.gene40142.t1

ATGTCAAATTCAGTGTCGGAAGATGATTTAACGATTCCGCCGAAACCGCGGAAAACAAAG

AAATTAAAGCAAGAAAATGAAGAAGATAATCTGAGTGATTTACCAGATTGTGTTATTCTT

CATATATTATCGTTTTTGAACGCGAAAGAAGCGGTTAGAACTTGCATTTTATCGAATAGA

TGGAAGAATGTTTGGAAATGTGTTCCTACTCTTGTATTGCATTCATCTGATTTTTCAACT

TTCAAGAATTTTACTAAATTTGTTTCTAAGATTTTGTCGCATCGCGATGGAACAATTGCG

CTGCAAGAGCTTGATTTTGAACGTGTAGGTAGTATTGAGCCTCACCTTCTTAAGAGAATT

GTGAATTATGCTTTTTCTCATAATGTGCAGAGATTGGGAATTTCTGTGAAAGGGGATATT

TGTCATATTCTGCCTTGTGTTTCTTCGTGTCGGACTTTAACGTCTCTTAAGCTTTCTGTT

TCACCTAAAGGGAGGCATAATTATGGGAGAACGTTGTTTCCGAAATCTTTGGATTTGCCG

GCGTTAACCAATTTGCATCTAGGGAATTTCGTCTTTTGTGCTAATGATAAAGGCCGAACT

GAGCCCTTTTCGGGTTTTAACAAGTTGAATAGTTTGGTCATTGACAATTGTACGGTGAAG

GATGCGCAGATTCTTTGCATATTAAGTGAGACACTTGTCAATTTGACTATGCGTAATCAT

TCTTCCGATGTTTACAAAATCGAACTATCCGCCCCAAGTCTTAGCACATTTGCGTTTACC

GGAACCCCTTATCAGAAACTTTGTGGGAGCAATCTTTCCTCGGTTAAACAAGTGAATATT

GATGCAGAAATGTTGTCTAACTACACTGAGCCTCCTTTGGTTTTGCTTAGCTGGCTGTTA

GAGCTTGCTAATATCAAATCATTGACGGTCTCTGCGAGTACTCTTCAGGTTCTTTCTCTA

ATTCCTGATTTGTTGAAGGATAAGCTCACTTCTTTGTGTAACTTGAAGTCACTTAAAGTA

CAACTAAAACCACTTTCATACGGATTATCCATGACCCTGAGAACGGCCAAGTTACAGAAA

GAAGTTAAAGCAGGGCCGGAACCATCTTCACCCATACCTGATGGAATAGTGGACTTTTTG

ATTCAAAACTCACCGTCGGCAGAAGTTGACATCGTAGATTGCTCAAGGTTCGGCGGCTCC

TTTGACCATCTGCCTCCATTTCCCGTGTCTTCCATTTTTCCTCAATTCCTTCAGCCTTCT

TCCTGTGAGTCTGTTGTGGATGACCTGCGCCAGCGGATACAACAGTTAGAGCAAGCAGTA

TTGCAAGTGCAGCAGTATATTCAACTAGCCCATGAGGAGATATCTCGGGAGAAGGAGGAA

ATGTCTGCCATCCAAGGGCATATGTCGATGTTGTCAAAGACCCTTGCTGCCCTAAAGCGG

CAGATGCAGACTATGGGGCTGTGGCAATGA

>MS.gene40140.t1

ATGATTCTATTCAGCTGGCTGCAAGCCCTTGCTAGTGTAAAATCATTGAAAGTCTCTTCA

ATTACTCTTCAGATTCTCTCCCTAGTTCCTGATTTGTTGGAGGTTGAGCCCCCTTCTTTC

TGTAACTTGAAGTCTATGAAAATAGAACTCAAACCACTTAGCTTAGATTTATCTTGTATA

CTAAAAGATGCCATGTTAAAGAAAGCCGATGCAAAGCACCCGATGAAGCGGGCAAGTTAA

>MS.gene40143.t1

ATGGCTTCTCGGAATCAGAACCGGCCTCCTCAAAACCACTCCAATGTTGGATCTGAAGAG

ATGGAGAGACAAAGAGTGTCTGGTAGAGCTAAAGCTAGAGCGCCGTTAACTGCAATGGTG

AACAACGCTACTGCAAGTAATGTAGAGGGAATCGATTCAACCGCAATTGAGTTCACAAAA

GATGATATAGAGGCATTGCTTAATGAGAAGATGAAAAAGGGAACTCCTTTTGATAATAAG

AAAAAAATGGAGCAGATGGTGGATCTTATTAAGCGGCTTAAGCTTTGTGTTCAATGGTGC

GCCAACAAGATTGAAACGCTTCATTCTGAAGTTGAGTCTGCTGTTAAGAAGTGTTCTGAT

ACTGAGATTGAGATGAAAAATAAGACCGAGGAGCTTCAATCAGATCTCAATTCAGCTAAC

GAGGCTTATAGAAGACTTGAAACAGAGAAATCAATCATTGCTGAGAGCCTTAATGATACA

AGAGATCTTAATAAGGAATTGCAGGATCGATTGAAATCTCTTAAAGATTCACAGAGTGAA

GCTATAAAGCATAAAGAAATGTTCCGAGAAGAACTAAAAAAAATTACAGATGATCGTGAT

CGCCTACAAGGAAAACTAAATGATGTCATGTTAGAACGGGAAAAATACATAAAATTTAAA

TATGAAACATACTCAAAATTGGATAAATTGGCAAGTAAAACAGAGGCACTTGAGGAGACT

TGTTCCTCTCAAAAGGAGAAAATAAATATACTGGAGCAAAAGTTACATGCTGAAAGGGAG

AAGTTAGAGATGGCTGATTTATCTGCTATGGAAACAAGAGCAACGTTTGAAGAGCAGAAA

AGATTCATTCGTAAACTACAAGATCAATTGGCAGATAAAGAATTTCAAGTAGTTGAAGGA

GAAAAGCTAAGGAAAAAACTGCACAACACTGTCTTGGAGCTGAAAGGAAATATTCGTGTG

TTTTGTCGTGTACGACCTTTACTACGAGACCGTTCAGAAACGAAGACGGTTGTTTATTAC

CCTACGTCAATAGAAATGCTTGGCCGGGGCATTGAGTTGGAACAAAATGCAGGACAGAAA

CAGCTTTTTACATTTGACAAGGTGTTTAATCATGACGCCTCTCAGCATGACGTTTTCACC

GAAATATCACAACTGGTGCAGAGTGCCCTTGATGGCTACAAGGTATGCATTTTTGCTTAT

GGACAAACAGGTTCAGGAAAAACCCATACAATGATGGGTAGGCCTGACTCTCCAGATCTC

AAAGGGATGATACCACTTTCTTTAGAACAGATATTCCAGACCAGTCAGTCTCTTAAAGAC

CAGGGCTGGAATTACAAAATGCAGGCATCAGTATTGGAAATATATAATGAGACCATCAAA

GATTTGTTATTGTCAAATCGGTCAAGTGGGATTGACCAAACACGAGCAGAAAATAGTGTT

CATGGAAAACAGTACACTATTAGACATGACAAAAATGGAAACACACATGTTTTGGACCTC

ACCATTGTCGATGTTTGTAGTGCCGATGAGATTTCCTCCCTCCTCCAAAAGGCTGCCCAA

ATCAGGTCAATGGGAAGAACACAAATGAATGAACAATCATCAAGAAGCCACTTTGTCGTT

ACTTTGCGCATATGTGGTATAAACGAGAACACTGAACAACAAGTACAAGGTGTCTTAAAC

CTGATTGATTTGGCTGGAAGTGAAAGACTTTCAAGGAGTGGGGCCACTGGGGAACGTTTG

AAGGAAACTCAGGCTATCAACCAAAGTCTCTCCTGTTTGAGCAATGTCATATTTGCTTTG

GCAAAGAAAGAAGAGCATGTTCCTTTCAGGGATTCAAAACTTACACACCTACTTCAGCCA

TGTCTTGAAGGGGATTCCAAAACTTTGATGTTTGTCAACATCTCACCTGATCAATCTTCA

ATTGACGAGTCACTTTGCTCTCTTCGATTTGCGTCTAGAGTCAATGCTGTGAAATTGGGG

ATTCACAATGTCAGACCTAGACATCCACTCAATGTTTAG

>MS.gene40144.t1

ATGTCAGCATACCGATGGAAAAGCTTCGAAGAGAACGAAGACCGCCCCTCCAAGCCTCGC

CGCTACGGCGTCACCGAAATGCGAAGCCCTCATTACACCCTCTTCAACCACAATGTTCTT

CAGGATATTTTTGAATCTATGGGAGATTATGTTGATGGATTGAAATTTTCTGGAGGTTCT

GATAGTTTGATGCCAAAAGCTTTTATCAAACAAGTTATTGATACTGCTCATCACCATGAT

GTTTATGTTAGCACTGGTGATTGGGCTGAACATATGATTCACAAAGGTCCTTCAGGATTC

AAAGACTATGTGGAGGAATGTAAGCAGTTGGGGTTTGACACAATTGAGCTGAATGTGGGT

TCCCTTGGAGTTCCTGAAGAAACCCTTTTGAGATTCGTCCGCTTGGTTAAAACTGGTGGT

ATGAAAGCTAAGCCTCATTTTGAAGTTAAGTTCAATAAGTCTGAACTTCCCAGAGGTGGT

GATAGGGCTTATGGGGCTTATATTCCTCCAGCACCTAGATCATATGAATTAGTAGAAGAT

GTGGATCTCTTGATTAGGAGAGCTGAGAGGTGTTTAGAAGCGGGTGCAGACATGATCATG

ATTGATGCTGATGATGTCTCCAAGCATGCTGACAATATGCGCGCAGATGTTATTGCGAAG

ATCATAGGACGCCTTGGTATTGAGAAGACTATGTTTGAAGCATCAAATCAGAGTGCATCC

GAATGGTTTATTAAACAATATGGTCCAAATGTGAATCTTTTCATTGATCATTCCAACTTG

GTAAATGTGGAGTGCATCCGGGGACGCAACTTAG

>MS.gene40138.t1

ATGATTCTTCGAAGTATGAAGAGAAGAAGGATCTATGAGGTTAAAAATGATAAAAAGGCT

GAAAATGGAGAAATTGAAGACAGGCTAAGTAGTTTACCTGATGGCGTTCTCCTTCACATT

TTGTCATTTTTGGACGCCACATATGCAGTTCAAACTTGTGTTTTGTCCACAAGGTGGAAG

TATCTTTGGAAACTTATTCCAACTCTTAATTTGCATTCTGAAAGATTTTATACTGTTAAG

CAATTTTCTAAATTTGCGTCTAAGATTTTGACTCTTCGTCTTCGTAAAAAGTCAGCTGCT

TTGGGTGCCCTTGATCTTGACTGTAGAGGTAATATTGAGCCTCAAATCCTTAAAAAGATT

TTGAATTATGTTTCCCCACATAATACCCACCTCCAGAAGTTAGAAATCTATGGCTATGGG

GATTCTAGTCTCATTATGAGATGCGTTTCTTCATGCCAGGCTCTTACATCTCTTAAACTT

GGTCCTATTAATTTTTCTAGAACAATCTTTCCAAAATCTTTGAATTTGCCAGCATTGACC

AGCTTAAATTTAAACACATTTGTCTTTTGTGGTGGTGATGACAATTGTGTTGAACCCTTT

TCCGCCTTTACCAAGTTGAATAGTTTGGTCATTGATAGATGTGCGATTAGGGATGCACAA

ACCCTCAGGATATCAAGCGAGACACTAGTAAATTTAGCTATGCATGAAAGTTCATATGAA

TTTGACAAAATTCAGTTATTTACTCCGAGTCTTTGTACCTTTACTTATACCGGTCATCCT

GATCAGAGAATATGTGGGAGTGGTCTTTCTTCTGTTAAACAAGTAAATATTGCTGCAGAA

ATATATTTTTGTTGGGAAAAGCCTGCTAGGCTTCTACTTAGTTGGCTGCAAGATCTTGCC

AATGTAAAATCATTGACAGTTTCTTCAACTACTCTTCAGATTCTCTCCTTAATTCCTGAT

TTATTAGAGGTTAAGCTCCCTTCTTTGTGCAACTTAAAGTCTATAGAAATAAAATTGGAA

CCGCTTGCAGACTATACAACTTTATTTGATGAATTGCAACGTGTCATGTTAAAGAAAATT

GCTGCTAAGTCAATGAAACAGCGTATAGAACGTTTGGAACCACCTCCCATACCAGATGGA

ATAGTTGCATTCTTGCTTCAAAACTCACCGTCCGCAAAAGTTGACATCACAACAGAGTAC

CCAAATGCTTTTAAGATTAAGCAGGATGATAGATCATCAAATGAAGATAAGGTGGAGAAT

TGCCAACCTAACGTAGCAACTGCTTGA

>MS.gene40139.t1

ATGAAGAAAGCAAAGCACTGTGAAAACGAGAATGAAGATAGATTGAGTGATTTACCCGAT

TGTGTTCTCCTTCACATTCTGTCATTTTTTAATACTAAACATGCCGTTCAAACTTGCATT

TTGTCCACAAGATGGAAGTATCTTTGGAAACGTATTCCCACCCTTACATTGCATTCTTCG

GGATTTTCCACTATGAAGAGTTTTTCCACATTTGTGTCTAAGATTTTGACTCTTCGTGAT

AGCTCAACTTCTCTGCATGCTCTCGATCTTGACTGTAATGGATGTAATGGTAATATCGTC

CCGTCTCAACTCCTCAAAATGATTTTGAAGTATGTTTCTTCTCATAATTCCCACCTCCAA

GAATTAGGAATCTCTGTTAGGGGTGATAGTACTCTCATTCTCCAATGTGTTTCTTCATGT

CAGGCTCTTACATCTCTTAAGCTTTCGATTTACCCTAAAGGTCGTTCTTGTTTTGAGAAA

ACATTATTTCCGAAATCTCTGAAATTGCCAGCATTAACTAGCTTGAGTTTAACAAATTTT

GCCTTTTGCGGTGATGAAAGCGGTCGTGTTGAGCTCTTTTTGGCCTTCAAAATGTTAAAT

AGTTTGGTCATTCGTAATTGCGAGGTAAGGGATGCACATTTCTTTGTCATATCAAGTGAA

ACCCTTGTTGATTTATATATCTATAATAGATCATGTAAAATAGCTAATGGTGCCACAATT

GAGTTATCTGCACCAAGTCTTTGTACCTTTAATTATATCGGTGATACTGCTCAGAGAATA

TGTGGGAGAGGATTCCCTTCTATTGCTATAGGTACTCTTCGGATTTTCTCATCATTTCCT

CATTTATTAAAGGATAAGTGTCCTTCTTTGTGTAACTTGAAGTCATTGGAAGTAAATCCA

GGTTACCAAAAGAAGTGGGTTTAA

>MS.gene40149.t1

CAGAACTCTTTTAATTTTGGCCATAGGAATATGCATAGGCTTTGTGATTGGAGCTGGTTT

GGTATTTTCAATTTTAGTCTTCTGCAGGCCTGGAAGGAAGCGTGTAGAGGTGGAGAAGAG

CGGTCCTCTGAGAACCGAGGCCATTAATGTTCATGGCAAAGGGGCTGATTCCAGTGTAAC

ATCATTATCAGACTCCAACGCGACTTTTGAGTCACCAAGAACATCGGAATGGAGTAACAC

GCCTTTCTGGTTAGAAGGACTTAGGAGGAAGAATGCTGTCTCAGTATGTGGAATTCCCAA

ATATTCTTACAAAGATATAGAAAAGGCTACATCTAATTTCACAACGATCATAGGCCATGG

AGCATTTGGTCCTGTTTACAAAGCAATCATGCCTACAGGCGAGGCAGTTGCGGTTAAAGT

TCTCGGTGCTAATTCAAGACAAGGAGAACAAGAATTTTTAACTGAGGTCCTATTACTTGG

AAGATTACATCATAAGAATCTTGTGGGTTTGGTGGGATATGCTGCAGAAAGAGGAAAGCA

CATGCTTCTTTACATTTACATGAGCAACGGCAGTCTTGCTTCTCATTTGTATGGCGAAAA

TCATGAGCCATTGAGCTGGAATTTGAGACTTAGCATAGCACTAGATGTTGCAAGGGTGAT

GGAATATCTACATTATTCGATCGATCCTCCTATCGTGCACCGTGACATCAAGTCTTCAAA

CATTCTATTAGACAAGTTTATGAAAGCCAAGGTCACCGACTTTGGGCTTTCCAGACCAGA

AATGATTAAACCTCGTCTGTCAAATGTCAGAGGAACTTTTGGATATCTTGATCCCGAGTA

TATCTCTACAAGAACCTTCACTAAGAAAAGTGATGTTTATAGTTTCGGTGTGCTACTGTT

TGAGCTTATTACTGGCAGGAATCCACAGCAGGGTCTCATGGAATATGTAAAACTGGCTGC

CATGGAAAGTGAGGACAAGATTGCATGGGAAGAAATTGTGGATCCGCAACTGAATGGAAA

CTATGATGTACATAAGCTTAATGACATGGCTTCACTTGCACTCAAATGTGTCAATGGTGT

CTCCAAAATTCGACCTTCAATGAGGAACATTGTTCAAGCATTATCTCAACTTTATAAGAA

GCCTAAGAGAAATTCTAGCCGAGTAGCTCCTCCTGCGTTAAATGAAGTGTCTGTTGAAGT

GATGCAGCCAGAAACTCGTGACTTTTCTATTGAGTGTTCAGAGGAGCTGCGCAGATTGCA

CAGCCGATAA

>MS.gene40148.t1

ATGTCGTCAGAGAAGGAAGATTCAAGCTCCACAATGAAACGCAAATTATCGTGCTTCGAA

CACTTTGATGCACTCTGGTTCTGCTACTCCCCAGTTTATCAAATGCGGCAGTATTACAGG

CTTGGTTCTCTTGATAACTGTCGCGGTAAATGGAAAGCATGGACTGACTGCTTAATGCTC

AAGACCAAACCCAAATCTCAAGTCGAGATGAAGTCCCAACATCAGCTGGCAGAATCACTC

AGAGTGACAGAGGCAAAGAGCCTCTTCCTTCCAGCTAATAGTGCAAACAAAACATCTCAC

CTCATAGCACTGCAAAGTTTGGGAGAACTGAATCTATTTAAAGCAGAATTAACAGTTGAA

GAAGATTTTGATGCTCCTATAGCAGGATGTGAACTTGTCTTCCAACTTGCTACACCTGTG

AACTTTGCCTCTCAAGATCCCGAGAATGACATGATAAAACCAGCAATCAAAGGTGTATTG

AATGTCTTGAAAGCATGTGCAAGAGCAAAAGAAGTCAGAAGAGTTATCTTAACATCGTCA

GCAGCTGCTGTGACTATAAACGAACTCGAAGGGACTGGTCATATTATGGATGAAACCAAC

TGGTCTGATGTTGAGTTTTTGAACACTGCAAAGCCACCCACTTGGGGTTATCCTGTCTCA

AAAGTGCTAGCTGAAAAGGCTGCATGGAAATTTGCCGAAGAAAATAACATTGATCTAATC

ACTGTGATACCTACTCTAACAATTGGTCCTTCTCTAACACAAGATATCCCATCTAGTGTT

TCCATGGGAATGTCACTTCTAACAGGCAATGATTTCCTCATAAATGCTTTGAAAGGAATG

CAGATCCTGTCAGGTTCGATATCCATCACTCATGTCGAGGATATTTGCCGAGCTCATATA

TTTGTAGCAGAAAAAGAATCAGCTTCTGGTCGATATATTTGTTGTGCTCACAATACTAGT

GTTCCTGAGCTTGCTAAGTTTCTCAGCAAACGATACTCTCAGTATAAAGTTCCAACTGAA

TTTGATGATTGCTCCAGCAAGGCAAAGTTGATAATCTCTTCTGAAAAGCTTATCAAAGAA

GGGTTCAGTTTCAAGCATGGTATTGCTGAAACTTTTGACCAAACTCTGGAGTATTTGAAG

ACTCAGGGGGCCTTGAAGAAGTAG

>MS.gene40146.t1

ATGTCAGCATACCGATGGAAAAGCTTCGAAGAGAACGAAGACCGCCCCTCCAAGCCTCGC

CGCTACGGCGTCACCGAAATGCGAAGCCCTCATTACACCCTCTTCAACCACAATGTTCTT

CAGGATATTTTTGAATCTATGGGAGATTATGTTGATGGATTGAAATTTTCTGGAGGTTCT

GATAGTTTGATGCCAAAAGCTTTTATCAAACAAGTTATTGATACTGCTCATCACCATGAT

GTTTATGTTAGCACTGGTGATTGGGCTGAACATATGATTCACAAAGGTCCTTCAGGATTC

AAAGACTATGTGGAGGAATGTAAGCAGTTGGGGTTTGACACAATTGAGCTGAATGTGGGT

TCCCTTGGAGTTCCTGAAGAAACCCTTTTGAGATTCGTCCGCTTGGTTAAAACTGGTGGT

ATGAAAGCTAAGCCTCATTTTGAAGTTAAGTTCAATAAGTCTGAACTTCCCAGAGGTGGT

GATAGGGCTTATGGGGCTTATATTCCTCCAGCACCTAGATCATATGAATTAGTAGAAGAT

GTGGATCTCTTGATTAGGAGAGCTGAGAGGTGTTTAGAAGCGGGTGCAGACATGATCATG

ATTGATGCTGATGATGTCTCCAAGCATGCTGACAATATGCGCGCAGATGTTATTGCGAAG

ATCATAGGACGCCTTGGTATTGAGAAGACTATGTTTGAAGCATCAAATCAGAGTGCATCC

GAATGGTTTATTAAACAATATGGTCCAAATGTGAATCTTTTCATTGATCATTCCAACTTG

GTAAATGTGGAGTGCATCCGGGGACGCAACTTAGGTAAAAATCATGCTTCTGTTCTTGGC

TCTTCGTATTTCCTGTTCTGA

>MS.gene40147.t1

ATGATGGCACGTATAGCGCTAATGCAGGATACAAATGGCTATTTCGTCAAACAGATAATA

TTATGCCGCAAGCAGCTGCATCTTGGAGATGGATTTGGTGTATTGCAGCCCCAGAGAAGG

ACACTTCAGGGTGGTTGCGGCGAGGAGCAGAATGTGATAGATCAGCTGAGGCACAACCAT

GGGTTTCTTTGCATCCCAATGGCTGGTTTGGCAACAGTGTTAAATGTTGATGGAAGCAGC

TTGGGTAATCCAAGATCATCTGGTTTTGGAGGTGTTTTACGGCATTCCGACGGTTCTTGG

TTGTATGGGTTTGCAGGGCATGTTGGCATCTCTTCCATTCTTCATGCGGAACTGCTAGCC

TTATATTATGGATTGCGCCCAGCTTGGGAGAAAGACTACGGGCACATCATTTGCTATTCT

GATTCTAATCTTGCTATTCAGTTAGTAACTACAGGGGAGTTGTTGGCTCGTGCTTGGACT

GTCCAGGTGTGTCACACTTGGAGGGAAGCAAATGTTGTGGCAGACTTTCTAGCAAAGATG

GGGGCAAGGAGTAATGTGGATTGGCATGATTTCTTTTCCCCTCCTGACGGATTGCAGCCC

CTATTGCAGGTTTTTTTGAACAAGAGCCCCTATTGCAGGTTGATACTTCCAGAGTGCTAT

ATGCACGTCGTTAGATCCTATCTGGATCTTTTTGTTTTGTCTTAG

>MS.gene40459.t1

TGATATGGGTGGAAATTATGCTGACATCAATTGGGAAGGACTTAGTTTTAATCTGACTCC

AACAGATTACATGCATGTCATGAAATGCACAAAAGGAGAAAAGTTTTCACAAGGATCCCT

CATTCGCTATGGAAACATTGAGATAAGCCCGGCTGCTGGTATCATAAACTATGGACAGGG

AATCTTTGAGGGACTAAAAGCATATAGAACCGAAGATGGGCGAATCCTTCTTTTCCGACC

AGAGGAGAATGCTCTACGCATGAAGATGGGGGCTGATAGGTTGTGTATGCCATCACCATC

AGTTGAGCAGTTTGTTGATGCTGTTAAGCAAACAGTTCTTGCCAATAAACGTTGGGTACC

TCCTCCAGGGAAAGGGACGCTTTATCTTAGGCCTTTGCTGATGGGAACAGGAGCTGCATT

AGGCCCGGCTCCATCACCTGAGTACACATTTCTCATTTATTGCTCCCCTGTTGGAAAGTA

CCACGAGGGCGGAAGACTAAACTTAAAAGTGGAGGATAAATTTCATCGATCAATAGCTGG

CAGTGGTGGAACAGGAGGAATCAAGAGTGTTACTAATTATGCCCCGATATATACTGCAGT

AACTGAAGCAAAAGCCAATGGATTTTCTGATGTCTTGTTCTTGGATTCAGCAACTGGAAA

AAATATTGAGGAGGCTACTGCGTGCAATATATTTGTTGTGAAGGAAAATGATATTTTCAC

TCCGGCAATAGATGGATCTATTCTGCCTGGGGTCACACGAAAATCCATCATAGACATCGC

CATTGATTTGGGTTATAAGGTCATAGAACGTTCCATATCAGTGGAGGAAATTATGAGTGC

TGATGAAGTGTTCTGCACAGGAACTGCAGTGGTTGTTACCTCTGTTGCATCTGTAACATA

TAAGGAAACAAG

>MS.gene40515.t1

ATGAAGAAAGCAAAGCACTGTGAAAACGAGAATGAAGATAGATTGAGTGATTTACCCGAT

TGTGTTCTCCTTCACATTCTGTCATTTTTTAATACTAAACATGCCGTTCAAACTTGCATT

TTGTCCACAAGATGGAAGTATCTTTGGAAACGTATTCCCACCCTTACATTGCATTCTTCG

GGATTTTCCACTATGAAGAGTTTTTCCACATTTGTGTCTAAGATTTTGACTCTTCGTGAT

AGCTCAACTTCTCTGCATGCTCTCGATCTTGACTGTAATGGATGTAATGGTAATATCGTC

CCGTCTCAACTCCTCAAAATGATTTTGAAGTATGTTTCTTCTCATAATTCCCACCTCCAA

GAATTAGGAATCTCTGTTAGGGGTGATAGTACTCTCATTCTCCAATGTGTTTCTTCATGT

CAGGCTCTTACATCTCTTAAGCTTTCGATTTACCCTAAAGGTCGTTCTTGTTTTGAGAAA

ACATTATTTCCGAAATCTCTGAAATTGCCAGCATTAACTAGCTTGAGTTTAACAAATTTT

GCCTTTTGCGGTGATGAAAGCGGTCGTGTTGAGCTCTTTTTGGCCTTCAAAATGTTAAAT

AGTTTGGTCATTCGTAATTGCGAGGTAAGGGATGCACATTTCTTTGTCATATCAAGTGAA

ACCCTTGTTGATTTATATATCTATAATAGATCATGTAAAATAGCTAATGGTGCCACAATT

GAGTTATCTGCACCAAGTCTTTGTACCTTTAATTATATCGGTGATACTGCTCAGAGAATA

TGTGGGAGAGGATTCCCTTCTATTGCTATAGGTACTCTTCGGATTTTCTCATCATTTCCT

CATTTATTAAAGGATAAGTGTCCTTCTTTGTGTAACTTGAAGTCATTGGAAGTAAATCCA

GGTTACCAAAAGAAGTGGGTTTAA

>MS.gene40516.t1

ATGATTCTTCGAAGTATGAAGAGAAGAAGGATCTATGAGGTTAAAAATGATAAAAAGGCT

GAAAATGGAGAAATTGAAGACAGGCTAAGTAGTTTACCTGATGGCGTTCTCCTTCACATT

TTGTCATTTTTGGACGCCACATATGCAGTTCAAACTTGTGTTTTGTCCACAAGGTGGAAG

TATCTTTGGAAACTTATTCCAACTCTTAATTTGCATTCTGAAAGATTTTATACTGTTAAG

CAATTTTCTAAATTTGCGTCTAAGATTTTGACTCTTCGTCTTCGTAAAAAGTCAGCTGCT

TTGGGTGCCCTTGATCTTGACTGTAGAGGTAATATTGAGCCTCAAATCCTTAAAAAGATT

TTGAATTATGTTTCCCCACATAATACCCACCTCCAGAAGTTAGAAATCTATGGCTATGGG

GATTCTAGTCTCATTATGAGATGCGTTTCTTCATGCCAGGCTCTTACATCTCTTAAACTT

GGTCCTATTAATTTTTCTAGAACAATCTTTCCAAAATCTTTGAATTTGCCAGCATTGACC

AGCTTAAATTTAAACACATTTGTCTTTTGTGGTGGTGATGACAATTGTGTTGAACCCTTT

TCCGCCTTTACCAAGTTGAATAGTTTGGTCATTGATAGATGTGCGATTAGGGATGCACAA

ACCCTCAGGATATCAAGCGAGACACTAGTAAATTTAGCTATGCATGAAAGTTCATATGAA

TTTGACAAAATTCAGTTATTTACTCCGAGTCTTTGTACCTTTACTTATACCGGTCATCCT

GATCAGAGAATATGTGGGAGTGGTCTTTCTTCTGTTAAACAAGTAAATATTGCTGCAGAA

ATATATTTTTGTTGGGAAAAGCCTGCTAGGCTTCTACTTAGTTGGCTGCAAGATCTTGCC

AATGTAAAATCATTGACAGTTTCTTCAACTACTCTTCAGATTCTCTCCTTAATTCCTGAT

TTATTAGAGGTTAAGCTCCCTTCTTTGTGCAACTTAAAGTCTATAGAAATAAAATTGGAA

CCGCTTGCAGACTATACAACTTTATTTGATGAATTGCAACGTGTCATGTTAAAGAAAATT

GCTGCTAAGTCAATGAAACAGCGTATAGAACGTTTGGAACCACCTCCCATACCAGATGGA

ATAGTTGCATTCTTGCTTCAAAACTCACCGTCCGCAAAAGTTGACATCACAACAGAGTAC

CCAAATGCTTTTAAGATTAAGCAGGATGATAGATCATCAAATGAAGATAAGGTGGAGAAT

TGCCAACCTAACGTAGCAACTGCTTGA

>MS.gene42380.t1

ATGAGTTCTCTTCTTCAACCACAAGGTAGAGATTGCGAGCAAGACAATAGCTCTAAACTG

TTGCCAACAATTGTGAAATTTGAGTCCACATTTGAGGTGGAAGCCACCAAATCAGTAGAA

AATGATAAGGAGGAGGGTATTTTCTTGACATGGGGAGGACCTTTGGGTGACTGTTACAAA

TGGAAAAATGGAAGAAAACCAATACTTCAAGGTCTAAGAGGCTATGAAAACCAGGACAAC

TCTTACTTAGCCATAATAGGTCGTTCTGGTTGTGGCAAGTCCACTCTTCTTGATGCTTTA

GAAGGCTGA

>MS.gene42381.t1

ATGGTCAATAACTCTTCTCTGGAGATCAATGAAGAAAGGGAAGATTTGGATGACTCAAAG

AGTTGCAAAGGAACTAGCAACTCAGAACCATCTTTTGGTATCACTAAGGAGCAATATGAG

CAACTTGTCACTCTTCTTCAGTCTCATCAAGCTTCCACAAGCAAGGTGAATCATGTGACT

TCAGGAAGATTAGGCTCTAAAACAAAACAAACAGGGAATATCCTAATCAATGGCCGCAAA

CAAGCACTAGCTTATGGAATCGCTCACCTACAATTAATTAACAAGGATTTTGAACAGGAC

CCTGAGATAGGGATAACTGGAGGACTATGCACAAAAGAAGCTATCCAAATTCTTGTTAAA

TCATATGATTCATCTGAAGTTAGTCACCAAGTTCAAAAAGAACTTTATGAAATTAAAAAA

AAAAGAAGATCTTTCGTGAATATGCATCGTGAAGCAGGCTATTATCGGCTACGTTTGCTT

ATCTGTGGTGCATTGGCTTTAAGTCTTGAAACTATGTTCTTTGAGATTGGTTCAAGTGGT

GAATCAATTCAGGCCAGAGGTTCACTGCTTGTATTTCTTGTTACATTCTTGACATTCATA

ACTGTTTCTGGATTCCCTTCTTTTGTGGAGGATATGAGGGAGCATTGGTTTACTACCCTA

GTTGGACTTCACCAAGGACATGAACAATTTGTGTACTTTATATCTTTGCTTTTTATTTCA

GTCTTCTTAGTTGAAGGTCTCATGATGATTATTGCAAGTATGGTTCCAAATTTCCTAATG

GGAATAGTCTTTGGTTCTGGAATCTTGGGAGTAATGATGTATGCATACCAAGGATTGTTC

AAGACTGAGTTTCAAGGTCTAACATTCACTATTAGCAGTCAAGTGTGA

>MS.gene001495.t1

ATGGCAACTAATCAGATGCACGAATTGTTACATCCGTGGCTGGCACGCAAAGAAATCAAG

GAGATCTCTGTATTGTTGGGAAGATTGACTTCAACAATATGGTTAGAAGAACTCCGGGAC

ACAACCGACTGTTTTGCTATAGACAATGCTATTGGGGTGGGAAAGATGGGAATGATGTAC

CAGGGAAATCTACCAAATGGTCAGTTACTAGCCGTTAAGAGACTATCTAACTCTCGTCTT

TTTGAAAGGCAGTTCCTTTTAGAAGCAACGATTATGAGTAGGTACAGACACAAAAATATA

GTTCCCATGCTTGGATTCTGCATTGAAGGAAAGGAAAGGCTTTTGGCATATGAATACATG

TCAAATGGAAGGCTTTCCAAATGGTTGCACCCTTTGAAAAGTGAAGTCATCAGACTGAAA

TGGCCTGAGAGGGAAAACATTGCACTTGGAATAGCAAGAGGATTATCATGGCTCCATCAT

AGCTGTGATTTGAGCATAGTGCATTTTAATATATGTTCACAATGCATACTGCTAGATGAG

AATTTTGAACCCAAAATATCCAATTTCGGGCAAGCCAAATTTATGAATCCTAACAATGAG

GATGATCTTGGCAGAATGTTTAAAGTAAACGATGGGAAGAAGGATGTCCATGACTTTGGA

AGTGTGCTCTTTGAACTGATTACAGGGAAAACATATAACGAACTGACTCGTTCATCCACC

ACCACTAATCTCTATGATAACCCTTCAAATTTTTATAATGCCATCGATAAATCTCTGCCC

GGACAAGGATTTGAAAACGGAGTATGTGCTCTCCTAAAGGTTGCTTGTGAGTGTGTTAAA

CCTATAAATCAAAGACCAACAATGCTTGAAGTTTACAACAATTTGAGCAATATAAGTAAG

TCACAATATGTCTCTAGTGATGATTCTAATCCAACCGGTGGATCACAAATTGCTTCTGGT

ATTACTATAGACGAAATTACCGAGTTATAA

>MS.gene028559.t1

ATGGCTTTCCTAATTTTCTCATGGATAGTATTAGGAGTTGCATTCTCCATGCTGATCATA

GGCACATTAGCAAACTCAAAATCAAGAGAATCTTGTGGGATATCAAATCATCGGTTTTTA

TCCATTGGAGGGATTTTATGTTTCATTCATGGATTGTTTACTGTTGCTTATTATGTTTCA

GCAACGGCCACAAGAAGGGAAGAATACAAGTGA

>MS.gene028560.t1

ATGTCAGCATGCCGATGGAAAAGCTTCGAAGAGAACGAAGACCGCCCCTCCAAGCCTCGC

CGCTACGGCGTCACCGAAATGCGAAGCCCTCATTACACCCTCTTCAACCACAATGTTCTT

CAGGATATTTTTGAATCTATGGGAGATTATGTTGATGGATTGAAATTTTCTGGAGGTTCT

GATAGTTTGATGCCAAAATCTTTTATCAAACAAGTTATTGATACTGCTCATCACCATGAT

GTTTATGTTAGCACTGGTGATTGGGCTGAACATATGATTCACAAAGGTCCTTCAGGATTC

AAAGACTATGTGGAGGAATGTAAGCAGTTGGGGTTTGACACAATTGAGCTGAATGTGGGT

TCCCTTGGAGTTCCTGAAGAAACCCTTTTGAGATTCGTCCGCTTGGTTAAAACTGGTGGT

ATGAAAGCTAAGCCTCATTTTGAAGTTAAGTTCAATAAGTCTGAACTTCCCAGAGGTGGT

GATAGGGCTTATGGGGCTTATATTCCTCCAGCACCTAGATCATATGAATTAGTAGAAGAT

GTGGATCTCTTGATTAGGAGAGCTGAGAGGTGTTTAGAAGCGGGTGCAGACATGATCATG

ATTGATGCTGATGATGTCTCCAAGCATGCTGACAATATGCGCGCAGATGTTATTGCGAAG

ATCATAGGACGCCTTGGTATTGAGAAGACTATGTTTGAAGCATCAAATCAGAGTGCATCC

GAATGGTTTATTAAACAATATGGTCCAAATGTGAATCTTTTCATTGATCATTCCAACTTG

GTAAATGTGGAGTGCATCCGGGGACGCAACTTAG

>MS.gene028557.t1

ATGTCTCCTGGTGTCATTCCAACAACCTGTACTCTAGTGTTTGATGTGCAATCTTTCCTG

CGTGGCTCTCAGTCCTTTGGTTTCCTGGCAACATGCATCAAATCCTCTACTTCCTCTCAA

TCTGTCCGTGCACCTACCCCACAGCCTTCATCTACTACTTCCTTGAGTACCATTGATCCC

AACTACCCTGTTTATTGTCCTACTTTGTCTTCTCCTCCTCTTTCTACTCAGCCCATGCAA

GCACAGCCCAAGTTTGGCATTGTAAAGCCTCAACTTCACCCCACTTATTGCTCACTACAG

TTCGGCGGCTCCTTTGACCATCTGCCTCCATTTCCCGTGTCTTCCATTTTTCCTCAATTC

CTTCAGCCTTCTTCCTGTGAGTCTGTTGTGGATGACCTGCGCCAGCGGATACAACAGTTA

GAGCAAGCAGTATTGCAAGTGCAGCAGTATATTCAACTAGCCCATGAGGAGATATCTCGG

GAGAAGGAGGAAATGTCTGCCATCCAAGGGCATATGTCGATGTTGTCAAAGACCCTTGCT

GCCCTAAAGCGGCAGATGCAGACTATGGGGCTGTGGCAATGA

>MS.gene028558.t1

ATGGCTTCTCGGAATCAGAACCGGCCTCCTCAAAACCACTCCAATGTTGGATCTGAAGAG

ATGGAGAGACAAAGAGTGTCTGGTAGAGCTAAAGCTAGAGCGCCGTTAACTGCAATGGTG

AACAACGCTACTGCAAGTAATGTAGAGGGAATCGATTCAACCGCAATTGAGTTCACAAAA

GATGATATAGAGGCATTGCTTAATGAGAAGATGAAAAAGGGAACTCCTTTTGATAATAAG

AAAAAAATGGAGCAGATGGTGGATCTTATTAAGCGGCTTAAGCTTTGTGTTCAATGGTGC

GCCAACAAGATTGAAACGCTTCATTCTGAAGTTGAGTCTGCTGTTAAGAAGTGTTCTGAT

ACTGAGATTGAGATGAAAAATAAGACCGAGGAGCTTCAATCAGATCTCAATTCAGCTAAC

GAGGCTTATAGAAGACTTGAAACAGAGAAATCAATCATTGTTGAGAGCCTTAATGATACA

AGAGATCTTAATAAGGAATTGCAGGATCGATTGAAATCTGTTAAAGATTCTCAGAGTGAA

GCTATAAAGCATAAAGAAATGTTCCGCGAAGAACTAAAAAAAATTACAGATGATCGCGAT

CGCCTACAAGGAAAACTAAATGATGTAATGTTAGAACGGGAAAAATACATAAAATTTAAA

TATGAAACATACTCAAAATTGGATAAATTGGCAAGTAAAACAGAGGCACTTGAGGAGACA

TGTTCCTCTCAAAAGGAGAAAATAAATATACTGGAGCAAAAGTTACATGCTGAAAGGGAG

AAGTTAGAGATGGCTGATTTATCTGCTATGGAAACAAGAGCAACGTTTGAAGAGCAGAAA

AGATTCATTCGTAAACTACAAGATCAATTGGCAGATAAAGAATTTCAAGTAGTTGAAGGA

GAAAAGCTAAGGAAAAAACTGCACAACACTGTCTTGGAGCTGAAAGGAAATATTCGTGTG

TTTTGTCGTGTGCGACCTTTACTACGAGACCGTTCAGAAACGAAAACGGTTGTTTATTAC

CCTACGTCAATAGAAATGCTTGGCCGGGGCATTGAGTTGGAACAAAATGCAGGACAGAAA

CAGCTTTTTACATTTGACAAGGTGTTTAATCATGACGCCTCTCAGCATGACGTTTTCACC

GAAATATCACAACTGGTACAGAGTGCCCTTGATGGCTACAAGGTATGCATTTTTGCTTAT

GGACAAACGGGTTCAGGAAAAACCCATACAATGATGGGTAGGCCAGACTCTCCAGATCTC

AAAGGGATGATACCACTTTCTTTAGAACAGATATTCCAGACCAGTCAGTCTCTTAAAGAC

CAGGGCTGGAATTACAAAATGCAGGCATCAGTATTGGAAATATATAATGAGACCATCAAA

GATTTGTTATTGTCAAATCGGTCAAGTGGGATTGACCAAACACGAGCAGAAAATAGTGTT

CATGGAAAACAGTACACTATTAGACATGACAAAAATGGAAACACACATGTTTTGGACCTC

ACCATTGTCGATGTTTGTAGTGCCGATGAGATTTCCTCCCTCCTCCAAAAGGCTGCCCAA

ATCAGGTCAATGGGAAGAACACAAATGAATGAACAATCATCAAGAAGCCACTTTGTTGTT

ACTTTGCGCATATGTGGTATAAACGAGAACACTGAACAACAAGTACAAGGTGTCTTAAAC

CTGATTGATTTGGCTGGAAGTGAAAGACTTTCAAGGAGTGGGGCCACTGGGGAACGTTTG

AAGGAAACTCAGGCTATCAACCAAAGTCTCTCCTGTTTGAGCAATGTCATATTTGCTTTG

GCAAAGAAAGAAGAGCATGTTCCTTTCAGGGATTCAAAACTTACACACCTACTTCAGCCA

TGTCTTGAAGGGGATTCCAAAACTTTGATGTTTGTCAACATCTCACCTGATCAATCTTCA

ATTGACGAGTCACTTTGCTCTCTTCGATTTGCGTCTAGAGTCAATGCTGTGAAATTGGGG

ATTCACAATGTCAGACCTAGACATCCACTCAATGTTTAG

>MS.gene028562.t1

ATGGATCAGGACACTTCAGGGTGGTTGCGGCGAGGAGCAGAATGTGATAGATCAGGTACT

TTTCTGGCTGCGTGTTGGTGGATATGGCGGGCTAGAAATTTATTGTGTATGGAGGATGAG

AACATGTCCATGTTGCAGCTGAAAAGGGAAATTTATAGGTTGGCATCTTTAATCAACAGA

GTCTTCAATTCTGTGAACAATGCAGCTGAGGCACAACCATGGGTTTCTTTGCATCCCATG

GCTGGTTTGGCAACAGTGTTAAATGTTGATGGAAGCAGCTTGGGTAATCCAAGATCATCT

GGTTTTGGAGGTGTTTTACGGCATTCTGACGGTTCTTGGTTGTATGGGTTTGCAGGGCAT

GTTGGCATCTCTTCCATTCTTCATGCGGAACTGCTAGCCTTATATTATGGATTGCGCCCA

GCTTGGGAGAAAGACTACGGGCACATCATTTGCTATTCTGATTCTAATCTTGCTATTCAG

TTAGTAACTACAGGGGAGTTGTTGGCTCGTGCTTGGACTGTCCAGGTGTGTCACACTTGG

AGGGAAGCAAATGTTGTGGCAGACTTTCTAGCAAAGATGGGGGCAAGGAGTAATGTGGAT

TGGCAGGATTTCTTTTCCCCTCCTGACGGATTGCAGCCCCTATTGCAGGTTGATACTTCC

AGAGTGCTATATGCACGTCGTTAG

>MS.gene028561.t1

ATGTCAGCATGCCGATGGAAAAGCTTCGAAGAGAACGAAGACCGCCCCTCCAAGCCTCGC

CGCTACGGCGTCACCGAAATGCGAAGCCCTCATTACACCCTCTTCAACCACAATGTTCTT

CAGGATATTTTTGAATCTATGGGAGATTATGTTGATGGATTGAAATTTTCTGGAGGTTCT

GATAGTTTGATGCCAAAATCTTTTATCAAACAAGTTATTGATACTGCTCATCACCATGAT

GTTTATGTTAGCACTGGTGATTGGGCTGAACATATGATTCACAAAGGTCCTTCAGGATTC

AAAGACTATGTGGAGGAATGTAAGCAGTTGGGGTTTGACACAATTGAGCTGAATGTGGGT

TCCCTTGGAGTTCCTGAAGAAACCCTTTTGAGATTCGTCCGCTTGGTTAAAACTGGTGGT

ATGAAAGCTAAGCCTCATTTTGAAGTTAAGTTCAATAAGTCTGAACTTCCCAGAGGTGGT

GATAGGGCTTATGGGGCTTATATTCCTCCAGCACCTAGATCATATGAATTAGTAGAAGAT

GTGGATCTCTTGATTAGGAGAGCTGAGAGGTGTTTAGAAGCGGGTGCAGACATGATCATG

ATTGATGCTGATGATGTCTCCAAGCATGCTGACAATATGCGCGCAGATGTTATTGCGAAG

ATCATAGGACGCCTTGGTATTGAGAAGACTATGTTTGAAGCATCAAATCAGAGTGCATCC

GAATGGTTTATTAAACAATATGGTCCAAATGTGAATCTTTTCATTGATCATTCCAACTTG

GTAAATGTGGAGTGCATCCGGGGACGCAACTTAGGTAAAAATCATGCTTCTGTTCTTGGC

TCTTCGTATTTCCTGTTCTGA

>MS.gene028563.t1

ATGTCCACGTACAGAACTTTGTTATACAATTTACCGAAGCTAGTCCAAGGAGTGTATCAG

AAGGAGGAGGACCCATCACCCAATCTAAAATCAAAACCTGGTTGTAAGACTGCTTTTGCT

TTCATTACTGAATTCCATACTATGGATCCTTGTCTTTTCGGCATATCCAGAAAAGTTATG

TCGTCAGAGAAGGAAGATTCAAGCTCCACAGTGAAACGCAAATTATCGTGCTTCGAACAC

TTTGATGCACTCTGGTTCTGCTACTCCCCAGTTTATCAAATGCGGCAGTATTACAGGCTT

GGTTCTCTTGATAACTGTCGCGGTAAATGGAAAGCATGGACTGACTGCTTAATGCTCAAG

ACCAAACCCAAATCTCAAGTCGAGTCATTGACTAAGTGA

>MS.gene028564.t1

ATGTCAGCATGCCGATGGAAAAGCTTCGAAGAGAACGAAGACCGCCCCTCCAAGCCTCGC

CGCTACGGCGTCACCGAAATGCGAAGCCCTCATTACACCCTCTTCAACCACAATGTTCTT

CAGGATATTTTTGAATCTATGGGAGATTATGTTGATGGATTGAAATTTTCTGGAGGTTCT

GATAGTTTGATGCCAAAATCTTTTATCAAACAAGTTATTGATACTGCTCATCACCATGAT

GTTTATGTTAGCACTGGTGATTGGGCTGAACATATGATTCACAAAGGTCCTTCAGGATTC

AAAGACTATGTGGAGGAATGTAAGCAGTTGGGGTTTGACACAATTGAGCTGAATGTGGGT

TCCCTTGGAGTTCCTGAAGAAACCCTTTTGAGATTCGTCCGCTTGGTTAAAACTGGTGGT

ATGAAAGCTAAGCCTCATTTTGAAGTTAAGTTCAATAAGTCTGAACTTCCCAGAGGTGGT

GATAGGGCTTATGGGGCTTATATTCCTCCAGCACCTAGATCATATGAATTAGTAGAAGAT

GTGGATCTCTTGATTAGGAGAGCTGAGAGGTGTTTAGAAGCGGGTGCAGACATGATCATG

ATTGATGCTGATGATGTCTCCAAGCATGCTGACAATATGCGCGCAGATGTTATTGCGAAG

ATCATAGGACGCCTTGGTATTGAGAAGACTATGTTTGAAGCATCAAATCAGAGTGCATCC

GAATGGTTTATTAAACAATATGGTCCAAATGTGAATCTTTTCATTGATCATTCCAACTTG

GTAAATGTGGAGTGCATCCGGGGACGCAACTTAGGTAAAAATCATGCTTCTGTTCTTGGC

TCTTCGTATTTCCTGTTCTGA

>MS.gene028572.t1

ATGGTGGATTCCATCAAAAGATTAGGCATGGAGTACAATTTTGAAGAGGAAATTGAAGCA

ACACTTGAAAGGAAACACACCATGCTTAGATTTAAGAGTTTCCAGAGAAATGAATATCAA

GGACTGTCACAAGCTGCATTTCAATTTCGTATGTTGAGACAAGAAGGGTATTACATTAGC

CCAGACATATTTGATAAGTTTTGTGACAACAAAGGAAAACTCAAGCATACATTTTCCGAG

GATATAAATGGATTAATGGCCTTGTTTGAAGCTTCTCAATTGAGCATAGAAGGAGAAGAC

TGTCTTGACAATGTAGGACAGTTCTGCTGCCAATATCTTAATGACTGGTCATCAACATTT

CATGGCCACTCTCAAGCGAAATTTGTGGCACACACCTTGACGTATCCAATTCATAAAACT

TTGTCAAGATTCACACCAACAATAATGCAATCACAAAATGCAGCATGGACAAACTCTATA

CAACAATTTTCAGAAATAGACACTCAAATGGTTTCATCATCACATCTCAAGGAAATTTTT

GCAGTTTCCAAATGGTGGAAAGACTTAGGTTTGGCAAAGGACTTGGAATTTGCTAGAGAT

GAACCAATTAAATGGTACTCGTGGTCAATGGCATGTCTCCCAGGTCCACAGTTCTCCGAA

GAAAGGATTGAGCTCACAAAACCTCTGTCCCTAATATACATCATTGATGATATATTTGAT

TTTTATGGAAACATTGAAGAACTTACTCTCTTTACAGATGCTGTTGAGAGATGGGACTTG

GCAGCTATTGAACAATTACCAGACTGCATGAAAGTGTGTTTCAAGGCTTTCTATGACATA

ACCAATGAATTTGCTCTTAGGACATACATCAAACATGGATGGAATCCTTTAACCTCATTA

ATAAAATCGTGGGTAAGACTCATGAATGCTTTCTTGCAAGAAGCAAAATGGTTTGCTTCT

GGAAATGTTCCAAATTCAGAGGAGTATTTGAAGAATGCCATAGTAAGCACTGGAGTACAC

GTGATACTTGTGCATGCTTTCTTTTGCATGGGTCAAGGTATAACTGAGAAAACCGTGTCT

CTAATGGATGACTTCCCAACCATTATATCTACAACAGCCAAAATTCTAAGGCTATGTGAT

GACTTGGAAGGCGACAAGGATGTTAATTGTGAGGGTAATGATGGGTCATATTCGAAGTGT

TACATGAAAGATAACCCAGGAGTTTCTATTGGACAAACAAAGGAGCATATGAGTGAACAA

ATTTCAGATGCATGGAAACAACTCAACAAGGAATGTTTGAACACAAATCCATTACCATCA

TCTTTTACTAAACTTTGTCTCAATGCTGCACGGATGGTACCTATAATGTACAATTATGAT

GGCAATACTTCTTCAAAGCTAGAGGAGTATGTGAAATCGTTGCTTTATGATGGTGGTTAC

TTACAAACTATTCATTCCCCAACATCAAAGCATTCTGCAGTATGA

>MS.gene028578.t1

ATGGAACAAGTAAATAACAATAGACTATGTGTGTCTATGGAAGAGTTGGGTTTCAATGCT

GAAAATGGTAGCAATAGGGGAAACTGGAGAAGGAACAAGCACCGTGATAATGATGGTGTC

CCAAAAGTGTTCACATCCAAGAACCTTGAGACAGAAAGAAAGAGAAGGGAGAAACTCACT

AGCAGACTTTTGGTGCTTAGGTCTTTAGTACCAATCATCACAAATATGAACAAAGCAAGC

ATTATTGAGGATGCTATCACTTATACCATGAAGTTGCAACATGAAGTTGAGAGTCTCACA

ATAGAGCTTGATGGAATGGAACCAAGAAGAGAGAAAAGAGTTGAGCCAAAACAAAGAGAG

TCTTCTGCTGTTGATGAGATGAATAAAATGGGGTTACAGGAAGAGGTGCAGGTAGCAAAA

GTTGATGGAAATAAGCTATGGATTAAGATGCTCATTGAGAAGAAAAGAGGAAGGTTCAAC

AAATTAATACAAGACTTGAATGATTTGAACATTGAAATGGTAGACCTCAGTGTTACTACT

ATGGCAGGAGCATATCTCATTACAGCCTCCTTGCAGGACATGGGTGGTGAACCACTTGAA

CTTTATCGAATTCCAACTATTATCAAGTATTAG

>MS.gene028569.t1

ATGTCGAAGAAGAAAGTGAGTGGAAGCACCATGACACTCAAGGACTTCCACGGCGGTTCT

ATTCCCTCCGATCTCCCTCTCCCTTCCGCTCCCGGCGTAACTGTTAGGCCTTCAGATCGA

GTTGGTTATGATCGGCCTGCAACGTGGGGGAATTCGATGGGGAGACCGGATCATTGGTCT

CGGCCCCACACGTCTCCGGCAACTAGACATTACGATGATAAATCGATGTTTCTTCCACAT

ACTGCTCCGATTGGTAGGAATTTTGATGAGGATGAGAGGAAGCCGTTGGATGGTAGTTCG

GCTCCGAGGAGAACGATTAGTGATGAGAGTATTCGTGCTCCTTTGCCTTCTCGTGTTGAG

GTGAAGCGAAGTAGTTCGTTGAGTAGGCAAGTGGCTGCTCCGGTGTCACCTGTTAATGTT

AATTCCTATTCGGCTAGGCTTACCGAGGCAACTCATGTGGGAAATAATTCCCAGAGTTTG

GGGGTTAGTGGTGGTGGTGGTGGTCATCCGAATGTGTGGTCAATGAGGAAGGAGGTGGCG

AATGCTGTTGAGTCAGAACAGTCTGCTTGGGCTAGTGCGAATGCTGTTTCCAAGTTGGCT

CATGCGAGTGCGCTTGATAAGGTATCATCCGGTAGATGGCAGACGGTTCATTATCAGACA

GAGGTTGAGGCGGTTAGACCTACTAGAGTGGAGGGTAGACCGCATGCGTATATTAATAGT

AATAGGATAGATACAGTGGTTGAAAAGGAGCATTCTGATGAAATGCTAGCAAGACATGCT

GAAAGGGGTTTAGTTATTGATAATCAGATGCAAGGTGGTGGTAAGAATGAGTTACTCGAA

CATGAAAGGTTGGGGGTTCATAAGTATTCAGATGTACGGCCTAGAAGTGTAGCTCAGTTT

TCCGATGGAGTTCAGCCTGCTCGAACTGATGGCAAAGCTGTTGGCTCAGAATCGCAGCAC

CCTATAGCTTCTGAACCTATTGAACGACCTAAACTTAAGTTGCTGCCGAGAACAAAGCCA

CTGGAAAGTTCCGAACCTTCTGTTATAGAACACACACAGGGATATCGTCAAGTTAATGAT

TCTGTCCACGTCGAAACTGTTTCTCAGGGACATGGACATGCTAATTTTGTGAAGTCTGTT

TCGGCTGGAACCGAGAGTCCAAAGGATCCAGGACAGCGTCCAAAGCTGAATCTGAAGCCT

CTGAAACCTCGGCCTGAGGTCCATGAACAGTCGGAAGCAAACCAGGAAAGAGACAGGAAT

GCTTTATTTGGTGGAGCTCGCCCTCGAGAACTAGTTTTGAAGGAGCGAGGGGTTGATGAT

GTTGCAATAAAAAACTATGACGTGGTTGAGAATTCAAATAGGGTTGAACAAAATACTCCT

AGGTCCGAGAAACACCATGATCATTCAATCCAGACTCGATATGTCGAAAAAACTGAGGAT

GGTCTCAATCAAAGAGCGGGCAGAAAACCTGAAAGGAAGGAGCAAAAGGTAGATGGTGAC

AGGGCTCCTGGACAGAGGAAGAATTGGCGCAGCGGTGATAATAACAATAATAATAACCGG

AGAAACCCGAGGGAAACTGACAGGCAGCAAGTTCCTGAGAGGCAACCTTCACCTGAAACA

TGGCGCAAGCCTGTGGAATCATCTCAAGGTGCCGGTGGCCCACGCTACGGTAGAGCAGCT

TCAGCCGTTGAACTTGCCCAAGCATTCTCTAAATCTGTGTCAGATCCCAAAGTAAATAAT

GATAGGTTTTCAGGTCAAAGGGGTTTGAATGCTGGCAGGACGCAAGTGCCCTTTTCACGG

CTTGTTGGTCCTACTTCAAGGCCTCAGATCAATGGTTATTAA

>MS.gene028579.t1

TTACTATTCTATGTACGGACATGTTGAGAAGCTAGCTCAAGAGATTTTAAAAGGCGCGGC

TTCCGTGGAAGGAGTAGAAGCAAAGCTATGGCAGGTACCTGAAACTCTGCCTGCGGAGGT

CGTTGGGAAGATGGGAGGACCTCCAAAGAGTGATGTGCCAATTATTACACCTGATGCGCT

ACCTGAGGCTGATGGTTTGTTGCTTGGCTTCCCGACAAGATTTGGAATGATGGCTGCTCA

GTTTAAAGCATTCCTCGATGCAACTGGTGGCCTATGGCGTACTCAGGCACTTGCAGGAAA

GCCTGCAGGAATCTTTTACAGCACTGGTTCTCAAGGAGGAGGACAAGAGACTACACCGTA

A

>MS.gene028574.t1

ATGAAGATGGCACAAGTGTTCCTATCAAAGAACACTGGGTCAGAAAGAAAGAGGAGACGG

AAACTCAACACCAAACTCTTCATGCTTCGGTCTTTAGTACCAATGGTCACAAATATGACA

AAAGAAAGCATTTTTGACGATGCTATTACTTATATCAAGAAGCTTAAAGATGAAGTTAAG

AGTCGCACGCAAGAGCTTCAAGCGATGGAAGCAAAAGAAAAATATGAGGAAAGAGCTGAG

CCAAAATTAGATGATTTTTCTGCTGCAGAAGAGATGAAAAAATGGGGGATACAGGAAGAG

GTTCAAGTGGAAAAAATGGATGGAAATAAGCTATATGTTAAGATGATCTTTGAGAACAAG

AGAGGAGGGTTCAAGAAATTAATGGAGACTATGAATGATTTAGGACTTGAAATGATAGAA

CCCAATGTAACAACCATAAAAGGAGCATATCTGATTACAACTTTCATAAAGGGAATGTGT

GGTCAGCCACTTGATGTTCATCAAGTCAAACATTTGTTGCAAGATATAATCAAGAGCATT

TAG

>MS.gene028575.t1

ATGTCTCTAACACCTAGGTGCCATGTCCCCTTGTTCCTTCTTATCCTTGTTTTCATTCTT

TCGCTCACATGGACAACTGAAGAATCTTCTCTTGAAAAGATTCTCCATAACCATGGCCTC

CCAGCCGGTCTCTTCCCTCAAAGTGTAAAATCTTTCAAATTGGACCAAATGGGTCATTTA

GAGGTACACTTAGATCGTCCATGTTTGGCTCAGTATGAAACAACAATGTTCTTTGACACT

ATTGTGAAAGCCAACCTCAGTTTCGGACAACTTAAGATTTTGGAGGGCATGTCTAGTGAA

GAGCTTTTCTTGTGGCTACCTGTTAAGGATATTATGGTTATTGATCCAACTTCAGGTTTT

TCGTTTCGTATGGACGGAAGAAAGGGCATTGGCTTTGAAGATCAGAGATAA

>MS.gene028573.t1

ATGTATCGCATTCTTCAACGCAGCGTTCATACCGTAACAGAAAAATCACCCAACAACATC

AAATCCATTTCACAAGACCTCTACAAAGAACAAAATCTCAAAACACTCGTTGAAAAATTC

AAAAAAGCTTCAGACATTGATCGTTTCCGTAAAAAAAACGGCATCTATGAAGACACCGTC

CGCCGCCTCGCCGGAGCTAAACGCTTCCGGTGGGTCCGTGACATTATCGAGCATCAGAAG

AGTTACGCCGATATATCAAATGAGGGTTTCTCGGCGCGTTTGATTACGTTATATGGGAAA

TCGAATATGCATAGACATGCACAGAAACTGTTTGATGAAATGCCTCAGAGAAACTGTGAG

CGTTCTGTGCTTTCTCTCAATGCGTTGTTGGCTGCTTATCTTCATTCGAAGCAGTATGAT

GTCGTTGAGCGGCTTTTTAAGGAGCTTCCTGTTCAATTATCTGTGAAACCTGATTTGGTT

TCTTATAATACTTATATTAAGGCTTTGTTACAAAAGGGTTCTTTTGATTCCGCGGTTTCG

GTGCTTGAGGAAATGGAGAAGGATGGTGTGGAAAGTGATTTGATTACGTTTAACACGTTG

CTTGATGGGTTGTACTCGAAGGGTCGTTTTGAGGATGGTGAGAAGCTTTGGGAGAAATTG

GGTGAGAAGAATGTTGTTCCGAATATCAGGACTTACAATGCAAGGTTGTTGGGATTGGCT

GTGGCGAAGAGAGCTGGTGAAGCTGTTGAGTTTTATGAGGAAATGGAGAAAAAGGGTGTG

AAGCCGGATCTTTTTAGCTTCAATGCTTTGATTAAAGGTTTTGCGAATGAAGGGAATTTG

GATGAAGCTAAGAAGTGGTTTGCTGAAATAGAGAAATCTGAGTATGATCCTAGTAAGGGA

ACTTATTCTATAATTGTTCCTTTCTTGTGTGAGAAGGGTGATTTGAAGACGGCTATTGAG

ATGGTTAAGCAGATATTTCACACTCGCTGCCGTGTTGATGTGTCGTTGCTGCAGACTGTG

GTGGACAAACTGGTGAGTGAGTCCATGGTTTCAGAGGCCAAGGAGATTGTGGAACGAGGT

AAAAACAATAATTACTGTCGCTATAAGCTAAATTCGCCAGCAGATGAGTGA

>MS.gene028565.t1

ATGGCACGTATAGCGCTAATGCAGGATACAAATGGCTATTTCGTCAAACAGATAATATTA

TGCCGCAAGCAGCTGCATCTTGGAGATGGATTTGGTGTATTGCAGCCCCAGAGAAGGGAT

TGTGGTTATGTTACTCGCCTATGGATATCACTTGGTTTCGACAATATCAATTTTTTCATG

GATCAGGACACTTCAGGGTGGTTGCGGCGAGGAGCAGAATGTGATAGATCAGGTACTTTT

CTGGCTGCGTGTTGGTGGATATGGCGGGCTAGAAATTTATTGTGTATGGAGGATGAGAAC

ATGTCCATGTTGCAGCTGAAAAGGGAAATTTATAGGTTGGCATCTTTAATCAACAGAGTC

TTCAATTCTGTGAACAATGCAGCTGAGGCACAACCATGGGTTTCTTTGCATCCCATGGCT

GGTTTGGCAACAGTGTTAAATGTTGATGGAAGCAGCTTGGGTAATCCAAGATCATCTGGT

TTTGGAGGTGTTTTACGGCATTCTGACGGTTCTTGGTTGTATGGGTTTGCAGGGCATGTT

GGCATCTCTTCCATTCTTCATGCGGAACTGCTAGCCTTATATTATGGATTGCGCCCAGCT

TGGGAGAAAGACTACGGGCACATCATTTGCTATTCTGATTCTAATCTTGCTATTCAGTTA

GTAACTACAGGGGAGTTGTTGGCTCGTGCTTGGACTGTCCAGGTGTGTCACACTTGGAGG

GAAGCAAATGTTGTGGCAGACTTTCTAGCAAAGATGGGGGCAAGGAGTAATGTGGATTGG

CAGGATTTCTTTTCCCCTCCTGACGGATTGCAGCCCCTATTGCAGGTTGATACTTCCAGA

GTGCTATATGCACGTCGTTAG

>MS.gene028566.t1

ATGTCGTCAGAGAAGGAAGATTCAAGCTCCACAGTGAAACGCAAATTATCGTGCTTCGAA

CACTTTGATGCACTCTGGTTCTGCTACTCCCCAGTTTATCAAATGCGGCAGTATTACAGG

CTTGGTTCTCTTGATAACTGTCGCGGTAAATGGAAAGCATGGACTGACTGCTTAATGCTC

AAGACCAAACCCAAATCTCAAGTCGAGTCATTGACTAAGTGA

>MS.gene028567.t1

ATGGCTAGTATCAAACAAATAGAAAAGAAGAAGGCATGTGTGATAGGTGGCACTGGTTTT

GTGGCATCATTGCTGATCAAGCAGTTGCTTGAAAAGGGTTATGCTGTTAATACTACTGTT

AGAGACCCAGATAGTGCAAACAAAACATCTCACCTCATAGCACTGCAAAGTTTGGGAGAA

CTGAATCTATTTAAAGCAGAATTAACAGTTGAAGAAGATTTTGATGCTCCTATAGCAGGA

TGTGAACTTGTCTTCCAACTTGCTACACCTGTGAACTTTGCCTCTCAAGATCCCGAGAAT

GACATGATAAAACCAGCAATCAAAGGTGTATTGAATGTCTTGAAAGCATGTGCAAGAGCA

AAAGAAGTCAGAAGAGTTATCTTAACATCTTCAGCAGCTGCTGTGACTATAAACGAACTC

GAAGGGACTGGTCATATTATGGATGAAACCAACTGGTCTGATGTTGAGTTTTTGAACACT

GCAAAGCCCCCCACTTGGGGTTATCCTGTCTCAAAAGTGCTAGCTGAAAAGGCTGCATGG

AAATTTGCCGAAGAAAATAACATTGATCTAATCACTGTGATACCTACTCTAACAATTGGT

CCTTCTCTAACACAAGATATCCCATCTAGTGTTTCCATGGGAATGTCACTTCTAACAGGC

AATGATTTCCTCATAAATGCTTTGAAAGGAATGCAGATCCTGTCAGGTTCGATATCCATC

ACTCATGTTGAGGATATTTGCCGAGCTCATATATTTGTAGCAGAAAAAGAATCAGCTTCT

GGTCGATATATTTGTTGTGCTCACAATACTAGTGTTCCTGAGCTTGCTAAGTTTCTCAGC

AAACGATACTCTCAGTATAAAGTTCCAACTGAATTTGATGATTGCTCCAGCAAGGCAAAG

TTGATAATCTCTTCTGAAAAGCTTATCAAAGAAGGGTTCAGTTTCAAGCATGGTATTGCT

GAAACTTTTGACCAAACTCTGGAGTATTTGAAGACTCAGGGGGCCTTGAAGAAGTAG

>MS.gene028576.t1

ATGTCTCTAACAACTAGTTCTCGTGTCTCCGTCTTCATTCTTTTCCTTGCCTCTGTCTTT

CCTCTCTATTGGACAGTCGAAGAATCTTCTCTTGAAAAGATTCTCCGCAACCATGGTCTT

CCAGCTGGTCTCTTCCCTCGAAGTGTGAAATCATTCAAATTCGATCAAATGGGTCTTTTA

GAGGTATACTTAGACCGTCCTTGCCTGGCTCAATATGAAACCACAGTGTTCTTTGATACT

ATTGTGAAAGCTAACCTCAGTTTCAGACAACTTCAAGTTTTAGAGGGTATGTCTCGTGAA

GAGCTTTTCTTGTGGTTACCTGTTAAAGATATCTTTGTTGTGGATCCATCTTCTGGTGTA

ATTCTCATTGATATTGGTTTTGCTTTGAAGTTTCTCGCCTTCTCTCGTTTTGATGAGCCA

CCAATTTGTAGATCTCATCTTGGACTTTCATTTCGTATGGGTGGAAGAAAAGGTACTGCA

TTGGAAGACCAGAGATAA

>MS.gene028570.t1

ATGTTAGCGAAGATCGGGTTGCCACCAAAGCCTTCTTTACGAGGGAATAATTGGGTCGTT

GATGCCTCTAATTGCCAGGGTTGTTCCGCTCAATTCACCTTCATCAATCGCAAGCACCAC

TGTAGAAGGTGTGGGGGTCTATTTTGCGGAAGTTGTACCCAACAAAGAATGGCTTTGCGT

GGACAGGGTGATTCGCCTGTACGTATATGTGAACCTTGTAAGAAGCTAGAAGAGGCAGCA

CGATTTGAGTTGCGACAGGGACGCAGGGCTGGGAGAGGAAGCTTGAAATCAGCACCTAGA

GATGAAGATGAAATTCTCAACCAGATTCTTGGTCAAAACGAGGAACTTCTTTTATCAGGA

AAGCAATCAACCAGTGACAAAGGTCGTAGTGGCCAGAGATCAGTTAGCGCTGCATCTTCT

TCAAGTACTACAGGATTTTCTATTCAGGACGAAGAAGATATACAAAAGATTATATCAACT

GAAACAACGAATAGTATGGCTGTTGATGTGGGATCCACCACTCCTGATGAGTTACGTCAG

CAAGCTTTAGCGGAAAAAAGTAAGTATAAAATTCTGAAAGGAGACGGGAAATCTGAGGAA

GCCTTGAAAGCTTTTAAGAGAGGGAAGGAGCTTGAGAGGCAAGCAGATGCTTTGGAAATT

CAGCTAAGGAAAGCGGCTCGGAAAAAGTTGTTGCCTTCTGGAAACATGTCGGATATGCAC

AACAAAGACATTTCTGTTGAGTCTGGCAGAAAAACAAAGTCACTTCCTCAGACTGGTAAA

GATAAGGATGATCTTACTTCAGAACTTCAAGAGCTGGGATGGTCTGATTTGGAACTACAC

AAAGAAGATAGAAAGTCAGCAAACTTGAGTTTGGAGGGTGAACTCTCCTCCTTGATTGCA

GAAACTTTTGCAAAGACAGGTGAAGAAAAGGGCAGCCGGATTGACAAGACTGAGGTTGTT

GCTATGAAAAAGAAGGCTCTAACGTTGAAGCGTGAGGGTAAGCTTGCAGAAGCTAAAGAG

GAATTGAAAAAAGCAAAAATCCTAGAGAAGCAACTGGAAGAACAGGAACTCCTTGCTGGT

GCTGAAGATTCTGATGATGAGCTATCAGCATTAATACGCGGCATGGATGATGATGATAAA

GAATTTCCAAATCTGCATGATCATGAGCATGGTTTTGATTTTGATAATCTCTTGGCCATT

TCTGATAATCTTGATGGTAATTTAGAAGTGACCGATGAGGATTTGATGGACCCTGAGTTA

GCTGGTGCTTTGGAATCACTGGGTTGGACTGAACCTGAAAATACATTTTCCAAATCTCAA

ACCTTTGACAAAGAAGCGTTGCTAAGTGAAATTCAGTCATTGAAAAGAGAGGCTCTTAAT

CAAAAGCGAGCAGGTAATACTGAAGAAGCAATGGCGATATTGAAAAAGGCGAAGTTATTA

GAAAGTGACTTAAACAACACTGGGTCTGATGACGATAATACTATGTTGCAAAAAGTTACT

CCTGTTGGGAAAAGTTTGAGTTCTGAAATTACTGGTAATGATAGAAATAATAATGCCGCT

TCCACGGTGGCACCAAAAAGCCGGTTGATGATTCAAAGAGAGCTTTTGAATTCGAAAAAG

AAGGTTCTTGCATTGAGAAGGGAAGGGAAAATGGATGAAGCAGAAGAAGAAATGCGGAAG

AGTGCAGTTCTTGAGCATCAGCTGATGGAGATGGACAATGCTCCGAGTCAATCATCATCG

ACGAATACTGGTAATGTCCTGCATGCAGCGAGTAGAAGTCCTCTAGTTGAGGAAGGAAGT

GGCGATGATGTAACAGATAAAGATATGTCTGATCCAGCATATCTTTCACTCCTTACGGAC

TTGGGTTGGAATGATGACAATGATAAATCTTCTAAATCTTCGAACAATCCTTCGAAGACA

TATGATGATAACTTTGTTCCAGTTGATGATACTTTTCTGAGTAAGCATTCTACAACTATG

TTTGTTGAAGCACCAAGAAAAAGTAAAGCAGAAATTCAGAGGGAACTCTTGAGCTTAAAA

AGAAAGGCCCTTGCTTTAAGACGGGAAGGGAAAACGGAGGATGCAGAGGAAGTCCTTAAA

ATGGCCAAAACATTGGAAGCCAAGATAGAAGAGATGGATGCTCTGAAGAATAATGTACAG

GTTGAGGCTCTTAAGAAGAAAGAACTCTTCAATTCTCCAGTTGATGTGGCAGTTGATGAA

GAAAGGGACATGGTTGTTTCAGAAGAGGATATGCATGACCCAGCATTGAATTCAATGCTT

ACTAATCTAGGATGGAAGGATGAAGGATTTGAGCCCGTGACCATAAAAGAAGAACCTGTT

AAGGAAGCTACTAGTACTGTCACAACTTCAAGGAACAAAGGAGAGATTCAAAGAGAACTC

TTAGCGTTGAAAAGGAAGGCACTTGCTTTGAGGCGCAAGGGAGAAATAGAAGAGGCTGAG

GAAATTTTAAAAAAGGCCAAGAATCTGGAAGCCCAACTGGAAGATTTTGAGAGCCAAAAC

AAGGACTTGTTGCTGAATGCATCCAAGGATAAAAAATCTGTTCCGTCTGAATCATCTCAC

GGAAAAAGTCCTGCAAACTCCCATTTTGCACATGATGAGCATCCATTATCTGCCGAGGTG

AGTGCTTCAAGTGAAAATCTCACAAAAAGGATGAAAGTCGAAAATATCATTGCTCATAGC

TCCTCAACTGGCCACTCTATGCATATGCCAGATCTACTTACTGGTAATGGCTGTAGCAGT

TCTGAAATTTTGTCTCAAAAACAAAAAGAAGAATATAAAGCAGGTTCAGTTAATTCATCT

CAAGCGGGTCCTACTATTCCTTTGGACTCCTCAGTGAATCTCAGCCAAGACCAGATATAC

AAAAACAACATTCAAACCCAAAGAAGAAAAGAAGTGACTGATGTTGATGAAAAGCCAAAC

ACAAACCAGTCAAATGTTGTTCCAGATTATGCTTCTCAGGACGACCTTTCTTTGCGTCAA

GAAATTTTGGCTCATAAAAGGAAGGCAGTTGCTTTGAAAAGAGAAGGAAAACTAACAGAA

GCCAGGGACGAACTTCGACAGGCCAAGCTGTTAGAGAAGAGGTTGGAGGATGGAAGTATG

CAGCCAAACACTGTCTCCACCAGCAATGTTTCCAACACATCAAATGTTGTTCAAAAGAAA

CAAGATTCCCCGAATGCTGCTGCAAAGCCGTTTACTAGTCGTGACCGTTTCAAGTTGCAA

CAGGAGTCTCTAGCACACAAACGTCAAGCCCTAAAGCTACGGAGAGATGGCCGAACAGAG

GAAGCAGAAGCTGAGTTAGAACGGGCAAAGGCAATTGAAGCGCAGTTGGAGGAGTTGGCT

GCTGAGGATGCCGATAAGTCAGATGCGGTGGATGATGTGAGTGTTGAGGATTTTCTTGAC

CCTCAACTCTTATCTGCTCTGAAAGCTACTGGACTTGCAGATTTAACTGTTGTATCTAAT

AAAAGCCCAGAAAAACAAGAGACTGTGAAACCCGTTGCCAAAATCGAGAACCCTAACCAA

GAGAAAATTCAGCTAGAAGAAAGAATTAAAGAAGAAAAACTGAAGGCGGTTAGTTTGAAA

CGATCAGGAAAACAAGCTGAAGCCTTGGATGCTCTTCGACGTGCCAAAATGTATGAAAAG

AAGCTGAATTCATTGTTAACATCTGGGTGA

>MS.gene028577.t1

ATGTCTCTGACACCTTCGTCTCTTGTTCCCATTTTCCTTTTGGTCGTTGTTTTCATTTTT

CCTCTCACAGGGATAGCTAAAGAATCTTCTCTTGGAGAGATTCTTCGAAACCATGGCCTT

CCAGCTGGTCTCTTTCCTCAGAGTGTGAAATCATTCAAATTGGATCAAATGGGACGTTTA

GAGGTACACTTGGATCGTCCTTGTTTGGCTCAATATGAAACCACTGTGTTCTTTGACACT

GTTGTAAAAGCCAACCTCAGTTTCGGACAACTTAAGGTTTTGGAGGGTATGTCTCGTGAA

GAGCTTTTCTTGTGGCTACCTGTTAAAGATATCATTGTTACTGATCCAAAATCTGGTGTA

ATTGTCATTGATATTGGTTATGCCTTCAAACGTCTCTCTTTCTCTCGTTTTGACGAACCT

CGCATTTGTAGATCTCATCATGGACTTGCGTTTCCAATGGGCGGAAGGAATGATATTGGT

TTTGGAGATCAGTGA

>MS.gene028571.t1

ATGGCACTGCATTTGCAATCATGCCTTTCCTCTTTGAAACCTCAAATCGTCCCAACAATT

GCACAACTTTCTCAAAACTTTAATAATCTCAAGCTTGATTCTCTTCACATTGCTAACAAG

TGGAGCATTAATGTTGAAGAAGAAAGGCGTAGTGCACTCATAAAACAACATAGAGATCTC

AGCAGCAATAATAATCATTCTGAGAAACTTGAGGTGGTTAAGCATGAACTTAGAAACGTT

GGTGAAAATTCACTCAAAGGTTTGTATATGATTGATGCCATGCAGCGCCTAAATATTGAT

TACCACTTTGAAGAAGAAATTGAATCATTCCTAAGAAGACAATATGTCGCTAGTGCTTGT

GGTGGAAGTAATCATCATAATCTTCATGAGACCGCACTTCACTTCAGGCTATTAAGACAA

CAAGGTCACTTTGTTCCCGCAGAGGTGTTTAACAAGTTCACTAACAAGGAGGAAAAGTTT

GATCCAAAACTAGGTGAAAATATCAATGGAATGATAGATTTATTTGAAGCCTCGCATCTA

AACCTGGCAGGGGAAGACATACTTGATGAAGCTGGAAAATTTAGCAGGAAGATCCTTAAG

GAAAAAATGGCACAATTTGATTTTCACGAAGCTATGTTTGCAAGGAGAACCTTAGAGTAT

CCTTTTCACAAAAATTTGCCAATGTTCACAGCTAGAAATTTCTATAGTCATCTATATAGC

ACAAATGCATGGTTTGGTTCTATGAAAGAAGTTGCGAAAATGGATTTCAGTTTGCTGCAA

GGCTTACACCATCAGGAGATTGTTCAAATTTCAAAATGGTGGAGAGAACTTGGTTTAGCT

AATGAATTACCATATGCTAGAAACCAGCCCCTTAAATGGTACATGTGGTCATTGGGATGC

CTCACGGATCCTACTTTATCTGAGGAAAGAATTGAGCTCACAAAACCAATATCTTTAATC

TACATAATTGATGACATTTTTGATATTTATGGGACTCTAGATGAGTTAACTCTGTTCACT

GAAGTTGTTTCTAGATGGGATATTGACACAGATATGGAGCAACTACCAAATTACATGAAA

ACAGGTTTCAGGGTCCTCTATGATCTAACAAATGAACTAAGCTCCAAAATATATAAGAAG

CATGGGTGGGACCCTAAAGACTCTCTTCGAAAGACGTGGGAGAGTTTGTGCAAAGCCTTT

CTAGTTGAAGCAAAATGGTTTGGTTCAGGGAAGTTGCCAAGTGCTGAAGAATACTTAAAG

AATGGGATAGTAAGCTCTGGAGTGCATATTGTGCTGGTTCACATTTTCTTTCTTTTGGGT

AAAGGATTAACTAAGGAAAATGTTCATACAATGGACACAACCCTATCCATTATTTCCGCC

CCTGCCACAATTCTTCGACTTTGGGATGATTTAGGAAACGCAGAGGATGAAAATCAACAA

GGAAATGATGGATCATACGTGAAGTGTCTAATGATGGACCAACCGGAGTATTATTGTACA

AGAAGAAGAGCAACAGACGAGGTTATGAATAAAATATCTAATGCATGGAAAAGCCTCAAC

CAAGAGTGTCTCTTTGATACCCATTTCCATAAAGCATTCACAAAGGTCTCACTCAATCTT

GCAAGGATGGTACCATTGATGTATAGTTATGATGATAAGCACTCCCTTCCTGGGCTAGAG

GGTTATGTCCATTCCTTGCTTTATGATAAAGTTTTTTGA

>MS.gene028568.t1

ATGAATTCGCACGAGATGAGCAGAACTCTTTTAATTTTGGCCATAGGAATATGCATAGGC

TTTGTGATTGGAGCTGGTTTGGTATTTTCAATTTTAGTCTTCTGCAGGCCTGGAAGGAAG

CGTGTAGAGGTGGAGAAGAGCGGTCCTCTGAGAACCGAGGCCATTAATGTTCATGGCAAA

GGGGCTGATTCCAGTGTAACATCATTATCAGACTCCAACGCGACTTTTGAGTCACCAAGA

ACATCGGAATGGAGTAACACGCCTTTCTGGTTAGAAGGACTTAGGAGGAAGAATGCTGTC

TCAGTATGTGGAATTCCCAAATATTCTTACAAAGATATAGAAAAGGCTACATCTAATTTC

ACAACGGTCATAGGCCATGGAGCATTTGGTCCTGTTTACAAAGCAATCATGCCTACAGGC

GAGGCAGTTGCGGTTAAAGTTCTCGGTGCTAATTCAAGACAAGGAGAACAAGAATTTTTA

ACTGAGGTCCTATTACTTGGAAGATTACATCATAAGAATCTTGTGGGTTTGGTGGGATAT

GCTGCAGAAAGAGGAAAGCACATGCTTCTTTACATTTACATGAGCAACGGCAGTCTTGCT

TCTCATTTGTATGGCGAAAATCATGAGCCATTGAGCTGGAATTTGAGACTTAGCATAGCA

CTAGATGTTGCAAGGGTGATGGAATATCTACATTATTCGATCGATCCTCCTATCGTGCAC

CGTGACATCAAGTCTTCAAACATTCTATTAGACAAGTTTATGAAAGCCAAGGTCACCGAC

TTTGGGCTTTCCAGACCAGAAATGATTAAACCTCGTCTGTCAAATGTCAGAGGAACTTTT

GGATATCTTGATCCCGAGTATATCTCTACAAGAACCTTCACTAAGAAAAGTGATGTTTAT

AGTTTCGGTGTGCTACTGTTTGAGCTTATTACTGGCAGGAATCCACAGCAGGGTCTCATG

GAATATGTAAAACTGGCTGCCATGGAAAGTGAGGACAAGATTGCATGGGAAGAAATTGTG

GATCCGCAACTGAATGGAAACTATGATGTACATAAGCTTAATGACATGGCTTCACTTGCA

CTCAAATGTGTCAATGGTGTCTCCAAAATCCGACCTTCAATGAGGAACATTGTTCAAGCA

TTATCTCAACTTTATAAGAAGCCTAAGAGAAATTCTAGCCGAGTAGCTCCTCCTGCGTTA

AATGAAGTGTCTGTTGAAGTGATGCTGCCAGAAACTCGTGACTTTTCTATTGAGTGTTCA

GAGGAGCTGCGCAGATTGCACAGCCGATAA

>MS.gene051299.t1

ATGTCAGCATGCCGATGGAAAAGCTTCGAAGAGAACGAAGACCGCCCCTCCAAGCCTCGC

CGCTACGGGCGTCACCGAAATGCGAAGCCCTCATTACACCCTCTTCAACCACAATGTTCT

TCAGGATATTTTGAATCTATGGGAGATTATGTTGATGGATTGAAATTTTCTGGAGGTTCT

GATAGTTTGATGCCAAAATCTTTTATCAAACAAGTTATTGATACTGCTCATCACCATGAT

GTTTATGTTAGCACTGGTGATTGGGCTGAACATATGATTCACAAAGGTCCTTCAGGATTC

AAGACTATGTGGAGGTTTGATCTTGCACCTTTTTTCTTATTGTGTGAATGTAAGCAGTTG

GGGTTTGACACAATTGAGCTGAATGTGGGTTCCCTTGGAGTTCCTGAAGAAACCCTTTTG

AGATTCGTCCGCTTGGTTAAAACTGGTGGTATGAAAGCTAAGCCTCATTTTGAAGTTAAG

GCTTATGGGGCTTATATTCCTCCAGCACCTAGATCATATGAATTAGTAGAAGATGTGGAT

CTCTTGATTAGGAGAGCTGAGAGGTGTTTAGAAGCGGGTCATGCTGACAATATGCGCGCA

GATGTTATTGCGAAGATCATAGGACGCCTTGGTATTGAGAAGACTATGTTTGAAGCATCA

AATCAGAGTGCATCGAATGGTTTATTAAACAATATGGTGAATCTTTTCATTGATCATTCC

AACTTGGTAAATGTGGAGTGCATCCGGGGACGCAACTTAG

>MS.gene051300.t1

ATGTCAGCATGCCGATGGAAAAGCTTCGAAGAGAACGAAGACCGCCCCTCCAAGCCTCGC

CGCTACGGCGTCACCGAAATGCGAAGCCCTCATTACACCCTCTTCAACCACAATGTTCTT

CAGGATATTTTTGAATCTATGGGAGATTATGTTGATGGATTGAAATTTTCTGGAGGTTCT

GATAGTTTGATGCCAAAATCTTTTATCAAACAAGTTATTGATACTGCTCATCACCATGAT

GTTTATGTTAGCACTGGTGATTGGGCTGAACATATGATTCACAAAGGTCCTTCAGGATTC

AAAGACTATGTGGAGGAATGTAAGCAGTTGGGGTTTGACACAATTGAGCTGAATGTGGGT

TCCCTTGGAGTTCCTGAAGAAACCCTTTTGAGATTCGTCCGCTTGGTTAAAACTGGTGGT

ATGAAAGCTAAGCCTCATTTTGAAGTTAAGTTCAATAAGTCTGAACTTCCCAGAGGTGGT

GATAGGGCTTATGGGGCTTATATTCCTCCAGCACCTAGATCATATGAATTAGTAGAAGAT

GTGGATCTCTTGATTAGGAGAGCTGAGAGGTGTTTAGAAGCGGGTGCAGACATGATCATG

ATTGATGCTGATGATGTCTCCAAGCATGCTGACAATATGCGCGCAGATGTTATTGCGAAG

ATCATAGGACGCCTTGGTATTGAGAAGACTATGTTTGAAGCATCAAATCAGAGTGCATCC

GAATGGTTTATTAAACAATATGGTCCAAATGTGAATCTTTTCATTGATCATTCCAACTTG

GTAAATGTGGAGTGCATCCGGGGACGCAACTTAGGTAAAAATCATGCTTCTGTTCTTGGC

TCTTCGTATTTCCTGTTCTGA

>MS.gene052579.t1

ATGGGTTTCTTCAGTTTTAACAACTCTTCTTCAAGATACGTTGGAATTTGGTATTACAAC

GTTACTTCAGCTTATGTTTGGGTTGCAAACAGAGAAAAACCAATTAAAAACAGAGAAGGT

TCTGTTACAATAAAAAATGATGGTAACTTAGTTGTTCTTGATGGACAAAACAACGAGGTT

TGGTCAAGTAACGTATCAAAAATATCGATAAACAATTCGCAAGCTGTTCTTCATAATGAT

GGGAACCTTATTCTTTCAGATAGAGAAAATAACAAAGAGATTTGGCAAAGTTTTGAAGAT

CCAACTGATACATATCTTCCAGGTATGAAAGTTCCGGCGAGTGGTGAAAGAGGAATTGGG

AAGGATGCTACTTTTTGTTCATGGAAATCGGAGAACAATCCTTCATTAGGAAACTATACA

ATGAGTGTGGATTCTGAAGCATCGCCACAGATAGTGATTATGGAAGGAGAGAACAGAAGG

TGGAGAAGTGGTTATTGGGATGGTAGAGTTTTCACAGGTGTACCAAATATGACAGGAAGT

TATCTTTTTGGTTTTAGGCTTAACACAAATGATAATGGAGAAAGGTATTTTGTATATGAA

GCATTGGAAAATAGTGATGAGGTAAGGTTTCAGCTTGGATATGATGGATATGAGAGACAA

TTCAGGTGGAATGAAGAAGAGAAAGAATGGAATGTGATACTAAGTGAGCCTAATAAGAAG

TGTGAGTTTTATAATTCATGTGGTAGTTTTGCAATATGTGATATGTCTGATTCAAGTTTA

TGCAAATGCATAAAAGGGTTTGAACCAAAGGATGTGAAAAGTTGGAATAGTGGGAATTGG

TCAAAAGGGTGTAAGAGGATGACTCCATTGAAGGGTGAGAGAAGTGGTAATAGTTCTGGT

GGAGAAGATGGTTTTTTGGTGCAAAGGAGTTTGAAGTTGCCTGATTTTGCACGTTTGGTG

AGTGCTGTTGATAGTAAGGATTGTGAGGGAAATTGTCTGAAGAATAGTTCTTGTACTGCT

TATGTAAATGCTATTGGAATTGGGTGTATGGTTTGGCATGGGGAGTTAGTCGATGTTCAA

AGGTTAGAAAATCAAGGAAATACACTCAACATTCGTCTTGCTGATTCTGATTTAGGTGAT

GGGAAGAAAAAGACCAAAATTGGGATAATATTAGGTGTTGTGGCGGGGCTTATCTGCCTT

GGAATCTTTGTATGGCTTCTATGCAGGTTTAAGGGAAAACTCAAAGTTTCCTCAACTTCT

TCTACTTCCAACATTAATGGTGATCTACCAGTTTCTGAACAAATCAAGAGTGGAAACTTA

TCAGCAGGATTTTCAGGATCAATCGATCTTCACTTAGACGGGAGCCCTCTAAATAATGCA

GAACTTTCATTCTTCAATTTTAGTAGCATTGTAATTGCAACAAACAATTTCTCAGAAGAA

AACAAGCTTGGACAAGGGGGATTTGGTCCTGTCTACAAGGGAAGGCTTCCAGGGGGAGAA

CAAATTGCGGTGAAGAGGCTTTCTAGACAGTCGAACCAAGGTTTAGATGAGTTCAAGAAT

GAAATGATGCTAATAGCTAAATTACAGCATAGAAATCTTGTTAGACTATTAGGATGTTCA

ATTGAAGGGGAAGAAAAGTTACTTGTATATGAATACATGCCAAACAAAAGTTTGGACCGC

TTCTTATTTGATCCAGTCAAGAAAACAAAACTAGACAGCTCAAGACGGTATGAAATCATC

GAGGGCATTGCAAGAGGACTACTTTATCTGCACCGAGATTCACGTCTTAGAATAATACAT

CGAGATCTAAAAGCAAGCAACATTTTGTTGGATGAAAATATGAATCCAAAAATTTCAGAC

TTTGGCTTGGCAAAGATATTTGGGGGAAATCAAAATGAAGGGAATACAGAGAGAGTAGTT

GGAACATATGGATATATGTCTCCAGAATACGCAATGGAAGGTCTATTTTCAGTCAAATCT

GATGTCTATAGTTTTGGCGTACTACTACTAGAGATTGTGAGTGGTCGCAAAAACACTAGC

TTTCGTGATTCGTATGACCCAAGTCTCATAGGATATGCGTGGCGTCTATGGAATGAAGAG

AGAATAATGGAGCTTGTTGATCCGTCCATAAGAGATTCCACTAAAAAGGATAAAGCTTTG

AGATGCATACACATAGGGATGTTATGTGTGCAAGATTCAGCATCTCATAGACCAAACATG

TCCTCAGTGGTGTTGATGCTTGAGAGTGAAGCAACAACTCTTCCCTTGCCTGTCAAACCT

TTGCTTACTTCCATGAGGAGATATGATGATACAGATGAATTTAATACAGAACCCCTTGAT

GCATCAATTGATCTGACAGTCACAGGGAGATAG

>MS.gene052582.t1

ATGTCTGAAAAAGGTTGTCAGCCTAATGAGTTCACTGTCGGGATACTTGTTCGTGGTTTT

TGTCGAGCTGGGAGAACTAAACAGGCGATGGAGTTTGTTGATGGGAAGATGGGGGGGAAT

GTTAATAGGGTTGTGTATAATACTCTTGTGTCGAGTTTTTGTAAACAGGATATGAATGAC

GAGGCGGAGAAATTGGTGGAGAGGATGAGGGAGAAGGGTTTGTTACCTGATGTTGTTACG

TTTAATTCTAGGATATCTGCACTTTGTAGCGCGGGGAAGGTTTTTGAGGCGTCGAGGATT

TTTAGGGATATGCAGATGGATGGGGAATTGGGACTACCTAAGCCGAATGTTGTTACTTTT

AATTTGATGCTTAAGGGGTTTTGTCAGGAAGGGATGATGGAGGAAGCTAGGTCTTTGGTT

GAGACTATGAAGAAAGGTGGGAATTTTGTTAGTTTGGAGAGTTATAATACGTGGTTGTTG

GGTTTGCTTAGAAATGGGAAGTTGTTGGAGGGTCGATTGGTTCTTGATGAAATGGTGGAG

AATGGTATTGAACCAAATATTTACTCATATAATATTGTGATGGATGGGCTGTGTAGAAAC

CATATGATGTTGGATGCGAGAAGGTTGATGGATTTGATGGTGAGTAATGGTGTTTACCCA

GATACTGTTACTTATACTACTCTACTACATGGCTATTGTAGCAAAGGGAAGGTTTTTGAA

GCTAAAGCTGTTCTTAATGAAATGATTAGGAAAGGTTGTCATCCAAATACTTATACTTGC

AATACGTTGCTGAATAGCCTGTGGAAAGAGGGGAGGAAGTCAGAGGCTGAGGAAATGCTG

CAAAAGATGAACGAAAAGAGTTATCAGTTAGATACCGTGACCTGTAATATCGTGGTCAAT

GGTCTGTGTAGAAATGGAGAATTGGAGAAAGCAAGTGAAGTTGTAAGTGAGATGTGGACT

GATGGAACAAATTCCCTTGGTAAAGAAAACCCGGTTGCTGGCCTAGTTAATTCAATCCAT

AATGTATCGACCAATGTACCTGATGTGATCACTTATACAACCTTAATTAATGGACTTTGC

AAGGTTGGGAAACTAGAGGAAGCCAAAAAGAAATTTATTGAGATGATGGCGAAAAACTTA

CACCCTGATTCTGTGACCTATGATACATTTGTATTGAATTTCTGCAAACAAGGGAAGATA

TCATCGGCTTTACGTGTATTGAAAGACATGGAGAGAAATGGTTGCAGCAAGACTCTTCAA

ACTTATAATTCATTGATCTTGGGGTTAGGAAGTAAAGGGCAAATATTTGAAATGTACGGA

TTGATGGATGAGATGAGAGAAAGAGGAATACATCCAGATATTTGCACTTACAATAATATG

ATCAATTGTCTTTGCGAAGGAGGAAAAACAAAGGATGCCACTTCTCTTTTACATGAAATG

TTGGATAAGGGAGTCGTCTCTCCCAATGTATCATCCTTCAAAATATTGATTAAAGCATTC

TGCAAGTCTGAAGATTTTAAAGTAGCATGTGAACTTTTTGATGTAGCTCTGAGTGTATGT

GGCCACAAAGAAGCCTTGTACAGTTTGATGTTCAACGAATTACTTGCTGGGGGAAAACTC

TCTGATGCAAAAGAGCTATTTGAAGCTTCGTTAGAAAGATCTCTCTTATCAAAGAACTTT

ATGTATGAAGATTTGATTGACAAACTTTGCAAGGATGGAAGGTTAGACGATGCTCACGGC

CTTCTTCAGAAATTGATTGATAAGGGATATTGCTTCGACCATTCATCATTCATACCTGTG

ATTGATGGCTTAAGTAAAAGGGGAAACAAACAGCAAGCTGATGAACTAGCAAGGATAATG

GAATTGGCTTTAGAAGAAGATAAAACTTTTGATAGGACGTACCGAAATGGAAACTCTATC

TTTCGTCGAAAATTACACAAGGATGGTGGAAGTGATTGGCAGGATATAATTAACAGGTAT

GACCATATGGTAGTGAAGATAGTCTTTTCATTCCACTTTTGGGTTCTGTTCTTTTATAAA

AGAGATGTTTTTAACTAG

>MS.gene052580.t1

ATGCAAATATTCGTTAAGACCCTCACTGGCAAGACCATCACTCTCGAAGTTGAGAGTTCA

GATACCATAGACAATGTTAAGGCAAAGATTCAAGACAAGGAAGGAATCCCACCAGACCAG

CAGCGTCTGATTTTTGCTGGAAAGCAGCTGGAGGATGGGCGTACCCTTGCTGATTATAAC

ATCCAAAAGGAGTCCACTCTCCATTTGGTGCTTCGTCTCCGTGGTGGTATGCAAATATTT

GTGAAGACTCTCACTGGAAAGACAATCACCCTTGAGGTGGAGAGCTCTGATACCATTGAC

AATGTTAAGGCCAAGATTCAAGACAAAGAAGGTATTCCACCAGACCAGCAGAGGCTCATC

TTTGCTGGAAAACAGCTTGAAGATGGCAGAACCTTGGCTGATTACAACATCCAGAAGGAG

TCAACTCTCCATCTTGTGTTGCGGCTTCGTGGTGGTATGCAGATTTTTGTAAAGACCCTT

ACAGGAAAGACAATTACTTTGGAGGTAGAGAGCTCGGACACAATTGACAATGTGAAGGCC

AAGATACAAGACAAGGAGGGTATCCCCCCAGACCAGCAAAGGTTGATTTTTGCTGGAAAG

CAACTCGAAGATGGTCGAACTCTGGCTGATTATAACATCCAGAAAGAGTCGACTCTTCAT

CTTGTCTTGCGACTTCGTGGTGGTATGCAGATTTTTGTTAAGACCCTAACAGGCAAAACA

ATTACTTTGGAGGTGGAGAGCTCAGACACAATTGACAATGTAAAGGCTAAGATTCAGGAC

AAGGAGGGCATTCCCCCAGACCAGCAAAGGTTGATTTTTGCTGGAAAGCAGCTTGAAGAT

GGCAGAACCTTGGCAGATTATAACATCCAGAAGGAATCCACCCTTCATCTTGTCCTTCGT

CTGAGGGGAGGCATGCAGATCTTTGTGAAGACTTTGACAGGAAAGACCATTACATTGGAG

GTGGAAAGCTCTGACACCATCGATAATGTTAAGGCAAAAATACAGGATAAGGAGGGAATC

CCACCAGATCAGCAGAGGTTGATCTTTGCTGGGAAGCAGCTTGAAGACGGTCGGACTTTG

GCTGATTATAATATTCAGAAAGAGTCCACCCTTCACCTTGTCCTTCGTCTGAGGGGAGGT

ATGCAAATCTTTGTGAAGACGTTGACCGGAAAGACCATCACCTTAGAGGTGGAAAGTTCA

GACACTATTGATAATGTCAAGGCAAAAATCCAGGACAAGGAGGGAATCCCCCCAGATCAG

CAGAGGTTGATCTTTGCTGGGAAGCAATTGGAGGATGGAAGGACCCTTGCTGATTACAAC

ATTCAGAAAGAGTCTACTCTTCACCTTGTTTTGCGTCTTCGTGGTGGATTTTAA

>MS.gene052581.t1

ATGTCGCTGATTCCAAGTTTCTTTGGTGGTCGAAGGAGCAATGTTTTCGATCCATTCTCC

CTTGACGTTTGGGATCCCTTCAAGGATTTTCCATTCTCCAATTCTGCACTTTCTGCTTCT

TCATTCCCTCAGGAGAATTCTGCGTTTGTGAGCACCAGGGTGGATTGGAAGGAGACCCCA

GAAGCTCATGTGTTCAAGGCTGATCTTCCAGGAATGAAGAAGGAAGAAGTGAAGGTTGAA

ATTGAAGATGACAGAGTTCTTCAGATTAGTGGAGAGAGGAACGTTGAGAAAGAAGACAAG

AACGATCAATGGCATCGCGTGGAGCGTAGCAGTGGGAAATTTATGAGGAGGTTTAGATTG

CCTGAGAATGCTAAAATGGATCAAGTGAAAGCTGCAATGGAGAATGGTGTTCTCACTGTT

ACTGTTCCAAAAGAAGAAGTTAAGAAACATGATGTTAAGTCTATTGAGATCTCTGGTTGA

>MS.gene052583.t1

AGATGGATCATCGATGATGCAACAGGACAAAGAATGAAACTAACCTGTGCACATTGGGTT

GCACATGCAACCCCAATGCTTGTTGAAGGTCTTGACAAATTGCCTATACAAGACATAGCT

AATCAAATTGCTAAGTCAGGTTTCAATTGTGTCCGTTTGTCTTATGCTACTTACATGTTC

ACACGACATGCAAATGATACAATTCGAGATACACTTTATAGTCTCGATATACCCAAGGAT

GTTGTGTCGGCTATTGAGAAACATAACCCTTTAATGTTGAACATGACTCATGTTCAAGCT

TATGAAGCTGCCATTGATGCTCTTGGTGAAAAAGGTGTTATGGTTCTTATTGATAATCAT

GTTAGTATGCCAGATTGGTGTTGTGATAATAATGATCAGAATGGATTCTTTGGTGATAGG

CATTTTCATCCTGATGAATGGCTTCAAGGTTTGGCTATTATAGCTAAGCACTTCAAGGGA

AAACCCAATGTGATAGCCATGGACTTGCGGAATGAGCTAAGAGGTGGACGTCAAAATTTG

CCCGATTGGTACAAGTATGTAACCCAAGGAGCAAGCACAATTCACAAACACAACCCAGAT

TTATTAATAGTTATTTCAGGGTTTAACTTTGACAATGACCTTTCATTTTTGAAGAAAAAG

ACCCTTGATCTAAACTTCACCAACAAATTAGTGTACGAGGCACACATTTATTCCTTCTCA

GGAAACCAAGATAGGTGGAAGTTGCAACCAATGAATTGGGTTTGTTCCTCTGTCATTGAA

ACCTTAAACGACCAAGCTGGTTTTCTTATTAGTGGTAACAATCCAGTACCTTTATTCATA

AGTGAATTTGGATATGACATGACTGGTGGCAATGCCGTGGACAACAAATTCATGCCATGC

TTTGTGTCTTATGCCGTCTCTAATGACTTGGATTGGAGCTTGTGGTCTTTTGGTGGAAGC

TACTATTTTAGACAAGGCAATGTTGGGGCTGGTGAGACATATGCTGTAATGGACTATGAT

TGGAAAAATTACAGGGACCCAAATTTCCCTCAGAAATTTCAGCTTCTCCAGAAGAAAATT

CAAGATCCAACTTCAAACCTCTCAAAGTCTCATATAATGTTCCACCCATTGACCGGTAAG

TGCGCTCACGTGAATGAGAGTAACAATGAACTTGTATTGGGAGATTGTAAGAGTCATAGC

CAATGGAGTTCTGAGGGAGATGGTTCTCCAATAAGGTTGATGGACTCTGCTAAGTGTCTA

AAGGCAGAGGGTGAAGGGCTTCCTGCAACCCTCTCAGAACATTGCTTGTTGCCGCAAAGT

TCCTGGAAATCTGTTTCAAAGACTGGTCTTCACTTGGCCACCTCTCATGGGAACGGATCC

CTTTTGTGCTTGGAAATGGATTCAGATTCCTCCAAGATAGTGACCAGAAAATGCATCTGC

ATAGATGATAATGATTCTTCATGTTTGGACAACCCCCAAAGCCAGTGGTTCAAACTTATT

TCAACCAATGTTTAG

>MS.gene052584.t1

ATGATTCAAACCGGTGTTAACCCAGAAACATATACTTTTAATCTTTTAATCCAGTCGTTA

TGTGAATCAAACGCGCTTGATCATGCACGTGAACTGTTTGATAAAATGTCTGAAAAAGGT

TGTCAGCCTAATGAGTTCACTGTCGGGATACTTGTTCGTGGTTTTTGTCGAGCTGGGAGA

ACTAAACAGGCGATGGAGTTTGTTGATGGGAAGATGGGGGGGAATGTTAATAGGGTTGTG

TATAATACTCTTGTGTCGAGTTTTTGTAAACAGGATATGAATGACGAGGCGGAGAAATTG

GTGGAGAGGATGAGGGAGAAGGGTTTGTTACCTGATGTTGTTACGTTTAATTCTAGGATA

TCTGCACTTTGTAGCGCGGGGAAGGTTTTTGAGGCGTCGAGGATTTTTAGGGATATGCAG

ATGGATGGGGAATTGGGACTACCTAAGCCGAATGTTGTTACTTTTAATTTGATGCTTAAG

GGGTTTTGTCAGGAAGGGATGATGGAGGAAGCTAGGTCTTTGGTTGAGACTATGAAGAAA

GGGAAGGTTTTTGAAGCTAAAGCTGTTCTTAATGAAATGATTAGGAAAGGTTGTCATCCA

AATACTTATACTTGCAATACGTTGCTGAATAGCCTGTGGAAAGAGGGGAGGAAGTCAGAG

GCTGAGGAAATGCTGCAAAAGATGAACGAAAAGAGTTATCAGTTAGATACCGTGACCTGT

AATATCGTGGTCAATGGTCTGTGTAGAAATGGAGAATTGGAGAAAGCAAGTGAAGTTGTA

AGTGAGATGTGGACTGATGGAACAAATTCCCTTGGTAAAGAAAACCCGGTTGCTGGCCTA

GTTAATTCAATCCATAATGTATCGACCAATGTACCTGATGTGATCACTTATACAACCTTA

ATTAATGGACTTTGCAAGGTTGGGAAACTAGAGGAAGCCAAAAAGAAATTTATTGAGATG

ATGGCGAAAAACTTACACCCTGATTCTGTGACCTATGATACATTTGTATTGAATTTCTGC

AAACAAGGGAAGATATCATCGGCTTTACGTGTATTGAAAGACATGGAGAGAAATGGTTGC

AGCAAGACTCTTCAAACTTATAATTCATTGATCTTGGGGTTAGGAAGTAAAGGGCAAATA

TTTGAAATGTACGGATTGATGGATGAGATGAGAGAAAGAGGAATACATCCAGATATTTGC

ACTTACAATAATATGATCAATTGTCTTTGCGAAGGAGGAAAAACAAAGGATGCCACTTCT

CTTTTACATGAAATGTTGGATAAGGGAGTCGTCTCTCCCAATGTATCATCCTTCAAAATA

TTGATTAAAGCATTCTGCAAGTCTGAAGATTTTAAAGTAGCATGTGAACTTTTTGATGTA

GCTCTGAGTGTATGTGGCCACAAAGAAGCCTTGTACAGTTTGATGTTCAACGAATTACTT

GCTGGGGGAAAACTCTCTGATGCAAAAGAGCTATTTGAAGCTTCGTTAGAAAGATCTCTC

TTATCAAAGAACTTTATGTATGAAGATTTGATTGACAAACTTTGCAAGGATGGAAGGTTA

GACGATGCTCACGGCCTTCTTCAGAAATTGATTGATAAGGGATATTGCTTCGACCATTCA

TCATTCATACCTGTGATTGATGGCTTAAGTAAAAGGGGAAACAAACAGCAAGCTGATGAA

CTAGCAAGGATAATGGAATTGGCTTTAGAAGAAGATAAAACTTTTGATAGGACGTACCGA

AATGGAAACTCTATCTTTCGTCGAAAATTACACAAGGATGGTGGAAGTGATTGGCAGGAT

ATAATTAACAGGGATGTTGGCAGTAATATCGCATTGAAAACTCTGAAGCGTGTAGAAAAA

GGTTGGGGTCAAGGAAGTATATCGAGTTTGCTGCCTCAAAAGAATGATTTTCTTGATTAC

TAA

>MS.gene052587.t1

ATGCTAAACAGCTCCAAAACTGTCATATGGGAAAGTTTTGATCATCCCACAGATACCTTG

CTTCCATATCTAAAGGTTGGTTTTGATAGGAAAACTAATCAAAGCTGGTTTCTCCAATCA

TGGAAGACAGATGATGACCCTGGAAAAGGCGCATTCACGTTGAAATTCAGCACCATTGGC

AAATCTCAGTTGTTTATGTACAACCATGATCTTCCTTGGTGGCGTGGTGGACATTGGAAT

GGAGAATTATTAGTAGGCATACCTAATATGAAACGAGATATGGCCACTTTCAATGTTTCT

TTGGTTGAAGATGACAACTATGTAGCGCTTAGGTATAACATGTTCGATAAGTCTGTCATT

ACAAGAATAGCGGTTCAGCAATCTGGTTTCTTTCAAACATTCATGTGGGACAGTCAAAAG

AGTCAATGGAACCGATACTGGTCTGAACCAACAGACCAATGTGATAACTACGGAACCTGT

GGATCAAACAGTATTTGTGACCCTTTTAACTTTGACGACTTTAAGTGTACTTGTTTACTT

GGTTTTGAACCAAAATTTCGACATGATTGGTATGAGAGAAGAGATGGGTCGGGAGGGTGT

GTAAGGAAGAAAGGTGTATCTGTTTGTGGGAATGGAGAAGGGTTTGTCAAAGTTGTCAGC

TTGAAAGTTCCCGATACATCAGTGGCAGTTGCTAAAGGTGATTTGAGTTTGGAAGAATGT

GAGAAAGAATGCTTGAGAAACTGCTCTTGTACCGCCTATGCCGTTGCTGATGTGAGGAAT

GGTGGAAGTGGTTGTTTGGCATGGCATGGGGATTTAATGGACGTTCAGAAACTTAGCGAT

CAAGGCCAAGATTTATTTTTACGTGTCAATGCAGTTGAACTAGCCAAAGCCAATACTCAC

AAAAAAAGCAAAGGAATCCTTGGTAAAAAGAGGTTAGCTGCGGTTTTGGTAGTTTCTATA

GTTGCAATTGTCCTCCTCCTTTCATGTATGTATTGCATGTGGGAGAAAAAAAGAAAGGAT

AAAACGCTGCGGCAATCATACCAAGATTCCTCTGGAGAGATTGGTGCTCAAAGCAACACA

CATCCAAATCTACCATTTTTTAGCTTTAAAACGATAATAACAGCTACAACAAATTTTAGT

CATGAGAATAAGCTCGGACAAGGTGGATTTGGCTCTGTCTATAAGGGTTGCTTGGTTAGT

GGACAAGAGATAGCAGTGAAAAGACTCTCCAGAGATTCAGGTCAAGGAAAAAAAGAGTTT

GAAAATGAAGTTACTCTTTTAGTTAAACTCCAACACAGAAATCTAGTGCGGTTGCTTGGT

TGTTGTTTTGAAAAAGAAGAAAGGATGCTAGTGTATGAATACTTACCAAACAAAAGCCTA

GACTTCTTTATATTCAATCAAAACCGAAGGCCATTATTGGATTGGGTTAAGCGTTTTGAA

ATCATTTGTGGGATTGCTAGAGGTGTTTTATATCTTCATCAAGATTCAAGGCTGAAAATA

ATCCATAGAGATTTAAAAGCCAGCAACGTTCTCCTTGATGCTGCAATGAATCCCAAAATC

TCAGATTTTGGTATGGCTAGAATATTTGGAGAAGATGAAATCCAAGCAAGAACAAGAAGA

GTGGTCGGAACATACGGATATATGTCACCAGAATATGCAATGGAAGGACGATATTCAACA

AAATCTGATGTCTTCAGTTATGGTGTCTTACTACTGGAGATTATTGTTGGCCAAAGAAAC

ACACATTGTGAAACAGGAAGAGAATCACCAAATTTAATTGGACATGTGTGGAAACTATGG

ACAGAAGGAAGAGGCTTGGATATTGTTGATCCAGCACTAAACCAGTCTTATCCCCCTGCT

ATAGTTCTGAGATGCATTCAAATTGGACTTTTGTGTGTGCAAGAAAATGCAATTAATAGA

CCATCAATGTTAGATGTTGTTTTCATGCTAGCCAATGAAACACCTCTTTGCCAACCAAAA

AAACCAGCATTTTTATTCAATGGAAACCATGACTTACATGAGTCATCAACTTCAGGAGGA

GGATCTTCAATAAATGAATTAACCGAGACTACTATTAGTGCTCGCTGA

>MS.gene052588.t1

ATGGACTCTGCTAAAAATCATCTCTACTTTGTCACAGCCAAAAATCATAAACACAACTTA

ATATTACATAATAGTTGGTTCCTTATAAACTCTTTGATTCTACTCCTTCTCACCTTCTCC

TTTTGCTCTTGTTCTTCTGATGTCATATCTATTGACAAACCAATAAGAGATGGTGATCTT

CTTGTTTCCAAATCCAAAACTTTCGCTCTTGGATTCTTCACTCCAGCAAAATCCACATCT

CGCTATGTTGGTATTTGGTACAACAATTTGCCAATCCAAACTGTTGTTTGGGTTGCAAAT

AGAAATAGTCCAATCAACGATACTTCTGGAATTCTATCAATCAATCCAAATGGAAATCTA

GTACTCAACCACAACCGTAGCACCATTCCCATTTGGTCTACCGATGTTTCATTACCACAA

TCACAAAGAAATAGCACCAGGGTTATAGCTCAACTATCGGATGTAGCAAACCTTGTTCTG

ATGATAAACAACACCAAAACTGTATTATGGGAAAGCTTTGATCATCCAACAGACACCTTG

CTTCCATATCTAAAGATTGGGTTTAATAGAAAAACTAATCAAAGCTGGTTCCTTCAATCC

TGGAAGACAGATGATGACCCTGGAAATGGTGCATTCACAGTGAAATTCAACAGCATTGTC

AAACCTCAGTTGTTTATGTACAACCATGATTTTCCATGGTGGCGTGGTGGACATTGGAAC

GGAGCAATATTAGTAGGTGCACCTAATATGAAACGAGATATGGCAATTTTGAATGTTTCT

TTTGTGGAAGATGATGATAACTATGTAGCCATCTCATATAACATGTTCGATAAGTCTGTC

ATAGCAAGGATAGTTGTTCAGCAATCTGGTTTCTTTCAAATATTCACTTGGAACAATCAA

AAGAGTCAATGGAACAGGTTCTGGTCTGAACCAACAAACCAATGTGATAACTATGGAACC

TGTGGATCAAACAGTAATTGTGACCCTTTGAACTTTGAGGACTTTAAGTGTACTTGTTTA

CCAGGTTTTGAACCAAAATTTCCACGTGATTGGTATGAGAGAAGAGATGGGTCAGGAGGG

TGTGTAAGGAAGAAAGGCACATCTATTTGTCGGAATGGAGAAGGGTTTGTCAAAGTTGCA

AGCTTGAAAGTTCCTGATATATCTGTGGCAGTTGCTAAAGGTGGTTTGAGTTTGGAAGAA

TGTGAGAAAGAATGCTTGAGAAACTGCTCTTGTGCTTCCTATGCGGTTGCTGATGTGAGT

AATGGTGGAAGTGGCTGTTTAGAATGGTATGGGGATTTAATGGACATTCAAAAACTTAAT

GATCAAGGCCAAGATTTATTTGTACGTGTCGATGCAGTTGAACTAGCCAAAGCCAATAAC

CACAAAAGAAGCGTAGGAGTCCTAGGTGAAAAGAGGTTTTCTGCAATTCTGGTTGCTTCT

ACAGTTGCAATTGTCCTCCTCTTCTCCTATGTGTTTTGCAGGTGGAAGAAAAAAACAAGA

AAGGATAAAATGATGCGGCAATTTAACCAAGATTCCTCTGGCGAAGAGAATGGTGAGCAA

AGCAACACACATCCAAATCTGCCCTTTTTCAGCTTTAAAACGATTATAACAGCTACAAGT

GATTTTAGTCATCAGAATAAGCTTGGACAAGGTGGATTTGGCTCTGTCTATAAGGGTTGC

TTGGTTAATGGACAAGAAATAGCAGTGAAAAGATTGTCCAGATTTTCAGGTCAAGGAAAA

GAAGAGTTTAAAAATGAAGTTAAACTTTTAGTCAAACTTCAACACAGAAATCTAGTGAGG

TTGCTAGGTTGTTGCTTTGAAAAGGAAGAAAGGATGCTAGTTTATGAATACTTACCAAAC

AAAAGCCTAGATTTCTTTATATTTGATCAAAACCAAAGGTCATCATTGGATTGGGGCAAG

CGTTTTGAAATTATTTGTGGGATTGCTCGAGGTGTTTTATATCTTCATCAAGATTCAAGG

CCGAAAATAATTCATAGAGATCTAAAACCCAGCAATGTTCTCCTTGACGCTGCAATGAAT

CCCAAAATCTCAGATTTTGGTATGGCTAGAATATTTGGAGAAGATGAAATCCAAGCAAGA

ACAAAAAGAGTGGTTGGAACATATGGATACATGTCACCAGAATATGCAATGGAAGGACGA

TATTCAACAAAATCTGATGTCTTCAGTTATGGTGTCATACTACTGGAGATTATTGCTGGC

CAAAGAAACACACATTGTGTAACAGGAAGAGAATCCCCAAATTTAATTGGACATGTGTGG

ACACTATGGACAGAAGGAAGAGCCTTGGATATAGTTGATCCAGAACTAAACCAGTTTTAT

CCTCCGACTACAGTTATGAGATGCATTCAAATTGGACTTCTATGTGTGCAAGAGAATGCC

ACAAATAGACCATCGATGTTAGAAGTTGTTTTCATGCTAGCTAATGAAACACCTCTTTGC

CCACCTCAAAAACCAGCATTTTTATTCAATGGCAACCAAGATTTGCAAGAATCATCAACA

TCAGGAGGAGGATCTTCAATAAATGAATTAACAGAAACTACCATCAGTGCTCGCTAA

>MS.gene052585.t1

ATGCTTGTTGAAGGTCTTGACAAATTGCCTATACAAGACATAGCTAATCAAATTGCTAAG

TCAGGTTTCAATTGTGTCCGTTTGTCTTATGCTACTTACATGTTCACACGACATGCAAAT

GATACAATTCGAGATACACTTTATAGTCTCGATATACCCAAGGATGTTGTGTCGGCTATT

GAGAAACATAACCCTTTAATGTTGAACATGACTCATGTTCAAGCTTATGAAGCTGCCATT

GATGCTCTTGGTGAAAAAGGTGTTATGGTTCTTATTGATAATCATGTTAGTATGCCAGAT

TGGTGTTGTGATAATAATGATCAGAATGGATTCTTTGGTGATAGGCATTTTCATCCTGAT

GAATGGCTTCAAGGTTTGGCTATTATAGCTAAGCACTTCAAGGGAAAACCCAATGTGATA

GCCATGGACTTGCGGAATGAGCTAAGAGGTGGACGTCAAAATTTGCCCGATTGGTACAAG

TATGTAACCCAAGGAGCAAGCACAATTCACAAACACAACCCAGATTTATTAATAGTTATT

TCAGGGTTTAACTTTGACAATGACCTTTCATTTTTGAAGAAAAAGACCCTTGATCTAAAC

TTCACCAACAAATTAGTGTACGAGGCACACATTTATTCCTTCTCAGGAAACCAAGATAGG

TGGAAGTTGCAACCAATGAATTGGGTTTGTTCCTCTGTCATTGAAACCTTAAACGACCAA

GCTGGTTTTCTTATTAGTGGTAACAATCCAGTACCTTTATTCATAAGTGAATTTGGATAT

GACATGACTGGTGGCAATGCCGTGGACAACAAATTCATGCCATGCTTTGTGTCTTATGCC

GTCTCTAATGACTTGGATTGGAGCTTGTGGTCTTTTGGTGGAAGCTACTATTTTAGACAA

GGCAATGTTGGGGCTGGTGAGACATATGCTGTAATGGACTATGATTGGAAAAATTACAGG

GACCCAAATTTCCCTCAGAAATTTCAGCTTCTCCAGAAGAAAATTCAAGATCCAACTTCA

AACCTCTCAAAGTCTCATATAATGTTCCACCCATTGACCGGTAAGTGCGCTCACGTGAAT

GAGAGTAACAATGAACTTGTATTGGGAGATTGTAAGAGTCATAGCCAATGGAGTTCTGAG

GGAGATGGTTCTCCAATAAGGTTGATGGACTCTGCTAAGTGTCTAAAGGCAGAGGGTGAA

GGGCTTCCTGCAACCCTCTCAGAACATTGCTTGTTGCCGCAAAGTTCCTGGAAATCTGTT

TCAAAGACTGGTCTTCACTTGGCCACCTCTCATGGGAACGGATCCCTTTTGTGCTTGGAA

ATGGATTCAGATTCCTCCAAGATAGTGACCAGAAAATGCATCTGCATAGATGATAATGAT

TCTTCATGTTTGGACAACCCCCAAAGCCAGTGGTTCAAACTTATTTCAACCAATGTTTAG

>MS.gene052586.t1

ATGGATTATCATGGTGTTCCAGAAATTTTTCTTTGGAACAAAAATAGAAGAGTTTATAGA

AGTGGACCATGGAATGGTAAAAGATTCAGTGGTGTACCAGAGATGCAACCAGTTACAGAT

TCAATTCAATTCAGTTTTGTTCAGAATGAACATGAAGTTTACTATTCGTTTTCAATTGGG

AAAGAATTATTGTATTCAAGACTCAGTGTGAATTCATTAGGTGAACTTCAAAGGCTAACA

TGGATAAATAGTAGAAATATTTGGACAAAATTTTGGTATGCACCAAAAGACCAATGTGAT

AATTACAAAGAGTGTGGTCCATTTGGTGTATGCGACACAAATGCTTCACCTGTTTGTAAT

TGTATTAAAGGGTTTCGACCGAAGAATCATCAAGCATGGAATTTGAGAGATGGATCTGAT

GGATGTTTGAGGAATAATGAGTTGGATTGTGAGAGTGATAAGTTTTTACATATGGTGAAT

GTGAAGTTGCCAGAGACAAGTAGTGTTTTTGTGAATAGGAGTATGAGTTTGGTTGAATGT

GGTAATTTGTGTCAAAGGAATTGTTCTTGTACTGGTTATGCAAATATTGAGATTGTTGAT

GGAGGAATTGGTTGTGTTATGTGGCTTGATGAACTTATTGATATTAGAGTTTATCCTGCT

GGTGGTCAAGATCTCTTTGTCAGATTAGCAGCTTCTGATGTAGGTGATGATGGGGTAGGA

GGAAGTTCTAATCACAAGATTGCTAAGGCTATAGGCATTATGGTTGGTGGTGCAACTATT

GTATTTTTGGTATTAGGAACATGTTACTTGTGGAGGAAGAAAAAATTGCAGTGTCTATTG

AAAGGGAAGAGTGATAAAAGAGGTTCATTGGAAAGAAGTCAAGATTTGCTTATGACTGAA

GGGGTGTATACAAGTAATAGAGAACAGACAAGTGAAAAGAACATGGATGACCTAGAATTG

CCATTCTTTGATTTTAATACCATAACAATGGCTACTAACAATTTCTCTGAAGAAAATAAA

CTTGGACAAGGAGGTTTTGGTATTGTTTATAAAGGTAGGTTGATTGAAGGTCAGGAGATT

GCAGTGAAAAGATTATCAAAAAATTCCGGCCAAGGCGTTGAAGAATTCAAGAATGAGGTC

AGGTTGATTGTGAAGCTTCAACATCGAAATCTTGTTCGGTTACTTGGTTGCAGCTTTCAA

ATGGATGAGAAGATGCTAGTGTATGAGTACATGGAAAATAGAAGCCTTGATGCCATTTTA

TTTGACAAAGCTAAAAGATCTTTGCTAGATTGGCAAACACGCTTCAACATTATAAGCGGA

ATAGCTAGAGGACTTCTTTATCTTCACCAAGATTCTAGATTTAGGATCATCCATAGAGAT

CTTAAGGCAAGCAACATTCTACTTGATGGAGAGATGAATCCAAAGATATCGGACTTCGGG

ATGGCAAGAATTTTTGGCACGGATCAGACAGAGGCAAATACAGTTAGAGTTGTTGGAACA

TATGGTTATATGTCTCCAGAGTATGCTATGGATGGAATCTTCTCAGTGAAATCTGATGTT

TTTAGTTTTGGTGTTCTCGTGATGGAAATCATAAGTGGAAAAAAGAATCGAGGTTTCTAT

TCCGCAAACAAAGAATTAAATCTTCTTGGTCATAGTTGGAAGTTGTGGAATGAAGGAAAT

GCTTTGGAACTGATTGACTCTTCCATTGCTAATTCATATTCACCATCTGAGGTTTTTAGA

TGCATACAAGTTGGACTATTATGTGTACAAGAGCGAGCGGAGGATAGGCCAACAATGTCT

TCAGTGGTGTTGATGTTGAGTAGTGAAACAGCAACAATAGCACAGCCTAAAAACCCTGGA

TTTTGCCTAGGAAGCAATCCTGTGGAAACTGATTCATCCTCAAGTAAACAAGATGAATCT

TGTACTGTGAACCAAGTTACTGTCACAATGGTAGATGGTAGATAG

>MS.gene052589.t1

ATGCATAATAATTTACTTCTTAACTCTTTGATTCTACTACTTCTCACATTCTCTTTTTGC

TCTTGTTCTTCTGATACCATATCTATTCACAAAACCTTAAGAGATGGTGAGCTTCTCATT

TCTAAATCAAAAACATTTGCTCTTGGATTCTTCACTCCAGGAAAGTCCACCTCTCGCTAT

GTTGGTATTTGGTACTACAATTTGCCGATCCAAACTGTTGTTTGGGTTGCAAATAGAGAT

GCTCCCATAAACGACACTTCTGGGATTCTATCCATCGACACAGATGGGAACCTAGTACTC

AACCATAAACTTAGCACCATTCCCATATGGTCTACTTACATTTCATTAACACAATCACAA

AGAAAGAGCACCAGTGCTGTTATAGCAAAATTATCAGACAAAGCAAACATTGTTCTGATG

ATAAACAACACAAAAACTGTCATCTGGGAAAGCTTTGATCATCCCACAGACACCTTCCTT

CCATATCAAAGGTTTGGTTTTGATAAAAAAACTAATCAAAGTTGGCCCCTTCAATCCTGG

AAGACAGAAGATGACCCTGGAAAAGGTGCATTCACGGTGAAATTCAGCAGCATAGGTATA

CCTCAGTTGTTTATGTACAACCATGATCTTCCTTGGTGGCGTGGTGGACATTGGAACGGA

GAATTATTAGTAGGTATACCTAATATGAAACGAGATTTGCAAACTTTTAACGCTTCTTTT

GTGGAAGAAGATAACTATGTAGCACTTTCATACAACATGTCTGATAAGTCTGTCATAGCT

AGGTTAGTTGTTCAGCAATCTGGTTTCATTCAAATATTCACTTGGAACAATCAAAAGAGT

CAATGGAACAGGTTCTGGTCAGAACCAACAAACCAATGTGATAACTATGGAACATGTGGA

TCAAACAGTAATTGTGACCCTTTGAACTTTGAGAACTTTAAGTGCACTTGTTTACTCGGT

TTCGAACCAAAATTTCCAAGTGATTGGTATGAGAGTAAAGATGGGTCGGGAGGGTGCGTA

AGGAAGAAAGGTGCATCAGTCTGTGGGAATGGCGAAGGGTTTATCAAAGTTGTAAGCTTG

AAAGTTCCTGATATATCTGGAGCAGCTGCCATAGATGGTTTGAGTTTGGTTGAATGTGAG

AAAGAATGCTTGAGAAACTGCTCTTGTACTGCCTATGCAGTTGCTGATGTGAGGAATGGT

GGAAGTGGATGTTTGGCATGGCATGGGGATTTAATGGACATTCAAAAACTTAGTGATCAA

GGCCAAGATTTATATTTGCGCGTCGATAAAGTTGAACTTGCTAATTACAACAAAAAAAGC

AAAGGTGTCCTTGATAAAAAGAGGTTGGCTGTAATTCTGGTAGCTTCTATAGTTGCAGTT

GTCATCCTCCTCTCCTGTGTGAATTACTTGTGGAAGAAAAAAAGAAAGGATAAAATGAAG

CGGCAATCAAACCGAGATTACTCTGCAGAAGAGAGTGATGCTCAAACCAACACACATCCA

AATCTACCCTTTTTCAGCCTTAAAACGATAATGACAGCTACAAGATATTGTGGTCATGAG

AATAAGCTTGGACAAGGTGGATTTGGCTCTGTTTATAAGGGTTGCTTGGGTAACGGACAA

GAGATAGCAGTGAAGAGATTGTCCAGAGATTCAGGTCAAGGCAAAGTAGAGTTTAAAAAT

GAAATTACACTATTAGTTAAGCTCCAGCACAGAAATCTAGTAAGGTTGCTCGGTTGTTGT

TTCGAAAAAGAAGAAAGGATGCTAGTTTATGAATACCTACCAAACAAAAGCCTAGACTTC

TTTATATTCGATCAAAACCAAAGGTCATTATTGGATTGGGTTAAGCGTTTTGAAATCATT

TGTGGGATTGCTCGAGGTGTTTTATATCTTCATCAAGATTCAAGGCTGAAAATAATTCAT

AGAGATCTAAAAGCCAGCAATGTTCTCCTTGATGCTGAAATGAATCCCAAAATCTCAGAT

TTTGGGATGGCAAGAATATTTGGAGAAGATGAAATTCAAACTAGAACAAAAAGAGTGGTT

GGAACATATGGGTATATGTCACCAGAATATGCAATGGAAGGACGATATTCAACAAAATCT

GATGTTTTCAGTTATGGGGTCTTACTACTGGAGATTATTGCTGGCAAGAGAAACACACAT

TGTGAAACTGGAAGAGACTCCCCAAATTTAATTGGACATGTGTGGACTGTATGGACAGAA

GAAAAGGCCTTGGATATAGTTGATGAAGCACTAAACCAGTCTTATCCCCCTGCTATAGTT

CTGAGATGCATTCAAATTGGACTCTTGTGTGTGCAAGAAAATGCGACGAATAGACCATCA

ATGTTAGAAGTGGTTTTTATGCTAGCCAATGACACGCCTCTTTGCGCACCTCAAAAACCA

GCATTTTTATTCAACGATAACAAAGATTTGCAAGAGTCATCAACGTCGGGAGGAGGATCT

TCAATAAATGAATTAACGGAAACTACCATCATCGCTCGCTAA

>MS.gene053602.t1

ATGTCTACCATGACAACCAAGTCCTCCAACTCACGAGATGGAATCACCTTCTATTTGGCA

CACCCCAATTTTCCACTACCTGTTCCAAAAGATGGAAGTGGTATTGGTCTCATGAGTAGC

GTTCAATTAGCTACTCCAAATTACACAAAAGAAAATCCCTTTGTGGCTGTGGAATTTGAT

ACCTTTGTTAATACATGGGATCCTACATATGATCATGTGGGGATTGATGTTAATTCAATC

AGCTCTTCTTACACAACCCAATGGTTCACTAGCTTGGACGAAAGAGGATATGATGTTGAT

GTTAGTTACAATTCTATGTCAAACAATTTAAGTGTCACCTTCACAGGTTATCGAGATAAC

AACACAATCGAACAGAATTTGTTTCAAATTGTCAATCTAAGAGAATTCCTACCTGATCGG

GTTGAATTTGGCTTCACTTCAGCAACAGGATTGTTTTGGGGGGAGGAACATACTCTTAGG

TCATGGTCCTTCAACTCCAGTTTAGATTTTGAAGCACGCAAAGACGAAGGAAAAAACAGG

ACCTTTGTGTTGTGGAAGAGGTGGAAGAAAGAAAGTGAAGAGGAAGATGGTGAATTTGAA

GAGTACATGGGAGAGGATTTTGGAAGAGGGACAGGACCAAAGAAGTATACATACGCAGAA

TTAGCACAAGCAGCTAATAATTTCAAAGATGAGCATAAACTAGGTCAAGGAAGATTTGGA

GGTGTTTATAGGGGTTTTCTTAAAGACACAAACTCTTATGTTGCTATTAAGAGGGTATCA

GAAGATTCACATCAAGGGAAAAAGGAGTTCGCATCAGAAGTAAAAATTATTAGCAAGTTA

AGGCATCGAAATCTAGTGCAATTGATTGGTTGGTGTCATGAAAGAAAAAGGCTTTTACTT

GTATATGAGTACATGCCAAATGGAAGTTTAGACATTCATCTTTTCAAAAATCAAAGTTTG

GTTAAATGGGGTGTTAGATATACAATAGCTAGAGGCCTGGCTTCTGCATTGTTATATTTG

CATGAAGAATGGGAACAATGTGTTGTACATAGAGACATAAAAGCAAGTAACATCATGTTG

GATTCAGAGTTTAATGCAAAACTTGGAGATTTTGGTTTAGCAAGGTTTGTGGACCATGCA

AAAGGTGCACAAACTACAGCTTTAGCAGGGACTTTGGGATACATGGCTCCAGAATGTGCT

ACCACAGGAAGAGCTAGTAAAGAAACAGATGTGTATAGTTTTGGAATAGTTACTTTAGAG

ATAGCTTGTGGAAGAAAACCCATAATAAACCTTAAGGCTCAAGAAAATGAAATACATATT

GTTGAGTGGGTTTGGGGGCTTTATGGAAGAGGGAGAATTGTTGAAGCAGTAGATCCAAGA

CTAGATGGAGATTTTGAAAAGGAGCAAATCAAGTGTATGATGATTGTTGGTCTTTGGTGT

GCTCATCCTGATCCTAATAATAGGCCTTCAATAAGACAAGCTATTCAAGTGCTTAATTTT

GAAGCTCCAGTGCCTAATCTTTCATCAAGTATGCCTGTACCAACATATCTTGAGGGCCCT

TTGAATTCATACACAACTCCATTTAGCATAAGTGGTTCAGAGAAAGGTCAAAGCAAGAAT

ATAATAAACTTTAATTCAAACACAAACTCTTCTGGGTTTACTACTACAACTTCTGAGGAT

GTTTCTCCCTCTATGTCGCTTCTCTATTCTAGATAA

>MS.gene053611.t1

ATGTCGAGAATGTCTGAGTCTAACGAAGAAAGCATGATTCTAGAAAAGATGAATAAAAGA

AGAAAATGTGAGAATCAAAGTAATGAAGAAAGTGAAGACAGGCTTAGTGACTTACCCGAC

GGTGTTATCCTTCACATTTTGTCATTTTTGAATACCAAACATGTCGTTCGAACTTGTCTT

TTGTCCAAGAGATGGGAACATCTCTGGAAACGTATTCCAACTCTTATGTTGTATGCATCA

AGATTTTCCACTGTTAAGCAATTTGCCATGTTTGTGTCTAAGATTTTGACTCTTCGTGAT

ACCTCAACTGCGCTGCACGCTCTTGATCTTGACCGTCATGGTGATATTGAGCCTCAACTC

CTTAAAAAGATTTTAAACTATGTTTCATCTCATAATACCCACCTCCATGAATTAGGAATC

ACTGTTAATGGTGATACTAGTCTCATTATGAGCTGTGTTTCTTCATGCCGGGCTCTTACA

TCTCTTAAGCTTGATGTTGCCTCTAGAGGTCGTCATAATATTGGACAAACATTATTTCCA

AAATCTTTGAATTTGCCAACATTGACCAGCTTGGAACTTATATGCATCACCTTTTGCGGC

GGTGAAAGCAGTTGTGCTGAGCCCTTTTCAGCCTTTAACAGGTTGAATAGTTTGGTCATT

TCTTATTGTACAGTTAAGGATGCACAAATCCTCAAGATATCAAGTGAGACACTTGCCAAT

TCAGCTATGCATCATAATTCCTCTAACTTTGCCCAAATCGAGTTATCTGCTCCTAGGCTC

TGTACCTTTACTATTACTGGTATGCCTATACACAAAATATGCGGCAGTGGTCTTTCTTCT

GTTAAACAAATAAATATTGCTGCACAAATTTATTCAAATTCGGAGAAACCTCCTATGGTT

CTATTGAGTTGGCTGCTAGACCTTGCCAATGTAAAATCAATGACAGTCTCTTCAACTACT

CTTCAGATTCTCTCCTTAGTTCCTGATTTATTAGAGGTTAAGATCCATTCTTTGTGTAAC

TTGAAGTCGATGGAAATAAAATTGGAACCACTTGAAGCTGAATTCGGATTACCCTACATA

GTGAAAAATGCCATGTTAAAGAAAGCTGCTGCCAACTCACGCAAAGAAGTTGCTAAGTTA

AGAAAGGCGTTTAAAGCAGGTTTGAAACCGCCTCTCATACCAGATGGAATAGTTGACTTC

TTGCGACAAAACTCACCATCGGTGGAAATTAACATCACAACAAAGCACATGAGTGATTTT

AATATTAAGCAGGTTGTAGAATCTATAAAGGACGCAAATATTATCAACTACAGCTCACAG

TTTACCGCGCCTGCCACTACCGCGCCTGCTTCGGCTGCTGAGTCTGCTTCTGCCACTGCG

CCTGCCACTGCTGTACTTCCAAATCTTCTTCTCTGTCACGCTGAAAAGGACAACAAATCA

TCAATTGAAGATAAGGCGAAGAAGCACTGA

>MS.gene053607.t1

ATGGTTTCTATATTCTGTTATGCAAGGGAAACAATTTTGCTTTTCCTGATAGTCCCTCTT

GCACATGCTCACCTTGTGTCTTTTGATTACCCTATGTTTAGTTACGATTGTAAAGAGCCA

CCAGAGCTTGATGGAGATGCCACCATTCTAGATTCTGACAAAGTTATCCGACTCACAGGG

TACCCTGATGACCCAAATAAGGCTTCTGGTGTTGGTCGAGTCACAAGTCCCAAACTTATC

AAACTTTATGAGAATAGCTCAAATCAAGGTTCTTATGGAGATGGTTTGGCATTCTTCCTG

GCAAGCTCAAATCTCCCAAAAGCTAATCATATAGGAGGTGGAGGGGGTTTTGGTCTTGTG

CCTGAAAGCAAAGTTGCTTTAAACTCAACTGAGTATTCGTTTGTGTCAGTGGAGTTTGAC

ACGTACCAAAATTCTTGGGACCCAAGATTCAACCATGTAGGAGTGAATGTCAATTCTGTG

GTGTCTGATACATCTATTGAATGGTTCACAAATGTTTCAGAAAGGATGGTTTACAATTGT

AGCATTGAGTACAGTTCAAGCAATAACTTTCTGACTGTTTCTTTCGCTGGATACAGATTG

AATGCATGGCAAGAGCCGCAAAACTTTTCACACATCATTGATCTAAGAGAATATTTAAAA

GACTATGTTATTGTTGGCATATCAGCTTCAACAGGAAAAGTAGATGAGGAACATATGCTA

ATGTCATGGTCTTTTAGTACAAGCCAACCAAGTTATGTTGATCCTGAGAAGAAAAGCAAC

TCAAAAACTACTTCTGATCAGGATATGGATGATGAATTTCGAATGAATGCTGGACCTAAG

AAGATTAGTTATTATGAATTGTTGAATGCAACAAACAACTTTGAAGAGACACAAAAGCTT

GGCCAAGGTGGTTTTGGCGGTGTTTATAAGGGTTATTTTAAAGACTCAAACTCAGTTGCA

GCTATAAAAAGGATATCAGCAGATTCAAGGCAGGGTATAAAGCAATACTCAGCAGAAGTG

AAGATCATTAGCCAACTGAGGCATAGAAATTTGGTGAAACTCAATGGTTGGTGCCACAAG

AAGAATGAACTCATCCTAATATACGAATACATGCCTAATGGCAGCTTAGACTTTCATCTT

TTTCGTGGAGGAAGCATCTTGCCGTGGGACTTAAGGTACAATATAGCTCTTGGGTTGGCC

TCGGCATTGCTTTATTTGCAGGAAGAATGGGAAAAATGTGTGATTCATAGGGACATAAAA

TCAAGCAACATAATGTTGGACTCTGACTTCAATACTAAGCTTGGGGATTTCGGTTTAGCT

AGGTTGATGGATCATGAGAAAGGGTCAGAAACCACAGGTGTAGCTGGAACCAGAGGATAC

CTAGCACCTGAATACATGGACACAGGCAAGGCTAGAAAAGAATCAGATATATTCAGTTTT

GGGGTTGTTTTGTTGGAGATAGCCTGTGGAAAAAAAGCCATACACCACCAAGAATTGGAG

GGTGAAGTATCATTAGTTGAATGGGTTTGGGAGCTGTATGGATTGAGAAATCTAATTGTA

GCAGCAGACCCAAAGCTATGTGGAATATTTGATGTGAAGCAATTGGAATGTTTGCTAGTT

GTTGGACTTTGGTGTGCTAATCCAGATAACACTTCAAGACCATCTATAAAGAAAGTGATT

AAGGTGCTCAATTTTGAAGCTCCATTACCAATTCTTCCACAAAACATGCCATTCTCAGCC

TCTCTGTCTCCTACAACTAATGAGCAATTTTTCTCCGTTCCTTCTTTCTTTAGGTCTACA

GGGGGGAATAACAAACTCCTTAGCACCGACACTTCTGATGGAATCGGTGTCCAATACTAG

>MS.gene053599.t1

ATGTTTTTGATGTTAGGCTTTTGTTTCAAGATTCTTCACTTCGGTTGGCATTTTAAGGGT

GCCCTACGTTTTCTCTGTGATTTTGGACGCATTCCCACAATTCGTTTATGTTTGGAAAAC

TTTGTTTTGGAAGTTTCTAAGAAAAAAATCCCATCTTTGGAAAATGGGTCTCACCCTAAT

ACCAATTCTTCGACTCAGAAGAGATTGAATGGTCGTTTGGAAGATCGGTTGGAAAGGAAA

GATGATAGTGAAATGCAGGTTCCTGATGTGGATGAGGTGTTTGATGTAATCACTCTGAGA

AAGTTGATAAAGATTGAGCGCAGGAAAGCTAATGCGGCGTTTGCGGAGCTTGATAAGGAA

CGGACAGCGGCTTCTTCATCTGCGGAAGAGGCAATGGCGATGATTCTCAGGCTTCAGAGT

GAGAAGAGCTCGGCTGAAATTCAAGCGAATCAATTCCGCAGAATGGCTGAACAGAAGTTA

GAGTATGATGATGAAGTGATCGAGTCTTTGGAATGGACGATCTCGAGGCATGAATCTCAT

AGGAGTGTGTTGGAAGAGCAGTTGAGGGTTTATAGAGAAGAACTCAAGCAATATCTTGGG

GAGGATGGGATAAACCAACTTGAAGCTGATGTTAGTAGAGATAGGAGTTTTGAGAATGAA

GCAGTTGATTCTGTAGTTAGCTCTTCTGAAAACGGATCACCGACCTTGTAA

>MS.gene053608.t1

ATGATCCATCTTGCAAACTCACTATCATTTGATTACCCCTACTTTAAGAATGGTGATGTC

AACTGGGAAGGAGATGCTTCTCCTTATAAAGGAGCTATTCAAATCACTTCTAATACCCTT

GACCAAAACAACAATTACAGTGTCGGGCGAGTCACAAGTTTTAAACAGATGCATCTCTGG

GATTTGAACTCTGGAAACCTCTCTGACTTCACCACCAAGTTTTCCTTTGTTGTTTATTCA

GACAAAAGAGATTATGGAGATGGAATGGTATTCTTCTTGGCTGATCCAGATCTCCCACTT

TTGAAGAATATTTCGCAAGGAGGTGGTCTTGGTCTTGTAGATGGAGATCAAGTGCTAAAA

TCAACTCAACACTCTTTTGTAGCAGTGGAGTTTGACACCTTCAATAATCCATCATGGGAC

CCTCCAGGTGAAGGCACTCATGTTGGCTTGAATTTCAACTCTATGAGGTCTAACATAACT

AAGCCGTGGTTGACAAATATTCAACAATGGAGAGTTTACAATTGCAGCATTGAGTACAAA

TCAAGCACTCTAAATTTGAGTGTTTCATTCACCATGTATGATGATGATGATAAACCAGTT

GAAGAGTACATATCATACAAAGTTGATTTGAGAGATTTCTTACCAGAGAGGGTTATAGTT

GGCTTCTCTGCTGCAACAGGAAGATTGTATGAGGTGCATACTCTCCGATCATGGTCATTT

AGTTCAAGTCTACTAAGTGATGAGACCAAGGATCAGGTAGTGGCACCAATTGCCAGTCCT

ACAATTAATGAAAAAGAAAACAAGATGGGATTGAAGGTGGGGCTTGGAATTGGAACAGGC

TTGGCTGTGAGTTTATCAGGGCTGCTTTGTACTCTCTTGTGGAAGAGGAGTAGAGAAAGA

AAAGAGTCAGGTTTTGATCTCAACATGGATGATGTATTCCAAAAGGGGTCTGGACCTAAG

AGGTTTTCCTACAATGAACTAGTGAGTGCAACAAACAAATTTTCAGAGTCAGATAAGGTG

GGGCAAGGTGGTTTTGGTGGCGTATACAAAGGTTATTTGAAAGAATTGAACTCCTACGTT

GCTATCAAGAGGATATCAAGAGAGTCTAGACAGGGAATACTAGAATATGCAACCGAAGTG

AAGGTCATAAGCCAGTTGAGACACAGAAACTTAGTACAACTTCTTGGTTGGTGCCACAGG

AAGAATGATTTTCTTCTTATATATGAGTTCATGTCAAATGGAAGCTTAGATTCTCATTTA

TACGGTAAAAAAAGCTTCTTGACATGGACGACTAGGTATAACATAGCATTGGGCTTGGCT

TCAGCATTGCTTTACCTACAGGAAGAATGGGAGCAGTGTGTGCTTCACAGGGACATTAAA

TCGAGTAACATTATGTTAGATTCATGTTTCAATGCTAAGCTTGGTGATTTTGGTTTAGCT

AGGCTTGTAGATCATGAGAAAGGGTCACAAACCACCCTTATAGCCGGGACAAGGGGTTAT

ATTGCACCTGAATATTTCACATCAGGAAAGGCTACTAAGGAATCTGATATATTCAGCTTT

GGAGTAGTGTTATTGGAAATAGCCAGTGGAAGGAAAGCCATTGAAAGAGAAGAAAAGGAG

GGTCAAATAAGTGTAGTGGAGTGGGTTTGGGAGCTCTATGGATTGGGAAAGTTCCTTGAA

GCAGTTGATCCCAAGTTATGTGGAGCATTTGATGAGCAACAACTGGAACGTTTGGTTATT

GTTGGCCTTTGGTGTGTTCATCCAGATTATTCATTCAGGCCTTCTATAAGACAAGTGATT

CAGGTTCTCAAATTTGAGTCTCCTTCACCAATACTTCCAGAAAAAATGCCTGTGCCAACC

TATCTTCCTCCAACAATAAAAGCACTTTTTTCTTCAATTTCGTCTATATATTGGACAAAT

AGTTAG

>MS.gene053606.t1

ATGTCACTGATTCCAAGTTTCTTCGGCGGCCGAAGGAGCAACGTTTTCGATCCATTCTCC

CTCGACGTTTGGGACCCCTTCAAGGATTTTCCTTTCAACAATTCTGCACTTTCTGCTTCA

TTCCCTCGTGAGAATTCCGCATTTGTGAGCACACGAGTCGACTGGAAGGAGACTCCAGAA

GCGCATGTGTTCAAGGCGGATCTTCCAGGAATGAAAAAGGAGGAAGTGAAGGTAGAGATT

GAAGATGATAGGGTTCTTCAGATAAGCGGAGAGAGAAGCGTTGAGAAAGAAGATAAGAAC

GATCAATGGCTTCGCTTGGAGCGTAGCAGTGGAAAGTTCATGAGGAGATTTAGATTGCCT

GAGAATGCGAAAATGGATCAAGTGAAAGCTGCAATGGAGAATGGTGTTCTCACTGTCACT

GTGCCAAAAGAAGAGGTTAAGAAGCCTGAAGTGAAGACCATTGATATCTCTGGTTAG

>MS.gene053601.t1

ATGGCAGCATCTTGCTACCATCACAAACCTCATCTTCTTGCTTTCTTTCATGTTACATTA

ACATTCTTGCTTCTTGTAACATCTCGTGCAGCTCCATTATCATTCAACTATGACCAACTT

GGTAGTGACAAAACAAATAACATGAATTTTTTTGGTGATGTCGAACAAGACAACCAAGTC

CTCCAACTCACAAAGTACGACAAAGACAGTTTAGGCAGAGTCACATATTCAAAATTGTTT

CATCTTTGGAACATAAACACAAGTGAAGTCACAGATTTCACTACTCGTTTCAAATTTTCT

ATCAACACCCCCAACAAAAGTTATCATGGAGATGGCATCACCTTCTATTTGGCACGCCCC

AATTTCCCTATGTCCCAAATAGATGGAAGTGGTATCGGTCTCGCAAGCCGCGTGCAATTG

AATAATCCAAATTACACAAAAGAAAATCCATTTGTGGCTGTTGAATTTGATACATTTGTT

AATGATTGGGATCCTAAATATGATCATGTTGGGATTGATGTTAATTCTATTAGCACTAAT

CACACTACACAATGGTTCACTAGCATGGATGAAAGAGGGTATGATGCTGAAGTGAGTTAT

GATTCAAGTTCAAACAATTTAACTGTCACATTTACAGGTTACCAAGATAACAAAACAATC

CAACAGCACCTGTTTTATGTTGTTAATCTGAGAGATGTTCTACCAGATTGGGTTGAATTT

GGATTCACTTCAGCAACAGGGTTTTTCTGGGAGTATCATACTCTTAGCTCATGGTCATTC

AACTCAAGTTTAGATTTTGAGGCAAAAAAAGATGGAACAAAAACCGGACTTGTTGTAGGA

CTCGGTGTTGGTGGAGCTATTGTTTTGATTTGTGTTATTGGGCTGGTTTGTCTCTTGAAA

TGGAAGTTGAGGAATAAAGGCATGAAGGATGAATCACATTTTGATCTTGCAATGGATAGT

GATTTTGAAAGAAGCTCTCTACCTAAGAAATTTACCTATGAAGAACTTGCTAGATCAACT

AACAACTTTGCAAATGAACACAAAATTGGAGCTGGAGGTTTTGGAGCAGTGTACAAGGGA

TTTATAAGAGACTTGAAAACTCATGTTGCAATTAAGAAGGTTTCTAAAGAATCAAATCAA

GGAGTGAAAGAATATGCATCTGAAGTCAAAGTCATTAGTCAATTGAGGCATAAGAATTTA

GTTCAACTCTTCGGCTGGTGTCATAAGCAGAATGATCTGCTTTTAGTTTATGAGTTTGTG

AAAAATGGAAGCTTAGATTCTTATCTTTTCAAAGGAAAAGGTTTGCTGACATGGCCAGTA

AGATATAATATAGCTAGAGGTTTGGCCTCAGCGTTATTGTACCTGCACGAAGAGTGTGAG

CAATGTGTGCTTCATAGAGACATAAAATCAAGCAATGTTATGTTGGATTCTAATTTCAAT

ACAAAGCTTGGAGATTTCGGGCTCGCTAGACTAATGAACCATGAGACAGAATCAAAGACA

ACTGTTTTAGCTGGAACATATGGATATCTATCACCTGAAGCTGCTACTAGAGGAAAGGCT

AGTAGAGAATCTGATGTATATAGTTTTGGAGTTGTTGCTTTGGAAATTGCTTGTGGTAGA

AAGGCAATTGAACCAAGTCTTAGTGAAGAACATATTTACTTGGTCGATTGGGTTTGGGAA

CTTTATGGTATTGGTGATCTTCTTAAAGCAGCTGATTCAAGATTATATGGTGAATTTAAT

GAGAAGGAAGTAGAGAGGCTAATGATAGTTGGACTTTGGTGTACTCTTGTAGATCATCTT

CAGAGGCCTATGATTAGGCAAGTTGTTCAGGTGCTTAACTTTGATGCTCCATTACCTAAT

CTTCCATTACAGATGAATGCATCTACCTATAATACATCTTTCAATTCTGTGTCCTCTAAA

TCTAAGATTTCTGGTTTTGAAAATAACCAAACTGGAACCTCAACTTCAAGTAATAGCTCT

CTCACAGGCTCATCACAATCAAGCACAACCTTTGAAGTCATATCTCCATCAGCTGCACTT

CTACATAAATAA

>MS.gene053605.t1

ATGGCAGCATCTTGCTACCATCACAAACCTCATCTTCTTGCTTTCTTTCATGTTACATTA

ACATTCTTGCTTCTTGTAACATCTCGTGCAGCTCCATTATCATTCAACTATGAACAACTT

GGTGGTGACAAAACAAATACCTTCAATATTTCAGGTGATGTCTATCAAGACAACCAAGTC

CTCCAACTCACAAAGTACGAGAGAGACAGTCTAGGCAGAGTCACATATTCAAAATTGTTT

CATCTTTGGGACAAAAAAACAAGTGAAGTCACAGATTTCAATACTCGTTTCTCATTTTCT

ATCAACACCCCCAACAAAACCCATCATGCAGATGGCATGACCTTCTATTTGGCACACCCC

AATTTTCCCATGTCCCATATATTTGGAGGCGGTATCGGTGTTGCGAGCCTTGTTCAATTG

AGCGATCCAAATTACACAAAAGAAAATCCATTTGTAGCTGTGGAATTTGATACCTTCGTT

AATGAGTGGGATCCTAGCTATGATCATGTTGGGATTGATGTTAATTCTATCAGCACTTCT

TACACAACGCAATGGTTCACTAGCATGGATGAAAGAGGATATGATGCTGAAGTCAGTTAC

GATTCAAGTTCAAACAATTTAACTGTCACATTTACAGGTTACCAACATAACAACACAATC

CAACAACACCTGTTTTATGTTGTTAATCTGAGAGATGTTCTGCCTGATTGGGTTGAATTT

GGATTCACTTCAGCAACAGGAACTTTTTGGGGGGAGGAACATACTCTTAGGTCATGGTCC

TTCAACTCCAGTTTAGATTTTGAAGCACACAAAGACGAAGGAAAAACAGGACCTGTTATA

GGACTAGCATTGGGATTAGGAATTGGTGGATTCATTTTGATTGGTGTATTGAGTCTTATT

TCAGTTGTGTTGTGGAAGAGGTGGAAGAAAGAAAGTGAAGAGGAAGATGGTGAATTTGAA

GAATACATGGGAGAGGATTTTGGAAGAGGGACAGGACCAAAGAAGTATACATATGCAGAA

TTAGCACAAGCAGCTAATAATTTCAAAGATGAACATAAACTAGGTCAAGGAGGATTTGGA

GGTGTTTATAGGGGTTTTCTTAAAGACACAGACTCTTATGTTGCTATTAAAAGGGTGTCA

GAAGATTCACATCAAGGGATAAAGGAGTTCGCATCAGAAGTAAAAATTATTAGCAAGTTA

AGGCATCGAAATCTAGTGCAATTGATTGGTTGGTGTCATGAAAGAAAAAAGCTTTTACTT

GTATATGAGTACATGCCAAATGGAAGTTTGGACATTCATCTTTTCAAAACACAAAGTTTG

TTGAAGTGGGGTGTTAGATATACAATAACTAAAGGCTTGGCATCTGCATTGTTGTACTTG

CATGAAGAATGGGAACAATGTGTTGTACATAGAGACATAAAAGCAAGTAACATCATGTTG

GATTCAGAGTTTAATGCAAAACTTGGAGATTTTGGTTTAGCAAGGTTTGTGGATCATGCA

AAAGGTGCACAAACTACAGCTTTGGCAGGGACTTTGGGATACATGGCTCCAGAATGTGCT

ACCACAGGAAGAGCTAGTAAAGAAACAGATGTGTATAGTTTTGGTATTGTTGCTTTAGAG

GTTGCTTGTGGAAGAAAACCTATAATAAACCTTAAGGCTCAAGAAAATGAAATACATATT

GTTGAGTGGGTTTGGGGGCTTTATGGAAGAGGGAGAATTCTTGAAGCAGTGGATCCAAGA

CTTGGTGGAGATTTTGAAGAAGAGCAAATCAAGGGCCTAATGATTGTTGGTCTTTGGTGT

GCTCATCCTGATCCTAATAATAGGCCTTCAATAAGACAAGCAATTCAAGTGCTTAATTTT

GAAGTTCCACTTCCTAATCTTTCATCAAGTATGCCTGTACCAACATATCTTGATGGGCCT

TTAAACTCTTTTTCAGCTCCATTCAGCACAAATAATTCAGAGGAAAGTCAAAACCAGAAT

ATAAAAAGCTTTAGTTCAACTTCAAACTCATCTTGCTATTATGTATCCTCTAAGACTCTT

GATTTTGAAAATAATCAAGCAAGGACCTCACAATCAACCTTTGATGTCATTTCTCCATCA

GATGCACTTCTAAATACATACCAGTAG

>MS.gene053604.t1

ATGCCAAATGGAAGTTTGGACATTCATCTTTTCAAAACACAAAGTTTGTTGAAGTGGGGT

GTTAGATATACAATAACTAAAGGCTTGGCATCTGCATTGTTGTACTTGCATGAAGAATGG

GAACAATGTGTTGTACATAGAGACATAAAAGCAAGTAACATCATGTTGGATTCAGAGTTT

AATGCAAAACTTGGAAATTTTGGTTTAGCAAGGTTTGTGGATCATGCAAAAGGTGCACAA

ACTACAGCTTTGGCAGGGACTTTGGGATACATGGCTCCAGAATGTGCTACCACAGGAAGA

GCTAGTAAAGAAACAGATGTGTATAGTTTTGGTATTGTTGCTTTAGAGGTTGCTTGTGGA

AGAAAACCTATAATAAACCTTAAGGCTCAAGAAAATGAAATACATATTGTTGAGTGGGTT

TGGGGGCTTTATGGAAGAGGGAGAATTCTTGAAGCAGTGGATCCAAGACTTGGTGGAGAT

TTTGAAGAAGAGCAAATCAAGGGCCTAATGATTGTTGGTCTTTGGTGTGCTCTTCCTGAT

CCTAATAATAGGCCTTCAATAAGACAAGCAATTCAAGTGCTTAATTTTGAAGTTCCACTT

CCTAATCTTTCATCAAGTATGTCTTTACCAACATATCTTGAGGGGCCTTTAAACTCTTTC

AGCTCCATTCAGCACAAATAA

>MS.gene053610.t1

ATGTCGATGAGAACATCTTATGAAGTGATGGGTCAAGGAGTTGGAGGCACGGAGAATCTT

CCTTTTCGATTTTCGGACTTAAAGAACTATCTAATGACAACTCGTCAAAAGGAGATGGTG

GCTGGTGAAGCAACTGTTATTCAAGAATTCTTTAGAAATGAAGCTCTCTCGAAACCAGGT

TTTTACTATGATATCCAAGTTGATGCTGCAGAAGACATAGCCAGTATTTTTTGGGCAGAT

GGAATTATGCAACAAGATTATGCTCTATTTGGTGATGTTGTCAGTTTTGATACAACTTAC

CGAACAAACAATAAATATCGGCCATTAGCTGCCTTTCTTGGTTTTGACAATCATCGTAAG

AGTGTGTTATTTGGTGCTGCGCTACTGTATGATGAGACATCAGCTACTTTTGATTGGTTG

TTTACAACTTTTTTGAAGTGTATGTCCAATAAGAAACCTCAAACCATATACACAGACCAG

GCTGGTGCGTTGTTGAAGTCAATTCCGAATGTTTTTCAAGGTGTTTTTCATGGTCTTTGC

TCATGGCACATGGGAGAGAATGCAAAGAAAAATCTCGGCTCTCGTGCAAACAGTGCATTT

TTCGATGAGTTAACTAATTTGGTTTCAAATGTCGAAGATGAATCAGATTTCGACTACAAT

TGGGATCAGATGATGAAAAACTGTTTTAATGGAAGGCCTACTTCGGACTTTAAGTGGCTT

GTTCAAACTCATTCAAATCGTATGCATTGGTCTTCAGTTTGGGTGAGGTCACATTTTACA

GCTGGTTTGAAAACGACACAGTTAAGTGAGTCTTTCAATGCTTTTCTTCGTGGATTTTTG

CAGCCAGACCATTCACTTGTTCGATTCTTTAGTCATTTCAACATTATGGTTCAGAGAATG

AGGGATAATCATGCTGAGTTGGACTTTAAGGCTGCAAACACTAGAACAAAAAATAATTAT

CCCAACAGTCAGCTTATGCGCGGTGGACTAGAGATGTGCGTCCGTCTGTCGATAAGCTGA

>MS.gene053609.t1

ATGGGGAGAGGAAAGATTGTGATTCGAAGGATTGAGAATTTGACAAGCCGTCAAGTGACT

TTCTCAAAGAGAAGAAAAGGGTTGATGAAGAAAGCTAAAGAACTATCCATTCTTTGTGAT

GCACAAGTTGGATTGATTTTGTTCTCCTCTACTCACAAGCTTTATGACTATGCAAGTTCC

AGCATGCAGTCAGTGATCGAGCGTTATAACAAGTTAGTAGAGGACCATCATCAAGCAATG

GATCCTACTTTGGAACTCAAGTTTTGGCAAAGAGAAGCAGCAAGCTTGAGGCAACAATTG

CAGCACTTGAATGATAGCCAAAGGCAATTGATGGGCCAAGAACTATCTGGTTTGGACTTA

AATGAGCTGCAGCATCTAGAAAGTCAATTGGAGATGAGTTTGAAAAGCATCCGCACGAGA

AAGGGTCTAAGTTTTAGTGATGAGATCAATGAACTACTCAAAAAGGGAAGCCTTAGTAGT

CAAGAAAATGAAGATCTTCGTAAGAAGATAGACCTCATTGGTGAGAAAAATGCAGAACTA

GAGAAGAGCAAAACAAAACCAAATTGCAACGGAAACGTTTATAACATTCTCGCCTGTAAC

AACCTCGCCATCGCCTGTAACAGTTTCCGGCACCGGCGTTCACCAACTTCATCACCGTCG

TCCTTTTCCACCGTCAAAATCACCGTCGTAGTTCACAATAATCATCACCGTCGTCGTTTT

CTGCCGATTTCGTCATCATCGTTCGCATTTCCAACTTTCTTCACCATAACCGCCACCGTG

GAAAAAACTACAGAGCAAGGTACATTGCCATCATTCAGTTTAGGACTTACACAAATGGAG

GAGGAGGATGAAAAGAATATCAATGACTCGTCATCTGCTGGTGATACAAATAAGGATCAT

AAAAAAAGAGAGGCTAAGGGCAAAAAGAAGAAGCATAAACCAATAGAGGCTAAGGAGCAT

AAAAAAAGAGAGGCTAAGGGCAAAGAAAATAAGGAGCACATAACCAGGAGTAAAAAAAGG

AAACAAGAGAATGAAGAACAATTGGAGGATGAACAAGAGAGGCTAAGGGCAAAGAAAAAG

GTCACTGGTTCATCATCTGGTGGTGATAAACATAAGGAGCAGAAAGAAGATGCTAGAAGT

AAGGAAAAGAGGAATAAGAAAAGGAAACAACAAAATGATGGTGAAGATTCTGATGAAGGA

AGTTTGAATGAGGAAAATGCAAAGCACAGACTGAGGCACAAGCTGAGTATACCTAAGGTA

TATGAAATTATGAATTCAATATATCGAAAGAGAAACAAAACAGAGATCATTGAGGTTTTG

AATAAGTCTGGCTTTGGTGGAATGTTGCATATCTGCAACTGGAAAAGAATCCACACTTTC

TTTGTGGATTGGGTTGTTAAAAACTTTGACAAAGATAACATGTGGATTGTATTAGGCAAG

AATGAGGTACTTCCATTGAAAGAGGAAGATGTACATAGGGTGTATGAGCTTCCAGTGGCA

GGGAAGAAGATTAACGTGGATCTATGTTCTGAGGAAGCAATAAAGAAATTAAGGAAAGAA

TTAGGTCTGACTGGAACTTATTCGGCTTCTGTGAGGGTGACAGAATTGGAGAGAATACTG

AAGACACTCGAGAAACCAAATGCGTGGGTGAAAGGTGCAATCTGTTATATCATTCACAAT

ATTTTGTGCCCCACTAATAGTAGCTTTGTATCTCTCCAATATGCACACATATTTGAAGAT

CCAGCTGGGGTATCTTCTTATAACTGGTGCTCCCATGTTCTTGAATATATGAAAGAAGGC

CTGCAAAATCCGGAAGTTGCAAATCCATTAGCGGACTTCCACTTCCTAATGATTAATTAC

ATGGACAAAATGGGGAAGAGAAGTCCTTTTCTGACAGGACAATACAAGCGGCCATCACTT

CGTGATTGGGATGTCATGGCGGCAAACCAAGAACTTCAGAAGGTCTATGACATTATGGGA

CTCGAGCATGGACTGACAGCCGGCGTAACCAGACTGCACAACACAATTGAAGGTCCCCTT

GTATTGTGTTTTGATGCAGATACTTGCCCACTCTCTAAGGCACAGATGCATCTTAATCAT

TGTAGGTCTTGCATACAAATCTACACTAAAACTGCTGAAACCTTGGAAAGAAGAATAGCT

GAGGCAAAAGTTGAAACTTCTGGGGAAAATGATGATGTTCCAAAAGATACAACCAACGAC

AGTCCAACTGAAGGTCAACCTGAACACGACAAGAATAACAGTTCAAGTCATGAAAAAAGT

ATGGAAGCACCTGATAGTGAAGAAAATAGAGGGGATGGAAGCACTGGAAGGGAAAAAAGT

GAAGAAAATCATGACAAAGATGTATCAGATGAGATTGATGATGATGATGGAGAAAATGAG

ATTGATGCCAAAATCCTTGATGATTCAGTGAGGAACGCATGCAATATTTCCGTTACACAG

GAAACAATCTTGAGGTTTCCAGAGTTTTTTGATGGGGGAGAAGCTTCAAATGCAGGTAAA

TCAGTTAAGACAAAATCTGAGACAACATCTGAAGCAAGCAATGTGAATCAAGAAAGGGTG

ATGGAGGAGGAAAACCAGGAGAAGGACACGGAGCAGGAGAACCTGGAGAAAAACACATCA

GTTAAGACAAAATCTGAAGCAAGCAATGTGAATCAAGAAAGGGTGATGGAGGAGGATAAC

CCAGAGAAGGACACGAAGCAGGAGAATGCAGAGGAGGATAAGAATTTGGAATTTTGTAAT

GACATTATTGTAAAGGCAACTCCGTTAAGGTCAGTGTTAGCAGATGAGGTAATTGACTTG

GACAATGACAAAAGTGTGAAGGCAAGGAAAAGAAAGACCAAGCATGACATATCTGGAAAA

GCCCCTGAGCGTGAAAGAAGAGCTGTAAAGAAATCGAAATACCTTGAAAGCCCTTATGAT

GAAGCTGTCTACGAGTCAAAATGCAATAAGATGCAAAAGGACATATCAACATTTGCATGG

AGCAATTCACTTGATGAGGAGGAGTTTTTGTACTGCTCGGAAAACGGCGCTCATGCTTAC

GCTCTACAAAGAAGAGACTTGTACACACTTAAAAAAGATGAATGGGTCAATTGCTTCGTT

ATAAATGCCTGGGTCAATTGCTTGAACTGGAATCAACAACCAGCTGACAAAATGACAAGA

CTGGTCACACCATTCATGAATTTTGTTGACTTGGAAAGACCTGATGCCCTGAATATAACA

GAAAAGGTTGCATTTGAACGATTCATCGAAAAGCTGAAAAAGTTCAAATACATGGATTGG

AAAACTATTGACCCTTCATGTCTAGAATATATAATGACACCAGCAATAATTGGAAATCCG

GGTAGTCATTATGTATCCTTCGTAGTGAATCTCAAGAGCCAAAAGTTACAATTCCTGAAC

AGCTTGGCAGGAGATCCACTGCACATGAAGAATGGAGACCCAACTGTGTACAAAAGAATG

TTTGATGTATGGCTGAAGGAGGTGGAAGCATTCGTGAAAGAATTGTATAAGAAAAGCAAC

ATCACAATGCCTTTCCAGTTTAGCAAATTTAAATGGGAAACACCAAAGGTGCCTATTCAA

ACTGATAAAGACAGTTGTGGAGTGTTTTGCATGAAGTTTCTTGCTGAATGGGAGGATGGC

AATCCAGAAATGGAATCTTTCAAAGGTTGGACCAAACTGAAAAAGCGTGGGGAGAATGGA

AAGGTTACAAGAATTATGGATATGAGAATTGAAATCTGTTCAACAATATTAACTGACTCC

AGCAATAGCAAAAGACAACATGTGGAGAAGGCAGCAACTTCAAACTACAAACAAGTGCTT

GAAAAGCTAGCATCTCCGATTAAATAA

>MS.gene053603.t1

ATGTCACTGATTCCAAGTTTCTTCGGTGGAAAAAGGAGTAACGTTTTCGATCCATTCTCT

CTTGACGTTTGGGATCCATTCAAGGATCTTCCTTTCACAAATTCACTTTCCAGTTCCTTC

CCTGAGCTATCTCGTGAGAATTCAGCATTTGTGAGCACACGCGTGGATTGGAAGGAGACT

CCAGAAGCGCACGTGTTCAAGGCGGATCTTCCAGGAATGAAGAAGGAGGAAGTGAAGCCC

CTTATGGTTCAAAAAAGCCTAATGCAACAAAAGGTGGTTCTATGGGTCTGTCTAACACTT

GATAACCAAAGGTTGAACTCAACTGATAATCCTTTTGTTGCTGTGGAGTTTGATATCTAT

CGGAATCATTGGGATCCACCTCTTGAACATGCCGGAATCGACATCAACTCTATGCTGTCT

GTTGCTAATGTTACATGCGTTGTTAATCAACATTTATCTTCCATAGTTGATCTTAGACTT

TATCTGCCAGAATTTGTTATTATTGGCTTCTCAGCTGCCACTGGAAATTCAACTGCTATA

CATAGCATCTCTTCATGGGATTTTAGCTCAACTTTAGAAGGACAACAAGATAACAACAAA

ACAAACACACAAGACCCAGTTACAAGGTCTCCATCTTCAAACAAAGCTCCCATTATGAAG

AAAAAGGCCATGACAGGACTAGCAGTGGGATTGGGAGCCGGTGGATTCGTTCTGATTTGT

GTATTCGTTATTTTGGTTTTCTTGTGGAAGAAAAGAAGGGAGGAGGAAGATGGTGAATTT

GAAGAGTACATGGGTGAAGATTTCGGAAGAGGGACAGGACCCAAGAAGTATACATATGCA

GAATTAGCACATGGAGCTAATAATTTCAAAGATGAGCATAAGCTAGGACAAGGAGGATTT

GGAGGTGTTTATAGGGGTTTTCTTAAAGACGCAAAATCTTATGTTGCTATTAAGAGGGTG

TCAGAAGATTCTCATCAAGGGATAAAAGAGTTTGCATCAGAAGTAACGATAATTAGCAAA

TTAAGGCATAGAAATCTAGTCCAATTGATTGGTTGGTGTCATCAAAGGAAAAAGCTCTTG

CTTGTATATGAGTACATGCCAAATGGAAGTTTAGACATTCATCTTTTCAAGAAACAAAGT

TTGTTGAAATGGGGAGTTAGATATACAATAGCTAAAGGCTTGGCATCTGCATTGTTGTAC

TTGCATGAAGAATGGGAACAATGTGTTGTACATAGAGACATAAAAGCAAGTAACATCATG

TTGGATTCAAAGTTTAATGCAAAACTTGGAGATTTTGGTTTAGCAAGGTTTGTGGACCAT

GCAAAAGGTGCACAAACTACAGCTTTAGCAGGGACTTTGGGATACATGGCTCCAGAATGT

GCTACCACAGGAAGAGCTAGTAAAGAAACAGATGTGTATAGTTTTGGAATAGTTACTTTA

GAGATAGCTTGTGGAAGAAAACCCATAATAAACCTTAAGGCTCAAGAAAATGAAATACAT

ATTGTTGAGTGGGTTTGGGGGCTTTATGGAAGAGGGAGAATTGTTGAAGCAGTAGATCCA

AGACTAGATGGAGATTTTGAAGAGGAGCAAATCAAGTGTATGATGATTGTTGGTCTTTGG

TGTGCTCATCCTGATCCTAATAATAGGCCTTCAATAAGACAAGCTATTCAAGCGCTTAAT

TTTGAAGTTCCATTACCTAATCTTCCATCAAGTATGCCTGTACCAACATATCTTGACGGC

CCTTTGAATTCATTCACTGTTCCATTCAACACAAATGGTTCAGAGGAAGAGTTCTGCATT

TGTGAGCACACGTGTGGATTGGAAGAGACTCCTGGAGCACACGTGTTAAATTCTGATTTT

CCAGGAATGAAGAAGGAGGAAGCGAAAGTAGAGATTGAAGATGATAGGGTTCTTCAGATA

AGCGGAAAGAGAAGCGTTGAGAAAGAAGATAAGAACGATCAATGGCATCGCGTGGAGCGT

AGCAGTGGGAAGTTCATGAGGAGATTAAGATTGCCTGAGAATGCTAAAATGGATAAAGTG

AAAGCTGCAATGGAGAATGGCGTTCTCACTGTCACTGTGCCTAAAAAAGAGAGTAAGAAT

CATGAAGTTAAAACCATTGATATCTCTGGTTAA

>MS.gene053600.t1

ATGTCACTGATTCCAAGTTTATTCGGTATCAGAAGGACTAACATTTTCGATCCATTCTCC

GTCGATCTTTTCGATCCAGATTTTCCGTGCCGTGATTCACTTTCTGCTTCCTTCCCTGCA

AATTCAGCATTCCTGAGCACATGTGTGGATTGGAAGGAGACTCCAGAAGCGCATGTATTC

AAGGCGGATCTTCCAGGAACGAAGAAGGAAGAAGTGAAGGTTGAAATTGAAGATGACAGA

GTTCTTCAGATTAGTGGAGAGAGGAATGTTGAGAAAGAAGATAAGAACGATCAATGGCAT

CGCGTGGAGCGTAGCAGTGGGAAGTTCATGAGGAGGTTTAGATTGCCAGAGAATGCTAAG

ATGGATCAAGTGAAGGCAAACATGGAGAATGGTGTTCTCACTGTAACTGTTCCTAAGGAA

GAAGTGAAGAAGCCTGAAGCTAAGACCATTGATATCTCTGATTAA

>MS.gene053668.t1

ATGCAGCCTCGTCGTTATCCATCCGGCGTTCCATTCCGATCTGTAATTCATCAGATCAGA

ACTCACATCGCACTCACCAAGGTACATAATTATCTGAATAACAATGTCAAATTCAGTGTC

GGAGATGATTTAACGATTCCACCGAAACCGCGGAAACAAAAGAAATTAAAGCAAGAAAAT

GAAGAAGATAATCTGAGTGATTTACCAGATTGTGTTATTCTTCATATATTATCGTTTTTG

AACGCGAAAGAAGCGAATTTTACTAAATTTGTTTCTAAGATTTTGTCGCATCGCGATGGA

ACAATTGCGCTGCAAGAGCTTGATTTTGAACGTGTAGGGAGGCATAATTATGGGAGAACG

TTGTTTCCGAAATCTTTGGATTTGCCGGCGTTAACCAATTTGCATCTAGGGAATTTCGTC

TTTTGTGCTAATGATAAAGGCCGAACTGAGCCCTTTTCGGGTTTTAACAAGTTGAATAGT

TTGGTCATTGACAATTGTACGGTGAAGGATGCGCAGATTCTTTGCATATTAAGTGAGACA

CTTGTCAATTTGACTATGCGTAATCATTCTTCCGATGTTTACAAAATCGAACTATCCGCC

CCAAGTCTTAGCACATTTGCGTTTACCGGAACCCCTTATCAGAAACTTTGTGGGAGCAAT

CTTTCCTCGGTTAAACAAGTGAATATTGATGCAGAAATGTTGTCTAACTACACTGAGCCT

CCTTTGGTTTTGCTTAGCTGGCTGTTAGAGCTTGCTAATATCAAATCATTGACGGTCTCT

GCGAGTACTCTTCAGGTTCTTTCTCTAATTCCTGATTTGTTGAAGGATAAGCTCACTTCT

TTGTGTAACTTGAAGTCACTTAAAGTACAACTAAAACCACTTTCATATGGATTATCCATG

ACCCTGAGAACGGCCAAGTTACAGAAAGAAGTTAAAGCAGGGCCGGAACCATCTTCACCC

ATACCTGATGGAATAGTGGACTTTTTGATTCAAAACTCACCGTCGGCAGAAGTTGACATC

GTAGATTGCTCAAGGTTCGGCGGCTCCTTTGACCATCTGCCTCCATTTCCCGTGTCTTCC

ATTTTTCCTCAATTCCTTCAGCCTTCTTCCTGTGAG

>MS.gene053669.t1

ATGTCAAATTCAGTGTCGGAAGATGATTTAACGATTCCACCGAAACCGCGGAAAACAAAG

AAATTAAAGCAAGAAAATGAAGAAGATAATCTGAGTGATTTACCAGATTGTGTTATTCTT

CATATATTATCGTTTTTGAACGCGAAAGAAGCGGTTAGAACTTGCATTTTATCGAATAGA

TGGAAGAATGTTTGGAAATGTGTTCCTACTCTTGTATTGCATTCATCTGATTTTTCAACT

TTCAAGAATTTTACTAAATTTGTTTCTAAGATTTTGTCGCATCGCGATGGAACAATTGCG

CTGCAAGAGCTTGATTTTGAACGTGTAGGTAGTATTGAGCCTCACCTTCTTAAGAGAATT

GTGAATTATGCTTTTTCTCATAATGTGCAGAGATTGGGAATTTCTGTGAAAGGGGATATT

TGTCATATTCTGCCTTGTGTTTCTTCGTGTCGGACTTTAACGTCTCTTAAGCTTTCTGTT

TCACCTAAAGGGAGGCATAATTATGGGAGAACGTTGTTTCCGAAATCTTTGGATTTGCCG

GCGTTAACCAATTTGCATCTAGGGAATTTCGTCTTTTGTGCTAATGATAAAGGCCGAACT

GAGCCCTTTTCGGGTTTTAACAAGTTGAATAGTTTGGTCATTGACAATTGTACGGTGAAG

GATGCGCAGATTCTTTGCATATTAAGTGAGACACTTGTCAATTTGACTATGCGTAATCAT

TCTTCCGATGTTTACAAAATCGAACTATCCGCCCCAAGTCTTAGCACATTTGCGTTTACC

GGAACCCCTTATCAGAAACTTTGTGGGAGCAATCTTTCCTCGGTTAAACAAGTGAATATT

GATGCAGAAATGTTGTCTAACTACACTGAGCCTCCTTTGGTTTTGCTTAGCTGGCTGTTA

GAGCTTGCTAATATCAAATCATTGACGGTCTCTGCGAGTACTCTTCAGGTTCTTTCTCTA

ATTCCTGATTTGTTGAAGGATAAGCTCACTTCTTTGTGTAACTTGAAGTCACTTAAAGTA

CAACTAAAACCACTTTCATATGGATTATCCATGACCCTGAGAACGGCCAAGTTACAGAAA

GAAGTTAAAGCAGGGCCGGAACCATCTTCACCCATACCTGATGGAATAGTGGACTTTTTG

ATTCAAAACTCACCGTCGGCAGAAGTTGACATCGTAGATTGCTCAAGGTTCGGCGGCTCC

TTTGACCATCTGCCTCCATTTCCCGTGTCTTCCATTTTTCCTCAATTCCTTCAGCCTTCT

TCCTGTGAG

>MS.gene055444.t1

ATGGCGGTGGAATACACATGCTGCGAATCGGAGTTCTTCATCCACATATTGGTTATCATC

TTCTTGGTGGTCTTCGCCGGTTTAATGTCCGGACTCACCTTAGGTCTCATGTCTCTCAGT

CTCGTTGATCTTGAAGTTCTTGCTAAGTCTGGTACTCCTCACGATCGTAAACACGCCGTG

AAGATATTACCTGTTGTGAGAAATCAGCATTTATTGCTTTGTACTTTACTCATTTGCAAT

GCCGCCGCCATGGAGGCACTTCCTATTTTTCTTGATAGTCTTGTTACTGCTTGGGGTGCT

ATCCTGATTTCAGTCACGTTGATTCTTCTCTTTGGTGAGATTATACCCCAATCAGTTTGT

TCTCGATATGGTTTGGCCATTGGTGCATCAGTCACTCCTTTTGTCCGCGTGCTTGTATGG

ATATGTTATCCAGTTGCTTTTCCAATTAGCAAGTTGTTAGACTATTTGCTGGGTCATCGA

CATGAAGCCCTTTTCCGTAGAGCCGAGTTGAAAACACTAGTAGATTTGCATGGTAATGAG

GCTGGAAAAGGTGGGGAACTGACACATGATGAAACAACAATAATTGCCGGGGCACTTGAA

CTCAGTGAGAAGACAGCCAGTGATGCTATGACTCCTATATCTGAAACATTTGCTATTGAT

ATTAATTCGAAGCTTGATAGGTAA

>MS.gene055443.t1

ATGTCAATAACACAAAACCTCACCATTTCAATCTCTTCTTCTTCATCTTCTTTCTTATCT

CCTTCCAATTTCAATTCCAGGAGTCAGGTTTCATTGCCTGTGAAGAGTGTCAGCATTTGT

AAATGCGTTGCTACACCCCAAGAAGCTGAGACTGCCTACAAGACAAGGGTCTCTCGCAAT

GAAAATTTGGGTAAACTTCAAGCTGGCTATCTCTTTCCTGAGATTGCTAGAAGAAGGTCT

GCACACTTGCTGAAGTACCCTGATGCAAAAATAATAAGCCTTGGGATTGGTGATACTACT

GAACCCATTCCTGAAGTCATAACTTCTGCATTGGCAAAGAAATCACATGCATTGTCAACC

TTAGAAGGATATAGTGGTTATGGAGCTGAACAGGGTGAAAAGCCATTAAGAAGTGCAATT

GCGTCAACATTTTACCCCGATCTTGGCATAGAAGATGATGATATATTTGTCTCAGATGGA

GCAAAGTGTGATATATCTCGTCTCCAGATTGTCTTCGGTTCGAATGTGAAAATGGCTGTG

CAGGATCCATCCTACCCGGCCTATGTCGACTCGAGTGTAATTATGGGCCAGACTGGTCTC

TACCAAAAGGATGTTCAAAAGTTCGCGAACATTGAATACATGAGGTGTAATCCAGAAAAT

GGTTTCTTTCCTGATTTGTCCTCTCTTTCTCGGCCAGATATTATTTTCTTCTGTTCTCCA

AACAATCCTACTGGTGCTGCAGCAACAAGGGAGCAACTGGTCCAACTAGTTCAGTTTGCT

AAGGACAATGGATCTATAATAGTATATGATTCAGCATACGCTATGTATATTTCTGGCGAC

AACCCCCGCTCCATCTTTGAAATTCCCGGAGCCAAAGAGGTTGCCATTGAAACTTCATCA

TTTAGCAAGTATGCTGGGTTCACTGGAGTTCGACTGGGTTGGACTGTGATTCCAAAGCAG

TTACTGTTTTCTGATGGATTTCCCGTGGCCAAGGACTTCAACCGTATTGTATGTACTTGT

TTCAATGGTGCATCAAATATTTCCCAGGCTGGTGGTCTCGCCTGCCTTTCACCAGACGGC

CTTAAGGCTATGCGCGGGGTTATTGGATTCTACAAAGAAAATACTGACATTATAGTGGAA

ACATTTGATTCTCTCGGGTTTAAAGTGTATGGGGGAAAAAGTGCACCATACGTGTGGGTC

CACTTCCCTGGGCAAAATTCATGGGATGTATTCAGTGAGATTCTGGAGAAGACACATGTG

GTTACAACACCTGGGAGTGGTTTTGGACCTGGTGGTGAAGGTTTTGTCAGGGTCAGTGCC

TTTGGTCACCGGGAAAATGTCTTGGAGGCCTGCAGAAGATTCAAGCAGCTATACAAGTGA

>MS.gene055445.t1

TGAAGATATTACCTGTTGTGAGAAATCAGCATTTATTGCTTTGTACTTTACTCATTTGCA

ATGCCGCCGCCATGGAGGCACTTCCTATTTTTCTTGATAGTCTTGTTACTGCTTGGGGTG

CTATCCTGATTTCAGTCACGTTGATTCTTCTCTTTGGTGAGATTATACCCCAATCAGTTT

GTTCTCGATATGGTTTGGCCATTGGTGCATCAGTCACTCCTTTTGTCCGCGTGCTTGTAT

GGATATGTTATCCAGTTGCTTTTCCAATTAGCAAGTTGTTAGACTATTTGCTGGGTCATC

GACATGAAGCCCTTTTCCGTAGAGCCGAGTTGAAAACACTAGTAGATTTGCATGGTAATG

AGGCTGGAAAAGGTGGGGAACTGACACATGATGAAACAACAATAATTGCCGGGGCACTTG

AACTCAGTGAGAAGACAGCCAGTGATGCTATGACTCCTATATCTGAAACATTTGCTATTG

ATATTAATTCGAAGCTTGATAGGGAACTGATGACTGAAATATTGGAGAAAGGACATAGCA

GAGTCCCAGTCTATTATGAGCAGTCTACTAACATTATTGGATTAATACTGATCAAGAACT

TGTTGACTATTCATCCAGAAGATGAATCGCCTGTAAAGAGTGTAACCATACGCAGGATTC

CAAGGGTTCCAGAAAGTATGCCGCTGTATGACATTTTGAATGAGTTTCAGAAGGGGCATA

GTCACATGGCCGTTGTTGTACGACAATGCGATAAAACCAAGCAACCATCTTCCAAAAATG

ATTCAAATGATTCTGTGAGAGAGGTGAAGGTGGATATTGATGGTGAAAAGCCTCTCCAAG

AGAAAGTCTTGAAACCCAAGATACCAATCCAAAAGTGGAAAAGCTTTCCAAATACGAACA

AGTCGAATAGGGGTTCTCGGAGCAGAAAATGGTCAAAAAATATGTACTCAGATATTTTAG

AGATAGATGGTAGTCCTCTTCCAAATATCCCTGAAGAAGAAGAAGCTGTTGGAATAATTA

CAATGGAGGATGTTATTGAAGAGCTTTTACAG

>MS.gene055447.t1

ATGGCGGTGGAATACACATGCTGCGAATCGGAGTTCTTCATCCACATATTGGTTATCATC

TTCTTGGTGGTCTTCGCCGGTTTAATGTCCGGACTCACCTTAGGTCTCATGTCTCTCAGT

CTCGTTGATCTTGAAGTTCTTGCTAAGTCTGGTACTCCTCACGATCGTAAACACGCCGTG

AAGATATTACCTGTTGTGAGAAATCAGCATTTATTGCTTTGTACTTTACTCATTTGCAAT

GCCGCCGCCATGGAGGCACTTCCTATTTTTCTTGATAGTCTTGTTACTGCTTGGGGTGCT

ATCCTGATTTCAGTCACGTTGATTCTTCTCTTTGGTGAGATTATACCCCAATCAGTTTGT

TCTCGATATGGTTTGGCCATTGGTGCATCAGTCACTCCTTTTGTCCGCGTGCTTGTATGG

ATATGTTATCCAGTTGCTTTTCCAATTAGCAAGTTGTTAGACTATTTGCTGGGTCATCGA

CATGAAGCCCTTTTCCGTAGAGCCGAGTTGAAAACACTAGTAGATTTGCATGGTAATGAG

GCTGGAAAAGGTGGGGAACTGACACATGATGAAACAACAATAATTGCCGGGGCACTTGAA

CTCAGTGAGAAGACAGCCAGTGATGCTATGACTCCTATATCTGAAACATTTGCTATTGAT

ATTAATTCGAAGCTTGATAGGGAACTGATGACTGAAATATTGGAGAAAGGACATAGCAGA

GTCCCAGTCTATTATGAGCAGTCTACTAACATTATTGGATTAATACTGATCAAGAACTTG

TTGACTATTCATCCAGAAGATGAATCGCCTGTAAAGAGTGTAACCATACGCAGGATTCCA

AGGGTTCCAGAAAGTATGCCGCTGTATGACATTTTGAATGAGTTTCAGAAGGGGCATAGT

CACATGGCCGTTGTTGTACGACAATGCGATAAAACCAAGCAACCATCTTCCAAAAATGAT

TCAAATGATTCTGTGAGAGAGGTGAAGGTGGATATTGATGGTGAAAAGCCTCTCCAAGAG

AAAGTCTTGAAACCCAAGATACCAATCCAAAAGTGGAAAAGCTTTCCAAATACGAACAAG

TCGAATAGGGGTTCTCGGAGCAGAAAATGGTCAAAAAATATGTACTCAGATATTTTAGAG

ATAGATGGTAGTCCTCTTCCAAATATCCCTGAAGAAGAAGAAGCTGTTGGAATAATTACA

ATGGAGGATGTTATTGAAGAGCTTTTACAGGAGGAGATATTTGATGAGACAGATCATCAT

TTTGAAGACTCATGA

>MS.gene055448.t1

GTACCTCCTCCAGGGAAAGGGACGCTTTATCTTAGGCCTTTGCTGATAGGAACAGGAGCT

GCATTAGGCTTGGCTCCATCACCTGAGTACACATTTCTCATTTATTGCTCCCCTGTTGGA

AATTACCACGAGGGAGGAAGACTAAACTTAAAAGTGGAGGATAAATTTCATCGATCAATA

GCTGGCAGTGGTGGAACAGGAGGAATCAAGAGTGTTACTAATTATGCCCCGATATATACT

GCAGTAACTGAAGCAAAAGCCAATGGATTTTCTGATGTCTTGTTCTTGGATTCAGCAACT

GGAAACAATATTGAGGAGGCTACTGCATGCAATATATTTGTTGTGAAGGAAAATGATATC

TTCACTCCGGCAATAGATGGATCTATTCTGCCTGGGGTCACACGAAAATCCATCATAGAC

ATCGCCATTGATTTGGGTTATAAGGTCATAGAACGTTCCATATCAGTGGAGGAAATGATG

AGTGCTGATGAAGTGTTCTGCACAGGAACTGCAGTGGTTGTTACCTCTGTTGCATCTGTA

ACATATAAGGAAACAAG

>MS.gene055446.t1

ATGTCAATAACACAAAACCTCACCATTTCAATCTCTTCTTCTTCATCTTCTTTCTTATCT

CCTTCCAATTTCAATTCCAGGAGTCAGGTTTCATTGCCTGTGAAGAGTGTCAGCATTTGT

AAATGCGTTGCTACACCCCAAGAAGCTGAGACTGCCTACAAGACAAGGGTCTCTCGCAAT

GAAAATTTGGGTAAACTTCAAGCTGGCTATCTCTTTCCTGAGATTGCTAGAAGAAGGTCT

GCACACTTGCTGAAGTACCCTGATGCAAAAATAATAAGCCTTGGGATTGGTGATACTACT

GAACCCATTCCTGAAGTCATAACTTCTGCATTGGCAAAGAAATCACATGCATTGTCAACC

TTAGAAGGATATAGTGGTTATGGAGCTGAACAGGGTGAAAAGCCATTAAGAAGTGCAATT

GCGTCAACATTTTACCCCGATCTTGGCATAGAAGATGATGATATATTTGTCTCAGATGGA

GCAAAGTGTGATATATCTCGTCTCCAGATTGTCTTCGGTTCGAATGTGAAAATGGCTGTG

CAGGATCCATCCTACCCGGCCTATGTCGACTCGAGTGTAATTATGGGCCAGACTGGTCTC

TACCAAAAGGATGTTCAAAAGTTCGCGAACATTGAATACATGAGGTGTAATCCAGAAAAT

GGTTTCTTTCCTGATTTGTCCTCTCTTTCTCGGCCAGATATTATTTTCTTCTGTTCTCCA

AACAATCCTACTGGTGCTGCAGCAACAAGGGAGCAACTGGTCCAACTAGTTCAGTTTGCT

AAGGACAATGGATCTATAATAGTATATGATTCAGCATACGCTATGTATATTTCTGGCGAC

AACCCCCGCTCCATCTTTGAAATTCCCGGAGCCAAAGAG

>MS.gene055449.t1

ATGTTCGTTTCTAAGATTTTGACTCTTCGTGATACCTCAACTGCATTGCAGGCTTTTGAT

CTTGACCGTCGAGGTAAGATTGAGCCTCAAGTCCTTAAAAATGTTTTAGACTATGTTTGC

TCCCATAATACCTACCTCCAGGAATTAGGAATCTCTGTCCATGGTGATAGTTCTCTCATT

CTGAGCTGCGTTTCTTCTTGTCGGGCTCTTACATCTCTTAAGCTTTCAGTTCACCCTAGA

GATAATAATGGTAGTGATACTGGAATATTATTTCCAAAATCTTTGAATTTACCTGCACTG

ACCAGCTTAGATCTAACGAATTTTGCCTTTTGCGGTGATGAAAACGGTTGTGCTGAGCCT

TTTTCGGTCTTTACCAAGTTGAATGGTTTGGTCATTCGTAGTTGTAAGGTTAAGGATGCA

CAAATCCTCAATATATCAAGTGAGACACTTGTAGATTTAGCAATGCATGATAACTCGCGT

GACTTTGCCAAAATCGAGTTATCTACTCCGAGTCTTTGTAACTTCACTTTTTGTGGTAGA

CCTGTTCAAAGAATACGTGGTAGCGGTATTTCTTCTGTTAAACATGTAAGTATTGAAGCT

GAAATGTATTCAGTAAGGGAGAAGTATCCTTGGATTCTATTCAACTGGCTGTTAGGCTTT

GCCAATGTAAAATCATTGAAAGTCACTTCAAGTACTCTTCAGATTCTCTCCTTAGTTCCT

AATTTATTGAAGTTTAAGATACCTTCTTTGTGTAAGTTGAAGTCACTGGAAATAGAACTG

GAATCACTTCAAATGGGATTACCCGACATACTGATGGAGTCCATGTTAAGGAAAGCTGCT

GCCAAGTCACTTAAGAAAGCCGCCAAGTTACGAAAAGCTTATGAAGAAGGTTGGAGACCA

CCGCTCATACCTAATGGAATGGTTGACTTCTTGCTACAGAACTCGCCTTCGGCGAAACTT

GAAATCTCGATGCGTTCAAATCCATCGAAAAAATCACTACTCAACGACGACAGCGAGAAA

ATGAGGCGCGCGTACGATTCTTCCAAGAAAATCAGGATTCAAGAAAAGATGAAAAAAATA

AGGCAATGTGAGACTGAAAATGAAGGAAGTGAAGAAAATGAAGACAAGCTAAGTGACTTA

CCTGAGTGTGTTATCCTTCACATTTTATCATTTTTGGACTCCAAACATGCCGTTCAAACC

TGTGTTTTGTCCACAAGATGGAAGCATCTTTGGAAACGTATTCCTACCCTTATATTACAT

TCCTCAAACTTTTCAACTGTCAAGAAATTTGCCATATTCGTGTCTAACATTTTGACTCTT

CGCGATAGCTCAACTTCGCTGCATGCTCTCAATCTTGATCGTCGTGGTGATATTGAACCT

CAACTCCTTAAAAAGATTTTAAATTATATATGCTCCCATAATACCCACCTCCACGAATTA

GGAATCTCTCTCCGTCGTGGAAGTGATCTAATTCTGAACTGTGTTTCTTCATGCCACACT

CTTACATCTCTTAAGCTTTCACTTGACCCTAGAAGTAGTCGTCGTTATTTTATTTCTGAA

GAAACATTATTTCCAAAATCTTTGAATTTGCCTTTGTTGACCAACTTAGATTTAACAAAT

TTAGTCTTTTGCGGTAGTGAAAGCGGTTGTGCTGATCCCTTTTTGGCCTTTCCGAAGTTG

AATAGTTTGGCCATTCGTTGTTGTAAGATAAAGGATGCTCAAATCCTCAACATATCAAGT

GAGACACTTGTCAATTTAGCTATACATTATCCTTTGGAAAAAATGAAGTTATCTTACCAT

GATACTTCATCCAAATTTGCCAAAATAGAGCTATCTACTCCAAGTCTTTGTACATTTACT

TTTATTGGTAGTCTTATTCAAAAAATATGCGGGAGCGGACTTTCCTCTGTTAAACAAGTA

AATATTGATGATTCACATCAGCTTTATGCTTCAGCGGGGAATGGTTTGATTCTACTCAGC

TGGCTGCTAGACTTTGCGAATGTAGAATCATTTAGAGTCACTTCAACTACTCTTCAGATT

CTCTCCATAGTTCCTGATTTATTGAAGGTTAAGCTCCATTCTTTATGTAACTTGAAGTCA

TTGGTAATAGAATTGATACCATTTCATGATGGATCTTCATCCCGCTCAATCGTAGATGCC

TTGTTAAAGAAAGATGCTGCCATGTCACGTAAAGAAGTTGCTAAGTTAAGAAAGACATTT

AAAGCTCGTTTGGAACCACCTGCCATACCTAATGGAATAGTGGACTTCTTGCGACAAAAC

TCACCGTCTGCGGAAGTTAACATCTTTACAGATTACCCGGATTGTTTTAATCTTAAGCAG

GTTGTAGAATCTATAAAGGGCGCTAAGATCATCAGCTATCGTTCCAAACTTGCCGCGTCT

GCCTCCTCCTCTGCCCCACCTGCTTCTGCTGCTGAATATGCTCCTCCCACTGCTCCTGCT

TCTGCCTCCGTGCCTGCTTCTGCCGCTGTGCCTACCTCTGCTGCACCTTCCAAACTTCAT

CTCTATATCGCTGAAAAGGATGATAAATCATCAACTGAAGATAAGAAGGAGAAGCGCCAA

TGTAACACCGACTCTCCACTTCTAGATAATGGGCAGTGA

>MS.gene055450.t1

AAAATGAGGCGCGCGTACGATTCTTCCAAGAAAATCAGGATTCAAGAAAAGATGAAAAAA

ATAAGGCAATGTGAGACTGAAAATGAAGGAAGTGAAGAAAATGAAGACAAGCTAAGTGAC

TTACCTGAGTGTGTTATCCTTCACATTTTATCATTTTTGGACTCCAAACATGCCGTTCAA

ACCTGTGTTTTGTCCACAAGATGGAAGCATCTTTGGAAACGTATTCCTACCCTTATATTA

CATTCCTCAAACTTTTCAACTGTCAAGAAATTTGCCATATTCGTGTCTAACATTTTGACT

CTTCGCGATAGCTCAACTTCGCTGCATGCTCTCAATCTTGATCGTCGTGGTGATATTGAA

CCTCAACTCCTTAAAAAGATTTTAAATTATATATGCTCCCATAATACCCACCTCCACGAA

TTAGGAATCTCTCTCCGTCGTGGAAGTGATCTAATTCTGAACTGTGTTTCTTCATGCCAC

ACTCTTACATCTCTTAAGCTTTCACTTGACCCTAGAAGTAGTCGTCGTTATTTTATTTCT

GAAGAAACATTATTTCCAAAATCTTTGAATTTGCCTTTGTTGACCAACTTAGATTTAACA

AATTTAGTCTTTTGCGGTAGTGAAAGCGGTTGTGCTGATCCCTTTTTGGCCTTTCCGAAG

TTGAATAGTTTGGCCATTCGTTGTTGTAAGATAAAGGATGCTCAAATCCTCAACATATCA

AGTGAGACACTTGTCAATTTAGCTATACATTATCCTTTGGAAAAAATGAAGTTATCTTAC

CATGATACTTCATCCAAATTTGCCAAAATAGAGCTATCTACTCCAAGTCTTTGTACATTT

ACTTTTATTGGTAGTCTTATTCAAAAAATATGCGGGAGCGGACTTTCCTCTGTTAAACAA

GTAAATATTGATGATTCACATCAGCTTTATGCTTCAGCGGGGAATGGTTTGATTCTACTC

AGCTGGCTGCTAGACTTTGCGAATGTAGAATCATTTAGAGTCACTTCAACTACTCTTCAG

ATTCTCTCCATAGTTCCTGATTTATTGAAGGTTAAGCTCCATTCTTTATGTAACTTGAAG

TCATTGGTAATAGAATTGATACCATTTCATGATGGATCTTCATCCCGCTCAATCGTAGAT

GCCTTGTTAAAGAAAGATGCTGCCATGTCACGTAAAGAAGTTGCTAAGTTAAGAAAGACA

TTTAAAGCTCGTTTGGAACCACCTGCCATACCTAATGGAATAGTGGACTTCTTGCGACAA

AACTCACCGTCTGCGGAAGTTAACATCTTTACAGATTACCCGGATTGTTTTAATCTTAAG

CAGGTTGTAGAATCTATAAAGGGCGCTAAGATCATCAGCTATCGTTCCAAACTTGCCGCG

TCTGCCTCCTCCTCTGCCCCACCTGCTTCTGCTGCTGAATATGCTCCTCCCACTGCTCCT

GCTTCTGCCTCCGTGCCTGCTTCTGCCGCTGTGCCTACCTCTGCTGCACCTTCCAAACTT

CATCTCTATATCGCTGAAAAG

>MS.gene055451.t1

ATGAGGCGCGCGTACGATTCTTCCAAGAAAATCAGGATTCAAGAAAAGATGAAAAAAATA

AGGCAATGTGAGACTGAAAATGAAGGAAGTGAAGAAAATGAAGACAAGCTAAGTGACTTA

CCTGAGTGTGTTATCCTTCACATTTTATCATTTTTGGACTCCAAACATGCCGTTCAAACC

TGTGTTTTGTCCACAAGATGGAAGCATCTTTGGAAACGTATTCCTACCCTTATATTACAT

TCCTCAAACTTTTCAACTGTCAAGAAATTTGCCATATTCGTGTCTAACATTTTGACTCTT

CGCGATAGCTCAACTTCGCTGCATGCTCTCAATCTTGATCGTCGTGGTGATATTGAACCT

CAACTCCTTAAAAAGATTTTAAATTATATATGCTCCCATAATACCCACCTCCACGAATTA

GGAATCTCTCTCCGTCGTGGAAGTGATCTAATTCTGAACTGTGTTTCTTCATGCCACACT

CTTACATCTCTTAAGCTTTCACTTGACCCTAGAAGTAGTCGTCGTTATTTTATTTCTGAA

GAAACATTATTTCCAAAATCTTTGAATTTGCCTTTGTTGACCAACTTAGATTTAACAAAT

TTAGTCTTTTGCGGTAGTGAAAGCGGTTGTGCTGATCCCTTTTTGGCCTTTCCGAAGTTG

AATAGTTTGGCCATTCGTTGTTGTAAGATAAAGGATGCTCAAATCCTCAACATATCAAGT

GAGACACTTGTCAATTTAGCTATACATTATCCTTTGGAAAAAATGAAGTTATCTTACCAT

GATACTTCATCCAAATTTGCCAAAATAGAGCTATCTACTCCAAGTCTTTGTACATTTACT

TTTATTGGTAGTCTTATTCAAAAAATATGCGGGAGCGGACTTTCCTCTGTTAAACAAGTA

AATATTGATGATTCACATCAGCTTTATGCTTCAGCGGGGAATGGTTTGATTCTACTCAGC

TGGCTGCTAGACTTTGCGAATGTAGAATCATTTAGAGTCACTTCAACTACTCTTCAGATT

CTCTCCATAGTTCCTGATTTATTGAAGGTTAAGCTCCATTCTTTATGTAACTTGAAGTCA

TTGGTAATAGAATTGATACCATTTCATGATGGATCTTCATCCCGCTCAATCGTAGATGCC

TTGTTAAAGAAAGATGCTGCCATGTCACGTAAAGAAGTTGCTAAGTTAAGAAAGACATTT

AAAGCTCGTTTGGAACCACCTGCCATACCTAATGGAATAGTGGACTTCTTGCGACAAAAC

TCACCGTCTGCGGAAGTTAACATCTTTACAGATTACCCGGATTGTTTTAATCTTAAGCAG

GTTGTAGAATCTATAAAGGGCGCTAAGATCATCAGCTATCGTTCCAAACTTGCCGCGTCT

GCCTCCTCCTCTGCCCCACCTGCTTCTGCTGCTGAATATGCTCCTCCCACTGCTCCTGCT

TCTGCCTCCGTGCCTGCTTCTGCCGCTGTGCCTACCTCTGCTGCACCTTCCAAACTTCAT

CTCTATATCGCTGAAAAGGATGATAAATCATCAACTGAAGATAAGAAGGAGAAGCGCCAA

TGTAACACCGACTCTCCACTTCTAGATAATGGGCAGTGA

>MS.gene055452.t1

ATGTTGTATGCATCAAGATTTTCCACTGTTAAGCAATTTGCTATGTTTGTGTCTAAGATT

TTGACTCTTCGTGATACCTCAACTGCGCTGCACGCTCTTGATCTTGACCGTCATGGTGAT

ATTGAGCCTCAACTCCTTAAAAAGATTTTAAACTATGTTTCATCTCATAATACCCACCTC

CATGAATTAGGAATCACTGTTAATGGTGATACTAGTCTCATTATGAGCTGTGTTTCTTCA

TGCCGGGCTCTTACATCTCTTAAGCTTGATGTTGCCTCTAGAGGTCGTCATAATATTGGA

CAAACATTATTTCCAAAATCTTTGAATTTGCCAACATTAACCAACTTATATCTAACAAAT

TTCGACTTTTGCGGTGATGAAGATGGTTGTGCTGAGCCTTTTTTGGCCTTTCCCAAGTTG

ACTACTTTGGTCATTCGTAGTTGTAAGGTAAGGGATGCACAAATCCTCAGGATATCAAGT

GAAACCGTTGTCAATTTTAAAATGCGCAATTATTCAGAAGACTTTGCAAAAATTGAGTTA

TGTGCTCCAAGTCTTTGTACCTTTACTTTTACTGGTACTCCTGTTCAGAAAATATGTGGG

AGTGGTCTTTCTGCTGTCAAACAAGTAAAGATTAAAGCAGATATGTTTTCAAGATGGGAT

GAGCCTCCTATGATTCTATTCGACTGGCTGCTAAACCTTTCCAATGTAAAATCATTGATG

GTCTCTTCAACTACTCTTCGGATTCTCTCATTAGTTCCTGATTTACTAGAGGTTAAGCTG

CCTTCTTTGTGTAACTTGAAGTCTATGGAAATAAAACTGGAAACCTGTAGTAATCAAATG

GCATTACCCAACCTGGTGAAAGAAACCATGTTAAAGAAAGCTGCTGCCAAGTCACGTAAA

GAAGCTGCCAAGTTAAGAAAGGCTTTTAAAGCACGTTTGGAACCACCTCCCATACCTGAT

GGAATAGTTGACTTCTTGCGACAAAACTCGCCATCCGCCGAAGTTAACATCACAACAAAT

TACCCGGGTTGTTTTAATCTTAAGCAGGTTGAAGAATCTATAAAGGGTGCAAAGGTTATC

AACTATCATTCGCAATTCGCCGTGCCTGCCTCCTCTGCTGCACCTGTTCATGCTGCTGAG

TCTGCTTCTGCCACCGTGCCTGCCTCGACATCACCTCCCAATCTTCATCTATGTTGCGCT

GAAAAG

>MS.gene068003.t1

CATTCCTGAGCACAGTGTGGATTGGAAGGAGACTCCAGAAGCGCATGTATTCAAGGCGGA

TCTTCCAGGAACGAAGAAGGAAGAAGTGAAGGTTGAAATTGAAGATGACAGAGTTCTTCA

GATTAGTGGAGAGAGGAATGTTGAGAAAGAAGATAAGAACGATCAATGGCATCGCGTGGA

GCGTAGCAGTGGGAAGTTCATGAGGAGGTTTAGATTGCCAGAGAATGCTAAGATGGATCA

AGTGAAGGCAAACATGGAGAATGGTGTTCTCACTGTAACTGTTCCTAAGGAAGAAGTGAA

GAAGCCTGAAGCTAAGACCATTGATATCTCTGATTAA

>MS.gene068004.t1

ATGCAGGTTCCTGATGTGGATGAGGTGTTTGATGTAATCACTCTGAGAAAGTTGATAAAG

ATTGAGCGCAGGAAAGCTAATGCGGCGTTTGCGGAGCTTGATAAGGAACGGACAGCGGCT

TCTTCATCTGCGGAAGAGGCAATGGCGATGATTCTCAGGCTTCAGAGTGAGAAGAGCTCG

GCTGAAATTCAAGCGAATCAATTCCGCAGAATGGCTGAACAGAAGTTAGAGTATGATGAT

GAAGTGATCGAGTCTTTGGAATGGACGATCTCGAGGCATGAATCTCATAGGAGTGTGTTG

GAAGAGCAGTTGAGGGTTTATAGAGAAGAACTCAAGCAATATCTTGGGGAGGATGGGATA

AACCAACTTGAAGCTGATGTTAGTAGAGATAGGAGTTTTGAGAATGAAGCAGTTGATTCT

GTAGTTAGCTCTTCTGAAAACGGATCACCGACCTTGTAA

>MS.gene068005.t1

ATGTCACTGATTCCAAGTTTATTCGGTATCAGAAGGACTAACATTTTCGATCCATTCTCC

GTCGATCTTTTCGATCCAGATTTTCCGTGCCGTGATTCACTTTCTGCTTCCTTCCCTGCA

AATTCAGCATTCCTGAGCACATGTGTGGATTGGAAGGAGACTCCAGAAGCGCATGTATTC

AAGGCGGATCTTCCAGGAACGAAGAAGGAAGAAGTGAAGGTTGAAATTGAAGATGACAGA

GTTCTTCAGATTAGTGGAGAGAGGAATGTTGAGAAAGAAGATAAGAACGATCAATGGCAT

CGCGTGGAGCGTAGCAGTGGGAAGTTCATGAGGAGGTTTAGATTGCCAGAGAATGCTAAG

ATGGATCAAGTGAAGGCAAACATGGAGAATGGTGTTCTCACTGTAACTGTTCCTAAGGAA

GAAGTGAAGAAGCCTGAAGCTAAGACCATTGATATCTCTGATTAA

>MS.gene068006.t1

ATGCAGGTTCCTGATGTGGATGAGGTGTTTGATGTAATCACTCTGAGAAAGTTGATAAAG

ATTGAGCGCAGGAAAGCTAATGCGGCGTTTGCGGAGCTTGATAAGGAACGGACAGCGGCT

TCTTCATCTGCGGAAGAGGCAATGGCGATGATTCTCAGGCTTCAGAGTGAGAAGAGCTCG

GCTGAAATTCAAGCGAATCAATTCCGCAGAATGGCTGAACAGAAGTTAGAGTATGATGAT

GAAGTGATCGAGTCTTTGGAATGGACGATCTCGAGGCATGAATCTCATAGGAGTGTGTTG

GAAGAGCAGTTGAGGGTTTATAGAGAAGAACTCAAGCAATATCTTGGGGAGGATGGGATA

AACCAACTTGAAGCTGATGTTAGTAGAGATAGGAGTTTTGAGAATGAAGCAGTTGATTCT

GTAGTTAGCTCTTCTGAAAACGGATCACCGACCTT

>MS.gene068029.t1

ATGGAGGAGGTGGAGAAGGTGAAGGCTGAGGCAGTGCAAATGATAGGAATGTTTCAAGTA

CTACCAAGGCTCGTAGTGTTCGATTTAGATTATACTCTTTGGCCTTTCTACTGTGAGTGC

CGCTCCAAGCACGACACACCTTCTTTGTTTCCACATTCCAGAGGCATCTTGAGTGCACTC

AAAGATGAAGGAATTGATGCTGCCATTGCTTCCAAATCACCAACTCCACACATAGCAACA

ACGTATCTTGACAAACTCAAAATCACCTCAATGTTTGTCGCCCAGGAAATATTTTACAGC

TCGACACACAAAACAGAACATTTTCAGAAAATTCATTCAAAGACCGGTGTCCCCTACAAC

TCTATGCTCTTTTTCGACGATGATAACAATAACATGAAACCGGTTTCAAATTTGGGAGTT

ACAAGTATTTTGGTTAACAATGGCTTAAATCTAGGAGTATTTAGAGAAGGGCTCACAAGA

TTTTCGCAAAACTGGGATGCCTCAAAGAACAAGCAGAAAAGGCGCAAGTAA

>MS.gene068052.t1

ATGAGTTCTCTTCTTCAACCACAAGGTAGAGATTGCGAGCAAGACAATAGCTCTAAACTG

TTGCCAACAATTGTGAAATTTGAGTCCACATTTGAGGTGGAAGCCACCAAATCAGTAGAA

AATGATAAGGAGGAGGGTATTTTCTTGACATGGGGAGGACCTTTGGGTGACTGTTACAAA

TGGAAAAATGGAAGAAAACCAATACTTCAAGGTCTAAGAGGCTATGAAAACCAGGACAAC

TCTTACTTAGCCATAATAGGTCGTTCTGGTTGTGGCAAGTCCACTCTTCTTGATGCTTTA

GAAGGCTGA

>MS.gene068041.t1

ATGAGCAATGGCAAGTACAAAAGTGTAGTACATAGAGCAGCTGTGAGCAATGGAGCTACA

AGAATGTCATTAGCTACGGTTATTGCGCCATCCTTGGACACCGTGGTTGAACCAGCTTCA

GAACTACTAGACAATGAAAGCAATCCAGCAGCATATGTTGGGATGAAACATATAGATTAC

ATGAAACTTCAACGAACCAACCAGCTTTACGGGAAGTCTGTGCTAAACAAAGTGAAAATA

TGA

>MS.gene068046.t1

ATGTTTTCGTTACCAATGACAATTGGCATGGTGATTTTTACATTGAGATATTTCTCTGGA

CCTGATGTGCCGAAATATGTGTTGTTCACCGTTGCTTATACTTGGTTCTGCTCCATCTCC

ATCATCATCCTTGTCCCTGCTGACATATGGACGACGCTAAATGATGCTGTAAATGTAACA

ATTTCCTTCTTATGGAGTTTATCATATTGGAGTACCTTCTTACTTACATGGGCTGTGGTT

CCCCTTCTTCAGGGTTATGAAGATGCTGGAGACTTTACAGTGAAAGCAAGATTAAGGACA

AGCTTACATGGAAACCTTGTTTTCTATCTGTCTCTTGGTTCTGTGGCTCTTTTTGGATTG

ATATTACTAATATCTCTGAACAAATTTTGGAGTGGTAGTGTAAGGGGTTTTGCCATGGCT

TGTTCTAACACATTTGGACTTGTTACTGGTGCATTTCTACTTGGATTTGGCATGAGTGAA

ATTCCAAAAGGCATTTGGTTAAATGCAGATTGGACCATTCAGCAAAAATTTCTTTCCCAC

AAAGTTGCAAAAATGGCTGTCAAATTAGATGATGCTCATCAAGATTTTTCAAATGCTATT

GTTATCACACAGGCAACATCAAAGCAAATGTCCAAGCGGGATTCTTTGAGACCTTACATG

AATATTATTGACAAAATGTTGGTTCAAATGTTGAACGAAGACCCTTCCTTCAAACCACAG

GGTGGGAGACTAGGAGAAAGTGACATGGATTATGATACGGATGAAAAATCAATGGCATCA

CTAAGACGTCGTCTTAGAAGAGCTCGTGAGCAGTATTATCGGTACAGAAGCGAATATACA

AAATTTGTCCTAGAAGCCCTCGAGCTGGAGGATACCGTAAAGAACTATGATCGTCGTGAT

TCTACAGGATGGAAATATATTTCATGCTTGAGGCCTGAACGAATAGGCAAAGTAGGTGCA

GTTTTGGATACAATTGAGTTTCTATGGCGATGTATATTAAAGAAACAACTTGAGAAATCA

TTGGCTGTTATATTGGGCTTCATGTCATTTGCTATTCTATTAGCAGAGGCTACCATACTA

CCCAGTGGAGTTGACTTGTCTCTTTTCTCTATCCTAGTACATGCTGCAGGACATACAGAA

GTGCTTGTGCAGTTAGCTGCTTTCGTCCCTTTGATGTATATGTGTGTTTGTACATATTAT

TCCTTGTTTAAAATGGGAATGTTTATGTTTTACTCACTGACACCAAGACAAACAAGCTCC

GTAAGCTTGCTTATGATATGCTCGATGGTGGCAAGATATGCTGCACCTATTTCATACAAC

TTTCTCAATCTCATCAATATTGGTGGGGATAGAAAAACCGTTTTTGAAAAAAAAATGGGA

AACATTGATGAAGCTGTTCCCTTTTTTGGGAAAGGATTCAACAAAATTTACCCTCTTATC

ATGGTTATATACACTTCACTAATAGCAGGCAATTTCTTTAACCGCGTCATCAACTATTGC

GGGAACTGGAAAATATTCAAGTTTCGTGATGATGCAGAAGATATGGATGGATTTGATCCA

TCTGGAGTAATAATTTTGCAGAAAGAGCGTTCTCTTCTTCAGCAAGGGCACAAAGTTGGC

GAACTTGTGTTCCCTTTAGCTAGGAGTTTTAGCATGAGTATGGATGTTGAGTCTGTCAAC

AGGGCCAAGGCACTGGATGAGAGTGTCGCAAGCGAAGATAAAACAACCATCATGGTAGAG

ACCAAAAATGAAGAAGCTGACATGAGCAGAAAAATCGGTGGCAGAAAGTATTCAGCCTTA

AGGACAAACCTGAATGAAGAGGGGTCAAGTAAAGACTTCGCCCTAGAAAGAAATTCTTCC

TCTTTGACAAATGATTCTCATACGGACACGAGTTCAGCACCATCTTCTGTGATAGCCTCA

AAATGGGAATCAATGATGCATGGATTTAAAAATTTAAAATCTAACATTGACTCCAATAAA

TTCCTTCCACTTAGTAATAATACTTATACATCGTCTCTAAACTCAACCTCTTCATTTGAA

TCTCTTGATGATATATTTGAGAGATTGAAACGTCCGCCATCAGAACACAAAGATTCTGGT

GGCGAATAA

>MS.gene068026.t1

ATGGCTTCTTCAATTTCATCTCTCTCTACTCCACACCTCACTTTCCCAAAACAAAACCTC

TCTACTACCTTCCCTCTCAAACGCCCTTCCCTTCTTCACTTCCCTTCTCAACCGTTATGC

CTCTGTCTCAACTCCGTCTCCGACGACAACCACAACAGTACAAACGATGGCGACGGTAAC

AACCGCCGGTGGGATTCGATGCTCAGCGAATTCGTAACCGGCGCAATTAAACAATTCGAT

TCTTATATGAACTTGCTTAGGAGAGGCCGTGCTGCTGATAAAGAAGACGGTGATGTTCAC

GATGATGATTGGGATTGGAATCGCTGGCGTCAGCATTTCGATCAGGTTGATGACCAGGAA

CGTCTTCTCATTATCCTCAAGTCTCAATTACGTCATGCTGTATATGTGGAGGATTATGAA

GAAGCTGCCAAGCTTAAGGTGGCGATCGCAGCTGCAGCTAACAATGACAGTGTTGGGAAA

GTGATAACTCTTCTCAAAAGAGCCATAAAAGAAGAGCGGTACAATGATGCAGCTTTTTTA

AGAGATAAAGCGGGTGCTGGACTTGTGGGTTGGTGGGCTGGTATTTCAAAAGATGTGAAC

GATCCACATGGTCTAATTATTCGCATAACTCCAGAGCATGGAAGATACGTGGCAAGGAGT

TATAGTCCTAGGCAACTTGCAACATCTGCTGCTGGTGTTCCTCTATTTGAGTTTTTTCTT

ACAATGGATAAAAAAGGTGACTTTAAGTCGCAGGCTGTGTACCTAAAGCGAAAAGGGTCC

TACCACGGTCCCCCAACAACGTCCTCTAAACCATTGGATGCTAGTGGGAGATCGAGTTCA

ATGGAATCTACAGAAGACAGAAGTGAGCTGTTTGTTGTGAGTACTGAAGATCCAGAAAGT

GGTGATGATAGGAATGATGGCTCTGACCCTGCTGAGGGAATACCTGGATTTCAGAATGTC

TTGAAAGATATGATTCCTGGTGTGAAGGTGAAGATTTTCAAAGTGATAACTCCAGAGAAA

GTAGACAAGGATCTAATGTCTAAGGTGATTGAAGAATTATTTGAGGAAGAAGAGAGTGAG

GATGAAGATGAAAATGGAGAAGATGACGGCGACGAAGACGACGGCGATGAAGATGACAGT

GAAGATGAAGACAAGGAAAATGATACAGAAATTCTGGATCTGGAAGACATTAAGTTGGAA

ACTGATCAAGAGGGAGATGATGGGATTGTGATAAATAGTGATCTTGGAACTTTTGCACGT

GAAGAACAGAATGAAATTGCTGTCAAAGTTGTCATTGGTGGTCTTGTGCAGAAACTTTCC

AGCAATTTATCCCCTAGAGATTTGCTTCGAGTTCCTGCTAAGCTGGAGATCAAGGAGCGT

CGTTCATTTTCCTTTACTGTTGAAAACGAAGTCAATCAGCTGGATGGTCCTGACAAAGGG

AAATCTTCATCAGATAAATCAATTAAGTTTCAAGGTCGTCGCAGAGTTGATAATGTTATT

TCTGATCTTGCAAAATTCATTGGCAAGGATAAAGTACCTGCAAAGGTGCTGAAAGAGGTA

GGAGAATTGATAAGTCTCACTCTAAGCCAGGCTCAGAATCATCAACCATTATCTGGGTCA

ACGATTTTCAATCGCATTGAAATACCAACTTCATTTGATCCCTTAAATGGCTTATATATT

GGTGCATACGGTGTTTACTCTTCTGAAGTTATTCAAATGAGACGTAGATATGGTCAATGG

CAAGAGGACGGTAGGGCGAAGGAGGCTTCAGATCTCGAGTTTTATGAGTATGTAGAAGCC

TTGAAAATAACCGGGGATCCTTATGTACCAGCTGGCCAGGTGGCATTTCGTGCAAAAGTT

GGAAAGGGGTACCAACTTCCACATAAAGGCATAATTCCTGAAGAATTTGGAGTGATTGCT

CGCTATAAAGGTGAAGGAAGATTGGCAGAGCCAGGGTTTCAGAATCCTCGATGGGTTGAC

GGCGAACTGGTGATTCTTGATGGAAAGCACATAAAAGCAGGGCCTGTTGTTGGATTTGTG

TATTGGGCTCCTGAGTATCATTTTTTGGTCTTCTTCAATCGGCTTAGGCTTCAACAGTAG

>MS.gene068035.t1

ATGGCCTCAACCAAATCCTTCATTGCAGCTTTACTTCTTGTTGTCACAATGTCAAGCATG

AGCCTAGAAGCTCGCCATCTATTGCAAACAACAACACAACCAAATTTACCTACCATTCCC

ACTTTACCAAAACCAACTACATTACCACCTTTGCCTTCAATTCCAAATATGCCTCAAGGA

AATCTTCCTCCATTGCCTACTATCCCTTCTATGCCTAAACTCACTATGCCACCACTTCCA

AGTCTTCCTACCAATATTCCAACAATTCCATCTCTCAACATTCCACCATTGCCAGCAGCC

ACTTCACTTCCCAACCTTCCTTCAATCCCAACCACTTTCCCCTCCTTCCCATTTTTTTCC

CCACCACCTTCAACTTCTAGCCCTTAA

>MS.gene068033.t1

ATGAATGGCTATCATGCCTGTACGAGGGTGGGCACTGAGAACGAGAGGGTTTCAGTTGTC

AATGCTGTGCCATCGATGCAAGATGGCTACATGTACGGAAATCAGTTTACGAGAGAGGGG

AATATTATGACTAGAAAGAAGATACAGATCAAGAAAATCGACAATATCAGTTCGAGGCAA

GTGACTTTCTCTAAGAGGAGGAAAGGGCTTTTCAAGAAAGCTCAGGAGCTTTCAACCCTT

TGTGATGCAGACATTGCTCTTATGGTTTTTTCTGCAACTAGTAAGCTCTTTGAGTATGCT

AGTTCAAGTATGGAACAAGTAATTGAAAGGCGTAATGGATATTCAGCAAATCACAGATTA

TTGGACCATCCATCTACTGATCAGCTTCAGGTTGAGAGCGACTCTACTTGCGACACACTG

CGCAAGAAATTAGAAGATAAATCTCGTGAACTGAGGCAATTGAATGGAGAAGATCTGCAA

GAATTGACAGTAGAAGAACTCCAGAAACTAGAGGCGCTTCTTAAAAGAAGTTTGTCTAGT

GTTTCCAAAATAAAGGATGAAATGTTTATGCGAGACATCGACACCCTTAAGAGAAAGGAA

GTGGAACTGATCGAAGAAAACCGAAGATTGAAACATGTGGTACCAGACCTTATAAATGGA

CGATGGCGACAATCATTGGAATCTGTTATTAGGGATTCATCTTTTTCTCCTGAAGACGAC

GATGGCAGTGACACTTCTCTCAAGTTGGGGTTACCATTTCTTAAATAA

>MS.gene068028.t1

ATGGAGAAGTCTCAAAACGGAGAAGAAAATGCACAACTCAACCTTTCCAACGATGGTTCT

ATTTGTGGCTACGATTCCCTTCATCATCTTCTCAAGGATAATCTTAAACCGCATCATTTC

CAGGAAGTCAATCGTTTGCTTACCGGGCTTAATTGTGGAAAAGTGCTTGAAACAATTGCT

CTCCCAGAATCTGCTACAGCTCTCTCTGTCAAACATGGTTTTGATGCCCAGGCTTTCTGC

TTTTGTGCTTATAAAGAACAATTAAGAGAACCTCGAGTTGTGAGGGTTGGTTTGATTCAA

AACTCTATTGCCATCCCAACAACTGCTCACTTTGTGGACCAAAAAAAGGCACTTTTTGAG

AAACTAGAGCCAATTATTGATGCTGCTGGTTCATCGGGGGTCAACATACTATGCTTGCAA

GAAGCATGGATGATGCCATTTGCCTTTTGTACACGGGAGAAAAGGTGGTGTGAATTTGCA

GAACCTGCTGATGGAGAATCAACAAAATTTTTGCAAAGCTTTGCAAAGAAGTATAACATG

GTCATTATAAGCCCAATTCTTGAGAGAGATGTGAACCATGGAGAGGTTATGTGGAACACT

GCCGTCGTAATTGGGAATCACGGCAATATAATTGGAATACACAGGAAGAACCATATTCCA

AGAGTTGGGGACTTCAATGAGAGCACATATTATATGGAAGGAAATACTGGTCACCCTGTA

TTTGAAACAGCATTTGGAAAGATTGCCATTAACATATGTTATGGTAGGCACCATCCTTTA

AATTGGCTAGCTTTTGGCTTAAATGGTGCGGAAATTGTTTTCAACCCTGCTGCCACCGTT

GGTGAACTCAGTGAACCTATGTGGCCAATAGAGGCACGAAATGCTGCAATAGCAAATAGT

TACTTTGTTGCTTCAATCAACCGGGTTGGGACTGAGACATTCCCCAATCCATTTACATCT

GGTGATGGCAAACCAGCACACGCAGATTTTGGTCATTTTTATGGATCAAGTTATATTTCA

GCACCAGATGCATCCTGCACACCATCTTTATCACGTAACAGGGATGGCTTATTAGTAACA

GACATGGATCTTAACTTGTGCAGGCAATATAAAGACAAATGGGGATTCCGAATGACTGCA

CGATACGAGTTGTATGCGGAGACACTTGCTCAATATGTGAAACCAGAATTTGAGCCTCAA

GTCATCCGTGACCCCTTACTTCATAAGAGATCTTAA

>MS.gene068011.t1

ATGGCTTCTTCATTGTTGCTCAAACTCGTTTTCAAATCCCTTCATCACTTCGCATGGCAT

CTTCTTGCTCTGGTGTATCCTATGTGTGCTTCCATACAAGCAATCGAGACCGATTCTGAT

GCAGAAATTAAGAATATAATCTCATATTGGATACTTCTTTCATTAATTTACCTATTTGAG

TATGCTTTTATGAGCCTTCTTCTATGGTTTCACTTCTGGCCGTACATTAAGCTAATGATC

ATCTTCTGTCTCATCATACCGGACTTTGGACGAGCTTCTTATGTTTATAATAACCTCATT

CGCCCCATGAAACTGAAAATAGTCACATGGAGGTTAAATAACTACTGGAGGAAGTGTTTT

GCTGAGAAAGATGACTTTTTAATGCATGCAGAGAGATATATGAAAGAGAACGGAACTGAA

GCCTTAGAGAAACTCCTTGCTAGCAAGAGCACAATGTGTAGGCCTGATGCAGAAGTGGCA

AATGAAATCATAGCTACTGATAATAAAGAAATGCTAAAGTCAAATGGAGAAAGACTCCAA

ATTGAGCACAAAGACATCAAAGACTCGGACGCTATTGAGAAAAAAGAACTTCCTGCTTTC

AAGCAAGAAGTACTTGGTCTCTATGCTAATACTCGGACGTTGCAGGATATTCCTGTCTTG

CCGAAAATTGGGCCAAGTCAAAATGCACCATTAGCCACAGTGGAAACCAAAGTAACAGCA

GAAAGAAACAGCGCTGGTGGAGAGGTTCCTCAGAGTTCTACTTCTGCACAGAAGGAAGTG

CAGAATGAGTGGACTTGTGCTTTATGTTTGGTAACAACATCGAGTGAGAAAATCTTGAAT

TCCCACCTGAGTGGAAAGAAACACAGGGCTGCTCTTCAGAGGCAAAAGGATGCAGAGGTG

ACAAATGAAATCATAGCTACTGATAATAAAGAAATACTAAAGACAAATGCAGAAAGGATC

CAAACAGAACACAAAGACATCAAAGATTTGGAGGCGGTTGAGAAAAAAGAAATTCATGAA

ACCAAGCAGGATATTCCTGTCATACCTAAAATTGGGCCAAGTCAAATTGGATCGTCAGCC

AAAATGGAAACCAAAGGAACAGCAGAGGGGGACAGGGCTGGTGTAGAGGTTCCTCAGAGT

TCTTCTACACTGAAGGATGTGCAGAAAGACTGGAGTTGTGCTTTAGGTTTGGCGACAAAT

GGAGATAGACTCCAAACAGAACACAGAGACATCAAAGATTTGGAGGCTATTGAGGAAAAA

GAAATTCCTGCAACCAAACAAGATATCCCTATCATGCCTAAAATTTTGCCAAGTCAAGTG

CATCATCAGCCACAGTGGAAACCAAAGGAACAGCAGAGTGACACAGTTGGTGGAGAGGTT

CCACAGAGTTCTACTATTGCGTTAAAGGAAGTGCAGAAAGAGTGGGCATGTGCTTTATGT

TTGGTAACAGTTCCATGTGAGAAAACATTGAATTCTCATCTTAATGGGAGGAAACACAGG

GCTGCTTGTGAAGCACCTTTGAAAGCAAAGAAGCTAAAAATCTACAAGGCCAAAGAGGAG

GTGAAACAGGAGAATTTTAGCAATAAGTTAAATTCTGAAGGTGGTATTCTTAACAACAGG

TTGAAGGGACCAGTAAGGATGCAAAAGCTCCATTTCATAGGACCCACACCTTCTCCACAA

GCTCTCAGTGGACCAACACCTTTCAGCGGACCAACATCATCTCCAATAAGAGTTCCATTT

TCATATCATAGAGGACCAACACCTTTCAGAGGACCAGCGCCTTTTGGAGGACCAACGCCA

CCTCCAATAAGAGTTCCATTTTCATATCATGGATCAGATGGATCATGGGCACGTCAACCA

GAGTTCACGTATTTCATTCCAACTCCTCCTAGTCCTCCACAACTCCTCCACTACGCCGCC

ACCGTCTCCGCTATTCTTCCTCGTTTCTATTTCAATAGTGTTGCATCGTTCTACTCCCTT

TTATTCGTTTCAACGGCGCCCCGCCACGGCGCCGCTACACCTCCAATACGGACTCCGAAA

TGTATCGTTGCTTTTCTGACGAGAGAAAGAGTGATCATGTGGTGTTTGAAATCCGAATCT

GCTCCGATTTCCGATCGACACAATCGCTTCTACGAATCGCCGTCGTTTTTGCCATTTCCC

CTTTTCAAAGATTTGGGAACAGATTTTTGTGTCGGAGAAGAAGAGAGAAGAGGGAGAGTA

ACGTCGAAACTGAGAGAGAAAGGGATGGAAGAGCATAATGGAGTATGGAGCATCATAGAG

CACTGGCCTCTTTTTGCTCTGGTGTATCCTATGTGTGCTTCCGTACAAGCAATTGAAACT

GACTCATATGCAGAAACCAAGGATTTGATCTCATATTGGATACTTCTTTCCTTAATTTAC

CTCTTTGAATATGCTTTTATGAGCCTTCTTCTACGGTTTCACCTCTGGCCATACGTTAAG

CTAATGATCATCTTCTGGCTCAGCATACCGGACTTTGGACGAGCTTCTTATATTTATAAT

AAGCTTATTAGTTCCATGAAACCGCAAGTAGTCACATGGAGGTTAAACAACTACTGGAGG

AAGTGGTTTTGTAAGAAAGATAAATTTTTAATGCAGGCAGAGAAATATATTAAAGAGAAT

GGAACTGAAGCCTTAGAGAAACTCATTGCCAACAAGAACACAATGTGTAGGCCTGATGCA

GAAGCGACAAATGAAATTGTGGCCACTGATAATAAAGAAATGCTAAAGACAAATGGAGCA

AGACTCCAAATTGAGCACAAAGATATAAAAGATTTGGAGGCAATTGAGAAAAAAGAAATT

CCTGCTACCAAGCAAACAAAGCATGAGTCCTTAATGAGGGAGCTTCTTGTGCAGACAAGT

GGAGAAAGGATCCTAACAGAACACAAAGACATCAAAGATCTCGAGGCTGCTGAGAAAAAA

GAAATTCATGAAACCAAGCAGGATATTCCTGTCGTACCTGAAATTGGGCCAAGTCAAAAT

GAATCATCAACCACAGTGGAAACCAAAGGAACAGTAGAGGGGGACAGGGCTGGTGGAGAG

ATTCCTCGGAGTTCTTCTACGCAGAAGGATTTGCAGAAAGACCGGACTTGTGATTTATGT

TTCACAACAGCAGATGAAATCTTGAATGCCCGCTTCAGTGGAAGGAAACACAGTGCTGCT

CTGATGAAGCAAAAGGGTGCAGAAGCGATAAATGAAATCACAACTACTGATGATAATAAA

GAAATACTAAAGGTGACAAATGGAGATAGACTCCAAACAGAACACAGAGACATCAAAGAT

TTGGAGGCTATTGAGGCAAAAGAAATTCCTGCAACCAAACAAGATGTCCCTATCATGCCT

AAAATTTTGCCAAGTCAAATGCATCATCAGCCACAGTGGAAACCAAAGGAACAGCAGAGT

GACACAGTTGGTGGAGAGCTTCCAGAGAGTTCTACTATTGCGTTAAAGGAAGTGCAGAAA

GAGTGGGCCATGCCTAAAATTTTGCCAAGTCAAATGCATCATCGGCCACAGTGGAAACCA

AAGGAACAGCAGAGTGACACAGTTGGTGGAGAGGTTCCAGAGAGTTCTACTATTGCGTTA

AAGGAAGTGCAGAAAGAGTGGGCCATGCCTAAAATTTTGCCAAGTCAAATGCATCATCAG

CCACAGTGGAAACCAAAGCAACAGCAGAGTGACACAGTTGGTGGAGAGGTTCCAGAGAGT

TCTACTATTGCATTAAAGGAACTGCAGAAAGAGTGGGTCATGCCTAAAATTTTGCCAAGT

CAAATGCATCATCAGCCACAGTGGAAATCAAAGGAACAGCAGAGTGACACAGTTGGTGGA

GAGGTTCCAGAGAGTTCTACTATTGCGTTAAAGGAAGTGCAGAAAGAGTGGGCCATGCCT

AAAATTTTGCCAAGTCAAATGCATCATCAGCCACAGTGGAAACCAAAGGAACAGCAGAGT

GACACAGTTGGTGGAGAGGTTACAGAGAGTTCTACTATTGCGTTAAAGGAAGTGCAGAAA

GAGTGGGCCATGCCTAAAATTTTGCCAAGTCAAATGCATCATCAGCCACAGTGGATACCA

AAGGAACAGCAGAGTGACACAGTTGGTGGAGAGGTTCCAGAGAGTTCTACTATTGCGTTA

AAGGAAGTGCAGAAAGAGTGGGCATGTGCTTTATGTTTGGTAACAGTTCCATGTGAGAAA

TCATTGAATTCCCATCTTAATGGGAGGAAACACAGGGCTGCTTGTGAAGCAGCTTTAAAA

GCAAAGAAGCTAAAAATCTACAAGGCCAAAGAGGAGGTGAAACAGGAGAATTTTAACAAT

AAGTTAAATTCTAATGTTGAGAATGGAGGTGGTATTGTTAACAACAGGTTGAAGGGACCA

GTAAGGATGCAAAAACTCCATTTCATAGGACCCACACCTTCTCCACAAGCTCGCAGTGGA

CCAACACCTTTCAGCGGACCAACATCATCTCCAATAAGAGTTCCATTTTCATATCATAGA

GGACCAACACCTTTCAGAGGACCAGCGCCTTTTGGAGGACCAACGCCACCTCCAACAAGA

GTTCCTTTTTCATATCATGAATCAGATGGATCATGGGCACGTCAACCAGAGTTCACGTAT

TTCATTCCGACTCCTCCTAGGTTGCCACCTCTGGATCCAAAAAGTTGTTGA

>MS.gene068009.t1

ATGTCGCAGCTTTCTATTCAACGAGCAAAGAAGTTGAAGTTTTTGATGCCTCAGATCTCT

GCTTTCGGAACCTCCCATTTCTCAACAGCGGCTCAACCATCTTCCACTGATCGCTATAAA

AGGGTTCCCAATCTTATTGGGGGAAGGTTTCTTGACTCTAAATCATCAAATTTCATCCAT

GTTTTAAACCCTGCGACACAAGAAGTTGTTTCGCAAGTTCCATTGACTACGGATGATGAG

TTTAAAGCTGCTGTATCTGCAGCTAAGAAGGCATTTCCGTCATGGCGTAACACTCCAGTT

ACGACGCGCCAACGTGTTATGTTGAAGCTCCAGGAGCTTATACGCAGAGATATGGATAAA

CTTGCTCTTAATATAACCACCGAACAAGGAAAGACACTGAAGGATGCTCAAGGAGATGTG

TTTCGTGGTTTAGAGGTGGTGGAACATGCTTGTGGAATGGGAACTCTACAAATGGGGGAG

TATGTTTCAAATGTTTCACATGGAGTCGACACATACAGTGTCAGAGAACCAATTGGTGTT

TGTGCTGGGATTTGTCCTTTCAACTTTCCTGCAATGATTCCATTGTGGATGTTTCCCGTG

GCTGTTACTTGTGGCAATACATTTGTTCTAAAACCATCAGAGAAAGACCCAGGTGCCTCT

ATAATGCTTGCAGAATTGGCACTGGAAGCTGGTTTGCCTGAGGGTGTCTTAAATGTTGTT

CATGGAACCCATGATGTTGTGAATGCCATTTGTGATGATGACGATATTAAAGCCATATCA

TTTGTTGGCTCCAATGTTGCTGGAATGCACATATATTCAAGAGCAGCAGCTAAAGGGAAG

CGTGTTCAGTCTAATATGGGGGCCAAAAATCATGCAATCGTCATGCCAGACGCAAATATT

GATGCTACTGTAAATGCTTTAGTTGCTGCTGGTTTTGGAGCTGCTGGGCAAAGGTGCATG

GCTCTCAGCACAGTTGTTTTTGTGGGAGGTTCACAAGCATGGGCAACTAAAATCATAGAG

CGTGCCAAAGCTCTTAAAGTAAACGCTGGAACGGAACCTGATGCAGATCTTGGTCCAGTT

ATCAGCAAACAGGCAAAGGAGCGAGTGCACAGATTAGTTCAATCTGGGGTCGACAGTGGT

GCCAGACTACTGCTTGATGGAAGAAATATAGTGGTCCCAGGATATGAATCTGGCAATTTT

GTTGGCCCCACTATCTTATCAAATATCACTGCCGACATGGAGTGCTACAAGGAGGAAATT

TTTGGCCCAGTTCTTCTTTTCATGGAGGCTGATAGCTTGGAAGATGCCATAAGCATTATC

AACAAAAACAAGTATGGAAATGGTGCTTCTATATTTACTACTTCTGGTGTTGCTGCTAGG

AAATTTCAGACTGAGATTGAGGCTGGGCAGGTTGGCATCAACGTTCCTATTCCAGTTCCT

TTGCCATTTTTCTCATTCACTGGCAACAAGGCGTCATTTGCTGGCGATCTCAATTTCTAT

GGCAAAGCAGGGGTTAACTTTTATACACAGATCAAAACAATAACTCAGCAGTGGAAGGAT

TCAGTTGGCAGTAGCAAGATTAACATGGCAATGCCAACCTCTCAAAAATCTTGA

>MS.gene068018.t1

ATGAATGATTTGGTGTCTGAGTACCAGCAATACCAGGATATGCTACTGCTGATAACGAAG

AGTATGAGGAGGAGGCAGAGGAGATTGGTGTTTGAGGCCTCTCTGAGTCAGCATCTTTGC

GCTTGGGGCAATGACGCAGCTAATGAGGTTCTAACCGAGGCGCGTCTATTGCTGCTCCCT

CTGGGCGCCTCCCTCTTCTCACCGAACCACCACCGGACCACCGTCATTTCACCACTGGAT

CACCGGCTACCAAATTCAATCAATTGGAAGAGTATTGAAGCATGGTTCAAAATTGAAGGA

TCGCTGAAAGACAAGAAGCTTGAAGGAGGAAAAGTAGAAGCTGCAGGCTTTGGGCATCAA

GAGAAGCTAATACAGGAGTTTTTGTCTCTATTTGCTTCTGTTTCGGTGTCCTTGACTGTG

CAGCCTCTGAGTTTCTCTTCATCAAATATGGATGAGAACATGAAACATTTTCAAAAGAAA

TTAACAAAGTTGGAACTCGAAGCTGAGCATCTTCTTTTAGCCCGACATCAGCTGGTTGAG

AATGATAAGTTGAGGAATGGGAACAGAGAAGCACTTACTGCGCTAAGGAAAAAGGCTCGG

ACAACAACTACTAGTGTTCCATCTCCTTTTGGATCAATAATGAAGGGAGTTTCAGGGACC

GGCTCAAGACCTTTGGTACAAGAGGTGTGTACCACCTGTGGTAACCATGACTCTTTTGAG

CAGACGTGGACGATGTTTCCAGGAACGGATCTGTTTGTGGGAATTCCATTCCATGCTGCT

CATACTATATTGGAAACAGATCAAGCAGAGCTAGACTTTGAGGCAAAGAAACTACAGAGC

ATTGTGAAGGACAAATCGTATCTTATATCGGAGACAGGTGCTCTCGCCGACTTGATAAGT

CCAGGAGTGCTTAAATCACTTGTAACCTTAAATGACAAACCAAAGTAA

>MS.gene068014.t1

ATGGCACGCCGTAGCTCCGGAGGAAGATCTGCTCGTCCAGCTCCACGTGCTGCACCACGT

CCTGCTCCAGTTAACCACGCTCCTCCTCCAGCTAATCCTCAGAGTGGTGGCGGCGGTATT

CTTAGTGGCATTGGTTCAACCATTGCTCAAGGTATGGCTTTTGGGACTGGAAGTGCAATG

GCACACAGGGCCGTGGATGCTGTTGTGGGTCCTCGCACCATTCAACATGAAGCAGTAGTT

AATGAGGCTGCTGCAGCTGCTCCTGCACCGACTTCAAGCTCTTTTGGCAGTGATGCATGC

AATATTCATTCAAAGGCATTCCAAGATTGCATCAACCACTACGGAACTGAGATCAGCAAG

TGCCAGTTTTACATGGATATGCTAGCCGAGTGCAAAAAGAACTCTGGAGCCACAATGAGT

ATGTAA

>MS.gene068039.t1

ATGGGTAAGCAACATTTGCAAGGTGGTAGGATTGGTTTAACTTCTGATGCTTTCTTGGTG

ATTAAGGTTCCTGATACACGTTTTTTACGTGTTGTGTCACGTTCTTTGTTTTTGGCTTTG

TTTTTTGTTGCATTTCCTTTCTTGGGGTCCTTTTTAAAAGGGTTAGTTGTTTCTGGATTT

GATGCTATTGTTGTTAATGCTTCAACTTCAACTTTTGTTTCCATCAATGTTGAGGTTTTG

AATTCCATTCTTCATGATTTGGGTGACGAGGGTCTTCTTAAAAAGGAGGATAAGGCTCTT

ATTATGAGCCCTCCGCGTGGTTTTGAAGGTGGTGATTCTTTGTTGAATTGGAATAGTGAG

GTTGATGTTGTTATGCATGATGAGTCTTATGATTTTGCGTTTGCACCAAGCTTTGAAGAT

GCTGTTTCGGCTGATCGTGTTCTGAAAATTGATGGCATTGTAGCTTTTCCTTTGAGCCTT

GATGACTCGTCAAATGCTGGTTTTAGAAAACAATCTAATTATAAAGTTGTGTATCTTAGG

CGTTATGATTCTATTTTTGTTGCATTGAGGAAAATTGGTGTCGAAAATAATTTGGTAGAT

TCTACACCAAGGAAGAGGCTTTGTCAGTTTGCAACAGTGGCCAAGACAACGGCTTTAGAG

GGTCTCGAAGATGCGCTCCTTGAGCCACCAAGACAGGATTCTGCCAAATCAAACAAGAAC

TTGAAGATCAAGTACCTGCCTGAGTTGTTGGGTGATTCTCTAGATGGTTACAAGCGAAGA

GTCTTCGTTGGTGTGGGTTTGCGTGAGGAAAACAAAGCAGCAGTTGAATGGTTTGAGAGA

AACTACCCAAAGAAGAGTACAAAATTTGAAATCCACAGTCTACAGGTAGCATCAGAAGAG

CCGAATGTGCCTCAAACTGATGTTTCTGCTTGGTTATCTAAGCATGTTAAGGCAGATGAG

TATGTTGTAATGAAGGCTGAAGCAGATGTGGTGGAGGAAATGATGCGGAAAAAGACAATT

TGCTTAGTCGATGAATTGTTCCTCCAATGTAACAATCAATGGTGGCAGACTGGGAAGAGA

AAGAAGAGTGGTAGAGCATATTGGCAGTGCTTGGATTTGTACGGAAGGCTGAGGGATGAG

GGTGTTGCAGTACACCAATGGTGGGGCTGA

>MS.gene068022.t1

ATGAACGTAGAAGAGGAGGTTGAAAAGCTGAGGGAAGAAATCAAGAGGCTTGGAAATGTT

CAAGCAGATGGTTCTTGCAAGGTCACATTTGGAACACTCTTTCACGATGATCGGTGCGCA

AATATATTTGAAGCACTCGTCGGTACACTAAGAGCTGCTAAAAAGCGTAAAGTAGTTGCA

TACGACGGTGAACTACTGTTGCAAGGAGTCCATGATAATGTGGAAATCACTCTTAATCCT

GCTCCTGCTGCTGCTAACTGA

>MS.gene068016.t1

ATGGAAATGCTGAATTCGTCTGAGAATGGGTCAATGGCCATGCACTGTACCAATGTAGCT

TCCGTTTCTGACGAAGGTGAAAAACCTCAACCCATTACCGCTTCTGTTCCACTCTCTCGT

GCTAGTGAACTCACCATTTCCTTTGAAGGCGAGGTTTATGTTTTCCCTTCCGTTACGCCA

GAAAAGGTGCAGGCTGTATTGTTACTCTTAGATGGGCAGGAGACACGCAATAGCGTACCC

ACTTCTGACTTTTCGGTGCAGCAAAACTGTCGGGACATTTGGGGGAAAAATGACCCTTTC

CGTAATTCAAAGCTTTCACGGAGATCTGCATCACTTGTTAGGTTTCGTGAAAAACGAAAA

GAGAGATGTTTTGAGAAGAAAATCCGGTACACTTGCCGTAAAGAGCTTGCTGAGAGGATG

CAACGGAAGAATGGACAGTTTGCATCATTGAAGGAAGAATGCAGTTCTCCTGCTGAAAAC

CAGGATTCTAGCAACAGCACTCCTTTCCTAGAATCCACTGAACGTAGATGCCAGCATTGT

GGAATTGGTGCAAAGTCTACTCCGGTAATGCGTCGAGGACCGGCTGGTCCAAGAACTCTA

TGCAATGCTTGCGGGCTCATGTGGGCAAATAAGGGAACACTGAGAGATCTCGGCAAAGCA

GGAAGGATTGCTTTTGAACAGAATGAACTGGATTTTTCAACAGATCCAGAAAATTCATGT

GCTGCACAGGACAAGAAGGAAAGCCCCCATGAAACAAAGCCTTTGCCAATGGATGCCAGA

CAATCACCTGAGATGATAATTGAGCAGTATATGCTCGAAGCTGCTGAAGCAGTTACTGAT

AACTTGTCAATCCAAGTGGAGAATAATGCTCTTGATCTGCACGAGCAGGATAATATTATG

GAGGATTTTGCTGATGCATCTGGGACTGAATTTGAGATTCCTTTAGGTTTTGATGATCAG

GTCGATATTGATGATTCCAATATGAGGATTTACTGGCTGTGA

>MS.gene068027.t1

ATGGACAAGTCCCAAAACAGAGAAGAGAATGAACAACTCAAGCTCTCTAAGTATGGGGAT

GGTTCTATTTGTAGCTACAATTCCCTTCACCATCTTCTCAAGGAAAATCTCAAACCTCAT

CACTTCCAGGAAGTCAATCGTCTGCTTACAGGGCTTAATTGTGGAAAAGTACTTGAAACA

ATTGCTCTACCGGAATCTGCCACAACTCTCTCCGTGAAACATGGTTTTGACCTCCAGGCT

TTCTGCTTTCATGCTGATAAAGAAGTATTAAGGGAACCTCGAGTTGTGAGGGTTGGTTTG

ATTCAGAACTCTATTGCCCTCCCAACAACTGTGCACTTTATCGACCAAAAAAAGGCTATC

TTTGAAAAAGTAAAGCCCATTGTTGATGCTGCTGGTTCATCAGGGGTCAACATATTATGC

TTGCAAGAATTTTGGATGATGCCATTTGGTTTCTCTACACGAGAGAAGAAGTGGTGTGAA

TTTGCAGAACCTGTTGACGGGGAATCAACTGAATTTTTGCGAAGCTTTGCACTGAAGTAT

AACATGGTGATTATAAGCCCAATTCTTGAGAGGGATATGAACCACGGAGAGATTATTTGG

AACACTGCCGTTGTAATTGGGAATCACGGCAATATAATTGGCATACACAGGAAGAACCAT

GTAACAAGAGTCGGAGACTTTACTGAGAGCACATATTATATGGAAGGAAATACAGGTCAC

CCTGTATTTGAAACAGAATTTGGAAAGATTGCCATTAATATATGTTATGGTAGGCACCAT

CCTTTAAACTGTTTAACTTTTGGCTTGAATGGCGCTGAAATTGTTTTCAATCCTTGTGCC

ACTGTTGGTGAACTCACTGAAGCAATGTGGCCAATAGAGGCACGTTATGCTGCAATAACA

AATAGTTACTTTGTTGCTGCAATCAACCGCGTTGGGACTGAGATATTCCCCAATCCATTT

ACTTCTGGTGATGGCAAGTCAGCACATACAGATTTGGGATATTTTTATGGATCAAGCTAT

GTTTCAGCACCAGATGCATCCTGCACGCCGTCTTTATCACGTAACAGTGATGGTTTATTA

ATAACAGACATGGATCTTAACCTGTGTAGGCAGCTTAAAGACAAGTGGTGTTTCAGAATG

ACTGCACGATACGAGTTGTATGCGGAGACACTTGCCAAGTATGTGAAACCAGACTTTGAG

CCTCAAGTCATCCGCGACCCCTTACTTCATAAGAGATCTTCATGA

>MS.gene068025.t1

ATGGTGTCTCATACAGTTGAACTTTTGAAGAACGAGATTCCTCTAGAGCAAGAATCAGTG

GTTTTAGCTGAAGACACTGTAAATGGTCTTGTTCTTGTCGATATCATAAATGGCTTCTGC

ACAGTTGGTGCTGGAAATCTGGCACCAAGGGAGTCCAATAGGCAAATTTCAGAAATGATC

AATGAATCAGCAAGGCTAGCTAGACTATTCTGTGAGAAGAAATTGCCAATTATGGTTTTC

CTTGATTCTCACCAACCTAACAAGCCAGAGGACCCTTACCCCCCTCATTGTATTGCTGGG

ACTGATGAATCAAATTTAGTTCCAGCGTTGAAATGGCTAGAGAATGAAACCAATGTAACA

ATCAGACGTAAGGATTGTTTTGATGGATACGTTGGCTCAATGGAAGAAGATGGTTCAAAT

GTTTTTGTAGATTGGGTGAAGAAGAATAAGATCAAAACTATGGTGGTGGTAGGTGTATGC

ACAGATATATGTGTTCTGGATTTTGTATGCTCCACAATGTCAGCTAAAAACCGTGGTTTT

CTCAAGCCTCTAGAAAATGTGGTGGTATATTCAAATGCTTGTGCTACCTTCAACGTCCCT

CTTGAAGTAGCCACAAATATCAAAGGAGCTTTAGCCCATCCTCAGGAGTTTATGCATCAT

GTAGGTCTGTATATGGCCAAAGAACGTGGAGCCAAGATAGCGAAGGAAGTGATGTTAGAT

GCAGCAGAGAAGATATAA

>MS.gene068036.t1

ATGGAGGGTGGAAACAGAGCTGAAGCTGAACGGTGGCTATACACCGCAAACAAACTCCTA

AGTGCACGTGATCTGCACGGTGCACGCTCCTTCGCGATCCGTGCCCGAGAATCTGACCCA

ACTTTCGACGCCTCTGAGCTTCTCTTAGCTGTCATCGACACTCTCCTCGCCGGAGAATCT

CGGATCAATGATCACCACCGTGATTGGTACGGAATTCTTCAAATTCTCCGATACACCACC

AACATCGACCACATCGCGAATCAGTATCGCCGACTTGCTCTGCTCCTTGACCCTAATCGG

AACCCATTCGCTTTCTCTGGTCACGCTTTCTCCCTTGTTCATGATGCTTGGTCTGTTCTC

TCAAATCCCGCTAAGAAAGCTATGTATGATAGTGATCTACGGTTGCTCACTACACCACCG

GTTCCGCCACAGCCTCAGCCCCAACCGGCTCATCAGTATCAGCAACCTCCACTGCAGCAG

CCTCCACAGCCAAATCCAACGCCGAGGAAAAACTCTAGATCGAGGGATGGCACTGGCGCC

ACCGCCACCGAGCCGACACCGTCTCGACAGAATCGGAACCCTAGCGAGGCAGGTGAGACG

ACTCGGCAGACGCGAACAGCGAGTGCTGCCGAAACGGAAGGTAACGTAAGTTTCTGGACA

TTGTGCCCTTACTGTTACGTTTACTATGAGTATCCGAAGGGGTATGAAGATTGTACCTTA

CGGTGTCAGAGTTGTCGGAGGGGGTTTCATGCGGTGGTGATACGTTCACCGCAGGTGAAT

GAGATAGATTCTTCGTTTTGTACTTGGGGATTTTTTCCATTAGGGTTTTCTGGTGATTCT

AAAGATGTGAATGGGGCTTCTTCCAATTGGAACCCTATTGCACCTTTGTTTCCATGTTCC

ATGAAAGGATCGTCTAATAGGAAAAAGAATTGGGTTTATTATGATGAAGAGGCAACTGCG

GCGTTTATTGATATTTCTGATCCGAGCGATAATGATTCTGATGATGGTGATTGGAGGGGT

GGTGCTGGTAAGAGCAGAGGGGTGAAGAGGTCAGGGGTTAGTACTAGTAAAAAGATTAGG

AAAGATTCCGGTAATGCTAGTGGTAGGGGAGCTGTTGGGAGACCTAGGAGAAGTGCTACT

GGTGCTGCATCTGGGAATGAGAAGAATGTTGGGGCTGTTGATGATGCTGCTGCCGCTGCC

GCTGGTGGTGTAAGGGCAGATACAAGTACTATTAAGAAAGCTGCACTGGGTAGTTCCAGG

AGGAGAGGTGCTGGTAACTTGGATTTGAATGTGGAGTTCAGTAATGATGTGGAGGAGCCA

TCCCGTGGAGTGCGTGCCCGTGAAGGGAATGCAACTGGCAATGCCGAAGATAATATTGAT

GGGGTTGGGTTTTTTGAGGGTCTTGATGAGTTCCTCAGTAGCTTACCCATTCTCAATCCG

GTGGGAGATGATAAGGTTAAGGGTCATTAG

>MS.gene068050.t1

ATGAGTTCTCTTCTTCAACCACAAGGTAGAGATTGTGAGCAAGACAATAGCTCTAAACTG

TTGCCAACAATTGTGAAATTTGAGTCCACATTTGAGGTGGAAGCCACCAAATCAGTAGAA

AATGATAAGGAGGAGGGTATTTTCTTGACATGGGGAGGACCTTTGGGTGACTGTTACAAA

TGGAAAAATGGAAGAAAACCAATACTTCAAGGTCTAAGAGGCTATGAAAACCAGGACAAC

TCTTACTTAGCCATAATAGGTCGTTCTGGTTGTGGCAAGTCCACTCTTCTTGATGCTTTA

GAAGGCTGA

>MS.gene068044.t1

ATGTCAACAAGAGGCTCATGGATAACTTTGAAATTCAGAGGTGTTGTTGATGTAGTTCGT

GCTAGTAGATTCCAATCAAGCTATGGTGGAGTCAAGAGGAACACTCCAATTGTGACAACA

ACTAGGGTTGAAAGTAGCTATTGGAATAGGAATCCTGGTCTGCAATTCTTTACCACCAAA

AGCAACACAAGCACTAATGCAAATAGAATAAAGAATGATGAGATTCAGCCTGAAGCCCCA

GCTTCTAAATTTTTCGCCTTCTCTTCTTGGGTTAAATGGGTTTTCTGTTCGTTACTTTCT

TTCGCACTACCATTCTGGAGTCAAAATTGGGGAAAACTTCAAAGAATAGAAGGAGAAGCA

GAGATTGTGATTGAAGGGGTTGAAAAAGTGGCAGAAGTAGTAGAAAAAGTTGCAACCTTA

GCAGAGAAGGTAAGTGAGGATGTTGCAGAGACGCTTCCTGAGGATGCTAAGCTAAAGAAA

ATAGCAGCGGTTGTAGAAAGTGCTTCAAAACAAGCTGTTCATGGTGCTCAAATAACAGAA

GAATTCATACACAAGGTTGAAAAAGTGACTAATGACATGGAGGATTTAGAATCATTCGTT

GAACCCTTAATTGACAAGATTGTGAAGAAAGAAACTCGAAAGAACTGA

>MS.gene068012.t1

ATGGCTTCTTCATGGTTTCTTAAACAGGCTTTCAAATGTCTTCAACATTTTGCATGGCCT

CTTCTTGCTCTGGTGTATCCTATGTTTCAGCTCTGGCCGTACATTAAGCTAATGATCATC

TTATGGCTCATCATACCGGATTTTGGACGAACTTCTTATGTTTATAATAACCTTATTCGC

TCCATGAAACCGCAAATAGTCACATGGAGGTTAAATAGTTACTGGAGAGATTGTTTTGCT

GAGAAAGGTAACTTTTTACTGCATGCAGAGAGATATATGAAAGAGAATGGAACTGAAACC

TTACAGAAACTCATTGGTAGCAAGAACACAATGTGCATGCCTGATGCAGAAGTGACAAAT

GAAACCATAGATACTGATAGTAAAGAAATATTAAAGTACTTAATGAGGGAGCTTGATCTT

GTGCAGAGAATAGATTTTCGTGCAACCAAGCAAACAACCTATGCTGATATTTTGGCAAGT

CAAAAACCATCATCATCCATACAAGAAACCAAAGGAACAGCGGAGAGTGACACTGCTAGT

GGGGAGCTACCTCAGAGTTCTGCTACACAGAAGGAAGTGCTGAAGAAGTGGACTTGTGCT

TTATGTAAATTGCCTTCCCGAGCAGGTAGAGTAACTCTGGGTAAACGTGGTGCCTCCAAA

AATGACTTCTTCAAAACATGGATTGAGTATCTTCAACTGAAGTGGCCACTTGTTGCTCTG

GTGTATCCTATGTGTGCTTCTGTACTAGCAATCGAGACCGATTCCTATGCAGAAACTAAG

GATTTGATCTCATATTGGATACTTCTTTCCTTAATTTACCTCTTTGAATATGCTTTTATG

AGCCTTCTTCTATGGTTTCACCTCTGGCTGTACATTAAGCTAATGGTCATCTTCTGCCTC

ATCATACCGGACTTTGAACGAGCTTCTTATGTTTATAATAAACTTATTCTCACCATGAAA

CCCCACATCGTCACATGGAGGTTAAATGACTACTGGATGAAGTGTTTTGTTGAGAAAGAT

AACTTTCTAATGCATGCAGAGAGATATATTTCAGAAAATGGAACTGAAGCCTTAGAGAAA

GTCATTGCCAACAAGAGACAGACAAATGACGAAAGACACGAAACAGAGAACAAAGACATC

AGAGATTTGGAAGCTATTGAGAAAAAAGAAATTCCTGGAACCAAGCAAGATATTCCTGTC

ATTCCTGAAATTGGGCCAAGTCAAAATGCATCATCAGCCACAGTGGAAACTAAAGGAACA

GCAGAGAATGACAGAGCTGGTGGAGAGTTTCCACAGGGTTGTACACAGAAGGAAATGCAG

AAAAGGTGGACTTGTGCTTTATGTTTGGTAACAACTACAAGCAAAAAAGACTTGAGTTCC

CACCTTACTGGGAGGAAACACAGGGATAATATTGAAGCTTTAATAGCAAAGAAGCAACCT

ACCCTAGAGAAGCAAAAGGATGTGGAAGGGACAAATGAAATCATAGCTACTGATAATAAA

GAAATACTAAAGACAAATGGAGAAAGACTCGAATCAGAGAACAAAGACATCAAAGATTTG

GAGGCTATTGAGAAGAAGGAATTTCCTTCAACCGAGAAAAGAACCTATGCTAATATTGTG

GCAAGTCAAACAGCATCATCAGCCGTAATAGAAACCAAAGGAACTGCTGAAAGTGTCACA

GCTTGTGGAGAGGAGGTTCCTCAGAGTTCTACACAGACGGCAGTGCAGAAAGAGTGGACT

TGTGCTTTATGTTTGGTAACGGTTAGGTGCGAGAAAACCTTGATTTCTCACCTGAGGGGA

AGGAGACACAGGGAAACTATGGAAGCATTGAAAGCAAAGAAGCAACCTACCCTGCAAAAG

AACCTATCAGAACCTATTAGGATGATTAATTCCAAAATAATATGTAAAGTCTGTAATATT

ATGCTTCCAAGTGAGGACTACGTGGCCTCTCACATAAAAGGGTGGAAGCACTTGTCTAAT

ATTAAAAGTTGA

>MS.gene068007.t1

ATGTCACTGATTCCAAGTTTATTCGGTATCAGAAGGACTAACATTTTCGATCCATTCTCC

GTCGATCTTTTCGATCCAGATTTTCCGTGCCGTGATTCACTTTCTGCTTCCTTCCCTGCA

AATTCAGCATTCCTGAGCACATGTGTGGATTGGAAGGAGACTCCAGAAGCGCATGTATTC

AAGGCGGATCTTCCAGGAACGAAGAAGGAAGAAGTGAAGGTTGAAATTGAAGATGACAGA

GTTCTTCAGATTAGTGGAGAGAGGAATGTTGAGAAAGAAGATAAGAACGATCAATGGCAT

CGCGTGGAGCGTAGCAGTGGGAAGTTCATGAGGAGGTTTAGATTGCCAGAGAATGCTAAG

ATGGATCAAGTGAAGGCAAACATGGAGAATGGTGTTCTCACTGTAACTGTTCCTAAGGAA

GAAGTGAAGAAGCCTGAAGCTAAGACCATTGATATCTCTGATTAA

>MS.gene068021.t1

ATGGAAGATGACGAGGTTGTCCGTGCGGTGGTAACCGGTACAATTCCGTCGTACTCGCTT

GAATCAAAACTCGGAGATTGCAAACGTGCGGCGGCGATACGTAACCAAGCGGTGGAGAGA

GTTACCCGAAAGTCACTCTCAGGTTTACCAATGGAAGGTTTACCAAGACGGTGA

>MS.gene068049.t1

ATGGGTGAGAGGAAAAAGGGTGGAATTACTGTGACATGGGAGAATTTGGAGGCCATTGTT

ACAAATGGAAAGAATAGAAAACTAATTCTGCATGGACTTACAGGTTATGCTCAGCCAGGG

AAGCTTTTGGCAGTAATGGGTCCTTCCGGCTGTGGCAAATCCACACTCCTTGATGCTTTG

GCAGGAAGATTGAGATCAAACATACAGCAATCGGGGGAGATTCTTATAAATGGCAAAAAA

CAAGCACTGGCTTATGGAACATCAGGCTATGTAACACAAGATGATGCTATGCTGTCAACT

TTAACAGCTGGTGAAACTTTATACTACTCAGCTCAACTTCAATTTCCAAACTCAATGTCT

ATAGCAGAGAAGAAGAGGCAAGCAGACATCACACTCGCAGAAATGGGCCTGCAAGATGCT

ATTAACACAAGGGTTGGAGGGTATGGTTCTAAAGGCCTAAGCGGGGGGCAAAGAAGGAGA

CTAAGCATTTGCATTGAGATTCTAACACACCCTAGACTTCTTTTCCTTGATGAACCAACT

AGTGGACTTGATAGTGCAGCTTCCTACTATGTTATGAGCAGGATTGCAAGTTTAAGTCTA

AGGGATGATATTCAAAGGACAATTGTTGCATCCATACATCAGCCTAGTAGTGAAGTTTTT

CAACTTTTTGATGACCTCTGTCTTCTTTCCTCTGGGGAGACAGTATATTTTGGTACAGCT

TCTGAAGCAAATCAGTTTTTTGCTTCAAATGGTTTCCCTTGCCCAACTCACTATAATCCT

TCTGATCACTACTTAAGGATCATAAACAATGATTTTGATCAGGACACTGAAGAAGGCTTT

GGTAAAGGAGTAGTTACTGAAGACGCAATTGGTATCCTTGTAAATTCTTATAAAGAATCT

GAAATTAAAAGTCAAGTTTTGATGGAAGTTACAAACATAAGTGAAAGAGATTTGAGTGCA

ATAAGGAATAGGAGGACCCACGCTCCATTTCTGACTCAGTGCATGGTTCTTATAAAAAGA

TCCTCCCAACAATTGTATCGTGATATCAGCAATTACTGGTTACGTCTTGTTGTCTTCATT

GCCATTGCTATAAGCCTCGGCTCTATCTTCTACCAAGTCGGTTCAAGTACTCGATCTATT

CAGGTCAGAGGATCGCTACTTTCATTTTTTATGTCAGTTTTGACTTTCATGACACTTGTT

GGTGGATTCTCTCCCTTGATTGAGGAAATGAAGGTGTTTAAACGAGAGAGATTAAATGGG

CACTATGGTATTACTGCTTTTCTGATTGGCAATATATTATCTTCTATTCCATACATGCTA

ATGATCTCTCTCATTTCCGGATCAATAGTGTGTTACCTTTCTGGACTACACAAAGGACTA

GAGCACTATCTATACTTTGCTTCCATCCTATTTGCCATTATGATGTGGGTTGAGAGCCTT

ATGATGGTTGTGGGGAGTATCTTCCCAAATTTTGTGATGGGGGTGATCATTGCTGGTGGA

GTTGAAGGACTTGCGATTTTAACAGGTGGAGTCTATCGAATTCCTAGTGATCTTCCAAAG

CCATTATGGAAGTACCCCTGCTACTACATTTCCTTCCTCACATATGCTTTCCAAGGATCA

TTCAAGAATGAATTTGAAGGCTTAACATTTGTTGGTTATCAAGAAGGAGGTACCATAACC

GTTAGTGGTAGAGATGCACTAACAGATATATGGCATGTGCAAATGGGTCACTCGAAGTGG

GTTGATCTTGTTATCATGTTTGGTATGATTGTTGTTTATCGAGTTCTCTTCTTGGTCATC

AATAAGGTCAAGGAGAAGTCAAACCCCACCTATCCTACCATAAATCGACCTCAAGCAAAA

ACTTTCTCTAGAACCAATATGGATGAACTCTAA

>MS.gene068030.t1

ATGAGTTCCATCGGCGAATCAACCACCAACCAAGAAAACTTTTCCCTCGGCGTCGCCAAA

CAACTCTTCTCCTTGGAAAAATCGGACAACAACATTGTGTTTTCCCCGTTGTCGCTCCAA

GTTGTTCTCAGCATCATCGCTGCTGGCTCCGAGGGTCCCACACAACAACAACTTCTCGAC

TTCCTCCGCTCTAAATCCATCGATCATCTCAACTTATTCGCTTCTCAGCTCGTTTCCATC

ATCCTCAAAGACGCTGCTCCTGCGGGCGGACCTCGCCTTTCTGCAGCCAATGGTGTGTGG

ATTGAACAATCACTTTCTCTTCAACCTTCCTTCAAAGAAGTTGTGTCTTCTAACTATAAG

GCCACCACTTTGGCTTCAGTTGATTTTCTGAACAAGGCAGATGAAGTGACTAAAGAAGTG

AATTTATGGGCTATAAAAGAGACAAATGGACTTATTGAAGAAATTCTTCCTCCAAGGTCA

GTCGACAACTTAACCAGACTCATCTTTACAAATGCATTGTACTTTAAAGGAGCATGGAAT

GAGAAGTTTTATGATTCAATGACGAAATACTATGATTTTCACCTTCTGAATGGTCTTCCT

TATGAGCAAGGAGAAGATGAGCGTCAATTCTCTATGTACATTTTTCTTCCAAATGCAAAA

GATGGTTTGACAGCTTTGGTTGAAAAGGAGTTAGGAGTGATTTTACCTTTCTCTCCTGGG

GGTTTGACAAATATGGTGGAGTCTCTCATGGATCAAAACCTCTACGTTTCTAACATATTT

CACAAGTCTTTCATTGAAGTAAATGAGGAAGGAACTGAAGCTGCCGCGATCACGGCTTGT

TTTACGCACATGCAACCCATGGGTATGCCTGTACCAATTACTCCAATAAACTTTGTAGCA

GACCATCCTTTCTTATTTCTAATTAGAGAAAATTTGAGTGGAACAATCCTCTTTGTTGGA

CAAATGTTGAATCCTCTTGCTGATAGATTTTAA

>MS.gene068042.t1

ATGGCTTCAACAGTTCCTCCTCAAGTTAACCAAAAATCCAACAATGGAATCACTACTTTC

ACAAGCGTGAAAACACTCTCAGAATCACCAGATTTCAACTCTATTCCATCCTCCTACACT

TTCACCACCAACCCACATGATGAAAATGAAATAGTAGCAGACCAAGATGAAGTCAATGAT

CCAATCCCAGTCATTGATTACTCACTCCTGGTCAACGGTAATCATGATCAACGGACCAAA

ACCATCCATGATATAGGCAAAGCTTGTGAGGAGTGGGGATTCTTCATGCTAACCAATCAC

TCTGTTTCAAAGAGTCTCATGGAGAAAATGGTTGACCAAGTTTTTGCTTTCTTCAATCTT

AAAGAAGAAGATAAACAAGTGTATGCAGATAAGGAAGTAACGGATGATTCAATAAAGTAT

GGTACAAGCTTTAATGTTTCAGGGGACAAAAACTTGTTCTGGAGGGATTTCATTAAAATC

ATTGTTCATCCTGAATTTCACTCACCTGATAAACCTTCTGGCTTCAGGGATAGGATAATC

CTTGCTCATTAG

>MS.gene068023.t1

ATGGGAAGCAACACTGAAGAATCATCATCATTTACTACTTTCAAGCGCAATAACGGCCAC

GTCGTCTTCCGTCTCCTCTGCCACGCGTCACGTATCGGCGCTTTCATCGGAAAATCCGGC

TGCGTTATCAAATCCCTTCAACAACTCACCGGAGCTAGGATCCGAATCGACGACGCGCCG

GTCGACTGTCCGGAGCGTGTCATCGTCGTCATCGTTAATCTCAACGGTGACGGAGATGTT

TCTTTAAACCCACAGGAGGCTTTGTTGAAGGTGTTTGAGAGGATTCTTGATGTTGCTGCT

GCGGAGAGTGATGGAAATGGTGTTGGTGATAGAGTGGTTTCTTGTAGGTTGCTTGTGAAT

GCTGGTCAGGCTGGTGGGGTGATTGGTAAAGGTGGGATGGTTGTGGCTAAGATTAGGGCT

GATACTGGTTGTAGAATTAGGGTTTTGAATGATAAGTTGCCGGCTTGTACTAAACCTTCT

GATGAAATCATTGAGATACAAGGCATTGCATCGTCTGTAAAGAAGGCCCTCGTTGCTGTC

GCTGGACGGCTTCAAGATTGTCCTCCACTTGACAGAACAAAGATGATGGGAACCAGACCT

CATGAAGCATTTCAGTATGAAACTTCTGCTGTTCCACATGAGGGCTTAACTGATCTGAAT

ATGGATTTCCGTCTGCAGAGGAGCTCTGCAATGCCCACTTCATCCATCAGGTCTAATGGT

GTCCCCTCTAAAAGTCATCCGTTGTCCGTTGAAGATAACAGAGTCTCATCTCTGGATCCG

GAAGCACTTAAGCAGGAAGTTACCTTTAGAATTCTTTGTTCCGGTGATAGGATTGGTGCT

GTGCTTGGGAAAGGTGGCAGTGTTGTAAAAGCTCTTCAGAACGAAACAGGGGCTACTATT

AGTGTTGGTCCTCCAGTATTTGAGTGCGAGGATCGGCTAATTACTATTACTGCTTTAGAG

AATCCCGAATCAAGATTTTCTCCAGCGCAGGAGGCTGTTGTGCTTGTTTTCTGCAGGTCT

ATTGAGTGTTGTATTGAGAAGGTGGTAGACTGGCGATCAAACAAGGAGTCATCTGTCACT

GCACAGCTAGTAGTCCCGTCCAACCAAGTGGGTGTTTTGTTAGGGAAAGGTGGAGCAATA

GTCTCAGAAATGCGGAAGGCCACATGGACTAGCATCCGAATAACTCGGAATGGTGAAGTT

CCAAAGTGTGCATCATTTAATGATCAAGTGGTACAGATATCAGGTGAACTTCCAAATGTT

CGAGATGCATTATATAATGCTACTAGAAGACTGCGAGATCATATTTTTCTCATAGCACAA

AACAGTGGTGGAACCGGTCCCTACAGGAGACCGAGGGACTCCATTCCTCTTGGTCTTGGA

GGCCAATCAGCTGTTGGCAGTAATCATGGTCCAAGCATTCATTCTTTATCTCAAAGCATG

GATCATCTCACCCTTTCTCGAAATTCAGGTCGTTCTGCTTCATCAGGGGTATGGGCACCA

AAGGCGGTGGGTGGGAAAAATTCCAGATATACCGATGATGCTGGCAGGAGATTGAGTCCT

CATGAAGGTGACTTAGAACTTGCCAGTGGGAGTAACACTGTCATTGGTAACGCTGCCATT

ATAACTAGTACAACTGTGGAAATTATGCTTCCCAATGATATTATTGGCTCTGTATATGGG

GAAAATGGTAGCAATCTGGATCAATTGAGACAGATATCAGGTGCCAAGGTTGTTTTTCAT

GAACCTCGTCCTGGAACAAGTGACAGAGCTAAAATTGTCTTATCGGGCACACCTGATGAA

ACTCAAGCAGCTCAGAGCCTTCTCCAAGCATATATTCTCAATGGATCATCATGA

>MS.gene068031.t1

ATGGGATCTATGAATCTGCCGTCGAAATTATCAATTACAGAGAATGAAGGTTCCAAAAGA

TCATTTATCCACAGCAGTTGTTGTGAGCCACGTTTTAGTGCATTAACTCCAAATGTTGCC

ATGAAGCCTTTGCCTTTTTTACAAAGCCGTTTCCAGAGAGGAAGAGCTGGGTGGACAGTA

GCTTTGGCGTTGAACACAGATGGATTGTCTGGTAACGGTGAGCAACAAAGCCTAAATGAT

TCTGGTTCCAGTTTAGGAGGCACTCGATTGGGGAGGATACTTAGTGCAGGTGGCAGACAA

CTTCTAGATAAGCTGAATTCAGCTAGAAAGAACTTCCCTATGAAAGTATTCCTGCTACTT

TTAGGTTTCTACACGGCAAATGCACTAGCCACAATCCTTGGTCAAACCGGTGATTGGGAT

GTTCTGGTCGCTGGTGTTGTGGTGGCTGCAATTGAGGGTATTGGTATGCTAATATATAAA

AAGCCACCTACAACGAGGACCGGGAGGTTGCAGTCTTTTCTTGTGATGGTGAACTACTGG

AAAGCAGGCATATGTTTGGGCCTATTTGTAGACGCTTTCAAGCTAGGCAGCTAA

>MS.gene068019.t1

ATGAAACGAAAGGATACAAGCATTGATAAAGTATATGATGCACGTGCATTAAGAGTAGTT

GTAGGAGACAAAAATGGAACTTTACATGGACCTGCAGTTCAGTGTTGCTATAGTCTTCTT

GACATTGTACACAGACTTTGGACACCGATAGATGGTGAATTTGATGACTACATCCTTAAT

CCGAAGCCTAGTGGCTATCAATCCTTGCACACTGCAGTTGAAGGTCCTGACAACTCGCCT

TTAGAAGTACAAATAAGAACACAGAGGATGCATGAGTATGCTGAACATGGACTGGCCGCA

CATTGGCTTTATAAGGAAACCAGAAATCCTTTTTCATCCATAGACAGAATGGATACACCT

GAAACAGATGCATCCTCATCCATAGACAGAATGGATGCACCTGAAACAGAAGCATCCTCG

TCTATAGACAGAATGGATACACCTGAAACAGAAGCATCCTCCTATTTTTCCAAAGACACA

GAAGAGGAAAATTCTTCAGATATTTTGCTGAGCAAGAATAAGTCATTGAAGGCTGGACAC

CCAGTCCTTAGAGTAGAAGGAAGTCACCTACTTGCTGCTGTTATCATCAGTGTAGAAAAT

GAAGCAAGAGAATTGCTAGTTGCTGTAATCTTTCAGCTAGCAGCTTCTGATGCAGTAGCT

GATAGAAGATCATTTTTCCAGGATAAGCGATGGGAAGCTTATGCACGGCTATACAAAAAG

GTGTCTGATGAATGGTGGTTTGAACCAGGACATGGGGATTGGTGTACTTGTCTAGAGAAA

TACACACTGTGTCGAGATGGTATGTATCACAAGCAAGACCAATTTGGGCGCCTTCTACCA

ACATTCGTCCAGGTTATCAATTTTACCGAGCAAGAAGAATCTGAATATTGGGACGTTGTA

TCTGCCGTGTTTGAGGGCAAGCAAGTGGATTGTATTGCATCGCAATCAAAATTAGACTTG

GTTCCGTCAACTTCTGTGGATGCTAGCATCAATAATAAGGTGCATCTTTTGAGAACAATG

CTTTCCTGGGAAGAGCAATTGCGTTCTGAAGTAAATATCAACCAAACAAAGCACGACGCA

AAGTTTGATGGTCCACGTGGTCCTCTTAACCTTGGAGAAGTGGTAGTTATATGTTGGCCT

AATGGTGAAATAATGAGGTTGAAAGCTGGTAGCAGTGCTGTTGATGCTGCACAAAGAACT

GGTTTGGAGGGAAAACTGGTTTTGATCAACGGACATTTAGCATTGCCTAATACAAAACTC

AAAGATGGCGATGTTCTTGAAGTAAGAATCTAA

>MS.gene068024.t1

ATGCAGTCAGCAGCAACAACAACACCCTTCTATCAACTCATCACCTTCCGACCAAAGCTT

CAAACTTTCCAACCAAAAATTCCAACCCTTTTCTTCACTCCAACAATGGCTTCAGTAACA

AGAGTAAAACCACTATCAGTCTTAGGTACTTCTTCCTCATCTTCATCAACAGTAGCAAGT

GATGATGGGTCTGTTACATTGATGGAGTATGTAGGTAAAGGAGGTGGAATTGGTGTGAAT

GATGATTTGGTGATTTTGATTGATCATATACAGTATGCTTGTAAGAGAATTGCAGCTCTT

ATAGCTTCTCCTTTCAATTATACCATTGATATGCCAAATAGTGGTTCTGATAGGGATGCT

CCAAAGCCTCTTGATATTATATCGAATGAAATTATCTTGTCATCACTTCAAAAATCTGGA

AAAGTTGCTGTCATGGCTTCTGAAGAAAATGATACACCAACTTGGATAAGTGATGATGGT

CCATATGTGGTCGTGACAGATCCCCTCGATGGTTCTCGAAATATTGATGCATCCATACCA

ACTGGTACAATTTTCGGTATTTATAAACGCCTTGAGGAGCTAGATAATCTGCCCACAGAG

GAGAAGGCTTTGCTGAATTCACTCCAAAGTGGAAATAGGCTGATAGCTTCTGGTTATGTT

CTTTATTCATCTGCAACTATACTCTGCATTACCTTTGGCTCTGGGACACATGCATTCACT

CTCGACCGTTCAACAGGAGACTTCATTCTGACAAATCCAAGCATTAAAATTCCTCCCCGT

GGGCAAATATATTCTGTGAATGATGCAAGATATTTTGATTGGCCTGAAGGCTTAAGGAAA

TATATTGATACTGTGAGACAAGGAAAAGGTAGATATCCTAAGAAGTACTCTGCAAGGTAT

ATATGCTCTCTAGTAGCTGATCTCCATCGAACTTTGTTGTATGGGGGTGTGGCGATGAAT

CCGAGGGACCATCTTCGTCTTGTTTATGAAGCAAACCCTCTTAGTTTCATCGTGGAGCAA

GCTGGTGGGAGAGGATCCGATGGAAAAAGTAGGATTCTTTCTCTTCAACCAGTTAAACTG

CACCAAAGACTTCCTCTCTTTTTAGGGAGTTTGAAAGACATGGAAGAGTTGGAAAGTTAT

GGAGACGTACAACAAAAAGTAAATCCTGGTTATGAGGTTTGA

>MS.gene068017.t1

ATGGAGGAAGCCAAGAAACTCAAAACAGTGGCTTGCTATGATGAAGAGTGTGACCGGTTT

AGCGATCTCTCAGACGAATTGGTATGTCATGTTCTATCTTTCCTCCCAACAAAAGCAGCA

TACAGATCAAGTGTTCTATCTAAAAGATGGGCATCGATCTGCACCACGATCCTCGACCTT

AACTTTGAAATACCAGAAGTAGTTTCAGGTTCGTCCACAGAAATTAAATCTGTATATGCA

GTTCTTCTAAGACGCACTGAAAATATAAGAAAACTACGTCTACATAATTATGATGGTTGT

CAACCATGTGACGTACACTTGTGGGTTTCAAAGGCATTAGATCTCAAGGTACAGGAACTT

CACTTGGAGTTTGGGAGCAGACCTAACCCTATATTGCCGCTAAGGCTATTTAGATCCGAG

TCACTCGTGGTCTTAAAATTGCGTGGTCATATTGAATTTCAACCAACACTTGATTCTTCT

TTTGATATACATTTACCATCGCTAAAGATTCTTCATCTCAGTTTATCTAAGCTTGATTTT

AATGAGGATGGTAGTGAGTATGATCTTCTTGAATTACTATCTGGTTGCCCACGTCTTGAG

GAATTTCTTTTTCATGGCTACTTGAAACAACCTATCAATATCTCTTTTCCCTTGTTAACA

AGGTTAGACCTCAATTTTTGGAAGTTTTCTGATAGTGGTTCTCCCATTGGAACCTTGCAA

ATTAATGTCCCGTCATCACTCGAAGTTTTGGATATTACTGATTTTTCTCCCAAAGAATAC

GAGTTGATAAACTTGTCTAATATTGATCGAGCTACTCTTAGCATTATAAAGTATGTAGAT

TTTAACAGTTTACATATACTCTTAAAAGGACTTTCTAATGTCAAATCATTGACCCTCACT

TCTGAAACAATTAAGTTTTTAAGCATGGAGGACAATCTTCACAACTTAAGTTTCCTTACC

TTTCATGAATTATTGTCTTTGTCGGTTGGAATATCCGAGAATTGCAATTGGAATGTGCTT

GTTGGCTTTCTTCAAAATGCTCCTAAGCTTAAGGATCTTGTCATTGCGAAAAATTTTAAA

AAAAACTCTCAGAAAGGAGGTTGGAAACTCGCGTTGGGTGGGACCATCGACGACTCCGAC

ATGTCTTTCAACTAG

>MS.gene068032.t1

ATGGATGAAGAGTATGATGTAATTGTCCTCGGCACTGGCCTCAAGGAATGCATCCTTAGT

GGTCTTCTCTCTGTTGATGGCCTCAAAGTGCTGCACATGGATCAAAATGACTACTATGGA

GGGGCTTCCACATCTCTTAATCTTACACAGCTATTTAAGCGATTTAGGGGAGATGACAAG

CCGCCAGAGGAATTGGGCTCTAGCCGAGAATACAATGTTGACATGATACCGAAGTTTATG

ATGGCCAATGGAGCTTTGGTTCGTGTGCTCATCCACACCGATGTTACAAAGTATCTGAAC

TTTAAAGCTGTAGATGGAAGCTTTGTGTATAACAAGGGAAAGATTTACAAAGTTCCAGCA

ACAGATGTTGAGGCACTGAAGTCACCTTTAATGGGTCTGTTTGAGAAGCGCCGGGCTCGG

AAGTTCTTCATTTATGTCCAAGATTATGAAGCAAACGATCCCAAGTCTCACGAAGGACTT

GATTTGAACCAAGTTACAGCAAGACAACTGATTTCCAAATATGGATTGGAAGATGATACA

GTTGACTTTATTGGTCATGCCTTAGCACTTCATCTTGATGACAGTTACTTGGATAAGCCA

GCTAAGGATTTTGTGGACAGAGTTAAGACTTATGCAGAATCCCTCGCACGTTTTCAAGGA

GGTTCACCTTACATATATCCACTATATGGACTGGGAGAGTTGCCTCAGGCATTTGCTCGG

TTGAGTGCTGTGTATGGTGGAACTTACATGCTGAATAAACCAGAATGCAAGGTAGAGTTT

GACGCTGATGGGAAAGCCATCGGTGTGACTTCAGATGGAGAAACAGCGAAATGCAAGAAA

GTCGTGTGTGATCCATCATATCTGCCTGACAAGGTTCAAAACGTTGGAAAAGTTGCGCGT

GCTATATGCATTATGAGCCATCCTATTCCAGACACTAACGACTCTCACTCGGCACAAGTC

ATTCTGCCACAGAAGCAACTTGGTCGTAAATCAGACATGTACCTTTTCTGCTGCTCTTAT

GCTCACAATGTAGCTCCCAAAGGAAAATATATTGCTTTTGTTACCACAGAAGCTGAGACT

GACCAACCTCAGGTGGAATTGAAGCCTGGAATCGATCTCCTTGGACCAGTCGATGAGATC

TTCTATGACATCTATGACAGATTTGAACCCACCAATGATCATGCTACTGATGGTTGTTTC

ATCTCCACAAGCTATGATCCCACAACACACTTTGAAACCACAGTGAAAGATGTGGTTCAG

ATGTACAGTAAGATCACTGGAAAGGCGCTTGATCTTTCTGTTGACCTGAGTGCTGCAAGT

GCTGCAGCTGAAGAATAA

>MS.gene068034.t1

ATGGCCTCAACCAAATCCTTCATTGCAGCTTTACTTCTTGTTGTCACAATGTCAAGCATG

AGCCTAGAAGCTCGCCATCTATTGCAAACAACAACACAACCAAATTTACCTACCATTCCC

ACTTTACCAAAACCAACTACATTACCACCTTTGCCTTCAATTCCAAATATGCCTCAAGGA

AATCTTCCTCCATTGCCTACTATCCCTTCTATGCCTAAACTCACTATGCCACCACTTCCA

AGTCTTCCTACCAATATTCCAACAATTCCATCTCTCAACATTCCACCATTGCCAGCAGCC

ACTTCACTTCCCAACCTTCCTTCAATCCCAACCACTTTCCCCTCCTTCCCATTTTTTTCC

CCACCACCTTCAACTCCTAGCCCTTAA

>MS.gene068020.t1

ATGGCTGCTGAATCAAGGAAACGTGCAATGGAGGCTTTGGAGAGAAGAATTCAAACTGAA

CATAAACTAAAAGAAAAGAAAAACAAAAGGGATATAAATCATCAACATATCCTGAAAGAA

AAGAAAATTATGAAGTCTATAAATGAAGATGTAAAATCACCTACTCCACCACCCTCTACT

TCTAATGATCCGTCCTTGCCTTTATGTCGTCCATCATCGGATACACCCAACAAAGGGAAT

TTCGGTCTTTTTGGTCGTGCCATTTCACAAGAGAAAGAAGATGGTCCAGAATATGCCCAA

CTTTCTGTGACTGTAAATGAAAATCTGCTCACAACTAATGGGGAGTTTTCTTCTGAAAGA

GGAGGTTCAGTTTCCGGAATATTGCATGAACTTCTTCAGAAGGGAGATGCAGCACAAAAG

TACATGCAAGGATCTAGAAGCATGAAAATCGACAGCTATATCCTTCTTGATAATTTTGTG

CAAGGACGTGCGCTTTCTTCATCTTCTCAGACAAGGGCTTTGCAAATTCATTCAAAACGT

TCTAAGAGGCATATGTCCATGAAACAACATAAAAAACATGGATCAATGGATCTTCCCAAA

GAGTTCCATAAATTTGACATTTTCAAGCCAATGCATGACATGTGGAAAGACTATATAAAG

TTGTTGCTTAAATCCACTGGGAATAATCAATTGGCTCAATGTCTCCTAGGTGCAGATCTA

CATGGTGCTATTATTTTAGTTGTGGAGTGTAAACTAACCCATTTTACTGGAACTGGTGGC

ATCATGATTCGTGAAACTGCAGAAGCTTTTGGGATAATTACTGAAGCTAATAAATTCCAA

GTTGTTCCGAAGAAGGGTTCTGTATTTGTACTACAAGTTGATTGCTGGAAAGCCACTCTG

CTTGGAGATAAACTAGATTCAAGAAAGGTTGGATTATGA

>MS.gene068043.t1

ATGGGACTTTATCACAGAAAACTCTTATCTGAATCTGAATGTGAACGCTTATTATGTCAT

GATGATTGTACCACAGCCACAAGCTCAAACTGTGTTAATTGCCTCAAATTCTGCAATACC

TACAACCCTCCACCACCACCATCTCCTGATCACACCAAACACAAAACCACCACTTACTTG

ATCATAAGTTTTTCTATTGTAGCAGCTATTTTCCTTGTTCTTTGCTGCTATGCCTTCTAT

GTCAAGTTTTTCTCCCGTAGGAGCAGGTCAAGAAGAAGAGCCTTGTTAACAAGGCAACAA

ACAGAACATGGTTTTGTTGTTGGTGAGGAACATGATGATGGGTCTGTTGTAGACCACCCC

ATATGGTATATTCGAACTCCTGGTCTTCAACAATCAATTATTAATGCAATCACTGTTGTT

AAGTATAAGAAAAGTGAAGGTTTGATTGATGGATCAGATTGTTCTGTTTGCTTGAGTGAG

TTTGAAGAAGATGAGAATCTTAGACTTTTGCCAAAGTGTAACCATGCTTTTCATTTACCT

TGTATTGATACTTGGCTTAGATCACACATCAACTGTCCTATGTGTAGGGCTCCTATTGTT

GTTGACCCTTTAAGGATTCCATCTATGGAGCCTAATGTTTTTGTGGAAAGTTCTCAGATT

GAAGTTTTGGAAAATAGTGGTGGAAATGAAGATAGTGTTGTTGCTCAATTGAGAAATGGA

GAAGAGGAAGCAGGGGAAGAGGTTGAGGAAAATGGAAGAAGGGTTTGTCAAGATGAGAGT

TCAGCTTTGGAGATAGTAAATATGCAGCCAAGAAGATCAGTTTCTCTTGATTCTTCTTCT

GCTGCTAAGATCAATCTTGCTCTTTCCAAGTTTCTATCAGTGGAATCTCAAAGGAATTGT

AATAGGGAGCAGGTAGGGGAAGTCAATGAACAAATTGTTTCAAAGAAGGTTGGTGAAAAT

GGGAATATGGTAAATATGCATAGTGCTCCTTGTTCAATGAATAGGTCAAGATCCTTCAAT

GGTAAACACCTACTATCCTTGTATAGTAGTAGTCAGAAGAAGAAAAATGCTCCACCAAGA

AGCTTTTGA

>MS.gene068051.t1

ATGGGTGAGGGGAAAAAGGGTGGAATTACTGTGACATGGGAGAATTTGGAGGCCATTGTT

ACAAATGGAAAGAATAGAAAACTAATTCTGCATGGACTTACAGGTTATGCTCAGCCAGGG

AAGCTTTTGGCAGTAATGGGTCCTTCAGGCTGTGGCAAATCCACACTCCTTGATGCTTTG

GCAGGAAGGTTGAAATCAAACATACAGCAATCAGGGAAAATTCTAATCAATGGCAAAAAA

CAAGCACTGGCTTATGGAACATCAGGCTATGTAACACAAGATGATGCTATGCTGTCAACT

TTAACAGCTGGTGAAACTTTATACTACTCAGCTCAACTTCAATTTCCAAACTCAATGTCT

ATAGCAGAGAAGAAGAGGCAAGCAGACATCACACTCGCAGAAATGGGCCTGCAAGATGCT

ATTAACACAAGGGTTGGAGGGTATGGTTCTAAAGGCCTAAGCGGGGGGCAAAGAAGGAGA

CTAAGCATTTGCATTGAGATTCTAACACACCCTAGACTTCTTTTCCTTGATGAACCAACT

AGTGGACTTGATAGTGCAGCTTCCTACTATGTTATGAGCAGGATTGCAAGTTTAAGTCTA

AGGGATGATATTCAAAGGACAATTGTTGCATCCATACATCAGCCTAGTAGTGAAGTTTTT

CAACTTTTTGATGACCTCTGTCTTCTTTCCTCTGGGGAGACAGTATATTTTGGTACAGCT

TCTGAAGCAAATCAGTTTTTTGCTTCAAATGGTTTCCCTTGCCCAACTCACTATAATCCT

TCTGATCACTACTTAAGGATCATAAACAATGATTTTGATCAGGACACTGAAGAATGCTTT

GGTAAAGGAGTAATTACTGAAGACGCAATTGATATCCTTGTAAATTCTTATAAAGAATCT

GAAATTAAAAGTCAAGTTTTGATTGAAGTTACAAAAATAAGTGAAAGAGATTTGAGTGCG

ATAAGGAATAGGAGGACCCACGCTCCATTTCTGACTCAGTGCATGGTTCTTATAAAAAGA

TCCTCCCAACAATTGTATCGTGATATCAGCAATTACTGGTTACGTCTTGTTGTCTTTATT

GCCATTGCTATAAGCCTAGGCTCTATCTTCTACCAAGTCGGTTCAAGTACTCGATCTATT

CAGGTCAGAGGATCGCTACTTTCATTTTTTATGTCAGTCTTGACTTTCATGACACTTGTT

GGTGGATTCTCTCCCTTGATTGAGGAAATGAAGGTGTTTAAACGAGAGAGATTAAATGGG

CACTATGGTATTACTGCTTTTCTGATTGGCAATATATTATCTTCTATTCCATACATGCTA

ATGATCTCTCTCATTTCCGGATCAATAGTGTGTTACCTTTCTGGACTACACAAAGGACTA

GAGCACTATCTATACTTTGCTTCTATCCTATTTGCCATTATGATGTGGGTTGAGAGCCTT

ATGATGGTTGTGGGGAGTATCTTCCCAAATTTTGTGATGGGGGTGATCATTGCTGGTGGA

GTTGAAGGACTTGCGATTTTAACAGGTGGATTCTATCGAATTCCTAGTGATCTTCCAAGG

CCATTATGGAAGTACCCCTGCTACTACATTTCCTTCCTCACATATGCTTTCCAAGGATCA

TTCAAGAATGAATTTGAAGGCTTAACATTTGTTGGTTATCAAGAAGGAGGTACCATAACC

GTTAGTGGTAGAGATGCACTAACAGATATATGGCATGTGCAAATGGGTCACTCGAAGTGG

GTTGATCTTGTTATCATGTTTGGTATGATTGTTGTTTATCGAGTTCTCTTCTTGGTCATC

AATAAGGTCAAGGAGAAGTCAAACCCTACTGTTTCTAGCATAAATCGACCCCAAGCAAAA

ACTTTCTCTAGAACGAATATGGATGAACTCTAA

>MS.gene068047.t1

ATGGAAACATTGATTTGGAGGGCTATCAAGTTGCCTATTTATTCTGTTGCGTTGGTCCCT

CTTACTGTAGGCAGTGCAGCAGCTTATTTACAGACAGGCATCTTCTCAGCTAAATGCTAT

TTTGTTTTATTGGCTTCTTCAGTTCTTGTTATTACCTGGCTCAACTTGAGCAATGACGTT

TATGATTTTGACACTGGAGTTGACAAGAACAAAAAGGAATCAGTAGTAAACTTGGTTAGA

AGCCGTACAGGAATCTTTGTTGTTGCTTACTTATGCCTTGCTCTCGGCTTCATTGGGCTG

ACTTGGGCCGCTATTGAGGCAGGAAACCTGCGTTCAGTATTGTTTCTCACTTGTGCAATT

ATTTGCGGCTATATATATCAGTGTCCACCATTTCGGTTGAGCTATCAGGGACTGGGAGAG

CCTCTGTGTTTTGCAGCATTTGGTCCTTTTGCCACTACTGCTTTTTATTTAGTACAAGGC

AGTGCAAGTAGCGTGACGAACCATTTCCCCGTGAGTGGAACAGTTCTTTCAGCATCAATC

CTCGTTGGCTTCACAACATCTCTTATCCTTTTTTGCAGTCACTTTCATCAGGTGGATGGA

GACGAAGAAGTTGGAAAATTATCGCCTTTGGTTAGACTTGGCACTGAAAGAGGTGCAGAG

GTAGTGAAAGTAGCAGTCTTGTCGCTCTATGCTCTTTTGGTTGCTTTTGGTCTAAGCAAG

GCACTTCCTCTCACTTGTATTTTCCTTTGTGCATTGACATTACCGATCGGAAACCTGGTA

GTTAAATTTGTCCAAGACAATCACAAGGACAAAGATAAGATTTTCATGGCCAAGTACTTT

TGTGTGAGGTTGCATGCTTTGTTTGGTGCTGCACTGGCTTTTGGATTGGTATTGGCTAGA

ATGGTTAACAGAAAGCTACTACTCCAATGA

>MS.gene068040.t1

ATGTGGGAATCAGAAAGTGAAACAGCAGCTGGAAGGGAATATGGGGGTGGAGTTCTCACC

TCAACCAAACATGGTGTTAAGATTGAAGGTTTTCATCAAAGAGGCAACTCATGGTATGTT

TCAGCTGATATTCCAAGTGATCTTCAAGTCCAAATTGGAGAAGCCAGCTTCCACTTGCAC

AAGTATCCTTTGCTATCTAGAAGTGGAAAGCTGAACAGAATCCTATATGATTCTCGCGAC

CCCGACTTGAATAAGATAGCTATGGATGATCTCCCTGGAGGGCCTGAAGCATTTGAACTT

GCAGCCAAATTCTGCTATGGAGTTGCTATTGATTTGACAGCAAGCAACATTTCCGGCCTA

AGATGTGCTGCTGAGTATCTTGAAATGACTGAGGATCTAGAAGAAGGAAATCTTATATTC

AAGACTGAAGCTTTTCTTAGCTATGTTGTTTTGTCTTCATGGAGAGACTCTATAGTAGTG

TTGAAAAGCTCTGAAAAGCTGTCACCGTGGGCAGAAAATCTTCAAATTGTCCGAAGATGC

AGCGAGTCTATAGCTTGGAAAGCTTGTGCTAATCCAAAAGGAATAAGGTGGGCGTATACC

GGAAGAGCGACGAAAGCTTCAAGCCCAAAATGGAACGAAATGAAAGACTCAAGCCCGAGC

AGGAATCAGCTAGTGCCTCCTGATTGGTGGTTTGAAGATGTTTCAATCCTTAGGATTGAT

CACTTTGTTAGAGTCATTACTGCTATTAAGGTAAAAGGTATGAGATTTGAACTGATTGGT

GCTGGAATAATGCATTATGCAACTAAATGGCTACCAGGACTGGCGGGGAACGACACTACA

ATCCAAGGAGAGGAAACAAGCAACAACAGTAATAGTAGTTTTAGTAGCGGTGACAACAAC

AGTAGCTGGAGAGGTGGACTTCATATGATTGTTTCTGGAACTAGAGATGAAACTTCAAGT

CTTCAAACCAAGGATCAAAAGATGGTTATTGAGAGTCTCATCAGCATAATTCCGCCGCAG

AAAGACAGTGTCTCGTGTAGCTTCCTTCTCAGGCTTCTGAGAATGGCAAACATGTTAAAG

GTAGCATATGCATTGATTACTGATTTAGAGAAAAGGGTTGGAATGCAGTTTGAGCAAGCT

ACACTTTCTGATCTTCTAATTCCTTGCTTTGAGAAAAGTGAAACTATGTATGATGTGGAT

CTTGTTCAGAGGTTGTTGGAGCATTTTCTTGTTCAAGAACAAACTGAAGGTTATAGTCCA

AGTAGACAATCCTTTTCTGATCACAAGCATGTGGGAGGTAACCTGAATGCCAAGGCAAGA

GTGGCAAGGCTTGTGGACAGTTATCTTACAGAGGTATCAAGAGATAGAAACCTTTCATCG

ACAAAGTTTCAGGTTCTTGCAGAAGCTTTACCTGAGTCGGCTAGAGTTTCTGACGATGGA

CTATATAGAGCAATTGATTCATATCTCAAGGCACATCCAACACTAACTGAGCATGAAAGG

AAGCGTCTCTGTCGTGTAATGGATTGTCAAAAACTCTCCATTGACGCCTGCATGCACGCA

GCTCAAAATGAACGACTGCCATTAAGGGTAGTAGTGCAAGTTCTATTCTCTGAACAAGTA

AAGATAAGCAACGCACTAGCCAACAGCTCTCTAAAAGGCAGCGTTGAATCTCAGTACCAA

CCAATGGTAACAAACAGGAAAACACTTCTAGAAGGGACACCGCAATCTTTCCAAGAAGGA

TGGACAACAGCTAAGAAAGACATTAACACATTAAAGTTTGAACTTGAGAGTGTGAAGACA

AAATATTTGGAGCTTCAACATGATATGGAAAATCTGCAGAAAAATTTCGATAAGCTGTTG

AAGCAAAAGCACACATCAGCTTGGACTAGTGGGTGGAAGAAACTGAGCAAACTTACTAAA

ATGACAAATGTGGAAAATCATGATATGGTTCCAATTTCTGAAGAACCGAACAGAAAGACA

ACTAGAAGGTGGAGAAACTCAATATCTTGA

>MS.gene068045.t1

ATGATTTATGTGCGTGGCTCAGATTTGGCATTTGTTCGGCTGAGCTCAAAATTCCCTTCA

TGTCGCAATGCGGTATGTAGAGTTAAAGCTGCGTTGACCTCTGGAGGAGGAGACCTAAAA

AATGAAGATCTGGTTGGCGTTGAATCAGCACAACTTAATGCGGTAGCGCTTGGAACACTG

GGAGCAGATACAGCTCTAACAGGCAGTGGCTTTTCTGATGATAACGATGACTTCGATTCA

GACAGTCCAACAAAAGGTTTTGCTTCCATACCTGAGGCTATTGAAGACATTCGGAATGGA

AAGATGGTAGTGGTTGTAGATGACGAGGACAGAGAAAACGAAGGAGATCTAATAATGGCA

GCACAGTTGGCAACACCTGAGGCTATGGCTTTTATTGTGAAGCACGGAACTGGCATAGTT

TGTATAAGCATGAAAGAGGAAGACCTGGAGAGATTAGAGCTTCCTTTGATGGTCAACAGT

CGGGATAATGATGAGAAACTCCGTACAGCATTCACTGTGACAGTGGATGCCAAATATGGT

ACCACCACAGGTGTGTCGGCTCAGGATAGGGCAACCACAGTTTTGGCCCTTGCATCCAAA

GATTCAAAACCAAGTGATTTCAACCGCCCAGGCCATATTTTCCCGCTAAAATACAGGGAA

GGTGGTATCTTGAAGAGAGCTGGACATACAGAAGCTTCAGTTGATCTTGCAGTACTTGCT

GGTTTGGATCCTGTGGCAGTTCTGTGTGAGGTTGTAGATGATGATGGTTCCATGGCTAGA

TTACCTAAGCTTCGCCAGTTTGCAGAGCGTGAAAATTTGAAAATTATATCTATTGCTGAC

TTAATAAGGTATAGAAGGAAGAGAGATAAACTAGTGGAACGTGCTGGTGCTGCACTAATA

CCAACAATGTGGGGGCCATTCACAGCTAACTGTTATAGGTCACTTTTAGATGGGATGGAG

CATATTGCGATGGTAAAGGGTGACATTGGGGATGGATGTGATGTTCTTGTGAGGGTACAC

TCAGAGTGTCTCACAGGAGACATATTTGGATCTGCCAGGTGTGACTGCGGAAATCAATTG

GCTCTTGCAATGCAACAGATTGAGGCTGCTGGCAGAGGTGTGTTGGTATATCTTCGAGGA

CATGAAGGAAGGGGTATTGGATTGGGCCACAAGCTTCGTGCTTATAACCTACAGGATGAG

GGAAGGGATACTGTAGAAGCCAATGAGGAGTTGGGATTGCCTGTTGACTCAAGGGAGTAT

GGAATTGGTGCACAGATATTGAGGGACTTGGGTGTTCGTTCTATGAAGCTAATGACAAAC

AATCCGGCAAAATATGTTGGGCTCAAAGGTTATGGTTTGTCCATTTCAGGTAGGATCCCA

TTGTTATCACTTATCACAAATGAAAACAGGAGATACTTGGAAACCAAACGTGTGAAAATG

GGTCACATGTATGGCTTGGAATTTAACAGCAAATTGAATGGTAATGCCAGTAATATTGAT

GATTCCAGTACTGCTCCTGGCACATAA

>MS.gene068038.t1

ATGGGGAGTGTTACTGAAGGAAAGTTAAGATTTTGTATCGACAGAGGCGGTACCTTTACT

GATGTTTATGCTGAAATCCCGGGCCATCGGAATGGGATAGTATTGAAGCTTTTGTCAGTT

GATCCTTTGAACTATGACGATGCACCGGTGGAAGGGATACGGAGGATTCTGGAAGAGTTT

ACCGGTGAGAAAATTCCGCGTAGCTCGAAAATACCTACTGAGAAGATTGAGTGGATAAGG

ATGGGTACAACTGTGGCAACCAATGCGCTTCTAGAGCGTAAGGGAGAAAGGATTGCTGTG

TGTGTGACTAGAGGCTTTCGTGATTTGCTTCAGATTGGTAACCAGGCTCGTCCGAGTATA

TTTGATCTTACTGTGTCAAAGCCTTCGAATCTTTATGAAGAGGTTGTTGAGGTGGAAGAG

AGAGTTGAACTTGTTCAGGCAAAGGAAGAAGAAGAAAGTCTGAGTGCTTCTTCGCCAATT

GTTAAAGGTATTTCGGGAGAGCTTGTTAAGATTGTAAAGCCTCTTAATGAAGAAGCGTTG

AAGCTTGTACTGAAAAATTTACTGGAGAAGGGAATTAGTTGTCTAGCGGTTGTTTTGATG

CACTCGTACACTTATCCTCAGCATGAACAACAGGTGGAGAGGTTAGCTTTGAGTCTGGGA

TTTAAACATGTTTCGATATCATCTGCTTTGTCTCCCATGGTTCGTGCTGTTCCTCGTGGT

CTAACAGCTAGTGTGGATGCTTATTTGACCCCAGTTATTAAAGACTACTTGTCAGGATTC

ATTTCCAAATTTGAAGAGGGGCTTAGCAAGTTGAATGTTTTGTTTATGCAATCAGATGGA

GGACTTGCGCCAGAGAGTACCTTCTCGGGGCACAAAGCAATTCTGTCTGGCCCTGCTGGT

GGAGTTGTTGGTTACTCACAAACTCTTTTTGGTCTTGAAACAGAAAAGCCATTGATTGGA

TTTGATATGGGAGGTACATCCACGGATGTGAGTCGATATGCTGGAAGTTATGAGCAAGTG

CTGGAAACCCAAATTGCTGGTGCCATAATTCAAGCACCTCAGCTTGACATAAACACTGTG

GCTGCTGGAGGTGGTTCAAAGTTAAAGTTCCAGTTCGGTGCTTTTCAAGTTGGACCAGAA

TCAGTTGGAGCACACCCAGGTCCGGTATGTTATCGAAAAGGTGGAGAGTTGGCAATTACA

GATGCCAATCTTGTTCTCGGTTATGTCATTCCTGATTATTTCCCATCCATATTTGGCCCA

AATGAGGACCAGCCTCTTGATGTCAAGTCAACAAGAGAAGAATTTGAGAAGCTTGCTGGT

AATATAAATGCTTATCGGAAGAATCAGGATCCATCTGCAAAAGACATGACGGTCGAAGAG

ATTGCACTTGGCTTTGTGGATGTTGCTAATGAGACAATGTGTCGTCCAATAAGGCAGTTG

ACTGAAATGAAGGGCCATGAGACAAAGAATCATGCACTTGCTTGCTTTGGAGGTGCTGGA

CCTCAACATGCTTGTGCTATAGCCAGGTCTCTAGGTATGAAAGAAGTGCTTATTCACAAA

TTTTGTGGGATTTTAAGCGCTTATGGAATGGGATTGGCAAATGTTGTAGAAGAAGTGCAG

GAGCCTTATGCTGCGGTTTATGGAACTGAATCTACTCTTGAGGCTTCACAACGAGAAGCT

CTGTTACTGAAACAAGTTAAGCAGAAGTTACAAAGTCAAGGATTTAAAGAGGAAAATATT

TCAACAGACACATATTTAAACTTGAGATATGAAGGCACAGACACTGCCATCATGGTCAAA

AGGAAAATAGTTAAAGATGAAATACCATTTGACTATGCTACCGAGTTTGTGAGCTTGTTT

CAGCAGGAGTATGGTTTTAAACTCCAGAACAGAAATATTGTGATTTGTGATGTTAGAGTT

CGTGGTACAGGAGTTACCAATATATTGAGACCACAAGCCATAGAACCTGCCTCTGGGAGT

CCTATAATTGAAGGCTACTATAAGGTTTACTTTGGAAATGGTTGGCAGGAAGCACCTCTC

TATAAGCTTGAAAAGCTGGGGTATGGACATACGATGTCTGGCCCAGCAATCGTCATGAAC

GGTAATAGCACTGTGATTGTAGAACCCAATTGTAGAGCCATTATAACCAAATATGGAAAC

ATCAAAATTGAAATTGATTCACCTGTGAGCAGCATTAAAATATCAGATAAAGTTGCAGAT

GTTGTACAACTCTCTATTTTCAATCACAGGTTTATGGGTATAGCCGAGCAGATGGGAAGG

ACTTTGCAAAGAACTTCAATTTCGACAAATATCAAAGAACGGCTTGATTTTTCTTGTGCT

CTATTTGATCCTCACGGGGGCCTAGTTGCAAATGCACCTCACGTTCCTGTCCATCTAGGA

GCAATGTCTAGTACAGTTCGTTGGCAACTCAATTACTGGAACGACAATTTGAATGAAGGA

GATGTTTTGGTTACTAACCATCCGTCAGCCGGAGGTAGCCATCTTCCTGATATAACTGTT

GTTACACCTGTTTTTTTTAATGGGAAGTTGGTATTTTTTGTTGCAAATAGAGGCCATCAT

GCAGAGATAGGGGGTATTACTCCTGGAAGCATGCCTCCATTTTCAAAGTCCATATTGGAG

GAAGGAGCTGCCATTAAGGCGTTTAAGCTTGTTGAAAAAGGTGTCTTTCAGGAAGAGGGG

ATTGTTAAACTTTTACAGTTCCCAAGCTCCGATGACCGTGGAACTAAGATTCGAGGAACT

CGCAGGATTCAAGATAACCTATCTGATTTGCAAGCACAAGTAGCAGCAAATCAAAGAGGA

ATTTGTCTAGTCCTGGAGCTTATTGAACAATATGGACTGGAAACTGTTCAAGCTTACATG

AATTACGTACAGATGAATGCAGAAGGAGCAGTAAGAGAGATGCTGAAGTCAGTTGGTCGT

AGAATTTCATCTGAGTCAAATGAAAACTCTGTGACAATTGAAGAAGAGGACTACATGGAT

GATGGATCTGTTATTCATTTGAAACTGAGCATTGACTCTAACAAAGGAGAAGCAATTTTT

GATTTTGGTGGGACAAGTGCAGAAGTTTATGGTAATTGGAATGCACCAGAAGCCGTAACA

GCTGCAGCTGTCATTTACTGCATTCGCTGTTTAGTGGATGTTGATATTCCTCTCAATCAA

GGCTGTTTGGCTCCGGTGAAGATTCATATTCCAGAAGGCTCATTTCTTTCTCCTAGTGAT

AGTGCAGCTGTAGTAGGAGGCAATGTCCTTACATCTCAGAGAATCACCGATGTTGTATTT

ACTGCATTTCAGGCTTGTGCTTGTTCGCAAGGTTGTATGAATAATCTTACATTTGGAGAT

GACACTTTTGGGTACTATGAAACCATTGGAGGTGGAAGTGGGGCTGGTCCTACATGGGAA

GGGACCAGTGGGGTACAGTGCCACATGACAAATACAAGAATGACTGATCCAGAGATATTT

GAACAAAGATATCCAGTGATTCTGCACAGATTTGGACTTAGAACGAACAGCGGGGGAGAT

GGATTTCACAGAGGTGGTGATGGGCTTGTCAGGGAAATAGAGTTTAGGCGCCCAGTTACC

GTCAGCATTCTTTCCGAGAGACGTGTCCATGCACCTAGAGGGCTGAAGGGAGGGAACGAC

GGTGCACGTGGTGCTAATTACATTCTAAAGAAAGATAAGCGAAAGGTTTATCTTGGTGGT

AAGAATTCTGTTGAGGTGCTTCCTGGCGAAATTCTTCAGATTTTGACTCCTGGGGGTGGT

GGATGGGGCTCTCCAGTGTAA

>MS.gene068037.t1

ATGGCATCAAGCAAATCCTTCATCACAACTTTACTTCTGGTTGTGACAATGTCAAGCATG

ACCTTAGAAGCTCGCCATCTATTGCAAACAACAACACAGCCAAACTTACCAACCATTCCC

ACTTTGCCAAAACCAACAACACTGCCACCTTTGCCTTCAATTCCAAATATGCCTCAGGGA

AATCTTCCTCCATTGCCTACTACTATTCCATCTCTTCCTAAGCTCACCATGCCACCACTT

CCTAGCTTTCCTACCAATATTCCAACACTTCCATCTCTCAACATTCCACCACTACCAGCA

GCCACTTCACTTCCAAACCTTCCTTCAATTCCAACCACTTTCCCCTCCATCCCATTTCTC

TCCCCACCACCTTCAACATCTAACCCTTAA

>MS.gene068010.t1

ATGGCTTCTTCATGGTTTCTCAAACTGGCTTTCAAATGCCTTTATCATTTTGCATGGCCT

CTTTTTGCTCTGGTGTATCCTATGTGTGCTTCCGTACAAGCAATTGAAACTGACTCATAT

GCAGAAACCAAGGATTTGATCTCATATTGGATACTTCTTTCCTTAATTTACCTCTTTGAA

TATGCTTTTATGAGCCTTCTTCTACGGTTTCACCTCTGGCCATACGTTAAGCTAATGATC

ATCTTCTGGCTCATCATACCGGACTTTGGACGAGCTTCTTATATTTATAATAAGCTTATT

AGTTCCATGAAACCGCAAGTAGTCACGTGGAGGTTAAACAACTACTGGAGGAAGTGGTTT

TGTAAGAAAGATAATTTTTTAATGCATGCAGAGAAATATATGAAAGAGAATGGAACTGAA

GCCTTAGAGAAACTCATTGCCAACAAGAACACAATGTGTAGGTCTGATGCAGAAGCGACA

AATGAAATTATGGCCACTGATAATAAAGAAATGCTGAAGACAAATGGAGAAAGACTCCAA

ATTGAGCACAAAGATATAAAAGATTTGGAGGCAATTGAGAAAAAAGAAATTCCTGCTACC

AAGCAAGATATTCCCGTCATGCCTAAAATTGGGCCAAGTCAAAATGCGTCATCAGCCACA

GTGGAAACCAAAGGAACAGCAGAGATTAACAAAGCTGGTGGAGAGGTTAGTCAGAGTTCT

ACACAGAAGGAAGTGCAGAAAGAGTGGACTTGTGCTTTATGTTTGGTAACAACATCAAGC

GAGAAAACCTTGAATTGTCATCTCGGTGGAAAAAAACACAGGGATAGTGTTGAAACATTA

ATATCAAAGAAGCAACCTACACTACATAAGCAAAAGGATGCAAAAGTGACAAATAAAATC

ATAGCTATTGATAATAAAGAAATAGTAAAGACAAATGGAGAAAGACTCCAAACAGAGAAC

AAAGGCATCAAAGATTTGGAGGCCATTGAGAAAAAAGAAATTCCTGCAACTAAGCAAATA

AACTACCCTAATATTGTGGCAAGTCAAAAGGCATCATCAGCCATAGTGGAAACTAAAGGA

ACAGCAGAGAGCGGCAAAGCTGGTGGCAAGGTTCCTCAAAGTTCTAATTCTACACAGAAA

GTGTGGACTTGTGCTTTATGTTCGGTAACAGCTACAAGCAAGAAAGACTTGAATTCCCAC

CTTAATGGGAGGAAACACAGAGATGCTAGTGAAGCAGCTTTGAAAGCAAAGAAACAACTT

GCTCTGCAGAAGCTGAAAAATTATCAGTCCAAAGAAGAGGTGAATCAGCTAAATGTTAGC

AATAAGCTAAATTCTAATGTCAAGAATGGAGAAGATATTCTTAACAAAGGGTTGAAGGGA

ACAGTGATGATGGATGATAAGGTGCAAAAACTGCAAAAGAAGGTGCAAAAACTGCAAAAG

AACCTGTGTGAGCCTGTTGGGATGCATAATTCCAAATTAATATGTAGTGTCTGTAATGTT

GTCCTTCATTGTGAGGCCAATGTGGCCTCTCACTTAAATGGGAAGATGCATTTGGCTAAA

ATGCAAAGCAAAGTTGATAGATTAAATAACCTTAGGGACATTTGCACTGCTTGA

>MS.gene068048.t1

ATGGCTACCTTTAATCCACGTATTCAACCAATGGTTTTGCAAACTAACTCACAAGTTAAT

TTGACCAAATTTGAAGGTGACCATGTCCTAAAGTCAATAGAAATGATTAGTGGAAATGTT

GAAGGAGGGAATAAGGATGGAAGCATAACAGTGACTTGGGAGAAACTTAGGGTCACTGTT

CCAAATGGACGCAAAAGGAAACCTATTCTTCAGGGTCTTACTGGTATTGCCCAACCTGGA

AGGCTTTTGGCTATTATGGGTCCTTCTGGCTCTGGAAAATCCACACTTCTTGACGCTTTA

GCAGGAAGATTAAGCTCAAACATGAAGCACACAGGAAATATTCTAATCAATGGCCACAAG

CAAGCACTGGCTTATGGAATATCAGGCTATGTAACACAAGACGAGGCTATGCTATCAAGA

TTAACGGCCAGAGAAACTTTATACTACTCAGCTCAACTTCAATTTCCAGATTCCATGTCC

ATAACAGAGAAGAAGGAACAAGCAGATCTTATACTCCGAGAAATGGGACTAATAGAAGCT

GTTAACACAAGGGTTGGAGGATGGGGTTCTAAAGGTCTAAGTGGAGGACAAAGGAGGAGA

CTTAGCATTTGCATCGAGATTCTAACACACCCAAGACTTCTCTTCCTTGATGAACCGACT

AGCGGACTTGACAGTGCAGCTTCCTACTATGTTATGAGTAGGATTGCACGTTTGAATTTA

AGCGACGGAATTCAAAGGACTATTGTTGTATCCATCCATCAACCTAGCAGTGAACTTTTT

GAACTTTTTCATGACCTTTGTCTTCTGTCTTCTGGTGAAACTGTTTATTTTGGTCCAGCC

TATGATGCAAATCAGTTTTTTGCTGCAAATGGTTTTCCTTGTCCAACTCTACACAATCCT

TCTGATCACTATTTAAGGATCATAAACAAAGATTTCGAAATGGATGTTGAAGAAGGATTA

GGTAAGGGAGTAACCACAGAAGAAGCAATTGGTATCCTTGTAAAGTCTTATAGATCATCT

CAAATTAGAAACCAAGTTAAAAAAGAAGTGAAAATTATAAGTATAAGTGACTCTGGTGCA

ATAGGAAAGAAGAGGATTCATGGTGCATTTACCACTCAATGTCTTGTTCTAATAAGAAGA

TCTTCTTTGCAATTGTTTCGGGACATAGGCAATTATTGGCTACGTTTAGTCGTCTTTGTT

ATGATTGCTATAAGCATTGGTTCTATCTTTTATGGCATTGGTACAAGTAGTGGCTCAGCA

TCTATTCAGGGAAGAGGTTCTCTGCTCACATTTCTTGTTTCAGTTCTGACTTTCATGACC

CTTGTTGGTGGATTCTCTCCTCTTTTGGAGGAAATGAAGGTATTTGAACGAGAGAGATTG

AATGGACACTATGGTGTTACTGCTTTTCTGATTGGAAATATATTCTCTTCTCTACCTTAC

ATAATAATGATCTCTGTGATACCTGGAGGAATTGCATACCAACTTTGTAAAATGCACAAA

GGACTAGAACATTTTCTATACTTCATTTCATTGCTAATAGCCATTGTAATGTGGGTTGAA

AGCCTTATGTTGGTTGTTGGAAGCATCTCTCCAAATTATGTAATAGGAATGTTCATTACT

GGTGGAATTGAAGGACTCATGATTTTAACAGCTGGATTCTATAGACTACCAAATGAACTT

CCTAAACTACTATGGAAATACCCTTTATACCAAATCTCTTTCCTTAAGTATGCATTTCAA

GGATCATTCAAGAATGAATTTGACGGGTTAAAAGTTGCTGTGGGGACCAACATGGTGAGT

GGTAGGGAAATATTGACTGATAAATGGCATGTTGAAATTGGTCACTCTAAGTGGGTTGAT

CTTGCTATCATGTTTGGCATGATTGTGTTGTATAGAGTTCTATTCTTGGTCATCACTAAG

AGCAAGGAGAAAATAAGTGGTCCTTAA

>MS.gene068015.t1

ATGGCGGCGGTGTCCAACGACGGCGCCCCGCTAGGATCCACCGTAATCTCACTAGTCAAC

AAACTTCAAGACATCTTCAGCCGTGTCGGAAGCCAATCCACCATCGACCTCCCTCAGGTT

GCCGTCGTCGGCAGCCAGAGTAGCGGCAAGTCTAGCGTCCTCGAAGCTCTCGTCGGCCGT

GACTTCCTCCCCCGTGGTAACGATATCTGCACACGAAGACCCCTTGTTCTTCAACTCGTT

CACATTCCACCTTCTAAACCCGAATCAGCTGAGTTTCTTCATTTACCTGGCCGCACTTTC

CATGATTTCTCCCAAATCCGCGCTGAAATTCAGGCTGAGACTGATAGGGAAGCCGGAGGG

AACAAAGGTGTTTCTGATAAGCAAATTCGTTTGAAGATTTTTTCGCCGAATGTTCTTGAC

ATTACGCTTGTGGATCTCCCTGGTATTACCAAGGTTCCCGTTGGTGATCAACCTTCAGAT

ATCGAATCGAGAATTAGAACTATGATTATGTCATATATCAAAGTTCCTACTTGTCTTATT

CTTGCTGTTACACCGGCTAATTCTGATTTGGCGAATTCCGATGCTCTTCAAATGGCTGGA

AATGCTGATCCCGATGGTCATAGAACTATAGGTGTCATAACCAAGTTGGATATCATGGAC

AGAGGTACCGATGCTCGGAATCTGTTACTGGGAAAAGTTATTCCCCTCCGACTTGGTTAT

GTCGGTGTTGTAAATCGTAGCCAGGAGGATATTCTGACAAACCGGAGTATAAAGGATGCT

CTGGTTGCTGAAGAGAAGTTCTTCCGCAGTCATCCTATATATAGTGGTCTAGCTGATAGT

TGTGGCGTTCCTCAATTGGCAAAGAAGTTGAACAAGATTCTTGCACAACACATCAAGGCT

GTGCTACCAGGGCTGAAGGCACGAATAAGTGCTTCACTTGTCAATCTTGCAAAGGAACAT

GCAAGCTATGGAGAAATCACGGAGTCCAAGGCTGGACAGGGTGCACTTATCCTGAATATT

CTTTCAAAATACTCTGACGCATTCACCTCCATAGTAGAGGGAAAGAATGAGGCGATGTCT

ACATCTGAGCTATCAGGTGGAGCACGGATTAATTACATTTTTCAATCCATATTTGTGAGG

AGTTTAGAGGAAGTTGATCCGTGTGAAGACTTGACCGATGATGATATTCGTACCGCCATA

CAGAATGCAACTGGACCTAGAGCAGCATTGTTTGTTCCAGATGTTCCGTTTGAAGTCCTT

GTACGGAGGCAGATATCTCGGCTATTGGATCCAAGTCTTCAATGTGCCAGGTTTATATAT

GACGAGTTAATGAAGATCAGCCATCGTTGTATGGTGACTGAACTGCAGCGATTCCCTTTC

TTGAGAAAACGCATGGATGAAGTTATTGGGAACTTTTTACGAGAAGGCCTTGAACCCTCA

GAGACAATGATCACCCATATCATAGAAATGGAGATGGATTACATAAACACTTCCCACCCA

AATTTTATTGGTGGAAGTAAAGCGTTAGAAGCTGCAGTGCAACAGACCAAGTCTTCTACA

GTTTCTAAGGTGAAGGATGCTGTGGAATCTGATAAAGGATCAGCTTCTGAAAGAAGTGGA

AAGTCTCGATCTATTCTTGCAAGACATGCCAATGGAGGAATGGCTGATCATGGTGTGCGT

GCTTCATCAGATATCGATAAAGTATTACATTCAGGAACCACTGGTGGATCTAGTTGGGGG

ATCTCATCTATTTTTGGTGGAGGAGATAACCGTGTGTCTGTGAAGGAGAATACAAATAGT

AAACATCATAATGATCCAGTTCAAAGTGTGCTTCCATCCTCTACAATTCATTTGAGAGAG

CCCCCTACTATCTTGAGGCCATCAGAGAGAAGTTCAGAGACGCTAGCTGTTGAAATTACA

GTAACAAAGTTGCTTTTGAGATCATATTACGAGATTGTCAGAAAAAATGTTGAGGATCTT

ATTCCTAAGGCGATCATGCACTTCTTGGTAAATAACACGAAGAGAGAGCTGCACAACGTC

TTCATCGCAAATCTATATAGAGACGATCTGTTTGAAGAGATGTTGCAAGAACCGAATGAG

ATAGCCGTTAAAAGAAAGCGCTGTCGAGAACTGCTCCGAGCTTATCAACAAGCTTTTAGG

GACTTAGACGAACTACCTCTTGAAGCTGAAACGGTTGAGTGGGGACACAGTTCGCCTGAA

ACAACTGGCTTGCCTAAAATTCGCGGATTGCCAACTTCTTCTATGTATTCTACTAGCAGT

TCAGGAGACAATATCTAA

>MS.gene068013.t1

ATGGAGAAAATGGGTGGGAGTGGGAAGAGATTTGGGGTGTTGTTATGTGCAGATGATTCA

GAATATGTGAAGAAAATGTACGGAGGGTATTTTGGGGTATTTGTGAGAATGTTGGAGGAA

GAAGGTGAAAACTGGGATGTGTATAAGGTGGCGCGTGGAGAGTTTCCAAAGGATGATGAA

TTGAATCTGTATGATGGTTTTGTGATCACTGGTAGTTGTAGTGATGCACATGGAAATGAT

ACATGGGTCTCTCAACTTCTCAATTTGCTTAAGAAATTGAATGACATGAACAAGAAAATC

CTTGGCATTTGCTTTGGTCATCAGATACTTGGCCGTGCAATTGGAGGGAAGGTGACTCGA

TCTCCAACTGGCTGGGACATTGGTGTCCGAAACATAACATTATCACCCTCTTTGCCATCT

CCTTTGTCTTCTCTTGAACTTCCAACGAGGCTTTCCATTATTCAATGCCACAGGGATGAG

CTAAGAGAGCTACCTGCCAAGGCTGAAGTGATTGCTAAATCAGATAAGACTGGAATTGAG

ATGTTTAGATATGGAGATCACATTATGGGAATCCAAGGTCATCCTGAGTACTCTAAAGAC

ATTCTTTTGAACATTATCGACCGTCTTATTCAGCGAAATTTCATCACGGAAAATTTTGCT

ATGAGATTGAAGGAGAAGGCTGGTATGTGGGAGCCAGATAAGGAGGCATGGAAGAGACTA

TGCATCAGCTTCCTCAAGGGTCGTTATTGTGAAAGAAATGGAATAGAAAATATAAATATT

GGTGGTATATTCTAA

>MS.gene068008.t1

ATGTTTTTGATGTTAGGCTTTTGTTTCAAGATTCTTCACTTCGGTTGGCATTTTAAGGGT

GCCCTACGTTTTCTCTGTGATTTTGGACGCATTCCCACAATTCGTTTATGTTTGGAAAAC

TTTGTTTTGGAAGTTTCTAAGAAAAAAATCCCATCTTTGGAAAATGGGTCTCACCCTAAT

ACCAATTCTTCGACTCAGAAGAGATTGAATGGTCGTTTGGAAGATCGGTTGGAAAGGAAA

GATGATAGTGAAATGCAGGTTCCTGATGTGGATGAGGTGTTTGATGTAATCACTCTGAGA

AAGTTGATAAAGATTGAGCGCAGGAAAGCTAATGCGGCGTTTGCGGAGCTTGATAAGGAA

CGGACAGCGGCTTCTTCATCTGCGGAAGAGGCAATGGCGATGATTCTCAGGCTTCAGAGT

GAGAAGAGCTCGGCTGAAATTCAAGCGAATCAATTCCGCAGAATGGCTGAACAGAAGTTA

GAGTATGATGATGAAGTGATCGAGTCTTTGGAATGGACGATCTCGAGGCATGAATCTCAT

AGGAGTGTGTTGGAAGAGCAGTTGAGGGTTTATAGAGAAGAACTCAAGCAATATCTTGGG

GAGGATGGGATAAACCAACTTGAAGCTGATGTTAGTAGAGATAGGAGTTTTGAGAATGAA

GCAGTTGATTCTGTAGTTAGCTCTTCTGAAAACGGATCACCGACCTTGTAA

>MS.gene071779.t1

ATGGCATCGGCTTTATTAGACAACCGTAACGAACCAAATTGGCTGCAACACAGAGGCGGT

GGAGCTGAATTCATGGGTAAAGCACCTAACCCTAACCCTAAATTTAGTAACAAAAAGAGA

ACTCAATCCCCATCAGATGACGCTTCCTCAATCAATCGACGATCCAACGATAATCATTCA

CAATACGTTACTTTCAACATCGAATCGTACACAAAGACGGAGCTATACGAGCTCAAAAAT

CGACTCGTATCAGAACTCGACCAAATTCGTCAGCTTAAGACTCGAATTGAATCAGGTGAG

TTTAAACCCAGGCTAAACCACAACGGTGGTGGTCCCACTAAGAAATCAGGGAGCAAGAAG

GTTTCCGGCAACAAACGACCTTTTCCTGCGGTGAAGGAATTGAAGAAATCAAAGTCAGAG

ATTGGGGATGCGATGAAGGCTTGTGGTCAGATTTTGACGAAGCTCATGAAAAACAAAAGT

GGTTGGATTTTTAACACTTGGATCTTTAACACTCCTGTAAATGCAACGGCTTTGAATCTT

CATGATTACTTTGATATAATTAAGCATCCTATGGATCTGGGTACTGTCAAGTCAAAACTT

GCTAAGAATGCTTACTCTACGCCGGCGGATTTTGCTGCTGATGTGAAGTTAACGTTCAAC

AATGCATTGACATACAATCCCAAGGGCCATGATGTTAACACTGCGGCGAAGCAGCTTCTT

GAAAAATTCGAGGAGCTTTATCGACCAATACACGAGAAATTTGATGAAAAAAGTTTTGAT

GATGAATTACAGGCTAGCTCGTGGAACCATGTTGAACCAGAGAGGGAAAGGGAGAGGGTG

AAGAAGAAAGATAATCCAATTCCAATCCCTCCACCGGTAGCAAAACGCCAAGAGCCACTG

CCTGAGCCTGCTAGTACTTCTAACCAGCCAAGCACTTCGAATCCCCCATTAGCTCAGTCA

CCAGTTCGCATACCCTCCCCAACTCGAGCACTTCCAGTGAAGCCCTTGAAGCAGCCCAAG

CCAAAGGCCAGGGATCCAAATAAGAGGGAAATGAATGTTGAGGAAAAACATAAGTTGGGA

CTAGGGTTGCAGATTTTGCCACCAGAGAAGATGGAACAAGTAGTACAGATCATAAGGAAG

AGAAATGGGCATTTGGAGCAAGATGGGGATGAGATTGAGCTTGATATGGAGGCTGTTGAC

ACGGAGACTCTCTGGGAACTCGATCGATTGGTTACCAATTGGAAAAAGATGGTGAGCAAG

ATTAAGCGGCAGGCGCTAATGGATAATAATAATGTGCCTTCAAACAAAGGCAATGGGGAA

TTGCCTGATAGGGAGAAGGTTGATGCAACCCCTCCTTCTGAGGGGAAGAAGCAGAAGAAG

ATAGATACTGTTGACGAGGATGTTGACATCGGCGATGATATGCCAGCAAACAACTTCCCA

CCTGTGGAGATTGAGAAAGATAAAGATATGGGTGCTACTGGTGGCGGTGCCAGTAGTAGT

TCCAGTGGCTCCAGCAGTTCTGGTAGTGATTCCTCGTCTAGTG
