# Supplementary material for: Identification of Genetic Loci Associated With Crude Protein Content and Fiber Composition in Alfalfa (Medicago sativa L.) Using QTL Mapping
Source: Front Plant Sci. 2021 Feb 18;12:608940. doi: 10.3389/fpls.2021.608940 (PMC7933732; doi:10.3389/fpls.2021.608940)
Supplement: Supplementary file 10 [file Table_8.docx]

### Table S8. Potential Candidate Genes of *qADF5C*

>MS.gene009876.t1

ATGGCGGTTTCCCGAGGAACAATGGAATCACGCATTGTTGCACTGGAACGTGGGTTTGAA

AGTCTGTTAGAAATGAGACAAACCATGGAAGAAGAACGGATTGAAGGCATTGAAATGCGC

AGACAAATGAATGATTTAATGGAAACTATGCGTGCAATAGTGGAACGATCTGCACAACAT

GATTCACACATGGATGATGATTCAACGTTGGATGGGGATAGTCCGCAACAGTACTATGGT

GTTCGAACTAGAGAAAAACACTCGAGAAACACGGAAGTTACAGTTTTTAATGGTGAAGAC

GTATTCGGGTGGACCAACCAAGTTGAGCGCCATTTTCAATCGAAAGAAATGAATGATACA

GAGAAGTTACAGGCAGTTATGGTAGCCATGGAAGGGAAGGCTTTGACATGGTATAAATGG

TGGAAGTTTTGTGCACAAAATCCAACATGGGATGACTTCAAATCTGCAGTCATACAAAGG

TTTCAACCGTCGATGCTGCATAGTCCCTTTGAATTACTGCTCAATCTCAAACAGACAGGA

ACGGTGAAAGAATACATAGAGCAGTTTGAATTATATGCAAGCCCTCTGAAATATAATGAA

CCGGCCTATTTGAAGGTAATTTTTTTGAATGGTCTTAAAGAAGGCATAAGGGATGAATTA

AAACTTCATCCACTGGAAGGATTATCTGAAATGATGGATTATGCACAAAGGATTGATGGA

AAGAACATTGCAGCGAACAAGGGAAGTGTTGGACTAAGTTCTATCGGAAAACCCATTAGA

ACCTGTAATAATTCCAGTACTGTCACTTGGGAATCTGGAAATAAAAGTCGTGTATCATCC

ACCAATAGGGCTGCATGTATTATGGATGAGACCAGTAGCACAAAAATTTCTGGATCTTTT

AGAGAAAAGGGATTTAGAGGATTATCTTATGCAGAAAGTGCACTTACCATTATCACAATG

GTGAAGATGAAGCATGGACGTACAATCTATTGCTCTAATAATGATGAAATTGTCCCACTT

CCACAACAAATTGATGCTTCAATTAGGCTGCAATGGGACCCTGGGGGGCACTTCATGTTC

ACCTTGAGGACAAGGCTGTTGTGGATTATTCTGAAAAGTAGAAAAGGGTTATTTGGAAGG

GAGAGACCAAGCTCTCTAATTCTTGGAGGGGAGAGAACCAAGACTCTCGAATTTCTTGGG

ATTCTATTCATGATCCTACGTTCTCACTTCAAAATCATGAGTTTGATCATGCTGCACACC

TCCAGCGATCCTGGCCGTATCACAGCTCAGAACCATGGCCGTGGATCACCGTGGATCGTA

GAATCATACGATCCTATATGGCAGCTCGCGAACTTGACTACATCTCTCTCAAGTCTCAAC

TTCTTATGA

>MS.gene009916.t1

ATGGAGAGATTATTATGGGACAATAATGGTCCAAGTGACGAGGACCAGAACTTCATGCTT

TTACCATCTGAGTTGAATTGCTTTCATGTGTGCTTCAAATTCTTGAAGAAGACTATTAAG

GAAAAATCCCAAAGCAACCCAAGAAGTTGCTCTAGAACTTTAGTAATTGTAAATGAAGCT

CATAACGATCTTCATGGAAAATACAGCCCAGGTTCCTTATTGAGGAAGCTGAAGACAAAA

TGGTTCCAGCTTGCCCGACTAAGCGATTATAAACACATTGTATTCATGAATGATTTAGAT

CTTGTGGAGCTGGAGAAAACTTGGCTCCAGCTTGGTCAAAACTATGAGGAACATAGCACG

ATCGTTTTAGAGATAGAGAAAGAGTGCTTGTATAAGTGCTTGGATATCTTCAACGAGAAT

AAGGACATAATTAAATTCAATGAAAACAAGTCTACAAGCCTAGGTTTCTCTGGAACTTCA

GTAGCTGTAAATGAAGATTGTTACAACTCTGGTTCCTTGTTGAAGAAGCTAACGAAAACA

TGCCTCCAGCTTTGTCTAGACGAAAAAGAAGAGACAGTCATTGATGGTTTAGATTATTTG

TATCTCAAGAGAGCATGGGACCAGATTGGCTTAAGCGACAAGGAACAAAGCTCAAGGCTT

TTGAAGATAGAAACAAAGTACTTGAAAATGTACATGGAATTCTTCTTTAAGAAGAAACTC

TTAACCAACCCAAGACGAGGTTTCACTGTAACTTCATTGCCTATAAATGAAGATCCTGAC

CATTGTATCGGAGAATACAGCTGCGGTTCCTTGTTGAGGAAGCTGAAGAAAACCTGTGTC

CAACTTGATGCAAACCATGATGCAGATTTTGTGGAGTTGAAGAATAAATGCGAACAGTAT

GCTCAAAGCTATGATCAACAGATCAAGTTGCTTACATCTATAGAGATACAGTGCGTGCGT

AGGTGCAGTAAATTCTTCAAGAATGAGCTTGTCAAGGTCATAGCCTGGGAGACAAATCTA

AGGAGATACTTGTATGCTGTTAAACTTGAAATTTCTACTCTATCATCAGCTCTTGGAGAA

AAGATGCCTGCTGGAATTATCCTTAAAATATCAGATGAGATAGCTGGGTTTTTGAAGCGT

AATGATGATAATGAGGATGACGTTCCTCCTAAATTTGATGACTATATCCTGTCCCAGAAC

AAGTTAGAAGAATATCGCACTTGGCTTCAAAAACTTCAAAATAAAAAGAACGAAAGGTTG

GAAAAAGTTTCTGGTTTTGTGAACACTGTGTGTGATCTATGTGCTGTTCTGGGTATGGAC

TCCTCCAGTATCTTAGCTGAGGTTCATCCAAGCTTGAATGATTTTACTGATGCTCAACCT

AAAAGCATAAGCATTCCCACGCTAGATAAACTTGATGGCAAGGTCTTGATGCTGAAAGAA

GAGAAAAAGCAGAGGCTTCACAAGTTCAAAAAATTAGCTTCTCAGTTAAATCGTCTGTGG

AATCAAACGGATACTCCTCGCGAGGAAAGGAATCTGGTTGACAATGTTACCTACACTATA

TCAGCTTCTGTCGATGAAGTCATTACTTCTAACGCCCTTGCTCTGGATCTGATTAAACAG

GCTGAAGTGGAAGTTGAGAGACTTGAACAGCTTAAAAGGGTGAAGGAGGAAGAGAACTCG

GAGAGATATATTCAGATGCTCTTATAA

>MS.gene009935.t1

ATGGGTTCTCTATGCGATTTTTGCGGAGACCAGAGGTCTTTGGTGTACTGCCGCTCCGAT

GCTGCTTCCTTATGTTTGTCGTGTGATCGAAATGTTCATTCTGCTAACGAACTTTCTAAG

CGTCATTCAAGAACACTTGTATGCGAGAGGTGTAATTTACAACCTGCATATGTGAGGTGT

GTTGAAGAGAAAGTTTCACTTTGTCAGAACTGTGATTGGTCAGCTCATGGTACCAATCCA

TCCTCTTCAACACACAAGAGGCAATCAATCAATTGCTTCTCTGGCTGTCCTTCGGCTTCC

GAGCTTTCTTCAATATGGCCCTTTTTCTCCGACATCCCTTCTACAGGTGAAGCATGTGAA

CATAAACTCGGTTTGATGAGCATTAACGAGAACAGTGACAACAGTGCCAGGGTTCCTCCA

GAAAGCAAGAGTGTGTCTAGTTCAGCTCAAGTGGCCGATCTTCCAAGCAAAAACAAATCT

GGCGTCGACACATCTTCAATACCCGAGTCAAGTGCCAAACCCCGTATTTTGAACCAGGCG

CCCGGATCTTCCAATGAACTTATGCCTAAGTTACTATGTCCCAGCAGAAAGGCTCCTGCA

CTATGTGAAGATGATAAGTTGCTTGGTGATTTCAACATAGATGAGATGGATTTTGAACTT

GAGAACTATACGGAACTTTTTGACTTTGCTCTCAACCACTCTGAAGAGTTTTTTGAAAAT

GGTGGTATTGATAGCTTATTTGAGAGAAAAGACATGTCTGCTAGTGCTGGTGATTCAAAT

TGTCAAGGCGCTGTTGCTGCTGAGGGGTCATCAGCAAGGTTTGTTAGTGCTATCCAACCA

GAATGCAGCAATGCTGCATCTGCAGATTCGATATTAAGTACTAAAACTGAACCAGTTATT

TATTTTACGGAAAGGCAATCAAACCTTTCATTTTCTGGCGTTAACAAAGATACTAGTGCT

GGTGACTATCAAGAGTGTGGTACTTCTTCAATGCTTCTCACAGGGGAGCCTCCCTGGTGT

CCTCCTTGCCCCGAGAATTCTATTCAATCTGCTAATCGCAGCAATGCTGTCATGCGTTAT

AAGGAAAAGAAAAAGAATCGAAAGTTTGATAAGAAAGTAAGATATGCTTCTCGGAAGGCA

AGGGCTGATGTCAGAAAACGTGTGAAAGGCCGGTTCGTCAAAGCTGGGGAAACATACGAT

TACGACCCTTTGAGTCAAACCAGAAGCTGCTGA

>MS.gene009906.t1

ATGCCAATGGCTTCCCGCTTCCTCTCCTCCCGTCACCTCCCAAAGGCTCTTGCTGCTCTC

GCGGTCTTTCCCACTGCTTACCTCTGTGTCAAGAACAAGAACAACACCTACGCCATCACA

CGCCCTCTCAACGCGCCCAAGAACGATCACCCCCAATTCAGTGTGTTTTTGCCTTGGCAT

ATGTTATATAGACTATATCAGACAAAGGAACGTCATTTTGTCCAGCAACTATTGGACCAC

ATTGATATGAGAGAGACGGAGGATAATCTGCTTCTCCGTCTGCATTTGTTACAACTCGAT

AGGGAAGATGTGAAGATCTATGTGGATCACAAAACTCTTACCATTAAAGAAGCTAATGGT

GGCAGTATCTCTCTTACCATTAAAGATGCTAATGGTGGTCGTATCTTTGGCATATTTCGT

GATAGAAGTCGATTCACTTGCACACTTGAGTTAACTGAGCAATATTGCAAATATCCGATT

ACAGCTAAGATGGAAAAGGGTATGCTCGAGGTTGTTGTGCCAAAAATGAATAATGAAGAG

GGTATGCTCAAGGTTGTTCTGCACGAAGGTGAGACGATTGATGTCAAGGTTGAATAG

>MS.gene009863.t1

ATGGAAAACCCTAGAAACAACAATTCAATGACGGAAGAAGAAGACGATCCCGGAAGTGCA

TTATCCTTCGACTCAACCGAATCAAGATGGGTTTTTCAAGAAGACGAGGATCCATCTGAG

ATCGAGGATTACGATGCTTCTGATATGCGCCATCAATCTATGTTCGATTCTGAAGATGAA

GATAATGCTGAAATGAAACTTATTCGTACTGGTCCTAGAATTGATTCGTTTGATGTTGAA

GCTCTTGAGGTTCCTGGTGCTCATACTCATCACTATGAGGATATGACCACAGGAAAGAAA

ATTGTGTTGGCTTTTCAAACTCTTGGTGTTGTCTTTGGTGACGTTGGAACAAGCCCATTA

TATACCTTCAGTGTTATGTTTAGAAAGGCACCTATTAATGATAATGAAGACATTCTTGGG

GCATTATCACTTGTCTTGTACACTTTAATCTTGATTCCTTTCCTCAAATATGTTCTCGTC

GTTCTCTGGGCCAATGATGACGGCGAAGGTGGTACATTTGCCTTGTATTCCTTGATTTGT

CGAAATGCAAAGGTGAATCTTCTTCCCAATCAGTTGCCGTCAGATGCCCGTATATCAGGC

TTTAGGCTAAAGGTTCCGTCCGCTGAGCTTGAAAGGTCTTTAAAAATCAAGGAAAGGCTA

GAGTCTTCAATCACTCTGAAGAAGGTTCTGCTATTATTAGTTCTTGCTGGTACTTCTATG

GTGATAGCTAATGGGGTTGTTACACCAGCAATGTCAGTACTATCATCTGTTAATGGCTTG

AAGGTTGGGGTAGATGCAATTCAACAAGATGAAGTGGTGATGATTTCTGTTGCTTGCCTT

GTCGTTTTGTTCAGTTTACAGAAGTATGGAACAAGTAAAGTTGGGCTTGCTGTAGGACCT

GCATTGTTTATATGGTTCTGTTCTCTTGCGGGCAATGGTGTATACAATCTTGTCAAATAT

GACAGCAGCGTTTTTAGGGCTTTTAATCCTATTCACATCTATTATTTCTTTGCAAGGAAT

TCAACTAAAGCATGGTATTCTCTTGGGGGCTGTCTTCTGTGTGCAACTGGCTCTGAAGCC

ATGTTTGCAGATCTTTGCTACTTCTCTGTTCGATCAGTTCAGATTACATTTTTATTTCTC

GTTTTGCCGTGCCTATTATTGGGTTATTTGGGTCAAGCTGCATACCTTATGGAACACCAT

GCTGATGCTGGTGAAGCCTTTTTTTCTTCTGTTCCAAGTGGTGCATTCTGGCCAACTTTT

CTCATCGCCAACATTGCTGCACTAATTGCAAGTCGGACTATGACAACAGCTACATTTTCT

TGCATAAAACAGTCAACTGCTCTTGGTTGTTTCCCTCGACTTAAAATAATTCACACCTCT

CGGAAATTTATGGGTCAAATCTATATCCCTGTCATAAACTGGTTCCTGTTGGCTGTTTCT

CTGGTGTTCGTCTGTACTATATCCAGCATTGATGAGATTGGAAATGCATATGGCATTGCT

GAGCTAGGGGTGATGATGATGACCACCATTTTAGTTACTCTTGTTATGCTTCTTATATGG

CAGATGCACATCGTCATAGTGCTGAGTTTCCTGGGAGTCTTCTTGGGTTTGGAGTTGGTT

TTCTTTTCATCGGTTTTGTGGAGCATAACAGATGGAAGTTGGATTATATTGGTCTTTGCT

GCAATTATGTTTTTTATAATGTTCGTATGGAACTATGGAAGCAAGCTCAAGTATGAAACT

GAAGTCAAACAAAAGTTGTCACCGGATTTAATGCGAGAATTGGGCTGCAATCTTGGGACA

ATACGAGCTCCTGGAGTTGGTCTACTTTATAATGAGTTGGTCAAAGGAATTCCAGGAATT

TTTGGGCATTTTCTAACCACACTTCCAGCAATACATTCCATGATTATATTCGTGAGTATC

AAGTACGTTCCAGTTGCCATGGTGCCTCAAAGTGAAAGGTTTCTATTTCGGCGAGTTTGC

CAGAGAAGCTATCATTTATTTCGTTGTATTGCCAGGTATGGTTACAAAGATGCTCGCAAA

GAAAATCACCAGGCTTTTGAACAGCTTCTAATGGAGAGCTTAGAGAAGTTTATACGTCGC

GAGGCTCAGGAGAGATCACTTGAAAGTGACGGGGATGAGGATACTGATTTAGAGGACGAG

TACGCTGGTTCTAGAGTTCTCATAGCTCCAAATGGGAGTGTTTACTCACTTGGTGTTCCC

CTTCTGGCTGATTTTAATGAATCATTCATGCCCAGTTTTGAACCCAGCACATCAGAAGAG

GCAGGCCCTCCGTCTCCCAGACCCCTTGTGCTCGATGCAGAGCAACTTCTGGAAAGAGAG

TTATCTTTTATACGCAATGCTAAAGAATCTGGATTGGTTTATCTTCTTGGTCATGGAGAT

ATAAGGGCAAGGAAAGACTCTTGGTTTATTAAGAAGCTAGTTATAAACTATTTTTATGCC

TTCCTGAGAAAGAACTGCAGAAGGGGGGTCGCAAATTTAAGCGTACCGCATTCACATCTA

ATGCAGGTTGGAATGACTTACATGGTTTGA

>MS.gene009848.t1

ATGTTAGGTAAACTTCCACATAGAACACTTCAATCACTCTCCAAAAAATATGGTCCAATC

ATGTCATTACAACTTGGTCAAGTTCCAACTATTATCATTTCATCTTCAAAAGCTGCAGAA

TCATTCCTCAAAACTCATGACATCGTTTTTGCAAGTCGAGCAAAGAGTCAAGGATCAGAG

ATCATGTCATATGGTTCCAAAGGGATGGCTTTTTCTGAGTATGGTCCTTATTGGCGTAAT

GTGAGGAAATTTTGCACTTTGAAACTTTTTAGTGCTTCAAAAGTTGAGATGTTTGGTCCT

ATTAGGAAGGAGAAGTTGGATGTTTTGGTTAAATCTTTGAAGAAAGTTGCTTTGGAAGGT

GAGGTTGTGAATGTGAGTGAGGTTGTAGAGAATCTTATTGAGGATATTGTGTATAAGATG

ATACTGGGTAGAGGTAAGTATGAGCAATTTGACATGAATAAGTTAGTTCTAGAAGCATTG

GCTTTGATGGGAGCTTTTAATCTGGCTGATTATGTACCTTGGCTAGGCGTTTTTGACCTT

CAGGGATTAACACGAGCTTGCAAAAAAACCAGTAAAGCCCTCGATGAGGTTTTGGAGATG

ATAATAACAGAGCACGAACAGACTACTAATACAGATAAAACTCGTAATGAAGACTTTGTA

GACATACTTCTTTCAATTATGCACCAAACCATTGATGTGGAGGGTGAACAAAATCTGGTC

ATTGATCGAACTAACATCAAGGCTATTTTGCTCGACATGATAGTGGCATCAATTCATACA

TCTGCTACCACGATTGAGTGGGCTTTATCTGAACTTCTAAGGCATCCAAGAGTGATGAAA

ATTCTTCAAGATGAGATACAAAATGAAGTAGGAAATAAGAGAATGGTTAAAGAGAAGGAT

CTGAAGAAGTTTAATTACTTAGATATGGTGGTGGATGAAACCTTAAGACTTTATCCTGTT

GGACCCTTATTAATCCCTCGTGAATGTAGAGAGAGCATAACAATTGATGGATATTTTATA

ACGAAAAAGACTCGAGTTATAGTAAATGCATGGGCTATAGGGAGAGATTCTAATGTTTGG

TCAGAAAATGCTGATGAATTTTACCCAGAGAGATTTATTGACAAGAAAATGAATTATCTC

GGTCAAGAGTTTGAATCTATACCATTTGGTTCTGGTCGTAGACGTTGTCCTGGAATTCAA

TTGGGTTTAATTACGGTTAAGTTGGTTATAGCTCAATTTGTGCATTGCTTTAACTGGGAA

CTTCCACATAATATTAGTCCTTCCAATTTGAATATGGAGGAGAAATTTGGACTCTCTATA

CCAAGAGCTCAGCATTTGCACGCAATACCGAGTTATCGTTTGGATGATGCTAAGCAAGAA

TAG

>MS.gene009910.t1

ATGGATTCCGTGTATATGAATCCGGCTGAACCGGTTGAGGCTCGAATCAAAAACCTTCTT

TCTCTTATGACTTTGAAAGAGAAGATTGGTCAGATGACCCAAATTGAACGTTCTGTTACT

ACTCCTTCTGCCATTAAAGATTTCACCAGTGGGAGTGTGTATTGTGCTCCACCAAATAGT

ACAACTGCTAAGAAGGAAGTGTCTTCTGATTGGGCTGATATGGTGGACAGGTTTCATAAT

CTGGCACTTGAGTCGCGGTTAGGCATACCAATCATTTATTGTACTGATGCTATTCATTGT

AACAATAACATCCATGGTACTACTATATTTCCTCACAACAATACTGTGCACTTCACTCTT

TCCGTGAATCAATTACCGATTCTACCCCTCCATCACTCTCTTCATGAAAACGGTCTCTTT

TCCACAATCACCACCCACCGCAACCTCCGGCAACTGCAACCGCCAAAGTCCAGCACCAAG

CTTCCTCGCCGACACAATCACCAACGGCTTCTGCTGCTGCTTCTCCTCACTTTTCCGACA

AACCTCCTCCACCAGATCACCACCACCGTCAACAACGCTCCCTTCTCCGTCTTCAGGAAT

TTCCACTTCGACGAAGGAGTTTCGGCCTACTCCTTCTTCCCGAAGACGTACCACTTCAAC

TCAAACGACGGCGAGAAGTTCTCCGGTGAGGTCGAATCTGAATCGGGTGAAGTTGTTGGA

AGCAATGCTCGCCCTCTGATCTCACCTGAATGCGATTACGGTTTCCTCTTCTTCTTCTCC

CGTGAATTCGGTTTGCCTCTTCTCTGGATCGAACAAAAGGAGATGGTGTTATGTCTTGCT

GAGATCGGTTCAGTTCCGGCGAGAGTTGTTGTTCTTCGCGGTTTCATCTTGAAGCGTGAT

TATGAAGTTATATGGGGACTTGTTGGACCACGCAGAATCTTTGGAAACCTTGGTGAATAT

TCAACTGTGAATTGGTTTTTCCTTGGTGGAGCCATTGCACCTTTTCTAGTTTGGCTTGCT

CACAAGGCATTCTGGGGACAAACATGGATACGTCAAATTCACATGCCTATCTTGTTGGGT

GCTACATCAATGATGCCTCCTGCAACTGCAGTCAACTTCACTAGTTGGATTATTGTTGCC

TTTATCTCTGGGTACATTATATTTAAATATAAACAAGATTGGTGGAAGCGCTACAATTAT

GTTATGTCTGGTGGTCTTGATGCTGGAACTGCCTTGTGTCATTGA

>MS.gene009939.t1

ATGGAGTTTATTGCTGAAGTTGGTTTTTTGCACCACCCTCGTCACCTTTTCACACCCTTC

TCTAATCCTCCTTACACAATTTCACCAAAGAGGCATATATTCCATCATGCACCACTACAC

GGAGAATATAGATTCATGTGTTGTCTGAAGAACCATAAATCAGTTAGAAGATTAGCTTCA

TTCACAACAACGCAGAAAGGAAATTCAACCACCAGTGTTGATGATATGTGCAAGATTGTT

TGGACTGTAGAAGCTGATTTAGAAGATGGTCATCTCCTGTACATAACTGGGGACCCTGCT

GTGTTAGGCTGCTGGAAACCCAACATGGCTGTACTAATGTCTCCTACGGAGCACACTAAT

ATTTGGAAAGCTGAATCTCAGATTGCTTTTGGCTTGAACTTTAAATATAACTATTTCATT

AAGGAAAAATCAAGGTCCTCAAGTGACATTATTTGGAAGCCTGGACCAGCATTTTCTCTT

TCAGTACCTCTGACAGTTCTAGAAGATAATGAAATTGTGGTGAGGGATTTATGGATTAGG

TCCAATTTCCATATTTCTTCAGCTCAAGCATGGAACCCCTGCACTGAGGAAACGTATCTT

CTAAAACAGCCCTCTATTTTTTTTCCAGTCAAAGATGAACGAAGGAATATGAGTCTCCTT

GAAAATGATTTCCTGAAGACCGAGACACTCATTTTGGAAGATCAATTATTTTTTGACAAT

GAGGACATGGCGATTTTGAGTAATAAGGATTCACATCCTATTAACGTTCTTTCTGAGAAT

TACCAGCCTGTTGAGGAACCCTGGTTACTCCATTCACTGCATTCGGTTATCTCTGAGGAT

AAAACAGAATCAAATGAATCTAAAACTAATGACATTGTGAAAGAACAGGTGAAGTTGGTA

GATTCAGAAGAGTTGTTACCTGAAGAGAGCAGTAATACAATTTTAAAAGACCCTGTTTCC

ACCATTATACTTATAAACTCATCCATATGTACCATGCAAAGAATTGCAGTACTGGAGGAT

GAAAAACTGGTTGAATTATTATTGGAACCAGTGAAGACTAATGTGCAAAGCGATAGTGTA

TATGTGGGGGAAATCACAAAGCTTGTTCCTTCTATGGGAGGAGCTCTTGTTGATATTGGG

AATTCTAGACCCTCTCTTATGGACATTAAGCCATACAAGGAACCATTTATATTCCCCCCA

TTTCGTCGAAGGACAAAGAAACAAGAAATTGATCTTAAGGGAAAAAATGATCACATGTCT

CGTGCTACTGACATTCCTGGTGGAATACGTGACATACATTCTGAGGATGATTGTTTGAAA

TCTGTACACAATGACTATGATGAACATGAAGCAGATGATGATTTTTGTCTTTCAGAAGTT

CTTAAGGAAAATGTGAATGGTAGTGTGGTTGATGAAGTAGAAGCTGACTTTGAGGATGAT

GTTGAGGGAGCTGATGTTCATATAGAAGGAAAAATGAATAACGGTTCCCTTTCTTTAGGC

ATGAATGGGTCTATCAATTCTCATATTCTGCCAACAAAAGATACAAAGAGAGAAATGGGA

GAAAATAAATGGATCCAAGTTCGTAACGGAACCAAAATAGTTGTCCAAGTTGTTAAAGAG

GGTCTTGGTACGAAGGGTCCCACTCTGACCGCTTTTCCTAAACTAAGAAGTAGATTCTGG

GTTTTGACTACACGTTGTGATAAAATTGGAGTCTCCAAAAAGATTTCTGGTGCCGAGCGC

ACAAGGTTGAAAGTTATAGCAAAGACATTGCAGCCAGAGGGTTTTGGTCTTACCGTAAGA

ACTGTTGCTGCTGGTCATTCTTTTGAGGAACTGCAGAAAGACTTGGAAGGTTTGCTTTCG

ACTTGGAAAAACATAATGGAAAATGCAAAGTCTTCTGCTCTTGCTGCTGATGAAAGGGTG

GAAGGAGCTGTTCCTGCTATTTTGCACAGGGCAATGGGTCAAACTCTCTCAGTGGTCCAA

GATTATTTCAATGAAAATGTTAAGAAAATGGTGGTCGACTCCCCAAGGACATTTCATGAG

GTGACCAATTACCTTCAGGACATTGCCCCTGATCTTTGTGATCGAGTTGAACTATATAAT

AAAAAGGTTCCCCTTTTCGATGAATACAACATTGAAGGAGAGCTTGACAATATCCTCAGC

AAAAGGGTTCCACTTGCTAATGGAGGTTCTCTAATCATCGAGCAAACTGAGGCGTTAGTT

TCTATTGATGTAAATGGAGGACATGGGATGCTTGATCATGATACTTCCAAGGAGAAAGCT

ATTCTAGATGTCAATCTTGCAGCTGCAAAACAGCAGCATAAATTTTATACACTATCTAGT

TTAAAGAAACTCCGTGTCAAAATACGATATCTTACTTTCAACAATGCTCTTGAGTAA

>MS.gene009861.t1

ATGAGAAACATTTTACTAAGGTCAACTGCACGAGCTTTGTTCCGCAGTGGTGGGAACTAC

CACCGTAACTTCTCAACTGCGGTGATAGCTCAGCCGAGACACCACCAGCATGGCGGTGAC

ACATATGGAAACTTCTACTGGCAGAGGATGTCAACTTTGCCAGAGAAAAAGGATCAGCAG

ATGGAGGAGAACAAGAATGATGCCAACAACAACAGTAATAACAGCAACGCCGTCGTTTCG

AGTTACTGGGGGATCAGTAGGCCTAAGGTTCTCAAGGAAGATGGAACTGAGTGGCCATGG

AACTGCTTCATGCCATGGGAAAGTTACAGTTCAGATGTGTCAATAGATGTTACCAAGCAT

CATGTACCAAAAACATTTGGGGACAAGCTTGCTTTCAGATCTGTCAAGTTTCTAAGAGTG

CTTTCTGATTTGTATTTCAAGGAACGATATGGTTGCCATGCAATGATGCTAGAAACAATA

GCAGCTGTCCCTGGAATGGTGGGAGGGATGCTTTTACACTTAAAATCTCTAAGAAAATTT

CAACATGCTGGTGGTTGGATCAAAGCATTACTTGAAGAAGCAGAGAATGAAAGGATGCAT

TTAATGACAATGGTTGAGCTTGTGAAACCAAGTTGGCATGAGAGGCTTTTGGTTATTACT

GCACAAGGGGTTTTCTTCAATGGATTCTTTGTGTTTTACATCCTCTCACCAAAAATAGCA

CATAGATTTGTTGGTTATTTGGAGGAGGAAGCTGTGATTTCATACACACAACATTTACAT

GCAATTGAAAGTGGTAAAGTGGAGAATGTACCTGCTCCTGCTATTGCAATTGATTATTGG

AGACTTCCTAAGGATGCAACTCTTAAAGATGTTATCACTGTTATCCGTGCTGATGAGGCT

CATCATAGGGATGTCAATCACTTTGCTTCTGATATTCACCATCAAGGAAAGGAATTGAAA

GAGGCTCCAGCTCCTGTTGGTTATCATTGA

>MS.gene009872.t1

ATGATTAGATTGGCAGCCGACGTGTCAGCATCTGAATATCTACATAAGCTTGTAGAAAAG

GGTTTGTATGACTTGTGTAAGCTGGTGATGGAGGCTCGGTTACGAGAAATAGAGAATGTC

GATGATGAAGGTGATGTCCCAACAGGAAAAAAAGAATATGAGACACAAGAAAAGGGGTTT

AAGGTTAAGCCTGGATTACAAAATCGTAGACGACTAAAGAGTTGTGTCGAGCTTATGTCT

AAGAAAAAAAGCAAAGCAACAACTGTAAAACCTACACCACCTCAAAGATCAAAGGATCAT

GATTCTCTAAGTCAAATGGAATCTGGGACATCAACTTTAGCACAATCAGCATGTGAGCTT

TCTCAGACAAGTGAGGACAACTTTAGTTTCACTAGATTATTAATGGAGCCACTCATTGAT

GGATGA

>MS.gene009888.t1

ATGCGAGGCCAGCCCTCAGCTGAACTTACCTTTATTCCAGAAGTTGAAAAGTACTGCCGA

GCCATCCGGAAAGAAACAAAACTCCGAAAACTTCGAGAAAAGGAAGGGACCAGCGGAGTA

GAGGTTGACACGGACGAGCAGTTAGACGAAGAGATGGCGGAGGGACAACCGCCACCACCA

CCACCACCTGAAGAAAGACTTCTAGGAGACTATGGGGCCCGTGACAGAAACCGAAACAGG

CTGACCATCACTAATCAGCCTGTGACGGTAAACAAGTTTGAGATCAATCCCGGTCTGTTG

CGGGAGTTGAAGGAGAATCAGTATGCGGGGAAGTACAATGAAGATGCCAACAAGCACTTG

AAGAGTTTCTTGATACTTTGTGAGACAGCTAAGGTACAAGGGCACTCTGAGGAAGCAAAA

AGATTGAGACTGTTCCCGTTCACTTTAACTGATGATGCTTATGAGTGGTTTGACTCTTTA

CCGGCTGGTAGCATAACAACTTGGAATGAGATGGAAGATAAGTTCTTGGAGCAATTCTTT

CCTACAGCTCTATTTGTGAGGAGGCGTCAAGACATTTCCAGCTTCCAACAGAAAGAGGGA

GAGTCCTTGGGAGAAGCTTATAAAAGGTACAAGAAGTTAATCTCAGCATGTCCCGAGCAC

AACTTTGATACTACGGTTCAAATGCAGATGTTTTGTAATGGTCTACGTCTGGCTACTAGA

CAAGTTCTCGACACAGCTTCCGGTGGTTCCATCAATTTCAAAACCGCCTCTCAGATAATC

AAAATCATTGAAGCTGTTGCACTTAATGAACAAATGGAGATGTATGATAGAACCGGTGGT

ACAAGGGGAGGACTCATAGATCTGAATCAACTTGAGCTCAAAAATGCTCAAAGCATACTA

ACTGGTAAGCAGATTCAAGATGCAGTCGCTGCTGAAGTATCAAAGAAAATGGCAGCACTC

AACTTAACTCAACCCCAAGTTGCTCCGGTGAATCAAATGAACACTGTTAAATGTGATTGG

TGCGCTGGCCCTCATTTCACTATGCATTGTGATGTGCCCATGGATGCAACCCAAGTGGAG

ATGGTGAACTACCTCAGCAGTCAAGGGAACCAGGCTGGAGGAACCATCCAAATTTCTCTT

GGAAAAATCAACAAGGTGGTTCCCAAAATCAACAACAAGGGGGTTATCAAGGAGGTTCAT

CAAACCAAGCACCGAAGAAGGCGGATTGGGAGTTAG

>MS.gene009868.t1

ATGAGAATCACAATTCTAAGGGACACTAGAAGGAAATTGGGCGGTGCTTTAGGATTCAAT

AGGTTGATTCATTCCGTGCCTCAATCTCCTCCTTTAGCTGGGAGCATTGACCATGGGATT

CAGTCGTTGAAGCCCGTTTTGCCTGAATTTAGTTCTCCAACGTTCTCTTTTGGTGGCTCT

ATGGAGCTCATGGCTGTGCCAAAAAGGAAGACTTCTCCTCATAAAAGAGGAATAAGAAAT

GGGCCAAAAGCTTTGAAACCTGTTCCTGTGCTAGTTCTGTGCAAATTTGTTGGAGAAAAT

TGCGATTCGTCCGGGACGAGTGGAGTTTCCTTTTTCACAGGGACTAGGGTGAAATGCGCT

TCTCTCAGTGCTTCTGCTAAATCTAAACAGTCGCGATGTTTGAAGTGA

>MS.gene009928.t1

ATGTTGTCCACATGGAAAGACGACCCCACCGCAGATTGTTGCAAATGGATGGGAGTTCGA

TGCAACAATCAAACAGGTTATGTTCAAAGACTTGATCTGCATGGTTCGTTAACACGCCAT

TTGAGTGGTGAAATCGTTTCTTCAATAATTCAGCTTGAACATCTAAAACATCTAGACCTC

AGTCATTTGTTAACTAGAGGCAACATCCCAAAATTTATTGGCTCCTTCAGCAACTTACAA

TACCTTGATCTCTCAAATGGTGTTTATGATGGGAAGATTCCTTCTCAACTTGGAAATCTT

TCAAAATTGAAACATCTTGATCTCAGCGGCAATGAACTCATTGGAGAAATTCCCTTCCAA

CTTGGAAATCTCTCACAATTGCAACAACTTGATCTCAGCTACAATGAACTCATTGGAGGA

ATTCCCTTCCAACTTGGAAATCTCTCACAATTGCAACACCTTGATCTCAGCTACAATGAA

CTAACTGGAGCAATCGCTTTACAACTCCAAAATCTTTCATTGTTACAAGAGTTATGGTTA

TGGGATAATGAGATTTCAGGCTTGTTACCTGACCTTTCGGTCCTTTCATCTTTGAGAGAG

TTGATGCTACATAACAATAAATTAATTGGAGAAATACCCGCGGGTATCGGATCACTTACG

AAGTTAGAGTATTTGTACCTAGGAAGCAACTCTTTTGAAGGTGTCCTCTCTGAATCTCAA

TTCACTAACTTCTCCAAATTATTAGGCCTATCTTTGTCTTCTAACTTGTTGACGGTGAAG

GTTAGTACTGATTGGGTTCCTCCTTTTCAATTAATGTATTTGCATCTAGCATCTTGTAAT

CTCAACTCTACCTTTCCTAATTCGCTTCTTAAACAAAATCACTTATTGAGACTTGACATT

TCTAACAACAATATCACCGGAAAGGTTCCCAATTTGGAACTCGAGTTTGCAGAAACTCCT

GAAATAAATTTGAGTTCAAATCAACTTGAAGGTTCCATTCCTTCTTTTTTATTTCAAGCA

GCAGCACTGCATCTTTCCCATAATAAATTTTCAGATTTAGCTTCCTCTCTATGCAACAAA

ATTAACCCTAACAATCTGGTTGTGGTGGATTTGTCAAACAATCAATTAAAGGGTGAGTTG

CCTGATTGTTGGAATAATCTAACCTCGTTAAAGTTTGTTGAACTGAGCAATAACAAGTTG

TCGGGTAAGATTCCATTCTCAATAGGTGCTCTTGTTAATATGGAGGCTTTGATCTTAAGA

AATAACAGATTGAGTGGACCACTTCCTTCCTCCTTGAAGAATTGTTCAAACAAATTGTCT

TTGTTGGACCTTGGAGAAAATATGTTTCATGGTCCAATACCTTCATGGATTGGAGACAGT

TTCCACCAATTAATAATCTTAAGCCTTCGATCCAATAACTTCAATGAAAGCCTGCCTTCA

AACATCTGTTATTTGAGAGAACTTCAAGTCTTGGATTTGTCACTAAATAGCTTATCAGGA

GGAATCCCAACATGTGTAAAGAATTTTACTTCAATGGCTCGGGACCCTATGAACTCAACT

TCATTGATGTATCATTCGTATGGAATCAATATTACTGATAATGAGGAAATCATTTTTATA

TATCACTTTGATCTATTCTTGATGTGGAAAGGTGTGGACCGACTCTACAAAAATGCAGAT

AAGTTTTTAAACAGCATTGATCTTTCAAGCAACCACCTTATAGGAGAAATACCAACAGAA

ATTGAGTACTTACTGGGATTGACATCCTTAAATTTGTCAAAGAACAATCTTAGTGGGGAA

ATAATTTCAGTCATAGGAAAATTCAAGTCACTTGAATTTCTTGATCTATCAAGAAATCAT

CTATCAGGCACAATTCCTTCCAGTCTAGCTCATATTGATCGACTCTCTGTGTTGAATTTG

TCAAACAATCAGCTTTATGGAAAAATTCCAATTGGAACGCAGCTGCAGACATTTAGCGCT

TCGAGTTTTGAAGGAAATCCTAATCTCTGTGGAGAGCCACTTGATATAAAATGTCCAGGA

GAAGAAGAGCCGCCAAAACATCAAGTGCCAATAACTGATGCAGGAGATGACAATTCAATA

TTTTTGGAAGCATTATACATGAGAATGGGGCTAGGATTCTTCACTACTTTTGTTGGCTTT

ATAGGGTCAATATTGTTCCTACCATCTTGGAGAGAAACCTATTCCAAATTCTTGAATGCT

TTGATATAG

>MS.gene009938.t1

ATGGGTAGTGAATATTCTTCTTCATCTGGTGTCCAGAAACGTAGAACAAGATGGTATGAC

CCTGAAGTGCCTGAGAGACAAAAGAAGGTTATTCGAGCTTTGTTGAAGAAGAATGACGAA

TTGCAGGACAAAGAAAGAAGTCTAGCAAAGGCAAAGGCTGTCATAGTTGTTTTAGGAGTT

ATGTTGTTGATCTCTCTCTGTTGTAATCTGGTTTGA

>MS.gene009911.t1

ATGGATTCCGTGTATATCAATCCTGCTGAAACGGTTGAAGCTCGCATCAAACACCTTCTT

TCTCTTATGACTTTGAAAGAGAAGATTGGTCAGATGACCCAAATTGAACGTTCTGTTACT

ACTCCTTCTGCCATTAAAGATTTCACCATTGGGAGTGTATACTGTGCTCCACCAAATAGT

GCAACTGCTAAGAAGGAAGTGTCATCTGATTGGGCTGATATGGTTGACGGTTTTCAGAAG

CTGGCGCTTGAATCACGGTTAGGCATACCAATAATTTATTGTACTGATGCTATTCACGGT

AACAATAACGTCTATGGTACTACTATATTTCCTCACAACGTTGGCCTCGGAGCAACTAGA

GATGCAGATTTAGTTCAAAGAATTGCAGCTGCAACATCACTTGAACTTAGAGCAAGTGGA

ACTCACTATACTTTAGCTCCTAGTGTATCTGTCTGCAAAGATCCTAGATGGGGAAGATGC

TATGAGAGTTACAGCGAAGACACCGAAATTGTCCAAGACATGACTTCTTATGTTTCAGGT

TTGCAAGGCCAACCTCCAGAAAATCATCCAAGGGGCTACCCATTTGTGGCTGGGAGGAAC

AAAGCTATTGCCTGTGCTAGACATTTTGTTGGAGATGGAGGTACAGAAAAAGGTGTAAAT

GAGGGGAATACAATATTATCATATGAAGACTTAGAGAAGATCCACATGGCCCCGTATGTG

GATTGTATAGAACAAGCTGTTTCAACCATTATGGTCTCATATTCCAGCTGGAATGGAGTC

AAACTCCATGGTCATCGATTTCTGATTAATGATATTTTGAAAGAAAAGCTAGGCTTCAAG

GGTTTTGTGATTTCTGACTGGGAGGGAATCGATGAATTATGTCAACCTTATGGTTCAGAT

TATCGTTATTGCATATCTACTTCCATTAATGCTGGAATTGACATGGTGATGGTTCCTTTA

AGATATGAACAATTCATGGAAGAGTTGACATCTCTAGTTCAATCGGGGGAAGTACCAATA

GCCAGGATTGATGATGCCGTTGAGCGGATTTTAAGAGTGAAGTTTATTGCTGAACTTTTT

GAATTTCCTTTAACTGACAGATCTTTGCTGGATACTGTTGGTTGCAAGATACATAGAGAT

CTAGCACGTGAAGCAGTTCGAAAGTCCTTGGTTTTGTTGAAAAACGGAAAGGAGCCTAGT

AAACCTTTCATACCATTGAATAAGAACGCCAAGAGAATCCTTGTTGCTGGAACTCATGCT

GATGATATCGGGTATCAATGTGGAGGATGGACATTTACTAAGTATGGATCTAGTGGCCAG

ATCACAATTGGCACAACTATATTGGATGCTGCTAAGGATGCTGTGGGACAGGAAACTGAA

GTTATATATGAGAAGTGCCCAACACCAGAATTCATAGAACGTAATGAATTTTCTTTTGCC

ATTGTTGCTATTGGTGAAGCCCCCTATGCTGAGTGTGGAGGTGACAATAAAGAACTTGTA

ATCCCATTTAATGGAGCTGGAATTGTCGACATAGTTGCTGATAAAATCCCAACACTTGTG

ATTCTAATATCTGGAAGACCTTTGGTCTTGGAACAAAGTTTGCTCGAAAAAGTAGAAGCT

CTTGTTGCGGTATGGTTGCCTGGTAGTGAAGGAAATGGAATCACAGATGTTATCTTCGGG

GATCATGACTTCAAGGGTAAACTACCAATGACTTGGTTTAGAAGAGTTGATCAGCTTGAT

CAACCTGTTGAAGGAGTGAATTCATGTGATGACCCCTTATTCCCTCTTGGTTATGGGCTA

GCTTATAATAACGAGAATTCACATGAATAA

>MS.gene009905.t1

ATGTCTACCGAAAAGCATGAAATCTTGGTGGTTCGCCTTAATGGCAAAAACTACTCAACT

TGGGCATTCCATTTCGAGATATTTGTCACAGGAAAAGATTTGTGGGGTCATGTTGATGGC

AGCACTCCAGTTCCTGACAAAGAGAAAGATAAGGTTGCACATGGCAAATGGGTAGTCAAA

GATGCTCAAGTCATGTCCTGGGTCCTAGGTTCTGTTGATCCAAACATTGTCCTGAATCTC

CGACCTTACAAAACAGCAGCCACAATGTGGAATTACTTGAAGAAAGTCTACAATCAGAAC

AACGCCGCTCGAAGGTTCCAGCTTGAACACGACATAGCCTTATTTAAACAGGATAGTCTC

TCCATCTCTGAATTTTACTCTCAATTCATGAATCTTTGGGCTGAATACACTGAGATAGTT

TATGCAGACTTAACTTCTGAAGGTTTAAGTTCCGTACAATCTGTTCATGAAACTACCAAG

CGAGATCAATTTCTTATGAAATTGAGATCTGAATTTGAAGGTATTCGGTCCAATCTCATG

CATAGAAACCCTGTACCATCATTGGATGCATGTTTCAATGATCTGTTACGTGAAGAACAA

CGTCTACTGACACAGTCCATCATTGAAGATCAAAAATTGTCTACTGTTCCAGTGGCTTAT

GTGGCACGAGGAAAGACAAGAAGTCATGACATGAGTGCTGTTCAATGTTTCTGTTGCAAG

AAATTGGGACATTATGCTTCAAACTGTCCAGACAAAGTCTGCAACTACTGCAAAAAAGAC

GGACATATTCTTAAGGAATGCCCAATAAGGCCACCAAGGAGAAATGTCACAGCCTTTACA

ACCTCGGTTGATTCATCTATTCCTAGCAACTCGGTCAATCCAGCACCGGTTCAGCAAAAT

GCTCCTACTGCTTCATCAGTGACCCCTGAAATGGTTCAACAAATGATCATTTTTGCCTTC

TCTGCTCTAGGAATATCAGGATCAACAATCAGGGAAGATAATCGCGAAGGGGCCTAA

>MS.gene009918.t1

ATGTCTTCGAATGATCAAAAGCTGGCCAACTTGATAACAACATTTTTCACCGTCGGTAAC

CCTTGCGTCAATTTCTTTTTATATGTTTTCCGTGATACTCCCTCTGAAACACTCATCGAG

AGACTCAAACTAGCATGGTCCCACGATTCATTGACCACCCTCAAACTCGTCTGTAACCTC

CGTGAGGTTCGTGGTACCGGCAAGTCCGATAAAGAAGGCTTCTACGCCGCCGCTCTATGG

CTTCATGAAAATCACCCTAAAACCCTAGCTTACAATGTTCCTTCACTTGCTGACTTTGGT

TGCTTCAAAGATCTCCCTGAGATCCTCTACCGTCTTCTGAATGGTTCTGAAGTTCGCAAA

ACGCAGAAAGAAGAGTGGAATGAAAGGAAATCTTCATCAAGATCAGAGATCATCTACAAT

GTTCCTGAAGAGTTGAAGGACACGAAATCTTCATCAAGATTAACCCCTTTTGTTGTAATT

GTATACGACAAGGAGAAGAAGAAGAAGAAGAAGAAGAAGAAGAAGAACAATGATGTTAAG

GATAACAAAGGGTGGAAGGGTACCAAGAAAGACTCCGAACTGACTGAAGCCGTGGCAGCG

AGAGTTAAGGATGAGAAAAAAGCTGTTCAGGATTTGCATCAAGAGAAGAGGTTTGCCTTG

GCTAAGAAGTTTAATGATTGTTACACTACCGACCCCAAATTTAAGTGTCTTTACGATAGC

ATCTGTACTCATTTTGCTGATTGTTTGAAGAAGGATCTTCAGTTTTTGAAATCTGGGTCA

CTCACAAAAATCAGTCTTGCTGCTAAATGGTGCCCTTCTCTTGGCTCATCCTTTGACAGA

TCATTGTTGTTGTGTGAGAGCATTGCCAAGAGGATCTTCCCCAAGGAGGAGTATGAAGGT

GTGGAGGAGGCGCATTATCTTTATAGGGTTCGTGATCGTTTGAGGAAACATGTGTTGGTG

CCGTTGAGGAAGGCGTTGGAGTTGCCTGAGGTGTTTATTAGTGCTAATCAATGGGGTTCG

ATTCCTTACAATAGAGTTGCTTCTGTGGCGATGAAGTTTTATAAGGAGAAGTTTTTGAAG

CATGATAAGGAGAGGTTTGAGAAGTACTTGAAGGATGTGAAGGCAGGAAATACTACTGCT

GCTTTGCTTCCTCGTGAGATTATAGAGTATTTGGAGGATGGAGATAGTGTTGAAGAGCAG

TGGAAGAGAACTGTTGATGGTTTGCTCAAGAAGGGAAAGATGAAAAATTGTCTTGCAGTG

TGTGGTGTGTCTAGGAGTATGCATAGGACCCCTGTGGCATTGGGGTTGTTGGTGTCTGAA

TTGAGCGAGGAACCGTGGAAAGGGAAGGTTATTACTTTCAGTATGGAGCCTCAGCTTCAT

GTGATTCAGGGGGGTGATCTGAAGTCCAAGACAGAGTTTGTGAGGAGCAAGGATGAAGGG

ATCAACACTGAGTTCCAGAAGGTTTTTGATTGCATATTGGATGCAGCTGTAAATGGTAAT

CTCAAGGAGGATCAGATGATTAAGAAGATCGTTGTCTTCAGTGATATGGAGTTTGTTGAA

GCGTCTGCAAACTATTGGGATCAGACCGATTACCAAGCAATCACTAGGAAGTATAGAGAG

AAAGGGTTTGGTTCTGCTGTACCTCGGATAGTTTTTTGGAATCTGAGAAACTCAAGGGTC

ACCCCAGTGCTGGCTGCTCGAAAAGGAGGAGTGACGCTGGTAAGTGGATTCTCTAAGAAT

CTGCTGAAACTGTTCTTGGATAATGAAGGCGAAATCAGCCCTGAAGAAGCAATGGAAGCT

GCCATTGCTGGCCCTGAGTATCAGAAACTAGTTGTTCTAGATTAA

>MS.gene009902.t1

ATGTTCATTAAACAGGTTGTGATTGAAGGTTTCAAGAGTTACCGAGAGCAAATTGCCACG

GAGGATTTCAGTCCTAAAGTTAACTGTGTTGTTGGTGCTAATGGATCTGGTAAAACAAAT

TTTTTCCATGCCATTCGTTTCGTGTTGAGTGATCTCTTTCAGAACTTACGCAGTGAGGAT

AGGAATGCACTACTCCATGAAGGGGCTGGACACCCAGTTTTATCTGCATTTGTGGAGATT

GTGTTTGATAATTCAGACAATCGTATCCCGGTTGACAAGGTGGAAGTGCACTTACGCCGG

ACAATTTGTTCGAAAAAGGACGAATATTTTCTAGATGGAAAACACATAACTAAAACTGAA

GTGATGAATTTACTGGAAAGTGCTGGGTTCTCGCGCTCAAATCCATATTATGTTGTACAA

CAAGGAAAGATAGCATCCCTAACCTTGATGAAAGCCTCTGAGCGATTGGATTTACTGAAG

GAAATAGGTGGTACTAGAGTTTATGAGGAGAGGCGTCGTGAAAGTGAGAAAATAATGAAA

GATACAGATAACAAAAGAGAACAAATTACCCAAGTTGTACGGTATTTAGAAGAGAGGCTG

AAGGAACTAGATGAAGAGAAAGAGGAACTTAGAAAATATCAGCAACTTGACAAGCAGAGA

AAATCCTTGGAATATGCCATATATAACAAAGAAGTTCAAGATGCTCAGCTAAAGCTTGCA

GAGAAAAAGCTCGGACCAAAGGTTTCTGAAGTATCAAAAAAGAAGTATAATGAGGCTCTT

GTTGCACATGAGAAATCCAAAGATCTGGAGAATAATTTGAAAGATATAATGAAGGAACAT

CAAAACTTTATCAAAGAAAAAGAAGTTATTGAAAAGCGACAAACAACAGCATTTAAGAAG

CACACAGAGCTTGAGCTTGATGTCAAAGACTTGCAAGAGAAGATCTCTCGAAATGAAAGC

GCCAAAGCTGCTGCTGAAAAACAATTGACGATAATTGTGAAAGAAATAAAAGAAGCAATG

GATGAACTGGACAAAATCAGTCCTATATATGATGGTCTGGTTCAGAGAGAAAAAGACATT

ACCATGAGAATCATGAAGCACGAGAAGGAACTCAGTATTCTCTATCAAAAGGAAGGACGA

GCAACACAGTTTTCAAGTAAAGCTGCCCGTGATCAGTGGCTTCAAAAGGAGATTGTTGAT

CTTGAACAGGTGCTCTCTTCAAACACGGCACAGGTGAAAAAGCTGATAGAAGAAATGGAA

TTGCTTAATGTTGAAATGCATGGCTGTGATACGGATATCGATAGCCGTAGAACAAAGATT

AATACTTTGGAATTTCAAACGGCCAAGTCACGTGAAGGGTTGAATAATAATATAGTAAAA

AGAAACGGTCTTCTTGATGAGAGGAAGTCCTTATGGAGCAGGGAGAAAGAACTTACTGCT

GAAATTGATAAACTGAGAGCAGAAGTTGAGAAGGCTGAAAAGAGTCTCAATGATACAATT

CCTGGTGATGTTAGAAGAGGGTTGAATACAGTTCAGAAGATCTGCAAATCTCAAAACATT

TCTGGAGTTCATGGTCCAATTATTGAGTTGCTAACCTGTGATGAAAAGTTTTTCACTGCA

GTTGAAGTTACTGCAGGAAACAGCTTATTTCACGTGGTTGTTGAAAATGATGACAAGGCA

ACTGAGATTATTAATCATCTTAACCGACAAAAAGGGGGACGTGTTACATTTATTCCACTT

AACAGAGTAAATGCTCCACGCATCACTTATCCACAGAGTTCTGATGTCAAACCTCTTTTA

AAGAAATTGAATTTTAAACATGACTACACTCCAGCTTTCAATCAGGTATTTGCTAGAACA

GTTATCTGCAAGAATCTGGATGTGGCTTCAAAGGTAGCTCGTACCAATGGTTTGGACTGC

ATAACACTGGAAGGTGATCAAGTAAGCAAAAATGGCAGTATGACCGGAGGATTTTATGAT

CACAGACGATCAAGGTTGAAGTTTATGAATACCATTAAGCAGAATGCTGACAGTATTCAC

ATCAAAGAGGAGGACTTGGTGAAAATAGACCAAAAGGTTAATGAACTTTTTACCGAGAAG

CAGAAGGTTGATGCTCAATGCGGTCATAACAAATCAGAGATAGAAGAGCTTAAGCGGGAC

ATTGCTAATTATAACAAAAAGAAGCCATTATTTTCTAAAGCACTTGTAAAGAAGGAGAAA

TCGCGTGAGGATGTCCAGAATCAGATTGAACAGCTTAAGGCTAGCATTGCTACGAAAAAA

GCTGAGATGGGCACAGAGCTCATTGACCATTTAACGCCAGAGGAAAAAAAGCTGTTGTCA

AACTGGAACCATGAAATTGAAGGCCTTAAAAAGGACCTTGTTGCTTGCAAGAATGATCGT

ATTGAGAAAGAAGAAAAGAAAGCGGGGCTGGAAACTAATCTAACCACTAATCTCAGGAGA

AGAAAACAAGAGCTGGAGGCTGTGATATCATCTGCTGATGATGACTCTTTGCATGGTGAT

GCTGAGTCAAAGAGTCAGGAACTTAGGGATGCTAAAGGATTGGTTGATGATGCATCAGAT

CAGCTTGAAAGAGTATCGGAAAGTATAATTGATCGAAACGGGCAAATTGCAAAGATCAAA

GATGAAATTAATAAATGGAAGTCTTTGGAGGACGAGTATAATAGAAAACTTCAGGAAGAA

GCTAAAGAATTGGAGCAATTGCTGGATAAAAAAAATGCATTTTCTGCTAAAGAAGAAGAG

TATACCAAGAAAATAAGGGAACTGGGCCCATTGACATCAGATGCTTTTGAGACGTACAAA

CAGAGGAATATTAAAGATTTGCAAAAAATGCTACAGAGGTGCAATAAACAATTAGAACAG

TTTAGTCATATCAACAAGAAAGCACTCGATCAGTACATAAATTTTACAGAACAGCGAGAA

GAAGTTAAGAAAAGACAAGCTGAACTTGATGCAGGAAAAGAGAAAATCAAAGAGCTAATA

ACAGTGCTAGATCAACAGAAGGATGAATCAATAGAACGCACTTTCAAAGGTGTTGCTATG

CATTTCGGAGAAATATTCTCTGAACTTGTACAGGGTGGCTATGGTCATTTGGATATGATG

AAGAAGGATGATGATCAAGACAAGGATGGTCCTCGGGAAGCAAAGCCAAAGGGGAGAGTA

GAAAACTACATTGGAGTGAAAGTGAAGGTGTCTTTTACTGGACAAGGGGAAACACAGTCC

ATGAAGCAGTTGTCGGGAGGTCAAAAAACTGTTGTTGCCCTTGCACTTATCTTTGCCATA

CAGAGATGTGATACAGCGCCCTTTTATCTTTTTGATGAGATTGATGCTGCTTTGGATCCA

CAGTACAGAACTGCTGTTGGAACTATCAAAATTCCTCATTGCATGAGAAATATAATTTCA

GACATAGCAAACACTCAATTTATCACAACAACATTCCGTCCAGAGCTAGTGAAAGTTGCC

GACAAGATATATGGAGTGACACATAAAAACAGGGCCAGCCGTGTTAATGTGATATCTGAG

AATGATGCTCTAGAATTCATTAATCAGGATCAGACACAAAATGCTGATTAA

>MS.gene009889.t1

ATGAACCCGCTAACACTGGTGAAGCGCATTCAACAAATCAATTCAAGAGAAGCTGCACTT

AACATAAGCGAAGACGCTTCATGGCACACTAAGTATAAAGATTCCGCTTATGTTTTCGTC

GGCGGTATTCCCTTCGATTTCACCGAGGGTGATGTGATCGCTGTTTTCGCTCAATATGGG

GAGGTTGTTGATATTAATCTTGTTAGAGACAAAGGTACTGGCAAATCAAAGGGTTTTGCA

TTTCTAGCGTATGAAGATCAACGAAGCACTAATCTTGCTGTTGATAATCTGAATGGAGCT

CAGGTTTCAGGCAGGATTATTAGGGTGGATCATGTTGATAAATATAAGAAAATGGAAGAG

GAAGATGAAGAGGAAGCAAAGCAGAAAAGGGAGGCCCGTGGTGTTTGTCGAGCTTTCCAA

AGAGGGGAGTGTACTCGTGGAGCTGGTTGCAAATTTTCTCATGATGAGCAAAGAGCTGCA

AATACTGGTTGGGGTGATAACGGGATCGCAAAAGGGGATAATGACAAATATGATGGTCCC

AAGAAAGAAAGAAGGTATGGCAACAACCAACCAGATCGCATTCCAGAAACTAGAGATAGA

GATTCACGTTCCAGAGCCCGTGAAAATGAGATGGAATTAGACAACCCATCCAAGAGAAGT

GATAGAAGAGAGGAAAAGGTGTTGCGAAGGCAAGATGGCGATGATAAATTTGTGGGAAGA

GAAAATACCAGTAGAAGAGAAGAAAAGAGACCAAGAGACCATCAAGATGATGAATTTGAA

CAGTGGCCAAGAGAAGATCGGCGTAGGAGAGAAGAAAAAAGATCAAGGAATGGTTATGAT

GATAGGGAACCTGAGCCAAGAGATCATAAAAGGGAAGACAGGAGGTCAATAAAGCAAGAT

GATGTGGAGTTTGAACCTAAGTCAAGAGATTCTGATCTTAGAGAAGATAAAAGACCAAGC

AGGAGGGATGTTGATGATTTTGGAAGCAAGTCAAGAGAAACACACGGTAGCAGGGAAGAG

AGGAGATCAAGAAAGCACACTGACGATGAATCTATGTTGAGGTCAAAAGAAGATAATGAT

AGGAAGCAAGACAACAGATCATATCAAAAAGACACTGATCGATCTGAATCAAAAGGAAGA

AATGATTCTGACCGAAGAGAAGAAAAGAGGTCCAGAAGGTAA

>MS.gene009919.t1

ATGGATTCCGTGTATATGAATCCCGCTGAAACGGTTGAAGCTCGAATCAAAAACCTTCTT

TCTCTCATGACTTTGAAAGAGAAGATTGGTCAGATGACCCAAATTGAACGTTCTGTTACT

ACTCCTTCTGCCATTAAAGACTTCACCATTGGGAGTGTGTACTGTGCTCCACCAAAAAAT

GCAACTTCTGAGAAGGAAGTGTCATCTGATTGGGCTGATATGGTAGACGAGTTTCAGAAG

TTGGCCCTTGAATCGCGGTTAGGCATACCAATCATCTATGGGACCGATGCTGTTCATGGT

AATAATAATGTCTATGGTACGACCATATTTCCTCACAATGTTGCCCTCGGAGCTACAAGA

GATGCAGATTTAGTTCAAAGAATTGGAGCTGCAACATCACTTGAACTTAGAGCAAGTGGA

ACTCACTTTACTTGTGCTCCTTGTGTGGCTGTCTGCAAAGATCCCAGATGGGGAAGATGC

TATGAGAGTTACAGCGAAGACACTGAAATAGTCAGAAAGATGACTTCTTATGTTTCAGGT

TTGCAAGGCCAACCTCCAGAACATCATCCAAGGGGCTACCCATTTGTGGCTGGGAGGAAC

AAGGCCATTGCTTGTGCCAAACATTTTGTTGGAGATGGAGGCACAGAAAAAGGTGTAAAT

GAGGGGAATACTATATTATCATATCAAGACTTGGAGAGGATCCACATGGCCCCTTATGTG

GATTGCATAGATCAGGGAGTGTCAAGCATTATGATCTCATATTCCAGCTGGAATGGAGTC

AAACTCCATGGTCACCATTTTCTGATAAATGATATTTTGAAAGAAAAGCTAGGCTTCAAG

GGTTTTGTGATTTCTGACTGGGAGGGAATTGATGAATTATGTCAACCTTATGGTTCAGAT

TATCGTTATTGCATATCTACTTCCATTAATGCTGGAATTGACATGGTGATGGTTCCTTTA

AGATATGAAAAATTCATGGAAGAGTTGACATCTCTAGTTCAATCAGGGGAAGTACCAATA

TCCAGGATTGATGATGCCGTAGACCGGATCTTAAGAGTGAAGTTTATTGCTGAACTTTTT

GAATTTCCTTTAACTGACAGATCTTTGCTGGATATTGTTGGTTGCAAGATACATAGAGAT

CTAGCACGTGAAGCAGTTCGAAAGTCCTTGGTTTTGTTGAAAAATGGAAAGGAGCCTGGT

AAACCTTTCATACCATTGAATAAGAACGCCAAGAGAATCCTTGTTGCTGGAACTCATGCT

AATGATATCGGATATCAATGTGGAGGATGGACATTTACTAAGTATGGATCTAGTGGCCAG

ATCACAATTGGCACAACTATCTTGGATGCTGTTAAGGAAGCTGTTGGGCATGAAACAGAA

GTTATATATGAGCAATGTCCATCAACAGACTTCATTGAATGTAATGAATTTTCTTTTGCC

ATTGTTGCTATTGGTGAAGCCCCCTATGCCGAGTGTGGAGGTGACAATACAGAACTTGTA

ATCCCATTTAATGGAGCTGGAATTGTAGACATAGTTGCTGATAAAATCCCTACACTTGTG

ATTCTAATATCTGGAAGACCTTTGGTCTTGGAACAAAGTTTGTTGGAAAAGGTAGAAGCT

CTTGTTGCAGCATGGTTGCCGGGTACTGAAGGAAATGGAATCACAGATGTTATCTTTGGG

GATCATTACTTCAAGGGTAAACTACCAATGACTTGGTTTAGAAGAGTTGAACAGCTTGAT

CAATCTGATGAGGGAGTGAATTCATGTGATGACCCCTTATTCCCTCTTGGTTATGGGCTA

GCTTGTAATAAGTAG

>MS.gene009864.t1

ATGAAAGATATTTTACTGTTCAACCTCAGAAGGATAAATGTTCTAATTCAGGGATTAACC

GTAATAAACGTACCTAATGATGAAGAGAACGTCTTTTGGAGTGGAGGAGGTGACAATGAG

TCCGGTGGTGGTATCGAGGATTGCGATTTTGATAGTGGGGGCGTGGTTCAGAGCTCCGTG

CCCATTAGCAACTCTGATCATCAAGGATCTATGATAGCAGTAATGGTTCCTGATTGA

>MS.gene009890.t1

ATGTCTCTGAGTGATGTAAACAAGAAATCAGAACTCATCTTCATTCCTGCACCAGGAATT

GGCCACTTAGCTTCAGCTCTTGAATTTGCAAAACTTCTAACCAACCATGACAAAAATCTT

TGCATCACAGTCTTCTGCATCAAGTTTCCAGGCACACCCTTTGCAGATTCATATATCAAA

TCAGTTTTATCTTCACAGCCACAAATTCAACTCATTGATCTTCCCGAAGTAGAACTACCT

CCACAAGAGCTACTAAAATCTCCAGAATTTTACATCTTGACTTTTTTGGAGAGTCTCATA

CCTCATGTCAAAGCAACTATCAAAACCATTTTATCAAACAAAATTGTTGGGTTAGTCCTA

GATTTCTTTTGTGTTTCAATGATTGATGTTGGAAATGAATTTGGTATCCCTTCTTATTTG

TTTTTAACATCAAATGTTGGTTTTTTAAGTCTCATGCTTTCCCTTAAAAACCGCCAAATC

GAAGATGTTTTCGATGATTCAGACCGTGATCATCAGTTGTTGAATATACCTGGTATCTCA

ATTCAAGTTCCTTCTAATGTTTTACCTGATGCTTGTTTTAATAAAGATGGTGGATATGTT

GCTTATTATAAACTAGCTGAGAGGTTTAGAGACACCAAAGGGATTATTGTTAATACCTTT

TCAGATTTGGAACAATCTTCTATTGATGCATTATATGATCATGATGAGAAAATCCCTCCT

ATCTATGCTGTTGGTCCTTTGTTAGATCTCAAAGGTCAACCTAACCCTAAATTGGATCAA

GCTCAGCATGATCTTATATTGAAATGGCTAGATGAGCAGCCAGATAAATCAGTTGTTTTT

CTATGTTTTGGAAGCATGGGAGTTAGCTTTGGTCCATCTCAAATAAGAGAAATGGCATTA

GGACTTAAGCATAGTGGAGTTAGGTTCTTGTGGTCTAACAGTGCAGAGAAAAAAGTGTTG

CCAGAAGGGTTTTTAGAATGGATGGAAATGGAAGGTAAAGGAATGATATGTGGATGGGCA

CCTCAAGTTGAGGTTTTGGCACATAAGGCTATTGGTGGATTTGTTTCACATTGTGGATGG

AATTCTATTTTGGAAAGTATGTGGTTTGGTGTGCCAATATTGACATGGCCTATTTATGCA

GAACAACAGCTTAATGCTTTTAGGTTGGTGAAGGAATGGGGTGTAGGTTTGGGGCTGAGA

GTGGACTATAGAAGGGGTAGTGATGTTGTAGCGGCCGGGGAGATTGAGAAAGGGTTGAAG

GATTTGATGGATAAAGATAGCATTGTGCACAAGAAGGTTCAAGTGATCAAAGAGATCGCT

AGGAATGCTGTTGTTGATGGTGGATCTTCTTTAATTTCTGTTGGAAAACTTATTGATGAT

ATTACAGGAAGCAACTGA

>MS.gene009877.t1

ATGGTCACAGATTCCAAACCCATTACCCACCACCGTGGTCTCATCGTCGGAAATTACACC

CACGACATCCTCCTCCACAACAACCACATAATCGCCGAATCCCTCGGCGGCGCCGCCTCC

TTCATTTCCACCGTCCTCGACTCCCTCTCTCTCCCTTTCCATCTCATCTCCAAAGTGGGC

CCAGATTTCTCCCACAATACACCCCACCTTCCTCTCGTGGTCCCCACTTCTCAAACCACC

CTCTTCCATGCCCACTTTGGAACAAATCACCCCGACCGGATTCTCAGACGGGTTAGATCA

TGTGATCCAATAACCCCGTCTGATATCCCGGCCGGGGCGAGGTTTTTATTCGGAATGGCT

GTTGGTGTTGCCGGGGAGATTCTCCCGGAGACATTGGAAAGAATGCTTGAGATTTGTGAT

TATGTTTTCGTTGATGTTCAGGGTTTGATTCGAAGATTTGAAGATGAAGATGGGTGTGTG

AGGCATGTGGGGTTAAAGGAGAGTGGGTTTTTTCATCTTTTGCCAAAGGTTGCGTTTTTG

AAAGCTTCTGAAGATGAAGCTGAGTTTATTGATTTGGAGGAAGTGAGAAAATGGTGTTGC

GTGGTGGTCACGCATGGGAAAGATGGGTGTGAGGTTTTTTGGAGAGATGGGTGTTTGATG

GTTGATCCGTTTGAGGCTTGTCAGGTTGATCCTACTGGGGCGGGGGATTGTTTTCTTGGT

GGGTTTGCTGCTGGAATTGTAAAGGGTTTGGGTGTGTATGATGCTGCTTTGTTGGGGAAC

TTTTTTGGTTCTTTGGCTGTTGCACAAGTTGGACCACCTAAGATTGATTTGAACTTGGTT

CAGGTTTGTTTCTCGGATTCATTTTTCCGTTGGCGAAATCATGATTTTCCTGGAACGAGA

ACCTGCGAACAGGATCACACGAACCGGGAACTCGCTCGCCCACATGGAGCAGCCGTGAAC

CAGAAGTGGTCTTGGATTCGCATTCCCGTTGCGGGTTTTCACTGTCGTGTGCCGCTCCTT

CGGGATTGA

>MS.gene009871.t1

ATGGCAAAAGCACTAGTTGAGGTGATGCCAGAGACTCGACATGGGTTATGTACATGGCAT

TTGATGCAAAATGGGATCAAACACCTAGGTAGCTTGATGAAAGGGGGAAGTGATTTCCTA

ACTGACTTTAAGAAGTGTATGTATAAATACGATCAAGAGGTGATGTTTGAAGCAGCTTGG

AGCAAGTTGGTTGCAAAATTTGATCTATTTGATAACAATTGGGTGAAATCAATGTATGGT

ATCAAGCAAAAATGGGCATCATGTCACATGAAGAAAGCTATCACTCTTGGAATGCGAAGC

ACTCAACTTAGTGAGAGTTTAAATTCCAGTTTCAAGGCTTGTATGAGACCTGGTATCAGT

GTCATTCAATTCTTCCAACACTTTGAGAAAGTTGTTGAGGAGAAACGATACAACGAGTTA

GTATGTGAGTATGAGTCTCGACATAAACTACCAAGGATGAGATATCAACATTCACCGATT

CTAATGCAACTCGCACAAGTCTGCTAG

>MS.gene009897.t1

ATGGTAAGAGGAGGAAATCATGGGGTCAATGGCGTTAAAATCTTTGGCAATGAAAATATA

AATGTCAATGACCCTTTCATGGATAGATTCTTATGGGGTCAAATGATTCACTACCCTAAC

CATCAACAAAACAATGCAAGTATGTTCCCTTACTCTTATGGTTCCAACCATGAGTTTATG

TGGCCAAATACTCAAGAATCAAGCTTTGTTGTTGATGGTGTCTTAGCAAATGAAGAGGCT

TTGAAGTGGACTAACTTCAACCAAACTTCCACATTGTGCCTTAAGGATATTCAAGGTTAT

GGTGAAAACACCAACATTGTGGGAAGGATAACTAAGAAAGAGACTTCCGAGGTCTTGATC

AAAGGACAGTGGACAAACGAAGAAGACAGGAAACTGATAAAGTTGGTTAAGCAATATGGC

GAAAGAAAATGGGCTCAAATAGCTGAGAAGTTAGAAGGAAGGGTTGGCAAGCAGTGTAGA

GAGCGATGGCATAATCATTTGCGTCCTGATATTAAGAAAGATAGTTGGAGTGAAGAAGAA

GAGAAGATATTAGTAGCTACACATGCCAAAATTGGAAACCGTTGGGCTGAGATAGCAAAG

AAGATTCCAGGAAGAACTGAAAATGCCATAAAAAATCATTGGAATGCCACTAAAAGGAGA

CAAAATTCAAGAAGAAAGAACAAGAAGAATGAAAATAATTCCAAGGGATCAAAACTAAAG

TCCTACATACTTGAAAACTATATCAAAACCAACACCTCCATCACTACAAAAATTACCAAC

ATACCCTCTTCCTCTCATGCGACACTTTCCTTTAGCCATGAAGAAAATCAATCAAATCCT

TTTTTCAATGAATTACCTTCTGAATCTTTTTCCAATGAATTACACTTCATACAACAAATT

TTGATGGATGATAATAATCAAAATGTTGTTGATGATGTGAATGAGAGTGAGCTTGTTCAT

TCAATCCAATACCCAAGTTACAACATGCACTTGGATACTCCTACCTCAATGAATAATTTC

CTTCCTTATGATCTTTACCTCTCTCAATTGTTGAATATGTAG

>MS.gene009925.t1

ATGTCCTCCGCCATCATTTTCGGCGGAGCAACCGCCGCTAAACCACCAACATTACCCAAA

AGCCACCGCACCATACTCTCCAACTTCCACTCACCAAAACTCAACCTCACGCGTTCACCA

TTTCCGAATAAAATCCAATTTTCCAATGGCGCCTCTGCCTCTCTCCGCTCCCTTCTCCGC

CTCCGTGTTTCTTCCAACGACGCTCAGTTCAACTCACTTCCCGAAAACAACCACCAACAC

AAACAACAACAACTTCAACCGAGTTTCACTGAGTTCATCACTTCTGAACGAGTCAAAGTG

GTGGCGATGCTCGCTTTGGCTCTTTCTCTCTGTAACGCTGATCGTGTAGTTATGTCTGTT

GCCATTGTTCCTTTATCTGTCGCTAATGGTTGGACTCGCGCTTTCTCTGGAATTGTTCAG

TCTTCTTTTCTGTGGGGGTATTTGCTTTCACCTATAGGTGGAGGAATGTTGGTGGATAAC

TATGGTGGCAAAGTGGTAATGGCTTGGGGTGTGGTATTGTGGTCACTTGCTACTTTTCTT

ACTCCCTTTGCTGCAGAGACTTCCCTTTTGGCTTTACTTGCTGTCCGTGCTTTGCTTGGT

CTGGCTGAAGGTGTTGCTCTTCCTTGCATGAATAACATGGTGGCCAGATGGTTTCCTCAA

ACTGAACGGGCAAGGGCTGTTGGAATTTCGATGGCTGGATTTCAGCTTGGATGTGCGATA

GGGCTCACACTTTCTCCCATTCTCATGTCGCAAGGCGGTATATTTGGCCCGTTTGTGATT

TTTGGTTTGTCTGGGTTTCTTTGGGTGTTGGTCTGGTTGTCAGCGACATCAAGCACTCCC

GACCAAAGTCCTCAGATATCAAAATATGAGCTGGAGTATATATTGAATAAAAGACAAACA

TCTTTTCCTGTGGAGGCAAAGAAAGTTAATGTTGCCCCTCCTTTCAGACGCTTACTTTCA

AAGATGCCAACATGGTCTCTAATCGCTGCAAACGCCATGCATAGCTGGGGGTTTTTTGTT

GTTCTTTCTTGGATGCCCATATACTTCAACTCAGTATATCATGTGGATCTCAGGCAAGCA

GCTTGGTTTAGTGCCGTTCCATGGGTTGTGATGGCTATCATGAACTACCTTGCTGGCTTT

TGGTCAGATATGATGATACAAAGTGGTACAAGTGTTACATTGACTCGTAAGATTATGCAG

TCCATTGGTTTTGTTGGTCCTGGGGTTAGCCTCATTGGTTTAGCTACTGCAAGAAACCCC

TCAGTTGCTTCTGCTTGGCTAACATTAGCCTTTGGCTTGAAATCCTTTGGTCACTCTGGA

TTTCTCGTGAACTTTCAGGAGATCGCACCACAGTATTCTGGTGTTATACACGGAATGGCA

AATACTGCTGGAACACTTGCTGCTATAATTGGAACAGTTGGAGCTGGTTTTTTTGTTGAG

CTAGTTGGTTCATTCCGAGGATTTTTGTTGCTTACATCAGTCCTATATTTTCTTGCTGCC

CTTTTCTACTGCCTTTACTCTACAGGAGAAAGAGTAAATTTTGATGATCCTGCGATGTTA

CGGATCTCTGTGGCAATGATGCATGTATGTTATGTGCAACTTTCTACCGGTGGTACAACC

CTCAACTACTTTCCAACCTTCTTCGGTTGTCCTCTTGGATGTCGTCAACTTAAAGATCTA

CCATCTTCGCACCGGTGGGTCCGTGTTGTTGATATCCGTCGCGGTTGTTCTAGTGTCGAC

TGA

>MS.gene009843.t1

ATGGGAAGCTATAGAGTCTGCGTGTGTTTCCGGCGGAGGTTTAAGTCCGGGGAGGCAGTG

GCACCTCAGGAAGTCAGGGAGATCTTCAACAAGTATGCTGAAGGTGGGGCCCACATGACT

CCGGACCAGTTCCGGAGGTTTCTCGTGGAGGTTCAGGGTGAGGTTGATGATGAGAAGGAT

GGACTTGATACACAGAAGGTGGTGGAGGAAGTGTTGCAGAAGCGGCATCATATTACGAAG

TTTGCAAGACATAATCTCACGCTTGAAGATTTTCATCATTATCTTTTCTCTACAGAATTC

AATCCTCCTATTAGATCTAAGGTTCATCAGGATATGACAGCTCCATTGTCCCATTATTTC

ATATATACTGGTCATAACTCCTATCTCACTGGAAATCAACTCAGCAGTGATTGTAGTGAT

ATCCCTATTATAAAGGCATTAAATAGAGGTGTGAGAGTTGTTGAGCTTGATATTTGGCCT

AATTCCACCAAAGATGATGTTCTTGTTTTACATGGAAGGACATTGACCACACCTGTTGAT

TTGATCAAATGTTTGAAATCCATTAAGGAACATGCGTTCGCTGCATCTCCCTATCCAGTC

ATAATAACTCTTGAAGACCATCTTACCTCAGACCTTCAAGCAAAAGCTGCTCAGATGATT

ACTCAAACATTTGGAGAAATGTTGTTCTGTCCAAATGATGAAAATTTAAAAAATATTCCG

TCACCTGAAGAACTGAAGTATAGGATCATGATATCGACAAAACCTCCAAAAGAGTACTTA

AATTCCAAAAGTGTGAGGGAGAATTCTGAACAGTTGCTGAAATCAAAATCAAAGGATTCT

GATGAAGACGAATGGGGAAAGGAAGTCGCTGATGTTGTACATGCACAAATTGAAGATGAC

AAGGGTGACTGTGACTCACATCACCAGAATGATGATGATGAATCAAATGATGATGACGAT

GAAACAAACCAGGAGAGTGAGTATAAGCGTCTAATCGCTATTCATGCTGGAAAACCTAAG

GGTAGTTTGAAGGAAGCCTTAAAAGTTGAAGATGATAAAGTGAGACGCCTTAGTTTAAGT

GAGCAAGCTCTTGAAAAGGCTGCTGAGTCACTCGGAACTGATCTTATTAGGTTCACCCAG

AAAAACTTTTTGAGGATATATCCGAAGGGTACACGTGTCACCTCGTCGAACTACAAGCCA

CTGGTTGGCTGGATGCATGGTGCTCAAATGGTTGCATTCAATATGCAGGGATATGGCAGA

AATCTTTGGCTGATGCATGGAATGTTTAGATCCAATGGGGGATGTGGTTATGTGAAAAAG

CCAGACTTTCTTATGAATGTAGGTCCAAATAATGAGGTATTCAATCCTAAAGAGAAATTG

GAAGTGAAGAAGACTCTTAAGGTGAAAATATATGTGGGAGATGGATGGAGTATGGATTTC

AAACAAACTCACTTTGACTCATATTCTCCACCAGATTTTTATGCTAGGGTTGGCATAGCT

GGAGTGCCAGCTGATGTAACAATGAAGAAAACAAAGACAATAGAGGATAACTGGATACCT

ACTTGGGAAGAAGAGTTCACATTCCCTTTAACTGTCCCTGAACTTGCTTTGCTCAGAGTG

GAAGTGCATGAATATGACATGTCTGAGACAGATGATTTTGCGGGACAAACATGTTTACCG

ATCACTGAACTAAAACAAGGGATCCGTGCCGTTCCACTTTACGATCGAAAAGGAGAGAAG

TACAACTCAGTTAGGCTTCTTATGAAATTTGATTTTATTTAA

>MS.gene009885.t1

ATGTCAAAGTTTGGGAGAGCCAGCACAGGCAATGACGTGACTGCTATTTTCAATTGCTCA

AACGCTGATGCAGCAGAAGGATTCGATTTAAAACTGTCTTTCTTTACATGCCTTCTAAGT

TCTCATTACTTGAAATCTGAATTTATTTGTCTTCACCCTGCTGCTGCTGTTTGGAATAGA

TCCCTTCTAAGTTCTTCGTACTTGAAATCTGAATTTATTTGTCTTCATCACCCTGCTGCT

GCTGTTTTCAAGAGAGCTGCAAATACTGGTTGGGGTGATAACGACAATGCAAAATGGGAT

AATAACAAATATGATGGTTCCAAGAAAGAAAGAAGAGACTATAAAGACGATGAATTTGAA

CATCGGTCAATAGAAGATCGACATATGAGAGAAGAAAAAAGATCAAGGAATGGTTATGAT

GGTATGCAGCCTGAGCCAAGAGATCATAGAAGGGAAGACAGGAGGTCAATAAAGCAAGAT

GATGTCGAGTTTGAGCCCAAATCAAGAGTTTCTGATGTTAAAGAAGATAAAAGACCGAGT

AGGAAGAATGTTGATGATTTGGGAAACAAATCAAGAGAAACACATGGTAACAGGGAAGAG

ATGAGATCAAGAAAGCACACTGACGATGAAGCTGTGCCGAAGTCAAGAAACAACAGATCA

TATAGAAAAGATACTGATCGATCTGAATCAAAAGAAAGAAACGATTCTGACCAAAGTAAA

GAGAAGAGGTCCAATAGTGGCAATTTGAGGTCTTGGTTTATCGTGGACAAGAATGGGTAT

TGA

>MS.gene009930.t1

ATGGCTTTTCGTGCCTTTAGATCACAATCAGTATCAACACAAGGAGCGTATAACTTAGCA

CAGAAAACAACAGAGGAGCCTGTTCCAAGCAAAGTTGCACAAAGCACGGTCACATGTTTC

TATCAAGCCAATGTAGCTGGATTTTGGAGAAATGTCTCAGTTTTATGGTGCAAAAACCTT

ATGAACCATACGTTACATGTCACGGTCGATAGCGTAGGAGGCGATTCACAATTCAGCTGC

AAGATTGATGTGAAGCCATGGCCCTTTTGGAGTAAAAAAGGATACAAAACATTTGAGGTC

GAAGGGAACCATGTGGAAGTTTATTGGGATCTTCGGAATGCCAAATTCACCGGTAGTCCT

GAACCGAGTAGTGATTACTATGTAGCGTTAGTTTCGGACGAAGAGGTTGTTTTGTTGTTG

GGAGATTACAAGAAAAAGGCATATAAAAGAACAAAGTCTAGGCCTGCACTTGTCGAGGCA

ATGTTACTTGTGAAGAAAGAAAACGTTTTCGGGAAGAAAAGTTTCTCTACGAAGGCGAGG

TTTGATGAGAAGAGAAAAGAGAATGAGATTGTTGTTGATAGTTTAACAGGTAATAGTACT

AATGATCCTGAAATGTGGATTAGCATTGATGGAATTGTTTTGATTCATGTTAAGAATTTG

CAGTGGAAGTTTCGAGGGAATCAAACTGTTATGGTTAATAAACAACCTGTGCAAGTTTTT

TGGGATGTTCATGATTGGTTGTTTAGTGGTTCTGGTTCAGGACCTGGACTTTTCATATTT

AAGCCAGGGCCTGTTGAAGATGAAAGTGAAAAGGAAGGTAGTGCTGTTCAAAGTTGTGAA

AGTGATGATGGTAGTCTTGGTTATCATTCAACTCTTAATAATGCTACTTTTGAGTTTTGT

CTTGTTCTTTATGCTTATAAGATTGAGTAA

>MS.gene009937.t1

ATGAAATGGGTCGAAGGTTCGTATGAAAAGGATGGTTCGAACGGTGGTTCGGAAAAGAAA

GGTGGAAGAATCCAGAAGGGTGGGTCGAAGAAGAAGAAAGGTGGTTCAAAGGCTGGTTCG

GCTGGTTCACAGAAGAAGGTTGGTGAAAAGGCATCTGGGTCCGGAAAAGATGTGGTGAAT

GACAAGATTCCCGACAAAAATGTGTCAAAGGTTGCTGAGAACCACTCATTGAAGGTAAGG

TTCCACCACAAAGGCTATTTTACATCTGACCCTGTTGTTGACTATAATAATGGTGAGATC

TACGAATTTGGTAAAAGGGACATAGATGAAGTCAATTTAGTAAATTTGGATAAGCTTGTA

AGAGAGATAGGTGTGAAGGGTTCATATCAGTTGTGGTATGTCAGTCCTGGTGCAGAGTTG

AAGGATGGTCTTAGTGCACTGAAGACAGATAGAGATACCATTAACTTCATCAATGAGTTC

AAAGGTGAGTCAGTAGCTGATTTGTATGTAGAAGGAGAAGAAGTTGTAGATGCCAGATAT

GATACTGATATTGAAGAGGTAGAAGAAGTTGTTGAAGAGCAGTCTGAATATGAAACAGAT

CCTGAATATGTTGCTGAAGGAGAAGATGGATATGCTTCATTTGCTGAAGAAGGAGCTGAA

AGTGTAGAAGGAGATGATAGTGTAGCTGGATCTCTGAATGATAGTGAGTATGATGAAGGT

TGGGAATGGACCACTGTTTTGCCTGAACAAACTGTAAATCCAACTGTTGTTGTGCCTTCT

GAAGTGTCTGCTGATAACAAATTGGTTGGTGTTGAGTCATCAAGGAATCCTGCTGAAACA

ACTCTTTCTGACTTTGAAGATGAGAATGGAGATTCATCAGATCTTGATAGCCCAAACTCA

GATGAAAGTGATAGAGGTAATACTAGATCTAGGAAGTTTAAGTATTCTGAAGGTGATGTT

GTGAGGTACCAATTAGATACCTATGAGAATCTCATCCTTCCTTCAAATGGTCCAAAATTA

TGGCCACAAGTTAACACTGAGCAACTCTTACCCCCTTATGTAAGAAGGATTCCTGGCAGG

CCCAAAAAACTGAGGAGGAAGAATAATGATGAGGCTACATCAGGTTCAGGTACAAGGCTG

TCAAAGAGGAACCAGAATATTGTTTGGTGTAAAAGGTGCAATTGTCTTGGCCATAATCAA

AGAACTTGCAAAGGGAAGTATGCTGCTGAAAGGATGATAACTCCAGGTGGAAATAAGGAC

AATACCCAGGTTGAGCAACCTGATGCACATGCTGCAGTTAACAATAGGATGAGGCCCAAA

AAGAAGCCTGCTACAAAGAAAAAGGCTAAGGCTACTTCAACATCACAAGCTGCTACTGCA

CCAGCCTCTTCTCAGCCTTCTGAACCTGCCACTTCTCAGCCTTCTGCACCTGCAAATGGT

CAAATTCCTCAGCCTCATGTTGCTGTTCATATTCCTGCACCTGCATCATCTGTTCAAATT

CATGCATCATCTGCAGCTGCACCTTCTGCACCTTCTGCAAGCATATCAGTACAGGCTGCA

AGCAACTCAGCTCAAAATACTTCACCATTCAAAATAGGAAAACACATTGTGGGAGATGAA

ACACTTAGTGTGCCTAGGAAAGTTAGGCCCACTGGTTTGAAGAGGAAGCTTGATGATGTG

GAAGTAATTGGGACTCAGCAATCAGTTAACAAAGTGTGA

>MS.gene009853.t1

ATGTCCTTACAACTTGGTCAAGTTCCAACAATTGTTATTTCATCTTCAAAGGCTGCAGAA

TCATTCCTCAAAACTCATGACATCCATTTTGCAAGTCGGCCGAAGACTCAAGCATCTCAG

CTCATTTTTTATGGTTCCAAAGGGTTGGCTTTTTCTGAATATGGTCCTTATTGGCGTAGT

GTAAAGAAACTGTGCACTTTGAAACTTTTAAGTGCTTCAAAAGTTGAGATGTTTGGTCCT

ATTAGGAAGAAGGAGTTGGATGTTTTGGTTAAGTCATTGGAGAAAGTTGCTTTGGTAGGT

GAGGTAGTGAATGTTAGTGAGATTGTAGAGAATCTTATAGAGGATATTGTGTATAAGATG

ATATTGGGTAGGGGTAAGTATGAGCAATTTGATTTGAAGAAGTTGGTTCAAGAAGGAATG

GCTATGCTTGGAGCTTTTAATCCGGCTGATTATGTTTCTTGGCTAGGCCCTTTTGATCTT

CAGGGATTTACACGAACCTGCAAGAAAACCGGTAAAGCACTTGATGAGGTATTGGAGATG

ATAATAAGAGAGCATGAACAAAGCACTAATGTAGACAAAGTTCGTCATGAAGACTTCGTA

GACATACTTCTCTCAATTGTGCACCAAACCGTAGATCCAGAGAATGAACAAAGTGATGTC

ATTGACAGAACTAACATCAAAGCAATTTTACTAGACATGATTGTGGCAGGAATTGATACA

GCCACTACCACGATTGAGTGGGCTTTATCTGAACTTTTCAGACATCCAAGAGTGATGAAA

AAGCTTCAAAATGAGATACAAAATGAAGTAGGAAATAAAAGAATGGTTGCAGAAAAAGAT

TTGAAGAAGTTTAATTACCTAGATATGGTGGTTGACGAGACATTAAGACTTTACCCTGTT

GGACCATTATTACTCCCTCGTGAATGTAGAGAGAGAATAACAATTGATGGATATTTCATA

AAGGAAAAGACTCGAGTTATAGTAAATGCATGGGCTATAGGGAGAGATTCTAATGTTTGG

TCAGAAAATGCTGATGAATTTTATCCAGAGAGATTTAGTGATAAGAAAATGAATTATCAA

GGCCAGGAGTTTGAATCTATACCATTTGGTTCAGGTCGTAGACGATGTCCTGGGATTCAA

TTGGGTTTGGTTACTGTTAGATTTGTTGTAGCTCAATTGGTGCATTGCTTTAATTGGCAA

CTTCCACATAATATTAGTCCTTCCAATTTGAATATGGAGGAGAAATTTGGAGTCACTATA

CCAAGGGCTCAACACTTGCACGCAATACCTAGTTATCGTTTGGAATGTGATGAGAAGCTT

GAATAG

>MS.gene009886.t1

ATGCCGGTAAAAGGGCCGTCAACTGCTTTGCAGCCTCTGCCTCCGATCAGGAGATGTATA

TCTGAAGTTATTGATCCTTCTGAGGTTAAAGAAGCAGTTGCTTGTTATTTGAACTCTGCT

GAAAAAACCACCCCTGAATCTGATTCAATGAGGCTTAGAAGGATGAAGGATCGGTTAAAA

GAAATGAAGAAAGTGTGGGATGAAGTTATGGAAGACGAGGAAGAAAATGAAAAAGAACAA

GAACAGGAAGAAGAGCCCTCTCCTGATGCTGAAGATGAAAAAGAACAAGAACAAGAAGAG

CACTCTCTTGTTGCTGAAGATGAAAAAGTCATATCTCAGGATGAATTGGGAAATGATTAT

GAAGAAGTGGTTAGTGTAGAGTGGATTGATAAGTGTTTAAGCCTTACTTTCAAGTGTCCA

TGTGGCAAGGGATATGAGGTTCTTATATGTGCAAACAATTGTTACTACAAATTGGTGTAG

>MS.gene009915.t1

ATGGGTTTGAATGCAAGAGACATGCAACGTCATTTGTTCAAAGTTCTTCATCTTTCAGTT

GAAAAGAGCTATATTTTGATGAAAAATCATCCAAGAGTTTCATGTGCTTTGTTAGTCTTC

ATCATCATGTATATATTTCTTTCTTGTATATACAATTTCTTGGTTTTCTTGTCTCCATTT

ATTGTGTTCACTTCAATTTTGGTTAGAATATTTTGGAGTTCGGAGGAGAAACTTGTTAGA

TGTGTGGAGAAAAAGGGAGATGAGAAAAAGGTTGAAGCGAACAAACCACCTAAGGTTCCT

AAGAATGAGAGACGCGGAATGCTTTATAAGTATCCATCACAAAATGCAACAAGTAGAAGA

AGAAATTTCACGGGGAAGAAATTGGATGTATATGGCGATTTAGAACAAAAAGCTAAGAAT

TTATCAGCAGCGTTTTGTAACGAGTTCACTAGAAAAAACACCGAGATTAGATCAGGATTT

AGATATTTTGAGAAAGAAATTGATCCTTTTGATTATAAATTTCCACCAAGAAAAGTTGAT

GGAATTGATCCTTTTGATTATAAATTTCCACCAAGAAAAGTTGATGGAATTGATCCTTTT

GATTATAAATTTCCACCAAAAAAAGTTGATGGAATTGATCCTTTTGATTATAAATTTCCA

CCAAGAAAAGTTGATGCTATTGATACTTTTGATTATAAATTTCGACCAAGAAAAGTTGAT

GCTATTGATACTTTTGATTATAAATTTCCACCAAGAAAAGTTGATGCTCCTAAAAATCAA

ACATTGCTTTCAGAGCCTTCAATGGTTGACCTTGTGACTTGTGGTACAAGTTATTATGAT

TGTCAAGAGAAAAGCACAGAAAAAATGGAAGATGAGAAAAAGGAAGTTGAGGATAACAGT

ACTAATAAAGTTGTAGAATTAAAAGAGGATGATCAAAAAAAGCTAATGGATCTTGGGATT

TGTGAGATGGAAAGTAACAAAAGGTTGGAGAGTCTTATAGCTAGAAGAAGGGCAAGAAAA

CTATTGAAGTTGGAGATTGAGAATGGTTTAATTGATATGGAATCAATTACACCTACCCAA

ATTGCACCATTGTTTATTGCAGCAAGAATTAACCCTTTTGATTCTCCAAGATATTTTGAT

GACATAGAAATACCTGGTTCTGCTCCATCTGTTTTGAGAAGTCCATTTGATATTCCTTAT

GAGCCTTTTGAGGAGAAACCAAATCTCAAATGGGATAGTTTTGATCAAGAATTCACAAAT

GAAATGCTATTAGAACTCAGACAAGACCTTCATGTTAGAGAGAATAGAATACCAAATTCA

AGAGTTAGAAGGCTTTCAGGTAGGGGAAATCACGGTAGGCCTGAAAAGTTAAACTCCAAC

GAAGGAAGTGAAAGTGAATTACAAGCTCCAAGTCCATCAAATGGAGGAGAGGAAACTACA

CATGAAGAGGAGGAGAAATGTAAAATTGACAGTGATGGAACGAAAGGTGGGGAAGTAGAC

AATAGCGATCCAACAAATTCCATTTCAGATCATGCAAGTGAACCAAACATAATTCCAACA

ACAAAAAATGTTGAAGTCCTTGATTTTCCAATATCTAGTACTGATGTTAGTAACATAAAT

GATTCTTTGTATGATTCTCTTTCAACTCCTGAAGATAAGAATAAGGAAAACACGTTGTTT

ACAAATGGGCTAATTCGTCATGCACCATCAGTTTCCTTAGCTTCTGATTTACAAGTTGAG

TTTTCGGAAATTGGTTCACCAACATTGACAACTGATGAGAGTCATGAAGATCTGTGGGGA

GAAAATGAAGTTAGCGAGCATGATGATATTTTAGAGGCAGACAATTGGAGTGACATTGGT

TCTTCATCTATTTCTTTACAAAATAAAGATGAAGAAAATGCAGCTCATGTGAGTTTTATG

TCATCAACATCCGATATACTTGACGATTCTCCAACTTATCCAACGAGCAGTGATCATAAC

ATCCTTGGTAATGTGAGACAAACTACTGGTGCATCTCAATATTCTTCGGATGTGTTAGGG

CGTTGGAAGCGGTTGATGAGGTTGATGGATACTCGTGTTGATCATTTACCACAAGAAAGG

CTTTCAGAAAATTTGGAGGGATGCAACCAGACACAGAATTTAATAAACAAGGCACAAGTT

ATGAATGATGTGAATGACTCAGCAGTCACCGAGCAAGAAAATACAAAGGACTTGAGGAGT

AATGAAGAGCCTAGTGCATCAGGAGTGCGGCAAGAAGTGGTTGATGAAGTCTCAACTATC

ACAAGTTCGTCGTCATCGTCTTCATCATCACCAAGGTCTGTATTGCCAATACCACATAAG

ACAGAAGCAGACCAAGAGATAAATGGTGTTCAACAATCTGACATGGAGAATGTGATACAA

GAATCAGTAAATGGTGAAGGTTCACTTGAATCCATGTCTCAAAATGTTCAGCCTTCCATG

GATGATTCAACTGATGAATCACATAATGGTGACTTGATTCATTCTCAGGAGGGAACTAAT

CTCTTGGAGAACTCCATTGAAGAATCAAATATGTCCAGTAACATGAATGATGCAGAAGTT

TATAACCAAGAAGACCAAGAGTTAAAGAATAATGAAAACAGTGAAATCAAATTTACTTCC

CAGGGTAGACAGGATACTTCAGAAGAACCATATAGACTAGCAGAGGTGCAATTGATTTCT

CCAATGCTAGAATCATCCTCAGAAAGTCACATAGAGAATGAAGGAGAATCCCTAGCATCA

TTGAGAGAAGAGGCTATCACAGAACCAGTTATCAATGCTTCTGCTAAAGATTTTGAAGGA

AAACACGATGACTTGAATGAAAATCAAACCGAAAATCATAGTTCAAAAGAAGAGAACTAT

TTAAAAAATGAATCAAACCAAGTGGTTAAAGATCACATTGAAAAGGATCAGTCAGACAAA

GGTGACATCTCTCAAGATCCTGCACTACCAATGGTCAATGAAGTAACCAATTCTGAAGAC

ACGTTAGGAGAATCTGACAAGATGGACAAAAATGAAGTCGCTGACAAAGAATTGCACAAA

AATAATCAAACCATGGTTTTATCTGAACTAGAAGGAGAAACTGATAAGATCACTGACATA

GTACATACGAATGATCCTTAA

>MS.gene009874.t1

ATGAGTTCCAACGAATCGGGCTGCATTTACGCCACCTTGATCCTCCACGACGATGGAATC

CCTATCACCGCGAAGAAGATCGGTACTTTGTTGAAGGCCGCTAACGTCACCGTTGAATCT

TACTGGCCAGGCCTATTCGCTAAGCTTGCTCAGAGCAAGAACGTCGATGATCTCGTTTTG

AACTCCGGCGCTGTTGGTGGTGCCACCGTTGCCGTATTTGCACCTGCTTCTGGAGGTGGA

GCCGCAACAACCGAACTGCAGCCGTTGAAAAGAAGGAGGAAGCCAAGGAAGAAAGTGACG

ATGACATGGGCTTTAGTTTGTTTGATTAAGTTTCTCTGTATAGTTTGTTGTAGCCAGACT

AGGACTCCTTTCAAGGGGGTGGCTGAATCCAAAACTTGCAATCAACTATCTCTTTATAAA

TTGTTTATCTAA

>MS.gene009842.t1

ATGTTTAAGCTCAAGGTGGCTGAACCACCAGAAGAAATCAACAATATTTTTAATAGGTAT

GCAAAAAATGGTACCATGACCATGGATGAATTGTACTACTTTTTAGTTGATTTTCAAGGA

GAAAAGGGTGGTGAAGCTACCCAAAAACATGCTCAAGATGTTTTCCACAGTTTAAAACAT

CTCAATATATTTCAAAGAAGAGGTCTTCATTTTGATGCCTTCTTTCGTTATCTCTTTGGT

GACCTAAATGGCCCCATAGGTGATCAGGTGCATCAAGATATGAATGCTCCATTGGCTCAT

TATTTTTTGTACACAGGGCACAACTCCTACTTAACTGGAAATCAATTGAGCAGTGAAAGC

AGTACTATCCCAATAATAAAAGCTCTGAAAAAAGGAGTTAGAGTAATTGAGTTGGATTTG

TGGCCAAATTCTAGAGAAGATGATGTGGAAGTTCGTCATGGAGGGACTCTGACTTCTTCA

GTGAAACTTAGAGATTGTTTGAATGCCATTAGGGACAATGCATTTGATGCCTCAAAGTAT

CCTGTTATAATAACTTTTGAAGACCATATAACTCCACCTCTTCAACGCAAAGTCGCCAAA

ATGGTGGATGATATATTTAGAGGTATGTTGTTTCGTCCAAATTATTCACATCAAATGAAG

AAATTCCCTTCACCAGAATCGTTGAAGGGGAAAATTCTGATTTCAACAAAACCACCGGAG

TCTCCTGAAAGTCAAAGCCAAAAGATACAAGAGGAAGAAGTTGAGTTGCTTGAGGACAAA

GATGATGGATCTAGAGTCAATGATGAGGATGAATTGGGTGATGAAAGTGAGGAGGAGGAT

GAGACTCTTGGATATAGAAATTTGATTTCTATACATGCTGGGAAGCCAAAAGGCAGTGTA

GAACATTGGTTGATGGAACATGATCAAGTTAGGCGTCTTAGCTTGAGCGAACAAGTTCTT

GAAGAAATCGCCAAAACTCATGGGAATGATATTGTCAGATTCACCCAAAGGAATTTGCTT

AGAATATATCCAAAGGGTTCACGTGTGGATTCCTCCAATTATGATCCTATGAATGGATGG

ATGCATGGAGCTCAAATGGTAGCATTTAATATGCAGGGACATGGGCATTATCTTCAATTT

ATGGAAGGAATGTTCAAAGCCAATGGTGGATGTGGGTATGTTAAGAAACCTGACATCTTA

TTGAACAATAATAAGATGTATGATCCTAGAGTATACCGACCAAAGAAAACTCTTCAGGTT

CACGTATACATGGGTGAAGGGTGGCAATCTGAATTTGGCCAAACACACTTTGATTTTTAT

TCTCCGCCTGATTTTCGTGTACAGGTTGGTATCCATGGTGTTCCAGTTGATACGGACACC

AAAGTAACTAGGACCAAGGAAGATGAGTGGGTCCCAGTTTGGAATGAGGAGCTAAACTTT

CCATTGACTTATCCAGAATTGGCCCTATTACACATTAAAGTTATTGAACGTGACTTTTCT

GGACAACATGATTTTGCTGGACAAACATGTTTACCTGTATCAGAGCTAAGAGAAGGCATT

CGTTGTGTTCGTTTATGTAACCGCAAAGGTGAACTTTATAAGCATGAAAAACTCATATTT

TTGTCTTCTTCTTTACCCGACGACCTGAAACGGGTTTTACCGACCCGCGTTTCGATCCGA

TCGGATCTCGCATTATACACAGTGAAATTTAGCGATTTTTGCATAGTTTTGATCGGAATT

GAAAGCCACGCACAAACAACCTCCCTCCACACCATCTCCGCCTTACTCTCCCTCCCATCT

TCATCCCTCCGTGACGCCATCTGCCGAGCTCGAACCGCCGTTAGATTCCCGTCATTCTCG

CACCGTCTCCACCTCCGAGCACTTGAGCTCTCCGTCGGCGTATTCCTCGACCGTCTCCCG

TCGTCAAAACCATCGCCGGTAGAAGAACCGCCGGTTTCGAATTCTCTCTTGGCAGCGATC

AAACGGTCGCAGGCAAATCAACGCCGGTCGCCGGAGAGTTTTCATTTCTATAACCAAAAC

GGAACGACGTCGTCTTTGTTGAAAGTTGAACTGAAACACTTCGTTTTATCGATTCTTGAT

GATCCAATAGTGAACCGTGTTTTTTCTGAAGCTGGTTTTCGTAGTTGTGATGTTAAACTC

GCTTTACTTCAACCACCGGTTCAATCATCAACCCGGTTTTTATCCTCTCCGCCGGTTTTT

CTTTGTAATCTTGAACCGGGTCGAACCGGTTTAACTCCTTTTCCTTTAGGGGTTGATGAG

AATTCAAGAAGAATAGCTGAGGTTATAGCAATGAAAGGTAAAAAGATGAATCCTTTGTTG

ATGGGTGTTTATGCAAAAGATGCTTTTAGGAATTTTGTTGAGTTGTTACAAAAGGGTTTA

GGTGGTGGTTTGTTTCCACCTGGGATGTCTGGTTTGAGTGTTGTGTGTGTTGAAAAGGAG

ATTGTTGAGTTTGTTAATGATGGTGGGAGTGAGGAGAAGATGGGTTTGAGGTTGAAGGAA

GTGGGATGTGAGGTGGAGAAATGTTTGGGTGCTGGTGTTGTTGTTGGGTTTGGAGAGATT

GAGGTTTTGGTTGGGGATGATGTGGATGGCGGGGGTGTTAGGTTTGTTGTGTCGGAGTTG

GGAAGATTGTTGGAGGTTTATGGTGAGAAGGTTTGGTTGATGGGTGTGGCTGAAACGTCG

GAGGCTTATTCGAAGTTTTTGAGGTTGTTTCCGGGTGTGGAAAAGGATTGGGATTTGCAT

TTGGTTACTGTGACATCTGCTACTCCTTCAATGGAAGGACTCTACTCTAAATCCAGTTTG

ATGGGGTCCTTTGTTCCATTTGGTGGATTCTTTTCTACACCTCCTGAAAGCAAAAGTCCC

ATTAGCTCTACAAATGCATCATTTACTCGCTGTGACAAATGCAATGAAAAATATGAACAA

GAAGTTGTTGATGCTTTGAAGGTAGATCCTGCTACTCTGGCATCAAATCACTCAACAAGC

TTGCCTTGGTTTAAAAAGGTTGTTGATGTGGATACACGTGGAGGACTTGATGCGGCAAAG

GAAAATGAAGAAAATACAAGCTTGAATGATAAGATATTGGGATTTCAAAAGAAATGGAAT

GATATTTGTCAACGTCTTCATCAAGCAAGGTCTCATGTCCCATCACTTGAAGTTTTACGG

TTTGGTTCAGGTTTTAACGAAAGCAGCAGTAAAGATCCATCACTTAATAAACTCCAATGT

TCTAGTCCATTTTCCTACATGCCAAAAGAGTTGCATGGTACTTTTCCATCAAAACAATTA

TCACTTATTCCAGTACATACTGGTACAGTTAGTGTCAATGTTAGAACTCCTTGGCAACAA

AATGACATGACGAAAGTTACAGAGACTCGACAAAATGACATGACGACTCCTTGGCTCGCC

CCTTCACGTATGGCCAATACGGGTGTACTGGAAAACAAATCATCTTCATCCCTTATTCCT

GTGACCACAGATTTAGGATTGGGAACGTTATATACATCAACATCAATTGCTCACAAGCCA

GATACTTCAGAATTTCAAGATAATATAAAGCATTTTGAGCCCTTTCCAGACTCTACTTCA

GCTGATTCTGTTGCTGTCAATGGAAATACATCACACCAGATTGCTAGATCCTCCTGCCCT

TCTTCAAATTTGGCAACAAAATTTGATTCAGTAGATTTCAAGTCTCTTAATAAACTACTC

TTTGAAAAGGTTGGCTGGCAGAATCAGGCGATATGTGATATCAACCGAACTCTGTCCCTT

CATAAATCCGGTGAAGGGAAGAGCAGAGACTTGCATGGTAGAGCAGACATATGGTTTGCT

TTTCTAGGACCAGATAGAATTGGAAAAAAGAAAATTGCTTCGGCACTTGCAGAGACTATA

TTTGGAAATACAGAAAGCATCATCTCTTTGGATCTCGGCTTCCAAGACAGGCTTTACCCA

CCAAACTCGATTTTTGAATGCCAAAAGTCATTAAGTTATGACTTGTTTATAAGGAAAACA

GTTGTGGACTATATTGCTGGGGAGTTGAGTAAAAATCCCCATTCCGTTGTCTTTCTAGAA

AATGTAGATAAAGCTGATTTTCTGGTGCAGAGTAGTTTGCTGCAGGCAATAAGAAGAGGT

AAATTTCCAGACTCACGTGGAAGGGATATAAGCATCAACAATGCAATCTTTCTTTTATCC

TCGTCTGTCTGTAAAGGTAACGGCTCTTCTACTTTGGTGGAAGGCAACTTGTTTTCTGAA

GAAACAATCCTTGAAGCCAAAAGATGTCAAATGCAATTACTACTTGGAGATACATCTGAG

GATGCCAAAAGAAGCTTTAGTACAAATGTCAAGATTGTGCGGAGGAAAGGGTTTTCCAAA

CCATCATTCATGAATAAAAGAAAACGAGCTGATACTAGTGACTTCAAAGAGGGAGCAGCA

AGCAAGATGCAAAAACAGGTTTGCGAGACATCAATGTCCTGTCTGGATTTAAATATGCCT

CTAGACGAGGGTGAAGAAGGTATGGACGACAATGACCATGAACGCGATTGTGTAGTGGAA

AACTCAGACTCATGGTATAGTGATTTCTGCGATAAAATGGATGAAAAAGTGGTTTTCAAG

CCATTCAATTTTGATGCGCTTGCTGAACAATTACTAAAAAGCATTAGTATACAATTTGAA

AAGACATTTGGTTCAGAGTTTCAGTTGGAAATTGATTACGAGGTTATGGCACAGATACTT

GCAGCTGCTTGGTTAGCAGACAAGAAAAATGCAGTGGAGGATTGGGTTGAAAGTGTACTT

GGCAAAGGCTTCATTGAAGCTCAGCAGAAATTCCACCCTGTAACTAAATATGTTGTGAAA

CTAGTTAATTGTGAGTCTATTTTTGTAGAAGAACCTGATCTTGGACTATGCCTTCCAGCT

AGCATTAACTTGAAGTAA

>MS.gene009945.t1

ATGCAAGAGGTACTAGCAGCAATGGTTACTTACCTCATATTTGCAAGAAGGTTTCTACAG

GGTTATGATTGTGACGGATATAGAATGAGAGAAGAAAATGGTTTTGTAGTAACCTGCTGG

CAGGACAGGTTTTCTATTACACTGGCTGCATCATTAGCTGCACACCATGGAGCAACATCC

GTGGCTGCATTTCAAGTCTATCTGCAGGTTTCGTTGGCAGTGTCCCTTCTTGCGGATAGT

CTGGCTGTTGCCAGGCAGGTTAGAGAGGAAGTTGAATTGGCTCTAAAGAAGCATTTGAGT

AGCATGAAACATTTGGAAAGAGCATATCTATCCCTGTCTTTACAGAAGCTTGAATTGAAG

GAGAAGAAAGAACCAAGTAATGCGACTGATAACTTTAGTCCTGCCAATAAAATTGGAGAG

GGTGGTTTTGGTTGTGTCTACATGGGACGACTGAAAAATGGAAAACTTGCTGCTATAAAA

GTTCTTTCAGCTGAATCAAGACAAGGGGTGAAAGAGTTCTTGACGGAGATTAGTGTGATC

TCTGAAGTAGAGCATGAAAATTTGGTTAAGTTGTATGGTTGTTGTGTTGAAAGAAATAGT

AGGATATTAGTCTACAATTACCTTGAGAATAATAGCCTTTCACAAACTCTTCTAATGGAA

GTATTCCTTTAA

>MS.gene009923.t1

ATGCCTCGTAAACGCTTCTTCGAAACACCAGAACCCAAAACAGGTTTAGAACATGCCCAA

TATTTTCTCAAGAAAATAGGGCTGGGGCCGAATAATTACTACTTCTGGAAACAAATGGGG

AAGGCTTTGGCGTGCACTTATGCAGTGATGGGTGCAGCGTGGTTGTTCAACGAGACATCG

CCATTGGGTTGGTGGACGTTGAAGCCAATGCCCAAGGAAGAGAAGGAGCTGGAACATTTG

TATCAGCGGCGTGAGTTTCCATATCCGGGAGACGAGGAAGCTATGGAGGAGTTTATTAAA

AAGGGGGGAATGATTGGAACAACTATTGGACCTAAAGGGATGGTGGAGAATGATATGGAT

GAGAGTGATTATAAGAAGGAGCTTAAGGATAAGAAGTTTGAGCAAGAGGCTCAGAAGCTT

TGGTTTAGGATGAGGAACGAAGTTATTGGTGAGCTTCAGGAGAAGGGTTTTGATGTAGAT

CAATGA

>MS.gene009929.t1

ATGGGTGATTTACAGATTCAACAAGAATCCATGTTGATCCCTCACAAACAGAAACATTCC

ATGATTATTCCAACATCACCAATGCTTCATCATCATCACACTCACAACAACTCTGTCAAA

CGCCGTTCATCACCATCATCATCTTCTGCTCACCAACCATCATCCAAGAAGCATTCTTTT

GACGCAAGCAATCTCGCCCGTAATGGCTTCTCCGCCATTACACTTCCTTTCAGTCTCCGT

GGGAATGCTCTTAGCCGCCGTGTCTCTGATCCATGCACCTTGCCGGATCAATCCATGCCG

GTAAAAGGGCCGTCAACTGCTTTGCAGCCTCTGCCTCCAATCAGTAGATGTATATCTGAA

GTTATTGATCCTTCTGAGGTTCAAGAAGCAGTTGCTCGTTATTTGAACTCTGCTGAAAAA

ACCACCCCTGAATCTGATTCAATTAGGCTTAAAAGGATGAAGGATCGGTTAAAAGAAATG

AAGAAAGTCTGGGATGAAGTTATGGAAGACGAGGAAGAAAATGAAAAAGAACAAGAACAA

GAGACCTCTCCTGATGCCGAAGAGGAAAAAGATGAAAAAGAACAAGAACAAGAAGAAGAT

CATTCTCTTGACTCTTTTGTTGCTGATGATGAAAAAGTCATATCTCAGGATGAATTGGGA

AATGATTATGAAGAAGCTGTTAGTGTAGAGTGGCTTGATAAGTGTTTGAGCCTTACTTTC

AAGTGTCCATGTGGCAAGGGATATGAGGTTCTTATATGTGCAAACAAGTGTTACTACAAG

TTGGTGTAG

>MS.gene009896.t1

ATGACTATCACCAAGGTCTACATTGTGTATTACTCATTGTATGGACATGTGAACACTATG

GCAAGAGAAGTACACAAGGGGGCAGCTTCAATTGAAGGTGTTGAAGCAACACTTTGGAGG

GTACCTGAAATTCTTTCGGATCGAATATTGGAAAAGATGAAGGCACCTCCTAAACCAGAT

GATGTAGCTGACATTAAGCCGGAACAACTTGTGGAGGCTGATGCTTTGATATTTGGTTTT

CCTTCACGTTTTGGTATGATGCCAAGCCAGCTCAAGGCCTTCTTTGACGCTACTGGAGAG

TTGTGGGCGTCTCAAGCACTCGCAGGCAAACCTGCTGGAATCTTCTGGAGTACTGGATTT

AACGGGGGTGGCCAGGAACTCTCAGCATGGACAGCTATAACTCAGTTAGCTCATCATGGT

ATGCTTTATGTTCCCCTTGGATACACCTTTGGAAGTGGCATGTTTGAGATGGATGAGGTA

AAAGGAGGCTCGGCTTATGGCGCTGGAACTTTCGCTGGAGATGGAACTCGTCAACCTACT

GAGCTAGAGCTTCAGCAGGCCTTTTACCAGGGTAAATACATTGCTGAAATTGCAAAAAAG

TTGAAAAGTTAA

>MS.gene009884.t1

ATGTTTGGTTTTTGTGTCTCAGATGATCTGAATGGAGTTCAGGTTTTAGGTAGGACTATT

AGGGTGGATCACGTTGAGAAGTATAAAATGATGGAAGATGAAGATGAAGAGGAAGCAAAG

CAGAAAAGGGAGGCCCGTGGTGTTTGTCGAGCTTTCCGAAGAGGGGAACCCGTGGAGCTG

GATGCAAATTTTCTCATGATGAACAATCATGTGTGCTAG

>MS.gene009850.t1

ATGTTAGGTAAACTTCCACATAGAACACTTCAATCACTCTCCAAAAAATATGGTCCAATC

ATGTCCTTACAACTTGGTCAAGTTCCAACTATTGTCATTTCATCTTCAAAATATGCAGAA

TCATTTCTCAAAACTCATGACATCAATTTTGCAAGCCGACCAAAGATTCAAGGAACTGAA

CTCATAACATATGGTTCCAAAGGGTTGACTTTTTCTAAGTATGGTCCTTATTGGCGTAGT

GCGAGGAAACTTTGCACTTTAAAACTTCTTAGTGCTTCCAAAGTTGCGATGTTTGGTCCT

ATTAGGAAGGAAAAGTTGGATGTTTTGGTTAACACTTTGAAGAAAGCTTCTTTGGTAGGT

GAGGTTGTGAATGTTAGTGAGGCAGTAGAGAATCTTATAGAAGATATTGTGTATAAGATG

ATATTGGGTCGGAGCAAGTATGATCAATTTGACTTAAAGAGGGTGGTTCAAGACGTAATG

GCTTTGGTTGGAGCTTTTAATCTGGCTGATTATGTTCCTTGGCTAGGTGTATTTGATCTT

CAGGGATTAACACGAGCATGCAAGGAAACGGGTAAAGCAATTGATGAGGTGCTGGAAGTG

ATAATAACAGAGCATGAACAAACCACTAATGTAGACAAAACTCGTCATGAAGACTTTGTT

GACATACTTCTTTCATTTATGCACCAAACCATAGATCTAGAAAATGAAGAAAATCATTTC

ATTGATCGAACTAACATCAAGGCAATATTACTAGACTTGATTGTGGCAGCAATTGATACA

TCTGCTACCGTGATTGAATGGGTTTTATCCGAACTTCTAAGACATCCACGGGTAATGAAA

ATCCTTCAAGATGAGATACAAAATGAAGTAGGAAATGAAAGAATGGTTGAAGAGAAAGAT

TTAGAGAAGCTAAGTTACTTAGATATGGTGGTTCATGAAACTTTAAGACTTTACCCTGTT

GCACCTTTATTAGTCCCTCGTGAATGTAGAGCGAGCATAACAATTGATGGATATTTTATC

AAGGAAAAGATACGTGTTATAGTAAATGCATGGGCTATAGGGAGAGATTCAAATGTTTGG

TCAGAAAATGCTGAAGAATTTTACCCAGAGAGATTTATCGACAAGAAAATGAATTATCAA

GGACAAGAGTTTGAATCTCTACCATTTGGTTCTGGCCGCAGACGTTGTCCTGGAATTCAA

TTGGGTTTAGTGACCGTTAAGTTGGTTATTGCCCAATTGGTGCATTGTTTTGATTGGGAA

CTTCCATATAATATTAGTCCTTCCAATTTGAATATGGAGGAGAAATTTGGACTCACTATA

CCAAGAGCTCAACACTTGCATGCAATACCGACTTATCGTTTGGCATGTGATGACAAGCTT

GAATAG

>MS.gene009846.t1

ATGTCCTTACAGCTAGGTCAAGTCCCAACTATTGTCATTTCATCTTCAAAAGCAGCCGAG

TCATTTCTCAAAACCCATGACATCGTTTTCGCTAGCCGGCCTAAGATTCAAGGATCTGAG

CTCATGTCCTATGGTTCCAAAGGGATGGCTTTTTGTGAGTATGGTCCTTATTGGCGTAGT

GTGAGGAAATTTTGCACTTTGAAACTTCTTAGTACTTCGAAAGTTGAGATGTCTGGTCCT

ATTAGAAAAGAGGAGTTGGATGTTTTGGTTAACACCTTGAAGAAAGCTTCTTTGGTAGGT

GAGGTTGTGAATGTTAGTGAGGTTGTAGAGAATCTTATAGAAGATATTGTGTATAAGATG

ATGTTTGGTCGGAGTAAGTATGAGCAATTTGACTTGAAGAGTTTGGTTCAAGAAGGAATG

GCTTTGATTGGAGCTTTTAATCTGGCTGATTATGTTCCTTGGTTAGGAATTTTTGATCTT

CAGGGATTAACACGATCTTGCAAGAAAGTCAGTAAAGCAATTGATGAGGTGCTAGAGGTG

ATATTAACAGAACATGAACAAGCTGCTAATGTAAACAAAACTCGTGAAGATTTTGTAGAC

ATACTTCTTTCAATTATGCACCAAACCATTGAAGTCGATGGTGAACAAAATCTCGTCATT

GATCGAACTAACATCAAGGCTATTTTGCTCGACATGATAGTGGCAGCAATTGATACATCT

GCTACCTCGATTGAATGGGCTTTATCTGAACTTTTAAGACATCCAAGAGTGATGAAAAAA

CTTCAAGATGAGATACAAAATGAAGTAGGAAATAAAAGAATGGTTGAAGAGAAGGATTTG

AAGAAGTTGAGTTATTTAGATATGGTGGTTGATGAGGCCTTAAGACTTTACCCTGTAGCA

CCGTTACTAGTCCCTCGTGAGTCTAGAGAGAGCACAACAATTGATGGTTATTTTATAAAG

GAAAAGACACGACTTATAGTAAATGTATGGGCAATAGGGAGAGATCCTAATGTTTGGTCA

GAAAATGCTGAAGAATTTTACCCAGAGAGATTCATTGAGAAAAAAATGAATTATCTCGGA

CAAGAGTTTGAATCTATACCATTTGGTTCTGGTCGTAGGCGTTGTCCTGGAATTCAACTG

GGTTTAATTACTGTTAAGTTGGTTATAGCTCAATTTATGCATTGCTTTAATTGGGAACTT

CCACATAATATTAGTCCTTCTGATTTGAATATGGAGGAGAAATTTGGACTCACTATACCA

AGAGCTCATCATTTGCACGCAATACTAAGTTATCGTTTGGATGATGCTCAACATGAATAG

>MS.gene009901.t1

ATGAGTCACAATGAAAAAACAAGCTTTTCATGGAGACAAAATTCTAATAGAAAATCGGCT

AGAAATCGACATGTTAGGAGCTGTACATTCAGGATTCCTTGGCAATCGGAATTCTCTCCC

ATTGAACTTGTCAAGGATCTTGCAGTAAGAGTGACAAGTGCTTTACGCTCGGTCTCTCGG

AGGAGATCATTAAACCGAGACCCTTCTTCCTTAGGAAGATCAGCATCAGCAGGGTCATCT

GCTGATTCTCATAGGACAGCTGCTGTTGAGGATTGCATTGAATTCATCCACTCTTCTTTT

TCTAGATCCAATTCCTCAACCAAAACATCCCACGAAGATTCTATAGAAGCTTCTTGA

>MS.gene009920.t1

ATGAAGTGGGAAATGGAAGTTCTTCCTCCTGCATCATACACACAAAACTCCAATTGGTGT

ATGGAAGATAGCATGACCACACAATGGACTCCAGAAGAGAACAAAATATTTGAAAATGCT

CTTGCAGTTCATGATAAAGATACACCTGATCGATGGCATAAAGTAGCTGAAATGATACCA

GGAAAGACGGTCGGTGATGTGATGAGACAATATAAGGAATTAGAAGATGATGTTTGTAAT

ATAGAAGCTGGGTTGATTCCAGTTCCTGGTTATAACACTCCTACTTCACCTTTTACCTTA

GATTGGGTGAACAGTTCTGGTTATGATGAATTCAGAGGAAGTGGAAAGAGATCTTCTTTA

GTTAGAGCTCCTGAGCAGGAAAGGAAGAAAGGAGTGCCATGGACTGAAGAGGAACACAAA

TTATTTCTACTAGGCCTAAAGAAGTATGGCAAAGGTGATTGGAGAAATATTTCGCGCAAT

TTCGTCATCACAAGAACACCAACTCAAGTAGCAAGCCATGCTCAAAAGTATTTCATAAGA

CAACTTTCAGGAGGAAAAGACAAGAGGAGAGCAAGCATACATGACATAACAACAGTTAAT

CTTTCAGAAAAAATTGGAACTTGTTCTTCAGAAGACACCGGTAATAGATCCACTTCACCA

CAGAATTCTATGTTACTTTCACACCAGCAGCAGCACCAACAAACTTCGACGGCAACAAAT

TTTCGTTGGAGGAATGATCAACAAAATGCTATGGCTTTGAATCCAGCTCACGAGCAAGTT

TTCATGGATCCTCATGGTTTTAACTCCTATGAGGTTAAAATGCAAGACCAAAATCTTCAA

AAAGGTCTTGTTCATGAGTCTTCTTATCCACATAACATGGTTTTCCAAATGCAACGTTCA

TCACAACATTATTCACATGCATAA

>MS.gene009907.t1

ATGAAAACCAATCAATTTAACTTGTGCTTTTATGCACTTCTTCTTATCTTTGTTGTAAAA

ACCGAGTCTTATGAAGTTGTGAAAAAAATTGAGGAAACAAAAAAATCAAAACCGGACATT

GGAGTAGATTGCTATACATATTGGTCAAACAAAGGTGCAACTTGGAAAGCCAACGACGGG

GAGTTGGGGAATGGTTGGTTTGGTTGTGGATTAGGAGGCATAAAAGGAATGGGATACAAC

AGTTATTTTTGGTGGAAAGGCCAAGCCTGGGGTAGTGAATGGTGGAATGGTGGTGGAAAA

GGTGGCGGTGGTGGAAAGGTTGGCGGTGGTAGAACATGGGGTGGTGCTGGATATGTCAAA

CCTATTTCTGGCAACGGAAAAGGTGGTGGTGGTGCAAACGACATAGAACTCCCTGATGAT

GGAAAGGGGACTGATATTGAGAAGGGGCGGTCGTGA

>MS.gene009922.t1

ATGGGGATGATGAGTATTAGAATCATATTGCTACTAGCTGCTTATTGCTTGCTTCCTCTA

TCTGTGGATGCCATGGTTCGCCACTACAAGTTCAACGTGGTGATGAAGAACGCCACAAGA

TTGTGTTCAACCAAGCCTATTGTAACCGTAAATGGAAATTTCCCTGGCCCCACCATCTAT

GCTAGAGAAGATGACACTGTTCTTGTTAAGGTGGTTAACCATGTCAAATACAATGTTAGC

ATCCATTGGCATGGAATAAGACAATTGAGAACAGGTTGGGCTGATGGGCCAGCATACATA

ACCCAATGCCCAATTCAACCAGGCCAAGTCTATAAGTACAATTTTACCCTCACAGGACAA

AGAGGAACACTTTGGTGGCATGCACATATACTTTGGCTTAGAGCCACTGTACATGGTGCT

ATTGTCATTTTGCCTAAGCTTGGAGTTCCTTACCCTTTTCCTAGACCTCACATGGAACAA

GTTATCATATTGAGTGAATGGTGGAAATCAGATACTGAGGCTATAATAAATGAAGCTTTG

AAATCTGGGTTGGCCCCAAATGTCTCTGATGCTCACACTATCAATGGTCATCCAGGGCCT

GTTCAACATTGTGCTTCACAAGAAGGATTCAAATTCCAAGCTGAACCAGGAAAAACCTAC

TTGCTAAGAATTGTCAATGCAGCACTAAATGAAGACCTATTCTTCAAAATAGCTGGTCAC

AAACTAACTGTTGTTGAAGTTGATGCTGCATACACAAAACCATTCAAAACAGACACAATA

GTAATAACACCAGGACAAACAACAAATGTACTTCTCAAAACCAACCGTGCAAGTGGCAAA

TACATGGTAGCAGCCTCAACATTCATGGATGCACCAATTGCTATAGACAATGTAACAGCC

ACTGCCACATTAAATTACCCAAATACCCTTGGTTCAACAATCACAACACTCACTTCACTA

CCTCCAATAAATTCAACAAAAATAGCTAACAGTTTCACTGACTCGTTAAAAAGTTTGAAT

TCCCATAAATACCCTGCTAGAGTCCCTAAAAAAATTGACCATTCATTGTTTTTTACTGTG

AGCCTTGGTGTTAACCCTTGTGCTACATGTGTGAATGGTAGTAGAGTTGTTGCAGCAATA

AATAATGTAACATTTGTCATGCCCAAAATTTCTCTTCTACAAGCACATTTTTTTAACATT

AGTGGAATTTTTACTGATGATTTTCCTAGAAAACCTGAAATAGTTTATGATTTTACTGGG

AATAAACAGCCAACAAATTTTAGGACTAATAAAGGGACTAGGGTTTATAGACTTGCTTAT

AATTCAACGGTTCAATTAGTTTTGCAAGATACTGGAATGATAGCTCCTGAGAATCATCCT

ATTCATCTACATGGATTCAATTTCTTTGTTGTTGGTAAGGGACAAGGGAATTTTAATTCT

AAAAAGGATACTAAAAAGTTTAATTTGGTTGATCCTGTGGAGAGAAATACAGTTGGTGTT

CCATCTGGTGGATGGACTGCTATCAGATTCAGGGCTGATAATCCAGGGGTATGGTTTATG

CATTGCCATTTGGAAATTCACACAACTTGGGGATTGAAGATGGCATTTGTTGTGGACAAT

GGTAAAGGACCAAATGAATCTTTACTACCACCTCCAAGTGACCTTCCCAAGTGTTGA

>MS.gene009899.t1

ATGGCTTCTATCACCATGTTGCCAATGGTTCCAACAACAGGAAGAGTCTTTGCAGCTACA

GGTGCAAAGGGTACTACAGGTGGTGGCAGTAGCAAGCAAGAGAAAGGTTTTTGGGATTGG

ATTGTTGGTGGTTTAACAAAAGAAGATCAGTTCTATGAAACTGATCCTATTCTCAAGAAG

GTTGAAGAGAAGAATAATAGTAGAGGCACTACTAGTAGAGGTACTACTAGTGGTAGAGGT

ACTACTAGTGGTAAAGGCACTACTGGTGGTGGCAAGAACTCTGTGGTAGTTCCACAAAAG

AAGAAGGGTGGTTTTGGAGGATTTTTTAACAAGGATTAA

>MS.gene009942.t1

ATGGTTCGAAAATCATCAAAGACACGCTCCAAAATTGCTGATGCTGGTGTTATTCAACCA

CTCATCTTCATGCTTTCTTCTTCCAACATTGATGCTCGTCAATCCTCTCTTCTTGCTCTT

CTCAACCTCGCTGTTCGCAATGAACGGTACAAAGATCTCTTTTTTTTTCTTGTTTCTCAT

CTGGGTTTTGCATTTGCCTTCATGGGTATTCGTTGGATTATGTTTTCTTTGTGTAATGGG

AAATGGGTTGCTTTAAATTTATGA

>MS.gene009862.t1

ATGAAAAACTTGTTACTAAGGTCAACGGCACGAGCTTTGTTCTGCAGTAGCCAGAGCTAT

CACCGCGGTTTATCAACTGCGGTGACTGTTCAGCCGAGACACCAGAATGGCGGTGGCGCT

CTTGGAAGCTTCTACTGGCAGAAGATGTCAACTCTGCCAGAGAAAAAGGATCAGCGGTCA

GAGGAGAATAAGAACAGTAACGACAGCAACACCGTCGTTTCGAGTTACTGGGGGATCACT

AGGCCCAAGGTTAAAAGGGAGGATGGAACTGAGTGGCCATGGAACTGTTTCATGCCATGG

GAAAGTTACAGCTCAGATGTGTCAATAGATGTGACCAAGCATCATGTACCAAAAACATTT

GCAGATAAGTTTGCTTTCAGATCTGTCAAGTTTCTAAGGGTTTTCTCTGATTTGTATTTC

AAGGAACGGTACGGATGTCATGCAATGATGCTGGAAACAATTGCAGCCGTCCCGCCAATA

GTCGGAGGGATGCTTTTACACTTGAAGTCTCTAAGAAAATTTCAACACACTGGTGGTTGG

ATCAAAGCATTACTTGAAGAAGCAGAGAATGAGAGGATGCATTTGATGACAATGGTAGAG

CTTGTGAAACCAAGTTGGCATGAAAGGCTTTTGGTTATTACTGCACAAGGAGTTTTCTTC

AATGCATTCTTTGTTTTTTACATCCTTTCACCAAAAACAGCACATAGGTTTGTTGGTTAT

TTGGAGGAAGAGGCTGTGATTTCATACACACAACATTTAAATGCAATTGAAAGTGGTAAA

GTTGAAAATGTACCTGCTCCTGCTATTGCTATTGATTATTGGAGACTTCCTAAGGATGCA

ACTCTTAAGGATGTTATCACTGTTATTCGTGCTGATGAGGCTCATCATAGGGATGTCAAT

CACTTTGCGTCGGATATTCACCATCAAGGAAAAGAACTGAAAGAGGCTCCAGCTCCTGTT

GGTTATCATTGA

>MS.gene009860.t1

ATGTCGGAATCAATTTGCAGATCCTTACGCGACGGCGCGTTAGAAGGAGAACTATCTCCC

ACTCTCACTATCAAAGACACTCTCTCTTCACCTTTCGCCTTCAACGTTTTCTCTCACATT

CTTCTTCAACTCTCCTCACCAAAATCTCACTCCAACACTGCTATTCTCATCGTTTCGCTT

TCTCGGAGTCCCTCGTTCTATGCTCATTTCTTCAAAAACAAGGGAATCGATCTTTCTTCC

TCTAACAAATGGATTCATGTTTTGGATTGTTATACAGATCCTCTTGGTTGGAAGGGTAAG

ACAAGGAAATCTGGAAATGTTACGATTCCTTCTGACCAAATTTCACTTGCTACTACAACT

TATAAATCAGTCAAGGATATGGATAAGTTGTTTTTGGCGATTACTGAATTGGGAAGAGGT

TTAGTTGGAGAAAATAAAGTCCGCGTTTGTGTTGCCATAGACTCGCTAAGTGAACTGTTG

AGACATGCATCTTTGCAGTCAGTCGCAGGCCTTCTAAGCAATCTGCGTAGCCATGATCAA

ATTTCAAGTATATTTGGATTATTGCATTCTGACCTTCATGAGGAGAGGGCTGCAGCTGCT

CTTGAGTACATGTCCTCCATGGTGGCTAGTGTAGAATCAGATCATCATTCTTCCGAGAAC

TCCTTATTGGAGAAAAACTTTACCCAAGGAAAATTTAATGTCAGACTCAAACGCAGAAAT

GGACGAGTTAGAGTGACGTGTGAAAAGTTTAAAGTTGAAGCAGGAGGAATCAGCTTTACT

TCTGCTTCAGCAGAAGATGGAACAACCGTTGCAGGCTTACTGCCGAAGGTACAATTCAAT

CTCCAGCTGTCGGAGAAGGAGCAAGTTGATAGGGCAAAGGTTGTGCTTCCTTTTGAACAC

CAAGGAAATGGTGCACCGATTCAAATTTATGATGGTAGAAGATCCCTTGAAGAGAGCAGC

AGTGAGGGGGCACCTATTTCAAAGGGTAAAACGGAGGACTCAGCCCTAGGTGAGATTATA

TATTTTCGTGATTCAGATGATGAGATGCCAGATTCTGATGAAGACCCAGATGATGATTTA

GATATATGA

>MS.gene009894.t1

ATGTCTAGATTGGCCTCAACAGGACTCTCTGAATCGCGTCCAACTGCCTATGGTTCTAAA

GGAGTTGATACATGGAATTTGAAGAGCTGGCAGAAGGAAATTAGTGACAATGATGTGTTG

GTCTTGGATGTGGCTGTCAATGGAGGGACCAAGGTTTCTGACAAAGAATTTTTTGTGACT

ACAGAGTTACTTATGAGGAAATTGCTAAAGGTGGATGGTATTAAGGCTGAAGGTGATGCT

AGGTTGCAGAGAAAAGCTGAGATTGTGCATTCGATTGCTCTATTTCGGGTTGACTTCGCA

GGAAGAGGAGAGCTTACTGAGCGCCAGATAATACCTACAACATCAACCAACAACACGAGA

TTGAGCCAAGGGAGACAATTGATCTAA

>MS.gene009875.t1

ATGGCATCAAAACTCTCACTCATTCTCTTTGTTCTAACAATCTGGACCCTTGATTCATCT

CAAGGAGCTTCAACTCATCAAGCTCCAGCACCATCAGTGGACTGCACAAACCTTGTCTTA

ACAATGGCTGATTGTTTGTCCTTTGTAACAAACGGTAGCACAACAACAAAACCAGAAGGA

ACTTGTTGTTCTGGTTTGAAATCTGTGCTCAAAACTGCTCCTTCTTGTCTCTGTGAGGCT

TTCAAGAGTAGTGCTCAGTTTGGTGTTGTCTTGAATGTTACCAAAGCTACTTCTCTTCCT

GCTGCATGCAAAGTTTCTGCTCCTTCTGCTACCAAATGTGGATTGACTGAAGCGCCTGCT

TCTGCACCTGCTGGAGGTCTCTCTCCACAATCACCAGCAGCTTCTCCAACATCTTCTGGT

GCAGCTTCTCCAACATCTTCTGGTGCAGCTTCAGGTTTGAATGGTCCTGTAAGTGAACTA

TCTCCAGTTCCAGCACCATCTCCAGGGAACACAGCTTCAAGACTATTCCCAATTTCAATG

GGATCCTTACTTGTTGGCCTATTGGTGGCTACAATGTCACTATTTTAG

>MS.gene009878.t1

ATGAGCTTCTTCTCAGAGTCTACATCACGACCATTTCGAACCATGAATGAAGATTTGGAG

AACAATAATGTAGAGACACCACATAACATTTCATCAAATTCTATTGTGAGAAAGAAATCA

GATATGGTTTTGGTGACAAATAATGTTCGGTTCCAAATACTAAGAAATGTGATGACAAAT

ATGAAAGAGGTGATGTTGGGTACTAAGCTTGTTGTGCTTTTTCCGGCTGTTCCTTTAGCT

GTTGCTGCTGATTTTTATAGCCTTGGAAGGCCTTGGATTTTTGCTTTGAGCCTACTTGGA

CTTGCTCCCCTAGCTGAACGTGTCAGCTTCCTCACTGAGCAAATTGCATATTTCACCGGC

CCAACAGTTGGAGGGCTTCTAAATGCAACTTGTGGTAATGCAACCGAGATGATCATAGCA

ATATTAGCACTTCATCAGAACAAAATTCATGTTGTCAAGTTCTCCTTGCTTGGTTCCATT

CTCTCAAACCTTCTCTTGGTTCTAGGCAGCTCACTCCTTTGTGGTGGCTTAGCAAACCTT

AAGAGGGAACAAAGATATGACAGAAAACAGGCAGATGTAAATTCATTGCTTTTGTTGCTT

GGATTGCTATGCCATTTGCTGCCATTGATGTTCAAGTATGCCTTGGCTGGTGGCAATCAT

TCTATAGCCAATTCTACTCTTCAATTGTCAAGAGCTAGCAGCGTTGTTATGCTTCTAGCA

TATGTTGCATACATCTTCTTCCAATTGAAAACTCATCGAAAAATATTTGATGCACAAGAG

GTGGATGATGAAGATGATGAAAAAGCAGTGATAGGATTTTGGAGTGCATTTAGTTGGTTG

GTGGGTATGACATTGGTCATATCTGTGCTTTCTGAGTATGTTGTGGGAACCATTGAGGCT

GCTTCAGATTCTTGGGGTATTTCTGTTAGCTTCATTAGCATAATTTTGCTACCAATTGTT

GGAAATGCTGCAGAACATGCTGGTTCAATCATATTTGCTTACAAGAACAAACTGGACATA

TCTTTAGGTGTTGCAATGGGATCTGCTACTCAAATTTCTATGTTTGTGGTTCCATTAAGT

GTGATTGTTGCATGGATAATGGGTATAAGAATGGATTTGGACTTCAATCTTCTTGAAACT

GGATGTCTTGGTTTTGCAATCATTGTTACAGCCTTTACTTTACAGGATGGAACTTCACAC

TATTTGAAAGGACTGATTCTTACTCTATGTTACATTGTCATTTCTGCATGTTTTTTTGTT

CTCAAAACTCCACAAATCAATCATTATGGTATTGCCATACTTTGA

>MS.gene009932.t1

ATGGCTGGAGCTATGCAGCCTATGAATTTTTCTGATTTTAAGTGTGAAATTCCCGAACTA

AGGGGTGAAAACTATAAGGTGTGGAAGGAGAGAATTCTTCTTCACTTAGGGTGGATGGAT

ATCGATTATGCTATTCGGAAAGACGAACCACCTGCAGTTACTGAAACTAGCCTTCAGGAA

GACGTTGATCTTTATGAAATGTGGGAGCGGTCTAACCATCTCAGTGTGATGTTCATAAAG

ACCAACATCTCTGCTAGTATCCATGGTTGTGTCGATCAGCATGATAATGTCCAAGCATTG

CTTAAGGCTATTGATGAACAATTCAAAACATCAGATAAGGCACTTTGCAACACTCTTCCA

CCTGAATATGGACCTTTCAAGATTTCTTATAACACACATAAGGACAAATGGTCAATTAAT

GAACTCCTGACCATGTGTGTTCAAGAAGAAGGAAGGTTGACTATGGAAAAGAGCGAGAGC

GTTTTTGTTGCAACTGAAAGAAAGAACATGAATCAAGCGAAACATAAGGGGAAAGGCAAA

ATACCACCTCAAGGTAGAATTAAGAAAGAATCCAAGTGTTTCTTTTGTAAAAAGAAGGGA

CACATGAAGAAGGATTGCCCCAAGTTTCAGAAATGGCTTGAAAACAAAGGGTATGACAAG

CCTAAGGAAACCACTTCCAAACTTCCAAAGCCCTTGTGGACTGAAGCTCTTAAAACGGCA

GTGTATATATTGAACCGTGTTCCAACCAAGGCTGTCCCAAAGACACCGTTTGAGTTATTT

AAAGGTTGGAAACCGAGTTTGCGTCATATACGCGTTTGGGGATGCCCGTCTGAAGTAAGA

GTTTACAATCCACAAGAAAAGAAACTAGACCCTAGAACTATAAGTGGGTATTTCATTGGA

TATGCTGAAAATTCTAAAGGGTATAGGTTCTATTGTCCATCTCATAGCACTAGGATTGTG

GAATCAAGAAATGCAAAATTTCTTCAAAATGACTTGATTAATGGGAGCAATCAATTTCAA

GACACTGGTTCTGATAAAAGTCAAATTGATGCTGAACCGTCCACTTCAAGTGACAGATTG

ATTGTCATTCAGAGTGCCCCTCAAGATCAAATGGGTATTAGACAATCAGTTGTTGAAGTT

CCACAAGCGGCCGATAACAATCTAGTAAATAAAGATGTTCAAGAATTTCCAGAAATTTTT

GAACAAATGGTTGAGCAACATGATCCTCCGGAAAATGTTGAACCAACATTAAGGAAATCT

ACTAGAGAGAGAAGGTCAGCTATACCTAGTAACTATGTTGTATATTTACAAGAATCGGAC

TATAATATTGGTGCTGAAAATGATCCTGAAACGTTTTCAAAAGCCATGAGTTCTAGAGAG

TCGAATTTGTGTGAGATTGTGGAAGGTTTAGAAGAGCATCAACTTCTTCAAACAAGAGCA

ATGGCTGGAGAAGCGGAGTATGCTGAAATGTTGACTGACTTATGTTGCAAGGGAGCGCAA

TGGCATCAAAAACCTGACGGATATCCTATCAGATTGACAACTGAGAATATAAAGCCGATT

CCGGCTTCATATTCACAGTTGTTTGTTACAATGTCGGTTAAATATTTGGTTTTTGAGAGC

AGTTTACTTTGGATGGAGATCGAATAA

>MS.gene009867.t1

ATGCAGCCTCGCCGGCGAAAGTGTGTCCGGACATCATCACCGGAGAAGGAATCAAAACAT

TTTATGAAAGCCATACTTCCCTCACCCATTCATTCCAAAGAAATAAGAATTCCAGATGAA

TTTATATCAAGATTTGGGAATGAACTCAAGAATGTTGCTACAATTACTGTTCCAGATGGT

CGTGTCTGGAAAATGGAACTGAAGAAACATGATGAGAACGTTTTCTTTTGCAACAAATGG

CAAGAGTTTGTAGAATACTATTCTATAGGATATGGTTGCTTCCTTTCTTTCAAATATGAA

GGGAATTCAAAGTTTAGTGCTATTATATTTGATGCAACTTCTGTTGAAATTTGCTATCCT

TTCAAAACTTCAAGTACCAACGGAGAACCAAAAACAAATTATCCTAGTTCTAGAAAAAGG

TACAAGGTTGAAACATGTGAGAGGCGTGGGAAAAAGGTCAAAAACATGTCTAAATATGCT

TCTAAAAGAGTAGAGGATGCTGCCAAGGAACTCAACCCAAACAACCCTTATTTTCGTTGC

AAAATCGTCAAGGGAAAATATGCGTATGTTAATTATGATTTTGCTACAAAGTACCTGAAG

CCAAATGTTCCTATAAAGCTCCAAAATTCTCATGGAGGGCAGTGGGAAGTGTTTGGCATT

ATTCATGATGTCAGATCATCAGCAATGCAAATAGTGAGGGGATTTTCTATATTTCAAAGG

GACAACAAACTGTCACATGGAGATTATTGTGTGTTTGAGCTGATCAAGAGGAGACCGATT

GTGCTAAAAGTCACAATGTTTCGTGCGGTTGACTATTGCGATTAA

>MS.gene009849.t1

ATGACACTAATGGCATCTCATCATTCTGATGATGAAGAAAATGAGGTTAGCAAACTTCCA

CATAGAACACTTCAATCACTCTCCAAAAAATATGGTCCAATTATGTCCTTACAACTTGGT

CAAGTTCCAACAATTGTTATTTCATCTTCAAAGGCTGCAGAATCATTCCTCAAAACTCAT

GACATCAATTTTGCAAGTCGGCCGAAGACTCAAGCATCTCAGCTCATTTTTTATGGTTCC

AAAGGGTTGGCTTTTTCAGAATATGGTCCTTATTGGCGTAGTGTAAAGAAACTGTGCACT

TTGAAACTTCTAAGTGCTTCAAAAGTTGAGATGTTTGGTCCTATTAGGAAGAAGGAGTTG

GATGTTTTGGTTAAGTCATTGGAGAAAGTTGCTTTGGTAGGTGAGATAGTGAATGTTAGT

GAGATTGTAGAGAATCTTATAGAGGATATTGTGTATAAGATGATATTGGGTAGGGGTAAG

TATGAGCAATTTGATTTGAAGAAGTTGGTTCAAGAAGGAATGGCTATGCTTGGAGCTTTT

AATCCGGCTGATTATGTTTCTTGGCTAGGCCCTTTTGATCTTCAGGGGTTTACACGAACC

TGCAAGAAAACCGGTAAAGCACTTGATGAGGTATTGGAGATGATAATAAGAGAGCATGAA

CAAAGCACTAATGTAAACAAAGTTCGTCATGAAGACTTCGTAGACATACTTCTCTCAATT

GTGCACCAAACCATAGATCCAGAAAATGAACAAAGTGATGTCATTGACAGAACTAACATC

AAAGCAATTTTACTAGACATGATTGTGGCAGGAATTGATACAGCCACTACCACGATTGAG

TGGGCTTTATCTGAACTTTTCAGACATCCAAGAGTGATGAAAAAGCTTCAAAATGAGATA

CAAAATGAAGTAGGAAATAAAAGAATGGTTGCAGAAAAAGATTTGAAGAAGTTTAATTAC

CTAGATATGGTGGTTGACGAGACATTAAGACTTTACCCTGTTGGACCATTATTACTCCCT

CGTGAATGTAGAGAGAGAATAACAATTGATGGATATTTCATAAAGGAAAAGACTCGAGTT

ATAGTAAATGCATGGGCTATAGGGAGAGATTCTAATGTTTGGTCAGAAAACGCTGATGAA

TTTTATCCAGAGAGATTTAGTGATAAGAAAATGAATTATCAAGGCCAGGAGTTTGAATCT

ATACCATTTGGTTCAGGTCGTAGACGATGTCCTGGGATTCAATTGGGTTTGGTTACTGTT

AGATTTGTTGTAGCTCAATTGGTGCATTGCTTTAATTGGCAACTTCCACATAATATTAGC

CCTTCCAATTTGAATATGGAGGAGAAATTTGGAGTCACTATACCAAGGGCTCAACACTTG

CACGCAATACCTAGTTATCGTTTGGAATGTGATGAGAAGCTTGAATAG

>MS.gene009859.t1

ATGTCCTTACAACTTGGTCAAGTTCCAGCTATTGTCATTTCATCTTCAAAAACCGCAGAG

TCATTCATCAAAACCCATGACATTGTTTTCGCAAATCGACCAGAACTTATAGGAGCACAG

ATCATGTCTTATGGTTGCAAAGGGTTGGCGTTTTCTAAGTATGACCCTTATTGGCGTAGT

GTGAGGAAACTTTGCACTTCAAAACTTCTTAGTGCTTTGAAAGTTGAGATGTTTGGTCCT

ATTAGGAAGGAAAAGTTGGATGTTTTGATTAAATCATTGGAGAAAGCTGCTTTGGTTGGT

GAGGTTGTGAATGTCAGTGAGGTTGTAGAGAATGTTATAGAGGATATTGTGTATAAGATG

GTGTTGGGTCGGAAGCATGAACAAACCGCTGACACAGACAAAACTTGTCATGAAGACTTT

GTAGACATACTTCTCACTTTTATGCACCAAAACATTGATCATGGGAGTGAACAAAATCAT

GTCATTGATCGAACTAACATCAAAGCTATTTTACCAGACTTGGTTGTGGCATCATTTAGT

ACATCTGCTACCACAATTGAATGGGCTTTATCCGAACTTTTAAGGCATCCAAAAGTGATG

AAAAATCTTCAAGATGAAATACAAAATGAAGTAGGAAATAAGAGAACGGTTGAAGAGAAG

GATTTGAAGAAGTTGAATTACTTAGATATGGTGGTTGATGAACCTTTAAGACTTCATCCA

GTTTCACCCTTACTACTCGCTCGAGAGTGTAGAGAGAACATAACAATTGATGGTTATATT

ATAAACGAAAAAACACGAGTTATAGTAAATGCATTCACAATAGCGAGAGATCCTAGTGTT

TGGTCTGAAAGTGCTGAAGAATTTTATCCAGAGAGATTTATTAACAAGAAAATGAATTAT

GAAGGACAAGAGTTTGAAAGTATACCATTTGGGTTTGGTCGTAGGCGTTGTCCTGGGATT

CAATTGGCTTTGAGGACTGTTACGTTAGTTATAGCTCAATTGGTGCATTGTTTTAATTGG

AAACTTCCATATAATATTAGTCCTTCCAATTTGAACATGGATGAGAAATTTGGACAATCT

ATACATAGAGCTCAACACTTACACGCAATACCAAGTTATCGTTTGGCAGGTGATGGCAAG

CTTGAATAG

>MS.gene009851.t1

ATGTCCTTACAACTTGGTCAAGTTCCAACAATTGTTATTTCATCTTCAAAAGAAGCAGAA

TCATTCCTCAAAACCCATGACATCGTTTTCGCAAGCCGACCGAAGACTCAAGGATCCGAG

CTCATGTCCTATGGTTCTAAAGGGATGCCTTTTTCTGAGTATGGTCCTTATTGGCGCAGC

ATGAGGAAATTTTGCACTTTGAAACTTCTTAGTGCTTCAAAAGTTGAGAAGTCGGGTCCT

ATTAGAAAAGAGGAGTTGGGTGTTTTGGTTAACAGTTTGAAGAAAGCTTCTTTGGTAGGT

GAGGTTGTGAATGTTAGTGAGGCTATAGAGAATCTTATAGAGAATATTGTGTATAAGATG

ATATTGGGCAGGGGTAAGTATGAGCAATTTGACTTGAAGAAGATGATTAAAGATGGACTG

ACTTTGATGGGAGCTTTTAATCTGGCTGATTATGTTCCTTGGCTAGGTGTTTTTGACCTT

CAGGGATTAACACAAGCTTGCAAAAAAACCAGTAAAGCTCTTGATGAAGTTTTGGAGATG

ATAATAACAGAGCACGAACAAACTACTAATACAGACAATCCTAAAGATTTTGTAGACATG

CTTCTTTCAATTATGCACCAAACCATTGGTGTTGAGGGTGAACAAGATGTCGTCATTGAT

CGAACGATCATCAAGGCTATTTTGCTCGACATGATAGGGGCATCAATTGATACATCTGCA

AACGTGATTGAGTGGGCTTTATCTGAACTTTTAAGGCATCCAAGAGTGATGCAAATTCTT

CAAGTGGAAATACAAAATGCAGTAGGAAATAAGAGAATGGTTCAAGAAAACGATTTGAAG

AATTTTAATTACCTAGATATGGTGGTTGATGAAACCTTAAGACTTTATCCTGTTGCACCT

TTATTAATCCCTCGTGAATGTAGAGAGAGCATAACAATTGATGGTTGTTTTATAAAAGAA

AAGACACGAGTTATAGTAAATGCATGGGCAATTGGGAGAGATCCTAATGTTTGGTCAGAA

AATGCTGAAGAATTTTACCCAGAGAGATTTATTGAGAAAAAAATGAATTATCTCGGACAA

GAGTTTGAATCTATACCATTTGGTTTTGGTCGTAGACGTTGTCCTGGAATTCAATTGGGT

TTAATTACGGTAAAGTTGGTTATAGCTCAATTGGTGCACTGTTTTGATTGGGAACTTCCA

CATAATATTAGTCCTTCCAATATGAATATGGAGGAGAAATTTGGACTCACTATACCAAGA

GCTCAGCATTTGCACGCAATACCGAGTTATCGTTTGGATGATGCTAAGCAAGAATAG

>MS.gene009895.t1

ATGGTTGTGAGTGAGATGAAGAAGAAAGCAGAACTAATCTTCATTCCTTCACCAGATATT

GGTCACTTAGTTTCATCACTTGAATTTGCAAAACTCTTAATGAATACTCACAACAATATT

TCCATCACAGTCCTTTGCATCAAATTTCCACATACAGCATTTTCTGATTCATACATCAAA

TCAGTTTTAAACTCACAACCACAAATCAAACTCATTGATCTTCCTCAAGTAGAATCACCA

CCAAAAGAACTATTGATATCACCACCTTGCTACATCAAAGCACTCATGCACACTCTCACA

CCTCATGTAAAATCCACCATTCAAACCATTTTATCATCTCATTCAAACCAAGTTGTTGGT

TTAGTCCTAGATTTATTTTGTCTTTCAATGATTGATGTTGGAAATGAACTTGGTATCCCT

TCTTATTTGTTCCTAACATCAAATGTTGGATTTTTAGGATTCATGCTTTCCCTTCAAAAC

CGTCGAGTCAACGACGTGTTCAATGATTCTGATCCTGAGTTATTGATTCCTGGTTTCGCA

AATTTAGTACCTTCTAGTGTTTTACCAAATGCTGCTTATAGTAGAGATGGTGGTTATGAG

GCCTATTACAACCTTGCTCGGAGAATCAACGACACCAAAGGAATTATTGTTAATACCTTT

TCAGGTTTGGAACAATATTCTATTGATGCATTATATGATCATGATGAGAAAATTCCTCCT

ATCTATGCTATTGGTCCTTTGTTAGATCTCAAAGGTCAACCTAACTCTAAGTTAGATCAA

TCTCAATATGATCTTATATTGAAATGGCTAGATAAGCAGCCAAATAAATCAGTTGTTTTT

CTATGTTTTGGAAGCATGGGAGTTAGCTTTGGTCCATCTCAAATAAGAGAAATAGCATTA

GGACTTAAGCATAGTGGAGTTAAATTTTTGTGGGCTATGAAATCTCCACCAAGAATCAAC

AATTACGAAGAGAAAAGATTACCAGAAGGGTTTTTAGAATGGATGGAATTGGAAGGTAAG

GGAATGATATGTGAATGGGCCCCACAAGTTGAGGTTTTGGCACATAAGGCTATTGGTGGA

TTTGTTTCACATTGTGGATGGAATTCTATTTTGGAAAGTATGTGGTTTGGTGTGCCAATA

TTGACATGGCCTATCTATGCAGAACAACAGCTTAATGCTTTTAGGTTGGTGAAGGAATTG

GGATTAGCAGTGGAGTTGAGAGTGAATTATAGAATAGGTAGTAAAGAACTTGTTATGGCT

GAGGAGATTGAGAAAGGGTTGAAGCATTTGATGGAGAAAGATAATATTTTGCTTAAGAAA

ATTCAAGAGATGGCTAGGAACGCTGTTCTTTGTGGTGGGTCTTCTTTCATTTCTGTTGGG

AAACTTATTGATATTATGATAGAAAGTAATTAA

>MS.gene009881.t1

ATGGCACCGGTTACTCGTAGAACCTCTTTCCCCAAAGTTCTCATTGAAAGAGACTCTGAC

TCTGAACAGAGTTCTTCTGATGAAGAAGAAGAAGAAGCACTCGAGGAGGAAGAAGAAGAA

GAAGAAAATGGGGTTGTTGAAAATGAAAAGATTGAGAAACTGGAAGTGGGTTTTGATGCT

AATAGGAAAGGGAAAAAACCCATTACGCTAACTCTCAGAAAAGTTTGCAAGGTGTGTAAG

AAGCCTGGTCATGAAGCGGGTTTCAAGGGTGCTACTTACATTGATTGCCCTATGAAACCT

TGTTTTCTTTGTAAAACTCCTGGACATACCACTTTGAATTGTCCACATCGGGTCACTACT

GAGCATGGTGTTGTTCCGGCACCCCGCAGAAAAACCTCTAAACCTTTGGAGTATGTGTTT

GAACGCCAGCTAAGACATGCCATTCCTACGATCAAGCCCAAATGTGTGATTCCAGACCAA

GTGAGTTGCGCTGTTATCAGATATCATAGTAGACGGATAACAAGCTTGGAGTTCCACCCT

ACGAAGAATAACATCCTGTTGTCGGGAGATAAGAAAGGACAACTTGGAGTATGGGATTTT

GAGAAGGTTTACGAGAAAGTGGTCTATGAGAACATACACTCTTGTATACTGAACAACATG

AAGTTTAATCCTACAAATGATTGTATGGTCTATTCCGCATCCTCAGATGGAACCATTAGT

TGTACTGATTTGGAGACTGGAATATCATCCTCTCCGATGAACCTGAATCCTGATGGATGG

CATGGGCCAAACACCTGGAAAATGCTTTATGGTATGGATATTCACTATGAGAAAGGTCTT

GTGCTCGTGGCCGATAACTTTGGTTTTCTTCACTTGGTCGACATGCGATCCAATCATAGG

AATGGTGATGCAGTTTTGATCCATAAAAAAGGTAGCAAAGTTACTGGTATCCATTGCAAT

CCAATGCAACCAGACATCCTTTTGACTTGTGGAAATGATCATTATGCGCGCATTTGGGAT

ATGCGTCACTTAGAAGCTGGATCATCCCTCTGTAGCCTTGAGCACAAGCGTGTTGTTAAC

TCTGCATATTTCTCTCCAATAACTGGAAACAAAATTCTTACTACGTCACAGGACAATCGT

CTTCGTATATGGGATTCTATCTTTGGTAATATGGCATCTCCCAGCCGAGAAATTGTGCAT

AGTCATGATTTCAATCGCCATTTAACACCCTTCAAAGCTGAATGGGATCCAAAGGATCCA

TTAGAGTCACTTGCTGTTGTTGGTCGTTATATAAGTGAAAATTTTAATGGCACAGCCCTG

CATCCTATTGATTTTATAGATACAAGTACAGGGCAGTTGGTAGCAGAAGTCATGGACCCG

AACATCACAACCATCAGTCCGGTGAATAAGCTTCATCCACGTGATGATATCCTGGCAACT

GGGAGTTCTAGGTCTCTGTTCATTTGGAAGCCAAAGGAAAAATCTGAGACGGTCGAGGAG

AAGGACGAAAGCAAAATTGTGGTTTGTGGAAAAGCTGAGAAGAAACGTGGTAAGAGGAAA

GGTGATAACATCGATGAATCCGATGATGATGGATTCATTTCCAAGCTCAAGAAGCCAAAA

TCCAAGCAGACTGAATGGAAATTGTCTCGCTGCTCGACAAAGGATAATCGCTAA

>MS.gene009882.t1

ATGTTTTCGTTAAGACTTCCCATGCCACTAGATAAAACTCTTATAACTTCTCCACCAAAG

ACAACTACTACTACCAACCATGTTCATCGGGGGATCACTCAGATACAAACTCATTCTCGA

TATTCTTCAAGGTTTGTTACCAAACGCGTCACTATTGCCGCGGCTTTTCAGGATCAGGTG

GACGAGGAAACTTCCGTGGACGGAGATTTTTCAGAAATGGAGCCATTGCCGGAGGAACTG

CAAGCGGAGCTGATGCCTAAGCATGTGGCGGTGATTATGGACGGGAATGGGAGGTGGGCA

AAGATGAGGGGGTTACCCCCGTCTGCGGGTCATGTAGCCGGTGTAAAGTCGTTGAGAAGG

ATGGTGAGGTTGTGTTATAGTTGGGGAATAAAGGTTCTAACTGTTTTTGCATTCTCAACC

GATAATTGGATTCGACCTAAGGTGGAAGTTGAATTTTTGATGAGCCTCTTTGAAAGAACA

ATAAATTCTGAAATTGAAGCACTAGAGAGGTGTCAAATTCTAACTGCTTTAACTAATTAA

>MS.gene009869.t1

ATGAGATTCGGCGAATCAGGCTGCATTTACGCCACCTTGATCCTCCACGAAGATGGAATC

CCTATCACTGTAATCTCCAAATCGCTCATAGGCCTATTCGCTAAGCTTGCTCAGAGCAAG

AACGTTGATGATCTCGTTTTGAACTCCGGCGCTGTTGGTGGTGCCGCCGTTGCCGTATCT

GCACCTGCTTATGGAGGTGGAGCCGTAGCAGCAGCCGAACTGCCGCTACTAGGACTCCTT

TCAAGGGGGTGA

>MS.gene009947.t1

ATGTGTTACATTCTTGAAACTATTTTTTGGCCGAACCAGGGTAGGGTCCCCATTCCAAAC

CAGTACTACAAAAAGCATTGTAAGGAAATTAACATCGAGGATCCAGGATTGCTTCTTGGT

GAAGGAATAAACTATGTTACACATTTCGACAGGGATGAAACAGGGGTGTATATGGGTAGT

GGAATTAGGATCACTAAGGAGTTTGGGTTCTTGCTTCCAACCAAGGTTAGGCTAGACTAT

GAGGGTCTAGAGAATGTGTAA

>MS.gene009914.t1

ATGGCTCAGACAGTGTGGCCTTGTTTGTCTTCTTGGGGGTTAAATAAGGTGCTCACTATG

ACAGTCGATAAATATGTATCATCTAATGATGTTGGAATATCGCACCTCAAAAATGCCCTT

ATGCCTGATGGTTTACTTATGGGAGGAAAGTATTTTCATACTCGTTGTTGCACTCATGTG

TTAAATCTGATTGTGAAGGAAGGTCTTAAGGATATTAAGCGTGAAATTGTGAGGATTCGT

GGTGCAGTGAGGTACGTTCAAGCTTCCCCTTCAAGGTTACAAAGATTCAAGGCATGTATT

GATCCACAAAAGGTTCAGTTTAAGGGTTTTATCAACATAGATATTGAGACAAGGTGGAAC

TCTACCTATTTAATGCTCAATGCTGCAGTTAAACACAAGCCAATATTTGCAATGTTGGAG

ATGCAAGATTAA

>MS.gene009941.t1

ATGCCCGACCAAAATTTAAACAAGGTCCAGATAGTGACAGCTGGTGCAGTGCCTCCTCTG

GTGGAGCTCCTCAAGATGCAAACTAACGGTATACGAGAATTGGCTACAGCCGCAATCTTG

ACGCTCTCATCTGCTGCAACCAACAAGCCCATTATTGCTGTTTCTGGAGCTGCTCCTCTC

CTTGTTCAGATTCTCAAATCTGGAAGTGTTCAAGGCAAAGTTGATGCCGTTACAACCCTC

CACAATATCTCCTCTAGCACCGAGAATCCCATTGAACTTCTTGATGCTAGCGCTGTTTCC

CCTCTTATCAACCTCCTTAAAGACTGTAAGAAGTATTCAAATTTTGCAGAAAAAGCTACA

TCACTACTCGAAATCCTTTCCAACTCTGAAGAGGGACGGATCGCAATCTCACTTACAGAA

GGTGGAATTTTAACCCTGGTAGAAACAGTGGAAGATGGGTCACTTGTCAGCACAGAACAT

GCTGTTGGAGCGTTGCTGTCATTATGTCAAAGCTGCCGGGATAAATATCGGGAACTAATC

CTTAAAGAAGGAGCGATTCCGGGTCTGTTACGGCTGACAGTAGAGGGCACAGTTGAAGCT

CAAGATAGAGCTCGCATGCTTCTGGATTTACTCAGAAATTCTCCTCAAGAAAAAAGACTA

GACTCCTCGGTTTTGGAGAAAATTGTTTACGACATGGCTGAAAGGCTAGATAGAGTATCG

GTAGATAAAGCTGCTGAAACTTCCAAATGA

>MS.gene009917.t1

ATGTCGAACGTGAGCATAAGAGCTGCAACATACAACGACATGTCTGAGCTTTGCCGCGAA

TATGATCTTATCTTGCCTTTGAGTACTGGAGATGAACATTTTCACGTCGCGGTCAGGAAC

GGCGGCCGTCGCAATATCGTCGGTTATGTCTGCTCTATTAAGAAGGAGGAGGAGGTTAAG

AAGGAGGTTAAGATTTACGCTGAAATCAGTCACGTCGTGGTTCTCGCAACCTATCAGAGA

CTTGGTATCGCTACAAAGCTTGTGACTGCCGCCCATAAGGATTTGGAACAGAATTATCAT

CCTGAGTACATCTCAGCGCTCGTCCCGAGGTACAACATTCCAGCTTTCATTTTGTTCCAA

AAGGCCTTGGATTACGATATTTATGAAGCTGATTGTACGAAAAACAGTTATCACTTTTAT

AAGCCGATATCTTCTCGGGAGACCAAATGA

>MS.gene009921.t1

ATGCTTTTTTCTGATGAAGGAATTAACTATGTCACATACTTTGGCAAGGATGACATAGGG

GTGTACATGGGTAATGGAATCAAAATTGCCAAAGAGTTTGGATTTTTGGTTCCAACCAAG

ATTAGGCTCGACTACGAGGGCATCAAAAACATGTATAAGCTGACACAACTAGGACCAATT

TACATTCCAACTGTTTTCCCCGCACGAGTTAAACAAGAACCCATTGAGATCATTTCAGAC

GATGACACTGACACTGAACAAACAAATGGTGAGGATGAAGCAAGAACTAATAATCAAATT

CAGCCAAATGCAGGTCTTGTCAACAATTTGCAAAGAACTAATGCGGTGCATTATCATCAC

TTTGAGAAGGATGTGGCAGCAGCTTTAGCCTCCATTGAAAAACAACAAGCCTTGAAGGTG

TTATAA

>MS.gene009940.t1

ATGTTGGGTCGGTCTGCATTTTCTAGACCTGGAGGTTTCAGACCAGAGAATTTAGGTCAG

AATACTATGGCCATGATTGGGAATGTTTGTTTTTCAGTGTTTGTTATTGGTGTTTTGGTT

TTCACTATCATGGCTGCTACTTATGAACCTGAAGATCCTTTGTTTCATCCTTCAACTAAG

ATTACAACATTGTTCACTTCTAAGTCCAATGCTACTTTTAAGTCTGATAACAGTGTTGTG

AAAACTGGTGAGGATTTTATGGCTACTAATGAGTCTGTGTTTGGCTCTATTCTTAATATG

ACTGATGTTGATAACTCGGTTAGCGGTGAGACTAATGAGGCTGAGGTGACTCAGTGTGAG

GGGAATTCAGGTCCTATTGATTGTAAGAACCCTGAAGTTTTTCATTTGATGATGAGGGCT

ACTATTGAGAAGTTTAAAGATGTTCATTTTTATAAATTTGGGAAACCTGTTCCGGGTTCG

AATGATAGTACCTGTGATATGGCGTGGAGGTATAGGCCGAAGGATGGGAAGGCTGCGGCG

TTTTATAAGGATTATAGGAGGTTTGTGATTGAAAAGTTTGAGAATTGTACGTTTAGTGTT

GTTAGTATGGGGGATTATCATACTGGTATGAATGCGAGGAAGAGGAAGAAGAATCAGAAG

GCTGGACTAGAGAAGACATCGTCGAATCTGGATCAGGTCAATGCGTTGCCGGTTGTTGGA

GAATTTGTGAATGACAGCCTCCCTGTAGTTGAGTCGGAAAGTTCATTTAGTCAAGGTAAA

TACTTGGTTTATGTGGGTGGTGGAGATAGGTGTAAAAGCATGAATCACTTCTTGTGGAGT

TTCTTGTGTGCTCTAGGAGAAGCTCAGTACCTAAACCGAACATTGGTTATGGATTTGAGC

ATATGTCTGTCTTCAATTTACACTTCATCGAAGCAGGATGAGGAAGGCAAAGATTTTAGG

TTTTACTTTGATTTTGAGCATTTGAAGGAGGCGGCGTCTGTGTTGGACAAGGATCAGTTT

TGGGCAGATTGGAGTAAGTGGCAACAAAAAGATGGGATGAATCTTCATCTGGTCGAGGAT

TACAGGGTCACACCAATGAAACTTATGGATGTGAAAGATGCTTTAATCATGAGAAAGTTT

GGAGATGTCGAGCCTGATAACTATTGGTATAGAGTCTGCGAAGGAGAGACAGAATCTGTT

GTTCAAAGGCCATGGCATTTGATATGGAAATCGAGGCGATTGATGGAAATAGTGTCTGCA

ATAGCTTCAAGGTTAAACTGGGATTATGACTCAGTTCATGTTGTGAGAGGGGAAAAGGCG

AGGAACAAAGAGCTTTGGCCAAATCTTGATGCACATACCTCCCCCGATGCACTTCTCTCA

ACCTTACGGGACAAGGTTGACGAAGGAAGAAACCTCTACATTGCTACAAATGAACCTGAT

GCATCTTTCTTTGATCCTCTGAAGGATAAGTATTCAACTCATTTTCTCAACGAGTATAAG

GAACTTTGGGATGAGACTAGTGAATGGTACTCCGAGACAACAAAACTCAATAACGGCGTT

CCAGTAGAATTTGATGGTTACATGAGAGTATCCATTGATACAGAAGTGTTTTTGAGAGGT

AAGAAGCAACTTGAAACTTTCAATGATTTGACCAGTGATTGCAAGGATGGTATCAATACA

TGTAATGTTGCTGAAAACTAA

>MS.gene009891.t1

ATGGCAAATAGGTCTTTTACCATCCTCCCACCAACAAATAAAAGAGTTGGGAACCTTACT

AACCCTAAATCAAAAACGACTAGAAACAACAGTCACAGGCCGGGGGGACTAGAGGAGGAA

GACGCAGGAATGCAGACAGAGACATATCTTCCTATTGAGTTAATCATCCAAATCCTATTA

AGGTTACCAGTCAAGTCTCTTATTCGTTTCAAATGCGTTTGTAAGTCATGGTTTTCTCTT

ATCTCTCAACCTGATTTCGCAAATTCACATTTCCAACTTACTGCCGCGACACACACCAAT

AGAATTCAGTTGATAACTCCATATCTTGAATCTCTATCTATAGATCTTGAATCATCGCTT

AGCGATGATAGCGCTTTTTATTCACCGGACATTAGTTCTTTGTTTGACGATGATGATTAT

TATAGTTCTTCTTCGTCGGACAGGGATGATTTAATGCCTCCCAAATATTTTTTTAAAATT

GATTTCAAAGGTTCATGTAGAGGCTTTATACTTTTGAACTGTTATTCAAGCCTCCGTATA

TGGAATCCATCCACCGGATTTCACAAACAAATACCTTTTACTACACTTGATTCCACTCCA

GATGCAAATTATTTCTATGGTTTTGGGTATGACGAGTCAACAGATGATTACTTGGTGCTT

TCAATGTCCTATGAACCGAGTCCGAGCTCTGATGGTATGCTATCTCACTTGGGAATTTTC

TCATTGAGAGCTAATGTGTGGAGCGAAATTGAGGGTGGTAAACTTTTGCCTTATTCGCAG

GACAGTATTTTAAACTTAGTGGAGTCGCTCTCAAATGGTGCTCTTCACTGGTTGGCTTAT

CGTGATGATATATCAATTCGTGTTATTGTTGGCTTTCATTTAATTGAAAGGAAACTTCTA

GAGTTGCGTTTGCCAAATGATATAGTGTATGGTCCTAGTAGTGTGTATGAATTATGGGTG

TATAAAGGATGTCTTGCTCTATGGGATCTTTCGTCTGATAAAGGTGCAGTTGAAATATGG

GTGATGGAAAAATACAATGTGCAATCGTCTTGGACTAAGACTCTTGTTCTGTCTTTTGAT

GGCCTTCCTACTCGCTGCTTTTGGCCGAAATACTATGCAAAAAGTGGTGATATCGTTGGA

ACAAACATGCATAGTTTATTGGCCAAGTATAATGACAAAGGACAGCTGCAAGAGCATCGC

TCCTACTGTGACAATGAATATGGATCCCTCAAGGTTATGTATACAGAATCTCTGCTTTCA

ATTCCTGGTGGTGACAGTGGTCAAGTTTAA

>MS.gene009857.t1

ATGTCTTACGGTTCCAAAGGGTTTGCTTTTTCTGAGTATGGTCCTTATTGGCGTAGTATG

AGAAAACTTTGCACTTTGAAACTTCTTAGTGCTTCAAAAGTTGAAATGTTTGCTCCCATT

AGACAAGAGGAGTTAGGTGCTTTGGTTATGTCGTTGGAGAAAACTGCTATGCTAGGTGAA

GTTGTGAATGTTAGTGAAGTTGTAGAGAATCTTATTGAGGATATTATGTATAAAATGATA

TTTGGTAAGAATAAGTGTGAGCAATTTGGCTTGAAGAAGTTAGTTCAAGAAGGAATGGCT

TTGGTCGGAGCTTTTAATCTTGCTGATTATGTTTCTTGGTTAGGCCCTTTCGATTTTCAG

GGAATAACACGAGCTTGCAAGAAAACGAGTAAAGCACTTGATGAGGTGTTAGAGGCGATA

ATAACTGAGCATGAGCAAACTGCTAGTGCAACCGGAACTCATCATGAAGATTTTGTAGAT

ATACTTCTCTCAATTATGCACCAAACCATAGATTCAGAAAATGAGCAAAGTCATGTTATT

GATCGAACTAACATCAAGGCAATTTTACTAGACATGATTGGAGCTGCAATTGATACATCT

GCTACCGCAATTGAATGGGCTTTATCTGAACTTTTAAGGAATCCAAGGGTGATGAAAACT

CTTCAAGATGAGATACAAAATGAAGTAGGAAATAAAAGAATGGTAGATGAGAAAGATTTG

AAGAAGTTTAGATACTTGGATATGGTGGTTGATGAAACATTAAGACTTTACCCTGTTGGA

CCCTTATTAATCCCTCGTGAATGTAGAGAAAGCACAACAATTGATGGATATTTTATAAAG

GAAAAGACACGAGTTATAGTAAATGCATGGGCTATAGGGAGAGATTCTAATGTTTGGTCA

GAAAATGCTGAAGAATTTTATCCAGAAAGATTTATTGACAACAAAATGAATTATCAAGGA

CACGAGTTTGAATCTATACCATTTGGTTCCGGTCGTAGACGTTGTCCCGGAATTCAATTG

GGTTTGATTACTATTAAATTGATTCTAGCTCAATTTGTGCATTGCTTTAATTGGGAACTT

CCATGTGATATTAGTCCTTCCAATTTGAATATGGAAGAAAAGTTTGGACTCACTATACCT

AGAGCTCAACAGTTGCACGCAATACCAAGTAATCGTTTGGCAAGTGATGCCAAACATGAA

TAG

>MS.gene009926.t1

ATGCAAGCTTATCTTCATCCAAATTCCAGTTTACCATATCTTTGCTTGACATGCCCTCTC

TCATGTGCTCCGATATTTGTCGACTTAGACAATGGAGCCACCGATTTTGTTAAAGATTTT

GTCTCAGAGCAATTAAATGAACAAAACCCTGCCTCGCATAACGAGTGTTCTGATGGAAGA

ACTTCCTCTATACATACTGCTGCATGTTTAAGTAAATATGGACACCTGGTTTATGTAGGA

AACTCAAAAGGGGAAATTCTCTTAATTGACAATAGAGATGGTGAGGTGTGTGCCATGGTT

CCTATTTGTGGTGGTTCTACAGTGAAGAACATTGTATTCAGCATGAATGGACAATTACTT

CTAACGAACTCAAATGACCGAATAATCAGGATCTATAAAAATCTTCTGCCACCGAAAGAC

GAAGTTAAAGCACTTAATGAATTAAATGAGAATAAAAAAGAACTAAATGGAGTTGAGAAT

GGTGACGGTGAGTGGGTTGCTGCTGGTTCTGCTAGCAAAGGAGAGCATAAGGTTTACATA

TGGGATAAAGTTGGACGTGTTGTGAACATCTTTGAAGGGCCTAAGGAAGGTCTTATCGAT

TTAGCATGGCATCCTATACGACCTATTGTTGTATCTCTTTCTCTGAAAGGTAAAGTTTAT

ATCTGGGCTAAATATTACTTTCAGAGCTGGAGAGCGTTTGCTCCTGATTTCATAGAGATA

GAGGAAAATGAGGAGTATGTGGAGAGAGAAGATGAATTTGATTTAAATCCGGATACTGAG

AAAGTAAAAGAATCGGATGTCAATGAAGACGAAGAAGTTGACATTTTAACAGTGGATGAA

GGTAGTGTTTTTAGTGATATTGAAGAAGAGTTAATCTACCTACCAGTAAGTCCTATTCCG

GATGTTCTCGAGCAACAAGACCAGCTCTTAGAAAGCTCTGTAAATATCGTGGACAACAAA

AACACTGGATCCCCACTTTCTGAACCAACTGGACATTTGACGAACCATGCCTTTAGTCCA

GTTAAAGATGTTGCTGGGGGATCACAAATTAAAGGAAAGGGAAAACCTTCGAAGAAGCCC

TCAAAAGCTAGCAACTCATCAAAGCCAAAAAATGAGCCAGTGGTATCCAAAGGAAAAGAG

AAGGCTACCTAG

>MS.gene009900.t1

ATGTCTGATTCCATGTCTGATAGTGAGTCCCAGCACTCACCCAAACATTCTTCACCCAAA

CCTGAATCATCCCAACCTGTCAGGACATACCATGAAAATCACCCTGTTCAGATCACAACA

ATTCGGCTGAACGGTGCTAATTACTTTCGTTGGTCACAGTCTGTTCGGTTGTATCTCAGG

GGGAGAGGAAAGATTGGCTACATCACTGGAGACAAGAAACAACCAGACAAGGCAGGCGCA

GATTATGATACATGGGATGCTGAAAATTCTATGGTCATGACATGGTTAGTCAATTCTATG

ACCGAAGAGATAGGTGCGAACTATCTCTGTTATGATACCGCAAAAGATCTTTGGGAGAGT

GTCTCTCAAATGTACTCTGATTTGGGAAACCAGTCCCAAATTTACGAATTAACTCTCCAG

CTTGGAGAAATTCGGCAAGCTGAAGATTCAGTCACCAAATACTTCAATTGTCTCAAACGT

ATTTGGCAAGATCTGGATCTCTTCAATGAATATGAGTGGAAGTCACCTGAGGACTGCAAG

CACTACAAGAAGATGGTGGATGTTAGCCGTGTCTTCAAATTTCTTGCAGGTTTAAATGTT

GAGTTTGATGAGGTTCGTGGCCGAATCCTTGGTAGGAATCCTATCCCTCCAATTGGTGAC

GTCTTTGCGGAAGTGCGTCGCGAAGAGAGTCGTAGGCAAGTGATGCTCGGAAAAAAGATA

GCCGCTGTCCCGCCACCGGTTGAAGGATCTGCCTTGGCCGTTCCTCAAGTCAATCGCAAA

TCTTTCCCTAATCCGCGAGGTGGTGACAAGACTGATTTAGTTTGTGACTACTGTGGCCGC

AATCGTCACACTCGAGAAACTTGTTACAAGCTACATGGTAGGCCAAACAATAGCAAGGCT

GGTAAGTTTGGTCACCGACCTATGCCTACAACCAACGATGCTGCTTCATCTCCATTCACT

AAGGAGCAGATGGACCACCTTCTTAAGCTTCTAAAGTTCAATTCATCTCCTAATACTCCT

GTTGGTACTGTGGCACAAACAGGGGGAGGGTCAAAGTAG

>MS.gene009873.t1

ATGATGGCAGGGGGATTGATTAGTTTCATCGGTTGGAAGTTGGGAGCGAAATTGGCAACT

GATGAAATTGGAATTGAGGGGAAATACACCATTGATATTGATTTTTGTAAAACTATACAT

ATGGTTAGGGACATAGATGGTGAAGGCAGGAATTTTAGATTGTTGGTATCCGACGAAGAT

TCAATTTTGTTGCCGGATCCATTGAGAGCTGACACAACAAATCTTGCAAACTGGCTTTAT

AATGATTTAGATCAACAGGTACCTCCAGGGCAACCTGCGCAAGATGAAGAAATGGGTAAG

GGGCAACAACCTATCGAAGGAAGGGAAAATGATGATGATTGGCGTCGAAGGATGGAGGCT

AAGGCTGACAGGACGTATGAGGAGGTTACACTCATCAGACAAATGCTTGCAGCACATATG

CAACAACTCAACATGCGATATCCTCCAGCCGGTCCACAATAA

>MS.gene009844.t1

ATGTCCTTACAACTTGGTCAAGTCCCATCTATTGTCATTTCATCTTCAAAAGCCGCAGAA

TCATTCCTCAAAACCCGTGACATCGTTTTCGCAAGCCGGCCTAAGAGTCAAGGATCTGAG

CTCATGTTATATGGTTCCAAAGGGATAGTTTTTTTTTCTTCTGAGTATGGTCCTTTTTGG

CGTAGTGTGAGGAAATTTTGCACTTTGAAACTTCTTAGTACTTCGAAAGTTGAGATGTTT

GGTCCTATTAGGAAGGAAAAGTTGAATGTTTTGATTAAGTCATTGGAGAAAGCTGCTTTG

GAGGGTGAGGTTGTGAATGTAAGTGAGACTGTAGAGAATCTTATAGAAGATATTGTGTAT

AAGATGTTGTTTGGTCGTAGTAAGTATGAGTAA

>MS.gene009879.t1

ATGGCAGCTTCCGATGGAAGCGAGTACTTCGAAATCGGATCAATCGGAAGCGAGAGTTTT

GCGAGAGCGTCGAATGCAGAATGGGTGGAGGAAGACGAAGAGGAGCTTCATTGGGCGGCG

CTTTCACGTTTACCGTCGCAGAAACGCATCAACTTCGCCGTCCTCCGTGCTTCATCTTCC

CGTCAACCTTCAAAGGAAAATACCGGCGAGAATTTAGTCGACGTGAGAAAATTGAATCGG

TTTAACCGTGAGCTCGTCGTTAAAAAAGCACTCGCTACAAATGATCAAGATAATTACAAG

CTTCTCTCCGCCATTAAAGAACGCCTCGATAGGGCTGGAATTGAGGTACCGAAGATTGAA

GTTAGGTATACGAATTTGACTGTGAGTGCTGATGTTCTAATAGGTTCACGAGCTTTACCG

ACTCTGTTCAATTACACTCGCGATGCATTAGAGGGTATTTTAACGTCGATGAAGTTGTTT

AGAGCTAAACGGCATTCACTTACTATTTTGGACAATGTTAGTGGCGTCATCAAACCAGGA

AGGATGACTTTGCTACTTGGACCTCCAGGTTCCGGCAAATCCTCTTTACTTATGGCTCTC

GCGGGAAAACTTGATAAAAATTTGAAGAAAACTGGTAGCATAACATACAACGGCCATGAG

ATAGATGAATTTTATGTTAGAAGGACTTCTGCATACATTAGCCAAACAGATAATCACATT

CCTGAACTGACAGTGCGAGAAACTCTGGATTTTGGTGCTAGATGTCAAGGGGCAGAAGAA

GGTTTTGCAGAATACACGAAAGATCTGGGCCACCTAGAGAACGAAAGGAACATACGACCT

AGTCCAGAAATAGACGCATTTATGAAGGCATCCTCTGTTGGGGGTAAAAAGCACAGTGTG

AACACTGATTACATTTTGAAGGTACTTGGTCTTGATGTATGTTCAGATACAATAGTTGGT

AACGAAATGACCAGGGGAGTCTCCGGGGGCCAAAGAAAGAGAGTTACAACAGGCGAAATG

ATCGTTGGTCCCAGAAAAACGCTATTTATGGACGAAATATCAACTGGGCTTGATAGCTCT

ACTACATACCAGATAGTGAAATGCATAAAGAATTTTGTTCATCAGATGGAGGCTACAGTA

CTCATGGCACTCCTTCAACCAGCTCCTGAAACATTTGAGCTATTTGATGATCTGGTGCTT

CTGTCCGAAGGGCATGTTATATATGAAGGCCCTAGAGAAGATGTGCTTGAATTTTTTGAG

TCAATAGGTTTTCAACTTCCACCACGTAAGGGGATTGCAGACTTCCTTCAAGAGGTCACC

TCTCAAAAGGATCAAGCACAATACTGGGCTGATCCTTCTAAACCATATGAATTTATCTCA

GTTCGTGAGATTGCAGAAGCCTTTAGAAATTCTAGATTTGGAAGGTATATGGACTCCTTA

CAGGCTCATCCATATGATAAATCGAAGTGCCATCCTTCAGCTTTGGCTCAAAAAAAATAT

GCTGTGTCCAAATTGGAGGTCACAAAAGCTTGCTTTAACCGAGAAGTCCTTTTGATCAAA

AGGCATAGTTTCCTTTATATTTTTAGAACCTTCCAGGTTGCTTTTGTTGGATTCGTCACA

TGTACAATATTCCTGCGAACAAGGTTGCATCCTACAGATGAGTCTTATGGGAGCCTTTAT

CTCTCTGCTCTATTCTTTGGGCTTGTTCACATGATGTTTAATGGGTTTTCTGAGCTTCCT

CTGATGATATCGCGGCTCCCAGTATTTTACAAGCAAAGAGATAATTTATTCTATCCTGCA

TGGGCATGGTCTTTTACCAGTTGGATTCTCCGTGTACCTTACTCTGTTATTGAGGCTCTT

ATATGGGCTGCTGTTGTATATTACAGTGTTGGATTTGCCCCTGCAGCAGGAAGGTTTTTT

CGCTACATGTTTATACTTTTTGTAGTGCACCAAATGGCATTAGGTCTTTTCGGGATGATG

GCTTCTATTGCACGGGATATGGTTCTTGCCAATACATTTGGCTCAGCTGCACTGCTGATT

ATTTTCTTGCTGGGAGGATTTATTGTACCTAAAGAAATGATCAAGCCGTGGTGGATATGG

GGCTACTGGTTGTCACCCCTTACCTATGGACAACGTGCAATTACAGTTAATGAATTTACT

GCTTCCAGATGGATGAAGAAATCAGCAATTGGGAACAACACAGTAGGTTACAACATCCTC

GTTTCAAACAATCTACCAGTTGATGATTACTGGTACTGGGCTGGTGCTGGAATTTTAATA

CTCTATGCAATTTTTTTCAATAGCATGGTCACTCTGGCCTTGGCCTATCTGAATCCACTT

CAAAAGGCACGCACAGTTATCCCTCTGGATGATGATGGTTCAGGAAAGGATTCTGTTAGT

AATCAGGTCTCTGAAATGAGTACCCATTCCAGGTCTAGAAGAGGCAACAGTAATACGAAG

GGGATGATTCTTCCATTTCAACCATTGACAATGACATTCCATAATGTCAATTATTATGTT

GATATGCCAAAGGAAATTAGAAATCAAGGCATAGTTGAAACAAAGTTGCAACTCTTATCA

GATGTAAGCGGAGTTTTCTCACCGGGTGTCCTGACAGCACTAGTGGGGTCTAGTGGGGCT

GGAAAGACCACTCTAATGGATGTACTTGCTGGCAGGAAAACTGGAGGGTATATAGAAGGG

GACATCAAAATATCTGGTTACCCAAAAGAGCAACAGACATTTGCCAGAATATCGGGATAT

GTTGAACAAAATGATATACATTCTCCTCAAGTTACCATTGAGGAGTCGCTGTGGTTTTCT

GCATCTCTTCGCCTTCCAAAGGAAATCAGCATTGATAAAAGACGTGAATTTGTTGAACAA

GTAATGAAACTGGTTGAGCTTGATTCTTTGAGATATGCTTTGGTTGGTATGCCAGGCAGT

TCTGGTTTATCAACAGAGCAGAGGAAGAGATTAACAATTGCAGTGGAGCTTGTAGCAAAC

CCTTCCATTATTTTTATGGACGAGCCTACATCTGGACTTGATGCTCGTGCAGCAGCTATT

GTTATGCGAACTGTTCGCAACACTGTTGATACTGGAAGAACTGTTGTCTGTACCATACAT

CAACCAAGTATTGATATTTTTGAAGCATTTGATGAATTACTTCTTATGAAACGTGGGGGA

CGAGTTATTTACGGGGGAAAGCTTGGTGTGCATTCACAGACATTGATAGACTATTTTCAG

GGAATCACTGGTGTCGCCCCTATTCCAAGTGGCTACAATCCGGCTACCTGGGTACTTGAG

GTCACTACACCTGCTGTTGAAGAGAGAATTGGTTCAGATTTTGCTGAAATTTACAAGAAT

TCAGCGCAATTCAGGGGGGTGGAGGCTTCTATTTTGGAATTCGAGCGTCCTCCTGCAGGA

TTCCAACCACTGAAGTTTGACACAATATATTCACAAAATCCGTTGTCCCAATTTTATCTG

TGCTTATGGAAACAAAATCTTGTGTACTGGAGAAGCCCGTCATATAATGCCATGAGGATG

TACTTCACCACAATCAGTGCTTTGATATTTGGTAGTGTATTTTGGGATATTGGTTCAAAA

AGATCATCAACTCAAGAGCTGTTTGTGCTTATGGGAGCTCTTTATTCTGCGTGCTTGTTT

CTTGGGGTAAACAACGCTTCTTCTGTACAACCAATTGTTTCAATAGAAAGGACAGTATTT

TATAGAGAGAAAGCAGCAGGAATGTACACTCCATTGGCATATGGAGCAGCCCAGGGACTT

GTAGAGATACCATACATTGCTGTTCAGACAATAGTGTTTGGCTTAATAACATATTTTATG

GTCAATTTTGAGAAGACAGCCGGGAAGTTTTTGCTCTATCTATTGTTCATGTTCTTAACC

TTTACCTACTTCACCTTTTATGGAATGATGGCTGTTGGTCTTACACCTTCACAACAATTT

GCTGCTGTTATTTCTTCAGCATTTTACTCCCTGTGGAATCTTCTTTCTGGTTTTCTTATC

CCAAAATCGCATATTCCTGGATGGTGGATTTGGTTCTATTATATATGCCCTGTTCAATGG

ACATTACGTGGCATTATAACATCTCAGCTTGGTGATGTAGAAACAAGAATTGTGGGACCA

GGATTTGAAGGCACTGTGAAAGAGTATTTATCTGTAACTCTTGGATATGATCAGAAAATC

AACGGAATTTCGTCCGTGGGTCTTTCTGTGATAGTTCTCATTGCATTTATTCTCCTATTC

TTTGGTAGTTTTGCTGCATCAGTCAAATTATTGAATTTCCAGAAGAGATAA

>MS.gene009944.t1

ATGAGTAGTAGTGGGTTTAGGGCTTCAATTCCAAACAGTGTGAAGAAAACGATCCAGAAT

ATCAAAGAGATCACAGGGAATCATAGTGATGAAGATATCTATGCAATGCTTAAAGAATGC

TCTATGGATCCCAACGAAACCACTCAGAAGCTTCTCCTTCAGGACACATTTCATGAGGTC

AAAAGAAAGAGGGACAAAAGAAAGGAGAATCTCAACAACAAAGAACATGTGGAACCACGC

GGGAGACCTGGCCCCCACGGACGGGGGCCTAGGGGTGGTCGGGGAAGTTTTGCTTCTCAT

AATATATCTCATGATACTAGTGGAAGAAATACTGCTGTTACAGGCAAAGAGAGTGGAGCT

CATCTACCTTCGGAGAAAGTTGCACCACATTTATCAGCTTCTCAAGAAATAGTATCTGAA

GGAAAAAGTTCCGGAACAAGCTCTGCGCCCATTATTGCCAATGGTCCCACAAATGTTGCT

TCTGGAACTATTAGTGGTGTGAGTCCTTCTCTTTCGTCTGCTGGGAATGGAGAGATAACG

GTTCAATCTTCTGGCAATAATACTAATAATAATGTGGATAGTGCTTCACCTTCCGATAAA

TCAAATGAGGTTGCAACAGTTGCTTCTGAAACTGGGTCGGCATCGTCATCGGCTGCTCAT

TTCTCCTCCTCTGATCCAGTAATGGTTCCATCGGATAATTCATGGTTTCCTGGTGCTGTT

GGTGCCATTAGACGCGAAATCGGAAATCAACATCAACCCTCTCTTGGGGAATCAAATGCT

GTTAGCTCAGCCAAGAACAAATTAACTGCCGCTTCTGAGACTGGTAGCTCTTCTGTGCAA

GGAAAGATTCAAGGTAAATCCCAGGGAGTGGCAAAGAATCATGTCAATGAGATACCATCT

CCATCATCATCAGTAACTCATGGAAGCCCTTCAGTTAGTCGCCCTTCCTCTAATTACAAT

AACCGGTCTCAACAATTGATTGGCCCTCAGAAAGCTGGTTCTACTAAGGAATGGAAGCCA

AAGCCAACAAATACATGTGGTCAAAATTCTGGACTTCCTGCTGCATCTGAGTCTCCTCCC

GTATCAGTCGAAGTAACTAGGCAGTTACAGTCTGCGTCTGGTGCCCTTGACACTGAAGAA

GCTACTTCAAAACTGCAGAGGAAGCTGGAAGAATTGCATCTTCCACAGCGTCAACACGTT

ATACTTCCAAATCATATCATAGTACCTGATTCTGAGAAGAACAAATTTTGCTTTGGAAGT

TTGGGAGTCAATTTTGGAGTCAATGCAACAAGCTACGTTAGTGGCCCAGATAGTGAAAAG

AGCTCCACACCACTTTCTGAAACATCCCAGGATATTGAAGAATCTGTGGAGGAGCAGAAC

TCAAGTCAAAATGGTGCACTTACGTCTGAGGTGGAAGATTATCCTGACCATCCACAATCA

CCCAATAAAGTACCTGTGAATTTAGAATCGAGTGAGATTGACGGGTCATCTAGTGCTATT

CAGGAGTATAATGAGTCTAAGCAAAACACTGCTTTGCCATCCGAAGGTCATCAATACCCA

GGCGTGCAAATTTCTCCAAATTACAGCTATGGTTTTGTGCCCCCAATGTTGGGTTCTCAG

CTCCCACCATTTGAAAATTCTGAGTCTCAGACACGTGATATTTCTCGGCTTCCAAACTTT

ATTGTCAATCCACAATATGATCCAGCTGGTTACTATGCCCAGCTTTACCGACCAGGTGCT

GATAGTGACGGCCGTCTTTCTCCTTTTGCTTCTGCTGGGGCTACCACCAAGTACAATGGC

AATGTTGCGGTGCCACCTGCTCCAAATGCTCAATCTCCTCAAGAGGGAGGTATCTTGTCC

ACAGTAGGTCAAACACCAGTTGCGACTCAAGCTGCAGGACTGATGCAAAGCTCAATACCT

GTTACCCAGCAACCTGCCCCTGTCTTCCGGCCAAGTGGAGTTCATGTACCCCCTTACCCT

CCAAACTACATTCCTTATGGTCACTATTTTTCACCATTTTATGTCCCACCTCCAGCAATG

CACCAATTTTTGGGTAATGGTGCATTTCCTCAACAACCTCAGGGAAGCACTATATATCCA

CCACCTCCAAATGTGTCAGCCCCAGGAATCAAATATCCTCTTCCACAATACAAACCTGGA

ACAAATGCAGAAAACCTGACTCACAATGTAATGCCAAATGCTTTTGGACTATATGGCTCC

TCTCCAGCTGGTTATAATCATAATTCTGCAGCGACTACCGGGAATTCGAACTCTCATGAG

GATCTCGGTTCTTCCCAATTCAAGGAAAATAACGTATATATCAGCGGACAACAGAGTGAA

GGTTCAGCGATGTGGGTGGCTGCACCTGGCCGAGACATGACTAATTTGCCCACTACTTCA

TTCTACAACCTTCCACCTCAGGGTCAGCATATGACTTTCGCCCCGACTCAGGCTGCCCAC

GGAACTTATGCAAGCATCTACCACCCCGCACAAGCAGTGACAGCTGCAGCAGTCCATCCG

TTGCTACAACAATCTCAAACAATGGCAGGACCTGGAGGAAATGTTTATCAACAGCCTCAG

CATGCACAGATGAATTGGCCAAGCAATTACTAG

>MS.gene009865.t1

ATGGGATTAAGACGCAAAGACGTTTACCCTTATATCTGCTTCAATGACCATCGCAAAAAT

CAATTATACTGGAAGAGATCCAGAGTACATGAAAGATATTTTACTGTTCAACCTAAGAAG

GATAAATGTTCTAATTCAGGATTGCCTTCAACACACAAGGGATTAACCGTAACAAACGTA

CCTAATGATGAAGAGAACGTCTTTTGGAGTGGAGGAGGTGACGATGAGTCGGGTGGTGGT

ATCGAGGATTGCAATTTTGATAGTGGGGCGTGGGTCAGAGCTCCGTGCCCGTTAGCAAGA

TCTATGATAGCAGTAATGGTTCCTGATAGAGTTCTCTACTTGGCTTTGCTAAATGCAAGG

GGTGGAGGTTTTCAATGTTTTCCAAAGGTAAGGAAAATAATGACAGGCCTAAAGAATGAG

TCTCCAAAGAAGACAGTGAAAGTGAAGAAACTCCCAAGGAAACCACAAATGATGAGCTAG

>MS.gene009845.t1

ATGCATCAAACCATTGATGTTGAGGGTGAACAAAATCTCGTAATTGATCGATATAACATC

AAAGCTATTTTGCTCGATATGATAGTGGCAGCAATTGATACATCTGCTACCTCGATTGAA

TGGGCTTTATCTGAACTTTTAAGGCATCCAAGAGTGATGAAAAATCTTCAAGATGAAATA

CAAAACGAAGTAGGAAATAAAAGAATGGTTGAAGAGAAGGATTTGAAGAAGTTGAATTAC

TTAGATATGGTGGATGATGAAACCTTAAGACTGTATCCTGTTGCACCCTTACTAGTCCCT

CGTGAATGTAGAGAGAACATAACAATTGATGGTTATTTTATAAAGGAAAAGACGAGAATT

GTGGTAAATGCATGGGCAATAGGGAGAGATCCTAATGTCTGGTCAGAAAATGCTGAAGAA

TTTTACCCAGAGAGATTTATTGAAAAGAAAATGAATTATCAAGGACAAGAGTTTGAAAGT

TTACCATTTGGTTCTGGTCGTAGGCGTTGCCCTGGAATTCAATTGGGTTTAACTACTGTT

AAGTTGGTTATAGCTCAATCTGTGCATTGCTTTAACTGGGAACTTTCACATAATACTAGT

CCTTCCAATTTGAATATGGAGGAGAAATTTGGACTCACTATACCAAGAGCCAGGGACGGA

CCCACATAG

>MS.gene009856.t1

ATGTCCTTACAGTTAGGTCAAGTTCCAACTATTGTCATTTCATCTTCAAAAGCAGCAGAA

CCATTCCTCAAAACTCATGACATTAATTTTGCAAGTCGACCAAAGATTCAAGGATATGAA

CTCTTGTCTTATGGTTCCAAAGGGATGGTTTTTTCTGAGTATGGTCCTTATTGGCGTAGT

GTGAGGAAACTATGCACTTTAAAACTTCTTAGTGCTTCAAAAGTTGAGATGTTTGGTCCT

ATTAGGAAGGAGGAGTTGGGTGTTTTGGTTAACACTTTGAAGAAAGCGTCTTTGGTAGGT

GAGGTTGTGAATATAAGTGAGGTTGTAGAGAATCTTATTGAGAATATTGTGTATAAGATG

ATATTGGGTAGGGGTAAGTATGAGCAATTTGACTTGAAGAAGTCTGTTCGAGAAGCAATG

ACTTTGACCGGAGCTTTTAATCTAGCTGATTATGTTCCTTGGCTAGGTTCATTTGATCTT

CAGGGATTAACACGAGCATGCAAGAAAACGAGTAAAGCACTTGATGAGGTGCTGGAAGTG

ATAATAATAGAACATGAAGAAACTACTAATGTAGATAAAACTCTTCATGAAGACTTTGTT

GACATACTTCTTTCATTTATGCACCAAACCATTGATCTAGGAAACGAAGAAAATCATGCT

ATTGATCGAACTAACATCAAGGCTATTTTACTAGACGTGCTTGTGGCAGCAATTGATACA

TCTGCGACCGCGATTGAATGGGCTATGTCTGAACTTCTAAGGCATCCAAGAGTGATGAAA

AATCTTCAAGATGAGATACAAAATGAAGTAGGAAATAAAAGAATGGTTGAAGAGAAAGAT

TTGAGCAAGTTAAGCTACTTAGATATGGTTGTAGATGAGACCTTACGACTTTACCCTGTC

GGACCCTTATTAGTCCCTCGTGAATGTAGAGAGAGCATAACAATTGATGGATATTTTATA

AAGGAAAAGTCACGTGTTATAGTAAATGCATGGGCTATAGGGAGAGATTCCAATGTTTGG

TCAGAAAATGCTGATGAATTTTATCCTGAGAGGTTTATTGACAAGAAAATGAATTATCAA

GGACATGAGCTTGAATCTATACCATTTGGTTCGGGGCGCAGACGTTGTCCTGGAATTCAG

TTGGGTTTAGTTACTATCAGATTGGTTATTGCTCAATTGGTGCACTGCTTTAACTGGGAA

CTTCCATATAATATTAGTCCTTCCAATTTGAATATGGAGGAGAAATTTGGACTCACTATA

CCAAGAGCTCAACACTTGCATTCAATACCGAGTTATCGTTTGGCAGGTGATGCCAAGCAT

GGATAG

>MS.gene009934.t1

ATGGGACACGGTACATTCATCTTTCATTCTAAAAAAGGATTCGCCGATGATGCTTACGAT

GACGACGAAGGAAAAACAAAGGATTTTAGCAAATTAGAGTTGAAACCGGATCACACAAAT

CGTCCTCTTTGGGCTTGTGACAATGGCCGCATTTTCCTTGAGACTTTCTCTCCTTTGTAC

AAACAAGCTTATGATTTTCTCATTGCCATCGCCGAGCCTGTTTGCAGGCCGGAGTCCATG

CACGAATACAATCTTACACCACATTCGCTGTATGCTGCGGTTTCGGTTGGTCTAGAAACT

GAAACAATTATAGCAGTTTTGAATAAGTTGTCTAAGACCAAGCTTCCTAAAGAGATGATT

AGGTTTATCCATGATTCTACTGCAAATTATGGGAAAGTCAAACTCGTTCTTAAGAAGAAT

CGTTACTATGTGGAATCTCCATTTCCAGAGGTATTGAAGACATTGCTCAGGGATGATGCT

ATATCGCGAGCAAGGATCACTTCTGAGGGTACTCATGGAGATGGATTTACAATCAGCAAA

GCACTAGGTGAGATCGAAGGCACTCATGACGAGTTGCTAAATCAAGCAGAAGTAGCGGCT

GTTGCAGAAGAGAAAGAATCTCATGCATTCGAAATTGATCCTTCTCAGGTTGAAAATGTA

AAGCAACGTTGTTTGCCAAATGCCTTAAATTATCCTATGTTGGAGGAGTATGACTTCAGA

AATGATACAGTGAACCCAGACCTCGAGATGGAACTAAAGCCCCAAGCACAACCAAGGCCA

TATCAAGAGAAGAGCCTTAGTAAAATGTTTGGAAATGGTAGAGCAAGATCCGGTATCATA

GTTCTACCTTGTGGTGCCGGAAAGTCCCTAGTTGGTGTTTCAGCAGCTTCCCGGATAAAA

AAGAGCTGTCTTTGTTTGGCAACAAATGCTGTCTCTGTGGATCAGTGGGCTTTTCAGTTT

AAACTATGGTCAACTATCCGAGATGAACAAATATGTCGTTTCACATCTGACAGCAAAGAG

AGATTCCGTGGTAACGCTGGAGTTGTTGTGACAACATATAATATGGTTGCCTTTGGGGGT

AAACGGTCTGAAGAATCTGAAAAGATCATTGAAGAAATAAGAAACAGAGAATGGGGATTG

CTGCTTATGGACGAGGTGCATGTGGTTCCTGCCCATATGTTTCGAAAAGTCATTAGCATC

ACTAAATCTCACTGCAAACTTGGGCTCACAGCTACACTTGTGAGAGAGGATGAACGTATT

ACAGATCTGAACTTCCTAATTGGTCCCAAACTGTATGAGGCAAATTGGTTGGATCTTGTA

AAAGGTGGATTTATTGCAAATGTACAGTGTGCTGAAGTATGGTGTCCAATGACAAAAGAA

TTTTTCGCTGAGTATTTAAAGAAAGAGAACTCCAAGAAGAGACAGGCCCTTTATGTGATG

AATCCAAATAAATTCCGAGCATGTGAGTTTCTCATAAATTACCATGAAAGGGCACGTGGT

GATAAAATTATAGTGTTTGCTGATAATCTTTTTGCTCTCACCGAGTATGCCATGAAACTC

CGCAAACCAATGATATATGGTGCTACAAGCCATGTTGAGAGGACAAAAATTCTACAGGCA

TTCAAAACCAGCAAAGATGTAAATACCATTTTTCTTTCCAAGGTTGGTGACAACTCAATC

GATATTCCTGAAGCAAATGTTATAATTCAGATCTCTTCACATGCTGGTTCAAGGCGTCAA

GAAGCACAGCGTCTGGGTCGTATTCTTAGGGCAAAGGGAAAGCTTCAGGATAGGATGGCT

GGTGGCAAAGAGGAATATAATGCATTTTTTTATTCCCTTGTGTCGACCGATACCCAGGAG

ATGTATTACTCAACTAAGAGGCAGCAATTTTTAATTGATCAGGGTTACAGCTTTAAGGTA

ATTACAAGCTTGCCTCCATCTGATGAAGGATCTCGCTTGCACTATCATCATCTCGATGAT

CAACTGGCACTACTCGGAAAGGTATTGAATGCTGGTGATGATGCAGTTGGATTAGAGCAA

CTAGAAGATGATGCAGATGAATTAGCTCTTAAAAGTGCTCGTCGTTCTCAAGGATCAATG

AGTGCAATGTCAGGTGCAAAAGGGATGGTTTATATGGAGTACAGTACCGGGCGTAACAAA

GGCCACAGATCAAGAGCAAGCCGAAAGATCCAGCAAAGAGACACCACTTATTTAGAAAAC

GATTTGGCTCAACCTAAACCCTCATGTGTGATTAAAAAGGAACCATGCAGTGGCGTCTGT

TCTTATACTCAAGTGTATCCTGGTGACCACACCACACATGGTTGA

>MS.gene009912.t1

ATGATATCGGGTATCAATGTGGAGGATGAACGTTTACCAAGTATGGATCTAGTGGCCAGA

TCACAATTGGCACAACTATCTTGGATGCTGTTAACTGTTAAGGAAGCTGGTGGACATGAT

ACTGAAGTTATATATGAGCAGTGCCCATCAAAAGAATTCATAGAGTGTAATGAATTTTCT

TTTGCCATTGTTGCTATTGGTGAAGCCCCCTATGCTGAGTGTGGAGGTGACAATAAAGAA

CTTGTAATCCCATTTAATGGAGATGGAATTGTAGACATAGTTTCTGATAAAATCCCAACA

CTTGTGATTCTGGTATCAGGAAGACCTTTGGTCTTGGAACAAAGTATGTTGGAAAAGACA

GAAGCTCTTGTTGCAGCATGGTTGCCCAGATTAGAAGAGTTCATCAGCTTAATCAACCTG

TTGAGGGAGAGAATTCATGTGATGGCCCTTTATTCCCTCTTGATTATGGGCTAG

>MS.gene009946.t1

ATGGTGGTCCCATCTATAATTTGTGCAAGGATGAAGAGAAAGTTAGCATCATTACAAAGG

GGTAATGCAAAAAGACCCATGGCCAAAGGTCTTGATGTTAGTAATACTTATGGGATCAGT

GAGAGACAACTGCTATCCAATCCACTTCTTAGAACCCCTCTTTCACCATTGATGAATGTG

GTCAATCAAGGAAACTTAGATACAACACATAATTACGGTGGAAATTCAATGGATTCAACA

TCAAGTGTTTCACCAACTATTTCTTCAGAAATACAAACATCAAGAGGGCTATCCATGCAA

CAATGTGACTCGAAAGAGGCAAGGATGAAGAGAAAACATTCATTGTTACAAAGAGGATGC

ACAGAAAAACTTATTGATAAAGTTGTACATTCTTCAGAATCTCAATCAATAAATATAGCG

AAGAAAGGTCAAATGACTCGAAGTCAAAGGAGAAGACATTCCAAAGGACTCCCTATTGCG

CGTTTGGATTTTGAAACTGTTTTTGTCGAAGGCGGCTCATCATCGAACGATACCTACGAT

GGGGGAGATAAAGAAAACATAAATTTGGATGACTTCAAAAATGGTCTATACAGCGATGCT

GAGATCAAAGGACCTCTTTATTTTGGATATCCAGACTTCACATGCAAATGGTGCAAAGCT

AAACTTTGGATTGAAGAGAGGGCAATAAAGTCTAGACCTCCGAACAATGATTCCGAATTT

TCGTTATGCTGTCAAAAGGGATATGTTGATATCCCTTATGTAGAGGAACCTCCAAAACTA

CTGTTATCATTGCTTGATGGAAGTGATCCTCGAAGCAAGCATTACCTAGAGAACATCAGA

GCATATAACAGTATGTTTGCCTTCACATCCCTTGGAGGCAAAGTGTTTTCAAAGCTCAAC

AATGGAAGAGGGCCTCCTCAATTTATATTAAGTGGACAGAATTATCATAGGATTGGAAGT

CTATTGCCTGAGCCTGGAAGTACTCCAAAGTTTGCCCAACTATACATCTACGACACTCAA

AACGAAATTCAAAATAGAACAAAAGTTTTTGATTCAAATGATGGTGAGGGTGGATTTGAT

CTTTCATTGGTTGAAGATTTGAAAAAAATGTTGGACGAGTTTAACCCTTTGTGCAAATCT

TTTAGAAAGATCCGTGATCTGGTTGAAGAGGGTTCACCTCCAAAGATGGCATTGAGGTTG

TTCAGGAAAAGGGAAAAGGACTCAAGAATGCACAATCTACCTACTGTTGATGAAGTTGCT

GGGTTGATAATAGGTGATTATGATGAGACGGAAGAAGGAAGAGATATTATAGTGGATGAT

GTAGGCAGAGGATTGAGAAGGATACATGAGACACATCCATTATACATGCCACTTCAGTAT

CCTTTAGTGTTTCCTTGCGGTGATTATGGCTATGAGGAGGATATACCTTACAGGGAATTT

GAAGACGAAGAAAAAAAGTCAAGAGGTTCAAGGGAAAGAGTTGCAATTAGAGAATACATT

GCTTTCAGAATTCAAGATAGGGATTGCGAGTTTGGAAACATTGTGTATGCAAGAAGACTG

ATGCAGCAATTTTCTGTTGATTGTTACACGATGATTGAAGCACAAAGACTATCTTATATT

CGGAATAACCAGGGAAATATTCGTTCCGATTTGTTGAGTGGATTACAAGAAGCAGTTGAA

ATGGGAGACAATGATCCTCGAAAAGCCGGACAACGTGTTATACTTCCAGATTCATTTACA

GGTTCACCAAGGTACATGTTCAATAATTGCCAAGATGCAATGGCAATTTGTAAGAGATAC

GGCTACCCGGATCTTTTCATCACAATAACTTGCAACTCAAATTGGAAGGAAATCAAGAAC

TTTGTTTCAGATAGAGGACTGAGTGCATCTGACAGACCTGATATCATTTCTAGAGTGTTC

AAGATGAAGCTGGACCAAATGATGACCGATTTTAAGAAAGATCACATTTTTGGTGCTGTG

ACTGCTGGTATGTATACCGTTGAGTTTCAAAAGAGAGGGCTCCCACATGCACACATTTTG

TTGTGGTTGGAAGGCAATAACAAACTGCACACAACTAAGGAAATTGATATGGTTATATCA

GCAGAGATTCCTCATCCACAGCTATATCCAAAGTTGCATTTGGCTGTTTCATCATTCATG

ATGCATGGACCATGCGGAAGGGCAAATTTGAATTCCGCATGCATGAATGGCAAGAGGTGC

ACGAAATACTTTCCAAAGAAGTTCGTAGATTCCACTTCAATAGATGAGGATGGTTTTCCG

GTATATAAAAGGAGGGACAATGGGGTTTGTGTGGAAAAAAATGGAACTCAATTGGACAAT

AGGTATGTTGTTCCTTACAATCCATTCCTCTTGATGAGATACCAAGCACATGTTAACACT

GAGTATTGCAATAAGTCTAATGCAATAAAGTATCTTTTCAAATACGTTAACAAAGGTCCT

GACAGAGCAAACCTCCAAATTAAGAAGAAAGCAGGAGATAAAGATGAGGAGGGCCCAATT

GATGAAATTAAGCGTTACTATGATTGTCGCTATGTCTCTCCATGTGAAGCAACTTGGAGA

ATATTTATGTTTGATATCCATGAGAAATGGCCTGCTGTTATGAGATTAGCTTTACATCTT

GAGGGTCAACAATGTGTTAGGTTCAAGGAAAATCAAAAGCTTCCGAATGTTGTCAGATAT

CATCAATCAGTCCCTACCATGTTTCTTGCTTGGTTTGTTGCTAATCAAAATTACAGTGAG

GGTAGAGATCTTACTTATGCCGAATTTCCATCAAAGTTTACTTATGTGCCTGACAAAAGG

ATATGGCATCCTAGGAAAAATGGTTTTCAGATCGGGAGGCTGTCTTACATTCCTGTAGGC

TCAGGTGAACTTTACTATATGCGGATTCTTTTGACCTTTCAAAAGGGTTGTAAGGGGTTT

GATTGTATTAAAACTGTGGATGGCAAGCTATATGGTAGTTTTCAAGATGCATGTTATGCT

CTTGGTTTATTGTCCGATGACAAAGAGTTTATTGATGGAATTTTTGAGGCTTCTAGATCA

CAGAGTGGGCAAATGCTTCGTGTTTTGTTTGTTAGACTTTTAATCATGAGTACAATGCAC

AAGCCTGATAATGTATGGAAAGCATGTTGGAAGTTATTGGCAGATGGAATTCTCTATGCT

CGACGACGGATCCTCAATATTCCAGGTATTTTTACCCCTTTTCAACTGTTATCATGA

>MS.gene009847.t1

ATGTCCTTACAACTTGGTCAAGTTCCAACAATTGTTATTTCATCTTCAAAAGCTGCAGAG

TCATTCCTCAAAACCCATGACATCGTTTTCGCTAGCCGACCGAAGATTCAAGGATCTGAC

CTCATGTCCTATGGTTCTAAAGGGATGCCTTTTTCTGAGTATGGTCCTTATTGGCGCAAT

ATGAGGAAATTTTGCACTTTGAAACTTCTTAGTGCTTCAAAAGTTGAGAAGTCGGGTCAT

TTTAGAAAAGAGGAGTTGGGTGTTTTGGTTAACACTTTAAAGAAGGCTTCTTTGGTAGGT

GAGGTTGTGAATGTAAGTGAGATTGTAGAGAATCTTATTGAATATATTGTGTATAAGATG

ATATTGGGTAGGGGTAAGTATGATCAATTTGACTTGAAGAAGATGATTAAAGATGGATTA

ACTTTGATGGGAGCTTTTAATCTGGCTGATTATGTTCCTTGGCTAGGCGTTTTTGACCTT

CAGGGATTAACACAAGCTTGCAAAAAAACCAGTAAAGCTCTTGATGAAGTTTTGGAGATG

ATAATAAAAGAGCATGAACAAACTACTAATACATACAATCCTAAAGATTTTGTAGACACA

CTTCTTTCAATTATGCACCAAACCATTGATGTTGAGGGTGAACAAGATGTCGTCATTGAT

CGAACGATCATCAAGGCTATTTTGCTCGACATGATAGGGGCATCAATTGATACATCTGCA

AACGTGATTGAGTGGGCTTTATCTAAACTTTTAAGGCATCCAAGAGTGATGCAAATTCTT

CAAGTGGAAATACAAAATGAAGTAGGAAATAAGAGAATGGTTGAAGAAAACGATTTGAAG

AATTTTAATTACCTAGATATGGTGGTTGATGAAACCTTAAGACTTTATCCTGTTGCACCT

TTATTAATCCCTCGTGAATGTAGAGAGAACATAACAGTTGATGGTTATTTTATAAAAGAA

AAGACACGAGTTATAGTAAATGCATGGGCAATTGGGAGAGATCCTAATGTTTGGTCAGAA

AATGCTGAAGAATTTTACCCAGAGAGATTTATTGAGAAAAAAATGAATTATCTCGGACAA

GAGTTTGAATCTATACCATTTGGTTCTGGTCGTAGACGTTGTCCTGGAATTCAATTGGGT

TTGATTACGGTAAAGTTGGTTATAGCTCAATTGGTGCACTGTTTTGATTGGGAACTTCCA

TATGATATTAGTCCTTCCAATATGAATATGGAGGAGAAATTTGGACTCACTATACCAAGA

GCTCAGCATTTGCACGCAATACCAAGTTATCGTTTGGATGATGCTAAGCAAGAATAG

>MS.gene009909.t1

ATGGGACATGAAACTGAAGTTATATATGAGAAGTGTCCATCAACAGAATTCATAGAATGT

AATGAATTTTCTTTTGCCATTGTTGCTATTGGTGAAGCCCCCTATGCTGAGTGTGGAGGA

AGACCTTTGGTCTTGGAACAAAGTTTGTTGGAAAAAGTAGAAGCTCTTGTAGCAGCATGG

TTGCCTGGTAGTGAAGGAAATGGAATCACAGATGTTATCTTTGGGGATCATGACTTCAAG

GGTAAACTACCAATGAATTGGTTCAGAAGAATTGAACAGCTTGATCAACCTGATAAGGGA

GTGAATTCATGTGATGACCCCTTATTCCCTCTTGGTTATGGGCTAGCTTATAATAAGGAG

AAGTCACATGAATAA

>MS.gene009913.t1

ATGTCCGCTTTAGATTATCTCTCACCTTCAACACACGCTACAATCTTTCTTCACTCTCAT

CACTTTCGAAACGCACGTCGTTTCGTTATTCCCAATTCACCTTCAATTAGGGTTTTAAAA

GATTCAATTCTTCTCAACAATTTCGGAAAATTCGAATTATGGAAAGGATTCAACTCTAAA

TTGAGCAATTTCGATGGACTTAGAACTGCTGCTAGCGGTGGTGGTCAGGAGAGTGATTCT

GGGGAGAAGAGTGAAGAGGGGAAAGGGGTTGAAGTTGAAGTTAAACCGGGTTCAGGTGGT

TCGAACCGGAGGAAGGAGAAGCAAGGGAAAGGTGGATGGTGGAGGTGGAAATGGCAACCG

ATGTTGAAAGCTCAAGAAGTTGGGGTTTTGTTACTTCAATTAGGGATTGTGATTTTTGTT

ATGAGGTTGCTTAGACCTGGGATTCCGTTACCGGGTTCCGAATCGCGAGCTTCAACGGTT

TTTGTGAGTGTTCCTTATAGTGATTTTTTGAGTAAGATTAATGGGGATCAGGTTTTGAAG

GTGGAGGTTGATGGTGTTCATATTATGTTTAAGTTGAAGACTGATTTGGAGGGTGGTGAG

GTTGCTGGTACTGGTAGTAGTGGTAAGTTGCAGCAGGAATCGGAGGCTTTGGTTAAGAGT

GTGGCACCGACGAAGAGGATTGTTTATACAACGACTAGGCCTAGTGATATTAGAACTCCT

TATGAAAAGATGTTGGAGAATGAAGTGGAGTTTGGATCACCCGATAGACGTTCTGGCGGA

TTTTTTAACTCGGCTTTGATAGCCATGTTTTATGTTGCTTTATTGGCGGGGCTTCTCCAT

CGATTCCCTGTAAGCTTTTCTCAGAATACTCCTGGTCAGTTTAGGAGCCGCAAATCAGGA

ATGTCGGCTGGTACAAAATCATCCGAAAAAGGTGAAACAATCACTTTTGCTGATGTTGCT

GGTGTTGACGAGGCTAAAGAAGAGCTAGAAGAGATTGTGGAATTTCTTCGAAATCCGGAT

AAATATGTACGACTTGGAGCTCGTCCTCCTCGAGGTGTTCTCTTGGTAGGTCTTCCGGGA

ACAGGCAAGACTTTACTAGCAAAGGCTGTGGCTGGAGAAGCTGACGTGCCATTTATAAGC

TGTTCTGCTAGTGAGTTTGTTGAGTTGTATGTTGGTATGGGTGCTTCCCGGGTGAGAGAT

CTCTTTGCAAGGGCAAAGAAAGAAGCACCATCCATAATATTTATTGATGAGATAGATGCC

GTGGCTAAAAGCCGTGATGGTAAATTTCGTATTGTAAGCAATGATGAACGAGAACAGACC

TTGAACCAGTTGCTTACTGAGATGGATGGGTTTGACAGCAATTCAGCAGTGATTGTTCTT

GGGGCAACTAATCGTGCTGATGTCTTAGATCCTGCGCTTCGCCGACCAGGAAGATTCGAT

CGAGTAGTTATGGTGGAAACACCGGATAGGATCGGAAGAGAATCCATCCTGAAAGTTCAT

GTTTCTAAGAAAGAACTTCCTCTTGCTAAGGATGTTTACATTGGTGACATTGCTTCTATG

ACTACCGGATTCACAGGGGCGGATCTTGCAAACCTAGTAAATGAGGCTGCTTTATTGGCG

GGAAGAAAGAACAAAGTTGTTGTGGAGAAAATTGATTTCATTGAAGCTGTGGAAAGGTCA

ATAGCTGGCATAGAAAAGAAGACTGCCAAGTTGCAAGGAAGTGAGAAGGGTGTAGTTGCA

CGACATGAAGCTGGTCATGCTGTAGTAGGCACTGCAGTTGCAAATCTTCTATCTGGACAG

CCACGTGTTCAGAAACTAAGCATATTGCCTAGGTCAGGAGGGGCCTTGGGCTTTGCTTAT

ATTCCTCCAACAAATGAGGACAGATACTTGCTTTTCATTGATGAATTGCGCGGCCGCCTG

GTGACTCTTCTTGGAGGACGTGCAGCAGAAGAAGTTGTATATTGTGGTCGAGTTTCAACA

GGTGCAATTGATGACATACGTCGAGCTACTGACATGGCATACAAAGCCATTGCTGAATAT

GGTCTTAGTCAGACAATAGGCCCTGTGTCAATTTCCACTCTTTCTAATGGCGGATCTGAT

GAGTCCGGGGGATCAGTTCCTTGGGGAAGGGATCAGGGACAACTTGTTGATCTTGTTCAA

AAAGAAGTGAAAGCATTGCTTCAGTCTGCACTGGAAGTAGCACTTTCCATTGTTCGAGCT

AATCCTACTGTTGTAGAGGGTCTTGGTGCTCAATTGGAAGAAGAAGAGAAAGTAGAGGGT

GAAGAGTTACAGAAGTGGTTAAGATTGGTGGTTGCACCAACAGAACTTGCAATTTTTATG

GAAGGCAAGCAACAAATCCTTCTCCCATTGCAGACCGGTTCCTGA

>MS.gene009931.t1

ATGGACACGAAATTAGAAGCGAATCTTAACGCAACTGTTTCTACTGTTAATGCGTATCCT

GAATCGCGTGTGGTTAATGCTATTAATGGTGATAGATCAAAAGGTTCTGTTCCGTTTGAT

GTACAGATTGTTGCTGAATCTTCATTTCGTTCGAGTTCATGGAGGTTTAGGACGAGGATT

TTGAAGGCTTTGTGCAGGAAAATTGATGTTGGGATTTCGTCAAATTCGACTACCGGCGAA

TTGATTGGTGGAAATAGAGAATGTCAGGTCTGGACTTGA

>MS.gene009893.t1

ATGGATGAAGACGAAGAAGACCCATATCTACCTATTGAATTAATCATTCAAATCCTACTA

AGGTTACCAGTCAAGTCTCTTATACGCTTCAAGTGTGTTTGTAAGTCTTGGTTTTCTCTC

ATATCTCAAAATCACTTTGCAAATTCTCATTTTCAACTTACCGCCAACGCACACACTCCT

AGAATTTTGTTCATAAATCCAGATCTTGAATCTCTATCTATAGATTTAGAAGCATCTCTT

AGCAACGATAGTGCTTCTTATTCACCGGACATTAATTTTTTATTTGAGGAATATGATTAT

GATAGTTCTTCTTCTTCGTTGGACATGGATTTTTCATCTTCTCACCCATATTTTTTAGAT

CTTGATGTTAGAGGTGAATGTAGAGGCTTTATACTTTTCTCCGGTTATTCAAGCCTTTAT

CTATGGAATCCATCCACTGGAGTTCACAGACAAATACCCTTTACTACTGTTCATGATTCC

GATTTAGAAGCAAATTATTTCTATGGTTTTGGGTATGATGAGTCTACTGATGATTACCTG

GTTCTTTCAATGTGCTATGATCCGAGTGCGGGTGGTTTGTTATCTCACTTTGGGCTTTTC

TCATTGAGAGCTAATACGTGGAAGGAAATTGAGGGTGGTGACAATTTGCATTATTCACAG

TCGTGTATGTATTCTAGAGTAGATACGCTCTTAAATGGTGTTATTCACTGGTTGGCTTTT

CGTAATGATAGATCAACGAATGTTATTGTTGGATTTCATTTAACAGAAAGGAAACTTATA

GAGTTGCCTTTGCCTAATGGTCCTAGAGTGTATGATTTATGGCTATTTAGAGGATGTCTC

GGTCTATCTGATATGTACATTGATAATGGTATAGTTGAAATATGGGTGATGAAAAAATAC

AACGTGCAATCTTCTTGGACTAAGACTCTTGTTCTTTCTTTTGGTGGCATTCCCATGCAC

TACTTTTGCCCAAAATATTGTACAAAAAGTGATGATATCGTTGGAACTGACGATAGTGTA

TTGGCCAAGTATAATGATAAAGGACAGCTGCTAGAGCATCACTCCTACTCGGACCATGAT

GATGGATCCCTAGTGGTAATGTATACAGAATCTTTGCTTTCACTTCCTGGTGGTGACACT

GATCAGGCTTAA

>MS.gene009883.t1

ATGGAGATGATGAAGAAAACAGAGCTATATCTTCCTCTTGAGTTAATTTTTCAAATCCTT

CTAATGTTACCTGTGAAGTCTCTTATTTGCTTCAAATGCGTTTGTAAGTCTTGGTTTTCT

ATTATATCTGATACCAATTTCGCAAATTCACATTTTCAACTTACCGCCAAAACACACACT

CCTAGAATTCTCTTGATAACTCCAAATTTGGAATCTCTATCTATTGATTTTGAAACATCA

CTTTACGATGATAGTGCTTCCTATTCATCGAATATTAGTTTTTTGCTTCCACAGTCTTTT

ACAGAACTTGATATCAAAGGTTCATGCAGAGGGTTTATACTTTTGAGCTGTGGTTCATGT

CTTTGTCTATGGAATCCATCCACTGGAGTTCACAAATTTATACCAAATTCTCTTATTGAT

TGCAATTTAGATGCATATCATTTATATGGTTTTGGGTATGACGAGTCAAGGGATGATTAC

TTGGTGCTTTCCATGTCATATGATCCCAATGATTATGATAAGTTAACTCGCTTAGGGCTT

TTCTCATTGAGAGCTAATGCGTGGAACGAAATTGAGGGTGATAATTGTTTCCCTTATTGC

CTGGCCCGTGAAAATTCCAAAGTAGAGCCGTTCTTAAATGGGGCTATTCACTTCTTAAAT

GGGGCTATTCACTGGTTGGCTCTTTCTTATGATATATTAACGAATGTTATTCTTGCCTAT

CATTTAATGCAAAGGGAGCTTCTAGATTTGCCTTTGCCAGCTGATATAACATCTGATTCT

AGTAAAGTGTATGATTTATGGGTATTTAGAGGATGTCTCAGTCTATGGGATATGGCACAT

GATAATGGTACGGTTGAAATATGGGTGATGGAAAAATACAACGTGAAATCTTCTTGGACT

AAGACTCTTGTTTTGTCTTTTGATGGAATTCCCACTCACTATTTTTGCCCGACATACGGT

ACAAAAAGTGGTGATATCGTTGGAACAGATGCTGGTAATGTATTGGCCAAGTATACTGAC

AAAGGACAGCTGCTAGAGCATCACTCCTTCTGTGGCAGTGAGTATGGATCCCTAGTGGTT

ATGTATACAGAATCTCTGCTCTCAATTCCTGGTGGTGACGGTGGTCAACCTAAGAAGATG

ATACGAAGAAGAAGAAGGAGGTCTTGA

>MS.gene009943.t1

ATGAGTGGTAGTGGGTTTAGGGCTTCAATTCCAAACAGTGTGAAGAAAACGATCCAGAAT

ATCAAAGAGATCACAGGGAATCACAGTGATGAAGATATCTATGCAATGCTTAAAGAATGC

TCTATGGATCCCAATGAAACTACTCAGAAGCTTCTCCTTCAGGACACATTCCATGAGGTC

AAAAGAAAGAAGGACAGAAAAAAGGAGATTCTCAACAACAGAGAACATGTGGAACCACGT

GGGAGACCTGGCACTCACGGACGGGGGCCTAGGGGTGGTCGGGGAAATTTTTCACCTCAT

GATACGACTGGAAGAAAAACTTCTGTGACAGGGAAAGATAGTGGAGCTCTTCTACCCTCT

GAGAAAGTTGCGCCACATTCATCAGCTTCTCAAGAGATAGTATCTAAAGGGAAAAGTTCT

GGAACAAGCTCTGCGCCCATTATTGCTAATGGTCCCACAAATGTGGCTTCTGGAACTGTT

AGTGGTGTGGGTCCTTCCTCTTCATCTGCTGGAAATGGAGACATAATGGTTCAATCTTCT

GGCAATAACAATAACAATGATGTGCATAGTGCTTCACCTTCCAATAAATCAAATCAAGTT

GCAACAGATGCTTCTGGAACTGGGCCGGCATCGTCATCGGCTGTTCACTTCTCCTCTTCT

GATCCAGTATTGGTTCCATCTGATAATTCATGGTTTCCCGGTGCTGCTGGTGCAATTAGA

CGCGAAGTGGGAAGTCAATACTCTCTTGGGGAATCAAATGCTGTTAATTCGACCAAGAAC

AAATTAACTGCTGCTTCTGAGACTGGCAGCTCTGCTGTGCAAGGAAAGATTCAAGATAAA

TCCCAGGGAGTGGCAAAGAATCATGGCAATGAGATACCCTCTCCATCAACACCAGTAACT

CATGGAAGCCCTTCAGTTAGTCGCCCTTCCTCTAATTATAATAACCGGTCACAACAGCAA

GTTGGCTCTCAGAAAGTTGGTTCTAATAAGGAATGGAAGCCGAAGCCAACCAATACATAT

AATCAGAATTCTGGACCTGCTATTGTATCAGAAGCTCCTCCTGTTTCAGCTGAAGTGACT

AGGCAGTTACAGTCCGTGTCAAGTGCCCTTGACACTGAAGAAGCAACTTCAAAACTGCAG

AAGAAGCTGGAGGATTTTCATATTCCACAGCGTCAACATGTTATACTTCCAAATCATATC

ATAGTACCTGATTCTGAGAAGAACAAGTTTTGCTTTGGAAGTTTGGGAGTCAATTTTGGA

GTTAATACAACAACCGATGTTAGTGGCCCAGATAGTGAAAAGAGCTCCACACCATTTTCT

GAAACATCTCAGGATATTGAAGAAACTGTGGAGGAGCAGCACCCGAGTCAAAATGGTGTG

GTTACGTCTGAGGTGGGAGACTATCCTGACCACCCACAATCACCCAGCAACGTACCTGTG

AATTTAGAATCGAGTGAGGTTGATGGGTCATCCAGTGCTACTCAGGAGTTTAATGAGTCT

AAGCAAGACACTGCTTTGCCGCCCGAAGGTCATCAATACCCGGGCATGCATGTTTCTCCA

AACTATGGTTTTGGTTTTGTGCCCCCAATGTCGGGTACTCAGCTCACATCATTTGACAAT

TCTGAGTCTCAGACACGTGATGTTTCTCGGCTTCCAAGCTTTATTGTCCAACCACAAGTG

GATCCTAGTTATTATGCTCAATTTTACCGGCCAGGTGCTGATAGTGATGGTCGTGTTTCT

CCGTTTGCTTCTGCTGGGGCTACCACCAAGTACAACAGCAATGTTGCGGTGCTGCCTACA

CCGAATTCTCAAACTCCTCAAGAGGGAGGTATTTTGTCCAACGCAGGTCAAACACCAATT

GCGACTCAAGCAGCAGGACTAATGCAAAGCTCAATACCTGTTACGCAGCAGCCCCTCCCT

GTCTATCGGCCGGGGGTTCATTTATCTCATTACCCTCCAAACTACATTCCTTATGGTCAC

TATTTTTCACCATTTTATGTCCAACCTCCAGCAATGCACCAATATTTGGGTAATGGTGCA

TTTCCTCAACAACCTCAGGCCAGCACTGTATATCCACCTCCCCCAGCTGTGGCAGCCCCG

GGAATGAAATATCCTCTTCCACCATTCAAACCTGGAACAAATGCAGCAAACCCTACACAC

CTTGTTATGCCAAACACTTTTGGAATATATGGTTCCTCTCCAGCTGGTTATAATCATAAT

TCTGCAACAACTGCCGGGAATTCAGCCTCTAACGAGGATCTTGGTTCTTCTCAATTCAAG

GAAAATAATGTATACATCAGTGGACAGCAGAGCGAAGGTTCAGCAGTGTGGGTGGCTGCA

CCTGGCCGAGACATGAATAACCTGCCCACTAGTTCATTCTACAACCTTCCACCTCAAGGT

CAGCACATGACTTTCGCCCCAACTCAGGCTGGCCACGGCCCTTTTACAAGCATCTATCAC

CCTGCACAAGCAGTGACAGCTGCAACAGTCCATCCGCTGTTACAGCAATCGCAGACAATG

GCAGGAGCAGTTGATATGGTAGGAACAGGTGGAAATGTTTATCAGCAACCTCAACATGCA

CAGATGAATTGGCCAAGCAATTACTAG

>MS.gene009924.t1

ATGGAGAAGACGAAGGAAATCCTGCAACGGAAGCTAAGCCAACAAGAATCGGAAGCCTTG

TCACGAATCCAATCGTTTCCTGCCATAAGAGCCGGCGATAACTCAAGCTTCTATGAACAT

TTCATTCTCACCGGAATCAAAGTTGAACAAGACAGCACTGGGAAGATGGCTAATGGTGCA

ATTGCAACTCTTGTGGATGAGGTTGGAGGTGCTTTAGTCCATCAAGAGGGTCTCCCCATG

AATGTTTCAGTTGACATGTCCATATCTTTTCTTTCAACTGCTCATGTTAATGATGAATTA

GAAATCACTTCTAGACTGTTAGGAAGGAAAGGAGGTTATTCTGGAACAATTGTTCTCCTA

AAAAATAAAGCTACTGGAGAATTGATAGCAGAAGGCCGGCATTCGCTATTTGGTAGACAT

AACAGCAAAATGTAA

>MS.gene009903.t1

ATGGCACGAACATTTATTACAGATGAAAAAGGGCGTGTTAGGCCTACTTCGTCTACACGT

CGATGTCGCAACAAACAAGTTACTTCATCAAAGAAGGGGCCTGTCGATGACCCACCTCGT

GAAGCCTATGAGCAACATCATGAAGCCAATGAGCCACATCAGGAAGTAGAGGAGCCACAT

CAGGATATAAGAGCTAATGGTGTGGAGATACATGGATATCCCGGTGGTCCCATAGACTGT

TCTATTCTTAAGACATATGGGGACCACGTGGCTAGACGAATTTGGGAAGGAGAGGATCGT

GGGGAGTTGAGGATCTTCAGCAACGGAAAAAAGATGAAGGGAGTTATCATTCATCACGAT

GAGGTTCGACGATTAGTTGAGGGGTCTGGACTCCTACCTCTGTTGAAATGCAGCTATGAA

ATGATTGACAAGGGACTCATTTCTGCTTTCGTTGAGAGGTGGCATCGAGACACCAATAGT

TTTCATCTTCCGGTCGGAGAGATGACCGTTACATTGGATGACGTGTCGTCTTTGTTACAT

ATACCTATCACGGGGGCATTCTTCACTATCAATGTATTTGACAGGGATCAAGCCGCAAGG

GTATTAACCGAACTATTGGGAGTTTCCATACCATTCGCTAGACAAGAGTTTACGATAACA

AACGCATCACAGGTTCGTTACTCTTGGTTGTTAGATCTGTATAATCAACGTTGTGAACAA

CGTCGTTGGGTGGAGGCTGCGAGAGCTTTTATATTATGGCTGGTTGGGTGCACACTATTT

AGTGATAAGAGTGCATTCGCAGTAAATGTTGCATATTTGGAGTGTTTTCGTGACCTCGAT

AGTTGTGGAGGATATGCATGGGGTGTTGCTGCCCTAGCCCACTTGTATGACAATCTATGC

GATGCAAGTCTTCATCACATGAAATCGATTTCTGGTTACCTAACCCTTATACAGGCATGG

GTTTACGAACACTTTCCTATTTTATGCATTGATTGTTGTAGAGTGCGACCAGATTATAAG

GAACAGCTACCTCGATCCATTAAATGGAAGCCGAGGAGAGATAAAGGCCAGGTGCTTCCT

TTCAGGCAGGCTCTTGATAATATTACTGTTGATCAAATTTGTTGGAATCCTTACATTGCA

CACAGAGATTTCCGGCCATTGCAAGATGCATCTTTTTACAGGGGTTGGATTAGGTGGGGT

CTTAAGATGTATGCACATCTTCCTGATAGGGTACTACGACAATACGGGCATGTACAAGGT

ATTCCTTCGTCACCAAACGATGTCACATGTCATTCGACCACTTCAAAGGATGTGGATCTT

ATGTTCACGCAGTATGCCATTCATGTGGTCGATCCCGGTCTCGTTGCAAATGACCCGTCA

GCCTGTGTTGATGGGTATATGGATTGGTTTCGTACGATCTCTCATCCTTACATCATACGA

CGAGATCCAAACCCAATTGTTTCAAGCTCGTCTACAGATGCAACATCATTGCTGAGGGCC

ATGCAGATTGTTAATACGCTTCTCGACAAACAATATATTGCACCTGATGGGATCGCACTT

GCAAATGAGTTGGTGGATTTGTTAACTACAGAGAAAGGTGTTAAAGACCGGTCAAACGAA

GAGCCATACAAGAAGAAAAAGAAAAAAAAGAAGAATTAG

>MS.gene009898.t1

ATGGAGACCCTCACTCTTCTCCTTACCCTAACAGCCTCCCTCTCTGCCTATTTCCTTTGG

TTCCATCTCCTCGCCCGAACCCTAACCGGTCCAAAAGCATGGCCCTTCATCGGTAGCCTT

CCGGCCCTATTCAAAAACCGGAACCGAGTTCATGATTGGATCGCGGAAAATCTCCGAGCA

ACTGGTGGTTCAGCCACATACCAAACATGCATTATCCCCTTTCCTTTCTTGGCTCACAAG

CAAGGGTTCTACACCGTCACATGCCACCCTAAAAACCTCGAACACATCCTTCGAACCCGG

TTCGATAATTACCCGAAAGGGCCTACGTGGCAAACAGCATTTCACGATCTTTTGGGCCAA

GGCATTTTCAACAGCGACGGTGAAACGTGGATAATGCAACGTAAAACCGCTGCCCTCGAA

TTCACGGCACGAACATTGAAACTAGCTATGGCTCGATGGGTGAACCGGTCCATCAAAAAC

CGACTTTGGTGTATTTTGGATAAATCGGTTAAAGATAATGTCTATGTGGATTTACAAGAT

CTTTTGTTAAGATTAACATTTGATAATATTTGTGGACTTACGTTAGGTAAAGACCCTGAA

ACTCTTTCACCGGCGTTACCTGAAAACCCCTTTTCTGTTGCTTTTGACACCGCCACTGAA

GCCACCATGCACAGGTTCCTCTACCCAGGTCTAATATGGAGGTTTCAAAAACTTTTCGGC

ATAGGATCCGAAAAAATGTTAAAACAAAGTCTCCAAATTGTAGAAACATACATGAACAAC

GCCATTTCAGATCGCAAGGAAACCCCTTCCGATGACTTAATGTCTCGTTTCATGAAGAAA

CGAGACATAGATGGAAAACCAATCAACGCAACAGTGCTACAACACATAATCTTAAACTTC

ATCTTGGCCGGAAGGGACACATCATCAGTTGCTCTCAGCTGGTTCTTCTGGCTGGTCATG

AACCATCCGAAAGTTGAGGAGAAAATAATCAAGGAGTTAACCACTGTTCTTGAGGAAACT

CGAGGAGGAGAAAAGCAAAAATGGACAGAGGATCCGCTTGATTTCAGTGAAGCGGATCAA

TTGGTTTATTTGAAAGCGGCATTGGCGGAAACACTGCGTTTGTATCCGTCAGTGCCGCAG

GATATCAAGCAGGCAGTCGTGGACGATGTTTTTCCCGATGGAACAGTTGTTCCAGCGGGT

TCAACGGTTACATATTCGATTTATTCTGTTGGGAGGATGGAGAAAATATGGGGGGAAGAT

TGCTTGGAATTTAAACCGGAAAGGTGGCTTTCGGTTCGGGGTGACCGGTTCGAACCACCT

AAAGAAGGGTTCATGTTTGTGGCTTTTAATGCTGGACCAAGAACTTGTTTGGGAAAGGAT

TTGGCTTACTTGCAAATGAAGTCAGTGGCTGCTGCTGTTCTGTTACGTTACCGGCTATTG

CAGGTTCCCGGTCACGTTGTGGAGCAGAAAATGTCTCTTACTTTGTTTATGAAGAATGGG

CTTAAGGTTTTTTTGCAACCACGTAAGCTTTGA

>MS.gene009858.t1

ATGTCAAATGGTGCCAAAGGGTTGGCTTTTTCTGAAAAGCAAGAGTTGGATGTTTTGGTT

AAATCTTTGATGAAAGCTGCTTTGGTAGGTGAGATTGTGAATGTTAGTGAGGCTGTACAG

AATCTTATAGAGGAAAATGTGTATAAGATGATACTCGGTCGGAGTAAGTGTGAGCAATTT

GACTTGAAGAAGTTGGTTCAAGAAGGAGTGGCTTTGACCGGAGCTTTTAATCTTGTTGAT

TATGTTCCTTGGCTAGGAGCATTTGATATTCAGGGATTAACAAGAGCCTGCAAGAAAACA

AGTAAATCACTTGATGAGGTGTTGGAGATGATAATATCCGAGCATGAACAAACTATTAAT

GTAGACAAAGTTTGTCATGAAGACTTCATAGACATACTACTCTCAATTAAGCACCAAACC

ATAGATCCAAAAAATGAACAGAAACATGTCATTGATCGACCAAACATTAAGGCAATTTTA

CTAGACATGGTTATGGCAGCAATTGAAACATCTGCTACCGCGATTGAATGGGCTTTATCT

GAACTTTTAAGGAATCCAAGAGTGATGAAAAGCCTTCAAAATGAGATACAAAATGAAGTA

GGAAATGAAAGAATGGTTGAAGAGAAAGATTTAGAGAAGTTAAGTTACTTAGATATGGTG

GTTCATGAAACTTTAAGAATTTACCCTGTTGCACCCTTATTACTCCCTCGGGAGTGTAGA

GAAAGCATAACAATTGATGGTTATTTTATAAAGGAAAAGACACGAGTTATAGTAAATGCA

TGGGCTATAGGGAGAGATTCTAATGTTTGGTCAGACAATGTTGAAGAATTCCATCCAGAA

AGATTTATTAACATGAAAATGAATTATGAAGGACATGAGTTTAAATCTATACCGTTTGGT

TCTGGTCGTAGACGTTGTCCGGGAATTCAAATGGGTTTGATCACAATTAAAATGGTTATA

GCTCAATTGGTACATTGCTTTAATTGGGAACTTCCATTTAATATTAGTCCTTCCAATTTG

AATATGGAGGAGAAATTTGGACTCTCTATACCAAGGGCTCAACACTTGCACGCAATACCG

AGTTATCGTTTGGCTTTGGCATGTGACAACAAGCATGATTAG

>MS.gene009852.t1

ATGTTAGGTAAACTTCCACATAGAACACTTCAATCACTCTCCAAAAAATATGGTCCAATC

ATGTCATTACAACTTGGTCAAGTTCCAACTATTATCATTTCATCTTCAAAAGCTGCAGAA

TCATTCCTTAAAACTCATGACATCGTTTTTGCAAGTCGAGCAAAGAGTCAAGGATCAGAG

ATCATGTCATATGGTTCCAAAGGGATGGCTTTTTCTGAGTATGGTCCTTATTGGCGTAGT

GTGAGGAAATTTTGCACTTTGAAACTTTTTAGTGCTTCAAAAGTTGAGATGTTTGGTCCT

ATTAGGAAGGAGAAGTTGGATGTTTTGGTTAAATCTTTGAAGAAAGTTGCTTTGGAAGGT

GAGGTTGTGAATGTAAGTGAGGTTGTAGAGAATCTTATTGAGGATATTGTGTATAAAATG

ATACTGGGTAGAGGTAAGTATGAGCAATTTGACATGAATAAGTTAGTTCTAGAAGCATTG

GCTTTGATGGGAGCTTTTAATCTGGCTGATTATGTACCTTGGCTAGGCGTTTTTGACCTT

CAGGGATTAACACGAGCTTGCAAAAAAACCAGTAAAGCCCTCGATGAGGTTTTGGAGATG

ATAATAACAGAGCATGAACAGACTACTAATATAGACAAAACTCGTAATGAAGACTTTGTA

GACATACTTCTTTCAATTATGCACCAAACCATTGATGTCGAGGGTGAACAAAATCTGGTC

ATTGATCGAACTAACATCAAGGCTATTTTGCTCGACATGATAGTGGCATCAATTCATACA

TCTGCTACCACGATTGAGTGGGCTTTATCTGAACTTCTAAGGCATCCAAGAGTGATGAAA

ATTCTTCAAGATGAGATACAAAATGAAGTAGGAAATAAGAGAATGGTTAAAGAGAAGGAT

CTGAAGAAGTTTAATTACTTAGATATGGTGGTGGATGAAACCTTAAGACTTTATCCTGTT

GGACCCTTATTAATCCCTCGTGAATGTAGAGAGAGCATAACAATTGATGGATATTTTATA

GCAAAACAGACTCGAGTTATAGTAAATGCATGGGCTATAGGGAGAGATTCTAATGTTTGG

TCAGAAAATGCTGATGAGTTTTACCCAGAGAGATTTATTGACAAGAAAATGAATTATCTC

GGTCAAGAGTTTGAATCTATACCATTTGGTTCTGGTCGTAGGCGTTGTCCTGGAATTCAA

TTGGGTTTAATTACGGTTAAGTCGGTTATAGCTCAATTTGTGCATTGCTTTAACTGGGAA

CTTCCACATAATATTAGTCCTTCCAATTTGAATATGGAGGAGAAATTTGGACTCTCTATA

CCAAGAGCTCAACATTTGCACGCAATACCGAGTTATCGTTTGGATGATGCTAAGCATGAA

TAG

>MS.gene009870.t1

ATGGCGAACCAGATCTTCTCTCGCGTTCTCCCCAAAACCCTAACCCTAACCACCTTCCTC

TATCGCTCCATCTCCACCACAACCACCACTCCCTCTTCCTCCGCTCTCTCCTTCCTCCGC

CGTCTCCGTCCCCTCTCTGCCGCCGCCATCACCTCCCGTCACATCCTCCTCCCCTTCCGC

GCTTTCTCCACACGCCCTACTACCTTTTCCCTCAACGATCCGAGTTTGTACGATGGATGC

GATTTTGAACACTGGCTTGTTGTTATGGAGAAGCCTGATGGTGATCCTACCAGAGATGAG

ATTATTGATAGTTATATCAAAACTTTGGCTAAGGTTGTCGGGAGTGAAGAAGTAGCTAGA

TATAGCATATATTCTGTTTCAACTAGACACTACTTTGCTTTTGGAGCTCTTTGTTTTGAA

GAGCTCTCCCACAAACTGAAAGACCTGCCCAAGGTTCTATGGGTTATTCCTGATTCTTAT

TTGAATGTGGAGGAGAAAGATTATGGAGGTGGTGCAATAAGGGCGAGCCCTTCATTAATG

GGCAAGCAGTTCCATATGATCCCAAGTATCATGAGGAGTGGGTTAGGAACAATGCTGATA

GGCCCCAAATGCAGGTGGAGGATACCCTCCCCCAACATGAGCGGACCTCCTCCTCTTCCT

CCTAACATGAGCCGACCTCCTCCTCTCAACAGTGGATATGGTGCTCCTCAAAATAACTAT

CCGGGGCAACAAAACCAGAACATAGCAGGGATGCCTCCAAATGCTGGATATGGTGCTCCT

CAAAATAACTATCCGGGGCAACAAAACCAGAACATAGCAGGGATGCCTCCAAATGCTGGA

TGGTCAAATAATCAGTAG

>MS.gene009933.t1

ATGCCATCGCGCTCCCTTGTGACACAACTTGAGAAGCAAGTAAATGATGTTGAGCAGTTT

TACCAATCCATTGACGTTCAACAGAATGATTGCAAATACAAAGGCAGGGAGAAGCCTCCT

ACCGGATCTAAGAAGGCGTTGCAACGTGCTTCAGAAGATATGCAAGAGGAGATCAGGCGC

AATTTTAACAAAATATTCAACGAGATAGCTAAGGATAAATGGGCGTGGCCATTTTTGGAT

CCTGTAGATGTTGAAGGTCTTGGGCTGTATGACTATTATCAGATCATTGAGAAGCCTATG

GATTTTAGTACTATAAAAATAAGAATGGAGGCTAAAGATGGCTCTGGTTATAAGAACGTG

AGGGAGATATATGCTGATGTAAGGCTGATTTTTAAGAATGCAATGAAGTACAATGATGAA

AAGAATGATGTCCATGTGATGGCCAAGACCTTGCTGGAAAAATTTGAGAAGAAATGGCTG

CATCTTTTGCCTAAAGTTGCTAAGGCGGAGAGTGACCTATCAAAGGAAGAAGTACATGAG

CAATTGAATAAAAGGCTTGCTCAAGAAGCAACTTATGCCAATATGACTAGGGAATTAAGC

ACTGAGCTGTCTAAGGTTGATATGGCTTTGAGAAGTCTCAAAACAACAGCGATTTCACAG

TGCAGGAAACTGTCACATCCGGAGAAATTAATACTTGCGAATGCATTCACCAAATTGTCT

CCTGATAACATTGTCAAGGCATTAGAGATAGTTAAAGAGAGCAATCCAAATTTCCAAGAT

AGTGTTGATATGGTGACCCTTGACCTTGATTCTCAGAGCGACTACACATTGTTTAGATTG

CATATGTTTGTTAAAAACACACTAGAAGTTCAAGAAGGAACAAGCGTTATTAATCATGAA

GACAACGTTGAGGAAATGAAAAACAACGCCAAAAAGAGGAGAATTGTGTGA

>MS.gene009892.t1

ATGGGAAATGGGTTTTACAATCCTACCACCACCAACAAAAGATTTGGGAACCTAACCCTA

AATCAAAACGACGAGAAACAACAGTCACAGGGACTAGAGAAGGAAGACGCAGGAATGCAG

ACAGCTGAGACATATCTTCCTATTGAGTTAATCATTTCTCTTCCTATTGAGTTAATCATT

CAAATCCTTCTAAGGTTACCAGTGAAATCTCTTATTCGTTTCAAATGCGTTTGTAAGTCA

TGGCTTTCTCTTATCTCTCAACCTCACTTTGCAAATTCACATTTTCAACTTACCGCCGCG

ACACACACGAATAGAATTATGTTGATAACTCCATATCTCAAAGCTCTATCAATAGATCTT

GAATCATCGCTTAACGATGATAGTGCTTCATATCCAATGGACATTAGTTTTTTGATTGAC

GATGATGATTACTATAGTTCTTCTTCATCTGACATGGATGAATTGTCGCCTCCCAAATCT

TTTTTTAAACTTGATTTTAAAGGTTCATGCAGAGGCTTTATACTTTTGAACTGTTATTCA

AGCCTCCGTATATGGAATCCATCTACCGGATTTCACAAACGAATACCTTTTACTACAATT

GATTCCAATCCAGATGCAAATTATTTCTATGGTTTTGGGTATGACGAGTCAACAGATGAT

TACTTGGTGCTTTCAATGTCCTATGAACAGACTCCTAGCTCAGATGGTATGCTATCTCAC

TTGGGAATTTTCTCATTGAGAGCTAATGTGTGGAGCGCAATTGAGGGTGGTAACCTTTTG

CCTTATTCGCAGAACAGTCTTTTAAACTTAGTAGAGTCGATCTCAAATGGGGCTATTCAC

TGGTTGGCTTTTCGTAATGATATATCAATGCCTGTTATTGTTGCCTTTCATTTAATTGAA

AGGAAACTTCTAGAGTTGCGTTTGCCAAATGATATAATCAATGGTCCTAGCAGGGCGTAT

GATTTATGGGTGTATAGAGGATGTCTCGCTCTATGGGATATTTTGTCTGATAGAGTTACA

TTTGAAATATGGGTTATGGAAAAATACAATGTGCAATCGTCTTGGACTAAGACTCTTGTC

CTGTCTTTTGATGGCAATCCTGCTCACTCCTTTTGGCCAAAATACTATACAAAAAGTGGT

GATATCGTTGGAAGAAACATGCGTGGTGCATTGGCCAAGTATAATGACAAAGGACAGCTG

CAAGAACATCACTCCTACTGTGACAATGCATATGTATCCCTAGTGGTTATGTATACAGAA

TCTCTGCTTTCAATTCCTGGTGGTGACCGTGGTCAAGTTTAA

>MS.gene009908.t1

ATGGCAACTGAGAAATCCCATGTTTATCACGAAAGACAAAGATTACAGTTCTGTCTCATA

CATACTCTCAACTCTCTCTTTCAGCAAAAAGATGCTTTTACTCGAGCAAATTTGAATGCA

ATTTCTGAAAAACTAGCACTTGATGAATCCTTCAACAATGAATCATCATGGACACCATTA

TCTATCCTTTTCAAGCCTCATCATAATGCATTGACTGGAAACTATGACATAAATGTTTTA

ACAGCTGCTTTGGAAGAGAAAGGAAAGAATGTAGTTTGGCACGATCGACGAAAGGGAGGA

TCTTCAGTTGATCTTGATGCATCAGAAGATGTTTTGATGGGGGTTGTGATCAATATTGCA

GTGAAAAGGTTTGCTGGGATTTGGAAAAGTAGACATTGGATTGCTTTGAGGAAGATTGAT

GGTGTTTGGTATAACTTGGATAGTGACCTATCTGCCCCGAAAAGTTTTCGTGATACTGAT

GAAGTAAGAGAGTTCTTGGATTCCAGCATAGTTCGTGGTGGTGAGGTTTTGCTTGTCATG

AATCAGAAACAGTCTTGA

>MS.gene009887.t1

ATGGTGACCGGTGAAAGCAACAAGACCATCGCCTTGGAATTTTTGTCTAATGCATTTGCT

GCGGGAGAAACGAGTAGACATGCTAAAGTTCGAGGCAAGAGGGTGGGATACAGTGCTGAT

GATATTGCTAGGGTGTTGGGGCTTACTAGGCCGGACGAGTGTGGTGTTATTGAGCGGAGG

AAGGAGTCTGGAGGAGGGGAGAGAAATGCTGATTATTGGGCTGGGTTACTTGCTGGTCTG

GTTCGAGAGGGGGCTGGTTGGAAAGGAAGAGGCGGAGTACCGCAGCGCATTGATGTAGTG

GATTTATTGCCGATATTCAAGGCTTGGTCAAACTTTCTTCTGACCACTATTGAGCGGACA

TCCGCTAAGGCTGAGATGACCCGTGCTCGCTTTTACATTTTGCAGGCTGTGCTTTCTGAT

GATGACATTGATGTTGCCAGGTTGATGTATGCGAGTTTGAAGGACCTCATCAACACCACT

GGGTCCACTGCTGGTCATTGTTGTCTGATTAACGCTCTCTGCCAGGAGGCACAGGTTCCT

TCAGAACCTACTGATATCTATCTCACATCTCAGCTTCCAATTTCAGACAAGACCCTGGTA

AAATATGAGAAAGAGCAAGAGAAGTTTGAGCGCGAACTCGCGCGACAAGGGGAGCAGCAG

GGTCAGCCTCAGATGATGGAGGAGGATCAGGCACACCAGATACCGCAGATGCCTCAGGTG

CAGCCACCTATGATGCAGCAGCAGCAGGGATACATCCCTCCTCATTTTGCACACTACACT

TATGCTATGGCCAATTGGGCCATGGATATCTCCTCCCGAGATCGGATACCACCACCAGCA

TTCCATGAAGAGTTTATTATTGCAGCTGATGCATATCGTCGTGGTCCTGATGCCATGACT

AATGCATATCAGAGATTTGCGAGCCCTGAGGACATGGAGAGGTACTTTGCTGAGGAGAGG

GCGAGGGGAGCCGCCAGAGAGGCTAGCATCAGAGAGGAGTACTACCGGATTCAGGCTGAG

CAGCCTAATACCGAGGAGCATCCACATTACTATCCATTTCCACCATCTGGACCAGGAGGG

TCGTCAGGAGCAGGAGGAGCACATTGA

>MS.gene009927.t1

ATGAAGAAATCAGCAAGCACCAATTTGCAATATCAGTCTAGTCAAAGAACAAAGAATTCT

AATTTTCAACATAGTTCAGAAATTGATTCAACAAAGAAGCAGAATTCAGCACTGAAAAGT

TTAAAATCTGTTGTCAAAATATTTGCTTTGATTTTCTCTGGCAGAAGAAAAACAGCTTCA

AAAATTACTGGAAGTGATGACAGAAAGAATACATCAAAACCAAGATTGATGTTATCATCT

TCAACTGATTTATCATCCGAAAGTAGTAACAAGAATTCATCAAAATGGAGATTTTCTTCT

TCCTATGCATCATCTAGTACTACAAGTGAACAGCTGGGAACTGGGAATTTCACTTTTGAA

GAAATTTACAAGTCAACTGCAAAATTCTCTACAGATAATCAAATTGGAGAAGGTGGATTT

GGAACTGTCTATAAAGGAAAGCTTAATGATGGAACAATTGTCGCCGTGAAGCGCGCCAAG

AAGGAAGCTTTGCAGAGTCACCTATATGAATTCAAGAATGAAATATACACTTTATCAAAG

ATTGAGCACCTGAATCTTGTGAGGTTGTATGGATATTTGGAACATGGAGATGAGAAGCTT

ATTATTGTTGAGTATGTTGGGAATGGAAATCTTAGAGAACATTTAGATGGTATTCGGGGT

GACGGACTAGAAATTGGTGAGCGTCTAGACATAGCAATTGATATAGCTCATGCAATAACT

TACCTTCATATGTACACAGATAATCCAATTATACATAGAGACATCAAAGCATCAAACATC

CTAATTAGTGAGAATCTAAGGGCTAAAGTGGCAGACTTTGGTTTTGCAAGATTGTCTGAA

GACCCTGGTGCAACTCATATTTCAACTCAAGTTAAAGGAACAGCTGGATATATGGATCCT

GAGTACTTGAGAACTTATCAACTCACAGAAAAGAGTGATGTTTATTCATTTGGCGTATTG

CTTGTTGAAATGATGACAGGAAGACATCCAGTTGAACCAAAGAAGAAAATTGATGAGAGA

GTCACAATCAGATGGGCAATGAAGATGCTCAAGAACGGAGACGCTGTGTTTGCAATGGAT

CCAAGACTGAGAAGAAGTCCAGCCTCCATCAAGGTAGTGAAGAAGGTTTTCAAGCTAGCT

TTTCAATGCCTGGCACCTTCAATACATTCAAGGCCACCTATGAAGAATTGTGCAGAGGTT

CTGTGGGGAATCCGTAAAGATTTTAAAGATGAAACCACTCCTCTTCCTACCTTACCTTCT

CATCAATCTGAAAATTTTCCTCAAAGAGAAAAGAATAAGCATATAACATTTGGTATTGAA

GATGATGACAGCTACAAATTTTCCTCTGCACCTAATCACATTCATTTATGA

>MS.gene009948.t1

ACCTCTGTGGATTTTTGACTGGTGTTGGTAGTGAAAGGGAAATACAAAAGGGTGGTAAGA

CTTACAAGTTGAATGTCATTGAATTGGAATCACTTGGGCATAAGGTTCAGTGTACTCTGT

TTGGTAGCTATGTTGATGATTTAAACAACTTTATTGCAAGTGGTGATGTCCAGAATGCCA

TTGTTATCATTCAACTTGCTAAAGCAAAAATCTTCCAAGACAAGCTTCATGTCCAGAACT

GTTTGAATTGTACAAGAGTTATTTACAACCCAACATGTGCTGATGGTTTGACGCTAAGGA

ATAGGCTTGCTGAGACTCCTGACACACCTTCACCACTATCTCTAACACAACTGCTACCTC

AAACCAAAGTTGACCCAAAGGAGGAGTTCCTTTATCATACTCCTAGAGCTACCATGCAAG

GCCTTAAGGATGCCTCTACTGATTCTGTCTTTGTTGTTGTGGGCACCATCAAGAGAGTGG

TGAACAAGGAGAACTTTTGGTACACTGCCTGTGTGTGTTCCAAGGCTGTCATTCCAGATT

CATCTATGTTCTTTTGTGAAAAGTGCGACAAGCACGTTAAAAAGGTTTTTCCTAGGTATT

GCTTGAGGGTGAGGGCCATTGACCAGACAGATTGTGCAACTCTGGTCATCTTTGACAGAG

ATTGCTCGACCCTCTTTAATAAGAGTTGTGCTGATCTCTTAGCTGAGCATGAAGTGGCTA

TCCATGAGGGTCAGCTTGCACCTGAGCTTGCTGCACTTATTGGGAAGACCTATTTATTCA

AAGTGGAGACCAAAGCAGATTTCAGTCCACGATTTGAACAGTCATTCCGTGTGCGCAAGA

TATGTCTTGACCCTGCAATCATCAAGGAGTTTATGTCAAAGTGGGAGAAGGAAGAATCCT

CATTCCTCAAGCTAACTAATGAGAGTGCATCTCTTAGTGTGTTGATGGACAAGGGCAAGC

ATACAATGCACATAGGAGAATCCAGTCTTTTATCTCAAGATCCAATTTCCCTATCTGAAC

CTGTGCAGAAGATTCAAAGGCAGCTTGCTAAGGTTCCTGAATCTGCTTCTGATGTGGTCA

AACAGGATTTGATGACTAAGTTTGGAGGTGTTGATTCAGAGCCTGATTGCCAGGAGATAC

CCCCCTCACATTCTGCATCTTCATCCAGCAAAAGGGCATCTCCCACAAATGATGATGATG

ACATGAATGTTCCTATTAAGAAGCTGATTAGGAGGACTGTCAAGATTGAGAAGATTTAG

>MS.gene009904.t1

ATGAACATCGAAGAAGAAGAAAATGTTGACACGGCTAACAATGAAATGAAACTTGAAGAA

CCACAATTAGTAGATTCTACCGCTGTATTTATCAATGATAAAGTGTTTGCTACACGTGGT

GATTTATTGAAATGGGCTAGAAATGTTGGGAAGGAAAATGGTGTTGTTGTTGTGATTTAT

AGGTCTGAAACAGCCACAGCAAAACCAGGAACTAGGACAAGACTAATCCTCGGTTGTGAA

AGAAGCGGAAAGTATAGACCTTGGAAGAATCCTAAACCTGAGAAACGAAAAATGGTCATG

GAGGCATGGGAAGGTGTAGTATATTGTTCTAATGAAGCTGAGTATCAGGAACAGTTGAAA

GTTTTCAAAGAAATTTGTATTGATTTTACCATTTTTCATGATTATGTACATGAACAGTGG

TTGATTCCTTATAAAGAAAGATTTGTTGAGGCATGGACAAACAAGGTTATGCACTTAGGG

AACACAACGACTAATAGAGCTGAGTCTTCACATTGGAGTTTGAAGAGGATATTGCAAGAT

AGCATGGGGGACATATGTAGTGTTTGGGATGCAGCTTCTAGCATGATCATACTACAACAC

AATGAGATAAAATCATCATTCGAAAAAAGCATATTTCAAAAGGAACATCGACATAGTACT

AGATTGTACGCTCACTTGTGTGGCCATGTTTCGAGACATGCATTATCGCACATTGCCAAT

GAGTTTGATCGGGTGAAGTACGTCGGTATTGATAAGTGTAGTTGTCGTTGCACTATTAGG

ACGACTCATGGTCTACCTTGTGCATGTGAACTAGCCAGATATAGTATGATGCATAGTGCC

ATCCCATTGGATGCAGTTCATATTTTTTGGAGGAGGTTAAATTTTTTCGATGATGGATTC

AACAAATCATCAGAGTTATCTATGAAACCTTTGATTGATGCATTAATGAGAAAATTTGAT

GAGCTTGATATGTGTGGAAAAATTGCACTTAAGAGTAAAGTACATGAACTTGTTTTCCCA

ATCACCACCTCAATGTGTCCACCGCCTATCAAAACAAATGTTAAGGGTGCACCAAAGATA

GGGAAGAGAAAGTTGTCTAAGAGACAAAAATCAACAAAGCGTGATCCATCTTGGTGGGAA

CATGTAGATGCAGCTATTGAATCCCAAGGTTCAGTACAGAAACTAACACCTACACCACCA

ATTCAGAAGTTTACAACACAACCATTGAAACAAAAGCTTACACCTCGACCTTCAGTCAAT

AAGGTTAATCAACCTAGACTATTACCCTTTATGGAATGGTTACATAAAGAGATCCACCAT

TTCATAGATGATGTTCTGGATGTAGGCCCGGATGGTAATTGTGGATATCGTGCAGTTGGC

GCTTTGCTTGGTAGAGGTGAAGACTCGTTGCCTCTAATTCGTCAGGAATGTTTGGAAGAG

CTTCTAGAGTGGAAGGCTGATTACGCAAGGATGTTTGGTGGGGAAGATTATGTTCAAGGT

ATGATCCAAAGTTTATATGTTGACGGATATGCAACTAGGGATAAGTGGATGACTCTTCCA

GCAATGGGTCATGTGATTGCTTCAAAGTATAATATCACACTAGTCTCATTGTCGGTGGAC

ATGCCAATGACTTTTTTCCCTCTTAGAAGTCCATTTTCACCATCATCTAGTCTCATTGTA

ATTACCTACGTTAATTACTGTCACTTTGTACAGGTTTATTTAAAGCCTAATGCTCCAATA

CCACCAGCAACAAATTTATGGAAAAAGTATCGCAGAGAAGAAGCTCGCACCTGGGAAACT

GCATATCTTACACGCATACAGTCTTGGTTGACAATATTTCCATATACATATGAACCTGTT

ATAAACCTTGGTGATCCTATTTAA

>MS.gene009936.t1

ATGTTGGCTCTTCAACCTTATTCTGTGGGACATCTAATTGATCAAGAGGTTAAAGCCTGG

AATGAGCAGTTGGTCCGTCAGTTATTTGCAAATCAAGGTAATCGCTTCATCAACAATGCT

CTCAAGATTTTGCTAAAGCAGAAAATAAAGATGACTTTAAATGACATTCATCCAAGCTTG

AGTGATTCATCGAATGGTGATCAACAAAGCATCATGAACAACACTATTGCTATACTAACT

GAGGGTGTTCATTCCTTGAAGCAGGAAAACAACAAAGGCTATAAAAGCCTAATGCTATAT

CAATGTCACTTAAGTGAAGGAGGTGTTCACTGCTACCGACTTAACACTTGGAAAATAGAA

AGGGAAGATCCTATTTTTAACCCCGGTGGAATTTTTAACCCTTTGAACTTTGCACCAACT

TTAGACCCTGAAGAGAAGGAAATTGCTAATGGTAGATTGGCAATGTTGGCATTCTTGGGA

TTTATCATTCAGCACAATGTCACAGGAAAAGGACCATTTGATAACCTCTTGGAACACCTT

TCAGACCCATGGCACAACACCATTGTTCAACCATTGAGTGGCGGCAACTAA

>MS.gene009866.t1

ATGGTTGATGGTCGTGAAATAACTGTCCAGTTTGCAAAGTATGGCCCTAATGCTGAGAGA

ATTCACAAAGGAAGGATTATTGAAACATCCCCGAGATCAAAGAGCTCAAGGAGCCGCAGT

CCCAGCAGGAGAAGGCATCACGATGATTACAGAGACAAGGGTTACAGGAGGAGAAGTCGC

AGTAGAAGTTATGATAGGTATGAACGTGATAGGTACCGTGGTGGGAGAGACAGGGACTAC

CGTCGCCGAAGCAGAAGCCGTAGTGCCAGTCCTGATTACAAGCGCCGTGGAAGGGGGCGC

TATGATGATGAGCGTCGTAGTAGAAGCAGAAGCAGATCAGTAGACAGCCGCTCCCCTGCA

AGACACAGTCCTAGTCCAAAAAGGAGCCCTTCTCCTAAGAAGAGTATTTCTCCTAAGAAA

AGTCCTAGTCCTAGTCCTAGTCCACGTCGCGAAAGTCCTGATACTCGTAGCCGTGATGGA

AGATCTCCTACTTCTCGCAGTGTCTCTCCACGAGGCCGTCCTGAAGCTTCCCGAAGCCCT

TCTCGAAATTCAAACGGTGATGAATGA

>MS.gene009880.t1

ATGGGCTTAGAAACTAGTAATGCTTTGATGCCATTCTCAGCTACATCGAAGATCCCTTTC

ACAATTTCAACACCAAAGACACGTTACTGTAGGACCATTATAGCTTATATGATGGTGGAG

TGTCTACCATATTTCATCAACGATGATAATTCACAACAACCAGATACTCCATGTTGCATT

GCAGTGCAATCTATAGCAGTAAACGATACCAACAATTGCATTTGTGATATTACGGAGAGT

GACAATTTGTCAATGGATCTCACAAAGGCAACGAAGCTACCTACTATTTGTGGCATATCA

CTCCCATGTTATGGCATGATACTTGAAAAATAA

>MS.gene009855.t1

ATGGTATTCCAATTTTATGTCCATAATATAATATATGAGAAACTTTTATGCTTCATTTTA

ATTTTGGAGGCTCTTGAAGGAGGTGATTCAAGCAACAAGGGAGAGGCTGTTGCAACAGCT

GATACTCCAGCAAGCAAACCGTTGGGAAAAAGGCCAGTTGAGAGTGTCGAAGGGATGTGT

GCGATCACCATTGAGTTTGGAGAAGGCTCTGCTACTAAAGAGTCGAAGATGGTTTGCGTC

AAGATGAAGATTTGA

>MS.gene009854.t1

ATGTCTTCTGCAGAAATAATAACTGCATTACTTTATTTCCTTCTCTTCACTTTCACATGC

TTTTTATTCAAACTCTTCTTATATCCAAAACAAAATACCATTATCCATAAAAAGCCACCT

GGTCCTCCAACAATACCAATAATCGGAAACCTTCATATGTTAGGTAAACTTCCACATAGA

ACACTTCAATCACTTTCCAAAAAATATGGTCCAATCATGTCCTTACAACTTGGTCAAGTT

CCAACTATTGTCATTTCATCTTCAAAATATGCAGAATCATTTCTCAAAACTCATGACATC

AATTTTGCAAGCCGACCAAAGATTCAAGGAACTGAACTCATAACATATGGTTCCAAAGGG

TTGACTTTTTCTAAGTATGGTCCTTATTGGCGTAGTGTGAGGAAACTTTGCACTTTAAAA

CTTCTTAGTGCTTCCAAAGTTGAGATGTTTGGTCCTATTAGGAAGGAAAAGTTGGATGTT

TTGGTTAACACTTTGAAGAAAGCTTCTCTGGTAGGTGAGGTTGTGAATGTTAGTGAGGCA

GTAGAGAATCTTATAGAAGATATTGTGTATAAGATGATATTGGGTCGGAGCAAGTATGAT

CAATTTGACTTAAAGAGGGTGGTTCAAGACGTAATGGCTTTGGTTGGAGCTTTTAATCTG

GCTGATTATGTTCCTTGGCTAGGTGTATTTGATCTTCAGGGATTAACACGAGCATGCAAG

GAAACGGGTAAAGCAATTGATGAGGTGCTGGAAGTGATAATAACAGAGCATGAACAAACC

ACTAATGTAGACAAAACTCGTCATGAAGACTTTGTTGACATACTTCTTTCATTTATGCAC

CAAACCATAGATCTAGAAAATGAAGAAAATCATTTCATTGATCGAACTAACATCAAGGCA

ATATTACTAGACTTGATTGTGGCAGCAATTGATACATCTGCTACCGTGATTGTATGGGTT

TTATCCGAACTTCTAAGACATCAACGGGTAATGAAAATCCTTCAAGATGAGATACAAAAT

GAAGTAGGAAATGAAAGAATGGTTGAAGAGAAAGATTTAGAGAAGTTAAGTTACTTAGAT

ATGGTGGTTCATGAAACTTTAAGACTTTACCCTGTTGCACCTTTATTAGTCCCTCGTGAA

TGTAGAGTGAGCATAACAATTGATGGATATTTTCTCAAGGAAAAGACACGTGTTATAGTA

AATGCATGGGCTATAGGGAGAGATTCAAATGTTTGGTCAGAAAATGCTGAAGAATTTTAC

CCAGAGAGATTTATCGACAAGAAAATGAATTATCAAGGACAAGAGTTTGAATCTCTACCA

TTTGGTTCTGGCCGCAGACGTTGTCCTGGAATTCAATTGGGTTTAGTGACCGTTAAGTTG

GTTATTGCCCAATTGGTGCATTGTTTTGATTGGGAACTTCCATATAATATTAGTCCTTCC

AATTTGAATATGGAGGAGAAATTTGGACTCACTATACCAAGAGCTCAACACTTGCATGCA

ATACCGACTTATCGTTTGGCATGTGATGACAAGCTTGAATAG

>MS.gene009970.t1

ATGGATGAGAAGATCTCTTGCTTGGTTGATTTGCAGATAAAGGGTGAGTTTCACAATGTT

AATGGAGGTTTGATGAACTTGTTAAGGAACTGTTTGGAGTCAGATGGGGACAACTCAAGA

AGCATTTTATCAGGTCATGTTGATCATTTCCAAAGCAATAAGTTTGAAGATGTTGGATGG

GGTTGCGGTTGGCGTAACATACAAATGCTTAGCTCACATTTATTGGCACAAAAGAGAGAA

ACAAAAGATGTCTTATTCGGTGGTTCAGGATTTGTTCCTGATATTCCATCCCTTCAAAGA

TGGCTTGAGATTGCTTGGGAAAGAGGTTTTGATGAATCTGGCTCTCATCAATTCAATCAT

GCTATCTATGGCTCTAAAAAATGGATAGGAGCTACCGAATGTGCTGCGCTTTTGCGTTCT

TTTGGTCTCCGATCAAGGGTGGTGGATTTTGGTCCTAAGGAATCTGAATCTCTCTATCTG

TCTGTCCCTGGTTCAAGTGTTGGTGGGCAGGATTTAGTGAGATCTGGTGATGAAAGGAAG

AGGAAAGCACCGAATGTTTCAGGACCAATGGATAGATATCTATCTCGTTGTGGTGGTGGT

GTTTCTCAAACAAGTTGTAGCAAGAATGTAGAGTCTTGTTCTTCTATTAATGCTACTGTG

AATAGAGAAAGTGGCGGTGAATGTGCGGTGAAAAGTGCTGCTAAACAAAGTAAAAATCAT

CAAGTTCTCATGGACTTTGTGTGGAATTATTTTTCAAATAAAAACTCGATTCAATTTGGC

TATAGGCGTGTTGTCTTCAGCGAAAAAACGCCCTTGTACTTTCAACATGATGGACACTCG

AGGACAATAGTTGGAATCCAAGTCAGACATCAACGAAATGGATTTCTGCATTATAATCTC

CTAGTTCTTGATCCTGGTCATAGAACGGCAGCTATAGAAAGATCACTAAGGGAGAGAAAT

GGATGGCAAAGATTCATAAAAAGAGGAGTGCACACACTGAGGAAGCAACAATACCAGCTG

TGTTATGTTGATCCTGGAATTGCCAGTGAGGAGGAGATGGAAAAACTCAAGACAGTGGAC

AGTGTCTTCATTGAACTTTAG

>MS.gene009981.t1

ATGGCCACACTAGAGAATCTTGCAGCCCCGTCGTCCGATCATTACCCCATCCTTTTGAAT

AGAACCCCTGTGCAGCGCTCTTACTCCCACAAGCGTGGTTTTCGTTACGAAAACGGATGG

CATGCTGAACCGGGTTTTAAAGAAATTGTGACTGATGCATGGCACTTGCACAGTGCACAT

TCGGTTATTCCTAGACTCACTGAATGCGCAGCTGATATGAAAACTTGGAGCAGGGATCAT

TGTAACAAGCTGAAGTCAAACATTGAAGAATGTAGGCGTAAAATCCAAACTGTTAGACTG

AGTAGCACAGGCCCCGACCAAGACGAAATTGTTAATTTAAGGAAGAAAATGAGCAGACTG

CTTTCTCAGGACAATGCATATTGGAGACAACGTGCTAAGGTTCATTGGTATAGAGACGGT

GACCGTAATACTAAATATTTTCATGCCTCTGCCACAGCAAGGAAGAAGATTAATCGGATT

TTGTCTCTCGAGGACGATTCAGGTAACAAGGTCTCTGATAGTCAAGGAATGTGTGGTTTT

GCAAAAAATTATTTTGAGAATCTATTTCAGAAACATAATAATGTTGCGGACCCTGTCACT

CAATTTATTCATCAGTCTGTTTTTGATGAGGACAATGTATTACTTACTGCACCTTTCACC

AAGGAAGAATTCCGGGCTGCGATTTTCTCTATGCATCCGGATAAAATACATCAACCGTCG

TTCCCTTTCTTCCCCCTACATCCAAACTTTAACCTCCACCATGTAGCTTCCATTAATGGT

GGTGCGTTGAGCTTCAACCTCCGCACTGCCGTCATGCCTCCAGTCCAGCCTTCCTCTGTT

CCAGATCGACATTCCGCCGTCAGATCCGTGCCGGTTTCGTGTCTATGTTTCCGTTCCGGC

AGCCTTTGGCTGCGACGGCGGTGGTGTTTGCCTTTGGTTCAACCTCCCTCTATTCCATGC

TCGACCTTCAGCTGTCAGATCTGTGCCGGTTTCGTGTCTCGGTTTCCGGTACGTTCATGG

CAGTTCCGGCAGCCTTTGGCTGCGACGGCGGTGTTTTTGTTCGGGTTCGAGTCTACTGTG

TTTGTGACGTGTCTCGCCTGTTGTGTTGGTTTGTAG

>MS.gene009969.t1

ATGGAACCACATACATGTTTGACTCTTGTTTTCTTTGTGCTTGTAAACTTGGCTGTTGGG

GTGTTAAGTAATGATCATTATAGCAGACATGATTTCCCCGTTGACTTTGTTTTTGGATCA

GGCACCTCTGCTTATCAGGTGGAAGGAGCTGCTAATGAAGATGGAAGAACTCCTAGCATT

TGGGATACCTTTGCACATGCTGGGTTTGCGCGTGGAGGAAATGGAGATGTAGCCTGCGAC

ATGTACCACAAATACAAGGAAGATGTGCAACTCATGGTTGAAACAGGCCTCGATGCCTAT

AGGTTTTCCATTTCTTGGTCAAGATTGATACCAAATGGTAGAGGACCCGTCAACCCGAAG

GGATTGCAGTATTACAACAATCTCATCAATGAACTCATTAGAAATGGAATCCAACCACAT

GTCACACTACACCACTATGATCTCCCACAGGCGCTTGAGGATGAATATGAAGGATGGCTT

AGTCGTGAGGTCATAAAAGACTTCACAAACTATGCAGATGTGTGTTTTAGAGAGTTCGGG

GATAGAGTCAAGTATTGGACTACTGTGAACGAGCCCAATATCTTCGCTGTAGGTAGTTAT

GATCAAGGAATCACCCCACCTAAGCGATGTTCTCCTCCATTTTGCGTTATAGAGAGCACC

AAGGGCAACTCATCATTTGAACCGTACTTGGTAGTTCATCATATTTTGTTAGCGCATTCT

TCAGCCGTGAGACTGTATAGAAGAAAGTACAGGGAGGAACAGAATGGATTTGTTGGTATC

TCAATCTATGCATTTGGGTCAGTTCCTCAAACAAATACAGAGAAAGATAGGGCAGCATGT

CAACGATTTCGCGATTTTTATTTGGGTTGGATTATGGAACCCTTGTTGCATGGAGACTAT

CCCGATTCCATGAAGACAAATGCGGGCGCGAGAATTCCTTCCTTCACAAGTCGTGAATCC

GAACAAGTTAAGGGTTCCTATGACTTTATTGGCATAATTCACTACATCAAGTTTAATGTC

ACAGACAATTCTGATGTGTTGAAGACCGAACTGCGAGATTTCATTGCAGACATGGCTGCA

AAGCCACTTGGTACAGAGGAAATATTTGTAGAGAACGAGTACCCATTCACACCATGGGCT

TTAGGAGAAGTGCTGGAAACATTCAAGACCCTTTATGGCAATCCACCTATATTCATTCAT

GAAAATGGCCAACGGACACTAAGCAACGCATCACTCCACCATGATGAATCAAGGGTGAAA

TACTTGCATGGATACATTGGCACCGTGCTTGATTCCTTAAGAAATGGATCAAATATGAAG

GGATATTTTGTGTGGTCTTTCATTGATGCATTCGAGTTGTTGGATGGATATGAATCGGTC

TATGGGCTATACTATGTCGATAGGAATGATCCAGAGTTGAGAAGATACCCAAAGCTATCC

ACAAAATGGTACAACCAATTTTTGAAGGGTACAAGATCCTCCCTAGTTGGTGCAATTGAA

CTCAATAACGATTCATCACTTGTTTCGGTTGGAGATTTACTTCAGTAG

>MS.gene009983.t1

ATGGAAGCCAAACAGCCGGCGAAAGATAAGAGGAACTATCAAATGGTGCGCGATCTGATG

ATTATGGAGACTAATTCTGGGCAGTCAACTGCTCCGGGTTTTGAGCAGGCTGGTGGACAA

GATCTCTCATACGGGGTCTTGAATGTGTTTGGCGGTGGTTCTCCTGAAGAGAGCCTGACT

GCAAAGATGAAGGCGGCAACAGATGCAGACACCAGTGGCGCTTGTGAGTTTGAGGCTGAA

AAGGCCAAGTACAACTTGTTTGTTGAGATTGTTGACAATTCCAATAAGGAAATGGAAGAC

GGAAACAAGACACTTTTTGACCGGATTGTTGCTCTTCAGAAGGATATCAATGATTTGGGA

TCTCCGAAGGATCTTCACGGTAGGATAGTAGGTTTGGAAAACGGTGTCGATGAGCATCAT

ACGACTTTGGAGGTGTCCAAGAAGGCTCTGGGGGAGGCCAAGTCGATCAGAGAAGGTACA

TCTCATCCAAAAGCTCATGAATGGGAAGCTTGTCCCTATATTGAAGAAGATTATAATAAA

GAAGCCATTGTTGGAGATTCTGGTGATGACATCCTCGTTGAAGGAGATCTTCAGGGTGAA

TTGGTGAATGAATCACCGCATCCCCGCCAATCACGTAGGAAACAAACTGATCTAAGGAAA

CAGAAGCAAGGAGGCAAGCTTCTTATTAACAAACCAATTCATTTCAATTCTCTCAAGGGT

CGCCTTGCATCTATGTGCCAGCCTAAACACCAGTCCATGGCTATTCGAAAAATTCATGCT

GGTGAAGAGGAAGTTCAGTTTCGGGGAGGATCCGCACCATGGCATTGGATAAAGCTGAGA

TATGGGTCCAGTGCTAGTCTCGAATGTCTCATAACAAGCACATCAGACCACTGCCCGCTC

CGGCTAGTCTGCACACCCAATCCGAGACGCAACAGGAATGGGAATCAGTTCAGGTTTGAA

AACTCTTGGCTGGTTGAACCAGAGTTTGTCCCTTTCGTTCAGAATTGTTGGGGAATGTAT

ACAAATGTGAATATCATAGACAAGATGGATCATTGTGCAGAAGATTTTTCACACTGGGAT

AAAGAACACAGCCAGAAAACTCGCAGAGAAATTGAAAAGGTGAGACGGAAACTGGAATTG

ACCCTATCTCTGGTGACTGCCTCGTACATCAATTATTTCAATGCTCTTGATCGACAAGAT

CTAACAATAGAAAATAGCACCTTGGAGAAGGTGACAAGAGAACAACGACAACCGGTAATA

GAGTTAGAAACGGAAGCTAATTGTGTCAAAGAATCAGTTCTTCAACTTAGTAAGAAGGCT

GACGAAACCCCATCTGTAATATTTGCTGCCGTTAACAAAAAGAACGAAAACAAGAAGCAA

CCACCATTTTCAATGGTAATGATGAATGTGGAATCACCTATTTGTATTCCTTACTCGGAG

CATTCAAATGCATCTTCTTCATCTTCTTTAGGACATCCAATACTTACATCTAGTGGCCAA

TGGCAGCAGAGTTTCCCGTACGCAAACACTTCTCTTCATCACAATCATTACTCAAACGCG

TCTCCCTGGTATTCATATCCATATAGTGGCAAATGGCAGGGTTTCCAATACACTGGTCTT

GAAGATCACAGGCCTTTTCTTGGCGATCACAATTGTGTGGGGATAGTGGCTGGGGAGCAT

CATTACCGAGAGCCTCCATCAAGGACATTGTTTGTTCAAAACATCGACGTTAATGTTGAG

AACTCTGTAATACGAACACTATTTGAGATTTATCGGTATCAAATGACGACCTTCACCAAA

TATGTGGGGCTTATGGTGAGGTCAAGGAGATCAGAGAAACACCAAACAAGAGAGACCGTA

AATTTATTGAATTTTATGATGTTAGAGCAGCAGATGCAGCATTAA

>MS.gene009950.t1

ATGTGTTACATTCTTGAAACTATTTTTTGGCCGAACCAGGGTAGGGTCCCCATTCCAAAC

CAGTACTACAAAAAGCATTGTAAGGAAATTAACATCGAGGATCCAGGATTGCTTCTTGGT

GAAGGAATAAACTATGTTACACATTTCGACAGGGATGAAACAGGGGTGTATATGGGTAGT

GGAATTAGGATCACTAAGGAGTTTGGGTTCTTGCTTCCAACCAAGGTTAGGCTAGACTAT

GAGGGTCTAGAGAATGTGTAA

>MS.gene009960.t1

ATGCCGGTAAAAGGGCCGTCAACAGCTTTGCAGCCTCTGCCTCCGATCAGTAGATGTATA

TCTGAAGTTATTGATCCTTCTGAGGTTAAAGAAGCAGTTGCTCGTTATTTGAACTCTGCT

GAAAAAACCACCCCTGAATCTGATTCAATGGATGAATTGGGAAATGATTATGAAGAAGCT

GTCAGTGTAGAGTGGGTTGATAAGTGTTTAAGCCTTACTTTCAAGTGTCCATGTGGCAAA

AGATATGAGGTTCTAATATGTGCAAACAATTGTTACTACAAGTTGGTCTAG

>MS.gene009964.t1

ATGTATGAGAATAGACTTGGTGATGCTGGTTATATTCATTTTAAGCTTGGAAGGACTAAC

AACCGTGGTCTTTTGATAGCAGTACAAAAGGAATATTTCACAGTTGTTAATTACAAGGAG

TTGCATTTCAATGATTGTGGTGATAGAGTAGCTCAATTGTTACATGTTGTGTTAGCATTT

CCATTTTCACAGTGCCAAAACAGTGGTGTTAGGCAAGAAGTTCTCGTTGTCAATACTCAC

CTACTATTTCCACATGATTCAAGTCTATGCCTTGTGCGGCTGCACCAGGTTTACAAAATA

CTTCAATATGTGGAATCTTATCAGAATGAGTACAAACTTAAGCCTTTACCAATTATACTA

TGCGGTGACTGGAATGGAAGCAAACGCGGACATGTTTACAAGTTCCTAAGGTCTCACGGG

TTTGTATCTTCATACGATACTGCACATCAGTACACTGATGCAGATGATCACAAGTGGATT

AGCCACCGCAACCATCTGGGAAACTCATGTGCTGTTGATTTTATATGGCTTCTGAATCCT

GACAAATATAACAAACTACTAAAATCAAGTTGGAGTGAAGCAGTATTCGGCATGTTCAAG

TACCTAATGCGAAGAGCTTCGCAGACAGAGAGTGATGCATTTTCTTTTCTAAAGGCTGAC

GATGAAGATTGTATTACATATTCTGGTTTTTGTAAAGCACTTCAGCAACTTAATCTAATT

GGTCCATGCTATGGTTTAAGTGTTGAAGAAACAAAGGACTTGTGGTTCCAAGCAGACATA

GATGGAAATGGTGTAATTGATTACAAACAATTTCTGCATCAAGTATGGAATCCAACAGTG

TCGGATTATCAAAGAGATGACAACAAGAATGATAAGCAAGATGATGGTCCAAATGATAGT

GAGAACGAGGAGGAAGAGGAGGAAACAATTGGTTTTAGTGTAAAAAATGCAGTGTTGTTT

CCACCAGAGGTTGAGAAAGGTAGATGGCCAGAAGACTATTCCCTTTCTGATCATGCAAGA

CTAACTGTAGTGTTCTCACCAATAACATTGGCATGCTCTCGCAACATGTTTTCTTGA

>MS.gene009967.t1

ATGGCTTCCATAAGCTATATCCTCCTCCTCATATTTTCCTTCTTCAATCTAGTTCATTCA

TCTAGCAATGCTTCAATCAATGTAACCAAACACTTCTCCTTCCATGATTTCAGCTTCACT

AACAACTCAAGATTAGTCCATGATGTGAAGCTTCTAGGAAGTGCAAAGTTCTCAGATGAA

AAGGGTTCACTTCAAATCCCAAATGAATCAGAAGAAACAGATATAAGACATCAAGCAGGT

AGAGGTTTATATTCCTTCCCTATTCGTTTGTTAGATCCAATCACCAAAACACCAGCTTCT

TTTCAAACCACCTTTTCTTTTCAGCTTAATAATTTAACTACTGCTTCTGATTTAAGTGAT

GATGGTGGTGGTAGTGGACTTACCTTTATCATTGTGCCTGATGAGTTCACTGTTGGAAGG

CCAGGACCTTGGCTTGGTATGCTTAATGATGCTTGTGAGAGCGATTATAAAGCGGTTGCA

ATTGAGTTTGATACACGAGAAAATCCAGAATTCGGTGATCCAAATGATAACCATGTTGGT

ATCAATTTAGGTAGCATAGTATCTACTAAAATTATCAATGTTTCTGATATTGGAGTTTCT

CTTAAGGATGGTTTTGTTCACCATGCTTGGATTGATTATGATGGTCCTCAAAGACGAATC

GATATTCGTTTAGGACTTGCTAATCAAGATGTTTATCCTACTAAGCCTATTTTCTCGGAG

TTTATGGATCTTTCACCTTATCTGAATGAGTATATGTTTGTAGGATTCTCTGCTGCAACT

GGTAACCATACACAAATTCATAACATACTTTCTTGGAACTTTACTTCCACTAGCCAAGCT

TTTCTTCGCTACCCTTCATCTGAAACATGTCAAGGTAAGATCATGCTTGAGAAAACTACT

ACAGAAACAACAGCTACCAATGAGAACTCTAAAAGAAACGAAACTCCACGGAGCTTTTTA

ATTTTTATGGCTGCTGTAGTGTTGGCTTTAGTTGTTTTGATTGGTTTTTACTTTATCAGT

AAGCATAGAAAAAGGGCAGCTAAATCAAACACTTCAATAGATGAAGAGATTCATAGGCCA

AGGCCTCCTAACAAACCGCGCAGATTCCGTTTTTCCGAACTTTCCTCTTCAACTAGATCA

TTCAATGAGATAGAGGTACTTGGAAGTGATAATAGAGGAGTTTTCTACAGAGGAAAGCTT

GCTAATGGAAATCAAGTAGCTTTGAAAAGATTCTCAGCGCAGTTTCTCAGTACACACGGA

TCCGATAAGAAGCGTTTGTTGAAGGAAATCAAAGTCATTAGCCATGTTCGCCACCCGAAT

TTACTTCCTATAAGAGGCTGGTGTCAAGACAACAACGAAATCGTTGCAGTCTATGATTTT

GTTCCTAATGGTAGCCTCGATAAATGGCTGTTCGGAGCCGGGGTTCTTCCGTGGACGAGG

CGGTTCAAAGTCATCAAAGATGTGGCCGACGGCTTGAGTTTCTTGCACACTAAGCAGCTA

GCTCATAAGAACTTGAAATGCAGCAGTGTGTTTTTAGATGTTAGCTTTAGAGCTGTTTTG

GGAGATTTTGGATTTGTGCTAATGGGAGCAGAATCAAAACAGTTTGAATCAGTAGTGTGT

AATGGTGCTGATGTATTTGAATTTGGTGTAATTGTGTTGGAAGTGATAGGTGGAAGGCCA

AGGGTGGAGGATACAGAAGAAGGAAAATCAGAAGAGAGGAACTTGTTGGATTTTGCATGG

AACTTGCATGAGATTAACGACAAAGTGAAGTTGGTTGATAGAAGAATGGGATCATTGATC

AATTTGGAGCAAGCGATTCGTGTTATGGAAATAGGGTTGCTTTGTACTTTGAATGAGAAC

AAAGGAAGGCCTACAATGGAGCAAGTTGTTGAGTTTCTTCATAACATGGAGAAGCCTATT

CCTGAGTTGCCAAAAACTAGACCTGTTGCTTTGTTTCCTTATAACAGTGCCAACACTGGA

CTTTGCAATAACTACTCTTGCACTTTGAAGCTGTGA

>MS.gene009979.t1

ATGGAAGGTGGTGGTGGTGGCAGTGGTGGTGGGAGGAGAGTTAGTTCAAGTCCAAGACCT

TGTTGTGGCCGGCGTGTGGTGGCGAAGAAGAAGCAAGGTCGTGGTGGTGCCGGTGATGGG

TTTATCAACAGTGTTAGGAAGCTTCAAAGACGTGAAATTTCTACCAAAAGTGTTCGTGGG

TTTAGTATCACTGATGCTCAAGAACGATTCCGCAATATCCGTTTGAAGGAAGAATATGAT

ACATATGATCCAAAAGGTCCTTCTTCCATCGTATTGCCATTTTTGAGAAAAAGGTCCAAG

ATTATTGAAATTGTAGCGGCTCAAGACATTGTTTTTGCCCTTGCACAATCTGGTGTGTGT

GCAGCATTTAACCGAGAGACCAATGAAAGGATATGCTTCTTGAATATAAGTCCTGATGAA

GTTATAAGAAGCTTGTTTTATAATAAAAATAACGACTCACTTATCACAGTTTCTGTCTAT

GCTTCCGACAGTTACAGTTCTTTGAAATGCAGAAGCACCAGGATTGAATATATAAGGAGG

GTTCAGCCAGATGCTGGATTTGCTCTTTTCGAATCTGAGTCTTTGAAATGGCCAGGTTTT

GTAGAGTTTGATGATGTAAATGGGAAAGTACTGACATACTCTGCCCAGGATAGCATATAC

AAGGTGTTTGACTTGAAAAACTATTCGATGTTATACTCTGTTGCTGATAAAAATGTACAA

GAGATTAAGATCAGTCCGGGGATCATGTTGTTGATTTATACTAAAACAAGCAGCCATGTC

CCTCTAAAAATCCTCTCAATAGAAGATGGTACTGTTTTGAAGTCATTCAACCATCTTATT

CATCGGAATAGCAAGGTGGACTTTATTGAGCAGTTCAATGAAAAGCTTCTTGTCAAGCAA

GAAAATGAGAACCTCCAAATCCTTGATGTACGTACTTTTGAGCTTACGGAAGTTAGCAAA

ACGGAATTTATGACGCCATCTGCATTTATTTTCCTGTATGAGAATCAATTGTTCCTGACC

TTTCGAAATAGAACTGTGGCTGTGTGGAACTTCCGTGGAGAACTTGTAACTTCTTTCGAG

GATCACCTTCTGTGGCATCCTGATTGCAACACAAACAACATATACATAACAAGTGACCAG

GATCTCATCATATCATACTGCAAGGCTGATTCTGATGAGTCATTGTCTGAAGGAAATGCG

GGATCCATCAATATCAGCAACATTTTAACTGGTAAGTGTCTTGCAAAGATAAGGGCAAGT

AATAGCTTTCCCATTGCAAAGGAATGCAACTGCGGTGACGACTGTTCAACTGGTGGCTGT

AATTCAAGGAAGCGAAAGCAGGACTCCAGAATAAGGAGCACAGTTAGAGAAGCCTTGGAA

GACATTACTGCTCTTTTCTATGATGAAGATCGCAATGAGATCTATACAGGCAACAGGCAT

GGTCTGGTTCATGTGTGGTCTAACTGA

>MS.gene009972.t1

ATGCCAAGAATTGTACCTTGGAAACTCCAAAGTTCCATTTTTTTTGTTTTTACTTTGTTA

TCTCTTTCTTTCTCTAGCATATTCACCATAACAAACAATTGCCCTTATACTATATGGCCC

GGCACACTTGCCGGCGCAGGCACGCCAGCGCTTCCATCGACCGGATTTCAGCTTGACTCA

GGTCAAGCTGCGAAACTAACAAGTGTTCCAGGGTGGTCGGGTCGAATATGGGCGAGGACT

GGTTGCACATTTGATGCATCAGGAATTGGTAAATGCCAAACAGGTGATTGTGGTGGAAGA

CTTGAATGTGATGGGAATGGTGCAGCTCCACCTACTTCGCTTTTCGAGATTACAATTGGA

CAAGGTGATCAACAAGATTACTATGATGTTAGCATGGTTGATGGATACAATCTCCCAATG

CTTGTTCTTCCAAGAGGTGTATATGGTAAAAGTGCATGTAATGCCACAGGTTGTGTGACT

GATATTAACAGAGGTTGTCCTAAAGAACTTCAAGTAGTTGGTGGTGATGGATACCAAGGT

AGTGTAGTTGGGTGTAAGAGTGCCTGTGAAGCATTTGGATCAGATCAATACTGTTGCAGT

GGACAATATGCTAATCCAAAAACATGCCAACCCTCTTTCTATTCCACAATTTTCAAGCAG

GCTTGTCCAAGAGCTTATAGCTATGCATTTGATGATGGCACCAGCACCTTCACTTGCAAA

GCCTATGAATATGACATTGTTTTCTGTCCTACCAGCAACAAGTAA

>MS.gene009998.t1

ATGATGTTGAGGCCAGGACCAATTCTTGCCTTAACAAGTGCAAGATTCAAAGAAAGTGAT

GAAGCAGAGAAGGTACCCCGTGTGTACATAAGGACAAAACATGACAGAGTGGTGAAGCCA

GAACAACAAGAAGCCATGATCAAGAGGTGGCCACCATTGAATGTGTATGAACTAGATAAT

AGTGATCATAGTCCCTTCTTCTCTACTCCTTTCATTCTCTTTGGTTTGCTTATAAAAGCT

GCAGCCGCTTTTGATGTTTGA

>MS.gene009975.t1

ATGGTGATGGAGGTTTTTATGAATCCTAACAAGGACCAAGCGGCGGGTGATTCTATTATA

ATTACGCAGTTTAATGTCTCCAATGCTATAAAAGATAGCATCTTGGTGAATTTTGGGGAG

TGTGGTTTGGCTGCATCACTGGGATCATTTCAAGTCAAGTATGTGAATCCAATCACTAAT

GTGTGCATCATTAGGGCTTCAAGAGAAGAGTATGAAAAAGTATGGGCTTCCATCACAATG

GTCAGAAGTATTGGAAATTTCCCAGTTGTGTTTAATTTGCTTGATTTATCTGGAAATTTA

CAGGCTACTAAGACTGCTGCATTAAAGTGTGAGGAAGCAAAATTTGAGCAGTTCAAACTT

ATGGTTGGTGATCGATTATCAGCTGAGGATACACATCGCATGAATAATCATCTTGCTAAG

ATCGAAGTTTTGGAGCATTGA

>MS.gene009974.t1

ATGGAGGTATTTGGAAAATCTATGGTTGCCGCTCCTTCAAATGTTATTTATCTGTCAAGT

ATTTTGGGCCATGATGGTCCAAGCCCTGTTCACAAATGCGACTGGAAATGCCAAAACGAA

AGTGTTTGGGGAAACATGTTTCGCTGCAAGCTGACAGGGCTGACTCACATCTGTGATAAA

AACTGTAACCAGAGAATTCTCTATGATAACCATAGCTCCCTTTGCCTAGCAAGTCGTCAA

ATTTTCCCCCTTACTCCAACTGAAGAACAGGCAGTGAGAGGCGTTCGAAGGAAGCTTGAC

GCAGCAGAGAATTCACACGTTGATAATAACGGTGGTTGTAAGCGTAGGCGGGATGCACAG

TTCCATCCTTCTCCTTTTGAGAGATCTTTCTCTGCTGTCAGTCCTATCTGCAGCCAAGTT

GGAGATGGCATGGATACAAACTAG

>MS.gene009973.t1

ATGGCGGGGACAGAGGAAGAAAGCGCAGTGAAGGAGCCTTTGGATCTCATAAGGCTGAGT

CTCGACGAACGTATCTACGTTAAACTCCGTTCTGATAGAGAGCTTCGCGGAAAACTTCAT

GTAATTTCTTCTACTTCCTTTCATCTCTTTTTTCTTTCACTGCTTGATTTCAATTTCAAT

TTCAATTTCAATTTCCAGGCTTATGATCAACATCTTAATATGATTCTTGGTGATGTTGAA

GAAATTGTTACTACTGTTGAAATTGATGATGAGACATATGAAGAAATTGTCAGGCATGAG

AATCATGGAATGCTCATGAACAAGGGTTATACTTATGGCAGCTTGGAGCTGTTGTTACAT

AGATGTGGCTCCATAAGCTTACACGCCAATGATTCTTGA

>MS.gene009985.t1

ATGGAGGATAGAATTAGTCAATTGCCCGACGACATCTTGTCGTATATCTTTACAACTTTA

TCCATGGAAGATTTGTCGAAAGCCATCTTAGTATCTAGAAGGTGGTGCAAGCTTTCTGCT

CTAAGGATAGATCTCTACTTTCATATCTTCAATGTGCTTGGCAGCAGTGAGAAGGAACTA

CTACCAGCCAGGTATCTCATGAATTTCCCTATCACAAAACTCGGGTTTCTCCCTACTATA

AAGAGAGTCATCAATTTAGATGGGAGTGCAAATGAATTCGTAAAACGTGTAGATCAGTTC

GTCAAGAATTTTCAGGGCACAATCGTCGATTCTTTCATGGTGAACTTTCATTTGGATTAT

GAACAAAGCAACACTATTGATCAATGGATACTTTTTGCAATTGCAAGGCGAGTTAGAAGT

ATCAATCTTCTCTTTCTAGGAAGGACTAATGTACATCACACTACTCGACACAATCGTTAT

AAATTTGACTTTGCGTTATTTTCGAAGACTAATGCTTCATCTCTAAACCATCTTCGGCTT

GAAAATTGTCTTGTTTGCCATCCCTTTAATTGCGACTTTATTCCATTCAAAAATTTGAGA

TCTCTATCGCTTGAAGAAGTAGAATTGGATGAATCTTTCATTGAAAATGTGTTATCTAAT

TGTCTACAACTTCAAGAACTTTTTTTATTTTTATGTGAGTTCAAATCATCAAAGTTGGAG

ATAATTATAGGGTGCTATATGGTATCCAATAATAAAAATCGGATGGAAGTAAACTTGATT

TTACTGGATTGCCTTAAACTACGTTCATTGGTTTACCGTGGATATATATGGTGTGGATTG

GATATCGTGAACATTAATACCCCTATGGTGAAGAGCATCGACTTCTCCATTTCATTCAAT

GAAGATCTTAATGCATTTGCTCAATGTGCAACTTTTCCTGAACTTGAGATTATGCATGTG

GACATATATTCGACGGTTGCAGCCCCAAAAACAACTCAACCATTCAAACATCTTAGACAG

TTGAACCTCATCCTTTCCTTGAGCTGGGAAATCTTAAATGATGTAGGGTATGATCTTTTG

TGGATTTTAAACATCCTCCAAGCTTCTCCTATTTTGCAGAAACTTTTAGTCATGGTGAGT

GAACAAGGTTAA

>MS.gene009978.t1

ATGGAGCAAGAAAACCAACATCCAATGATGATTGTGCATGATAATGAAGAACAACAAGAA

GAAGAAGTGGAAGATTTTTCTTTTTCTTTGAAAAGAACCTTTCCACATGTCAAGGACAAT

AACAACAACGTTGTTACAACTGTGGTTGTGACATCCTCTTCTGTTAGCAATGGTAGTAAT

GGTCATAGCAATGAAACTTCAACAATTGTCACCGACTTAGATGATTTTTCTTTGCAAAAA

GATGAACAAGTTGAAGAGAAAAGAAATGTTGGTGAATGTAACAGTGTGAAAGAACAAGAT

TGTGTACCTGTTGGACTTGGACTAGTTGCTGAGTTTGTTGGAGAAGAACCCATAACAGAT

GATGCTATTTTTGGCTCTGAATCTTCTGATATATTCCTCCAGAATAAGAACAGTGTTTAC

TTTGACAAACAACAAGGAGTATGGAAATGTCACCATTGTACATGGTCGACCAAGCGATTC

GACAGTCCTAGGACTGTTCCCATGTGGAATCTTAAAGGGTACCCTGACTTGCTGATGAAT

GTCAAAACTTTGATTCAACATGGACCTTGTTTTGTTTGGGAAACTAAAGGCTATGAAGTT

AATGGACTTGACAGAGTTCACAACGGCGATGCTACCAATCATGAGGAGAGTAAACTTGAT

GAACAAGTGGAAAATTCAGCTATTCAAACTAGTTTACGCGGTGAAGTTCATGGAATTAAT

AGAGTTCAAAATGATGATGTTACCAATCACGAGGAGAGTAAACTTGATGAACAAGTGGAA

GTTCATATACTTCATGGAGTTCAAAACGGCGATGCTACCAATCATGATGATAACGTTCCT

TCCAGTTCAGAATTATCTTCTTTGCATAACTCATCCGATAAACAAGCAACACCTGCTGTG

AATTCTAACGAAGAAAATGGAAAGGAACTTAACTCGATAGAAGAAATTGACCATCAAGAG

AAAGAGTATGATGTTGAGTTAGTAATTGCCAAACAAGAGACACATGATTTGTATTGTCCT

AATTGCAAATCTTGCATTACTAAAAGGGTTATCCTCAAGAAAAGAAAAAGGAACATTCAT

GTATTAGACAAGAAAGGAAAACGCGATAGATTGGACATTGTAGTTGACAATGATGTGGTC

AATCCTGATAGTACTACACATGAAGCTAACCAAGGTAATTATGCAAAAGTAACATCTGAA

ATTACCAGTCTGGATCCACCTCCTGCTCCTGCTGCTGCTGCTGCCGACGATGATGATCAT

CCTGAGAAAGAAGTGGAAGTATTCAGATGTTTATCATGCTTCAGCATTTTTATTCCTAGT

GGAAAAGGTTTCAATCTGTTCCGTAATTTTGGTGGTGCCAGCAAGGATGAAACTCCGCAA

AGTTCTTCAAATATAGCTGCAAGCAGTTTGCAAAATCCTTCAAATATACCAAGCTCCAGT

TCAAATTGGTTCATCTCTTTATTTACTTCAAATAAGAGGAAAACAGCTACTGAACCAGCA

GGTGACACTTCCCAAGAGCATTCTACCACTGGTCCTGCAGACCAGAACCAGTCAACAATT

ACTTCACCTTTACTATCCTCCCCTGATATTGGTCATCCAGAAGGTACACTTGGTGATGCA

CATCTGATTAAGAATGTGAAACCGACGTCAGATGTTAATCATGGACGTGGAACAATTAAT

TCTACAAGTTCCTCAAATGGAGTGCAGAATGTTGTGCAGGATTTCATAGATTTTTCAGAA

AAAGAACAATCGCTGACCAGAAAACCTAGGACCGATAACAGAGAGAAAAACAAGACTTCA

GTGGATACAACAAATACAAATACTGTTGAAGTTACGTCCTCAACGAACTTTTCTAACGGG

ACGGTATCTGAGTACAAAAGTGTTAAATCAGTAACTACTACTTCTAGTGAAACATTTGTT

AATTCTGGAGCAACTGCAAAAGGCACCATTCTGAATCATTATCAAGAGAAACCTGAATTT

CATGTTCCGACAAGTACAACCGTAGCGTCCCTAATTGTCGAGAAGTTACCGAAAGATGTG

AGCAAAATGCCTGAAATTGTTAAGAATAATGATTATGATTCCTCTTTGAGGCAAGATGGA

GCACAACCACCAGTTCAGTCACTTGACAGCACAAGTTCTGCAATAGATGCCATATTTCCA

TCAAAAACAGATATTACATTGATTGATAAAGTGCGAAAAGATATCAATGGGAAGATAAAT

CCTTCTATTATAAATGAAAATACAGGTGATGTGATTGTTGTTGTTGATGAAGACAAAAAT

GAATTAACAACATTGCAGACAGAAGATAATGTTACAAGGGATGGTGCTATTGTGACAGAA

TCTCAAACTCAAGTAGTTATTGGTGAACAATCTAGAAATGAAGTTGGTGAACCAAAAAAG

TGGGAAATTGTTAAAAGCATTGTGTATGGTGGCTTAGTTGAGTCAATCACAAGTCTAGGA

ATTGTTTCATCTGCAGCTAGTTCTGGTGCTACCCCATTGAATATTATTGCATTGGGATTT

GCAAATCTGATTGGTGGACTTTTCATCCTTGGTCATAATCTTAAAGAGTTGAAAGATACC

CACTCTAGAGGTCAACAACTGCAAACCAATGTGGTAGATCGGTATCAAGAACTACTAGGA

AACAGATCGAACTTCGTGTTTCACGCCGTTATAGCTGTTTTTTCATTCCTACTATTTGGT

TCTGTCCCCCTCATTATCTACGGCATCTTGATTAATAAGAACTACTATGATGAAGTTAAG

GTTGCAATAGTGGCAGCAACTTCTGTTGCATGCATCATTCTGCTTACTGTTGGGAAAGTT

TACACAAGCAGACCACCTAAATCCTACATCAAAACTGTGTTATACTATGTCACAATGGCA

CTTGCAGCCTCAGGACTATCTTACATAGCAGGCAAACTACTCAAGGATCTTCTAGAGAAA

TTCAGCCAATCAGAGTCAGGTTTTGCTATTAACATGCCAATATCAGACACAAGTATGGAA

ACAGCATGGATGTCTTACTGA

>MS.gene009977.t1

ATGTTATCATCTTGCAATTGCAATTGCAATAGAATCATATTCTCTTTCCCCATTAACAAA

CTATTATTGAAACATACACAACTGAAACTGAAACAGAAACAGAGACAGAGACAAACCGTG

TTGGTAGCAGCTTCCATTCATCACGACAGCCTCAGAGTTCTCGAATGGGACAAACTCTCC

GATTTGGTCTCTTCCTTTGCCACCACTTCCTTGGGTCGTCATGCCCTCAAGGATCAACTA

TGGTCCCAGAATCAAACTTACGAAGAAAGTCTCAAACTTCTTGAAGAAACCAATGCTGCA

GTTGAAATGCACAAACATGGCTCTTGCAGGTTGCAATTTGGACATATTGATGCTATAATG

GTGCAAACTGCAATTCAAAGTGCACGGAGAACCATACTGGTAACTGGCTATGAGGCAAAT

GCTGTTTTGGCTCTTCTTCAGACTGCTGACACCTTGCAGGGTGACTTAAAAGCTACAATT

AAGCAAGACAAAGATTGGTATAGTCGTTTCATGCCTCTTACAGAAGTGATAATGGATTTT

GTCATCAATCGATCTTTAGTTAAAGAGATAGAACAAGTTATTGATGAAGATGGATCGATC

AAGGACTCTGCGAGTTCGGAGCTTAGAAAGTCACGTCAACAAGTACAAGTGCTTGAGAGA

AAGGTACAACAATTAATGGAAAGCTTAATCAGAAGTGAAAGGAGTGAAACATCAATTCTC

GAAGTGAATAACATTGATGGCAGGTGGTGCATAAGAATTGATTCTGGACAGAAAACAAGT

TTTAAGGGTCTATTGTTGTCCAGTAGTTCAGGAGTTGGAAGTACTATCGAGCCACTTTCT

GCTGTTCCCTTAAATGATGAGTTGCAGCGAGCAAGAAGTTTGGTAGCAAAGGCTGAGGCA

GATGTGCTGTTGGCATTAACCAGAAAGATTCAGCTGGACGTTGATGATATTGAAAAGATA

CTAGACAGTTTGGTTCAACTAGATGTGATTAATGCCCGTGCTACCTATGGTCTTTCATTT

GGAGGATCAAATCCCAATATATTTCTTCCTGATAGGAATAGCTCTTCTACTGCTGAATCC

TTAACAAGGAATGACACTTTGAATGGACCATTACCTGAAAATAGGGAATGGATATTGTAT

CTTCCCAAAGCTTATCATCCTTTATTGCTCCAGAGCCATAGGGCAAATTTAAAGAAGGTT

AAAGAGGATGTCAATATTGCTACTTCAGTTTCTGCACTGGACAAAGCCCAACCACAGCCA

GTACCAGTTGACTTTTTGGTATCTAATAAAACTCGTGTTATAGTTATAACTGGCCCTAAT

ACTGGCGGTAAAACGATATGTTTGAAGACTGTAGGATTGGCTGCTATGATGGCAAAATCA

GGTCTCTATGTTCTTGCTTCTGAATCTGTGCAAATTCCTTGGTTTGATTCTGTTTTTGCT

GACATTGGTGATGAGCAGTCCTTATCACAGTCGCTGTCTACGTTCTCTGGCCACTTGAAA

CAGATCAGTAATATTAAATTACAATCAACAAGGCAGTCACTGGTGCTACTAGATGAGGTT

GGAGCAGGAACAAACCCCCTTGAAGGAGCAGCACTAGGAATGTCATTATTGGAATCTCTT

GCTCATGACGGTTGTTTATTGACTATAGCAACGACTCATCACGGTGAACTGAAAACCCTG

AAATACAGTAATGAGGCCTTTGAAAATGCATGTATGGAGTTTGACGAAGTGAATTTGAAG

CCAACTTACAAGGTTCTATGGGGCATACCTGGGCGCTCAAATGCTATTAATATAGCCGAG

AGACTTGGACTGCCCTCTGTTGTTATAGATGCTGCTCGGAAGCTATATGGTTCTTCTAGC

GCGGAGATTGATGAGGTAATTACTGATATGGAGAAGTTAAAACAAGATTACCAACGGCTA

TCAACTGAAGCAGATCATTATCTAATGCAATCCAGAGGACTTCACGATAGTCTATTGAAA

ACCAGAAGGAAGATCGCTGAACATAGTACAAGTCTAAGACTTAAAAAACTGAGAGATGTA

TCCGAGGCTGCAGCAATGTCAAGATCCATTCTTCACAAGAAGGTCAGGGAGCTTGATGCA

TCAGCTAAGAAAACCTTACAGCACGGCAAAGCCATTAAGAGCTCTCGTGTATCAACAACA

AACAACCTCCGTACTGCAGCAGATAATAAAGAACCTACTAGTACAAATAAAAGTCCATCT

GATGTTAAAAAAATTGACAAATCATCAACAGATAGGTCTGCAGTCCCGAAGGTTGGTGAT

ACAGTATATGTCTCTTCCCTTGGGAAAAAAGTGACAGTTTTAAAAGTGGACTCACCAAAA

GGAGAAATATTAGTTCAAGCTGGAATCATGAAGCTGAAGCTAAAAGTAACTGACATTCAG

AGATAG

>MS.gene009953.t1

ATGCCTGTCATTTCTACTGCATCTTCTGCATCTTCTAGCCAGGGATCACCTGGGTCGTCC

CAGGCTAGTGTAGTTGCTCCTGCTATGGGTTCTGCTCCTCCTCCCACTTACATGTGCCCT

CCAGTACATCCAGGATATTATGGCACCATACCTCCTTTTGGGACCTTCCTTCCTCCGAGA

TATCATTTCTCACCATATTCAGTCCCACCACAAAACCTTCCCCTTCCCCAACAACAACAA

ATCCCTCCACAACAAAACCCACCCTTTCCCCAAACAGTTAATCCCTTCATAACAAAACCC

ACCTTTCCCCAACAACAACCAAACCCACCACAATCCCAACAAGAAAATGTGGTCCAAGAG

GGTGGTGGAAAACTGGTGCTGTTTGATGATGTTTTCCCGGATTATCCGAGAGATTCTCGT

CGTCGATTAATCTTAAAGCCTAGAGGAAACTCAGCTGCTGCACGTTTTTCGGATTTACTT

AGAAGAGCCCGTATTCGGTTTGAGGAGACAAGAAAGCGGCCACATTGGATTGACCAGCTA

TCTTTGATGAATTGGTTAAGTACTGGGCGTCTGAGGCGTTCAAGGAAAAATCCGACAAAT

CAAAGAAGAATCGGGCGTCGGAAAAGGCTAGGAGGTTCCGTCGGGATCCGTTTATTAACG

AGCTTCACGACGAAATGCACCAAAAATAGGTCCGGCCAATACTGCGACGAGCGTGCTAGG

AGAGTGCAGGGGGAGTATGACCGGCAGCTTGCTGAGGCTATTTCTCAAGGGTCTACTGTT

GATCACTCTTTGAGCTTCAGCACTTGGAAAAAAATTGCTGGCGACAAGAGGAAGGGAAAA

CTACATGGTCTTGGTAACTTAGCCGCTAACTACCGGAAAGGAGCTTATTCACAGATTGAC

ACGAGCAGTTCACGCAGCAGATGGCGAGTCAAGCGGCCGTTATACAAGATTTGCTTAACC

GCCAAAGATCATATGAGGAGCATCTGGCTCAGTTTAGACAAGCACAGGCTCAAGATCCCT

CCCACACTAGTGCACCGGTTTTAA

>MS.gene009986.t1

ATGGTGAAAATATGGATTGAAATATGCATAATATCAGCTCGTGGAGTAAGAGCATCACAT

TCACTATGGAAACGTCAATGGTATGCTACTGGTTGGGTTAATCCAAACAACAAATACATA

ACCAAAGTAGATGCTTCAACCAACACAAACCCTCTTTGGAGAACAAAGTTTTCTATGCAA

CTTGATAACTCTGATCCAAATTTCCATGATCTTGCATTGAACGTTGAAGTTTATAGCAGA

GATCCTTTTTTCTTTACGGAGAAACTTCATGGTTCAGCTACTATTCTTCTCAAAGAGTTT

CTTGCAAAAGGGTTGCAGAATGATGAGGGTTTAAGGCAAGGGAGTGAAGAAGTTGGAAGC

TATCAGTTAAGGAAGAAGAAATCTGGTAAACCAAGTGGGTTTGTTGATGTTTCGGTTCGT

GTTTCTGAAGAAGATGAACCAAATTCTCACTCAGGTAATGGAGGAGGAATAGAGCTCTTA

GATAATGGTAATAAGGATAGATTTGGACAAGGCTATCACCAACAAATGGATCCAGCTTCG

TTCAATGGACCTCATAAACAAGCACACACTAATGTACCCTATTCACATCCAGTGCCATAT

CCTACAAACTATTCTAACCCTTATGTGGGTGGACCAAGCTACCCTGCAGCTTCTGGACCA

AGTTACCAACCACCAAGAGCTCCACCACCACCACCTCCACCTTCAAATGTTGGTTATGCT

CCCAATTTTCATCAAAGCAATGATGGATTGACACCGAGTTATTTTAATATGCCATCATCA

TCAGGGACAGCTCCTAGGCAGAGGGGACCTCCAGGATTTGCAATGGGAGCAGGTGCTGGA

GCATTAGCAGCTGGTGCTGTTATGTTTGGTGATAACTTCATGTCAGGATTTGATGTGCCT

TCAGGTGCTGGAGCATTAGCGGCTGGTGCTGTTACCTTTGCAACTGATCCTCTTTTCTAA

>MS.gene009963.t1

ATGATAACTTTGAATAAAGAGATTGTTGATCTCATCGATTATGAAGAGGGAGATGGAAGA

ATTCATAACATTCACTTTGCATCCATTCGCGAAACCGACGACAAATGTGAAACATTTCAT

AGGGGAAAGAATGTTGTTCTTTTCTTAGAGGAACGTGTTAAGGGTGTCCAATTTACGCCC

TCAAAATCTATTGATGAGTGCCTTAACTTGTATGTCTTTCCGAACATTAAAGGATTAAAA

AGAAAGGCACGGTACCTGGCATACATGGTGAAGGTTCTATTACTGGCCGCTGAAAAACGG

ATAACAGGGATGACTTTCGAAATAAGAGGCTTGAGTTGGCTGCGAATGGGGAAGGCTCTG

CAGAGAGACCTCTATGGAGATCAAGATGTTCGTCAAATTGAGCATTATCTGACGCTTCCA

TTATTACAAATGGTCTCTAAAGAGCGTTCTCTACTGAAACATGGACACATCCTTTTAAAT

GGATGGAAAGGATTTCTGGTGTCGTTGCAATTCTTTGCCGAACTAATCCATTGCAAACCA

TGGCTGAATTAA

>MS.gene009951.t1

ATGGCTAATCTTGCTGAGTTTTCTCCAATTTCTGAAATATCAACTGAAAAAAATTTCTGG

AATGTGAAAGCTAGGGTCATTCGATTATGGCAAGTCTCTGACTTTAACAGGAACACTTTG

CCATTTTCGACTGAGATGGTATTGATGGATGAAGCTGGAAACAGAATCCATGCTACCATA

AAAAAAACCCTTCTTTACAAGTTTAAAAATGACATTTTTGAAGGGAGATGCTTTAGCTTT

GAGAATATGGGTGTTGCTAACAATGGAGGTAACTATAGGACCACTAGACACAACTTTAAA

CTGAACTTTCAGTTTGGCAGCAAGGTTGTTTTTCTACCAAATCTCTCAATCACCAAGTCT

CCATATAATTTTGTTCCAATCCCTGAAATTGTTGGAGGGGCTTATGACACGGATTACTTG

CCTGACCTCTGTGGATTTTTGACTGGTGTTGGTAGTGAAAGGGAAATACAAAAGGGTGGT

AAGACTTACAAGTTGAATGTCATTGAATTGGAATCACTTGGGCATAAGGTTCAGTGTACT

CTGTTTGGTAGCTATGTTGATGATTTAAACAACTTTATTGCAAGTGGTGATGTCCAGAAT

GCCATTGTTATCATTCAACTTGCTAAAGCAAAAATCTTCCAAGACAAGCTTCATGTCCAG

AACTGTTTGAATTGTACAAGAGTTATTTACAACCCAACATGTGCTGATGGTTTGACGCTA

AGGAATAGGCTTGCTGAGACTCCTGACACACCTTCACCACTATCTCTAACACAACTGCTA

CCTCAAACCAAAGTTGACCCAAAGGAGGAGTTCCTTTATCATACTCCTAGAGCTACCATG

CAAGGCCTTAAGGATGCCTCTACTGATTCTGTCTTTGTTGTTGTGGGCACCATCAAGAGA

GTGGTGAACAAGGAGAACTTTTGGTACACTGCCTGTGTGTGTTCCAAGGCTGTCATTCCA

GATTCATCTATGTTCTTTTGTGAAAAGTGCGACAAGCACGTTAAAAAGGTTTTTCCTAGG

TATTGCTTGAGGGTGAGGGCCATTGACCAGACAGATTGTGCAACTCTGGTCATCTTTGAC

AGAGATTGCTCGACCCTCTTTAATAAGAGTTGTGCTGATCTCTTAGCTGAGCATGAAGTG

GCTATCCATGAGGGTCAGCTTGCACCTGAGCTTGCTGCACTTATTGGGAAGACCTATTTA

TTCAAAGTGGAGACCAAAGCAGATTTCAGTCCACGATTTGAACAGTCATTCCGTGTGCGC

AAGATATGTCTTGACCCTGCAATCATCAAGGAGTTTATGTCAAAGTGGGAGAAGGAAGAA

TCCTCATTCCTCAAGCTAACTAATGAGAGTGCATCTCTTAGTGTGTTGATGGACAAGGGC

AAGCATACAATGCACATAGGAGAATCCAGTCTTTTATCTCAAGATCCAATTTCCCTATCT

GAACCTGTGCAGAAGATTCAAAGGCAGCTTGCTAAGGTTCCTGAATCTGCTTCTGATGTG

GTCAAACAGGATTTGATGACTAAGTTTGGAGGTGTTGATTCAGAGCCTGATTGCCAGGAG

ATACCCCCCTCACATTCTGCATCTTCATCCAGCAAAAGGGCATCTCCCACAAATGATGAT

GATGACATGAATGTTCCTATTAAGAAGCTGATTAGGAGGACTGTCAAGATTGAGAAGATT

TAG

>MS.gene009968.t1

ATGCCGAAATTCTCCATCATTCTCACCTTCATCGCTCTCATCATCATCTTCCCAATCCTC

ACAACAGCTGAACACGAAGAATCATCCGTTCTCCGGTTACCGTCACAAAACGTCTGTTCC

GGAACAACGCCGTCATCGTGTCCGGCGAAATGCTTCCGAACAGACCCGGTTTGCGGAGCC

GATGGTGTGACATATTGGTGCGGTTGCGCTGAAGCTGCGTGCGCCGGAGCTAAAGTTGCG

AAATTGGGTTTCTGTGAAGTTGGGAATGGTGGATCTGCAACGTTTCCTGGTCAGGCTCTT

CTTTTGGTTCACATTGTATGGCTCATTGTTTTAGGATTTTCTGTATTGTTTGGATTTTTT

TAG

>MS.gene009999.t1

GTGGAGGAGGTTGGTGCTGGTACAAAATCAGGTGCCTTATGGAGAATTCTGGCTACAAAG

TCTCTTGCATAGACCTCAAAAGTGCAGGAATTGATCAATCTGATGCTGATTCTGTTCTTT

CTTTTGATGATTATAATCAACCCCTCTTGGATTTCATGTCTTCTTTGCCTGAAAATGAAA

AGGTAATATTGGTGGGACATAGTGCAGGAGGGTTGAGTATTACCCATGCTTGTCACAAAT

TTGCCAAGAAAATATGTTTAGCAGTGTATGTGGCTGCAACTATGCTCAAATTGGGATTTT

GTACAGATGAAGATCTAAGAGATGTGAGTACTTCATTTTCCTATATCAACAACTAA

>MS.gene009990.t1

ATGAAAATCCATCACATCCCTTGCCTAGAAGACAATTATTCATATTTGATTGTTGATGAG

ACTACTAAAGAAGCTGCTGCGGTGGACCCAGTTGAACCCGAGAAGGTTCTAGAAGCTTCT

AATTCGCTTGGTCTTACTATCAAATTCGTCCTCACTACTCATCATCACTGGGATCATGCT

GGTGGAAATGAGAAGATAAAGCAATTGGTGCCTGGAATCAAGGTCTACGGTGGCTCAATT

GATAATGTCAAAGGTTGCACTGATGCACTTGAAAATGGTGATAAGGTGCATCTTGGAGCA

GATATTAATATTTTGGCCCTTCACACACCTTGCCACACCAAAGGTCACATAAGTTATTAT

GTTACTGGCAAAGAGGATGAGGACCCGGCTGTTTTTACTGGAGATACATTGTTTATTGCT

GGTTGTGGGAAATTTTTTGAAGGAACTGCAGAACAAATGTATCAGTCACTCTCTGTAACA

TTAGGTTCATTACCAAAGCCAACTCGAGTTTACTGTGGCCATGAGTACTCAGTGAAGAAC

CTACAATTTGCTCTAACAGTTGAGCCAGACAATTTAAGGATACTGGAAAAATTAACCTGG

GCTCAAAATCAGCGGCAAACTGGCCAACCAACAATTCCTTCAACCATTGGGGACGAGTTG

GAAAGCAATCCATTTATGCGGGTTGATCTACCTGCAATCCAGGAGAAGATGGGGTTCAAT

TCACCAGTTCAAGCTTTAGGAGAATTAAGGAAGGTGAAAGACAATTGGAGGGGCTAA

>MS.gene009988.t1

ATGAAACAGGGCAAGAAACTTAGGATTCTGTATGCAGGGAAGGTTGTTCATGGACTTTGC

ATTAAGGATGTTTTTGATTTTGATAACTCAGTTAGTAGTGCATTTGCTGAGTATTATTAT

GTTTGTGATGCTGTAGACGATGCAAAGAGAGTTTATGAGAACATGGTAGAAGAAGCTTGT

TTGAACGTTGCTGACTCGCTGATTGGTGGCATTGTCTCGATGGGGAGGATCAAAGAAGCT

GAGATGATCTTTATGGATTGA

>MS.gene009995.t1

ATGGAAAATGCTTATGGAAGGTTTAAGAGATCTAGGTTTTTGATTGATGAAAATGTGATT

AGGGCTATGGCTTATGCATATATAAAGAAGAGAAAATTCTATGAATTGGGTCAGTTTGTT

AGAGATGTTGGTTTAGGTAGGAGAAATGTAGGGAATCTTTTGTGGAATCTCTTATTGCTT

TCTTATGCTGCTAATTTTAAGATGAAGAGTTTGCAGAGAGAGTTTGTTAGAATGGTTGAA

TTAGGGTTTCGTCCTGATGTTACGACGTTTAATATTCGCGCTTTGGTGTTTTCGAGAATG

GCGCTGTTTTGGGATCTTCATCTTAGTATTGAACATTTGAGAATTGAGAAGGTGGTTCCT

GATTTGGTTACTTATGGTTGTGTTGTGGATGCTTACTTGGATAGAAGGCTTGGAAGGAAT

TTGGAGTTTGTTTTGAATAAAATGGATGTGGATGATTGTCCACAGTTGTTGACGGATCCG

TTTGTGTTTGAGGTTTTAGGTAAAGGTGACTTTCACTTGAGTTCGGAGGCATTTATGGAA

TACAAGACACAGCAAAATGAGTGGAGTTATAGGGTTTTGATAAAGAAATACCTTAAGAAA

TATCATCGGAGGGACCAAATATTTTGGAACTACTAA

>MS.gene009989.t1

ATGGAAACTTTGGGTGGTAATTCATCATCATCACCATCATTATCATCAGCATCGTCATCA

TCATCTTTTGGAGGGATTAATACTGATGATAACAATGTTGGTGTAACTTGGTATGGAATG

AGGTTACCTGTTAACCCCTTTTTGTCACCGATTTCGTTTTTGTTGGATTATTCTGGAATT

TTGCGTTCGAGTAATGATTCTGAGGGTATGATTGTCAACAACGGAGTTTCTGGTTCGGAG

TTACGATCTCAGGTTGATGCTGGTGGTGCTGTTGCTGGGAGTAGTGCAGGGGAAGTTGCA

ATAAGGATTATTGGAGCCGGAGAGAATATTCATAATCAGGTTGGGGAAGTAGGTTATGAT

GATTGTGGTGAGGAGCTTATTGGTGAGAGAAGTGGAATGCCTGGATTGGGTGAGAATGAT

GCCGAGGGTCGTGGTGGAATTGAGGCTAGTGAAGGGGTTCCTCTTGTGTCTTCATCTTCT

TCGTCGTCGCTGGCTGGTAGTGGACAGGTTGATGGTGAGGCTGCTGGGAATGGAACAGAA

AATAACAGGGATTCGTCTTCTCATCAGAGATATGATATTCAGCTGGTTGCCAAGTGGATC

GAGCAGATTCTTCCTTTCTCGCTTTTGTTGTTGGTTGTTTTCATTCGACAGCATTTACAA

GGTTTCTTTGTCACAATTTGGATAGCAGCTGTGATGTTCAAGTCAAATGAAATTGTTAAG

AAGCAGACAGCTTTGAAGGGAGATAGGAAAGTCTCTTTACTTGCTGGTATCGCTTTTGCT

TTCATTCTTCATGTGATATGCATTTACTGGTGGTATCGAAATGATGATATTTTATATCCA

CTGGCCATGTTACCTCCTAACCCAACACCTTTCTGGCATGCAATATTCACGATCTTGGTC

AATGATATATTGATGCGGCAAGTTGCAATGGCTTTTAAGTGTATCCTTCTTATATATTAC

AAAAAGGGGAAAGGTCATAATTTCCGTCGACAGGCTCAAATGTTAACTCTTGTTGAGTAC

ACGCTGCTGTTGTATCGTGCCTTGTTGCCAACACCAGTCTGGTACCGGTTTTTCTTGAAT

AGGGAATATGGAAGTCTCTTTTCGTCACTGACCACAGGGTTGTATTTAACTTTCAAGCTA

ACATCTGTGGTTGAGAAGGTCCAATGCTTTATTTCTGCCTTGAAAGCATTATCAAGAAAG

GAAGTTCATTACGGGGTCTATGCCACAGCAGAACAGGTTACTGCTGCCGGTGACCTATGT

GCTATATGCCAAGAGAAGATGCATTCCCCTATCTTGTTGCGCTGCAAACACATTTTCTGC

GAAGATTGTGTTTCTGAGTGGGCTCTGCACTTGCAGAAAAATCAAACGGTTTTTGATCTG

ATTTCAATGATGTCACAGTCGAGTGAGGTTCTCGACATCTCGTTGAGGCTTAGGTGCCAA

CGAGAAGCAGCTCTGAGCAACGCTCCTGTCACTACTGATTTTGCCTCCGAGATATCCTGA

>MS.gene009993.t1

ATGCTGCGTTACTCAAACCGGTTGATATTTGGACTAATGGAAACATTCCCTGATTTTTTT

ATGTGGAATCCGCTTAATGCAACGGATGTGGATAATTACAACAAAGTATTGAGAAAATTG

TTACATGATTTGAAAAATCAAGCTGCAACTGGTGATTCTATGAAAAAATATGCTACCGCA

AATGTAACAGGTCCAAATTTTCAGGATATATATAGTCTAATGCAGTGTACACCTGATATA

AATTACTTGGATTGCAATCAATGTTTGGTTCAAGCTATAGCTAATCTTCCATCTTGTTGT

GGAAATAAGATAGGTGGGAGAGTTATTAGACCAAGCTGTAATCTTAGATATGAAAATTAT

AGGTTCTATGAACTTAATGTTCAACCTCCGCCGCCGCCACCTACTTCACCATCTACTAAT

CACAAAGGAAAGGATCACACATCAAGAACTGCCATCATCATAGCAGTCCCAGTTGTTACT

ATTGTTTTGTTGCTATGTTTCATCTGCATCTATTTAAGACTAAGAGTGAAGAAACCAAAT

GAAAGTACTAAAATTCCTTCAGAAGATGATGAAGAAATTACAACCTTTGAGTCATTACAA

TTACCCTTTGACACTTTAAAAGTTGCTACAAATGACTTCTCTGATTCTAATAAACTTGGG

GAAGGCGGATTTGGAGCTGTATATCAGGGTAGGCTCTCTAATGGACAAGCGATCGCTGTT

AAAAGGTTGTCCATTAATTCTGGCCAAGGAGATAAAGAATTTAAGAATGAAGTGCTTTTA

ATGGCTAAACTTCAACACCGGAATTTAGTTAGACTACTTGGTTTTACTATTGAAGGGAGA

GAAAGACTACTTGTCTATGAATTTATTCCCAATAAAAGTCTTGACTACTTCATATTTGAT

CCACTGAAGAAAGCACAATTAGATTGGAAGAAGCGCTTCAAAATCATTCAAGGTATTGCT

CGAGGCGTTCTTTACCTTCATGAGGATTCTCGACTGCGTATTATACACCGCGATCTCAAA

GCAAGCAACATTCTCCTAGACGAAGACATGAATGCTAAGATTTCAGATTTTGGCATGGCA

AGACTAATTCTATTGGATCAAACCCAAGCAAATACAAGTAGAGTTGTTGGAACCTACGGA

TATATGGCACCTGAGTATGTAATGCATGGAGAATTTTCAGTGAAATCAGATGTCTTTAGT

TTTGGTGTTCTGGTTCTTGAGATTATAAGTGGCCAAAAGAATAGTTACATTCGTCACGGG

GAAAATACGGAGGATCTATTGAGCTTTGCATGGAGAAGTTGGAGGGAAGGAACAGCTGCA

AATATTATTGATTCATCACTATACAACAGTTCAAGAAATGAAATCATGAGATGCATTCAT

ATTGGTTTACTTTGCGTCCAAGACAACGTAGCTAGAAGACCAACCATGGCCACCATTGTA

CTCATGCTAAGTAGCTATTCTCTCACTCTCTCTTTACCTTCAGAACCTGCATTTTTTATG

GACAATAGAACTAGAAGCCTTCAAGAGATGAGGCTGTGGGAAGAGAATTCAGGGACAACA

AGATCGAGTCAATCCACAAGTAAATCAGCTACAGAATCAGTAAATGAGGCTTCATTTACT

GATCCATATCCTCGTTAG

>MS.gene009976.t1

ATGGACAAGAAAATCCTTGATCTCATATTAGTCCCTAGTGGTCTCTTAATAATGGTTGCA

TACCATTTATGGCTTCTCTACCAAATCGTTAAACACCCCACAAAAACTGTCATTGGTGTC

AATTCAATCAATCGTCGTTATTGGGTTCAAGCTATGATGGAGGATGTGTCCAAGAATGGT

GTTTTGGCAGTGCAATCTTTGAGAAACAACATAATGGCATCAACCCTTTTGGCTTCAACA

GCAATAATGCTAAGTTCTCTAATTGCTGTGTTAATGAGTAGTAGAAATGAGGGGAGAAGT

GTTGTATCATTAGTGTTTGGTGATAGAACTGAGCTAGTTTTATCAATCAAATTTTTCTCC

ATATTGGTTTGTTTCATGTTAGCTTTCTTGTTGAATGTTCAATCTATAAGGTACTATAGT

CATGCAAGCATACTCATCAATGTTCCTTTCAAGAAATTGTCATCAAACCTTAGACAACAG

AAGCTGACAGCTGAGTATGTTGCCAATACAGTTAACCGTGGAAGCTATTTCTGGTCACTT

GGTTTGCGTGCATTTTACTTTTCCTTCCCTCTTTTCATGTGGATCTTTGGACCTATTCCT

ATGTTGTTTTCTTGTTTTGCACTTGTTTCTATGCTCTATTTCTTGGATGTTACATTTGAA

TGTGGATGGGCTGCTATTGGAGTTGATGATGCTGACTGTGTTGGAGATGAAGTGCACAAG

CAACAACACCATGTTGACATGGAAATGCAAGAGCCAATTAGAAATTAA

>MS.gene009952.t1

ATGGGTGAGGAAGCAAAACAGGAACAACCAAAGGTTGAGGAGAAGCAAGAGGAGAAGAAA

GAAGAAAAGCCAGCAGAGGAAAAGAAGGAGGAAAGCAAAGAGGAAAAGCCAGAAGAAGAG

AAAAAGGAAGAACCTAAACCACCAGAACCATGTGTACTTTTTGTGGATTTGCATTGTGTT

GGATGTGCAAAGAAGATTCAAAGATCTATAATGAAAATGAGAGGAGTTGAGGGAGTAGTT

ATTGACATGGCTAAAAATGAAGTTACCATAAAAGGTATAGTGGAGCCTCAAACTATATGC

AATACAATTACCAAGAAAACAAAGAGAAGAGCAAATGTTATATCTCCATTGCCTCCAGCA

GAAGGAGAACCTGTCCCAGAAGTTGTTAATTCTCAGGTTAGTGGACCAGAGACAGTGGAA

CTTAATGTGAACATGCACTGTGAGGCTTGTGCTGAGCAACTCAAGAGAAAGATACTCCAA

ATGAGAGGTATACTTTGTTTCTTCTTGATTTCAATATAA

>MS.gene009987.t1

ATGATTACTGTGTATTCCAAGAACGGTGAACTTGATGAAGCTGTGAAGCTTTTTGACAAA

ACTAAGGGTGAAAGAAATTGTGTATCATGGAATTCAATGATGTCGAGTTACATTCATAAT

GGCCAGCACAGTGAGGCATTGAAATTATATGTGACTATGAGCAGATATTCATTGCTTCAT

GCACACTTAGCCAAAACGCCATACCAGGAAAATGTTTACGTTGGAACTGCTCTTGTGGAT

TTCTATTCCAAATGTGGTCATTTGGCTGATGCTCAAAGGTCATTTACCAGCATCTTTTCA

CCCAATGTAGCAGCATGGACAGCTCTTATAAATGGTTATGCATACCATGGATGTGGATCT

GAGGCGATTTCACGCTTCCGCTCAATGTTAGATCAAGGCGTTGTTCCAAATGCTGCTACT

CTTGTAGCTGTTCTTTCTGCCTGTAGCCATGCCGGTCTAGTTGACGAAAGTTTGGAATTT

TTTCACTCAATGCAGGTAAACTACAGAGTAACCCCCAACAATAGAGCATTACACATGCGT

GGTGGATCTTCTTGGTTGGTCAGGCCGTGTGAAAGAAGCAGAAGAGTTTATCATACAAAT

GCCTATCAAAGCAGATGGTGTAATTTGGGGAGCTTTGATTAA

>MS.gene009994.t1

ATGAAGTTCCAATTCTTCGTTGTTCCATTTCTTCTTCTAAGCTTCATATCATCTCTCTTT

AACTTCACTCTAGCTGACTTAATCTCAGACAAAAATTCTCTCCTTGAATTCTCTTTTACT

CTCCCACATGCCTTAAGATTGAACTGGAATAACTCAACTCCTATTTGCACTTCATGGATT

GGCATAACTTGCAACCGAAATGAAACTAATGTCATAAGCATCCATCTCCCGGGAATTGGA

TTAAAGGGTTCCATTCCAAACAACAGCAGCCTAGGAAGACTTGATTCCCTTAGAATCCTA

AGCCTTCATTCCAACGAACTTAGTGGAAATCTACCTTCTAACATTCTCTCTATTCCTTCA

CTTCAATATGTTAATCTTCAACACAATAATTTCTCAGGCCTAATCCCTTCTTCTATCTCT

TCAAAACTCATTGCATTGGATCTTTCCTTTAACTCTTTCTTTGGAGCTATTCCTGTATTC

AACCTTACTAGGCTCAAATACTTGAATTTGAGCTATAATAACTTGAATGGTTCAATTCCA

TTTTCCATTAATCATTTCCCTAACAGTTCTTTTGTTGGAAACTCTCTCTTATGTGGTTCA

CCTCTCAAAAATTGTTCTACAGTCTCTCCTTCTCCATCTCCTTCTCCGTCAACTACTCGA

TATCATAAGTCTACAACTTCAAAGAAATTCTTTGGAGTAGCTTCTATACTTGCTTTTGCA

ATGGGAGGAATAGCCTTTCTTTCTCTCATAGTTTTGGTGATCTTTGTGTGTTTTTTTAAG

AAGAGAAAGAGTAACAGCAGTGATGATATACCGAAAGGAAAGACGAAGAATGAGGATTCA

ATTTCAAAAAGTTTTGAGAGTGAAGTGTTGGAGCATGAAAGGAACAAATTGTTCTTCTTT

GAGGGTTGTTCTTATAGTTTTGACCTTGAAGACTTGTTGAAGGCTTCAGCTGAAGTACTT

GGAAAAGGAAGCTATGGAACAACATACAAGGCTAAATTGGAGGAAGGACTGACAATTGTG

GTTAAAAGGTTAAGAGAAGTTGTGGTCGGAAAGAAGGAGTTCGAGCAACAGATGGAGGTT

GTTGGAAGAATTGGAAGACATCCTAATGTCATACCACTTAGAGCTTATTACTATTCCAAA

GATGAGAAACTTCTTGTTTATGACTACATGCTAGGTGGAAGCTTGTTTTCGTTACTTCAT

GGAAACAGGGGTGAAGGAAGAAATCCATTAAACTGGAATTCAAGACTGAAGATTGCGCTG

GGAGCTGCCAAAGGAATTGCTTCCATCCACAAAGAGGGAGGTCCAAAATATTTTCATGGC

AACATCAAGTCGACCAATGTACTCATAACACAAGAACTTGATGGTTGGATCACTGATGTT

GGACTTACTCCCCTTATGAGCACCTTATCAACCATGTCAAGATCCAACGGATACCGTGCT

CCAGAATTAACCGAATCAAGGAAGATTGCCACTCAGAAGAGTGATGTTTACAGCTTTGGT

GTAATCCTTCTTGAAATGCTTACAGGGAAGATTCCATTAGGATATTCTGGTTATGATCAT

GACATGGTTGATCTTCCAAGGTGGGTTAGGTCAGTGGTTCACGAGGAATGGACGGCTGAA

GTTTTCGATGAGGAGATGATTAGAGGTGGAGAGTATGTTGAAGAGGAGATGGTGCAGATG

CTTCAGATTGCATTGGCTTGTGTGTCAAAGGTGGTAGATAATAGGCCAACAATGGATGAA

GTTATTAGAAACATAGTAGAAATTAGGCACCCTGAGTTGAAGAAAAGTACATCTTCTGAA

TCTGAATCTAATGTATAA

>MS.gene009958.t1

ATGACGCAGAAATCAAAACTTTTCAAGGGCCAAACTAAGAAGAAATCGATTCCTGCTAAT

CGTCATGGCAAAGTACCTCAAACTCGCAAAGGGAAGAGATTTGTGAAGCCAACGAAAGTT

ACAAAGGATATGGATGCTGATCGTGAGGTGAGCAAATTCATCAACCATTGCAATGAAGTT

AAAGCAGCGACTGTAGCTACAAAGGACGGTGGTTATCTAAGCATTGTTAAGACAGCACCA

GAGTCTGCAAGTGGTGCAGATAAATAG

>MS.gene009992.t1

ATGAGTGGCTTCCATAGAAACAGCAGCCCTAACCGCAGCCCTGCCGGCGGCTCTAACCGG

AGTGGTGCCGGCGACTCTAACCAGATCCCTGCCGGCGACTCTAACCGGAGCCCTGCCAAT

GTCTCTCATCCCAAACTTGACCTTAAACACCCTTACCCTCCCGAATTACTAAAAAATGTC

AACCCTGACAACGTTAAGGCTCCTTACGCTCTCACCCACGAGTTGAGAGATAAAGTTTGT

GCGGTCCTTGAGAAGCAAGGGAAAGTGCTGTGCGAATCAGGGAAGCATGTGCAAGAAATA

ATTCAAGCTCTTCTACGTAATGATATGGATCCTGATAAGACGGCAGCATTTCTAGGTGTC

TGGCTGCAGTTCTGA

>MS.gene009971.t1

ATGGATTTGCAGAATAATATGCAGCCTGAATTGGGAAAATTGGAGCAGATTGTGTATCAA

TTTCTTTTAAAGTGTTTACACATCATTTTGGACTCGAGGGTACCGTTGTTACGTCAACAT

GATAGAAGCGGTGAGTTATCAATGGGTGGTTCTCGTGTGAGGAGGAGTGATAAATGGTTT

AATTTCGCCTTAGGTGATCGACCGTCTGCTCTTGATAACTTGAACTTTTGGCATAGGAAT

TTGATGGATCCAATGATAATTGACATTATACTAGTTCATCAAGAGAATGGTTCTTCTGTT

GAGACAGTTATTGAGAGGTGGAATATTCAATATGAATGTCCAAGAGGAATGGCTCCTCAA

ACTAGTGACGCTACGGTCTCTTACAAGAAAACATACCAGAAGTCGATTATACTGTTTCGT

GCGCTTTATTCTCAAATGAGGCTTCTCCCAACTCATAAGATATTTAGGCAACTAAGTGCA

ACAAATCAAAGTTGTAATTTTGATATTGTCTATAAGGTATCTTCCTTTAGTGATCCATTT

TCTAGGGAAGAAGGAGGGATGATGGGAGAATATATTTTCACACCCATTGACGCGCTTCCA

GGACGCCTTAGCTTATCTGTGACTTACCGTACTACATTATCTGATTTCAATCTTCAGTGT

TTAACATTACCAACAAAAATAATAGCTGATTATGTTGGAAGCCCGAATACCGATCCTTTA

AGGTATTTCCCTTCTTCAGTAAAGGGTGTTCGTGCGCCTCCGTCTTCTTCGCCACTTGAG

CGCCCTCATAGCTGGACGCCTGGTTTTCATAAAGCAGCGCCTTTTGTACAGAACCATCAG

TATGTTGGATCAACACCGGTGCATCGCGGTTCTCACAAGCCATTTGATTTTCCATCTCCA

CCTACTGATAATTTCAATAATAGATATCATAACCATCGAATACAAAGTCGGTCAAGGTCT

ACAAGTTATGATGAGTATCAACTTTCTCCTCCATTCTCATCCTCACCATCTCCTTCGCCA

CCTATACATATTGGAAATACGTTGCATACCCGTATGCGTTCTGAAACATCACCTGTGACT

ATACCTCATCCCATAATGGGAAAAAGCTCTAGAAATCTTTCTCCTAATTTTTTAGATCCT

AATAGAAATTCTCTACCTCCATTATCTCCCAGAAGGAATGATACATCGTCACATGAGTCT

CCATCTGGAGTCAGGTCATTCAGGAAAATAGAATCTCTAAAGATTGGTCAAAAGATTGTC

AGAGATAGCAAGGATGATTCGGGGCGGTTCTCTGGATTGTTATCTTCTAGTGACTCACCA

CGCATTGGAGCTTCGAGAACATCAAGTAGTAGATTATCTTTTCAGGATGACTTGGATGAT

GGTGACTTTTCGTGTCCTTTTGATGTTGATGATGTTGACCCTCCCAATGTTCTGTCAAGC

CATAATGTGGATGGGAGGAAGAGTGCTTTGGAGTCCCCTTCAACATCATTACCAATAGGG

AGAAAATCACAAGATGCTGCTGTTGGTGTTCTTGTGCACATGCTTAGAACAGCGCCTCCT

TTGCGCCAAGATTCAAGCTGTTATTCATCACATTCCCTGAAGGCCGAGCTCGATGGAGGA

GTTGCTACTGCTTCCGGATTCTTCATGCCTCGAAAGACAACTGACGCACTTGAAGAGCTT

AGGAGTTACAGAGAAATGAGAGACCTTCTTCTTTCTAAGAGTGGTACTCGGATCCTGAAA

AGAGATGAAACTTAA

>MS.gene009984.t1

ATGGAGAATAATAACAACAATTCTTCTTCTTCTTCTTCACCTCCTCCTCAGCCACCTCAA

CCTATCGATATCCTTCTCGATCCTCTGCGACACCTGATTGTTACCAATGATCAAGCAGCA

CTTCACAATTTGCATCTGTTGTTTCCTGAATCAGAGGTTATCGCCGAATCTTGGCGACAG

ATTGCTCCAGCTACTTCACCTACTTCGCTTTGGAGGTACGGATTTATCGGAGTCGACGAG

TCGATAAAGGTTGGGATGAAGAGAAAGGTTGATTTAAGCACTATGTGGAAGTTCTTCGAA

TTTGCCGATAAAAAGCATATATCTGCAACTTCTCAAGATCAAAGAATGGCTGGAATTGAT

TCTTTTCCGGTAACCACTCAGTATCCTAATACTGGGCTACGAGAGATTTATTTACTGATA

CCGGATTTAATGGGCAACGACCCTGTTCTAAACCTTGAAGCGGCTACTAAACTTGGAAGG

CTGCTATCAAGTGGCAAACCTTTACCTAAATTGGTGATTGCGGAAAATGTTCTCCCTCAG

TTAATGACGTTTTTGGTAAGGGATGATATACAACTGCAGCTTCTTAGCTCCAGTATTGAT

CATGCAGTAAGAGATCAGGCATTATGGGCTTTAGGTAACATCGCTGCTGATTCTGAGACA

ACAAGGGACCTAGTTGCAAATCATGGCGCACTTTTTCAATTATTATCTCTCATGTGGAAT

CCTTCCACAAGAAATACAAGTACATGGAATATTGCTACATGGGCCTTCTCAATCATGTTC

ATTGGAAATGGTCCTGTTTCTGTTGAACAGATACAACCAGCACTGCCTGCCCTTCATAAA

CTCTTACATCAGCCTGATGAAGAAGATGCGCTGATTGCATGCCAAACTATTTCTGTCCTT

TTTGAGTGGGGCTCAAACGGTATAATCGGAGCTGTCATTGCAGCAGATGTTTTCCCAGGG

CTTGTAGCATTACTAAAGTTTCCGACGTCCAGAGTTCTGGTACCAACTCTGCAGATTATA

GGATATATTGCTGCTGGTGATTATACTCACACTGAGATTCTAATCCAGAGTGGAGTTCTT

TCGTGTCTGAAATCTCTTGTTACGCATACTGATGAAGTCGTTGCCAAGAAAGTATGTTGG

GTAATATCCAACATAGTCTGTGGGAAAGGTGATCAGTTACAGGCCGTCATTGATGAGGAT

CTTATTAGGCTTCTTGTCAATATTACAAAGATTGAATCCGGCATTAGGAAAGAGGCGGTA

CTAGCTATTGTTAATGCCGTGTATCATGGAGATGAGGAGTCAATTCGGTACTTGGTGAGT

GAAGGGTGTACAGAGACATTATGCGATCTTTTGACATGTACAGACAAGGCTGTGGTGATA

GTCAGTCTAAATGGATTGAAATTACTGTTAGAAGGAGGAGAATTGTCCAATGCACGCATT

AAAGTTGAAGATTGTGGGGGATTGCAGGAGATTGCAAGATTGAAAAGGGATGACAAAAAA

GTCATTAATGAAATGGCTGAGAGCATTTTGAGGAACTATTGGCCCACATATTCCTCGAAA

GGTAAATTGAAAGCAGCTATTGAGGATGAGGAATGA

>MS.gene009957.t1

ATGGGTGCCGTAGTTGAGTGTTTACCGTCGTCGCAACAAGCGGAGGAGCTGGCGGTTCTG

TCACTGAACAAGCTGCCGTTAGGGTTCCGCTTCCGGCCTACTGACGAGGAGCTTGTTGAT

TTTTATTTAAGGATGAAGATCAATGGAAACGGTGATGAAGTTTGGGTTATTCGTGAAGTT

GATGTTTGTAAATTTGAGCCTTGGGATTTGCCTGATTTATCGATCGTAAGGAACAAGGAT

CCAGAGTGGTTTTTCTTCTGTCCAATGGATCGGAAGTATCCGAATGGGAGCAGATTGAAC

CGCGCAACAACTAATGGGTATTGGAAGGCAACAGGAAAAGATCGAAGGATCAAGTCTGGT

GCTACCTTGATTGGAATGAAGAAGACTCTTGTTTTCTACGCTGGTCGGGCTCCGAAGGGG

ACGAGGACTCATTGGGTTATGCATGAATATAGACCTACCTTAAAAGAGCTTGATGGCACC

AACCCTGGACAGAACCCATATGTCCTTTGTCGCTTGTTTAAGAAGCAAGATGAGAGTCTT

GCAAGTTCAAACTGTGGTGAAGCGGAGCAGACTACTTCAACTCCTACGACTGCCAATTAC

TCTCCAGAAGAAATACAGTCTGACCTAAGTCTGGTTCCTGTATCTTCCTCACTAGCGACA

GAAGATGAAAGGCACCTAGCAGCTATCCCTGAGAACTCTGAGGAAGCAATGTCCCACGTT

ATAACCCCAGCTGATTGCTATAGCGATGCATGTAATGCTTCTGATGCACAACATCAGAGT

TTAGAAGTACCGGCTGCAGAGGAAGACCAGCTGTTAAACTTGGACATATTTGATAGCCCA

CGATTTGAGCTTGAGCCATGGGATGATAAGTTATTCTCCCCAGCCCATGCACACTTTCCA

CCAGAATTTGGCTATCAGGCCAACAATGAATTAATTCAGTATGGTACAAACGGGACAGAT

GTTTCAGACTTTTTCAACTCGTGTGTTAATTGGGACGAGTTCTCCAGTGAGGCGTCTATC

AGTCTTGATCTGAGCCCAACCATTTTTAAAATTCCAGAAAATGGGTCGTGCAGCGACTCG

GATGTGGAAATGGCCAATATGATGAATCTGCTAGCATCACATGATTATCCAGAGGGAGTG

ACTCCACAAAATAGTGATGTAGGGTTGTTTCAGAACAATTCCCAGATGACTCTTTCTAAT

GATAGCATGGGCCAAATGACCACTGTGGTAAATGAATATGAGCAAATGTGGAATTCGGAT

ACTGTTGTCGATGGCGGCACTGGAATTAGGATAAGGTCTAGACAAGGACAAAGTGAACAA

CCAGACATGAACCCGGTGATGCAAGCACAAGGAAGTGCACCAAGGAGAATACGATTGGGA

GGATTTGTTGCGCGTTCCCTTGTTTCTGAGGAGACGACGGAAGATGGGAGTTGTGCATCA

GAATATGAACAATCGAGGATTTTGGACAGTGACACTGGTATAAGGATAAGGAGGACTCGA

CAAAGAAGAAATGAAGAGCCAAACATGAACCCAGTGATTCAAGCACAAGGTAGTGCCCCG

CGTAGAATACGATTAGGAGGATTTGTCAAGCATTCCCTTGTTTCCGAGGAGACTACAAAA

GATGAGAGTTTGCAGGAGACGGAAGCTTCAGAAAACCATGCTGCTGCTGAGAGTGCTGCC

GATTCAGAGAAGACATCTCCCATGTCAGCAAGAGAGATCTGTGAGCAACATGTTACAACT

GCAGAATCCAACGTGGGGTCGAAAAACTTTCTGTTGGGCAGGGTTCGGTGCACTTCAAAG

ACATCCTCCATTAGCGCTATGCGGTCTACTGTTCTTGCATATTCTGCCATTGTAATGGTC

TCGCTAGTATTGCTTGTTAATATTTGGGGATATCTTAGATTTAGAAATGCTTACTAG

>MS.gene009966.t1

ATGGACATTCCCGACGGAGTAAGCATCAAAGTCCACGCCAAACTCATCGAGGTTGAAGGT

CCAAGGGGTAAATTGGTGCGTGATTTCAAACATTTAAACCTTGATTTTCAGTTAATTACT

GATGAAGAAGGGAAGAAGAAGCTAAAGGTTGAAGCTTGGTTTGGATCAAGGAAAACCTCT

GCTGCTATTCGTACTGCTCTTAGTCATGTTGATAATCTCATCACTGGTGTTACTAAGGGT

TACCGTTACAAGATGCGTTTTGTTTATGCACATTTTCCAATTAATGCTAGTATTACGAAT

ACTAATTCTGCTATTGAGATTCGTAACTTCCTTGGTGAAAAAAAGGTGAGAAAAGTTGAC

ATGCTTAACGGAGTCACTATCCTTCGATCTGAAAAGGTGAAGGATGAGTTGATTTTGGAT

GGAAATGACATTGAACTTGTCTCCAGATCTTGTGCCCTTATTAACCAGAAATGTCATGTT

AAGAAGAAAGATATCCGGAAGTTCCTTGATGGTATCTATGTTAGCGAGAGGGGAACCATT

GCTGAAGAATAG

>MS.gene009961.t1

ATGGTTGATCTTGATCATCACACTCATAACAACTCTGTCAAACTCCGTTCACCATCATCA

TCTTCTGCTCACCAACCATCATCCAAGAAGCATTCTTTCAACACAAGCAATCTCACCGGT

AATGGCTTCTCCGCCATTACATTTCCTTTCAGTCTTCGTGGCAATGCTCTTGGCCTCTGT

GTCTCTGATCCATGCACCTTGCCGGATCAATCCATGCCGGTAAAAGAGCTGTCAACTGCC

TCCGATCAGGAGATATGA

>MS.gene009956.t1

ATGGAGAATTTGAAGATTGGAACATGGAGTGGTAGAGCCAAGTGTATAGGAGCTATTATG

TGTGTTGGTGGAGCATTAGCTACCAGTCTCTACAAAGGGAAGGAATTTTATATTGGTCAT

CATCATCATCATAGTCACCATTCTGCAAAGATTAGTGCTGTTGCAGCCCACAAAACTCAC

ATGTTTCGAGGCACTTTCTTTTTGGTTGGAGCCTGCTGCTCTTACACTGCTTGGTTTCTT

CTTCAAGTAAAATTGGTGAAAGTATTTCCATTGAGGTATTGGGGAATAATGCTATCATGT

GCTATGGCCGCAATTCAGTCAGCAGTAATAGGTGCATGCGTAAATCCCAGTAAAGCTGCT

TGGAGATTAGAGTGGAATCTTCAGCTTATTACTATACTTTACTCGGGAGCATTGGCTAGT

GCTGCTACATTTTGTTTACTAACATGGGCAATTAAAATTAAGGGACCTACTTACCCTCCA

ATGTTCAATCCATTGGCTCTTGTTTTCGTTGCCATATCAGACACTATCATACTTGGTGAA

CCACTAAAAGTTGGAACGAATGAAATGCCACGTTTGCCTCAAACAAGTGTTGCAGCTGCA

GAATTATCAACAAGCATGGATGATCAGCCTATTGTGTCTCAATCAAAAGCTGTTGTAGTA

CCAACTTCTTCACCAAATGAGACCGGAGCATTGGCTACAGCTGCTACATTTTGTTTACTA

ACATGGGCAATTAAAATTAAAGGACCTACTTACCCTACAATGTTCAATCCATTGGCTCTT

GTTTTCGTTGCCATGTCTGACACTATCATACTTGGTGAACCATATAGGGTTGGAACGAAT

GAAATGCCACGTTTGCCTCAAACAAATGTAGCAGCTACAGAATTATCAACAAGCATGGAT

GATCAGCCTATTGTGTCTCAATCAAAAGCAGTTGTAGTACCAACTTCTTCACCAAATGAG

ACCGTACATCTTGATATTGATAAAAATGATAAAAACTGA

>MS.gene009949.t1

ATGGTGGTCCCATCTATAATTTGTGCAAGGATGAAGAGAAAGTTAGCATCATTACAAAGG

GGTAATGCAAAAAGACCCATGGCCAAAGGTCTTGATGTTAGTAATACTTATGGGATCAGT

GAGAGACAACTGCTATCCAATCCACTTCTTAGAACCCCTCTTTCACCATTGATGAATGTG

GTCAATCAAGGAAACTTAGATACAACACATAATTACGGTGGAAATTCAATGGATTCAACA

TCAAGTGTTTCACCAACTATTTCTTCAGAAATACAAACATCAAGAGGGCTATCCATGCAA

CAATGTGACTCGAAAGAGGCAAGGATGAAGAGAAAACATTCATTGTTACAAAGAGGATGC

ACAGAAAAACTTATTGATAAAGAGAATGAAAATGCGGACTATGCATTAAGGTATCGGAAA

AAGAAAGTTTCAGATGCACCACGAAGTGTACCTAATCAACCCGTTATAAGTGGTACACCA

TTCATTCCTTCTCAAACAAGTATAACATCACTTGCATCAACCTTAACTCATTCAAGTTTG

TTAAAGAGGTCAAGAGCAAAATGTTCTCATCCAACACCCGAACAAAGAAGTCCATTATCC

AATGTTTCCAATATTACAGTTGTACATTCTTCAGAATCTCAATCAATAAATATAGCGAAG

AAAGGTCAAATGACTCGAAGTCAAAGGAGAAGACATTCCAAAGGACTCCCTATTGCGCGT

TTGGATTTTGAAACTGTTTTTGTCGAAGGCGGCTCATCATCGAACGATACCTACGATGGG

GGAGATAAAGAAAACATAAATTTGGATGACTTCAAAAATGGTCTATACAGCGATGCTGAG

ATCAAAGGACCTCTTTATTTTGGATATCCAGACTTCACATGCAAATGGTGCAAAGCTAAA

CTTTGGATTGAAGAGAGGGCAATAAAGTCTAGACCTCCGAACAATGATTCCGAATTTTCG

TTATGCTGTCAAAAGGGATATGTTGATATCCCTTATGTAGAGGAACCTCCAAAACTACTG

TTATCATTGCTTGATGGAAGTGATCCTCGAAGCAAGCATTACCTAGAGAACATCAGAGCA

TATAACAGTATGTTTGCCTTCACATCCCTTGGAGGCAAAGTGTTTTCAAAGCTCAACAAT

GGAAGAGGGCCTCCTCAATTTATATTAAGTGGACAGAATTATCATAGGATTGGAAGTCTA

TTGCCTGAGCCTGGAAGTACTCCAAAGTTTGCCCAACTATACATCTACGACACTCAAAAC

GAAATTCAAAATAGAACAAAAGTTTTTGATTCAAATGATGGTGAGGGTGGATTTGATCTT

TCATTGGTTGAAGATTTGAAAAAAATGTTGGACGAGTTTAACCCTTTGTGCAAATCTTTT

AGAAAGATCCGTGATCTGGTTGAAGAGGGTTCACCTCCAAAGATGGCATTGAGGTTGTTC

AGGAAAAGGGAAAAGGACTCAAGAATGCACAATCTACCTACTGTTGATGAAGTTGCTGGG

TTGATAATAGGTGATTATGATGAGACGGAAGAAGGAAGAGATATTATAGTGGATGATGTA

GGCAGAGGATTGAGAAGGATACATGAGACACATCCATTATACATGCCACTTCAGTATCCT

TTAGTGTTTCCTTGCGGTGATTATGGCTATGAGGAGGATATACCTTACAGGGAATTTGAA

GACGAAGAAAAAAGTCAAGAGGGAAATATTCGTTCCGATTTGTTGAGTGGATTACAAGAA

GCAGTTGAAATGGGAGACAATGATCCTCGAAAAGCCGGACAACGTGTTATACTTCCAGAT

TCATTTACAGGTTCACCAAGGTACATGTTCAATAATTGCCAAGATGCAATGGCAATTTAT

AGAGGACTGAGTGCATCTGACAGACCTGATATCATTTCTAGAGTGTTCAAGATGAAGCTG

GACCAAATGATGACCGATTTTAAGAAAGATCACATTTTTGGTGCTGTGACTGCTGGTATG

TATACCGTTGAGTTTCAAAAGAGAGGGCTCCCACATGCACACATTTTGTTGTGGTTGGAA

GGCAATAACAAACTGCACACAACTAAGGAAATTGATATGGTTATATCAGCAGAGATTCCT

CATCCACAGCTATATCCAAAGTTGCATTTGGCTGTTTCATCATTCATGATGCATGGACCA

TGCGGAAGGGCAAATTTGAATTCCGCATGCATGAATGGCAAGAGGATGGTTTTCCGGTAT

ATAAAAGGAGGGACAATGGGGTTTGTGTGGAAAAAAAATGGAACTCAATTGGACAATAGG

TATGTTGTTCCTTACAATCCATTCCTCTTGATGAGATACCAAGCACATGTTAACACTGAG

TATTGTCCTGACAGAGCAAACCTCCAAATTAAGAAGAAAGCAGGAGATAAAGATGAGGAG

GGCCCAATTGATGAAATTAAGCGTTACTATGATTGTCGCTATGTCTCTCCATGTGAAGCA

ACTTGGAGAATATTTATGTTTGATATCCATGAGAAATGGCCTGCTGTTATGAGATTAGCT

TTACATCTTGAGGGTCAACAATGTGTTAGGTTCAAGGAAAATCAAAAGCTTCCGAATGTT

GTCAGATATCATCAATCAGTCCCTACCATGTTTCTTGCTTGGTTTGTTGCTAATCAAAAT

TACAGTGAGGGTAGAGATCTTACTTATGCCGAATTTCCATCAAAGTTTACTTATGTGCCT

GACAAAAGGATATGGCATCCTAGGGAAAATGGTTTTCAGATCGGGAGGCTGTCTTACATT

CCTGTAGGCTCAGGTGAACTTTACTATATGCGGATTCTTTTGACCTTTCAAAAGGGTTGT

AAGGGGTTTGATTGTATTAAAACTGTGGATGGCAAGCTATATGGTAGTTTTCAAGATGCA

TGTTATGCTCTTGGTTTATTGTCCGATGACAAAGAGTTTATTGATGGAATTTTTGAGGCT

TCTAGATCACAGAGTGGGCAAATGCTTCGTGTTTTGTTTGTTAGACTTTTAATCATGAGT

ACAATGCACAAGCCTGATAATGTATGGAAAGCATGTTGGAAGTTATTGGCAGATGGAATT

CTCTATGCTCGACGACGGATCCTCAATATTCCAGCTATTTTTAATGCACTCATTTCGAAG

AGTTCATTAACTTCCACAGACGGTTATACTGTTTGCATCAAAGTTTTAGCTAAATTGACC

TTTAAGTTCTAA

>MS.gene009982.t1

ATGGTGCTGCTCATCACCGGCCAGAGTGGAGGCTATACCGAACGTTTAGATAGCATCACA

GCTCCATTCTTTTTCACAAATTTTCCACCGGAGGCAACAATAACGGAGCTATGGAAGTTG

TTCCCAAATTATGGAAGTGTAGGGGAGGTTTTCATCCTACTGAAGGTGGACAAGTAG

>MS.gene009965.t1

ATGGGTAACACGGAGAGAATTCAAGGTTTGAAGGAATGGTTCAAATCTTCACAAGCACTT

CTTAGCATGTTATTAGTGCAAATTTTTGCAACTGGGATGCAACTTCTATCAAGAGTCATC

TTGGTTCAAGGGACTTACATTTTTGCACTCATTGCTTATCGACATATTGTTGCTGCTATT

TGTGTTGCTCCATTTGCACTCTATTTTGAAAGAGAAAAGGAGAAGAAATTCAATTGGAGT

GTTTGGTTTTGGCTTTTCCTTAACGGATTAGTGGGGATGACAATGGCTCTAGGGTTATTC

TATTATGGTCTTCGAGACACAACTGCTGCTTATTCAGTCAATTTTCTCAACTTGGTACCT

ATTTGCACATTCTTAACCTCTATAATATTCAGAATGGAAAATTTGAAGATTGAAACATGG

GGTGGTAGAGCTAAGTGTATAGGGACTATTATGTGTGTTGGTGGAGCATTAGCTACCAGT

CTGTACAAAGGGAAGGAATTTTTTATTGGCCATCATCATATTCACCATTCTGCAGAGATC

AGTGCTGTTGCAGCTCACAAAACTCACATGCTTCGAGGCACTTTCTTTTTGGTTGGAGCC

TGCTGTTCTTACACTGCTTGGTTTATTCTTCAAGTAAAATTGGTGAAGGTATTTCCGTTG

AGGTATTGGGGGACCATGCTATCATGTGTTATGGCGGCGATTCAATCAGCCTTAATAGGC

GCATGCGTAAATCAAAGTAAAGAAGCTTGGAGATTAGAGTGGAACCTTCAGCTTATTACT

ATACTGTACTCGGGAGCATTGGCTACTGCTGCTACATTTTGTTTACTATCATGGGCAATT

ACTATTAAAGGACCTACGTACCCTCCAATGTTCAATCCACTGGCTCTTATTTTCGTTGCC

ATATCCGAGGCTATCATACTTGGCGAACCGTTAAGAGTTGGAACGTTGCTAGGCATGGTT

TTGATCATAATGGGACTGTACTACTTTTTATGGGGTAAAAGGAATGAGGTACCTCGTTTG

CCTCAAACAAATGTAGCAGCTGCAGAATTATCAACAAGTATGGCTGATCACTCTACTGTG

ACTCAATCATCAGCTGTTGTAGTGCCAAGTTATTTACCAAATGAAAGTGTACATCTTCAC

ATTACTAAAACTGAGAAAATCTGA

>MS.gene009991.t1

ATGCTGCGTTACTCAAGCCGGTCGATATTTGGACTAATGGAAACAGCATCTGGATTATCT

ATGTGGAATCCGTTTAATGCAACGGATGTGGATAATTACAATAAAGTATTGAGAAAATTG

TTATATGATTTGAAAAATAAAGCTGCAACTGGCGATTCTATGAAAAAATATGCTACATCA

AATGTAACAGATCCAAATTTTCAAGATATATATAGTCTCATGCAGTGTACACCTGATATA

AGTTACTTGGATTGCAATCAATGTTTGGATCAAGCTATAGCTAATCTTCCATCTTGTTGT

GAAAATAAGATAGGTGGGAGAGTTATTAGACCAAGTTGTATTCTTAGATATGAAAGTTAC

AGGTTCTATGAACTTAATGTTCAACCTCCGCCGCCGCCACCTACTTCACCATCTACTAAT

CACAAAGGAAAGGATCACACATCAAGAACTGCCATCATCATAGCAGTCCCAGTTGTTACT

ATTGTTTTGGTGCTATGTTTCATCTGCATCTATATAAGACTAAGAGTGAAGAAACCAAAT

GAAAGTACTAGAATTCCTTCAGAAGATGATGAAGAAATTACAACCTTTGAGTCATTACAA

TTACCCTTTGACACTTTAAAAGTTGCTACAAATGACTTCTCTGATTCTAATAAACTCGGG

GAAGGTGGATTTGGAGCTGTATATCAGGGTAGGCTCTCTAATGGACAAGCGATCGCTGTT

AGAAGGTTGTCCATTAATTCTGGCCAAGGAGATATAGAATTTAAGAATGAAGTACTTTTA

ATGGCAAAACTTCAACACCGGAATTTGGTTAGACTACTTGGTTTTACTATTGAAGGGAGA

GAAAGACTACTTGTCTATGAATTTATTCCCAATAAAAGTCTTGACTACTTCATATTCGCA

AGCAACATTCTCCTAGACGAAGACATGAATGCTAAGATCTCAGATTTTGGCATGGCAAGA

CTAATTCTTGTGGATCAAACCCAAGCAAATACAAGTAGAGTTGTTGGAACCTACGGATAT

ATGGCACCTGAGTATGTAATGCATGGAGAATTCTCAGTGAAATCAGATGTCTTTAGTTTT

GGTGTTCTAGTTCTTGAGATTATAAGTGGCCAAAAGAATAGTGGCATTCATCATGGGGAA

AATAGGGAGGATCTATTGAGCTTCGCATATAGAAGTTGGCGGGAAGGAACAGCTGCAAAT

ATTATTGATCCATCACTATACAACAGTTCAAGAAATGAAATCATGAGATGCATTCATATT

GGTTTACTTTGTGTCCAAGACAACGTAGCTAGAAGACCAACCTTGTCTACCATTGTACTC

ACGCTAAGTAGCTATTTTCTCACTCTCTCTATACCTTCAGAACCTGCATTTTTTATGGAC

AGTAGAACTAGAAGCCTTCAAGAGACGAGGCTGTGGGAAGAGAATTCAGGGACAACAAGA

TCAAGTCAATCCACAAGTAAATCAGCTCCAAAATCAGTAAATGAGGCTTCATTTACTGAT

CCATATCCACGTTAG

>MS.gene009959.t1

ATGCCGGTAAAAGGGCCGTCAACTGCTTTGCAGCCTCTGCCTCCGATCAGTAGATGTATA

TCTGAAGTTATTGATCCTTCTGAGGTTAAAGAAGCAGTTGCTCGTTTTTTGAACTCTGCT

GAAAAAACCAACCCTGAATCTGATTCAATGAGGCTTAAAAGGATGAAGGATCGGTTAAAA

GAAATGAAGAAAGTGTGGGATGAAGTTATGGAAGACGAGGAAGGAAATGAAAAAGAACAA

CAACAAGAAGAAGAGCCCTCTCCTGATGCTGATGATGAAAAGGATGAAAAAGAACAAGAA

CAAGAAGAAGATCACTCTCTTGACTCTTTTGTTGCTGAAGATGAAAAAGACATATCTGAG

GATGAATTGGGGAATGATTACGAAGAAACTGTTAGTGTAGAGTGGATTGATAAGAGCTTA

AGCCTTACTTTCAAGTGTCCATGTGGCAAGGGATATGAGGTTCTAATATGTGCAAACAAC

TGTTACTACAAGTTGGTCTAG

>MS.gene009980.t1

ATGGCGGAAAGTAACTCCATTGATATGAATGCAACTACCGGTAATGAACGTGATGCTAGT

GATATTGTGATTCCTTCAAGTTGCACTGATTTTACTAGGAATACTGAAAAATCTATCCTC

TGTCCATGGGGTAGAGATTCAAATGCTAACAAGACTGAGATTGTAGAAAAGGAGGAGGAT

GGTTCTTTGGAGAAAACTGAATTGACTCATCATGTTCCTAATCCTATTACCAGTGATAAG

ATTATTGGTTGCAAAGCAGAGAAGGACAATGCTCCAGAAACTGTTAAGCAGTGTGGACAA

TCTTCAAGGGATTCAAATGCTTGCAAGAATGTGACTGGTTGGAACAAAGAAAAGGAGGAT

GGTTCTTTGGAGAAAAGTGAATTGACTCATCATGTTCCTAATCCTATTACCACTGATATG

ATTATTGGTTGCAAAGCGGAGAAGGACATTGCTCCAGAAACTGTTAAGCCGTGTGGACAA

TCTACAAGGGATTCAAATGCTTGCAAGAATGTGAGTACTGGTTCTAACAAAGAAAAGGAG

GACGGTTCATTGAAGAAAACTGAATCCATTGATGTTCCTATCAGTGATGATAAGAATTTT

GATTGCAAAATGGAGAAGGTCAATGCTCCAGTCAATGCTAAGGAGTGTGGACCATCATCT

GCAACTCCAATTAGGAACTTGGATATGGGTGGGAGTGGGAATGTGCTGATAAAGGTTCAA

GACTTACGCCCAAATTTTAATGATGTCAATTTGTTGGTAAAAGTAGTAAATGTTAAGAGG

ACATGTCGCAAGGGTCCAAGACGAGTAAATGTGTGCATTGTTGGTGATGAAACAGCCACC

ATCAAGCTTCGAGTCACAAATAATCAAGTTGATATCGTCAAGAAAGGATCGACTCTTATA

TTATACAAGGCAAGGATTAATATGATGAACGACTCGATGGAGCTCATGGTGCCCGCACAT

GGAAATATTCAAGTCCAACAGGAATCTGCAACCTTCACGGTTAAAGAAGATAACAACATG

TCTATGATTCAATACGATGAAGTTCAAGTTGAAGTATGA

>MS.gene009962.t1

ATGCCAATTCTAACATTTGGTCATTCTAAGTTAACTGCTGCTACACTAGCTCTTCAATTG

CCATGTAATTGTATTAGGAAGATAAGGTTTATTACAAAGATTCAATGTTCTGTTGCTGGT

AGGACGTATAGTTATAGGACCGGTGCTGCTGATTCGCGGGGATCCAAACGGGACTCTGGC

GAAATTCAAAGGAAACGGGGAGGTGCATCGTCGTTGTATGCCCGTCCGAGTTTATCTGAA

ATTAAGAAAGATAAGGCCACGTTGCGTGAAATTGTTTATGAGTTCTTGCGAGGAATCGGC

ATTGTTCCTGATGAACTTGATGGTCTTGAACTTCCTGTTACGGTTGATGTTATGAAGGAG

CGTGTTGATTTTCTTCACAGTTTGGGGCTTACGATTGAAGACATTAACAACTATCCTCTT

GTACTGGGATGTAGTGTCAAGAAGAATATGGTTCCTGTGCTTGATTACCTTGGTAAATTG

GGAGTTAGGAAATCCACGTTAACGCAGTTCTTGCGGAGATATCCACAAGTGCTTCATTCT

AGTGTGGTTGTGGATCTTGTGCCGGTGGTCAAGTATCTTCAGGGGATGGATATCAAACCG

GATGATATTCCTCGTGTTCTTGAGAGGTATCCTGAAGTGTTGGGATTCAAACTTGAGGGA

ACCATGAGTACATCTGTTGCTTATTTGATTGGAATCGGTGTTGGAAGAAGAGAACTCGGA

GGCATCTTAACTAGATTCCCGGAGATTTTGGGGATGCGGGTTGGTAGAGTCATCAAGCCT

TTTGTGGAGTATCTGGAAAGCTTGGGTATTCCAAGGCTAGCTATTGCTAGACTGATAGAG

ACACAACCTTATATTCTTGGATTCGATTTGGACGAGAAAGTGAAGTCAAATGTCAAATCC

CTCGAAGAGTTTAATGTTCGGCAGACATTGCTTGCCTCTATAATTGCTCAGTATCCTGAC

ATCATTGGAACCGACCTAGAGCCGAAGCTTGCAGATAAGAGAAGTGTGCTCAATTCTGTG

CTTGATTTGGATGTTGAGGATTTCGGCTTAATCATTGAGAAGATGCCGCAGGTAGTTAGC

CTCAGTACTACACCTATGCTGAAGCACGTTGATTTTCTTAAGGATTGTGGGTTTTCTGTG

GATCAAATGAGGAAGATGATTGTTGGATGCCCTCAACTACTTGCTTTAAACATTGATATC

ATGAAACTTAGCTTCGATTACTTCCAAAGTGAGATGGAAAGGCCTTTGGTAGATTTGGTT

GAGTTCCCAGCATTCTTCACTTATGGTTTGGAGTCAACTATAAAACCTAGGCATAACATG

GTTGCTAAGAAAGGATTGAAGTGTTCTCTGGCATGGATGCTTAATTGTTCTGATGAGAAA

TTTGAGCAACGAATGGACTATGACACTATTGATATGGAAGAGATGGAAATGGAACCATCA

TTTGACATGAATTCATTGATGCGGCCAAGGAGCGATGAATCAGATTCTGATTATGAAGAC

AGTGATTATGATGATGATGATGATGATGATGATGATGAGTAA

>MS.gene009955.t1

ATGCAAAACAACAACACAAACAACGGTGGTGGTGGAATGGACGAGTTACTAGCAGCATTA

GGTTACAAGGTTCGTTCATCATCGGACATGGCTGACGTAGCTGAGAAACCTGAACAACTT

GATATGGTTATGGGTAGTGCTCAAGAAGAAGGAATCAACCATCTTTCTGCAGATACTGTT

GAGTGTCTAGCTAGCATCAGCGTTGAGAATGTTGAATCCAACAGACGTGCTCTTCGTGAA

CTCCTTTTCACCGCCCCTGGTGTCCTGAAGTATCTTAGTGGAGTTATCCTCTCTGAGGAA

ACCCCTCTACCAATGCACCACTGCTGGTACTCACTCCCAACATTTTGTGTTAAAAGCAAG

CCTTTTGTTGATGTCTTGAAGGAAGCTGGTGTGCTTCCTGGTATTAAGGTTGACAAGGGT

ACCGTTGAGCTTGCAGGGACTGATGGAGAAACCACCACTCAGGGTCTCGACGGACTTGGT

GCACGCTGTATCAAGTACTATGAAGCTGGTGCACGTTTCGCTAAATGGCGTGCCATGCTC

AAAATTGGCTCCAACGAGCCATCTGAACACTCTATCCATGAGAATGCCTATGCTTTGGCC

CGATACGCAGTCATATGCCAAGAGAATGGACTTGTACCAATTGTTGAGCCTGAGATCCTT

GTTGATGGACCTCATGACATTCAAAAGTGTGCTGCTGTTACCGAGCGTGTCCTTGCAGCA

TGCTACAAGGCCTTGAATGACCAACATGTCCTCCTTGAAAGCACTCTCTTGAAGCCTAAC

ATGGTTACCCCTGGATCTGATGCTCCAAAGGTTGCACCCGAGGTTGTCGCTGAGCACACC

GTTAGAGCTTTGCAGCGAACCGTACCTGCTGCGGTTCCCGCTGTTGTTTTCTTGTCTGGT

GGACAGAGTGAGGAAGAGGCCAGTGTCAACCTCAATGCCATTAACCAAGTCAAGGGTAAG

AAGCCATGGACTCTCTCTTTCTCATTTGGAAGGGCACTTCAACAAAGTACCCTTAAGGCA

TGGTCTGGAAAAGAAGAAAATGTGAAGGCTGCTCAAGAAGCGTTGTTGACAAGGGCTAAA

GCTAATTCTGAGGCAACTCTTGGAACTTACAAGGGTAACTCCAAACTTGGTGCCGGTGCA

TCAGAGAGTCTTCATGTTAAGGACCATAAGTACTGA

>MS.gene009997.t1

ATGGCACCTAAAAGAATTAACACGGGTAAGAGAAAGCAAGGCGAAACATCCGCAACCCAA

CCCCCACCCCAAAACCAGTACTATGATCAGGAGAGATTTAGGTTTAGGTACCACCAGAAC

AGATTCAATGCATTGCTTAAACAGTCCTTGTGGACTGAGAGAGTTGTTCAAATTAAACCG

GATGGACCATATGGCAACTTTCACAAACTTTTCCTTGAGCAAGGGTGGGCCAGACTCCTA

AACCCCATAACCAAAATCAACCAAGAGTTGGTGCGTGAGTTTTATGCTAACGCCCTTCCA

GAGAATCCTCAAACCGACCCATTCACTTTTGAAACTTTTGTGAGAGGTCGAAAAATCAAA

TTTGATCGTGATGCCATAAACACATTCTTAGGAAATCCTTTTGACCTGGATGTTGAGGCT

GAGGATGGAATTGATGATTTCCATAAGAAACAAAACCTAGGTCACTTTCTCCTTGATGAT

GTCCACAATGAGATAAAAAAAATTCATAATGCTAGAGGGGGAAACTTATGA

>MS.gene009996.t1

ATGGCCACCAACCATGGTGTTCTTTCATCTCTTGACAATGCCAAGACACAAAGCTACCAT

TACTTGGCAATAGTGATAGCAGGAATGGGATTTTTCACTGATGCATATGATCTCTTCTGC

ATCACAGCCGTTACAAAGCTTATCGGTCGATTGTATTACTCTGATCCGACTAGCCATAGT

CCAGGAATACTTCCAACAAACGTAAACAATGCAATAACCGGTGTTGCGTTGTGTGGCACC

CTTGCAGGGCAACTCTTCTTTGGCTGGCTTGGTGACAAACTTGGTAGAAAAAAAGTTTAT

GGCATAACTCTTACTACCATGGTTGGTTTTGCTTTAGCATCAGGTCTTTCATTTGGTTCC

ACGTCGAAAACCGTGGTGACTAGTCTTTGTTTCTTCAGATTCTGGCTAGGATTCGGCATC

GGTGGAGACTATCCTCTATCAGCTGTGATCATGTCTGAATATGCTAATCAGAAAACACGA

GGAAGTTTTATTGCTGCTGTTTTCGCTATGCAAGGAGTTGGAATATTGGTTGCTGGAGGC

GTAGCCATGTTCGTCTCGAAACTATTCTTATCGTACTTTCCAGCTCCTGATTTTGAAACT

GATCCTGTTCTATCTACTCAACCAGAAGGTGATTTCGTTTGGAGAATTGTGTTAATGTTT

GGTGCTGTTCCTGCAGCATTAACTTACTATTGGAGAATGAAAATGCCGGAAACTGCGAGA

TACACTGCTTTGGTTGAAGGAGATCACAAGAAAGCCGTTGAAGATATGGCAAAAGTTCTT

GATAGGAACATAGTTTCAGAAGAATCTAATACCAGGATTGCTATCAGGCCACTCGAGACA

CCCTCTTATGGACTCTTCTCTTCGGAGTTTCTTAATAGGCATGGCCTTCATCTTCTAGGA

ACAACTAGTACTTGGTTTTTATTGGACATTGCTTTCTATAGTCTTCAACTGACACAAAAA

GATATTTATCCAGCTAGTGGACTTGTATACAAGGCTTCCAAAATGAATGCTATACAGGAA

GTGTTTCAGCTCTCTCGAGCGATGTTTGTGGTTGCCTTGGTTGCCACAGTACCTGGATAC

TGGTGCACGGTTTTCCTAATTGAGAAGATCGGACGATTCAGAATCCAGCTCGTTGGATTC

CTTGTTATGTCTGTTTGTATGTGGTTTCTAGGACACAATTATATATCATTCAGGGGAGAA

GAATCTGCATGCAAGAATGGTTCAAAATATGGTTTTTGTAATGGAAACCCAGTCATGTTT

GCTATACTTTTTGGACTTACACTTTTCTTTGCTAACTTTGGACCTAATAGTACTACATTT

ATAGTGCCAGCAGAGCTGTTTCCTGCAAGGCTCCGTTCGACATGTCATGGGATATCCGCT

GCAGCTGGAAAATCAGGAGCTATTGTAGGTGCCTTTGGGGTGCAGAGCTATATAGGTAAT

GCTCATGATAAATCAAAACGAACCAAGCAAGCAATTATGGCCCTTGCGGTTGTGAACTTG

CTTGGATTCTTTTTCACTTTCTTGGTGCCTGAAACACAAGGTCGATCGCTTGAAGAAATC

TCGGGAGAGGAAAAAGATTTCCAAGGAAATGATGCAGATGAAGAAATAAGCGGTGAAAGA

AATGGAACAAGAAATGCTAGTGTCGATAAATCTCCAGGAACTTCAATGGTTTAG

>MS.gene009954.t1

ATGTCGAACTTCAAGAGCAAGTACCATGATGAGCTTATTGCCAATGCTGTCTACATCGGC

ACACCCGGCAAGGGTATTCTTGCTGCTGATGAGTCAACCGGAACAATTGGAAAGCGTCTA

GCTAGCATCAGCGTTGAGAATGTTGAATCCAACAGACGTGCTCTCCGTGAACTCCTTTTC

ACCGCCCCTGGTGTCCTTCAGTACCTTAGTGGAGTCATCCTCTTTGAGGAAACCCTCTAC

CAAAGCACCGCTGCAGGCAAGCCTTTTGTTGATGTCTTGAACGAAGCTGGTGTGCTTCCT

GGTATCAAGGTTGACAAGGGTACCGTTGAGCTTGCCGGAACTGATGGAGAAACCACCACT

CAGGGTCTTGATGGACTTGGTGCTCGTTGTGCTAAGTACTATGAAGCAGGTGCACGTTTC

GCTAAATGGCGTGCAGTGCTTAAAATCGGCCCCAATGAGCCATCTGAGCACTCTATCCAT

GAGAATGCCTATGGTTTGGCCCGATATGCAGTCATATGCCAAGAGAATGGACTTGTACCA

ATTGTTGAGCCTGAGATCCTTGTTGATGGACCTCATGACATTCAAAAGTGTGCTGCTGTT

ACCGAGCGTGTCCTTGCAGCATGCTACAAGGCCTTGAATGACCACCATGTCCTCCTTGAA

GGCACTCTCTTGAAGCCTAACATGGTTACCCCTGGATCTGATGCACCAAAGGTTGCACCC

GAGGTTGTTGCTGAGCACACTGTTAGAGCTTTGCAGAGAACCGTACCTGCTGCAGTCCCA

GCTGTTGTTTTCTTGTCTGGTGGACAGAGTGAGGAAGAGGCCAGTGTCAACCTCAATGCC

ATCAACCAAGTCAAGGGTAAGAAGCCATGGACCCTTTCCTTCTCTTTTGGAAGGGCACTT

CAACAGAGTACCCTCAAGGCATGGTCTGGAAAAGAAGAAAATGTGAAGGCTGCTCAAGAA

GCTTTGTTGACAAGGGCTAAGGCTAATTCTGAGGCTACTCTTGGAACTTACAAGGGTAAC

TCTAAACTTGGTGCTGGTGCCTCAGAGAGTCTTCATGTGAAGGACTACAAGTATTGA

>MS.gene010001.t1

ATGTTAGTGAATGAAGAGATAACCATTCCTAAGAGAGAGGAAAGTGGTGCTGATTCAATA

GGAACAGCAACAACTATCTTGAAGCAGCACTTTGTGTTAGTACATGGCATAAGTGGAGGA

GGTTGGTGCTGGTACAAAATCAGGTGCCTTATGGAGAATTCTGGCTACAAAGTCTCTTGC

ATAGACCTCAAAAGTGCAGGAATTGATCAATCTGATGCTGATTCTGTTCTTTCTTTTGAT

GATTATAATCAACCCCTCTTGGATTTCATGTCTTCTTTGCCTGAAAATGAAAAGGTAATA

TTGGTGGGACATAGTGCAGGAGGGTTGAGTATTACCCATGCTTGTCACAAATTTGCCAAG

AAAATATGTTTAGCAGTGTATGTGGCTGCAACTATGCTCAAATTGGGATTTTGTACAGAT

GAAGATCTAAGAGATGGTATACCCGACCTGTCTGAGTTTGGAGATGTATATCATCTAGGA

TTTGGATTGGGAAAAGATAAACCTCCAACAAGTGCTTTGATGAAAATGGAATTTCAACGG

GAAGTCTTCTATCATTTGTGCCCTAATGAGGATTTTACCTTAGCAGCAATGTTGTTGAGG

CCTGGACCAATTCTAGCTATAACAAGTGCAAGATTCAAAGAAAGTGATGAAGCAGAGAAG

GTACCTCACGTGTACATAAGGACAAAACAAGACAGAGTGTTAAAGCCAGAACAACAAGAA

GCCATGATTAAGAAGTGGCCACCATTGAATGTGTATGAAATAGAAAATAGTGATCATAGT

CCATTATTCTCTACTCCTTTCATTCTCTTTGGTTTGCTTGTAAAAGCTGCAGCTGCTTTT

GATGTTTAA

>MS.gene010000.t1

ATGTTAGTGAATGAAGAGATAACCATTCCTAAGAGAGAGGAGAGTGGTGCTGATTCAATA

GGAACAGCAACAACTATCTTGAAGCAGCACTTTGTGTTAGTACATGGCATAGGTGGAGGA

GGTTGGTGCTGGTACAAAATCAGGTGCCTTATGGAGAATTCTGGCTACAAAGTCTCTTGC

ATAGACCTCAAAAGTGCAGGAATTGATCAATCTGATGCTGATTCTGTTCTTTCTTTTGAT

GATTATAATCAACCCCTCTTGGATTTCATGTCTTCTTTGCCTGAAAATGAAAAGGTAATA

TTGGTGGGACATAGTGCAGGAGGGTTGAGTATTACCCATGCTTGTCACAAATTTGCCAAG

AAAATATGTTTAGCAGTGTATGTGGCTGCAACTATGCTCAAATTGGGATTTTGTACAGAT

GAAGATCTAAGAGATGGTGTACCTGACCTGTCTGAGTTTGGAGATGTATATCAGCTAGGA

TTTGGATTGGGAAAAGATAACCCTCCAACAAGTGCTTTGATTAAAAAAGAATTTCAAAGG

AAAATCATCTATAACTTGAGCCCTCATGAGGATTGTACCTTAGCTGCAATGATGTTGAGG

CCAGGACCAATTCTTGCCTTAACAAGTGCAAGATTCAAAGAAAGTGATGAAGCAGAGAAG

GTACCCCGTGTGTACATAAGGACAAAACATGACAGAGTGGTGAAGCCAGAACAACAAGAA

GCCATGATCAAGAGGTGGCCACCATTGAATGTGTATGAACTAGATAATAGTGATCATAGT

CCCTTCTTCTCTACTCCTTTCATTCTCTTTGGTTTGCTTATAAAAGCTGCAGCCGCTTTT

GATGTTTGA

>MS.gene010002.t1

ATGTCTAGTGAATCGGCTCCCAAACCTCGATTTGTATTATGTCCTAAATGTAGGCTGCTG

CTTCAAGAGCCTCAAAATTTTGACGTGTACAAGTGCGGTGGATGTGGCACGACCCTTCAA

GCTAAGAAACGAAAAAGCAGAGCTGCAAATTCAGAATCCAGTTCAAACGAGACTGATGCA

GCTGCTTCTAGAAATGCATCAGATCTTGTTTCTGGTGATAAACAATACAGCAACGGAGAG

AAGCTAGTTAGTCCTCAAAAGAATGTTTCAAGAGAAAAAGCAACTAATTCGTTGTTTGTA

GATTCTTCTTCAGAGGGGAATGAAGGAAGGAATCAAATTGAAAATAGAGAATGCAATGGG

GAGAAGCCGGTTACTACTCAGGAGAATGGTTCGAGAGAAAAAGCTGTTTTTTCTTCCTCG

GGAGAATGTTCTTCAGAGGAAAATACTGAAAGGGATCAAATTGAAAATGGTGAATGCAAT

GAAGAGAAAACAGTTACTTCTCAGGAGAATATTTTGAGGGCAAAAAGAACTAGTTCTTCC

TCCGGAGAATGTTCGCTGGATGGAAACTGTGAAAGGGGTCAAATTGTAAAGGGTGAATGC

AATGAAGAGCAACCAGTTATTTCTCAGGAGAATGATTTGAGGGAAGAAGCAACTAGTTCT

TCAGAAGAATGTTCATTGGATGGAAGTAGTGGAAGCGTTCAAATTGAGAATGGTAAATGT

GACGAAGAGAAGCTTGTACTGTTTAATTTATCAGATGAAGAACCAGAAGATGAGGTGGAT

ATCTATAAGTTATCAGATATCAGAAGGCATACAGTGTCCAACAGAGGTTACTCAAATGAG

CTCCCACAGGCCTCAGCAGAAGTGATGGCAGACAATTCAGTTGAAAAACAAAATGAGACT

AACTTGAAACTAGAAGAAGAAGAAGAGCAAAGTAATGGAAACATGCCATTAGAAAGAACT

GGAAATCAGTTAATTAGTGCACTGGACAGAGAAGATGCCAGTGATGAGAAATCAGCTCTA

GTAGGCGTGAAGTCTGAAGTAGACATCGGCGAAAGTGATTTAGAAGTAGCAAGAGAGATA

AATAATCGAAACTTGTCACAAAGAGCAAACCAGAAGTTAGTTTCCGGATCTGGATCAGAT

GGACAATGTGTCAATAATGACAAATTGGTTCTAGTAGGCGACAGCTCAGCAACAGACGTT

AATGGACCCAATAATGCAGACTCGAAAGAATTAAATGGTGAAAACTTGTCATTACAAAGA

ACAGAAGAGGACATTGGTGGAAATGCATGTACGGACGAAAGGTTGAGTACTGAGAACTTT

GCGTCTGAAAAAGGAAACATTTCATATGTTTCTCGTCATGAACTTAAAGAAGGTACATTT

GATAATCATGCATACCCTCCCAAAAACATTCGCCATAGATTTGACGATTTAAGGTCAGCA

GGTACATTTGACAGTGCAGAAGTAAATAATCTGAGTTTGGAGATTAATGGTGCGCTTGGG

GAATTGTCTAAATCTCCAACAGCTAGAAGTTCTCATGCTTATGATGGCAGTGTCTCTTCT

AACGATGGAATGGATGAGCGGTTCCTCGGTCAAAATTTATATTCTTTTGAGGGAGGCTCC

AGAAAGGGAAAAGGTGCTGTTAAAAGTAGCATGTTATATGAAGATGTTGAAATGCAAAGT

CAGTCGAATTTTCCCAATAGAATGTATCAAAATGAGGTTCTGGAGACTAAAAGAGGTGAT

CATGCAAATCGGATGCGAACAAAAACTGACGAGTTTCCGTTTCCATACAAAATGCCTCTC

CATGGAAGTAGTCCCCACTCTGGCTATGAAAGTGGAAGTCCATCCAATCAAATACACAAT

GAACTCTACCTCAGTTCAAGCTATGTTTCACCTGACTCCGTCGAGGACCCTGACCAGGAG

AAGATGAAACTGTTGAGAATGGTTTATAAATTGCAGGATCAGCTCAACAGAACCAACCAT

GCAAACAAGGAAACAAACGAAAGACTCTCTGCGGGAAACCATATTTCTTCATACCAAAGC

CATGATTCACACGAAGGGAGATTTTATCATGGTTTGGATTACCCTAGGGGGGATGCAAAT

GCAAGCTATAGTCATGGCATCAACATGCACCAAAGGCGGCATAACTTTTCGCATGTTCCT

TATTCAACCGAGCCAACAAGCAATGCACACCATATTGATCATCCTTATTTCAATTGTTGT

CCTCAAGAGGGGCAACACTTTGGAGAGTTTCCTCCGTGTTTTCCTTACCAGCGTGAAGAT

TTGTATCGGCCTCATCCAGTTCATAGTCGTTGCTTATCTCAGCATTCTTATCCTTCAAGT

CCGCAATGGTTAATAAACTCAAATCATGTTCACGGCCGTGAAACAAAATCTTGTGAACAG

AGATACAGGGCTACCGAGATGAACTATACTAGAACTAGGGACAAGCCGAGTTTGACTAAG

CGACATTACCGGCCAGTAGCAGGTGCAGCACCATTTGTCACTTGTCATAAATGCTTAAAT

CTGCTGCAGCTTCCCGCAGATTTTCTCCTTTTCAAAAGAGTATGTCATAAGCTCAAATGT

GGTGCATGTCAAGAGGTACTTAAATTTTCTCTCCAGAACAAAAGTCATGTAGTTTCTTAT

ACTCCAAATGCTGTTGGACCACCATCAAGTGACCTTGATATGCAAACAAAGCCAATAAAT

GGCATCAATACTCAGTCTGAACCTCATGTTGCGGATCGCGTGTCATATTCCGATGATTAT

GGTCATTCTGTCAGTAAAAGCTATTCCTCAGAAGGAGATCCTGTTTCTGTTGCACCGTTT

CACCACTCGCATGCGAGTGCACACAATAATCCAAGTGTCTCTCCTGATACTGTTGATGCT

ATAACAGAGAAGGAGAAAACTGCTTCAAGAGGACCTAGCACGAGTAAGGCTCCATCCAAT

ATGTCATCAGAAGGAGAGGCACCACAGTCACTGCCAAAACCTTCAGCACTTCATCAACTG

ATGGGTTATTCCTCTCCAAGTCAAGTGCTAAGAGGTGCTCCAGTCGATGAAGGAAAAGAG

AGCTAA

>MS.gene010004.t1

ATGGCGGTTTCAGGGTTGAGAAGTAGCTGCGGTTTACCATTGATTCATCATCACAATCAT

CTTCGTTTCTCTTCTTCCTTCACGAACCTCAATCTTCATTCTAAGTCAAAGCGTTTCACT

CTCTTTGCTCGTTATGCTCAAGCTCAAGACCTTTTCTCCTCTCGTCGATTCCAAGACTTC

ACGTCTTCCATCAGAAGTGTCGGTGCTACAGGAAAAGGATGTTTGATAAACATTGATAGT

ATCGAAAAGCTACCTAAGCTTGTAGAGGATATTGTTCAGACATCTGTGGATACTGGTCCT

CGGGGTGTTCTTAGATTGGCTCAAGGTGTTCAAGCCTTCCTTGGGGTCGGTCAAGAGTGG

TTAACGGATGTATCCAAGGTCTGA

>MS.gene010006.t1

ATGATGTCTCTAGGTCGAACTGAAGGTGATAGTTCTTCAAGCAATTTCAAAAGTAGGGAC

CTTCCAGCTATGTACCCAGAGCTATTTGAAGATGATCCAGTCTTTCAATATCTACGTGAG

ACTAAAAAAAGGGCCGAAAGAGAACGTCTTCGTCACATGCGTAAGTCAGTTGAAATTACT

AAGATTCCAGTTGGTCAGATTGTTGAACAACAAGTAACAATTAATGGAAATGTTGAGCTA

CGACGAATTTATGTTCCTGATCCTAAAGTGAGAGAAACAATGGAGTATATGGTCCAAATT

GCTTGGGAACGTCAAAGCTCCAATCAACCTTTAAGGTCGCAGGATCAGACGGCAATAGAC

TACTATAATGTTGTGTTACGCCATCTTCCACAACCGATTAATGGTCCAAACCAACCTAAT

AACTATATACAGCCACGGTGGCTGCAGAAACTTAACAACCGATGGATACGCAAAGTAGAA

GCAACAATGACCAATATTCGTCGTGAGAATCTTGAATTTGGGGATGCCAGTCCCGAAAAT

ACACAATTGTGCTGCATTTGTCTGGAAAATTTGGCTGCTGGAGAAGAAATTGAAAGACTG

GACTGTGCACATAGATTTCACATGAGTTGCATCAAAGACTGGCTCCTAGCTATAAGGAAT

GTATGCCCTCTTTGCAATACAAGGGCATATCATTTGATTTAA

>MS.gene010003.t1

ATGGCGGTTTCAGGGTTGAGAAGTAGCTGCGGTTTACCATTGATTCATCATCACAATCAT

CTTCGTTTCTCTTCTTCCTTCACGAACCTCAATCTTCATTCTAAGTCAAAGCGTTTCACT

CTCTTTGCTCGTTATGCTCAAGCTCAAGACCTTTTCTCCTCTCGTCGATTCCAAGATAGT

ATCGAAAAGCTACCTAAGCTTGTAGAGGATATTGTTCAGACATCTGTGGATACTGGTCCT

CGGGGTGTTCTTAGATTGGCTCAAGGTGTTCAAGCCTTCCTTGGGGTCGGTCAAGAGTGG

TTAACGGATGTATCCAAGTCAACAAACTCATCCGCTGCGTTACCGACAGATATGCAGCTT

GGTCTTCTGTCCCCCTTATATTTGAGGAGGTTGTTTGAACGTCTGGGGGCAACCTACATC

AAATTAGGACAGTTCATAGCATCCGCACCAACACTATTTCCAGCTGAGTATGTACAAGAG

TTCCAGAATTGTTTTGATAGAGCTCCTCCAGTTCCTTTTGAGGTAATTCAATCAATATTG

CGTAAGGAATTAGGAAGACCAATAGAAAGTGTGTACGAATATGTTGACCCAACACCCCTT

GCTTCCGCTTCTATAGCACAGGTTCATGGTGCAAGGCTAAAAGGCTCACAGGAGGATGTG

GTTATAAAGGTCTTAAAACCAGGAATAGAGGACATATTAGTAGCAGATCTTAATTTTGTT

TATGTTGTTGCTCGGATATTGGAGTTCTTGAATCCTGAAATAAGTCGGACATCACTGGTT

GGTATTGTCAAAGACATACGAGAGTCTATGCTTGAAGAAGTTGACTTTTACAAGGAGGCT

GCAAATATTGAGGCTTTCAGGAGATATTTAGAAGCAATGGGGCTCACTAGGCAAGCTACA

GCTCCAAAAGTGTATCAAAACTGCAGTACGCGGCAAATTTTAACCATGCAAAGACTATAT

GGTGTTCCTCTTACTGACCTAGACTCAATTAGGTCATTAGTTACTAACCCAGAAGCCAGC

CTTATCACTGCTCTCAATGTGTGGTTTGGAAGTTTGCTTGCATGTGAATCTTTTCATGCA

GATGTGCATGCTGGGAATCTGTGGCTTCTACGTGATGGCCGTATCGGATTTCTTGACTTC

GGAATTGTTGGTCGTATATCTCCCAAAACATGGGCGGCTATGGAAGTCTTCTTAGGATCA

ATTGCCATCGATGATTATGACTCAATGGCATCTTCCTTAATTCAAATGGGTGCTACAAAC

AACGATGTTAATGCTAAGGCCTTTGCCAGAGATTTGGAGAAAGTGTTCTCATCAATGAAG

GAGTTGGACACTGAAATAGTTGTAGCAACTAGAAGTGGAACAGCCAGAAATCCAGCTGCT

ATATCTGCTAATGTAGTTGTTGATGAGAGGCAGATGAATGCCCTATTTCTTGATGTGGTT

CAGGTTAGTGAATCTTATGGATTGAAGTTTCCTCGAGAATTTGCGCTTCTTCTGAAGCAG

CTCTTGTATTTTGACCGGTACACACGGTTGTTGGCTCCCAATTTGAACATGCTTCAGGAT

CAGAGGATTTCCATTGCTTCCAATAGAAGAAGCAACTATACATAA

>MS.gene010009.t1

ATGGGTAGGCGAAAAAGTAAACCTCATCGATCAGGTGGGATAATAGTAGAAACAAATGCT

AGTGCTGAAACAGAATTGAATAAACATGCCGTAGGAGCTGGAGGAAAAGAAAATAATGAT

TTTGGTGATATTTATAAACCTTATTTTGTTGAGGTTGACCGGTCTGGTTGGTTGTCCGAT

GAACACCTTGATATTTCTGAAGTTGTTCTTAGGGATTTAAATATAGGAGAAGGCTTTTAT

GGATTTGAATTGTATGAGGATATTTATCAGGATCCCCAATTCTCATTAAGATTTCGGTTG

TGCAATATTGGCAATGATCTTGGTCGATGCAAACTTGGCCATTGGCCAGTGTTACCTTAT

ACTGATATTCATCTAGAGTTTGTTAAAAGAGCTACGGTTGACAGTACCGAAACATGCACG

GTGTTATTATCAGGTATTTTTGATGGACCTGATGAAAGTGTTTCTGGTCTTGTTCATTTG

GCGAGTATGAAATTTGTTACATTGAGGGCAATTCTTGGGATTAGGCTTTCGGAGGACATT

CCTTCCCTTAGGGTGAGGGTTGAGGTACTAAAGAGTGCCTTCGATGCATGCGAGTCACTT

CTTGAGGCTTCTAGAAAACCTTGGAAAAAGAGTATGATGAATGTTATGTCTTGGTTACGA

CCAGAAATAATGACTTCAGATATTAAGTATGGGTTTAGTAGCTATATGGAAATGGAAGTT

GATTTGCAGACAGACATGGCAGATGACGGTGGCTATGCAGGGAAATGTTCGAGGTTCGAT

CCTGCTGGTTTTTATGAAGCCATCAAGCCTTCAAAAACAGAGCCAATGCTTGAAGATGAC

ATACCAGAGTTACTTCCTGAACTGAGGCCATATCAACGGCGTGCAGCTTTGTGGATGGTA

AAACGAGAAAAAGCTACGGAAGAAAGTCAGGCTGAAATAGAAAGAAATCAGTTTCATTCT

CCATTATGTGTGCCGGTGGATTTTCTGGACACAAGGTCTAAAATGTTTTTCAACCCATTC

AGTGGAAATATTTCCTTGTGTCCAGAGACTTCGTCACCTTATGTATTTGGAGGCATTCTT

GCTGATGAGATGGGATTGGGAAAAACTGTAGAATTGCTTGCTCTCATCTTTGCTCACCGG

GGATCAGCATCTGAAAGTGACATACTAATTGATTCAGTGCCCCAAGTCAACGGCGACGAA

AAAGTCGCTTTAAAAAGACTCAAAAAAGAGCGTGTGGAGTGCATATGTGGAGCTGTGAGC

GAAAGTCTCAAATATGAAGGACTATGGGTTCAGTGCGACATTTGTGATGCTTGGCAACAT

GGAGATTGTGTTGGTTATTCAGCTAAAGGAAAATCACTGAAATCAAAACGAGGATTGGAA

AGCAAGACATCTAAAACCACCATAGCTGTGACGAGTGGGGAATATGTATGTCAGATGTGC

TCTGAACTAATACAAGCTACTGAATCTCCTATTGCTTCTGGTGCCACTCTCATTGTCTGC

CCAGCTCCTATTTTGCCCCAGTGGCATGACGAAATTATACGTCATACACGCCCAGGAGCC

TTGAAAACTTTTATTTATGAAGGTGTGCGTGACACATCTTTCTCCAATTCATGTCTGATG

GATATCGGTGATCTTGCCAGTGCGGACATTGTATTAACTACATATGATGTGCTTAAAGAT

GATTTGTTCCATGATTCTGATAGACATATAGGTGATCGACACCTTTTGAGATTTCAGAAA

AGGTACCCAGTTATCCCAACTCTTCTTACCAGAGTATACTGGTGGAGGATTTGTTTGGAT

GAGGCTCAAATGGTGGAGAGTACTGTTGCTACCGCTGCTACTGAAATGGCTCTAAGACTC

CATTGCAAGCATCGTTGGTGCATTACAGGGACCCCAATTCAGCGAAAGTTTGATGACTTA

TATGGACTTTTAAGGTTCACCAAATCCAGTCCTTTCAATATATACAGATGGTGGAGTGAA

GTAATTCGAGATCCTTATGAGAAGGGAGATATGGGAGCTACGGAATTTACACACAGAGTT

TTCAAACAAATCATGTGGCGTTCTTCTAAACAACATGTTGCAGATGAATTGGAATTGCCC

TCCCAAGAGGAGTGCCTCTCTTGGCTTACACTATCACCAGTAGAAGAGCACTTTTACCAG

AGGCAACATGCAGCTTGTGTTAGAGACTCCCATGAAGTTATTGAAAGTTTGAGAAATGAT

ATTCTTAATAGAAAAGTCCCAGATTCTGTCTCTTCGAGTGGTTCCTCCGATCCATTAATC

ACTCAAGCGGAGGCAGGGAAGCTATTGCATGCTTTGCTAAAGCTTCGCCAGGCTTGTTGT

CACCCCCAGGTTGGAAGTAGTTCTGGATTGCGTTCCCTGCAGCAGTCACCTATGACAATG

GAGGAAGTACTGACGGTCCTTATAAGCAAGACCAAGATAGAAGGTGAGGAAGCTCTCAGG

AGGTTAGTTATTGCTCTGAATGCTCTTGCAGCAATAGTTACAATACAAAACGATTTTTCT

CAAGCTGCTTCGCTGTACAATGAATCTCTTACTTTAGCTGAAGAGCATTCTGAAGATTTC

CGTCTGGATCCATTGTTAAATATCCACATTCATCACAATCTTGCCGAAATATTCCCCCTG

GCTGAAAATTTTGCTTTGAACTTGCCATCCAAAGGAAAACAATTATCTGGGACTTCCGCA

GTTAATACTGCCAAAAAGCATTATATTGTAAAGGTTGATAATGATCAAGTGAAGAGGCAT

AAAATCAGTAACTGTGGTGACATAAGTTTAACTGGGGCTGCTTCAGATCCATCAAATGTT

GCATCTAGTAGTTCAGAAAATGGATTAAATGATAGGGAATCTGATGATTTGTCGGCCAGT

TCTGTCAAATATCTGAAAGCACAATGTGAAGATTCAAAACATAAATACTTATCTGTTTTC

AGTTCAAAGCTAGTTGCAGCTCAGCAAGAGTTTCAAAGTTCTTACATGCAGGTTTGTAAT

GCATATCACGACACCAGTACAAATCAAACTACATTTTGGTGGTTAGAAGCACTTCACCAC

GCTGAGAAGGATAAGGATTTCTCAACTGAGTTGATTAGAAAGATTGAAGAATGCATCTCA

GGAAATTCAAAATCGTCGAGATTAGCTGCTCGTTTTCGGAGCATAAGCTCTCTGACGTAT

GAAATTCAAACCGGATTAGATCAGCTAGTAGCCTCAAGGAAAGTGGTATTAGATCGTCTG

TTAGAAATAGATCAGACGATGGAAAACCCAAAAGATGAAGATATTGAGCGCGTTGGTAAA

TGTCGAAATTGTCAACCTAATTGTGATGGCCCTCCATGTGTTCTGTGTGAGCTAGATGAA

TTGTTTCAGCATTACGAAGCTAGACTCTTTGTTCTCAAGAACGAACGTGGAGATATAATT

TCATCTGCTGAGGAGGCAGTAGATTTTCAAAAGAAAAGTTTCGCACGGAATCATTTCCTG

TCAAACCTATCAAAATCTAATCAAAGTTCATCAGTATCTAATATTGATAATGAAGAATCC

AGAAAAAGGAATGTTGGGCAGAAAGTTGTGACTTCAAGATCGGCATCTATCCTGGAGGTT

CTTCTTGGAGTTATAAAGAACTATTGCAAGACTCGATTTGGAAAGGACAGTGCCTCAGCA

GCGACCAAGCATTTACATATTCTTGAGGGAATGCGAAAGGAATTTGTCTATGCAAGGTCT

TTGGCATCAGCTCAAGCTCAATATCTGCGTGCTCATGATGAAATAAAGATGGCGGTTTCT

CGTTTACACCTAAGGGAAAATGAAGACGATAAATCTCTTGATGCTTTAGGTGAGAATGAA

TTATATGCGGCTAGCTCAAACTTTACCCAGGAGAAGTTTATGTCACTGGCCTTGTTGTCA

CAGATAAAAGGAAAGTTACGCTACTTAAAGGGTTTGGTTCAATCTAAGCAAAAACTGCCA

TCGGAAAGTCCAGATAACTCTTCATGCACTCAAGATACAAATGCTATGTCAAACTCTACG

GAAGAGAAGGGTGAACTCATACCTAAAACTTATGAGGAATCATGCCCAATTTGTCAAGAA

AAGCTAGGCCATCAGAGAATGGTGTTTCAATGTGGGCATGTTACTTGCTGTAGATGTTTA

TTAGCTATGACTGAGAAAAGACTTAAACATAGCAAGACTATTACTTGGGTGATGTGCCCT

ACATGTCGACAGCATACTGATTTTCGAAACATTGCCTATGCTGTTGATGCACAAAAGGAA

TCTCCCAATTCGTCAATGCTGCATACAATTGACAATTGTGAAAAACACGAAGCATCCATT

ACTGTTGAAGGCTCATATGGAACCAAGATTGAAGCAGTCACAAGACGAATCTTGTGGATA

AAGGCTACAAATCATAACTCAAAAGTTCTTGTATTTTCAAGTTGGAATGATGTACTTGAT

GTATTGGAACATGCCTTTGCCACTAATAACATCACCTTCGTCCGGATGAAGGGAGGCAGG

AAAGCACACACTGCCATCAGTCAATTTAGAGGAATACAGAATGGCACAAAAGACTGTGAA

TCTATTCAGGTGTTATTGCTTTTAATCCAACATGGAGCCAATGGCCTCAATCTTTTGGAA

GCACAGCATGTGGTTCTTGTAGAGCCATTACTCAATCCAGCTGCTGAAGCACAAGCAATC

AGCCGTGTACATCGAATCGGTCAAAAACAGAAGACCCTCATTCACCGTTTCTTAGTAAAA

GACACAGTTGAAGAGAGCATATATAAATTGAACAGAAGCAGAACCAATCATTTATTCATT

AGTGGCAACACAAAAAATCAAGATCAACCTGTTTTGACTCTAAAGGATGTGGAATCCTTG

TTGGCAAGAGCACCAAGAACTGCGCCGGAAATTGATGAAAATCCTAATAATACAAATACA

AACCTGAGAGACCTGCCACCATCACTTGCAGCGGCAATAGCGGCCGAGAGAAGACATAAT

GAGCATAGAACATGA

>MS.gene010005.t1

ATGAACACTGGTGAGATTCAAGTTGGTCAGAATGTCGAACAACAAGCAACAGTTGGTGGA

AATGTTGAGCAACAACCAATTTATGTTCCAAATCCCGAAGTAAGAGAAATGATGGAACAT

AAGATGACAACTACATTACCCACCCATCAACAAAAGGTCCAAATTGCTTGGGAACGTGAA

CGCTCGGGTCAACCTTTAAGGATGCAGGATAAGGCGGCAATAGACTACTATAATGTTGTG

TTACGCCATCTTCCACAACCAATTAAGGGTCCAAACAAACCTGTGTTCCCCCATCTTCAG

CAATCAGATTATGATGCTCAAAACGAGGTATTCAACATGATGTATCTTTTAAGTGATTAA

>MS.gene010008.t1

ATGAACACTAAAGGTCGAACTGAAGGAGATAGTTCTTCAAGCAACTTCCAAAATATGAAC

CTGCCAGCTATGTACCCAGAGCTATTTGAAGATGATCCAGTCTTCAAATATCTACTAGAG

ACTACAAAGAGGGTTGCAAGAGAACGTCTTCATCACATGCATAAGCCAGTTGAAACTGCA

AAGATTCCAGTTGGTCAGATTGTTGAACAACAAGTAACAATTAATGGAAAAGCTGAGCTA

CAACGAATTTATGTTCCTGATCCTACAGTGAGAGAAACGATGGAGTATATGGTCCAAATT

GCTTGGGGACGTCAAAGCTCCAATCAACCTTTAAGGTCGCAGAAGCTTACCAACCGGTGG

ATACGCAATGTAGAAGCAATGATGACCAATATTCGTCGTGAGAATCTTGAATTTGGGGAT

GACAGTCCCGAAAATACACAATCATGCTGCATTTGTCTGGAAAATTTTGCTGCTGGTGAA

GAAATTGAAAGACTAGACTGTGCACATAGATTTCACATGAGTTGCATTAAAGACTGGCTC

CTAGCTGTTAAGAATTTTATAGGAAAATAG

>MS.gene010010.t1

ATGAGTTCCGTCGATACAATCAATGGAAACTCTGCAAAATCTTTCAAAGAACATGTTCCA

GGGGAAAGCCTGGAACCAAAACAGGATGTTACTGAAGTAAAAGCACAAGTTGATGCCATA

TGGGAACAAATGAATAAAGGAGTGTCCAATAACGTTATTAGCAGTTTTAAAAGCAAGCCT

AACTCTACTGCAAAAACTACGACAAAGAAGATATCTTTAAACTTGGATGCTGTCCTTGAA

CAAATCAAGAAGAAAGCAGAACCTGAAATTTTTGTAGGCTATAGCTCATCTTCAAAAGTC

CATAGAATCTATCTTCCACAAAGCAACAAAGTAATTGTTAGCAGAGATGTCAAATTTCTT

GAGTCAAATAGTTGGAATTGGGAAGATTGTGAGAAGTTTGAATTTCAGGAGGAGAATGAA

GATGTTGATGATGAAACTGTTAGAGGAACTAGATCACTTTCAGACATCTACCAAAGGTGC

AACGTTGCTGTCATGGAACCAGAAGGATATGACAAAGTTAGATATGAATTCTTGACACAA

ATGCTTGGTGTATGCAGTTTCAGAGTCAAGGAGGAGTGTTGA

>MS.gene010007.t1

ATGAACCCTGAAGAAGGCAACAACACTTCTCACTTTAACAATGGAGGACCGAGAAGTGTG

CCGTCCATCTCGGCTAGAGATGATATGTCATTCTCCTCCTTGTATCCGGCTATTGGAGGA

CAAACTGGAGTTGCACCTGTTGTAGGATCTGATTATCAAAGAAGTGAAACTGTTGTAGAA

AATAGTAGTAATGTCAATTCCATTTCGTCATTCAACGATGGAATTAACACCACTACTACT

GTAGTGTTCTCTGCCTTCTCTCTAGACACATCCCGCCTCCCTTTCCCTCAAATCCAACAA

CTCGTTCCAAGATGTAGATCCGATGTCGGTGGTGCGACGAGGCATGGCCCTCCGCATCAC

CTCACCTCCGCCGACCTTTGCGCCGATGGGTTTGGGAGATTGAAGCAACAATGCGCCGCG

GTGGCTCACGGTGGTTCGTGGTGGCTCACGGTGGTTATTGCGATGTGGTGCTGTAGAAAC

CCGAAAGGGTTGTTTTCGTTGAAAGAGCTGCGTGGCAGATCTAGTTACTGTTCGGTGGTG

GCTAGTTTTGTTAGTGTATATCCGGGAAATGTTGTTCAGATCCGCCGTTTCGCTCCACCA

TTAGTTCTCTTTTACTCCACCGACAGACGCCTTCTCCTTGTCACCGGTGGCCGCCTTTTT

ATGATTTGGGTTGTGTTTTCGCTTCAAACCGGCGTCGCTACCCGTGTTTTGTTGTTGGAT

TTCGTCTTCCTCTAG
