# Supplementary material for: Identification of Genetic Loci Associated With Crude Protein Content and Fiber Composition in Alfalfa (Medicago sativa L.) Using QTL Mapping
Source: Front Plant Sci. 2021 Feb 18;12:608940. doi: 10.3389/fpls.2021.608940 (PMC7933732; doi:10.3389/fpls.2021.608940)
Supplement: Supplementary file 11 [file Table_9.docx]

### Table S9. Potential Candidate Genes of *qlignin1C-2*

>MS.gene40896.t1

ATGATGAGGGGAAAAAGCAACGAAGAGCCATGGCTGGTGGTCGATGGCACGGGAGAGGCA

CATCTGTTAGAGTCTGGAGAACATGAGATCATGAGACGTACTGGTCTCACCGCAACTGAC

CTTCAAATTCTTTACAACCCTTCCACTATTCTCGGACGCGAGAGAGCAATAATCGTCAGC

ATGGAACACATCAAAGCCATCATCACTGCTTACGAAGTTTTCCTCCGAGACCCTTCTTCC

ACTTTTGTTGAGGAACTTCAAGCCAGGATCCTCCGTTACGATGCAGCCACCACTCCTCTC

CCGTTTGAATTTGCAGCCCTTGAGGTGTGTCTTGAGGCCGTTTGCAGAATGTTGAAGAAT

GAAACAAAGATTCTGGAACAAGAGGCACGTCCTGCTCTAGATGAACTAGATTCAAACATA

AGTGGTAACAGTTTGGTTGTTGTTGGAGAGTTGAAGATCCGGTTGGATGACATAACTGCT

CGGGTTCAAAAGGTGAACGATGAATTAGATCATTTGTTCCTTGATGCAAATTTGAGTGAG

ATTCATCTCACCAAAAAACAACAAATGGAAGAAAACTCCTCTACCACCACCACAGCTGCC

ACTGGCGAGCTTGAGCTGGACATGCTCGAAGCATACGTCGATCAGATTAACTATACTCTC

CGCAAACTCTCCACGCTGAGGAAGAATGTTGAGGACAAAGAGGAGTACATCAACGATGTG

CTAGTTTATCAACAGGAACGTAGCGCACAAAAGAGATTCAAGCTGACCATTGCACACGTG

GCATTAACTGGATGTATTACTGTGATTGGTAGTTTACGCAACAACGTTTATAATGACGGA

GTTTTTCTCTTCACCGTTGGTATCTGCATTGCTGTGTTCATATTCTTGTATATGGGTGCC

ATTGCCTGGTTGAAGCACAAATGCTTGTTAGATTAA

>MS.gene40893.t1

ATGACAGCCCAACCAAACTCGGATCTGCGACCGACGGTAGATGAAGTTCAGCCATGGCTG

TCGGTCGATAGTACTGGAGATGCACATGTGTTTAAGGCCGGTGAGCTTGACATCATGAGA

CGTACTGGCCTCACCTTATGTGACCTTAGAAAAATCTTAGACCCCCTCTTTTCTTCCCCT

TGTGCTTATTCCACTATTCTCGGACGAGAAAGAGCCATCATCGTCAACTTGGAACACATC

CAAGCCATAATCACTGCCGATGAAGTTCTCCTCAAAGACCCTTCTTTTGCTCAGGAACTT

CAAGAAAGTGTTCGTAACCACGACTCAACCACCACTCCATTTGAATTTGCAGTGCTTGAG

GCATGTCTTGAAGCGGCTTGCAGCGGGTTGGAGAAGGAAGCAAAGATGCTAGAGCAAGAG

GCACATACTCCTTCTCTAGATGAACTCAAATTGAAGACCAGCACTGAACTACTTAACAAT

TTGGAAGGTCTTTATAAACTTATGAGCCGATTGGTTGACATAACCGACCATGTTCAAATA

GTGAAAGAGGGTTTGGGATATTTTCTTTTTGATGAATCAAATATGGCTACCTACTCCACT

GCCAACGACAAGTTCGTCGTTAAGGAGCTGAAGATGCTCTTGGAAGCATACTACTTTCAG

ATTCTCAGTACGCTCAACAAACTCTCCAAGCTGAGGAATTATGTTGATGACACCGAGGAC

AACATCAACATTGTGCTGAAAAACATACCGGAATATCGCCTACAATTTACAGCCACATTG

TCGACAATAGCTGCTATTTTGATTGCCTTTATATTTTTTGCCACATTATAA

>MS.gene40895.t1

ATGACTACCCAACCAAACTCACACAAGCGACCCACCGGAGATGAAGTTCAGCCATGGGTG

TCGGTCGACAGTACCGGAGATGCACATATGTTTGAGGCTAGAGAGCTTGACATCATGAGG

CGTACTGGCCTCACCTTACGTGACCTTAGAGGAATCCTAGACCCTCTCTTTTCCTACTCT

TATACTTATTCCACTGTTCTCGGGCGAGCGAGTGCCATCATCGTCAACGTGGAACACATC

CAAGCCATAATCACCGCCGACGAAGTTTTCCTCAGAGACCCTTCTTTTGTTCAGGAACTT

CAAGCAAAGGTACGTAAGCGTGACGCAACAACCACTCCGTTTGAATTCACAGTGCTTGAG

GCATGTCTTGAAGCGACTTGCAGCGTGTTGGTGAACGAAGCAAATATGCTAGAACAAGAG

GCACCTACTCCTTCTGTAGATGAATTGAAATCGAAGACTAGTACTGAAATACTGAACAAT

TTGGAAGGTCTTTATAAACTTAAGAGCCGGCTAGTTGACATAACCGATCGTGTTGAAAGA

GTTGAATATGGTTTGGAAGCTTTTCTTTATGATGAATCAGATATGGCTAGGTACTCCACT

TTCAACGACAAGTTTGACGTTGAGGAGCTGGAGATGATCTTCGAAGCATACTACTTTCAG

ATTCTCAGTACTCACAACAAACTCTCCAAGGTACCTTTCTATTTGCCTTTTCACTATGCT

ATTAGCATCTACAAATAG

>MS.gene40894.t1

ATGACAACCCAACCAAACTCGGATCTGCGACCGACGGTAGATGAAGTTCATACATGGCTG

TCGGTCGATAGTACTGGAGATGCACATGTGTTTAAGGCCGACCCCCTCTTTTCTTCCCCT

TGTGCTTATTCCACTATTCTCGGACGAGAAAGAGCCATCATCTTCAACTTGGAACACATC

CAAGCCATAATCACGGCCGATGAAGTTCTCCTCAAAGACCCTTCTTTTGCTCAGGAACTT

CAAGAAAGTGTTCGTAACCACGACTCAACCACCACTCCATTTGAATTTGCAGTGCTTGAG

GCATGTCTTGAAGCGGCTTGCAGCGGGTTGGAGAAGGAAGCAAAGATGCTAGAGCAAGAG

GCACATACTCCTTCTCTAGATGAACTCAAATTGAAGACCAGCACTGAACTACTTAACAAT

TTGGAAGGTCTTTATAAACTTATGAGCCGATTGGTTGACATAACCGACCATGTCCAAATA

GTGAAAGAGGGTTTGGAATATTTTCTTTTTGATGAATCAAATATGGCTACCTACTCCACT

GCCAACGACAAGTTCGTCGTTAAGGAGCTGAAGATGCTCTTGGAAGCATACTACTTTCAG

ATTCTCAGTACTCTCAACAAACTCTCCAAGTTACCTTTTGTTATGGAAATGTGA

>MS.gene40892.t1

ATGACAACCCAACCAAACTCGGATCTGCGACCGACGGTAGATGAAGTTCAGCCATGGCTG

TCGGTCGATAGTACTGGAGATTCACATGTGTTTAAGGCCGGAGAGCATGACATCATGAGA

CGTACTGGCCTCACCTTACGTGACCTTAGAAAAATCTTAGACCCCATCTTTTCTTCCCCT

TGTGCTTATTCCACTATTCTCGGACGAGAAAGAGCCATCATCGTCAACTTGGAACACATC

CAAGCCATAATCACTGCCGATGAAGTTCTCCTCAAAGACCCTTCTTTTGTTCAGGAACTT

CAAGAAAGGGTTCGTAACCACGACTCAACCACCACTCCGTTTGAATTCGCAGTGCTTGAG

GCATGTCTTGAAGCGGCTTGCAGCGGGTTGGAGAAGGAAGCAAAGATGCTAGAGCAAGAG

GCACATACTCCATCTCTAGATGAACTCAAATCAAAGACCAGCACTGAACTACTGAACAAT

TTGGAAGGTCTTTATAAACTTATGAGCCGATTGGTTGACATAACCGACCGTGTTCAAATA

GTGAAAGAGGGTTTGGGATATTTTCTTTTTGATGAATCAAATATGGCTACCTACTCCACT

GCCAACGACAAGTTCGTCGTTAAGGAGCTGAAGATGCTCTTGGAAGCATACTACTTTCAG

ATTCTCAGTACTCTCAACAAACTCTCCAAGCTGAGGAATTATGTTGATGACACCGAGGAC

AACATCAACATTGTGCTGAAAAACATACCGGACTATCACCTACAATTTACAGCCACATTG

TCGACAATAGCTGCTATATTGATTGCCTATATATTTTTTGCTACATTATAA

>MS.gene40975.t1

ATGCTTCCAATGTGCAAGTGTTTACCTATGAAGCCGATGATTCTTTACATATCAACGACT

TTGGAGAATCAAGGAAGAAGGTTTTGGAGATGTCACGATTGGCAAAAAAAGTACAAAACC

TGTAATGAGTGGATTTGGGATGATGAACTTGGTCCAGCAACCAGACCAATGACTCGTTGC

TATTCAGCTAAAGCAGAGTCTGCACCTTTCAATGTAGCTGTGATTCATGAAGATGAGTCT

GCACCTGTGAATGGACCTGTGATTCGTGAAGCTGAGTCTGCAAAGTCTGGAAGGTCTGCA

AACCAATGTTGCAATTCTGGTGAAATCTGGGAGAAGAAGAAAGACAAATGGAAAATGAAG

GTGTTGGCTGAGAAGAAGAAGATGTTTCATACATAG

>MS.gene40976.t1

ATGGCGAATCCAATGGAGGTGTCAAACTCGAATTCGAAGATCGCAACGTCTGGAGAATCA

TCACGACCTAATACGGATATGGGTATTGCTGTTAAGTTTCATTATGGGGGTAGGTTTGTT

AGGGATTGGGTTATTTGTTACAACGGGGGAGAAGAAACGTTGATTGAAGGTTTAGATCCT

GATAAGTGGTGTTTCTTTGAGTTAACTGGAATATTGGAAACTGATTTGCTAGTTAAAAAA

CCTTTTAGGCTGTGGTGGATGTTAGATGAAGAGGTTAGTTTTAGGGTGATTAAAGATGAC

GCCACTGCTGAATTTTTAAAAGATTATGCACTTAAAAAAAACTCTGTTGTGAATGTTTTT

GTTGAACATAATGTGGATGAAAGTATTGTTGTAGGTCATAATCCTAATTATGATGTTGTT

GGTGAGGAGTTGGGTGAGAGTTATCAGGTAGGTAACAATGAGAAGGGTGAGAGTTCTCAG

GCAGTCAATACTGACAAAGGAAAAAGGCCATTAGTGTATAGTGATAATGAAGAAGGGGAT

GTGGGTGGTCTAAGAAGCAGTGATGATGAAGATGATGATAGGTATATTGACAGTGAAGAA

GAGAGAGCACTTGGATTTGAAGATGGGTTTGAAGAAGTGGATAATGACAATTTGAACATA

GACAGGAAGGAGTATGAGAGTGAAGAGTTAGATTCTGATGATCCTGATTTGAGTGGTGAT

GAGAGAGCACCTGCTTATGATGTATTTAACCCCAGCCAACTAACCAAGGATTATGTGTTC

ACAGTTGGCATGGATTTTAAGAGTCTTAAAGAGTTCAAGGATGGTGTAAGGGAGTGCAAG

GTGGCCTACAACCATCCATTTAAATTAAAGACATGGTATGGGGACCACACATATGGTAGG

GTTTTAGAAAACCGTAGTGCTGATGTAAACTTTGTTACCAAATATGCCCTTGATAACATG

AGGACCTCTGAGATGAAGGTTAGTGATATAATGTCAGATCTGAGAACACACAAGTCAGTT

GGAGTGTCATTTTACATAGCTTGGATGGCAAAAAAGAAGGCCAACGAACTCATAGAAGGG

GATGCAAGGAAACAGTATACCTTACTGTGGAGGTATGCAGCTGAGCTTCAGAGGGTGTCC

CAAGGAAATAGGTATATTGGAACTAACAGGAGATGGGTATTTATCTCTGATCAACAGAAG

GGGCTCATTTCAGTTTTAGAGGAGTGGTCTGACATAGTTGAACACAGACTATGTTTGAGG

CATCTTTATGCAAATTTTGAAAAGAGATTTGGAGGTGGTGCTTTGATCAGGGATCTGATG

ATGGGTGCAGCCAAAGCAACTTACTATCAATTGTGGAAGTCAAAGATGGATGAGCTGAAG

AAGGTTGACTTGAAAGCATGGGAGTGGCTAATGGGACATGACCCTAAAATGTGGTGTAAG

CATGCTATTTCTTATTACCCTAAATGTGATGTCTTAATGAACAACATATCTGAAGCCTTT

AATTCAACAATATTAGTTGCTAGAGAAAAACCAATACTGACCATGGCTAAATGGATTAGA

ACATATTTAATGAATAGGATGTCAACCAATAGGGTTAAGCTTGAAAGATGGAACTATAGG

ATAATGCCTATGCCTAGGAAGAGATTAAATAGAGAAATTGAGAAGTTTGGTGGTTGGGTT

GCCATATGGGGTGTTGCTGAAGAGTTTGAAGTTCATCAAGTTGGAGGAAGTCTGGCCTTT

ACTGTTAATGTGGCCAAGCAAACATGTTCATGTAATTTTTGGGAGTTGGTTGGGATTCCT

TGCAGGCATGCCATTACAGCAATGTCTAAAACATCAAAGGACCCAGAAAATTATGTTTCT

GAATGGTATAGCAGAGAAATGTATGAAAGGTGTTATGCACACAATGTGAGTGCTATCAAT

GGTCAAGACATGTGGCCAGAGGTAGAATGTGAAGAAATGTTACCTCCTGCTTATAAGAAG

GCACCAGGAAGACCAAAAAAGCTTAGAAGGAGAGAAGCTGATGAAGCACCATCATCCCAG

GGTAAGTATAAAAGATCTGGAACTACTTACAGGTGCACAAGGTGTGATCAGTTTGGCCAT

AATGCTAAAGGATGCAGAAGTTTAACTATCAACCCTAATGCTCAAAAAAGAAAGAGAAAG

CCACCAACACATGCAAGGGGTGATGACAATGATGACAATGAAGCTAGTGCAAGTGAAGCT

GGGAATGGAGCAAGTGCAAGTGGTGCAGGTGAAGCTATTGCTGGATCAAGTGCAAGTGCA

AATGCAAGTGCAAGTCAAGAAGGGACTGGGGAGTCTATACTGAGTACTCCAGACATAAGT

GAGCCTGTGCAGGGTGCAAGTAAAAAAAGGAAGGTTGGGAGCAATACAGGATTCATTCCT

CCAAAACAGAGTAAGAAACCTAAAACAAATCCAATGGCCTTTATGTCTTCATCTATTAAG

CCAAGTGCTACAGTTCAAACAACAGTTCACACCACCAAAAAATCACACACATTAATAGAG

CCAACTGCAAATGTCAACACTGTGGTTCACACATCAAAAAAATCACACACATCTGTTAAG

CCAACTGCAAATGTGCACACTGTGGTTCACACATCAAAAAATGCGCACACATCTGTGAAG

CCCTCAGCTAATGTAACCACAGTTGTTAACCCACCAACTACTGTCATGAAGAAACCTGGT

ATGCACAAAAAGCCACCAAGAAAACCTTCTGTTAAGACCTTAGATGTTGTTAGGGCCATT

GTGGAGCCAATAGTGAAAGTGAAAGCACCTGTTAGGAGGAGTGGCAGAACTTTCTGGAAA

AGTCCTGCAATCAAAAATGGTCCAGGGAAAGACATGGACAACCCAATTGAGGTGGTTGAT

GAAGAAGTTGTAGAGGCAAATGGCTCAAGGAAAGATGCTGCTGAAGCTGCCCCAAGGAAA

TTCAGAGATGAAGGTGGAAGTTGTTTGGCCTTGTTAAGAAGTGTGGAGAATGTGAAATAT

ATTTAG

>MS.gene40981.t1

ATGGATGCGGATCCACCACCGTATTTGCTTGATGAATTGATCTCTAAAAGAGAAAGACGC

ATGGAGGAGGAGGAGACGACAGCACTGCCGTATCTTCCTGAAGAATTGGTAACTGAAATC

CTTCTAAGGTTACCGGTGAAGTCTCTTATTCGTTTCAAATCCGTTTCCAAGTCATGGTTT

TCTCTTATTTCAAATCCTAGTTTTGCAAATTCACAGTTTCAGATCACTGCTGCAACACAC

ACTCGTCGAATTCTGTTCGTAACAGAACTGCTCGTAACAGAACTGTTTGTAACAGAAGCT

CCTCAATTTCGATCTATAGCTTTAGATTCATTATTCACCGCTGATTCTGCTCCTGTTCTA

CTGAACCCTAATTTTTCGCTTCCTAAATCTTATTTTGATTTTAAAGTTAGAGGTTCATGT

AGAGGGTTTATATTGGTGGAGTCTTTTCCAATCATGTGCTTATGGAATCCATCCACCGGA

GTTCATAGACAAATACCTTCGCCTCCGCCTTGGCCTAATCATTCAAGTATACCTTTTTAT

GGCTTTGGGTATGACGAGTCAACAGTTGATTACTTGGTGGTTTTAGTATTCTATCATTTT

ATTCAAAACTCAGATGATGTGCTATCTCACATTGGGATTTTCTCTTTGAGAGCTAATGTG

TGGAACGAAATTGTGGGTCCTGCACATTTGCCTTTTTGTGATAATCTTACTCACTTTGCT

TATACTGTGGTAGAGTCGGTCTTCAATGGGGCTATTCACTGGTTGGCTATTTGTCCTGTT

ACAGGTGACCATCTTGTTGTTGCCTTTGATTTAATGGAAAGAAAGTTTGTAGAGATTCCT

CTCCCAGATGATATTGGTCGTTTGAAAATTTGTAGTTTATGGGTATTTAGAGGATTTCTC

AGTCTATGGGTTTTGAGGGACGAGGATAAAGTTGATATATGGGTGATGAAAGAATACAAA

GTGAATGGTGATATTGTTGGAACAAATAGACCTAGTGGGTTGGTGAGATATGATGACAAA

GGAGAGTTTCTAGGGCATAACTATTATAGTGAATCTTCACAACATGCATTCCGACTGGCT

ATGTATACAGAGTCTCACTCCCCAGTGACAGTGAGCAAGCTTAAGAAGGTGGCACAAACA

AGAGGAGTGAGGTAG

>MS.gene40980.t1

ATGAAGAAAAGCACCGATGGGATTCTTAAGAGGACTGAAAATAAGCACATTCAAACTGTG

ACTTCAACTCTTAATTCAAGAAAGTTGACAAACTCAAAGGAGGCAAAAACAAACAATGGA

AACTCTAAACAAAGGACTTCTTTGACATCTATAAATAGCTTTAAAAGCTCTGAGTTTGGG

AGGTCTACCCCATTGGTTGCAGTTGCCAAAAGCCAGACTTTTGAGGCATCAATACCTGTT

GATCAGAATTCAAACCCTGCGAAAAGTGAAAAGCCGAACAGAGAAGATGACGATGTTAAT

TCGACAACTTCAAGTCTTACTCCTGGTAAGAAGAGCAACGGCTCTGGATTCTCCTTCAGA

TTGGAAGAACGAGCTGAAAAGCGAAGAGAGTTCTTCTCAAAGTTAGAAGAGAAAATTCAG

GAAAAGGAAGCTGAGATAAGTAATCTGCAAGAGAAATCGAAGGAAAGCCAAGAGGCAGAG

ATCAAGAAATTAAGAAAGAGAATGACATTCAAAGCAGCTCCAATGCCAAGTTTCTATAAG

GAACCTCCTCCAAAAGTTGAGCTGAAAAAGATACCTACAACACGCCCAAAATCACCAAAG

CTTGGAAGAAACAAAGGATCTATTGTGAACAACAATTCAGAAGATAAATCCAGTTCTATC

CCACATGAGAAACAACAGAAGAATGATTCAACCAAGGCAAAGGTAAAAGGCTATAAAGAT

GTGAGTTCAAAGAAACCAATCAAAAAAACTCAAGCCAAGGTACAATCTAAGGAAATCGCG

GTCACGAAAACAGAAATGGATTCTGTCATGTCTTCCACAAACGAGAATGGGATAGAACAA

GAATCACAGAATGGTCTTGCTCAGAACAGTGTGCTGGTCCTTAATGCTTCCACACCAGAA

ACTGTATCCTATGAAGTTACTGTGGGAGTGTAG

>MS.gene40982.t1

ATGGTAGAGTCAGTCTTCAATGGGGCTATTCACTGGTTGGCTATTCGTCACGATAGACGT

GGGTATGTTATTGTTGCCTTTCATTTAATGGAAAGGATACTTCTAGAAATTCCTCTCCCA

AATGATGATGATTTTGAATATAGTTTCAAAGATTGTAATTTATGGGTATTTAGAGGATTT

CTCAGTCTATGGGTTTCGGGGGACAAGGGTAAAGTTGATATATGGGTGATGAAAGAATAC

AAAGTGCAGTCATCTTGGACTAAAACTCTTGTTCTTACTACTTATGATACCATTCATAAC

GTTGCCCTTGTATGTTGCACAAAAAGTGGTGATATTGTTGGAACAGATTACCGTACTGGA

TGGGTGAGATATGATGACAAAGGAGAGTTTCTAGAGCATATCTACTATTGCATAGATTCA

CGAAATGAATTCCGATTGGCTATGTATACAGAGTCTTTGCTTTCACTCCCTAGTGACAAT

GAGGAAGCTTAA

>MS.gene40977.t1

ATGCTGAACGCAACACTCACTATTGTAAGGGGAAGACATTGGGACGAGAATGAAATTGAA

GAAGACCCAAGTGAAGTAAAAGGTACTAAAGCCTTCTCAAATGAAGTGAATTCCACAGCA

AGGACAAACAGGGGCCAACATCAATTTGATGTCAATAGCAACATTCAGAAAGATCAAGGG

TTAAGGATGACACTATCTGAAAAGCTTGTGGGAGTAGCTGATGGAACCATGACTGAACTA

GAATGGGTGATGCGTAATGTGCAGATCGAGGTTCAAAATTTTGAATTCTTCGCGGACATA

GTGGTGATGGACATGGCTAACTATACAATGGCGTTAGGCAGATCTTTTCCTAACTACATC

AAAAGCTCGAATCAACATGGAATACATGGAGATTGTGCTTATGTCAAAAGGTCAGTACAT

GATATATCATCTCTCACAAAGGAATCCTGGGAAAGATACAGTACTGAATGCCATGCATTG

GAAATATTTGATCCTTATGGTTCCCATGGAAGCATAGAACCGACATGTGCAAATCAAGAA

GATATCAAAGAAAACAGGAGCTACTGA

>MS.gene40978.t1

ATGAACCTCTTGGCACTACAAGATGGTTGCTTAAATTACATAGAAGATGGAGGTGTAAAT

ATGAATGCTGGATTTAGACGGAAAGAAAAGAGGCTAGATGAAGAGGCGCTGAAGAGCAAG

CAATCTAGCAAGAGTACTGACGACAAACGATGCAGAAACCGTGAGAAAGTGAAGAATCAA

CACTCCCGTTTCACCGATTATACCCCGTTGAACTCATCACGAGAAACCATCCTCCACGAA

TGCTTTAACTCAGTGTTCAAGGAGGCGGGCATCAAGCCACCCCAACCGTTAAAGGAGTCT

GCAATGACGGATAAGTCAAAATATTGCCGCTTCCACCGATGCCATGGTCATGACACCAAT

GACTACTATCAACTCGAGGATGCGATCGAAGAACTGGTTAAAAAAGGGAAGCACCGCTTC

ACCAAGGACGTGCATCGCAAGCATGAAAAACGAAAAAGAGATTCGTCGCACAAAGCTTCC

CTTCATAGTGAGTCTCCAAGGAAAAGAAAAGTAATAAGCGTCGTGGAAAGAGGAACAAAG

AGTCACGATGGCACCGATGAGGGAATGGAGAAATTTTGTGTTTTCTTGGGAGCCATCATG

GGGGTATTTGCTGCCCCGCGGAATGCCTTAAAGGGTTCAACCAAGAGGAAAGTGTTTGAG

TTAAGGCTGGTGCATGTCGAAACACCGAGCCCAAAGAACACAAAAACAAGACCCATCCGA

GGGTTCACCGACAAAGAAAAGATCAATGGAGGATCGAGTGAAATCTCTTCGCTTGTTATA

GTCACTGTGATTCGCGGCCACGACGTCGCCAAGTGTTTGAGAGACGAGGGACGCTTTGTT

GACATCTTGTACCAGGGCACTTTTTAA

>MS.gene40984.t1

ATGAGGTTACCGATTAAGTCTCTTATCCGTTTCAAATCGGTTTGTAAATCATGGTTTTCT

CTTATCTCTTATAATCACTTTGCAAATTCACATTTTCAACTTACTTCTTCAACTCACACT

CCTAGGATTCTGTTCTTAACCGTAAATTCACAGTTTCAACCTTCAGGTCCAACACTCTTC

ACCTATACTCCTGAATTTCGATCTATAGATTTACCGTCATTGTTTACCGACGATGTTTAT

AATGAACCTGTTCCACTGAACCCTAGTTTTCCACTTCCGGAATCTCATTTTGATCTTCAT

ATTAAAGGTTCATGTAGAGGGTTTATATTTTTGCACAATTGTTCAAAATCTTATATATGG

AATCCATCCACTGGATTTCATAGACAAATACCTTTCCCTCCTAATGCTTCACATTTAAGT

TTTTATGGTTTTGGGTACGACGAATCAACAGAGGATTACTTGGTTGTTTCAGTTTCATAT

GATTACTTGGGGATTTTCTCATTGCGAGCTAATGAATGGGAAGAAATTGTGGATACTGTC

CATTTGCTTTATTTTAGTGAAGACTCTTCCTTTGTTTATCCCTTAGTAGAGTCAGTCTTT

AATGGGGCTATTCACTGGTTGGCTTTACGTCTTGATACACGTGACTATCTTGTTGTTGCC

TTTGATTTAGTGGAAAGGAAACTTGTAGAGATTCCTGTCCCAGATGATATTGATTATCGT

TCTACGGATTGTGGTTTATGGGTATTTAGAGGATTTCTAAGTCTATGGGTTATGGTGGAC

GACGATACGGTTGATATATGGGTAATGAAAGAATACAAAGTGCAGTCATCTTGGACTAAA

GCTCTTGTTCTTACTACTTATGATACCGTTCATGGCGTTTCCCTTGTATGTTGCACAAAA

AGTGGTGATATTGTTGGAACAGATTGCCGTACTGGATTGGTGAGATATGATGAGGAAGGA

GAGTTTTTAGAGCATACCTACTATTGCAAAGATTCACGAAATGGATTCCGATTGGCTATG

TATACCGAGTCTTTGCTTTCACTCCCTAGTGACAGCGAGGAAGCTTAA

>MS.gene40983.t1

ATGGTGATGCCATTACTCGCTTTCATTGTTGCTGCACTTTTCATTCCCAATGGAGTAGTC

TTTGCAATTCCCTCCACTGTTCCCGCATTCCTTTGGTCATCTCATTATAACTTGATCTCA

GACAATGGATTGAAGGAGTCCGTTAATTATCAGGTCATTTCTCCGAAAGACCTAGCAAAG

TCTGTTTTATCTGAAGCAGGATGGTCAAATTTTCTGTGCAAAGGGAAGAAATTTCAGGAT

CCTCTCGATCTGGCACTTCTATTTGTTGGTGGAGAGTTACAGTCTTCAGATTTAAGCTCG

AACAAGCATGCAGACTCAGCTCTTTCTCACTTGCTCAAGGACTCTTTTGTCAGATCCAAC

ACTTCCATGGCATTTCCTTATGTTTCTGCATCAGAGGATGTGAACTTGGAAGACTCATTG

GTTTCTGGATTTGCTGAAGCCTGTGGAGATGATTTAGGAATTGGCAACGTTGCTTTCCTT

GGATCTTGCTCTATGGGCACTGGAAATCGTGAAGAAACTGCAGCTTTACACTCAGTTCAA

GCCTATTTGACCAAGAGGAAGGAAGAGAGCCACAAAGGGAAAACAGATTTGGTTGTGTTC

TGCAATGGTCCTCAAGCTTCACAAAATGTTGACAGAACACAATCTGAAGGTGAAGTTTTA

TCTGAGCTTATCAGCACTGTAGAGGAATCTGGGGAAAAATATGCAGTACTTTATGTATCA

GATATCTCGAGGTCAATCCAGTATCCTTCTTATAGGGATCTGCAAAGGTTTCTAGCAGAA

AGTACAACAGGGAATGGATCAACCAATTCTACAGCCTGTGATGGAGTCTGCCAGCTTAAA

TCATCACTTTTGGAGGGACTTTTAGTG

>MS.gene40974.t1

ATGATGCAAAACATTTTGCATCTGGATGACCATTCTAGCACCAGTAGAGGAGTGGATGAT

CAGACTCTCACTACTCCTAGTCAAAGTAAAATTGACAGGTCTCCAATGATGGACCGAGTT

TTTGTTGAACTCATGCTGGAGCAGGCGTATAAAAGGAAAACGATTAGCCGTACGTTCACG

AGGCAAGCATCGGGAGACATGGCAGAGTCCTTGAACATTATATTAAGGTGTCGGTACGGT

AAGGTTGTGTTGAAAAAATGTTTCAGTTGGGATAAGAGACAACACAAGGTTGTGGCTGAT

GATCAACCACTCCCAAACGCAACTTCGAATCTAGGAGGAGATAATGATTTTACCGGAGAT

ACCACTCAGCCTCTTGCTAATGCAGATAATGAAGGCAGTGAGAAGAACTCTAGTAGAGAT

GAAGGCGGTGAGAGGAACTCTACCAGAAAAAGTAATGTTATGACCATGCTTCAGGCTATA

CCAGACTTGGATGACGAGCTGATATTAGATGCATGTGATTTTCTGGAAGATAAAAGGAGA

ACAAGGATTGGATTCTAA

>MS.gene40979.t1

ATGGAGAAGAACCAGCCTGTTTTTGTCAAAGATGCTGTTTTGAAATTGCAATTGGCCCTC

CTTGATGGCATCCAAAGAGAAGACCAGCTGTTTGCAGCTGGATCTGTGATATCAAAGAGT

GACTATGAAGATGTTGTAACTGAAAGGTCCATCACTAATTTGTGTGGGTATCCATTGTGC

CACAATGCTTTGCCAACTGATCGTCCTCGGAAGGGTAGATATCGGATTTCATTGAAGGAG

CACAAGGTGTATGACCTACACGAAACTTACATGTTTTGTTCCTCCGGTTGTGTGATTAAC

AGTAAAGCTTTTGCTGGGAGCCTGCAAGATGAGAGGTGCAATGTTTTAGACCTGGAGAAA

CTGAACAATGTTCTTAGGTTGTTTGGAAATTTGAATTTGAACTTGGAACCAATGGAAAAT

TTTGGAAAGGATGGAGAATTAGGTTTTTCTGACTTGAAAATCCAGGATAAAACAGAAACC

GGCACTGGGGAGGTGTCTTTGGAACAGTGGGCTGGACCATCAAATGCAATTGAGGGTTAT

GTACCAAAACAAAGAGACAATGATTCTAAGGCTTCTAAGAAAAATGCTAAAAAAGGGTCC

AAAGCTAATTGTGGCAAGTCGAATGATTATAAAAGTTTAATTGGCAGTGAGTTTGACTTC

ATGAGTACAATAATTACACAAGATGAGTATAGTGTTTCGAAAGTATCATTGGGGCAAACA

GACACAACTGGTGATCATCAAATTAAGCCGCCATCCATATTGGAGAAGCCAAAAAGGGTT

GGCAATAAAGTGGTCAGGAAAGATGAAAATATACAAGATCTTTCTTCATCTTTTGAGAGT

ACTGTAAATTTAAGTACCTCAACAAAAGAGAAGGAAATAGCGAATTCATGTAAAGATGTT

CTCAAATCCTCTCATTCTGTTGAAAAGAAAGTTGTCCACTCGATTACCATTTCAGAAAGA

GAATGTGATGCAGAACAGAATGATTCTGAAAGGAAATCGATACAACTCAAGGAGGAAAAT

AGTATAGTTGCTGCTAACGATGATGCTTCCACTTCCAATTTAAATTCTGCCAATGTTGAA

GAGAAATTCCTAATTGAAAAAGCAATTGAATCATGCCATACTAAACCAAAATCTTCTCTT

AAATCTAACGGTAAAAAGAAACTCAGTCGCTCTGTTACTTGGGCTGATGAGAAAATCAAC

AGTTCTGGGAGTAAAGATCTTTGTGCAGTGAAAGAATTTGGAAACATTAAAAAGGAGTCT

GGTGTGGCTGATAACGTAGATTCTGCTGATGAAGATATGTTACGTTGCACATTAGCAGAA

GCTTGTGCAATTGCCTTGAGCCAAGCATCAGAAGCAGTTGCTTCTGGAGACTCGGATCCC

AATGATGCTGTATCGGAAGCAGGAATCACTATACTGCCACATCCTCCCAATGCTGTTGAG

GAAAGTACTGTGGACGATGATGACATTCTAGAAACAAATTCAGTTACTCTGAAATGGCCA

AAGAAACCTGGAGTTTCCGAATTTGATTTGTTTGACTCTGAAGACACGTGGTTTGATGCT

CCACCAGAAGGTTTTAGTTTGACTTTGTCACCTTTTGCTACTATGTGGAATGCCTTCTTT

TCGTGGATAACATCGTCTTCTTTGGCATATATATATGGGAGGGATGTAAGTTTTCATGAA

GAATTTCTATCAGTTAATGGGAGAGAATATCCTAGCAAAATTGTCTTGACAGATGGTCGT

TCATCTGAAATAAAACAAACATTAGTTGGTTGTCTTGCTCGAGCTTTACCTGCTGTTGTT

GAAGAGCTCAGGTTGCCAATACCCGTAGATATATTAGAGCAAGCGATGGTACGCTTGCTG

GATACAATGTCGTTTGTGGAAGCACTACCAGCATTCAGAATGAAACAGTGGCAAGTGGTT

GTTCTCTTGATCATTGATGCATTGTCTGTTTCTAGAGTGCCTACTCTTATCTCATACATG

ACGGACAGGAGGGACTTGTTTCTCAAGGTTCTGAGTGGTTCACAAATAGGCAAGGAAGAG

TATGACGTTTTGAAGGATTTCATTGTACCACTTGGCCGAGCACCTCATTTCGCTTCTCAA

AGTGGAGCTTGA

>MS.gene41000.t1

ATGCATTTGAAGCTAGGTCAAATTTCAACCATAGTAATTTCATCACCAGATATAGCACAA

GAAGTGCTTCAAACCCATGATCTCTCATTCTCAGATAGAACAATTCCTCAAGCAGTAGCA

GTTCTTGATCATGAACATTTTAGCTTACCCTTTATGCCAGTTTCAGATCTTTGGAGAGAC

CTAAAAAAATTATGCAAAAACCATTTGTTTTCTAGCAAAACACTTGATGCTAGCAATGAA

CTTAGGTGTAAGAAGCTTCAAGAGCTTTTAAGTGATATTGATAGAAGCAGCTTAACTGGT

GAAGCAGTAGATGTTGGAAGAGCTGCTTTTAAGACTTCATTGAATTTTTTGTCAAACACT

TTTTTCTCTATGGATTTTGTTAACTCCACAGGTGAAACTGATGAGTATAAAGATATTGTT

GAAAATCTTGTGAGAGCAATAGGAACACCAAACATGGTTGATTTTTTTCCTGTTTTGAAG

ATGTTTGATCCACAAGGTATCAAGGCAATATCAGCTACTTATGTTGAAAAGATGTTGCAA

ATCATTGATTCCTTTATCACCAAAAGGTTGAAGTTAAGAGAAAAGGAAAATTATGTCACA

AATGATGACATGTTAGATAATTTGCTCAACATCTCTCAAGAGAATGCCCAAAAGATGGAC

AACACTAAGATTAAACATTTGTTTCTTGATTTATTTGTTGCTGGGACTGATACAACTTCC

TACACAATAGAACGAGCAATGGCAGAACTAATCCACAACCCACATGCAATGTCAAAAGCC

AAAGAAGAACTTGAGGAAATAATTGGTATAGGAAATCCAATTGAGGAATCAGACATTACT

AGACTCCCATATTTGCAAGCAATAGTAAAAGAAACATTACGTTTGCATCCATCAGCTCCA

CTTTTGCTACCAAGAAAAGCCAAGATAGATGTCAAAATTCGTGGATACATTGTTCCTAAA

GAAGGTTCAACATCTAATACTGGACGCGTAAGGGAGGCAGGGCATGCTTCCCGTAGGAGA

GAGCGTCGCCAACGGCTGGAGGAGGAAGGTGGTGTTCCTCTGAGGCGTCGGGGTGCTAGA

GGACGATGTGCTGATGTCGAGCATCAGCCGGAGCAGCAACAGTATATGGACATGGAGCAG

GAGCAGCCTGATGATCAATATGTGTTGTTTATGTTCATAAGGAAACCTCGTTCTTTCTCA

GTCATTACTTCATCGATCGTGTGCTCAACCACTACACGAAATGATGTTGATGTTGAAAAT

GAAAGATATTCGTTGACCTTATCAATTTTCAATCTACCTGGTCGCCACTCTGGCAAGGCG

AGTGAACATTGGATGGTGGATCCGAAAGAGTACCGATCAACTCATATTCATGTTTTGATT

AATTGTGCAGAGGTTAAACCATACCTTAAGAAATTCAAGACCTTTTATGAAGAATTTGGT

GATGATTCACCTGGTTATGTACATGAATATTTTCCCGGTTGGCTTGATGCACAAGTTGAA

GGGGATCATGTTGATCATGCTGTTTTGTTGACTCCTAATGAAGATAAAAATGACGAGGAT

GATTCTGGTTCTTAA

>MS.gene41023.t1

ATGTCCTACCGCCCTAACTACCAAGGCGGAGGCCGCAGAGGCAACTCCTCATCTTCCAAT

CGCGGCGGAGGCCGCCGTGGCGGTGGCGGCGGTGGACGTGGTGGCGGTGGCCGAGGTGGC

GGTGGCCGAGGTGGCGAACAGAGATGGTGGGACCCTGTTTGGCGTGCTGAGCGCCTTAGA

CAACAACAAGCAGAGAAGGAGGTGCTTGATGAGAAAGAATGGTGGAGAAAGATAGAAACA

ATGAAAAGAGGAGGGGAACAGGAGTTGGTTATAAAGCACTACTTCAGTATTGCAGACCAG

CAAACCCTAGCTGATATGGCTTATCAACTTGAACTTTACTTCCATGCATATAATAAAGGG

AAGACTCTTGTTGTTAGCAAAGTTCCTTTGCCTGATTACCGTGCAGACCTTGATGAGCGT

CATGGATCAACACAGAAGGAGATTAAAATGTCCACAGACATAGAGAGAAGAGTAGGAAAT

CTTTTAAATAACTCACAGTCAACCTCACAATCAACGGCTACGGCAGCTGCTAGTATGCCT

TCTGCATCAACTGATATGGGACATAAAAAAACAATGACCACTATAAACTCTGCTTGTTCA

CAACAGACTGATACTTCAAAAGAGAAACTCAGTGTTGCACTCAAGGAAAGGCAGGAACTG

GAGCAGGCAAGTGGTAGTTTGAAGGAAATGAAATCATTTAGGGAGAAGCTTCCTGCATTT

AAAATGAAGTCTGAGTTTCTGAAAGCAGTTCAAGGAAATCAGGTATTGGTAGTTTCGGGA

GAGACAGGCTGTGGCAAAACAACACAGCTGCCACAGTTCATTCTGGAAGAAGAAATATCT

TGTTTGCGTGGAGCTGACTGCAACATAATCTGTACTCAGCCTCGTCGTGTGTCTGCAATC

TCTGTTGCAGCTCGTATTTCTGCTGAAAGGGGAGAAACTCTTGGCAAAACTGTTGGATAC

CACATCCGTCTTGAAGCAAAACGTTCTGCGGAAACACGCCTTCTTTTTTGCACCACTGGT

GTATTACTGCGGCAACTGGTACAAGATCCAGAGTTGACTGGTGTTAGTCATTTGCTGGTG

GATGAAATTCATGAAAGAGGCATGAATGAAGACTTCTTAATAATAATTTTACGCGACCTT

CTTCCTCGGCGTCCAGATTTACGCCTCATTCTAATGAGTGCCACAATCAATGCTGATTTG

TTCTCGAAATACTTCGCAAATGCCCCAACAATGCACATACCGGGATTTACTTTCCCTGTG

GTGGAGCATTTCCTAGAAGATGTGTTGGAGAAAACTAGATATAGCATCAAGTCGGAGTCT

GACGATATTGAGGGGAATTCAAGGAGAAAAAAGAAACAACAAGATTCTAAGAAGGATCCT

TTGGCTGAAATGTTTGAGGACGTTGATATAGATACTCATTACAAAAGTTACAGCTCGGGT

GTTAGAAAATCTCTTGAAGCTTGGTCAGGTTCACAGATTGATTTGAGTCTGGTAGAAGCA

ACAATTGAATATATATGTCGAAATGAAGGTGGTGGAGCAATTCTTGTATTTCTTACTGGC

TGGGATGAAATCTCTAAGCTATTTGAGAATCTTGAAAAAAATTACTTACTTGGAAACCGT

AGCAAGTTCTTAATCCTTCCAATACATGGTTCAATGCCTACCATCGACCAATGTGAAATA

TTTGACCGTCCTCCCCCTAATAAAAGAAAAATTGTGCTAGCAACAAATATTGCTGAGAGT

AGCATCACCATAGATGATGTTGTGTATGTCATAGACTGTGGCAAAGCAAAGGAAACCAGC

TATGATGCTCTTAATAAGCTTGCCAGCTTGTTGCCATCATGGATTTCAAAGGCTTCGGCA

CGTCAGAGACGTGGTCGTGCTGGTCGAGTACAACCTGGAGTTTGTTATAGATTGTATCCA

AAACTGATACACGATGCTATGCCAGAGTATCAGTTACCTGAAATCCTCCGAACACCTTTG

CAAGAGTTGTGCCTGCACATTAAAAGCTTGCAGCTGGGAACTGTGGCACCATTTTTAGGG

AAAGCACTTCAGCCGCCAGATTCTCTTGCTGTTCAGAATGCTATAGAACTTCTGAAGACA

ATTGGGGCATTAGATAATAATGAGGAGCTTACTCCACTGGGTCGGCATCTTTGTACTGTA

CCTTTGGACCCAAATATAGGGAAGATGCTTCTCATGGGTTCTATCTTTCAGTGTCTCAGT

CCTGCCTTAACTATTGCTGCTTCTCTTGCTTACCGCAACCCATTTGTCCTACCTATAAAC

AGAAAAAAAGAAGCTGATGAAGCAAAAAAATCTTTTTCTGCTGACTCTTGCAGTGATCAT

TTAGCCCTTCTTGAAGCTTTTGAACAATGGAAGGACGCAAAAAGTAGGGGGGATGAAAAG

AATTTTTGTTGGGAAAATTTCCTATCCCCAGTGACCTTGCGGTTGATTGATGATATGAGA

ACACAATTTCTCGATTTGTTATCTGATATTGGGTTTGTTGACAAGTCAAAGGGTGTCCAA

GCTTACAACCAATACAGTCATGATTTGGAGATGGTTTGTGCAATTCTTTGTGCTGGTCTC

TACCCTAATGTTGTGCAGTGCAAAAGAAGGGGACACCGAACAGCATTCTACACTAAAGAA

GCCGGGAAGGTTGACATTCATCCCTCTTCTGTTAATGCCGGAGTTCATTCCTTCCCTCTG

CCCTACTTAGTATATAGTGAAAAAGTGAAAACAACCAGCATCTATATTAGAGACTCTACC

AACATTTCAGATTATGCCCTACTTCTATTTGGTGGTAATCTTGACCCTAGCAAAAATGGT

GAGGGCATTGAGATGCTTGGGGGTTACCTTCATTTCTCTGCTTCCAAGAGTGTCATTGAG

TTGATTAAGAAATTGCGTGGGGAACTTGACAAGCTTCTAAACAGAAAAATTGAAGAGCCA

GGTTTTGACATTTCTGATGAAGGAAAAGCAGTGGTTGCTGCTGCTATTGAATTGCTTCAT

AATCAAGTCATGCAATGA

>MS.gene41020.t1

ATGGCCGATAGTTTAAAGCAAAATAATGTTGATACATCTGGCATGCGGTTTCATTCTAAT

GCCAGAACCACATTAGCTTTTGTTACACATAGAGCTAATGGCGAGCGCGAGATATTGTTT

TTCCGAAATCCTAAATCTGATATGCTTCTGGATAAGTCAGAGCTTGATCATAACCTCATC

AAGCAGGCCAAAATATTCCATTATGGTTCCATCAGTTTGATTGACGAGCCATGCAAGTCG

GCTCATCTTGCTGCCTTGAGAATCGCTAAAGACTCTGATTGCATTCTCTCGTATGATCCG

AATTTGAGATTGACACTATGGCCATCAGCCGAGGCTGCTCGAAATGGTATAATGAGCATT

TGGGATCTAGCCGATGTAATAAAGATCAGTGAAGATGAGATTACTTTTTTGACTGGTGGT

GGTGATCCTTATGATGACGATGTTGTATTAAACAAGCTTTTTCACCCTAATCTCAAGCTT

CTAATTGTTACTGAAGGGTCGGAAAGTTGTAGATATTACACCAAGGATTTTAAGGGCAAA

GTTAGAGGTGTCAATGTTAAACCTGTTGGCACAACCGGTGTTGGTGATGCATTTGTTAGT

GGGATTCTCTACAAGATAGCTTATGACCCAAGTATTTTCGAGGATGAGGAAGGTCTCCAA

AAAGCGTTAAAATTTGCAATGGGCTTTGATGTTTTATCCAAAAAAAACATTGCAATCACT

GACGATGACCACTCCCGACCTACTTCTCCACTGCCGCCTGTTCTCGAACCTCAGACTTCC

GATTGCCCCGTTAACCATCACCTTTTCAAACTCGGGTTTGGTTTGAAGGCAGACAAGGTT

GCTGAATGTCTTCGCCAACTAGGGGATTTGTTAAATGGCTTCGATGTCCCCCAAAAGGCG

AAAATCTGTTTCCAGAAATTTCTGTTTTCCGATCTCAATTCTTGTGGTAGTGGTGTACTG

CCTCATATTGATGAAAAAATGCTCAAAACTTCTCTCCATGGTCCCTTCATATTGCAGGTC

GATGAAATTATCCATGGCTGTTGTCCTCTACAAATTCAAAGGTGCCTGATGTTCAAAATG

ACCGATGGTGTTCAAACAGTTTATGGAATAGAGCTCAAGCCTATTGAAGCCCTTCAAGTT

TGTGCGCCTCGTGGATTAAAGGTCGAAATCTCTAATGTTGAGGTGAGACGTGGACTATTG

ATTCTGGTTCCTGAAAATATTAAGGTATTGGGTGGGGGATTAGTTAAGCAGCTAGATGAA

GAAGTTCTGAAAGAAGTTCTAAAGCCCGTGGTTGATGATCCTAGTCACAGTAGCTCAACG

TTGAACATCACTGATGCTGTCCAGGCAAGCAACCAAGGTAAAACTGATGATCTCATAACC

GAAAGCTCCAACAAAACAAGCGGACTGGTTGTTTGTTTTGGTGAAATGTTAATAGGTTTC

GTGCCATCAATGGGTGGAGCGTCTCCAGCCGAAACGGCTGCTTACAAGAAAGCTCCTTGT

GGTACCGCAAACGTGGCAGTTGGCATCTCTAAGTTGGGGGGTTCATCTGCATTTATAAGC

AAGGTTGGAGCAAATGAATGTGGGTATATGTTGGCTGATATTTTGAAGAAAAATAATGTT

GATACATCTGGCATGCGGTTTGATTCTAATGCCAGAACCACGTTGGGTTTTATTACATTT

GGAGCTGATTGCAAGCGCGAGATATTGTTTTTCCGAAATCCTAGCGCTGATATGCTTTTG

GATAAGTCAGAGCTTGATCATAACCTCATCAAGAAGGCCAAAATATTCCATTATGGTTCC

ATCAGTTTGATTGACGAGCCATGCAAGTCGGCTCATCTTGCTGCCTTGAGAATCGCTAAA

GACTCTGATTGCATTCTCTCGTATGATCCGAATTTGAGATTGACACCATGGCCATCAGAC

GAGGCTGCTCGAACTGGTATAATGAGCATATGGGATCTAGCAGATGTAATAAAGATCAGT

GAAGATGAGATTACTTTTTTGATTGGTGGTGGTGATCCTTATGATGATGATGTTGTATTA

AACAAGCTTTTTCACCCTAATCTCAAGCTTCTTATTGTTACTGAAGGGTCGAAGGGTTGT

AGATATTACACCAAGGGTTTTAAGGGCAAAGTTAGAGATGTCAATGTTGAACCTGTTGAC

ACAACTGGTGCTGGTGATGCATTTGTTAGTGGGATTCTCTACAACATAGCTTCTGACCCA

AGTATTTTCGAGGATGAGGAACGTCTCCGAGAAGCGTTATGTTTTGCCATTGTATGTGGC

GTGATCACGGGGACGGAGAGAGGGGCACGGGGACAGAGAGAGGGGCAATTCCTGGATTAC

TTGCAAAAGATGTTGTCCAGCAATCTGATGCAATATAGGAATATCAGCTGTTATAAAATG

TAA

>MS.gene41022.t1

ATGAACAACTTATTCAGCGGTTCATTTTCTCGCTTCCGTAGTGAAGAAGTTTCACCGGAC

CGGCACCACGTCATCGAGATGACAGACGGTGCCGGAGCCAGAACCGGAGCAGGAGCAGGA

GGTAGAATAAACCTCGACAAATTCTTCGACGACGTCGAAGGAGTAAAAGACGATTTAAAA

GAGTTGGAATCAATCCACCAACGACTTTCTAAGACTAACGAACAAACCAAAACGGTTCAC

GATGCTAAAGGAGTGAAGGAGCTTCGTTCACGTATGGACGAAGAAGTTTCTGTGGCGTTG

AAGAAGGCGAAGATGGTGAAGCTGAAGCTGGAGGCGTTGGAAAGATCTAATGCGGCGAAC

CGGAATTTGGAAGGGTGTGGACCGGGTTCGTCGTCGGACCGGACGAGGAGTTCGGTGGTG

AATGGGTTGAAGAAGAAGTTGAAGGATTCAATGGAGAGTTTTAATCGTTTGCGTGAAGTT

ATTACGACGGAGTATAGAGAGACTGTTCAACGACGTTATTTTACTGTTACTGGAGAGAAT

CCTGATGATAAGACTCTTGATCTATTGATCTCTACTGGTGAAAGTGAGACCTTTCTTCAG

AAAGCAATACAAGAACAAGGCAGGGGAAGAATTCTGGACACCATCAATGAAATTCAAGAG

AGGCATGACGCTGTCAAAGACTTGGAGAAGAGTCTACTGGCATTGCACCAAGTGTTCCTC

GACATGACAGTTATGGTACAGTTTCAAGGTGAGCAATTGGATGATATCGAGAGCCATGTG

GCGAGGGCGAGCTCGTTTGTGCACACTGGTACCGAACAGTTACAAACTGCAAGGAAGCAC

CAGAGAAACACAAGGAAATGGACCTGTTATTGTATTATACTTCTATTGATCATTGTCTTG

ATCGTGGTTCTTGCCACGGTGAAACCATGGCAACATAATGATAGTGGAGGCGGTGGGGGT

AATCAGCCTGCTCCAGCACAGACTCCCCCGTCACCTCCTCCTCCTTCGGGTGGTACTTAA

>MS.gene41019.t1

ATGGCTAGTGCAAGCACTGGTTCCTTCCCTATCTGCCACCACAGACTCTTCAACTTTTCC

TTTACTACTCATTCCTCCATTTCCTTTAACTTCCCACACAGAACAGCAACAACAACCATT

TCTACAAAACCCTTCAATTTAATCCCTCAATTATTCAACCCTCTTCATCCACCTCCTTCA

TTTTCATTCCATGAGGAGGAAGAACAACAATACTCTGAATCTGAATCTGAATCTGAAACT

GAAATTGAAACCTTACAGAACTCAGACCTAAAACAGGAAGCAGAATCAACCCAATCAAGG

AGACTCTTTGTTGGAAACTTGCCATTTTCATTATCTTCTTCCCAATTGGCTCAACTCTTT

GGAGAGGCTGGAAATGTTATATCTGTGGAGATTGTGTATGATGACATCACAAATAGAAGC

AGAGGATTTGCGTTTGTTACAATGGAGAGGGTGGAGGATGCTGAAGAATCAATTCGAATG

TTTGATGGCACTACAGTTGGAGGTAGGGTTATTAAGGTGAACTTCCCAGAAGTGCCCAAA

GTAGGCAAAAGGGTACAAATAGGCTCAAACTATAGAGGTTATGTTGACAGCCCTCACAAG

ATCTATGCTGGAAACCTTGGTTGGGATATGACTTCCCAGGATCTTAGAGAAGCCTTTGCT

GAGCAATCAGGCTTACTGAGTGCCAGGGTCGTCTACGAAAGGAACAACGGAAAATCTCGC

GGATATGGATTTGTCTCTTTCGAAACTGCTGAGGATGTAGAGGTTGCTTTGAGTGCTATG

AACGGCGTGGAGGTTCAAGGCCGACCTTTACGACTGAATTTGGCTGTGGATAACAGGAAG

CCTTCATCTCCTCCGTTTTGTAATGATGGCGAGGGACTAGAGCCTAAGTCTTATACAATC

GGCTTTACCGACTTTACCGTGCAGTCGACTGTAGACTGTTAG

>MS.gene41029.t1

ATGGAGAGTGAGTGGAAAGCTAATCAGATATCACCAAATTGTCCAAGATGTGGTTCTTCA

AACACAAAATTCTGCTACTACAATAACTACAGCTTAACTCAACCAAGGTATTTTTGCAAA

GGATGCAGAAGGTATTGGACAAAAGGTGGATCCCTCAGAAATGTTCCTGTTGGTGGTGGC

TGCAGAAAAAACAGAAGAGGTAACAACAAAACATTAGTAAGACATAGATCCTTTGACTCT

CTCACTTTCAAAAACTCACCTAATTCTCACACTACTTTAGATAACAATAACTCAATTGCT

CATCATTCTTATGATCCTATTTTGAGTACTAATATTTCATCTTCTAGCTCCTCATTGGTG

AATGATAGACCCAATATTGATCTTGCATTGGTTTATGCAAATTTCCTCAATCAAAAGCCT

GATTCTCATAGTTCCGATCAAATGCGAAAAACAAATTTTGATCCTTGTCTAGAAAATTCA

AGGCTATCGATTGTTGAAGGCGGTACGAGTAGTTTAAATTTGTCTGAATTAGAACTTGGT

TTCAAAGGGTGTTTGAATCTTAATCCAGAACAATTACCATCCACAACAGTAACACATTTT

AGTGAGTTCAATTCTATGCAAACCCTTCAAAAAGATAGAATTGATCATTGTAGTAATATT

CATGATGATGCTGTGAATGTGAATTTTGAGCTACCACCTTTACCAGGTGAAGAGGATTCA

TCATCACAAGATCATGTATTGTGGTCCAATTCTGAAATGATGATTAACCTTCCATTTCAA

GTCACTCAACCTCCACCACCATTTCTTGGTCCTGATCAAATTCATGATGCAGACTTGTTA

ATCGGTAATTGGAGCCCTTTTGATTTTCCAAGGGATGCTTAA

>MS.gene41001.t1

ATGCATTTGAAGCTAGGTCAAATTTCAACCATAGTAATTTCTTCACCAGATATAGCACAA

GAAGTGCTTCAAACCCATGATCTCTCATTCTCAGATAGAACAATTCCTCAAGCAGTAGCA

GTTCTTGATCATGAACATTTTAGCTTACCCTTTATGCCAGTTTCAGATCTTTGGAGAGAC

CTAAAAAAATTATGCAAAAACCATTTGTTTTCTAGCAAAACACTCGATGCTAGCAATGAA

CTTAGGTGTAAGAAGCTTCAAGAGCTTTTAAGTGATATTGATAGAAGCAGCTTAACTGGT

GAAGCAGTAGATGTTGGAAGAGCTGCTTTTAAGACTTCATTGAATTTTTTGTCAAACACT

TTTTTCTCTATGGATTTTGTTAACTCCACAGGTGAAACTGATGAGTATAAAGATATTGTT

GAAAATCTTGTGAGAGCAATAGGAACACCAAACATGGTTGATTTTTTTCCTGTTTTGAAG

ATGTTTGATCCACAAGGTATCAAGGAAATATCAGCTACTTATGTTGAAAAGATGTTGCAA

ATCATTGATTCCTTTATCACCAAAAGGTTGAAGTTAAGAGAAGAGGAAAATTATGTCACA

AATGATGACATGTTAGATAATTTGCTCAACATCTCTCAAGAGAATGCCCAAAAGATGGAC

AACACTAAGATTAAACATTTGTTTCTTGATTTATTTGTTGCTGGGACTGATACAACTTCC

TACACAATAGAACGAGCAATGACAGAACTAATCCACAACCCACATGCAATGTCAAAAGCC

AAAGAAGAACTTGAGCAAATAATTGGAATAGGAAATCCAATTGAGGAATCAGACATTACT

AGACTCCCATATTTGCAAGCAATAGTAAAAGAAACATTACGTTTGCATCCATCAGCTCCA

CTTTTGCTACCAAGAAAAGCCAAGATAGATGTCAAAATTCGTGGATACATTGTTCCTAAA

GGTGCACAAGTCCTAATTAATGAATGGGCCATGGGAAGAAACCCTAATATTTGGGATAAT

CCAAATTTATTTTCACCTGAAAGGTTTTTGGGTTCAGAAATTAATTTTAAGGGCCAAAAT

TTTCAATTGACACCATTTGGTAGTGGTAGAAGAATGTGTCCTGGAATGCCACTTGCTATA

AGAATGTTACATACCATGCTAGGATCGTTGATTAATTGTTTTGATTGGAAGCTGCAAAAT

GGTGATAGTGAAATTGGTCAACCCTTGCGAGCTATTCCTTTTAGAGTAAATAAGGTGTAA

>MS.gene40992.t1

ATGACCAACCTAAGATGTCTTCAGATCACTAATAATATAAATAATCTAGAAGGGTGTGAA

TTGTACACTGTGCACCTCCTTGAAGGTCTTGAGTGGTTGTCTGATAAATTGAGGCACCTT

TTTTGGGACTCATTCCCACTTGAGTCTTTGCCATCAACCTTTTGTGCTGAATGGCTTGTA

CAAATTAGCATGTCCCATAGCAAGCTAAGAAAGCTTTGGGATGGAATTCAGAGGCTTGAC

AATTTAATGATCATTGACCTTAGCAACTCTAAAGATCTGATTGAGATTCCGGACTTATCA

AGAGCACCAAATCTTCAAATACTATCTCTTTCTAATTGCGTGAGTCTCTGTCAAATCCAT

CCATCCATTTTCACTGTGCCCAAGCTTAGAGAGCTACATTTACGTGGTTGCAAAAAGATT

GAAAGCCTGAAAACTAACATTCATTCAAAATCTCTTGAAAGTCTAGATCTCTCGGATTGT

TCATCTCTTGTGGAATTTTCTGTGACATCAGAGGAAATGACATGTTTGTTGTTAAGTCGC

ACCGCTATACATGAATTTCCTTCATCAATGTGGCGGAATAGAAAACTCACTTATTATCTC

GATCTAAGTGAATGTAAGGAGCTTAACATTGTTGGGAAGAAGTTATCAAATGATCCAGGA

TTAGAGTCTCTTACATATTTGAACCTCTCAGGATGCACACAAATCAATACATTGAATATG

TGGTTCATCCTTGATGGCATGCGATCATTAATAGATCTCGTTCTGAATGAGTGCTGCAAC

TTAGAAGCTCTCCCTGACAACATCCAAAACTGTTTAATGTTGGAAAAGCTTTATTTAGAT

GAATGCATGAACCTTAAATCCCTACCAAAGCTTCCTGCATCCTTGCAAGAATTGAGGGCC

ATTAACTGCACTTATCTGGACCTAGACTCCATTCAAAGAGAAGTGCTTGAGAACTACGAC

GTGCATAGATTAGATGATCTTGATGCACTCTCTTTCCGTAATATCTCTTTACTTCCTGGA

TCCCAGGTTCCGTGCGAGTTTGCTTATCAAACAACAGAGGCTTCAATTGTTATTCCTCAT

ATTCCAAAATCAGGCTTATGTTCATTCATTTTATGCATCATTCTCCCAAAATTGCTCGAT

CTTAAAGACCACAACATTATCTGTACTATCTATGAACATGGAAAAGGAGTCCGCCAATAC

TCCTTATGTCATAAAACAAGGGCATCCGGATGCACTAAAGCTCCCTCATATGCAAGCGGT

AAAAATGGAATATTAAATTCAGATCATGTAATGTTAGGCTTCTTTTATAGCGACGTTTTG

GTGAAGGAGGGGAATGAAAGTGGAGGTGATCATTACGACCTTTCATTTAAATTTGATACC

ATAGACTGTTCACGTAGTATTTACGAAGGCCAGTGGTCAACTGACGGGATAAAAGGTTGT

GGGGTCATACCTGTATACAACTTGGATGGTAGAAGAAGAAGCAACAATGTTGAAATTGTT

GAATTAGCATCATGTGATCAAGTCTCTGATGAGTCTGATCAACATTCGAAATTTGACATT

GATGAGTTGCAACACCGAGCAATTGGAGCTGAAGGTAGAGACTCTACACACGGAAATGAA

GATGACCAGCAACAACAGAAAAAGAGGAGGACTTGA

>MS.gene41002.t1

ATGTTCTTGGTTCCGATTGACTCACGGTTTCAAAGGGCTATGTCTGGTCTTACGGCGCAA

TTCGTATGTCCTTGGCCTTGCTTGGTCTCACATACGCCGAAGCCTACTCTGAAGCCTGTC

TCAACTCGTAGTTTTGCACAGGTTCTTCTCAACAAAGTGAACGTCCCTTTGTCTGATCTC

CCGAAACCTTGTTTGAAAGATCCTGCCAACAAAATTGCTCGTGAAAAGGTGACCACTAAA

GAAAATCTTGTTAAGAATGTCCCTAAAAGGCATGCACAAGGCTGTAGTGCTGTGGTTGAT

GAAGATCCTATTATTCTTGACATAATGAGGTCTAAGGAAGTAACAACTAATGTGTTAGTG

GGTGATGTTATAAACCTTGAGGAAGAACTATATTCTAGCGCTTTCGTTGGGGCAATTTAG

>MS.gene41006.t1

ATGGAGGGAAATTACAACCATGTTCCTTTCCAAACAGAACCACCTTTGTATCCTCCAACA

TGTGGTTCAGTTTGGGATGCTACCTTTAACCACTCTACTGCAAACTTAGGAGCCAACTTG

GGTCTTAATAGTGACCAAAACAAGGGTCCTAAGAGGATCAGACTGAAATACATTGAAACC

TTTAATCGAGACTCGGACTCAGCTCATAGATCAAGACGGAAGGAAGATCGGTATTTTTCC

TATTTGAGTAAAAAAGTTAAAGAAATTGAGGCTCAAATAACTGATCTTCGCTCACAAGTT

GAATTAAAACAAAACCAAAAGCACCGTTTGATGTTGGAGCGTGTGACGTTGATGGATCAA

GTGACAATTCATCATAAAGAAGCACATCTTAAAGACGCTGAAAGTGAAGAACTGAAAATC

CAAGTGAAGAAATTAAGGGAACTCTACGTCGATCAACAAGCTGAGGAACTAGCACAGCTG

ATTAATGGGAATGATACTAATGTTCAACCAATGTCCAATGATTTAGACTCAAATTATGTT

TCAAAACCATATGATCAAGGACTATTACAAAGCCAAATGTGGACGCCACCTATGTCGGAT

GAGCACGAAACAGAACACAACAACATGGAATTGGGCATGGAATGGATGGATCAGAACCCT

AATATTGACGATCTATTTCTATGA

>MS.gene40995.t1

ATGGTTGCAAACGAAACTTCACGGTTTGAGAGCAGTGAGATGCTGGCAACGTTCTTGATT

TCTACGCCACTGTTGTCGGAGTCATGGAGGATGTGCAGTCAGGCCAAAGCCTCCGCCGTC

AACCTCCGCAGTTTTGTGGTGGAGCGTGTTGACAACGTCGTGTATGTAGCTTTCTCCGGC

GTTCAAATGGCCGGCGGGGGATCCGATCCGAGTTGGAGAACGGTGGAGCCGTTGGAAAGC

ATCGGTGGAGTGCCGTTGTTTTCGACGTGTCGGAATAAGGAAAAGGAGGAGCCGGTGAAG

GTTCACTCCGGGATGTTGAATCTCTTTTCTTCCCTTTTCAAATCAATCCAAGACCAGGTG

TTGGGTATATTAGAAAATACAGACACAAAATCACTTGTGATCACTGGCCATTCCATTGGA

GGAGCTATAGCATCTCTATGCACTCTTTGGCTTCTTTCTTACCTCAAAACCATCTCTTCA

TCGTTGCCAGTAATGTGCATCACTTTTGGTTCACCATTACTTGGTAACAAATCATTTTCC

CAAACCACTTTTGGTTCACCGTTACTTGGTAACAAATCATTTTCCCAAGCCATTTCCAGA

GAAAAATGGGGTGGAAATTTTTGTCATGTAGTATCAAAACATGACATAATGCCAAGATTG

CTATTTGCACCTATAACACCCCACACTTCTCAGCTAAATTTCGTGCTTCAGTTTTGGCGC

TTCTCCAGGACTTCACCAGAATTTGGAAAGCTTGCAGTTCAAGTTTCCGAGAAAGAAAAA

GCTGAGTTGTTCACTTCTGTATTGGATTCTTTGGAAAATGGTGAAGCAGCTGATGCTTCA

GTGCCAATTTTGTTTCATCCTTTTGGAAGCTATCTGTTTGTTTCAGAAGAAGGTGCTTTA

TGTGTGGACAGTCCACACACAATTATTACGATGATGCGTTTGTTACTTTCAACTAGTTCT

CCAACTAATAGTATTGAGGAACATTTGAAGTATGGAGAGTTAGTTAATAGGTTGTCTTTG

GAAATATTGAATAAGAAAAATACTAAGCTTAGGAACATTCCTAACTCAAGCTATGAGGCA

GGGCTTGAATTGGCCATCCAATCTTCTGGTTTAGCAAATAAGGAATCTGCAGCGATACCA

GCCATAGAATGTTTGAAGTCAGCAAGGAGAATTGGTCTATCACCAGCTCTAAAAGCTGTA

AACCTACAACTATCCTTAGCCAAGGTTGTACCTTTCAGAGCCCAAATAGAATTGTACAAA

AGTTGGTGTGATAAACAAGATGATCAAATGGGATACTATGACACATTCAAAACAAGAGGA

TCTGCCAAAGTACACATGGAAGTCAACAAGATCCGTTACAAACTCGCTAGATTCTGGGAC

AGTGTGATTGATATGTTTGAAAAAAATGAGCTACCACATGACTTTGACCAAAGAGTCAAA

TGGGTCTACGCTTCACAATTCTATAAGCTTTTGGCTGAACCACTTGATATAGCTGACTAT

TATAAGCAAAGGAAGCATTTAGAAAAGGGTCATTACATAGAACATGGTAGAGCGAGAAGG

TATGAGATTTTTGATAGGTGGTGGAAGAATAGAGAAGTTACTATTGGTGAAGAAAATAAA

GAGAGAAGCACATTTGCAAGTTCAACACAAGATTCATGTTTCTGGGCTAAAGTTGAAGAA

GCAAGGGATTGGTTGAATGGTATGAGAAGTGAAAGAGACACTAACAAGTTGGATACATTG

TGGGGGAAAATTGAAAACTTTGAGAAATATGCCATTGAGTTGATTCAAAAAAAGGAGGTG

TCTATTGACGTTCTGGCTAAGAATTCAAGTTACAGTACATGGGTGGAAGATTTAAAAGGG

TTTAGACAATTGAGAGCAAACGTGCAGAGGTTTCCTCAACAGTTTAGATGA

>MS.gene41030.t1

ATGGAATTGCCAAACACAAAACATGCATTTTGCATATGGCATATTGTCGCAAAGCTGTCA

AGTTGGTTTTCTTTTCCACTAGGTTCGAGATATGATGACTTTAAACATGAGTTTCATAAG

GTGTATCATTTAGAATGTGCAGATGATTTTGAACGAGAATGGAAAGTTGGTGTTGCTGTC

AAAATTAGAAATCAAGCTGGAGAAGAGGCACAAATGCGTCAAAAGTATCATAATCCTCAG

ATTACAACACATTTTCCTATTGAAGAACATGCCTCGTCTATACTCACACCTTATGCCTTT

AAGTTGCTTCAACATGAGATCGAATTATCTGTAAAGTATGCAGCAATTGAAGCTGACAAT

GGCTCTTATGTTGTGCAACATCATACCAAAGTTGATGGTGGTCGTATATCTCTAATTCGA

TGTTTGGAAGTCGAATCATTAAAGACAAGTGAACGAGAACAGGTTGCCACAAGAGAGTTA

GAAAATATCATCATAAAGATAAAAGGCATGCCTAAAACTCAAGAACATGTGATTGATTTG

GAAGCGGATGTTTCCAATAATGATGACTGTGACGTTGAAAATCCTGTTGTTTCAAAAACA

AAAGGTCGGCCAAAAGGATCAAGACCAAAAGGAGGGGTTGAAGCTGCCAAGAAGCCTCGT

CGTTGTCATGTTCCAGATTGTGGTGGAACTAATCACGACTCACGAAATTGTCCAAACAAA

AAGAGAAAAATGGAAGTGTTGCCTTCTCAATCTCCTAATAAGATCATGAAGGGTTGGAAG

TAG

>MS.gene41024.t1

ATGACGAATTTCCCCCCTCAATCACCCTCCTTGAAGAATAATGAATTTTTTCCTGTTACA

TCTGCCTCCAGCACCATTGGCAACAAGATGTTGTACGCTGAGCGTCTTGTTATTGAACTG

AGCAACCCCGATCTACGAGAAAATGCTCTTCATTTGCTCTCCAAGATGACAGATTTGTTT

CAAGAACTCGCACCGTTATTATGGAATTCAATTGGTACTATTGCAATATTTTTACAGGAA

ATAATTACAATATATCCTGCTTTTTCACCAGAAAATCTTACTGCTGCACAATCAACTCGA

ATATGCAATACCCTTGCTCTACTTCAGTGTGTGGCTTCCCACCCTGACACAAAGTTTTCA

TTCATGAAAGCTAACATACCTATATATCTGTATCCCTTCCTTAATACATCAAGTAAGTTA

GCTCCATTTGAAGATTTAAGACTTGCGAGTCTTGGAGTCATTGCTGCCATGGTAAAGGTT

AAGACTAAGGAAGCGATAGGCTTCCTTCTCGCAACTGAAGTAATGCCGTTGTGCCTACGC

AATATGGAAATTGGCAAAGAATTGTCAAAAACTGTTGCAACATTTATCGTTGAGAAAATT

TTGTCAGATGATGATGGTTTGGCGTATGTTTGTGCTACACCAGATCGTTTTTTTGCAGTT

GGTCGAGCTTTGGACAGGATGTTGGCAAGTGTTGACAATCAACCTTCACCTCGTCTTTTA

AAGCTTATGATTCCGTGTTATACAAGTCTCACAAAAAATGTAGGGTTGTTTAAGCCGAAC

CCAACTCCACCATCTTAA

>MS.gene40990.t1

ATGGGTTTTAAGGAGAAACATGAGTGCACTGAACCTGTTTGTGAAAATGCCGCAGAGAGT

GTGGTTCTTGATAAAGATGAAAACTTTACCGTTTTGGAAGATGCCCAGTTGGTTAAAATG

GGAGAAGAAGATATGTGTCGAATGGTGGGTGAGATTATGGAAATAATTCGAGGTGAAAAT

GGTTCGGTTTCTGTGGAAGAGCGATTAGAAAAGATGGGTTATTGTGAATTGAATGTTGAG

GTTTTCGATAGAGTTTTGAAAAGGTGTTTTAAAATTCCGCATTTGGGTTTGAGGGTTTTT

AACTGGTTGAAGCTTAAGGATGGTTTTCGTCATACAACACAGACTTATAATACCATGTTG

TGTCTTGCCGGAGAAGGTAAGGAGTTTGGGTTGGTGAAGAAGTTGGTGGAGGAAATGGAT

GAATGTGGAGTTCAAAAGGATGTAAATACGTGGACCATTCTTGTGTCTCTTTATGGGAAG

GCGAAGAAAATCAGTGAAGCTTTGTTGGCGTTTGAAAATATGCAAAAGTGTGGCTGTGAA

CCTGATGTGGTTTCGTATAGAACGATCATTCGTTTGCTTTGTAGTTCGGGTAAGGGTGAT

ATTGCTATGGAGTTCTATAGGGACATGGTCAAGAAGGACATTGTACTTGATGATGTAAGG

TTGTATAAGATGCTTATGAACTGTATGGCAGAATCAGGAGATGTTGCAGCTGTTGGTTTG

CTTGGAAATGACATGACTCGGTTGTGTCTGATGCCGGAAAACAGTGTTTTTGGGTGTATG

CTTAAGAGCTTTTGTATTTCTGGTAGGATTAAAGAAGCTTTGGAATTGATTCGTGACCTC

AAATATAAAGATGTAGTTCTTGAACCTGAATATTTTGAGACCTTAGTGAGAGGGCTGTGT

AAGGCAGGAAGGATTTCAGACGCTTTAGAGATTGTTGAAATTATGAAGAGAAGGGACATT

GTTGTGGGGAACGTTCAAGGGATTATCATAAATGGCTATTTACGGAGAAACGATGTTTGC

TTGGCACTTGATGTGTTTCAAAGCATGAAAGAATCTGGTTATGTGCCTACAGTTTCCTCA

TATACTGAACTGATACAGCATCTGTTTAAGTTGAGTAGGAATGAAGAAGCTTGCATGCTG

TATGATGAGATGCTCGGAAAAGGAATTAAACCAGATATTGTGGCCATAACAGCCATGGCT

GCAGGCCATGTTTCACAAAATCGTATATCCGAAGCCTGGAAAATTTTCAAGAGTATGGAA

AGCCAAGGCATCAAGCCTACATGGAAGTCGTATTCGGTATTTATTAAGGAGCTTTGCAAG

GCTTCAAAGACCGATGACATTGTCAAAGTATTGTATGAAATGCAGTCTTCAAAGATTGTA

ATCAAAGATGAAGTATTCCGTTGGGTTATAACTTACATGGAAACCAAGGGGGAGTTTGCT

TTAAAAGAGAGAGTGCAAAAGATGCATACAACATCTAAACTCTATCCTGAAAATTTTGAG

GAGTCTGAAAAACGAGTGTCATTGAGAAATGAGGTGGAAGAAGATAGAGGAGTTGACCAA

CCAAAATCAGAAAAGGTAGACTGCTCATTACTAATTCCAATTCTCAAGACTTACAGTGAG

CAAGATGTTCGCGATGTATGTAGGATTCTTTCATCCTCTCTAGATTGGTCTTCAATCCAA

GAAAAGTTAGAGATAAGTAACATTGAGTTCACCCCAGAATTTGTTATGGAGATAATGCAA

AGCTGCAGTATGCATGGCTGCTCGGTGCTAAATTTCTTTTCGTGGGTGGGAAAGAGACCT

GGCTACAGACACACCACAGAATCATACAACATTGCAATCAAAATTGCAGGGCGCGGGAAA

GATTTCAAGCACATGCGCAGCCTGTTATATGAAATGAGAAGAAACAATTATCCAATAACA

TCAGAAACATGGACGATCATGATCATGATTTACGGCCGAACAGGTTTGACAGAGATGGCC

ATGAATTGTTTCAAAGAGATGAAAGATGGTGGTTATTGCCCAAGTAGGAGTACATACAAG

TATCTGATCATCGCCTTATGTGGGAGGAAAGGAAGGAAAATTGATGATGCTCTCAAAATA

TACGGTGAGATGATTAATTCCGGACATGTCCCAGACAAAGAATTGATTGAAACCTACCTT

GGTTGCTTATGTGAAATGGGTAGCATTGTAGAGGCTAGGAAATGCATAGATTCACTTCAA

AGATTCGGCTACACGGTCCCTCTTAGCTACTCGTTGTTTATAAGAGCTCTTTGTAGAGCT

AGAAAAGTGGAAGAAGCATTGAAATTAGTTGAAGAAGCTGGTGCAGAGAAAACTAATGTT

GAAAAGCTAACTTATGGAAGTATTGTACATGGTTTATTACAAAAGGGTAAACTAGAAGAA

GCATTAACCAAGGTGAATACAATGAAACAAGAAGGAATAACACCTACAATACATGTTTAC

ACATCTTTAATAGTTCATTTCTTTAAAGAGAAACATGTAGAAAAAGCTATTCAGATATAT

GAGGAAATGCTAGAATCGGGTTATGAACCAAATGTTGTTACATATTCGGCACTTATACGT

GGGTACATGAACGTGGAGAGGTATAATGATGCATGGAATGTGTTCTACCGTATGAAATTC

AAAGGACCATTTCCTGATTTTAAAACATATTCTATGTTCCTTTCTTGTCTTTGCAAAGTT

GGAAGATCTGAAGAAGCAATGAGACTTCTTACTGAAATGTTGGAGAGTGGGATTGTTCCT

AGTACTGTTAACTTTAGAACAGTTTTTTATGGGTTGAATAGAGAAGGGAAACAAGGTTTA

GCACGTGTTGTGTTGCAACAAAAATCAGAACTTATTAGAAAGCGCAAGCTCATAACTTGA

>MS.gene40993.t1

ATGGCAATTTGTTACAGGAAAAGCACATTTGTAGAAACTTATAACAACATCATATTGCCT

AACAATGGACCTAAATTAAGGCCAACTGTTGATGCATTACCAATCCATCCTCCATGTTTT

AGGAGGACAATAGGGAGACCCAAGAAGGCTAGAAGAAAGACAAATGATGAGCCTACAAAC

TCTAGAAGGTTGAAGAGGGTAGATGGTACTGTCAGATGTAAAAGGTGTAATGAATTAGGA

CACAATAGAAGAACATGTAAGGGTAAAACAGCTGCAGAAAGAAAGATACCAAAGGGTGGA

AACAAGACAAATACTAGTCTAGCACAACCTGAGACTCAAGCTGCATCTATGAGACAACCT

GAGACTCAAGCTGCACCTGTTTCACAAACTGGATCTCAAACAGGAACTCAAAATGCCAGT

CATGCTAGGAGGCAAGCAGGAACTCAAGCTGCATGA

>MS.gene41016.t1

ATGGCGGTTATCACCTTACTAACGGCGGCACTCACGGCGGCGTTTCTAGCTTCAGCATAC

GCCGGCGACACAAACAAAGTATTTTCTCCTTGCACAGACACACGCGTCCAGAGATCCGAC

GGATTCACGTTGGGATTGGCGTTCGCTTCGAAGGAGAAATTCTTCTACAACAATAACAAT

AGCATTCAGTTATCTCCGTGTGATTCTAGACTCTCGCTTTCCAATTCCAATTCTCAGATC

TCTTTGTTTAGACCTAAGGTCGATGAGATCTCGTTGCTTACTGTTAATTCTTCTTCCTTC

GTCGCGGACTCGTATGGCTATATGGTTGCATTTGCTGGTCGGAAATATGCAGCAAGATCC

CCTCCTGCTTTTATTGCAAATGGCTCATATACTGTGACCAGTTTTACTCTCGTTCTTGAA

TTTAAGAAGGGCAGGCTGCAAAATTTGTTCTGGAAAAGAGATGGGTGTTCTAAATGCCCG

AAAAACTCAAAAGCTGTCTGTCTTAACGGTCAGGACTGTGCACTACCAACTTCTAGTTGC

AAGACCCATTCAGGAACTGTGGATTGCAGCTTAGGTATACAATTAGCATTCTCTGGCACA

GATAAACACCTATCAGCTCTTAACTCTTGGTATGAAGTGAAAAACCTTCGCCAGTATTCG

CTCTACGGCCTTTATTCAAATCTGAGAAGTTCACTCACTAGCCAGTATGATAAGTTTTTC

TAA

>MS.gene41015.t1

ATGGTTCCCATTTTCAGCCTCTTGCTTCTTTTAATTAATTACTTCATGAGTATTCATGGA

AGAGTGATGTCTTTAGAAGAATCCCATATAAGTTTACCCTCAGAAAATCTACAAAGTTTT

ACCCACTTACACTTTTATTTCCATGACATCCTAGATGGTGAAAAACCAACTACACTCAAA

ATTATCAATCCACCCAATGAATCATCTCATGGTCCTTTTGGAGCCACTTACATACTAGAC

AACCCTTTAACTAAAGAACCAAATTTAAGTTCAGAGCTTATTGGAAGAGCCCAAGGAACC

TATTCTTTGGCTTCTCAACAAGGTGATTTTGCATTTAAGATCGATATTAATTTTGTTTTT

ACAGCTGGAACCTACAAAGGGAGCAGCCTCACCATGCTTGGGAGGAATGTTATTATGGAT

GAAGTTAGAGAAATGCCTATTGTTGGAGGAACTGGTGCTTTCAGATTTGCAAGGGGTTAT

GCTTTGGCTAAGACTATTTGGTATAATTCTACATCTGGAAATGCTATTGAAGAGTTTGAT

ATTATAATTTTACATTTCTAA

>MS.gene41007.t1

ATGTCCATAATTCAATGTATTGGAGGATTTGTAGCCATCCTAATCTTCCTATACATCCAC

TACTGGAGACGCAATAGAGATGAATTTGCACCAATTAATTGGCCAATAATTGGTATGCTA

CCGGGGCTTTTGCGTCACCTGTCCAATTTCCATGATCATACAACCTTAATTTTGAAACAT

CATGGAGGAACATTTCGATTTGAAGGAGCTTGGTTCACAAACACAAGCTTTATCGGTACC

AGTGATCCGATGAATGTTGATCACATCGCGAGCAAGAATTTTGGCAACTACGGAAAGGGG

TCCAACTTCAAAGAAATTTTTGATTTTTTTGGAGATGAGAACAATGTTTCATTCATTTCC

AAATGGAAAACCTTCAAGAACTTTTTCCAACAAATTATGAAGAAAAAGTTGGAGAACTGC

CTACTACCATTTCTTAATGATGTATCTGAAATAGGTGCTCAGGTGGACCTGGAGGATGCT

TTGAGTAGGTTCACTTTTGATAGCATCTGCACTATTGCATTTGGATTTGATCCTAATTGC

CTTCCAAACAAGTTTAATGAGCTAAGAGAAATTGCTTATCAAAAATTTCTTCCTGTTATA

GATGAGGTGATAATTTACCGGCACTTCATACCACGTTGTCTATGGAAGCTGCAAAAATGG

CTCGACGTTGGTCAAGAAAGGAAGTTAAAGGAAGCCAAAAAAAATCTTGATAGTTTCTTG

TATGAAAGTATAACATTTTCCAAACAAGAGCAAAGCAAGTGCAGCAGCAGTGAAGAAATG

GATGACTTTGTAAAAGCGCTGATGAAGGATGGATCTGGAAAGGGGGAAATGGACGAGAAG

TATCTTAGAGACAATGTACTCTCTCTCTTTTTAGCAGGAAATGGCACAATTAGTTCAGGT

CTCAGTTGGTTTTTCTGGCTTGTTTCAATTCATCCTATTCTCGAAGACAAAATTATTCAA

GAAATCAAAGATAACTGGCCCACACAAGAGGAGAATCAGACTCCTTTGAGAGATGTGGAT

CTTGATAAGCTAGTGTACCTCCATGGAGCTCTATGTGAAGCCTTAAGGCTTTATCCTCCT

GTACCTTTTGAGCATATCTGTGCAATCAAATCCGATATACTACCTAGTGGAGAGCGTGTT

AGTCCAAATACAAGGTTACTGTACTCTTTGTACGCTATGGGAAGGATGGAACAAATATGG

GGAGAAGACTGCATGGAATTTAAGCCTGAGAGTTGGATATCAGAGAGAGGACGCATTATA

CATGTACCATCTTACAAGTTCATTGCTTTTAATACAGGACCTAGAAGTTGTATGGGTAAG

GATATCAGCTTCATTCAAATGAAGATGGTCGCAGCTGCTTTGTTACCGAATTTTCACATA

AAGGTGGTGGAAGGTCATCCTGTAACCCCAAAGCTTTCTTTTGTTCTTCACATGAAACAT

GGCTTGAAGGTTAAAGTCACTAAGAGATGCATTTGA

>MS.gene41012.t1

ATGGCATTTGCTGAAGAGCTAGAAACTCTTCATCACAAACCAGCAACTCCACTTCATCCC

CCAACCAAGTCACCGGTTCATAAACCATTAGCTAGCCCTCCTCACCACAACCACTCACCA

TCCCATGCACCTTCTCATGTTCACTCTCCTTTACATCCTCCTCACCCTGCTAAACCCCCA

ACCCATCACCATCACCACCACCACCAGCATCAACATCACTCACCATCCCCTACCCCTTCC

CATATTCACCCTCCTTTACATCCTCGTCACCCAGCAAAACCCCCAACTCATCATCATCAT

CAACATCACTCTCCTGCTCATTCTCCTATTAAGCCTCCGGTTCACACTCCTTTACATCCT

CCTCACCCTGCCAAACCTCCAACCCATCACCGCCACCAACATCACTCACCTTCCCCTTCC

CCTTCCCCTTCCCATGTTCACACTCCTTTACATCCTCGTCACCCTGCAAAACCCCCCACC

CATTATCATCTTCACTCTCCTACACATGCCCCTATTAAGCCTCCGGTTCACAAACCTTTA

CTTCCTCCTCACTCTGCAAAATCCCCAACCCACCACCATCACCATCCTCCGGCTCATGCC

CCTACTCACACACATGTTGTTTCAAAAAGCTTGATAGCTGTTGAAGGAGTTGTTTATGTC

AAATCATGCAACCATACTGGTGTTGACACCCTAAAGGGAGCTACACCGCTTCCTGGTGCC

ATTGTGAAGCTCCAATGCGACAACGCCAAATACAAGTTGGTACATAAAGCCAAGACGGAT

AAGAAAGGTTACTTTTATATTGGAGGTCCAAAGAATATTGTAGGTTATTCAACTCGCCAT

TGCAACGTTGTTTTGGTTAGTGCACCAAAAGTACTAAAACCCTCAAATCTTCATGGTGGT

CTTACTGGGGCTCTTCTTAAACCTCTGAAACGATCAATGTCTAAGGGTGCTTCTGTAAAA

CTCTACTCTGTTGGACCGTTTGCATTTGAGCCCAAATGTCATCATTAA

>MS.gene41026.t1

ATGGCTCCTTTTGCATTCTCATTCTTCTTCCTTTTCCTCTCACTCTATGCTTCATCATCA

GAAGCAGGATCTATCGGAATCAACTACGGTAGAATAGCTGATAACCTACCAACACCATCA

AAGGTAGTAGAGCTTTTGAAAGCACAAGGTTTCAGCCGTGTAAAGCTTTACGACACCGAC

GCCACCGTGTTAACCGCTTTGGCAAACTCCGGCATCAAAGTCACCGTCGCCATGCCAAAT

GAGCTTCTCTCCTCCGCTGCAGCCGATCAATCCTACACCGACACATGGATCCAATCCAAC

ATCCTTAACCACTACCCCGCCACACAAATAGAAGCCATTGCTGTCGGAAACGAGGTTTTC

GTAGACCCGAAAAACACGACCAACTACCTCGTACCCGCCATGAAAAACGTTCATGCTTCT

CTCCAAAAACAAAACCTAGATAAACAGATTTCAATCTCTTCACCTATAGCACTGTCTGCT

TTACAATCTTCTTACCCTACCTCAACCGGTTCATTCAAAACCGAACTTGTTGAACCGGTT

ATTAAACCGATGCTCGAGTTTCTACGTCAAACCGGTTCTTATTTAATGGTTAATGCTTAC

CCTTTTTTTGCTTATGCTGCTAACTCAGACACAATCTCGTTAGATTATGCTTTGTTTAAG

CAAAACCCTGGTGTTGTTGATTCTGGTAATCATTTGAAATATGATAATCTTTTCGACGCT

CAAATCGACGCCGTTAATGCTGCCATGTCTGCACTTCAATATGATGATGTTAAGATAACT

GTTTCTGAAACGGGGTGGCCTTCATTAGGTGATAGCAATGAGATTGGTGCTGGTCAAGAC

AATGCTGCTTCTTACAATGGTAATTTAGTGAAAAGGGTTTTGAATGGAAGTGGGACCCCT

TTAAGACCTAAGGACCCACTTAATGTGTTTTTATTTGCTCTTTTTAATGAGAATCAGAAA

ACGGGTCCCACTTCTGAGAAAAATTATGGTTTATTTTACCCTACTGAGAAAAAGGTTTAT

GATATTCCGTTGACGGTGTCGGGGACTAATCAATCTTCGACACCGATAGCGGAGTCTCCA

GTGGAGTCGGCTCAGGCTCCAGTGGCGGGTGTAGGGAGGAGCAAGGTGCCGGTTAGTGGT

GGTGAGGTGTCGGTTGTGGAGAATAAAGGGCAAACATGGTGTGTGGCTAATGGTGGTTAT

TCCGAGGAGAAGTTGAAACACGCGCTTGATTATGCGTGTGGAGAAGGTGGAGCTGATTGT

GCGCCGATTCAACCCGGTGCCACGTGTTACAATCCTAATACTTTAGAGGCACATGCTTCC

TTTGCTTTCAATAGTTACTATCAGAAGAAGTCACGTGGGGGTGGCACGTGTGATTTTGGT

GGTGCTGCTTATGTTGTTACACAAGCTCCAAGGTATGGAAAATGCGATTTCCCTACAGGA

TACTGA

>MS.gene41028.t1

ATGGGTCGACCAGAACCCTGTGTTCTCTTCTCTCACACCTTCGTTCATTCTCATCTCGAT

GAGTACGTCGACGAGGTTGTTTTTTCGGAACCTGTTGTAGTCAGCGCTTGTGAGGTTTTG

GAACAGAGTACAACATCGGTTGCACAAGCAGTACCACTTGTTGGGGCGACTTCACCTCCA

TCCTTTGCCATAGAGGTGTTTGTTCATTGTGAGGGAGAGACGAGGTTTAGAAGACTTTGT

CAGCCTTTTCTCTATTCACAATCTACATCCAATGTATTAGAAGTGGAGGCCGTTGTCACT

AGTCATGTAGTTGTGAGGGGCAGTTATCGCAGTTTGAGTTTGCTTATATATGGGAACACA

GCTGAGGATTTGGGTCAATTCAACATTGAATTTGATGACAATGCGTTGACTGATCTTGTT

GATTCTACTGAGGGGAAGCTTGAAGACTTGCCACTTGCATTGCATTCTACTAATTTTTCT

TTTGAAGATTCACGTTCTTTGAATGTCTTGTCTATTCCAGTTCCTGTAGCAGATATTTCT

CTTGAAGTCAAACTTTTTTTACAGTTGATGTTAAAGATTTTAGAATTATCAGAGACTGGG

GGTAAGGGACATATTGGGGATGATGGACATAAAGTTGTAAGTACTGTAGTATCGGCAATA

TCTTCCTACATTTCTGGTGACATCTGTGAATCAATCAGTGGGAGATATCAGTCAGGGAAG

AGAACTGAGAAGTTTGAAGAGTTGCACAATGTTGTTAACGGGGCAAGGAAAGAGCTCACT

GATGTCTATAGGGTTTTTCGTAAAAAGATTGGGAGTGAATCCTCTGAACGTTCATCAGAT

TATTCTGATCTGGAGACTGAAATATTGGATTCCAAAACACTGGTGGATATGTTTAACCAG

ATTAATCATTTCAGAAGGCACTCTTCATCTATAGGAGATCACTTTCTTTCTCGGAGTGAG

CATGCTCTATTAGGATTGAGCATGGCTTATTTACTGTGTTCTGGTAGGGAGAGCTGTTTT

CAGTTTGTTAATAGTGGGGGAATGCAGCAGATTGAAATGTTTTTTGCTAAGGATGTCCAG

AATTCTACTACTATTACGCTTCTGCTTCTTGGTGTTGTAGAGCGGGCTACTAGATATGCT

GTTGGATGTGAAGGCTTTTTAGGTTGGTGGCCTCGGGAAGATGAAAGCATCCCCTCTGGT

GTCAGCGAAGGTTATAGCCATTTATTGAAGTTGATACTGTCAAAACCTCGGCATGATGTT

GCCTCTCTTGCAACCTATTTGCTTCATCGCTTACGATTTTATGAAGTTGCTTCGAGATAT

GAGTCTGCAGTTCTGTCTGTGGTGGGAAACACCAGTACTTTTGGCAGGGTAACAGACGTT

ACTTTGAACATGCTCAGTTCTGCAGAAGTTCTGCTCAGAAAGCTCCTGAAATTGATAAAT

TCACGTGGTCCAATTGAAGATCCTTCTCCAGTGGCTTGTGCAAGTAGATCACTGATTACT

GGTCAAACAGATGGATTGTTGTCATATACAACAACTTCCAACTTGATCAGCTCTTCAAGT

TGTTGCTTTTCAGATTGGGATATTGATTCACATTTATTGGGACTGTTAAAGGATAGGGGA

TTTCTTTCATTATCAACCGCGCTTTTGTCATCATCTATACTGCGTGCGGAAAGGGGTCAT

GTCATGGAGATATTCATGGATGTTATATCATCTATTGAGGCTGTGATTCTTTCATTTCTT

TTTTGTCGTTCAGGCTTGATATTCCTATTACAAGATCCCGAACTTTCTAGTACTTTAATC

CGTGCCTTAAGGGGTGGTCATCACGGCAGCAAGGAAGATAGTATTCCTCTTCGATATGCT

TCCGTTTTGATAACAAAGGGTTTCTTTTGTAGTCCAGTGGAGATAGGAACAATAATTGGG

ATGCATCTAAAAATGGTTAATGTGATTGATTGTTTGCTTTCATCAAATCCACAATCGGAA

GAGTTCTTATGGGTTGTGTGGGAATTGTCTGCTCTCTCAAGGTCTGACTGTGGGCGCCAA

GCATTGTTTGCTTTCGGAAATTTTCCAGAGGCTGTTTCCGTCTTGATTGAAGCATTAAGT

TCTACCAAGGAATCTGAATCTGCTGGGAAAAACAGCGGATCTTCACCAGTAAACCTTACA

ATATTTCATTCAGTTGCTGAGATCATTGAAGCCATTGTCACTGATTCCACATCAGCATCT

TTGGGCTCTTGGATTGGACATGCTATAGAACTTCACAGGGCTCTGCATTTCTCCTCTCCA

GGTTCTAATAGAAAAGATGCTCCCTCACGGCTGTTGGAATGGATAGATGCTGGTGTAGTA

TACCACAAACATGGCGGTATTGGTCTCCTACGTTATGCGGCTCTATTGGCTTCTGGAGGA

GATGCTCAATTAACTTCAACTAGTGTCCTAGTTTCAGATTTGACTGATGTTGAAAATGTC

GTTGGAGAGTCTGGCTCTGATATTAATGTCATGGAGAATCTTGGAAAGTTCATATCTGAC

AAGTCTTTTGATGGTGTTACTCTTCGTGATTCTTCTCTATCACAGTTGACAACATCAATA

AGGATTTTGTCTTTTATCTCTGAGGACCCGGCTGTTGCTGCGTCTCTCTATGATGAAGGT

GCTGTTACAGTTATTTATGCCATTTTGGTCAACTGCAGATTTATGCTTGAGAGGTCCTCA

AACAGTTATGATCACCTTGTAGATGAGGGTACAGAGTGCAATGCTACGTCGGACTTGCTA

TTGGAACGCAATCGTGAGCTGAGCATAGTTGATCTTTTGGTTCCTTCTCTTGTGCTACTG

ATTACACTTCTGCAGAAATTACAGGAAGCAAAGGAACAACACCGAAATACAAAATTAATG

AACGCTCTTTTAAGAGTCCACAGGGAAATAAGCCCCAAGCTAGCTGCATGTGCAGCCGAG

TTATCATCTCCGTATCCTGATTATGCGATTGGTTATGGAGCTGTCTGTCATTTGATTGCG

TCTTCCCTTGCTTTTTGGCCAGTTCATGGCTGGAGTCCTGGTCTTTATCACACCCTTCTT

GCCAGCGTTCAGGGTACTTCATTGTTGACTCTAGGTCCAAAAGAAACATGCAGTTTACTC

TATCTTTTGAGTGATTTGTTTCCTGAGGAGGATGTGTGGCTTTGGATTGGTGGCATGCCT

TTGTTAACTACGCGAAGAATGTTGGCTGTTGGAACCCTGTTGGGGCCTCAAAAGGAGAAG

CACGTCAACTGGTATTTGGAATCTGGACCCCTTGGGAAGCTGGTTAGCCAGTTGGCACCA

CACCTTGACAAAATTGCTGAGATTGTACAGCACCATGCCATTTCTGCATTAGTAGTCATT

CAAGATCTGCTGCGTGTTTTTGTAATTCGCATTGCTTGCCAAAATGTTAAGTATGCATCT

ATGCTTTTACAATCAATATTGTCATCGATCGCTAGTCTTGTTTCAGATTCATCTCCATCA

GATACTGATACATACAAGGTTTTGAGACTTCTTGATTTTCTTGTTAGCCTATCTGAGCAT

CCACTTGGCAAGGGCCTATTGTTGAAACTGGGCACTCTTGAGACGCTAACAAAAGTACTT

GACAGATCTTTTGTCATTGTTGATGGAAAACCAACTCCTGATGGTAGAAGTTCTGCAAAG

TACAACTTTAATTTTTTGAGTTGGTGTCTCCCAGTATTCAAGTTTATCATGCTTCTTTTT

AATTCCGAAACTTCTCAATGTTACTCCCGAAGACATGACTTTAAATTTTTTGAAAACATG

AGTGACAAAGATTATGCCCTCATTTTACATTATCTTTTGAAGAGTTGCCAGGTCCTACCT

GTTGGCAAAGAGTTACTGGCTTGTCTTATAACTTTGAAAGAATTAGCTTCGTGTAGTGAA

GGTCAAATGGCATTTGAAGCAATTCTTTCTGGTATCCACTATAATGCTCGTGAACTTGAT

CAGAAAGATGATATGGATGTAAATAATAATGTTCCTAGTATAGCAGAATGGAGAAAATGC

CCTCCTTTACTTAACTGTTGGATGAAGTTGTTGAGATCAATTGACACAACAGAGGGTTTG

TCGCCTTATGCAATTGAAGCTGTCTATGCTTTATCTATGGGTACTCTGCAGTTTTGCATG

AATGGGAACAGTTTGATTTCTGACAGAGTTGTCGCATTGAAGTACCTCTTTGGACTTTCT

GATGATGTGACAAGATCATTTGACTTTCCTGAAGAAAATATAAACTACATACTAGATTTG

AGTACTATATTAAGTTCAAAGGCTACCGTAGATGACTGTTTGGTCACCTCTCACATGCAA

ATCCCATTATACCAGGTTTCTGAGTCAGTGAAATCATTGTCCTCAATATTGCAAAGGCCT

GTTGGTTCCATGGAGTTGGATGACGTTGTTTTACCTCAAAATGATGTTTTAGTTTTTCCA

AAGGCACTTCACATGCTTGAAAACAGCATTGAAAAGATTGACGATCATCTTTACATTGGA

GGGCTTGGTGATAAATTTCTTTGGGAATGCCCAGAAACAGTACCTGATAGATTAACACAA

ACAAGTCTTGCTGCCAAAAAGAAATTAACAGCAATAGATGGATCAGTGAGACGAGGTAGA

GGAGAAAGCTTTCAAACTGATGTCTCTTCTTTTTCACGTGGCATAGCACAGACCACTGTT

TCATCTGGTCCTACGCGTAGGGATTCTTTCCGACAGCGCAAGCCGAATACCAGCAGACCC

CCATCTATGCATGTGGATGACTATGTGGCGAGAGAGAGAAATGATGGGGTTACTAATGTA

ATAGCAGTGCCAAGAACAGGATCTACTGGTGGGAGACCCCCATCAATCCATGTGGATGAA

TTTATGGCGAGACAAAGGGAACGCCAGAACCCTTCAGCAACAGTAGTGGGAGAAGCCGTG

GGACATCTAAAAAATGCTTCTCCGGTGAAAGCAACAGATGTAGAGAAGTCAAATAAATCT

AATCAATTGAAAACGGACCTTGATGACGATCTTCAAGGAATTGATATAGTTTTTGATGGA

GAGGAGTCTGACTCTGATGACAAATTGCCATTCCTTCAACCGGATGATAACTTGCAGCAG

CCTGCTCCAGTTATTGCTGACCAAAGTTCTCCCCACTCAATTGTTGAAGAGACAGAAAGT

GATGCTGTTTCTCGTATGGGTACTCCATTGGGATCTAACATTGATGACAATGCCCAGAGT

GAATTTTCTTCAAAGGTTTCTGGTTCACGACCTGATATGGCATTAACTCGTGAATCAAGT

GTTTCTTCGGATAGGAAGTATGGCGAGCAGGCTGATGACACAAAGAATGTTCTTCAAGCT

AAGATTGCTGGTGGATACGATTCTGCAACAGCAAATAGTTCATTTCCAGTGTCTCTCTAC

AACAATCCATCAACATCTACGCAGTTACCAGTTGATTCAAGGACTGCTTCTCAGAATTTC

TTTTTAAAGAACAGTCCGCAACATGGTGGTATTGCTACAGGTTCCCAAGGAATGTATGAC

CCGAGATTTTTTCAGAACCAACCTCCTCCTTTACCGCCCATGCCACCCCCGTCAACAGTC

TCACCTGTAATATCACATGGTTCCGATTCAGTGCATGGTCAGATAACCTCATTCGTTAAT

TCTCCAGCAGGTGCACGCCGTCCAGTTTCATTCCAAGGACAATCAGATTATTCATCTCCA

TTCAACAATGGTCCAATTGCACCATCATTTTCATCTTCTGTTCCTATGCCAGATTCCAAA

TATTCTAGACATTCTATATCTTCTCCCAGTGGACCTAGTAGACATGCTCCTCCACTGCCG

CCTACACCACCCCCTTATGCATCTAGTCCATATAATTTATCATCATCTATCAATACTTCT

GTCTCCCAACCTGCTCCATATAATCAGACAGGTATTGGGAATACCGAACTTTCTCAGGCC

TCCATTGCTCATTCAGGAGCAAGATTGTCAGCTTATCCACTAAATCCCTCGATGATGCCC

CCGGGGTACAGTAGGCCGACATCTGTGCCAATGACTGTCTTCAGTAACCCCTCAAATCAG

CAACAGAATGAGAACCAGCCAAGCTTCTTGCATAGTATCTCTGTCCCCCAAGCCTCCTTT

CCGTCAATGCATACAGTTACTCAGTTGCAGCCGCTGCAGCCCCCCCAGCAGCTTCCACGC

CCTCCACAACCACCTCAGGTACCTAGGCCACCTGTTCAAGCCTTACAACAGTTAGAGCAA

GGGATGACCATGCAGAGCAATGTTCAAGTGCATGAGTTACAGATGCTGCAACAGTCTCAG

GTTTCTTCAATGCAGACATATTATCAAACTCAGCAGCAACAGCTTTCACATGAGCAGCAA

CAGCAGCAAGTTCATCATGCTCAACATATGGGGAATGCTCAATCACAGGAGCATGCAGAT

GCTGGAATGTCATTAAACGAGTATTTCAAGTCTCCAGAAGCCATTCAGTCTTTGTTGAGT

GACCGGGAAAAACTTTGCCAGCTGTTGGAGCAGCATCCAAAATTAATGCAGATGCTTCAG

GAAAGGTTAGGCCAGCTGTAG

>MS.gene40996.t1

ATGCACAATTCAACTTTTACCAAAGTACACTTGCCTGCTATCCCTATGAACATTTTTGAG

TTCAAGCCATTCAATGAAATTCTCTCTTCAACTGTCGAGGAAGTGTCTACGGATGTTATT

GGTCATGTAATTGAAAGAGGTGATATAAGGGAAACTGAAAAGGATGGAAGGAAAAGCAGG

GTTATTGATCTCACTTTAGAGGATCTTGAAAACAACCACTTGCGTTGCTCTCTTTGGGGT

GCTATGGGAGTTGCTAATGCCTTTAATGGGACTAAGCTGATACTTAATGGCGATTTGCCT

GAAGTCGTTGCGTACATGACAGGATCTGTCAAGGTTCGGTGCTTGCCACTATATGTGAGA

TCGAAACTGGGGTTGATTGGTACTTTCGATCATGCACCCAGTGTGCTTCATTGGCCACCG

TTGACAATCCGTTGTCGCAAGTGTAAAACTTGCAAGGGTGCAGTCCCAAGGTTCAAGCTG

CATGTCATCGTGATGGACGACACAGGGTCCACTACTTTTGTTCTTTTTGATCGCAAGGTG

ACCCAGTTTATTGGGCATCTCTCTGCTCCTCCTAAACCCTCTTCCAAATCCATTGATGAT

GAATACGATACTCCGATTCAACCTCGAAACCTTGTCTATCAAGACGTTGATGATGATGAT

GATACTATTCCTCAGTTTTCTGCTATCACTGACTTCGATTCTCCAATCGGTCAAAATTCT

CCTGAAAATCTTCAACCAGATAAAGTAGAAGAAGCTCCTCTTCCTAAGGTTCCACTGTTT

TTCGAAGATTCTTCTCTTCGTAAACAAAAATCTCCTGAGAATCGCCAACAGCTTAATGTA

GAAGAATCTCCTCTTCCTCGCGAGGTTCAACAGGATTTGGATAATTGTTCTCGTATTACT

TGCATTAATGGATTATTAGGTCAAAATTCTAATGAGAATCACCAACCAGTTATGGTTCAA

AAAGCTCCTCTTCCTAACGAGGTTCTAGAGGATATTGAGAATTACTCTTCTAGTCAAAAT

CATGTGGAGAATTACCTACCAGTTGAGGTAGAAGAAGCTCCACTTCCATTTGAGGTTCCT

CGGGGTTCAGATAATTGTTCTCGTGGTCAAAATTATTCGAAGATTCGCCAACCAGTTAAG

GCAGAAGAAGCTCTTGTTCCAAAGGAGGTTCCACGGACTTTGGATGAGTGTTCTCTTAAA

GAGGAAAAGAAGACTAAGGTTAAGGTGCAAGGGCGGCGCCGTCTTTGCAAGGCTGCGGAT

AAAGAGGCAAGCAAAAGTGTGGCAGATGACGAGCCTACGTTTGATGGTGATCTTGTGGAT

TTTGATTCGCCTATTACGGTGCGGAAAAATGTGATTGAGATTGAGGAGAGTAGGGTGACA

AAGCCTGTTGAGCGTTTTGTGCGAGGGAAAGAAATTTTTGAAGATGAGGGTTTGGAGTTT

GGGAGTGCTGGATCTTCATTTTCACCAAAACAAGATCCCCATGACATCTCATCAAAGGAT

ACTAAGAATGATAGTGGTGGTCTTGAGTATGAATCAGATGATTCAGTTCAGGTGTTGGAT

CACTTTGAGCCTGAGAATGATAATGGTTCTATAACATTGAATGACCCACGATCTACATAT

AAGTTGCAATCTAAGATTGCAAAAATGTTGTATCCACATCAGCGGGAAGGATTGAAGTGG

CTCTGGTCATTGCATGTTCGGGGAAAAGGTGGAATCTTGGGAGATGATATGGGTTTGGGA

AAAACAATGCAGATTTGTGGCTTCCTAGCTGGACTGTTTCATTCCCGTTTAATTAGAAGG

GACAAAGGTGTTCTTCTCACCACATATGATATTGTCCGCAACAATACCAAGTCCTTAAAG

GGGCATAGATATTTTGATGATGAGGACAATGAAGATGGCCCTACATGGGACTACATGATA

CTTGATGAGGACAGTGTGAACTTTGACAATCACTGGCTTCGTAGAAACCCACCTTATAGA

ACCCAAGCACTCAGAGAGCTAAAAGTTTGCTCGAGATACCCAGCGCTCATCGCATTATCA

TTCACACCACTGCAAAATAATCTCAAGGAATTGTGGGCACTGTTCAATTTCTGCTGCCCT

GACTTGCTTGGTGACAAAAAATGGTTCAAAGATAAATATGAAACACCTACACTTAAAGGA

AATGATAAAAATGCTTCGGCCAGAGAGAAATGTATTGGTTCGTCTGTAGCAAAGGAGCTA

AGGGATCATATTCAGCCTTACTTTTTGAGACGTTTAAAGAGTGAGGTTTTCAATCAAGAT

ACGGAAAAAACAACTGCAAAACTTTCTCAGAAACGAGAAATCATTGTGTGGCTTAGATTG

ACCAATGTTCAGCGGCATCTTTATGAAGCATTTTTGAAGAGTGAGATTGTTCTCTCAGCA

TTTGATGGTTCACCACTGGCTGCCCTTACGATTTTGAAGAAAATATGTGATCATCCGCTT

CTGTTGACTAAGCGGGCTGCGGAGGATGTATTGGATGGGTTGGAATCAATGCTAAAGCCA

GAGGAGGTTAATGTTGCGGAAAAATTAGCGATGCATATAGCAGATGTTGCAGAAACATCT

GAAGACAAGCATGATGTATCATGCAAAATAGTTTTCATCATGTCATTATTGGATAATTTG

ATCCCTGAAGGGCATAGGGTGCTTATCTTTTCTCAGACTCGCAAGATGCTTAATTTAATT

CAGGATTTCCAAGATGGTATTGGAGCTCCAATATTTCTGTTGACATCTCAGGTTGGTGGT

TTAGGACTGACACTTACAAGAGCAAATCGTGTGATTGTTGTTGATCCTGCTTGGAACCCT

AGTACTGATAATCAAAGTGTTGACCGAGCATATCGTATTGGTCAAAAGAAAGATGTTATT

GTGTATAGACTGATGACATCCGGAACTGTTGAAGAAAAGATCTACAGGAAGCAAGTATAT

AAAGGAGGATTATTCAAAACAGTTTCTGAACAGAAAGAACAAACTCGGTACTTCTCCCAG

AAGGATCTCAAGGAGCTTCTCAGCCTCCCAAAAGACGGATTTGACGTGTCTGTTACCCAA

CAACAATTGGATCAGACACATGATTCTCAACATATAGTGGATGCCTCTTTCCACGCACAT

TTAGAGTTCCTGAAAAGTCAAGGCATAGCAGGAATTAGTCACCACAGTTTACTGTTTTCA

AAGACAGAGCCAGTTCAGGAGGCTCCTGCGTATGAAGTAGAAAATAACCACTGGAAACCT

AATCCTAATGCTAGATATACTGGAACTTCATCATCATCTTTCACATGA

>MS.gene41025.t1

ATGGAGTTCAGAACTCGTGATTACCAAACTGAGAAGGAATCTCACGCGCTTCCTCGTCTA

CGCGCCGATGCTCATCCCCTCTCTCCTCCACCTACATCTCTACTCCTCCCACAGGTTAAC

GTTGTTGTCGATGATGGGAATGCTGACTTCTTTGATCCACTAAGAGGAGGAATTAATAAT

GATGCAAAGGCTAGCCCTCCAGAACCTGTCAACTTGAATGAAGTTGCTAGTGATCCACCA

ATTAAGGAGTGGACCTCTTTCAGGAGACTACTAATGCAGAGGTTTCCTGTCTCAAAAATG

GTTTCACTTTCTTCAATGCCCGATGTTTTAATGAGAAGTGGAAAATCACATGACAAATCT

TCAACAAGTATGCACCTGGAGGAACTAGATGATCCACAAAAATTTGCAGATGAGGGTGTC

AAGGTGATTACTTGGCAGGATTATGTTTCTCGACTACACGAGCTCAAAGATGAAATTACC

CGGTCTTGGCTTGCTGATGACCGTGTAACATCCTTAAAGTTATCTATAAAGGTTGCTAAG

CTTTTGGTGGATACTTCAGTATCTGAGTTTTATCCCACTCTATTTGTCCTTGTCACAGAT

ATCATGGACATGATTGGGGACCTGGTTTGGAAACGCATAAAGCAGAAAGCAGAATTTACT

GAAGATGGAACTTTACTCTGCAACTTGGCAGAAAACTTTGAAGCAAGCAATATTTGTGCC

GATGCCAAAGAAACTTGCTATAACTGGTTCAGCAAAATTGGCGCTGTGCAAGATCTTCTT

CCACGCATTTACTTGGAGATGGCATTATTGCCTTGCTGGCGTTTTCTGCTCGACCAGCCT

ACAGACAGTCTCCAACGCTTGGTGATGATGACAAGAGGATTAGGAGATCCAGTGGCATCT

GCATATTGCCGTCTATATATGGCTCATTGTGCTCAGAAGCTGCCATCACATGACATAGGT

TATCTTGTTACATGTGTTAATGATCTAAGAGTTATTTTGACGCAAACCTTAGCTGCCAAT

GAAAGTACTCTTGGAAATTTCAAAAATAATAAAAAATTGCAAATCAGCCTGATGGAACCA

ACCATTGAGTATATTATGAAGTGTATATTTAGCGGGTTATCTCAGAGACGAGTCAATGAA

GTTCTATCAGAGCTTGGATTGTTGAAGAATCAACAGAATTTTGAGACTGTTTCATGTGTT

TCGGTTGTTCTTCATCATTTACTGAAAGAACTCCCTATTGAAGTAGTCATTTCAAATGTT

GTGAATATCCTTCATCTCATTGAATTTAACAAGGATACTTCCTTTCATCAGCACCTGAAT

TACAGATTGCTTGGGTTCAGGCTGTATGAAGGGAAATGCCCGGTTAACATTGTCAATGCT

GTGTTAGATAAAGTTATGCAGATTATTGTCCCGTACGAGAGTCTTGATGAGTACCTGAGT

GTCGTAGATGCTTATGTTGATCTTATACTTCAGAATCACATGAATAATCATTTGGACATC

ATTTTGAGAGGCATTTCAGAGCGTGCTTCGAACGGAGGAGTTACAGTAGATGAAATGTCA

AGCTTGCAATCTCTCTTGGTGAAGCTTCTGTCTCATTTTGAGTGTCTGGAAGATGTGTTT

TGTCTGAATCATTTTCCTGAAATCTTAGATGTAATGCATGGGAAATCACAAGATGTTGTC

TTTTTGTACATCCTTAATATGGCGACGAGGAGTGGTCATATTAGGGATCCGACAAGCATA

CACTTGCTTTCTGAAATTTCTCAGGCTCTACATGATAATATGGAATTTACGAATAAGGAT

GATGATGGGCAAGTGGCACGTTCAGTATCTCGCTTTGTGCACATGGTAGATTATGGAACG

GAGATGGAATCCCATTTAGCATTTCTGGTTGATTGCCGAGCAAATTTTGGTAGATTCAAC

GAGCTTAAGGAAACTCTCGTTCACTCCAGCAATTCTTTAGCAATTCAATCTTTGAAATGT

ACTAAGAAAGATCTGAGTTTTTTCAAGTCCTGTGTCACATTTAGCGAAGTCACAATACCT

TCTATCTCTGGTCAGAGACAATTTGATCTTTTTCTAGAGACTGCAGAGGTTGCATTCTTA

GGGGGCTTGGTTTCTCATGTAGATGGATTGATTGATTCAGGAATTGGCTGTTTGCATGGC

TTTCGAACTCCAACTGATGTTGAAGGGCTAGTTTCATCTATTAGAAAGCTGTGTAGCTTC

TTAATTATGGTCCCAGGTAATACCAGTGTACCAGTTACCTACTTCCCTAATAACTTATAT

ACATTGATCAGCTCCCAATCATGGTTTGACCCAAAAATGAGGACACGGATTTTTTCTGCT

ATCTTATTATTATTAACAACTTTATCACAAAAGACTATGCCATATCATGCAAATACACAG

ATACCAGGCAATGACATGTTATACTATGGGGATTTATCTTACAAGCAAGAACTTGTTTCT

TTGTCTAAGGTTGTTCTTGAGAACCTACTTTGTGCCATTCAGGAAGAACCTTCTCAGGCT

GCTCGAGGAAGTATGGCACTTGAAGCTTGCAATTGCATTTTATCATCTTTTCTGCTAAGC

AATGAATTATCCTCTACTTGCCTTACACTGATTGGAACAGCCAAGTCATGTTTGAGTGCC

CAAGACAGATGCCTCCAGTCAACCATTCAACTTGTAAACAAGCAATTGCCAGATTTTGCA

GGGGCCATTTCAACTTCTGTGTGA

>MS.gene40998.t1

ATGGTTCAGCTCAGAAAGATGATAGAATTCTCATTGCTTCTAATGAGGTTCAGGAAGCTG

ATGCAACTACTGAAACATCACTTCAGAAAGTTGACACAACTGCTGAAGATGGGAAAGTTG

ATGAAGTTCCTGCATAATAACTTCAGAAGGTTGATGAAATTGAAGAAGCTTCTGGAGAAA

TACCTCAGAAAGCTAACAAAACAAGAAGCTTTTAGTGGAGAAAGATCCTTTAAATCTTCT

ACTGCTGAAACAATTTCAAAATCTGAAGTTGATGTTACAAACATCATGAGTGAACTCCAG

TTTGATGAACAAGAAATTGTTCTAAAATCTGATATGGGAAATGACCCTCTGTCATTAAAA

GATCAAGATTTGCATGATGTTGCTAAATTTCTAGCCACACACATGCTTAGTGAAAACACT

ACCACCAAGGAATCTGATGAAGATGAGCTAGCTAGATATCAAGCCGGTTCTGATCTGAAT

GTGGAGGAATTCAGCTCAGACTTCAATGCAAGTGTCTTTCAAGATTCTCAAATGGAAAAT

GATGGAACAATTCCTGCAACTCAAGTTCAGAATGTTGAAGAAAACATTCCATTTGTTGAC

CATGTGTCTACAGATCCTGAAACATATGGATATGTTAAAAGAGACACAAATTAG

>MS.gene41008.t1

ATGGTTGTTAAGATGATGAAATGGCGTCCATGGCCCCCACCAATTTCCCGTAAATTCGAA

GTCAAACTCCTCATCAAAACTCTCTCCGGTGGAATCGATCTCTCGCCGGAGAATACATTC

GCCGTTGAAATTCGTTGGAAAGGTCCTAAACTTGCTCTTAGTTCGCTTCGTCGGAACGCG

GTTGTGAGAAATTTTACTGGTGAGGCTCATCCTAAGGGAGATGAAAACGATGTCGTTTTG

TGGGATGAAGAGTTTTGTAGCTTTGTTAATCTTTCTGCTAATAAGGAGAATGGTTTTCAT

CCTTGGGAGATCGCTTTCACTGTTTTCAACGGTTTGAATCAGAGGCCAAAGAACAAGATT

CCTGTGGTTGGAACAGGTTCATTGAATCTAGCTGAGTATGCATCTGTTGTTGATCAGAAG

GATTTTGATTTAAGCATTCCACTTACAATTCCTGGTGGTTCTGCTGTTGACCCTTCTCTT

TCACTCACTATATCAATCAGCTTAGTCGAGCTAAGAGTGGCTCAAGAGAATAGTGAGCTT

GTTCATAAATCAATAGTGCCTGTTGCATCACCCTTGAACCAATCAGGAGAGTCAGAGAAA

GACGAGGTTTCCACTATCAAAGCTGGTCTTAGGAAAGTTAAGATTTTGACTGAGTTTGTA

TCAACAAGGAAATCAAGGAAACCGAGCCGTGAGGAAGAGGGAAGTGAGGGAAATTACTCT

GGCAGGAGTGAGGATGGCGAGTACAATTACCCTTTTGACTCTGACTCTCTTGATGATTTT

GAGGAAGGGGATTCTCATTCAGAAGAGGTAAAGGAGGATTCCAGTGTGCGGAAATCGTTC

AGCTACGGGAAACTGGCCTTTGCAAATGCTGGTGGATCATTCTATTCGAGCATGAGAGTG

AAGGGTGATGACGAGGATTGGGTTTACTACAGTAATCACAAATCAGACGTTGGAAGTTTG

CCAAAAGAGGATTCTATTGTTTCTTCCTCTGAGCCTTATGTAACGCAAAGTTCAAGACGC

AGCTTATTGCCTTGGAGGAAGAGAAAGTTGAGTTTCAGATCTCCTAAATCTAAAGGAGAG

CCCTTGTTGAAGAAGGCATATGGAGAAGAAGGTGGTGATGATATTGATTTTGATCGCCGG

CAGCTTAGCTCTGATGAATCTATTTCATTTGGGTCACATAAAGCTGAGGACGATTCAGGT

GCAAATCGAACATCGGTATCTGAGTTTGGGGACGACAACTTTGCAGTGGGCAGTTGGGAG

CAGAAAGAAGTAATGAGCCGTGATTGCCACATGAAACTTCAGACACAAGTCTTCTTTGCC

TCAATTGATCAGCGCAGTGAACGTGCAGCAGGGGAGAGTGCTTGCACAGCTCTTGTTGCT

GTAATTGCTGATTGGTTCCAAAACAACCGCGACCTAATGCCCATAAAGTCCCAGTTTGAT

AGCTTAATTCGAGATGGCTCATTAGAGTGGAGAAACTTATGTGAAAACCAGACATATAGG

GAGCGATTCCCCGACAAACATTTTGATCTAGAAACAGTTGTTCAGGCCAAAATTCGCCCC

CTTTCCGTTGTTCCTGAAAAGTCCTTTATCGGTTTCTTTCATCCAGAAGGAATGGATGAG

GGAAGGTTCGATTTTCTGCATGGCGCCATGTCTTTCGATAACATTTGGGATGAGATTAGT

AATGCCGGAAAGGACTCCACAAGTAACGATGAACCTCGGATTTTTATTATCAGTTGGAAT

GACCATTTCTTCATCCTTAAAGTTGAAGCCGATTCTTACTGCATCATTGACACTCTTGGA

GAGAGGCTTTACGAAGGATGCAATCAAGCATATATCTTGAAATTCGATGGCAACACGGTA

ATTTACAAAATGCCAGATGCCACTCAATCGTCAGACGAAAACACAACCGGTGAACAACAA

ACCGTTGCAGATGTATTAGAGCACAATGACAGGCAGGTTCAGCAAATCAATGACAAGGAG

TTGGAGTCTGGTGCTGAGACAGGAGATCAGTCGAAAACTGAGCGTGAGGAAGACGAGGTT

TTGTGCAGAGGGAAAGAAGCATGCAAAGAATACATCAAAAGTTTCTTAGCAGCAATACCT

ATTAGAGAATTACAAGCAGATGTGAAGAAAGGGTTAATATCATCAACTCCACTTCATCAT

AGACTACAAATTGAGTTTCACTATACTCAATTGTTGCAGTCTTGTGATGTTGTTCCTGTG

GCTGAAGAAGCATCTGTCACAGTAACTGAGGTTAACACATAA

>MS.gene41011.t1

ATGGCATTTGCTGAAGAGCTAGAAACTCTTCATCACAAACCACCAACTCCACTTCATCCC

CCAACCAAGTCAACGGTTCATAAACCATTAGCTAGCCCTCCTCACCACAACCACTCACCA

TCCCATGCACCTTCTCATGTTCACTCTCCTTTACATCCTCCTCACCCTGCTAAACCCCCA

ACCCATCACCATCACCACCACCACCAACATCAACATAACTCACCATCCCCTACCCCTTCC

CATATTCACCCTCCTTTACATCCTCGTCACCCAGCAAAACCCCCAACTCATCATCATCAT

CAACATCACTCTCCTGCTCATTCCCCTATTAAGCCTCCGGTTCACACTCCTTTACATCCT

CCTCACCCTGCCAAACCTCCAACCCATCACCGCCACCAACATCACTCACCTTCCCCTTCC

CCTTCCCCTTCCCATGTTCACACTCCTTTACATCCTCGTCACCCTGCAAAACCCCCCACC

CATTATCATCTTCACTCTCCTACTCATGCCCCTATTAAGCCTCCGGTTCACAAACCTTTA

CTTCCTCCTCACTCTGCAAAATCCCCAACCCACCACCATCACCATCCTCCGGCTCATGCC

CCTACTCACACACATGTTGTTTCAAAAAGCTTGATAGCTGTTGAAGGAGTTGTTTATGTC

AAATCATGCAACCATACTAGTGTTGACACCCTAAAGGGAGCTACACCGCTTCCTGGTGCC

ATTGTGAAGCTCCAATGCGACAACGCCAAATACAAGTTGGTACGTAAAGCCAAGACGGAT

AAGAAAGGTTACTTTTATATTGGAGGTCCAAAGAATATTGTAGGTTATTCAACTCGCCAT

TGCAACGTTGTTTTGGTTAGTGCACCAAAAGTACTAAAACCCTCAAATCTTCATGGTGGT

CTTACTGGGGCTCTTCTTAAACCTCTGAAACGATCAATGTCTAAGGGTGCTTCTGTAAAA

CTCTACTCTGTTGGACCGTTTGCATTTGAGCCCAAATGTCATCATTAA

>MS.gene41014.t1

ATGAACAACTCAACTGATTCATCTGGCTTCTTTGGCTCCAATGACATAAGTGGATTTGGT

TATGGCATAGGAATTTCCATTGGTATTCTTTTGCTCATTACAACAATCACACTCACCTCC

TACTTTTGCACAAGATCACAAGTTCCAAATCCTCCTAGGAGAAGAAACAACAATAACACA

TCTGAATTTCTTGAACCTCAACATTCAATACTTGATTTTGGTTTAGATGAAGAAACAATA

ATGAGTTATCCAAAGATGTTATATTCTGAAGTGAAGCTTAACAAATATGATTCCACATCA

ACATGTTGTTCTATATGTTTAGGTGATTACAAAGGAAGTGACATGCTTAAGATGTTACCT

GATTGTAAACATATGTTTCACCTTAAATGTATAGAACCTTGGTTAAGGATACATCCTTCT

TGTCCTCTTTGTAGAACTTCTCCAATTCCAACACCTCTTTCAACACCTTTGGCTGAAGTT

GTTCCATTAGCAACAAGAAGAGATTCATAG

>MS.gene40997.t1

ATGCATTTGAAGCTAGGTCAAATTTCAACCATAGTAATTTCATCACCAGATATAGCACAA

GAAGTGCTTCAAACCCATGATCTCTCATTCTCAGATAGAACAATTCCTCAAGCAGTAGCA

GTTCTTGATCATGAACATTTTAGCTTACCCTTTATGCCAGTTTCAGATCTTTGGAGAGAC

CTAAAAAAATTATGCAAAAACCATTTGTTTTCTAGCAAAACACTTGATGCTAGCAATGAA

CTTAGGTGTAAGAAGCTTCAAGAGCTTTTAAGTGATATTGATAGAAGCAGCTTAACTGGT

GAAGCAGTAGATGTTGGAAGGGCTGCTTTTAAGACTTCATTGAACTTTTTGTCAAACACT

TTTTTCTCTATGGATTTTGTTAACTCCACAGGTGAAACTGATGAGTATAAAGATATTGTT

GAAAATCTTGTGAGGGCAATAGGAACACCAAACATGGTTGATTTTTTCCCAGTTTTGAAG

ATGTTTGATCCACAAGGTATCAAGGCAATATCAGCTACTTATGTTGAAAAGATGTTGCAA

ATCATTGATTCCTTTATCACCAAAAGATTGAAGTTTAGAGAAGCTGAAAATTATGTCACC

AATGATGACATGTTAGATACTTTGCTCAACATCTCTCAAGAGAATGCCCAAAAGATGGAC

AACACAAAGATTAAACATTTGTTTCTCGATTTATTTGTTGCTGGGACTGATACAACTTCC

TACACAATAGAACGAGCAATGACAGAACTAATCCACAACCCACATGCAATGTCAAAAGCC

AAAGAAGAACTTGAGCAAATAATTGGTATAGGAAATCCAGTTGATGAATCAGACATTACT

AGACTCCCATATTTGCAAGCAATAGTAAAAGAAACATTACGTTTGCATCCATCAGCTCCA

CTTTTGCTACCAAGAAAAGCCAAGATAGATGTCAAAATTCGTGGATACATTGTTCCTAAA

GGTGCACAAGTCCTAATTAATGAATGGGCCATGGGAAGAAACCCTAATATTTGGGATAAT

CCAAATTTATATTCACCTGAGAGGTTTTTGGGTTCAGAAATTAATTTCAAGGGACAAAAT

TTTCAATTGACACCATTTGGTAGTGGGAGAAGAATGTGTCCTGGAATGCCACTTGCTATA

AGAATGTTACATACAATGTTAGGATCATTGATTAATTGTTTTGATTGGAAGCTACAAAAT

GGTGATAGAGATATTGATCAACCCTTGCGAGCTATTCCTTTTAGAGTAAATAAGGTGTAA

>MS.gene41021.t1

ATGAATGATTTATTCTCCGGCTCTTTCTCCCGGTTCCGCAACTCGGATCAAGTTTCGCCG

GACAACCATCACCATGTGATCGAAATGTCATCGCCGAACACCGCACAAACCGGTGTTCAT

CTTGACAAATTCTTTGAAGAAGTCGAAGGTGTTAAAGAGGAGTTGAAGGAGCTAGATCGT

CTCTACGAAAGCCTTAGAGTCTCCCATGAGAGGAGCAAGACCCTTCACAGCGCGAAAGCT

GTGAAGGATATTCGCTCCAAAATGGATGCTGACGTGGCTTTAGCTCTTAAGAAAGCTAAA

CTTGTTAAACTCCGGCTCGAGGCACTTGACCGGTCCAATGAGGCTAGTAGGAGTTTGCCT

GGTGCTGGACCGGGTTCGTCCTCGGACCGGACTAGAACATCTGTGGTGAGTGGTTTGAGG

AAGAAGCTTAAGGATTCCATGGATAGCTTTAACAACCTTAGACAACAAATATCCTCTGAA

TATAGAGAAACTGTACAACGTAGATACTATACTGTCACCGGAGAGAATCCTGATGACAAA

ACCGTTGACCTCCTCATTTCCACTGGTGAAAGTGAAATATTTTTACAAAAAGCAATCCAA

CAACAAGGTAGAGCAAACATCATGGACACAATCCAAGAAATTCAAGAGAGGCATGACACA

GTGAAAGAGATAGAGAGAAATCTCATGGAGCTTCACCAAGTGTTTATGGACATGTCTGTT

TTGGTTCAATCACAAGGTGATCAATTGGATAATATTGAAAGCCACGTGGCACGTGCTAAT

TCGAAGAAAACCGCCGAGAATGATCATTCCAGAGGCCCACCAAGAGTTGTGGGAGAAAGG

AGGTTCAGTGGTGACCGTAATGGCGGATGGCCAAGAGACATAGCTTTGGGGGACCTGGTG

GTAGACCTAGAAAGTGGTTTTCATGCACTGTGA

>MS.gene41031.t1

ATGAAGGATCCAGTTACTGTCTCAACAGGCATAACCTACGACAGAGAAAGCATCGAAAAA

TGGCTCTTTTCATCGGAAAACAAAACATGTCCTGTCACAAAACAACAACTCACACATGAT

GATGCAAATGATCTCATTATTCTCACACCAAACCACACTCTTCGAAGGCTCATCCAAGCT

TGGTGCACCATGAATTCTTCTTATGGAATCGAAAGGATTCCAACTCCAAAACCTCCAACT

ACAAAAACCCTTATCGAAAAACTCCTCAAAGAAGCTTCTGATTCTTCAGATTCACCTCAT

TTACTTATCCAAACTCTAAAAAAGCTCAAAATAATTGCATCCGAGAGTGAGTCAAATCGA

CGGTGTATTGAATCGGCCGGTGCAGTCGAGTTCTTAGCATCAATAGTTACAAAAAACAAC

ACAAGTTGTTCATCTTCATGTTCAACAACGGAATTAATTGAAGCTAGCTTTGATGATGAT

GATGTTGAAGGTTTTTCTTTTGACTTCAAAATAGGTGCAGAAGATGAAGCTATAAACATC

CTTTACAATCTTCAATTATCAGAACAAGGTTTGAAAACTCTCCTAAACTTCAAAAATGGA

GAATTTTTAGACTCTTTAATGAGATTATTGCAAAAAGGTAACTATGATTCAAGAACATAC

GCAATCTTTCTGTTGAAATCTATGTCAAAAGTAGCTGATCCATCAAAACTAGCAAATCTA

AAGACCGAGTTTTTCGTCGAGTTAGTTCAACTTCTCAAAGACCAGGTATCAAAGAAAGCA

TCAAAAGCAACACTACAAACGTTAATTCAACTCGTTGAATTAGGAAGAAATAGAGTAAAA

GCAATTGAATCAGGTTGTGTTTCTGCTTTGATAGAACTTCTTCTTGATTGCAAAGAAAGA

AAGCCATGTGAGATGATTTTGGTGCTTTTGGAGATGTTATGTCAATGTGCTGATGGAAGA

TTTGAACTTCTAAGCCATGGATGTGGTTTAGCTATTGTTTCAAAGAAGATTCTTAGGGTT

TCAACAATGGCTAATGATAGAGCTGTGAGGATTCTTCTATCTGTTTCAAGATTTTCTGCA

ACACATTTTGTTATTCAAGAAATGTTACGAATTGGTGTTGTTGCAAAGCTTTGTTTGGTT

CTTCAAGTTGATAGTGGAAATAAGGCAAAGGAGAAAGCAAGGGAGATTCTTAAATTGCAT

TCAAAGTCTTGGATGAATTCTCATTGCATACCTTTTAATTTACTTGCTTCATACCCAACA

AGTTGGTAA

>MS.gene41018.t1

ATGGCTATGCAACCTGTTTATCTTAAAGAACATGAAGGAAATGTCCACAATTCTGTTGGA

CAGTTTTCATCTGTGACTTCAGCTCCATGGTGGAGTAATGCCTATGGATCTCAACCTGTT

TATGGCGGAGAGTCTTGTGGCCAAATGAAACCTTTTTCACTAGAGCTTTCCAACTACATA

GACCAAATTGCTCCGAGTAAGAACTTAGTTCGAGGAGTTGAACAATTGTTTGATAAAGGA

CATACAAACCAATTCACCATTTTTCCAGATGATTGTAAGATGTCAGTTGATACACCGAAT

CATCAAGCAACCTTATCACTGCAATCATCATTTGCTGCTGAGCCACTTAACCGTTTTGAG

CTAGGTTTTAACCAGCCTATGATCTGTGCAAAATATCCTTATATGGATCAATTTTACGGG

CTCTTCTCAACTTATGGACCTCAAATCTCGGGACGTGTTATGCTTCCACTTAGCATGACA

TCTGATGACGGACCAACATACGTGAATGCTAAGCAATACCATGGTATCATCAGACGCAGA

CATTCTCGTGCCAAAGCTGTGCTTCAGAATAAATTGATAAAGCGTAACAAGCCATATATG

CACGAATCGCGTCATCTACATGCAATGCGTAGACCAAGAGGATGCGGTGGTCGTTTCTTG

AACACAAAAGTTTCTGCTAATGGAAACGGTAAAGGCGGGAGTGAAGAGAACATAAACACT

GGTGGCCTACAGCTGCAGTCCAGTGGTTCTCAGAGTTCCGAAGTCTTACAATCTGAGGTT

GGAACTTTAAATTCGTCAAAGGAGACAAACGGAGGCAGTCCAAACGTCTCGGGGTCAGAG

GTGACTAGCATGTATTCACAGGGAGGTCTTGATAGCTTTACTGTCAATCATATCGGATCT

ACTGTCCACTCTTTGGGAGACATGATCGATACTGGACACGGTATTGTCATGCCAACAAAA

TGGTTTGCAGCAGCTGGTAGACAGCTGCTGGAACCATAA

>MS.gene40987.t1

ATGAACAAGCTCAAGAAAATGGCACTTAAGAATCTATCTGAAGAAGAAATTGCTGGATTA

AAAGAAATGAACAAGATGATCGACACAGACAATAGTGGTCATATTACTTTTGAAGAACTC

AAGGTTGGACTGAAAACATTTGGCGCCAATCTAAAAGAATCTGAAACTTACGATCTAATG

AAAGCTGCAGATGTTGATAACAGTGGCACGATAGACTATGGAGAATTCATAGCTGCAACA

TTACATTTAAACAAAGTTGATAGGGAAGATCATTTACATGCAGCATTCTCATATTCGATA

AAGATGGAAGTGGATACATCAGAAAAGATGAGCTTCAAAAAGCTTGATGGAAGAATAGAC

TACAATGAATTTGTGGCTATGATGCATAGAGGGAATGCAGAAATGGGTAAGAGGGGTCGA

AAAGGTAGCAGTAGTTTTAGCATGGGATTTAGGGAGGCACTACCAGTATGTTAA

>MS.gene40994.t1

ATGAAGAAGAACAATCGTGGAAAGGCAAACAACCGTGGAAAGATGAACAACCATGGAAAG

GCGAAGATGAAGGAGACATCAACGAACAACCGTGAGTCATTTGCGAAGAAGATGAATGAT

AGAAACGCGCATCACGATTTCATGCCAGAGCCATTTGTCCATATGATAAAGATAAGATTC

CATCATAAGGGAATTTTCACATCTGACCCGGAACTGGCATATTTAAATGGGGAAATATTT

GAACCTGAAGGTACTTGGGATATGGATCAGATTAACACATTAGATTTGGAGAAATTTGTA

ACAGTGATAGGATATCCTGAAACAACTAAGTTTTGGTATGTAGTCCCAGGAAAAGGCCTA

GCTGTTGGGCTTAGAAAGTTGGGCTGTGACAAAGATATCCATGAGTTTGTAAGACTTCAT

AAGGGCCTCAATTATGCTGATCTGTATTTGGAAACACCTGATCATAACCAAACCACTCCA

ACCATTAGAAATGCGACACCTAGCACCCTTAAGCTGATGTGTACCGAGGGTAATGAGGGG

AACATTGATATTGAGGCTAATGAGGTGAACAATGAAGTTGAGGGTAATAATGAAGGGGAT

AAGGATAATGAGGGTAATGAGGGTGGTGATGAGTTTAGTGAATCTGGTGAGGATGACTAT

GATGTAGGTTCTGATCTTGGATCTGGATCAAAATTTGATGATAGTGATTTTGATGAAAAT

TGGGCTTGGACTGAAATACTTCCAGAAGAGACAGTGAATCATGTAAAAGAGGGTAACAAT

GATGTGGATGGTGGTGGATCTGTAGAGGTGGGGGTTATTATTTCATCAAGAAATCCAAAC

CCAACTACTATTGAAGACTTTGTTGATGAAGATGGTGACAGTGAAGACTTAGACACACCA

GATGAGGAGAGTGAAGATGGTGGTAAGAAGAAATATCCCAAGTTCAAAGTTGCTGATGGT

GATGAGCCTGTGAGATTTGAGTTAGGTCAGATATTCACTTCTACAGACCTTGTTAGAACT

GCTGTGAAAAAGTATGGTTTGAAATCTAGGAAGAATGTGCATCTAGAGAAAAATGAGAAG

ACTAGAATTGTTGTTAAGTGTACTGATACATGTCCATTTTATATGAGAGTTATTAAGTCT

ACTCACAAGACTTACTTCCAGGTTGTTAGCTTAGATCCTATACATAAATGTCGCACAAGT

GGTAAGAACAGACAAGAAAAACCTAAACTCATTGCTAGGAAATTGATTCCTATTCTTAGA

CACACTCCTGGTATGCGAATTAAAGCTTTACAAACAGAATGTAAAACTAGGTGGGGTCTG

ATGCTAACTAGATTTCAGGTTTATAGAGCAAAAACAAAGGCTTTGGAGATGATTGAGGGA

AGTATGGTGGAATAG

>MS.gene40988.t1

ATGGAGACGGAGGCAGAGGCAGAGGCGGATCCACTTTATCTGCCTCATGAATTGATCGTC

CAAATCTTAGTGAGGTTACCGGTTAAGCCTCTTATACGTTTCAAATGTGTTTCCAAGTCA

TGGTTTTTTCTTATTTCAAATCCTCGTTTTGCAAATTCACATTTTCAGATTACTACTGCA

ACTCACACTCGTCGAATTCTGTTCTTAACAGAAACTCCTGAATTTCGATCCATAGCTTTG

GATTCATTGTTTACCGATCATTCTGCTCCTACTTTACTCAACCCTAATTTTATGCTTCAC

AAACTTGTTGATCTCAAAATTTTAGGTTCATGTAGAGGGTTCATGTTTTTGTACTCTTCT

TCAAACTTTTACCTATGGAATCCATCCACCGGAGTTCACAAACAAATACCTTGTTCTAAT

TTTTCACATTTAAGATTTTATGGTTTTGGATATGACGAGTCAACGGACGATTACTTGGTG

GTTTCACTGTCCTACGATAATATTCAAAACTCAAATGATATGCTATCTCGCTTGGGGATT

TTCTCATTGAGAGCTAATGCGTGGAACGAAATTGTGAGTCCTACCAATTTGCGTTCTTGT

AGAAAGGCCCCGTTCTCTCGATATCCCGAAGTAGAGTCGGTCTTCAATGGGGCTATTCAT

TGGTTAGCTTCTTCTTATGATATAGGTTTTTTTGCTGTTGTTGCCTTTCATTTAACGGAA

AGGAAACTTGTAGATTTTTCTCTCCCATATGATATTAATTATCGTTCTAGGTATTGTGGT

TTATGGGTATTTAAAGGATTTCTAAGTCTATGGATTTTGGGGAGCAGTTCAGTTGATATA

TGGGTGATGGAAGAATACAATGTGCAGTCATCTTGGACTAAATCTCTTGTTCTTAATATG

GATATCCCTTGCATCCCTTGCATTTTTCCTATGTGTTGTACAAAAAATGGTGACGTTGTT

GGAACAGATCGCCATACTGGACTAATAAGATATGATGACGAGGGAGAGTTTCTAGAGCAT

ACTTACTACTGTAAAGGTTCAGGACGTGGACGTGTGTCTATTTATACCGAGTCTTTGCTT

TCACTCCCCAGTGTCGGTGAGCAAGATTGA

>MS.gene41005.t1

ATGTCATTTCAACATATTCCAGATTTTCATAGGATAATCTTGCAAGATAAGAAACTTAGG

GTTCCTAAGATTTATGTGGAGAAATATTGGAAAGGAATATCAAACCCTATATTCCTTAAA

TTCCCAAACGGTGTTCAACAAGAAATATTTTGGGTGGAGAGTGGATCATATTTTAAAGTT

AAGATATTTGGTGCCAATACTTTAGAAATAAATTATTCCAATATCAAATCCGTTGATGAT

GGTGCTGAAGCCACAAAAAGAGGTCAAGAAAGTGATGATCAAGCCACAAAAGAAGTTCAT

GAAAGTGATGAAGTGAGTGATAAGGGTTCGGATGAGGTTGAAATTCCAAAGCAAGTTCAA

AGAACTAGAAATGGCAAAAGAAAAGTGAGTATGGATTTTGCCACTACCCAACAAAAATTC

TCAGGTAGTAACAAAGTAGCCATGGTGAAGAAGGCTAGAAAATACCCAACATCTGAAGCT

TTAATCGAGAGAGCCAATAATGAAAATCCTTTTTTTGAAATTATAATGTCACATACTTAC

GCCAATGGTTATTACTTGTGGATACCAAGTATATTTTCAAGAGAACACTTGAACAATTTT

AAAGGGACTGCAACAATAAGGGTTGGTAAAGAAAGGACTCTGGAAGTGAGTTTGAGGTAT

TATGATAGCAAGAAAAAATCTTGTATGAGTGGTGGTTGGAAATTATTCAACAGTAAATAT

AATTTGCAAGTTGATGATGTTTGCAAATTTGAGATGATTCAAAGGAAACCAATTTCATTC

AATGTTACTATTATTCGTGCTCGTGCCAGAAACGAACCAACCTCAAAGAAATTCCTAGGT

TATAAAGGGATCTCTTGTGGTGTAAAGAAGGAAGACATTGCAGGAACTTCTAGGAGCCAT

TCAAAAGTAAATTTTCTTGAGGAAGATCGTGATGAGATGGAGCATAACACATTCAAGGTT

ATGGTGAAGAATAGAGTCCCGGGTGTACCAAAGGAGTTTATGAAAAGGGGATGTCATGAA

AATATTGTGGAGTTGAAAATGGGGAGAAAGTCATGGTTCGTAAAAGTCAAGTATTATGAG

AGTATTTGTGGCTGCAGATTCAGTAAAGGTTGGGGAAAATTTATGAAAGAATGCGAAGTG

GAGATTGGAGATACTTGTCTCTTTAAGTTAGTTGATGAGAGAAAGTTTGTGTTTGATGTC

TCAATTGAAGGAAAAAAACCATTATCTGTTTGTTCTAATAAGTTGCCAAAGGCTGAGTAT

ATGGCAACAGTTGAAGCTGCCAAAGAAGCCTTATGGCTTACGGGTTTGGTGAGAGTTTTG

GGTGGAGATGTGTTGTCCAAGTCTCTTAGAGTCCTAGACGTTTAG

>MS.gene41034.t1

TGACACGCATTGAAGCAAGAAGTGAAAAGTTTGACATGTTTATGCAACTAGACATAAATT

CTGAGCTATATCCCTTAAAGATGGGACAGAAATTTTCTCTTGCACTGGTTCCAACACTCA

ATCCTGATGGCACCCCAGATACTGGCTACTATAATCCGGGTAATCGGCAATCAATTGCTG

ATAACTATGAATATGTCATGTATGGAAAGCTTTACAGAGTAACAGAGGGTTCAGGAGGGC

GTGAAAAAGC

>MS.gene40986.t1

ATGGATGCGGAAGCGGAGGTGGATCCACTTTATCTGCCTCATGAATTGATCGCCCAAATC

TTAGTGAGGTTACCGGTTAAGTCTCTTATTCGATTCAAATCCGTTTCCAAGTCATGGTTT

TCTCTTATTTCAAATCCTAGTTTTGCAAATTCACAGTTTCAGATTACTGCTGCAACACAC

ACTCGTCGAATTCTGTTCGTAACAGAAACTTCTGAACTTCGATCCATAGCTTTGGATTCA

TTGTTTACCGATAATTCTGCTCCTACATTACTCAACCCTAATTTTGTTCACAAACTTGAT

TTACCTATCAAAATTATAGGTTCATGTAGAGGGTTCATATTTTTGTACTCTTATCCAAAC

ATTTACCTATGGAATCCATCCACCGGAGTTCACAAACAAATACCTTATTATAATGGTTTT

TCACGTTCAAATTTCTGTGGTTTTGGGTATGATGAGTCAAAAGACGATTACTTGGTAGTT

TTGGTATTCTATATTCCAAACTCAGATTATGTGCTATCTCGCTTGAGGATTTTCTCATTG

AGAGCTAATGCGTGGAACGAAATTGTGAGTCCTCCCCATTTGCCTTGTAGTGAGGTCCCG

TGTTCTGGTTCTGATTATCCCGTGGTAGAGTCGGTCTTCAATGGGGCTATTCATTGGTCA

GCTTTTCGTTATGAGATAGGTTTCTTTGTTGCAGCCTTTCATTTAACGGAAAGGAAACTT

CTAGAGATTCCTCTCCCAAATGACATTGAATTTTGGTCTACAGATTATAGTTTATGGGTA

TTTAGAGGATTTCTCAGTCTATGGGTTTTGAGGTACAAGGACAGAGTTGATATATGGGTG

ATGAAAGAATACAATGTGCAGTCATCTTGGACTAAAACTCTTGTTCTCACCATGGATACT

ACCATTCCTCGCATTTCCCCCATTTGTTGTAGAAAAAGTGGTGATATTATTGGAACAAGT

AGCCGTACTGGATTGATTAGATATGATGACGAAGGAGAGTATCTAGAGCATACCTACTAT

TGTAAAGGTTCAACATACGTGTCTATTTATACCGAGTCTCTGCTTTCACTCCCCAGTGCC

GGTGAGCAAGATTAA

>MS.gene40991.t1

ATGGAGAAGAAACGAAAGATTGTGCAGTATAGGGAGAGGCTAGATAAGACCCTTGCCTCA

CCTGATCTTACAAATGTTGAGATACTCAAAAAGCTTGTCAAAAGTCAACTCCTACCATCA

TCAGAATTAGAAGATGAAGGTGGTGAATATAAGGAGAAATTCGTAGAACATAAAACTGCG

GAAATATCTAACTTTCTTGATATGTTAAGGAGTACTTCAGATGATCATGGAAGGTCTAAT

ACATCACAAAATGATTGGAAATTAAAACAGGATGGCGAAGAGTTCCGTGTTATGTATCGT

GAAGGACCGGAGGGAACTCCCTTTCATACAATGTTAGTGGAAGGCTTTGTGGACGGACCT

GTTGATGTTTGTTTATGTATCTCATGGCAGACTTCTCTTTACAAAAAATGGTGGCCTCAG

TCTACAATTCCTACTTTCAAAATCTTATCATGTGAATGTTTACAGAAGGTTCAGATTGGG

GAACAAATATCACTAGTGAGGATGAAGGTTTCGTGGCCTCTGTCTTCAAGGGAAGCTGTT

GTGCACTATTATCTGTTTGAGTACTTTCAAGACGACTTAATTGTTGTTCTTACGAATTCG

GTCCCTGAGTCAAAGAGCATCATGGGGACCCTTTCTGGTTTCAACGATGACGCAATTCCT

GAAGTGAAGGATGTCGTGAGAGTTGATTTGGTGGGAGGCTTTGCTTTGCAGAAGGTGACA

TCAGAAAGAAGTTACTTTCGGACAATAGCGAATATGGATATAAAGGTGGATTTTGTTCCT

CCATCCTTGATAAACTTTATTTCAAGGCAGCTCATTGGCAATGGTTTCAGACTTTATCAG

AAGGTTGTGGCTTCTGTGATGAGCAGTGATAAAGAAGAATTCAGCAAGGCCTTGGGGGAT

CCATTGTATGCTAGAATTCGCAAGGCTCTATATAGCACGACTACTAGTGCATCAGAGGTT

GCAAATAGTGGAGAGCTTCAACAAGTTGCAAAAATTCACCCTGCTGAATACCTTGTTGAA

AGTAAGCCAGGTGGAGAAAAAGATGCGTCTGAAGAGGATAACATAAGTCAATGTTCAAAT

AATGACATGTCACCAACAATGGATGCAACAGTACTAGAAAATAGCAAAGCATTTGGTGAG

ATTGTAGAATTAGATAAGGAAGAGATTATACAAAGTGTGGAGGATGATGAGAAAGTAAGT

GTCATTCCAAACGAGGAATTAGGCGCAATTGCACAAAAAGGAAAGAGGGGTATAGTTATC

CGCTCGGATGTCAAACAGGCTATAGAAACATTAGATAAGGCCATTTCAATGATTAGGGAA

TATCGATTACATTCCGAGATGGCCTCGACTAGCTTTGCCAATGAAGAGTCCCCTTGCATG

GAAGAAATTGATAGAGTTGATTCATATTCCACAAAAATTATTAAACCTTGTGATATAAAT

GAGGTTATTGTTGAGGTACCAAATAAAGATATATTGGAGGGAACTTCACTAGAAGCACCT

TCGACAAACTCTGGCATCCAAAACTTAAGGTATTCTGGAATAGATCCAAACTTAAAGGAA

ATAAATCCCAACAAAATGATTCCAACTCTATCAGAGCAGAGTCTTTCAGGACCTACTGAG

GCAAGCCAGGTTGGTTCATATTCTTTGGACAGGGGAGCGATTATGGACCAAACAACATAC

CAAAACAAGCAACCAAACACTGATTTGGTCCAAGACATGTCTTCAGATGACAAAGTCAAG

TCAAGAAGGCAGAAAAAAACTAACACCATTGTGACTCGAGCTGCAAACATTGATTTAGCA

AATGAAAGTACTTTTGTGTGGTTGAAGGAATCCTTAATTACATCTTCAACAATGACAAAG

AAATATCTCTCCAAACCTCTTTCAACCTTCTTCAACTTTATACGAACACGAACCTTAACT

TCGATTCCTCAGCGTGTATCACCTTATCTTAAACCTCAATCTCCATCACCACCCTTTACA

CCGTATCGTATTCATAATCTATATCATAATTACGATTATTCTTCATTTGGGTCTCTACGA

TTTTGCAAAAAAAGTTTGTGTTCAAAACCTGCGCACAATTTTCAACCAACATGCTGGAAT

TGTCACGCTGTTCCTCAATCAGCACCTTTCTTGTTCTGCCAGTCGTGTCGTTGCATTCAA

CCTGTTGATTGTTCAAATGACTATTTCCATATTTTTGGATTGGAGAGGAAGTATGACGTA

GGGGGTATTGATTTGGAGGGGAAGTACAAGGAGTGGCAAAAGAAACTACATCCTGATTTG

GTGCATTCAAAATCTCAGGAAGAAAGAGATTATGCTGCTGAACAATCTGCAAGAGTGATT

GATGCATATCGTACACTTAGCAAACCTTTGTCAAGAGCAATTTACATGTTGAAGCTTGAT

GGAGTGGAAGTTGATGAGGAGCAGACAATTTCAGATCCAGAATTATTAGCAGAGATTATG

GAAATCAGGGAAGCCGTTGAAGAAGCAACTAACTCTGAGGCTTTGAATCAGATTCGCTCT

GAGATGCAGGAGAAAATGCAAAGTTGGTCTAATGCCTTCGCTGATGCTTTCCAAAAGCGG

GACTTTGAAGAAGCAAAGAATGCAATTAGAAGAATGATTTATTATACTCGTGTAATTGAG

GAAGTTGTAAAGAAGCTTTGA

>MS.gene40999.t1

ATGCCTCTCACACCCTCTGCATCATCTAGCCAGGGATCAACTGGGTCCTCCCATGCTAGT

GCAATTACTCCCACCATAGCTCCACCGTTCTCATCACCACCTGGTTTTCCTTATTCAGGT

TATCCACCTTTTGGGTCTTCCCCACCTATTGGGCCTCCACCTTCTTACTTTCATTCGGGA

TATCCACCTTACTTTGGGTCTTCCCCACCTTTTCCATATGGAATGACCGAGTTTCCATAC

CTACCACATTCCCAACCCCAACACCCGCCACAAAACCCTCCACAACCCCAAAACCCACCA

CAACCCAAAAATATGGTCGAAGAGGAAGGTGTAGGCCAGATGAAAGGTGGTGGACAGGTG

CAACAGGTTGATGATGTTCTGCTAATTTATCCGACTGACGCAAGGGGTCGTGGAAGACTT

AAGGCGAAGTGGAGGATGAGGAGAAAGATCGCTGGTTTAGGCTCTTTGAGGCAAGGAGTT

CAAGCCAAAGTCAGAGCAAGCGAAAAAAAATCGGGCGTCCGAAAAGGGGGTTGCATTCAC

AGCGGTGGTTGCTTAAGCAACAGAGAGCATGCTGAGCGCTTGGCCAGGAGACTTGGGAGG

GAGCCGTTTATTAACGAGCTTCATGATGAGACACACAGACATATGTCCGACCAATATTGT

GACGAGCGTGCTAGTAAGTATGATCGACAGCTTGCCGAGGCTTTTTCTCAAGGGTTATAT

GTTGATAATAGTTTGGCGATAAAACCAAAGGAAAGCTATATTGTGCCGATTGACTCAAGA

GAAGCTCACCCAGCAAATGGCGAGTCACGAGACCGGTGCAAGATTTGCTTAGCCGCCAAC

GAACATATGAGGAGCAGTTGGCTCAGTTTAGAAGGAGGAAAGGAGGTGGAGAGCGATGAT

GAGGAGTGA

>MS.gene41033.t1

ATGAATCAAGATATCGAAGTTACACCATTTTTTCTTTGCCCTATTTCACTACAACTCATG

AAGGATCCAGTTACTGTCTCAACAGGCATAACCTACGACAGAGAAAGCATCGAAAAATGG

CTCTTTTCATCGGAAAACAAAACATGTCCTGTCACAAAACAACAACTCACACATGATGAT

GCAAATGATCTCATTATTCTCACTCCAAACCACACTCTTCGTAGACTCATCCAAGCATGG

TGCACCATGAATTCTTCTTATGGAATCGAAAGGATTCCAACTCCAAAACCTCCAACAACA

AAAACCCTCATTGAAAAACTCCTCAAAGAAGCTTCTGATTCTTCAGATTCACCTCATTTA

CTTATCCAAACCCTAAAAAAGCTCAAAACAATTGCATCCGAGAGTGAGTCAAATCGACGG

TGTATTGAATCGGCCGGTGCAGTCGAGTTCTTAGCATCAATAGTTACAAAAAACAACACA

AGTTGTTCATCTTCATGTTCAACAACGGAATTAATTGAAGCTAGCTTTGATGATGATGAT

GTTGAAGGTTTTGCTTTTGACTTCAAAATAGGTGCAGAAGATGAAGCTATAAACATCCTT

TACAATCTTCAATTATCAGAACAAGGTTTGAAAACTCTCTTAAACTTCAAAAATGGAGAA

TTTTTAGACTCTTTAATGAGATTATTGCAAAAAGGTAACTATGATTCAAGAACATACGCA

ATCTTTCTGTTGAAATCTATGTCAAAAGTAGCTGATCCATCAAAGCTAGCCAATCTAAAG

ATCGAGTTTTTCGTCGAGTTAGTTCAACTTCTCAAAGATCAGATATCAAAGAAAGCATCA

AAAGCAACGCTACAAACGTTAATCCAACTTGTCGAATTTGGAAGAAATAGAGTAAAAGCA

ATTGAATCAGGTTGTGTTTCTGCTTTGATAGAACTTCTTCTTGATTGCAAAGAAAGAAAG

CCATGTGAAATGATTTTGGTGCTTTTGGAGATGTTATGTCAATGTGCTGATGGAAGATTT

GAACTCTTAAGCCATGGATGTGGTTTAGCTATTGTTTCAAAGAAGATTTTGAGGGTTTCA

ACAATGGCTAATGATAGAGCTGTGAGGATTCTTCTTTCTGTTTCAAGATTTTCTGCTACA

CATTTTGTTGTTCAAGAAATGTTACGAATTGGTGTTGTTGCAAAGCTTTGTTTGGTTCTT

CTAGTTGATAGTGGAAATAAGGCAAAGGAGAAAGCAAGGGAGATTCTTAAATTGCATTCA

AAGTCTTGGATGAATTCTCATTGCATACCTTTTAATTTACTTGCTTCATACCCAACAACT

AGTGGGTGA

>MS.gene41032.t1

ATGAATCAAGATATCGAAGTTACACCATTTTTCCTTTGCCCTATTTCACTACAACTCATG

AAGGATCCAGTTACTGTCTCAACAGGCATAACCTACGACAGAGAAAGCATCGAAAAATGG

CTCTTTTCATCGGAAAACAAAACATGTCCTGTCACAAAACAACAACTCACACATGATGAT

GCAAATGATCTCATTATTCTCACTCCAAACCACACTCTTCGTAGACTCATCCAAGCATGG

TGCACCATGAATTCTTCTAATGGAGTTGAAAGGATTCCAACTCCAAAACCTCCAACAACA

AAAACCCTCATTGAAAAACTCTTCAAAGAAGCTTCTGATTCTTCAGATTCACCTCATTTA

CTTATCCAAACCCTAAAAAAGCTCAAAACAATCGCTTCCGAGAGCGAAACAAATCGTCGG

TGTATCGAATCAGCCGGTGCAGTCGAGTTCTTAGCATCAATAGTAACAAAAAACAACACA

AGTTGTTCATCTTCTTGTTCAGCAACAGAATTAATTGAAGCTAGCTTTGATGATGATGAT

GTTGAAGGTTTTGCTTTTGACTTCAAAATAGGTGCAGAAGATGAAGCTATAAACATCCTT

TACAGTCTTCAATTATCAGAACAAGGTTTGAAAGCTCTCCTAAACTTCAAAAATGGAGAA

TTTATAGACTCTTTAATGAGATTATTGCAAAAGGGTAACTACGATTCAAGAACATACGCA

ATCTTTCTGTTGAAATCTATGTCAAAAGTAGCTGATCCATCAAAGCTAGCCAATCTAAAG

ACCGAGTTTTTCATCGAGTTAGTTCAACTTCTCAAAGACCAAATATCAAAGAAAGCATCA

AAAGCAACACTACAAACGTTAATTCAACTTGTCGAATTTGGTAGAAATAGAGTAAAAGCA

ATTGAATCAGGTTGTGTTTCTGCTTTGATAGAACTTCTTCTTGATTGCAAAGAAAGAAAG

CCATGTGAGATGATTTTGGTGCTTTTGGAGATGTTATGTCAATGTGCTGATGGAAGATTT

GAACTCTTAAGCCATGGATGTGGTTTAGCTATTGTTTCAAAGAAGATTCTGAGGGTTTCC

ACCATGGCTAATGATAGAGCTGTGAGGATTCTTCTATCTGTTTCAAGATTTTCTGCAACA

CATTTTGTTATTCAAGAAATGTTACGAATTGGTGTTGTTGCAAAGCTTTGTTTGGTTCTT

CAAGTTGATAGTGGAAATAAGGCAAAGGAGAAAGCAAGGGAGATTCTTAAATTGCATTCA

AAGTCTTGGATGAATTCTCATTGCATACCTTTTAATTTACTTGCTTCATACCCAACAAGT

GGGTAA

>MS.gene41010.t1

ATGGCCAAGACTCTGTTATTGTGTTTTCTCTTTCTCCAAATAACCACTTTCATAGCATTT

GCTGAAGAGCTAGAAACTCTTCATCATAAACCAGCAACTCCACTTCATCCCCCAACTAAG

TCACCGGTTCATAAACCATTAGCTAGCCCACCTCACCACAACCACTCACCATCCCATGCA

CCTTCCCATGTTCACACTCCTTTACATCCTCCTCACCCCGCTAAACCCCCGACCCACCAT

CATCACCACGACCAGCAACAACATCACTCACCATCCCCTACCCCTTCCCATACTCACCCT

CCTTTACATCCTCGTCACCCAGCAAAATCCCCAACTCATCATCATCATCATCAACATCAC

TCTCCTGCTCATGCCCCTATTAAGCCTCTGATTCACACTCCTTTACATCCTCCTCACCCA

GCCAAACCTCCAACCCATCACCGCCACCAACATCACTCACCTTCCCCTTCCCCTTCCCAT

GTTCACACTCCTATACATCCTCGTCACCCTGCAAAACCCCCCACCCGTTATCATCATCAC

TCTCCTGCTCATGCCCCTATTAAGCCTCCGGTTCACAAACCTTTACTTCCTCCTCACTCT

GCAAAATCCCCAACCCACCACCATCACCATCCTCCGGCTCATGCCCCTACTCACACACAT

GTTGTTTCAAAAAGCTTGATAGCTGTTGAAGGAGTTGTTTATGTAAAATCATGCAACCAT

ACTGGTGTTGCCACCCTAAAGGGAGCTACACCACTTCTTGGTGCCATTGTGAAGCTCCAA

TGCAACAACGGCAAACACAAGTTGGTACTTAAAGCCAAGACGGATAAGAAAGGTTACTTT

TATATTGGAGGTCCAAAGAATATTGTAGGTTATTCAACTCGCCATTGCAACCTTGTTTTG

GTTAGTGCGCCAAAAGTACTAAAACCCACAAATCTTCATGGTGGTCTTACTGGGGCTCTT

CTTAAACCTGTGAAACGTTCAATGTCTAAGGGTTCTTCTGTAAAACTCTACTCTGTTGGA

CCGTTTGCATTTGAGCCCAAATGTCATCATTAA

>MS.gene41017.t1

ATGGAAGGTTATCTTAAGGATTTCGAATTGGAAGACAAGGATCGTTCAATCGAAGCTCTC

ACTAGATGGAGAACTGCCGTTACTTTGGTCAAGAATCCTCGTCGAAGGTTTCGAAACGTT

GCAGATCTCGCCAAACGTGCTCTAGCTCAAGAAAAGCAGAAGAAAATTCAGGGAAAGCTT

CGGGCTGTTATCACCGCTCAACGAGCAGCACTGCATTTTACGGATGCTATTGGTACACCT

GAATTCAAGGTATCAGAAAAGACTAGAGCAGCTGGTTTTGGTATCGAACCGGATGATATT

GCATCAGTTGTTCGAAGCCATGATTTCAAGAACTATAAAAAGGTTGGTGAAGTTCAAGGG

ATTACAAGTAAACTTTCCGTCTCAGTTGATGAAGGTGTCAGTCAAGACAGTATACATAGT

AGGCAAGAGATTTATGGGCTCAACCGTTATACCGAGAAACCATCTAAAAGTTTTCTGATG

TTTGTTTGGGATGCACTGCATGACTTAACACTGATCATTCTAATGGTTTGTGCTTTAGTT

TCCATAGGCATAGGGATTCCCACAGAAGGGTGGCCAAAAGGTGTTTATGATGGTGTTGGA

ATCTTACTTAGTATATTCTTGGTTGTCACTGTTACTGCTATCAGTGACTATCAGCAATCT

CTGCAGTTCTTAGATTTGGACAAAGAGAAGAAAAAGATTTCTATTCATGTTACCAGGGAT

GGTAAAAGACAGAAGGTTTCAATTTATGACTTGGTAGTTGGAGATATAGTTCATTTGTCA

ACTGGTGATCAAGTTCCAGCTGATGGAATTTTCATTCATGGATATTCATTGCTAATTGAT

GAATCAAGTTTGTCAGGTGAGAGCGAACCTGTAGATATAGACAATCGAAGACCTTTTCTT

CTTTCGGGAACCAAAGTGCAAGATGGTCAAGCGAAGATGATAGTTACAACTGTTGGCATG

AGAACTGAATGGGGAAAGCTGATGGAAACTTTGAGTGAGGGAGGAGAAGATGAGACTCCA

CTGCAGGTGAAATTGAATGGAGTTGCTACAGTAATTGGTAAAATCGGTTTGACTTTTGCT

GTGCTGACGTTTTTGGTCTTGACAGCAAGGTTTGTGATTGAAAAAGCAGTTAATGGAGAC

TTCGCTAGTTGGTCTTCAGAGGATGCACTGAAGCTACTAGATTACTTTGCCATTGCTGTA

ACTATAATAGTTGTTGCGATTCCTGAAGGATTACCGTTAGCTGTAACACTTAGTCTTGCA

TTTGCAATGAAAAAATTAATGAATGATAAGGCGCTTGTCAGACATCTTTCTGCTTGTGAG

ACTATGGGTTCAGCTAGTTGCATTTGCACTGATAAGACAGGAACGTTGACGACCAACCAT

ATGGTGGTTGATAAAATTTGGATATGTGAAAAGACTGTGGAGATGAAAGGTGATGAAAGT

ACCGATAAGTTGAAATCAGAGATTTCTGATGAAGTTCTAAGCATCCTTTTGCAGGCTATA

TTTCAGAATACTTCTTCGGAAGTAGTTAAAGACAAAGAAGGAAAACAGACAATACTGGGA

ACACCAACAGAATCAGCAATATTGGAATTTGGCTTGGTTTCAGGTGGTGATTTCGATGCA

CAGCGTAGATCCTGTAAGGTACTTAAGGTTGAGCCTTTCAATTCAGACAGGAAGAAGATG

TCGGTGCTAGTGGGTCTTCCTGATGGAGGGGTCCGAGCTTTCTGCAAAGGTGCATCAGAA

ATCGTGCTGAAAATGTGTGATAAAATCATTGATAGTAATGGAACAACTATTGATCTTCCT

GAAGAGAAAGCAAAGATTGTGAATGACATTATAGATGGATTTGCCCATGAAGCTTTGAGA

ACTCTTTGTTTGGCTGTCAAAGACATAGATGAAACCCAAGGAGAAACCAACATCCCGGAG

AATGGATATACTTTGATAGCCATTGTGGGAATCAAGGATCCTGTGCGCCCTGGAGTTAAG

GAAGCTGTTCAAAAATGTTTAGCAGCAGGAATAAGCGTTCGCATGGTCACCGGCGATAAC

ATAAATACAGCTAAGGCTATAGCTAAAGAATGCGGTATACTTACTGAGGGAGGTGTAGCT

ATAGAAGGACCAGAGTTTCGCAATCTGTCTGAGGAGCAAATGAAGGATATCATACCTAGA

ATTCAGGTAATGGCACGATCCTTGCCTCTTGACAAGCATACCTTGGTAACCCGTTTGAGG

AATATGTTTGGTGAGGTTGTTGCTGTTACTGGTGATGGAACCAACGATGCTCCTGCACTG

CACGAGTCAGACATTGGACTTGCCATGGGCATTGCCGGAACCGAGGTTGCCAAAGAAAAT

GCTGATGTCATTATAATGGACGACAACTTCACTACTATTGTCAAAGTGGCAAAATGGGGA

CGGGCCATATACATAAACATTCAAAAATTTGTGCAGTTTCAGTTAACAGTCAATGTTGTT

GCTCTGATTACTAATTTCGTTTCTGCATGCATCACTGGTGCTGCTCCACTAACTGCTGTT

CAATTGCTTTGGGTTAACTTGATTATGGACACTCTTGGTGCATTGGCTTTGGCTACTGAA

CCTCCTAATGATGGACTTATGGAAAGACAACCAGTTGGAAAGAAAGCAAGTTTTATTACC

AAACCAATGTGGAGGAATATCTTCGGTCAAAGTTTGTATCAACTGATTGTCCTTGGAGTT

CTAAATTTTGAGGGGAAGAGGCTGCTGGGACTAAGTGGCCCAGATCCAACTGCTGTACTC

AACACTTTGATATTCAACTCCTTTGTATTTTGCCAGGTGTTCAATGAGATAAACAGCAGA

GAAATTGAAAAGATAAACATATTCAGAGGCATGTTTGACAGCTGGATATTTTTAAGTGTT

ATTCTCGCCACAGCGGTATTTCAAGTAATCATAGTTGAGTTCCTGGGAACATTTGCCAGC

ACTGTCCCTCTAACCTGGCAATTCTGGTTACTCAGTTTGTTATTTGGTGTACTTAGCATG

CCTTTAGCTGCTATCCTCAAATGTATACCAGTTGAAAGAGATACCACAACAAAGCATCAT

GATGGTTATGAGGCACTGCCTTCTGGTCCTGAAAGAGTGTGA

>MS.gene41027.t1

ATGGAAGAAAATCAAGAGAGTACAAGTGTGTTAGCAGCAGGTCCATTCTTTGGTGAGTCA

ATTAACCCTGCATGTGCTTTTGGATCTGCTTCCATTGCTGGAACATTCAGGAACCAAGCT

TTCTATTGGGTTGGTCCTTTGGTTGGTGCAGCTGTTGCTGATCTTCTTTATGACAATGTG

TTGTTCCCTTCTCAGAATTCAGATTCTATTAGAGAGGTTTCAGATGGATGGAACAGTTAG

>MS.gene41003.t1

ATGCATTTGAAGCTAGGTCAAATTTCAACCATAGTAATTTCTTCACCAGATATAGCACAA

GAAGTGCTTCAAACCCATGATCTCTCATTCTCAGATAGAACAATTCCTCAAGCAGTCGCA

GTTCTTGATCATGAACATTTTAGCTTACCCTTTATGCCAGTTTCAGATCTTTGGAGAGAC

CTAAAAAAATTATGCAAAAACCATTTGTTTTCTAACAAAACAATTGATGCTAGCAATGAA

CTTAGGTGTAAGAAGCTTCAAGAGCTTTTAAGTGATATTGATAGAAGCAGCTTAACTGGT

GAAGCAGTAGATGTTGGAAGAGCTGCTTTTAAGACTTCATTGAATTTTTTGTCAAACACT

TTTTTCTCTATGGATTTTGTTAACTCCACAGGTGAAACTGATGAGTATAAAGATATTGTT

GAAAATCTTGTGAGAGCAATAGGAACACCAAACATGGTTGATTTTTTTCCTGTTTTGAAG

ATGTTTGATCCACAAGGTATCAAGGCAATATCAGCTACTTATGTTGAAAAGATGTTGCAA

ATCATTGATTCCTTTATCACCAAAAGATTGAAGTTAAGGGAAGCGGAAAATTATGTCACA

AATGATGACATGTTAGATACTTTGCTTAACATCTCTCAAGAGAATGCCCAAAAGATGGAC

AACACAAAGATTAAACATTTGTTTCTTGATTTATTTGTTGCGGGGACTGATACAACTTCC

TACACAATAGAACGAGCAATGGCAGAACTAATCCACAACCCACATGCAATGTCAAAAGCC

AAAGATGAACTTGAGCAAATAATTGGAATAGGAAATCCAATTGAGGAATCAGACATTACT

AGACTCCCATATTTGCAAGCAATAGTAAAAGAAACATTACGTTTGCATCCATCAGCTCCA

CTTTTGCTACCAAGAAAAGCCAAGATAGATGTCAAAATTCGTGGATACATTGTTCCTAAA

GGTGCACAAGTCCTAATTAATGAATGGGCCATGGGAAGAAACCCTAATATTTGGGATAAT

CCAAATTTATTTTCACCTGAGAGGTACAAAATGGTGATAGAGAAATTGGTCAACCCTTGC

GAGCTATTCCTTTTAGAGTAA

>MS.gene41004.t1

ATGGGGGATGATTCATTGATTAATTTGGACAACTCGAGGGCTAATTGGACTCCGTCTCAA

GACCAATATTTTCTTGAGCTTTTGCTGTCTCATGTTCACAAAGGCAACAAAACTGGCAAA

GTATTTACCAGATTAGCTTGGGCAGACATGACTGAACAATTTAACAGCAAATTTGGGTTC

AAATATGATGTAGATGTGTTGAAGAATCGTTACAAACGATTCAAGAAGCAATACTATGAA

ATAAAAGCGATGGCTAGTCAAAATGGATTCCAATGGGACGGGAGACTAAACATGATCACA

GCTAATGATAAAACATGGGAAGAGTATATTAAGGCCCACCCCGATGCTCAAGTGTTTAGA

AAAAAACTTTTTCCATGTTATAATGATCTATGCATAATCTATGGCCACACAGTTGCTGAT

GGGAGATACAGCCTTTCATGTTTTGATGAAGGTTTTGAATATGAAGAAAATGCTTCAAAA

GAACTGGATGACCATACTAGCACCAGTAAATTAGTGGATGATCAGACTCCTGCTACTCCT

AGTCAAAGCAAAATCGATTGGTCTCCAATGATGGACAGAGTTTTTGTTGAACTGATGTTG

GACCAGGTGCGTAAAGGGAACAAGGCTGGTCGTACTTTCACGAGGCAAGCCTGGGGGGAC

ATGGCAGAGTCATTTAACAATAGATTTGGATGCCACTATGGTAAGGTTGTGTTGAAAAAC

CGTTTCAATGTCCTAAGTAGGCATTATTCCTCTATAAATGAACTTCTTGGAAAAGAAGGT

TTCAGTTGGGATAAGACACAACACAAGGTTGTGGCTAATGACCATGTTTGGCAAAAATGC

ATCAGGGTAAATCATAAGTTCCGGCTGTACAGAATTAAAAGCATGCCTTTTTATTCTGGC

ATGTGCATTGTATGTCGTGATGAAGCTCCTGCAGATTGCAAGTCAAATCATGGAAGGAAA

TCTTATGGCAGCAAGAACTTTGCCCCCGACCCTAATGCATCATTGCATATAGGTGGTGAA

AATAATTTTATCGGAGATACTCAGCCCCTCCCTTATGCATCATTGTATACAGGTGATGGA

AGTAATTTTAGCAAAGAACCTCTCCCTGATACAGCATTCCATGTAGGTGGTGAAAATAGT

TTTACTAGGGATACTATTTCTCAGCCTCTACCTTACGCAGCATTGCATATAGATGATGAA

AATAGATTGACTAGAGGTACTCAGCCACTGCCTAATGCAATTTTGAATCTAGGCGGCGAA

AATAATTTTACCGGAGATGCCATTAATGCAGCATGGCATGTAGGTGGTGAAAATAGTGTT

ATCAGAGATACCATTACTCAGCCTCTCCCTAATGCAACATTGGATATCGGTGGTGAAAAT

AATTTTACCAGAGATGCTATTACTGAGCCTCTCCATAATGCAACATTGCATATAGGTGGT

GAAAATAATTTTACCTTTATAGGTGGTGAAAATAATTTTATCGGAGGAGCTCAGCCTCCC

GCTAATGCAGATAACGAAGGCGGTGGAAAGAACTCTACCAGAGATGAAGGCATTGAAATG

ATCTTTACCAGAAAAACTCAGCCTCAGAAAAACTCTACAAGAGATGAAGGTGGTGAAAGG

AACTTTACCAGAAAAAGTCAGCCTCAAAATGCAGATAAAGAACCGTTGCTGCTTCTAAGT

GCTGGAAAAAATGTTTCTAGTCAGAAAAAAAGACATCAAACAAAAACGCCGGCAACATTG

AAAGAGCCTAAGAAGGCAAGAAACTACAATGAAGGCATGTCAGTTGCGTTGAAGCATATG

GCAGTTGCAGTTACATCGCTAACGAATAAAACAAGAAAAGAAGATAATTTTTCTGTTGGT

AATGTTATGAAGGTGCTTCAGGCTATTCCGGACTTGGATGACGATCTGATATTAGATGCA

TGTGATTTTTTGGAAGATGAGAAAAGAGCAAGGTTGTTTCTGGCATTGGATGCTAATTTG

AGAAAGAAATGGTTATTGAGAAAGCTTCGTTCATAA

>MS.gene40985.t1

ATGGCAGAGGTGGCGGAGCTTATGGCGGAGGTGGATCCACCACCGTATCTGCCTGATGAA

TTGATCACCAAAATCTTAGTGAGGTTACCAGTTAAGTCTCTTATTCGTTTCAAATCCGTT

TCTAAGTCATGGTTTTCTCTTATCTCTGATAATCACTTTGCAATTTCACATTTTCAACTT

ACTGCTGCCACACACATTCGTAGGATTCTGTTCGTAACCGGAAATTCACAGTTTCATCTT

TCCTCTCCAACACTCTTAACTGCTACTCCTGAATTTCGATCTATAGATTTACCGTCATTG

GACGATGATTATAATGAACCTGTTCCACTGAACCCTAGTTTTTCACTTCCGGAATCTCAT

TTTGATCTTCAAATTAAAGGTTCATGTAGAGGGTTTATATTTTTGCACACTTGTTCAAAA

GCTTATATATGGAATCCATCCACCGGATTTCATAGACAAATACCTTTCTCTCCTAGTGTT

GCATATTTAATGTTTTGTGGTTTTGGGTATGACGAGTCAACCGATGATTACTTGGTGGTT

TCACTTTCGTATGATTACTTGGGGTTTTTCTCATTGAGAGCTAATGAATGGGAAGAAATC

GCAGATACTGTCGATTTGCCTTTTTGTAGTAGGGCCTTTTCCTTTGTCTATCCCTTAGTA

GAGTCGGTCTTTAATGGGGCTATTCACTGGTTGGCTTTAAATCTTGATACATGTGACTAT

CTTGTTGTTGCATTTGATTTAGTGGAAAGGAAACTTGTAGAGATTCCTCTCCCAGATGAT

ATTGACTATCGTTCTACAGATTGTGGGTTATGGGTATTTAGAGGATTTCTAAGTCTGTGG

GCTATAGTGGACGAGGATACGGTTGATATATGGGTAATGAAAGAATACAAAGTGCAGTCA

TCTTGGGCTAAAACTCTTGTTCTTACTACTTATGATACCGTTCATGGCATTTCCCTTGTA

TGTTGCACAAAAAGTGGTGATATTGTTGGAACAGATTGCCGTACTGGATTGGTGAGATAT

GATGAGGAAGGAGAGTTTTTAGAGCATACCTACTATTGCAAAGATTCACGAAATGGGTTC

CGATTGGCTATGTATACGGAGTCTTTGCTTTCACTCCCTAGTGACAATGAGGAAGCTTAA

>MS.gene40989.t1

ATGGGAAACAACTGTGTCGGAACGAGAACCTTTTTTTCGAAGGATGAATCATCATCACGC

GCAACTTTTCCAAGTCCATCTTGCTGGTCACGTTCCAAGAAAGATAGTGCACACAAAACA

AGTCCAACAAAACCAAAAGAATCTGTTCAGAACAATCCTCCACCGGTAATGAAAATCGAA

AAGGAAGATGAAAAGCCACCACCACAGCAGCAACATCAACAACAACATCATCAAAAACCG

CGACAAAAACCACAAACCAATGAAGCTGTTGTTGCTACACCTGCAAAACCAAAGAGACCT

CACAATGTGAAGAGACTAGCAAGTGCAGGGCTAAAAGCAGATTCAGTTTTACAGCGAAAA

ACGGTTAGTCTTAAGGAGTTTTATACTTTAGGACCAAAGCTAGGACAAGGGCAATTTGGA

ACTACATTCCTTTGTGTCGAGAAATCTACTGGGAAAGAGTATGCATGTAAGTCAATCATG

AAGAGGAAGTTGTTGACTGAAGAGGATGTGGAAGATGTGAGGAGGGAGATTCAGATTATG

CATCACTTGGCTGGCAGTTCGAATGTGATTTCGATTAAGGAAGCTTCTGAGGATGCAGTT

GCTGTTCATGTTGTTATGGAGTTGTGTGCAGGTGGTGAACTTTTTGATAGGATTGTGGAA

AGAGGGCATTATACAGAAAGAAAGGCGGCGAAACTTGCAAGGACTATTGTTGGTGTTATT

CAGTCATGTCATTCTCTTGGTGTGATGCATCGCGATCTTAAGCCGGAGAATTTTCTTTTT

GTTAATCAGCAGGAAGAATCTCCCCTTAAGGCAATCGATTTTGGATTATCTTGTTTTTTC

AAACCAGGTGACATTTTCAATGATGTAGTAGGAAGTCCATATTATGTTGCACCTGAAGTT

TTACGCAAGCGATATGGTCCAGAAGCAGATGTGTGGAGTGCCGGTGTCATCTTGTACATT

CTCTTATGTGGTGTGCCTCCATTTTGGGGCGAATCGGAACAAGATATATTTGAGGCCATT

TTGAATAATGATCTTGATTTCTCATCGGATCCATGGCCTAGTATCTCTGAAAGTGCAAAA

GACTTGGTTAAGAAGATGCTTGTCAGAGACCCTAGTAAGCGGTTAACAGCTTTTGACGTT

CTCCGTCACCCGTGGATCCAAATTGATGGAGCAGCACCAGACAAGCCTCTAGATTCTGCA

GTTTTGAGTCGCATGAAGCAGTTTACTGCAATGAACAAGCTCAAGAAAATGGCACTTAGA

GTTATTGCAGAGAATCTATCTGAAGAAGAAATTGCTGGATTGAAAGAAATGTTTAAGATG

ATCGACACAGATAATAGTGGTCATATTACCTTTGAAGAACTCAAGGTTGGACTGAAAAGA

TTTGGTGCCAATCTAAAAGAATCTGAAATTTACGATCTAATGAAAGCTGCAGATGTTGAT

AACAGTGGCACGATCGACTATGGAGAATTCATAGCTGCAACATTACATTTAAACAAAGTT

GATAGGGAAGATCATTTACATGCAGCATTCTCATATTTTGATAAAGATGGAAGTGGATAC

ATCACAAAAGATGAGCTTCAAAAAGCTTGTGAAGAATTTGGCTTTAGAGATGTCCCCTTG

GAGGAAATGATCCGAGAAGTTGATCAGAACAATGATGGAAGAATAGACTACAATGAATTT

GTGGCTATGATGCATAGAGGGAATGCAGAAATGGGTAAGAGGGGTCGAAAGGGTAGCAGT

AGTTTTAGCATTGGATTTAGGGAGGCACTACCAGTATGTTAA

>MS.gene41009.t1

ATGAGCAGGTTAGGCATTGTGATAGTGAATGTGATTCGGAACATTGCAGGACAAATTATT

TATAAAGAGATTGTCTGGTCTAATTTTGATTGTGCAGGATGGTGCAAGATAGTTATGGAG

ATGAAATTTCCTACCCAACCCATGTTCCTGTTTAAGTTTAAGATTGAGTCGTGTTCTTTA

CCCGGATCATATAAAAGCAGCAACTTTAAGTACTTTGTTGTCAAGTGGTTGAAGAAGAAG

GACATAACCTCCTGTCTCCTGACATTGTCTTATGATAAGTTTATGGATTCGATGCTTTCA

AAGGATTGCAAGACATTTAAGCTGATTGATGATACCGGACATGCTTGGAACTGTACGAAT

AATTTTGAACGGTCACCTTTGAGGCAATGTAAAATTGGTGGAGATTGGAAGCATTATTGC

ATAAGTTTAGGACTGTGTGAAGGAATGGTGATAAAGATTGGTGCTCCGTCCCTTGAATAC

AATGAAGTTTTCTTTATCACATTGAAATATTATGGATTGGATTGA

>MS.gene41013.t1

ATGACATTTGAAGAAGAACAAGTTGATGATGTTGATGAGATCCAGAAAGAACCTCACATA

GGAGCTACTCAAGATGTCAGAACCTCTCAAATACAAAGGAGCATCGATAAGGTTACGGTT

CCTTTTTTTGTCACCAAATTTCCAGATGAGGTGGCAAACGAAGAGCTTTGGAAGATTTTT

CTTCAGTATGGTAGAGTAGGAGAAGTGTTTATCCCTAAGAAGGTAGACAAATGGGGGAGG

AGGTTTGCCTTTGTGAAGTTTAGGGAGGTGAAGTCGGTGGAAGAATTAGAGAATAAGCTG

AAGGATGTATGGTGGGGAAATCATAAGCTCGAGGTTAATAGGGCTAGATTTGGGAAAGAA

GAGACGGAAGGGCCGGTACAATCACAACTGAAGAGGAAGGAGATAGAGCATAGGGCTGTC

GTGGCTGAGACTTCTTTCCAGAGTGTACTGGTGGGTAACAAGCAGATCGCGAAGGTGGGT

GGAAGAGGTGTCGAACTTCCGGCAATGGAGTTTCTTCCGGCGGAGGACCTTCTCGAGGAG

CTGAAGAGTTGTTTTGTGGGCCGTCTCACTCATCATCTCGAGTCAGATGCCTTACAAACA

CTCTTGTTCATGGAAGGGTGGCGTAACATTAAGGTGTCCCCCATGGGTGAGAAGCTTGTG

CTGCTTAAGGAGGACAGCTTGGGGGTTTTGGCAGCAGCGAGGGAGGAGAAGCAGGTTTGG

TGGAAGGCAACGTTTGTTGAGGTGGTGCCGTGGTCCCCGATTCTAGTGGCTTCGTCTAGA

AGGGTTTGGGTCCAGCTGAGAGGTATTCCTTTACATGTCTGGTATGAAGATTTTTTCAAG

AAAGTTGCAAATCTTTTTGGTGTTTTCTTGGATTTCGATGAGGATACGGTGTGTAGGAAG

AGGTTTGATGTTGCCAATCTACTCATTTCAACTAATAGATTGGGTAGGATTGATGAATGT

GTGAATGTCAAGGTGATGGGAGCGGTGTTTCGTATTTGGCTGGTGGAAGGGTTGCCGTCA

ATTCAGGAAGGGGAAGGGAGTGTGGAAGAGGTTTCAGAGTCTTCTAGGGAGAGGTGCGAT

GCTTGGGCGGAGGAGGATGATGGTGATAGGCGGCCGGAAGTTGCAGGGGAGGAAGAGGAC

CTTTTGACCGATGAAGAGAGTGAGGAAGATGAAGTGGATAGACAATCCGTACAGCAAGGT

ACTGGTCTATTCCCTATGACTTCTGTAGGACAAAAGGGGAGTATGGAGGGGACCCACCAG

GCTGAACAGCAGGGAGAAAAAGACTTTGTGGAAAGGGTGGCTGACACACCTGTTAGATAT

GGACAAGTGGCAAAGCTTCCCCAACAATCTTTGTTGTTGGGAGATGGGAAACAAGTGGGT

GGCGATGATGTGGGGATGTGTGAGCCACGTGGTCAGTTGGGAATGGAGGGAACAGAGACC

AATTTTGAAATTCCATCCTTAGCTGCACGTGACGTTGAAGTTAACTTGAGGCAAAATGTC

AATGTTGTGGGGCCGCAAGAGGTTGTGCTAGAAAATGGGTCAGCGGGTTTATTTGCTGGA

CCGGACCCTCCAGTTCCTAACTGGGTTGCTGGGCCTTCAACTCCAGCCCATTCAAATCCT

GATTCTGAAGAAACAATTTCAGAATTTTCTAATAGAGCGGATATTGGTTGTTCTAGGAGG

TGTATTAAGAAAAAAGCTTTTATTAATGTAAGACCAAGGGCCTTGGGTAATGCTAAATGT

ATGTTTTTTGCTCAAGCAATTAAAGAGGGAAAGGGACCTCAAAGGAGGAAAGGAAACTTA

CGGCAGGGAAGGAAAAATAAGGGGGTTAGGCGTAGGGAGGAGATTGATGAAGCTTTGGTG

CGGGGAGGAGGTTTTGTGCGAGATGATGAGGCTTTAGGGGCTGAGAGGATGACACAACCG

GCTGTGCCTTACTGCCCAGACACGCCGGCAGCGAGCTCCCATTTGCTAGCGACTCCGGCT

TCGGGTTTGCGTTTACTCTTGGATGAGCCCAACTCTATGGTTCCGGAGACTCCTATTCCT

TCATTATCGGATTCTAGTAGGAAGGATTTGGATGCATCTCGTCTTCATACTCTCCAAAAT

GGCTGTTTGCATCAGTTAGCAGGAACAGCAGCCGCTGGGTTTTGGATCTTGGTTCAGCGG

CCTTTGTCACCTGTATCATGGTGTATTGGCTGCTGA

>MS.gene41043.t1

ATGTTGGAGAGACTGATTCCTATTTTTGATGGAAAGGAAGATGCCTACTGGTGGTTGATC

CAGTTAAAACGGTACTTTGAAGCCAATATTTGGATCACAGAGAAAATGAAGGTCGACTGG

GTTACTCTGTATGCTTTCAGAGGAGAAGCTTCCAATTGGTGGTCTTATTGGAAGAAGGGC

AATCAGAATGCGACGTGGAAGATATTTGAAAGAGGTTTTATTCAGAAATTCATCCCAGAC

CTATGGGATATGCTTGAGTCTGCAGAAGGCGAGGAGCAAGAAAGCCATGAGTACATTATG

AATGAGAACACAGAAAACTATGAAGAGTCAAAGGAGGTTGGTAAGGAGACAAACTCTGGA

TCGCTGCTTAGTTTGAGCAGCATCGAACCACCACCAGAACGGTCACATCAGGAATTTCAT

GTGACACCATTCACAAAATGCATGGTACAAAAATCTGCAGGTCACAATGAATCGATGGTA

GTGTTTGAGACAATCATCGATGCAGCAACTAAGACACAAAGATGTGAGAAGGAGAGAAGG

ATTGTTCCCGATCTGCACCCTCCACAAGAGACACCAGATCCGCCTTCATCACAATCACCA

CCACCAGAGTCGCTAGACTCTAGTCAACCTGCGACAGATCTCCAACGGAGAGAACCGCCA

CCCAAACCACCGGATTGGTCGTCTTCAGATGCAACAAGAGACAGGCCCCGATCCAAGGAG

GAAGGGAAAGAGCCAACAATTAATACTCAGCACATACCACCGCCGCTGAAATGGCCGGAA

CCGACTGGCATCAACCAACTTGCAGTGGTTCGACCGCTACCGACGACGAGCAACACACTT

GCAACTGAACGGTTACGTTTCAACGTCGCTTCAGTTGGGAAGGATTGTGAGGTCAAAAGG

ATAACAGGGTCAATTGTTAAGGTTGGTATCTCTTTTAATCTAATGGATCAGACCCAAGTT

ATTTTTTGTTTGGTATGGAAGGTATTAGAAATGTGGACAGGAATAGAGTTACTAGGTTCG

GTTCAATTTAATGATAGCCATTGGGTTATGAAAAAGGGGGTGGCTATAGTAGATGATACA

AACCAGAAGGAGAAAAACAGTGGAACAGATTGGTCAGGTTGTATGTTGGATCTAACAATA

AACCTAGTGGGTCAGGCACAACACAATTCTAGTAAGTGGGTGAGAGAAATTAAAAGGTGG

AGCAATGTAGAGTCACCAGATTCCAAAAATAGCATAAAACATAGACAACCCTATGATTGT

AATTTAGAGGTTTTTGCATCAAACCTTTTCAAACAATCATTGGTGATTCATCCTTCAGTC

ATAAAGATGCTATTGATGAAGAGGCTCTTAAAGGATAACATATGTACAAATATTCTCAAG

CACACTATTTTTCAATTAGATCAAATAGGAATTCCATCTCAAGGTCATTGGAGCAGCCCA

TATTTGATTTCTGATAAGTTTATCCAAGTGAAAGGTGAAGGAGTTACTAAGTACAACAAA

GTGGTTTTATCTATATTTTTGAAGACACGCGTAATTCTTTTTATGAGCAGGAGGAACATG

GAAGTGGAAAGGGAATTATCACTGCCATTTACTCTTAATATTGAGATCTCTTCAATGGTG

GTGATGGCTTTGATTGATGATGTATTAGGCCCTTGGCGTGTGAAATATATTAGTTGTCGG

TTGCCAACCAATTACAATGGGAGTGGAATTGTGTGGATGACTCGATCATTTGAAATGGGC

TTGTCCGGCTCCCATCAATGGGATCCTAGAGGAAATGACTCGGTTTTCGAACACTACAGC

ACAATGAGAGTTTTTGGGGAATTCATTTATCAGGAAATTCAATGTCTCAACGCCATTGTC

CTAGTTGTCGCCTTACTTTGTGCTGTAGCAACAGTTGTCTTGCTGAAATTGTTCATGTTC

AATTGCTCGGGTGACTTGCACGCTACTTGTGTCAAGGCTACTCTAGTAGTTAGGAATGAC

AAAATGTACTCAAACCAATTCTCTCATCTCAGCCTTGAGGACACGGCTGTTTTTCTAGTG

GTCGACAGTACTTTGTCACTCTACTTCAAGTTATGGGACCCTGGTGGACACTTTTGTTAT

CCTTCACCTTGA

>MS.gene41066.t1

ATGGCAGAAGCAAAACCCCACAACCGCGGCAGAAGATGTTGTTGCTGCCTTTTTGGCATA

ATTTGGAAACTCCTCGTAGCAATCATCGTTCTCGTTGGTCTAATAATCCTTATTTTTTGG

CTCATTGTCCAACCTCGAACCTTCAAGTTCAGTGTCAATGAAGCAAAACTCACCAAATTC

AACTATAGCGACGACACCAACACCCTCCATTACAACCTCGTACTCAACTTCACGGCCCGT

AATCCAAACAAAAAACTCAACATCTACTATGATGTAATCGAAGGACATGCGTCTTACGAA

GGGACTAGGTTTGCTTCAACAAAGGTTATAACATGGTTAAATTCATTCCGACAATACACT

AAGAGCTCGAATCCTATGAGTGGTGTTTTCTCTGGGCAACATGTTGTGGTTTTTGATCAT

GATCAGGTTTCTGACTTTGAACGTGATAAGAAAGATGGAGTTTTTCATATTGATATGAAA

TTATACTTTGAGATGAGGTTTAGACTTGGTGATTATATTGGTCCATCTACTAAGGGTAAT

ATTAAGTGTAGGCTTATTGTTCCTTTTGTTGCTAATGGAACTAAGGTCATGAAAGCATTT

GAACCCACCACGTGTGATGTAAATTTCTAA

>MS.gene41064.t1

ATGGTGGATATGTGTGTGAAAAGCTTGTGCGTGAAAACTGTTAATGACACATCTGAATTT

GAACTCAACAGGAAATGGACATGGATAATGTTGGTAATTCTCAGGAACTCATCATTTCAC

AAGAAAGTGCACACCAAACTACTACTGGTCTCACTCCCTCATCGTAGTCGACTTCATTGG

ACAACCTTGGAGGCTTTGATGTGTGCTAGAAGTTGGCTATGGAGCAAGGAGAATAATGGT

GATGTGAATTCTCAATTATGTAATGAGTTTGCAACTTTGCTTAATGAAATAGAACCTGAT

GATGAATGTGAAATATTGACTAGTGGTGTTACTTCCCATTTTGAAGAGTGA

>MS.gene41047.t1

ATGCCTCAAGGAGATCATATAGACCGTCACATAAAAGAAAATGGCCGCCGTTTCGATCAC

GAAACCCGCAAGCGCAAGAGAGAGGCTCGTGAGGTTCACAACCACTCTAAAAAGGCCCAG

AAGACTATTGGTCTTAAGGGAAAGAGGAATGCCAAGAAAAATTATGCCGAAAAGGCACAG

ATGAAAAGGACTTTGGCGATGCATGAAGAGTCAACATCTAGGCGCAAGGCTGATGATAAT

GTTCAGGAAGGAGCTGTTCCTGCTTATCTTTTGGATCGTGAAAACACAACCAGAGCAAAG

ATTCTCAGCAACACCATTAAGCAAAAAAGGAAGGAGAAGGCTGGGAAATGGGATGTTCCT

CTACCTAAGGTACGTCCTGTGGCTGAAGATGAAATGTTCAAAGTGGTCCGCACCGGTAAA

AGAAAGACTAAGCAATGGAAGAGAATGGTTACAAAAGCAACATTTGTTGGGCCTGGTTTT

ACCAGAAAACCACCAAAGTATGAGCGATTCATTCGTCCCACTGGATTGCGTTTCACTAAA

GCTCATGTCACTCATCCTGAACTTAAATGCACATTCAATCTTGAAATTATTGGAGTGAAG

AAAAACCCTAATGGCCCCATGTACACCTCTCTTGGTGTCATTACCAGGGGATCTATAATT

GAGGTGAATGTCAGCGAACTTGGACTGGTTACACCTGCAGGGAAAGTTGTGTGGGGTAAA

TATGCCCAGGTTACCAACAACCCAGAAAATGATGGTTGCATAAATGCTGTTTTACTTGTT

TAA

>MS.gene41062.t1

ATGACTATGTCTATATATGTTCCATTGCAACAAGAAGATCCAGATGATGAATATCCCAAT

CAAAAAGTGGACAAGGAAGCTCAATATTCAGGTGATGCTGATGAAATGGTTTCTCAACCA

CAAAGAAGCAAGGGTGGTCTTATCACTATGCCTTTCATCATTGCAAATGAGGCACTTGGC

AATACTGCAAGCTTAGGAATTTTGCCCAACATGATATTGTATTTGATGGGACCTTTCAAG

CTTCATCTTGGAGAAGCTAATCAAATACTCCTCTTATCTGCTGCAGCCAGCAAGTTCATG

CCTGTGGTTGGTGCTTTTGTTGCAGATTCTTATCTCGGTCGATTCTTAAGTGTTGGATTA

GGTTCTGCTGTCAGTTTCCTGGGAATGGCATTGTTGTGGTTAACAGCAATGATCAAACCA

ACGGAAGGCGATCAATCACCAACATCATGGGAAATGGCAATGTTAATATCTGCCTTTAGT

CTCATGTCAATTGCAGGTGCTGGTGTTTCATGTTCCATGGCATTTGGTGCAGACCAAGTA

AATATAAAAGATAACCCTAATAACAATAGGGTCTTGGAAATGTTTTTCAACTGGTATTAT

GCTTTTGCAAGTATCTCTGCGATAATAGCTCTCACTGTAATAGTATATATCCAAGATCAT

TTTGGTTGGGAAATTGGTTTTGGAGTTCCAACAGCACTCATGTTTTTATCCACACTGTTA

TTCTTTCTTGCTTCTCCTCTTTATGTAAAGATTCAAAAAAGAACCAACTTGTTCGCTAGT

TTTGCGCAAGTAATCGTTGCTTCCTATAACAACAGAAAACTTCCATTACCGCCTAAAAAT

TCACCTCAATTTTACCATCACAACAAGGACTCAAATCTCGTGGTTCCAACTGATAAACTA

AGGTTTCTGAACAAAGCTTGTGTTATTAAGGAATTTGAACAAGATATTGCATGTGATGGT

TCAAGAATAAACCCCTGGAATCTATGCACAGTAGATCAAGTAGAAGAATTAAAAGCCATT

GTGAGAGTTATTCCATTATGGTCTAGTGGGATCATGATGACCCTTAGCATTGGAGGCTCA

TTTTCATTGCTTCAAGCTAAAACCTTGAACAGACATATCACCTCCAACTTTGAAGTTCCA

GCGGGATCTTTGAGCGTTATCAATATAGGTACGGTAATTATATGGATAGTTTTCTATGAC

CGCGTTCTTATTCCTTTAGCATCGAAGATAAGAGGGAAACCAGTTACGATCAGTGCAAAG

AAAAGAATGGGAATTGGTTTGTTTTTATCTTTTCTCTACTCGGTAAATGCAGCGATTTTT

GAGACTATAAGGAGAAGAAATGCGAACGATGGTGTTTTGGAAATGTCGGCATTGTGGCTT

GCACCTCAACTTTGTCTGGCTGGTATATCTGAAGCAGTCAATTTCATAGGCCAAAATGAG

TTTTATTACACAGAGTTTCCAAGTACTATGTCTAGTGTTGCTGCTTCGCTTTCTGGATTA

GGAATGGCTGCAGGAAGCTTGGTGTCTTCTTTGTTGTTCAGCATTGTAGAAAACACTACT

TCAAGAGGAGGAAATGATGGGTGGATTTCTGATGATATTAACAAGGGTCATTTTGACAAG

TACTCTTGGCTTATAGCTGGAGTTAGTGCTCTTAATATACTCTATTATCTAGTTTGCAGT

TGGGCTTATGGACCTGCAGTTGAAGAGTTAACTAATGATTAA

>MS.gene41037.t1

ATGGCAACGTATTTTCATAATAATTCAGAAATCCAAGGTGGTTCTGACGGCCTTCAAACA

CTCATTTTCATGAATCCATCAGCTACCAATCACCCCGGCAACACTTCCCTCCACCACCAA

CAACACCAGAACTTATCTCACGCGCCACCACAGTTTGTCGGGGTCCCACTCTCGGCAGAA

CAATCAGTACATGGCCATCATGAGGTTTCTGCTTTGCATGGCTTCCCACCGCGCATGCAG

TACAACATGTGGAACCCCGCCGACCCTAATTCAGCGGCGCGTGAAGCTACACGCTCTACT

CAGGGACTGTCATTAAGCCTTCATGCTCAAGGGTCGGGTGAGGACGCGCGTGTTCCCGGT

GGTGGGTCGGGTTCGTCTGCTTCAAACGGCTGTGTTTCGGGGATACAGAGTGTGTTATTG

AGTTCTAAGTATTTGAAAGCTACACAAGAATTACTTGATGAAGTTGTTAATGTTAATGGT

GGGATTAAGGTTGAAAATGTTAAGAAGAGTTTTGAGAAAAATAAGGTTGTTGGAGAATCT

TCAACTGCGGTTAGTGGAGAAGGTGGTTCTGTTGGTGGTGGTGATGGAAATGGGAAACGT

AGCACTGAGTTATCAACTACTGAGAGACAAGAAATTCAGATGAAGAAAGCTAAGTTAATT

AACATGCTTGATGAGGTGGAACAAAGATACAGACAATATCACAACCAAATGCAGATGGTG

ATTTCCTCGTTTGAACAAGTTGCAGGAATTGGTTCAGCAAGAACCTACACTGCCCTAGCG

CTACAAACAATCTCCAAGCAATTTCGATGTTTAAAAGATGCAATTACCGGCCAAATTAGA

GCTGCGAATAAGAGTTTGGGAGAAGATGATAGCTTTGGTGGCAAAATTGAAGGGTCTAGG

CTCAAATATGTTGATCATCATTTAAGACAACAAAGAGCTATTCAACAATTGGGAATGATC

CATCATAATGCTTGGAGACCTCAAAGAGGATTGCCCGAAAGATCTGTTTCTGTTCTTCGT

GCTTGGCTTTTCGAACATTTTCTCCATCCTTATCCCAAGGATTCAGACAAACACATGCTT

GCAAAACAAACAGGACTTACTAGGAGCCAGGTTTCAAATTGGTTCATAAATGCTCGAGTT

CGTCTTTGGAAACCAATGGTGGAGGAAATGTACATGGAAGAGATGAAGGATCAAGAGCTG

AATGGTTCCGAGGACAACAAATCAAGCAAAAATACCGATGACGATCCTTCGATGAAAACA

CCAACTCCACAAGAAAGAGTTCCAACCTCGGAAACAGAATCCAAAAGTTTCAATTCCAAA

CAAGACATTCCTATGGTTTCAGTTTCAACACCATCAACTTCACCTATTGGTGTGAATGTT

AGAAACAATTCAGGATTCAGCTTCACGGAACTTGATGGAATAACACAAGCAAGTCCAAAG

CGAACGAGGAACCATGAAATTCTTCAATCACCGAATCATGTGAAGTCCAATGAAACAACA

ACTAATAATGAACAAATTTCAATGAAATTTGGTGATGATAGACAAAGTAGAGATGGTTAT

TGTTTCATGGGAAACCAAACTAATTTCATTGCTGGTTTCGGTCAATATCCAATGGAGGAA

ATCGGAAGATTTGATGCGGAACAATTTGCACCAAGGTTTTCAGGTAATAATAATGGTGTT

TCACTCACTCTTGGTTTGCCTCATTGTGACACATTATCAGGAACACATCAAAGTTTTATG

CCAAACCAAAACATTCAACTAGGAAGAAGATTGGACATTAGCGAAACAAATGAGTTTGGT

GACTCTTCAGCTGCGTTTGAGAGTATCAACATGCAGAATCCAAAGAGGTTTGCCGCACAA

TTGTTACCGGATTTTGTGGCATAA

>MS.gene41055.t1

ATGGGAATTCCTCAAGCCATGGTTGCTCTACACGAACGAGCATCGTTCGTGAAAGACTCG

CTTCACAAAAGCCAAACCATTACCGATAACATGGTTTCGATTCTTGGTTCGTTTGATCAT

CGTCTTTCTGCGCTTGAAACCGCAATGCGTCCTACTCAGATTAAAACGCATTCGATTCGA

AGTGCGCATGATAATATTGATAAGACTTTGAAAGCTGCGGAGGTTATTTTGGGGCAGTTT

GATCAGACGCGTAAGGCGGAGGCTAAAATACTGCGAGGGCCACATGAGGATCTAGAAAGC

TATTTGGAAGCAGTTGATCAGCTTAGAGCTAATGTTCGTTTCTTCAGCAGCAAAAAGAGT

TTCAAAAGCAGCGACGGTATCATCAACCATGCAAACGGCTTGCTTGCTAAAGCTATCTTA

AAGCTGGAAGATGAATTTAAACATCTTTTGACAAATTACAGCAAGCCTGTGGAACCTGAT

CGCCTTTTTGACTGCTTACCAAACTCTCTAAGGCCAGCAGCGCAGCAGAATGCCGGTGGC

GGCGGCAAAAACCATCCAGAAAAACAAAGCATAGAAACTGTCACTTTCACCCTCCCAACT

CTTATTCCTCCCAGAGTTATACCATTGCTTCATGAATTAGCAGAACAAATGGTTCAAGCG

GGCCATCAACAGCAGCTCTTTCGAATCTATACAGACACCCGTGCTGCAGTCCTGGAGACG

AGTCTAAGGAAGTTAGGTGTAGAGCGGCTGAGTAAGGATGATGTTCAAAAAATGCAATGG

GAGGTTTTGGAGGCTAAGATTGGAAACTGGATTCATTACATGAGGATTGCTATCAAATTG

TTGATTGTTGGGGAAAAAAAAATCTGTGATCAAATATTTGATGGACATGATTCTGTGAAA

ACTCAATGTTTTGCTGAAGTCACTGGTAGCAGTGTGGCTATGCTTCTTAGTTTTGGAGAG

GCAGTTGCCAGAAGCAAGAGGTCACCTGAAAAACTATTTGTTCTTCTCGACATGTATGAG

ATAATGAGAGAACTTCAACCAGAGATTGGGATACTGTTTGATAGTAATGTTTGCGCTGAA

ATGCGGGAAGCTGCAATCAATTTGACAAAGCGACTTGCTCAGACAGCCCAAGAAACCTTT

GGTGACTTTGAGGAAGCAGTTGAAAAAGATGCTACTAAAACTGCTGTGCAAGATGGAACT

GTGCACCCTCTAACCAGCTATGTTATCAATTACGTAAAATTTCTCTATGATTATCAAGCA

ACTCTGAAACAACTTTTTCAAGAGTTTGATCCCGCCGATCCAGAATCTCAGTTAGCATCT

GTTACAACAAGAATTATGCAGGCTCTCCAGAATAACCTAGATGGAAAGTCTAAACTATAT

AAAGACCCAGGACTAACCCAGTTATTTCTTATGAACAATATCCATTACATAGTGAGATCT

GTGAGAAGGTCTGAGGCAAAGGACATGTTGGGGGATGACTGGGTACAAATACACAGAAGA

ATTGTACAGCAGCATGCAAATCAGTATAAAAGGATCTCTTGGGCAAAGATCTTGCAGTGC

CTCACTGTCCAAGGAGGTAATTCATCTGGTGGAGGGGACAGCAACAGTAATATTTCAAGA

TCATCGGTTAAAGAAAGGTTCAAGACATTTAACACTCAATTTGAGGAGCTTCATCAGAGA

CAATCTCAGTGGACTGTTCCTGATAGTGAATTGCGAGAATCACTTAGACTGGCTGTTGCT

GAAGTTCTTTTGCCTGCCTATAGATCTTTCCTTAAGCGTTTTGGGCCCATGATTGAGAAT

GGCAAAAATCCTCAAAAGTACATCAGATACTCTCCCGAGAACCTTGAACAAATGCTCGGT

GAATTTTTCGAAAGCAAGGCGTGGAGTGAACAGAAACGGTGA

>MS.gene41057.t1

ATGGCTTCATTTATCAATTATGCAACATGCATTTCAATCATAGTTGACAAACTTAAGTTT

CCTCGCCTTAGTTATTCCGACTTGAAGTACATTGCCTTCACTATCTATACCATAATCCTA

TCTACTTTCTTCTTCATTTCAAGCCTAATTATTTTCAGCCTCTTCATAGCCAACTTTAAA

CCCCAAATTCCAACCGTTGATCTTGACTCCGTCAACATCACCAACCTTAATACCGAAGCT

GTTAACTTAACGGCCACATTTGATTTATCATTTATTTTTGAAACTCCCGAAAACCATAAC

CGAAACGTTTCTTACCGTGACCTCGAGGTTAGGGTTTTGTGGTCAGGAAAAGACAAAATT

ACCCTTGCCAAGAAGATCTTGCTCCCGTTTTCACAAGTGGAAAACAACGTGACTAAGATC

CGAGCTGAGTTAACGGTGATTGATGGGTTTACTAACAGCATTGATGTTTCAACTGGAATT

GATGCTGAAAGAGATAGTGGAGCGGTTCATATTGGTGTGAGATTGATGGGTTTGTTTATG

TTGAGGGATGGTTTTTCAAAAGCTATGAGAATTGATTTCGATCGAATTGATGTGAAGTTC

CCACCTGGTAGCAACAATGGTACCTATGTGCTGGTTCCTCGCCCCATATTCCCAAATCCA

ATTTTATGGTCTTATGTTATGTAA

>MS.gene41067.t1

ATGGCTCTGTCGATTAGCGGTTTAAGAGCACCCATTCAAGCCACTGCCACTTTCGATAGA

GACATCTTTATCAACTCACTCAAACTTAGATTCAGAACATATCCGGTTGAATTTGATCCT

ACCAACACTTGCAATGAAGAAGATGACGGTGAGTCCAACAATGAAGAAATAACTTTGCTA

AATAAGATTCCTCTATTGGAAGGCATAGAATGTAGTGATTCCAATGACAAGGCTAGCATC

GCTTTAACCGCTATTGAAGACACTCCAAGTTACAAATCTGGAACAAAATGA

>MS.gene41039.t1

ATGAACTCATTTTCCGTGATAAATTCATCTATGAAGGCGAAGGTGGTGGAGAAAGGAGAA

GGGGGTCGAGATGCATCTCTGTATTCAAGGCCATCGCAAAGGCAGGTCATATTACGTATA

TATCATGGTGGGTTGTTCATGGAATCACCTAGCAACATCTATGTTGGTGGTAAAATGGAA

GAAATGAATTGTGGTTGGGATGTCGATGTCGTGTCCTACATGGATATTTCAAAACTGATT

GAAAGTCTAGGGTATAGTGATTTCAGAAGCACCTCAACCTCGAGGATCACAAGCATAAGT

CGTTAG

>MS.gene41040.t1

ATGGCAAGTCACTCCCATTCACAACCCTCAAACACCTTCACCTCTCTCTCCCACCGTTGG

CGAGAAGATCTTCGCCACATACGCGACCGTGCAAACTCCTTCACAACACGAAACCTTCCT

TCCACCACCTTCGACGACGTCGATTTCGTCAAAAACTTGCGCCCTAAGCTTTCCGAGTTT

CCTAAGCGAACGAGTATTCGAATGAATCTCTCTGCTATTAGGGATGCTATTGTTTCCGAA

GTTGAAGACGATATGGATGGAATTTTGGGTAATAATAAGAGGTTTAATTGTAGTGAGTTT

TGGAATGGGGATAATGAACAACAATGGTGTAAGGATTGGGAACCAATTCGTGTGTTTAAG

AAACAGTTCAAGAAAAATACCCAGTTTTTGGAGAAGTTGAAGTCAAGTTTGAAATCAATG

TGCAATGAGACTGAAGATTCAAAGGAAGTTCGCCCATTGGATGTATCTGAACTTCTGGCT

TATTTTGTTAGACAGTCAGGACCATTTTTGGATCAACTTGGAGTCAAGAGAGATATATGT

GACAAGATTGTGGAAGGCTTGTATAGCAGACGTAAGAACCAACAACAACTCCACTCCTTT

TCTGGGGGAGAATCTTCGGTTCTTGGGAATGGTAACCTAAATGATGAATTGGATTCGCGG

ATAGCAAGTGTTCTTCAGAGTACAGGGCACCGGTATGATGGTGGATTCTGGACAGACCAT

GCAAAACATGATCTGTCAGACAATCAGAGGCATGTTGCAATAGTCACGACTGCTAGTCTT

CCTTGGATGACCGGAACTTCTGTAAATCCACTATTTCGTGCTGCTTATCTATCACAATCT

TCAAAAAAGAAAGTTACTCTGCTAATTCCATGGCTTTGTAGATCAGATCAAGAACAAGTT

TATCCCAGCAATCTCGCTTTTCTCTCACCAGAAGAGCAGGAGGTTTATATACGAAATTGG

CTTGAGGAAAGAATTGGATTTGAAGCAGAATTCAAAATCTCCTTTTATCCTGCTAAGTTT

TCTAAAGAAAGGCGAAGTATAATGGCTGCTGGCGATATTTCTAAATTTATACCTGTCAGG

GATGCTGATATTGCTATATTGGAAGAACCAGAACATTTGAATTGGTACCATCATGGTAAA

TGTTGGACGAAGAAATTCAATCATGTTGTTGGTATTGTCCACACAAATTATTTAGAATAT

ATCAAGAGGGAGAAAAATGGGGCCCTCCAAGCATTCTTTGTGAAACACATAAACAACTGG

GTTACAAGAGCATACTGCCACAAGGTTCTCCGTCTTTCTGCAGCCACTCAGGATTTACCA

AAGTCTGTAATTTGCAATGTTCATGGTGTGAGTCCCAAGTTCTTAAAAATTGGAGAAAAG

ATCGATGCAGACAGAGAGCTTGGACAGAAGCCATTCACAAAAGGGGCATATTTCTTGGGA

AAGATGGTATGGGCAAAGGGATATAAGGAATTGATTCATTTATTAGCAAACCATAAAAAT

GACCTTGACGGCTTCAAGATTGATGTATTTGGTAATGGAGAGGATGCTAATGAAGTTCAG

AGTACAGCTAAAACGTTGGATTTGAATATCAACTTTCAGAAAGGAAGGGACCATGCAGAG

GATTCTCTTCATGGGTACAAAGTTTTCATTAACCCTAGTGTTAGTGACGTGCTCTGCACA

GCTACAGCAGAGGCACTTGCGATGGGAAAGTTCGTAGTTTGTGCCGACCATCCTTCAAAT

GAGTTCTTTAGGTCTTTCCCAAACTGTTTGACTTACAATACATCTGAAGAATTTGTGGCA

AAAGTTAAAGAAGCGTTAGAAAATGAGCCCCGTCTTCTTACACCTGAGCAAAGATATCAA

CTTTCTTGGGATGCTGCTACTCAAAGATTTATGGAATATTCCGAGCTCGACAGAGTGGTG

AACATCGAGAAGGGTGCTACAAAATCAAATAAAAATAATGGAAAGCTCATTGCTAATTCA

CTTTCAGTGCCTAGTCTCACCGAATTAGTAGACGGAGGATTAGCATTTGCTCACAACTGT

CTCACTGGGAATGAATTCCTGCGATTATGTACCGGAGCAATTCCTGGGACGCGTGAATAT

AACAAGCAGCATTGTGAAGACCTTCAACTCTTGCCTCCACAGGTAGAAAATCCTACCTAT

GGCTGGTGA

>MS.gene41059.t1

ATGGCTTCCTTCATTGCAAAATCCATATCCATCATGTTTGACACCGTTTCTTCAGTCACT

TCCGAAGTGAAGGCCATTTTCTTCACTCTCTGCACCATGTTCCTTTGCATTTTCTTTGGC

CTTACAAACCTTGCTTTTCTTAGCCTATTTATAACCCCATTTCAGCTCAACACCCCCCTT

GTTCATCTCGACTCTATCACCGTAACATCCCTCAATACTGAAGCCGTTGATTTAACGGCT

ACATTGAATTTAACATTATTTTTCCGTGCTCCCAACTACGATCATCAAATCGTTTCTTAC

AGAGACGTTGAGGTCATCGTTTGGTGGTCAGGAAAAGATGATGTTTTTCTTGGCAGAACA

ATCTTGTCACCATTTACCCAAGCGCCTGATAGTGTGACTATGATTAGCGCTGGGGTAACG

GTACCCCCTGGGATCAGTAACGACAGTGATATTGCTATCGGACTTGGTGCTGAAAAAGAT

CGAGGATCGGTGCAAATTGGGGTGATTTTGTTGGGGTATATGATGACGGATAGTGCTTCA

GTATCAATGACGTTCAAGTTTGGCCCAAATGCTGTAAAATTTCCAGCTGGTAGCCAAAAT

GGTTCGTGGACTTGGGTGATTCCTCGCCCAATATTGATGCATAATTTCATGGTGTAG

>MS.gene41041.t1

ATGAATACGGAAGAGCTCGTGCAGCATACAATTGAGGCCACATTAGAGGTGGCAACCTTA

GACCTTGGTACAACGTCTCAGGATCATGGAGAGGAATGTAATGCGCATTCAGATCACATC

CCCGCAACAACATCTCATTTGGTAACTCTTATAACCCGCAACCCTATTACACTTGTTAAT

ACTTTTAGCATGCAACTTGATAATGTCTCTAATGAGATGGTCAGAAATGATATGGAAGGG

AACCACAAATTTATTTTATCTTCCGTCAAAGAAAACACACTAGATGTCAGTAGCATCGTG

ACTCCAGATGATGTCCTGGTAGATCCGGTACTGCAAAAAGATATAGATTTTATGCAAACT

TGGTTAGCAAAGGCAGTTGTGAATGATGCGCCTGTTACACAGGTAGTATCAAAGTCTCAG

AAAAAGAAATTGCAAAAGGCTTCATACCAAACCCGCTCTCTGGGCTCTCTTCCGTCGTCT

AAATGA

>MS.gene41054.t1

ATGTCCAACGGTAGAAGTACGCCATTTCCCGTCATCTATAGCGACGGCGAAACCGAAACC

ATCCTCGGAATTCTAAGGCTGTACCCCACCTCAGATATGAATACTCTTCTTTTCATCCTC

AGTGAAAAGATCAAAATCTTGCCACATCTGTTTACTGTATTTCTTGCGAAACAAGGCAGC

ACCTGGAAAATCCCTTTCTCATTCAAGATCAACATTGCCGAATTCGATAATAAACACGGC

GAGTACTATTTATATGTCAAGTCTTCGGTGAAGATGAACAAGAACCCATCTCAGAAATTC

AAGCCTCTGAACAAAGCTGGGAATTTAAGTGATGAATACGTTGAAAGGCTGAGAAGAGGA

CCGATTGGACAAGGCAAAGTTGGTGTCTGTGGAGAGTGTTTCATGGAGAAAATAACAGGG

ATTGATGCTGGTTTCCATTTGTGTATTGGTGATAAGGAAATTTTAGGGTTTCGAACAACT

GCGGGTCCGAGTTCCCGGCCAGTGAGAAGTTCCGGCAAGAATGGTCACTGA

>MS.gene41044.t1

ATGACTGAATCTTTCATTGTATGTACAGGCATTTTTATCGGCGATGACAAAGTTATCCAC

TTTACTAGGCGTGGACAAGAAGTAGGAACCGGTACTGTACTAGATGTTCTCCTCCTCAGT

TCAGCACCAGCCAGGTCTCAAGAAATTTGCCCAAATTGCACTCCATCCCAAGACGACCAT

GGGGTCGTCGTCTCATGCCTGAACTGCTTTCTGGCAGGAGGTGTCTTGTACCGTTTTGAG

TACTCTGTCTCCCCAGCTCTCTTTCTTGCGAAAGCACGCGGTGGAACATGCACTCTTGCA

GTCTCCGATGAAGACGATGTTGTGGTTCATCGAGCTCTGTATTTACTCGAAAACGGCTTT

CGTTGCTATAACGTGTTCAAGAACAACTGTGAAGACTTTGCCATCTATTGCAAAACCGGG

TTAGTTGTTGTTGATGAAAGAACAATTGGACAGAGTGGCCAAGCAGTTTCCATCATAGGA

GGACCTCTTGCTGCTGTTCTATCAACGCCGCTCCGCTTAGTGACTACAAATGTTTATGGA

ATGGCGGCAACTGCCGTTGGAGTTTATTGTGCTAGCAGGTATGCTGCAGACATTGGAATG

AGGAGGGATGTGGTGAAAATACAAGTGGAAGAGTTGACAGGAAGGTTGGCTACTGGGTTA

CTGCAGGTTATTGAACCACAAGTTTCGATGAATCTGGCCCCCCCTCAATCTACTCAGATT

GTTAGTCAATGA

>MS.gene41053.t1

ATGGAACTGCCAATGAGCAACAACAGCGGAAGCGGCGGTGCTCACAGCGGAAGAAGAGAA

AGTGTGTACTGTAAGGAGTGTGTGATTGCGGAAGTGACAAGGGTTAAGGCTGATTTCCAT

TTGTGTGTTCGTGATGAGGTAATTATAGGGTTTAAGACGTCGGCGGGACCAATTTTCCGA

CCGGTGAGGAATTCCGGCGAGGATGGTTAA

>MS.gene41048.t1

ATGAAAAATGTTGGAAAGAATGAGATTAGTCAAAGCAGCAAAAATAATGAGAATATTTGC

TATCGTTGTGGAGGCAAAGGCCATTGGAGTCGTACATGTCGTACGCTAAAGCATCTAGTT

GATCTTTACCAACAATCACTTAAAAATAAAGATAAAAAGGCTGAAACTCATTTTGCATGT

GAAGATGATGACTATGATTATGGTAATATGGATGTTACCCACTTAGATGCTGCTGATTTC

TTTGTTGATCCTGATGGAAAGATTGATCATCTCATTGGAGATGGAAGTGTCCAGAAATAG

>MS.gene41046.t1

ATGGCGGAGGTCGCGGAAATTATGGCGGAGGTGGCGGAACCGTATCTACCTGATGAATTG

ATCACCCAAATCCTAATGAGGTTACCGGTTAAGTCGCTTATCCGTTTCAAATCCGTTTGT

AAGTCATGGTTTTCTCTTATCTCTGATAATCACTTTGCAAATTCACATTTTCAACTTACT

TCTTCAACTCACACTCGTAGGATTCTTTTCTTAACCGTAAATTCACAGTTTCAACCTTCC

TCTCCAACACTCTTCACCGCTACTCCTGAATTTCGATCTATAGATTTACCGTCATTGTTT

ACCTACGATGATTATAATGAACCTGTTCCACTGAAACCTAGTTTTCCACTTCCGGAATCT

CATTTTGATCTTCTAATTAAAGGTTCATGTAGAGGGTTTATATTTTTGCACACTTGGTCT

GAAGCTTATATATGGAATCCATCCACTGGATTTCATAGACAAATACCTTTCCCTCCTAAT

GCTTCACATTTAAGTTTTTTTGGTTTTGGGTATGACGAGTCAACAGATGATTACTTGGTG

GTTTCAGTTTCGTTTGATTACTTGGGGTTTTTCTCATTGAGGGCTAATGAATGGGAAGAA

ATCGTGGATACTGTCCATTTGCCTTTTTGTAGCAAGGACTCTTCTTTTGTTTATCCCTTA

GTAGAGTCAGTCTTTAATGTGGCTATTCACTGGTTGGCTTTACGTCTTGATACACGTGAC

TATCTTGTTGTTGCATTTGATTTAGTGGAAAGGAAACTTGTAGAGATTCCTCTCCCAGAT

GATATTGACTATCGCTCTACGGATTGTGGTTTATGGGTATTTAGAGGATTTCTAAGTCTA

TGGGTTATGGTGGACGAGGATACAGTAGATATATGGGTAATGAAAGAATACAAAGTGCAG

TCCTCTTGGACTAAAACTCTTGTTCTTACTACTTATGATACCATTCATAACGTTTCCCTT

GTATGTTGCACAAAAAGTGGTGATATTGTTGGAACAGATTGCCGGACTGGATTGGTGAGA

TACGATGAGGAAGGAGAGTTCATAGAGCATTTCTACTATTGCAAAGATTCACGAAATGGA

TTCCGATTGGCTATGTATACAGAGTCTTTGCTTTCACTCCCTAGTGACGACGAGGAAGCT

TAA

>MS.gene41052.t1

ATGGAACTGCCAATGAACAACGACCGCAGAAGCGGCGGCGCTCACAATGGAGGAAGAGAA

AGTGTGTATTGTAAGGAGTGTGTGATTGCAGAAGTGACAAGGATTAAGGCTGATTTCCAT

TTGTGTGTTCGTGATGAGGTAATTATAGGGTTTAAAACGTCGGCGAGACCAATTTTCCGA

CCTGTGAGGAATTCCGGCGAGGATGGTTAA

>MS.gene41065.t1

ATGTCGGTGAAGGAGTGTGATCATCACAAAGGAAGAAAAAGAATACTCTTCCAAAGAATA

TTTTGGGCAATCATGGTATTCCTCTTCTTAGTCCTACTAACAATTCTCCTCATATGGGCA

ATTCTAAGACCAACAAAACCTACCTTTATCCTCTTAGACGTAACCCTATATGCTTTCAAC

GCATCACAACCAAATTTCTTAACCTCCAACTTCCAAGTCACACTTTCATCTCGAAATCCC

AACGATCACATCGGTGTATACTACGATCGTCTCGATACCTACATGACCTACCGGAGCCAA

CAGATTACTTACCGGACCGCCATACCGCCTTCTTACCAAGGTCACAAAGAATATGACGTT

TGGTCGCCGTTTGTTTATGGAAATGATATACCTGTTGCTCCTTTTAACTCTGTGAGCCTT

TCACAAGATGAAAATAACGGTAATATGTTTGTTATCGTTAAAGTTGACGGTAGAGTTCGG

TGGAAGGTTGGTGCTTTTATTTCTGGTCGTTATCATATACATGTTCGTTGCCCTGCTTAT

ATCTCACTTGGTAACAATGGTAGAGGTGTTGATATGGGAGAAAACGCCGTTAAGTATCAA

GTGAATCAACGTTGCTCCGTTAACGTTTGA

>MS.gene41051.t1

ATGAACTTTTTGGAGAGAAATATGCTTCAATGCCGTGACGGTGGTTCCCGACCGGTGTCT

GTGGCGGAAGCTTTGGTTTTTGAACGTGGTGAAATGGAGAGAAGGATGCTTAAGTTTAAG

ATGGAAAGAGAGGCGTTTTTAATGAGAATGGCAAACAATGTTGAGTCTTTTAGAGTGCAA

CAGCCGATGGATAATGGCGGAGGAAGCCGTGTGTATTGTAAGGAGTGTGTGTTGGCGGAA

GTGACAAGGACTAATGCTGGTTTCCATTTGTGTGTCCGCGATGAGGTAATTATAGGGTTT

AAGACGTCGGTGGGACCTATTTGCCGACCGGTGAGAAATTCCGACGACGATGGTCACTGA

>MS.gene41061.t1

ATGATTAGCGCTGGGGTAACAGTACCCCCTGGGATCAGCAACGACAGTGATATTGCTATC

GGACTTGGTGCTGAAAGAGATCGAGGATCGGTGCAAATTGGGGTGATTTTGTTGGGGTAT

ATGTTGACTGATAGTGCTTCAGTATCAATGACATTCAAGTTTGGCCCAAATGCTGTAAAA

TTTCCAGCTGGTAGCAGAAATGGTGCCTGGTCTTGGGTGATTCCTCGCCCAATATTGATG

CATAATTTCATGGTCTAA

>MS.gene41049.t1

ATGTCTGACGGTGGAAGAAAACCATTTCCCGTCGTCTACAACGACGGCGAAACCGAAACC

AACCTCGGAATCGTCATGGTGTACCCCACCACAAATATTAAAACTCTTCTCTCCAAACTC

AGCCACAAGATTGGAATCTCACCGCATCAGTTTTCCGTTTTCATTGTCGACCAAAATAGT

GACCGGAAAATCCCTTTCACCGCTAAGGTCAGCATCCCCGCAGTGGTGTGTGACAACGGC

GCGTATGGTTTTTACGTGAAGCGGTGTGGGAGTTATAAGAATGCTTTGGTAAAGAAGAAT

ATGAACTTGTTGGAGAGAAATATGCTTCAATGCCGTGACGGTGGCTCCCTGCCGGTGTCT

GCGATGGAAGCTTTGGTTTTTGAACGTGGTGAAATGGAGAGAAGGATGCTTAGGTTTAAG

ACGGAAAGAGAGGCGTTTTTGATGAGAATGGGAAACAATGTTGAGTCTTTTAGAGTGGAA

CCGACGATGAATGATGGCGGAGGAAGCCGTGGAGGAAGAGGTGTGTATTGTAAGGAGTGT

GTGTTGGCGGAAGTGACAAGGACAAATGCTGGTTTCCATTTGTGTGTTCGTGATGAGGTA

ATTATAGGGTTTAAGACGTCGGTGGGACCTATTTGCCGACCGGTGAGAAATTCCGACGAT

GATGGTCACTGA

>MS.gene41042.t1

ATGGTGAGGATAAGGCCATTTTATACTAGGAGTCAGAGGATAGCAAAATGCTTGTCTTGC

GAGCTATGCAACCAACTTCTGACTGATGCCACCAAAATCATTATATGCTTTCATACTTTT

TGCAAGAAGTGCATTTATACTAAACTAAGTGATGAAGATTTGGATTGCTGTCCAGTATGT

GACGTTCATTTAGGGGTGGATCCTTTGACAAAACTGAGGCCGGACTTCAATTTGCAAGAT

GCTAGAAACCGCTTATTTCCTGTTGGGAAAGAGGAGGAGGCACTGAAATACCTCCCAGCA

GACCTAATGAGAGCAAGTGGGATTGATCCTTATGGGTTAAGGGCTTCAAGTTCAAGTGCT

AAAAGAGGACTAATGGAATCAAAAGACAACAATAGTTTGGAAGGAAAAAACAAGAAAGTT

AAGACTGAGGAGGCTGCTGAGGTTGGAGATACAAGTGCCAACTAG

>MS.gene41050.t1

ATGGAACTGCCAATGAACAACGACCGTAGAAGCGGCGGCGCTCACAGTGGAGGAAGAGAA

AGTGTGTATTGTAAGGAGTGTGTGATTGCAGAAGTGACAAGGATTAAGGCTGATTTCCAT

TTGTGTGTTCGTGATGAGGTAATTATAGGGTTTAAGACGTCGGCGAGACCAATTTTCCGA

CCTGTGAGGAATTCCGGCGAGGATGGTTAA

>MS.gene41058.t1

ATGGCACCTCTGTTGAATTCAAAAACAGCATCCATGATTGATGAACTACTTCAAGTTTTT

CGATTAGGCTTCCGGGAAATTCCTTCTGATGTAAGAGGAATTGTTTACTTTCTCTGCAGC

TTCATCTTTGCCATAATTTTTGTCAATTGTTCTGCCTTCATTTTCTTTTTCATACTAAGT

CCCATCCTACCTCACTACCACCCCACCATCAAACTAAACTCTATCAACGTTACATCGCTT

GATACTCATGTTGTTAATTTAACGGGAACATTTGACATAACCTTTGATTTCAACACTTCC

AAATGCAATGATCATAGTATTATTTCATATCATGACATTGAGGTCAAGGTTTGGTGGTAT

GGAAATAAGGATATTACCCTCGCCACAACCCGGCTGTCTCCTTTTAGTCAAAGAACCCAT

AGTGTGACTGAAGTCGGAGCTATTTTAAAGGTGGCTGATGGATTCAATAACGACAGTGAT

GTGGTGAAGGGGATTGCTTCTGAATTGGCTCGTGGATCGGTTAATTTTGGAGTAAGTTTG

TTTGGTTTGGTTCGGTTTGAGGATGATTTTTCGGAGTCACTAAAATATGTGTGCAATCAA

GTTGTTATCGTGTTTCCACATGGTAGCAACAATGGTATTTGGAAGACTTTAGGGTTTCCT

TGCCCCAAATTGTATCGTAAATTAGTAATTTAG

>MS.gene41063.t1

ATGGCTGACTTCACTCCTACTTCTTCTAATTGCACCCCTACAACTTCTTCTTCTTCAATT

TGCTTCCAACGAACGGTTACATTATATGATTGGTGGCTCGTTAAATCATCCCAAGGAAAC

AACCACTGTTTAGCCATCTCCGGCATCTCTTCCACAAAGGAAGAAGCGGTTTGGGTGTTT

AATTCCGCTCCTATTATCAAAAGATATGATGAGGTTTCCCTTAAGACTGTCGATGCTTGC

TTTAGAGAAGAATCAGAAATCGGTACTGATTCGGGCAATGTTGTTTTGGATAATGTATCT

GCATTTTATCAAGAAATTTTCTCTAACGGTGTGGAAAAGTCCATTCCTACTTCGTCGGTA

TTGCCTGAAGAGGCCCTAGAGAATTGCAAGACTCCATTCCCCGGAGATGAATGCAAAGTC

TTAAAGATGAGTAGGGTTGATGTTGCTTGTGGTAGTGGTAAAAATAGACGTAGTACCAGG

TTGCATAATATTAAAGTCTGTCAGCAGAAGAAGCAGAAGCAGCATCCTGAGTGTAGAGGT

CCTTTCAAGCATCCAGATGGAGAGCCGAGCTCTACTTCAAAGGCCCTGGAAAATCATGAT

TCGGACACTGTTGTTCCGGATAATGTACCAGCAAGTCTTCCAGAAATATCATTTGATGGT

AAAAATTTAGACATGTTTGTAAATGATTTGTAA

>MS.gene41038.t1

ATGAAGGCAGAGGTACTTGGTGTTGATCCAAGAGAAGTTGATGTTCCAGTGGTAGGAGGT

CATGTAGGAGTCACAATATTGCCTCTTTTATCACAGGTTAAGCCTCCCAGTAGCTTCACT

GCAGAAGAAACCGAATACCTGACAAATCGCATTCAAAATGGTGGAACAGAAGTTGTTGAG

GATTGCTTCAAAGAGTCCTAA

>MS.gene41045.t1

ATGTCAGACCACCTTAAAAATTGTTCCTTCACAGGTGGATTTCAGATGCCATCTCGTCCC

CATCTCGAGCTTACAAGACTTGATGTATCTCACAATGCAATAACTGGTCAAATGCCGAGC

AGTAATATCAGTTATATTTTTCCAAATTTGGAATCTCTAGATATGTCTCTAAATGCGATC

CATGGTCCAATTCCTCATGAGCTTTGCCGTATGAGCACATTAAATACATTGGATCTTTCG

AACAATCAATTGTCTGGAGAAATACCATTTAACATATATGGAGTTGGGAAGCAACTCGAA

TCCTTGGCACTTTCAAACAATATGCTTGATGGACCAATACTTCCTATGTTAAGTAATTTG

GCATACTTATCATTGGAAGGAATTGACTTATCCCACAATAAACTAAATGGAACTATCCCG

TGTGAGCTTGGAAACATGACAAGAGTTCGAGCATTAAACTTCTCTAACAATTATTTGACT

GGGAAAATTCCAGTTACATTTTCTAATTTGGTACATGTAGAGAGTTTGGATCTTTCTTTC

AACATGTTGAGTGGTAAAATTCCTCCTCAACTAAGTGAGTTGACCTTCCTTGAAGTATTC

ACTGTGGCGCACAACAATTTATCAGGTGCAACACCCGAAATGAAAGAACAATTTTCCACA

TTTGATGAAAGCAGCTATGAAGGTAATCAATTTCTCTGTGGACCTCCGCTGCCAAAGAGT

TGCAATGACAGTGAACAAATACCTGCTACATTACCACATGGATCAGACACAAATGGAGAA

AATGATGTTGGGTGGATATGTATATTTTTTGTGTGA

>MS.gene41056.t1

ATGGTTAACTCTGAAATTACGGAAACAGTTGCAGCTTCACATTCTGATGCACCTACTCAT

GATGTTACTGCCAATACTTTTAGATTTGCATTGCAAAATGTTTCGGACGTAATTCAACAG

GGTTTGTTGCCTCAACCTGCCTCTCCCATGTTGGAATTATCTATTATAGTTGCTCACGAT

GGCGTGCATTCACTTAGAGTCGAGCAAACACATTCAGATGATCAGGACATGAATGTTCCT

ACTTCTCCAGTTGTTTCTGCTCCGGTTCCATCTATTGTAGAGCACGTTGACGTGTATGAG

GATATTGTTTCGGCCACGGAAGTGAGTGATTCGTCGGTTCAGCACACTATAGCGCAAGAG

GAATTACTTTCTGCCTGTTCTGCATAA

>MS.gene41060.t1

ATGTACTCGGTAAATGCAGCGATTTTTGAGACTATAAGGAGAAGAAATGCGAACGATGGA

GTTTTGGAAATGTCGGCATTGTGGCTTGCACCTCAACTTTGTCTGGCTGGTGTATCTGAA

GCTGTCAATTTCATAGGCCAAAATGAGTCTTATGACACAGAGTTTCCAAGTACTATGTCT

AGTGTTGCGGCTTCGCTTTCTGGATTGGGAATGGCTGCAGGAAGCTTGCTATCATCTTTA

TTGTTCAGCATTGTAGAAAATACTACTTCAAGAGGAGGAAAAGATGGGTGGATTTCTGAT

GATATTAACAAGGGTCATTTTGACAAATACTCTTGGCTTATAGCTGGAGTTAGTGCTCTT

AATATACTTATCTAG

>MS.gene41069.t1

ATGGCCGGAAATCAACTCAACGGCGCTTATTACGGCCCATCAATCCCTCCTCCACAGCAA

CCGAAGCCTCGTCGACACCGCAACGACGACGGTTGCGGCATCTGCAGCTGTCTCTGCGGC

TGCGTTCGTGGCTGTTGCGGCTGCATTTTCAACTGCATCTTAGGCTTAATCTGCAAAATC

ATCACCACAATCATCGTCATCCTCGTGATCCTCGGGTTCCTCTTCTGGCTCATTGTTCGA

CCTAACGTCGTCAAATTCACCGTCAACGATGCAACCCTAACGCAATTCAATTTCACCGAT

AACAACACACTTCACTACGATCTCGCTCTTAACGTCACGGTTCGTAACCCTAATCGAAGA

GTTGGTATTTACTATGACACAATTGAAACGATGGCGTTTTATAAGGATGTTCAGTTTGCG

AATCAAACCCTAGGGAGATTCTTTCAGCATCATAAGAATACGAGTTTTTTGAACCCTGTT

TTCAAAGATAAACAAGTTGTTGTTTTGAATGAAGATCAGAATAAGGAGTTTGATAAGGAG

AAAATTGATGGGGTTTATGGAATTGATTTTAAGATGGTGATTGAAATGAGGTTTAAATAT

TGGTTTTTTAAGATTGGACATGCGAAACCTAAGGTTCGTTGTGATTTGAAGGTGCCTTTG

AAATCACATAACGGTTCTTCTTCTTTGGGGCATGGTTTTGAAGCTACTGAATGTGATTGG

GATTACAAATGGAGATTATGGCATTAG

>MS.gene41068.t1

ATGGACCAAGAAAACCCCAACCCACCATTAGAACAACCCTCAAAACGTAGCACAAGATGT

TGCTTCTGTCTCTCCCGTGTCTTATGGATACTTCTTTGCACAATAATCATCCTAGCTTCC

CTCATCATCCTTGTTATTTACATCGTAATCACACCACGTTCCTTCAAATTCCATGTTAAC

CAAGCCAAACTCACTCAATTCAACTTCACCGACAACAACTCCACCTTAAACTACAACCTA

GTACTCAACTTCACTGCTCAAAACCCTAACAAAAAACTCAAAATCTACTATGATGTCGTT

GAAGCCAACGCTTTCTATAAGGGATACAATTTCTCTGCCACGGATATGAACAGGCCATTG

CGCACACTTCAGGATACTAAAAGTGTGGATTACCGTATGAGTGCTGTTTTTATTGGTCAA

CATGTGATGATGCTTGATCGTGATGAGGTTGATGAGTTCCAAGAAGATTACAAAATTGGG

ATTTTTCGTATTGATATTAAGATTTATTTTAGTATAAGGTTTAGGCTTGGAAATTTTATA

TTTGGTGACACCAAAGCCCAAGCAAAATGTGGTCTAAAGGTTCCTCTTAGTTCTAATAAT

AATAGTGGGAAAATGGTTGATCCATTTGATCCCACCAAGTGTGAGGTGGATTTCTGA

>MS.gene41070.t1

AATGTTCTTGGAGTGCACGTGACAATTGGTAACAATTTAGCTGACAATTTGGACTTAATT

GTTCATTGCAAATCTGCAGATGATGACATTGGAGTACATCTTCTTCATCATAGGGATATT

TTTGGTTGGCATTTTGGAACTAACTTTTTTGGAGAAACAAGATTCTATTGTTCCTTTCAA

TGGAATGATGAGATAAAATGGTTTGATATATACGCAGACCGTAGAGATTTTCAT

>MS.gene41082.t1

TGCAATCTACCTTGCTATTTCCATGGTTTTGCTTTCCTCAGTAGCAATGGCAGCTGATCA

CGTTGTGGGTGATGAAAAGGGTTGGACTGTTGATTTCAACTACACTCAATGGGCTCAAGA

CAAAGTTTTTCGTGTTGGTGACAACCTTGTGTTCAATTATGATAACACCAAGCACAATGT

CTTCAAAGTGGATGGTAAACTCTTTCAAAGCTGCACTTTTCCATCAGAAAATGAAGCACT

TTCCACCGGAAAAGACGTTATTGCACTGAAAACCGAAGGAAGAAAGTGGTATGTTTGTGG

AAAGGCCGATCATTGTGTTGCTCGTCAAATGAAGCTTGTCATCAATGTACTAGCAGAAGG

TGCACCTTCACCTTCTTCGTCTGCTCACTCTATTGTATCATCCATCTTTGGAGTCATCAT

GGTAGCCACAATTGCCATTGCAACCATCTTCTAG

>MS.gene41079.t1

ATGGAAGAAACCAAATCAAAGTTTAAGAGGATTTGTGTCTACTGTGGTAGCAGTTCAGGG

AACAAACCTACTTACCAAGAAGCTGCTGTTGAACTAGCAAAAGAAATGGTTGAGAGAAGG

ATTGATTTGGTTTATGGAGGTGGTAGTGTGGGTCTGATGGGTCTTGTTTCTCAAGCAGTT

CATGATGGCGGACGCCATGTTCTTGGTGTTATCCCTAGGAGTCTCATGCCAAGAGAGATA

ACTGGTGACCCTATTGGGGAAGTGAGAGCTGTATCTGATATGCATCAAAGGAAAGCTGAA

ATGGCTAGGCAGGCTGATGCATTCATTGCCCTTCCTGGTGGATATGGAACCCTTGAAGAA

TTGCTGGAAATTATTACTTGGGCTCAACTTGGGATCCACAGCAAACCGGTGGGTCTATTG

AATGTGGATGGATTTTACAATTCATTGTTGTGTTTCATTGACAAGGCCGTTGATGAAGGC

TTTATTTCCCCAAAGGCACGTCGCATTATCGTGTCAGCATCCACAGCCAAGGAATTGGTT

AGGGGATTAGAGGAACATGTACCTGAGCAAGATGAATATGTATCCAAGTTGGTGTGGGAA

GAAAGACTAAATTACGTGCCCGAATCAGAAGTTGCCATGTGA

>MS.gene41080.t1

ATGGCATGCTCAGCTACTACTACCTCTGCTTCTTCTCTCTTCTCATCTCTAAACCCTAAA

TCCTCACTTTTCTCTCCCAAATTACCCTCTTCTTCTACTCTCTCTATCCCTAATTCTCTT

CCTAAACCATTCTCTCTCCCTTCACTTTCCTTCACTCGCCCCAATCTTCAACATTCTTCT

CGCCGTAGTAGCTTCCTTGTTAAATCCTCTAGTGAGCTTCCATTGGTTGGAAATGCAGCA

CCTGATTTTGAAGCAGAGGCTGTTTTTGATCAGGAGTTTATCAAGGTCAAACTCTCTGAC

TACATTGGGAAGAAGTATGTCATCCTCTTTTTCTACCCATTGGACTTCACCTTTGTTTGC

CCAACAGAAATCACCGCTTTCAGTGACCGACATGCAGAGTTTGAGGCAATAAATACCGAG

ATTTTGGGTGTTTCAGTGGACAGTGTGTTCTCGCATCTTGCATGGGTTCAAACAGATAGA

AAGTCAGGTGGCCTTGGTGACTTGAATTATCCTCTGGTTTCCGATGTCACCAAATCCATA

TCAAAATCTTACGGTGTTCTCATTCCTGATCAGGGAATTGCATTGAGAGGATTGTTCATT

ATTGACAAGGAAGGGGTTATTCAGCATTCTACCATCAACAACTTGGGAATTGGTAGAAGT

GTTGACGAGACAAAGAGAACACTCCAGGCCTTGCAGTATGTGCAGGAGAACCCAGATGAA

GTTTGCCCTGCTGGATGGAAGCCTGGGGAGAAGTCCATGAAACCAGACCCAAAACTTAGC

AAAGATTACTTTGCCGCGGTGTAA

>MS.gene41081.t1

ATGTCCGGTAACGCTGGACCTTCCGACCGTGGCTCGCCGATGCCGACCGGAGCACAGATT

CGGCCGCCGTTGAAGCGTCATCTTGCGTTTGTTTCGACGAAACCACCGTTTGCACCGACT

GATGAGTATCATACTTTCGCTGCTGTTGATTCTAGGAAAGTCGCCGATCATGTCAATGAA

GCCGTTATTGTTAGATCTCCGGTCATAAAGCGGAAGAATGGAATGAATGAAAGTGAAGGA

GACTCGCAAAAGTCAAGTAACAGCCCTGGATACACTAATGTAATGAACAGCCCCTTCAAA

ACTCCTTTGTCTGCAAAAGGGGGAAGGGCAAACAAGTCAAGGGCATCCAAAGAAGGCAAA

TCATGTCCTCCAACACCTATCTCAAATGCTGGTTCCCCTTCTCCGCTCACTCCTGCTGGT

AGCTGTCGTTATGACAGTTCCTTAGGTCTCTTGACAAAAAAGTTCATCCATTTGCTCAAG

CGTGCTGAGGATGGTATTCTTGACTTAAATAAAGCAGCAGAAACTTTGCAGGTGCAAAAG

AGGAGGATATATGACATAACTAATGTTTTGGAAGGCATTGGTCTCATCGAAAAGAATATC

AAGAACAGAATATATTGGAAAGGAATTGAATCTTCTACGCCTGGTAATGTGGATGGTGAT

ATCTCTTTGCTTAAGTCGGAAGTTGACAACCTTTCTCTGGAAGAGCAGAGACTAGATGAT

CAGATAAGGGAAATGCAAGAAAGACTGAGAAGTTTGAGCGAAGATGAAAACAACCAGAAG

TTCCTTTTTGTGACCGAGGAAGATATTAAGGGCCTACCTTGCTTCCAGAATGAAACTTTG

ATAGCAATCAAAGCTCCGCATGGAACCACCTTGGAAGTTCCTGATCCTGAGGAAGCCGTT

GACGAACTACAGCGGAGATATAGAATCATTCTAAGGAGCACAATGGGTCCAATTGATGTC

TACCTTATCAGTCAATTTGAAGAGAAATTTGAAGAGATTAATGGTGCTGAGCCCCCCGCA

AGCTTACCTCTTGCTTCAAGTTCAGGGTCTAATGAGCAACAAGTGACAGAAATGGTACCT

GCTGAATGCAGTGGAAAGGAACTTGAACCTCAAGCTCTGCTCTCTTCTCAGACATATTCT

GATTTAAATGTTTCACAAGAGTTTGCTGGGGGAATGATGAAGATTGTCCCTTCAGATGCT

GATAATGATGCTGATTATTGGCTTCTATCAGATGCTGGAGTTAGCATAACAGATATGTGG

AGAACAGATTCTAATGTTGATTGGAGTGGAGTGGACATGCTTCATCCTGACTTTGAAATG

CTCTCGAGGCCACAAACTCCATCGCCGGGACTTGCGGAAGCACCATCGACTGTAGCTAAC

CCTAATCAGAGATGA

>MS.gene41078.t1

ATGGATTGTACAGTATCATTTCCTAGTGTGCATTTTGTTCCATTGGGAAGAACATTATGC

AGAAACCAAATAATCACTTTAGCAGCAAATTCAAATAGAAGAAGAAAAAATCCAACAAAG

AATCTTCGATATCCACGCCATGATAAGCTACCTCCAGAGTTTGGTGTTAACTTATTCTTG

AAGAAACCTACCACCACAACCACTGAACAAACCCATGATGATAAGGATTTCGATGAAGAG

AGTAATGAGGAGGAACAAAATGTGGATACTGATGTTGTTTGGGAATCCGATGAAATCGAT

GCTATATCGTCACTTTTTCAAGGTAGAATCCCACAAAAACCGGGGAAATTGGATCGAGAA

AGACATCTTCCTCTTCCGTCTCCTCACAAGCTTCGACCACTAGGACTACCTACACCAAAA

AGACTACCAGCTATGGTCTCTACTCGCAGTTCTGTGGATAAGAAATTATATAAAAGCCCG

AGTTTTTTAGTTGGGTTGGCGAGAGAGATTAGCAGGCTTAATCCAGATGAAGATGTGTTG

ACGATTCTTGGGAAAGGTTTATTGGTTTTGGCGAGTCGTGGGCTGTTGGAGGCGTTGATT

AAGGGTTTTTTGAAAGGAGGAAACCTTAGACTTGCTTGGAAGGTAATTGCAGTTGCTACA

AGGGATAAAAGAATGCTGGATCCTAGCATTTATGCAAAATTGATAATGGAACTTGGAAAG

AACCCTGATAGATGCAGACATGTTTTGCCATTGTTAGATGAACTCGGAGCACGAGATGAT

CTGAATTTGAATTCACAGGATTGTACCGCTATAATGAAAGTCTGTGCTAAGATGGGGAAG

TTTGAAGTTATGTACACCACTGTAATTCATAGCCGCTACGCAGAGAAGAAATACAGAGAG

GCATTGGAGTTAGTTTGGGAAATGGAGGCTTCAAATTGCCTCTTTGACTTTACAGCTTAT

CGTATAAAAAAGGTGTTTGTTGCTTTGAACGATCTATCGAGAGCTGTGCGGTATTTTTCA

AAACTTAAAGAAGCAGGATTTTCTCCCACTTTTGGTTTATACAAGGACATGCTTGAAATT

TACATGGCCTCCGGGAGGATAGCAAAGTGCAAAGAGATCTGTAAGGAAGCCGAGATAGCA

GGTTTCACATTACATTAA

>MS.gene41077.t1

ATGTTCTCCTCCTTCTGGCGATCCATCGATCGCTTCTCCCTTCAACACTTCAAATATGTA

ATTAGGGAATTGCAGAAAATCAAAGCTGTGAACGAGCATAATAGGGAATTTGTGGTGGAT

TTGTTGCAGTGTATTGTGGAGATAGTAACATATGGCGATAGACAAGATCCGTTGATTTTT

GAATGTTTCATGGAACACCAAGTGCTTGCGGAGTTTGTTCGCATACTCAAGACCAGTGAA

AATTCAAAAATTGAGGCACCGCTGCTTCAGTATCTGAGCATAATGATTCAAAACATGGAT

AATGAACAAGCAATTTACTATTGCTTCAGCAATGGTTATATTAACAGCATTATTTTGCAT

CCCTACAAGTTCGACGGAGGAGACATAGCTTCGTACTATGTGTCTTTTCTAAGGGCAATT

AGTGGTAAAATAAATAGAGACACACTATGCCTCCTTGTGAAGGTTCATGGGGATGCTGTA

ATCTCCTTTCCATTGTACACTGAGGCTTTAAGATTTGCTCATCACGAGGAAAAGATGATT

CAGACAGCTATACGTTCATTAGTTCTCAATATATACAATGTTAGTGATGACATGGTGTAT

CAGTTTATATTGGCCCATCCAGTTTCCGATTATTTCTCTGACCTAGTTCATAGATTAAGA

GATCTATGCTTCTACTTAGACAGTATTCTCCATGATAAAGGGGAGAAGGAGACTCAAACA

CGAAGAAATGGGCTGATTCTTCAATCTGATAAAATCGTGGATGAACTGTACTACTTTAAA

GATATTCTTAGTGTTGGCAAGCCAAGTTTGACAAGACTGGTGACTGAAAATCTACTGAAT

GGGATTGTGTTTCCTATATTACTCTCATTACTAGCATCAAAGAATAATGATGAGTCAGGT

TTATCTTCTGTCACTTCACTATATATCGTATCCCGCATTCTTCAAGTCGTTGGTGGAAGA

AGCATGATTAACAACGTGGCGTGTTTCATCTTGTATCATTTTTTGAACTTAAATATGAGA

GTTGAAAGTGAAGAGAATACTACTGATGGCCATGATGATGTTAAACCTTTTGCGAAGTGC

TTGACTGAAATTGAAAGAACTATTTGTTATGCTCCAGAGTTAAATGGAGCTGAGAGCATC

GATGAGAACTATTTAGGGTCTCGTTGGGAAGATTTCACTTCTGGTTTCAGCACTGATGAA

TTTTGTTCCAAAAGGAGAGGAGTACTTGACTTTGTTTTCTCCGAGGATCGCAGCCTAGTG

CTAGCATCTATATTTCTGTTGCTAATTTTGGCAGAAAGTAAAGATCTTGATTGTTTCTTA

TCTCCAATGATAGGGCTATATCAGACTGGGGTTGTTATGTTGCAAAATAATGGTGCGTCA

ACTTCTAAGTCTGCGGATGCAAGTATCTTCAGTAGATTTATGCCTGAGATTTTAAACGCT

GTCCTGAAGGTTTTAGCCTTCCAGCAATCCCTCTCTGCAATGATGCTATGGCATGTTGGA

TGGTGCTTACGAAAGTTACTTAGCTTCAACAGAGAGGGACTTGATAGTGATAATCTCCTC

CTGTTTACTACTTCATATGATCAGTCTCGTGCTCACTTTTTGAAAGAACTTGATGGAATT

TGGTTTGATCATATACCCGATGCTCTAAGAACTGAATGGGAGAGCTGTATAAGAGCTCTT

GAACAATCTTCCCAATATATAGATCCATTATTTATGTTCGAGGTTGTTCTTGACCAACAA

TCTACCAATGGAGAAACATCTTCATATTTTGCTTGGCAAAGGATGGATGATGCAATCAAG

GCATTTATTCTACACTTCCAGCTGAAGACGTTCATTTTCAAAAGTGTATTGGTTGAGAAG

CCATTTTTGAACACTATTCCCAGTTCTTCCAATGATTCTGGAGCAAATGGAAATTTAAAT

GTTTCATCTGCTAGCTTTGGGTCCAATGTTTCTTTGGAATGTGGAATGCCATGCCGGATT

GCATTTTCAAATTCTGAAATTAGGGATGTATATGTAATACCAGTGGCATATGGAATGACT

GGAAAGTTGATTCTTGCAGAGAAACACCCTTTCCGCAGTCGACATGGAGTTGTGATTGCG

ATTGCTCCATTGGCTGGGTTATGTCCTAAGATTGATGAAGACCACCCCTCGTGGCTGCTT

CTTCGAATAAGAAATTTTGATCCACAATTTTATACAATTAAAGCCAGAAACAACAACCTA

AGCATGTCAGATCATTTGGCAGATGGGAGATGGACCCTCGGTTTCTCAAATGCAAAGGCA

TGCGAGGAAGCTCAATTGGCAATTCTAAATGAAGTTGCCAAGCAGAGATCAGCCGTGGAG

TACATGCTTGCTCCGTTACTTCAGAATGATCTCGGATTAGCTGTAGACAAAGGTAGTTAA

>MS.gene41102.t1

ATGGATCATAGGGAAATTCATGCTTGTAAGTTTTGCAACCTGACTTTTTCAAGAGTGAAT

GCCTTGAATGGTCACATGAGAGTCCACAGGGAGGAGAAAAATAAGGCCATGAAAGAAATG

ATCACTACTCTCAACCAACATGAGGCGAAGAACCAAAAAGTCACCAATCCAAGTCCAAGT

CAAGGAGGAGAAGAAATTGATTTGGAACTAAGACTTGGTCCAACCAAGGCTACTCCTAGC

ACTAAGTTGGACCTAGACCTCAAGTTATAA

>MS.gene41105.t1

ATGAGTCGAAGAAATGGAAGTGGTCCAAAACTTGACTTGAAACTTAACCTATCACCACCA

AGGGTTGACCACAGAAGAGTTGAGTCATCACCAACAAGATCAGCAACAGTGTCACCTACA

TCCCCACCAAGTTCATGTGTGTCCACTGAGCTAAACCAAGAAGATGGAAACAACCAGCAG

CAGAGATACTCTAACAGCCCAGAAGCCACTTCCATGGTTTTGGTTGGTTGTCCTCGTTGT

CTTATGTTACCCTGTTGCCAGTTCTGCATTCTTCTGCTAGTAGGACATGTTAGCTTTTAA

>MS.gene41104.t1

ATGTCATTGTATATCGGTAAACTGTCAGAACGTGTTCGGAGAGATGAACTTGAGCGAGTT

TTTGGTAGGTTTGGGCGTTGTAATGTTCAGGTGAAAAAAGAAGGTTATGGTTTTGTTGTA

TTTGATTTCCGTTCAGATGCGGAGAAAGCTTTGAGAGAATTGAAAGGTAGAAAGATATGT

GGGGAGTTGTTGACTTTGATGTGGTCTAATCAACAGCCGGAGTCAAATTTTGCAAAATTT

GATAGGGGTGGTGAAAGAAAAAGAAATACCTATGAGTTGCAAGGTAGGAGGATTTTTGAC

AGATTGGGCGAAAGAGAAAGGAAAGAAGGTTTTGATGGATGGGGGAAATGGAATCCCAAT

ACGGATATTGTTGAAAGGGGTAAGTCTGTTGAAGAGGGGGAATATCGTCAAGATAGTTTT

AAGTACTGCGATGTAGAAGAAAAAGATCGCCGAGGAGACTTTCCAGATGAAGGTGGCTAT

GTTGTTCCTAACCAGGATCACAATGGTAGAGGGGCCGAGCCAACTCATGACAAGGCAACT

GACTGTGGGAATGCGAATACTTTGGAATTTGATCGCTACAAACTTTCCTGTGGCTATGAC

AGAAAGGATGATAAAGAAGATTACTCTAGTGGTTCTCTTTTAGCAAATTCCCAAGAGAAT

GTGGGCAGAGCACGGATTGGTGAGGAAACCTTGAATCGTCCAAACAGTTCAAAATTAAAG

CATACTGGTTATAGACGTGGAGAAGCAGGTCATAAGATGCAAAATTGTAGGAAAGACCAC

TCTTTGCGGAGAAAATATAACAGATTGGATGATGAGCATGCTGATAAAATAGACAAAAAG

CGTAGAGTTGAAGATGACATTGAGTCTGGATGGGGTTCTTGGGTGAATCCGCAGTCAAAT

GGAGATGCTTTATTGATGAGCCACCAAAGAGATAAGTGGAGGGTGTCTGGTTCAAGGAAT

GACTGTGCGCCATTGAGAAATGAATCATCCCCGGTAGCAAAGGACACTGAGGAGTATGAA

GGAAAAAAGCAAAGCAGAAATGAAACAGAATCACCTAAAAGATCCAGGGAGAAGAAGATA

TCAAGGCGATCAGTCTCATCCTCTTTGCTATCAGATCACTCTGCATTTCGCTCACTTACA

CATTCTCAATCATCCAAATCACTGCCTAGGTCTACTTCATATTCTAGATCAAGATCAGTG

TCTTCTAGATCACATTCTTCATCTTCAAAGTTAGCATCTTCCTCAAAATCTCAGAATTGT

AGAGGAAAAAAATTACATTCGATGAGGTCGAGCTCCCCTACTTTGTCTGTATCACTCAAT

CAGTCTTTACCGTCTTCTCCAAATAAAATCCAGTTTAATTTAAAAAGCTCATCCATTAAT

GGCGCTGCGCTTAAGTCTGTGGATCATTTGGTTGCTCGGGGACAAGGGATTGGGAGTACA

ATGGAATTGGAGAATTTACAATCCAAGGACTCAGATATTACTGTAAATGGACAAGCTGCA

GTGTCCACTACAGCGGTGGATGCCAAGGAAAAGGACCAGCACGTGCAGGAGGACAATAAT

GAGAATTTAACCAAACCAGTTATTGCAGAGAAATTGTCTCCCAGAAGGGTAAAATGGGAA

GAGGGCTTTCAGCATCCTAGAACACTAATAGCGGATGATATTTCAACTGAAGTTCAGAAG

CCAACTCTGGAAACTCTTATTACTCCCCGTTCTGGCTGTTCAACCATCATATCTACAGAG

GAAATGTGCATGGTTCTGAATAAAAGCGGCCTTGAACTTCCAGATGGCCAGGACATAAAA

TTAACAACAGATGACTTCTTTGGTGCTGCTCGGTTGTGGCCTTGGTATATTATTTATTAC

CGTAGGTTGAAGAAGGGCCCAATTTCAATTGAGAACTATGCTAGGCGGGTTGCTCAGAAT

CAGGAATTTGGCATTGTCGATAAGTACATAAGAAGTAGCAGTGGATGGGGAGAATTCAGT

CCCGAGAACTCCTGA

>MS.gene41106.t1

ATGGAATATACCAACTGGTCTCCATTTCTCAAGGAAAACTGCGAACGATATCTTGCTAGG

AAGAAAGAAGAGGACAGAAAGTATTTATCTGAAATTCTTAGTCCTGCTGTCAAGACCAAC

ATTGCTGAGGAGCGTCCTGTGACATCCGGCACGCTTTTTGTGATGTCTGACCCATTTCTT

GCTGAAAGAGTTGCTAAGAGGCTAGAGGAAGAGCCTCTTGAGACGACCAATCCGGTTTGC

AACCCTTTTCCTGCTAAGAAAGAAAAGGAAAGTAAGTTCGTAATGACCGAACCGCTTGTT

GCTGAGAAGCGTCCTGTGATGTCTGACCCGTTTCTTTTTGAGAAGCCTCCTCACATGTTT

CTAAAGATATTTTTTGCTTATCAGCAAAAAGTGGAAAAACTTGCTGAGATGCTAAAAATA

GAGAGCATAGAGGGAGAGCCTCAAGTCACGACAGACCCTCTTGTTGCTAAGAAGCATCAT

GTGTCGACCATGTTTCTAGACCTTTTTATTGCTTATAAACAAGAAGTGGAAAGAGTTGCT

AAGATGCTAAAAAAAGAGAGCATAGATGGAGAGCCTTTTTTGACGACTGACCCTATTCTT

GCTGAGAAGCCTCCCGTTCTTGCTTATCAGCAGGAAGTGGAAAGTTCTAAGAAGAAAGAA

GAGGAAAGTAATACAACCAACATTGCCGAGAAGCATACTTTGACATACTTTGACGACAAA

CCTGTTTCACAACCTTCATCTTCAAGTGATACAGCTACATGA

>MS.gene41101.t1

ATGTTCACACAAAGCATGTTCATCGCAAAACCCTACGCTCCATCAATCTCAATCCGACCG

GTGTGGTCATTCAATCTCGAATCAGAGTTCAAACTCATCCGCTCTTTCGTTGATTCACAT

CCGATCATCTCAATGGACACCGAATTTCCCGGCGTTGTCGTCCGCCCTGACGGCATCACC

GACTTATCATCTTACCACCGAACACCTGCCACTCATTACTCCGTCCTCAAGGCGAATGTC

GACGGTCTTAATCTCATCCAAGTTGGTCTCACTTTATCAGATGCCGAGGGTAACCTTCCG

AAATTAGAAAAGGAGTTTCTAATTTGGGAATTTAATTTCAGTGATTTTGATGTTGCACGT

GATAATCACGCGCACGAGTCAATTGAATTGCTGAAGAGTCAGGGGATTGATTTCGAGAAG

AATAAGGAATTTGGGATTGACTCATGGAAATTTGCTGAATTGATGATGAGTTCTGGACTT

GTGTGTAATGAGGAGGTTAGCTGGGTGACATTTCATAGCGGATATGATTTCGGATATTTG

GTGAAAGCGTTGACGCAGAGAGCTTTGCCTGATGAGCTTGCTGAGTTTCTGGTGCTGGTT

AGGGTTTTCTTTGGGGAGAGTGTTTATGATGTGAAGCATTTGGTGAAGTTTTGTGAAGGG

TTGTATGGTGGTTTGGATAGAGTTGGGAAGACGCTGAATGTGGATCGTGTTGCTGGGAAG

AGTCATCAGGCTGGGTCTGATAGTTTGCTGACTCTTCATGCTTTTAGGAAGATTAAGGAA

GTTCACTTTGGGAATGATGTTGATCGATTGATGAAGTATGCTGGTGTGTTGTATGGATTA

GAAAATGTGGTTTAA

>MS.gene41100.t1

ATGTTTTCACAAAATATGTTCTTTGCAAAACCCTCCACTTCATCAATCTCAATCCGACCG

GTGTGGTCATTCAATCTCGAGTCAGAGTTCAAACTCATCCGGTCATTCGTGGATTCACAT

CCGATCATTTCAATGGACACCGAGTTTCCCGGCGTCATCATCCTCCCTGACGGCATCACT

GACTTATCATCTTACCACCGAACCCCTGCTGCCCACTACTCTGTCCTCAGGGCGAATGTT

GATAATCTTAATATCATCCAAGTCGGTCTGACTTTATCTGATGTTGATGGTAATCTTCCG

AAATTGAGAAATGGAAACTCTGAGGAGTCTCTGATTTGGGAATTCAATTTCAGTGATTTT

GACTTAGAGCGTGATATTCACGCGCACGAGTCTATTGAATTGCTGAAGAGTCAGGGGATT

GATTTTGAGAAAAACAAGGACTTTGGGATTGACTCCAGGAGATTTGCTGAATTGATGATG

AGTTCCGGACTTGTGTGTAATGAGGAGGTTAGCTGGGTGACATTTCATAGCGGTTATGAT

TTCGGATATTTGGTGAAGGCGTTGACTCAATATGCTTTGCCTGAAGAGCTTGCTGAGTTT

CTGGTGCTGGTTAGGGTTTTCTTTGGGGAGAGTGTTTATGATGTGAAGCATTTGGCAAAG

TTTTGTGAGGGGTTGTATGGTGGTTTGGATAGGGTTGGGAAGATGCTGAATGTGGATCGG

GTTGTTGGGAAGACTCATGAGGCGGGTTCTGATAGTTTGCTGACTCTTCATGCTTTTAGG

AAGATCAAGGAAGTTTACTTTGGGAATGATGATGGTCAACTGATGAAGTATGCTGGTGTG

TTGTATGGATTAGAAAATGTGGTTTGA

>MS.gene41103.t1

ATGACGCTTGGCTCAGCCGGATCCAGTGTCGTCGTTCCAAGGAACTTCAGATTGCTGGAG

GAGCTTGAACGAGGTGAAAAAGGTATTGGTGATGGCACTGTTAGCTATGGAATGGATGAT

GGTGATGACATTTACATGCGCTCTTGGACTGGCACCATCATTGGCCCCCATAATACTGTA

CATGAAGGCAGAATATATCAATTGAAGCTGTTTTGTGACAAAGATTACCCAGAAAAGCCG

CCAAGTGTTCGGTTTCATTCACGAATCAATATGACTTGTGTTAACCATGAAACTGGCGTG

GTTGAACCAAAGAAATTTGGGCTTCTTGCAAATTGGCAAAGAGAGTACAGCATGGAGGAT

ATACTGACCCAGCTGAAGAAAGAAATGGCAGCCCCTCATAACCGGAAGCTTGTCCAGCCT

CCAGAAGGAACTTACTTTTAG

>MS.gene45440.t1

ATGACTATGTCTATATATGTTCCATTGCAACAAGAAGATCAAGATGATGATACAAATCAA

AAAATCAACAAGGAAGTTCAATATTCAGGTGGTGAAATGGTTTCTCAACCACAAAGACGC

AAGGGTGGCCTTATCACTATGCCTTTCATCATTGCAAATGAGGCACTTGGCAATACTGCA

AGCTTAGGAATTTTGCCCAACATGATATTGTATTTGATGGGACCTTTCAAGCTTCATCTT

GGAGAAGCTAATCAAATACTCCTCTTATCTGCTGCAGCCAGCAAGTTCATGCCTGTGGTT

GGTGCTTTTGTTGCAGATTCTTATCTCGGTCGATTCTTAAGTGTTGGATTAGGTTCTGCT

GTCAGTTTCCTGGGAATGGCATTGTTGTGGTTAACAGCAATGATCAAACCAACGGAAGGC

GATCAATCACCAACATCATGGGAAATGGCAATGTTAATATCTGCCTTTAGTCTCATGTCA

ATTGCAGGTGCTGGTGTTTCATGTTCCATGGCATTTGGTGCAGACCAAGTAAATATAAAA

GATAACCCTAATAACAATAGGGTCTTGGAAATGTTTTTCAACTGGTATTATGCTTTTGCA

AGTATCTCTGCGATAATAGCTCTCACTGTAATAGTATATATCCAAGATCATTTTGGTTGG

AAAATTGGTTTTGGAGTTCCAACAGCACTCATGTTTTTATCCACACTGTTATTCTTTCTT

GCTTCTCCTCTTTATGTAAAGATTCAAAAAAGAACCAACTTGTTCGCTAGTTTTGCGCAA

GTAATCGTTGCTTCCTATAACAACAGAAAACTTCCATTACCGCCTAAAAATTCACCTCAA

TTTTACCATCACAACAAGGACTCAAATCTCGTGGTTCCAACTGATAAACTAAGGTTTCTG

AACAAAGCTTGTGTTATTAAGGAATTTGAACAAGATATAGCATGTGATGGTTCAAGAATA

AACCCCTGGAATCTATGCACAGTAGATCAAGTAGAAGAATTAAAAGCCATTGTGAGAGTT

ATTCCATTATGGTCTAGTGGGATCATGATGACCCTTAGCATTGGAGGCTCATTTTCATTG

CTTCAAGCTAAAACCTTGAACAGACATATCACCTCCAACTTTGAAGTTCCAGCGGGATCT

TTGAGCGTTATCAATATAGGTACGGTAATTATATGGATAGTTTTCTATGACCGCGTTCTT

ATTCCTTTAGCATCGAAGATAAGAGGGAAACCAGTTACGATCAGTGCAAAGAAAAGAATG

GGAATTGGTTTGTTTTTATCTTTTCTCTACTCGGTAAATGCAGCGATTTTTGAGACTATA

AGGAGAAGAAATGCGAACGATGGAGTTTTGGAAATGTCGGCATTGTGGCTTGCACCTCAA

CTTTGTCTAGCTGGTATATCTGAAGCAGTCAATTTCATAGGCCAAAATGAGTTTTATTAT

ACAGAGTTTCCAAGTACTATGTCTAGTGTTGCTGCTTCGCTTTCTGGATTGGGAATGGCT

GCAGGAAGCTTGGTATCATCTTTACTGTTCAGCATTGTAGAAAACACTACTTCAAGAGGA

GGAAAAGATGGGTGGATTTCTGATGATATTAACAAGGGTCATTTTGACAAATACTCTTGG

CTTATAACTGGAGTTAGTGCCCTTAATATACTCTATTATTTAGTTTGCAGTTGGGCTTAT

GGACCTGCAGTTGAAGAGTTAAACTTGAATCTCATCCCCAAAGGTGACTCAAAGGATGAA

GATACTCAAACATATTTATACACTCAACAGCCCGGGAATCTGAGCGATGTAGGACTAGCA

ACAAACTACAAACATCTTCAACAATCTCAAGCAGGTGAGCCGCCCTCGAGAAGGGGTCTA

GCCGTTGGACAAGGAATTGCACACAAAGGAGTCATGTCAGAGATGAAATCGCGCAAGTGT

CGTACACATGGAATGGCAACTTGA

>MS.gene45443.t1

ATGTCGGTGAAGGAGTGTGATCATCACAAAGGAAGAAAAAGAATACTATTCCAAAGAATA

TTTTGGGCAATCATGGTATTCCTCTTCTTAGTCCTACTAACAATTCTCCTCATATGGGCA

ATTCTAAGACCAACAAAACCTACCTTTATCCTCTTAGATGTAACCCTATATGCTTTCAAC

GCATCACAACCAAATTTCTTAACCTCCAACTTCCAAGTCACACTTTCATCTCGAAATCCC

AACGATCACATCGGTGTATACTACGATCGTCTCGATACCTACATGACCTACCGGAGCCAA

CAGATTACTTACCGGACCGCCATACCGCCTTCTTACCAAGGTCACAAAGAGTACGACGTT

TGGTCGCCGTTTGTTTATGGCAATGATATACCTGTTGCTCCTTTTAACTCTGTGAGTCTT

TCACAAGATGAAAATAACGGTAATATGTTTGTTATCGTTAAAGTTGACGGTAGAGTTCGT

TGGAAGGTTGGTGCTTTTATTTCTGGTCGTTATCATATACATGTTCGTTGCCCTGCTTAT

ATCTCACTTGGTAACAATGGTAGAGGTGTTGATATGGGAGAAAACGCCGTTAAGTATCAA

GTGAATCAACGTTGCTCCGTTAACGTTTGA

>MS.gene45441.t1

ATGGCTGACTTCACTCCTACTTCTTCTAATTGCACCCCTACAACTTCTTCTTCTTCTTCT

TCTTGCTTCCAACGAACGGTTACATTATACTATTGGTGGCTCGTTAAATCATCCCAAGGA

AACAACCACTGTTTAGCCATCTCCGGCATCTCTTCCACAAAGGAAGAAGCGGTTCGGGTA

TTTAATTCCGCTCCTATTATCAAAAGATATGATGAGGTTTCCCTTGAGACTGTCGATGGT

ATATATATTTTCAAGAGTTTTTTATTTGGCTTTCCTGAAAACTGGGAAACCTCTGTTCTA

GCTTGCTTTAGAGAAGAATCAAAAATCGGTACTGATTCGGGCAATGCTGTTTTGGATAAT

GTATCTGCATTTTATCAAGAAATTTTCTCTAACGGTGTGGAAAAGTCCATTCCTACTTCG

TCGGTATTTCCTGAAGAGGCCCTAGAGAATTGCAAGACTCCATTCCCCGGAGATGAATGT

AAAGTCTTAAAGATGAGTAGGGTTGATGTTGCTTGTGGTAGTGGTAAAAATAGACGTAGT

ACCAGGTTGCATAATATTAAAGTCTGTCAGCAGAAGAAGCAGAAGCAGCATCCTGAGTGT

AGAGGTCCTTTCAAGCATCCAGATGGAGAGCCGAGCTCTACTTCAAAGGCCATGGAAAAT

CATGATTCGGACACTGTTGTTCCGGATAATGTACCAGCAAGTCTTCCAGAAATATCATTT

GATGGTAAAAATTTAGACATGTTTTCGGAAAAGGCCACGAGAGATTGCAATAAGTCGTTC

CTTGAAGTTGAACAAGATATGTCAATTAAGATGAGTAAGGTCAATGATGTTCATGGTAGT

GGCCGGACTAGACGTAGTGGCCGGTTGCATAATGCTAAGGACTGCCAGAAGAAGCAGCCT

GCGTCTGGAGATCCTCTCAAGAATCCAGAGAAAGAGCAGAGCTCTACCTTGAAGGTTGTG

GAAGATCATGATTTGGATACTGCTGTTCCTGATAATGTATCAACAAATCTTCTAGAAATA

TTATCTGAAGCTGTGGAGAAGTCTTTTCCTACTTCTTTGGTATCACCGGATAAGACCACG

AGAGATCGCAACAAGGCACTTCTTGAAGATGAACGGGACATGTCAATTAAGACGTGTAGG

GTCAATATTGTTCATGGTAGTAGCGGGAATAGACGTAGTGCCCGGATGCATAATGTTATA

CCCTACCAGAGGAAGCAACCTGCTACTGGAGATCCTGCAACACATCCAGATAAAGACCAG

ATCTCTGCCTTAGCAGCCTTGGAATTGAGTGATGGAGGACTGGAAAGTCTATCGACACCG

GTTCAGTCAAAAAAGGGAAGAGTGAACACAATGTCTGGGCAGGTCACTAATAAATTGAGT

TCCAGAAATTCCAAAACTTTTTCAGCAAAAACTGAAGGATGTTATAAGAAAAAAGGAAAA

ATGATTAAACATGCATCTTCTGTAAAATCTCCTCGAGGCAGGGATTTAAGTCACTCGAAC

AAGGGAAGTCAACAGAGATTATCCACAGTTTCTCCAGAGTCATTGAGTCTCAAAATATTC

AGATCTGGAAGGTGGCTTCTACCTCGCTTAGAGTTTTGGCGCAATCAAACACCTATTTAT

AATATGGACCGTGAGATTACAGAAATTCAGGAAGGCTCGTCTTTGATATCACCTTTTAGA

GGCTCTTCGTCATAG

>MS.gene45442.t1

ATGAAGGTGACACAGGGTCAACTCGAAGGATTAGCCAAGGCTCATGAAGAAACTCAGAAA

ATGAATGAGGCTGCTATTAAGAACTTGGAGATGATGGAAGTAGTTAAGGAAGTAGCAGCA

GCTGAAAAGGGATTGAAGGGAGCAAATTCTCTTAAAGGCGAAATCAAGGAGGAAGAAAAA

TTGCAGCAACTAGTTAAGCTTCTGGCACAATTTAAGGTAAATTTTCCAATCAGAGAAATG

ATGAAGAAGAGCCCGTATTGCATAAAATTTATGACATACCTCTTACTACCAAATGGTAAA

CCATTAGAAGATGCAATGATTGTTGTTACCAAAGGGTGTAGTACGGTTTTTGAAGACAAG

CCTCGAAAGAAGAAGGAAGATCCAGGACGTTTTAACATACCTGTTACCATCGAGGATTTT

TTATTTAACATAAGTGATATGTGA

>MS.gene45444.t1

ATGGCAGAAGCAAAACCCCACAACCGCGGCAGAAGATGTTGTTGCTGCCTTTTCGGCATA

ATATGGAAACTCCTCGTAGCAATCATCGTTCTCGTTGGTCTCATAATCCTTATATTCTGG

CTCGTTGTCCAACCTCAAACCTTCAAATTCAGTGTCAAAGAAGCAAAACTCACCAAATTC

AACTACAACGACGACACCAACACCCTCCATTACAACCTCGTACTCAACTTCACGGCCCGT

AATCCAAACAAAAAACTCAACATCTACTACGATGTAATCGATGGTCATGCGTCTTACGAA

GGGACTAGGTTTGCTTCAACAAAGGTTGTAACATGGTTAAATTCATTCCGACAATACACT

AAGAGCTCGAATCCTATGAGTGGTGTTTTCTCTGGGCAACGTGTTGTGGTTTATGATCGT

GATCAGGTTTCTGACTTTGAACGTGATAAGAAAGACGGAGTTTTTCATATTAATGTGAAA

TTATACTTTGATATGAGGTTTAGACTTGGTGATTATATTGGTCCACATACTAAGGGTAAC

ATTAAGTGTAGGCTTGATGTTCCTTTTGTTGCTAATGGAACTAAGGTCATGAAAGCATTT

GAACCCACCACGTGTGATGTAAATTTCTAA

>MS.gene46320.t1

CTCCTCGAATTGCTGCCTTTCGAATGGTTCCACAAGTGTAGGGAGAAAAGTGTCAGACTT

GCATTGCCTACTATTTAGTAAATGGGGCAGTTCAAGAAAGGGATGCCTTATTCAACATGA

TTTGTCCTCGAGCAGTGGTCAAGGTCCTGTGAGCTGTAGAAAACACTATTTTACATTTTC

TAAGACGGGTAGGAGTGTAAATTTGCTTCCATTTGCTACCTCTGATGATGGTATGACTGT

CAACGGGAGTCCACAAGCTGACGCCAGTGCTAACCTTGAAAAAATGAGGGTGAAATTGAA

TAGCTCTCTTGAAGATGAAGACTTTTATGATGGACTTGTTCAAGCTTTATATGATGCAGC

CAGGGTTTTTGAGCTAGCAATTAAAGACCATAAATCGTCATCACGTGGATCCTGGTTTTC

AACAGCTTGGCTTGGGGTAGACCAAACCGCATGGGTGAAGGCATTGTCATGTCAGGCTGC

CGTGTACTCCTTATTATATGCTGCAAGTGAAATTTCATCCCGAGATGATAGCAGAGATAG

AAATGTCAATGTATTTGTTCAAAGGAGTTTGCTAAGGCTATCTGCTCCCTTGGAGAGCTT

AATCAGAGAAAACTTATCTGCCAAACAGCCTGAAGTGTACGAATGGTTTTGGTCTGAGCA

AGTTCCAGCTGTAGTGACGTCCTTTGTTAATAAGTTTGAAGGGGACGGGCGCTTCACTTC

TGCCATTGCTTTGTCTGAAAAAACTAAGGGTTTGAGCAGTGCAAGTGATGTATCACTTCT

CCTGCTTGCACTTACATGCATTGCTGCAATTGCTAAACTTGGCCCGGCAAAAGTTTCTTG

CTCACAATTCTTTTCCATGAGCACTGAGATAACTGGTAGTTTGATGGACATGCTGGTTGG

TTTAATTCCTGTAAGTCAAGCTTATAATTCTATAAAGGATGTTGGTCTGCACAGAGAATT

TCTTGTACATTTTGGTCCTCGAGCTGCAGCTTTTAGAGCAAACGTTGAGTGGGGTTCAGA

AGAGGTTATTTTCTGGGTAAATCTGGTTCAGAAGCAGTTGCAGCAGGCTATTGATAAGGA

GAAAATATGGTCAAGACTGACAACATCTGAAAGCATTGAGGTTTTGGAGAAGGATTTGGC

TATATTTGGATTCTTTATTGCTTTAGGAAGAAGTACACGAACATTTCTTTTGGCAAATGG

TTTCGATGGTCCCGATGATCCAATTGAAGATTTCATCAGGTATCTTATTGGGGGAAGTGT

TTTATACTACCCACAACTCTCATCCATTAGTTCATATCAATTGTATGTTGAGGTAGTTTG

TGAAGAGCTGGATTGGCTTCCTTTTTATCCGGGAATCACCAGCATCACAAAACAGTCTCA

TGGGCATAGTAAACCAGAAGGTCCTCCAAATGCAGAAGCAGTGACCCAAGCATTTGCTGT

TTGCTCTTATTGGATGCAGAGCTTTATTAAATACAGCACATGGCTTGAGAGTCCTTCAAA

TGTAAAAGCAGCTGAATTTCTGTCCAGAGGGCACAACAAGTTGATGGAGTGCATGGATGA

ACTTGGGATGATAAAAGATAAGACATTGGAGACTGATGCCAAGAAAATAGTTGACGGGCA

AAGATCCACAATTCAGTCAACGATAAAGACTCGGGCATTAAAAAGTGTCGAAGAAGCTGT

GATAAAGCTTGAAAAGTTGCTTCAAGAATTGCATGTGTCAAGCTCTAGTTCTGGAAAGGA

GCATTTGAAAGCAGCCTGTTCTGACTTGGAAAAAATACGGAAACTTAAGAAAGAAGCTGA

ATTCCTGGAGGCATCTTTCCGAGCAAAAGCTGATTCTCTGCAAGAGGGAGTTAATAGTGA

TCAAACCATCACACCGGTTGGTGAGGAGGACAGGTTTATGAAAGGGAAAAGCAGAAAGAA

CGACAATGTAAAGGTGGACAGGAGCAAAAGACAAATTGGAAAATCTCGTGGATTCTGGAG

CATATTTGTACCTCCTATACCCAGAAAGCCCGACCCGGAACCTGATGTGGATGCTCATGA

AAATTATATTGAACAGCCTGCACCAAATGTAGGGGTTGTGGACCAAGAACCCAATGAAAT

CCTTCGCTTTGAGCTTCTAAGAAATGAGCTGATGGAACTTGAGAAAAGGGTCCAAAGAAG

TGCCTATCAGTCAGAAAATAATGAGGATTTGATGATCAGTGATGATGGTGCCCGTTATAA

CGGTGATGCTGGAGGTGTTCAGATGGTCAGAGTTCAGAAGAATGAAAATTTCATACAGAA

ATCTTTTGACAAACTAAAAGAAACGGGAACGGATGTCTTGCAAGGAACTCAACTTCTTGC

TATTGATGTTGGTGCTGCCTCGGGTTTGGTCAAAAGGACCTTGATAGGTGATGAATTGAC

TGAGAAGGAGAAGAAAGCACTTAAAAGAACCTTAACTGACATGGCTTCAGTTGTTCCTAT

CGGCATTTTAATGCTTCTTCCT

>MS.gene46321.t1

TTTGCTAAGGCTATCTGCTCCCTTGGAGAGCTTAATCAGAGAAAACTTATCTGCCAAACA

GCCTGAAGTGTACGAATGGTTTTGGTCTGAGCAAGTTCCAGCTGTAGTGACGTCCTTTGT

TAATAAGTTTGAAGGGGACGGGCGCTTCACTTCTGCCATTGCTTTGTCTGAAAAAACTAA

GGGTTTGAGCAGTGCAAGTGATGTATCACTTCTCCTGCTTGCACTTACATGCATTGCTGC

AATTGCTAAACTTGGCCCGGCAAAAGTTTCTTGCTCACAATTCTTTTCCATGAGCACTGA

GATAACTGGTAGTTTGATGGACATGCTGGTTGGTTTAATTCCTGTAAGTCAAGCTTATAA

TTCTATAAAGGATGTTGGTCTGCACAGAGAATTTCTTGTACATTTTGGTCCTCGAGCTGC

AGCTTTTAGAGCAAACGTTGAGTGGGGTTCAGAAGAGGTTATTTTCTGGGTAAATCTGGT

TCAGAAGCAGTTGCAGCAGGCTATTGATAAGGAGAAAATATGGTCAAGACTGACAACATC

TGAAAGCATTGAGGTTTTGGAGAAGGATTTGGCTATATTTGGATTCTTTATTGCTTTAGG

AAGAAGTACACGAACATTTCTTTTGGCAAATGGTTTCGATGGTCCCGATGATCCAATTGA

AGATTTCATCAG

>MS.gene46443.t1

ATGTTATTAGTCGAACCAACTTCTTGGTCTCTCCATCACCACCAACCTCCATGGTTCAAT

TCTCAAATCCATGAAAACTCACCTCATTACAACCTCAAAAACATCGAAGATGAAGAATTA

GACACAACCACCACCGTAATAAAACCCGAAGACGAAAAGGAATCAATGTTTGAGAAACCC

TTAACACCTTCTGACGTAGGAAAACTCAACCGTCTTGTTATCCCAAAACAATACGCTGAA

AAATACTTTCCACTCGGCGGAGCAGACTCCGCCGAGTGCAAAGGACTTTTACTAAGTTTT

GAAGATGAGTCAGGGAAGTGTTGGCGTTTCCGTTACTCATATTGGAACAGTAGTCAAAGC

TATGTTCTAACAAAAGGTTGGAGTCGTTATGTTAAAGACAAACGACTCGATGCTGGCGAT

GTCGTTCTGTTTGAACGACATCGTGTTGATAGTCAGAGACTTTTTATCAACTGGAGGAGG

AGGAGTGGGGATGATCAGTCGGCGTATGTTAGCAGAGGGGTGGGACATCATGGTAAGGGT

AGTGATGGGAATGGTAAGAATGAGGTTGTTAATGGTGTAGGGTGGTCCAGAGGGTTCTAT

TCTGCGCATGTTCCTTATCCTGCGCATCATCATCAAGTCTTGCCATACCAACATGATCAA

TGTCTTCATGCAGGTCTTAATATCTCTTCAATGCTATCTCATCTTATTTTTTGCATTCAC

ATTCTAATCAAACAA

>MS.gene46444.t1

ATTCAATGCCAGTGACCTCCTAGAGAGGAGTCGCAATGGACGCATTGTGTTTGCAGGTGA

TTCAGTGGGCAGAAACCAATGGGAGTCATTACTTTGCATGTTGTCAAAAGGGGTCTCTAA

CCTCTCAAAAATATATGAAGTTAATGGAAGTCCCATAAGCAAACACAAGGGCTACTTAGT

GATGAAATTTGAGGATTACAACATGACTGTAGAATATTATAGGGCACCTTTCTTATCTAT

TATAGGTCATCCACCACAAAACTCATCTAGTGACATTAAAATGACTATCAGACTTGATGA

GTTACATTGGTATTCTAATAACTGGAAGGGAGCAAATGTTCTTGTTTTCAATAATGGTCA

TTGGTGGAACTTAGACAAAACTATCAAGTCGGGTATCTATTTCCAGGAAGGTGGGAAGGT

AAATGTGACTATGACAGTGAAAGAAGCTTTTAGGAGATCCTTACAGACATGGAAATCATG

GGCATTGAGTAATCTAAATCCTATGAGGAGTTTCGTCTTCTTTCGTAGCTATGCCCCTGT

TCATTACAGGAATGGCACATGGAATGAAGGAGGAAACTGTGACAAAGACAAGGAACCAGA

AAACGATCCTGCAAAACTCGAAGCTGATCCGTATTACAACGTATTTATATCTGATGTTGT

AAAAGAGATGCAATATGGAAGCTGGAAAGTCAACTTTTTGAACATCACATATCTTTCGGA

ATTGAGGAAAGACGGTCACCCTTCCAAATATCGGGAACCAGGAACCCCACCCGATGCTCC

TCAGGATTGTAGCCACTGGTGTTTACCCGGAGTGCCAGACACGTGGAATGAACTTATCTA

TGCCCAACTCCTCTCTAAGAAATATGGCATCAACAAAAGTTTTCCAGAAAGCGAAGAACG

CAGCTGA

>MS.gene46445.t1

ATTCAATGCCAGTGACCTCCTAGAGAGGAGTCGCAATGGACGCATTGTGTTTGCAGGTGA

TTCAGTGGGCAGAAACCAATGGGAGTCATTACTTTGCATGTTGTCAAAAGGGGTCTCTAA

CCTCTCAAAAATATATGAAGATTACAACATGACTGTAGAATATTATAGGGCACCTTTCTT

ATCTATTATAGGTCATCCACCACAAAACTCATCTAGTGACATTAAAATGACTATCAGACT

TGATGAGTTACATTGGTATTCTAATAACTGGAAGGGAGCAAATGTTCTTGTTTTCAATAA

TGGTCATTGGTGGAACTTAGACAAAACTATCAAGTCGGGTATCTATTTCCAGGAAGGTGG

GAAGGTAAATGTGACTATGACAGTGAAAGAAGCTTTTAGGAGATCCTTACAGACATGGAA

ATCATGGGCATTGAGTAATCTAAATCCTATGAGGAGTTTCGTCTTCTTTCGTAGCTATGC

CCCTGTTCATTACAGGAATGGCACATGGAATGAAGGAGGAAACTGTGACAAAGACAAGGA

ACCAGAAAACGATCCTGCAAAACTCGAAGCTGATCCGTATTACAACGTATTTATATCTGA

TGTTGTAAAAGAGATGCAATATGGAAGCTGGAAAGTCAACTTTTTGAACATCACATATCT

TTCGGAATTGAGGAAAGACGGTCACCCTTCCAAATATCGGGAACCAGGAACCCCACCCGA

TGCTCCTCAGGATTGTAGCCACTGGTGTTTACCCGGAGTGCCAGACACGTGGAATGAACT

TATCTATGCCCAACTCCTCTCTAAGAAATATGGCATCAACAAAAGTTTTCCAGAAAGCGA

AGAACGCAGCTGA

>MS.gene80127.t1

ATTCAATGCCAGTGACCTCCTAGAGAGGAGTCGCAATGGACGCATTGTGTTTGCAGGTGA

TTCAGTGGGCAGAAACCAATGGGAGTCATTACTTTGCATGTTGTCAAAAGGGGTCTCTAA

CCTCTCAAAAATATATGAAGTTAATGGAAGTCCCATAAGCAAACACAAGGGCTACTTAGT

GATGAAATTTGAGGATTACAACATGACTGTAGAATATTATAGGGCACCTTTCTTATCTAT

TATAGGTCATCCACCACAAAACTCATCTAGTGACATTAAAATGACTATCAGACTTGATGA

GTTACATTGGTATTCTAATAACTGGAAGGGAGCAAATGTTCTTGTTTTCAATAATGGTCA

TTGGTGGAACTTAGACAAAACTATCAAGTCGGGTATCTATTTCCAGGAAGGTGGGAAGGT

AAATGTGACTATGACAGTGAAAGAAGCTTTTAGGAGATCCTTACAGACATGGAAATCATG

GGCATTGAGTAATCTAAATCCTATGAGGAGTTTCGTCTTCTTTCGTAGCTATGCCCTGAA

TGGCACATGGAATGAAGGAGGAAACTGTGACAAAGACAAGGAACCAGAAAACGATCCTGC

AAAACTCGAAGCTGATCCGTATTACAACGTATTTATATCTGATGTTGTAAAAGAGATGCA

ATATGGAAGCTGGAAAGTCAACTTTTTGAACATCACATATCTTTCGGAATTGAGGAAAGA

CGGTCACCCTTCCAAATATCGGGAACCAGGAACCCCACCCGATGCTCCTCAGGATTGTAG

CCACTGTGTTTACCCGGAGTGCCAGACACGTGGAATGAACTTATCTATGCCCAACTCCTC

TCTAAGAAATATGGCATCAACAAAAGTTTTCCAGAAAGCGAAGAACGCAGCTGATCCAAT

TTTCATCAGACCAGGAAGAATGTTAAGTTTTGTTAGCTATACATAA

>MS.gene80249.t1

CTCCTCGAATTGCTGCCTTTCGAATGGTTCCACAAGTGTAGGGAGAAAAGTGTCAGACTT

GCATTGCCTACTATTTAGTAAATGGGGCAGTTCAAGAAAGGGATGCCTTATTCAACATGA

TTTGTCCTCGAGCAGTGGTCAAGGTCCTGTGAGCTGTAGAAAACACTATTTTACATTTTC

TAAGACGGGTAGGAGTGTAAATTTGCTTCCATTTGCTACCTCTGATGATGGTATGACTGT

CAACGGGAGTCCACAAGCTGACGCCAGTGCTAACCTTGAAAAAATGAGGGTGAAATTGAA

TAGCTCTCTTGAAGATGAAGACTTTTATGATGGACTTGTTCAAGCTTTATATGATGCAGC

CAGGGTTTTTGAGCTAGCAATTAAAGACCATAAATCGTCATCACGTGGATCCTGGTTTTC

AACAGCTTGGCTTGGGGTAGACCAAACCGCATGGGTGAAGGCATTGTCATGTCAGGCTGC

CGTGTACTCCTTATTATATGCTGCAAGTGAAATTTCATCCCGAGATGATAGCAGAGATAG

AAATGTCAATGTATTTGTTCAAAGGAGTTTGCTAAGGCTATCTGCTCCCTTGGAGAGCTT

AATCAGAGAAAACTTATCTGCCAAACAGCCTGAAGTGTACGAATGGTTTTGGTCTGAGCA

AGTTCCAGCTGTAGTGACGTCCTTTGTTAATAAGTTTGAAGGGGACGGGCGCTTCACTTC

TGCCATTGCTTTGTCTGAAAAAACTAAGGGTTTGAGCAGTGCAAGTGATGTATCACTTCT

CCTGCTTGCACTTACATGCATTGCTGCAATTGCTAAACTTGGCCCGGCAAAAGTTTCTTG

CTCACAATTCTTTTCCATGAGCACTGAGATAACTGGTAGTTTGATGGACATGCTGGTTGG

TTTAATTCCTGTAAGTCAAGCTTATAATTCTATAAAGGATGTTGGTCTGCACAGAGAATT

TCTTGTACATTTTGGTCCTCGAGCTGCAGCTTTTAGAGCAAACGTTGAGTGGGGTTCAGA

AGAGGTTATTTTCTGGGTAAATCTGGTTCAGAAGCAGTTGCAGCAGGCTATTGATAAGGA

GAAAATATGGTCAAGACTGACAACATCTGAAAGCATTGAGGTTTTGGAGAAGGATTTGGC

TATATTTGGATTCTTTATTGCTTTAGGAAGAAGTACGCGAACATTTCTTTTGGCAAATGG

TTTCGATGGTCCCGATGATCCAATTGAAGATTTCATCAGGTATCTTATTGGGGGAAGTGT

TTTATACTACCCACAACTCTCATCCATTAGTTCATATCAATTGTATGTTGAGGTAGTTTG

TGAAGAGCTGGATTGGCTTCCTTTTTATCCGGGAATCACCAGCATCACAAAACAGTCTCA

TGGGCATAGTAAACCAGAAGGTCCTCCAAATGCAGAAGCAGTGACCCAAGCATTTGCTGT

TTGCTCTTATTGGATGCAGAGCTTTATTAAATACAGCACATGGCTTGAGAGTCCTTCAAA

TGTAAAAGCAGCTGAATTTCTGTCCAGAGGGCACAACAAGTTGATGGAGTGCATGGATGA

ACTTGGGATGATAAAAGATAAGACATTGGAGACTGATGCCAAGAAAATAGTTGACGGGCA

AAGATCCACAATTCAGTCAACGATAAAAGACTCGGGTTCTTTTGATGAGGCATTAAAAAG

TGTCGAAGAAGCTGTGATAAAGCTTGAAAAGTTGCTTCAAGAATTGCATGTGTCAAGCTC

TAGTTCTGGAAAGGAGCATTTGAAAGCAGCCTGTTCTGACTTGGAAAAAATACGGAAACT

TAAGAAAGAAGCTGAATTCCTGGAGGCATCTTTCCGAGCAAAAGCTGATTCTCTGCAAGA

GGGAGTTAATAGTGATCAAACCATCACACCGGTTGGTGAGGAGGACAGGTTTATGAAAGG

GAAAAGCAGAAAGAATGACAATGTAAAGGTGGACAGGAGCAAAAGACAAATTGGAAAATC

TCGTGGATTCTGGAGCATATTTGTACCTCCTATACCCAGAAAGCCCGACCCGGAACCTGA

TGTGGATGCTCATGAAAATTATATTGAACAGCCTGCACCAAATGTAGGGGTTGTGGACCA

AGAACCCAATGAAATCCTTCGCTTTGAGCTTCTAAGAAATGAGCTGATGGAACTTGAGAA

AAGGGTCCAAAGAAGTGCCTATCAGTCAGAAAATAATGAGGATTTGATGATCAGTGATGA

TGGTGCCCGTTATAACGGTGATGCTGGAGGTGTTCAGATGGTCAGAGTTCAGAAGAATGA

AAATTTCATACAGAAATCTTTTGACAAACTAAAAGAAACGGGAACGGATGTCTTGCAAGG

AACTCAACTTCTTGCTATTGATGTTGGTGCTGCCTCGGGTTTGGTCAAAAGGACCTTGAT

AGGTGATGAATTGACTGAGAAGGAGAAGAAAGCACTTAAAAGAACCTTAACTGACATGGC

TTCAGTTGTTCCTATCGGCATTTTAATGCTTCTTCCTGTTACTGCCGTTGGGCATGCAGC

AATGTTGGCTGCCATTCAGAGATATGTACCATCCCTGATTCCATCCACTTATGCACCAGA

AAGGTTGGATCTCTTGAGGCAGCTTGAGAAAGTGAAACAAATGTCAATGTCAACCAGTGA

TGTAGATTCAGATGATGAAATGGATGAAGTTAAATGA

>MS.gene81721.t1

ATGGAAGAGGAAAATGAGAACCTAACTATTGCCACACTATTTAATATCAAAGAGAAAAAG

AAGAAGAAAAAGAAGAAAAAGACGAAGGCAAATAAAGAGAGCGAAAGAGTTGCTGATGAT

TCTGCTACAGCGGCAAATGATGAAGGTGGTGGTGGTAGTACCAGCAGTGGTGCTAACCCT

AAGGTTTCTTTGTTTGATATCTCTGTCGAGAATTTCTTTGAAGACATGGACACCATTGCC

AAACTCTGTGGGGAAGAAGAACGCAACACTGCTGTTGAGCAAAGCGAGATCAAGAGAATG

TTTTCTTCCGTCACTTTTTTAAGAGAATGGAAGGATTTTAAATATCCATCTAAAAGCATT

AGGTTTACTTACGGAATTGGGAGCTCTGAGTGTTATGAGAGAAATGATATCAAAGATGAC

ATAAACTTACCTCAGTTTTCATCTGCCACTGTTCCTAAGCATAATATGCAAAAGGAGGAG

CAACTTGGGGATGCCAAACCTCAAGAATCCAAAGACTTTGTGATGAATGTTGGAGGTTCT

GTATGGGCATTAGATTGGTGTCCTAGGATGCATGGGGAGCCTGATTGTTCAATTAAATGT

GAGTTCATTGCTGTTGCTGCTCATCCGCCTGGTTCATCTTATCACAAAATGGGTGCCTCA

CTTACTGGCAGAGGTGCTGTGCAAATATGGTGTCTTCTAAATATCAGGGAACACAGCGAA

GAAGTATCATATCTCCCCGGGAAGAAGGAAAAAAAACCTAAAAAAGACACATGCACAAAT

GACAAATCGATAGAAATAAAGAGGCCTAGAGGAAGGCCTAGAAAGAATCCTACTGAAAAC

AACGAAGCAATATCTCCTATTACAAACAAGAGAAAAAGAGGAAGACCAAAAAAGAATCCA

ACTGTAATAGCAGTGGATAGTACAAATTGTGGGACCAAGTACATAGCATCTGGTAGTGTC

TCAAATGGGAATAATGAAAACAATGAAGAAATACTTCGTATTACATACAAGAGTAAAAAA

AGGCCTAAAGGAAGACCGAAAAAGAATTCAAAAGATGGAACAGTGAGTGATCCAAATTGT

GAGAACCAATTTGTTCCTCTTACTGTTCAACTTCCAGATTCAGCTGAATTTATTTCTCCA

GATGTAGTTCCTGGCAGTTCTGATGAACATCATTCACAACAATTTTCTAATACAAAGGGA

AAGAATGCCAAGAAAGCTGCTGCGAATACAAAGGGAAAGAATGCTAAGAAAGCTGCTCCG

AATACAAAGGGAAAGAATGCCAAGAAAGCTGCTTCTGCATACGATTCAGAAACTCTTGTT

GCAAGAAGTAGGTTGGATATTAATCATAGGGAAAGAAGTTGTAGCCCAGATACAAGTAGG

CCATTATTGATTCAGTGTGAGAATGAGGCAAATCATCAACCACATGGTAGCCCTGTATTG

GAACCTCAAGCATCTACTTGTCCCATTCCACAAAATGTAGCATTTCCTAGAGTTGTGTCA

TGCCTAGCTCATAATGGAAAGGTGGCATGGGATGTCAAATGGAGGCCTCTTAGCAATCTT

GATTCCTCATGCAAGCATCGAATGGGCTATCTTGCTGTCTTGTTGGGCAATGGATCTCTG

GAAGTGTGGGAGGTTCCTCTTCCTCATGCACTGAGAGCAGTTTATACGCAGAAGGAGGGC

ACAGATCCTCGTTTTATAAAATTGGAGCCTGTATTCAAATGCTCAATGTTGAAACGTGGC

AGCTTACAGAGCATTCCACTGACAGTGGAGTGGTCAGTTACTCCCCCTCATGATTATTTA

CTTGCTGGCTGCCATGATGGAACGGTTGCTCTGTGGAAATTCTCTACAAATTCTTCATCT

AAATGTGATGATACAAAGCCTATACTTTGTTTCGGTGGGGATACTGTTCCTATTAGAACA

GTTGCATGGGCTCCTTTTGAAGGTGATCCAGAGACTTCTAACTTAATAGTAACCGCAGGA

CATGAAGGCCTCAAGTTTTGGGACCTACGTAATCCATTTCGTCCTCTGAGGCTCCTCCAA

CCTTCCCAGAGAATCATTTACAGTCTGGATTGGCAGTCAAAGCCAAGCTGTATCATTATG

TGTTTTGAGGATGGAACAATGAAAACCATTAGCTTGGCGAAGGCTGCAAATGACCTCCCT

GTCACCGGAACGATATACACTGGAAAGAAACAACCTTGGTTGCATGGTACTACGTATTCA

TCTTATGCCATTTGGAGTGTTCAAGTGTCACCGATAACAGGCATGGTTGCATATTGTGGT

GCAGATGGTGCTGCTGTCCGTTTTCAGCTAACAACTAAATCAGTGGAGACTGATCATTCG

CACAATCGACTTCCATTCTTTCTATGTGGATCAGTGACTGAAGAGGAATCAACTATCATT

GTCAACACTCCAGTATCAAACTCTCCTTTCCCATTGAAGCTGCAAGAAAGAGGCAGATAT

TCAGAATCTTTCCGAGACTTATTATCCAAAAGCAGAATTGCGTTTAATCAAATGATAAAA

GCTTCTACTGATGATTGTCAGATTTTAGCTCTTTGTGATGGTGATAATATGGGTTTGGAA

TCTGTATCTGAGGAAGCATTGTCTTCTCGGGAGCAAACCAAAAGACCAAAACTGAGCTGT

AGCAGAAAGAAAAAACCAGCTGAAAGCACAGCTCTTGCTTGTAGAGATGGTGCGCCGACA

AATACTCCCGGAATTGATAATGAAAAGCCGGATTCTGGGACTACTCCCGAAGTCTTTCCT

CCTAAGATGGCAGCATTGCATAAAGTGAGATGGAACATTAACAAGGGTAGTGAGAGATGG

TTGTGCTTTGGGGGAGCTAATGGACTTGTACGTTGTCAGGAAATTGTTTACTCTAATATT

GATAAGAAAATGGCCTTGAAGAGATGA

>MS.gene81716.t1

ATGAGCGTTCCGAAAGTGGTAGCAGCGACAATCCGGTTGAGAGTTCCAGCTGGAGGAGCA

CGTCCGGCGCCTCCTGTGGGACCTGCGCTAGGTCAGTACCGACTGAACCTTATGGCTTTC

TGCAAAGACTTCAACGCACGGACTCAGAAATTCAAGCCTGATACACCAATGGCTGTTACA

ATAACAGCTTACAAGGACAACACCTTTGAATTCACTGTTAAATCTCCTTCCGTCTCGTGG

TACCTGAAGAAAGCTGCGGGTATCGAATTGGGAAGTACCCGTCCCGGTCATGTGACCGCC

ACCTCCCTGTCACTTCGGCACGTGTATGAGATCGCTAAGGTGAAACAGTCTGATCCTTAC

TTGCAGGGTATGCCACTTGAGTCCATTTCTAAATCCATTATTGGTACAGCTAGGAGCATG

GGAATCAAAATTGTCAAAGATCTTGAATGA

>MS.gene81718.t1

ATGAAGGTTGTTGCTGCCTATTTGCTTGCTGTTCTTGGTGGCGACAACACCCCCTCCGCC

AAAACCATCAAGGACATCCTCGGATCCGTTGGAGCAGACGCAGAAGATGATAGGATTGAG

TTGTTTTTGTCTGAAATTAAGGGCAAAGATATAGCTGAGGTAAGCTTTGATCAATCCCCT

GTAGTCAATCGCAGCCGCTGTAGCAGTGTTGCATAA

>MS.gene81714.t1

ATGGATGCCGGAGATTTGTGCGAAAATAAGGGGTCTGGAATTTTTCCTGAGCATGGTGCT

ATCTTCATGTCCAATAGGAGTACTTTGAAAGAATGCTTTGAGAGGAATTTGTTTGGGTTA

CCGGGTAATTTTTTTGATTTTGTTAAAAACGTCAAGGCAGGAATGATTTTGTTCCTTTTC

GAATTTGAAGAGAGGAAACTTCATGGGGTTTTTGAAGCGATAACAGATGGTGGCATGAAC

ATTGCTCCTCGTGCATATGTTTCATCTGGAAAGCAGTTCCCTGCACAGGTTAAATTCACC

CGAATCTTGCGCTGCAACCCTCTTTTTGAAAATGAATTTTGTGATGCTATTCGAGCTAAT

TACTTTACCAAGTACAAATTCAACTTTGGTTTGTCTGAAGATCAGGTTCGAAGTCTGATG

TGGCTATTCAATTCAAGAAAATGTGAAGTTCCACGCTCTCTTCATCAGAAGAAAAGGAAA

ACAAGAAACTGGGATTTTCAGATTATTGAAGATGTGCTAAAGAAAGGGGGGGTTACCAAC

CCTCCGAAAAGGAAGCTCATTGTGAACCATGGTACTCCAGTAACTGCTGAGCAAGAGGTG

GAAAAGCTTTCTCTGTCTCCTGAGTCTCTCGGAAAGGGTGAAAGCATGCATTTGGATGTT

GATGCTTATGATCCTGAACACCCTGGCTTCAATCAATCAGTGGCATCTGGGGCTCACTCT

GCTGCCAGCTTTGAATCGCGTGAGCTTCCTACATTGCAAGAGAAGAAAGAAAACTTCCAT

ATTTTTGAGGATGGTAATGAAGATTTTATACCCTTATGTTCAACTGACCATTCTGACCTT

GAAGATGGGGAGCTTTGTAATTCTAGCGATGGTTCAGATGAGGAACAGATAGAGTTAGAT

ATGCTTATTGGAAATGATGATTCTTCTATCCCTGTTCCACGATTTCTGCTGAGTGATAAA

GAATCTGACAAGTTAGGTGACTTTTCCTCTGATGCTGTAAGTGACTTCCAGTCCAAAGAT

GAAAGTGATCATTTGAATTCCCTTCTCTCAAAGGGTATGTATTGTGATAAGCCCAAACAA

AAAACCAGTGTGTTTTCTCGCTTAAGTGTTTCTTTGAAGGGTATTGCTTCGAAGAATCAG

AATGACGCCAATAGAAAGGACTTGGTGAATAAGAATAACATGTCAAGAAAAAATAAGCAA

TATCAATATGAAAGCATTAAAGATGCCACACAACAGAGGGAAAATGGTACAACGTATAAA

AGAGCTAGTGTGTTTCTGCGCTTGGCCGGTGCTTCAGATGCTGTTTCTCCACAAGTCCCC

TCTATGACAGGACTATATGAAAGAACTGGCGGCCAGAAGAAAGTTTGGTAG

>MS.gene81707.t1

ATGGCCACTGGTGAAGGCGAAACACTCCTTGCACTCTCAACTTCTGCGAAGGTGTTGTTA

GATAAGACTCTTTCTAGTGATCTCGCGGACAACTTCCAGACCACCAAGCTTGTTGCTTCA

CTCTTGGAAAAACTGAAGTCAATTTTGGCGAAACTTCAAACTGTACTTCAGAATGCTCAA

GAGAAACGAATCACCACAATGACCCTGAAGATTTTTGAATCTATTGCTGTACCAGCCTTT

TACCCAAATATTTTGCTCGTATTAGATAACTTGTCGGGTGCACAATCTGTCAATTGGATA

TATCTGATGGATATCTTTAATGCTGTGGAAATGGGAAGTTGTAAAAGGCTCACTAAGTTG

CCGGAGGACATGGGGAAATTGGTTAATCTACGTCACCTTGACGTTAGTGACACTGCTTTG

AGAGAGATGCCCATACATATAGCCAAACTAGAAAATCTCCAAACTTTGTCTGATTTTGTT

GTCAGCAAACATAATGGTGGACTGAAGGTTGCAGAGCTGGGAAAATTTCCCCACCTACAT

GGAAAGCTTTCCATCTCAGAATTGCAAAATGTCAATGACCCCTTTGAAGCAGTTCAAGCC

AATATGAAGATGAAAGAGCGAATAGACGAGTTGGCTTTGCAATGGGATTGTGGGATGCAA

TCAGTACAGACAATTGATACTGAGTTCTATGGAAGTGATAGTTCTCCTTCATTACAACCG

TTTCCCTCATTGGAGACTCTGCAATTTCAGAATATGCAAGAGTGGGAGGAGTGGAACCTG

ATTGGAGGTACGACTAAGGAGTTTCCTTGTCTAAAAACTTTGTCACTACGTAAGTGCCCG

AAAATCTCGATTAATGTGTGGGAGGAGTGGAACCTGATTGGAGGTACGGCTAAGGAGTTT

CCTTGTCTAAAAACTTTGTCACTACGTAAGTGCCCGAAACTTAGAAGGAAACATCATAGA

GGACATTCGATTAATGTGTGGGAGGAGTGGAACCTGATTGGAGGTACGGCTAAGGAGTTT

CCTTGTCCAAAAACTTTGTCACTACATGTTCACATCATAGAGGACATTCACTACGTGATT

AATGTGTGGGAGGAGTGGAACCTGATTGGAGGTACGACTAAGGAGTTTCCTTGTCTAAAA

ACTTTGTCACTACATGTTCACATCATAGAGGACATTCACTACGTAAGTGCCCGAAACTTG

GAAGGAAACATTCACTACGTTGTGAAATTATCCATATGGATGTCAAGTTTGAAGCTTCTA

TACTTTTTCAGGTAG

>MS.gene81724.t1

ATGGACTCACAAAAGCACCTTTTCATCCTCTTATGTCTTTCCCTATTACTTGTACTTCTC

TCAGGAGAAATGGGGCAGCAAGTTGAGGCCACTCGCCATCTCAATAATCCTTCAAAAAAA

TTGTTTGTTTTTGGAGATTCGTATGTTGATACTGGCAACTCCATAAAAACCATATTTGAT

TCATGGAAAGTACCTTATGGCATGACCTTTCCCGGAAAACCAGTTGGGAGATTCTCCGAC

GGCAGAGTTTTAACTGACTTCCTTGCCAAGTATTTGGGATTGAAATCACCAATCACTCAC

AATTTATGGAATACTAATTCAGTGCCTAAGCATGATTTGAAGTATGGGATGAACTTTGCC

TACGGTGGTACAGGTGTATTTGAAGTATCTTCTATAGGCCCAAATGTGACCCAAATCAAT

TCCTTAATCAAGCTCATTCAAGAAAATGTCTACACTCCCTCAGATATCACCAAATCTATA

GCTTATGTCTCTGTTGCTGGAAATGACTACAATTATTATTTGGAAACAGGAGGCGCTTTC

CTGGGTTTCCCATCTTTCCCATCATTCATTGAATCAGTGATTGAACAAACAACCACCAAT

TTGATTCATCTTCAAAGCTTAGGATTTGAGAGAATTGTTGTGGGAGGTTTACAACCCCTT

GGTTGTCTTCCTCAAGCCACTGCTGAAACCTCATTTCAAAGTTGCAATAGTACATTTAAT

GACCTTGTAGCTCTCCACAATAACCTATTGAACCAATCTGTTACAAAGTTGAACCAAGAA

ACCAATGATCACACCACATTCACAATTCTTGACATTTTCGATAGTTTCAGGTCAGTGTTG

AATCACCCTTCTAGCCATAACATTGAGGAACGTTTGAAGCCATGTTGTGTTGGAGTAAGC

AGTGAGTATAAGTGTGGAAGTGTAGATTATAAAAATTTAAATAAGGTTAAGAAATATTTG

GTTTGTGAAAATCCTGAATCAACTTTCTTTTGGGATCAGTTGCACCCAACACAGGCTGGT

TGGAATGCTGTGTATAATGAGTTAGAGAAGAAAGATCTTTATCAAATTTTGTATTAG

>MS.gene81726.t1

ATGATTTGCATTTCCGGCGAATCAGAGGAGGCTGAAACAACCACATTCTCCGCCGTCCAC

GACGACATAATCCAAACTCACATACTCACACGCCTTGATGGCGCCGCTCTTGCTTCCGCC

GCAAGCACCTGCTCCCAACTCAACTCTCTCTGTTCCGACGAACATCTCTGGGCAAACGCA

TGCTATTCTACGTGGCCTTCCACCAACATGCCACGTGTTCGCCATGTAATCTCAACGTTC

TCCAATGGCTCCCGCTCTTTCTTCGCAGACACTTTCTCTTCACATCATCAAAGGGATACA

GCTTCAACAATTCATGATCGAACCCTAAGTTTAATCTCAGCCGTTGATATTTTCCATTGT

AAGGGTCTAGTGTTTTCCAAAGTAGTTGAAACTGAAACCGTGACCACCCGGTTCCGGTGC

TCTCCGTTTCGTGTTGACCTACTAGATAACAAGGATGTGATCAAATATCCAGTTGGTAGT

GATGAAAACCTAGGGGAGGAGTTGAGGCTGAGCTGGATTTTGATCGATCCGGTGAGTCAA

CGGGCGGTGAACGTGTCGAGTGGCAAGGTGATGGAGGTGGTGCAGCGGAGTGGGGAGGTG

AAGTTGCTGTTTGCAACGGTGGTTTACGGAGAAACAGCGACGGCGACGGAGGTGGCGTTG

TGTAGTGTGGTGGTGATGTTGGCGGAGGAGAATATGGAGGTTAAAGAGGTTAGTTTGCAA

GTGGAGGATATGGATGGAAAACACGTGAATGGGAGGGATAGTTTGGTAATTTTGCGAAAG

GCATTGGAAGGTAGGAGAAAATGGAAGGGAATTGGGGAAGAAGAAGGTTATAGGGAATTC

GTAAAGAAGAAGAAAGAAAGGGAGGAAAGAAAAAAGAGGGCACAAAGGAGATTGGATATG

ATGTGTTTGCTTACAGGTCTTGCGTCGTTGGCCTTCATTGCACTCTTTGATTTGTGA

>MS.gene81711.t1

ATGTTATCGTTTCGCTTTCGTTTGTCTTGTTTTACACATTACAGAAGACTCCTCTTCCTT

CTCCCTTCTCCAACCAACATCTCCAGAAATCAATGTTGCTTCTCTGTTAAACCCATTAAA

CAAAATAATAATAACCTTCAATCTTCTTCTTTCTCCAAAGATTCTCCCTTTTCAGGATTA

GAGAATGTTTTTGTGAGCTTCCTCTTTGGAAAGAAAAGGGCAACAGATGTTGCTCATATG

GTATGGAAACATGTTGTCCAGAAAGGAGATACAGTCATTGATGCGACATGTGGCAACGGT

TTTGACACCTTAGCATTGCGCAATTTGGTTGCTGATGAGTCACATAATGGTTATGTGTAC

GCATTGGACATTCAGAAAGATGCTTTAGATAAGACTTCACTGTTACTGGAAGAATCGCTT

AGTTCCAATGAGAAACAACATGTCAAGCTCTTCAATATTTGCCATAGTAAAATGGAGGAA

GTTGTTCAAAGCAATGCCCACGTTAGGCTTGTTGCCTTCAACTTAGGTTATCTTCCGGGA

GGTAACAAAGAAATAATAACAAGATCAGAAACAACATTACTGGCATTAGAAGCTGCAAAG

AGAATTCTAATACCAGGAGGGCTTATTAGCATAGTGGTTTATGTTGGGCACCCTGGTGGA

CGGTAA

>MS.gene81709.t1

ATGTCGCAGGAACATCCGCAGCTCGTTGAAGCTCCAATCCTTCATCGTCAATCGATTCGT

CGAAGCTCCAATCGAACACCTTCCGATCTGAAACTACTCGCTCAGGTAATGTGCATTGAT

TTGAAGCTGTATAAGGAAACAAAATCTCAGAAGGCACATAAAGTGTTCGATGAAATGATT

GTCAGGAATTTGTTTACTTTGTCTGCTATGATTGGGGCTTATAGGATAGTGATATATAAT

GCTGGTGTTATTGTGAATCCTAAGGGAGAAATGAAAGGTTCTGCTATCACAGGACCAATT

GGTAAGGAGTTTGCTGATCTTTGGCCTAGGATTGCAAGTGTTGCCAATGCCATTGAATCA

TTCACGGGCAGGGGAACTTCTGCCACTCTGACCAAAAGGTTTCCTGCGGATATTGTTGGC

TTTTCTTGTGAAAAGTTCCGCAGGGAATGTGAGCAATCTTACGCCACTCTTTCCCTCGCT

TCCTCCGCAAACTTGCTTCCAGTAATGGACAGTGAGGCTTTGGAGAGAAGTGAGATGTTG

AAGCCACTTCAGATCAATGCTTTTATACCACTAAGACCACAGATACATAGTGTCACGAGA

GATGCAGGTAGCAAGGGCACCATGAATGAGTTCACCATATCACCATCTTTAATTCGCAAC

ACTGACAGACAATTGAAACGTTCCCAGTTAGAGTGGTCATTGCTTCGGGTAGTGAACGAA

GTTTCTCACACTTCCACACTGCAATATAAATGA

>MS.gene81710.t1

ATGGAAGAAGCTCTGATGGAAATAGATGACTGTTATCAGGAGCAGATCATGCCTGAGGCT

GATACTCTTCCATTTGCTAGAAGCTATCAACTTGAGGCATTGGATAAAGCGATTCGCGAA

AATACTATAGTGTACTTGGAGACTGGTTGTGGAAAGACTTTGATAGCCATCATGCTTCTT

CGAAGCTACGCATATCACCTCCGAAAGCCTTCTCCTTATATTGCTGTTTTTTTGGTTCCC

AAAGTTGTTTTGGTTTCTCAACAAGCTCAGGCTTTGAGAAATCACACTGATTTGAAAGTT

GGAATGTATTGGGGAGACATGGGTGTTGACTTCTGGGATGGAGCTATATGGAAAGAAGAA

ATGGAGAAACATGAGGTGCTTGTTATGACTCCTGCAATATTGCTTTCTTGCTTGAGGCAT

AGCTTTATCAAACTGAATATGATAAAGGTTTTAATAATGGATGAATGCCATCATGCTTCT

GGTAGACACCCATATGCTTGTATCATGACTGAATTTTATCACCATCAATTAAGATCTGGT

ATCACCGAGCTTCCTCGAATTTTCGGGATGACTGCATCCCCAATTAAGTCGAAAGCGGCA

AATTCTAAGTCGACCTTGTCAAAAAATATTCGGGAACTAATGACTTTAATGAATTCAAAG

GTTTATACTTGTGTGAGCGATGCTGTCATCTCCAAATTCATACCGACATCAACCCCAAAA

TTCCAGTTTTACGATGAAAATGTAATTTCATATGCGCTATTTGCAGAATTAGCCGATAAA

CTCAGCAATTTAAAACAACAGCACGAGCTTGACGTCGCAAGTTCAAATTTCATAAAATCA

ACTGTTGACTCTGCACACAAGAAAATAGCAAAGATTCATACTCACTCAATTTTTTGCTTG

GAAGAGCTTGGTGTTTGGTTGGCTTTGAAGGCCGCAGAGTCTTTATCATCCATTGAAATT

GAAACTTTCTTATGGGGCAACTCGGGCGATCAAATTGTGAAAGATTTTTGTTCGTCCGCT

ATTCTGACACTGCAAAGTTACATACCATCTGATCCTCAGTGGACTATCGGTGACAATATG

AATTCCGATATGGAAAAGGGGCTATTGACCTCCAAAGTGTGTTGTCTTATGGACTGTCTA

CTTGATTACAGGAGTTTTACTGAAATGAGATGCATAATTTTTGTGGAAAGGGTCATTACA

GCTATTGTCCTTGAGATTCTTTTGAACACTTTGCTTCCGAAATACAATAGCTGGACCGCT

AAATTCATTGCAGGAAATGGTAGTAAATTACAAAACCAAACAAGGAAAAATCAAAATCAA

ATTGTGGAAGATTTCCGTATGGGATTGGTCAACGTCATTGTTGCGACATCAATTCTTGAG

GAGGGTTTAGATGTTCAAAGTTGCAATTTGGTTATTAGATTTGACCCATCTCCCACCGTG

TGCAGTTTTGTACAGTCCCGTGGCCGTGCCAGAATGCAAAATTCAGATTATATATTGATG

GTTAAGAGTGGGGATTCAGTTACACGTTCTCGATTAGAGAAATACCTTGATGGTGTGGAA

ATGATGAGGAAGGAGTCCTTGCATCATTCTTCCCTTCCATGTGAAAGTCTTGAAAGTGAT

CAATTTAATGAGCAAGCTTACCGTGTTGCAAGCACTGAAGCTGTTGTCAATCTTAGTTCT

AGCATTACTTTGATATACTTGTACTGCTCACGACTTCCAGCAGATGGGTACTTTAAACCG

ACTCTAAGGTGGGACAAACAAACTGGAACGTTGTATCTTCCTAAGAGTTGTCCTCTACAA

GCTATTAATGTACAAGGTGACTCAAAGTTCTTAAAAAATATTGCATGTCTTGAAGCATGC

AAACGACTTCATATGATTGGAGCTTTGACAGATAATCTTGTTCCTTCTATAGTCGTTGAA

GAAGCAGAGGTGGAGGACTTTGGGAATGAACCATATGATGAGGATCAACCAAGTTATGTA

CCGCTTGAATTGGTGAATCGTATGCCAAACAATAGTAACACAATATACTATTGTTATTTA

ATAGAGCTAAAACAAAATTTTAGCTACGATATCACTGTGCAAGACATTTTTCTTGCAACT

AGAGTCAAGCTTGATCTAGAAACTGAATGCATGCAATTTGACATGTGTTTCGATAGAGGT

AGCTTATCTATAAACTTGAGATACAAAGGATCCATTAATCTCTCGCCGGATCAGGTTCTT

TTGTGTAAAAGATTCCAAGTTAATGTCCTTGGAATTCTTATGAACCATAAAATGGATATA

GAAACTGTTTCCGACAAATTCTGTTTGGAAGATGATCTTGGGTTTGATTATCTTCTGCTG

CCATCTATTGCTATAGAGGAAACACCAACTGTTGATTGGATAACTATTAATTCGATACAT

CCATCTATAGTTAAGTGTCTGCATCATGAAGCAAATATATGGACCGAAAAGGGTTTAGTT

TGCCCTTGCATATTGCGAAATGCTTTGATTTGTACTCCTCATAATGGTCGTACCTACATC

ACCACTGGTATAATGGAATTGGATGGAAATTCAGCTCTGGAAGTAGGGGATGGTGAAGTG

ACCACGTACAAGAACTACTTTGGACAAAAACATGGCATTCAATTGCGTTTTGAGCATCAA

CGCCTACTTAAGGCAAGACACGTTTTTCCAGTAAAAAATTATTGTCACGGATATAGGCAA

TCGAAGGACAAAGATGTGAGCAAGACCTTTGTTGAGTTACCTCCTGAACTTTGCTCTATA

ATCATGTCACCAATATCAGTCAGAACGCTTTATTCATTTTCATTTATTCCATCGATCATG

CATAGACTTGAGTCATTGCTTGTAGCTTTGAACTTTAAAAAGATGCATTTGAATCATTGC

CCGCAGAACGGTATTCAAACATTCAAGGTCTTGGAAGCAATGACCACAAAGGCATGCAAG

GAGACCTTTCATTATGAGTCTCTGGAGACTCTAGGAGATTCTTTTTTAAAATATGCTGTT

AGTCAACAGCTTTTTAATATGTATGAAAATCATCACGAGGGTCTTCTTAGTGTGAAGAGG

GAGAAGATCATTTGTAACGCTGCCCTCTGTAAGTTAGGTTGCGGTTCTATACTTCCGGGC

TTCATACGGAATGAGCCTTTTGATCCTAAGACCTGGATCATTCCTGGAGTTAAATCAAGA

TGTTTTAAATTAGAAGAGACCGTTTTCAAGGGGACAAAAATATATACTCGCGGAAATAGA

AAATTGAAACGGAAAGTTGTTGCTGATGTCGTTGAAGCCCTAATTGGTGCATTCCTTAGC

ACTGGCGGTGAGATGGCTGCATTGTTGTTTATGGATTGGGTTGGCATCAAAGTGAATTTG

AATATTACACCCTATGAGAGGCAACTCAATGCTTGTCCAGACAATCTAGTTAATGTCAGC

TATCTAGAATCCCTCTTGAAGTACTCGTTTAGGGACCGTTCGCTGTTAGTAGAAGCTATG

ACACACGGTTCTTACATGCTGCCTGACGTGCCAAGATGCTATCAGCGACTTGAATACTTA

GGAGACTCGGTGTTGGATTATCTAATTACTACACATTTGTATAATGAATATCCTGGCCTG

ACACCGGGGCAGTTAACTGATATGCGGTCAGCTTCTGTGAACAATGATTGTTATGCAATG

TCTGCCATCAAGGTTCAACTGCATAAACATGTACTCCATGCCTCTCAGGAGTTACATAAA

CATATTGCAGCTACCCTTGATAAGTTTGACGAACAGTCTGCATCACAAACTTTTGGATGG

GAGTCCGAGGCTTCATTCCCCAAGGTACTTGGAGACATCATAGAGTCTCTAGCTGGTGCT

ATATTTGTTGATTCAGGATACAACAAGGAGGTTGTGTGGCAAAGCATAAGACCTCTTTTG

GAACCCCTTGTGACACCTGAAACATTAACGATCCATCCGATCAGAGAACTAACTGAACTG

TGTCAGAAAATGAATTATACTATGGAGAAGAACTTGTCCAGGAACGACGGCGTTACTAGC

TGTAGAATTGAGGTAATTGCAGATGGTATAATTCATCAATACGAGTACAAAGGTTCTACT

GACAAGAAAACAGCTACAAGACTAGCCTGCAAGGGAGTTTTGAATTCCTTGCAGTTGAAG

GAAACACAAGACCAGTGA

>MS.gene81708.t1

ATGATATCATTTACCTTGGGAGCTCTCCCTGTCCTCAAGAATCTGTTTATTGAGGGTTGT

AAAAATCTGAAATGGATACTAATTGCAGAAGACGGGTCGCAAAAGAGTCTCTCATTTCTT

AGAAGCATCAAAATATGGGATTGTAATGAACTGAATTCATTTCCCCTAGGCGGATTGCAC

ACTCCAGACCTCATTCATTTTGTAGCGTGGAAGTGTCAGCAGCTTCAGTCACTACCAGAA

GCAATGAATAGTCTAAATGACCTTCAAGAAATGGAAATTGATGATATGCCAAATCTTCAA

TCTTTTGCCGTAGATGATTTGCCTATCAGTTTGCGGGAACTGACTGATGGCTCTGTTGGA

GGGATTATGTGGAATACCGAGCCAACTTGGGAACATCTCACTTGTCTTTCAGTGTTGCGA

ATTAATGGCAATGCTACGGTGAACACGCTGATGGTGACACTGCTGCATCGCTTGTTACAC

TATGCATCAGTGGTCTTAATAATACAAGAATTGATGGGATGTGGCTTCAACATCTCACTT

CTCTCCAAAACCTTGAGATTGTTAACGCTCCAAAACTCAAGTCATTGCCAAAAAAAGGAT

TTCCTTCCTCTCTTTCGGTACTAA

>MS.gene81713.t1

ATGGGAGCACTTAGCACATACTTCAACTATAATTGTTACCCTTATCAACCTATTCAAACT

ACCAACAACTTAGAAATCACTACTTTTCAACATCATGAACAAGAACAATTTGTGCAACAA

AACCACCATTTTCCCTCTAACTATTTGAATGAAACTTCTCTTTTAGATGAAAGCTTCATT

TTTCAAACTCCTTGTTTTTACTCAAATGAAACTTACCCTAACCTTTGTCAAGATCAACTA

CTTGTTGACTCCACTTTTTCTTCTCAAAATGATGGCTTTGTTTCAATGAATGAAATTTTC

CCAAATGAGGAAGATTTCACCAATTACCTTACATGTCCAAAACGCCAAAAGCTATGCTAC

GAGGAGAAAAGAGAGGAACCACAACAAGAGCTTTTGAACTCAACCAATTTTTTTGTGGAT

GAGTTTATGACTAACCCTAACCCTTTTGCTTCCTTTGAGGCAGAACCCTTTGCTGCTTCA

AAAAAAATTGTTGATGTTGTTCAATGTGAGAAGAAAGTTGCTGAAAGGACTATCTCGTCG

CAGAGTATCGCGGCGAGAGAAAGGAGAAGAAAGATAACTGAGAAGACACAAGAACTTGGG

AAATTGGTTCCTGGTGGACCAAAGATGAACACAGCAGAGATGCTTAATGCTGCTGCTAAT

TATGTCAAGTTTCTTCAAGCTCAAGTTGGAATGCTTCAACTTATGGAAACATTTAGCAAG

GAAGAAAAGGAACCTCCTCCAAGTGAAGATCTACACAAACTACTTCTTTCTCCATTTGTT

CAAGAGAAACTGTATTCAGAAGAAAAGTGCTTTGTTCCAAAAGAGTTTGTCACCACATTG

AGCAATCATAATAATGTTCAATCAAAACCTACCATTCTTAAGGGTCTGAAACAGCTTGCT

GGGACTGAGATTGAGAAGAAACCAAAGCAAGAATAG

>MS.gene81725.t1

ATGGACTCGTATAAGCAACTTTTCATCTTCTCATGTCTTTCGCTGGTTCTTGTACTTCTC

TCAGGGCTGCAAGCTGAGGCCAGTCGTCACCTCAAATATCATCCTACAAAAGAATTGTTC

GTTTTTGGAGACTCTTATGTTGATACCGGCAACACCCAAAAAAGTAAACCTGGTTCATGG

AAAGAACCTTATGGCATAACCTTTCCCGGAAAACCTGCCGGAAGATTCTCCGACGGTAGA

GTTTTAACTGACTTCATTGCCAAGTATTTGAGATTGAGATCACCAATCCCTCACAAATTC

AGGAACATTGTGCCAAAACATTATTTAAAATACGGGATGAACTTTGCATATGGTGGTACA

GGTGTATTTGATACATTTACTTCAGGACCAAATATGACAACTCAAATCGATTCCTTCAAC

CAACTCATTCAAGAAAATGTCTATACTCATTCAGATCTCAACAAAGCTATTGCTTATGTC

TCTGTTGCTGGAAATGACTACAATCATTACTTGGCCACAAATGGCTCTCTCCCGGGTTTT

CCATCTTTCATTGCTTCAGTGGTTAAACAAACAACGATCGATTTGATTCATCTCCAAAGC

ATAGGATTTAAGAGAATTGTTGTAGGTGCATTACAACCACTTGGATGTCTTCCTCAGACC

ACTGCTCAATCCTCGTTCCAACGTTGCAATAGTACATTTAACGACCTCGTAGCTCTCCAT

AACAACCTATTGAACCAATCCGTGACCAAATTAAACCAAGAAACCAAGGATCACACCACC

TTTACAATTCTAGACATTTTTGACAGTTTCGCGTCCGTGTTGAACCACCCTTCAAGCCAT

AATATCAAGGATCGTTTGAAGCCATGTTGCGCGGGAGTAAGCATTCAGTATAATTGTGGA

AGTGTAGACGAGAATAATGTTAAGAAATATTCGGTTTGCGAAAATCCGGAATCAAGTTTC

TTTTGGGATGGGTTGCACCCAACACAAGCTGGATGGAATGCTGTATATAGTGAGTTGGAG

AAGAAAGGTCTTCACCATATTTTGTACTAG

>MS.gene81728.t1

ATGGAATCACAACCTCCGGTCACCGGAACCACCGCTAGAACCACCACCGAATCCTCACAA

TCTCAACCACAACAACAACAACCACCACCCTCTCTCCCTCCACCCACATCCTCTACCCCT

CCCATCTCAGCACCATCACATCCTTCCCCAATCCCTAACCCTAACCCTAATCTAATCCAA

ACTCCAAACCCTAAACCCCCAACTCCACTTCCTCCCCAATCACGTCCTCCTCCGTCTTCA

TTCACCCGAAATCCTCCTCCGACACAGTCACATTACTCTCACTTCTCATCAATCCCACCT

TCCGCTAATCCTTCACAAGCTTCATCTTTTCCGTCCAATCCCGCTTCATCGATTTCCTCG

GCTTCTGCGCCGAGAGGCGGTATGGCAATTGGTGTACCCGCGCACCACCAGAGCCCTTCG

CCTCCGTTTTCTTCTTCCTTCAGTCAGCATTTTGGTGGAATGGGTCGTTCTGATTCTACA

ACAAATTCTAACACCTCCCAGGTGCGAGCGCCAATGCAAGGAATGGGGACGTTAGGATCT

TTCGGTTCGAATTCACAAATGCGGCCAGGTGGAATGCCTCCACATCAACAGAGACCTGTT

CAATCGTCTCTTAGACCACCGCCGCCTTCTGCTCAAAATAATCAACCTGCTGGTTCTCAG

AGTTTCCAAGGTCATGGACTTATGAGACCGTCATCTGTGGGACCACCTTCAGCTCCATCT

CCAAGTGCATCACAAGGTATGCAGTCAATTAATCAGCCATGGTTGTCGTCTGGGCCACCA

GGGAAGCCTCCTTTGCCATCCCCGGCCTATAGGCAGCAAATAAACCCACAATCCTTGCAG

CAAAGGACGCATATTTCCCAGCAGCAGCAATCCATGCCAACAGCTTCACAGCAGCAGCAA

TCTTTGCCTTCTAATCAAACCCAGGAGCATTTTGGGCAACAAGTTTCGTCTTCAAGAGCT

CCACATGTGCCTCACCAGCCACAGGTTACAAGGCTACAGGGGCCAGGAAATCAGAAACCT

TCATCACTTGTGGCTGGACAGTCCGGTGCAGTTCAACCAGGGAGTCAAAGTAGATTACCA

AATACTTTACCAAATGCAGATATAGAAGAATCGGGCAAGAGTGTTCTCAGCAAAAGAAGC

ATCCATGAGCTAGTCCATCAGGTTGATCCATTGGAGAAGTTAGATCCTGAAGTTGCAGAC

ATTCTTGGCGATATTGCAGAAAATTTTCTGGAGTCTATAATTAGGTCTGGTTGCTCACTA

GCCAAACATCGGAAGTCAACAACTTTGGAAGCCAAGGACGTACTTTTACATCTTGAGAAA

AATTGGAATATTACGCTTCCTGGATTTGGTGGTGATGAGATTAAAAACTACAGAAAACCG

CTTTCAACTGATATTCACAAAGAGCGCCTAGCGGCTATAAAGAAATCAATGATAGCAACT

GAGGCTGCACATCCTAAGGGCTCTGCTGGTCAGGCTTCTGGTAGTGCAAAGGGTAGTCAG

GCAAAGCTACCTTTCAATGCCATTGGCTCTCCCAACCTTAAAAATCCATAA

>MS.gene81727.t1

ATGGCTTGTGGATGTTCATCAACAGCAATGGCAGAGAAGATTTCATGGAACTGTGCACTG

TTTGTGGCATTGATGTTGGTTTTGAGCTTCTGCGAATCAAACACAAGTGATGATGAGTTG

AATGTTCAGATACAAAATATGCTTCAAGAGAACGATTTTAGTATTAGTAATGCGAACAAC

AACAAAGTGTGTGATGAGATATACGTTGTTGGCGAAGGAGAGACACTTCAAACAATAAGT

GAAAAATGTGGTGATCCTTATATTGTTGAAGAGAATCCACATATTCATGATCCTGATGAT

GTTTTCCCTGGCCTTGTTATCAAGATTAACCCTTTCTCTGTTAGTTCATAA

>MS.gene81712.t1

ATGGACATATGGATTCAAGCTCATAACCTCCCTTTTGGATTTATGGCTGAATCAATGGGC

CATCTCTTGGGAAACCATGTTGGAAAACTGCTGAAATATGATTTTAACAACAATTATGGT

ACATGGCGTAAATACATGCGCCTTAGAGTTACCATGAATGTCAATGAACCGCTGAAGCAG

TGTTTGGAATTTGAAAGGGAAGGTGCTGATCCGGTGACCGTGGTTTTTAAGTACGAAAAC

CTTGGCAATTTCTGTTATATTTGCGGATTGCTAGGCCACACTGATGCTTTTTGCCCCAAA

AGACAAGAGACAGGTGCACAGAGGCAGAAAGGTGGGGAACGTCCAGCTTGCAGTAAGATT

CATAGTCGAGTCGGAAAAGTCAAGATTGGTAGAAACCCTGTCACAAAGGAGCTGATATTT

GCAAAATACATTGGGGCACAAAACAGCAGCAATGAATGGATTACTTTTGACCCTTTCAGT

GCAGATTTCAATGCTTGGCCAGTCGTGGAAGTCGCTGCTGCTGCACCAGGCCCAAACATA

CTTCAACTAGCAGGAACGGGAGAACCTTCTGAAATGCGTGCTGAACAGGAAGAGGATGAG

GCCAGAATTGCAAGCTTAATTAACCCTCAACCTATGGGGCAGGTAAGGATGACTACTGTG

CTGCCACTCAAAATAAGCACAAAAGATGATGGTTTAAAACAGCTGAAGCGAAGCCGGGTT

GATGATTCAGGTGCTGAAATGGAAGTCTCTGATGTTGATGCTGTTCAGACAAACATAGGT

GTTGAATGCCTTTCGACGACATCTTCTGATCATTACCCACTGTGGCTCACTTGCAAATCG

GTTACTGCTGTCAACAGAAATCCAAAACGCTTTAAGTTTGAAAATGCGTGGCTCGCTGAA

CCTGATTTTAAGCAACAAGTGCAGCAACGCTGGAAGCTCTATCCTGAAGAAGTAAAGTTC

AAAAGAAAATAG

>MS.gene81720.t1

ATGGAGGTTGAAACTCACTCTTCAAACCCTTCCTCTTCATTCTCATACGACGTTCACCCT

TCGGATTTTCGGAGCTCCGAATATCCCGACCCGACATTTCATCTCTCCCTTCACGATGAT

CCGCCATTAACTCCTCAGCCCTTCCAGTTTTCCGGACCTGACGCGCTTGAAAGCGCGTCT

TCTCATTCCAACGCCGCCGTTCATGTTCAGAAAGTCTACCGTGGTTACCGCACACGGCGG

AGGTTGGCTGATTCCGCCGTCGTCGCCGAAGAGCTTTGGTGGCAAGCGATAGATTTTGTT

CGGTTGAATCATAGCACAATTTCGTTCTTTAATTTGCCTGAAACTGCTGCGTCTCGTTGG

ACACGGGTCAAACTCAATGCTTCCAAGGTCGGTAAAGGTTTGTCCTTAGACGCCAAAGCA

CAGAAATTGGCTTTTCAGCATTGGATTGAAGCTATTGATCCACGCCATCGCTATGGGCAC

AACTTGCACTATTATTATGAAGAATGGTGCAAAACTGATTCTGGTCAGCCTTTCTTTTAC

TGGTTGGATTTAGGAAATGGCAAAACTCTTGATCTTGATCGCTGTCCCCGACCAAAGCTC

CGAAAACAAGGCATAAAGTATCTTGGACCGCAAGAAAGAGAGCACTATGAATACATTGTC

TGCGAGGGAAAAATTATACACAAGCAATCTGGTGATTTATTTCATACGAAAAATGACTCT

GATGATGCCAAGTGGATATTTGTAATGAGCACCTCTAAGAAACTTTACGCTGGCAAGAAA

AAGAAGGGATTGTTTCATCATTCTTCTTTTTTGGCTGGTGGAGCTACCTTAGCTGCCGGA

CGGCTTGAAGCTGAGGATGGAATTCTGGAGTCCATCTCTGCATATAGTGGACACTATCGG

CCAACAGACGACACACTCGATACCTTCTTATCATATCTAAAAACAAATGGTGTCAATCTT

GATGAAGTAAAGGTACGCAAACCAAATGATGACCCTGATATGTACGAGGATGGGAAGTTC

GGCGAGGGAACCTTAACATCTGAAAGTTCTATGACGTCTAACGTATCTGAGGAAACCGAG

AATACTCCATCCGTAGACAAGAAAGACGTGGACAAGAAAGACGCTCCTCAACTCAGTTTG

GAGGTTAGTAGTTATAAAAGGACTCTATCAGGTGGTCTTCAGAGTCCAAGAACGGCCGAG

GTGCCCAAGACTTCTATATTGCAAAGAATCAATTCCAAGAAGGGTTCAAAGTCCTACCAA

TTGGGACATAGACTTTCGCGTAAATGGTCAACTGGAGCTGGTCCGAGAATCGGGTGTGTT

GCTGATTACCCTGTGGAGCTTAGACTACAGGCCTTGGAAATGCTTCACCTTTCGCCAAAG

CTTCCACCCTCTCCTATGTCATACATGTCAGGTGCTCTTATTTCGCCCGCCGTTTCTCCA

ACGCCCAATACCGTGCACATTTACAATGATTGA

>MS.gene81715.t1

ATGGCGGAACATTCATTCAAGTACATCATCGTTGGTGGTGGAGTTTCAGCTGGTTATGCA

GCAAGGGAGTTTGTGAAACAGGGAGTTAAGCCTGGGGAGCTGGCAATCATATCCAAAGAA

GCGGTAGCACCTTATGAACGTCCTGCTCTAAGCAAGGCTTACCTTTTTCCGGAGTCTCCT

GCTAGACTTCCTGGGTTCCATACCTGTGTTGGAAGTGGGGGAGAAAGATTGCTTCCAGAA

TGGTACAATGAGAAAGGGGTACAGTTGCATCTTAGTACAGAAATTGTAAAAGCAGACCTT

GCTGCAAAATCTCTGACTAGTGCAAAAGGAGAAACATTCAAATACCAGACTTTGGTTATT

GCAACGGGCTCAACTGTTATAAGGTTGACCGATTTCGGTGTAGAAGGAGCTGATGCCAAA

AATATATTTTATTTGAGGGAGGTTGATGATGCCGACAAATTGTACGAGGCAATCAAAGGA

AAGAAGAATGGGAAAGCTGTGGTTGTTGGGGGAGGATACATTGGTCTGGAGTTGAGTGCA

GTTTTGAGACTCAATAATATTGATGTTACCATGGTCTACCCAGAACCTTGGTGCATGCCA

AGACTTTTTACTGCTGAAATAGCTGCGTTCTATGAGGGATATTATGCAAATAAGGGGGTC

AATATCATTAAAGGAACTGTTGCTACTGGATTCACCTCTAATTCCGATGGAGAGGTAAAA

GAAGTCAAACTAAAGGACGGAAGGGTACTGGAAGCAGATATCGTTGTTGTTGGTGTTGGA

GGAAGACCTCAAATATCCCTATTCAAAGGGCAGGTTGAAGAGGAGAAGGGTGGAATCAAG

ACTGATTCCTTCTTCAAAACAAATGTTTCCAATGTATATGCTGTTGGCGATGTTGCTACG

TTCCCTTTGAAATTGTACAATGAGGTGAGAAGAGTTGAACATGTTGATCATGCTCGCAAA

TCAGCTGAGCAGGCTGCGAAGGCCATCATTGCAGCAGAGGCAGGAAAAACAGTTGAAGAG

TATGATTATCTTCCATACTTCTATTCCCGCTCATTTGATCTGTCTTGGCAATTCTACGGC

GACAACGTTGGTGACACAGTGCTATTTGGAGACAACAATCCTGCATCATCAAAGCCTAAT

TTTGGAACATACTGGATTAAAGACGGGAAAGTTATTGGTGCCTTTTTGGAGGGCGGAACT

CCTGATGAGAACAAAGCTATTGCAAAAGTTGCAAGAGCATTGCCTGCAGTGAAGGATGTG

AATCAACTTGCAAAGGAAGGCATTACTTTTGCCAGTAAATTTTAA

>MS.gene81723.t1

ATGGACTCACAAAAGCAACTTTTCATCCTCCTATGTCTTTCCCTAGTACTTGTACTTCTC

TCAGGACAAATGGGGATGCAAGTTGAGGCCACTCGCCATCTCAATCATCCTTCAAAAAAA

TTGTTTGTATTCGGAGATTCGTATGTCGATACTGGCAACACCATAAAACCTATATCCGAT

TCATGGAGAGTCCCATATGGCATAACCTTTCCCCGAAAACCGGCTGGAAGATTCTCCGAC

GGCAGAGTTTTAACTGACTTCCTTGCCAAGTATTTGGGATTGAAATCACCAATCATTCAC

AAATTATGGTATAATAACATAGTGCCCGAACAGGATTTGAAGTATGGGATGAACTTTGCA

TATGGTGGTACAGGTGTATTTGATACATTTTCTTCAGGACCAAATATGACAACTCAAATT

GATTCCTTCAACCAAGTCATCCAAAAAAATGACTATACTCTCTCAGATATCACCAATTCT

ATTGCTTATGTCTCTGTTGCTGGAAATGACTACAATCATTACTTGGCCACAGATGGCTCT

CTTTTGGGTTTCCCATCTTTCATTGAATCAGTGATTAAACAAATAACCACCAATTTGATT

CATCTTCAAAGCTTAGGATTTAAGAGAATTGTTGTGGGTGATTTACAACCTCTTGGTTGT

GTTCCTCAAGCCACTGCTGAAACCTCATTTCAAAGTTGCAATAGTACATTTAATGACCTT

GTAGCTCTCCACAACAACTTATTGAACCAATCTGTTACGAAGTTGAACCAAGAAACAAAG

GAACACACCACATTCACAATCCTAGGCATTTTCGATAGTTTCAGGTCGGTGTTGAATCAC

CCTTCTAGCCATAACATTAAGGAACGTTTGAAGCCATGCTGCATCGGTGTAAGCGATGAG

TATACTTGTGGAAGTGTAGATTCTGAACATACTTCTAATAAATATTTGGTTTGTGAAAAT

ATTGAATCAACTTTCTTTCGGGATCAGTTACACCCAACACAAGCAGGTTGGAATGCTGTG

TATAATGAGTTGGAGAAGAAAAGCCTTCATCAAATTTTATACTAG

>MS.gene81722.t1

ATGGCAAGTTTAGAACTCAACAAACAATCCATAAGATCTTTTGCCTTTGAAGTTGTCAAC

AACGAAATCCAACATCGACTCATCCCTCTACTCCAAAACCAAAACCTAGACCAAGTTGTT

TTTGATTTGCAAGATGTTTTCAAAAGATTCTCTTTTGATAGTATTTGTAGATTTTCCTTT

GGTTTAGACCCTATGTGTTTAGAAACATCCTTACCTATGTCGGATTTTGCTCTTTCTTTT

GATTTAGCATCAAAATTATCAGCTGAAAGAGCCATGGTTGTGTCTCCATTAATTTGGAAG

ATCAAAAGATTTTTCAACATGGGTAGTGAAAAAGAGTTAAAAAAAAGTATTAGCATCATT

AATATGTTAGCAAAAGTTGTTATAGATCAAAAGAGAAAATTAGGTTTTTCCCATCACAAA

GATTTGTTGTCAAGATTCATGAGTACTACAATTCATGATGACATGTTTCTTAGAGACATA

GTCATAAGTTTCTTATTGGCTGGTCGTGACACCGTGGCATCATCACTCACAAGTTTCTTT

TGGTTATTAGCCAAAAATCCTGAAGTTGAGAAAGAGATTTTGTTAGAAGCTGAACAAGTA

ATTGGACCACGTGATGATGAAAAAAATAATTATGGTGTAACGAATTTTGAACAACTTAGG

AAGTTGCATTATTTACAAGCTGCAGCACATGAAAGTATGAGACTTTATCCACCAATTCAA

TTTGATTCAAAGTTTTGTTTGGATGATGATGTTTTACCAGATGGTACAAAAGTGAAAAGT

GGTACTAGGGTTACTTATCATCCTTATGCAATGGGTAGGTTGGAGGAATTGTGGGGTCCA

GATTGTTCAGAGTTTAAGCCTCAAAGGTGGTTGAAAGATGGTGTGTTTCAACCTTCAAAT

CAATTCAAGTACCCTATTTTTCAAGCTGGGTTAAGGGTTTGTATTGGTAAAGAAATGGCT

TTGATGGAACTTAAAAGTGTGGCAATTTCTTTGCTTAGAAAGTTTCATATTGAATTGGAA

GATAATACAATGTTTCATGGTAACCCAAGATTCTCTCCTGGACTAACTGCCACTTTTGCT

TTTGGTCTTCCTGTATTTGTTCGTCCTAGAGGAACCAACTGA

>MS.gene81719.t1

ATGGAAGAGACAATCACGGCACACGTTTTGATTTTGGACAATCACAGTACAATCTTCCTT

CTCCATAACCTTATATGTTCTGATTTTGTTCAACAACACAAACTCACCGGTTACCAACAT

TCACCGCCGTTTTCAAACTCGTTGTCGTCATTCTCTTATGCCTCTGTTTATCAGCATTGC

TACTCTAACCTCAACCATCAATTGAAATTTGATTTCCCTATTTTAGCATCCAGTTCAAGA

GACAATAAATTAATCCTGTTGCCTTTACTTTTAATTCAGTTGATTAATTTAAGTTTCTTT

TTTAAAAAATTTGTTGCATTAGTGGAGAAAACTTTTAGATCTTTTGAAGAAGATGAAGAA

GAGAGTGATCACATAAATGAAAATTGTGAGGATAATTTAACAGAAAATGAAGATGATGAA

CCTAAGGTTGGCATGAAATTTAATTCTACAGAGGAAGTTGACAAATATTACAAGAATTAT

GCAAGATGTTTAGGTTTTGGAGTTAGCACAATCAGTACAAAAAAGGGAGATGATGGAAAA

AAGTATTTTACTCTAGGGTGTAACCGTGCAAGAAGCTATGTGAGTAAGTCCAAAAATCTT

TTAAAGCCAAATCCTAGCATGAAAACACACTGTAAGGCTAGAATAAATGTATGTGTGTCT

TCAGATGGAACAATTACTATTTCTAAGGTTGCTCTTGAACATAATCATGACTTAATAAGT

CCAAGCAAATCAAGATATTTTAGATGCAACAAGAATATAGACCCTAGTATAAAAAGGAGA

TTGGAGGTTAATGACCAAGCTGGGATTAATGTAAGTAGAAATTTTCGGTCTATGGTTGTT

GAAGCAAGTGGATATGATAACCTTACATTCGGGGAAAAAGATGTTAGAAACTACATTGAC

AAAGTGAGGCGGCTTCGACTTGGGACAGGAGACGCTGAAGCCATTCAAAATTATTTTGTT

AGAATGCAAAGACAAAATAGTGAATTTTATTATGTAATGGATGTGGATGACAAAAGTCGT

TTACGAAATGTTTTTTGGGCAGATGCGAGATGTAGGGCTGCTTATGAGTATTTTGGTGAA

GTAATAACCTTCGACACCACTTACTTAACAAATAAATATGACATGCCTTTTGCTCCTTTT

GTTGGAGTAAATCATCATGAATGTATGCGTGGGCGTGCTCCACAAGCCATAATTACTGAC

CAGGATGGAGCAATGAAGAATGCAGTTGAGGCCGTCTTCCCGAAAGCTCGCCATCGATGG

TGCTTGTGGCATATAATGAAAAAGGTTCCAGAAAAATTGAGTAGACTCTCTAACTATGAG

TCTATCAAAATACTTTTGCATGATGCGGTGTATGATTCTTTGAGCATAAGTGATTTCATG

GAGAAGTGGGAAAAAATGATTGAATGTTATGAACTTCATGATAATGAATGGTTGAAAGGG

TTGTTTGATGAGCGTCATCGTTGGGTTCCTGTGTATGTGAGAGACACATTTTGGGCTGGA

ATGTCAACAACACAACGAAGTGAAAAAAGTAAAAAGGTATTTGATACAGTTAAAGACATA

ATGTTCAAGGTATTCTTCAATGAGAAAGACTTTGAAATACGATGTACATGCTGTTTATTT

GAGTTTAAGGGTATTTTATGTAGACATATCCTTTGTGTTCTTAAGCTCACAGCTAAAACA

GAGTCAGTGCCCTCTTGTTATATATTGCCAAGATGGAGGAAGGATATTAAGCGAAGATAT

ACACTTATTAAAAGTGGTTTTGATCAATTAGCCGGAAATGAAGAGTTGCAGCGTGTAGCT

AAAGCCTGCGATGCCTTTTACGGATTTGCTTCTTCATGTATCCATACTGAAAATGATTTA

TCAAAAGTAATGGATAGAATAGAGAACTTAAAACTGGAGATGACATGTCAAGAAAGTTTT

TCAGAGATCACTGAAGGAGATAACTTAGTTCAAAATCAGATGACTAAAATTCTTGATCTT

GTAGCGACTCGAAGTAAAGGGCGTCCTCCTTCAAAAAGGAAGGCTTCTAAAGTTGATCAA

ATTGTGAAAAAGAAGCTTGCTGGAAAGAAAGGACAAAAAAGCAACAAAAAAAGTAACACT

TATCAAAGTCAAGAAGAGGGGCCTAATATATCTAAAGTTCAAGAAAATCAAGAGTTCTCT

ATATCTCAGACCATTGATCTTGATGTGATTGACACACAAGAGAGCATTCAAGTAAAACAA

ATGTTAGCTCCCTTCAATCCAAATCAAAAATATCTTGATGTTAATCAGGTTCCATATTCT

TCTCAAAGCATAGACCACAATGTTTCATATTTAGAACTTTTACAGGCACAACACCATATA

AATGATCATTCATCATCATGA

>MS.gene81717.t1

ATGAAAACAGAAATCAAAATCAAATGGGTGTTGATTCACGGAATGGAGACAAGAAAGAGG

AAGAAGAACAAACAGAAGCATGAAGATTCCAATTTCAGAAACCGGAAACTCGCCGGAGAG

GGAAGAAGAGGTTGTTATCGGCGGAGAGACGGTGGTGACTGGATGCGAAATCATGACCGG

AAGAGGATCGGAGCAGATACGGCGGCGGCATGGACGGCTCACGATCGAAAGTTTGGTCTG

GTTTGA

>MS.gene81769.t1

ATGGACGGAACCTGTAATCAAAATCCACCACTGACTTCCACCGTCGGAGACCCGCCAGAG

CCATCACCGGAGAGCCTCCGTCACATCAGCCTCTCAGGTAGTGTGTGGACTCATCCTCCA

AACGCCGTCACCGGAGAGGAAGAAGAGCGTGTCAGCCACCACCACCACCACCGCCACTCT

TCACCGGAGGAAAACTTCCATTCTCCCTTTAATCTTCACCTTCTTCTCTCCACCTACACT

TCAACAACACCAACAACAGCTTTCTACAACCTTCTCGAAAGTGCGATTAAGATGGCTTGG

ATGGAAGAAAATGATGGGAATGGAAAAGAAAAGGAGTTAGGCGGAGAAAATGGTTTTTTG

AAAGTGAAAGAACCATCTGTTAGTGCTGGTGGAGATGATACTGTTTCGGGGAATAAAAGA

TCTGAAGTCAAAGACGATATTTCTTTCGCCAACATTCTGCGTTCGAGGAACAAGTTTACA

GACTCTCTTGCTTTGTATGAGCGTGTTTTGGAGAGTGATGGTGGAAATGTAGAGGCCCTC

ATTGGAAAAGGGATATGCTTGCAGATGCAGAATATGGGTAGGCTTGCTTTTGATAGTTTT

TCAGAAGCTGTTAAGTTGGATTCTCAGAATGCTTGTGCTCTCACACATTGTGGTATTCTT

TATAAAGAAGAAGGTCGCTTGATGGAGGCGGCTGAGTCATATCAAAAGGCGTTACGGGTG

GATCCTACATACAAAGCAGCTGCTGAATGCTTAGCCATTGTTCTAACGGATATCGGTACC

AACATAAAGCTTGCAGGAAACACTCAGGAGGGTATTCAAAAATATTTTGAAGCTCTCAAA

ATAGATCATCATTATGCGCCAGCATACTATAACCTTGGCGTGGTCTATTCTGAAATGATG

CAATATGACATGGCCCTGACTTTCTATGAAAAAGCTGCATCAGAGAGGCCTATGTATGCT

GAAGCATATTGCAACATGGGTGTCATTTATAAAAATCGAGGGGATTTGGAAGCGGCTATT

ACTTGTTATGAGAGGTGTTTAGCTGTTTCACCTAACTTCGAGATTGCCAAAAATAATATG

GCTATAGCTTTGACAGATTTGGGAACAAAGGTTAAACTGGAGGGTGACATTAACCGTGGT

GTGGCATTTTATAAGAAAGCTTTGTATTATAATTGGCATTATGCTGATGCTATGTATAAT

CTTGGGGTTGCTTATGGTGAGATGCTTAAGTTTGATATGGCTATTGTGTTCTATGAACTT

GCCTTCCATTTCAATCCACATTGCGCGGAAGCTTGTAACAATCTAGGCGTTATATATAAA

GATCGTGACAACCTTGATAAAGCTGTAGAATGCTACCAGCTTGCTTTGTCAATCAAACCG

AACTTTTCACAGTCATTAAATAACCTTGGTGTTGTATACACTGTCCAAGGCAAGATGGAT

GCTGCTGCAAGTATGATTGAGAAAGCTATCGTTGCAAATCCGACATATGCAGAAGCATAC

AATAACCTAGGAGTTCTTTACAGGGATGCTGGTGATATTGCTCTTGCAATTAATGCTTAT

GAACAATGTCTCAAGATTGATCCCGACTCTCGAAATGCTGGCCAGAACCGCTTGCTTGCA

ATGAACTACATAGATGAAGGAAATGATGACAAACTTTTTGAAGCTCACAGGGATTGGGGT

TTGCGATTTAGGAGGCTATATCAACAATTTACATCATGGGACAACTCAAAAGATCCTGAA

AGGCCTCTTGTGATAGGATATGTATCTCCTGATTATTTTACACACTCTGTGTCATACTTC

ATTGAAGCTCCCCTTATCTATCATGACTACACCAAATATAAAGTGATTGTTTATTCAGCA

GTTGTCAAGGCAGATGCAAAAACCATTCGGTTTAGAGAGAAAGTCATAAAGAAGGGTGGG

ATCTGGAAAGATATTTACGGGACTGATGAAAAGAAGGTTGCTGATATGGTTAGAGAAGAT

CAAGTTGATATTTTGGTAGAACTTACTGGTCATACTGCAAACAATAAGTTGGGAATGATG

GCATGTCGACCTGCTCCAGTTCAGGTGACTTGGATTGGTTATCCCAATACTACAGGATTG

CCTACAATTGATTATAGAATCACCGATTCACTGGCAGACCCTCCTGGAACAAAGCAGAAG

CATGTTGAAGAGTTGGTCCGATTACCAGATTGCTTCCTTTGTTACACTCCTTCCCCTGAA

GCTGGTCCTGTTTGTCCAACTCCTGCTCTTTCCAATGGTTTTATTACATTCGGTAGCTTT

AACAATCTTGCCAAGATTACTCCTACAGTATTGAAGGTTTGGGCAAGGATACTGTGTGCA

ATTCCAAATTCTCGCCTCGTGGTAAAATGTAAACCATTTTGCTGTGATAGTGTTAGACAG

AGGTTTCTTTCAACCCTAGAACAGTTGGGATTGGAACCACTACGAGTTGATCTTCTGCCC

CTCATTCTTCTTAACCACGATCATATGCAAGCTTATTCTCTGATGGACATCAGTTTGGAC

ACCTTTCCATATGCTGGGACAACTACGACATGTGAATCTTTATACATGGGAGTTCCATGC

GTTACAATGGCTGGTTCAGTGCATGCACACAATGTTGGTGTTAGTCTTCTTAGCAACGTT

GGTCTGGAGAATTTGGTTGCCAAAAATGAAGATGAATATGTTAAATTGGCCATGAAGTTA

GCGTCTGACATTCCAGCATTACAAAACTTGAGAATGAGTCTTCGAGAGCTCATGTCCAAG

TCCCCTGTCTGTGATGGAGCAAACTTCATCCTTGGTCTAGAGTCAACATATAGGAATATG

TGGCACAGATATTGCAAAGGAGATGTTCCTTCTTTGAAACGCATGGAATTGTTAGAACAG

TCTGGTTCTGCCGATGAGAACTCTGAGCCAACAAGGGTCGTAAACGTAAGTGAGGGCAGC

CCTGGATCTGTCATGGCCAATGGATTCAACTCATCGCAGCCACCGAAACTTAATATTAAC

GGATGCGAAGAAAATGGTGTGTCGTTGAAATTCAGTAGTAAGCAGGGAGTGGTGGGTCCT

TGA

>MS.gene81773.t1

ATGACTGATATGAAATTTGAAGAACTAGAAGTTCTACTGGAACCAAGGGTTGACCTGTAT

GATCTTGCTGCAAATGAATTTGACTTCAGAGAAAATTTTGTGGATATGGGTTGGATGGAA

TACTTCTTTGCAGAAAAGAAACCTATCTACACCGAGCTTGTAAAAGAATTTTGGATGAGT

GCAAGGGTTGATAATGGGAGCATCTGTGGTTATGTTCAAGGAAAGGTAGCTATAATCTCT

TTGGAATTTTTGCCACAGGTTATTAAGTGCTATGCTGAAGGTGTGAAGTTTTCTGATGAA

TGGAATGAGGAAGAAATCAAAGAGGAACTTCAACGAATAATTTTCGAGAAGCCAAACGGA

AAGAGTTATTCGGACTTGAATCCGGAATACAAGGTTCTATACAAAATTATCTCTGGATGT

ATCTTACCTCTAAGAAATTCTTCCAAAATAGTATCTCGGAGTTATAAGTTTATAATGTGG

CACTTTGCCAAAAAGATAAGTATCAATCTTCCTGAGTTGATACTTGATTTTTTGAAGAGA

GCTGTTTGGTCCTCAAGATCTGGAAAGGTTAAGACTATCCCTCTTGGAAGAGTGATTTCT

GGTTGTCTTGAAACTTTTGGAATCATTGGTAGTCTACAAAGATCCAAAGACTTCAGTATC

GTACAAGCCTTAAACCCCTCTTTTGGGCCACTTTTTAACCAAATAACTCTGGCAAGGTTG

AAAGTTCGTTCGCGAGATGTTGTTGAAAAACTTCTTAAAGAAGATGAAGACAATGAAGAA

GTCATATTGGAGTACCGTAAAACTATAGCTGAAGATTTTCTCAGAGCTAATCCAAATTAC

ATATTTCCAACTGTTCGGTATGAAGAGAAGACTATGACGAGGACAACAAAGATATAG

>MS.gene81729.t1

ATGGAATCACAACCTCCGGTCACCGGAACCACCGCTAGAACCACCACCGAATCCTCACAA

TCTCAACCACAACAACAACAACCACCACCCTCTCTCCCTCCACCCACATCCTCTACCCCT

CCCATCTCAGCACCATCACATCCTTCCCCAATCCCTAACCCTAACCCTAATCTAATCCAA

ACTCCAAACCCTAAACCCCCAACTCCACTTCCTCCCCAATCACGTCCTCCTCCGTCTTCA

TTCACCCGAAATCCTCCTCCGACACAGTCACATTACTCTCACTTCTCATCAATCCCACCT

TCCGCTAATCCTTCACAAGCTTCATCTTTTCCGTCCAATCCCGCTTCATCGATTTCCTCG

GCTTCTGCGCCGAGAGGCGGTATGGCAATTGGTGTACCCGCGCACCACCAGAGCCCTTCG

CCTCCGTTTTCTTCTTCCTTCAGTCAGCATTTTGGTGGAATGGGTCGTTCTGATTCTACA

ACAAATTCTAACACCTCCCAGGTGCGAGCGCCAATGCAAGGAATGGGGACGTTAGGATCT

TTCGGTTCGAATTCACAAATGCGGCCAGGTGGAATGCCTCCACATCAACAGAGACCTGTT

CAATCGTCTCTTAGACCACCGCCGCCTTCTGCTCAAAATAATCAACCTGCTGGTTCTCAG

AGTTTCCAAGGTCATGGACTTATGAGACCGTCATCTGTGGGACCACCTTCAGCTCCATCT

CCAAGTGCATCACAAGGTATGCAGTCAATTAATCAGCCATGGTTGTCGTCTGGGCCACCA

GGGAAGCCTCCTTTGCCATCCCCGGCCTATAGGCAGCAAATAAACCCACAATCCTTGCAG

CAAAGGACGCATATTTCCCAGCAGCAGCAATCCATGCCAACAGCTTCACAGCAGCAGCAA

TCTTTGCCTTCTAATCAAACCCAGGAGCATTTTGGGCAACAAGTTTCGTCTTCAAGAGCT

CCACATGTGCCTCACCAGCCACAGGTTACAAGGCTACAGGGGCCAGGAAATCAGAAACCT

TCATCACTTGTGGCTGGACAGTCCGGTGCAGTTCAACCAGGGAGTCAAAGTAGATTACCA

AATACTTTACCAAATGCAGATATAGAAGAATCGGGCAAGAGTGTTCTCAGCAAAAGAAGC

ATCCATGAGCTAGTCCATCAGGTTGATCCATTGGAGAAGTTAGATCCTGAAGTTGCAGAC

ATTCTTGGCGATATTGCAGAAAATTTTCTGGAGTCT

>MS.gene81752.t1

ATGCCAATCTCATGTTCACTCTCATCATCATTCATTCTTCCAAACCCAAACCCAAACCTC

CATCCAATCCAATTCCAAACCAAATTCCGAACCAAAAACAACCACCACTTCCCGAACCTA

AAAATCCGAAGCACCTCCCTTCGAACCAACAACAACAACACAATCCAATTCCTAAAACCA

TACCTAACCTCCCAACAAAAACCAATCCTCTACGGTTGGCTCTGCAGCGCAATCTCCGTC

TACTCCCTTTCCAACCTTCTCTCCAAATTCTCCGCCGTCACCACCGCCACAACAACCGTC

GACGTCACGCAGGGATTCGCGCTAGGCGGTTTGGTTCTTGTTCGGTTGATTGCCACGTAT

GCGCAACACGCGCTTCTTTGGGAAGCTTCGTTGAACGCGGTTTATGAGGTTCGTGTTCAT

GTGTTCGATCGTGTGTTGCAGAGGGAACTTGCGTTTTTTGAAGGTAACGACGCCGTTTCG

AGTGGGGATAATGCTTATAGGATTACTGCTGAAGCTTCTGATCTTGCCGCTACCTTATAC

GCTCTTCTTAACACCATAGTACCAAGTTCTCTGCAATTTTCTGCAATGATAATGCATATG

TTGGCTATAAGTCCTGAACTTTCTTTGATATCAGCTATGGTTATTCCTTGTATGGTTCTA

GTTGTTACCTTTCTTGGTCAAGAACTTCGCAAAATATCTAAGAAGGCACATATTAGCATT

GCTGCTCTCTCAGCTTATCTGAATGAGATGCTGCCCGCGTATCTCTTTGTGAAAGCGAAC

AACGCAGAGTCACTTGAGAGTGTCAGGTTTAAGAGACTGGCCCTGATGGACTTTTCTGCA

ATGCTGAACAAAAAAAGGATGAAAGCAGTAATTCCCCAGGTTATTCAGGCTATTTATTTT

GGAGTTCTTTCCATACTTTGCGCAGGTTCGGTGGTGATTTCAAGAGGTTCATTTGATCGC

TGTAGCTTGGTTTCATTTGTGACTTCATTGCTTTTCTTAATTGAGCCTATTCAGGATGTA

GGAAAAGCTTACAATGAATGGAGAGAAGGAGAGCCAGCTATTGAACGCTTGTTTGCTATG

ACAAAGTTCAAAAATAAGGTGGTTGAGAAACCAGATGCTGTTGATTTGGACTATGTTACA

GGGGACCTGAAATTCTGTGATGTCTCCTTTAAGTACAGTGATGGCCTACCTCATATCTTA

AATGGTTTGAACCTTCACGTTAGGCCTGGAGAGATAGTTGCTATTGTTGGCCCGTCTGGG

GGAGGAAAAACAACACTTGCAAAACTGTTGCTTCGGCTTTATGACCCTATATCTGGTTCT

ATACTGATTGATAACGAAAACATCCAGAACATTCGTTTGCAGAGTTTAAGAAGACATGTT

GGTGTAGTTTCTCAAGATATAACACTTTTTTCAGGTACAGTTGCTGAAAACATTGGTTAT

AGGGATCTGACAACAAAAATTGACATGGAGAAAGTGAAGCATGTAGCTCAAACTGCTTAT

GCAGACGAGTTTATTAGAAAACTTCCAGAAGGATACAATACCAATATTGGACCAAGAGGC

TCAACTTTAAGTGGAGGCCAGAAGCAAAGGCTGGCTATTGCAAGGGCTTTCTATCAAAAT

TCTTCTATATTGATTCTGGACGAGGCAACTTCTGCGTTGGATAGTAAATCTGAGTTGTTA

GTGAGACAAGCTGTGGAGCGTTTAATGGAAAATCGCACGGTGCTTGTGATCTCTCATCGC

TTGGAAACCGTTATGATGGCTAAAAGAATATTCCTCTTGGATAATGGAAAGCTTGAAGAG

CTGCCTCGTTCTACTATGTTGAATGGTCACATGGATTCATTGCTATCTTCTGGTCATATT

GTTTGA

>MS.gene81743.t1

ATGATTCATTTTGTTGCTGTTGTTCTTGCAATTGTAGAGGAAATTGTGAATGTCATTGTT

GAAGGAGGTGTTGTTCAGGCTTTGATTAAACATCTTCAGCCGCCAGCTGAAAACAATTAT

GTTCAGAAGCCGTTACCATTTGTACATGAGGTTGAGAAAGGGAGTTCTTTCACACTTGGA

GTTCTTGCTGTTAAGGTAACTTCACTTCCACCTTTTCTCTATTCTCATCATTCAATATTT

CCTTCTCAAAACATTGTTACGTTGTGGAGGAATGATAGGGTAGAGATGATAGTAAACGAC

CAAGGGAATAGAACAACACCTTTTTATGTTGCATTCACTGACTCTCAAAGAATGATTGGT

GATCCTGGTTTCAATATCGCTGCTTGCAAACCAACCAACATTGTTTTCGTTTTGACTATG

TCGATTTTGGACAGTTTTCTCATTGTTGCCAAAGACCAAGAAGTTGCTGATGCTCTTATC

CAACTTAAAGTTCAAGACAATTTGACTCCTGATGTGTGCAAGGCTGGAAAGGGGAGGCTG

CCTGGATGCTTTTCAAGGGTTTATGGAATGCAAGTTGCTCCGTATGTCATGTTGCGTGAT

CCAAAAAATAATGAGATTGAAGTGAAAGTTGAAAAGAAAAATGACAGGGTGTATTTTACG

GATGGTTGGTCCATGCTGAAGAATTTCTACAAAATTCAAGGTGGATCTTGGATGACCGTC

ATATACGCAAACAAAAACCTTTTTTTGATTAAAATGCATGATCTTTATAATGAAGAGGTT

TTGTATCCTGTCTCCAATCCTCCCAAGAGGTTTCTGTTGCAAATTGGAGCTGCGTTATTT

CCGACTGTCACATGTTCCCCCAATGTTCTTTTCCTCCCTACCACCTTTTATCATACGTAT

ACAAAGGTGCTTACAAATGACGATGTTATGTCCGGTAGCTTGAAGTTAGGTTGGTTAGGA

TTTTGTCAAGCGGCGTTTCCATCTGAAGCATGTGAGATGACTGTTGTTGATTGTTTGGGA

TGCAGCTGGAAATGTCTGTTGAAGTTGAACCTTACTACTGATATGACCTGCAATTTATCA

GGTGACTGGCTGAAGATTTGTGATGCGCGTAACTACAAAATAGGGGATGTGGTTAAGCTT

GGTGTCATGGGGGAGTATAATAATACTGTTGTTTATCTCACTGCACCACCAATCCGTTTA

GAATCAATCAGCCACTGCGCTGGAACTATAGCTTCATATTCGTGTTCTCTTTTCTTTAGG

TTTGATATGGGAGATGTAATTATTGATGGTAAATCCTGA

>MS.gene81770.t1

ATGTCAACAACCATTGATGTCCCAGAACCAAGCAACATTAACAAGGAAAAAACAATTCTA

GTTGCACCTACAAGGCCTGGAGGATGGAAAAAGGGAACAGCCATAATGGATTTCATTCTA

AGGTTAGGTGCCATAGCAGCGGCTATTGCCGCTGCCGCCTCAATGGGAACAAGTGATCAG

ACTCTCCCTTTTTTCACTCAGTTCTTTCAATTTGAAGCTAGCTATGATAGCTTTACAACC

TTCCAGTTTTTTGTTATTTCAATGGCAATAGTAGCTTCCTACTTAGTTCTATCCCTACCA

TTCTCTGTCGTAGCCATCATTCGCCCCCATGCGCCTGGACCAAGGCTTTTCCTTATTATC

TTAGATATCGTGTTTCTTACTCTGGCCACTTCAAGTGCTGCTGCAGCTGCTTCCATAGTT

TACTTGGCACACAATGGAAATCAAGACACAAACTGGCTAGCCATTTGCAACCAATTTGGA

GATTTCTGTGCACAGACAAGTGGAGCAGTAGTTGCATCATTTATTACTGTGGTTGTTTTA

ATCTTGTTGGTTGTGATGTCTGCTTTGGCTCTTGGGAAGCATTGA

>MS.gene81750.t1

ATGCCACAACTAGATTTCGAAACCCTAGTTTCCGCCTTATCCGGCGTTGCCGCCGACCGG

AAAGTCGCTTGCGAACCAACTGATGAGGCGGAAGACTCACCACCGGAATCATTCTGGCTC

TCTAACGACGCTGAATACGACTGGTGGGACAGAAACGCCGTCTACGAACGAAACGAATCA

ACAAAAGCAAGCTCAATTTCCATAAACCCTAACTCCGCTTCAAACTCTCAACGATTCTCC

AAGAATTTGAAGAAATCAAAAGCTACGACAATCATCGGTTTACCGAAATCACAGAAAGCT

ACGTTAGCAGAAGCTAGAGGCTGCCGTCGGAATCATCACAAGCCGGGAAACACGAGGTTG

TTTCCGAAACGAAGTGCGTCGATCGGAGGAAAATCTGATTCTACTGTTTTTGAACCTTCT

TCGCCGAAGGTTTCTTGTATAGGTAGAGTGAGATCGAAACGTGGACATAATCGAAGGTTG

AGAACTCGTCAGAGATCGATTTCTTCCACCGCTACCGGCGTCTCCGCCGCCGGCGTTGTA

AGACAGAAATCTTCACGTAGTACTCAAAGAAAGAAGAAAACTGGTTTTATCGAAAGCGTT

TGCGCGATTTTCAGGAGTCACCGGAGAGAGAAAACTGTTCAGAAATCGATTCTTCCGGTG

GAAGATTCTTCAACGAAGAAGAAGAGAAACAATGGAAGAAAAACGCGGGAAGGAAGTACG

GTGAGTAGAACGGTTGAAGAAACGGTTCACAGTGAACCGGCCGGTTTGGGTTCGATGAGC

CGGTTTGCGTCGGGGCGTCGGTCCGAGTCATGGGGAGTAGGTGAAATTGAAAACCACGTG

TGA

>MS.gene81767.t1

ATGGCAATGGTAAGTGGAAGTATAAGGCAGCAGAGTGTGTGCTTGAGTATGCAGCTGAGA

GGAACAGGATTGAATTCTCATGTTCCACTGCCTTCTCAGCACTCCCTCATTCATAGTCAT

CACAACAACAACAACAACCATTCTCTCGTGCTTCATAGCCGCTTCCATCTCACGGTGGCG

GCACCCAAAGTTTCCGCGGGAAGCTACAGCCCCACCGACGACGATGGAGTCTCTCTGGGC

ACCATGAAGCTGCCTGTCAACATAGACCTTCAGAGATTCGATTCCCTGCTCTTTCAGTGG

GCAAACAGCCTTTGCCAGGGAGCCAGTCTACCACTTTCCACGCCTCTCAAGGTTGACAAA

ATACCCGGTGGAGCAAGATTAGGGTTTATAGACATAGGGGAAACAGAAGTTCTTGTGTAT

ATTGACTGTTTGGTTTTTAAACCAAATGAAAGTTCTCCTCCAGTTTTCCAAGCGACCAGA

CATGGGCGCATGAAAGATAAGGTGCCGCCGGGTGAGCCCAGAATCATGAGGAGCCTTATG

GAAGCTCTTCAGAAGTCAGTTCAGATTGCCTCATTGTGA

>MS.gene81772.t1

ATGCCATTTCACCAACACCAACACAATCACCATCACCATCACCATCACCGAGCCCTAAGT

TGTACCAAAACGTCATTTTTAAAGATTGCGGCAATAGACAAGGCAATAAAATCCCAACAA

GTAGTCAGAGAAATTACGGAAAAATATCCTTCTTCTGACCCCATCAGAGAGTGTTTTGAT

GATTACAGTACCGTGGTTGCTGAGGTAAAAGGTGCATTGGGTGAGGATCCGGAAATGATA

GGCTTGGCTGTCAAATATGCTGGTGATGCAGTTGACCAGTGTGAACGTAGCTTAGCTAAC

GAAAAGATTGTTAATATTTCTTCCATTGCTACACTGAATCATGAGATGGAGTTGTATACT

GATATTGTAGTGGTAGCTGGAGGCCATCTATGA

>MS.gene81771.t1

ATGTCAACCAAAATTGATATTCAAGAATCAAGCAAAGATACTAAAGGAAAAGCTGTTTTG

GTTGCAGCACCTGCAAGGCCAGGAGGATGGAAAAAGGGCATAGCCATAATGGATTTCATT

TTAAGGTTAGGTGCCATAGCAGCAGCTCTTGCCGCTGCCGCCTCGATGGGAACAAGTGAT

CAATCTCTTTCTTTCTTTACTCAGTTCTTTCAGTTTGAAGCTAGCTATGATAGCTTTCCT

GCATTCCAGTTTTTTCTTATTGCAATGGCTATAGTGGCTGGCTATTTGGTCTTGTCTCTA

CCTTTTTCTATAGTAGCCATCATACGTCCCCATGCAACTGGACCAAGGCTTTTTCTCATC

ATCCTAGACACCGTATTTCTTACTCTGGCTACTGCTAGTGCTGCTTCAGCTGCTTCCATA

GTTTACTTGGCACACAATGGAAATCAAGACACAAACTGGCTTGCCATTTGCAACCAATTT

GGAGATTTTTGTGCACAGACAAGTGGAGCAGTTGTGGCATCATTTATTACTGTGGTTGTT

TTAATCTTGTTGGTTGTGATGTCTGCCTTGGCTATTGGGAAGCATTGA

>MS.gene81736.t1

ATGGCCACTGGAGTTAGCAGAAAAATTTCTGCAGCATCAGCTCGTTCTCACACCAGAAGG

GCCAACAAATCCTCTTCTTTTCAGCTTCCTTCAGGAATTCTTAGAACAACACTAGCAGTG

TTGTTTATTGGGGTTATAGCATGGGCTTATCAAGTTACGCAAGCTCCTCCTCCAAAAATA

TGTGGCTCTCCGGATGGACCACCTATAACAGCACCAAGAATCAAACTAAGAGATGGAAGG

CATTTGGCATACAAAGAGCACGGTGTTCCTAAAGACGTGGCAAAGCATAAAATCATCTAT

GTCCATGGTTTCAGCAGTTGCAGGCATGACGCTGTGGTTGCCAACACCCTATCACCCGAT

GTTGTCAAGGAATTAGGGGTCTACATTGTATCCTTTGATAGACCTGGTTATGGAGAAAGT

GATCCCGATCCAAATCGTACATTAAAGAGCATTGCCTTAGATATAGAAGAGCTTGCTGAT

CAATTGGAATTGGGGTCCAAATTCTATGTCATTGGTACTTCCCTGGGTGGGCAAATTGTT

TGGAACTGCCTTAAGCACATACCTCACAGGCTGGCAGGTGCAGCGCTCTTGGCCCCAGTC

GTCAACTTCTGGTGGCCTGATCTTCCTGCAAACTTAACGGCCGAAGCCTATTCCCAATGG

AAATTACATGACCAATGGGCGCTTCGTGTTGCTCACTACACGCCATGGCTAACATACTGG

TGGAACACTCAAAGATGGTTCCCAATTATTAGTGTGATTACTGGTAGTCCAGACATCCTT

TCGAAACAAGACAAAGAGCTTGTAAGACAACAAGGTGAATATGAAAGCCTCCACCGTGAC

TTGAATATTGGATTTGGAAAATGGGAATATACTCCTTTGGATCTTCAAAATCCATTTCCA

AACAATGAAGGTTCTGTTCATCTTTGGCAAGGAGATGAAGATATTATGGTTCCTGTCACA

CTACAACGATACATCGCGCAAAACCTTCCGTGGGTTCACTACCATGAACTTCCAGGTTCT

GGCCACCTCTTCCCTCATGCTGGTGGTGTGAGTGAGACTATCATTAAGTCACTTTTAGGT

GTGAAGTAG

>MS.gene81749.t1

ATGGCCTTGCAGATTTACTTCTCAATCCTTTCCATAGCTGCTTTGGCTCTATGCACTTCA

GCAAATCACGATTCGGTTAATTTTAGCTATACTGGACCAAATGGTCCTGTGAAATGGGGA

ACATTAAGTCAATCATTTGCAGCATGCTCAAATGGGAAAGCACAAAGTCCAGTGGATCTT

GCAATGACAAATATTGTTGTGAACAATGTATTGAAATCCTTAGACAGAAACTACCTTCCT

ACAAATGCCTCACTTGTTAACCACCAATACAGTATTGGGGTGCATTTTGAAGGAAAAGTG

GGAGATATTAATATAAATGGAATGAATTATTCATTGAAACAACTTCATTGGCATGCACCT

GCAGAGCACAGGGCTCATGGTCGTATACATGAAGCCGAGCTCCACTTAGTTCACTTCACT

GAAGATAACAATAACATAGCAGTTGTGGCACAGCTCTACAGATTGGGTGTTCCTGATCCT

CTAATCTCCAAGATTGAAGACAACTTCTATAAGCTAGCCAATGAGAGCCGTGCAGGCAAT

AAAGATGCTAACATTGCTCTTGGCACCTTTGATGTAAAGGAATTAAATAAAAAGATCTAT

AGATATTATAGATATGTTGGCTCTCTCACTACTCCTCCATGCAAAGAAGGTGTCATTTGG

AACGTCATTGGCAAGGTTAGGACACTCTCCAAGAAACAACTTGAACTTCTAAAGGCACCA

CTCGGTGTAGAGTTTCAGCACAACGCAAGGCCTCTTCAGCCATTGAATGGTCGCAAAATT

GAGATGTACAACTACCACACATAG

>MS.gene81744.t1

ATGGGTTCCAAGGCGATGGCTATGGCAGCAGAGAAGATAAGCACGGCGGTGCGACGGCAA

GCCGTTGAGTTAACCGATGCCGCCGCCTCTAGAATACACTTCTTGCTTCAGCAACGGCAA

AGACCGTTCTTAAAGCTCGGCGTCAAAGCTCGCGGATGTAATGGTTTATCTTACACGCTA

AATTACGCAGATAAAAAAGAGAAATTTGATGAATTGGTTGAAGATAAAGGTGTTAAGATT

TTGATTGATCCTAAGGCTCTTATGCATGTTGTTGGAACCAAAATGGATTTCGTTGATGAT

AAACTAAGATCTGAGTTCGTTTTCATTAATCCAAACTCCAAAGGCCAATGTGGTTGTGGT

GAATCTTTCATGACAAAACCAAGTACTGGAGCCTCTAAGCAAGGAAATGGTTGA

>MS.gene81755.t1

ATGCGTGGGGAGGAGCTCCAGAAAAGTGCTCTCTTAGCTCGTGCATTGGTTCGCACTACA

ATAGCAAGATACCAGACAAATTGTCAAATTGATCCAAAATCCTTGAAGTTTAGGAAGAAC

GATTATGGCAAACCTGAGGTAGACTGGCAATACGCTGACGATCGGAGCCTACCACCACTA

CATTTTAATATCTCACACACTTCTTCTTTGATAGCATGTGGAGTAACTGTCGGCTCACCG

ATTGGAATTGATGTGGAAGAGAAGCAAAGGAGGTTAAAGAATGACATTTTAGCCTTTGCT

CGTCGATACTTTTCTCCACATGAAGTAGAAATGCTAGCTCACATTGTAGATCCTGAGCTT

CGGCGTCAGGAGTTTATTAAATTATGGACTCTGAAGGAGGCATATGTAAAAGCACTGGGA

AGGGGCTTCTCAGCATCTCCTTTTAATACTTTCACTATTCGATTAAGAGACCATGTGAAA

GGAGGTATCCATGTTCCACCCCATGTGATTTCTAAGGAAGCTGAAATTACTGTTGAGCCT

TCTGGTGACCTGAAGAATCTCTCAAGCAATTGGCATTTTGTGCTTTTAGAGTTAATTGGT

TCTCATTATGCCGCCGTTTGCATAGAAAAGGACAACACCAATGCAGACAAAGGGAGCATT

CCAGTAAATCTGATTATACGGAAAACAATCCCATTTGTTGAAGATGAATGTATTTCCGGA

ACAGACTCTGTGGAAGTCATTGGGGGCTTGAGTAAACTGCTAGTGTGTTGA

>MS.gene81763.t1

ATGTTGTTCTGCGTGCATTTCTTGTTGAGCACGCATGTCTTTTTAAGCAGTGTGCACATC

GTCCATGAGTGCTTCTTGCTGGATCGGTTATGGATCAGAAGGACATTCTTCTTGAACATG

AGTATTATGTTGTTCTACGTGCATTTCTTGCTGAGCACGAATGTCCTTTTCAGCAGTGTG

CACATCGTCCATGAGTGCTTCTTGCTGGATCGGTTATGGATTGGAAGGACATTCTTCTTG

AACATGACAGGTGTGTCCTTTGCTGGTTCATTAACACTTCTTTTCCTTTTTGATGTCACC

TTGCCTTTAGCCTTCTTCAGTTTACATGCAGAAGGCACATCAGCCAAGGCAAGGAGATGT

GCCTTGACCGATGTGCCTTCTGCATGTAAACTTAAGAAGGCTAAAGGCAAGACGACATCA

AAAAGGAAAAGGAGTGTTAATGAACCAACAAAGGACACACATGCTGAAAAGACATGTGTG

CTCAACAAGAAATGCACAATCGATCCAGCAAGTACACTTCTGACCGATGTTCAGGCTAGA

TGTCGGGAGGAAAGTAAAGGCAAGAGGATAACAAAAAGGAAAAGGAGTATTAATGATCTA

GAAAAGGACACATATGCTGAAAAAGAAATGTGTGCTCAACAAGAAATGCACTCAGAACAA

CATATTACTCATGATGAAGAAGAACATCCTTCCGATCCGGAACCGATCCAACAAGAAGCA

ACCCTGGCCGATGTGCATGCTAGATGTAGGGACGAGGCTGAAGGCAAGAATATAGAAAAA

GGCAAAAGGACCTACATTCAAGGAAAAAATTAG

>MS.gene81737.t1

ATGTATGATGCTACTTGGGTACCATCAACTTCTAATCCATCGGGCATTGGTTCTTCACTA

GCTTTTGGTGGCACTACAACTCCAAAACAGGCTACGACTGAAAAGGGATTTGAAACCGAA

CCGTCAGCTGCTCATGGGACAAAAGAGACCGAGCCAGCTCAGGTTGCTCTTCAGCTTGAC

ACAATTGTTCAGAACCAAGGGAACGAAATTGCTGATAACACAGATGCAACACAGGTTGAA

ACACATGAAGATGAAATCCCAATACCTGTGAACCAAGGGGAAGCAATTCCGATCCAAGAG

GAAACACATGATGCGCAGAAGGAAGTAGTTACTATCAGATCAGAGGTTGTGCCGGCAGTT

TTACCGACTCCTATGGTAATTCCCACTTCTCATATTCCTGTACTTCAAAAGAAAAATTTT

AGTATGCCTCTTGAGAATGTTTCCGATGAGCTAATTGATAATAATCTGGAAGGAAATTAT

ATGCATGTTTTATCTCCCGTAAAAAATATACATCTTGATATCAGTAGCATTGTGGCCCCG

GATAATGCTAAGATTGATCCGGTGCTACAGAAAGAATTAAACTTTATGCAGACTTGGTTG

GCAAAGGCAGCAGAGACCGAAACACCATTTATAGAGGTTGTTTCCAAGTCTGATGAAGGA

TAA

>MS.gene81754.t1

ATGAACACTTCCCACCGCCGTCACTCTACATCGTCGCCACCCGTTCCCGGCGAATCAAAA

CAAAACCCAACAACAGCATGGAACTGTTACCTCCGAGAACTCTCAAAGCAAAGAAAATTC

ATGGAAGCATTAACCGTATACCGTCACATGCTCCGTTCATCCTTCTTCCCCAACACATTC

ACCTTCCCTGTTCTCCTCAAATCCTGCGCCCTCCTCTCCCTCCCCTTCACCGGTTCCCAG

CTCCATTCCCACCTCCTCAAAACCGGGTCACAACCCGACCCATATACCCACTCTTCCCTC

ATCAACATGTACTCCAAAACCTCACTTCCTTATCTTGCACGCAAGGTGTTTGATGAAAGT

CCTCTAAACCTTACGATCTCTTACAATGCCATGATTTCCGGGTACACTAATAATACGATG

ATTGTTGAGGGGGTTAAGCTTTTTCGGAAAATGTTATGTGAAAATCGGTTTTTGGTTAAT

TCGGTTACAATGTTGGGTTTGGTTTCGGGTATTATGGTACCGGAGAAGTTAAGACTTGGG

TTTTGTTTACATGGTTGTTGTTTTAAGTTTGGTTTTGAGAATGATTTGTCGGTTGGGAAT

AGTTTTTTGACGATGTATGTTAAGTGTGGTGAAGTGGAGTATGGGCGGAAGGTGTTTGAT

GAGATTTTGGTTAAGGATTTGATTACGTGGAATGCGATGATTTCGGGGTACGCGCAAAAT

GGGCACGCACGGAGGGTTTTGGAGATTTATCGTGAGATGAGGAAGGTGGGTGGGGTGAAT

CCTGATCCTGTTACACTGCTTGGTGTTTTGTGTTCTTGTGCAAATCTTGGTGCGCAAGGA

ATTGGAAGGGAGGTGGAAAAGGAAATTGATCGATTTGGGTTTCGTTCGAATTCTTTTTTG

ATGAATGCTTTGATTAATATGTATGCTAGGTGTGGTAATTTGGTTCATGCTCGTGAGGTT

TTTGATTGTATGGATGAGAGGAGTAAGAGTGTTGTATCTTGGACTGCTATTATAGGTGGG

TATGGGATTCATGGGGAAGGTGAGACTGCGGTGGAGCTTTTCGATGTAATGGTTAGGTCA

GGCGTGAAGCCGGATCGAACAGTTTTTGTGAGTGTTCTTTCAGCTTGTAGTCATGCTGGG

TTGACTGAGAAAGGGTTGGAGTATTTTTACGAGATGGAGAGGAAGTATGGTTTGCAGCCT

GGTCCCGAGCATTATTCCTGCTTGGTTGATCTTTTGGGTCGGGCTGGTAGGCTGAAGGAT

GCAATGGATCTCATAGACTCGATGAAGGTGAAACCGGATGGTCCTGTTTGGGGAGCACTT

CTTGGTGCTTGCAAGGTTCATAGAAACGTAGAATTAGCAGAAGTGGCTTTTCAACATGTT

ATTGAGATTGAACCTACAAATATAGGTTACTATGTTTTGTTGTCAAATTTGTACTCTGAT

ACTAAAAACTTAGAGGGGGTTTTGAAAATTCGAGTCATGATAAGGGATCGAAAGCTTAGA

AAGGATCCCGGTTGCAGTTATGTCGAATATAAAGGAAAAATGCACCTTTTTTACTCTGGG

GATACAAGTCATCCCCAAAGCAAGGAAATATATAGAATGTTGAATGAATTGGAAAATCTT

GTGAAGGATATTAATGGAAAAGACCACAAGTGTCATAGTAAGAGTGAAGAACCGTCAATT

GGTGCTGGCGTGCACAGTGAAAAATTAGCAATTGCGTTTGGTCTGTTGAACACTAGGCCT

GGAACAGATATTACTGTCATGAAAAACCTGCGAGTGTGTGTGGATTGTCATGTGTTCTTT

AAGTTGGTTAGTAAGATTGTTGATCGCCAATTTATTGTTAGAGATGCCACTCGCTTTCAC

CGTTTCAAGAATGGAGTCTGTTCTTGCAAGGACTACTGGTAA

>MS.gene81774.t1

ATGTCAACCACTATTGATATGGCAGAATCAAGCAACGCTCATGTTAATGAAAAAGCTGCT

TTAATTGGAGCCACTGCAAGGCCTGGAGGATGGAAAAAAGGCATAGCCATAATGGATTTT

GTTCTAAGATTAGGTGCCATAGCAGCTTCTCTTGCTGCAACTATCACCATGGCAACTAGT

GATCAGATTCTTCCTTTCTTCACTCAGTTCCTTCAATTTGAAGCTAGTTATGATAGCTTT

TCAACTTTCCAGTTTTTTGTTATTGCAATGGCATTTGTGGGTGGCTACCTGGTCTTATCC

CTACCATTTTCCATAGTAACCATCATTCGCCCTCATGCACCTGGACCAAGGCTTTTCCTC

ATCATCGCAGACACGGTGTTTATGACATTAGCCACTGCTAGTGGTGCTTCAGCAGCTGCC

ATGGTTTACTTGGCACACAATGGCAATCAAGACTCAAACTGGCTTGCCATTTGCAACCAA

TTTGGAGATTTTTGTGCTCAAACAAGTGGAGCAGTTGTGTCATGTTTTATTGCTGTGGTT

GTTTTAATGGTGTTGGTTGTGATGTCTTCTTTGGCTCTTGGGAAGCATTGA

>MS.gene81766.t1

ATGAATACTTTCTCTCCTTCCTCCACAATTCTACCTTTCCCTTCACATACCAAAAACTCC

ATTTTCTTTGCCCCTTTTAAAGGTAGTAAAACCAACCAATTTTCTTTTTCTAAGTCAAGG

ATAATTGCGTGCTCTTCTCCACAGGATGCAGGCTCCAATGAAATCACACCAAACAACTTT

TGCATTATTGAAGGGCCAGAGACGGTTCAGGATTTTGTTCAGATGCAGGTGCAGGAAATT

CAAGATAATATAAAGAGTAGACGCAATAAAATCTTTCTTCTTATGGAAGAGGTTAGGAGA

TTGCGTGTGCAGCAGCGGCTAAGAAGTATACAAAGAGCTTTTAGTGAAGAGGGAGAGGAA

GATGCAAATGAGATGCCGGAAATTCCATCATCTATTCCTTTTCTTCCTCATGTGACACCC

AACACTTTGAAGAAGCTCTATCTAACAGGCGCATCTTTCATATCTGCAATAATTGTATTT

GGCGGGCTTATTGCACCAACGCTGGAGCTGAAATTGGGTATAGGTGGCACGTCATATGAG

GATTTCATAAGAAGCTTGCATTTGCCTTTGCAACTAAGTCAAGTTGACCCAATTGTCGCA

TCCTTTTCGGGTGGAGCAGTTGGCGTGATTTCAGTACTGATGTTAATTGAGGCTAATAAT

GTTGAGCAACAAGAGAAAACAAGGTGCAAGTATTGCCTTGGAACTGGTTACTTGGCTTGC

GCTCGGTGTTCCACAAGTGGTGTATGTTTGAACATTAATCCGATTTCGGCATCTGGTACT

TCTGTTAGACCCCTGCAGGTTCCTACAACGACAAGGTGTCCAAATTGCTCTGGTGCTGGA

AAGGTTATGTGTCCAACCTGCCTTTGCACTGGGATGAAGATGGCAAGTGAACATGACCTG

AGAATTGATCCATTTGACTAA

>MS.gene81768.t1

ATGACATTACTGACAGAGCGTCTTTTAACGGATTTCATTCAGGGTACTATTGGACAAACT

TCTAATAATGTAATACTTGGGGGAAACAAATCCGATGTTGATGCTGAACAAAAGGTAACT

TCCCGCGAGGGTAAAGAATTGGCTGATGGGCATAATTTCCTTTTCTTTGAAACTGTGAGT

TGCAATATGCTTGATATTCACCTTTTGTTTGAATTATACGATTTCTTTCTACTTATTTTG

AAAAAACACATGCAGAGTGCAATCACAGGCATCAATACAAATTTTCCCGAATACCAGGGA

CCCGAGAAGATTGTCATCCGACGTTACAGTGACTTTGTTTGGTTACGTGATCGCCTTTTC

GAGAAGTTCAAAGGCATTTTCATTCCTCCTCTTCCAGAGAAAAGTGCTGTAGAGAAATTC

CGTTTCAGTGCTGAGTTTATTGAGATGAGGCGACAGGCATTGGATGTATTTGTAAATAGA

ATAGCATCACATCATGAACTTCGACAAAGTGAGGATCTGAGGACATTTTTGCAGGCAGAA

GAAGAGACAATGGAGAGGTTAAGGTCCCACGAGACTGGTATCTTCAAGAAGCCATCTGAT

TTGATGCAGATTTTTAAGGATGTTCAATCAAAAGTCAGCGATGTTGTGCTTGGGAAGGAG

AAGCCAGTGGAAGAATCAGATGCTGAATATGAGAAGCTAAAACATTACATTTTTGAACTG

GAAAACCACTTGGCTGAAGCTCAAAAGCATGCATATCGTCTTGTGAAGAGGCACAGAGAG

TTGGGACAATCACTGTCAGATTTTGGAAAAGCTGTGAAACTTCTAGGAGCTTCTGAAGGA

AATGCACTTGGGAAAGCATTCTCTGAACTTGGGATGAAGTCAGAGATTTTATCAGTCAAG

CTGCAAAACGAGGCACAACAGCTTTTGATGAATTTTGAAGAACCTTTGAAAGACTATGTC

CGTGCTGTACAATCTATTAAGGCAACAATAGCCGAGAGGGCCAATGCCTTCAGGAGGCAA

TGTGAACTGGCTGAAACAATGAAGCTGAAGGAGATTAATCTTGACAAACTCATGTTGATC

CGCTCTGAAAAAGTGGCAGAAGCTGAGCGTGAATATCACGAGGCAAAGAGTGAACAAGCA

ACAAAGACATTTGAGACAATTGTGAAATTAATGAATGAAGAAATAGGGCGTTTTCAGGAA

CAGAAAACCTTAGATATGGGGATTGCTTTCCATGAATTTGCCAAAGGTCAGGCACGCCTA

GCAAACGGTATTGCAGATGCATGGCGAAGTTTGCTTCCTAAACTAGAAGCATGTTCCTCT

TCATAG

>MS.gene81765.t1

ATGCAGATTTTCGTGAAAACCCTAACAGGGAAGACGATTACTCTCGAAGTCGAGAGTAGC

GATACCATCGACAATGTCAAAGCCAAGATTCAAGACAAAGAAGGTATCCCACCGGATCAG

CAGCGTTTGATTTTTGCTGGAAAACAGCTTGAAGATGGAAGAACCCTAGCTGATTACAAT

ATTCAAAAGGAGTCTACATTGCATCTTGTGCTTAGGCTTCGTGGTGGTATTATTGAGCCT

TCTTTGATGGCTTTGGCTAGGAAATACAATCAAGACAAAACCATCTGCCGCAAATGCTAT

GCTCGTCTGCATCCAAGGGCTGTGAACTGCAGGAAAAAGAAGTGTGGTCACAGTAATCAG

TTGAGGCCAAAGAAGAAGATTAAGTAG

>MS.gene81747.t1

ATGGCCATGGCTGGAACAGGAACTCTGAGTTTTTCTCAATTCTCAGTTCATAGGCCTGTT

TCATTTTCCAGAACAAAACTACTGCCCAGAAACAGAATTTGCATTAGAGCCATGTCAGAA

ACAGAAACATCTTCTTCCACTTCAACTTCATCATCAACATCAGTTTCAATTACTCCTCCT

CCAAATTTCAAACCCCCTGAACCTAAACGCTTTGCTATAAGATCTGACAAGACTCTTGAG

ATTCTTGGAGCTTCCCTTCCCTTGCTCTTTCGCTTTGCTACTGGAGTTTTTGTTTCTGGG

TATTCTTTCTCATTTGTTTCCAAGGATGAAATTCCACCAAATGAATATACTTTTCAGCTT

TCTGGCATTACAGTAAAAGAAACATCAAAGATAGGCGCTCGACCAGAGAAGCCTATTGAG

ATATATGAATTTGAAAGCTGTCCATTTTGTCGAAAGGTTAGAGAAATAGTTGCTGTTTTG

GACCTTGATATTCTTTTCTATCCTTGCCCAAGAAACGGCCCGAATTTTCGTGCGAAGGTT

GTTGAGATGGGTGGTAAAACACAGTTCCCTTACATGGTTGACCCAAACACTGGCGTTTCG

ATGTATGAATCAGACGAAATAATTAAGTATTTGGTTGGAACATATGGCGATGGAAACATT

CCTCTTACTTTATCGCTTGGATTTTTAACGTCATTGACTTGCGGTTTTGCTATGCTTGGT

CGCATTACAAAGGGAACATCATATACTCCAGCGAAGTTACCACCGAAGCCACTTAAATTA

TGGGCATATGAGGGATCTCCTTTCTGCAAAATCGTACGTGAAGTACTTGTGGAATTGGAG

CTGCCACATTTGCTTGTCAATTGTGCTAGGGGTAGCCCAAAACGAAATATTCTATATCAG

AAAACTGGACATTTCCAGGTACCTTTCTTGGAAGATCCAAACACTGGTGTAGAAATGTTT

GAGAGTGCAGAAATTATAGAGTACATTAGAGCAACTTACACTCTTTAG

>MS.gene81734.t1

ATGACTTCAGAGGAACATAGAGGAGTTTCCCATGATTTTGGAATAGAAGAGGATGATGAT

GATCTATTTGAAATTGATCTAGAGAGAGTGAATTGCATTCCACCACTACCTTATAATTAT

TGGGAAAGTAATTACTTCATTTCTACAGGAGAAGCTCTTCTTGCCAATTGTTTGCTACCA

ATATCCCATATTTCTAGTGCTGTTCCAGCTTGTAATAATGTTGTGTCTTTTGGAGGGAAC

ACCAATGTTTTCGTTATCACTGAGCCTAAACCATTGGGGGAATATCTTAGGTTACCTTTC

TTGGGGGATTTTGGATTTATAGGTGAGAAAATGAAAGCAAAATTTTATTTCCAGTTTCAG

ACTTAA

>MS.gene81746.t1

ATGGTCCTGTTTTGCATGAAAGATAACAACACAAACACAAATACCACTACAATGACTCAC

CAATCATCTACTAAATCTCATGACCAAATTGATATTGAATTGAAGAAACCTCATCAACTC

AAAGATAACAATATTAAGGAGCAACAACAACAAGAAGAAGATGACACAAACGTTGAAATT

GCATATATGAAGAAGGTGGTTTTGCCTATTGTTGAGGAAGAAGAAAGTGGAAGAGAGAGG

TTGAAGAGACATAGAGTTGAAATGGCTGGAAGAGTATGGATTCCAGATATGTGGGGACAA

GAAGAGTACTTGAAGGATTGGATAGATTGCACAACATTTGATCCTCCTTTGATGTCTGCT

AGTAAAATTGTGACTGCTAGAACAGCTTTGGTTCAAGAAGCTACTAGGATTCAGATTCCA

CTATAG

>MS.gene81757.t1

ATGGCCAAAGACCCCGAGAGCGCGAACCCAAATCAACCACCACCCACAACCCGACCCGTC

GGTGGCACCGAATACGGCTGGTGCAAAGCCGTCCCCGGCGGCACCGGCTCCACCGTATTA

AGCCTCCTTCTCTCAAAACCACCACAAATCCCTCATCTCCAAAACGCCCTTCACAAACTC

CAAAATTCCCACCCTATCCTCCGTTCAAAGATCCGCTTCGACACCACTACCAACACTTTC

CTCTTCATAACTCCTCCAAATCCCACCGTTCAAATCCAATCCTTCGATCTTCAGCAAACT

GAACAAATCCTCAACGCTCACGATCACGACGATCTCGTCAATCCTTTCCACAAGCTACTC

GAACACGAGATGAACCTTGACAGTGACACGCGGCGAGATCGTAACGACGTTGACGTTGAT

GTTATGTACGCTAGCACGTACGCGATTGATGAAAAACGGTTAGCGGTGTTTCTACGGTTG

CATACGTCGGCGTGTGACCGTGCTGCGGCGGTTGCGTTGTTGAAGGAACTGTTGCTGCTT

GTTGCTGGAAGAGGTGATGGTGGTGGAATGATGAAGGAAGATAAGGTGAATTTGGCGATT

GAGGATCTGGTTCCGGAAGGGAAGAAGAATAAACCGTTTTGGGCGCGTGGATTAGACGTG

CTTGGTTATTCGCTTAATGCGTTTCGGTTTTCGAATTTGAGTTTCGTGAATGCTGATTCG

CCACGAAGGAGTAGGATGGTGAGGTTGCAGCTCAATGCTGAAGAAACCAAGAGGTTGCTT

GATGGATGCAAATCAAGAGGAATTAAACTTTGTGCGGCACTTGCAGCAGCTGGAATGATT

GCCGCATGGGTCTCTAAGCGCCTTCCTGATTATCAAACAGAAAAATATGCTGTAGTAACA

CTCATTGATTGTCGCCCACTTCTTGATCCAGTACTTTCCAGCAGCCATTGTGGATTTTAC

CATTCTGCCATTTTAAACACCCACGATGTCTGTGGAGAAACCTTATGGGAGTTGGCAAAG

AGAAGCTACACATCCTTTGAAAATGCTTTGAACTACAACAAGCATTTCTCAGATATGGCT

GACCTGAACTTCCTCATGTGCAAGGCCATTGATAACCCTGGTTTGACACCATCGTCTTCC

CTTAGAACTGCATTGGTATCTGTATTTGATGACCTTGTCATTGATGATTCGAATGAAATG

CATGAAGAGCTTGGATTGGAAGACTATTTGGGTTGCGCTTCCGCACATGGTGTTGGCCCA

TCCTTAGCCATGTTTGACGCCATCCGGAATGGAAAGTTGGATTGTGCTTGTATATATCCT

TCACCCCTTCATTCGAGAGAGCAGATTCAAGAACTAGTTGATCATATGAAGAGAATACTT

GTGGATGCTTGTAACAGTGAAAATCAATAA

>MS.gene81758.t1

ATGACCGAACACAAAGGTAGAACCGTTGGCGGCACAGAATGCAGCTGGTGCAAGGCGGCT

CGCGGCGGCACCGGCGTCGCCGTCATAACATTACGCACTTCAAAACCACCAAACATACAC

CGTTTCCAAACAGCACTTCACAAACTCCAAAATTCCCATCCTATCCTCAGGTCCACTCTT

CTCCAACACACAAACACCTTCTCCTTCCTCACATCCCCCACACCTTTCCTCCAACTAACA

ACCCACGACCTCTCATCACAACAAAACGGCACCATTTCACTCTCCCCTCTCCAACAAATT

CTAGAACTTGAACTCAACAACGACAACGAGTGGCGCGACGCAAAGCGTGACTCAAACGAA

ATGTTTTTCGGCAGTGTTTACGCACTGCCGAACAACGTTTGGGTTGTTGCATTGCGGCTC

CACGTCGTCGCATGTGACCGCACGACAGCGGTGTCACTTTTGGGAGAGTTGCTTGTGCTC

ATGGAAGAAAAGGAAGAGATCCTTGAAGAAAATGATAAGGAAGTTAAACATGAGGTTAGT

TTGGCTATTGAGGATCTTGTTCCTCGTGAGAAAACAAAGAAGGCTTTGTTAGCACGTGGT

TTTGACATGCTTGGTTACTCACTCAACTCACTTAGGTTAACGAATATGAAATTTTGTGAC

ACCAAAGCAACTAGGTTTTCACAAGTTGTAAGATTGCAACTTAATCAAGATGACACTAAG

GGGGTTCTAGCTGGTTGCGCGTTGAATGGGATAAAAGTGTGTGGGGTATTGAGTGCTGCG

GGTTTGATGGCTGCCCATGGCTCAAAACGTGGTTCCAAGAAGTATGGTATTGTTACTCTC

ACTGATTGCAGATCCACACTCCAATCTCGTCTCTCCGACAATTTTGGTTTTTACCACTCT

GCCATTCTCAATTCCCACGAGATGAAAGGAGGGGAAACTCTATGGGAGCTTGCAAAGAAA

ACCTATGGAGCCTTTGCAAACTCAAAGAAATGCAACAAACATTTTTCAGATATGGCAGAC

TTAAACTTTCTGATGTGCAAGGCCATAGAGAATCCAGGGTTGACGCCATCTTCTTCGTTA

AGAACGGCGATCATGTCGGTGTTCGAGGACACGGTTATAGACGACAGTATTAAGAAGCAA

AGAGAGGTTGGTGTAGAGGACTATATGGGATGTGCTTCTGTTCATGGGGTGGGTCCATCT

ATTGCCATTTTTGATACCATAAGAGATGGAAGCTTGGATTGTGCTTGTGTCTATCCAGCA

CCGTTACACTCTAGAGAACAGATGCAAGAAGTTGTTGGTAAGATGAAGGCTATCCTTATT

GATGCTGCTAAGACATTCAAGAAATGA

>MS.gene81745.t1

ATGCTTAGAGGTGAGGCGAGTAGCACGGTGAATGTTCCGAGAAAGGGTAGTTCTAGTTTG

GGTGAGGATTTGTCCGAGAGCTTAGGGGATATATCTAGTGAGAGTAATTACAATCAGGCG

AAGATTAAAGTTATTGGTGTAGGAGGTGGTGGATCTAACGCGGTGAATCGTATGATAGAG

AGTTCGATGCATGGAGTTGAGTTTTGGATTGTTAATACTGATGTACAAGCTATGAGAATG

TCGCCTGTGTTTCCTGAGAACCGTCTGCAGATTGGTTTGGAACTTACGCGAGGTCTTGGA

GCTGGTGGTAACCCTGAGATGGGAATGAATGCTGCGAAAGAAAGCAGAGAGTCGATTGAA

GAGGCGGTTTATGGAGCTGATATGGTCTTTGTTACAGCTGGAATGGGTGGTGGAACCGGC

ACAGGTGGTGCTCCGATTATTGCTGGTGTTGCAAAGTCAATGGGTATACTGACTGTTGGG

ATTGTCACGACCCCTTTTTCATTTGAAGGACGAAAGAGATCCATTCAAGCACAAGAAGGA

ATTACAGCTTTAAGAGATAATGTCGATACTCTAATAGTTATTCCAAATGACAAGCTATTA

ACTGCAGTTTCTCAATCTACCCCTGTAACGGAAGCATTCAATCTTGCTGATGATATTCTT

CGACAGGGCGTTCGTGGCATATCTGATATTATTACAATCCCTGGGATGGTAAATGTTGAT

TTTGCTGATATTCGAGCTATAATGGCCAATGCTGGTTCTTCACTGATGGGGATAGGAACT

GCAACTGGGAAAACAAGGGCAAGAGATGCTGCGTTAAATGCTATTCAGTCTCCTTTACTG

GATATTGGTATAGAGAGGGCTACTGGAATTGTGTGGAACATAACTGGTGGGAGTGATCTG

ACTTTGTTTGAGGTAAATGCTGCTGCAGAAGTTATCTACGACCTCGTGGACCCTAGTGCT

AATTTAATTTTTGGAGCAGTAATAGATCCTTCACTTACTGGTCAAGTAAGCATAACATTA

ATTGCAACCGGATTCAAGCGTCAAGAGGAACATGAAGAGAAGCCACTACAGACTAGTCAG

CTCACTCAGGGAGATACAACTGTCAATTTCAATCAGCGACCTTCCTCGTTCTCTGATGAT

GGTAGTTTGTTTGAGATACCTGATTTCTTAAAGAAGAAAGGACGCTCACGCTATCCTAGG

GTTTAA

>MS.gene81739.t1

ATGTCTACACAAAAGAACCTTTGGCTTTTGCTCTCTATTTTCTCCCTCAACTTAGCCATC

ACCACATTAGCCTCAGACCCTGATCCAGTTCAAGACTTCTGCATACCAAACCCAATATTA

GCTTCCATTAAAACACATCACACCTTTCACACCATTCTCCCATGCAAAAACTCCTCACAA

GTCATCACCAATGACTTTGTCTTCTCCAACATGAAAACATCAGGAAACTTCTCAGAAACA

GGTTTAGCAGTAATACCTGCAAACCCTACAAATTTTCCAGGTCTCAACACACTAGGGATG

TCATTTGCAAGAACAGATATTGAAATTGGAGGAATCAATCCACCACATTTTCATCCAAGA

GCCACTGAACTAATACATGTGATTCAAGGAAAAGTGTATTCGGGTTTTGTTGATTCTAAC

AATAAGGTTTTTGCTAGAATACTTGAACAAGGTGAGGTTATGGTGTTTCCTAGAGGACTT

GTGCATTTTATGATGAATGTTGGTGATGAAGTTGTTACTTTGTTTGGTAGTTTTAATAGC

CAGAATCCTGGAGTGCAGAAGATACCTTCTGCTGTGTTTGGTTCAGGGATTGATGAGGAG

CTTTTACAAAAAGCTTTTGGATTGAGTTCTAAACAGATTGGAACCATGAAAAGAAAATTA

GATCCTAAACAAGAAAGGTAG

>MS.gene81751.t1

ATGTCAGGCAAAAAGAGAAGAAAATTGAAATTGTTAAGTGATACAATTGCTAAGAGCAGA

AGAGAAGCAACAGAGGTGGAAAATGATAATTTGGAGCTGCAAACATGGGCTGATCTCCCT

GCTGAACTATTAGAAATGATCATATCTCGTTTAGCCTTAGAGGACAATGTCCGTGCTTCT

GCGGTTTGCAAGAGTTGGAATTTTGTTGCCAATGCTGTACGCATGGTGAACCAATCACCA

TGGTTGATGTATTTTCCAAAATTTGGTCAGTGGTATGAATTCTATGACCCTGTGCAGCGG

AGGACCTATTCCATTGAGTTTCCTGAGTTGAATGGATCTAGAGTTTGTTACACAAAAGAT

GGTTGGTTACTGCTATACCGGCCAAGAACTGATCGAGTGTTTTTCTTTAATCCTTTTACT

CGGGAGACTATCAAAATGCCAAGATTTGAGATGACATACCAGATAGTTGCGTTCTCTTGT

GCTCCAACATCACCTGACTGTGTATTGTTCACTGTTAAGCATGTTAGTCCTACTATTGTG

GCCATCAGCACATGTCACCCTGGGGCAACTGAGTGGGTCACTGTTAATTACCAAAACCGT

CTGCCTTTTGTTAGTAGCATATGGAATAAGCTTGTGTTCTGTAATGGACTCTTTTATTGC

CTGAGTCTAACGGGTTGGCTAGGGGTATTTGATCCATCTGAACGCACTTGGAGCGTTTTA

TCGGTACCTCCACCCAAATGCCCAGAGAATTTTTTCGCAAAAAATTGGTGGAAGGGGAAA

TTTATGACCGAGCAAGAGGGAGATGTTATAGTAATGTACACATGTTCTAGTGAAAACCCT

ATTATTTTCAAGTTAGACCAGGCCTCAATGGAATGGGAAGAGCTAAAAACACTAGATGGA

GCAACTCTATTTGCTAGTTTCTTGTCTTCTCATTCAAGGACTGACCTCCTTGGAAATATG

AGGAACAGTATCTACTTCTCTAAAGTTCGTTTTTATGGAAAGCGTTGCATATCATTCTCT

CTTGATGACTATCGCTACTATCCTCGTAAGCAGTGGCATGACTGGGGTGAACAAGACCCT

TTTGAAAGCATATGGATTGAACCACCAAAGGATTTTTCTGGATTCTCATGA

>MS.gene81764.t1

ATGTACTACAAAGGAGTCTACCATCTATTTTACCAGTACAATCCCAAAGGAGCTGTATGG

GGTAACATTGTGTGGGGGCACTCAGTATCAAAAGATCTCATAAATTGGAAAGAACTTCAA

CCTGCACTATACCCATCTAAACCGTTTGACAAATACGGTTGTTGGTCCGGGTCGGCCACA

ATCATCCCAGGTCAAGGACCTGTCATCCTCTACACTGGAGTCATTGACAAGCGAAGCAAT

GAGGTCCAATGCATTGCTATACCTGCGAATGCATCTGATCCGTTACTCACAGAATGGGTT

AAGCCTGACCGATTAAACCCAATTGTGACTGCGGATCACCACATGAACGGATCAGTGTTT

CGAGACCCTACAACTGCTTGGTTGGGCAAGGATGGGCATTGGAGGATACTTGTTGGTAGC

AAAAGGGAAGATATGGGACTAGCCTATTTATATAGAAGTAGGGATTTTGTCAAATGGACC

CGAGCCAAACACCCGATCCACTCGGCTAAAACCACAGGGATGTGGGAGTGTCCGGATTTT

TACCCGGTTTCTTTGGAAGGAAAAAACGGGTTAGACGCATCGACAATAGGTAATAGTGTT

AAGCATGTGCTAAAGAATAGCCTTGACATGACGAGGTACGAATACTATACAATTGGAACG

TATATCCAAAATAAGGATAAGTATATACCGGATAAAACTTTCGAGGACGGTTGGGGTGGC

CTTAGATATGATTATGGCAACTTCTATGCTTCCAAATCATTCTTTGATCCAAGCAAGAGT

CGAAGGATCATATGGGGATGGGCAAATGAATCAGATACCAAAGAAGATGATGTTAAGAAA

GGATGGGCAGGAATTCAGGCAATTCCAAGAACTGTGTGGCTTGATCCTGGTGGAAGACAG

TTAAGACAATGGCCAGTTGAAGAATTAAATAGGTTAAGAGAGAAAGAAGTTGGGATAAAC

AACAAAAAGGTAAAGAAAGGTGGTTATGTTGAAGTGAAAGGGATTACTGCTGCTCAGGCT

GATGTGGAAGTTACTTTCACATTCTCAAGCTTGGACAAGGCAGAGGCAATTGATCCTAAG

TGGGTGAATGCAGAGGATATCTGTGCACAAAAGGGTTCAAAGGTTCAAGGTGGGATTGGA

CCATTTGGACTTCTGACATTGGCTTCCAAAAAGCTTGAAGAGTATACTCCTGTGTTCTTT

AGAATTTTCAAAGCTTCAAATAAACACGTGATTCTCATGTGCTCTGATGCAAAAAGTTCC

TCTTTGAATAGAGAATTATACAAGCCATCATTTGCAGGCTTTGTAGACGTGGATTTGGCA

AATAATAGGAAACTTTCTCTTAGGAGTTTGATTGATCACTCTGTGGTGGAGAGTTTTGGA

GCTGGAGGGAAAACAAATATCTTGTCTAGGGTTTATCCAACGCTAGCAGTGAATGAAAAA

GCTCATTTGTTTGTGTTCAATAACGGCACTGAGCAGATAACTGTAGAGAAACTAAAGGCA

TGGAGCATGAAATCTGCTAGTCGAAATTAA

>MS.gene81762.t1

ATGGCAACCGAGTCTTGCATCCGTCTTCTATACACATGCAGAAACATTTTCCATATCAGA

CAAGTTCACGCCAACGCATTAATCAACGGAACATTCAACAATCTCATTGTTGCAAACAAG

CTCCTTAACTTCTATATCCAACACAAAGCAATCAACGATGCACATTACCTGTTTGATGAA

ATGCCAACAAGAGACCCAACAACTTGGAGTATCATGGTTGGTGGATTTGCCAAGCTTGGT

GACTATACAAACTGCTATGCAACCTTTAGGGAGATTCTTCGATGTAATGTTACGCCAGAT

AACTACACTTTGCCGTTTGTTATTAGAGCTTGTAGGGATAGAAAGGATATTCAAATGGGT

CGAATGATTCATGATGTGGTTTTGAAACATGGGTTGGTATTGGATCATTTTGTTTGTGCT

ACGCTTGTTGATATGTATGCGAAGTGTGCGGTGATTGAGGATGCTCGGAAGTTGTTTGAC

GTGATGGTTAGTAAGGATCTTGTGACGTGGACGGTTATGATTGGTTGTTATGCGGATTGT

GATGCGTATGAGTCGTTGGTTTTGTTTGATCGAATGAGGGAAGAAGGGTTTGTTCCTGAT

AAGGTTGCTATGGTGACCGTTGTTAATGCTTGTGCGAAGTTGGGAGCTATGCATAGGGCT

AGGTTTGTTAATGAGTTTATTTGTAGGAATGGTTTGTCTTTGGATGTTATATTAGGGACT

GCGATGATGGATATGTATGCCAAGTGTGGATGTGTTGACTCTGCAAGAGAGGTTTTCGAT

AGGATGAAAGAGAAAAACGTTATTTCGTGGAGTGCCATGATTGCTGCTTATGGATATCAT

GGGAAGGGGAAAGAAGCTCTGGACTTGTTTCATATGATGTTGAGCTGTGGAATTTCGCCG

AATAGGATAACCTTTGTCTCCCTCTTATATGCTTTTAGTCATTCAGGATTGACCAACGAG

GGTCTTCACTTCTTCGATTCAATGTGGAGAGATTATGGTGTTAGACCTGATGTCAAACAT

TATACTTGTGTGGTTGATCTTCTTGGACGTGCCGGGAGGCTAGACGAGGCATTGAAATTG

GTTGAGACCATGAATGTTGAAAAAGACGAGAGGCTGTGGAGTGCTTTGCTTGGAGCATGT

AGAGTTCATGGGAACATGGAATTGGCAGAAAAGGTAGCCGAGTCTCTACTTGAACTACAG

CCAAAAAATCCAGGGGTTTATGTATTACTATCTAATATTTATGCAAAAGCCGGTAAGTGG

GAAAAAGTGGCAGAATTTAGGGATTTGATGACACAGAGGAAGCTGAAAAAAGTTCCTGGA

TGGACATGGATCGAAGTAGATAACAAAACATATCAGTTTAGTGTTGGAGATAGATCTCAT

CCTCAATCGAAGGAGATCTATGAAATGTTGACTAGCGTAATTAAGAAGTTGGAGATGGTT

GGTTATGTACCTGATACAGAATTTGTGTTGCAAGATGTTGAGGAGGAAGTTAAGAAAGAA

ATGTTGTATACACATAGTGAAAAGCTTGCTATTGCATTTGGCTTAATTGCCATCCCAAAC

AAGGGTGATCCTATTAGGATCTCTAAAAATCTGAGAGTCTGTGGTGATTGTCACACGTTT

TGTAAGATGGTATCAGAAGTTATGAAAAGGTCCATAATTGTTCGGGACGCAAACCGCTTT

CACCATTTTAATGAAGGGGTTTGCTCATGTGGGGACTATTGGTAG

>MS.gene81733.t1

ATGGACCTCCAAAACGACGCCGTTTCCAAACCAATTTCAGTCCTCTCTTGCATTGCGATG

GCGTTGCTCTACGTCGTTACCTTATACGCTCCCACCTTCCTCCTCCGTCTCCCACCTCCT

TCTTCTTTCACCAATTTCATGATCCGACGCTTCCTTTGCGCCGTCGTTTCAACCACTCTC

TCCCTCTTTCTCACTCCTTTCATCCTTCCCGTGCAAACTAGGGACTTGCCGTACATTTTG

GGCGTTTATGGCATCCGAGTGGATCACATGTGGCAGGCTGTGTTCATTCCTCTTGCTTTG

ACCTCTCTAATGTATGCTGGCTCTTTGTTCCTCAAGTCTCTTCTGTTGTTTGATTTTTGG

AGGCAACATTCATTTTTTGGTGGCGAGATTTCCTTTGATTCCCTCAAATGTGCCGTGACG

AGATTTATTGACTGGTTGTCTGAAATTTCATCAAATGTTTTGACTTGGAGAAACTATGTT

GTGGCACCCCTTACTGAGGAGTTGGTGTTTAGGGCGTGCATGCTTCCTATACTTCTCTGT

GGAGGGTTTAAACCATACAGTGCTATGATTCTTTGCCCCATTTTCTTCAGCTTATCTCAT

TTAAATCATTTCATGGAGATATACACCAAACAAAACTACAGAATAATGAAGGCTGCTATG

GTTATAGGACTCCAGCTTGGCTACACTGTTGTCTTTGGGTCATATGCTTCTTTTCTTTTC

ATTCGAACTGGTTATGGCTATGATATATGTCTGGTCTGA

>MS.gene81731.t1

ATGTTGATTCATCTCTTCCTTTACATCCTATGCTTCTTGTTGTTCCCTCAATCCCTACAA

TCACAAGCAAATGCTTTTTCTCGTCCACCTTCTGGAAAATTCCTCTTCACTCATCATGAA

CGTTCAGATTCTGACCCTCAACAGGTGCACATTTCGTTGGTAGGGAAAGATCATATGAGA

GTGTCGTGGGTAACGGAAGAAAAAGACTCAGAATCTTTGGTAGAGTATGGAATAAAAGGA

GGTGAATATAGCAAAAAAGCAATTGGGGAACACACCTCCTACCGTTATTTCTTGTATAAA

TCAGGCAAGATCCATCATGTGGTAATTGGGCCTCTAAATCCAAGCACTACTTACTTCTAT

AGATGTGGAGGATCAGGTCCTGAGTTTTCTTTCAAGACACCCCCACTCAAGTTGCCTATT

GAATTTGTGGTTGTGGGGGACTTGGGACAAACGGAATGGACGAAATCAACCTTAAAACAC

ATTGATAGCAAAGACTACGACGTGTTTTTGTTGCCCGGAGATCTATCCTATGCCGATACC

CATCAACCACTTTGGGACTCATTCGGTCGGTTGGTGGAACCCTACGCAAGCCAGAGGCCG

TGGATGGTTACCGAAGGCAACCATGAGATTGAGAGTATCCCTATTATCCAACCACATGCC

TTCAGATCCTACAATGCTCGTTGGCTCATGCCCTACAATGAGAGTGGTTCAACCTCTAAC

CTCTACTACTCATTTGAAGTTGCAAGCACTCATATCATCATGTTGGGTTCTTACACTGAT

TTTGATGCACACTCCGAACAATATAAGTGGCTTCAATCTGACCTAGCTAAGATTGATAGG

AAGAGAACACCTTGGGTGATTACCTTGTTGCATGCACCTTGGTATAATACTAATGAGGCA

CATGAAGGTGAAGGGGAAGATATGAGACAGGCCATGGAAGATTTGCTTTATGAGGCTCGT

GTTGACTTGGTTTTTTCGGGGCATGTCCATGCATACGAACGCTTTACAAGAATCTATGAC

AATAAGGCTAATCCATGTGGTCCGTTGTACGTGACAATTGGTGATGGAGGAAACCGTGAA

GGACTTGCATTAAAGTTTAAGAAGCCTCCAAGTCCACTCTCATTGTATAGAGAGGCAAGC

TTTGGTCACGGAAGGTTGAGAATAGTCAACGAAACACATGCAAATTGGTCATGGCATCGT

AACAATGATAGTGAAGCATTTGTAGCTGACGACATATGGATGAAGAGTTTGAGCAACACA

AAAGAATGTTGGGAGAGCTTAGGGCAAAAAAATTCTCATGAAGAGCTCTAA

>MS.gene81759.t1

ATGGTAGAGCGGGAAGGCTTCGCGTTTCCTATTGCTATAGAATATGAGGATCTGCCTGAA

TTTTGCACGCACTGTAAGAGTATCAGACACAATGTCACTTCATGCCGTTGGTTACATCCA

CGAAGGGAGAACAATGTTGAGCAGCCAATCGACAAGGATAAGAAACCAGTTCATTCTCAA

AGACCAAAACAATGGTGGAAGCCTAAAGACAACCCAGAGGGTGTTGGTTCATCCAAGGCC

TTTGCTAAGGCGGCACCGATTCAGCAAGATGATATTAGTAATGATAATGTCGCCTTTGGG

ATAGAAGCTACGGTGCAGCAAATTGATGGAGGTGATGACACCTTACGGTTGGACGCATCT

TAG

>MS.gene81741.t1

ATGGATAATCTTCCTGTTGAAGTGATTGGTAATATACTATCCCTTCTGGGGTCGGCCCGC

GATGTTGTGATTGCATCCTTAACTTGCAAGAAATGGAGACAAGCATGGCGTTATCATCTC

CATACTCTTGAATTCGTCACTTTTGATTGGCCTGTTTATCGCGAATTATCATCTAGTACG

TTGGAAATAATTATTACTCAAACAATTTTTCAAACCAAAGCATTGCGGTGTTTAACGATT

AAAATGGATGATGTTCATGAATTCTCGGCTGCATCGGTGATTGCTTGGTTTATGTATACG

AGGGAGGATTTGCGTCGGTTGCATTTTTATGTGAACACACCGCCGATGTTTAATATCATT

GAGAAATGTGCTAGGCAGAAGTTGGAAGTGTTGGTGTTGGGTCAGAATTTTATCACGCGT

GTGGAGCCGAGTTATCTGAAGTTTCCTTGTTTGAAGTCACTTTCGTTGAGTTTTGTCAGT

ATCTCGGCGTTGGATTTGAGTCTTTTGCTTAGTGTGTGTCCGAGGCTTGAAACATTGGCT

TTGGTTAGTCCGGAAATCGCTATGTCGGATTCACAGGCTTCCATGGAGCTTAGTAGCGGT

TCTTTGAAGGAATTCTCTGTTGAGTCGTTTGGTTTGGATAAATTTGTATTGGAGGCAGAT

TTACTTGAGTGCTTGCATCTTAAAGATTGTACCTTTGAGGTTTTTGAATTTATTGGGAAG

GAAGGTTTGAAAGTGTTGAAGGTTGATGATGTGAGTGTCATTCATCTTGATATTGGTGAG

AATGCTGATAACCTTGAGTATGTAGACATAAGCAACTTCACAATTATGTGGCCAAAGTTT

TATCATATGATTTCAAAAGCATCTAAGTTACGTAGACTTCGGCTGTGGGAGGTTGTTTTT

GAAGATGACGACGAAGTTGTAGACTTGGAAACGATTTCTGTTTGTTTTCCTCAATTGATG

CACTTGTGCTTAAGCTATGATTTGAGAGATGGTGTACTTAATTACGGCTTGCAAGGTTTT

TCTCTGATGAGGAATGTGGTGGTGCTAGAACTTGGATGGACTTCAATCAATGATCTTTTT

GTGGAATGGGTAGATGGACTTTTGGAAAGATGTCCTAACCTGAAAAAATTGGTCATTCAT

GGTTTTGTTTCAGAGCCCTATATCGCCATTGTTGTCGTCCTCCGGCGGCGGGATGGTAAC

CGTTTCGGCTCCCTCCTCCCTCAATTTTTGATTCGCACCAGATTCCGAACTGAAAAATCT

AGATCTGAAATCTTCTCTTCCTGTTACCGTGTTGCTGTTGTGGTTCGCGGAGGTTCACGG

CAGCAGTACTGTGCTCCGCTCCGTCGTGATGATGGATTTAAAAGCTTTCATTGGAGAACG

GTCGCCGAGCCAGAATCTCATAAAAGAATACATCTCTCAAAAGCAAAGTCCAGCTACAAC

AACTATAACAAGAAAAGGCAAAAAAAGAAAGAAGAAGAAACAGTGAAGATGGGTTCTCAA

TGCTCAAAACAAGTTGAAAGAAGAAAGACAATCCATACCGAAAAGAAAACTCTAACCGAA

CTTAAAACCTCAGGAGAAGACTACCCTGGTTCTGAATATCATCCATCAGATAGAAAAAAC

TGGATGAATGGTCTCAACCCAGAGAAAGTTCATATCAATCAAATTGTATGGCCAGGAACA

CATGACTCTGCTACCAACAAGATTGGTTTCCCACTCATCACTCGTCCTTTTGCTCAATGC

CAGTCTTTGTCCATCTATCGACAACTCGCTTTAGGCACACGTGTTATTGACATTCGTGTT

CAAGAAGACAGACGTGCGTGTCACGGAATTCTTGTTACTTACAGCATCGATGTTGTTATT

AAAGATATCAAAAAGTTCTTGTCGGAAACACAATCTGAGATCATAATTCTTGAGGTTAGA

ACAGAATTTGGTCATGATGATCCACCTGAATTTGATAAGTATCTTGAAGAACAATTAGGT

GAGTTTTTGATTCATCAAGATGATCATGTTTTTGGAAAAACCATTTCGGAGTTGTTACCT

AAGAGGATTATATGCGTTTGGAAGCCGAGAAAATCGCCCCAGCCTAAAGCAGGGAGTTCT

CTGTGGAGTGCAGGGTTTTTGAAGGATAATTGGATTAATACAGATCTTCCATCAACCAAG

TTTGATGGGAATTTGAAGCATTTGAGTGAGCAACCACCGGTTACGTCGAGGAAATATTTT

TATAGGGTGGAGAATACGGTTACTCCGGTGGCGGATAACCCTGTTTTGTGTGTGAAACCT

GTAACGAGACGCATTCATGGATATGCTAGGCTCTTTATTGCTCAGTGCTTTGCTAAGGGA

TATGTGGATAGGATTCAGGTCTTTTCCACGGATTTTATTGATGAGGATTTTGTTGATGCA

TGTGTTGGACTCACTCATGCAAGGGTTGAAGGAAAAGCCTGA

>MS.gene81742.t1

ATGAACTGGATTCAAAACTTGAAAATTGAGTTACCATGTAATGAAACATCTCCTAAAATT

ATAGAAGAAGATTGTTCAACTCAAAACCAAGCTACCATTCTTGATCCTAAAGTAGCTCGA

AGTAAAGGGTGCCCTCCTTCGAAAAGGAAGACTTCTACAGTTGATCAGATCGTAAAGAAG

AAGCTTGCACAAAAGAAAACTAAAAAAAGCAACCAAAATAGTAAAAAAAATAAAGGTCAA

GAAATGGGCCCATGCACATCTAGAGGTCAAGAAATTGAACATGGAGTATTTTACGGATCT

CAACTTGGTGATGGAATTGGCACACAAGAAAGCATTCAAGCAAACAAAGGATATACTAGT

CAAGTAAATCAGCATAAGCAGAACTATGTCTCGAGCAGTGAAAATGGTAGTGTGAATACT

GTAGCTCCCTCCAATCCAAATCAAGAACATCTTGCTGAAGCTCCTTATTATTCTCAGAAA

TTTGTATGTTTTACGAATGGAAGTGAAATCCGAAGGCTTGCCTGCTTCGGTAACGGGGAG

CTGCTGTTTGTGGGAGGTGGTGCGCGCAGTGGGATTGTGTTTGGGAGTTTTTTGATACTG

CTGTTGTTGCTTTCAGCTGATGTAATGTGGCTGTTGTGTGCTGGTATCTGTAGCAGGTTT

TTGGAGGTCTTGGGCTGA

>MS.gene81760.t1

ATGCCACTAATCGATCCAAATCACATTTTTAATGCGTGGAGTGCTGTTTTTGGAGTGCAG

CGGTGGGGTTTGGGCTTCCTGTTGTGTGTGAATGTGGCCTGCTGGTGTCTTGATGCTGAA

GGGACTGGTTTTTTTGGGTCCTGTCATGGCTGCAGTTTTGCTGTTGTGGTGCTGGTGGTG

GCCTGTCCTGTGATGCGGTTTGTGGCCGCAGCAGTGGCAGTTAGCAGGTGCGGTTCTGTG

TTAAGTGTTATAGCTGATGTTGTTATTCAGCTGGCTTGA

>MS.gene81756.t1

ATGCTAGGGAAAAGAATGGTATCATTGAAGAAACTGGCCAAGAAAGTGAAAGCTAGTGGT

GGAGCTGGAGCTGACTCAAACACTGATCCTCCCTACAATGAATGTTTGTTGAAGGGTTAT

GAAGAAGAATTCTCCACAAGCACAACTCCAACTGGTTTTTTTGCACTTTATGTGGGGGAA

GAACACCAGAGACATGTGGTTCCAACAAGCTATCTTTCTCACCCTTTATTCAAGATGCTG

TTGGAGAAGTCATACAATGAGTTTGGATTTGAGCAGAGAAATGGTTTGGTAGTTCCATGT

AGTGTTTCAACATTTCAAGAGGTTGTAAATGCTATTGAATGTAACAATGGCAAGTTTCAC

TTGGGCAAGATTTTTCAAGATTTTGTTTAA

>MS.gene81775.t1

ATGGTGTATTGGTCGTACCCGCCGACGGGGAGGGAAATTGCGGTAACCGCCGCCGTGTTT

ACGATCGGTGCTTCGTTGTTCGGTGTCGGAGCTTATCTTTCGTTTGTTAACGTTGCTCCA

CAGCAGGCTCGTGCGAAAGAACGCAGTGAAGCAATGAGGAATTATCTCAGCAAGCGCTTC

G

>MS.gene81735.t1

ATGGGAAGTATCACTTCAGATCCACTAATTCTTGGGAGAGTGATAGGAGATGTTATTGAC

TATTTCACCCCAACCACAAAAATGACTGTAATTTATAACAACAAAGAAATCTTCAATGGA

TATGAACCCTTTCCTTCTTCAGTTACCACCAAGCCAAGGATTGAGATTGGAGGAGAGGAC

ATGAGGTCCCTCTTTACACTGGTCATGATAGACCCGGATGTTCCTGGCCCAAGTGATCCT

TATATGAAAGAACATTTGCACTGCCACATAAAGAAAATAAACTTGATTGTCATGAAAGCA

CTACTCACAGACCAGAATTTACTGCATAGCTTTCAATCTCGGCGTTCTATAAGATCGAAC

GGTTAA

>MS.gene81761.t1

ATGTCTTCTGCTCGTCACGAGAAGGAGAAAGGCGTCAACGTTCAAGTCCTCCTTCGTTGC

AGGCCTTTCAGTGAAGACGAATTGCGGAGCAATGCTCCGAAAGTTGTTACTTGCAATGAT

TACAATAGAGAAGTATCAGTTTCTCAGAATGTTGGCGGCAAGCATTTTGACAGAGTTTTC

ACTTTTGATAAGGTCTTTGGTCCTTCTGCGAAGCAAAAAGATCTATATGATCAAGCTGTA

GTTCCAATAGTAAACGAAGTTTTAGAGGGTTTTAATTGTACTATATTTGCATATGGTCAA

ACTGGTACAGGGAAGACATACACAATGGAAGGTGAATCCAAAAAAACCAAGTGTGGGCCG

AATGGAGAGTTGCCTTCAGAAGCAGGAGTCATACCTAGGGCTGTTAAGCAGATTTTTGAT

ACTCTTGAAGGCCAGAATGCTGAATATAGCGTGAAAGTCACTTTTTTAGAATTGTATAAT

GAGGAAATTACTGATTTGCTTGCGCCAGAGGAACTTTCAAAAGCTTCTTTGGAGGAGAAG

CAAAAAAAGCAGCTTCCTCTTATGGAGGATGGTAAAGGTGGCGTTCTTGTAAGAGGTTTG

GAAGAAGAAATTGTAACAAGTGCAAGTGAAATTTTTACTCTGCTGGAAAGAGGGTCTGCC

AAACGGCGTACTGCAGAAACTTTATTGAACAAACAGTCAAGTCGGTCACATTCTTTGTTT

TCCATTACAATCCACATCAAAGAAGCAACCCCAGAAGGTGAAGAACTTATAAAATGCGGC

AAACTAAATTTAGTGGATCTAGCAGGTTCAGAAAATATTTCTCGTTCTGGTGCTAGAGAG

GGTCGTGCAAGAGAAGCGGGAGAAATCAATAAAAGTTTACTTACTTTAGGGCGGGTTATC

TCTGCACTTGTGGAGCACCTTGGTCATATCCCTTACAGGGATAGCAAGTTGACGCGTTTA

TTGCGTGATTCACTTGGCGGCAGAACTAAAACATGTATCATAGCAACAGTCTCTCCTGCA

GTTCATTGCTTAGAAGAGACCTTGAGTACATTGGATTATGCACATAGGGCGAAGAACATA

AGAAATAAGCCTGAGGTTAACCAAAAACTGATGAAAACAACTCTTATCAAGGATCTCTAT

GGTGAAATTGAGCGTCTTAAAGGAGAGGTTTACGCTGCACGTGAGAAAAATGGCATCTAC

ATACCAAAGGACAGATACATTCAGGAGGAGAATGAAAAGAAGGCCATGAGAGATCAGATT

GAGCAGATGAATATTGCATTAGATAGTCATCAGAAGAAACTTGAGGAATTGCAAAACAAA

TTCAATGACCAATTGTTGCAGAGCTCACATTTAAGCACAAAACTCGATGCCACCGAGAAA

AACTTGAAGCAGACCAGCATATTGCTTGCCAACACAGAGGAAGAATTAAAGAAATGTCGG

TATACTCTGAAAGAGAAAGATTTTATCATTTCTGAGCAGAGGAAAGCAGAAAATGCTCTT

ACTCATCAAGCATGCATTTTACGAGCTGATTTGGAGAAAGCTGTTCAAGATAATGCTTCA

TTGGTCTTAAAAATTGGTAGAGAAGATAAATTGAACTTAGATAACAGAGCTGTGGTGAAT

AATTTCCAAGTGGAGCTAACCCAACAAGTTGGTTCTCTTCGTAACACTGTGGCAACATCA

TTGTCTCGACAGAATGAACACCTTGAATGTGTGGAGAACCTTTGCCATTCATTTATGGGT

ATTCATGATAAGGAAGTTGCCGATTTGAAACAGAAAGTTACAACTCTGAGGGCTTTGTAT

ATATCTCATGTTGAAGCGATGCAAAATGTTGTGCGTTTGCATAAGTCGGGTTCTGATGCT

AGCTTTCAGGAACTATCATCTCTTATATCCTCTAATAGTCACTTCATTGAAGAATTTCTT

GTATCCGAGGCCACCAAAGCAGGTTCTATACTTGATGATCTTCAGACTTCTCTTTCGACT

CAGCAGGGTGAATTGACACTTTTTGCAAGTGAGCTACACCATAGATTAAGTGCCAATGCT

GAGCAAATAAAGGATATATCTGAGTGTACTCATGAGTTCGAGGATACAATTTTGAAAGAA

GCAAAAAGTCTTGAAAACTTTGCGTCTGCAGCTGATGAAATGCAAATGAAGAGTATCGCT

GAGTTTAAAAAAGCTTATGAGGAACAATCAAGATCTGATGTAGAGAAACTTATAGCTGAT

GTGACTAGTTTATTATCAAATCACGCTTGTCGTCAAATTGATTTGGTAGATACAAAGCTT

GCAGATCTTACAAAAAATGGCATTGCCAGCAAGTCATTCGTGGATGAACATGTATCATCA

ATGAAAGATATTGTCTCGTGTTCAAAGAGAAAGTGGCACGGCATATGCACGCAGGCAGAA

AAAGATGCCAGAGACACTGCCGAGTTTTCTGCTGCTAAGCATTCTTGTATGGAGGAGCTC

CTGCAGCAGAGTATCAATACTGCTCAATCAGCTTTCCAAAATACAAAGAAGACACATGAG

GTTGTAAATGAGACTGGAGCTAAACATGTTTCAGCAGCAGTGTCACTTATCAGGGATGCT

ACCGATAGCAATATGCAACATGACATTGAAATTAGTTCTGCTCGGGTTACAGCTGAGGAA

GATGTGACGAAGAACAGTGACGATGTCCTTCAACAGTTTGATGACATGTCTGTACAAGAA

AGAGATTCTGTATCTGGTATGTTGAATGTTGTCAAAACTCATGTGAATACACTTGAGACC

TTCAGAGAGGATCACTCTACCATAGCAACTTCAATTGAAGAAAAAGCAAGTGAAATTTGT

CAGGAACAATATAGGGACTATGAGCCAAGTGGAACTACCCCAATAAGATGTGAACTTGAA

GTTCCAAGTAGAGCAATAATAGAGTCTCTTCGATCCTTGCCAATGGAAACTCTTATTGAA

GAATTCCGGGAAAACAATTTGTATGAATCATTTGAATCATTCGATATAAAGGAGTTGAAA

CCATCACTAATACCTCGTTCTCCTCTCAGCCAAGTAAATGCAACTAAGGGAAGAACAAGG

TATTAG

>MS.gene81748.t1

ATGGCTTTGCAGATTTACTTCTCAATTCTTCTTATTGCAACTTTGGCTCTATGCACTTCA

GCAAATTATAATTCGGTTAATTTTAGTTATATTGGACCAAATGGTCCTGAGAAGTGGGGA

ACCCTGAGTCCATCGTTTGCAGCATGCTCAAATGGGAAAGCACAAAGTCCAGTGGAACTG

GTAATGAATGATATTGTCATGAACCAAGAATTGAAACCCTTAGACAGAAACTACCATCCT

ACAAATGCTACACTTGTTAACAACCAATTCAACATTGGGGTACATTTTGAAGGTAAAGTG

GGAGATATTAATATAAATGGAATGAATTATTCATTGAAACAACTTCATTGGCATGCTCCT

GCAGAGCACCGGGCTCATGGTCGTTTACATGAAGCGGAGCTCCACCTAGTTCACCTCACA

GAAGATAACAATAACATAGCAGTTATTGCAGCCCTCTACAATTTGGGTGATCCTGATCCT

CTAATCTCCAAGATTGAAGACAAGTTCTATGAGCTAGCCAATGAGAATCGTGTGGGCAAT

AAAGATGCCAAAATTGCCCTTGGCACGTTTGATGTAGAGGAAATAAATAAAAGGATCCAT

AGATATTATAGATATGTTGGCTCTCTCACTACCCCTCCATGCAAAGAAGGTGTCATTTGG

AACATCATTGGCAAGACTGATTTCAATCTGTCTTCACACCTCGATCATGCATCAGCCACA

TTTGTGAGTATTCAAAATCTCTCTACCTTGCCAACTCCAGTTGCTACGTTTTCAATAGGC

CATATGAGGATCGACACCATACAAACTTGTCAGCGTTGA

>MS.gene81740.t1

ATGGAAATTGTGACAACCACACTGCAAAGTATTGCTTCTCAGATGGAGCAACTCGCTCAA

CAGATGCTTCAACAGAGTGTGATTATGACAGAATTGAGCAAAAAGCTTGGGTCCAAGGGG

GTGACTCTAGAAGGCAAAACCTCTAGTGGTGATTCAGTTCAGAGCGAAATTGAAGAAACA

GAGAAGATGAAATTGGACTTCGATTTGAACTCATCGACAAAGGAGAAGGAAGAAGTTTCA

TTGTTGACTGGGACAATGTTTTCACGATTGATGAATTTTCAATCCTCACCGGAAAACATT

CACACAGAATCGAGTATTGAAAGAAAAGGGAAGCAAGAGGCTACAATCACTCAAAAACCG

CCGCTGAATCTGTCAAATCCGCCTTCGACGGAACCACCGCCGGACTCCGGTCAACCCGTA

ACAACAGTTCCACGGCGAGCACCGCCGCCGAAACCACCAGATCTACATGATTTTGTTGAT

GGGGAAGTCTACGCGAAATCGAGCGTCAAGAAGGGAGGGAAGGTACAGATGAGTCTTGGT

GGACCGTCGTTAAAGTCGTCGGAACCACCGTACTTCGGTTGCAATTCCGTTCACGTGATT

GTAATTGTTGCTGAAGCTAATGTGACTGATTCTTCTCAAGCAAAATATTCTTGGGGTTAT

GGCATAGAGAGGAGTGGCAAGCTTATACAGTGGGTTAAAGGTCTACTTGATGGACCAAAT

AAGTTTTTATGTCCTTCCTTGGTAATAAGATTAGTTATGCAGATGCTTACTTTAATTCTT

GTCATGAAATACAAAGGGGAAGATGGGAACCTCTTGATGATTCCCCCTAGTTTTTTATCA

CTATGTGATGATTCATTGGTGCTGCCAATTGGGGAAAATATGCATGTGTACATGGTGGGA

TTTTATGATGATAAAAGTGTTTTTTGGAAAACTGCATTGATTGAGAAAGATAGCAAATAT

GAGCATGTTTCTAGGACATACCGAGCTTGTATTCAGATTGGAGCAAAATTACATAACCTT

GTTGTGTTGAATGCAATGATGTGTGGGTATGGAAGTAAAGTACACTTTGTCCCTTCTTTC

AACATGATATCCTGGTGTTCATCCTTTTTTATGAACCAACAAGACTCAAGGGAATTCGAT

TTTCCTATGAGGCAAGTTTGGTGCAAAGGAAAGATGTTGCAGCAAGGGATGCATATTTCA

GCCTTGAAGCTTTTAAACATTTCAGTGATCACATGCTCTTCCAATCCACATTATTTCAGT

GATTATTTGCTCTCCATGCATGGACTAGTTCTCATGGACTCCATTGAGCTCAACAGGCTG

ATTGAACAAGCTTATGATAATCCACACGAGGCATTATCGCAAATCAAGCGGCACCTCCTC

ACCGAACGTGCCTTCAAAGAGGTTGGAATAGAGTTTATGGATTTATATAGCTACTTGATT

CTGGTTTATGAGAATGAGCCTCTTGAGAAAATTACAGATGCATATCTTGATCAATATCTG

TGGTATGAAGGCGACAAACGTTATCTCTTTCCCAACTGGATCAAGCGCAGATTCAGAGCC

ACCGCCTCTTTTGGTTTACAAATGGTGTCAAGGCATCAACAATTTGCAAGGTGTCTGGGA

CACCAGCGATGGCTTCTTCGTTTGGTTCTAGACCACAACATTGCTGATTATGTCACTGCA

AAAAACAATGTTGTGTTGTCATATAAGGACATGAGCCACACAAATTCATATGGTCTTATG

CGTGGACTGCAGTTTGCATCTTTTGTTGTTCAATATTACAGCCTAGTATGGGATCTTTTA

CTTCTTGAATTGACTCGAGCAAGTGAGATTGCTGGTCCACCGCAGATGCCTAACGAGTTG

ATTACTTATTGGGACACGAAAGGGGAGACCAGACATCCGATTAGGCTGTATCCTCGGTAC

ATAGACCGAGTTCACATATTATTTCGCTTCACACATGTGGAGGCGCAGGATCTCATTCAG

CGATATCTCACTGAGCATCCTGATCCTAACAATGAGAACATGGTGGGCTGGATTTCCACG

TATTCTTCTAGGGAATGTGGATTATCCACCCTTACGGTTCCTAGAAGAAGACGTGGGAAT

CCAGCTGTGGAAAATGTTAACCTACAGGGTTTACAACATGTTGTGCAACAACTTGTTAGA

ATTGTTGCTGGGCAGCAACAAGGAAACCAAACACATGAAAATTTTGAAGGTCAACAAGAA

ATTAGGCAGGTTGATCAGGTGGCAGCAGCCCCAACAAGGGGGATTGAGAAGTTGGAAGTG

TTGGTGTTGGGTCAGAATTTTATCACGCGTGTGAAGCCTAGTTATCAGAAGTTTCCTTGC

TTGAAATCACTTTCATTGAGTTTTGTTAGTATCTCAGTGTTGGATTTGAGTCTTTTGCTT

AGTGTGTGTCCGAGGCTTGAGACGATGGCTTTCGTTAGTCCGGATATTGCTATGTCAGAT

TTGCAGGCTTCCATGGAGCTTAATAGTGGTTCTTTGAAGGAATTTTCGGTTGAGTCGTTT

AATTTGGATAAGTTTGTATTGGAGGCGGACTTACTTGAGTGTTTGCATCTAGAGTTGTGT

TTATTTCACCAATGTAGATATCTAGAGGGACATTGA

>MS.gene81730.t1

ATGGCGGAAGAACAACCATGCCAAGCACCTGAAGGCTACCGTCTCTGCACAAACAATTGC

AGCTTCCTCGGTAATCCAGCCACCATGAATCTCTGCTCAAAATGTTACGGGGATGCTTCC

ACCAAATCCACCATAAAAAACACTCTCTCATCATCTTCATCCGTCATCGCATCCCCCGCT

TCACCGTCGCTTTCCACATCGGAGCCGATCGTTCAATTCACCAATCCAATGGTTACATCG

AGTTCTGTAACCACCAGTTCTGTTTCTTTTTCGGTTCAACCAAACCGGTGCTTGTTGTGT

AGAAGGCGCGTAGGGCTGACCGGGTTTAAGTGTAGATGTGGGTCCTTGTTTTGTGGGTCC

CACAGGTACCCGGAGAGACACGGTTGTGGATTTGATTTTAAGATGGTGGGGAGAAAGGAA

ATAGCTCAAGCAAATCCTTTGATTAGAGCGGAGAAGTTGAGGAGGATTTGA

>MS.gene81753.t1

ATGTCTGAACAGCTAGAAGAAAATATGGCAAGCTCAGACGAAACGTTTCCATCTGCAGAT

ATTGCTGCAGAACTGAATCCTGGGAATAATACTGAGGTTCAAGTTGATCCTATATCTTCT

GAGGTTGCTGCCTCCAAGGAAATGAAGGAGGAAGCTGCAGCCAAACAGAAGAAAAAGCAA

ATAGAAAAGAAGGAATATTTGGATAAACTAAAATCAGCAATTATAATTTCAAGCATTGTT

GTGGCTCTGGCAGGAGCAGCATTTGCCATAACCAAGAAATTGAAAGAGAAATGA

>MS.gene81738.t1

ATGGCCACTGGAGTTGGTAGAAAAATTTCTGCAGCATCAGCTCGTTCTCACACCAGAAGG

GCCAACAAATCTTCTTCTTTTCAGCTTCCTTCAGGAATTCTTAGAACAACATTAGCAGTG

TTGTTTATTGGGGTTCTAGCATGGGCTTATCAAGTTACGCAAGCTCCTCCTCCCAAAGTA

TGTGGCTCTCCTGATGGACCACCTATAACAGCACCAAGAATCAAACTAAGAGATGCAAGG

CATTTGGCATACAAAGAGCATGGTGTTTCTAAAGATGTTGCAAAGTATAAAATCATCTAT

GTCCATGGTATCAACACCTGCAGGCATGACGCTGTGGTTGCCAACACCCTATCACCCGAT

GTTGTTAAGGAATTAGGGGTCTACATTGTATCCTTTGATAGACCTGGTTACGGAGAAAGT

GATCCCGATCCAAATCGTACGTTAAAGAGCATTGCCTTAGATATAGAAGAGCTTGCCGAT

CAATTGGGATTGGGGTCCAAATTCTATGTCATTGGTTTTTCCATGGGTGGACAAATTGTT

TGGAACTGCCTTAAGCACATACCTCACAGGCTGGCAGGTGCAGTACTCTTAGCTCCAGTC

GTCAACTACTGGTGGCCTGATCTTCCTGCAAACTTAACGGCCGAAGCCTATTCCCAAATG

AAATTGCATGACCAATGGGCGCTTCGTGTTGCTCACTACACGCCATGGCTAACATACTGG

TGGAACACTCAAAGATGGTTCCCAAATTTTAGTGTGATTACTGGTGATCCAGACATCCTT

TCGAAACAAGACAAAGAGCTTGTAACTAAGTTGATGGAAAATAAAGAGAATTATGTGAAG

CAGATAAGACAACAAGGTGAATATGAAAGCCTCCACCGTGATTTAAATATTGGATTTGGA

AGATGGGAATATACACCTTTGGAGCTTCAAAATCCATTTCCAAACAATGAAGGTTCTGTT

CATCTTTGGCATGGAGATGAAGATATTATGGTTCCTGTCACACTACAACGATACATCGCG

CAAAACCTTCCGTGGATTCACTACCATGAACTTCCAGGATCTGGCCACCTCTTCCCTAAT

GCTGATGGTGTGAGTGAGGCTATCATTAAGTCACTTTTAGGTGTGAAATAG

>MS.gene81732.t1

ATGGAAAAATTGAACTCAAAGCTGTACTTGGAGAACTGTTACATTATGAAAGAGAATGAA

AAACTGAGGAAGAAAGCAAAGCTTCTGAATGAGGAGAACCAGGTTCTGTTATCTCAGCTG

AAACAGAAGCTTTCAAAGGGGGGTGGTAGCAGCAACCAGAATGGCAATGGTCCAAGCACT

ACTCTTAACCTTGGTCAAAACAATCCAAGTTCCAACAATTAA

>MS.gene81776.t1

ATGGTGTATTGGTCGTACCCGCCGACGGGGAGGGAAATTGCGGTAACCGCCGCCGTGTTT

ACGATCGGTGCTTCGTTGTTCGGTGTCGGAGCTTATCTTTCGTTTGTTAACGTTGCTCCA

CAGCAGGCTCGTGCGAAAGAACGCAGTGAAGCAATGAGGAATTATCTCAGCAAGCGCTTC

GGTGATTGA

>MS.gene029696.t1

GTTTGTTTGGCGTAAGAAGATTGAGCGTGATGTTGTTCAAGGTGTGCCTATTGATGAATT

TTCAGTTAAAGCTGAGAAAAAGAGACAGAGAGAAAGGATGGCGGAAATTGAAAAGGTGAA

AAAACGAAGAGAAGAGAGGGCACTTGAGAAAGCACGACATGAGGAAGAAATGGCATTGTT

AGCTAGAGAACGTGCTCGAGCTGAGTTCCAGGACTGGGAAAAAAGAGAAGTGGAGTTTCA

TTTTGATCAAAGCAAGTTTAGGTCTGAAATTAGATTGCGTGAAGGACGTGCCAAGCCAAT

TGATATCCTTACCAAACATCTTGATGGCTCTGATGATTTGGATATAGAAATAAATGAACC

ATACATGGTCTTCAAGGGTTTGACAGTGAACGAAATGGAAGAGCTCCGTGATGATATCAA

AATGCATTTGGACTTTGACAGGGCCACGCCAACCCATGTAGAATATTGGGAGGCACTCCT

TCTGGTGAGTGATTGGGAGTTAGCTGAAGCTAGAAAAAAAGATGCAATTGATCGAGCTAG

GGTGCGTGGAGAAGAACCTCCAGCCGAGGTGCTTGCTGAACAGAGAGGTCTGCATAACAG

TGTTGAACCAGATGTGAAGAAGCTTTTGCAGGGGAAGACACATGCAGAATTGGAAGCTTT

GCAGGCGCATATTGAGTCAGAAATGCGTACTGGTGCAGCAAAGCTTGTTGAGTACTGGGA

GACTGTTTTAAAGTACCTCCGCATATATAAAGCCAAGGCTTGTTTAAAGGAAATTCATGC

CAAAATGTTACGCAAGCATTTGCTACGCCTTAAGAAACCATCAGAGGGTGAAGATAAATT

GGAAGATGCTCATGTTATGAAATCTGAGGAGGATATTGAGGATTATGTTAAATTCCAATA

TGCAAAGAGATCACTTTCTCCCGAACCCATTAAAGCAGAGGGTCAAGAAGGTGAAGTAGA

AGATGAGGCTGGGTCCTTTTCACCAGAACTGTTTCATGGTGATGAAAATGAGGAAGCTAT

TGATCCTGAAGAGGACAGAGCTCTACTGGAACAAAAACGTTTGTCTGTAAAAGAAGAGCA

GCAGAGACGAATTCAGGAAGCAATGGCATCAAAGCCTGCTCCATCTGAAGATAATTTTGA

GATGAAAGCCATGAAGGCTATGGGAGCTATTGAAGAAGGAGATGTAATGTTTGGCTCTGG

TGCAGAAGTAAATCTGGATTCACAGGTCTATTGGTGGCATGACAAATACAGGCCCAGGAA

ACCAAAGTATTTCAACCGTGTTCATACTGGATATGAGTGGAACAAATATAATCAGACTCA

CTATGATCATGACAATCCACCTCCAAAGGTTGTACAAGGGTATAAATTTAACATATTCTA

CCCAGACCTTGTAGACAAGACACAAGCTCCAACTTACACTATTGAGAAGGATGGCAGCAA

TGGTGAGACTTGCATCATCAGATTCCATGCAGGGCCACCATACGAAGACATTGCTTTCCG

CATTGTAAACAAAGAATGGGAATATTCTCACAAGAAAGGTTTTAAGTGCACATTTGAACG

TGGAATTCTACATGTATACTTCAATTTCAAACGCCACCGCTACCGCAGATAA

>MS.gene029694.t1

ATGAGTTCTGAATTCGTTAACGGAAGCAGTAGCAGTGGTATGCAGAACCCGCAATTTGTA

TTTGAGAACATATCGCCTAACACCTCTGTCGCGGATCCTAAAGGTCCGTCATCCCAAGGT

GTGATCGGTAGTAATGGAATGAGTCCTTTGGGTGGATTGACCAATGCGGAAAAACGTTCT

GAGAGACGCACGTCGGATGATCCTCTACATAACTTAAGTTCCGTTCCCAAACGTGGTAGA

GGAATCAGCGTTGAAGGACCGTTGTCTTTGCGTCGTAAAAGAAATAGAACTGAAGCTCAT

TCTACAGCATCGCACAATACATCTGATACATCTATGAATTCTGAATTCGTTAACGGAAGC

AGTAGCAGTGGTATGCAGAACACACATTTTGCTCGCGTGTCCAATCAAACGGAATTATTA

AGATCAATGGTTCTTGATTTCGACAGTATATCAGATGATGAATTACGTCATATCGTTCCT

ACTGCTCAAGGTGCATCTTCCAACAACACGTCATCGCTTATACACGAAATGGCCGAATGT

GAACTATTCTTCGATGTTGGAGACCCAGTTTTGTGTTGTGGTTATTGTGGTGCAAAGATG

TGGTTAGGGGAACGTGTTGACGGAAGTGGGACTGAACATCGTGATCTGTTTCCATTATGT

TGCATGAAGGGAAAGGTGCACCTACCCCACCTTGAGAAGCCTCCGGAACTTTTATATAGT

CTGTTACACGGTGATCATCCAAAGAGTAAACACTTTTTGGATAATTTTAGAGCTTACAAT

AGTATGTTTTCTTTCACATCAATGGGAGGTAAGATTGATCGCACAACAAATGATGGGGGT

GGACCTGCAAATTTTGTTCTAAGTGGTCAAAACTATCATCGCATGGGTAGTCTGATGCCG

GAGTCGGATTCTACGCCGAAGTTTGCACAGTTGTACATATATGACACCCAAAACGAGTCT

AGGAATCGAATGAAGCATTTCAGTGGGGGTGATGATGGTCAATCCCTTGATGAGTCTTTG

GTTGAGAGCTTGAAGAATATGGTTGACCAGCACAATGAGTTGGCGAAAAAGTTTCGACAG

GTGAGGGATCATGTCGAAAGAGGTGGCCCATCCAATTTTTATATCCGACTATTTGGAAAT

CGGTCAAAGGATTCTAGAATGCATAATCTACCTACCTGTGACGAAGTTGCGGCTCTAATA

ATTGGTGATGAGAATGAAATCGAAAAGGGTCGAGATATTATTGTTAGGAATTCATCTGGG

CTGTTAGAAAGAATATATGAGACACATGTTTCATTTATGCCTCTTCAGTATCCACTTCTG

TTTCCGTTTGGCGAAGATGGATATCATAGGAATATTAAAATAAGATCTGAAAACCACGGG

AACGTTGAGCGTAAAAGAAAATTTGTTACCATGAGGGAGTTCATGGCGTATAGGATCCAG

GACAGACATGCTGAGTACGGTAACATTGTGCGTTCTAGGAGACTATTCATGCAGTTTGCC

GTGGATTGCTTCACGATGTTGGAGACTCAAAGACTATCTTACATTAGAAATAACCAGCAG

ACTATACGTTGTGGCTATTTAAATGGTTTGCATGAGGCTATGACGAGTGGTGAGACCAAC

GCACAAGATATTGGTCGTAGAGTAGTGCTACCACCGTCTTTTACAGGAGGAAGGAGATAT

ATGTTTAACAACTGTCAGGATGCCATGGCTATTTGTAAGAGATTTGGGTATCCCGACCTG

TTTATCACTGTTACATGTAACCCAAAATGGCGAGAGATACAAGATTTTCTTCAAATCCAC

GGTCTCAAGGCGAACGAAAGGCCTGATATTATTTGTAGAATCTTTAAGATGAAACTGGAT

CAATTGATGGATGATTTGAAGAATGATGAACTTTTCGGAAAAGTTGATGCAGGTATGTAC

ACGGTTGAATTTCAGAAAAGAGGGCTTCCTCATGCTCATATTTTGTTGTGGTTAAGTGGA

GATTGTAAACTGCGTACATGCACTGATATCGATAAGATAATATCCGCTGAACTTCCTGAT

CGAAAGCTTTATCCGAAATTGTTTGAGGCAGTCACCTCCTTCATGATCCATGGACCTTGC

GGGGATATTAATCATCGATCGCCATGTATGAACGCTGGTAAATGTACCAAGTACTACCCA

AAAGGATTTCAGTCAACAACTACAGTCGATGAAGATGGCTACCCCAAGTACAAGCGGCGT

GACAGCGGAGTGTCGTTTGAGAAGAATGGTGCTACCATTGATAGCCGGTATGTTGTTCCG

TACAATCCGCACCTTTTAGTCCGCTACGGAGGTCACATAAATGTTGAATATTGTAATAAA

TCAAACTCGATCAAGTATTTGTTCAAATACGTCAACAAAGGTCATGATAGATCAACGATC

GGGGTGTCGAAAAAGGACAATACAACTGATAAGGATGAACCTGTTGATGAGATACAACAA

TACTATGACTGCAGATATGTTTCACCGTGTGAATCTGTTTGGAGAATTTTTAAATTTGAT

ATTCATCACAAATGGCCGTCAGTTATGAAACTAACATTTCATCTGGAGAATGAACAATCT

GTGACGTTCGACGACGATGAGGATATTGATGAGGTTGTTGAAAGGGGTGAACGTTTGTGC

ACAATGTTTCAAGCGTGGTTTAGAGCAAATCAAATGTATGCCGAAGGTCGAGACTTGACA

TACTCTGAGTTTCCAACGAGATTTAGGTATATAAAAGATGAGAGAAGGTGGCAGCTTCGT

CAAAGAGGAGAACAAATAGGAAGACTTCAGTACACTCCACCTGGTGTTGGAAATTTATTT

TACATGAGGTTATTGTTGAATGTTCAGAAAGGATGCCAGGGGTATGAGTGCTTGCGCACT

ATAAATGGGAAGAAACATAACAGTTTTCAGGAAGCGTGTGATGCTCTTGGGTTATTATCC

GACGACAAAGAGTTCATTGATGTTATCAAAGATTCGGGCGAACTGTACTCCGGTCCGCAA

CTCCGTAAGTTGTTTGTTCATCTTATGACAATGTCTTCAATGAAAGATCCAGGTGATGTT

TGGAGTGCAACCTGGAAGTTACTGTCAGACGATATTGTGTACAACAGAAGGCGGTTGCTA

AAAAACCCAGCCAACTAA

>MS.gene029695.t1

ATGATTAAGAACAGAACAATATCATGGAACTCTCTCTTCTCAACCATCATCACCATCCTT

CACTCCTTCATTGCACTCTCACATCCAATCTCTTCACCACTAAGAGGAGCCAAAATTCCT

GAAAGTGATTTCAAAACTTTGCCCAATGGTCTCAAGTACTATGATTTGAAGGTTGGCGAT

GGAGCTGAAGCTGTGAAAGGATCGCGTGTCGCAATTCACTATGTTGCTAAATGGAGGGGA

ATCACCTTCATGACTAGTAGACAAGGAATGGGTGTTGGAGGAGGAACGCCCTACGGATTT

GATGTAGGCGAATCTGCGAGAGGAAATGTCTTAAAAGGTTTGGATGTAGGAGTAGAGGGC

ATGCGAGTTGGAGGCCAGCGGTTGTTAATTGTTCCTCCTGAACTTGCCTACGGAAGCAGG

GGAGTCCAAGAAATTCCTCCAAATGCAACAATAGAGATGGATATTGAACTGCTTGCCATC

AAACAAAGTCCATTTGGGACTGCCGTAAAGATTGTTGAAGGCTAA

>MS.gene034649.t1

ATGAAACAACAAGATGATCATGCCTTATCTGAAGACAATTTCACCCCTGATCATACTTCA

CCTTCTATTCCTTCACCAAGAAAATCACCTAGTCTCATCCCTTTTGACAACCAATCATCA

CATTTTCCAAACTTGGATGTGGAATCAGAAGAAGGTTTTAATACGATGTTTCTTGATCTG

GTCAAGCTTATCCCCTTTAACCAAAACCCCTATCAGTACTACTCCATGACTGGAGGCTTT

CATCCAGATGAAATGCATGAGATTTCTCAAGGACAAACGACACCATACCTCCTTTGCCCC

TTTAACCTCCTACATATCACCCACAAACCTTCTGATAACTCTCTTCTTGATGCTGCTCGC

AATTTTCTCACCTCACTAACACTTGATCCACAAATCAACAAACCCACTTCTTCCTCTCCT

TTACCCGAACCCAGCCACCTTGGTTCACTTGCTTCTTTTGTTAACACTGATACATCCAAT

TCTAATGGTCTTCCTAAGATACCATCTATGAATGTTAATATGCCTCCTGCTACTAATGTT

GTTGAAAGAGGACCAACAAGCTTCGATGTGATCCAAAACTTGATTGGTTTACAAAAAACT

GTTGCTAGCTATGGTAACCGTCAAGTGATTATTGACAACTGGATCCTAGGAAACTTTCTG

TCTGCTGTTGCACCAACTTTGCCTGCTCCTTTCTTACCTAATATTGTGTATCCCATCGTG

CCTGTTATATTAGTTGCTAAGCCCACTGATCCCACTCCACCTCCAGCCTGA

>MS.gene034644.t1

ATGACTTATAAGGAAAAGGGTGTTCATGTTGGCAGCACTTCTTGCGGAACGAGGAAACAA

GGTAAACTTAATGAGAGGGGACTTATTCTGAACAAATCTGAAAAGGTAAGATATAAAATG

CTTTTGAACAGAGACATTACACCCAATAGGTATCCCGATTCAGAAGCTTTAAGGGCATTA

GAAAATGCCACACACCCAAACCACATCGTTTCATTTAGTTTGGGTAACATAGAATATAAC

ATGCCCCTTAATGAATTTTGTGACATAATGGGCTTCGCAAGCACGGGGATCATTCATGTA

TCTCGTAAGCATGATACTAGGCCTCAAGATTATGACCAACATGAGTTTTGGTTTAAAATA

ACTGGGCGGAACCATTATGGATCTAAAACTGCCAAGACTAGTATGATACATAACCCTGTT

TTCCGATACTTATATAGAGTTATGGCCTGCACCATTGTTGGTCTGCCGAAAACTGCAACG

GTGAGGACTGATGAGCTATTCCTTTTATGGGCTATGGTTAAGAAATGTCCTGTAAACACT

GGCTACTACCTTTTAAACCATTTGGCATATGTGACGGCTCAACCCAAAGGTCAGATAGTG

GCGGGGGGATTGGTTAGTTTCATTGCTTGGAAGTTGGGAGCGAGAAGTGCAGCTAAAGAA

ATTGACATTGAGGGGAACTATACCATTGATCTTGATTTCTGTAAACAAATTCATATGGTT

AGGGATCTAGATGGGAACAACAGGAATTTTAGATTGTTGATATTCGACGAAGATTCAATT

TTAGCAGCCGGCAAAGATGAAGAAATGGGTGATTACCAGGAGAATCAACAACCTATGGAA

GGAATGCAAGATGATGAAAGGATGGAGGCGAAGGTTGACAAGACAAATGAGGATCTTGCA

CTCGTCAAACAAATGCTTGTAGCAACGATGCAACACCTGAACATCCAATATCCTCCAGTC

GGTCCACAGTAA

>MS.gene034648.t1

ATGGATGTTGTTTATGTGAATCCAAAGGATAAGGCAATGTTTTCTAGAAAATGGGATTAT

AAAACAATTGCAAATGGATGGCTAGTGGATTTAGGTGATATGGCTTCAAGAGGTCAAAAT

CTGAAACCACACTTTGATGCTTTGGGATGGACAAATTTCCTATCTCTGAAGGAACTGCAA

TACACACGCCTCACTCGTGCTTTTTATGCCGCTGTGAAATTCAGTTCAAACAACAATATG

GCCATTACTCTCAAAGGCATTTCTTTCAAACTTAATCTGGAAGTTATCTTGGCCTCAGAA

GATCGCCCAATAGCTGTCAACCTCAAGCCAAAGTGTCACTTGTTTCACAACATCTGTGTT

CGCACCATCCTCACTAGAGCCGAAAGTTGGGATAAGGTCACTGACACAGACTTGTTAGTT

GTTTATCACTTGCTGAAAAAGAAACCTCTTAACCTAGGTTATTTAATCTTAGCACATATG

AAAAATTCATCTGTTCACCGTCGCTCTGCACTCTATGCTATGGTGTTGACTAAGATTTAT

AGAGAATTTAATGTTCCTCTAGATGATGAAATTGGTTCAGATGAATGTTGTGTTATATGT

GGTCAAACTGTAGGAAAAGTAGAAGAACCTGAGGCTGAGGCTGAAGATGTTTTACATGAG

GATGAGCATGTTCTGTCTGAGGAAGAATATGAGAGTTCAGAACCCTCTTATACTGAAGAT

GACTCTGAGTTTGAGAAAGAGGATGACCCTTAG

>MS.gene034642.t1

ATGGATTCGGATAAAGTTCCATCTCCGAGGGATTTGTTGAAATGTTGTGATTGTGGATGT

AGTTGTTCCTTGGTGGAACAATGTTCTGAAAAATGGATGCGTTCTGTTAAGCGAAAGCAC

AATGAGTTTAAATTGGATAGTGAATTACCTGTGTCGTCGGTTGCGCGGGTTGATATTATG

AATGAATGTGTTGTGTTACGTGAGATGGTAAGTATGCAGCAGAGAACGATTCAGGATTTG

AATGAGGAGTTGGAGGAGGAGAGGAATTCTGCGTCCACAGCTGCGAATGAGGCAATGTCG

ATGATTTTGAGGTTGCAGAGGGAAAAGGCGGAAGTTCAAATGGAAGCTCGTCAGTTTAAG

CGTTTTGCAGAGGAGAAAATGACGCATGATCAGGAGGAACTTATGTCTTTGGAAGATTTG

TTGTACAAGAGAGAGCAGATAATTCAGTCCCTTACATGCGAAGTTCAGGCTTATAAACAC

AGGATGATGAGTTTTGGGTACACCGAGGATGAGGTCGAAGGTGATCAATATGATGATATT

CCTCCATACGAATACCCTCCTTTAAAATGTAATGTCATGTATAATGGCATGGATGCTGAT

AATGACGACACTGATATTGAGAAATATGCATTTGGTGAAACTCCTAATGACCGTTTGAGG

AGCTTGGAGAATAGGATTTCTCAAATGGAGAAAACTCCCACTTACAGCCAGGTGGATGGT

GATTTTATTGGAAAAAATGTTACGGAGAAAGTAATTGTTGGACAATCTCCAAATTTGACA

AGCCACGCGAGGAAATTTTCCTCTGACTCAGCAACGTTCGGTGGGATGTGTAAAGAAATA

GGTTCCGAGTTCCCTACAGAGTCTCCCAAGAACAATAACAAGTATAAGAAGGATTATTTC

TCTCAACCAGAGGACAATTCAAATTTGAAGAAGGTAGATAATGCTTCAGAAGGTGATGAT

ACGAGTGACAGAATTTATACAATTGACTCTGTTCATTCTGGGGCACCGGATAATGGCTTT

ACAGGTTCCAAAGCTGGAGGTTTCGATGATTATGCAACCAGTCCAAAGGAATCAGGGAAT

CATCATGCTGATTTCGAAGATCCCTACATTAAGAAGCTGTACATGAGGCTTCAGGCACTT

GAGGCTGATAGGGAATCAATGAGGCAGGCAATCATTTCAATGAGCACAGATAAAGCACAG

GTTGTGTTACTGAAGGAAATAGCTCAGCATTTGTGCAAAGAGATGTCACCGCAAAGAAAA

ATGAGTACGAGCAAGCCTGCAAGATCTCGGTACTTGTCAATTTTTAAGACATTGCCTTTG

TATACCGATCTTCCCCCCTCACAAAAAATACTCTGA

>MS.gene034651.t1

ATGCTTAAAAAACTCGTTCAAAGAATCACACAATTTCCTATTCCACAACAAACATACGCA

ACCACACGTTTCCGTCATTTATACTCCACTCTCTCCATTTACGATAACATCCCAAAGAAA

CTGAAGAAATTCGAGAGAAAACCATTGGTAACAAGTATCAACGAACTAAAACGCCGAGGT

AGAAAGAGGAGAAAAGAGAGACAAAATGTGAAAGAGATTATTTTACAATCTCCTGAAAAT

GGTTTGTTTGTTCAAAAATTGATTCCTGTTGCCAAACAAGTTTATGCTGCTAGATCTGAA

GTATTGTCTAGTGTTTCTAGACTTGTCAACTTTATTGCCATTTATTCATGCAGCATATGT

GGAGACGTTCATGTTGGTGATCCACCACATAAAATTAAAACATGTAATGTTAGAGGAAGC

TTGTCAAGTAAAGAACACAGTTGGGTCAAAGGTGGTATCGAACATGTTCTGCCTCTTGTA

GAATCATTTCATCTGTATGATCGAATTGGGAGGGCTGTTTCACATAATGAAATGCTTCTT

GTGGACCGAATTCCAGCAATTGTTGAACTGTGTATCCAAGCAGGTGTTGACATACCTGAG

TACCCTACCAGAAGACGGACCTTTCCTGTTTACTGTGTTGCTGGTAGGATAATTGATTTT

GAGAAAAGATTTCCTAAAGAAATTTCTCTTGAAAAAGATATAGACGAATATGGATTTCCA

TATATGAAAAAGAGATCTGATCAAGACACTAATTCTATGGAAATGCATTGTGATGACATC

CAAGCTGTTGCTGTCCGAGGCATGAAAGCCTGGAATAAAATGTGCACCGGAGCTTCAAAA

CTTATGGAGAAATATGCTGTACAAACTTGTGGATATTGTCCAGAGGTACAAGTGGGACCT

AAAGGTCATAAAGTGCGAAATTGTCAAGCTTACAAACACCAGATGAGGGATGGGCAGCAT

GAATGGCAGGAAGCTACAGTAAATGATTTTGTACCTCCAGTCTACGTCTATCATATTCGA

GATCAAGAACCCGGTAAGCCTTTGGTAAATGAGTTGAAAAGATATTATGGTATGTTGCCT

GCAGTTGTTGAGCTATTTTCACAGGCTGGGGCACCAGTTGAAAAGATTTATGGACATACA

ATGAGAGATGTTGTAGTCCCTGACATGGATGAGGAAAAGTGGGTTGTTTAG

>MS.gene034646.t1

ATGACGAACAAGAGAAAGGATGTTGTTGATGATGGTTCATCTGATGCCAAGAGGAAAAAG

GTTGCTGATGATGCTAGCTCTTCTAATGGCAAGAAAAAGAAGGGTAAGGCTAATGAACTA

GGAATTATCCTAAAGAAATCGGCTGAAAGGGAAAGGTTCGACAAACTTGCTGGAAGAGGA

ATCGCAGTGAACAGGACTGACACATCAATTGCAGAAAATTGGATTTATTCTGATTTTGGT

CCACAAGTTCCTGAAGAGCAGCATGATGAAGATGACGTACCGCCTGATGACCAGGTGGAA

CATCAAGCACTCCCGGAGGTTCAAGATGAGCAGGAATGGCGTGCAAGGATAGAGGATGAG

GTTCGAGGGACAAGGTCCGATTTTCAACATATAAGGCTGGACCAACAAAGGCAGGTTCAA

GATGAGCAGGAATGGCGTGCAAGGATGATGGCTGAGGTCGAAGGTGCAAGGTGTGATGTT

CAACAGATGAGGCAGGACCAGCGAAGGCAAGGACATATTCTCGACCTAATGATGCAGCAC

CTTAACATTGAATATCAACCTCCCGGTGGATTCTAA

>MS.gene034652.t1

ATGGAGAGAGGAAATACGATGGGGATATCTAGGACCCAATCAGATCAGCTGGCGGAGTCG

ATGGTGGCGGCTCTAAAGTCGCCACAGTCGAGTGATCACTCGACAAACGGAGCATTGGAA

GAGAGTGGAAGACTTTCGAGAAAATCAAGCCGGAGGATAACGGCTGCGTCGCCGGGCCGT

GGAGGAAGTAAGAACTCACACATAAGGAAAACAAGAAGTGCTCAATTGAAGATTGATTTT

GATGAGTTGGGTAGTGGTGCTGCTCTTAGTCGTGCTTCAAGTGCCAGCTTGGGTCTCTCA

TTTGGGTTCACTGGCTTCACTATGCCTCCTGATCAAATTGCTGATACCAGACCATTTAGT

GATGATGATATGATTCCTGAGGATATTGAAGCCGGAACAAGGGCAAGGATAAAGTTTCAA

ACTGAACCTACTGAACCTATATCTCTAAAGTTCACTGATGTGACCTACAAGGTAGTACTC

AAAGGCATGACATCAAGTGAGGAGAAGGATATCTTGAATGGGATAAGTGGTTCTGTAAAT

CCAGGTGAAGTTTTGGCATTGATGGGTCCCTCAGGAAGTGGAAAGACAAGTCTTTTAAAT

CTCCTTGGAGGAAGAATAAGCCAGCCCACAGTTGGTGGTTCTATCACTTACAATGACCAA

TCATATTCCAAGTTTCTCAAAAGCAGGATAGGATTTGTGACACAAGATGATGTTTTGTTT

CCTCACCTAACTGTGAAAGAGACATTAACGTACGCAGCTCGGTTAAGACTTCCAAAGACA

TTAACTAAGGAGCAAAAGGAACAAAGAGCTTTAGATGTCATCTATGAACTTGGCTTAGAA

AGGTGCCAAGATACTATGATTGGCGGTTCATTTGTTCGCGGAGTATCCGGTGGAGAGAGG

AAGAGAGTTTGTATTGGCAATGAGATTATAATCAATCCTTCCATTCTGTTTCTTGATGAA

CCAACATCTGGTTTGGATTCAACAACCGCTTTGAAGATCGTTCAGATGCTGCAAGACATA

GCAGAGGCTGGAAAAACAGTAGTGACAACAATTCACCAACCATCAAGTAGACTGTTTCAC

AAATTTGACAAGTTGATCCTTCTTGGGAAAGGAAGTTTGCTTTACTTTGGAAAAGCATCA

GAATCAATGGATTACTTCCAATCTATAGGATGTTCACCGCTTATTTCCATGAATCCAGCG

GAGTTTTTGCTAGACCTTGCTAATGGAAATATAAATGATGTTTCTGTACCGTCGGAATTG

GAGGATAAAGTTGGAAATGCAGCAGTTGAAACATATAATGGAAAACCATCACCAGCAGCT

GTCCATGAGTATCTAGTGGAGGCATATGAATCTCGAGTTGCGGAAACAGAGAAGAAAAAA

ATAATGGTTTCTGTACCTCTAGATGAAGATTTGAAGGCGAAAGTGGTTTCTGCTAAAAGA

GAATGGGGAGCAAGATGGGATGAGCAGTTTTCCATACTTTTTTGGAGAGGAATCAAGGAA

AGGAGGCATGACTATTTTAGCTGGTTAAGAATCACACAAGTTTTATCGACTGCAATCATC

TTAGGATTACTCTGGTGGCAATCCGATGCTAAGAACCCAAAAGATCTGCAAGATCAGGCA

GGACTGCTTTTCTTTATTGCGGTGTTCTGGGGATTCTTTCCAGTTTTTACAGCAATATTT

ACATTTCCTCAAGAAAGAGCCATGTTAAATAAGGAACGCGCAGCAGATATGTACAGATTA

AGCGCATACTTCCTTGCTAGAACAACGAGCGACCTTCCATTAGACCTGATATTACCAGTG

CTTTTTCTCCTTGTTGTTTACTTCATGGCTGGTCTAAGACTAAGTGCTGCTCCCTTTTTC

CTCACCATTCTTACTGTTTTCCTCTGCATCATTGCAGCTCAGGGTCTTGGACTTGCTATC

GGTGCTACGCTTATGGACTTGAAAAGAGCAACAACTTTGGCTTCGGTAACGGTGATGACC

TTCATGCTGGCTGGAGGATTTTTCGTCCAG

>MS.gene034650.t1

ATGGGCAACAACTGTGTGGGGCCAAACCTCGGTGGCAATGGTTTCCTTCAATCCGTTTCC

GCGGCCGTATGGAAAACCCGTCCACCAGAGGCAAGGCTTCCTCCTCCTAACGCCGAAGAT

AAAAACAAAACTCCGTCAACACCGGAAAATGCTGTAACAGGTTCCTCGAAACCGGAGTCT

AGTGCAAGTGGGAAAGTTTCTGAACATGTTTCGGTTCAGAGCACACCTCCTGAACAGGTG

AAAATAGCAGCCACAGAAATAAAACCGCTCGAACATGAAAAGCCGGTTAAAGCAGCAGCA

TCTAGTGCTGTTGCTGCTGGTGCCAGTGCTGGTGGTGGTGGTGCTGCTGCTGCTGGTACT

GGTGGTGGTGGTGCGGGTGAGGGTGATGCAGATAAACCTAAGAAACCGACACATGTGAAA

AGAGTATCAAGTGTTGGACTTCAGGTAGAATCTGTATTGGGAAGGAAAACAGAAAACATA

AAAGATTTTTACAGTTTGGGGAGAAAATTAGGTCAAGGGCAATTTGGGACAACATTTCTA

TGTATGGCAAAAGGGACTAATAAGGAATTTGCATGCAAGTCAATTGCAAAGAGGAAATTA

ACAACACAAGAGGATGTTGAGGATATAAGAAGAGAGATTCGGATAATGCACCATTTGGCG

GGTCATCCGAATGTGATACAAATAGTTGGTGCTTATGAAGATGCGGTTGCGGTTCATGTT

GTTATGGAGTTATGTGCCGGTGGTGAGCTTTTCGATAGGATTATACAGAGAGGACATTAT

AGTGAAAGAAAAGCTGCTGAACTTACTAGGTTGATTTTAGGTGTTGTTCAAGCTTGTCAT

TCTTTAGGGGTTATGCATAGGGATTTGAAGCCGGAGAATTTTCTATTTGTTAGTCATGAA

GAAGAATCGGCACTTAAGACGATTGATTTTGGACTCTCGGTGTTTTTTAGACCAGGTGAG

ACATTTACTGATGTGGTTGGAAGCCCATACTATGTTGCTCCTGAAGTTTTACGGAAGAAC

TACGGTCAAGAATGTGATGTTTGGAGCGCTGGGGTGATCATTTATATTCTACTCAGTGGA

GTTCCACCATTTTGGGATGAAACTGAACAAGGAATATTTGAGCAAGTATTGAAAGGAGAG

CTTGACTTTATTTCTGATCCGTGGCCAAGCATATCTGAAAGTGCAAAGGATCTTGTTCGA

AGAATGCTTATAAGGGATCCTAAGAAGAGGATGACGGCACATGAAGTTCTTTGTCATCCT

TGGGTGCAGGTTGGAGGTGTTGCTCCTGATAAACCGCTTGATTCCGCAGTCTTATCTCGT

TTGAAGCAATTCTCTGCAATGAACAAGCTCAAAAAAATAGCCATTCGAGTAATTGCTGAA

AATCTGTCCGAGGAGGAAATTGCAGGACTTAAAGAAATGTTCAAGATGATAGACACAGAT

AATAGCGGACAGATCACTCTTGAAGAACTGAAAAATGGCTTAGAAAGAGTTGGTTCGGTT

CTTAAGGATTCTGAAATCAACTGGTTAATGCAAGCGGCAGACGTTGATAATAGTGGTACC

ATAGACTATGGTGAATTCATAGCAGCCATGCTTCATCTAAACAAGGTTCAAAAGGAGGAT

CATCTATTTGCAGCCTTTAACTACTTTGACAAAGATGGGAGTGGATACATTACAAAGGAT

GAGCTCCAACAAGCCTGTAACCAGTTTGGCCTACAAGATGATCATCTAGATGATATAATA

CGTGAAGCTGATCGAGATAACGATGGTCGCATTGACTACAGTGAATTTGTTGCAATGATG

CAAGACACTGATTTTGGCAAAAAGGGGCTATAA

>MS.gene034640.t1

ATGGCTGCATCCCATACTGTTTGCCTCATGTTGGTATTTATAGCTAGTTTTCTTTTGTTA

GTCCAAACTGGGCTTTCTGATGATGTTACCGATCCACCAATTGGATCCCCTACTGAATAT

ATTTATGCAGAAGGGCCATTGTCTTCGTATGAGAAGTATTTGACTGATTGTGCAACTAAG

TTATACCCAATTTGTGGTGAGGAGATATTTTCTACTGTATTTTTTGGTAATACAACTTAT

AGCAATGTATGTTGTTTAAATCTTGTGAATGATGTGGGAAAACGCTGTCATGATGGTCTG

GTGAAATATTTTCTGTCATCTCCTAAATTCAAGAAAAACATAATTTCAATTTGGGAAAGG

AGTGATCAAGTTTGGAATGATTGTGCATCTAGAGCACAAGATATTACTCCAGTAGAAGCT

GAACCTCCAGTAGAAGCAGCTGAAGCTCCATATAATGTCTCTATGTTTGTTGAGGAATTT

AACTTTGCAAACCTGCCCCTTAAAGGCCCTCAGGTGTGTATTATTCTAAAATGGAAGCCT

CCGCCAATTGGTTCAATCAAGATAAATGTTGATGCACGGTGCTTTGGTGATGGAAAGATA

GGTTGGGGCCTTATTGTTAGAAATTCTGCAGGTCTGGCCCTGTTTGCAGAATCTAAGGCA

TATGGTATATCCGTGTCTCCGTTGTTGGATGCATGCATGGGCGAGAGATGGTGCCTTGCT

TGGATCTTGGAAAAACATTTCGCCAACGCAATTATTGAAACTTATACAGAAATTGTGGCG

AAGAGACTCTATGGTGGTCCTTTGCAGGCCAAAATTGAACCTTTAATCCGAGATTGTTTA

GACTATCTTTCTAAGTTATTGAATGTCTTTGTCTGTAGTATAAGTAGAGAACGTAACGCA

GTGGTTCATAGCGTTGTTAACATGTCTGGGTGTAGGATGTGGAAGGGATGTATCCCGGAT

CTTTGTTTAGCAGATTATTGTAATCCTCTTTTAAATGCAAAAGCTGGGTTTATGTAA

>MS.gene034643.t1

ATGTATCCCAATCATCTCAATCTCGATGGCTTGACCTTGTCCGAGGAAGGTTTAACCCTA

AACCTAGAGTCTAAACCTGAAATCGCTGCTGTCCTCAAGCACTGCCTCATTGGTCGTGTG

CTTGCTGATCGTGAAATCCAGTTTGCCTATTTCAGCGAGCGTATGTCTCATGCTTGGAAG

CCAGGCAAACAGGTTACAATTACTAAGTCTGTGAATGATCGCTATTTGTTCCAATTCCAT

CACAAGGTTGATGCAAACCATGACAATTTTCATATTGTCATGGATCGAATCTCACCTGGT

GTCGTCCCAAACTTTGTCCCTCTCAACCATATCGATTTCTGGGTTCAAGTACATGCTCTG

CCCTTTGGATTTATCCAGCCGAAAGTAGGTCAAGGTCTAGGCAGTTTCTTAGGAACCCTA

AAAGCTTATGATGGCCGCAACTCAATCCATAGCTCCTATATGCGGATCAAAGTTGCTATT

GATGTCACTGTGCCTTTGAAGAAAGCATGGCCTGATTGGTACTGCTGCAACCAACCGCGC

TGGCTGCAAGATCCCATTCCCGCATCTGTGCCACGCCAAACCAACGCTGCTGGTGGCACT

GGTGCTGTTCCTGCTGGCTGCGACACACATGCTGCTGGTACGGGAACTCTGACATCTTTC

AAAGACCGTATGCTTGCTTTCCAGCACCAACTCACTGCCATGAAACAAGATGTTATTGCT

GCCCAAAATGCAGCGCATGCTAAAAAAGGTAACGGCTCTAATTCTGCTTGCAAAGTGCAA

CTTCTGGCTTCCTCCTCTACAGGAAATTCGGCCTCTAGTTTACTGCCAGGTCACCCACTA

GTGCTTGGTTTACCTGCTGTTCCGTTTGTGACTGAAAATAATGAGGAAGTGAATGAGGAC

AATGGCTCGGAGTTGAAGAAAAGGAAGCGCATGTTTGCCCTTCAGAATGGAGAACCATTT

GCTGCTGATGGGACTCTTGTGGCACCTACTTCCGATCACTACCCTATTCTTGTAAATGTT

GCGCCTATACCTCGGCCTCATGTTCATAAACGACATTTTCGTTATGAAAATGCGTGGCAA

CTTGAACCGGGGTTTAAGGAGCTAGTTACTAACTCTTGGCAGGATCATTCAACAAGTACT

CTTATGCCGAAATTATCTTTATGTGCGGAAGATATGTGGGAATGGAAGAGGAATCATTGT

AACAAACTGAAAAAGGATATTGAAGATTGCCGTAAGGATTTGGAAGAAATACTGCTGCAT

GCTTCCGGGGAGCATCAAAACCGCATGCTAGAGCTTAGAAAACGCATGCAACGTCTGCTG

TCCCAAGACAATGCCTATTGGAGTCAACGCGCTAAAACTCATTGGTACAAGGATGGCGAC

AGAAATACAAAGTTTTTTCATGCCTCGGCAACAGCCTGA

>MS.gene034647.t1

ATGACGAACAAGAGAAAGGATGTTGTTGATGATGGTTCATCTGATGCCAAGAGGAAAAAG

GTTGCCGATGATGCTATCTCTTCTAATGGCAAGAAAAAGAAGGGTAAGGCTAATGAACTA

GGAATTATCCTAAAGAAATCGGCTGAAAGGGAGAGGTACGACAAACTTGCTGGAAGAGGA

ATCGCAGTGAACATGTATCCCTATAAAACTACGTTAAATGCATTAGGTATTAGAGAAAAT

GTTAGAATTCTGGAAACTGGTAAGATTGTGGTCGGGGAGCCGATAGATTACATTGCCAAG

AAGTTAGGAATAAAATTTTATTCTTATCATCCTCAAATTCCGGATTACAAGGGAATTGAT

ATTGATACACTTATTCAAATGAAAATGATTAAGGATGTAAGTGGGGGGAAGAGAGAAAAA

TTTCAATTGAGGGTTGGCAACCAAGACTCATTTATATTGCCAAATCCTGATAGGACTGAC

ACATCAATTGCAGAAAATTGGATTTATTCTGATTTAGGTCCACAAGTTCCTGAAGAGCAG

CTTGATGAAGATGACGTACCGGCTGATGACCAGGTGGAACATCAACCACTCCCGGAGGTT

CAAGATGAGCAGGAATGTCATGCAAGGATAGAGGCTGAGGTTCAAGGGGCAAGGTCCGAT

TTTCAACATATGAGGTTGGAGCAGCAAAGGCAGGTTCAAGATGAGCAGGAATGGCGTGCA

AGGATGATGGCTGTGGTCGAAGGTGCAAGGTCTGATGTTCAACATATGAGGCAGGACTAG

>MS.gene034645.t1

ATGGCTCTCACCCATATACACACCCATATACATGTTATGATTCCAGATTCCCCAATTGAA

TTCTTTTCTGCAGTCAAATTATCCTTTAAGGCACGTGTTGAAGTTGATTCGAGTTTTGTC

GAGAAATGCACTGTTGATTTTGTTGACGAGTGGAACGTTGTTTCACCTTATGGAAATATT

GTAACGCTTAAGTTAACAAAAAACTTCTTTAATCCAACTATAACCAATGGTTGGAATACA

CTAAAACAATACCACAATTTCCCAACCAACGTGGAGGTAATGTTTGCTTATTATGGAATG

AATTTATTCAAAGTCCAAGATTTTCGTCAAGTCAATTTCTTCAGCCAACTGTCTGCCTTT

CATAGTAGAAGTTTGAAGCGAAATGAAACTAAGTTTTTTGATATTCAATTACGGGAGGGT

CTATTAACAAGAAACAAGCTGGTTAAGTCAACTATACTTGCTTGA

>MS.gene034641.t1

ATGACCAAAAGACTTAAGCATCGTCCTGCAAAAGTGCCTATAGCTCATTTTAGGGAGCTT

TGTGAATTCTGGAGTAAGCAGCCTATACAAGCATTGGCTGAAAGTAACGCTAAAAATAGA

GCTCAATTGAACGTGAAAAGGAAAAAAGAGAGCCCACTCAATCAGAAATGTTTTTTGAAA

CTCGAAAAGGAAGTAGAGGAAAGCAATTGGATGAGGAAACTGGAAAAGTATTTGTATGTA

AAAGGAAAGGAGCGTCCCGACAGGTTACGTTGTTATGGGAGAACAGTAACTAAAACTTCC

TTAAAGCGAAAGGATGAGATTAATGCTTTGAAACAAGCACATAGTCAAGAGGTATCTACT

TTAAGGCATGAATTTCAAGATCAGATTGATAGATTGCAAAATGCGTTTAAGACCGTCATA

CAACAATGCAATCCTCAATTAAATATGGAGTCAATAGAACATTTGCTAGGATTATCTCAT

GGAGATGCTAATAGTTCCCCAAAGGATAGTAGAGCACAAATGCATTCCTCTACTTCAATT

CATACTCCATGTCCTGAAAAGCAAGTTATCAATGAAGATGCTGTACAGGATGACATAGAT

GAGGAATTTCAAGAGGAAGACGTAGATGATTAA

>MS.gene034653.t1

ATGTCTGTTGCAGCTGAGGATATTGAAGCCGGAACAAGGGCAAGGATAAAGTTTCAAACT

GAACCTACTGAACCTATATCTCTAAAGTTCACTGATGTGACCTACAAGGTAGTACTCAAA

GGCATGACATCAAGTGAGGAGAAGGATATCTTGAATGGGATAAGTGGTTCTGTAAATCCA

GGTGAAGTTTTGGCATTGATGGGTCCCTCAGGAAGTGGAAAGACAAGTCTTTTAAATCTC

CTTGGAGGAAGAATAAGCCAGCCCACAGTTGGTGGTTCTATCACTTACAATGACCAATCA

TATTCCAAGTTTCTCAAAAGCAGGATAGGATTTGTGACACAAGATGATGTTTTGTTTCCT

CACCTAACTGTGAAAGAGACATTAACGTACGCAGCTCGGTTAAGACTTCCAAAGACATTA

ACTAAGGAGCAAAAGGAACAAAGAGCTTTAGATGTCATCTATGAACTTGGCTTAGAAAGG

TGCCAAGATACTATGATTGGCGGTTCATTTGTTCGCGGAGTATCCGGTGGAGAGAGGAAG

AGAGTTTGTATTGGCAATGAGATTATAATCAATCCTTCCATTCTGTTTCTTGATGAACCA

ACATCTGGTTTGGATTCAACAACCGCTTTGAAGATCGTTCAGATGCTGCAAGACATAGCA

GAGGCTGGAAAAACAGTAGTGACAACAATTCACCAACCATCAAGTAGACTGTTTCACAAA

TTTGACAAGTTGATCCTTCTTGGGAAAGGAAGTTTGCTTTACTTTGGAAAAGCATCAGAA

TCAATGGATTACTTCCAATCTATAGGATGTTCACCGCTTATTTCCATGAATCCAGCGGAG

TTTTTGCTAGACCTTGCTAATGGAAATATAAATGATGTTTCTGTACCGTCGGAATTGGAG

GATAAAGTTGGAAATGCAGCAGTTGAAACATATAATGGAAAACCATCACCAGCAGCTGTC

CATGAGTATCTAGTGGAGGCATATGAATCTCGAGTTGCGGAAACAGAGAAGAAAAAAATA

ATGGTTTCTGTACCTCTAGATGAAGATTTGAAGGCGAAAGTGGTTTCTGCTAAAAGAGAA

TGGGGAGCAAGATGGGATGAGCAGTTTTCCATACTTTTTTGGAGAGGAATCAAGGAAAGG

AGGCATGACTATTTTAGCTGGTTAAGAATCACACAAGTTTTATCGACTGCAATCATCTTA

GGATTACTCTGGTGGCAATCCGATGCTAAGAACCCAAAAGATCTGCAAGATCAGGCAGGA

CTGCTTTTCTTTATTGCGGTGTTCTGGGGATTCTTTCCAGTTTTTACAGCAATATTTACA

TTTCCTCAAGAAAGAGCCATGTTAAATAAGGAACGCGCAGCAGATATGTACAGATTAAGC

GCATACTTCCTTGCTAGAACAACGAGCGACCTTCCATTAGACCTGATATTACCAGTGCTT

TTTCTCCTTGTTGTTTACTTCATGGCTGGTCTAAGACTAAGTGCTGCTCCCTTTTTCCTC

ACCATTCTTACTGTTTTCCTCTGCATCATTGCAGCTCAGGGTCTTGGACTTGCTATCGGT

GCTACGCTTATGGACTTGAAAAGAGCAACAACTTTGGCTTCGGTAACGGTGATGACCTTC

ATGCTGGCTGGAGGATTTTTCGTCCAGAAAGTCCCAATATTCATTTCTTGGATCCGCTAC

ATGTCTTTCAACTACCACACTTACAAACTATTGCTCAAAGTGCAATACGAACATCTCACA

CCTATTGTAAATGGAGTCAAAATTGACGGTGGTTTGACAGAAGTAGTTGCTTTGATTGCC

ATGGTCTTCGGTTACCGTTTCTTGGCATATCTTTCGTTGCGAAGAATGAAACTTCAATAG

>MS.gene034655.t1

ATGTATATTGCAGCTGAGGATATTGAAGATGGATCTAGGACAAAGTTTCAAACAGACCCT

ACTCTTCCTATATATCTAAAGTTCACTGATGTGACCTACAAGGTAGAACTCAAAGGCATG

ACATCAAGTAAGAAGAAAGATATCTTGAATGGCATAAGTGGTTCTGTAAATCCAAGTGAA

GTTTTGGCAATGATGGGTCCCTCAGGAAGTGGTAAGACAACTCTTTTAAAACTCCTTGGA

GGAAGGCTAAATCATCATCCCAAAATGGTTGGCGGTTCTATCACTTACAATGACCAAACA

TATTCCAAGTTTCTTAAGAGCAGGATAGGGTTTGTGACACAAGATGATGTTTTATTTACT

CACCTAACTGTGAAAGAGACATTGACATACGCAGCACGTTTAAGACTACCGAAGACATTC

ACTAAGGAACAAAAGGAAAAAAGAGCTTTAGAGGTCATCTATGAGCTTGGCTTGGAGAGG

TGCCAAGATACTATGGTTGGCGATTCGTTTGTTCCCGGATTATCCGGTGGAGAGAGGAAG

AGAGTTTGTATTGGCAATGAGATCTTAATCAATCCTTCCATTCTGTTTCTTGATGAACCA

ACATCTGGTTTGGATTCAACAACAGCCTTAAAGATTGTTCAGATGTTGCAAGACATGGCA

AAGGCTGGAAAAACAGTAGTGACAACAATTCACCAACCATCAAGTAGACTCTTCCACAAA

TTTGACAAGTTGGTCCTTCTTGGGAAAGGGAGTTTGCTTTACTTTGGAAAAGCATCAGAG

GCAATGAATTACTTTCAATCTATAGGATGTTCACCGCTTATTTCTATGAATCCAGCGGAG

TTTTTGCTAGACCTTGCCAATGGAAACATTAATGATGTTTCCGTACCATCGGAGTTGAAG

GATAAATTTCAACTTGGGAATGAAGCACCAGCACTAGTCCATGAGTATCTGGTAGAGGCA

TGTGAATCTCGACTTGCAGAAACAGAGAAGAAAAAAATAATTGTTTCCGTGCCACAAGAT

GAAGCTTTAAAGACCAAAGTATGTTCATCACCTAAAAGACAATGGGGAGCAAGTTGGGAT

GAACAATTTTCCATACTATTGTGTAGAGGAATCAAGGAAAGGAGGCATGACTATTTTAGC

TGGTTGAGAATCACACAAGTTTTATCCACTGCAATCATCTTAGGATCACTCTGGTGGCAA

TCAGATGCTGAAAACCCTACCGATCTGCAAGATCAGGCAGGACTACTTTTCTTTATTACT

GTGTTCTGGGGACTTATTCCAGTTTTTACAGCAATATTTACATTTCCTCAAGAGAGAGCA

ATGTTAAATAAGGAACGTGCAGCAGATATGTACAGATTAAGCGCATACTTCCTTGCTAGA

ACAACGAGCGACCTTCCATTAGACCTGATATTATCAGTGCTTTTTCTACTTGTTGTTTAC

TTCATGGCTGGTCTGAGACTAAGTGCTGCTTCCTTTTTTCTCACCATTCTTACTGTTTTC

CTCTGTATCATTGCAGCTCAGGGTCTTGGACTTGCTATCGGTGCTGTGCTTATGGACTCG

AAAAGAGCAACAACTTTGGGTTCGGTAACCGTGATGATCTTCATGCTAGCTGGAGGATTT

TTCGTCCAGAAAGTCCCAATATTCATTTCTTGGATCCGCTACATGTCTTTCAACTACCAC

ACTTACAAACTGTTGCTCAAGGTGCAATATGAACATCTCACACCTATCGTAAATGGAGTC

AAAATTGACAGTGGTTTAACAGAAGTAGCTGCTCTGATTGCCATGGTTTTCAGTTACCGT

TTTATGGCATATCTTTCGTTGCGGCGAATGAAACTTCAATAG

>MS.gene034654.t1

ATGTTCACTGAAGGTCTCGATGAAAACGCACATGTAGATGATGGTAGAGGGTTGGGATTA

TCGCCACCGTCAAAATTCAAAACTGGTCATCTTCCTGTTAACAAGTTAGATGTTTCCGCA

GTTGAGACATTTGATAGCGGTTCAAATAGTGACATAGACGCCAACGTTGATTCGGAGGAG

GAAGTTTATGGTGGGATGTATTCTTTGGATTCATCACCACAAGACAGTAGAGTACCCAAC

GAAACACTTGCTGGAAGACATGGAATGGCGAGGGATCCGGTGATGAGGGGTGCTGCTAAT

GGAAGGCTGAATGCGTACCGCGGTAGTCAGTGGCAACACCACGGACCACCGCACCGGCAT

GTTTCGCCGGACGTGAGGAATCTTGGGGGGGATAGGGATCCAAGCAGGGTGCCACAAAGG

GGTGGGAATGAGGGTAATTGGATCGACAGGTATGCAAGACGTGATCAGCAGGTCCAACAG

AGGCAGTCACGACGTGATTATATACGTCCAGTAGTGAAAGGAATGATGTCCGTGAATAGC

AGAAGCGCGTTTTGGTTTCAGCTGGAAACCAGAGAGTGTGCGACATTAAATCAGATGGCA

ACGGAAAGGCTTCAGACTTATCACCTAAACGATGGGGCGTCTATGCCGCTTATTCAGAAT

AGGGTGGAAGACGCGGGTTTTAAGGACATTGATATAATACCAATTGGGGCTGACAAAGTT

TTTGTTCATTCACTTTCGGGAGCGGATGTTTCTGGTATTTTGAAAGAGGCAAAACAATTT

TTTGATTTAATTTTCTCTAATATGGTGGCTTGCAAGAAAGAGGTTTTACCGTTTCAAAGG

GGAGCTTGGATTCGGTTGTATGGAATTCCATTACACGCGTGGAATCAAGGCTTTTTTAAA

TTATGTGTTCATGAGTGCGGAAGGTTTTTAAAAGCAGACAATTGTTCTTTGAATAGAGAA

AGGTTCGATTATGCAAGAGTATTGATATCTACGTCGAATTTGAATGTGGTTAATTGTTCC

GAACAAATTCTTGTAGATGATTCGATGGTGGAGATAAAAATCATAGAAGAATGGGGCTTC

AACTTAGGTGATGACGCTTGTTTATATGATGAGGAGGATAAAGCGGTGTCCGAACAGCCA

GACAATGAGTACGTGCATGAGGATTTCCAAAATATTGATAATATTGACAAAATAGCTGAT

AAACTTGTGCAGGACTTAGTGGAAACGGAAGACTTGGAATGTGATGACTTGAATGTAAAC

GGTAAGGCAAATCCAGTGGCTACAAACTCCATTAAGGTGCTCGACAATTTGCATCCTGAT

GGCAGTTTGGACAGTCCAGCGGTTGCTCCACAGAAAAATGAAGAACCTATTATGTCCATG

TCTTCTGTTTGTAAGGACGGATCCAAGGAGGACACAGTCAAACAACTCCCGGTGGTGGCA

GATATGAATGATGCCGTGTCAGGTGTGGGGGGTACAACACCTTCAGAAGCGACACAAGGT

TCAGCTGAAGAAGTGGTATCAAACCGAAAGAAAAGACAAAATAATCACATTTCATGTTCA

GCAGGTTCATTGGGTAACTCAGGTCTGTGGAGTATGGATTGGCTGCAAAACGTCAACAAA

GGAGGTACATGTCTTGTTTCTTCAAAAAACAAGCGGTTAAAAAAGGTGCGGAAGGACAAT

GGAGGCAGTGGTGGGAGATCACTCAATAATGTTGTTACAAAAAAGGCGGGTGGCTTTCTT

CGACATCCAGTTCTCACATTAAAGAAGGTGGCAAGACTTCCAAGAAAGGATAGAGCGGAG

GTTATGAAGGTATTGCGGGATTCAAAAATTATGAAATCCTTGAAACAAAAAATAAGTAAT

CGACGTCGGCACAGACAGAGAAACGCAAGATCGCTTGACGCAGTGAGTCTTAATTCTCAA

AATCTATCTTCATCTATGGTTTCTGTGAGTAATGATTGGAGGCACTGGGTAGCTTTAAAT

GGCAATGATGTGTCGAAGGCAGCATATATTGAGTCTTTTGGTAAAGCTATAGGGGTTTCT

TATAAAGAAAGTTGTCACAACAAGTTTAGTGTGCTAAACCGGTATAAAAAGGTTGAGTTA

GGGCCAGTGTTGACACCTATGGTGGTGGGGCGTGTTGAGGAGGATGTCGGAGTGTAG

>MS.gene034656.t1

ATGCAGTTCACTGATGTGACCTACAAGGTAGAACTCAAAGGCATGACATCAAGTAAGAAG

AAAGATATCTTGAATGGCATAAGTGGTTCTGTAAATCCAAGTGAAGTTTTGGCAATGATG

GGTCCCTCAGGAAGTGGTAAGACAACTCTTTTAAAACTCCTTGGAGGAAGGCTAAATCAT

CATCCCAAAATGGTTGGCGGTTCTATCACTTACAATGACCAAACATATTCCAAGTTTCTT

AAGAGCAGGATAGGGTTTGTGACACAAGATGATGTTTTATTTACTCACCTAACTGTGAAA

GAGACATTGACATACGCAGCACGTTTAAGACTACCGAAGACATTCACTAAGGAACAAAAG

GAAAAAAGAGCTTTAGAGGTCATCTATGAGCTTGGCTTGGAGAGGTGCCAAGATACTATG

GTTGGCGATTCGTTTGTTCCCGGATTATCCGGTGGAGAGAGGAAGAGAGTTTGTATTGGC

AATGAGATCTTAATCAATCCTTCCATTCTGTTTCTTGATGAACCAACATCTGGTTTGGAT

TCAACAACAGCCTTAAAGATTGTTCAGATGTTGCAAGACATGGCAAAGGCTGGAAAAACA

GTAGTGACAACAATTCACCAACCATCAAGTAGACTCTTCCACAAATTTGACAAGTTGGTC

CTTCTTGGGAAAGGGAGTTTGCTTTACTTTGGAAAAGCATCAGAGGCAATGAATTACTTT

CAATCTATAGGATGTTCACCGCTTATTTCTATGAATCCAGCGGAGTTTTTGCTAGACCTT

GCCAATGGAAACATTAATGATGTTTCCGTACCATCGGAGTTGAAGGATAAATTTCAACTT

GGGAATGAAGCACCAGCACTAGTCCATGAGTATCTGGTAGAGGCATGTGAATCTCGACTT

GCAGAAACAGAGAAGAAAAAAATAATTGTTTCCGTGCCACAAGATGAAGCTTTAAAGACC

AAAGTATGTTCATCACCTAAAAGACAATGGGGAGCAAGTTGGGATGAACAATTTTCCATA

CTATTGTGTAGAGGAATCAAGGAAAGGAGGCATGACTATTTTAGCTGGTTGAGAATCACA

CAAGTTTTATCCACTGCAATCATCTTAGGATCACTCTGGTGGCAATCAGATGCTGAAAAC

CCTACCGATCTGCAAGATCAGGCAGGACTACTTTTCTTTATTACTGTGTTCTGGGGACTT

ATTCCAGTTTTTACAGCAATATTTACATTTCCTCAAGAGAGAGCAATGTTAAATAAGGAA

CGTGCAGCAGATATGTACAGATTAAGCGCATACTTCCTTGCTAGAACAACGAGCGACCTT

CCATTAGACCTGATATTATCAGTGCTTTTTCTACTTGTTGTTTACTTCATGGCTGGTCTG

AGACTAAGTGCTGCTTCCTTTTTTCTCACCATTCTTACTGTTTTCCTCTGTATCATTGCA

GCTCAGGGTCTTGGACTTGCTATCGGTGCTGTGCTTATGGACTCGAAAAGAGCAACAACT

TTGGGTTCGGTAACCGTGATGATCTTCATGCTAGCTGGAGGATTTTTCGTCCAGAAAGTC

CCAATATTCATTTCTTGGATCCGCTACATGTCTTTCAACTACCACACTTACAAACTGTTG

CTCAAGGTGCAATATGAACATCTCACACCTATCGTAAATGGAGTCAAAATTGACAGTGGT

TTAACAGAAGTAGCTGCTCTGATTGCCATGGTTTTCAGTTACCGTTTTATGGCATATCTT

TCGTTGCGGCGAATGAAACTTCAATAG

>MS.gene034657.t1

GGTAATCTCAATTTGATAACACAAGCATTGGAAGCAGTTGGTTGTCGGATGCAGGTAATA

CCTGATCCGACAACCGTTCATTTCCATCTACCAAACCACCTCTCAGTTCGAGTGCACAGA

GAATATGATAAGTTCATTGAAGAACTGACGAGTTACTTTCCCCACGAAAAGGACGGCATC

CTCAAATTCTACGGTGAATGCTGGAAGATTTTCAATGCCCTGAATTCGTTGGAGCTGAAA

TCGCTTGAGGAGCCACTCTACCTTTTTGGGCAGTTTTTTCAGAAGCCTCTTGAATGCTTG

ACATTAGCCTATTATTTGCCTCAAAATGCTGGAGCCATAGCCCGGAAGTATATTCAGGAT

CCACAGTTGTTGTCTTTCATAGATGCTGAGTGTTTTATAGTGAGCACCGTCAACGCTTTG

CAGACTCCAATGATTAATGCTAGCATGGTTCTGTGTGACAGACACTTTGGTGGGATTAAC

TACCCTCTTGGGGGTGTTGGTGGGATTGCAAAGTCCTTGGCAAAAGGTCTAGTTGATCAG

GGTAGTCAGATACTTTACAAGGCAAATGTCACGAGTATTATCACCGAGCAGGGCAAAGCC

GTAGGAGTGAGGCTTTCAGACGGCAGAGAGTTTTTTGCCAAAACCATAATATCAAATGCT

ACCAGATGGGACACATTTGGAAAGCTTGTAAAAGGAGAATCACTACCAAAAGAGGAAGAG

AACTTCCAAAAAGTTTATGTTAAAGCTCCATCTTTTCTTTCAATACACATGGGGGTTAAA

GCAGAGGTTTTACCACCAGATACAGATTGTCATCATTTTGTGCTGGAGAACAATTGGACC

AGTTTGGAGGAACCATATGGAAGTATCTTTTTGAGTATACCGACTATACTTGATTCATCA

TTAGCTCCTGAAGGTCGTCATATCCTTCATATATTCACAACTTCTTCAATGGACGATTGG

CAGGGTCTCTCAAGAACCGAATATGAGGCAAAGAAGCAGGTGGTAGCAGATGAAATCCTA

AGCAGATTAGAGAAGAAATTGTTTCCAGGTCTTCGAGCATCCATCGATTTTATAGAGGTA

GGGACACCTAAGACACATCGACGATACTTAGCCCGTGACGAGGGTACTTATGGACCAATG

CCACGCAGCATTCCAAAGGGGTTGTTAGGAATGCCGTTTAACACAACAAGCATAGATGGT

CTTTACTGTGTTGGAGACAGTTGCTTCCCTGGACAAGGTGTTATTGCTGTAGCTTTCTCA

GGAGTTATGTGTGCTCATCGAGTAGCTGCAGATATTGGGTTGGAGAAAAAGTCACCTGTT

TTGGATGGCATGCTGCTTGGGTTGCTTGGTTGGTTAAGAACACTGGCATGA

>MS.gene034658.t1

ATGCAGTTCACTGATGTGACCTACAAGGTAGAACTCAAAGGCATGACATCAAGTAAGAAG

AAAGATATCTTGAATGGCATAAGTGGTTCTGTAAATCCAAGTGAAGTTTTGGCAATGATG

GGTCCCTCAGGAAGTGGTAAGACAACTCTTTTAAAACTCCTTGGAGGAAGGCTAAATCAT

CATCCCAAAATGGTTGGCGGTTCTATCACTTACAATGACCAAACATATTCCAAGTTTCTT

AAGAGCAGGATAGGGTTTGTGACACAAGATGATGTTTTATTTACTCACCTAACTGTGAAA

GAGACATTGACATACGCAGCACGTTTAAGACTACCGAAGACATTCACTAAGGAACAAAAG

GAAAAAAGAGCTTTAGAGGTCATCTATGAGCTTGGCTTGGAGAGGTGCCAAGATACTATG

GTTGGCGATTCGTTTGTTCCCGGATTATCCGGTGGAGAGAGGAAGAGAGTTTGTATTGGC

AATGAGATCTTAATCAATCCTTCCATTCTGTTTCTTGATGAACCAACATCTGGTTTGGAT

TCAACAACAGCCTTAAAGATTGTTCAGATGTTGCAAGACATGGCAAAGGCTGGAAAAACA

GTAGTGACAACAATTCACCAACCATCAAGTAGACTCTTCCACAAATTTGACAAGTTGGTC

CTTCTTGGGAAAGGGAGTTTGCTTTACTTTGGAAAAGCATCAGAGGCAATGAATTACTTT

CAATCTATAGGATGTTCACCGCTTATTTCTATGAATCCAGCGGAGTTTTTGCTAGACCTT

GCCAATGGAAACATTAATGATGTTTCCGTACCATCGGAGTTGAAGGATAAATTTCAACTT

GGGAATGAAGCACCAGCACTAGTCCATGAGTATCTGGTAGAGGCATGTGAATCTCGACTT

GCAGAAACAGAGAAGAAAAAAATAATTGTTTCCGTGCCACAAGATGAAGCTTTAAAGACC

AAAGTATGTTCATCACCTAAAAGACAATGGGGAGCAAGTTGGGATGAACAATTTTCCATA

CTATTGTGTAGAGGAATCAAGGAAAGGAGGCATGACTATTTTAGCTGGTTGAGAATCACA

CAAGTTTTATCCACTGCAATCATCTTAGGATCACTCTGGTGGCAATCAGATGCTGAAAAC

CCTACCGATCTGCAAGATCAGGCAGGACTACTTTTCTTTATTACTGTGTTCTGGGGACTT

ATTCCAGTTTTTACAGCAATATTTACATTTCCTCAAGAGAGAGCAATGTTAAATAAGGAA

CGTGCAGCAGATATGTACAGATTAAGCGCATACTTCCTTGCTAGAACAACGAGCGACCTT

CCATTAGACCTGATATTATCAGTGCTTTTTCTACTTGTTGTTTACTTCATGGCTGGTCTG

AGACTAAGTGCTGCTTCCTTTTTTCTCACCATTCTTACTGTTTTCCTCTGTATCATTGCA

GCTCAGGGTCTTGGACTTGCTATCGGTGCTGTGCTTATGGACTCGAAAAGAGCAACAACT

TTGGGTTCGGTAACCGTGATGATCTTCATGCTAGCTGGAGGATTTTTCGTCCAGAAAGTC

CCAATATTCATTTCTTGGATCCGCTACATGTCTTTCAACTACCACACTTACAAACTGTTG

CTCAAGGTGCAATATGAACATCTCACACCTATCGTAAATGGAGTCAAAATTGACAGTGGT

TTAACAGAAGTAGCTGCTCTGATTGCCATGGTTTTCAGTTACCGTTTTATGGCATATCTT

TCGTTGCGGCGAATGAAACTTCAATAG

>MS.gene034659.t1

ATGCTCAATTCCCCTATACTCTACGTATCTAACCACACCCCCATCATCTACCATCACACC

CCACCTACAAAGTTTCAATCTTTACCTTCATGTAGCTATGGAAGAGCTGAATTTTCATAT

GGGTCATGCTCAAATGGTGGTAGAAGAAAAAGGGTGGTGGTGAAAGTGAAAGGGATGATG

AAAGTGGATGAAGAAGTGGTTGAAAGTGGGAAGAGAGGGGATTTTGGTGAAGGGTTTGAT

GCAATTGTTATTGGTTCTGGGATTGGTGGGTTGGTTGCTGGGACTCAGTTGGCTGTGAAA

GGTGCTAGAGTTTTGGTTTTGGAGAAGTATGTTATACCTGGTGGGAGTTCTGGCTTTTAT

CATAGGGAGGGGTATACTTTTGATGTTGGTTCTTCTGTTATGTTTGGTTTCAGTGATAAG

GGTAATCTCAATTTGATAACACAAGCATTGGAAGCAGTTGGTTGTCGGATGCAGGTAATA

CCTGATCCGACAACCGTTCATTTCCATCTACCAAACCACCTCTCAGTTCGAGTGCACAGA

GAATATGATAAGTTCATTGAAGAACTGACGAGTTACTTTCCCCACGAAAAGGACGGCATC

CTCAAATTCTACGGTGAATGCTGGAAGATTTTCAATGCCCTGAATTCGTTGGAGCTGAAA

TCGCTTGAGGAGCCACTCTACCTTTTTGGGCAGTTTTTTCAGAAGCCTCTTGAATGCTTG

ACATTAGCCTATTATTTGCCTCAAAATGCTGGAGCCATAGCCCGGAAGTATATTCAGGAT

CCACAGTTGTTGTCTTTCATAGATGCTGAGTGTTTTATAGTGAGCACCGTCAACGCTTTG

CAGACTCCAATGATTAATGCTAGCATGGTTCTGTGTGACAGACACTTTGGTGGGATTAAC

TACCCTCTTGGGGGTGTTGGTGGGATTGCAAAGTCCTTGGCAAAAGGTCTAGTTGATCAG

GGTAGTCAGATACTTTACAAGGCAAATGTCACGAGTATTATCACCGAGCAGGGCAAAGCC

GTAGGAGTGAGGCTTTCAGACGGCAGAGAGTTTTTTGCCAAAACCATAATATCAAATGCT

ACCAGATGGGACACATTTGGAAAGCTTGTAAAAGGAGAATCACTACCAAAAGAGGAAGAG

AACTTCCAAAAAGTTTATGTTAAAGCTCCATCTTTTCTTTCAATACACATGGGGGTTAAA

GCAGAGGTTTTACCACCAGATACAGATTGTCATCATTTTGTGCTGGAGAACAATTGGACC

AGTTTGGAGGAACCATATGGAAGTATCTTTTTGAGTATACCGACTATACTTGATTCATCA

TTAGCTCCTGAAGGTCGTCATATCCTTCATATATTCACAACTTCTTCAATGGACGATTGG

CAGGGTCTCTCAAGAACCGAATATGAGGCAAAGAAGCAGGTGGTAGCAGATGAAATCCTA

AGCAGATTAGAGAAGAAATTGTTTCCAGGTCTTCGAGCATCCATCGATTTTATAGAGGTA

GGGACACCTAAGACACATCGACGATACTTAGCCCGTGACGAGGGTACTTATGGACCAATG

CCACGCAGCATTCCAAAGGGGTTGTTAGGAATGCCGTTTAACACAACAAGCATAGATGGT

CTTTACTGTGTTGGAGACAGTTGCTTCCCTGGACAAGGTGTTATTGCTGTAGCTTTCTCA

GGAGTTATGTGTGCTCATCGAGTAGCTGCAGATATTGGGTTGGAGAAAAAGTCACCTGTT

TTGGATGGCATGCTGCTTGGGTTGCTTGGTTGGTTAAGAACACTGGCATGA

>MS.gene034661.t1

ATGGTGATTCATTTTCGGTTTATAAAAGCCATAGTAACAACTGAAGAGGGTTTCTTGCTT

GATCCTTCATGTCATGAGGTTATTCCATTAGTTCAAAAATTACAGCAACAACTTCAACCC

TCCGAGGTTCCGTTTGAGTTTCAAGTGTTGGGGTTTGCTCTAGAAGTTGTTTCTAATTAT

TTGGATTCTAATGTTGCTGACCTTGAAAGAGCTACTTATCTTGTATTGGAGGAATTGGCT

AGGAATGATAGTATCGATAATCACGAACGTGTTATAATCTTAAAGAGTAATCTTACTGAT

TTGCGTGAATTTGTGGAGGAG

>MS.gene034822.t1

ATGATTTGCATTTCCGGCGAGTCAGAGGCGGAGGAAACAACCACAATCGCCACCGTCCAC

TCCGACATAATCCAAACACACATACTCACCCGCCTCGACGGTCCTGCTCTTGCTTCTGCC

GCAAGCACCTGCTCCCAATTCAACTCTCTTTGTTCCAATGAACATCTATGGGCCAGCATG

TGCCACTCAACTTGGCCTTCCACCAACAGTGAACGTGTTCAGCAAGTAATCTCAAACTTC

CCCAATGGCTTCCGCTCTTTCTTTGCAGACTCTTTTTCCTCTCATCATCAAAGGGATATG

ACTTTAACAAATCATGACGAAACCCTAAGTTTAATCTCAGCCGTTGATATTTTCCACCGT

GAGGGTTTAGTGTTTTCTAAAGTAGTTGAAACTGAGACAGTAACTGGGTGGTTCCGGTGC

TCTCCGTTTCGTGTTGACCTACTTGATACAAAGGATGTGATAAAATATCCAGTTGTTGGT

GATGAAAATAACGTCACATGTCATGATCTTGGGGAGGAGCTGAGACTGAGCTGGATTTTG

ATTGATCCGGCAAGTCATCGGGCGGTGAACGTGTCAAGCGGGAAGGCGGTGGCTGTGCAG

CGGCACTGGCTGAGTGGAGAGGTGAAGGCGCAGTTCGCAACGGTGGTTTATGGAGAAACA

GCCACGGCGTTGGAGGTGGCGTTGTGTAGTGTAGTGGTGATGTTGGCTGAAGAGAGTATG

GAAGTTAGAGAG

>MS.gene034823.t1

GCTGCCGTGTACTCCTTATTATATGCTGCAAGTGAAATTTCATCCCGAGATGATAGCAGA

GATAGAAATGTCAATGTATTTGTTCAAAGGAGTTTGCTAAGGCTATCTGCTCCCTTGGAG

AGCTTAATCAGAGAAAACTTATCTGCCAAACAGCCTGAAGTGTACGAATGGTTTTGGTCT

GAGCAAGTTCCAGCTGTAGTGACGTCCTTTGTTAATAAGTTTGAAGGGGACGGGCGCTTC

ACTTCTGCCATTGCTTTGTCTGAAAAAACTAAGGGTTTGAGCAGTGCAAGTGATGTATCA

CTTCTCCTGCTTGCACTTACATGCATTGCTGCAATTGCTAAACTTGGCCCGGCAAAAGTT

TCTTGCTCACAATTCTTTTCCATGAGCACTGAGATAACTGGTAGTTTGATGGACATGCTG

GTTGGTTTAATTCCTGTAAGTCAAGCTTATAATTCTATAAAGGATGTTGGTCTGCACAGA

GAATTTCTTGTACATTTTGGTCCTCGAGCTGCAGCTTTTAGAGCAAACGTTGAGTGGGGT

TCAGAAGAGGTTATTTTCTGGGTAAATCTGGTTCAGAAGCAGTTGCAGCAGGCTATTGAT

AAGGAGAAAATATGGTCAAGACTGACAACATCTGAAAGCATTGAGGTTTTGGAGAAGGAT

TTGGCTATATTTGGATTCTTTATTGCTTTAGGAAGAAGTACACGAACATTTCTTTTGGCA

AATGGTTTCGATGGTCCCGATGATCCAATTGAAGATTTCATCAGGTATCTTATTGGGGGA

AGTGTTTTATACTACCCACAACTCTCATCCATTAGTTCATATCAATTGTATGTTGAGGTA

GTTTGTGAAGAGCTGGATTGGCTTCCTTTTTATCCGGGAATCACCAGCATCACAAAACAG

TCTCATGGGCATAGTAAACCAGAAGGTCCTCCAAATGCAGAAGCAGTGACCCAAGCATTT

GCTGTTTGCTCTTATTGGATGCAGAGCTTTATTAAATACAGCACATGGCTTGAGAGTCCT

TCAAATGTAAAAGCAGCTGAATTTCTGTCCAGAGGGCACAACAAGTTGATGGAGTGCATG

GATGAACTTGGGATGATAAAAGATAAGACATTGGAGACTGATGCCAAGAAAATAGTTGAC

GGGCAAAGATCCACAATTCAGTCAACGATAAAAGACTCGGGTTCTTTTGATGAGGCATTA

AAAAGTGTCGAAGAAGCTGTGATAAAGCTTGAAAAGTTGCTTCAAGAATTGCATGTGTCA

AGCTCTAGTTCTGGAAAGGAGCATTTGAAAGCAGCCTGTTCTGACTTGGAAAAAATACGG

AAACTTAAGAAAGAAGCTGAATTCCTGGAGGCATCTTTCCGAGCAAAAGCTGATTCTCTG

CAAGAGGGAGTTAATAGTGATCAAACCATCACACCGGTTGGTGAGGAGGACAGGTTTATG

AAAGGGAAAAGCAGAAAGAACGACAATGTAAAGGTGGACAGGAGCAAAAGACAAATTGGA

AAATCTCGTGGATTCTGGAGCATATTTGTACCTCCTATACCCAGAAAGCCCGACCCGGAA

CCTGATGTGGATGCTCAT

>MS.gene034825.t1

CAGCTCCCAATAATTCACGAAAATCACTACCAATTGAAGCATGTGATTTTTCCAAAGGTC

GATGGGTTTGGGACGAAACTTATTATTCACATAAGTTGTATGATGAAAATTGTCCCTTTC

TTGATCCCGGATTCCGGTGCCGGCAAAATGGAAGGAACGACGAAGGTTATCGTAAATGGA

GATGGCAACCTAATGATTGTGACCTTCCAAGATTCAATGCCAGTGACCTCCTAGAGAGGA

GTCGCAATGGACGCATTGTGTTTGCAGGTGATTCAGTGGGCAGAAACCAATGGGAGTCAT

TACTTTGCATGTTGTCAAAAGGGGTCTCTAACCTCTCAAAAATATATGAAGTTAATGGAA

GTCCCATAAGCAAACACAAGGGCTACTTAGTGATGAAATTTGAGGATTACAACATGACTG

TAGAATATTATAGGGCACCTTTCTTATCTATTATAGGTCATCCACCACAAAACTCATCTA

GTGACATTAAAATGACTATCAGACTTGATGAGTTACATTGGTATTCTAATAACTGGAAGG

GAGCAAATGTTCTTGTTTTCAATAATGGTCATTGGTGGAACTTAGACAAAACTATCAAGT

CGGGTATCTATTTCCAGGAAGGTGGGAAGGTAAATGTGACTATGACAGTGAAAGAAGCTT

TTAGGAGATCCTTACAGACATGGAAATCATGGGCATTGAGTAATCTAAATCCTATGAGGA

GTTTCGTCTTCTTTCGTAGCTATGCCCCTGTTCATTACAGGAATGGCACATGGAATGAAG

GAGGAAACTGTGACAAAGACAAGGAACCAGAAAACGATCCTGCAAAACTCGAAGCTGATC

CGTATTACAACGTATTTATATCTGATGTTGTAAAAGAGATGCAATATGGAAGCTGGAAAG

TCAACTTTTTGAACATCACATATCTTTCGGAATTGAGGAAAGACGGTCACCCTTCCAAAT

ATCGGGAACCAGGAACCCCACCCGATGCTCCTCAGGATTGTAGCCACTGGTGTTTACCCG

GAGTGCCAGACACGTGGAATGAACTTATCTATGCCCAACTCCTCTCTAAGAAATATGGCA

TCAACAAAAGTTTTCCAGAAAGCGAAGAACGCAGCTGA

>MS.gene034824.t1

GAGTGTAAATTTGCTTCCATTTGCTACCTCTGATGATGGTATGACTGTCAACGGGAGTCC

ACAAGCTGACGCCAGTGCTAACCTTGAAAAAATGAGGGTGAAATTGAATAGCTCTCTTGA

AGATGAAGACTTTTATGATGGACTTGTTCAAGCTTTATATGATGCAGCCAGGGTTTTTGA

GCTAGCAATTAAAGACCATAAATCGTCATCACGTGGATCCTGGTTTTCAACAGCTTGGCT

TGGGGTAGACCAAACCGCATGGGTGAAGGCATTGTCATGTCAGGCTGCCGTGTACTCCTT

ATTATATGCTGCAAGTGAAATTTCATCCCGAGATGATAGCAGAGATAGAAATGTCAATGT

ATTTGTTCAAAGGAGTTTGCTAAGGCTATCTGCTCCCTTGGAGAGCTTAATCAGAGAAAA

CTTATCTGCCAAACAGCCTGAAGTGTACGAATGGTTTTGGTCTGAGCAAGTTCCAGCTGT

AGTGACGTCCTTTGTTAATAAGTTTGAAGGGGACGGGCGCTTCACTTCTGCCATTGCTTT

GTCTGAAAAAACTAAGGGTTTGAGCAGTGCAAGTGATGTATCACTTCTCCTGCTTGCACT

TACATGCATTGCTGCAATTGCTAAACTTGGCCCGGCAAAAGTTTCTTGCTCACAATTCTT

TTCCATGAGCACTGAGATAACTGGTAGTTTGATGGACATGCTGGTTGGTTTAATTCCTGT

AAGTCAAGCTTATAATTCTATAAAGGATGTTGGTCTGCACAGAGAATTTCTTGTACATTT

TGGTCCTCGAGCTGCAGCTTTTAGAGCAAACGTTGAGTGGGGTTCAGAAGAGGTTATTTT

CTGGGTAAATCTGGTTCAGAAGCAGTTGCAGCAGGCTATTGATAAGGAGAAAATATGGTC

AAGACTGACAACATCTGAAAGCATTGAGGTTTTGGAGAAGGATTTGGCTATATTTGGATT

CTTTATTGCTTTAGGAAGAAGTACACGAACATTTCTTTTGGCAAATGGTTTCGATGGTCC

CGATGATCCAATTGAAGATTTCATCAGGTATCTTATTGGGGGAAGTGTTTTATACTACCC

ACAACTCTCATCCATTAGTTCATATCAATTGTATGTTGAGGTAGTTTGTGAAGAGCTGGA

TTGGCTTCCTTTTTATCCGGGAATCACCAGCATCACAAAACAGTCTCATGGGCATAGTAA

ACCAGAAGGTCCTCCAAATGCAGAAGCAGTGACCCAAGCATTTGCTGTTTGCTCTTATTG

GATGCAGAGCTTTATTAAATACAGCACATGGCTTGAGAGTCCTTCAAATGTAAAAGCAGC

TGAATTTCTGTCCAGAGGGCACAACAAGTTGATGGAGTGCATGGATGAACTTGGGATGAT

AAAAGATAAGACATTGGAGACTGATGCCAAGAAAATAGTTGACGGGCAAAGATCCACAAT

TCAGTCAACGATAAAAGACTCGGGTTCTTTTGATGAGGCATTAAAAAGTGTCGAAGAAGC

TGTGATAAAGCTTGAAAAGTTGCTTCAAGAATTGCATGTGTCAAGCTCTAGTTCTGGAAA

GGAGCATTTGAAAGCAGCCTGTTCTGACTTGGAAAAAATACGGAAACTTAAGAAAGAAGC

TGAATTCCTGGAGGCATCTTTCCGAGCAAAAGCTGATTCTCTGCAAGAGGGAGTTAATAG

TGATCAAACCATCACACCGGTTGGTGAGGAGGACAGGTTTATGAAAGGGAAAAGCAGAAA

GAACGACAATGTAAAGGTGGACAGGAGCAAAAGACAAATTGGAAAATCTCGTGGATTCTG

GAGCATATTTGTACCTCCTATACCCAGAAAGCCCGACCCGGAACCTGATGTGGATGCTCA

TGAAAATTATATTGAACAGCCTGCACCAAATGTAGGGGTTGTGGACCAAGAACCCAATGA

AATCCTTCGCTTTGAGCTTCTAAGAAATGAGCTGATGGAACTTGAGAAAAGGGTCCAAAG

AAGTGCCTATCAGTCAGAAAATAATGAGGATTTGATGATCAGTGATGATGGTGCCCGTTA

TAACGGTGATGCTGGAGGTGTTCAGATGGTCAGAGTTCAGAAGAATGAAAATTTCATACA

GAAATCTTTTGACAAACTAAAAGAAACGGGAACGGATGTCTTGCAAGGAACTCAACTTCT

TGCTATTGATGTTGGTGCTGCCTCGGGTTTGGTCAAAAGGACCTTGATAGGTGATGAATT

GACTGAGAAGGAGAAGAAAGCACTTAAAAGAACCTTAACTGACATGGCTTCAGTTGTTCC

TATCGGCATTTTAATGCTTCTTCCTGTTACTGCCGTTGGGCATGCAGCAATGTTGGCTGC

CATTCAGAGATATGTACCATCCCTGATTCCATCCACTTATGCACCAGAAAGGTTGGATCT

CTTGAGGCAGCTTGAGAAAGTGAAACAAATGTCAATGTCAACCAGTGATGTAGATTCAGA

TGATGAAATGGATGAAGTTAAATGA

>MS.gene034827.t1

ATGTTATTAGTCGAACCAACTTCTTGGTCTCTCCATCACCACCAACCTCCATGGTTCAAT

TCTCAAATCCATGAAAACTCACCTCATTACAACCTCAAAAACATCGAAGATGAAGAATTA

GACACAACCACCACCGTAATAAAACCCGAAGACGAAAAGGAATCAATGTTTGAGAAACCC

TTAACACCTTCTGACGTAGGAAAACTCAACCGTCTTGTTATCCCAAAACAATACGCTGAA

AAATACTTTCCACTCGGCGAGCAGACTCCGCCGAATGAGTCAGGGAAGTGTTGGCGTTTC

CGTTACTCATATTGGAACAGTAGTCAAAGCTATGTTCTAACAAAAGGTTGGAGTCGTTAT

GTTAAAGACAAACGACTCGATGCTGGCGATGTCGTTCTGTTTGAACGACATCGTGTTGAT

AGTCAGAGACTTTTTATCAACTGGAGGAGGAGGAGTGGGGATGATCAGTCGGCGTATGTT

AGCAGAGGGGTGGGACATCATGGTAAGGGTAGTGATGGGAATGGTAAGAATGAGGTTGTT

AATGGTGTAGGGTGGTCCAGAGGGTTCTATTCTGCGCATGTTCCTTATCCTGCGCATCAT

CATCAAGTCTTGCCATACCAACATGATCAATGTCTTCATGCAG

>MS.gene034831.t1

ATGGTGTATTGGTCGTACCCGCCGACGGGGAGGGAAATTGCGGTAACCGCCGCCGTGTTT

ACGATCGGTGCTTCGTTGTTCGGTGTCGGAGCTTATCTTTCGTTTGTTAACGTTGCTCCA

CAGCAGGCTCGTGCGAAAGAACGCAGTGAAGCAATGAGGAATTATCTCAGCAAGCGCTTC

GGTGATTGA

>MS.gene034829.t1

ATGCTATATGCAACCTTCAAGAAGATTGGAGGATTGGAGCTAATGGCATGCTTCATGGAT

GTTGGTCTTGAAGACAGTACTTCGACAGAGCCTCTAGGGATGGTGAGATACATGATGATC

AACATTGATGTGTTTGATTTTGAAATTGATGTTATAGTGACAAATGACAACAAAGGTCAA

GACGTACTGCCTACTGATTCTGGAAAGATCATTCATGGAAACTACTAA

>MS.gene034828.t1

ATGGAGGAAGCATGCGGTGAAGGACGCAAAGGAACACATGGCAGGGGTAGAGCGTCTCCA

CAGTCGTATTTCTCCTCTCATCCCTTGGTCGTCGTCGACTCGACCGCCGCCACCGTCAGC

GTTGAATTTTCTCCTGCGGCCTCCTCTCTAAGTCTCTCTGTCCCGCTGCCTCTTCCGCTT

CCCGTTCATTCAACACCAACACCATGCCCCTGTACGATGAACACTCTGATGAGTCCAATG

TTGCCGATCGCTCTCCCGCACTCTCCGCGACGATTTCTCTCAGTTAGCCTGATCCTTGCA

CAGCTTGGAAAGATTCAGGCTTCTGTCAAGAAGCCCTTGGTGCCTAAGATCAAAGCAACG

GTGGAAAATGGATGTGCTTATGAGGTTGAGAATGCATTGGTAGCTCACAATGACCCTAAA

TACCAATGCACTAGACACCATTTCAAGCTCAATCTTATTGATAAAACTAAGTTTACCAAG

GTTGAAGCTTCTAACATACCTGTGAATCATTTCGATTTTGTAGCCTTTAGAAACATTTTG

GAAGCGGACAGGGAGGATAAACATCTAGTAATTTATACAACACTTCATTTTAACGTTGAT

GATCAGCTTGGTAAGATACAAGCCTCTGCAAAAAAGATATTGGTACCCAAGTTCAAAGCT

GTTGTTCAGGAAGGATCTATTGTGGTCCCTTTGGCGGATGATATGCTAAAATCAAAGAAG

ATGACCATCAAATATTTGATTGAATCCACTGATGATTTGTTGCCACCCAATGAAGTTCAA

ATTTTTGGCCATGCAAGGTCATATATATCTGATGATTATGAATTCAGAAACAAATCGCAT

AGATCTGAACCACCACAAACCAAATCCAACCGCTTCACAATGGAGGAAGCATGCGGTGAA

GGACGCAAAGGAACACATGGCAGGGGTAGAGCGTCTCCACAGTCGTATTTCTCCTCTCAT

CCCTTGGTCGTCGTCGACTCGACCGCCGCCACCGTCAGCGTTGAATTTTCTCCTGCGGCC

TCCTCTCTAAGTCTCTCTGTCCCGCTGCCTCTTCCGCTTCCCGTTCATTCAACACCAACA

CCATGCCCCTGTACGATGAACACTCTGATGAGTCCAATGTTGCCGATCGCTCTCCCGCAC

TCTCCGCGACGATTTCTCTCAGTTAGCCTGATCCTTGCACAGCTTGGAAAGATTCAGGCT

TCTGTCAAGAAGCCCTTGGTGCCTAAGATCAAAGCAACGGTGGAAAATGGATGTGCTTAT

GAGGTTGAGAATGCATTGGTAGCTCACAATGACCCTAAATACCAATGCACTAGACACCAT

TTCAAGCTCAATCTTATTGATAAAACTAAGTTTACCAAGGTTGAAGCTTCTAACATACCT

GTGAATCATTTCGATTTTGTAGCCTTTAGAAACATTTTGGAAGCGGACAGGGAGGATAAA

CATCTAGGAAGCCTAAGTATTGTCATAATCATTGCTTCTGTTAAACATGGTGCATTGAAA

GAGATGACTGATAACAATCCTATGGATGTTATTGAAGGGGATGATTCAAACCGCAAAGAT

CTTATTGAGCAGCTTTAG

>MS.gene034830.t1

ATGAAGAATCAACAAATCCATTCATTTTACAAAAGGAAGGCCGATGATATTGTAAGAGAT

GAAGAAGTTATAATTTCTTCATCTGAACCTGAACAAGTTTGTGAGAATCGAAGAATTGAA

GAAGATGAGCCTCGTCCTTCAAAGGTTAATAGAGTTAACCCGGAAGACATTGAGAATTCT

TTAGAACGTGATCCTGGAAAGCGTATTCCAATTTGGCAATATCCACCGAATCAAAAGGAT

GCAATACGAAGAGCGTATCTAAAATGGGGTCCTTATCAAATGAAATTAGAAAACTTTCCC

TTGTCCGGTGAGCATAAACATCCAAGGCGGTTTCAAGACACTTGGTTCAGCTTGTTTCCT

TCATGGCTAGAATATTCACCATCGGAAGATGCTGCATATTGCTTACAATGTTATCTCTTT

AGCAAAAAACCAAGCGGACGTTACGGATCACAAGTATTCATTTCTACGGGTTTTAAAGCT

TGGAAGAATGTTAGGAATGGAAAACAATGTTCCTTTCTTAAACACATAGGGAAGGATCCT

TGCTCACCACATAACAATGCAATGAAAGATTGTCAAGACTTTTTGAATCAAGATATGCAT

CTTAGGAATGTTATTGAAGTGCAAAGTTCGAGCCAAATTCTGAATAATAGACTATGTTTG

AAGGCTTCAATTGACACTGTTCGTTGGTTAACACTTCAAGCTTGTGCTTTTAGGGGTCAC

TATGAAGGAAGTGAATCAAGAAATCAAGCATCTTACAATGATGAAGTTGCAAAAGTTGTG

TTGAAAAATGCTCCCGAAAATTGCAAGTATACTTCACATCAAATTCAAAAAGAGCTCTTG

CAAATTCTTTCTAGTAGGGTGAGAAAACATATTCGTGAAGAAATTGGTGATTCTAAATTT

TGCATTGTTGTTGATGAAGCTCGTGATGAGTCAAAAAAGGAACAAATGGCTCTTGTGTTA

AGATTTGTTGATAAAGCTGGTTTGATACAAGAGAGATTTTTTGATGTGGCACGTGTTAAT

GACACTTCTTCGTTAACTCTTAAGGAAGCAGTGTGTGGTATACTTTCTCGACATAACCTT

GATGTTTCTAATATTCGTGGCCAAGGGTATGACGGTGCTAGCAATATGAGAGGAGAATGG

AATGGTTTACAAGCACTTTTTATGAAAGATTGTCCTTATGCCTACTATGTCCATTGTTTT

GCTCATCGATTGCAACTTGCCTTAGTTACTGCATCAAGAGAAGTTACATCAATTCATCAA

TTCTTTGAGAAGCTGGCTTTTGTTGTCAATGTTGTTGGTTCTTCTACTAAACGCCATGAT

GAGCTACAAGCTGCCCAATCAAAAGAAATTGAAAATTTGTTAGAGAATGGGGAGATTGTA

ACTGGTAAAGGTAAAAATCAAGTTGGTACCGTGAAAAGAGCCGGAGATACTCGTTGGAGT

TCACATTTCAACTCTATTTGTAGCTTGATAAGTATGTATGAAGCAACTTGTGTAGTTTTG

AAAAAAATTGCAAAAGAAGCAAAAAAATTTGCACAACGTGCGGATGCTGATAGTTCTTAC

AATTACTTGAAGTCTTTTGATTTTATTTTTATCTTGCACTTGATGAAAGAAATTATGGGG

ATAACTGATGTGCTTTGTCAAGCCTTGCAAAAACAATCTCAGGATGTTGTTAATGCTATG

CTTTTGGTTTGTTCAACAAAAGCTCTTATTCAAGAATTGAGAGAAAATGGTTGGGATAAG

TTGTTTGCTAGTGTGGTATCTTTTTGTGAAAAACATGATATTGAGATTCCCGACCTCAAT

GATTGTCATTCAGCTTCAAGATTTGGGCGCTCTCGCCTTGAAGAGAATCAGTGA

>MS.gene034832.t1

ATGGGTCGTGTCAGAACCAAAACCGTGAAAAAATCCTCACGCCAAGTAATCGAACGTTAC

TACTCAAAAATGACCCTAGATTTCCACACCAACAAAAAAATCCTTGAAGAAGTTGCATTG

ATTCCATCAAAGCGTCTTCGCAACAAGATCGCTGGATTTTCAACCCATTTGATGAAGAGG

ATTCAAAAGGGACCGGTTCGTGGAATCTCGTTGAAGCTACAAGAGGAAGAGCGTGAGAGG

AGGATGGATTTCGTTCCTGATGTTTCTGCTATCAGAACTGACCATATTGAGGTTGATAAG

GAGACTCTTGATATGCTTGCTGCTCTTGGTATGTCCGAGATTCCCGGGGTTGTTCAGGCT

GAACCTGTTCCTGTTCAGCAG

>MS.gene034927.t1

GTGAAGTTCCTCAAGATGGAGACCAGGTTGAGATTCATTACTATGGTAGACTTGCAGCAA

AACAAGGATGGCGTTTTGATTCAACCTATGACCACAAAGATGAGAATGGTGATCCTAATC

CTTTTGTCTTTGTCCTTGGTTCTGGTAAGGTAATTGCTGGAATTGATGTGGCAGTGAGAT

CAATGAAAGTCGGTGGTATTCGCAGAGTCATCATACCTCCCTCACTTGGGTATCAGAACA

CATCACAGGAACCTATCCCGCCTAATGTAAGAAATCCTTTTATGATCCAGAATTACATAG

TTACGTTTGATTAA

>MS.gene034925.t1

ATGAAGAAGCTTGTGGAGAAGCAAGAGGAGTGGTTGAGTAAGCTTGTGAAGACACTTGAG

GACAAGGAAAAGGAGAGGGCGTTAAGAGAAGAAGAGTGGAGGAAACAAGAGGCCAAAAGG

GTGGAAAAAGAACAAAAGTTTTGGAGTAATGAGAGAGCATGGATTGAAGCAAGGGATGCT

GCTTTAATGGAAGCTTTGAAGAATTTAGGAGGTAGAGAAATGATTAAGGTTGAAGAAGCT

CATGATCATAAGGGTGTAAATGGAATAACTGGAGCAGAGATTCAAAATCAAAATGAAGAT

GGGAGTGAAATCTTGAATAGTACTGTGAGAGGTTCTGATAATTGTACTCTAAGGGATGAT

TCTAAGGAAGGAAACAGGAAGAGAAAGGAGAGTTTTTACTTCAACAATAATGAACAATGT

TCTATGTACAATCAAGGAAGTGGTTATTGTGATGTTAATGATCAAAGAGAGGAGATTGTG

AAACTACAAAGGAATGATCATGGTTCTTCACCTTCTGATTCTAATGTTGGAAATGTAGCT

AATGGTGATACTTGCTTTCCTTTCATGATTAGTGAGGGTGGAAATTTGTGGGAGAATTTT

GGCTTGAAGATCAATAAAGTAAACCAAAACCATTAA

>MS.gene034926.t1

ATGTCTTCTTTCAAGAGCAAATATGAAGATGAACTCATTGCCAATGCTGCTTATATTGGC

TCTCCCGGAAAGGGTATCCTTGCTGCTGATGAATCTACTGGCACAATTGGAAAGCGATTC

GCTAGCATCAATGTGGAGAATGTTGAAGCCAACAGACGTACTCTTCGCGAACTCTTGTTC

ACTGCCCCTGGTTGTTTAGACTGCCTCAGTGGTGTTATCTTGTTCGAAGAAACACTATAT

CAAAAAACCGCTGCAGGAGTACCCTTTGTGGATGTGTTGAAGAAAGCTGGAGTTCTTCCG

GGTATCAAGGTTGACAAGGGAACCGTTGAACTTGCCGGCACAAATGGAGAAACCACAACT

CAAGGTTTGGATGACCTTGGTCAGAGGTGCAAGAAGTACTATGAAGCCGGTGCAAGGTTC

GCTAAATGGCGTGCCGTGCTAAAGATCGGTCCAAATGAACCATCTCCCTTGTCAATCCAC

GAAAACGCCTACGGTTTAGCTCGTTATGCCGTCATATGCCAAGAGAATGGTTTGGTTCCA

ATTGTAGAGCCTGAGATTCTAGTGGATGGATCTCATGACATCGCAAAATGCGCTGATGTA

ACCGAACGTGTTCTTGCAGCATGTTACAAGGCTCTAAGTGACCATCATGTTCTTCTTGAA

GGTACTCTTTTGAAGCCTAATATGGTTACACCAGGATCAGACTCTCCTAAAGTTGCTCCT

GAGGTTATAGCCCAACACACAGTCCGAGCTTTGTTGCGAACCGTTCCTGCTGCGGTTCCT

GCTATAGTTTTCTTGTCTGGTGGACAAAGTGAAGAGGAAGCAACTATTAATCTTAATGCA

ATGAACAAACTTAAGGGAAAGAAGCCTTGGACTCTTACATTCTCTTATGGAAGGGCACTT

CAGCAAAGTACTCTTAAGGCATGGGGTGGAAAAGATGAAAACATTCCTAAAGCTCAAGCT

GCATTGCTTGTTAGGTGTAAAGCAAATTCAGAAGCAACTCTAGGAACTTATCAAGGTAAT

GCTAACCTTGGTGATGGTGCTTCAGAGTCTCTTCATGTTAAGGACTACAAATACTAA

>MS.gene034931.t1

ATGGCCCCTCACACCGCACAGTTTCAGAACCGCCACAATCAAGGTGGAGCACACTCACAC

AGTAGTTCAACTGCCTTCTCAGAATTCAGAACCGCCACAACCCATAAGGGCAAACTCGCC

CTCAAGAAACGTGCTCCAAAACTCGTTGAAACTGGGAAGAAAACGTTGTTGCTTCATGGA

ACCAAAACAAGTGCGGTTTTGAATGCTGTGTTGACTCAGATTTATCATTTGAAGAAGGAA

AGTGCGATTAAGTATAGTCGTAAGAATGATAATATTAAACCTTTTGAAGCTGGTGGTGAA

ACCTCTTTGGAGTTTTTCGCACAAAAAACTGACTGCAGTCTCTTTCTGTATGGATCTCAC

TCAAAGAAGAGACCTAATAACCTTGTTCTCGGGAGAATGTATGATTTCCATGTCTATGAT

CTGATAGAAGTTGGTATTGAAAATTTTAGAGGCATGGAGTCATTTGATTATGATAAGAAA

ATAGCTCCAAAGGAAGGATCAAAGCCTTTTATGGCTTTCATTGGGGAAGGATTTGAGTCT

GTGAACGAACTTAAACAGCTGAAGGAAGTTTTACTTGATCTTTTTCGCGGAGAGGTTGTG

GAGAATTTAAATCTTGCCGGAGTGGACCGTGCATATGTATGTACTGCCCTGTCTCCAACT

AAGGTGTTTTTCACACACTGTGCATTGCGGTTGAAAAAATCTGGCACAGTTGTCCCAAGA

ATGGAATTGGTAGAAGTTGGTCCTTCCATGGATATGGTTATTCGCCGGCATCGTCCTCCT

AATGAAAGTGTGATGAAGGAAGCCATGAAAACTTCAAGGGACAAGCCAAAGAAAAAGGAG

AAGAATGTCAAAAAGGATCCCCTACAAGGAAAGATTGGAAATGTTTATATTCCAGATCAG

AAGATTGGAGAAATGGTTTTGCCTAATAAATCGAAAGGAGTGAAGAGGGAGCGCCGAGAA

GCCAAGCAGCAGAAACATGGGAGTGATGAGCATGCATCGAAAAAGCAGAAGGATGAATCT

TCCTAA

>MS.gene034933.t1

ATGGAAATGCCTGCTAGAAGATCTAATTACTCGATTCTAAGTCAAGTTCCCGACGATCAG

TTCTCCTACGCCGCCGGCGCTAGTGCTCCGGCACCGTCGTCGTCGAGCGACGGAAAAGCT

AGTAGAGGCGGTTGGGAGTTAGTCGGCGATCATCGGCAAGGGAACAATCGGATCGGGAAT

TTGAATTTGTATTCATCGATCGGATTACAGCGGCAGTCGAGTGGGAGTAGTTACGGCGAA

AGTTCGTTAGCCAGCGGCGGTGATTATTATGCGCAGACGCTGTCGGCGGCGGCTGCTAGT

GAGGTTGATGCGTTTGGTTATTTGCATGAGGTTGGTGAGGTGAGGGGGAAGTTGGCGGAA

AGTGCAGCGTGGAACGGGGGATCGTCTGGGAAGAGCTGGGCGCAGCAGACAGAGGAGAGT

TATCAGTTGCAGCTTGCGCTGGCGTTGAGGCTTTCGTCGGATGCTACTTGTGCTGATGAT

CCGAATTTTCTGGATCCGGTGCCGGATGACGCGGCTTCGAGACTTTCGGCTTCGGCGGAG

GCTGTTTCGCATCGATTTTGGGTGAATGGCTGCCTATCATACTCCGACAAAATTCCTGAT

GGCTTTTATCTGATTCATGGGATGGATTCCTATGTCTGGACTGTGTGCACTGATCTGCAG

GAAAACAGCCGAATCCCATCAGTTGATACTCTAAAGTCTGTACATCCCCGCATTGATTCT

TCCCTTGAAGTAGTTTTGGTGGATCGGCGCAGTGATGCCAGCTTGAGAGAACTGCAAAAC

ATAGTTCATAACATCTCTTGTAGCAGCATAACAACAACAGAGGTTGTGGATCAGCTTTCC

AAGCTGGTTTGCAACTGTATGGGGGGTTCAGCTTCTGCTGGGGAGGATGAGTTCCTATCT

TTCTGGAGGGACTGCAGCAATAATCTGAAAGATTGCTTAGGATCTGTTGTTTTTCCCATA

GGTAGTCTATCTGTTGGCCTCTGCAGGCATCGTGCCATATTATTCAAAGTGCTAGCTGAT

GCTATTGATTTACCATGTCGAATTGCAAAGGGCTGTAAATATTGTAAAAGGGATGATGCT

TCTTCTTGTCTTGTTCGATTTGGGCTTGATAGGGAATATCTTGTTGATTTAATTGGGAAG

CCAGGACACTTATCTGAGCCTGATTCCTTGCTCAATGGTCCATCTTCCATCTCATTTTGT

TCACCCTTGCGCTTTCCAAGACTTAAACCAGCTGAAACTACAATTGATTTCAGGTCACTT

GCCAAGCAGTATTTCTCAGACTGTCTGTCTCTTGAGCTTTTCTTTGACAACAGTTCAGCA

GAACAGCTGGATGGGAAATGCAATGAAAGTAATAACCTTATGCCAATTTCAAGTGACATC

AACATAAATTCTCACCTTCCTTTGTATCCGCAAGCTCCTCTTCCAAGCACACTTGATCAA

GCTTCTGAAACATATATGTTGTGTAACCCTCCTCAGAACATTATAGAATCAACAACTGTG

GGCAAGTATCCACCACCTTTAAAGCATAAGCGTCCTGTTGCTATTTCCAATGATGATGTC

ATTGAGGGTAGGAGGTTTATTGAAGGAAGTCAACTAATTCCTAGCAAGCCTACTCGAGAG

ATTACCTTTGACATGGAGGATTTGGCCATACCATGGAGTGATCTTGTTTTAAAAGAAAAA

ATCGGATCAGGTTCTTTTGGAACTGTACATCGTGCTGAGTGGAACGGCTCGGATGTTGCT

GTTAAAATTTTGATGGAGCAAGATTTTCATGCAGAACGCTTCAAGGAATTCATGAGGGAG

GTTGCAATAATGAAACATTTGCGGCATCCAAACATTGTTTTATTAATGGGGGCAGTCACT

CAACCTCCTAACTTATCAATTGTCACCGAGTATTTATCAAGGGGTAGCTTATATCGGCTG

TTGCATAGACCTGGTGCCAAGGAAGTGTTGGATGAGAGGCGCAGGCTTAGTATGGCTTAC

GATGTGGCAAAAGGGATGAATTATCTTCACAAGCGTAATCCCCCCATTGTTCATAGAGAC

CTGAAATCACCAAACCTTCTTGTTGACAAGAAATACACAGTGAAGGTTTGTGATTTTGGG

CTTTCCCGCTTAAAGGCCAACACATTTCTCTCTTCCAAGTCAGCTGCTGGGACCCCTGAG

TGGATGGCTCCAGAAGTTCTTCGTGATGAGCCATCAAATGAGAAGTCTGATATTTATAGC

TTTGGGGTAATCATGTGGGAGATTGCGACATTGCAGCAGCCATGGGGTAATTTGAATCCA

GCACAGGTTGTCGCTGCCGTTGGCTTCAAGCATAAAAGGCTTGAGATCCCACGCAATTTG

AATCCACAAATAGCTGCAATAATTGAGGCTTGCTGGGCAAATGAGCCCTGGAAACGTCCT

TCTTTTGCCAGCATTATGGATTCTCTAAGGTCATTGCTCAAACCCCCTACACCTCAACCT

GGTCTTCCAAGCACACCATGTCTCACTTGA

>MS.gene034928.t1

ATGGAATCCATCAATGTGGTGGTAGATGATATGGTTAAAGAACCAATGCTTGAAGTTGTA

CCAGGTGTTAAAGCATATCCTGAAACATCTGCTCAAGAGAAAGTAAGGCCTGAAACTGAT

AAAGAACCTGAGACTAACTCTAAAGATACTGAACTGGAGAATGCAGAATCAAGCAAAGGA

CCTTCAATCAGAGTTCAGAAGAATCATCATCAAGAGCTCATTATTGGAGATCCAGATCAA

GGAATCAGAACCAGGAGATCTAATGATGTTGTCTCTAACTCCTGGGGAGTAAAAGATATC

TGTTTGTGCTACTTGAGGGGGAGTAAACTTATGTGTCCCTGA

>MS.gene034930.t1

ATGGAGAATTCACGAAGACCGTTTGATAGATCAAAAGAACCAATTTTGAAGAGACCCAGA

TTGATCAAAGATCCGGATCATGCTCCGAATTCGAGTGCTCGACCAATTCAACAAAGAAAA

CAAGTGAATTCTGGGGTAGCAACGTTGTCTTCTGCCTCACGGTTCAGACAGAATGATAGA

GACTTGGAAAGCAGTGATTTTGGTGGTGGTTATGAGCCGCAGCCGATTCGCTTTGAGGAC

CTGGTGGCTCAGTATAAGAGTGCACTTGCAGAGCTTACTTTCAATTCCAAACCGATGATT

ACCAACTTGACTATAATTGCAGGGGAGAATCAAGCTGGTCAGAAGGCTATTGCTGCTACT

ATTTGTGCTAACATTCTAGAGGTTCCTAGTGAACAAAAGCTGCCATCTCTTTATCTCTTA

GACAGTATTGTTAAGAATATTGGGCGGGATTACATAAAATACTTTGCCGCCAAACTACCC

GAGGTATTCTGCAAGGCATACAAACAGGTTGATCCTTCTGTCCGTCAAAGTATGAAACAT

CTTTTTGGAACTTGGAAAGGAGTCTTTCCTCCTCAGACCCTTCATGTTATTGACAAGGAA

CTGGGCTTCACTCCAGCAATCAATGGTTCGTCTTCAGCATCTACTGCACTCAGGAGTGAT

TCACAGTCACAGCATTCGCCGCACAGTATTCATGTGAATCCCAAGTATTTTGAAAGACAT

CGTCTTCAGCAGTCCAGCAGGACTGAAGGAGTTGTTGGTGACATGACTGGAGCTATTGCA

AACTCAAATGACGACCATGAGATGACTGATAAAGATTTGGGTGTTGCACGAGCATGGGTG

GGTTGTAGTGTTTCTAATAATCAGCGCACTCATAGAGATCCATTCGATGATTCAGTTCCA

GAAAAGAGCATCAGTGCATCCTATGAAGACAATGAACATGGTTCCAATCTTTCAAGGAAT

TTGGGCATGGGCATTGGAAGAACTGATGGTAAAGTGGCCAAGTTAGGACACAGAAATTTG

TACGGCAAAGCTGCTGCTGGTGTTTCAGGGACCATATCTGGGCAAAGAAATAGTGTTGGC

CTCAAGCATAGTTTTTCAAATACTGAAGCATCTATGCATCAGCCAACACGAAACATAACT

GGCATACAGAGAAATGTGATATCAAGTAGCTGGAAAAATTCTGAGGAAGAGGAGTACTCG

TGGGATGAAATGAACGCGGGATTGACTGGTCATGGTGTCCGTAACAACTTGGGCAATGAT

GCTTGGACCGCTGATGATGAGAATTTGGAAGCTGAAGATAACCACCACCAAATCAGAAAT

GTTTTTGGGGCAAATGTTGATAGAGAAATGCCCAATAGATCTCAAGCCACTGAAAAGAAA

GAATTACATGCATTTCAGCATCATCCATCATTATCATGGCAATTGCAGGATCAGCAATCA

ATTGATGAGTTGGATAGGAAACCGGGTCACTTGGATGGATCTATGTTAATGTCTGGTTCT

TTACCAGCCAATGCAAGTTCTTCTGCTGTCAGGATGGGGAATCGTGCTTTCCTTCCAAAT

GCAAGAATAGGATTGGCAGAAATTGAGGGACAACAATTTCATTCTGTGGGATCTGAATCC

CCTTCTGGGCAGTCACCTTTGCAACATCGGTCTCCATCGCCACCAAGTATTGACCACTCC

CATCTTATGAAAACTTTGGCTATGCAAGACCATCCTCACACTCGTAAAACATCTAATTTT

TTGGGAGGTCCGCACAGCCAATATAATAAAGATTCTTCTCCCACCCTTTATCCTAATATT

CATGTTGGAGACTTGCGAAGATCATCACAACTAAAAGAATTGCATGGGCCATTACCTTCA

GCGGGTTTTCAGCCAAGGTATCAGCAACAACTAAGCTCTTCACACGCTGAAGGAACTGTT

AAGACTGAGAAGCCACCTTTGTCCAAAGTTTCTCTAGCCAGAAAAACCTCAGAGCAGCCA

GCCACAAGTCATACAAAAGATGCAAGTGTGAAGAATGGAGCATTTTCCAACATGCCTACT

ACTAGTAGTCTTCCATCTCTATTAGGGGCTCGGCCTTCTCAGTCAGGAAGTTTTTCCGCT

GCCAAAATAATTTCATCAGTGTCCGCAAATGTCTCTCCATCTTCACCTGCTTTGCAGAAA

AGACCTCAAAGAAAGGCTGGACAACCACTAAGGACATCTACTATATCACCAACTTCCTCG

AATGTTAGCAGCGCCTCAGCCCAGTCATCGGGTGCCACCAATCATACCTCAAATCCACTT

GCAAACCTTTTAAGCTCATTAGTTGCAAAGGGTTTGATATCGACAGTAACCGAGACACCA

GCCGAGGTGCCGCCTGAGATGCTGAGTCGATTGGAAGATCACTGTGACAGCTTTAGTACC

AGTAGCTCTATGCCTGTTGCTTCATTGTCTGGTTCTGCAGCTATTCCACTCCCTTCTACC

AAAGACGAGTTAGATGATACTGCGAAAACACCTATGTCGTTATCTGAATCAACCAGCACA

GATATTAGAAATGTCATTGGCTTTGAGTTTAAGCCTAATGTAATCAGAAAGTTGCATCCA

TCTGTAATTAGTGGATTATTTGATGATTTTCCTCATCATTGCAGCATTTGCGGCCTTAAA

CTTAAATTACAAGAACAGTTTAACAGACACTTGGAGTGGCATGCCACAAGAGAAAGAGAA

CGCACTGGTTTAATTACGGCATCAAGATGGTATCTGAAGTCAAGTGACTGGGTTGCTGGC

AAAGCTGAATGTCCATCTGAGAATGAGTTTACTGATTCTGTAGATTCACAAGACAGCGAA

CCAGACAAAAATCAAGAGGATGCAATGGTTCTAGCAGATGAAAACCAATGCTTGTGTGTG

CTGTGTGGTGAGCTATTTGAAGATGTTTACTGTCATGAAAATAGTGAATGGATGTTCAAA

GGGGCTGTTTACTTGACTAACGCTGATATCGATACTGATATGGGAATCAAAGATGTGATT

TCTGGAAGGGGTCCCATCATTCATACAAGATGCTTATCAGATTACTCGTTATCGAGTGTC

GTCAAGATGGAACAGGATTAA

>MS.gene034932.t1

ATGAATCCAAATGATGAAAAAAACTCTCCCAAATTTTCTTCTTCATCTTTGATGGAATAT

GAAGCATCAGCTATAGGTGGTGGTACTGGTAGTGATGTTCCTGTGCCTATGGAGTGTTTG

CAGATGAGCCCAGTTCCACCATTTCTTTCAAAAACATTTGATTTGGTTGATGACCCTTCT

TTGAATCCGATCATATCATGGAGTTCCAATGGTGTTAGCTTTGTTGTTTGGGATCCTTTG

GAGTTTGCAAGAGTTATCTTGCCTAGACACTTCAAACACAACAATTTCTCCAGTTTTGTT

CGTCAGCTTAATACTTATGGATTCCGCAAGATTGACACCGACAAATGGGAGTTTTTCAAT

GAAGGTTTTCAGAAAGGTAAGAAGCACTTGTTGAAAAGCATTCAAAGGCGCCGCACGTCT

CAATCTCAGCAAGTTGGTAACTATGTTGGATCTTCTTCTGATGCAGCAGGGAAATCTGGA

GTCGAGGTTGAGATTGAAAGATTGAGGAAGGAGAGGACTGTGTTAATGCAAGAAGTGGTT

GATTTACAGCAGCAACAACGAATGACAGCTAGTCACGCAGGTAATGTGAATCAAAGGCTT

CAATCTGCTGAACAAAGACAGAAACAAATGGTATCTTTCTTGGCCAAGTTGTTTTCAAAT

CCGGACTTTTTAGCGCGACTTAAGCAGAAGAAGGAACAGAAAGATATAGAATCTCCAAGA

GTTAGAAGAAAGTTTGTTAAGCAAAACCAAAATGAAGAAGAAAATGTCAAAGATGGAAAG

ATAGTGAGGTACCAACCAAATTGGGGGAATATAGACATGTCTTGTGAAACTCAAGAACTA

AGTCCAGTTTCCATTGAAAACTCTCCTCACTATCTTTCACTTGATCTACCTAGAGAAATG

ACTAAAGGTGAAGAAGATTTTACTTCGCAAATTGAGAATATTTCATCAGATGAATATGCT

GCAATGCATGGAATTTTCATGTCAAATTCAGAAACTAATATTATTGGCGAAGGATCATCT

AGTTTTGGACTTGATGAACCCCTTTTCAAAGGAAAAAATGTTATTAGTCCAAATGAAGAA

GTTATTCCTGAGAATTTTGAAGGGTTTCAATCTATTGGGACTGAAAGTATTATCAAGCAA

GAAGATATATGGGACCCTAACTTCAATGTTGGTGCTGTTGCTACTTCAAACTGTGGGAAT

GAGATGTGGGATCCTTTGAATTATGGAGTACCTGACTTTGGAGTTGTTACAAGTGGTGAC

ATGTCAGATATGTGGGATATTGGTTTTGGAAGTTTGGGTATTGATAATTGGCCAGCTGAT

GAATCTCCTTTTGATGAAATAGATAGTCAAGGTGGTAAGCATGATAGACCTACCTAA

>MS.gene034929.t1

ATGGCTCAATCATCAACTGAAGTGAAAGAAGATATGGAACAACTAATGGGATTAGAGGAA

TGTTATGAAAAATATGAAGAATGCTTTAGCAGAAGGATGAATATGGAGGCTCACTTGGAT

TACATCTACACCCAACACCATAAGCCTTGA

>MS.gene034934.t1

AAAGTACACAGTAAAGTTACAGCTTCATCTGTCACCAGTTATGTTACAAAGGTTCATGAA

TCATTCTTTGATAATGCATTCCAAAGATTGACCAAAAGGTCAATTGTGAAAATGGAGGTA

GGTGAAAATGATAGAAAGAAGCCAGAAGCAGAGATTCCATGGATCCCTTCAACCCCTGTT

AAGCCAGTTGTGCCAAAATCAGCTCCGATCTGTACTCCAAAAGCTAATGGAGCATTTGCA

TGTGTTGAATTCTCACATGGTGGAGAGAAAAACAGAGAATCCCATGATGGGATTGTACCT

GCGGCTGCAATTCCAGACATTGGAGGTGAAAATGGTAAAATCTGTGACAAGACAGGTTTT

GAGAATGTTTCATGTTGGAGTGGTCTGGGTTTTAATGAATCTTTGATTCCGACAGAGGCG

GCTTCGGCGAATTCTTGTGCAACACAGCTAGGAAACATTAATGGATTGAATGATCTGTTT

GTTTCTTCGGTTATCGGTGACAATTCGAGGGATCCTCATGGACATGAAACATCCGACAAT

GCTTGTTGTAGCAAGAGAAATTATGAGGATGACCCTGCCGGAAAGCTTGACAAGGATGAA

ACTCCACCAAACAAAGAGCTCTATGACCCTGCCGTAGAATTTGCTGCTGTTTCTTCACAA

CTGAAGGAGATTTACAACCCAGATGAGGGAAACAGCCTCTGTACTGGTCTGAATAAAACA

CCAGAAAAAAAGCCAAGGAGAAAAAAGCACCGCCCCAAGGTCATTAGAGAAGTCAAACCC

AAAAGAACTCCAAAGCCAGCTACCCCAAAGCCTGCTCAAGCAAAAGAAAACACAACCGGC

AAGAGGAAGTATGTGAGAAGAAAAGGATTGAACACTTCTACTACTCAAACAGAAGTGACA

AGTGAATTAGCTGAAAAAAAACCTGAAGCTGCCAAATTGTCATGTCAAAGGTCCTTAAAT

TTTGATATAGGAACAAAAGACGAGAGTTCTACAGGCAGAGAAAATGAAAATCCAACTGCA

CTTTTTGATGGTGTAGCCGTACAAGAAACCAATTTCGGCCTTGTCTGTGATTTAAACACT

TCAGTTAAGCATGCATCAAGCAGTTCCATGTCATTACCAGAAGATACACAAGCCCCAGAT

ACATCTTCACAAAGCACCAGCCCCGGGGCAAACTCAAAAGAAAACCCAACGGGCAAGAAG

AAACGTGTGAGAAGGAAAATGAACAAGATTTCTGCCCCTTCAACAGAAATGACAGGTGAA

TTGACTACAGAAAAGAGGTGTGAATTGGCCGAACCAACCAGTAAAAGTTCCATAAATTTT

GACAAAGGAGGAGTGGAAGAGAGTTCTGCAGTCAAAGAAAATGCAACTATACATCTGAGC

AAAGAAAATGAAGTCACTGATGGAACAAATCCAGACGTTCCATTAACAGAAGATACACAA

GCCACAAAATCATCTTCAAGAATGCCCCATGAAGCAAAGCCAAAGAAAAGCCCAAGTGTC

AAAAGGCAGTATGTGAGAAGGTCAGGATTGAACAAGTCTTCCACTCCTACAGAAGTATCC

GGAGACTTGCCTGGAAAAGTGATGCAAGAATCTGCCATAACTTCTTGTAGAATGTCCATA

AATTTTGATAGAGGAGCAAATGATGAAAGTTCTGCAGATAGAGAAAATGGAACTGTGCAC

CCGTGCAAAGAAACTGGAGCAGAAATACAGGAAATTAATGTAGGCCTTGACGATGATATC

CAGACTTTTATGAACCCGGCAGTAGAAAATAATTACCTGTCATTTTGTAATAATGAACAA

ACTCCAACTGTGCATCCGTGCAAAGAAACTGCTGCAGTAATGCAGAAAGTTGATGTAGGC

ATTTCCTATGATATGAAGACTTTCATGAAGCAGGCAACAGAAAATAATTACATGTCATTT

TGTAGTAATGAACAAAACTCAAGTACATCCCCGTCACAAACCAACCCCCTTGGGGATAAG

TCAAAAGAAAAGCTGACTGGAAACAAGTATGAGAGAAGAAAACGGTTGAACAAGTCTCCT

ATTTGTCAAACAAAAATGACAGGAGAACTGACTGGATCAATGATGCCAGATTCTAAAGAA

ACGCCAATGAGAAGGTTCTCGGATTTTGACATGGGAACCGAAGATGAAAGTTCTGCATGC

AGGCAAGTTTTAAATGTGCATATTGGCGACGCAGTAGAGGAAACACCTGCAGGCCTTGCC

TATAATAAAGATACCTGGATGAAGCAGGCATTACATAGCTACATGCCATTACCTGAAGAC

GCACAAGCCCCAAGTACATGTCCTTCAAAAGGTAATCATCCTGGAGCAAAGCCAAAAGAA

AACTCAGACGGCGAGAAGAAGTGTGTGAGAAAGAAGAGATCGAAAATGACTTCTACTCCA

ACAAAAAGGACAGGCGGATTGACTGAACCAATTATGTCAGAACCTACTACAATATCATGT

AAAATGTCCATAAACTTTGACAAAGGAGGAAGAGATGAAAGTTATATGTGCAACGAAAGC

CTGACCAGCGATCAAAACACCCTGGTGAAAGAGATATTACATTATTGCGCGTCATTATCT

GAAAACACACAACCTCCAAGTACATGTCTTCCCGAAAGCAATCCTCCCGAGGCAAAGCAG

AATGCCAGGAACGAAAACAAAAGAAAGGGCCTCGCAACTGTTGAGGATGGAAACATCAGT

AACAGTCAAGTTTCAACAATAAAGTTACAAATGGTTGGTTGCGAGAGAGAGCATGCTGAA

ACCATTGAACATGCAGATAACAGCAGCATGAATCTAATTGGCGCACACTATAATGGATTG

GCCTCATACCAGTCAAAGTTTCCGCTTCAATTTCCAAATATCCAGAAGAAAAGGAGAACT

GAGAAAGGGAAAACTTCTAATTCTCATATTACATCTTCTGTGATTACCGAAAATGGAGTA

CCACTAATATTCGCTCCCGAAGATGCTCAAATGCATCCTTATGCATCAAACAACAACTCT

TGGATGTATGATTTTGGATATAATGCACCTGTATTCCCAATCATAAATGAATCTAGAGAA

AATTATATCCATAATACTCAAACATTTGATGAGTTCAGATTATCTTTGAGAAGGGTGACA

GAAAGATCTCAATTCCCAGCTGAAACTTCTGATTATAATTCTCTAACGAGAATTAGAAAT

TTTATCGAACCAAATTATACAGCAAATCAACTGGACTTTTCAGACCAACAAACAATTAGA

GATGCAGAAAGACCTCAAACATGTATTGACGTCTTAGTTGAAGATATACCTGTATCATGT

GTAAAAAAGAAGCGAAATAGAAAGAGAAGTGCTCTTTCCAGTTCAGCGCCTCCCAACACA

GATCAGAATCAGATGCAACAGTACCATAATGTTGCCTTGGGAAATCACCACTTGGCACTA

GGAAAGTCATCAGATACTGCTCGCGGAGGAAGACAGAAGAAAGGGTATAATGTTGAAGCA

TTAGTTAAGCACTTTAGACGGCTAAACATAAACGCAGGAGTGGGAGACATTGTCTTATAC

AAGCAGAATGCACTTGTTCCCTTTCAGGGCTCATTTGATCCCATAAAAAAACAGCGTCCA

CGACCGAAAGTTGACCTAGATGAGGAGACTGATAGAGTGTGGAAGCTTTTGCTGTTAGAT

ATAAATCATGATGGGGTTGATGGAACAGATGAAGAAAAGGCCAAATGGTGGGAAGAAGAA

CGTAAAGTGTTCCGTGGACGAGCAGACTCATTTATTGCACGAATGCATCTTGTACAAGGA

GACAGACGATTTTCTCGATGGAAAGGATCAGTTGTGGATTCAGTTGTAGGAGTTTTCCTC

ACTCAAAATGTCACGGACCATCTTTCCAGCTCTGCGTTCATGTCCCTTGCTGCTCGGTTT

CCCAAAAAGTCAGGCAGCACAGCTTGTGACGGAGAAGGCACCAGCCAAGCGGTCAACAAA

CAACAAGTGGAGATAGTGGAACCGGAAGATAACACAGAATGTGATGTGAACTTATTGAAT

CAATCTGTTTGCAACCAGAGTTCTATGACAAAAGACATAATTGAGCATTCTGGAGAAAAA

GCTGTCAGCAGCAATGATTCCTGCAGAATTACCAGTAGCCCAATTAGCCTAACAGATGAA

TCAAACTGCAAACTAACAGAATCACCTCAATCCTCTGGTCCAATGGTTATGATTGAGGAA

GGGGAAGAAAAATCATGTTATGATGGTGCTGGGAAAGAGTTAAATGACATAGTTTCGTCC

CAATCCTCTGTCATTTCATCTCAAATATCTGGAGATTTTTCAAATGATCAAAATCCTGAG

AAGATAGGATCATGCTCAGATAGCAACTCAGAAGTTGAAGATCTGTCAAGCACAGCAAAG

TACAACAGTGTTGAAGATCTGTCAAGCACAGCAAAGTACAACAATTACGGTTCTTTCTGC

AAACTTCTTGAAATGGTAAGTTCAACCAAGTTTTATGAAATTGAAAGTCAAAGAAGCAAA

TCAACTGAGAACATGAGAGATGTCACTCACAGCTCTTTGGAAGAATCCATCATCCCGTCC

CATGAATGTAATTTGAGACTCACCCACAACTCTGAAGCTCATGATCCTTTCAAAGCAGAA

GCTTCATCAAGTGGCATCTTAAAGAATAAACATGAAAATGAGATGAATACACCTAGTTTT

CAAACAGCCAAGTCTGCAGGCCTTGTTGAGGTTACTCATTCTCAAACTATTGCATCTCAA

GTCCATCCTCAGGAACAGACTAATCACATGCAGCAAAACTTTTTCAACAGTTCTGAACAA

ACTCATGATCTGATTCGGAATGAAAGGGATTTAAACCTTGGTGATCACAAAGATGTTGTG

AGGAGTGAAACCAATGAGATAAGTTCTACCCCAATTAAAGTAAAGACCAAAAGTCAATTA

AAGGAGGAACAGGAACAATTTGACTGGGATAGTTTACGAATAAAAGCACAAGCTAAGGCT

GGGAAAAGAGAAAAGACAGAAAACACCATGGATTCTTTAGACTGGGATGCCGTGAGATGC

GCAGATGTCGGTGTAATTGCTGATGTTATCAAAGAGCGGGGCATGAACAACAGGCTTGCC

GAGCGTATTCAGAAATTCCTGAATAGGGTGGTTGATGATCATGGAAGCATTGACCTCGAG

TGGCTGAGAGATGTTCCACCTGACCAAGCAAAAGAATATTTGCTGAGCGTAAGAGGACTG

GGATTGAAAAGTGTGGAGTGTGTGCGGCTTTTAACACTGCACCATCTTGCCTTCCCGGTA

GACACAAATGTTGGACGTATAGCAGTACGCTTGGGATGGGTGCCTCTCCAGCCACTACCC

GAGTCACTACAGTTGCATCTCCTAGAAATGTACCCGGTGTTGGAGTCCATACAAAAATAT

CTTTGGCCTCGACTGTGCAAGCTAGATCAAAAAACATTATATGAGCTACATTACCAGATG

ATTACATTTGGGAAGGTCTTCTGTACAAAAAGCAAACCAAACTGTAATGCATGCCCGATG

AGAGCAGAATGTCGACACTTTGCAAGTGCATTTGCTAGTGCAAGGCTTGCCCTGCCTGGA

CCAGAGCAGAAGAGTATGGTTTGTGTATCTGGAAACGGTGTGACTGATGAGAACCCACCT

GTAGTCATGAGTCAGTTGCACTTGCCTCTTCCTGAGAACACAAACCAAGTGGTGGAAGAA

ATTCCCGAAACAGAAGTGAGCGGACAACTAGCAAAATCTGAAGTGAATATTTGCCAACCT

ATCATTGAAGAACCGACAACACCAGAGCCAGAATGTTCGCAGCTAGAACTAAGTGATATG

GAGGATGCCTTCATTGATGATCCATGTGAAATTCCTACCATTAAGCTTAACATGGAGGAG

TTCACTCTTAATTTACAAAACTATATGCAACAAAACATGGAACTTCAAGAAGGTGAAATG

TCAAAGGCTCTGGTTGCTTTGCATCCAGAAGCTGCTTCCATCCCTGGGCCCAAGCTAAAG

AATGTTAGCCGATTACGAACAGAGCATTGTGTTTATGAACTCCCGGATATGCATCCTCTT

CTGGAAGGGTGGGAAAAACGAGAACCCGATGATCCTGGCAAATATCTTCTTGCTATATGG

ACTCCAGGAGAGACGGCCGATTCAACACAGGCACCTGAGTGCAAATGCAACTCTCGCGAA

GAATGTGGACAGCTTTGCAATGAGATGGAATGTTTCTCATGCAACAGTTTCCGTGAAGCA

AATTCACAGATAGTCAGAGGCACACTTCTGATACCATGTCGAACTGCTATGCGTGGGAGC

TTTCCACTTAATGGCACCTATTTCCAAGTCAACGAGGTATTTGCTGACCATGGGTCTAGC

ATTAATCCTGTCAGTGTTCCCCGAAGTTGGATCTGGAACCTCAATAGGCGAACGGTATAT

TTTGGAACCTCAACAACATCAATATTTAAAGGTAAAGAATGCAGACCATGGATTATCAAC

ACAAGAAATTCAACAAGCCTTTTGGAGAGAAACGTGATGCATCACAGTGCCACTATAATG

TGCCACCTACCACCTATCGCTTAG

>MS.gene034935.t1

ATGGAGGTAGGTGAAAATGATAGAAAGAAGCCAGAAGCAGAGATTCCATGGATCCCTTCA

ACCCCTGTTAAGCCAGTTGTGCCAAAATCAGCTCCGATCTGTACTCCAAAAGCTAATGGA

GCATTTGCATGTGTTGAATTCTCACATGGTGGAGAGAAAAACAGAGAATCCCATGATGGG

ATTGTACCTGCGGCTGCAATTCCAGACATTGGAGGTGAAAATGAGGCGGCTTCGGCGAAT

TCTTGTGCAACACAGCTAGGAAACATTAATGGATTGAATGATCTGTTTGTTTCTTCGGTT

ATCGGTGACAATTCGAGGGATCCTCATGGACATGAAACATCCGACAATGCTTGTTGTAGC

AAGAGAAATTATGAGGATGACCCTGCCGGAAAGCTTGACAAGGATGAAACTCCACCAAAC

AAAGAGCTCTATGACCCTGCCGTAGAATTTGCTGCTGTTTCTTCACAACTGAAGGAGATT

TACAACCCAGATGAGGGAAACAGCCTCTGTACTGGTCTGAATAAAACACCAGAAAAAAAG

CCAAGGAGAAAAAAGCACCGCCCCAAGGTCATTAGAGAAGTCAAACCCAAAAGAACTCCA

AAGCCAGCTACCCCAAAGCCTGCTCAAGCAAAAGAAAACACAACCGGCAAGAGGAAGTAT

GTGAGAAGAAAAGGATTGAACACTTCTACTACTCAAACAGAAGTGACAAGTGAATTAGCT

GAAAAAAAACCTGAAGCTGCCAAATTGTCATGTCAAAGGTCCTTAAATTTTGATATAGGA

ACAAAAGACGAGAGTTCTACAGGCAGAGAAAATGAAAATCCAACTGCACTTTTTGATGGT

GTAGCCGTACAAGAAACCAATTTCGGCCTTGTCTGTGATTTAAACACTTCAGTTAAGCAT

GCATCAAGCAGTTCCATGTCATTACCAGAAGATACACAAGCCCCAGATACATCTTCACAA

AGCACCAGCCCCGGGGCAAACTCAAAAGAAAACCCAACGGGCAAGAAGAAACGTGTGAGA

AGGAAAATGAACAAGATTTCTGCCCCTTCAACAGAAATGACAGGTGAATTGACTACAGAA

AAGAGGTGTGAATTGGCCGAACCAACCAGTAAAAGTTCCATAAATTTTGACAAAGGAGGA

GTGGAAGAGAGTTCTGCAGTCAAAGAAAATGCAACTATACATCTGAGCAAAGAAAATGAA

GTCACTGATGGAACAAATCCAGACGTTCCATTAACAGAAGATACACAAGCCACAAAATCA

TCTTCAAGAATGCCCCATGAAGCAAAGCCAAAGAAAAGCCCAAGTGTCAAAAGGCAGTAT

GTGAGAAGGTCAGGATTGAACAAGTCTTCCACTCCTACAGAAGTATCCGGAGACTTGCCT

GGAAAAGTGATGCAAGAATCTGCCATAACTTCTTGTAGAATGTCCATAAATTTTGATAGA

GGAGCAAATGATGAAAGTTCTGCAGATAGAGAAAATGGAACTGTGCACCCGTGCAAAGAA

ACTGGAGCAGAAATACAGGAAATTAATGTAGGCCTTGACGATGATATCCAGACTTTTATG

AACCCGGCAGTAGAAAATAATTACCTGTCATTTTGTAATAATGAACAAACTCCAACTGTG

CATCCGTGCAAAGAAACTGCTGCAGTAATGCAGAAAGTTGATGTAGGCATTTCCTATGAT

ATGAAGACTTTCATGAAGCAGGCAACAGAAAATAATTACATGTCATTTTGTAGTAATGAA

CAAAACTCAAGTACATCCCCGTCACAAACCAACCCCCTTGGGGATAAGTCAAAAGAAAAG

CTGACTGGAAACAAGTATGAGAGAAGAAAACGGTTGAACAAGTCTCCTATTTGTCAAACA

AAAATGACAGGAGAACTGACTGGATCAATGATGCCAGATTCTAAAGAAACGCCAATGAGA

AGGTTCTCGGATTTTGACATGGGAACCGAAGATGAAAGTTCTGCATGCAGGCAAGTTTTA

AATGTGCATATTGGCGACGCAGTAGAGGAAACACCTGCAGGCCTTGCCTATAATAAAGAT

ACCTGGATGAAGCAGGCATTACATAGCTACATGCCATTACCTGAAGACGCACAAGCCCCA

AGTACATGTCCTTCAAAAGGTAATCATCCTGGAGCAAAGCCAAAAGAAAACTCAGACGGC

GAGAAGAAGTGTGTGAGAAAGAAGAGATCGAAAATGACTTCTACTCCAACAAAAAGGACA

GGCGGATTGACTGAACCAATTATGTCAGAACCTACTACAATATCATGTAAAATGTCCATA

AACTTTGACAAAGGAGGAAGAGATGAAAGTTATATGTGCAACGAAAGCCTGACCAGCGAT

CAAAACACCCTGGTGAAAGAGATATTACATTATTGCGCGTCATTATCTGAAAACACACAA

CCTCCAAGTACATGTCTTCCCGAAAGCAATCCTCCCGAGGCAAAGCAGAATGCCAGGAAC

GAAAACAAAAGAAAGGGCCTCGCAACTGTTGAGGATGGAAACATCAGTAACAGTCAAGTT

TCAACAATAAAGTTACAAATGGTTGGTTGCGAGAGAGAGCATGCTGAAACCATTGAACAT

GCAGATAACAGCAGCATGAATCTAATTGGCGCACACTATAATGGATTGGCCTCATACCAG

TCAAAGTTTCCGCTTCAATTTCCAAATATCCAGAAGAAAAGGAGAACTGAGAAAGGGAAA

ACTTCTAATTCTCATATTACATCTTCTGTGATTACCGAAAATGGAGTACCACTAATATTC

GCTCCCGAAGATGCTCAAATGCATCCTTATGCATCAAACAACAACTCTTGGATGTATGAT

TTTGGATATAATGCACCTGTATTCCCAATCATAAATGAATCTAGAGAAAATTATATCCAT

AATACTCAAACATTTGATGAGTTCAGATTATCTTTGAGAAGGGTGACAGAAAGATCTCAA

TTCCCAGCTGAAACTTCTGATTATAATTCTCTAACGAGAATTAGAAATTTTATCGAACCA

AATTATACAGCAAATCAACTGGACTTTTCAGACCAACAAACAATTAGAGATGCAGAAAGA

CCTCAAACATGTATTGACGTCTTAGTTGAAGATATACCTGTATCATGTGTAAAAAAGAAG

CGAAATAGAAAGAGAAGTGCTCTTTCCAGTTCAGCGCCTCCCAACACAGATCAGAATCAG

ATGCAACAGTACCATAATGTTGCCTTGGGAAATCACCACTTGGCACTAGGAAAGTCATCA

GATACTGCTCGCGGAGGAAGACAGAAGAAAGGGTATAATGTTGAAGCATTAGTTAAGCAC

TTTAGACGGCTAAACATAAACGCAGGAGTGGGAGACATTGTCTTATACAAGCAGAATGCA

CTTGTTCCCTTTCAGGGCTCATTTGATCCCATAAAAAAACAGCGTCCACGACCGAAAGTT

GACCTAGATGAGGAGACTGATAGAGTGTGGAAGCTTTTGCTGTTAGATATAAATCATGAT

GGGGTTGATGGAACAGATGAAGAAAAGGCCAAATGGTGGGAAGAAGAACGTAAAGTGTTC

CGTGGACGAGCAGACTCATTTATTGCACGAATGCATCTTGTACAAGAGTTCTATGACAAA

GACATAATTGAGCATTCTGGAGAAAAAGCTGTCAGCAGCAATGATTCCTGCAGAATTACC

AGTAGCCCAATTAGCCTAACAGATGAATCAAACTGCAAACTAACAGAATCACCTCAATCC

TCTGGTCCAATGGTTATGATTGAGGAAGGGGAAGAAAAATCATGTTATGATGGTGCTGGG

AAAGAGTTAAATGACATAGTTTCGTCCCAATCCTCTGTCATTTCATCTCAAATATCTGGA

GATTTTTCAAATGATCAAAATCCTGAGAAGATAGGATCATGCTCAGATAGCAACTCAGAA

GTTGAAGATCTGTCAAGCACAGCAAAGTACAACAGTGTTGAAGATCTGTCAAGCACAGCA

AAGTACAACAATTACGGTTCTTTCTGCAAACTTCTTGAAATG

>MS.gene034937.t1

TTTAAGAAAAATATGGAGGTGTTTCAAGTAGCTAGTGAAGGAGTTGGTATGCAACTTGCA

TTTATTTGGAGGAAAATAAGAGAACCTTTGATCGTGCCAATGCTGAGAGCTGCAGTGTTT

ATCTGCCTAGGCATGTCATTGATGATATTGGTCGAGATAGTTTACATGGGAGTTGTCATT

TGTTTTGTCAAGTTGTTTAAAAGAACACCAGAGAAGCGTTATAAATGGGAGACTATTAAG

GATGATGTTGAGTTGGGAAGCTCTAATTACCCAATGGTTCTTGTTCAAATCCCAATGTAC

AATGAAAGAGAGGTTTATCAGCTATCAATTGGTGCTGCATGTGAACTCTCTTGGCCTTCT

GATAGAATTATCATACAAATTCTTGATGATTCAACTGACCCAACAATCAAGGAGTTGGTA

CAGGAAGAATGCCTAAAATGGGGAAACAAAGGTGTGAACATAAAGTATGAAGTTAGGGAC

AATAGAAATGGATACAAAGCAGGTGCACTAAAGGAGGGAATGAAGCATAGCTATGTTATA

CAATGTGAATATGTTGCCATCTTTGATGCTGATTTTCAACCTGAACCTGATTTTCTCTGC

CAAACCATTCCTTTCCTTGTCAATAATCCTGAAATTGGTCTCGTTCAGGCTCGCTGGAAG

TTTGTAAATGCGAATGAATGTTTGATGACGAGGATGCAAAAGATGTCACTTGATTATCAT

TTTACAGTTGAACAAGAAGTAGGGTCTTCTACTTATGCCTTCTTTGGCTTCAATGGGACT

GCTGGTGTATGGAGAATTTCGGCGTTGAATGAAGCTGGTGGGTGGAAAGATAGAACCACA

GTTGAAGATATGGATCTGTCTGTACGTGCCAGTCTCGAAGGATGGAAATTCTTATACCTC

TCCAATATTCAGGTTAAAAATGAATTGCCGAGTACTTTAAATGCCTATAGATACCAACAA

CATCGATGGTCATGTGGGCCAGCCAATCTTTTCAGGAAAACAATTCTGGAGATTCTCACA

AACAAGAAAGTGACTTTGTGGAAGAAAACATATATGGTTTACAGTTTTTTCTTTGTCCGG

AAGATTGTGGTCCACATAAACACATTTGTGCTATATTGCATTGTTTTACCTACAACTGTT

ATGGTGCCTGAGGTTATGGTCCCTAAATGGGGAACTGTTTATATCCCTTCCATCATCACC

CTTCTAAACGCAGTTGCAGCACCAAGGTCATTGCATTTAGTTGTATTTTGGATTCTCTTT

GAGAATACTATGTCTCTAAATCGAGCAAAGGCAACAATTATTGGTCTACTAGAGACTAGT

AGAGTAAATGAATGGATTGTCACCGAAAAACTTGGAAATGCTCTTAAGGACCAAGATAGT

AAAGGACTTGAAAAGCTTCAATTTAAGATTGGAAACAG

>MS.gene034936.t1

ATGGATCTGGATTTTCTCATTCACGCTCTAATTCCATCATGGAACTCCGTTGCTTTACTC

GCCGGATTTTTTACTTACTTAGCCATTGTTGGATCCATTCTCCCTGGAAAACTTGTTCCT

GGTGTTGTTCTCTCTGATTCATCTCGTCTTCATTATCGCTGCAATGGTTTATTTTCTCTT

GTTCTGTTGGTTGGACTTCTTTGGATCAGTGCCAAGATGGAATTTGTATCTCCTACTGCC

ATAGCAGATAGAGGACTTGAGCTGCTGTCTACAACTTTTATCTTCAGTTTTCTTGTGGCC

CTCATACTCTATTTTTCCGGTTGCAAGTCACGAAGTAAAGGTTCATCGCTAAAACCTCAT

ATCAGTGGAGACCTGATACATGATTGGTGGTTTGGAATACAACTAAATCCTCAGTTCATC

GGTATTGACCTCAAATTTTTCTTCGTTAGAGCTGGAATGATGGGGTGGCTACTTATCAAT

CTATCAGTTCTTGCTAAGAGCATTCAAGATGGTACTTTGAGCAAATCAATGATTCTCTTC

CAGCTATTCAATGCACTATACATCCTGGACTACTTTGTACACGAAGAGTACATGACATCC

ACATGGGACATAATTGCAGAGAGACTGGGCTTCATGTTGGTCTTTGGAGATCTAGTGTGG

ATTCCTTTTACTTTTAGCATACAGGGCTGGTGGCTCTTGAGGAACAAGGTTGAGTTAACA

AATGCAGCTGTTGTAGCTAATTGCTTTGTCTTCCTTATTGGATACATGGTATTTCGAGGA

GCAAACAAGCAAAAGCATGATTTCAAGAAGAATCCAAAGGCTCCTATATGGGGTAATCCT

CCAAAAGTCATTGGAGGCAAGCTACTTGCGTCTGGTTATTGGGGTGTTGCTAGACACTGT

AACTACCTAGGGGACTTGCTGCTTGCTCTCTCTTTTAGCTTACCTTGTGGCATCAGTTCA

CCAGTTCCATACTTCTATCCAATTTATCTTCTTATTCTGTTAATCTGGAGAGAAAGAAGG

GATGAAGCTCGTTGTGCAGAGAAGTATAGAGATATATGGTTAGAGTATCGTAAACTTGTT

CCATGGAGAATTTTGCCTTATGTTTATTAA

>MS.gene034947.t1

ATGGCTTCTTGCGACGACGACGACGATTTCTCTCTCCTCCACGACGATAACAACCACCAC

TCTCACCACCACCACCTCCACCAAACCTTCGCACCTCACCACCACCACAACCACCACTCT

CACCACCACTTCTCCACAGCTTCAGTCCAATCCAAACACATCACCACCTCCGTCTCCGCC

GCCGTCGCCGACGACGACGGAGACGACACCGATGACTACGGTAATCCATTCGACGATGAC

GCTGGTAGCGAGAAACGTAAAGATCGAGATCGCGATGAGATCTGTGATGCAACGACGTCG

TATGGATTCAACAACAAACGGTCAAAACCTGTTTCTAGCGGTAACGGTGGAGGAAGCGCC

GGAGTAGGAGCAGAGTATAGAAAAGATCGAGAAGAATGGAGTGATACAGCGATAGTGTGT

TTACTCGAAGCTTATACAGAGAAATTTACTCAGCTGAATCGAGGGAATCTAAGAGGAAGA

GATTGGGAAGAAGTAGCTTCAATGGTGAGTGAAAGATGTGAGAATCAATCGAAGAGTGTG

GAACAGTGTAAGAACAAGGTTGATAATTTGAAGAAAAGGTATAAGCTAGAGAGACATAGA

ATGAATAATGGTTGCATTTCTGCTAGTCATTGGCCTTGGTTTAAGCAGATGGAACATATT

GTTGGGAATTCGCTTCCGGCCAAGTTTGCTGATGATGATAAAGCTATTGTTCCTGCTTCC

ATTTCACCTAGGCAATCTAAGAGATATGGAGTGGCTACGTCCAGCAGTGGAGGTCAAGTA

AACAGCCTGAAGTCCAAAGCATTGTCAAACCTTAGATGGCGGAGAGTTGTTCTAAAAATT

AGTGGATCGGCACTTACTGGATCCGATACCTGCAATATTGACCCTAAGGTGGCCATGTTA

GTTTCAAATGAAGTTGCAATAGCTTCCCGTCTTGGTGTGGAGGTAGCAATTGTTGTTGGA

GGTCGTAATTTCTTCTGTGGAGATGCATGGGTAACTGCAACTGGTTTAGAAAGATGTACT

GCATACCAAGTTGGCATGATGGCAACTGTGATGAACTCCTTACTGCTTCAATCAACTTTA

GAGAAGATGGGTGTTCAGACTCGTGTACAAACTTCAGTTTCAATGCAGGCGTTTGCTGAA

CCGTACAATAGGCAACGGGCCATCAGGCATCTTGAGAAAGGAAGAGTTGTTATATTTGGT

GGCATTGGTTTTGGTGCTGGTAATCCACTTTTCTCAACCGACATATCTGCTGCTCTTCGG

GCTTCAGAGGTTAATGCTCAGGCTGTCCTCAAAGGTACCAACGTCGATGGCGTATATGAC

TGCAACTCACGAGACAACAATTTCACTTTCGAGCACATATCCTTCAGGGAGCTAGTGTCT

AGAGGTGTCACCTCTATGGATATGTCAGCCCTGACATTCTGCGAGGAAAATGCCATTCCT

GTTGTAGTTTTTAATCTATTGGCACCCGGGAACATTTCAAAAGCTTTGTGTGGAGAACAA

GTTGGCACACTTATTGACCAAACTGGAGCTATAAGCTAA

>MS.gene034944.t1

ATGAAGGGTGAAACAGTGAACGTAACATGGCTAGGTGGTATTTGGCCAGTTTCACGCAAG

AGTGGTTCAGATGAAAATAATGAAATTGGAATCATGGCATTTGAGGTTGCAGGGTTGATG

TCTAAGGTTGTTAATTTATGGCATTCTTTGAGTGACAATGAATTAATGAATCTAAGAGAA

TGGATAGTAAGTTCTGTTGGTGTCAAAATGCTTGTTTCTGATGATGAATATTTCTTGATG

GAGCTTACAAGGAATGAGATACTTAATAACTTTCAATCTTTATCACAATCTGTGGCAAGG

TTAAGCAAGAGGTGTTGGGAATATAGATTGAAAAAGATGGAGAAAAAGGTGAAGAAAATG

GAAAGATTTGTTTGTTCTTTGTCGCTTTTGTCACAAGAGCTTGAGGTGTTAGCTGAGTGT

GAACAAACTTTAAGGCGAATGAAGTTGACTCGCGATGTTGTTAATAAGGCGAAATTGATC

GAGTTTCAGAAGAAAGTTATGTGCCAGAGGCAACAAGTGCAAAATGTAAGAGATTTGTCT

CCTTGGAATAGAAGTTATGACTATATTGTTAGGTTGTTGGCAAGATCATTGTTCACAATT

CTTGAGAGAATCATACTTGTGTTTGGAAATAGTCATTTACCAATTGAAAATCTAAAAAAT

GATTCCTCAGGAACAATGAATGATGCTAATAATCGTCTGGCGCGCAACCATTCCTTCCCT

GCTCTCCATGTCATGCATTCTTCGGTTCATCCTTCTCCCGAGACGAATCTAAATGAATTT

TGTTCAGGGCCTATTGGAAGAAAGAACAAGAGCAAGAAAAAGAAGAAAGAGCAACAAGTG

CTACTTCATTCACAAGATTCATCATGTGAAAAGTTTCTTCCCTCAGAAGGTAAGCAATTG

AAATATATTGGATCCTTCAAAGGTTGCATATCTGTTCAAAATGATTCTCATGTAGTACAG

AGTTGTATACCAACTAATGGTAGTTCTATGAGGAAAAACATTGATGTCAACACGAAATCG

CTCCTCAATAAACCGTCTCTCTTCCACAGAAGTAGAGTTTATTTTAAACTATCTCTCAAG

GAAAAGTTAAAACCGATTCCATCAACTCTTGGTGATGCAGCTTTAGCTATACATTATGCA

AATGTAATTGTATTGATAGAGAAGATAGTGTCATCACGTCGCGCAAATACAATCGATGTT

CGAACGAGAGACGATCTTTACAATAAGCTACCAACATCTATAAGAACAGCTCTGAGGGGT

AAGCTTAAATGGTATGCGAAAAGTAAGCTCGAAACAGATTGGAATGTGGTACTCAAACAG

ATATTGGAGTGGTTAGCTCCACTTGCACACAACATGGTAAAGTGGTATTCTGAGAGGAAT

TTTGAGAAGGAATATACTAGTTTGAAAGCAAATGTTTTACTTGTACAAACTCTTTACTTT

GCAAACCAAGCAAAAACTGAAGCTGCAATAGTTGAACTCCTTGTTGGTCTTCATTATTTA

CCGACGACGATGAAGACCGGATTCACGGTGTACAAGCTCTACAGAGCCCTAACTTACGCC

GCGTCTCCTTTAATCCGTCTTCACCTGCGGTGGCGCAGATTCCGCGGCCTAGAGCACCTT

CAGCGGTGGCCGGAGCGGCTAGGTCATCCCTCTCAGCCTCGGAAGCCAGGTCCACTCGTA

TGGTTCCACGCCGTTTCATTAGGCGAAGGAATGATTGCGATTCCAGTGATCAAACACTGC

ATTCGGAAAATGCCGAACTTGAATGTCCTCATCACCATCACCACTCTCTCTGCATTTGAA

GTATTGAGCAAAAAGATTCCAAGTGAAGTCATTTTACAGTTTTCACCGGTTGATACGCCT

TCTTCCATCCGTTCTTTCCTTCATTACTGGAAACCAAGTGCTATTGTGCTAATGGAAAGC

GAGCTCTGGCCAAATCTTATCATGGATGCTTCCAAAAATGGTATAACACTGGCACTGTTG

AATGCTCGGATTTCTGAAAAGTCCTTTAAACTTTGGTCAGGGCCAGTGCTTCTTCCATTG

ATTTCGTTGATGCTATCCAAGTTTTCCTTGATTGTTCCATTGAGTACAGAGCAGGGTATC

CGGTTTCAGCTACTGCAAGCCCCTCCTTATATCATAAACTTTTCTGGTGATCTAAAGTAT

GTAATAGAAGACTTTGGAGTCAACGAATGTGGCAGAACGAATATAGATAACCTGAGACAA

CAGCTTTCTCACAAGCAGGTTTGGATGGCTTCTTCAATTCATAGGGGAGAAGAAGAAATA

ATATTAGGAGTTCACAGTGTTCTTATGCAGCTGCAACCTAATATAATGACTATTATTGTC

CCTCGGCATCCGCAGCAAGGGCGAGAGATTGCCAAAAAATTGGAGAGACAAGGACATGAT

ATAGTTTTGAGATCACAACATGAGAGGCTTAAGCCAGAAACAAATATTTATGTGGTGGAC

ACTTTGGGCGAATTAAGACAGTTGTACACATTAACACCAATAGCTGTTATTGGGGGTTCA

TTCCTCCCTGGTTTGTCTGGCCATAATATCTCAGAAGCTGCTGCTGCTGGTTGTGCTATT

CTGACAGGTCGTCATGTTGGCCATTTCTCCCACATGGTACTAGAAATGCAACAATTGAAT

CCTCTGTCGGTTCTTCAGGTTTCTGGAAAACTGGAGCTTGAAAAATCTCTCACTGAGCTC

TTCACAAATACAACACTTCTTGAAGCTCGTCGTACAGCTGCAAAGGAAGCATTTTGTATG

TTGTCCAGTGATATTGTTGCAAACATATGGAGTTTGCTAAATTTTCATATTTTTAGTAGA

TTGTTTGCTGAGATGAAGCCTCATAATATTACAGCTAAGACTATGAGAAATGAAGCTTCA

TAA

>MS.gene034945.t1

ATGGCATGCATGTGGCGTAAGAATGTCTGCAGCGGGAAGAAACTGCCGTCAGACATACCG

CGAGGTCACTTGGCCGTGACAGTAGGAGAGACAAACAGGAGGTTTGTGATAAGAGCTGAC

TACCTGAACCATCCAGTTCTTCAGGAACTGCTAGACCAGGCATACGAAGGATATGGCTTC

AACAAGAGTGGTCCTCTGTCTATACCTTGTGATGAGTTCCTGTTTGAAGATATTCTTCTT

TCCCTTGGAGGAGGAACAGTTGCACGGCGGCCGTCTTCCCCTGTGCTTACAAAAAAGCTA

GATTTGAGGTTTTTGAAAGACGCTGTACCACTACTTGAAGCCTTTGACAGCAAAAGAAGC

AACAACTACAAGAATTAA

>MS.gene034946.t1

ATGATGAACTATGTCATCATGTTCTTCTTCTTCCTCTTCCTAAGCATCTACATTGTACCA

TGTCTAGCACATAATGACACACAAGCACTTACTCTCTTTCGCCAACAATCTGACACACAC

GGTCAACTCCTTACCAACTGGACCGGACCTGAGGCCTGCTCGGCCTCATGGCACGGTGTC

ACTTGCACGCCAAACAACCGCGTCACAACCCTCGTACTTCCTTCCCTCAATCTCCGAGGA

CCCATCGACGCTCTTTCTCCCCTTACCCATCTCCGCCTCCTCGATCTCCATAACAACCGC

CTGAATGGCACTGTTTCAGCCTCTCTCCTCTCCAACTGCACAAACCTCAAACTCCTCTAC

CTTGCCGGAAACGACTTTTCCGGCCAAATTCCACTAGAAATCTCCTCCCTAAACAACCTC

CTCCGTCTTGACCTCTCCGACAACAACCTCGCCGGAGATATTCCTAACGAAATCTCCCGT

TTAACTAACCTTCTCACATTAAGACTTCAAAACAACGCATTCTCTGGTAACATCCCTGAC

CTCTCTTCCGCTATGCCGAATCTCACAGAGCTCAACATGACAAACAATGAATTCTACGGC

AAAGTACCAAACACAATGCTTAACAAATTCGGAGATGAAAGCTTCTCCGGCAATGAAGGC

TTATGTGGTTCAAAACCTTTCCAAGTTTGTTCCTTAACAGAGAACAGTCCTCCTTCTTCC

GAACCGGCTCAGACCGTTCCTTCGAACCCGAGTTCTTTCCCTGAAACAAGCGTGATCGCA

AGACCAAGAAGCCAACACCATAAAGGGTTAAGCCCTGGAGTAATTGTAGCAATTGTGGTG

GCAATTTGTGTTGCATTGTTAGTGGTAACATCGTTTGTTGTTGCTCACTGTTGTGCCAGA

GGGAGAGGTGTTAATAGTAATTCCTTAGCAGGTAGTGAGGCTGGGAAGAGGAAGAGTTAT

GGTAGTGAGAAGAAGGTGTATAATTCAAATGGTGGGGGTGGTGATAGTAGTGATGGGACT

AGTGGAACTGATATGAGTAAGCTTGTGTTTTTTGATAGGAGGAATGGGTTTGAGTTGGAG

GATTTGTTGCGTGCATCTGCTGAGATGCTTGGGAAGGGTAGTCTGGGAACTGTTTATAGG

GCAGTGCTTGATGATGGTAGTACAGTTGCTGTCAAGAGGTTAAAAGATGCAAATCCTTGT

GCTAGGCATGAATTTGAACAGTATATGGATGTGATTGGGAAGCTTAAACACCCTAATATT

GTTAAACTTAGAGCTTATTATTATGCCAAGGAAGAAAAGCTTCTAGTCTATGATTATCTC

TCCAATGGGAGCTTGCATGCTCTTCTTCATGGGAACCGTGGACCAGGGAGGATTCCATTG

GATTGGACAACAAGAATAAGCTTGGTATTGGGAGCAGCAAGAGGGCTTGCTAGGATCCAT

ACAGAGTACAGTGCAGCAAAAGTGCCACATGGGAACGTGAAATCATCCAATGTACTACTT

GACAAGAACGGTGTTGCTTGCATATCTGACTTTGGGTTGTCACTACTGTTAAACCCTGTT

CATGCAATTGCACGATTGGGTGGGTACAGAGCACCTGAACAGACAGAACAAAAGAGACTG

TCTCAGCAGGCAGATGTCTACAGTTTTGGAGTGTTGCTGTTGGAAGTTCTCACAGGGAAA

GCTCCTTCATTACAGTATCCTTCCCCAGCGAATCGCCCTCGTAAGGTTGAAGAGGAAGAA

ACTGTGGTGGATCTTCCCAAATGGGTTCGATCAGTCGTGAGAGAAGAGTGGACTGGCGAG

GTTTTTGATCAAGAACTTCTGCGATACAAGAACATTGAGGAAGAGCTTGTGTCAATGCTG

CATGTTGGGTTGGCTTGTGTGGTGCAGCAGCCAGAGAAGAGGCCAACTATGGTAGATGTT

GTTAAAATGATTGAGGATATTAGAGTGGAGCAATCTCCTCTGTGTGAGGACTATGATGAA

TCACGCAATTCACTTTCACCTTCCATTCCCACTACTGAAGATGGTCTTGCTTAG

>MS.gene034943.t1

ATGCACAACAACAGCAATGTTTCAGTTGGGTCACCACAACAACTAGCAACAACAAACACA

TTACCTCAAGAAATGAACATGAACATAAGTGTTGGAAACAGTATTGTAGCTACTATTGGA

ACATCAATAGTTGCTTTAGAAGCACCAACGGTCACACCAACAACAACACCAGGAAGTTTG

GATTTGTTTGGTAAGAAAAAGAGAGGAAGACCAAGAAAATATGATGCTGATGGAAACCTA

AATCCAAGTTACAAGAAGATTGTTAAAACACCAACACCAATACTAACATCACCACCAGGA

TTCACTTTGTCTACAAATGAGTTTTCCTCCAAAAAGGGTCGTGGTGAGGTGTTTCCAAGC

ACAGCTGCTGTGGATTTTACACCACATGTAATCACAGTTTATGCAGGAGAGGATGTTGGT

GGAAAGATCTTGTCATTTGCACAGAAGAGTCCAAGAGGAATATGCATTCTTTCTGCCAAT

GGAGCAATCTCTAAAGTTGCACTAGGTCAACCTGGTTCTACTGGTGGTAGCATTTTGACT

TATGAGGGACGGTTTGAGATACTATCTTTGTCTGGATCTTACACGGCTTCCGACAATAGT

GGCATAAGAACTCGCGAGGGTGGATTGAGTGTCTCGCTAGCTGGTCCTGACGGTCGAGTA

ATCGGAGGTGCTGTTGCTGGTGTTCTTATAGCTGCTGGCCCCATACAAATTGTAGTGGGG

AGCTTCGTGTCAAATGGTAACAACAACAAGCCTCTTAAAAGGAAGTACCAACGTGAACAA

ACAGTGGCTTCTCCAACTTCAACTGGTCCAGAAATAGTCACAGCAGCACGACCTATCTCA

CAAGCAAATATTCATGGTGAAAACTTTATGATACCTATTATGTCTCATCAGCTTCCTGAT

CAAATCCAGAGAGAATCTATCAATGTTTCAAGTGATAAACTGAACTTAGATGCCACTCCT

GATGATGATACTTGGAACAGTTCTGATGAATATTCAGATCAAAGGACATCCCCTGACATC

AATATTTCTTTGCCTGATGAATAG
