# Supplementary material for: Identification of Genetic Loci Associated With Crude Protein Content and Fiber Composition in Alfalfa (Medicago sativa L.) Using QTL Mapping
Source: Front Plant Sci. 2021 Feb 18;12:608940. doi: 10.3389/fpls.2021.608940 (PMC7933732; doi:10.3389/fpls.2021.608940)
Supplement: Supplementary file 12 [file Table_10.docx]

### Table S10. Potential Candidate Genes of *qlignin5C*

>MS.gene09356.t1

GGGAAATTGAAGCCTAGCTCCAAAATGCTTGAGAATGTTGCCGGGCAGAAAGTGAAAGCC

GATGAAATAGCAACAGTTAAGATTGGTCTTGAGAGGCCTAATGGTGTCGGTGAAAGCAAA

CCAAGTCTTAATGGTTCTGCAACTAATGGAAATAGCACTGCTCCTGCTAGCAAGGCACAG

GTTGTTGGTTGGCCTCCAATAAGATCGTTTAGGAAAAATTCATTGACCACTGCTTCAAAG

AACAATGAAGAGGTTGATGGAAAATTGGGATCAGGAGGTGCTGTGTTTGTGAAGGTCAGC

ATGGATGGTGCTCCGTATTTGAGAAAAGTAGACTTGAAGAATTACACTGCATATTCACAA

CTATCTTCTTCTCTTGAGAAGATGTTCAGCTGTTTCACCATAGGTCAATGTGAATCTCAT

GGAAATCAGATGCTGAATGAAACCAAGCTGAGGGATCTGCTTCATGGTTCAGAATATGTT

ATTACTTATGAGGATAAAGATGGTGATTGGATGCTTGTGGGTGATGTTCCCTGGGAGATG

TTTATTGATACATGCAGGAGGCTAAGAATCATGAAGAGCTCTGATGCCATTGGTTTAGCT

CCCAGAGCAGTTGAAAAAAGTAAAAGCAGGAACTAA

>MS.gene09355.t1

GCACCGGTGCATGGGAGAGTGCATTGACAAATACATTGTCTCCCGGAGATCGTACTGTAT

CTTTTCTAATGGGCCAATTCAGCTTGCTTTGGATTGAACAACAAAAACGCCTCAACTTTA

AGGTTGATGTTGTAGAAAGTGAATGGGGTCAGGGTGCTGATCTTGATGCTCTAGAATCAA

AACTTGCTTCAGATAAAACACATACCATAAAGGCAATTTGTATTGTTCACAATGAGACAA

CAACAGGGGTCACAAATGACTTGACTAAAGTGAGAAAAATCCTTGACAAGTACCAGCACC

CAGCACTCATTATTGTTGATGCTGTGTCTTCCATTGGTGCTCTTGATTTTCGCATGGATG

AATGGGGTGTAGACGTGGTAGTGACTAGCTCTCAGAAAGCTCTCTCTCTTCCTACAGGAA

TGGGAATTGTTTGTGCAGGACCTAAAGCCATTGAGGCTTCAAAATCTGCTACATCACTTA

GATCTTTCTTTGATTGGAATGGTTACTTGAAATGCTACAACTTGGGAACTTATTGGCCAT

ACACTCCTTCCATTCAGTTGCTCTATGGTCTAAGAGCAGCCCTTGACTTGGTTTTTGAGG

AAGGATTTGAAAATGTGATTTTAAGGCACAAGCATTTAGCCAAAGCAACCAGGCTTGCAG

TAGAGGCATGGGGATTGAAGAATTGTTGCCAAAAGGAGGAGTGGTATAGTGCTAGTGTGA

CTGCTATTGTTGTTCCTCCTAACATTGATAGTGGTGAAATAGTTAGTAGGGCATGGAAGA

GATACAATTTAAGCTTAGGAGGTGGATTGAACAAAGTTGCTGGGAAAGTTTTCAGAATAG

GACATGTTGGCCATCTAAATGAGTTGCAACTTTTGGGTTGTCTTGCTGGTGTGGAGATGA

CACTCAAAGATGTGGGTTACCCTGTCCAGCTCGGAAGTGGAGTTGCTGCTGCAAGTGCTT

ATTTACTAAACAATGTTCCTATAATCCCTTCAAGAATTTGA

>MS.gene010034.t1

ACCAAAAGTAATTTGAATCTTAAAGCTACTGAACTTAGATTGGGACTTCCAGGATCACTT

TCTCCTGAAAGAGATTCATCAGATTTTTGTTTAAGAAGTTCAAAGCAGTTTGATGAAAAG

CCTCTTTTTCCTTTGCACCTCAAAAAAGATGATCATCTTTTTGAATCAAAGCCTGCTGTT

TTAGGTAACAAAAGAGGGTTTTCTGATGCCATGAATGTTTTCTCAGAGGGGAAATTGAAG

CCTAGCTCCAAAATGCTTGAGAATGTTGCCGGGCAGAAAGTGAAAGCCGATGAAATAGCA

ACAGTTAAGATTGGTCTTGAGAGGCCTAATGGTGTCGGTGAAAGCAAACCAAGTCTTAAT

GGTTCTGCAACTAATGGAAATAGCACTGCTCCTGCTAGCAAGGCACAGGTTGTTGGTTGG

CCTCCAATAAGATCGTTTAGGAAAAATTCATTGACCACTGCTTCAAAGAACAATGAAGAG

GTTGATGGAAAATTGGGATCAGGAGGTGCTGTGTTTGTGAAGGTCAGCATGGATGGTGCT

CCGTATTTGAGAAAAGTAGACTTGAAGAATTACACTGCATATTCACAACTATCTTCTTCT

CTTGAGAAGATGTTCAGCTGTTTCACCATAGGTCAATGTGAATCTCATGGAAATCAGATG

CTGAATGAAACCAAGCTGAGGGATCTGCTTCATGGTTCAGAATATGTTATTACTTATGAG

GATAAAGATGGTGATTGGATGCTTGTGGGTGATGTTCCCTGGGAGTAA

>MS.gene010032.t1

ATGGAATGTCAACGCTCGGATGGAACGGGGCTAGTAAGGTGGTGGACAGAAATTGATCCA

ACTGAGGAGATTAATGGTGGGCCCAAGAAAAAAGAGGTCTCAACAAAGAAGAAGATTTTT

GCTCCGAATCTTACAAAATCGGATCAAATAAATCAAGCCTTAGCAGAAGGAATGACGGAG

AAGGACGAATTTCTGTATGAGAATGGGGGAGGTGCAGAAGCGCTTTGGAAGCTGATAGAT

TTGGAAGCCCAACAAAAAAATGCAAGCCCACAAAATTCTATGGTGGGGGACAGCCAGAGG

GGCCCACGTGGGGCCAAAGGAAAGGAAGAAGAGAAAGGTGTGAACAGCAGGGTCCCACAA

GGGGACAGCCGCCAGACATCACACGCAAAACAAGGCCAGCTTGTCTTGTGTGACAACACT

CAACAGCAGGTGGTCCCACAGGGACAAAAACCAGCACAGCAATCATTAAGCATTCATTAC

AAAATGACTGGCACGGATTACAGAAGTCTTTTTACTAAGGAATCTGCACCAATGCCAATC

AATGCTGCTTTTAGTACAAAAGGTGGACCAGTTTCAAACTTTGATGAGTAG

>MS.gene010015.t1

ATGACGCTTTCTAAGTGTTTCTCTCACTCTGGACCCAACAATGCAATACCAGTTTCTGGG

TTGGTTGAATCTGGTGAAATGGAATCTCTTGTTGTGGTTTCGTTTTATAAGTTTGCGGAT

TTCCCTGATCATGCTGTTTTGCGTGTGCCCTTGAAGCAATTATGTCAACAATTGCGTGTT

TCAGGCGGTATCATTCTTGCGCCTGAAGGAATTAATGGCAGCCTATGCGGCACGCGGGAG

TCGGTGGAGAAAGTTCTTGCATTTATTCAGAGTGATGATCGTCTGAAGGGGCTAAGACGT

ATTGAATCGCCTGTCAGTCCTGAGGAGGAGGCCATCCATCACGGTAATCATGGACACAGT

GCCAGTTCTCCTCTTGCTGCTGGGGAAGACGCACCTTTCCGATGGGATCATGTTAGAGTC

AAGTTGAAGAAAGAGATTGTCACTCTTGGGATGCCTACGGTATCACCTATTGAAAGGGTT

GGAAAATATATTGGCCCAAAGGAATGGAATTCTTTGATTAGCGATCCTGATACTGTGGTA

ATTGATGTGCGAAATAACTATGAAACAAGAATAGGAAAGTTTAAAGGAGCAGTTGATCCT

TGTACGACATCATTTCGTGAATTCCCATCTTGGGTTGAGGAACGTTTCGAGCTTACCAGA

AAAGATGTTGAGCATCCAAAAGATGATGTAAACCATCCAGACCAAAGTGCTGAAAATGAA

ACGAAGAATCAGAAACAACATTTACCACGTGTTGCAATGTATTGCACTGGGGGTATTCGT

TGTGAGAAAGCTTCAAGTCTACTTCTCAGCAAAGGTTTCAAAGAGGTTTATCACCTGGAA

GGTGGGATCCTGAAATATCTTGAGGAAGTTCCGGAGAGACAAAGCCTTTGGGAGGGAGAG

TGCTTTGTTTTCGACAAGAGAGTCTCCGTTGAGCATGGTTTGGCACAAGGAAATTTCAAG

CTGTGCTATGGTTGTAAGCAGCCTGTGAGTGATGCTGACATGGAATCCCCAGAATATGAA

TATGGAGTCTCCTGTCCTTACTGTTTTGCACAGAAATCGGAAGAAGAAAAGGAGAGGGCT

CGAGCTCGACAAAAACAATTTGAGAGATGGGGGATCATAGGTGGTCCTGACAAGGGCCGC

CGGCCGACACGCGAACAGGACAGTGCTAGCAGGGATGAAAATCAACTTTCAAAGTCTGTT

TAA

>MS.gene010013.t1

ATGTCGTTGGATTGCAATGTGTATGTTGCAAATGCTCTTGTTACGATGTATGGCAAGTAC

TCTGGTGGTATTGGTGGAGGTTGTGATCATACTTCGGATTATGCATGGATTGTTTTCGAG

TCGATGGAGTATAGAAATCTTATATCTTGGAATTCGATGATTTCAGGTTTTCAATTCCGT

GGACTTGGCGACAAAGCTATTGGTCTGTTTGCGCATATGTATTGCAATGGAATTGGGTTT

AACAGTACCACATTACTCGGTGTTTTATCTTCTTTGAATCATTGCACGAGTACTTCGGAT

GACATCAACAACTCACATCACCTGAAGAACTGTTTTCAATTACACTGTCTCACAGTTAAA

AGTGGTTTTATTTCAGAAGTTGAAGTGGTAACTGCATTGGTAAAATCCTATGCAGACCTA

GGGGGTCACATTTCTCCCTGTTTTAAGCTCTTCCTTGATACAAGTGGCAAACGTGATATT

GTTTCATGGACTGCTATTATTTCTGTGTTTGCGGAGCACGATCCTGAGCAAGCTTTCCTT

CTTTTCTGTCAACTTCATCGAGAAAATTTTGTGTTGGACCGACACACATTCTCAATTGCC

TTAAAAGCTTGCGCTTACTTTGTCACTGAGAAAAATGCAACCAAAGTTCACTCACAAGTA

ATTAAACAAGGGTTTCACGATGACACAGTTGTTTCAAATGCCTTGATACATGCTTATGGG

GGAGATGCACTAGAGCTCTTTAAGCGAATGGATGTTCATCCAGATTCTGCAACCTTTGTT

GCTCTTCTCGCGGCATGCAGTCACGCTGGACTTGTTGATGAAGGCATGAAATAA

>MS.gene010027.t1

ATGCCGAATCCCACTCCAACAACCCCCCACCGGTCCAGAACCCGACCATCCACATCAACC

TCCCGTCCCGTCCAACCGGTCCAACCACGAACCCCCTTACGCCAACTCCTCCGAGTCGCA

TCCGTAGCAAGCGGAATCCAATTCGGTTGGGCTTTACAGCTCTCCCTCTTAACCCCTTAC

GTTCAACAACTCGGTATCCCTCATAAATGGGCGAGCATCATCTGGCTCTGTGGACCGGTC

TCCGGTTTATTCGTCCAACCGTTAGTCGGTCATTTAAGCGACCGATGCTCCAGCCGGTTC

GGCCGTCGGAGACCGTTTATTTTAGTCGGCGCGGCGTCGATCGTCCTTGCCGTTGTTATA

ATTGGTTATGCTGCTGATATTGGTTACTTGATCGGTGATGATATAACGCAGAATTATCGT

CCTTTTGCTATAGTTGTTTTTGTGATCGGGTTTTGGATTCTTGATGTTGCTAATAATGTT

ACTCAAGGTCCTTGTAGAGCTTTACTCGCTGATCTTACTTGCAATGATGCTCGAAGGACG

CGTGTTGCAAATGCCTATTTCTCCCTGTTTATGGCTGTTGGTAACATTCTTGGCTATGCA

ACTGGATCATACAGTGGCTGGTACAAGATTTTTACTTTCACACTTACTCCTGCTTGCTCT

ATTAGTTGTGCAAATCTCAAGTCTGCGTTCTTTCTCGACGTTGGTTTCATTGCGGTAACG

ACATATCTCAGTATTGTGTCAGCTAATGAAGTGCCTCTAAGTTCAAGTGGGGCAGCACAT

ACTGGAGAAGGGGCAGGAGAGTCGGGTAGTGCGGAAGAAGCTTTCATGTGGGAATTGTTT

GGGACATTTAAATATTTTTCAATGCCTGTATGGATAGTACTGTCTGTAACTGCTCTGACA

TGGATTGGATGGTTCCCATTTATGCTCTTTGATACTGATTGGATGGGTCGGGAAATTTAT

GGTGGTGATCCAGAAGGAGGCCTTATTTATGATACTGGGGTTAGAATGGGAGCACTTGGT

TTATTGCTTAATTCAGTTGTTCTTGGAGTAACATCATTGCTCATGGAAAGGCTATGCAGA

AAGCGAGGGGCTGGTTTTGTGTGGGGACTCTCAAATATCTTTATGGCTATTTGCTTTATT

GCAATGTTAGTATTAACCTATGCGGCAAATAGCATTGGCTACGTAAGTAAAGCTCAACCA

CCACCAACGGGCGTTGTGATCGCAGCAATAGCAATCTTTACCGTTCTTGGGTTTCCAATG

GCAATCACTTATAGTGTTCCATATGCCTTAATTTCAACGCATATCGAACCATTGGGACTT

GGCCAAGGGTTATCAATGGGTGTCCTGAATCTGGCAATAGTGGTCCCGCAGATTGTGGTG

TCCCTGGGAAGTGGACCGTGGGATCAATTATTTGGTGGAGGAAACTCTCCAGCCTTTGCC

GTGGCAGCTGTTGCAGCCCTTTTGAGTGGACTCCTGGCTCTATTAGCTATTCCCCGAACT

CGTACGCAAAAGCCTAGAGTCCGCATATGA

>MS.gene010031.t1

ATGGAGCCTTTGATGATTTCCAACCCTAATTTGCTTGATTGCTGCAAATGTTTTCAACCC

TTAACTATTCCTGTCTTTCAGTGTGACAATGGTCATATTGTTTGCTCTACCTGCTGTACT

AAACTTAGGAACAAATGTCATGAGTGCTCGTTGCGCATTAGCTCAAAACGTTGTAAAGCC

ATTGAGAATCTATTGCTATCTATCGAAATGTCATGTCCAAATGCAAAACATGGTTGCAAG

GAGAAAATAAGTTACACTGGAAATAGGAAGCACGAAGGCGAATGCATCTACCTTCCATGT

TACTGCCCCCTTTCAGGCTGTGACTTTGCTGCATCATCGGAAGTGTTGTCCAACCATTTC

AGCCATAAACATGGGGATTCTCAAATCAAATTTTCTTATGGTCACTCCTTCATTGTCTCT

TTGAAGTCTAACGATGAAACCATTGTTCTTCAAGAGGAAACTGAGGGAAAACTATTTATT

CTCAATAATAGATCCACGCTTTTGGGAAATGGAGTCAATATTTGCTGCATTGGTCCCAAC

TCGTCTGAGTCCGAGTATAGTTATGATATATTGGCTAGGTCTCGGATATGCAAACTGACA

TTACAGTCTTTTGCGAAGAACGTCCAACAGGTTGCTTTAGCAACTCCTTCATCAGAGTTG

CTTGTGATTCCATTTGGTTCTTCTGAACCTCTCCAGCTAGATATTTGCATAACTCCCATG

ATGCAAATCTACGTAGAAGACCTGACTGGAAAGACGATTCCCCTGAGGGTTGAGAGTTCA

GACACAACAGTCGATGTGAAGAAGAAGATTCTTGACAAGGAAGGGATCCCAATGCACGAG

CAACGTCTGATCATTGACGGTAAGCTACTAGAGGACAACCGTACCCTTGCCAGTTACAAT

ATCCAAGAGAAGTCAACCATCAGCCTGGTCCTCCGCCTCCATGGGAGCTAG

>MS.gene010033.t1

ATGGATGAATGGGGTGTAGACGTGGTAGTGACTAGCTCTCAGAAAGCTCTCTCTCTTCCT

ACAGGAATGGGAATTGTTTGTGCAGGACCTAAAGCCATTGAGGCTTCAAAATCTGCTACA

TCACTTAGATCTTTCTTTGATTGGAATGGTTACTTGAAATGCTACAACTTGGGAACTTAT

TGGCCATACACTCCTTCCATTCAGTTGCTCTATGGTCTAAGAGCAGCCCTTGACTTGGTT

TTTGAGGAAGGATTTGAAAATGTGATTTTAAGGCACAAGCATTTAGCCAAAGCAACCAGG

CTTGCAGTAGAGGCATGGGGATTGAAGAATTGTTGCCAAAAGGAGGAGTGGTATAGTGCT

AGTGTGACTGCTATTGTTGTTCCTCCTAACATTGATAGTGGTGAAATAGTTAGTAGGGCA

TGGAAGAGATACAATTTAAGCTTAGGAGGTGGATTGAACAAAGTTGCTGGGAAAGTTTTC

AGAATAGGACATGTTGGCCATCTAAATGAGTTGCAACTTTTGGGTTGTCTTGCTGGTGTG

GAGATGACACTCAAAGATGTGGGTTACCCTGTCCAGCTCGGAAGTGGAGTTGCTGCTGCA

AGTGCTTATTTACTAAACAATGTTCCTATAATCCCTTCAAGAATTTGA

>MS.gene010018.t1

ATGGAAATTGCATTAACTTTCACAGATGGTTCGAAGTCAACAACCTGCATGCTTTTAGAT

GCATCAGCAGGAGGTACCATGAAGAACATAACTGCAGCAGAGATGCGAGAGCTCATTGAC

AACATGTTCTTGAAAGAGTATCACCCTCAAAGCAAGAATATGGGTGTGGTGAAGAAGCAT

GGAGTTCTAATATTGGAAACACATGATGCCTTACTAGAAAGCAATAAACTCCTTAGTGAC

AAAATTGAAGCACTAGCTAAGAGGTTGGAAGCGCAAGAAGTAGCAAAGATGTCCATCAAC

AGTGTAAGCTACAATTTTCTGAGTAAGCTCATGAAAGTGGTGCATGCCTTTCAGCAAGTT

TTGGGTTATCTGAAGAACATGTCAAGTACATGGGTGTGTATACCAGACAACAACGAAATC

CCTAATCCAACACTTTCAATTCCAGCTGGCTAA

>MS.gene010011.t1

ATGTCGTTGGATTGCAATGTGTATGTTTCAAATGCTCTTATTACGATGTATGGCAAGTAC

TCTGGGGGTATTGGTGGAGGTTGGGATCATACTTCGGATGATGCATGGATTGTTTTCGAG

TTGATGGAGTATAGAAATCTTATATCTTGGAATTCGATGATTTCAGGTTTTCAATTCCGT

GGACTTGGCGACAAAGCTATTGGTCTGTTTGCGCATATGTATTGCAATGGAATTGGGTTT

AACAGTACCACATTACTCGGTGTTTTATCTTCTTTGAATCATTGCACGAGTACTTGGGAT

GACATCAACAACTCACATCATCTGAAGAACTGTTTTCAATTACACTGTCTCACAGTTAAA

AGTGGTCTTATTTCAGAAGTTGAAGTGGTAACTGCATTGGTAAAATCCTATGCAGACCTA

GGGGGTCACATTTCTGACTGTTTTAAGCTCTTCCTTGATACAAGTGGCGAACGTGATATT

GTTTCATGGACTGCTATTATTTCTGTGTTTGCGGAGCACGATCCTGAGCAAGCTTTCCTT

CTTTTCTGTCAACTTCATCGAGAAAATTTTGTGTTGGACCGGCACACATTCTCAATTGCC

TTAAAAGCTTGCGCTTACTTTGTCACTGAGAAAAATGCAACCGAAGTTAACTCCCAAGTA

ATTAAACAAGGGTTTCACGATGACACAGTTGTTTCAAATGCCTTGATACATGCTTATGGG

GGAGGTCTGGCTCCTTAG

>MS.gene010014.t1

ATGAAAGGGTGTACTTTTGTTACTAATGTTCATGATTTCACCTTTTCTCCTTCTTTGATT

TGTTCCAACTCGTTTATTCCATTACAAAGTAATATTTTTCCTAGTAATGTCACATTGAAA

CCATTGAGGGCAAAGGTGGGCATTTCTCATAAACAAAAAGGTAAGAGTGAATTTGTTGTG

GGATGTGCTAGTTGGGGATTGGTGAATGAGTTGAAGAGAGAACTTGAAGAAGGGGAAACT

GATGATGAAAAGGGTAAAAGTGGAATGACAAGGTATAGAGAAAAATGTGGAGAAAGGGAA

GGGGTTGTGGAGTTTTTGGAGTGTTTGGAAAAGGAAGCAATAATGGGTGATGATGAAGGG

AAAGAGCCTAATGATTACAATCGAAGGGCTCAGATATTTGATAAAAGCTCTGAAGTATTT

CAAGCACTTAAGGAATCAAATGATCATGTTTGA

>MS.gene010021.t1

ATGGAAGAAGTCATGAATTTCATCATGCGTGCTTTTGAATTAGCAAAAAATCTTGAACTA

GAACTACCCAACATGGCCAACCACCCTGAAATGCTATTCTCATCCATTGATGATGTAGAA

AAAGCATTTAATGCTGCCAAAGAAAGAGTAATGATGATGTTGTCATCACAACATGACACA

ACAACAACAACAACAAAACCTTCTAATTCCTTTGCTCAAATTCTGTCTCATGATGTTTTC

ATGCAAAAAACTCAACATGCTCAAATTGGTGGAAGTGGAAGTGCAATTTCAATGCATGCA

CATTCAATGGATCAATTGCTTCTAATGCAACACCCTTTTGATGTGAGTGTTCTAATTGAG

AATAAGATGATAAGTGGCGGTGGTGATGGCGGTGACGTTCAGTTGTTAAGGTCTAGGGAA

ACTTTGAGGATTGGTGAAATGGGTGGGAGGGATATGGAAGGTTTAGATAGATCAAAAGGG

TTAGAAGGAGATCAAATGCAAGCAATAGAAACACCACCTTCAAGACCAAAAAAATGCAGC

AGGAAGAATGATTTGGAGAAGAGAACAGAGATGCATCCAGCACCTCAGATTGGAAACACT

GAGATGCCACCAGAAGATGGCTTTACTTGGAGAAAATATGGCCAAAAAGAAATACTTGGC

CGCAAGCACCCAAGAAGTTACTATAGGTGCACACACCAAAAATTATATTTGTGTCCAGCC

AAGAAGCAAGTGCAAAGACTTGATGAAAACCCTAACATAGTTGAAGTAACATATAGAGGT

GAACACATTTGTCACAAATCCTTAACAGCACCATCATCATATCCACCACCACCAAACCTA

CTTGTGAACATTAGTACCATTGAAACTGCCATTTCTTCCCAAGGGTGGCCACCTGCGGTG

AACCACGACCTCAGGGGCGGAGACGGTACTGGTGGACCCTCTACTTCAAGATTCGGCATT

GATTATCCGGTGGCCGATATGGCCGATGTAATGTTCAACTCGGGTAGTAGCAGTGGCAAT

AGTATGGAATCACTCTTTTATCTAACTGAAGATAAAGGTGAACCTGATGAGGAGAAAAAC

TGA

>MS.gene010029.t1

ATGGATGATATACGAATCTTTAAATGTCAAAACTGTCTCTTTGTGGGTTCTGTTGACGAT

TCTGATGATGGCTTCTTCTACTGCCCTAAGTGTAATGTTCGGTTTGATGGTGTTTTTGAC

AATGATGTTGATGATGTGATTGATGTCTACACTCTCAGATTCCGACGCAAGCAATCTGCT

GCAATCAAAGCCGAAGAAAACTCTCAACCTTGCTCTGAATATGATGCCTGGTCCAAATTT

GTTAATAATCTCTTAGAAGATGAAACTCAACAGAATGATGATATTCATGTAAAAGAACAG

AATTTTGATGGGACAGGTGTCTCACATCCCAGAGATTTTGGAAGCTCTAAACTTAAAACC

TCAAAATACTACCACGACCACAACCAGATAAGGATGCGGTACGTGATGGGGTTGCAGATG

ATTATTGAGCTTCAATGTGAAGCACTAGTGAAGGAGTTTAAGGTTACTCCTTTAATATGT

GGCTTGGTTGGACCAATATGGTTGAGGTTTATCTCCAAGACTGGTGTTTTTGATGATGAC

CGGGCTTATACAGTAATGTGCAACTCTGAGATGCAGAAAGAAGGAGAACCAGAAGATTAT

AACATACGTGGCAAATACAGAGCAGAACCCCACAACATGTTTGGTCAACGATTTGTAATG

ATATGGTTTAGGTATTTGAAGAACAGGATTCCTCTAGCTTGTACAATTGCTGTTTCATAT

CTGGCATGCCATATTGCTAGGGAAGCCATCCTACCATCAGACATGATGAAGTGGACACTC

GAAGGGAAGCTTCCATATTTTTCTGCCTTTGTTGAACTCGAAAGGCTCATGAAACATGAA

TGGCTTGCATGTCCCATCAGTTCAAGTGTTATGTTCAAGCCCCAGCGAGCTATACCGGTA

CAAAAGCTAGAATCATTTGCTGCATCAATTGCTCATTGCATAGGCTTGGAGTTACCTCCT

GTGAACTTTCATGCAATAGCATATCGTTACCTTAAAAATTTATCTCTTCCTGTCGAAAAG

ATTCTTCCTTATGCATGCCGTATTTATGAATGGTCGATGCCTCCAGACTCGTGGTTATCT

TTTACCAAGGATTATTTTAGGTTACCTACTCATGTTTGTGTTGTATCAATTCTGGTGATA

GCTATAAGGATTTTGTATAACATAAATGGTTATGGAGAATGGGAGAAAAGTTTATCTCAT

AATGTTGGTGCCAAAGACAATGGCGAAATGGGTACCACTTTTGCCTCTCATGACGGGCAT

GTTTTTGGTAAAGATTCTGCCAACGATCCAATAGGATGTCAAAAACACAAAATGGATTCT

GCTGTGCTTCTCCAACACCTTCATGCAATATATAATGAGATTGCAGATATGCACGAGTAT

TCAAAGGACTTACCTACTTATCTCAAATATTGTAGAGATGTTGTCTTTGATGGATCAAAA

GAACGATCTTATGACAATTATGAGGAACAAAGAATGTTTGAAATCCTATGGAATTTTTAT

CAGAATGAAGAGAATACCACCAAACCATCAGAACATGCAGGGAAATCCAATACTGCTTTT

AACCGATCGAGGTTGACGGATGAAGGGCGCATTAGGAGGACATCTAAGATGAAAAAGGTA

AGAAGAGAATGTTTCAATGAGCCCTCTCATGACGATGGCAACTGCCATGTAGATGACTTT

CCTGGAAGTTTGAACAATGATAATTCCTCTGGGACATCGCCATATGGTGAAGACTCAGTT

TCCAACAAGAACGGCGGTTCAGGAAAATCTGTGGTCAATGAAGCCATTACACAAATAAAA

TTAGACATGGAGGAAAACCTATTTTGTTATGTACCACCTCGTGTCAAGCCGAAGAACCTT

GATCATGTTCACTATGTCAGGAAGAAGGATAAAGGTGCCATGACTTATGTTGCTCATGCT

GATTATTACATTTTGCTTCGGGCTTGTGCCAGAATTGCACAAGTTGACATTAGGATTTTG

CACATCGGTGTTTTGAGACTTGAGAAAAGACTTGCTTGGCTGGAGAAAAGGGTTGATCAA

TGCTTGCATTTGAAACCTTCCAGTATTTCTCGTCAGTTTTGTAGTGTTAAGACCACTGAA

AATGTTTCAGAAGATGTACCTTAA

>MS.gene010017.t1

ATGGAGGAATTAATTAAGAAGATTGACACACCTCCTCCTGCATGTTTTAATGAGAGTGTG

TTAAATCCCAATCAAATATGGAAAACTGGCAATGTTATGAAAACTGGGATCCCCCCTATT

GCCGCAACAATTGTCATGGTCGTATTTTTGTCCCGTCTTTTATTCTTTATCTATAAACCC

CTTCATCAACCTCGCATCATTTCACATATCACTGCTGGTTTTTTAATACAAAACCTACAG

CATTTCTTCCCGTCTTCTTTCAGCTACCTTTTCCCTGTAACTAATATGATCAACATTGAA

GTTCTATCAAATATTGGTGTCATATACTATGCATTCCTAAGTGGTTTAGAGATGAACTTG

AATACCATTTTACATGTTAAAAAGAAATCTGCAACTATTGCAATTTTTGGAATCATTTTT

CCAATGGTGATGGGACCGGCTTTATACCTTCTACATCGAAATTTTTATGGAAAGCGTGGT

GGATATAAACTTGAAGAAAATACAACAAATGCTTGTGTAATATGGACTTTAGTTCTCACT

GTGACAGGTTTTCCAGTTATAGCTCACACCCTTTCTGAGCTCAAACTTCTTTATACTGGT

CTTGGTAAGGTTGCATTAACAACAGCTATGATCAGTGACACATATGCTTGGATTTTATTC

ATATTATTTGTTCCATTTTCAGTTAATGGTACAAATGCAATATACCCAGTGTTAAGCACT

GTAGTATTTGTTTTCATTTGCATCTTTGTGGTGCATCCTATAATTGTAAAGGTCATTAAT

CGCAAGACAGAAAGAGATGAATGGGATGGTAACCAATTAGTCTTTGTGGTGATGGGACTT

TTTGTTTTCTCATACATAACAGATATTCTTGGCACACATGACGTTGTTGGTGCATTTGTG

TACGGGTTGATTTTACCTCACGGTAAATTTGCTGACATGGTCACATCAATGACTAATGAT

TTTGGTGGTGGGTTTCTAGCACCGATTTACTTTATTGGCAGTGGATTGAAACTCATGTTC

GTGCCCGGTTTCGATAAACCAAATTGGCTTACACTTGTCATTATACTCTTGTTATGTGTT

CTAAAGATTTTAAGCACTTTGTTTTCCACTTTCTTGTTTGGCATGCGTACTCGAGATGGT

TTTGCTTTAGGCTTGCTTCTCAATACCAAGGGTGTCGTTGCATTAATAATGCTCAACATT

TCATGGGATAGAATGATTATTACTCCACCAACCTATGTTGTTTTAACATCAGCAGTTATT

TTAATGACTATAGTGGTAGCTCCCGTCATAAATGCCATATACAAGCCCAAAAAGAGATAT

GAGCAGAACAAGCTAAAGACTATACAAAAGCTCAGACTTGATGCAGAGCTTAGGATTCTA

GCATGTGTTCATAATACTCGTCAGGCTGTAGGGGTTATTAGCCTCATTGAATCTTTCAAT

GCCACTAGACTTTCCCCTATGCATATTTTCGCACTTTACCTTGTTGAACTCGTTGGACGT

GATGGTGCACTTGTTGCTTCTCATATGGAGAAGCCTAATGGCCAATCTGGAACACAAAAT

CTTACGAGATCCCAGATAGAGTTAGAAAGCATTAATAATAAATTTGAAGCACTCATAGAG

GCATATGATGCTATCAGAGTCCAAACGTTAAATGTTGTGTCCGAATATGCAACCATTCAC

GAAGACATATACAATTTAGTTAATGAAAAACATACAAGCTTGATTCTCCTTCCATTCCAT

AAACAACTAAGTTCGGAAGGTGCATTTGAAACAATTAATGTTGCGTTCAAAGATATAAAC

TTGAATGTGATGCAAAGTGCACCTTGCTCTGTGGGATTATTTGTTGATCGTAATCTTGGG

TCATTACCAAAAATGAATTTTCGTATTTGTATGATCTTTGTTGGAGGTCCTGATGATCGT

GAAGCATTAGCTGTTGCATGGAGGATGACAGGACACTTAGGAACCCAACTATTAGTGGTT

CGAATGCTCCTGTTTGATAAAGCAGCAATAGTGGACACCACAAGTCATGATGAAGCAAGG

GGCATATTATCTATTGTAATGGATAGTGAGAAGCAAAAGGAATTAGATGATGAGTATGTG

AATGGATTTAGATTGACAACGATGAACAACAATGATTCTATATCTTATTCAGAAGTTGAT

GTCCATTCTGCTGAAGATATTCCCACATCCCTCAATGAGATAGAAAAAATTGGTTGCGAT

TTATACATAGTTGGACAAGGAAATCGTAGGAACACTCGAGTCTTTTTAAACTTATTGGAA

TGGTGTGACTGCCCAGAACTTGGGGTTTTAGGGGATATTTTAGCATCAAACAATTTTGGT

TCGGGCTCATCAGTGCTAGTTGTGCAACAATATGGGTATGGAGGAATGGTTTTGGGAAAG

CAACCAAACCAGGTGACCGGCAATAATGATGCTTTTGAGGTGTTGTAG

>MS.gene010030.t1

ATGGCTGTTTCAACTAGTTTCTATGGTGCTAAATTGGAACCTTTGTTCCTTAAATGTTGT

TCTTCTTCTTCAACATCATCTTCCTCTTATGTTACTACTCATTTATCATTTTTTGGTAGT

AACAAAAAGAGCTTTGTTCAAAGTAGAGGTTCTATTCGTTGTGATGCTTCATCTTCTGAT

GTTTTGGTTGATCCTAGTGATAATGCTAAATCTGTTTCTGCTCTACAACAGCTTAAAGCT

TCTGCAGCTGATAGATATACAAAGGAAAGGAGCAGCATTGTCGTTATCGGGCTAAGTATT

CATACTACACCAGTCGATGTGCGCGAAAAACTTGCCATACCAGAAGCAGAATGGCCTAGA

GCCATTGGTGAGCTTTGTAATCTGAATCATATTGAAGAAGCAGCAGTTCTGAGCACCTGC

AACCGAATGGAGATATATGTTGTCGCACTTTCTCAGCACCGTGGTGTCAAAGAAGTCACT

GAATGGATGTCCAATACAAGTGGTATACCTGCATCAGAGCTGTCAAAGTATCTATTTTTG

CTATACAACAAGGATGCAACACAACACCTTTTTGAAGTATCAGCAGGTCTTGATTCTCTT

GTAATGGGAGAAGGTCAAATCCTTGCACAAGTCAAACAAGTTGTGAAAGCAGGACAAGGA

GTTAATGGCTTTGGTAGAAACATCAGTGGTCTATTCAAGCACGCAATCACCGTCGGTAAA

AGGGTCCGAACTGAGACTAACATTGCAGCTGGTGCAGTTTCTGTTAGCTCGGCTGCAGTT

GAATTAGCCTTGATGAAGTTACCTGATCAAGCCTCACAAGGCAATGCAAGAATGCTAGTT

ATTGGAGCTGGCAAAATGGGGAAGCTTGTGATCAAACATTTGGTTGCAAAAGGGTGTAAA

AAGATGGTAGTTGTTAATCGAACTGAAGGGAGAGTTGCAGCAATCCGTGAAGAAATAAAT

GATGTTGAGATAATTTACAAACCCCTTTCGGAAATGCTCGAATGTATTGGTGAAGCAGAT

GTTGTTTTCACAAGCACTGCATCAGAGAATCCTTTGATTTTGAAACAAAATGTTAAGGAA

CTTCCTTTAGCAAGCGAAGAAATGGGAGGGAAACGCCTCTTCGTTGATATTTCTGTTCCT

AGAAACGTAGGTTCATGTGTCGATGATCTTGAGTCTGTTAAGGTTTACAATGTTGATGAT

CTTAAAGAGGTTGTGGCTGCCAACAAAGAGGACAGGCTAAGAAAAGCAATGGAAGCACAG

GTGATCATCGGCGAAGAATCGGGACAATTTGAAGCTTGGAGAGACTCATTAGAAACTGTT

CCTACCATTAAGAAATTAAGGGCTTATGCTGAAAGATTAAGGGTTGCTGAGCTTGAGAAA

TGTTTAGGTAAAATGGGTGATGATATAAATAAGAAGACACAAAAAGCTGTGGATGATCTT

AGTAAAGGGATTGTGAATAAAATGCTTCATGGTCCAATGCAACATTTGAGATGTGATGGG

AGTGATAGTAGGACTTTGAGTGAGACACTTGAGAATATGCATGCTTTGAACAGAATGTTT

AGTCTTGAAACTGAGGTTTCTGTTTTGGAGCAGAAGATTAGAGCTAAGGTGGAGCAAAAA

CCACAGTAA

>MS.gene010023.t1

ATGGCTTCTTCTGTAGTGTTGGAAGCACCATCATCACATTCACATTTAACAACAAACCCA

TTATTCTCTTCTTCTTCAAAATTTCATTATCTTAACAATAATGCTTCTAGATTCTACGCT

TTCCCTTCAAAATCCAGAGTTTTTCTCACCCTTACTTCCACCCCTAATCATATAATCAGA

CATGCTTCCGGAATTGATTCTTCTTCTACGGTTCAAGAACCTGAACCGGTTATGGAATTG

GGTCTTGACCCGATTCGAGATAGGAGACGGGTTGTGAGGGTTGCGTGGGAGAAACTGGTG

GTAGTGCTTGGAGGGGGATCATTTGGTACAGCAATGGCTGCTCATGTTGCCAATAGAAAG

GATCAGTTAGAAGTCGTGATGCTTGTTCGAGATCCCGAAGTTTGCTCCTCTATCAATGAG

AGCCACTGCAATCGTAACTACTTCCCGGATCACACACTGCCGGAAAATGTAGTCGCAACA

ACTGATGCAAAATCGGCTTTGCGTGATGCGGATTACTGCTTGCATGCTGTGCCTGTTCAG

TTCAGCGCAGCATTTCTTGAAAGTGTCGCTGATTATGTTGACCCAGGCTTGCCATTCATA

TCTCTCAGTAAAGGCCTGGAGCTTAATACACTGAGGATGATGGCTCAAATTATTCCTCAA

GCACTGCGAAACTCTCGCCAGCCTTTTGTTGCACTGTCCGGGCCTTCTTTTGCTCTGGAA

TTAATGAATAAGCTACCTACAGCAATGGTCGTAGCATCAAAAGACAAAAAATTGGCAAAT

GCAGTTCAGCAGCTACTAGCTTCAAATCATTTAAGAATCAGCACATCCAGTGATGTTACA

GGAGTTGAAATAGCAGGTGCCCTTAAGAATGTGCTTGCAATAGCAGCTGGGATTGTAGAA

GGTATGAATCTTGGTAACAACTCAATGGCTGCTCTTGTCACGCAGGGTTGTTCTGAAATA

CGGTGGCTTGCGACAAAGATGGGTGCAAAGCCAACTACAATAACTGGTCTGTCTGGAACT

GGAGACATAATGCTGACATGTTTTGTCAGCCTTTCGAGAAATAGAACTGTTGGTGTGCGT

CTTGGATCAGGCGAGAAGCTTGAGGACATACTTAATTCCATGAATCAGGTAGCAGAAGGT

GTCTCAACGGCTGGAGCTGTGATTGCTTTGGCGCAAAAATATAATGTAAAGATGCCGGTA

TTGACAGCAGTCGCTCGTATTATTGACAATGAACTTACTCCCAAGAAAGCTGTTTACGAG

TTAATGAGCCTTCCTCAGGTTGAAGAAGTATGA

>MS.gene010026.t1

ATGAGGAATCGATTGGTGCAAATTTCAGACTTTAGAGAAGTTGTGTCGTTATGGAAGTAT

CTTGGTGTTCCCTTGACTGAGAAAGCGCCTCGTAAATCAGATTTTCAATTTGTTTTAGAT

CAAGTTAACAGAAAACTTACTGCTTGGAAAGCCAAAAATTTATCTTTTGCCGGTAGAGTG

ACTCTAGCAAAAAGTTTGATTGAAGGTATCCCTATGTCCTATGATGACTGCCATGATGCC

GAAGAGTTGTTTGGAAGAGATTGA

>MS.gene010020.t1

ATGGCTGCAGAAGATGACAATTTTGAAGATGAAGCCAATGACTTTCCTGTACCAAATGCA

GGAGTTGTTTGGGAGGAAATTGTAGAGGATGTGGAAAATGTGGGTGGTGAAGTGCTGGAG

GGGGTTCCTATGATTGAATACCCAGTAGGAAATGTGGGAGAAAAAGGGCTGAATGTTCTT

ATAATTGAATACCCTGTAGGTGACAGTGAGGGAAATGTTGTGGATGACGGAAATGCTGGT

GACATGGTGGTAAATGAGGGAAATGCTGAGAATATGTTGGAAAATGAGTGGAATGAGGGT

AATGTGGATAAGCCCACTGAGTTTGGTGAGGGATTAAATAATGACATAAATATTGATGGC

CCATTTGTGGTTGACAAAACTGGCGAGGAGGAAACTACAACTGGTCTAGAGGAAGAATTG

AGGGATGTGCTTGATATTAACTTAGAAGATGACAATGATGCTTTCTGGCTAGGTGAGGAA

GGTGAAGATAATGCTCTAGACATTACCTTTGGAGATTCAGAGGAAGAGGTGGGGGATGAA

GATAATTTTGGAGTTGTAGTTGGTGAAGAAATAGAGAAAGGAGGTGAGGTTTTAGCAGGT

CCATCAACTGTTGGTGAATCAACTCAGGAGAAGCAAATCAGCCACATACAAATGAAGGGA

ATCAGAGTGAAGTACAAGCTAGTCAGCCAGAAACAAATCAAGGGAATCAGGGTTAAGTAG

>MS.gene010019.t1

ATGATGTCTGATGCTGACACTTTAGTATCAGAAGAACAAACTCTTGTTGAATCAACAATC

ATTGTAGCCTTACATGCTTTTGTTTCAGAAGCTACAATTGATCCTCAACCTATTAGTGTA

GCCCTACCATCTTCAGAAGCTACACTTAACACTCTTGAACTCCCCACTGAATCGGACATC

ATAATCACAACAGATTCAGATGTTGAAGATGAGCAACCTATTCAAACGGGTACTACCTTA

GAATCATCTTCACCATTTGTTTTAGAGTATGTTCATGATGCACCCTTTGTTCCTCCAAAC

CCATCTGTTGTTATTGAAACTTCAAGCACATATGCACCTTCTTCCTCAACAATCATTAAT

CCACAACTTACAAATGTCTCACCCCCACCTACCCTTTTGTTAGAATCTGATATATTAAAG

GAGGTATGTGAGAACATCTTCAAGGACTTGAACAAACTGGTGCATAACAGAAGCAACTTT

GTTCATAAGGAGAATTATGCAGATGAATGGACTGCTCTTAGAGAAAGAGTTGATTATGTG

ATGTGTGAACTTCAGAAGTTATCTCTAGAAGCACATAATCAGGCTTTGAACACCCTCAAG

GACTGGTTCAAAGAAGTTGTTAAGAGTATGGAAGAAGTATATGTTAACATGAATCAGAAG

AAAAGCAAGCTGTATATCTCAAACCCTCTTGTCTACATGGATGCTTCTAGTATCATCTTA

TCAAGTGTCCATTCAGAGAACCCTGACCTGGTTAACAAAGTTGAAGATTCAAGCTGA

>MS.gene010028.t1

ATGTGCAATCCAAAGCCATCTTCACCAACTTCACCATTTCTTTCTCCAACAAAAACTCAC

CTCATAAATCCTCACACCAAAGATTCTTATTCAAATCCTCCTACCCTTGATGATGATCAT

GTTCAAGATGAAATTCATAGCTGGCCTACTCTTAAAGAGGCCATAACAGAAATCAAAGAA

ATAGGAAAAATATCAGGTCCAACAACAATAACTGGTTTATTGTTATATTCAAGAGCTATG

ATATCAATGATTTTTCTTGGATATCTTGGAGAGATGGAACTAGCAGGAGGTTCACTTTCA

ATAGGCTTTGCAAATATCACTGGTTACTCAGTGATATCAGGATTAGCCATGGGAATGGAA

CCAATTTGTGGACAAGCTTATGGAGCAAAACAATGGAAGATACTTGGCTTAACACTTCAA

AGAACAGTGCTTCTTCTTCTTTCAACTTCAATCCCTATCTCATTCATTTGGATCAATATG

AAAAGAATCCTATTGTTTTCAGGTCAAGATGAAGAAATTTCATCAATGGCTCAAAGACTT

GGAGCAATGCTTTTCACAACATTGATGAGAAACCAATGGGGAAAGTTTTTCACAAATGAC

AAAGAAATTCTTGAGTTAACATCTATTGTGTTACCAATTGTTGGACTTTGCGAGCTCGGA

AATTGTCCACAAACAACAGGTTGTGGTGTCCTTAGGGGAAGTGCAAGACCAACAATTGGA

GCTAATATAAATTTAGGATCTTTTTACCTTGTTGGTATGCCAGTTGCAATCCTTTTGGGA

TTTGTGGCTAAATTGGGGTTTCCAGGGTTGTGGATTGGGTTACTTGCAGCTCAAGGCTCA

TGTGCTATGCTTATGTTGGTTGTTCTTTGTAGAACTGATTGGAATTTGCAAGTTCAAAGA

GCTAAAGAACTCACAAAAAGTTCAACTAGTGATGATGTTGATGCTAAATTACCTACATTT

ATGGAAGGTAATGTGAACAAGAATAATGTTCATGGTTGTCTTGAAGAAATTGTTATCACT

CATGATGTGTTTACTAAGAAATCTTCACTTGAAACAGATCCACTTATCATAACATCTACT

ACTACCAATTGCATTGAAGATTAG

>MS.gene010024.t1

ATGGATCCTCACCATCATTTGGCTTTTATTTTTGGAATACTAGGTAACATCATTTCCTGC

ATGGTGTACTTGGCACCCCTGCCAACATTTTACCGAATATGGAAAAAGAAGTCGACGGAA

GGATTCCAATCGTTGCCTTACTTGGTGGCTCTATTCAGCTCCATGTTATGGTTATATTAT

GGTTTAGTCAAGAAAGATGCTTTTTTACTCATTACCATTAATTCATTTGGGTGTGTCGTT

GAAACAATATATATTGTCACATACCTAATCTACGCCACCAAAGATGCCAGGATCTTGACG

ATTAAACTGTTCATGGCGATGAATGTGGGCTCTTTTGCGATGATTCTCCTTACCACCTTG

TTAGCTATGCATGGTTCTCTCCGTGTGATAGTCCTTGGATGGATATGCACATCTTTTGCG

ATTTGTGTTTTTGCATCACCTTTAACCATTATGGCAAAAGTTATTCGAACAAAGAGTGTT

GAGTTTATGCCATTCAACCTATCATTTTTCCTCACACTAAGTGCCATTGTGTGGTTTTTT

TATGGTCTCTTAATGAGGGATATATGCATTGCTATTCCAAACGTGTTGGGTTTCATCTTG

GGGCTACTTCAAATGTTACTATATGCTATTTACAACAAAGGTGTAAAGGAGGAGTATGCC

CTAGAGCCAATGACAAACATTGTTATTGTGAATCCTTTGGGAATTCCGTGTGAAGTATTC

TCTCTCCCAGTTATAGATAACGTGAATAAAATTGAAAAAGAAGGTGCAGAAGAGATGGAG

AAAAGTGTGGAAAACCCAACATGA

>MS.gene010025.t1

ATGAGGAATCGATTGGTGCAAATTTCAGACTTTAGAGAAGTTGTGTCGTTATGGAAGTAT

CTTGGTGTTCCCTTGACTGAGAAAGCGCCTCGTAAATCAGATTTTCAATTTGTTTTAGAT

CAAGTTAACAGAAAACTTACTGCTTGGAAAGCCAAAAATTTATCTTTTGCCGGTAGAGTG

ACTCTAGCAAAAAGTTTGATTGAAGGTATCCCTATGTCCTATGATGACTGCCATGATGCC

GAAGAGTTGTTTGGAAGAGATTGA

>MS.gene010012.t1

ATGGGTTGCCGCGATTTGGTTTCTTCGAATTCAATGCTCAAGTCTTATGCCATTCACGGT

CGAGCTAAAGATGCACTAGAGCTCTTTAAGCGAATGGATGTTCATCCAGATTCTGCAACC

TTTGTTGCTCTTCTCGCGGCATGCAGTCACGCTGGACTTGTTGATGAAGGCATGAAATAA

>MS.gene010016.t1

ATGGATAATAATATCCCTGAAAGTGTTAGAAAGAAAGTGATAATCAAAGAACTTGTTAAG

GGTCAAGAAGCTGCAACCAAACTCAAGATTCTTCTTCAGAACGAGAATCCCTATGGAGCT

GATCATCTTGCTGCAAACGTGCTGAGATCTTTCACTGAGGCTCTTTCTATTATCTCTCAA

TCAAACTCAAGTTGTGATGATTTTCTGAATCTGATTAAATCTGCAGATTCAATTAGTGAA

AGTAGAAAGAAGGGAAGAAGAGGTTGCTACAAAAGAAGGAAGAGTGCAGCTGAGATATGG

ACCATAGTTTCCCAAACCATTGTTGATAATCATTCATGGAGAAAGTATGGGCAGAAGAAA

ATCATGGATTCTGAATTCCCCAGCATGATTCTGTAA

>MS.gene010022.t1

ATGTCTTACACCGATCACGGCGGCGATCGCCGTCACCGTCGCCGGAACCACCCCTCTTCA

ATTTCCGCCGTTTTCCTCTACCTCACTTTCCATTCAATCATCACCCTCGTCGATGCTTCC

ATTCACGACTACCAAAACGAAACGTTCATCCGTCGTGCTAATTCGTTTTTCTTTCATGGT

GGTAGTGAAGGTCTTTACGCTTCTAAACCTATTGAGTTTAACCATTCTCTGGATAACTTT

CCCACTGGTAAATCTTTCATCAGGTTTGAGTCTATCAACTTTCGAAGGACAAAAGAATCT

GCTCAAAAGGCAAGTTCGATGCAACAAAAGACAGGGTTGGTTGAAGCTATAATAATTAAG

GTAAGAGACAGGAATAACATTGGGGGTGTTTATTTGAACTCTGATGCTATATGCTGCACA

CCAGAGCTTGCTAAAGAAGGTTCTTGTAAGTTAGGAGAGGTTATCATCCGTGAGAACCCT

GATGAACCGAACGGTCCGAAACGCTTGCAAACGTTTTTTGAAGGACAAAATGAAGAGACT

AACATGGTCATTCAAACTGTTGATATTAATAGTACTGGGATGTATTATTTGTATTTCATG

TTTTGTGATCCTGAACTGAAGGACACGGTGATTTCCGGAAGAACTGTGTGGAGAAATCCG

GATGGTTATCTACCCGGGAAGATGATGCCATTGATGACTTTTTATGGTTTGATGTCTTTG

GCTTATCTTTTTCTTGGTCTTGTTTGGTTTCTCTGGTTTGTAAAATATTGGAAAGATGTT

ATACAGTTGCATTACCACATTACTGCTGTTATTGGCCTAGGCATGTGTGAAATGGCTCTG

TGGTATTTTGAGTATGCAAATTTCAATTCGACGGGAAGCAGGCCAATGGTAATTACAGTA

TGGGCCGTGACTTTCACTGCTGTCAAGAAGACAGTGTCACGGCTTCTTCTTTTGGTGGTT

TCTATGGGCTATGGAGTAGTCCGTCCAACACTTGGTGGTCTAACCTCAAAAGTCCTTCTT

TTTGGTGTGGTGTATTTTGTGGCCTCGGAAGCACTTGAACTGGTGGAACATCTCGGTAAT

ATTAATGACTTCTCTGGAAAAACAAGGCTTTTCCTGGTGCTGCCTGTGGCTCTGTTGGAT

GCTTGCTTTATTCTTTGGATCTTTTCATCATTATCTAAAACCCTTGAGAAGCTTCAGATT

AGGAAAAGTACTGGAAAACTTGAGTTGTACAGGAAGTTTACAAATTCTCTTGCAGTGACG

GTGCTGCTTTCAGTATTATGGATTGGCTATGAGTTGTACTTCAATGCATCAGACCCTTTA

GGGGAACTGTGGCGAAGAGCATGGGCCATCCCAGCTTTCTGGACTTTGCTTGCTTATGCT

CTCCTGATAGTGATCTGCATTCTCTGGGCCCCATCTCGAAATCCAACCAGGTATTCGTAC

TCGGAAGAGACGGGAGATGATTTTGATGAGGAAGCTGTCGCAGTTGTTGGAAGTGGAGTT

AAGATGTCTGGAGAAATGTCTACCATGCTAGAAAGAAAGGATAGAAAGGCATCGTCTACA

TCACTTGCTACAGATCATCATGTATTTGGAGTTATTGAAGATCTAGAGGAAGACAAGAGG

GAGTGA

>MS.gene010036.t1

ATGTCTCTACCAAGGCTAGGAATAGGTGATGAAGAAAGTAAAAACAATGTTACTTTGTTG

GAAAAAAGTGTGTGTTTGAATGGTTCAAAACCAAAAGAGTTCAACTACATGGGATTATCA

TCCTCTAATTGTTCATCAGTGGATAGTTCAGTTCCAAAAATTCAATCTTTTAAAGATGAG

ACCAAAAGTAATTTGAATCTTAAAGCTACTGAACTTAGATTGGGACTTCCAGGATCACTT

TCTCCTGAAAGAGATTCATCAGATTTTTGTTTAAGAAGTTCAAAGCAGTTTGATGAAAAG

CCTCTTTTTCCTTTGCACCCTCAAAAAGATGATCATCTTTTTGAATCAAAGCCTGCTGTT

TTAGGTAACAAAAGAGGGTTTTCTGATGCCATGAATGTTTTCTCAGAGGGGAAATTGAAG

CCTAGCTCCAAAATGCTTGAGAATGTTGCCGGGCAGAAAGTGAAAGCCGATGAAATAGCA

ACAGTTAAGATTGGTCTTGAGAGGCCTAATGGTGTCGGTGAAAGCAAACCAAGTCTTAAT

GGTTCTGCAACTAATGGAAATAGCACTGCTCCTGCTAGCAAGGCACAGGTTGTTGGTTGG

CCTCCAATAAGATCGTTTAGGAAAAATTCATTGACCACTGCTTCAAAGAACAATGAAGAG

GTTGATGGAAAATTGGGATCAGGAGGTGCTGTGTTTGTGAAGGTCAGCATGGATGGTGCT

CCGTATTTGAGAAAAGTAGACTTGAAGAATTACACTGCATATTCACAACTATCTTCTTCT

CTTGAGAAGATGTTCAGCTGTTTCACCATAGGTCAATGTGAATCTCATGGAAATCAGATG

CTGAATGAAACCAAGCTGAGGGATCTGCTTCATGGTTCAGAATATGTTATTACTTATGAG

GATAAAGATGGTGATTGGATGCTTGTGGGTGATGTTCCCTGGGAGATGTTTATTGATACA

TGCAGGAGGCTAAGAATCATGAAGAGCTCTGATGCCATTGGTTTAGCTCCCAGAGCAGTT

GAAAAAAGTAAAAGCAGGAACTAA

>MS.gene010035.t1

GCACCGGTGCATGGGAGAGTGCATTGACAAATACATTGTCTCCCGGAGATCGTACTGTAT

CTTTTCTAATGGGCCAATTCAGCTTGCTTTGGATTGAACAACAAAAACGCCTCAACTTTA

AGGTTGATGTTGTAGAAAGTGAATGGGGTCAGGGTGCTGATCTTGATGCTCTAGAATCAA

AACTTGCTTCAGATAAAACACATACCATAAAGGCAATTTGTATTGTTCACAATGAGACAA

CAACAGGGGTCACAAATGACTTGACTAAAGTGAGAAAAATCCTTGACAAGTACCAGCACC

CAGCACTCATTATTGTTGATGCTGTGTCTTCCATTGGTGCTCTTGATTTTCGCATGGATG

AATGGGGTGTAGACGTGGTAGTGACTAGCTCTCAGAAAGCTCTCTCTCTTCCTACAGGAA

TGGGAATTGTTTGTGCAGGACCTAAAGCCATTGAGGCTTCAAAATCTGCTACATCACTTA

GATCTTTCTTTGATTGGAATGGTTACTTGAAATGCTACAACTTGGGAACTTATTGGCCAT

ACACTCCTTCCATTCAGTTGCTCTATGGTCTAAGAGCAGCCCTTGACTTGGTTTTTGAGG

AAGGATTTGAAAATGTGATTTTAAGGCACAAGCATTTAGCCAAAGCAACCAGGCTTGCAG

TAGAGGCATGGGGATTGAAGAATTGTTGCCAAAAGGAGGAGTGGTATAGTGCTAGTGTGA

CTGCTATTGTTGTTCCTCCTAACATTGATAGTGGTGAAATAGTTAGTAGGGCATGGAAGA

GATACAATTTAAGCTTAGGAGGTGGATTGAACAAAGTTGCTGGGAAAGTTTTCAGAATAG

GACATGTTGGCCATCTAAATGAGTTGCAACTTTTGGGTTGTCTTGCTGGTGTGGAGATGA

CACTCAAAGATGTGGGTTACCCTGTCCAGCTCGGAAGTGGAGTTGCTGCTGCAAGTGCTT

ATTTACTAAACAATGTTCCTATAATCCCTTCAAGAATTTGA

>MS.gene010038.t1

CTCTTGAACAATGGAAATTTAGTTCTCAGAGATGAGAAGGATAACAATGAAGAAAGTTTT

TTGTGGCAAGGCTTTGACCATCCTTGTGATACACTATTACCTGGAATGACGTTTGGATAT

AATCGAAAATTGGATTTTTATTGGAATCTCACTGCTTGGAAGAATGAAGACGATCCATCT

TCGGGAGATTTATATGCATCTGTGGTGCTTACAAGCAATCCAGAAAGCATGATATGGAAG

GGCTCGACTAAAATCTGTAGGTCAGGACCATGGAATCCTCTCTCTAGTGGAGTTGTCGGA

ATGAAACCTAACCCGCTTTATGATTATAAAGTTGTCGACAATGAAGATGAAGTGTATTAT

CAATTCGTGCTCAAGAATAGTTCCGTTACCTCTATAGCGGTACTCAACCAAACCCTATTA

ATTCGTCAACGCCTTGTTTATGTTCCTGAATCCAAAATATGGAGTGTTTACCAAATCATG

CCATCGGATACTTGTGAATATTACAATGTTTGTGGAGCAAATGCACAATGCACGATTGAT

GGATCGCCAATGTGTCAATGTTTACCTGGATTTAAGCCAAAATCACCACAGCAATGGAAT

TCAATGGACTGGACACAAGGATGTGTGCGTGGTGGAAATTGGAGTTGCGGGATCAAAAAC

CGAGATGGGTTTCAGAAATTTGTTAGGATGAAGTTACCGGATACTACAAATTCTTGGATT

AATCTAAACATGACACTTCAAGATTGCAAAACCAAATGCTTGCAAAATTGTTCTTGCACA

GCTTATACCTATCTAGACCCAAAAGGAGAAGTCAGTGGTTGTTCTCTTTGGTTTAATGAT

CTTATTGATTTGAGACTTTCGCAAAGTAGCGAAGGCGATGATCTCTATATTCGAGTGGAC

AGAGATTCGAATTTTGGTAAGGGCTTTTAG

>MS.gene010037.t1

ATGGAACTTCAAGACACAGCAGCAACTAAAATAACCATTGGTCTTCCATTGGGTTTAGCA

CTTCTCTTTGCTTGTTTACTCTTCATTTGTGTCTTCTTTTGTTGCTTATTACATTGGAAT

AAGCTCAAATTTTTGTTTCCATATTCTTCTGGGGTCATTAATTCTCAAGCTCAGATACAA

ACTGACTTAACTTCCTCTCCTCAGAAACCAGCATTTCCTTTTGTGGTGATGAAGCAGAGC

TATGCTGAGAGCTTGCCTGTGTTGATGCCAGGAGATGAGATCCCAAAATTCATAGCCATG

GCATGTCCATGTAAACCTCCAACAGATGAAAGTATCACAATCCATGTGCACAAGGAAGAA

ACAAATGGTTTTTTGCAGTGA

>MS.gene015192.t1

ATCAAAATGTCTCGAAGATATGATAGCCGTACAACAATCTTCTCTCCTGAAGGACGTCTT

TACCAAGTGGAGTATGCAATGGAGGCCATTGGAAATGCTGGTACTGCCATAGGAATCTTG

TCGAAGGATGGGGTTGTTCTGGTTGGCGAAAAGAAGGTGACATCCAAGCTTCTGCAAACC

TCAACATCAACTGAGAAAATGTACAAGATTGATGATCATGTTGCATGTGCTGTTGCTGGG

ATTATGTCCGATGCCAACATCCTAATCAACACTGCTAGGATCCAAGCACAACGTTACTCA

TTTGCTTACCAAGAGCCAATGCCTGTTGAACAGTTGGTTCAATCTCTTTGTGATACCAAG

CAAGGTTACACACAATTTGGTGGTCTCCGTCCGTTTGGAGTCTCTTTCCTGTTTGCAGGA

TGGGACAAAAACTTCGGCTTTCAACTTTACATGAGCGATCCTAGTGGAAATTATGGCGGT

TGGAAAGCCGGTGCTATTGGTGCCAACAACCAGGCAGCACAATCAATTCTAAAACAGGAC

TACAAGGATGATATCACAAGGGAAGAAGCAGTTAACCTTGCACTGAAAGTTCTGAGCAAA

ACTATGGACAGCACCAGTCTTACCTCGGATAAGCTTGAACTTGCAGAGGTTTTTCTTGCA

CCATCTGGAAAGGTCAAGTATCAAGTTTGCTCCCCAGAGAACTTGACTAAGCTGTTGGTG

AAGTCTGGGGTGACCCAACCAGCAACAGAGACTGCTTAG

>MS.gene015191.t1

ATGAAGCTCTCAACATTACAACAAAGTTACATCAACCGTAGAACAAACAGCTTCAGAGGC

TTAGATTCAGATAACGGCGCCGGCGCCGTTAAGTCACCGGCGACAATTTTCTGGCTAGTA

CTTCACGGTGTTTGCTGCTTAATCAGCCTCGTTCTCGGCTTCCGATTTTCTCGTCTTGTT

TTCTTCTTTCTCTTTTCAACTTCATCCACTAATATTTACACCGTTCCATTCACCTCCGGC

ACCGGCGCCGGAATCACTGTTCCGGTGGAAACTCAACAGAATGTTCTAACAAACGGGAGT

TCGGTAGTGGCGAGTAGAGTCGTGGTTGGGAGACACGGGATCCGGATTCGGCCATGGCCG

CATCCGGATCCTGTGGAAGTAATGAAAGCGCATGGAATAATCTCTAGAGTTCAAAATGAA

CAGAGATTGTTGTTTGGAGTGAAGAACCCTAGAAAAGTGATTGTTGTGACACCAACGTAT

GTGAGGACTTTTCAAGCGATGCATTTGACTGGTGTGATGCATTCTTTGATGCTTGTGCCT

TATGATCTGATTTGGATTGTGGTGGAAGCTGGTGGTGTTACTAATGAGACTGCCTCGATT

ATTGGAAAATCTGGATTAAAGATTATTCATGTTGGGTTTAATCAGAAAATGCCCAGTTTG

TGGGAGGATAGACATAAAGTTGAATCTTTGATGCGTCTTCATGCTTTAAGGATTGTGAGA

AAAGAGAAGCTGGATGGAATTGTGATGTTTGCTGATGATAGTAATATGCATAATATGGAG

CTGTTTGATGAAATTCAGAGTGTGAAATGGATCGGTGCTGTTTCGGTTGGAATACTCCTT

CATTCTGTTAATGCAGCTGAAATTTCTTCATTGGTTCAAAAAGAAGGAGATGAGGAAACC

ATGCCAATGCCGGTGCAAGGTCCTGCTTGTAATGGTACTGATAAGTTGGTCGGGTGGCAC

ACCTTCAATTCATTACGATATACGGGAAGGCGTGCAGTTTACATTGATGACCGGGCACCA

GTGTTGCCGACAAAATTTGAATGGTCTGGGTTTGTGTTGAATTCCAGATTACTTTGGAAG

GATGTTGACGATAAGCCAGAGTGGATTAAGGATCTTGATGCATTGGATGGGGATGGTGAG

AAGATAGAGAGTCCACTGTCTTTGCTCAAGAGTACCTCTGTGGTAGAACCACTAGGGAGT

TGTGGACGCCATGTTTTGCTTTGGTGGTTGCGGGTTGAAGCTCGCACAGACAGCAAATTC

CCTGCTCGATGGGTGATTGACCCTCCTTTGGACATCACAGTCCCATCGAAACGCACTCCA

TGGCCAGACTCTCCTCCTGAACTCCCATCTAATGAAAATGAAAAAGTGTTCGCTGCAGGC

ACAGAAGAGCATTCGAACACGCACACTACAAAGACTAAAACACCCAGATCCAGACGCAGT

AGAAGTAAGAGAAAGCATGACACCAAAGTGATTGGTGTACAGGTCTCTACACATTCTGAA

CAAACCGAGATATGA

>MS.gene049888.t1

CACTTTGGTCTCTAAGGATGGAACCTTTGAGATGGGTTTCTTCAAACCTGGTAAGTCCTC

AAACCGCTATGTAGGAATATGGTACAAAAATATCCCCGTTCGAAGAGTGGTTTGGGTTGC

AAATCGAAACAATCCAACAAAAGACGATTCGAGCAAGTTGATCATAAGTCAAGATGGAAA

CCTTGTGCTTCTCAACCACAATGACTCTCTTGTCTGGTCAACAAATGCATCAAGAAAAAG

AAATTCTTCAAGTCCTGTTGTGCAGCTCTTGAACAATGGAAATTTAGTTCTCAGAGATGA

GAAGGATAACAATGAAGAAAGTTTTTTGTGGCAAGGCTTTGACCATCCTTGTGATACACT

ATTACCTGGAATGACGTTTGGATATAATCGAAAATTGGATTTTTATTGGAATCTCACTGC

TTGGAAGAATGAAGACGATCCATCTTCGGGAGATTTATATGCATCTGTGGTGCTTACAAG

CAATCCAGAAAGCATGATATGGAAGGGCTCGACTAAAATCTGTAGGTCAGGACCATGGAA

TCCTCTCTCTAGTGGAGTTGTCGGAATGAAACCTAACCCGCTTTATGATTATAAAGTTGT

CGACAATGAAGATGAAGTGTATTATCAATTCGTGCTCAAGAATAGTTCCGTTACCTCTAT

AGCGGTACTCAACCAAACCCTATTAATTCGTCAACGCCTTGTTTATGTTCCTGAATCCAA

AATATGGAGTGTTTACCAAATCATGCCATCGGATACTTGTGAATATTACAATGTTTGTGG

AGCAAATGCACAATGCACGATTGATGGATCGCCAATGTGTCAATGTTTACCTGGATTTAA

GCCAAAATCACCACAGCAATGGAATTCAATGGACTGGACACAAGGATGTGTGCGTGGTGG

AAATTGGAGTTGCGGGATCAAAAACCGAGATGGGTTTCAGAAATTTGTTAGGATGAAGTT

ACCGGATACTACAAATTCTTGGATTAATCTAAACATGACACTTCAAGATTGCAAAACCAA

ATGCTTGCAAAATTGTTCTTGCACAGCTTATACCTATCTAGACCCAAAAGGAGAAGTCAG

TGGTTGTTCTCTTTGGTTTAATGATCTTATTGATTTGAGACTTTCGCAAAGTAGCGAAGG

CGATGATCTCTATATTCGAGTGGACAGAGATTCGAATTTTGGTCATATACATGGGCGTGG

GAAGAAATTGGTCATGGTGGTTTCCATCACAGTTTCAATGTTGCTTGTGATGCTATTGGT

ATTATCCTACGTTTATATATTCAAACCAAAGCTTAAAGGGAAAAAAGAAAGAGATGGAGG

AGAACATGAAGATTTTGAGCTTCCTTTCTTTGATCTAGCTACAATAATTAAGGCCACCGA

TAACTTCTCAACCAACAATAAGCTTGGTGAAGGTGGTTTTGGACCCGTATACAAGGCTAC

ATTGCAAGATGGACATGAAATTGCAGTCAAAAGACTTTCAGGAAATTCTGAGCAAGGATC

GAAAGAATTTAAAAACGAAGTCATATTGTGCGCTAAATTGCAACATCGAAATCTTGTTAA

GGTTCTTGGTTGTTGCATTGAAGGAGATGAAAAATTGTTACTCTATGAATACATGCCAAA

CAAAAGCCTTGATTCATTTATTTTTG

>MS.gene049897.t1

ATGCTTACAACTTCGATGAAGAACACACCTTGTAACCGTTCTAAAGTCCCTCTCATGATC

AGCTATGGTGGCATAATCGTACCAATACCTTGGGGACACCAAAATCATCACTATACCTAC

ATAGGCGGTATCAACAGAATCGTTGCCGTTGATCGTGATATCAAGCTTTCCAAACTCATC

GCAAAGCTGTCTACAATGATGTATCCTAATTTTTGCTTCAAGTATCAACTTCTTGGTCAT

GATCTTGATACTTTGATCCCGGTTTTAAACGAGGAAGATTTCGATGATATGATGCTCAAG

TATGATCATATGTGTCGTATTTCGCCAAACCCTGCCAAGTTGAGGCTCTTTCTCTTCCCT

GTTCCTACTCCACCGGCTGAAGATTTACCTCCGCCTCCACGACGTCCGCATCAGAAGGTT

GCCTTTACAAAAGAGGAGATGAAGGAGTTTCAAGAACTGAATGTTGTGAATGATGGATTC

GAAAAGATTCTAAAATATGTTATGAAGTGTTTTTGA

>MS.gene049891.t1

ATGACACCATTTCAAACGCTATATGGCCGACCCCCACCAGGCATCCCAGACTATGTTCCA

GGTTCAACCATCATAGGATCTCTGGATTCTACCTTGCAACAACAACAGATCCTACAACTT

CTTAAAATCAATCTCAAAGCTAGCAGACAGAGCATGCAAGACAAGGCAAATAAAAAGAGA

TCTGAATGTACCTTTGACACTGGCGACTGGGTTTTGCTTCGTCTCCGACCATACCGACAG

CAAACAGTGCACCGTAGAAATTCTCAGAAGCTGGCCAAACGCTACTTCAGACCGTTCAAA

GTGTGCCGTCGAATTGGGGCTGTCGCATATGCTTTAGAGCTTCCTGCTTCCTCCCGTATC

CACCCAGTATTTCATGTTTCATTGTTGAGGAAATATCATGGTGAAGATCCTCAGAGTCAT

TACACCCCTCTACCATCAACTCTTTCTAACTATTTTTCTGAGGGGAAAGATCTAGCTCCT

ACCAGTGAAGAAGAAAGCAACGCACCCAGTAAGGAGGACACAGAGAGAAAAACAGGAGAA

GAGTTAAAAGTGGGTTTCAATCATACTACAAGCTCTACTGAAAGTACAAGAGAAAATCTA

AATACAACTCTCTCTCCAATCTATCAAACGCATTCTCCACCACTTGATCCAAATGAGTCT

TCTAGATCTGCTCAAACCGTATCTGGACCGTCCATTCAAAAGGGACCGTTGGCTCCTCCT

TTCCTTGATGACCACACAGCTGTTTCCAATAACAAGGAATTCACTCCCCAACGAGCCACT

CAGCAGCATCCAAGTGTCCCAGAAGTGACTCCTACTCTGTCCCAGGCTGACCACGCGCTC

CCCACTATTCTACCAAACAGTCAAGACACGGGCCCCACTTTTTCAACAAACGCGCCTTCC

CACACACTAGCTGGACCGCATTCTACAAACCCACTGGACTCTCACCCACCCAACAACTTG

AACCTTGAGGACAAGGTTCCTCTTGACCCGCAGAGTATTGATATGAACCGACCCAGAAAC

ATTAAGAAACCCTTTTGGCTCAAGGATTTTATTACTATGTAG

>MS.gene049920.t1

ATGGAAAATGCTTATGGAAGGTTTAAGAGATCTAGGTTTTTGATTGCGGAGAATGTGATT

AGGGCTATGGCTTATGGTTATATAAAGAAGAGAAAATTTTATGAGTTGGGTCAGTTTGTT

AGAGATGTTGGTTTAGGTAGGAGAAATGTAGGGAATCTTTTGTTGCTTTCTTATGCTGCT

AATTTTAAGATGAAGGGTTTGCAGAGAGAGTTTGTTAGAATGGTCGAATTAGGGTTTCGT

CCTGATGTTACGACGTTTAATATTCGCGCTTTGGCATTTTCGAGGATGGCGCTGTTTTGG

GATCTTCATCTTAGTATTGAACATATGAGAAGTGAAAAGGTTGTTCCTGATTTGGTTACT

TATGGTTGTGTTGTCGATGCTTACTTAGATAGAAGGCTTGGAAGGAATTTGGAGTTTGTT

TTGAATAAAATGAATGTGGATGATTGTCCGCGGTTGTTGACGGATCCGTTTGTGTCTGAG

GTTTTAGGTAAAGGTGACTTTCACTTGAGTTCGGAGGCGTTTATGGAATACAAGACACAA

TACCATAAGTGGATTATAGGGTTTTGA

>MS.gene049919.t1

ATGGCGTTGTTTTGGGATCTTCATCTTAGTATTGAACATATGAGAAGTGAAAAGATGGTT

CCTGATTTGGTTACTTGTGGTTGTGTTGTCGATGCTTACTTGGATAGAAGGCTTGGAAGG

AATTTGGAGTTTGTTTTGAATAAAATGGATGTGGATGATTGTCCGCGGTTGTTGACTGAT

CCGTTTGTGTTTGAGGTTTTAGGTAAAGGTGACTTTCACTTGAGTTCGGAGGCGTTTTTG

GAATACAAGACACAACAGCATGAGTGGAGTTATTGGGTTTTGATAAAGAAATACCTTAAG

AAACAGTATCGGAGGGACCAAATATTTTGGAACTACTAA

>MS.gene049892.t1

ATGGCACTCCGAGGATTTTTGGGCCTCACTGGTTTTTATAGGCGCTTTGTACGCCACTAC

GCCACACTCGCCGTTCCCCTCACCGACTTATTGCGTTCCACTAAATTCTCATGGAGTACA

GATGCCACTCGGGCATTTACAGAATTAAAAAATAAAATTACAACTGTGCCAATATTAGCC

CTGTCGGATTTCTCCAAACTCTTCGTGGTGGAAACTGATGCTTCTTCTGTTGCCATTGGT

GCAGTCTTGACACAAGATGGTCACCCTTTAGCTTTCTTCAGTAAAAAAATGTGTCCTCGC

ATGCAAGGTACCTCTGTCTATGTCCGTGAGATGTATGCTGTCACAGAATCAGTCAAGAAG

TGGCGTCAGTACCTAATTGGAAGACACTTTCATATTTATACAGATCAGAAAAGTTTGAAA

AATGTGTTGACACAAAAGATACAAACTCCAGAACAACAGAAATGGGCAACTAAGCTACAA

GGGTTCAGCTTTGAGATTTTTAACAAACCTGGGAAATCAAATCAAGTTGCTGATGCTCTT

AGCCGCAAATACATGGACGAAGCTACTTTGTTGATTGCAACCTCATCTACAGTTCCAGCA

GTGTTGAAACGCCTCCAACATTATTATGAGACAGAAGATGCAGGACAAAAGTTAATCACT

AAAACCCAATCGGATAACCAATGTAAAGGTCTTTTTAACTTCAAAAATGGGTTGTTGTAT

TTCAAGAACAGGTTATACATACCAGACATTCCAGAACTCCGGGAAGCCTTCCTCCGTGAG

TATCATTCAACACCCACCAGTGGACACTCTGGTACAAAAGCAACTCTGGCCCGCATTGCT

TCTTCCTTCTACTGGCCAACTATCTCCAAAGACACCAAACTCTTTGTATGGGAAGAACTA

ACTATGGATTTTATCACTCACTTACCAAATTTCCATGGACACACTGCCATTTGGGTCATC

TGTGACCGGTTGACCAAATATGTTCATTTCTTGGCTCTTCCCACCCACTATACCGCTACT

GATTTGGCCAATCGATTCTCTGTGGAGATTTGTCGCCTGCATGGAATACCAAAATCAATA

ACATCAGACCGAGATCCAATTTTCCTAAGTTCTTTTTGGAAGGAATTATTTCGTGTGCAA

GGGACCAGTCTCAGATACAGCACAGCATACCATCCTGAGACTGATGGTCAAACGGAGGTT

GTTAACAGATGTTTAGAAGCTTACCTACGGTGTTTTGCCAGTGACCGTCCTCGTACATGG

TACAAGTTCTTGCATCTCGCATAG

>MS.gene049909.t1

ATGGTGTTAAGAAAAGTGGGTAAGTATGAAATTGGAAGAACAATTGGTGAAGGAACATTT

GCGAAGGTTAAATTCGCTCAGAATACTGAAACTGGTGAAAGTGTTGCTATGAAGATTCTT

GATCGTAGCACCATCATCAAGCATAAAATGGCCGATCAGATCAAGAGGGAGATTTCCATT

ATGAAGCTAGTCAGACATCCATATGTTGTTCGCCTGCATGAGGTTCTGGCGAGCCGTACT

AAGATATATATCATATTGGAGTTCATCACAGGTGGTGAATTGTTTGATAAAATTGTACAC

CACGGGCGTCTTAGTGAAGCTGAAGCTAGGAGATACTTCCAACAACTTATTGATGGTGTA

GATTATTGCCACAGCAAGGGAGTTTATCATAGAGATTTGAAGCCTGAAAACCTTTTACTT

GATTCACTGGGAAATATGAAGATTTCAGACTTCGGTTTGAGTGCATTGCCAGAACAAGGG

GTTAGTATGCTGCGGACAACTTGCGGGACTCCTAATTATGTAGCTCCCGAGGTACTCAGT

CACAAAGGTTATAATGGTGCTCCAGCAGATGTTTGGTCTTGTGGAGTTATCCTTTATGTT

CTGATGGCTGGATATCTTCCCTTTGATGAGCTTGATCTAACCACGTTATACAGTAAGATT

GACAGAGCCGACTTTTCATGCCCTGCTTGGTTTCCTGTGGGAGCGAAATCTTTGATACAG

AGGATTTTGGACCCAAACCCTGAACATCGTATAACCATTGAGCAGATTCGAAATGATGAG

TGGTTTCAAAAAAGCTATGTTCCTGTCCATCTCCTTGAGTATGAAGATGTAAATCTGGAT

GAGATAAATGCTGCATTTGACAGTGCTGAGGATGAGGGGGATAATCCACAGTGTGAGATT

GCGGACACGGGTCCTCTGATTCTTAATGCATTCGACATGATAATGTTATCTCAAGGCTTA

AATCTTGCAACAATCTTTGATCGTGGACAGGACACTATGAAGTACCAAACACGCTTCATC

ACTCAAAGGCCAGCGAAAGTAGTTTTATCAAGTATGGAAGTTGTGGCACAATCAATGGGA

TTTAAGACGCATATTCGTAACTATAAGATGAGAATAGAGGGTATTTCAGCAAAAAAGACA

TCTCATTTCTCAGTTATACTGGAAGTATTTGAAGTTGCTCCCACATTTTTCATGGTGGAC

ATTCAGAAAGCAGCTGGAGATGCTAGTGAATATCTCAAGTTTTACAAGAACTTTTCTAGC

AATCTTGAGGATATCATCTGGAAATCCCCTCATGAAACAAGCAAATTGAAGACCTCCAAG

ACTAGAAGCAAAAGACATTAG

>MS.gene049889.t1

ATGGGTTTCTTCAAACCTGGTAAGTCCTCAAACCGCTATGTAGGAATATGGTACAAAAAT

ATCCCCGTTCGAAGAGTGGTTTGGGTTGCAAATCGAAACAATCCAACAAAAGACGATTCG

AGCAAGTTGATCATAAGTCAAGATGGAAACCTTGTGCTTCTCAACCACAATGACTCTCTT

GTCTGGTCAACAAATGCATCAAGAAAAAGAAATTCTTCAAGTCCTGTTGTGCAGCTCTTG

AACAATGGAAATTTAGTTCTCAGAGATGAGAAGGATAACAATGAAGAAAGTTTTTTGTGG

CAAGGCTTTGACCATCCTTGTGATACACTATTACCTGGAATGACGTTTGGATATAATCGA

AAATTGGATTTTTATTGGAATCTCACTGCTTGGAAGAATGAAGACGATCCATCTTCGGGA

GATTTATATGCATCTGTGGTGCTTACAAGCAATCCAGAAAGCATGATATGGAAGGGCTCG

ACTAAAATCTGTAGGTCAGGACCATGGAATCCTCTCTCTAGTGGAGTTGTCGGAATGAAA

CCTAACCCGCTTTATGATTATAAAGTTGTCGACAATGAAGATGAAGTGTATTATCAATTC

GTGCTCAAGAATAGTTCCGTTACCTCTATAGCGGTACTCAACCAAACCCTATTAATTCGT

CAACGCCTTGTTTATGTTCCTGAATCCAAAATATGGAGTGTTTACCAAATCATGCCATCG

GATACTTGTGAATATTACAATGTTTGTGGAGCAAATGCACAATGCACGATTGATGGATCG

CCAATGTGTCAATGTTTACCTGGATTTAAGCCAAAATCACCACAGCAATGGAATTCAATG

GACTGGACACAAGGATGTGTGCGTGGTGGAAATTGGAGTTGCGGGATCAAAAACCGAGAT

GGGTTTCAGAAATTTGTTAGGATGAAGTTACCGGATACTACAAATTCTTGGATTAATCTA

AACATGACACTTCAAGATTGCAAAACCAAATGCTTGCAAAATTGTTCTTGCACAGCTTAT

ACCTATCTAGACCCAAAAGGAGAAGTCAGTGGTTGTTCTCTTTGGTTTAATGATCTTATT

GATTTGAGACTTTCGCAAAGTAGCGAAGGCGATGATCTCTATATTCGAGTGGACAGAGAT

TCGAATTTTGGTCATATACATGGGCGTGGGAAGAAATTGGTCATGGTGGTTTCCATCACA

GTTTCAATGTTGCTTGTGATGCTATTGGTATTATCCTACGTTTATATATTCAAACCAAAG

CTTAAAGGGAAAAAAGAAAGAGATGGAGGAGAACATGAAGATTTTGAGCTTCCTTTCTTT

GATCTAGCTACAATAATTAAGGCCACCGATAACTTCTCAACCAACAATAAGCTTGGTGAA

GGTGGTTTTGGACCCGTATACAAGGCTACATTGCAAGATGGACATGAAATTGCAGTCAAA

AGACTTTCAGGAAATTCTGAGCAAGGATCGAAAGAATTTAAAAACGAAGTCATATTGTGC

GCTAAATTGCAACATCGAAATCTTGTTAAGGTTCTTGGTTGTTGCATTGAAGGAGATGAA

AAATTGTTACTCTATGAATACATGCCAAACAAAAGCCTTGATTCATTTATTTTTGATCCA

ACTCAAAGCAAATTGTTAAGCTGGTCAATGCGTTTTAACATCTTGAATGCAATAGCTCGA

GGAATTCAATATCTTCATCAAGACTCTAGATTAAGGATCATACATAGGGATCTCAAGGCG

AGTAATATTTTACTAGACAATGAAATGGATCCAAAAATTTCAGATTTTGGCATGGCTAGA

ATGTGTGGAGGTGATCAAATTGAAGGGACAACAAGAAGAATAGTTGGTACATACGGTTAT

ATGGCACCCGAATATGTGATTCATGGGTTATTCTCTATTAAATCAGATGTATTCAGCTTT

GGTGTATTATTGCTTGAAACAATAAGCGGAAAGAAAAACAGAACACTTACCTACCATGAA

CATGATCACAATCTTATTTGGCAT

>MS.gene049910.t1

ATGGAGTGTGCTCAACAAGTATTTGATCACATGAATAGAAGAAATGTTGTTGCTTGGACA

AATTTAATGAAGGGTTATGTGCAAAACTCAATGCCAAAACATGCTATACATTTGTTTGAA

GAGATACTGTTACATTCAGAGTGTTACCCTTCAAATTACACTCTTGCTATTGCCCTTAAT

GCTTGTACATCTTTGCATTCATTGAAGTTAGGAGAACAGTTACATGCTTACATAATCAAA

TACCATGTTGATTTTGACACAAGTATCGGCAATGCGCTTTGTAGTTTATACACGAAATGT

GGTGGTAGGTTGGAATTTGGTCTAACAGCATTTAGGAGAATCAAAGAAAAGGATGTTATT

TCTTGGACTGCAGCTATTTCTGCTTGCGGCGAAAAAGGTGAAGCTATTAAGGGTGTGAGA

GTTTTTGTTGAAATGCTTTTAGATGAAGTGCAGGTTCAACCTAATGAGTACACTTTAACT

AGTGTCTTGAGTCAGTGCTGTGAGGTTAAGTGCTTAGAGCTTGGGATTCAGGTTCATGCT

TTGTGTACTAAATTAGGGTACGAGTCAAACCTACGTGTTCGGAATTCTTTATTGTATATG

TACCTTAAATGTGGTTGTATTGTTGAGGCTCAAAGGTTGTTTAAAGGAATGGATGATGTT

AATTTGGTAACATGGAATGCTATGATTGCTGGACATGCACAAATGATGGAGCTTTCAAAG

GATAATCTTTCTGCATACCAAAAAGGAATTGAAGCACTCAACCTTTTCTCGAAGTTGAAT

CAGTCTGGCATGAAACCTGATCCGTTTACCTTTTCAAGTGTGTTGAGTGTTTGCAGTAAA

ATGATGGCTTTAGAACAAGGTGAACAGATTCATGCTCGGACAATCAAAACCGGGTTCTTA

TCAGAGGTTGTTGTAGGTTCTTCTATGATTAATATGTATAACAAATGTGGGAGCATTGAG

AGAGCAAGCAAAGTTTTTCTAGAGATGTCTATTAGAACGATGATATTATGGACTACAATG

ATTACTGGTTTTGCACAGCATGGTTGGTCAAAGCAAGCATTGAATCTTTTTGAGGATATG

AAACTTGTAGGTATCAGACCAAATCCGGTCACTTTTGTCGGCGTTTTATCAGCTTGTGGA

AGTGCTGGAATGGTCAATGAAGCATTCAATTACTTTGAGATTATGCAAAAGGAATATAAA

ATTAAACCTGTGATGGACCATTATGCTTGTTTGGCTGATATGTTAGTGAGGCTCGGACGG

GTAGAGGAAGCTTTCGATTTGATAAAGAAAATGGATTATGAGGCTAGCGAGTTTATTTGG

TCAAATTTGATTGTTGGTTGTCTAAGCCATGGGAATCTAGAATTGGGTTGTGATGCTGCA

GAAAAGTTGTTAAGTCTCAAACCAAAAGATACAGAGACATACAAATTGCTGTTGAATGCG

TACGTCACAGCCGGACGATATGACGATGTTTCTAGGGTGGAGAATATAATGAAAGAAGAG

AAAATTGGAGAGTTAAAAGATTGGAGCTGGATTAGCATCAAAGACAGAGTGTATTCATTT

CAAACAAATGACAAAGCAGACATAGAAAGTTCAATAAGTAAATCATTGGAGGATTTACAT

ATCAAAGCAAAGAATCTTGGATATGAGATGTTAGAAAGTGTGGAAATAAGTGACAAAGAA

GAGAAAGAAAAGACATCCTCACCCACCATTTATCACAGTGAGAAGCTAGCCATAACATTT

GGGTTGGAGAATTTGCCAAATTCTTCACCAATAAGAGTTGTTAAGAATACCTTAATGTGC

AGGGATAGTCATAATTTTGTTAAGTACATCTCAACAATGACTAGTAGGGAAATCATTGTT

AAAGATAGTAAGAGGCTACATAAATTTGTCAATGGACAATGCTCGTGTGGGAATATTGGT

GGTTTTCTCTGA

>MS.gene049902.t1

ATGGAGCACACCAAGATCCCTCTCATGATCAGCTATCGTGGAACAATCGTCCCGATGCCT

TGTGGACCCCAAAATCATCTTTATACCTATGTAGGCGGTATCAATAGAGTCATCATTGTT

GATCGTGATATCCAGCTGTCCAGCCTCATTCAAAAGCTTTCCACGATGGATTCTAATTTT

TGCTTCAAGTATCAACTTCCTGGTCATGATCTCGATACTTTGATCCCGGTTTTAAACGAG

GAAGATTTCGATGATATGATGCTCAAGTATGATCATATGTGTCGTATTTCGCCAAATCCC

GCCAAGTTGAGGCTCTTCCTCTTCCCTGTTCCTACTCCACCGGCTGAAGATTTACCTCTG

CCTCCAAGACGTCCGCATCGGAAAGTTAGCTTCACAAAAGACGAGATGAAGGAATTTCAA

GAACTCAATATTGTGAATGATGGATTCGAAAAGATTCTAAAATATGTTATGAAGTGTTTT

TGA

>MS.gene049899.t1

ATGCTTACAACTTCGATGAAGAACACACCTTGTAACCGTTCTAAAGTCCCTCTCATGATC

AGCTATGGTGGCATAATCGTACCAATACCTTGGGGACACCAAAATCATCACTATACCTAC

ATAGGCGGTATCAACAGAATCGTTGCCGTTGATCGTGATATCAAGCTTTCCAAACTCATC

GCAAAGCTGTCTACAATGATGTATCCTAATTTTTGCTTCAAGTATCAACTTCTTGGTCAT

GATCTTGATACTTTGATCCCGGTTTTAAACGAGGAAGATTTCGATGATATGATGCTCAAG

TATGATCATATGTGTCGTATTTCGCCAAACCCTGCCAAGTTGAGGCTCTTTCTCTTCCCT

GTTCCTACTCCACCGGCTGAAGATTTACCTCCGCCTCCACGACGTCCGCATCAGAAGGTT

GCCTTTACAAAAGAGGAGATGAAGGAGTTTCAAGAACTGAATGTTGTGAATGATGGATTC

GAAAAGATTCTAAAATATGTTATGAAGTGTTTTTGA

>MS.gene049894.t1

ATGCAACCGATACCATTGCCAAGTCTGCTTCATTTCCTGATGGCAGCACTTTGGTCTCTA

AGGATGGAACCTTTGAGACCTGGTAAGTCCTTAAACCGCTATGTAGGAATATGGTACAAA

AATATCCCCGTTCGAAGAGTGGTTTGGGTTGCAAATCGGAACAATCCAACAAAAGACGAT

TCGAGCAAGTTGATCATAAGTCAAGATGGAAACCTTGTGCTTCTCAACCACAATGACTCT

CTTGTCTGGTCAACAAATGCATCAAGAAAAGCTTCAAGTCCTGTTGTGCAGCTCTTGAAC

AATGGAAATTTAGTACTTAGAGATGAGAAGGATAACAATGAAGAAAGTTTTTTGTGGCAA

GGGTTTGACCATCCTTGTGATACACTATTAGCTGGAATGAAGTGTGGATGGAATAGAAAA

TTGGGTTCAGTATGGAATATGACTTCTTGGAAAAATGAAGAGGATCCATCTTCAGGAGAT

GTAATTCAAGTTATGGTGCTTACAAGTAATCCAGAAAGCGTAATATTGAAGGGCACGACT

AAAATCCATAGGACTGGACCATGGAATGCTCCGTCAAGTGGAGTAGTCAGATTGAAACCT

AATCCACTTTACGATTTTGTATTTGTCAACAATGAAGATGAAGTGTATTATAGATACACG

CTGAAGAATAGTTTTGTGATCTCTATAGTGATTGTCAACCAAACCCTAGCAGTTCGACGT

CTCCTTTATGTTTCTGAATCAAAAACATGGAGTGTTTACCAAACCACGCCATTGGATGCT

TGTGATTATTACAACATTTGTGGGGCAAATGCACAATGCATCATTGATGGATCGCCAATG

TGTCAATGTCTACTTGGATTTAAGCCAAAATCACCGCAACAATGTAATTCAATGGACTGG

ACACAAGGATGTGTCCGTATTGGAAATTGGAGTTCTTATACATACTTGGATGCAAATGGA

GCAGTCAGTGGTTGTTCCCTCTGGTTTGGTGATCTTATTGATTTAAGAATTTCACAAAGT

AGTGGTCAAGATCTCTATGCTCGAATGGACATTGATTCTTCAAATTTCGGAGGTCTTGCA

CATGGGCGTGGGAAGAAAGTTGTCATGACGGTTTCGATCACAGTTTCAATGTTGCTTGTG

ATGCTATTGGTATTCTCCTACGTTTATATATTCAAATCAAAGCTTAAATGTAAGATTCTT

AATTAG

>MS.gene049908.t1

ATGGAGCATCAATGCTTCAATTCCCATCCCAATTCCGGCAATTCCTCACCCACCTCTCGT

GAATTGCTCGAAAACGATCACCACCACCGTTCATTCGATGAACCACCACCTTCCAACGGT

CCCAGAGTGAAGCTCATGATTCGCTTCGGTGGAAAAATCGAACTCAGGCATCACGACGAT

CAAAACTATTCCTACATCGGCGGTGACACCAAAATCCTCACCGTCGATCGTAGTATCAAA

TACTCTAACCTCATTGATAAGCTATCATCAATGACGTTTTCTGATCTTTGCCTAAAGTAT

CAACAACCTGGTGGAGAGCTCGATTCTTTGATCTCAGTATTCAACGATGACGATCTCGAA

AGTATGATGTTTGAGTATGATTGCATGTGTCGCGTTTCACCTAAACCTGTAAGGATGAAG

GTTTTTCTCTTCCCTCTCCCTGTTAACAATGCACCTTCTGATTCTTTGGATTCCGCTTTC

AATCTTCCGGTGGTGGAAGATTCGAAATCCGACGGACAGATGTTCGTCAAGATGCTCAAT

TCCGTTCATCGTCCGCCGCCGGTGGAAGATTTATCTCCGCCGCCGTCGACGATGAAGACG

AGTCCAGATTATCTTTTTGGATTAGATAACAATCAGTCGAAAATTCCGGTGACTGAACCG

GATTTCGCGGCGAAGGATGCGGAATGTGGGACCGAGACGGTTACAGAAAAAGAGATTCAG

GAAATTCAGATGGTGGAAACAGTGAATGATGACGAAAAGAAAGTGAAAGTTAACGGAGAA

AACGACGGAATTAACGGTAATGCTGAACAGCAAGTGAAAATTGACGGAGAAAACGGTGGA

AATAACGGTGGTGTTGATGTTTCCTCTGAAGAAAACACGGAAAGAGTGATTCCGTTAGTT

ACTAGTGAACCTTCTGCTGAGGATCCGGTTCATGAAGTACAACCCGGTTCATTTTGTTCC

TGTTCGATTCAGGTTCCTGAACCGGTTTCGGTTCAGTCATCCTTTACTTCAGAAATGTAT

AATGTTGTTGTTGCTGGTGCTGGTAGTGGTTATTCGATGGGGTATGTAAATGAACCGTTA

CCGGTTTATTTGATTCCAACACCTTCTGGATTGTATCAAGCTATGAGGCCGGTTACTGGA

CCAACGGGTCAACCGGTTTATTTCGCGTATTCGCCGATTGTGAACAATGGTGGTGGATAT

TCGCATGGTTCGTATGTGGCTAACAGATCAGCTTTGCCATTGTAG

>MS.gene049911.t1

ATGAAGTGGGCTGAGAATGGCGCGAATGGGAAGGAGGCTGGTGGAAGTAATGTTGCTCGA

AAAGACAAGGGCAAGCAGAAGGCTGTTGTTGAGGACCACCCACTCAAGATAAGGTTCCAC

TACAATGGATACTTTACTGCTGACCCTCATGTTTGCTACAAAAGAGGTGAAACCTACGAG

TTTGAGGGTACATGGGATTTAAATGAAGTGAACTTAATTGATTTGGAGAAATTGGTGAGG

GAAGTAGGTGTGACAGGGGATTATACACTCTGGTATCCTGTAGCTGGGGGAGAACTGAAA

GAAGGTGTTAGAGTAATCAAAACTGTTGGAGATGTGGAGAGGTTTAAGGATGAGTACAAG

TTAGAGGAAACTGTCAATTTTTACTTGGAACATCTAAGTTTGGAATATCTTGAGAAAATA

TATTCAGATGAAGAATTTTTCTATTCTGGTGAAGAACCTGAAAGTGAAACAGATCCTGAT

GTTGATCTAGATGACAGTGACTATGATGAGGAATTTGACTGGACAACTGTGTTGCCTGAC

CAGCTAATCAATCCAACACCTGTTGATGCTTCAAGTGGTAGTGTTCAGGCTCTGGTAGCT

GTTAATGCTTCAAGGAACCCTGATGCAACAACTCTGGAAGATTTTGATGATGAGGATGGA

GATTCAGACTATCTAGAGAGTTCATGCTCTTCTGGAGATGAGAGAGGTAAGCAAAAGTTT

ACAAAGTTTAAGTTGTCTGATGAAATTACTTTTAAGGTAGGACAGATATTCACAAATGCT

GAGTTGGTGAGGAGATCTGTTAAAGAGTATGGGTTGCAACAAAGGAAAGATGTCTGGACT

AAGAAGAATGAGAGTAAAAGGATTGTGGTGTCAGCCAAAATGTCCATTCCACATGAGGAA

ACTGATACCAATTCTGAAACACTCACCTGGGCTAAGAAAAGGGCTTTGGAACTGATTCAT

GGTGGAATAGATGAGCAATATGCTCATCTTAGAAATTATGCTGAGGAGTTGCTGAGGAGT

AATCCAGGTAGTACTGTTAGGATTAAATGTGCAGTTGGAGATGCAGTAAAACTTAGGGAT

TTTATGCTGAGGTATGATGGAGATATTTGTCCTATGATTAACAAACTGTTTGATAAGTAT

AAGAAAGAAGCTGAGGGTTGGTCTCCAAATTGGAGTGGAGATAGGGACTATGCAAGATTT

TCTGTTTCAGATGGGAGTGATGCATATGTGGTTAATCTCAAAGAAAAGACATGTGCATGT

AGAAAATGGGAACTAACTGGCATTCCTTGTCCTCATTCAATTGCTTGTATCTGGTACAAC

CAGCAAAACCCATATGAGTTTGTTGCTCATTGGTACAGGAAATCCACATTTCTAGATACT

TATGACAATCTGATACTTCCATCAAATGGACCAAAGTTGTGGCCAGAAATTGACTTGCCA

CCTATATTGCCACCTTACATGAGAAGGGCACCTGGTAGGCCCAAAAAGCAAAGAAAGAGA

GCAAATGATGAGCCAAAGAAACCTTCTGCTTCTGCGGCTGCATCTGGTACTCAATCTTTG

GTCAAAAGGAACCAAGTCACTGTGAGGTGCACAAGGTGTGGTGTCTTGGGTCATAACCAA

AGAACATGCTATGGTAAACAAGCTGCTGAGAGGAACATTCCAGCAGGGGGTAACAAGCCA

CCACCTGCTGCCAACATTGTCAACACTCAGCCACCACCTTCTGTCACAACTGGATCTTCT

GTCACAACTGGACCACCAGCTGCTAGTAGCTATACAGCAGGTGCAAGCAACACTTTGAAT

GTGACCAAAACTGTCAATGTGACCAAAACTGTTAATGTAACCAAAACTGTGAATGTGACC

AACTCTGTGCCAATTGCTGCCACTAACGCTAAACCACAAGTTGTGGGAGAGAATGTGTTA

ATGGTGCCAAAGAAGGGCAGGACCACAGGTACAAAAAGAAAGAGTGGTGATTTGGAGCCT

TTTGGGACACAACAATCAGTGAACAAGAAATGA

>MS.gene049903.t1

ATGCTTACAACTTCGATGAAGAACACACCTTGTAACCGTTCTAAAGTCCCTCTCATGATC

AGCTATGGTGGCATAATCGTACCAATACCTTGGGGACACCAAAATCATCACTATACCTAC

ATAGGCGGTATCAACAGAATCGTTGCCGTTGATCGTGATATCAAGCTTTCCAAACTCATC

GCAAAGCTGTCTACAATGATGTATCCTAATTTTTGCTTCAAGTATCAACTTCTTGGTCAT

GATCTTGATACTTTGATCCCGGTTTTAAACGAGGAAGATTTCGATGATATGATGCTCAAG

TATGATCATATGTGTCGTATTTCGCCAAACCCTGCCAAGTTGAGGCTCTTTCTCTTCCCT

GTTCCTACTCCACCGGCTGAAGATTTACCTCCGCCTCCACGACGTCCGCATCAGAAGGTT

GCCTTTACAAAAGAGGAGATGAAGGAGTTTCAAGAACTGAATGTTGTGAATGATGGATTC

GAAAAGATTCTAAAATATGTTATGAAGTGTTTTTGA

>MS.gene049890.t1

ATGGGTTTCTTCAAACCTGGTAAGTCCTCAAACCGCTATGTAGGAATATGGTACAAAAAT

ATCCCCGTTCGAAGAGTGGTTTGGGTTGCAAATCGAAACAATCCAACAAAAGACGATTCG

AGCAAGTTGATCATAAGTCAAGATGGAAACCTTGTGCTTCTCAACCACAATGACTCTCTT

GTCTGGTCAACAAATGCATCAAGAAAAGCTTCAAGTCCTGTTGTGCAGCTCTTGAACAAT

GGAAATTTAGTACTTAGAGATGAGAAGGATAACAATGAAGAAAGTTTTTTGTGGCAAGGC

TTTGACCATCCTTGTGATACACTATTAGCTGGAATGAAGTGTGGATGGAATCGAAAATTG

GGTTTAGTCTGGAATATGACTTCTTGGAAAAATGAAGAGGATCCATCTTCAGGAGATGTA

ATTCTAGTTATGGTGCTTACAAGTAATCCAGAAAGCGTAATATTGAAGGGCTCGACTAAA

ATCCATAGGACAGGACCATGGAATGCTCTTTCAAGTGGAGTAGTCGGATTTAAACCTAAT

CCACTTTACGATTTTGTATTTGTTAACAATGAAGATGAAGTGTATTATAGATACACGCTG

AAGAATAGTTCTGTGATCTCTATAGTTATTGTCAACCAAACCCTAGCAGTTCGACAACGT

CTCCTTTATGTTCCTGAATCAAAAACATGGAGTATTTACCAAACCACGCCATTGGATGCT

TGTGATTATTACAATGTTTGTGGGGCAAATGGACAATGCATCATTGATGGATCGCCAATG

TGTCAATGTTTACTTGGATTTAAGCCAAAATCACCACAACAATGGAATTCAATGGACTGG

ACACAAGGATGTGTGCGTAGTGGAAATTGGAGTTGTGGGATCAAAAACCAAGATGGATTT

CAAAAATTTGTTGGGATGAAATTTCCAAATACTACAAATTCTTGGATTAATCGACACATG

ACACTTAACGATTGCAAGATCAAATGCTTACAAAATTGTTCTTGCACAGCTTATACATAC

TTGGATGCAAATGGAGCAGTTAGTGGTTGTTCCATCTGGTTTGGCGATCTTATTGATTTA

AGAATTTCACAAAGTAGTGGTCAAGATCTCTATGTTCGAATGGACATTGATTCTTCAAAT

TTCGGGAAAAAAGAAAAAGATGGAGCGGGACATGAAGATTTTGAGCTTCCTTTCTTTGAT

CTAGATACAATGATCAAGGTCACCGATAATTTCTCAATCAACAATAAACTCGGTGAAGGT

GGTTTTGGACCAGTATACAAGGGTACATTATTAGATGGACAAGAAATTGCAGTCAAAAGA

CTTTCAGGAAATTTTGGGCAAGGATTGATAGAATTTAAAAATGAAGTCATATTGTGTGCT

AAATTGCAACATCGAAATCTTGTTAAGGTTATTGGTTGTTGCATTGAAGGAGAGGAAAGA

CTCATACTCTACGAATACATGCCAAACAAAAGCCTTGACTCATTTCTTTTTGATCCAACT

CAAAGTAAATTGTTAAGTTGGTCATTGCGTTTTAACATCTTGAATGCAATTGCTCGAGGA

ATTCAATATCTTCATCAAGATTCCAGATTAAGGATCATACACAGAGATCTAAAGGCAAGT

AATATTTTATTAGACAATGAAATGGACCCGAAAATTTCAGATTTTGGCTTGGCTAGAATG

TGTGGAGGTGATCAAATTGAAGGGAAGACAAGCAGAATTGTTGGTACATACGGCTATATG

GCACCGGAATATGTGATTCATGGATTATTCTCTATAAAATCAGATGTATTTAGCTTTGGT

GTATTACTGCTTGAAATAATAAGCGGAAAGAAAAATAGAGCACTTACCTATCATGAACGT

GATCACAATCTTATTTGGCATGCCTGGAGATTGTGGAATGAAGGAACTCCACATAATTTG

ATTGATGAATGCTTAAGAGAAACATGTCTTCTACACGAAGCTTTACGATGTATCCAAATT

GGTCTATTATGTGTGCAACATGATGCTAATGATAGGCCAAACATGAAATATGTGATTACG

ATGTTGGATAGTGAAAGTACCTTACCTGAACCAAAAGAACCTGGATTTTTAATTCAAAGG

GTATTGATTGAAGGACAATCTTGTTCTAAAAGCCAAACATCTTCTGATAATGGAATAACT

ATCACACGATTAAGTGCTAGATAG

>MS.gene049895.t1

ATGGATACTGCACCTGGTCAAAGTCTCTATCCATTGCACCATAGCAAAACTATTCACCTG

GTTAGGCATGCCCAAGGAGTTCATAATGTAGAAGGAGAAAAGAACCATGATGCTTACTTA

TCTTATGATTTTTTTGATGCAAACCTAACTCCTCTTGGCTGGCAGCAGGTTGAAAATCTG

CAAAAGCATGTGAAGGCTATCGGGCTTTCCAAAAAAATTGAACTAGTTGTGGTTTCCCCA

TTGTTAAGGACAATGCAAACGGCTGTTGGAGTCTTTGGTGGGGAAGCAAACACTGATGGA

GTTAATGAATCTCCTCTGATGATTGAAAATGTGGGAAACAGTGATCATCCTGCAGTTTCT

AGTCTTAACACCCCACCATTCGTTGCAGTAGAGCTTTGCCGAGAGCAAATGGGACTCCAT

CCATGTGATAAGAGAAGAACTGTCAGCGAGTACCGACACATGTTTCCAGGAATTGATTTT

TCATTGATTGAAACTGACGACGACACTTGGTGGACACCTGAAAGAGAGAAGAAAGAAGAA

GTTACTGGTAGAGGACTCAAGTTTTTGGAATGGTTGTGTACACGTAAAGAGAAGGAGATA

GCTGTTGTTACACACAGCAGTTTTCTGTTTAATACCCTTAGTGCTTTTGGAAATGACTGT

CATCCAAATATTAAGACTGAAATGTGTGCACATTTTGCTAATTGTGAACTACGTTCCATG

GTTATTGTGGATAAATGTATGATTGGATCGAATAATTCAACTACCAATTATCCTGGCAAA

ATTCCTTATGGTCCGGATCTTCCCAGTGATGCTACTGACTAG

>MS.gene049912.t1

ATGGAGTACCAAAGCTTCAATTCCTCACCCAACTACCATGAATTGCTCGAAAACTATAAG

CATCCATCATCCTACACTTCCAAAGTCAAGCTTATGGTTAGCTTTGGCGGAGAAATCAGA

CCTAGCCTTCAGGACGATCATCATTTTTGGTACATTGGCGGAACCACCAAAATCATCATG

GTTGATCGTAATATCAAATTCTCTGACCTCGTTGAAAAGCTTTCTTCAACGATGTTTGCT

AATGCTTGCTTCAAGTACCAGCTCCCTGGTGAAGATCTCAATGATTTGATCTCCGTTCAC

AACAATGATGATCTCGAAAATATGATGGTCGAGTATGATCGTATGTGTCGCGCTTCTCCA

AAGCAACCGGTAAGGTTGAGGCTCTTCCTCTTCCCTGTTCGTGCTAACAACAACAAAGAC

CACAATTCAAGACCTTATAGTCTTGTTGAGTTGCTCAATTCCGTTCACGTTCCGACTTTT

GAAGATTCATCTCCTCCGCCAGCTGAAGATTCAACTCCACCTCCTCCTCCTCCTCCACTA

GAAGATTCAATTCCACCTCCTCCGTCATCAACAACCACTAATCCCGAGTTGAGTCAGGTG

GAATGTCCGGAGACTTTGCCGGATTTCGCAGCAAAGGATACGAAGTGTGGGCCGGAGACG

GTTATCGAAACCAATATTCAGGAAATTCAGAAGGTGGAATCTGTGAAGGATGAACAGCAA

GTGAAAGTTGACGAAGAAAACAACGGTGTTGAAGTTTACCCTGAAGAAAGCGCGGAAAAA

GTGATTTCGTTAGTTACTGATGAACCTACTGCTGCTGAGGATCCGGTTCAAGAAGCTCCT

CCCGGTTCGTTTTGTTCCTGTGGAGTTCAGTTTCCTGAACCGAGTTTGGTTCCTGAACCA

ATTTCGGTTCAGTCATCCTTTACTCCAGAAATGCACAATGTGGCTGCTGATGGTTATTAC

TCGATGGGGTATTCAACTGAACTGTTACCAGTTTATTTGATTCCAACATCTTCAGGATTG

TACCAAGCAATGAGGCCGGTGACCGGACCAACTGGTGAACCGGTTTATTTCGCGTATGTG

CCGATTGTCAATGAGGTTGATTACAATGGTGGTCAGTATGCACCTAACAGATCAACCTTG

CCATTGTAG

>MS.gene049898.t1

ATGGAGCACACCAAGATCCCTCTCATGATCAGCTATCGTGGAACAATCGTCCCGATGCCT

TGTGGACCCCAAAATCATCTTTATACCTATGTAGGCGGTATCAATAGAGTCATCATTGTT

GATCGTGATATCAAGCTGTCCAGCCTCATTCAAAAGCTTTCCACGATGGATTCTAATTTT

TGCTTCAAGTATCAACTTCCTGGTCATGATCTCGATACTTTGATCCCGGTTTTAAACGAG

GAAGATTTCGATGATATGATGCTCAAGTATGATCATATGTGTCGTATTTCGCCAAATCCC

GCCAAGTTGAGGCTCTTCCTCTTCCCTGTTCCTACTCCACCGGCTGAAGATTTACCTCTG

CCTCCAAGACGTCCGCATCGGAAAGTTAGCTTCACAAAAGACGAGATGAAGGAATTTCAA

GAACTCAATATTGTGAATGATGGATTCGAAAAGATTCTAAAATATGTTATGAAGTGTTTT

TGA

>MS.gene049896.t1

ATGGAGCACACCAAGATCCCTCTCATGATCAGCTATCGTGGAACAATCGTCCCGATGCCT

TGTGGACCCCAAAATCATCTTTATACCTATGTAGGCGGTATCAATAGAGTCATCATTGTT

GATCGTGATATCAAGCTGTCCAGCCTCATTCAAAAGCTTTCCACGATGGATTCTAATTTT

TGCTTCAAGTATCAACTTCCTGGTCATGATCTCGATACTTTGATCCCGGTTTTAAACGAG

GAAGATTTCGATGATATGATGCTCAAGTATGATCATATGTGTCGTATTTCGCCAAATCCC

GCCAAGTTGAGGCTCTTCCTCTTCCCTGTTCCTACTCCACCGGCTGAAGATTTACCTCTG

CCTCCAAGACGTCCGCATCGGAAAGTTAGCTTCACAAAAGACGAGATGAAGGAATTTCAA

GAACTCAATATTGTGAATGATGGATTCGAAAAGATTCTAAAATATGTTATGAAGTGTTTT

TGA

>MS.gene049915.t1

ATGGAGTGTGCTCAACAAGTATTTGATCACATTCACACGAATAGAAGAAATGCTGTTGCT

TGGACAAATTTAATGAAGGGTTATGTGCAAAACTCAATGCCAAAACATGCCATACGTTTG

TTTGAAGAGATGCTGTTGCATTCAGAGTGTTACCCTTCAAATTACACTCTTGCTATAGTC

GTTAATGCTTGTACATCGTTGCTTTCTTTGAAGTTAGGAGAACAGTTACATGCTTACATA

ATCAAATACCACGTTGATTTTGACACAAGTATCGGCAATGCGCTTTGTAGTTTATACACT

AAATGTGGTGGTAAGTTGGACTTTGGTCTAAAAGCATTTAGGAGAATCAAAGAAAAGAAT

GTTATTTCTTGGACTGCAGCTATTTCTGCATGCGGCGAAAAAGTTGACGCTATGAAAGCA

TTCAATTACTTTGAGATTATGCAAAAGGAATATAAAATTAAACCTGTGATGGACCATTAT

GCTTGTTTGGTTGATATGTTAGTGAGGCTCGGACGGGTAGAGGAAGCTTTCGATTTGATA

AAGAAAATGGATTATGAGGCTAGCAAGTTTATTTGGTCAAATTTGATTGCTGGTTGTCTA

AGCCAAGGGAATCCAGAATTGGGTTGTGATGCTGCAGAAAAGTTGTTAAGTCTCAAACCA

AAAGATACAGAGACATACAAATTGCTGTGGAATGCGTACGTCTCAGCCGGACAATATGAC

GATGTTTCTAGGGTGGAGAATATAATGAAAGAAGAGAAAATTGGAGAGTTAAAAGATTGG

AGCTGGATTAGCATCAAAGACAGAGTGTATTCATTTCAAACAAATGACAAAGCAGACATA

GAAAGTTCAATAGGTAAATCATTGGAGGATTTACATATCAAAGCAAAGAATCTTGGATAT

GAGATGTTAGAAAGTGTGGAAATAAGTGACAAAGAAGAGAAAGAAAAGATATACTCACCT

ACCATTTATCACAGTGAGAAGCTAGCCATTACATTTGGGTTGGAGAATTTGCCACATTCT

TCACCGATAAGAGTTGTTAAGAATACCTTAATGTGCAGGGATAGCCATAATTTTGTTAAG

TACATCTCAACAATGACTGGTAGGGAAATACCGACAGAACTATCCTAA

>MS.gene049905.t1

ATGCTTACAACTTCGATGAAGAACACACCTTGTAACCGTTCTAAAGTCCCTCTCATGATC

AGCTATGGTGGCATAATCGTACCAATACCTTGGGGACACCAAAATCATCACTATACCTAC

ATAGGCGGTATCAACAGAATCGTTGCCGTTGATCGTGATATCAAGCTTTCCAAACTCATC

GCAAAGCTGTCTACAATGATGTATCCTAATTTTTGCTTCAAGTATCAACTTCTTGGTCAT

GATCTTGATACTTTGATCCCGGTTTTAAACGAGGAAGATTTCGATGATATGATGCTCAAG

TATGATCATATGTGTCGTATTTCGCCAAACCCTGCCAAGTTGAGGCTCTTTCTCTTCCCT

GTTCCTACTCCACCGGCTGAAGATTTACCTCCGCCTCCACGACGTCCGCATCAGAAGGTT

GCCTTTACAAAAGAGGAGATGAAGGAGTTTCAAGAACTGAATGTTGTGAATGATGGATTC

GAAAAGATTCTAAAATATGTTATGAAGTGTTTTTGA

>MS.gene049917.t1

ATGGGGTTAATACTTAATAGTGATGATAACAATGGTAGTGTATCTTGGTATGGAATGAGG

TTACCTGTTAACCCCTTTTTGTCACCGATTTCGTTTTTGTTGGATTATTCTGGAATTTTG

CGTTCGAGTAATGATTCTGAGGGTATGATTGTCAACAACGGAGTTTCTGGTTCGGAGTTA

CGATCTCAAGTTGATGCTGGTGGTGCTGTTGCTAGGAGTAGTGCAGGGAAAGTTGCAATA

AGGATTATTGGAGCTGGAGAGAATATTCATAATCAGGTTGGGGAAGTAGGTTATGATGAT

TGTGGTGAGGAGCTTATTGGTGAGAGAAGTGGAATGCCTGGATTGGGTGAGAATGATGCC

GAGGGTCGTGGTGGAATTGAGGCTAGTGAAGGGGTTCCTCTTGTGTCTTCATCTTCTTCG

TCATCGTTGGCTGGTAGTGGACAGGTTGATGGTGAGGCTGCTGGGAATGGAACGGAAAAT

AACAGGGATTCGTCTTCTTATCAGAGATATGATATTCAGCTGGTTGCCAAGTGGATCGAG

CAGATTCTTCCTTTCTCGCTTTTGTTGTTGGTTGTTTTCATTCGACAGCATTTACAAGGT

TTCTTTGTCACAATTTGGATAGCAGCTGTGATGTTCAAGTCGAATGAAATTGTTAAGAAG

CAGACAGCTTTGAAGGGAGATAGGAAAGTCTCTTTGCTTGCTGGTATCGCTTTTGCTTTC

ATTCTTCATGTTATATGCATGTACTGGTGGTATCGAAATGATGATATTTTATATCCACTG

GTCATGTTACCTCCTAACCCAACACCTTTCTGGCATGCAATATTCACCATCTTGGTCAAT

GGTATGGACTTATTCATGTAA

>MS.gene049913.t1

ATGAAACAAACAATCATCCCTTGGAAATGCCTTATAGATGACACAACTGCACCATATTCT

GAAAATTCAAACCCTAACCCTAACCCTAACTTTAATCAAGGTAACACCATAAAAACCAAC

CCTTCTCCCACCATAATCCCTTGGAAATGCCTCAAAGATGTTGAGACCAACCCTCCTAGC

ATCCCTAAACCACCAATAACCCAACAAAAACAAATCAAAACATTTGCTCAAGTAGTTTCT

CATCTTTATGATGTTCCTACTTCCCAGCTACCACAACCGGTTCTAAAGGGAGATAATTTC

TCAGTTTCAATTTTAGAAGAGGAGTACATGGTGGGATTGGAGACATGCAAATTCAATCTC

CATGCCAGAATCATCTGGCCAAAAGGATCCACTCCCCTTACAGTGTTTGACTTGCGTTCC

AAATTATATCTGCCCTATGGAAGGATCTCAGCCAGTGGGGAGTATCCTTCTTAG

>MS.gene049907.t1

ATGAATGGTGTTGAAGTTGAACGACCATTGAAACTTCACTTCATTCCGTATCCAGCATCT

GGACACATGATGCCCCTTTGTGATATAGCAACTTTGTTTGCATCACGTGGCCAACATGTC

ACAATCATCACCACTCCCTCCAATGCTCAATCCCTTACCAAAACTCTCTCATCCGCCGCC

GTCCGTCTTCACACGGTTGACTTCCCCTATCAACAAGTCGACCTCCCTAAAGGCGTTGAA

TCTATGACCTCCACGACTGATCCCATCACCACTTGGAAGATTCATAATGGCGCAATGCTC

CTCAACGAAGCGGTTGGGGATTTCGTTGAGAAGAATCCACCGGATTGTATCATTGCTGAT

TCCGCGTTCTCATGGGCTAATGACTTGGCCCACAAGCTTCAAATCCCGAATTTAACATTC

AATGGATCGTCTCTTTTTGCTGTCTCGATCTTTCATTCCCTTCGAACAAATAATTTACTT

CATACAGATGCTGATGATGATTCAGATTCAAGTTCCTATGTCGTTCCAAATCTTCATCAT

GATAATATCACCTTGTGTAGTAAACCACCAAAGGTTCTCAGCATGTTCATAGGAATGATG

CTTGACACAGTGCTTAAAAGTACAGGCTACATCATCAACAACTTTGTTGAACTTGATGGA

GAAGAGTGTGTCAAACACTACGAGAAAACCACGGGTCACAAGGCTTGGCATCTTGGTCCA

ACTTCCTTTATTCGTAAAACTGTTCAAGAGAAAGCAGAGAAGGGAAATGAGAGATTTCGG

TCGATGATTTTGTTTTACTTATCATTGCATATTTCAATTTTCACTGTGGTACTACGTTGA

>MS.gene049918.t1

ATGGACCTAGGGATGTTTCCTGAAGACCAAAGGATCTATGATCCAACCTGGATGGACATT

TGTGCTGAACTGTATGAGCTAGATGAAAACGGAAAGAAAGGAAAGACTCCATTTTATTCT

GCTTATGATCTTGACATGGTTGATCTTCCAAGGTGGATTGAGTCTGTGGTTTGTGAGGAA

TGGACGGCTGAAGTTTTCAATGAGGAGATGATGAGAGGAAGAGAAAATGTTGAAGAGGAG

ATGGTTGAGATGCTTCAGATTGTATTGGCTTGTGTGTCAAAGGTGGAAGGTAACAGGCCA

ACAATGGACAAAGTTGTTAGAAACATAGTAGAAATTAGCCAGCCTAAGTTGAAAAAAGAG

TACTTCATCTTCTGA

>MS.gene049906.t1

ATGGAGCATACACCTCGTAACGGTTTCAAAGTCCGTTTCATGATTAGTTATGGTGGAATA

GTCGTCCCTATACCTTGTGGATACCAAAATCATCGCTATATTTACATAGGTGGTGACAAC

CGAATCGTCGCCGTTGATCGTGATATTAAATTGTCCAACTTTTTCACAAAGCTTTCTGCC

ATGATGGGTTCTGATTTTTGTTTCAAGTATCAACTTCCCGGTCAAGATCTCGATACTTTG

GTCCCGGTCTTAAACGAGGAAGATTTCGATGATATGATGTTCAAGTATGATCATATGTCT

CGTATTTCGCCAAATCCTGCCAAGTTGAGGCTCTTCCTCTTCCCTGTTCCTACTCCACCT

GCTGAAGATTTACCTCCGCCTCCACGACGTCTGCATCAGAAGGTTACCTTTACAAAAGAT

GAGATCAAAGAATTTCAAGAACTGAATGTGGTGAATGATGGATTCGAAAAGATTCTAAAA

TATGTTATGAAGTGTTTTTGA

>MS.gene049914.t1

ATGGCCATGGCACTTCGAAGACTCACTTCCACCATCAACAAACCAACTTCTCTCTATCGC

TTGTCATCTTCTCTCTCCGCTCAACACACTCACAAATCTCACCCCGATTGGATTAAACAG

CTCAACGATCCTCTTGGTGTAGTGGATCCTGAGATCGAAGATATAATCGAACTTGAAAAA

GCTCGTCAATGGAAGGGACTAGAGCTTATACCTTCAGAAAATTTTACGTCGTTGTCGGTG

ATGCAAGCGGTTGGATCGGTTATGACGAATAAATACAGTGAAGGTTATCCTGGTGCTAGA

TACTATGGTGGAAATGAGTACATTGACATGGCTGAGACTTTGTGTCAGAAGCGTGCATTG

GAAGCTTTTGGGTTGGATCCAGCACAATGGGGAGTCAATGTGCAGTCATTATCTGGATCT

CCTTCTAACTTCCAAGTTTACACTGCTTTATTGAAACCTCATGAGAGAATTATGGCACTT

GATCTCCCCCATGGTGGGCATCTATCACATGGATATCAGACTGACACCAAGAAGATATCA

GCTGTATCTATATTCTTTGAAACAATGCCATACAGGTTGGATGAGAGCACTGGTTATATT

GACTATGACCAGATGGAGAAAAGTGCTGCACTTTTTAGGCCAAAATTAATAGTTGCCGGT

GCTAGTGCTTATGCCCGTCTTTATGATTATGCGCGTATTCGCAAGGTCTGTGATAAACAG

AAGGCAGTTATGTTAGCTGATATGGCACACATCAGTGGATTAGTTGCTGCAGGTGTTATT

CCTTCTCCTTTTGATTATGCAGATGTTGTAACAACCACAACACATAAGTCACTTCGTGGG

CCACGTGGGGCTATGATATTCTTCAGGAAGGGTCTGAAAGAGATAAACAAGAAAGGGCAG

GAAGTGCTGTATGACTACGAAGACAAAATAAATCAGGCTGTTTTTCCTGGGCTTCAAGGT

GGTCCTCACAATCACACTATTACAGGCTTAGCAGTTGCATTGAAGCAGGCTATGACACCG

GAATTCAAGAATTACCAGAAACAAGTTCTTAGTAACTCCTCGACATTTGCACAGAGCTTG

TTAGAGAAAGGCTATGACCTTGTATCTGGTGGAACTGAGAACCATTTGGTGTTGGTAAAC

TTAAGAAACAAGGGCATTGATGGCTCAAGGGTTGAGAAGGTGTTAGAATCAGTTCATATA

GCTGCCAATAAAAACACTGTTCCAGGAGATGTGTCTGCAATGGTTCCGGGAGGGATCAGA

ATGGGAACTCCTGCTCTTACATCTAGGGGTTTTGTTGAGGTTGATTTTAAAAAAGTAGCT

GAATACTTTGACGCGGCTGTCAGAATTGCCTTACAGATTAAGGAAAATTCTAAAGGCACA

AAGTTGAAGGATTTTGTGGAAGCTATGGAGTCAGATTCACAAGTTCAATCTCAAATCGCT

GATCTCCGCCGTGAAGTAGAAGGTTATGCTAAGCAGTTTCCCACAATTGGGTTCGAGATA

GAGACAATGAAGTATAACAAGTGA

>MS.gene049916.t1

ATGAATTTGGTTATTATTGATTTTTGCAGGATTGTTGATGAGACTACTAAAGAAGCTGCG

GTGGTGGACCCAGTTGAACCTGAGAAGGTTCTAGAAGCTTCTAATTCGCTTGGTCTCACT

CTCAAATTCGTCCTTACTACTCATCATCACTGCCACACCAAAGGTCACATAAGTTATTAT

GTTACTGGCAAAGAGGATGAGGACCCGGCTGTTTTTACTGGAGATACATTGTTTATTGCT

GGTTGTGGGAATTTTTTTGAAGGAACTGCAGAACAAATGTATCAATCACTCTCTGTAACA

TTAGGTTCATTACCAAAGCCAACTCGATACTCAGTGAAGAACCTGCAATTTGCTCTGACA

GTTGAGGCAGACAATTTAAGGATACTGGAAAAATTAACCTGGGCTCAAAATCAGCAGCAA

ACTGGCCAACAAACAATTCCTTCAACCATTGGGGACGAGTTGGAAAGCAATCCATTTATG

CGGGTTGATCTACCTGCAATCCAGGAGAAGATGGGGTTCAATTCACCAGTTCAAGCTTTA

GGAGAATTAAGGAAGGTGAAAGACAATTGGAGGGGCTAA

>MS.gene049900.t1

ATGGAGCACACCAAGATCCCTCTCATGATCAGCTATCGTGGAACAATCGTCCCGATGCCT

TGTGGACCCCAAAATCATCTTTATACCTATGTAGGCGGTATCAATAGAGTCATCATTGTT

GATCGTGATATCAAGCTGTCCAGCCTCATTCAAAAGCTTTCCACGATGGATTCTAATTTT

TGCTTCAAGTATCAACTTCCTGGTCATGATCTCGATACTTTGATCCCGGTTTTAAACGAG

GAAGATTTCGATGATATGATGCTCAAGTATGATCATATGTGTCGTATTTCGCCAAATCCC

GCCAAGTTGAGGCTCTTCCTCTTCCCTGTTCCTACTCCACCGGCTGAAGATTTACCTCTG

CCTCCAAGACGTCCGCATCGGAAAGTTAGCTTCACAAAAGACGAGATGAAGGAATTTCAA

GAACTCAATATTGTGAATGATGGATTCGAAAAGATTCTAAAATATGTTATGAAGTGTTTT

TGA

>MS.gene049904.t1

ATGGAGCACACCAAGATCCCTCTCATGATCAGCTATCGTGGAACAATCGTCCCGATGCCT

TGTGGACCCCAAAATCATCTTTATACCTATGTAGGCGGTATCAATAGAGTCATCATTGTT

GATCGTGATATCCAGCTGTCCAGCCTCATTCAAAAGCTTTCCACGATGGATTCTAATTTT

TGCTTCAAGTATCAACTTCCTGGTCATGATCTCGATACTTTGATCCCGGTTTTAAACGAG

GAAGATTTCGATGATATGATGCTCAAGTATGATCATATGTGTCGTATTTCGCCAAATCCC

GCCAAGTTGAGGCTCTTCCTCTTCCCTGTTCCTACTCCACCGGCTGAAGATTTACCTCTG

CCTCCAAGACGTCCGCATCGGAAAGTTAGCTTCACAAAAGACGAGATGAAGGAATTTCAA

GAACTCAATATTGTGAATGATGGATTCGAAAAGATTCTAAAATATGTTATGAAGTGTTTT

TGA

>MS.gene049901.t1

ATGCTTACAACTTCGATGAAGAACACACCTTGTAACCGTTCTAAAGTCCCTCTCATGATC

AGCTATGGTGGCATAATCGTACCAATACCTTGGGGACACCAAAATCATCACTATACCTAC

ATAGGCGGTATCAACAGAATCGTTGCCGTTGATCGTGATATCAAGCTTTCCAAACTCATC

GCAAAGCTGTCTACAATGATGTATCCTAATTTTTGCTTCAAGTATCAACTTCTTGGTCAT

GATCTTGATACTTTGATCCCGGTTTTAAACGAGGAAGATTTCGATGATATGATGCTCAAG

TATGATCATATGTGTCGTATTTCGCCAAACCCTGCCAAGTTGAGGCTCTTTCTCTTCCCT

GTTCCTACTCCACCGGCTGAAGATTTACCTCCGCCTCCACGACGTCCGCATCAGAAGGTT

GCCTTTACAAAAGAGGAGATGAAGGAGTTTCAAGAACTGAATGTTGTGAATGATGGATTC

GAAAAGATTCTAAAATATGTTATGAAGTGTTTTTGA

>MS.gene049893.t1

ATGCCTCCCAAACCTGCAAACAAAGATCTGGATGAAGTAAATCAGCAGCTGGGAGCTTTG

CAAACCCAGCTGGAACATCAACAGATTATGCACGACACTCGTCATGAATCTCTGCAGCAA

GCCATGGCAACTGGGCAAGAATCTCTTACTACCATGATGAACAGCTTAAAGGAGCAGATT

GCTTCTATGGGTCTGCGTTCACAACAGTCCCTTCCCTCTTCATCAGGTATAACAAAATCC

ATTCCTCACTCACTTGGTTTAGACAGTTCGAACAATAATTCTGCTGCCTTCACTTCACCC

ACACAACCCCCTTCACCAGATCTGGCTGACCAATTTTTCAAATTTTACCAAATTCCACCG

GAACAAAGACTTCCATTGGTCAGTTTTTACATGAAAGGTGACGCTTTAAGCTGGTTTAAA

TGGATGTATGACAACCAACAACCATCTGATTGGCCGTCTTTTACTCGCAACTTGGAGTTG

CATTTTGGCCCCTCAACTTTTGAGAATCACCAGGCTGAATTGTTTAAATTGCGCCAAACC

GGCAGTGTTTCCGACTATCAAGCTCAGTTTGAAAAGTTATGCAATCGTGTTTTAGGATTG

TCACCTACTGCTATCCTCAACTGTTACATTTCCGGCCTTACCCCTGAAATTCGTAATGAT

ATTGCGGTTCACAAACCTCAAACAATCACAGAGGCAATTGGCTTATCTAAACTACTTGAG

GCAAAAATTAAAGACACCAAACCAAAGTTTTTCAAACAATTTTCCCCAAACCCCCCTAAC

CCACAAAAGCCAGCCACACATCCACAAACACACTTAACCAATCAACCTATCCCACCCCCT

AAAACTGCTACCCCTACCCTCCCAAACAGGCAACAATCACTAAACCCAAATACCTTCCCA

ATTAAACGTGTGACTGCCGCACACATGCAAGAGCGAAGAGCACAAGGTCTTTGTTACAAT

TGCGATGAAAAATACATCGTTGGTCATCGTTGTGCAGCTGGAAATTTTTTATTGTTACTT

GAGGATGATCCTCCTGACTCCACCGCGGATGACCAAACTGCCGCGTTAGAAAACCTATCA

GCACCTGATGAACCACTCAAGCATTGA

>MS.gene049929.t1

ATGGATACTGAAGCGCGGAAAATTCTGCACTATGCAGCAGATCAAAACAAATCCCTTTGT

TCCTTCTGGAAATTGTGCATTTTCATAGCTGAAGTTGCATTTCGAGCGGACAATAGCAAG

TTAGACTATTATGGCCTGGAGTTTATGGCTCGATGGATTGTAAAGGGTGAACGTTCAAGA

CCTCCCATGTTGCTTTATGTAGATGGTTTAGTCGTGTCAGCTCTGATGACTGCTGGTAGA

ACCTATAATTCTGAGCTGCTAGGAGCAGCATGGGCTGTTTTGGACCTTTCCTTGCGTAAA

AAGAAGCTTCCAAATCCAGAATCTTATCTCACAAAGATACATGCCCTTGCATCATTGGGC

CATCTACAAAAGGCAGTTGGCACTCTGCATGATTATGAGAATGCTTATGGAAATTCTGAT

CAAGAAGCTGACAACCTCTTCTTTTGGTTAACTGTATTATCCTAA

>MS.gene049931.t1

ATGGATACTGAAGCGCGGAAAATTCTGCACGATGCAGCAGATCAAAACAAATCCCTTTGT

CCCTTCTGGAAATTGTGCATTTTCATAGCTGAAGTTGCATTTCGAGCGGACAATAGCAAG

TTAGACTATTATGGCCTGGAGTTTATGGCTCGATGGATTGTAAAGGGTGAACGTTCAAGA

CCTCCCATGTTGCTTTATGTAGATGGTTTAGTCGTGTCAGCTCTGATGACTGCTGGTAGA

ACCTATAATTCTGAGCTGCTAGGAGCAGCATGGGCTGTTTTGGACCGTTCCTTGCGTAAA

AAGAAGCTTCCAAATCCAGAATCTTATCTCACAAAGATACATGCCCTTGCATCATTGGGC

CATCTACAAAAGGCAGTTGGCACTCTGCATGATTATGAGAATGCTTATGGAAATTCTGAT

CAAGAAGCTGACAACCTCTTCTTTTGGTTAACTGTATTATCCTAA

>MS.gene049934.t1

ATGGATACTGAAGCGCGGAAAATTCTGCACGATGCAGCAGATCAAAACAAATCCCTTTAC

CCCTTCTGGAAATTGTGCATTTTCATAGCTGAAGTTGCATTTCGAGCGGACAATAGCAAG

TTAGACTATTATGGCCTAGAGTTTATGGCTCCATGGATTGTAAAGGGTGAACGTTCAAGA

CCTCCCATGTTGCTTTATGTAGATGGTTTAGTCGTGTCAGCTCTGATGACTGCTGGTAGA

ACCTATAATTCTGAGCTGCTAGGAGCAGCATGGGCTGTTTTGGACCGTTCCTTGCGTAAA

AAGAAGCTTCCAAATCCAGAATCTTATCTCGCAAAGATACATGCCCTTGCATCATTGGGC

CATCTACAAAAGGCAGTTGGCACTCTGCATGATTATGAGAATGCTTATGGAAATTCTGAT

CAAGAAGCTGACAACCTCTTCTTTTGGTTAACTGTATTATCCTAA

>MS.gene049922.t1

ATGTCAACTCAGCCATCAGCGTCTGGACCAGTAAAGTGCACTGTCAAGTTGGAGAATGAT

GGTTCTGTCCACGACGCTTGGTACTTGATACCAGTCGAGACAATATCGGAACTATTTCGC

TACTTCAACGAATATTTCATAAGGTACTATGAACCAACTCGTAGGGTGAAAATTTTTTAC

TGCATAACGCCATACTACTCGTTGGAAACTGTCCATAACGACTGGAAATTAAGCTACGAT

GTCAAGCATCTTGAAACCGACCGTGAGGTTGAAGACATGTTACGATGGACAACAAACGTA

GGGGACCCTTTGTATTTGTATGTTACAATTCAATTATCTAACTAG

>MS.gene049921.t1

ATGGCTTCTAAGGGAAAGGGTGTTCATGTTGGCAGTACTTCTTCCGGAACGCAGAAACAG

GGTAGACTCAATGAGAAGGGACTTATTCTTACCAAATCTGAAAAGGTAAGGTACAGAATG

CTTGTGAACAGAGACATTGTACCCAATAGGTACCCTGATTCAACCGCTCTACAGGCATTG

GGTATAGAGGACATTGTCACGACACTACTTAATAACGTAGGCTGGAAAGATTTCGTAGGC

GAGACGCACTACGCCTTTGAAACTAACCATTTGGCATATGTGGTGGCACAACCAAAAGGT

AAGATAGTGGCGGGGGGATTGGTAAGTTTCATTGGATGGAAGCTGGGAGCGAGAACTGCA

ACACAAGAAATTGGCATTGAGGGGAACTATACCATTGATATCGAGTTCTGTAAAAATATA

CATATGGTAAGGGATCTAGATGGGGACAACAAGAATTTTATATTGTTGGTATTCGACGAA

GATTCAATCTTGTTGCCAGATCCATCAAGAACTGACGTAAGAAATCCAGCAAATTGGCTT

TATATGGATTTAGATTCACGGGTACCTAAAGAGCAGGCTGGCAAAGATGAAGAAATGGGT

GATTACCGGGAGGAGCAACAACCTGCGGCTGGAATGAAAGACGACGATGATTGGCGTCGA

AGGATGGAGGCGAAGGCTGACAGGACGTATGATGAGGTTGCATGCATCAAACAAATGCTT

GCAGCAACGATGCAGCAACTCAACTTCCAATATCCTCCACCCCGTCCAGAATAA

>MS.gene049933.t1

ATGGTTGTTGTTCACGGCCTTCAAGGTTTCGCCTCTACACTTTCTCCGTTTTCTTGCGAC

GTTGAGAGAATGACTTCACTCTCCTTTCCGTTTCATCCTGATCACCGTTCAAGTGCTTAC

GTTCACGCTCCCGGCGCCGCCGTGCTTCCTGCTGTTATGCGCCTCATGCTACGAGTTTCA

ATGCTCAGGCTTCGTAATAACGGTTTCAGCATCCTCTCCCTTGCGACGAAGTTATGA

>MS.gene049928.t1

ATGTTTTTAAAAGGAGGAGAACCAATTCTGGTTATTAGGGTTTGTAATAACATCCCTACT

ATTGTTACATACCGAAGAAAATCAAAACATACCTATGAATCTGATTTGGAATACTATGAG

CGTAAATATTACACAAAATTGAAGGATGATTACTACAAAATTGAAATCTCAGATTCGATA

TATCGTTGTCCGTTTTGTTACAGCAAAGATTACTCCTTGTCCGAACTTTTGAGACATGCT

TCCAGAATATCAGGTAACTCACGTAAGCCGATTAAAGATATTGCTAGACATACTGTTTTG

ATAACATATATTCTAAGAATTCTTACGACGTTGAAAGAGGAGATCGACGTTGCTCGGACA

AACGAAGCGTTTCCGATGTCCGTGGAACCGGTTGAAGAACATTCAGTCACCGAACCTATT

GTTAATATTGAGGAGACTGATGAGCACGTGGAAGTGCAAACTCACTTTTCATATTCTCAG

TCTGCAAGAAATTCCTCTATGTCAGATGTAAATAGTTCTCCCAAGAAAGATAAATCTTGT

TTCCCTCATTCTGATTCAGTGAGCACTGTTGTTGAAATTATACAATCAAAACCTATTCTG

TCAGCAACTTCTGAATATTATGAATCGGAAATTGAAGGCACGCCATCCAACCAACTTCCC

AAAATTGTTGAGTTTCAAAATACAGATGGTAGGAAGATTGCCATGCCTGATGATGCACAT

GTTCAGGAAGAAAAGGATTCTGACTCTGGGAAAACATATTCGGATATGTTGACTTCAGGA

AAGCTGTCGTGTTCTGATCTTGAACCTTCTTCTTCTCATATAAGGCACGCATCAGATGTG

AAGGAGACTAAGAATTTCATGTTTGTTCCAACTATTAATCCAAATGATGGAGGAGTTTGC

AACGCCGTGTCAAACACTCATGCGTTCAAGGAAGGTGATGATTCAATATCAACACGTCAC

CCATATAACAAAGGAGATGGCAATTTAATATTGGGGAATGGTGTTTTTGACAGGACATAG

>MS.gene049923.t1

ATGGCTGAAGAAAATGATTTCCAACCTGAAGAACTTCAACATCCTCCTTCATTAAGACAA

CATATAATGGATGGCGAAGATGACTTTGATGTATCGTCGCCTGCACCTACCCCAAAAGGA

TTAACTCTAGAAGATGTTATGGAAGAGATCAAAGGCTTGAAACTAATTGTCTCTGAAGTG

GCTAATAAACAAAACACAGCTGAAAAGTATGGTACTATTTTGGCAATGTTGCTTGGTATT

AAATTTTTCTGTTGTATAACAGGGAATTACGGATCAGGTGGTAGGATTACGGGGTTCTCT

CTCTTTGCCGTTCAAGCCACTGTTGAAGCTCTGGAAGTGATTGCAGTTTCACAAGGGAAA

GATGCTTCCACTAACTTTGAGAAGCAACCAATTAATGACCCAGAATTTCTTACTGAACAA

GAACGTTTCGATAAGGAACTTGCTGACATGGACTGGAGTTCTAAATCTGGAAATGACGAT

GACTACTTCGGCGTAGGGAGGTTTGGGTGTAGCAGCAGCGTGTTCGCTGGAGTGGAAAGT

ATGGCTGATCGTAGTGTTGACGTAAGTGTGTTGCAGAGGAATGTTTCAAAATCTGTTGAG

AGACCATTCATCCCTGATTTCTGGTTAAAGGAAGATACACCAGCTAAGAGCTATAAGACT

GAGAAACAAACTTTGCTGGTAAGTCGTAGCGACTTTAAAAGACACACGCGAAGTAGTCAG

ACACCTCCAACATTCTCTGGAGATGGTGTAAACAAGAAATTGTTTAAAGAAACCCCTTCA

CCAAATCCAACACCAGTTGGTTCTTTGAAAACAAGTACCAAACTGTCTCAATCCGGGAAG

AAGGTCACGGTAAAACTGGAGGAATCTACGCCAAAAAGGACAAGGAAGTCGGTGGGGGAA

ACGTCAAAGGGAAAGGATACAGCTGACCCAACTTCATTAGAAATGAAAAGACTTGTTGTT

AGAGATGCTCTAGTGTGCACCTATCTTTTTCATCATGCATCCGATAGTAATTTGAGAAAC

GAGACACTTGTCTGGACAATGAGGTTGACGGCAACAAGAGCTGAACTTAAATGTCTGACG

CCTGGTGAACCCATTAATGAAGTGGTATGA

>MS.gene049924.t1

ATGACTTTGTTTACGAAAGTTTCAAAAGAAGATCTGCTGGTGAAACTTAACGATGTGTGG

GTGCGCTCACTCAAACTAACGGTTAATGTGTTCAAGTTTGAGAGAGAGTTGGTATCTGTT

GTTCCGGAGGAGGCTGTGACTTCGGTTAGGGTCCATTCGCAGGGTGGGGTTTCTAATCAC

AATCTCGTTGTGGAAGGTAATTCCTTTGCAGCGGCGACAGCGTCGTCTCCGGTGGTAAGG

TCGACGTCCGTTCAAGGGATGCTGGAGATCTCCTGTGAAGTGCCAGCAGGAAGGATGCAG

GAGCTGTCCGAGAGTGTCATCGGGTTATTGCTACCACACGTGAATATGCAAATGATCAGG

GAACAACTTGTTATTGCTGGGATGGGGAATATCAATGTCAAGTTTTTGGGTGGAGACCAA

GTGCTCATTAGCTCTCCTATCAGTGGCATTTTAAAAGAATCCCTGCTTCTCAGGAAGGAC

TGGTGGCTTCTGTGGTTCAGCGGCTTTTCTCGGTGGTTTGTTGATTTGCCTCTACCCGGT

CGCTGTGTATGGTTGGCTATCTATGGGGTTCCTGTCCATCTGTGGTGTTTGGAAGTGTTT

CACAAGGTGGGAGCCCTCTTTGGAGTAGTTCAGGAAGTGGCAGAGTTCACCTCATCTTTT

GAGCTTGCAAGGGTTAACGTGCGTCTCCCTAACCCTTTTCCGGTGTCAGCGTTGGTATCT

TTGTTGGCGGAGGGTTGGATGTTTGTTGCGTTTGTTAGGGATGAGATCCCACCACATCAT

CATTTTGCTGGATTCTTTCCAGCACCGGCAGGATCTGCGGTGGCCGGTGAGATGTTCGCC

GGAGATGGTGGTGTCTCCGATGAGGAAGCTTAG

>MS.gene049926.t1

ATGTCCGTGGAACCGGTTGAAGAACATTCAGTCACCGAACCTATTGTTAATATTGAGGAG

ACTGATGAGCACGTGGAAGTGCAAACTCACTTTTCGTATTCTCAGTCTGCAAGAAATTCC

TCTATGTCAGATGTAAATTGTTCTCCCAAGAAAGATAAATCTTGTTTCCCTCATTCTGAT

TCAGTGAGCACTGTTGTTGAAATTATACAATCAAAACCTATTCTATCCGCAACTTCTGAA

TATTATGAATCGGAAATTGAAGGCACGCCATCCAACCAACTTCCCAAAATTGTTGAGTTT

CAAAATACAGATGGTAGGAAGATTGCCATGCCTGATGATGCACATGTTCAGGAAGAAAAG

GCTTCTGACTCTGGGAAAACATATTCAGATATGTTGACTTCAGGAAAGCTGTCGTGTTCT

GATCTTAAACCTTCTTCTTCTCATATAAGGCACGCATCAGATGTGAAGGAGACTAAGAAT

TTCATGTTTGTTCCAACTATTAATCCAAATGATGGAGGAGTTTGCAACGCCGTGTCAAAC

ACTCATGCGTTCAAGGAAGGTGATGATTCAATATCAACACGTCACCCATATAACAAAGGA

GATGGCAATTTAATATTGGGGAACGGTGTTTTTGACAGGACATAG

>MS.gene049935.t1

ATGATTGATAAGAAGGTTAATCAGTTCTTCTGTTCTCATAAGGAACGAGCAAGTGTTCTT

GCCGCTAACAACTTGAAAGTCAGGCAACCTGGTGATCTGAGTGATAAACTTGAAATTGTT

TCACATAAGAAGTTGAACAACTTTGTGCACATGGCTTCACACTCATTAGGACACACCAAT

CTGGTTCTTCAGCCTCAAACCCACATAAACACGCCAACAACACACAAAAACTCCTTTCCC

ATTACTACCTTCTCTCTCAACCCTCCCGAGTTCAAATATATACCACCTTTTGTACCTTAT

CCTTTTCAAACAGCTCATCCGTACATCCTTACCGATCAGAACCAACAACTTCAGACCAGC

AAAAACTCAGACCATGTACTCAAACAAGCCATGGCCTTTGAACACCTAGAAACCAACACT

TCAGTTCCTTTGATGGTTGAACCAATCACTACTGTTCTGCCCAGAAAGGTTAAAAGGAAA

TCACCCACTAACTCCAGTCCAAAGAAGAAGAAACGTGATAAATCTGATGTGGGAACACCT

GTGAGAACCTTATGTATGGTTGACCGAGCAACTCCATTTGTTACTACTGCTGTCAAACCC

AATGTTGAGACTTATGTGAAGAATGCTCACAGTATTAATGTTGAAGGATTCCTCAAAACT

ACTTCCTCTTCTGAGAATGATGAAGGTAACTCTAATGCCTCTCTGATTTCTGATACACCT

GTATCTAGTGAAAAGTCAGTTTTGGAGAACTTGTGTGTTTTAAATAGAACTGTGCCCATA

GACATGGATCCTTCTAATGATGGTAAGGTGACAAACTTCAATGTTCCTGCACAAGAATAT

GGAGACATAAATGTTGAGGGAAATGGTGAAACATCCTTGAGCAAATTTGATAACCATGAG

AATACTGCTACCATTGGTGTAAATAAGAATTCTGGTTCTGGGACTACTTCAGCAGAGGAA

GTGAACTGTGAAAAGATGATTGAAGAAGGCCTTGCTAATAATGAATCAAAAGAGCAACTG

ACAGATATCTTTACTAAGGCTTTGGATGCTGAACAGTTTGAACATCTAAGAGGCAAGTTA

GGAATTTGCCTACATGATGAAGCCTAA

>MS.gene049930.t1

ATGATTTTAAATCATTGTAGAAACCTGGCAGATAGGGATTTCTTTGTCAGTGTGGTGAAT

CCCGAAGAGGTATGTAACATATCAATATTTGAACTTGACTATTTGAATTTGAAGAGAGGA

GGAGAACCAATTCTGGTTATTAGGGTTTGTAATAACATCCCTACTATTGTTACGTACCGA

AGAAAATCAAAACATACCTATGAATCTGATTTGGAATACTATGAGCGTAAATATTACACA

AAATTGAAGGATGATTACTACAAAATTGAAATCTCAGATTCGATATATCGTTGTCCGTTT

TGTTACAGCAAAGATTACTCCTTGTCCGAACTTTTGAGACATGCTTCCAGAATATCAGGT

AACTCACGTAAGCCGATTAAAGATATTGCTAGACATACTGTTTTGATAACATATATTCTA

AGAATTCTTACGACGTTGAAAGAGGAGATCGACGTTGCTCGGACAAACGAAGCGTTTCCG

ATGTCCGTGGAACCGGTTGAAGAACATTCAGTCACCGAACCTATTGTTAATATTGAGGAG

ACTGATGAGCACGTGGAAGTGCAAACTCACTTTTCATATTCTCGGTCTACTAGAAATTCC

TCTATGTCAGATGTAAATAGTTCTCCCAAGAAAGATAAATCTTGTTTCCCTCATTCTGAT

TCAGTGAGCACTGTTGTTGAAATTATACAATCAAAACCTATTCTATCCGCAACTTCTGAA

TATTATGAATCGGAAATTGAAGGCACGCCATCCAACCAACTTCCCAAAATTGTTGAGTTT

CAAAATACAGATGGTAGGAAGATTGCCATGCCTGATGATGCACATGTTCAGGAAGAAAAG

GCTTCTGACTCTGGGAAAACATATTCGGATATGTTGACTTCAGGAAAGCTGTCGTGTTCT

GATCTTGAACCTTCTTCTTCTCATATAAGGCACGCATCAGATGTGAAGGAGACTAAGAAT

TTCATGTTTGTTCCAACTATTAATCCAAATGGTGGAGGAGTTTGCAACGCCGTGTCAAAC

ACTCATGCGTTCAAGGAAGGTGATGATTCAATATCAACACGTCACCCATATAACAAAGGA

GATGGCAATTTAATATTGGGGAACGGTGTTTTTGACAGGACATAG

>MS.gene049932.t1

ATGGTTGTTGTTCACGGCCTTCAAGGTTTCGCCTCTACACTTTCTCCGTTTTCTTGCGAC

GTTGAGAGAATGACTTCACTCTCCTTTCCGTTTCATCCTGATCACTGTTCAAGTGCTTAC

GTTCACGCTCCCGGCGCCGCCGTGCTTCCTGCTGTTATGCGCCTCATGCTACGAGTTTCA

ATGCTCAGGCTTCGTAATAACGGTTTCAGCATCCTCTCCCTTGCGACGAAAGTGAGATTC

TTGAGGAAGAAGGATCAAAACGGTGCCGTTGGGGAAAAGGAGATCTGCGATGCTGTTGTT

GCTGATGAGAGACAAGAGGGATCGTGA

>MS.gene049925.t1

ATGGAATCCATCAATGTAGTGGTAGATGATATAGTTAAAGAACAAATGCTTGAAGTTGTA

CCTGGTGTTGAAGCATATCCTGAAACATCTATTCAAGAGAAAGTAAGGCATGAAGCTGAT

GAAGAACCTCAGACTAACTCTGAAGATATTGATCTAGAGAATGCAGAATCAAGTAAAGGA

CCTTCAATCAGAGTTCAGAAGAATCATCCTCAAGAGCTCATTATTGGAGATCCAGATCAG

GGAATCAGAACCAAGAGATCTAATGAGGTTGTCTCCAATTCTTGGGGAGTAAAAGATATC

TGTTTGTGCTCCTTGAGGGGGAGTAAACTTTTGTCCCTGAATGTTCCTGTGGATGTTGGA

ACATCCTGA

>MS.gene049927.t1

ATGCTGGCAGCAGATCAAAACAAATCCCTTTGTCCCTTCTGGAAATTGTGCATTTTCATA

GCTGAAGTTGCATTTCGAGCGGACAATAGCAAGTTAGACTATTATGGCCTAGAGTTTATG

GCTCGATGGATTGTAAAGGGTGAACATTCAAGACCTCCCATGTTGCTTTATGTAGATGGT

TTAGTCGTGTCAGCTCTGATGACTGCTGGTAGAACCTATAATTCTGAGCTGCTAGGAGCA

GCATGGGCTGTTTTGGACCGTTCCTTGCGTAAAAAGAAGCTTCCAAATCCAGAATCTTAT

CTCACAAAGATACATGCCCTTGCATCATTGGGCCATCTACAAAAGGCAGTTGGCACTCTG

CATGATTATGAGAATGCTTATGGAAATTCTGATCAAGAAGCTGACAACCTCTTCTTTTGG

TTAACTGTATTATCCTAA

>MS.gene049936.t1

ATGGAGAAAAGTGCTGCACTTTTTAGGCCAAAATTAATAGTTGCCGGTGCTAGTGCTTAT

GCCCGTCTTTATGATTATGCGCGTATTCGCAAGGTCTGTGATAAAAAGAAGGCAGTTATG

TTGGCTGATATGGCACACATCAGTGGATTAGTTGCTGCAGTTGTTATTCCTTCTCCTTTT

GATTATGCAGATGTTGTAACAACCACAACACATAAGTCCCTTCGTGGGCCACGTGGGGCT

ATGATATTCTTCAGGAAGGGTCTGAAAGAGATAAACAAGAAAGGGCAGGCTGTTTTTCCT

GGACTTCAAGGTGGTCCTCACAATCACACTATTACAGGCTTAGCAGTTGCATTGAAGCAG

GCTATGACACCGGAATTCAAGAATTACCAGAAACAAGTTGTTACTAACTCCTTGACATTT

GCACAGAGCTTGTTAGAGAAAGGCTATGACCTTGTATCTGGTGGAACTGAGAACCATTTG

GTGTTGGTAAACTTAAGAAACAAGGGCATTGATGGCTCAAGGGTTGAGAAGGTGTTAGAA

TCAGTTCATATAGCTGCCAATAAAAACACTGTTCCAGGAGATGTGTCTGCAATGGTTCCT

GGAGGGATCAGAATGGGAACCCCTGCTCTTACATCTAGGGGTTTTGTTGAGGATGATTTT

AAAAAAGTAGCTGAATACTTTGACGCGGCTGTCAAAATAGCCTTACAGATTAAGGAAAAT

TCTAAAGGCACAAAGTTGAAGGATTTTGTAGAAGCTATGCAGTCAGATTCACAAATTCAA

TCTCAAATAGCTGATCTCCGCCGTGAATAG

>MS.gene049958.t1

ATGGATTCGGAATGGGAAACTCTTCATATAATCTTAACAAAATCACTCACCATCCTTTCC

TGGCCTCCGATTACTTTGCTTTGTCCTTTATATGTTTCTATTCGGACGTTGGAGAGTGAT

TGTCGTTACAGTAATCAACGATGTCTTGTTTTTTGGGTTCTCTTTGCTTTCTCTATGATC

ATTGAACGTGAATTTGCAGTGTTATTTACTTGGCTCCCATGGTGGTCTCATGTGAAAGCT

GTGGCAACTATCCTATTGTTAATACCTTATTTTGGTGCTGCGCAATACATCTACAAGCTC

TTAATTAAACGCTACTGTACTGGGAACATGTGTGCATGGACGAGGACGATGACCATCTTC

AATCTGAAAATCACACATTTTGAATTGAGCGAGGATGGAAACATACTTTCTGAATCGGAT

ACGGGCAGAAAAGCATTTTTAGAATCGGATGGTAATAGCAAAAGTGTTGAAGAATCGGAT

GATAATAGCAAAAGTGTTGAAGAATCGGATGATAATAGCAAAAGTGTTGAAGTCTCTGCC

CAGACAATTATTACAACTCATTTACAAGAGAAGAAACTTTTAGTATATCAGGGGAGAGAT

GATCTTGGAGCTGATTGTGACAAGACAAACAGCGGTTATTATACAAGTAAAAAGAAAGTC

CAGAAGGAATGGAGTTGTGCTTTATGTCAAATTAGTACTGCAAGTGAAAATCACTTAGGG

TCACATCTTCAAGGGAAGAAACACAAGGCTAAGGAAAAAGAACTTCGAGTTGGGTTACAT

GCAACAAATATACCGTATGTTCTATCCTTTACGCAGCAAAGAATCAAAGGAATGGTTCTA

CTTAAGAATCTCAACCAAATTGCAAATATTCTGAATCCAGTTTCTAGATCCATAATATGG

TGTGAATGGAAAAAGCCGGAATTCGGATGGACAAAACTCAATACCGACGGTTCTGTAAAT

AAAGAAACAGCTGGTTTTGGTGGTCTGCTTCGTGATTATCGGGGTGAACCAATTTGCGCA

TTTGTCTCGAAAGCTCCCCAAGGAGATACTTTCTTGGTGGAACTTTGGGCTATATGGAGA

GGCCTTGTTCTCTCTTTAGGTCTTGGGATAAAATCAATATGGGTAGAGTCAGACTCCATG

AGTGTTGTGAAGACAATCAATAAAATGCAACAATGTCCAAAAGCTGAAACCTGTGTGAAA

AAAATCTGGAAACTCTTGAGTAAGTTTGATGAGTCTCGGATTTCTCACTCATGGCGCGAA

ACTAATAGAGCAGCAGATCATCTTGCAAAGATGGCTGTTTGTGGAAATGATGTGGTTTTG

TGGCCAGCTGATTTTTCACATAGCCTTTGCAATATCATTCAAGACGATGCCAGAGGAACA

AAATATCTAAGACGGTGA

>MS.gene049939.t1

ATGGAATCCATCAATGTGGTGATAGATGATGTGTCTCAAGAACCAATTCTTGAAGTTGTA

CCAGGTGTTGAAGCATATCCTGAAACATCCACTCAAGAAGAAGTAATGCATGAAACTGTT

GAAGAACCTGAAACTAACACTGAAGATACTGAGCTGGAACATGCAGAAACAAGTAAAGGA

CCTTCAATTAGAATTCAGAAGAATTATCCTCAAGAGCTCATTATTGGAGAAACAGATCAA

GGAATCAGAACCAGAAGAGCTAATGATGTTATCTCCAATTCCTTTAGTGCATTTGGGTTT

CGCGGTAGGGTTTCACCGTCGTCCACACTTCAGCTCTCTTACTGCACCTTCTTAAGGATT

CCCACGATGTCAACGCCTGGAGTGTCGGGGGTGGCATCACTAGGTGAAGGTGATTGTGGT

GATGAGGGAACCAATGGTGAGAAGGATAAGAGATGA

>MS.gene049943.t1

ATGGGAGTGAGCAATGACATATTGAGTTCATTGCCCGAATCATTGCTTTCGGCCATCGTT

TCCTTTCTGCCGTTCAAAGAAGCGGTGAGAACTTCTATTCTCTCTAAAGATTGGGTCAAT

GTATACAAATCAACAAGAAAAATAGAGTTCAATGAACTCTTCTTTGTTAACGTTGATCAA

TCTCCTGAAATTGTTGATGCTCAAAGAAGGGCATTTTCAGCTTTCGTGAGAAAATGGATT

GAAAAACACGGAGAAGATGTCATTGACAAATTCTCTTTAACAATTATGTTGCCTGAATTT

TTTAATGAGCTCATTGATCAATGTTTTCGTTTCGCTTTAAGAAATGGAATCAAGGATTTG

GAGCTTGATTTCACTTATCAACCTTATGATATTGTTTCAGCAGTAGTTGTTTTGCCTACT

TATTTTTATGGTTGTAGTGGGTTTGAATCTTTGAAATTGTATGCATGTAAATTTGATGCT

AGTCAATGTGTTAATTTTCATTCACTCAAAAAAATTTCTTTGGGTTACATGGGAATAAAG

CTACATGCTATTAAGGCACTTTTGACCAATTGTGAAAAGTTGGAGTGTTTGAGTTTTTTG

AAATGCTGGAATTCGGAAGGAGATTTTATTTTGAATGAAGAATATATGGGGTTGAAAAAA

TTGGTTATCAATAGCTGCGATTTTAAGTTTAATATTTTTAGAGTAAATGCCCCAAATCTA

AAGGTTTTCAATTATCATGGATTGATGATACTTTTTGCCGTTGATATTAAATCTCCTGCA

TTGGATGAGGTTAATCTTGACTTTTCCCTTGAGCATCGTGGTCAAGGACATGGTTCTTAT

CTTTACAAATTTTTAAAAGAGTTCTCTTCTGCTAAAATTATGACCGTATGCAGCTACTTG

CTTCAGGTTATTTATACCGAAGGAGGACCAAAACGAATGTATCCTAGCATGAATGTTAAG

CATCTGATACTGAAGACCGCTTTGCACCATTTTGAGTTTATGGGAGTATCTTTTATGCTA

AGAAGCTGCCCTTACTTAGAGCGTCTCACAATTGAAATTGTTGACGAAAGACACCTGTTT

GGTTATGAAGCAATGTTCAATGTTATCCCAGAGATATACTGGGTTGACTCTATAAAAGTT

TACAAATGTGTGAAAAACTGTTTGAAGGAAATCGAGATTAAGAGCTTTAAGGGAACTAAA

AATGAGTTGAACGTGATTACTTACTTTCTCACTTATGGAAAGGTCTTGAGAAAGTTGAGT

ATCAATTTCCTTAGAGATGAATGGAATGTCGCTTGTCCCCGTAAAGTAGTAGAGCATTTT

CGAATGATTCCAAAAGCTTCAAATAATTTGGAGATATCAATCTATTAG

>MS.gene049945.t1

ATGTCTCGAACAGGAAGAGGTAAAACTGCCACATCGGTGCCAAAATTCGATGCCAAAAAG

TTTCTCTCTGTAGAATGTGAAAGCAGAAAGGGGTTACTTGCTCGTCGGAGTATCATTCAT

GAAAGGGTGCTCTCGATACCCGGAGATGATATGCCTCCAATCCCTATTCAGATTGAGAAG

AGAGGTTGGGAGTACTTAGTGACATATCCAGGAGAAAAGGAGGCATATGCTGAGTTAGTC

GTTGAGTTCTACTGCAACGCGTATTGTCCACAGAAAGATAAGCAAAAAGGGATAAGGATG

AATGGCCAGCCTTCAAAAAAAAATGTCAACACTGTTAGCCTCTTGAAGGAGTTATGTAAG

GATGGAACTTACTGGATGTTGGATTCAACCGGAAAGCCCAAAAAGTTTCCCTCTTCGTCG

CTTGTGCCTTTACCAAAAATTTGGTCAGCATATATTGGGTCCAATCTAGTTCCATGCTCG

AATGTTTCAGATATTAAAGTGGAGAAAGCTGCCCTCATTTCAGCAATTATGACCGGGAAA

GTAATTGATGTCGGGAAAATTATTCATGCTCAGATTCAAAACATCGCCAACTCGGCCACA

AATGCGTTGGCATTCCCTCGGTTGATCACCCTGTTATGTGAAAAGGCCAAGGTTGACATG

AAATCTCTCCCTCCTTTGAAGAAACATACTGTTATTGATTGGAACTATGTCCGCAAGTAC

TGTGGGGTGGATGACGATGACGATGTTGATGAGCCAAAAGGGAAAAGGGTTCGATGGCAG

TCAGGTGTAGGGAGCTCCCACCAATCAGCCCAAACCACTCAAAGTCGTGAGGAGAGAATT

GATGTTTTAGAAGGAAATGTGCAAGCCCTTCGTAGAGCTGGTCATCATAACATGAAGTAA

>MS.gene049938.t1

ATGGCCGCGCTGAGCCATTCTCGCGCCACGCTCGTCAATTTAACAATAGCCACCCGTGGT

TATCCTCCTGATGACTGTATTGAAATTGAGCTATCTACTCCAAATCTTTGTAGCTTTACT

TTTATTGGTTCTCCTTTTCACAAACTCTATGGGAGCAAGACCAATCTCGCTTCTATCAAA

CACCTAGACATTGATGTAAAATTGATGGCATGTTCTGCCCGGTATTCATCGTTTCTACTC

AACTGGCTGATTGAACTTACTAATATCAAGTCATTGACGCTCTCTTCACCTACTCTTCAG

GTTCTTTTCTTAGTTCCTGATTTACTGAAGGTTGAGCTCTCTTCTTTGAGTAACCTGAAG

TCACTGAAAGTAATGAGGAAAAAACCTTCATCCATACCTGATGGAATAGTGGACTTTTTG

ATTCAAAACTCACCCTCGGCAAAGGTTGACATTATAGAGGTTGACATTGAAGATTGA

>MS.gene049937.t1

ATGGCACCTTCTTCCAAAAGTCATGGACGCAACTTTTCCTCTCCTCCTCCTCCTATCGAT

TCATCACTTCCTTCTGATGTATCCGTGTGTCATGGATCTAAAAGAAAGAAGAGAAAGACA

TCTAACGATCCATCTACACGCACCAACGCCTCCTCCGCCGCTGATACCAATCGGCAGTCT

CGATCTCGACTATCTCTTGCTGAACCTTCCAAAAAATTACCAGAAAGAAAAGCTAGGGTT

GGGGGAAAACCCTCTGATAACGCCAAAAAAGAAATGGCACTGCGAGTCCGAAGCAAAGGT

GCTGGGAAATCTAGGGTTGAAAGCATAAGCAAACTGTTCTCAAGCTCTGACCACAATATT

TTAGTTGGGGGTCAAGGGAGTAATTGGGAACTTTCAAAATCTGCGAGAGGGAAATCCATT

TCAGGTAGCAGCAAACCTCAACCTGATGTACTACCCGAGAACATGTCCGGCTCTGACCTC

AATATTTTAGTTGGGGGTCAAAGGAGTAATTGGGAACTTTCAAGATCCGAGAGAGGGAAA

TCCATTTCAGGTAGCAGCAAACCTCAACCTGATATACTACCCGAGAACATGTCCGGCTCC

GACCACAATATTTTAGTTGGGGGTCCAGGGAGTAATTGGGAACTTTCAAGCTTTGAGAGA

GGGAAATCCATTTCAGGTAGCTGTAAACCTCAACCTTTGAAGTTTAGCACCAAACCTAAA

CCTTTAGAGAAGGTTCCGGTTGATGAAGCCTCTCCCAAATCCAGTTCACCTGCCACCGAA

CCTCAACAACCTCTCACTGATGATGATGATGATGATGATGAGGACTTGAGTTACATGAAC

TCTCATCTCACTTTTGAACAAATGGTGGAAAACATGAAAAAATTAGATGAACTCATGTTG

GCTGAACCCGAATCCGAATCCGACGACAATCTTATGCAGTGCAATTGTGAGTTCAACAAC

TGCACATGTCTTCAATCTTATCGTGAATATCTTGCACATGACTATGCTGAGTTGGATTTT

AGGGCAGTCTAG

>MS.gene049947.t1

ATGGGAGTGAGCAATGGCATATTGAGTTCATTTCCCGAATCGTTGCTTTTGGCCATCGTT

TCCTTTCTGCCGTTCATAGAAGCGGTAAGAACTTCTATTCTCTCTAAAGATTGGGTGAAT

GTATACAAATCAACAACAAAAATAGAGTTCAATGAACTCTTCTTTGTTAAAGTTGGTGAA

TCTCCTGAAATTGTTGATGCTCAAAGAAGGGCATTTTCAGCTTTCGCGAGAAAATGGATT

GAAAATCACACAGAAGATGTCATTGACAAATTCTCTTTAACAATTGGGATGCCTGAATTG

TTTAGTGAGCTCATTGATCAATGTGTTCGTTTCGCTTTAAGAAATGGAGTCAAGGATTTG

GAGCTTGATTTTACTTATCAACCTTGTGATATTGTTGATGATTGGGTTTGA

>MS.gene049944.t1

ATGGGAGTGAGCAATGACATATTGAGTTCAATGCCCGAATCATTGCTTTCGGCCCTCGTT

TCCTTTCTGCCGTTCAAAGAAGCGGTAAGAACTTCTATTCTCTCTAAAGATTGGGTGAAT

GTATACAAATCAACAACAAAAATAGAGTTTAATGAACTCTTCTTTGTTAAAGTTGGTGAA

TCTCCTGAAATTGTTGATGCTCAAAGAAGGGCATTTTCAGCTTTCGTGAGAGAATGGATT

GAAAATCACAAATGGAGTCAAGGATTTGGAGCTTGA

>MS.gene049942.t1

ATGTCAATTGGTCTTGAAGTTTCACTTACTATAATTAGAACATTTGTTGATGCATTTTAC

ATTATTCATATCTATATTAGGTTTCAAACTGCTTATATTGCTCCTTCATCTCGTGTTTCT

GGTCGAGGAGAACTCATTATAGATTCCTCAAAGATTGCATCAAACTACATGAAAAAAGAA

TTTTGGTCTGATCTTGTTGCAGCACTACCACTTCCTCAGATAACCAAGGCAAGTGGAGTA

ATGATGGAAAAAGCTTGGGCTGGTGCAGCTTATTATTTGACACTCTATATGCTTGCTAGT

CATGGTGAAAGCGATGAACCAGAAGATTCAGTTGCTGGTACACTTGAGCTTAAGTCTTTA

TTCTTATGA

>MS.gene049950.t1

ATGTCCTCACACGCAGTGCAATTGCGTATCAAGAGCAATCATGTGGTTATGGACAATGGT

ATACTACAAGTGTATTTATCCAATCCTGGTGGATTTGTAACTAGAATACAATATAATGGC

ATTGATAATCTGCTTGAAGCTCTTAACGAAAAGAATAATAGAGGGTATTGGGATGTTGTT

TGGAGTGAAACAGGAAGTGCAGGAACAACCGGGACGTTCGAGCGGGTTGTGGGAACAAGT

TTTAATGTGATAATGGAAACAGAAGAACAAGTTGAAATCTCATTTACAAGAACATGGAAT

CCATCTCTAACGGGAAAGTTGTCACCTCTTAATATAGACAAAAGGTATGTGATGCTACGT

AATTCCTCAGGATTCTATTCATATGCTATCTTTGAACACCTAAAGGAGTGGCCAGCTTTC

AACATACCTCAAATCAGGATTGTTTATAAGCTTCGTAAAGACAAGTTTCATTACATGGCT

GTAGCTGATAATAGACAAAGGTTGATGCCTCTTCCTGATGATCGATTACCAGGAAGAGGA

AAAGAACTTGTTCCCCCAGAAGCTGTTTTGCTTGTAAATCCCATCGAGCCAGAGTTCTTA

GGAGAGGTTGATGACAAGTACCAATACTCAAGTGAGAACATAGACCTTAAGGTTCATGGA

TGGATAAGTGCAGAGTCTGAAACAAATCCAGCAACAGGTTTTTGGGTAATCATTCCAAGC

AATGAATTTCGATCAGGTGGTCTTGTTAAACAGAATCTTACCTCCCATGTTGGCCCCATC

AGCCTTGCTATGTTTCTTAGTGCTCATTACGCAGGAGAGGATATAGTTCTTAAACTCCAG

CCTAATGAGCCATGGAAAAAAGTTTTTGGGCCAACTTTTGTTTATCTTAACAACTTGTTG

GATCATGAGGACCCACTAGCTCACCTCTGGGAGGACGCCAAATTTCAGATGAACAAGGAA

GTACAAAGTTGGCCCTATGATTTTCCAGCTTCAGATGATTTTCAAAAGGCTAGCCAACGG

GGTAGCGTTTGTGGCACGTTACTAGTTCGTGACAGGTGTGTAAGTGACAAGGACATAATA

GCACAAGGTGCATATGTAGGTTTAGCCCCACCAGGAGATGCTGGATCTTGGCAAAGAGAA

TGCAAGGGATACCAATTTTGGAGTAAAGCAAACGAGGAAGGCTACTTTTCAATCAATAAT

ATACAGAGTGGTGATTACAATCTCTACGCATGGGTTCCAGGTTTCATTGGAGAATATTGG

AACAATGTTGTCCTCAAAATAACCCCAGGTTGTGAGATAAATGTTGATGACATAGTCTTT

GAGCCTCCAAGAGATGGTCCAACATTATGGGAGATTGGAATTCCTGATCGCTCTGCTGCT

GAGTTCTATGTTCCAGATCCTAATCCTAAATTCATAAACAAGCTTTTTATAGACCATCCT

GACAAGTTTAGGCAGTATGGCTTGTGGGAAAGATATGCAGAGCTATACCCAACTGAAGAT

TTGATTTATAATGTTGGTGTTAGTGACTACACAAAAGATTGGTTCTATGCACAGGTCACC

AGGAAGAAAGATGATGGCAGTTACCAAGGAACTACATGGCAAATTAAGTTCAACATGGAT

GATGTGCAAGAAAATGGACTGTATAAATTAAGATTAGCTCTTGCATGTGCAAATGTCTCT

GAATTACAGGTTAGAGTGAATGATGTAAAACAAGATCCTCCACTATTTACTACGGGGGTG

ATTGGGAAGGACAATGCAATAGCTAGACATGGAATTCATGGACTCTATTGGTTATTCAAC

ATTGGTGTGCCAAGTATTTTGCTATTCAAAGGAGATAATACTATTTTTCTAACACAAACT

ATGGCTACTGGTCCCTTGGCCCTCTTTCAGGGAATAATGTATGATCATATTCGTTTAGAA

AGCCCCTATTAA

>MS.gene049952.t1

ATGGCGACGTTCGAACTTTATCGTAGGTCGACGATCGGACAGTGCTTGACAGATACTTTG

GATGATATGGTTCAGAATGGAACTCTTAGTCCTGAACATGCCATTAAGATTCTCGTTCAG

TTTGATAAGTCTATGACTGAGGCTCTGGAGACGAAAGTTAAGAGCAAGTTTTCTATCAAG

GGACATCTTCATACATACAGATTTTGTGACAATGTCTGGACCTTCATCTTACAAGATGCT

TTGTTCAAGAGTGAAGACAAGCAAGAGAATGTTGGACGTGTTAAAATTGTGGCATGTGAC

TCAAAGTTGCTTGGTCAATAA

>MS.gene049941.t1

ATGAATTTCGGAAACCATTTCGACTTTCACAGAGCCTTTACATGTGCCGGCATGAGTTTC

AGATTATCCATCGTAATCGTCGTCTTCAGTCCTGATATGCTCATTTTTGTCAAAATTGTC

TCTCCAGTAGTATGCTCCTGCAGAAAAGGCAGTTCTTTCCCTCACACAGAGTTCAGGAAA

CCCACTGCAATAATTCTTCCAACAATAGAAGTTCCTAGATATAGGCCTGCACCAATTGTT

GCATCATTCTCATCTAGAGGCCCTTCAAGCCTAACAAAAACATTCTCAAGCAAATAA

>MS.gene049940.t1

ATGGGTGAAGAATTATCAGGTTTGACAGTCAAAGAATTACAGGGTTTGGAGAACCAATTG

GAAATCAGCCTTCGTGGTGTTCGTATGAAAAAGGAGCAACTTTTTATGGATGAAATACAA

GAACTAAATCGAAAGGGAGACATCATAAACCAAGAAAATGTCGAACTGTATCTTAAGGTA

AACCTAATTTGTCAAGAAAATATGAAATTGAAAAAGAAGGTCTATGGAACAAAAGATAAG

AATGGAACAATCAGAGTTTTGAGTCTCACAAATGGTGTAGGCATAGGAGACGATCTAAAT

GTACCTGTGATCTGTGAATCTCCAGCTCAGCCAGCCACAGCAACAACACTACAAGGCCCC

TTCAGGAACTACAAAACTAGGGTATGA

>MS.gene049956.t1

ATGCCGGGAGAGTTACTGAATCACTCGGGAACCCGCAAGATTGAGTGTTTAGAGGAGATA

GGTGATGGGGTTTGGGAGTTGGATAGGGCATCCCAAACTCTGACTTTCACAGGGTTGGTT

AGGACAACCGAAGGAATAAGAGGTTTAGTGGTGTCATCTGTCATTCATCATGTTACCTTG

GAGGTAGTGTTACCATTAGAGAGGATGATTAGTACATTAGGTCTTGATAGAGCTAGGGAG

AATTCGACCTCTGAAAGGGCTAGAGGACCAAGGCCTCGTTTTCGTCGTACCGCTCGCATT

AGTGTGCGTCCCCCCGTTGTTTATCATTTTGAGAATGATCCCGAGGAGGATGGAGCAGAC

ATGACCGTGACTGATGTGGAGGTGAGTGGACGTGCTGGAGATGAAGTGGATCCCTCTGAA

GACCTTGGTGTTGGTGGTGCTAATGAAGATCTCCCGAATGGTTGTTAG

>MS.gene049955.t1

ATGACAACGCTAAGATCGAATTGGCCTTCAAGGTTACGTCAACTTCTGTCAAGTGAAGGT

GCAATTGGTCCTTCTATTAAGCTCGATTCTGAGCCTCCTCCTAAGGTCAAAGCCTTCATT

GAGAAGGTCATCCAGTGTCCGTTACAAGATATAGCTATACCACTATCTGGCTTTCGGTGG

GAGTACGACAAGGGGAATTTTCATCACTGGAGACCATTGCTGCTTCATTTTGATACATAC

TTCAAGACTTATTTATCGTGTAGAAATGACCTCACTTTGTTAGATAATCTAGAAGTTGAC

AGCCCATTACCAAAACATGATATTCTGCAAATATTGCGGGTGATGCAAATAATTTTAGAG

AACTGTCCAAATAAGAGCACCTTTGATGGCATAGAGCATTTCAAGCTTTTGCTAGCATCA

ACTGATCCCGAGATTCTTATTGCTGCATTAGAGACTCTTTCTGCACTTGTAAAAATTAAT

CCCTCTAAGCTTCATGGAAATGCAAAGATGGTTAGCTGTGGTTCAGTGAACAGCTCTCTC

TTATCGCTAGCACAAGGTTGGGGAAGCAAGGAGGAGGGACTGGGATTGTACTCTTGTGTA

ATGGCAAATGAGAAAGCCCAAAATGAAGCACTAAGCTTGTTTCCTTCTGATGTAGAGATT

GGTGGTGATCAATCTAATTATCGCATAGGCACTACTCTTTATTTTGAATTACATGGACCT

AGTGCACAAAGTGAAGAACGCAGTGCAGATACATCTTCCCCCGGCATGAGAGTCATACAT

ATGCCAGATTTACATCTCCGCAAAGAAGATGATTTGTCATTGCTGAAGCAATGCATTGAA

CAATACAACATTCCTTCAGAGCTCAGATTTTCGTTGCTCTCTAGAATTAGATATGCTCAC

GCCTTTCGGTCTCCTAGGATATGCAGGCTTTACAGCAGGATATGCCTACTTTCTTTCATT

GTGCTGGTTCAATCTGGCGATGCTCATGATGAACTTGTGTCCTTTTTTGCTAATGAACCG

GAATATACAAATGAGTTAATTAGAATTGTGCGATCTGAAGAAACCATATCTGGATCTATC

AGAACACTCGCAATGCTTGCATTAGGAGCCCAGTTGGCAGCATATACTTCATCGCATGAA

AGGGCACGGATACTTAGTGGATCTAGTTCAAGTTTTGCCGGTGGGAACCGCATGATACTT

CTGAATGTGCTCCAGAGGGCTATCTTGTCATTGAAAAATTCTAACGATCCATCGACCCTT

GCCTTTGTTGAGGCACTTCTTCAGTTTTATTTGCTTCATGTGGTTTCAACGTCAACTTCT

GGGAGTAGTATTAGAGGTTCTGGTATGGTACCTACATTCTTGCCGCTTCTGGAGGATTCT

GATCCTGCACATGTTCATTTAGTGTGTTTTGCTGTGAAAACCCTTCAGAAGCTTATGGAT

TATAGTAGCTCGGCTGTATCTTTGTTTAAAGAGTTGGGGGGCATTGAGCTTTTGTCTCAG

AGATTATGGAAAGAAGTGCAAAGGGTCATTGAAGTTGGAGAAAACGATAACATGTTCATC

GCTGGTGAAAGTTCAAGGCATAGTACTGATCAATTGTACTCCCAGAAGAGGCTCATAAAG

GTCTCCCTCAAGGCACTTGGTTCTGCAACATATGCACCTGCAAACGCCACAAGATCTCAA

TATTCTAACGACAATTCATTGCCTGCTACTCTATGCTTAATATTTCAGAATGTAGATAAG

TTTGGAGGTGATGTTTACTACTCAGCTGTTACTGTCATGAGCGAAATAATCCATAAAGAT

CCTACATGTTTTTCTATTCTGCATGATATGGGTCTCCCCGATGCTTTTTTGTCATCAGTT

GGATCTGAACTGCTTCCTTCATCAAAGGCTTTGACATGTATTCCGAATGGTCTTGGGGCC

ATATGTCTCAATGCCAAAGGGCTAGAGGCCGTTAGAGAATCTTCGTCACTGCGGTTCCTT

GTTGATATTTTCACCAGCAAAAAGTATGTCTTAGCCATGAATGAGGCTATTGTTCCTTTA

GCAAATGCTGTGGAGGAACTTCTACGTCATGTGTCCTCATTGAGAAGCACTGGTGTGGAT

ATTATCATTGAAATCATCCATAAGATTGCATCTTTTGGTGATGAAAATGGTAGAGGATTT

TCTGGAAAAGCTAATGAAGGTACTGCAATGGAAACAGATTCTGAAGTCAAAGAGAATGAA

GGCCATGGTTGCATTGCGGGCACTTCATATTCAGCCGCAGAAGGCATAAGTGATGACCAG

TTTATTCAGCTATGTGTCTTTCATTTGATGGTATTGACTCATAGGACTATGGAAAATTCT

GAAACATGCCGGCTATTTGTGGAAAAATCAGGAATTGAATCTTTGTTGAAGTTGTTATTA

CGACCCACTATTGCTCAATCCTCAGAAGGCATGTCCATTGCTTTACACAGCACCATGGTA

TTTAAGGGATTTGCTCAACATCACTCAACTTCTCTGGCACGAGCTTTCTGTTCCTCTCTT

AAAGAGCACTTGAAGAAAGCATTAGCTGGGTTTAGTGCAGCTTCTGAACCTTTGTTACTG

GATCCTAGAATGACAAATGATGGTGGCATATTTTCGTCCCTTTTCCTTGTTGAGTTCCTA

CTATTCCTTGCTGCAGCGAAAGACAACCGGTGGGTGAGTGCGTTGCTTACAGAATTTGGA

AATGGCAGTAAGGATGTCCTTGAAGACATTGGGAGCGTCCACCGTGAAGTTCTGTGGCAA

ATTGCTCTACTTGAAAATAAGAAACAAGGGATTGAAGAAGAGGGTTCTTGTTCTTCTGAT

TCCCAACAGGCAGAACGTGATGCAAGTGAAACCGAGGAGCAAAGGATCAATTCTTTCAGG

CAGTTACTTGATCCATTACTGAGGAGGAGGACATCAGGATGGAGCATTGAATCTCAGTTT

TTTGACCTTATAAACATGTATCGAGATTTGGGTCGTTCCACTGGTTTCCAACATCGATCA

ATTTCTGCTGGGCCAAACATACGATCAAGCTCCAGTAACCAGTTGCATCATTCTGGGTCT

GATGATAATGCTGATTCTGTTAACAAGAAGGAATCTGACAAGACTAGGTCTTATTATACC

TCTTGTTGTGACATGGTCAGATCACTTTCATTTCACATTACCCATTTGTTCCAAGAGTTA

GGAAAAGTAATGCTGCTACCATCACGTCGACGTGATGATATTGTGAATGTAAGTCCTGCT

TCAAAATCAGTGGCTTCTACTTTGGCATCAGTTGCTCTAGATCATATGAATTATGGGGGC

CATGCAAATCATTCGGGAACAGAAGAGTCCATATCAACCAAGTGTCGGTATTATGGGAAA

GTGATTGACTTCATAGATAGTATGCTAATGGAGAGGCCAGATTCATGCAATCCTGTTCTG

TTGAATTGCTTGTATGGACGTGGAGTTATTCAATCTGTATTGACCACCTTTGAAGCTACC

AGTCAGCTGCTCTTTTCAGTTAATCGGGTCCCTGCCTCGCCTATGGATACTGATGATGCA

AATGCAAAGCAAGATGACAAGGAGGATACAAATAACTCATGGATTTATGGTTCTTTAGCT

AGTTATGGGAAATTGATGGACCATCTAGTAACCTCCTCTTTTATATTATCATCATTCACA

AAGCATTTGCTTGCACAGCCCCTTACAAATGGTGACACACCTTTTCCAAGGGACCCTGAG

ACTTTTATGAAAGTCCTCCAATCCACAGTGTTGAAGACCGTGCTTCCTGTTTGGACTCAT

CCCCAGTTTGGTGACTGTAGTTATGAATTTATTTCTGCAGTTATCTCCATCATTAGGCAT

GTCTATTCAGGGGTTGAAGTAAAAAATGTAAATGGCAGTGGTGGTTCTCGTATTACTGGA

CCGCCTCCTAATGAAACAACTATTTCTACCATTGTAGAGATGGGTTTTTCGCGGTCTAGA

GCAGAGGAAGCTTTGAGGCATGTTGGGTCGAATAGTGTAGAGTTGGCGATGGAGTGGTTG

TTCTCACATCCAGAAGAAGTACAAGAAGATGATGAACTTGCTCGTGCACTTGCAATGTCC

CTTGGGAACTCTGAATCAGACACCAATGATGCCGTTCCAAATGCAAATGAAAATGAAGCT

GTTCAACAGCTTGAAGAAGAGACAGTACAGTTTCCTTCTGTTGATGAGTTGTTATCTTCT

TGCACAAAACTTCTGATGAAAGAACCTCTTGCTTTTCCTGTCCGCGACTTACTTGTGATG

ATATGCTCTCAGGATGATGGTAAACATAGATCTAGCGTTGTCTTATTTATTGTTGACCGA

ATCAAAGAGTGTGGTTTGGTTTCTAGCAATGAAAATTACACCATGCTGGCTACTCTTTTT

CATGTTCTTGCTTTAATTCTTAACGAGGATACTGTGGCACGGGAAGCTGCTTCCAAGAGT

GGGTTGATCAAAATTGCCTCTGATCTACTCTACCAGTGGGATTCTAGTCTTGATGGCAAG

GAGAAACAGCAGGTGCCAAAATGGGTCACAGCTGCTTTTCTTGCCTTAGACAGGTTGCTG

CAGGTGGATCAAAAATTAAATTCTGAAATTACAGAGCAGTTGAAGAAGGAAGTTGTGAAT

AAACAGCAGGCATCAATTACTATTGATGAAGATAGGCAAAACAAGTTACAGTCTGCACTG

GGGCTCTCAATGAAGTATGCCGATATACATGAGCAGAAGAGACTTGTTGAGATTGCGTGT

AGTTGTATGAAGAATCAACTTCCCTCCGACACAATGCACGCTGTTCTGCTTTTATGTTCC

AACCTTACAAGGAATCATTCTGTTGCGCTTGCCTTTTTGGATGCTGGTGGTTTAAGTCTA

CTTCTTTCGTTGCCAACAAGTAGTCTTTTCTCTGGGTTTGACAATGTTGCTGCCAGTATT

GTCCGTCATATTCTTGAAGATCCACAGACACTCCGTCAAGCAATGGAATCCGAGATAAAA

CACAATCTTTTAACTGTGCCTAACCGGCATCCAAATGGAAGGGTCAATCCTCGCAATTTT

CTTTCAAATTTAGCTTCTGTGATTGCTCGGGATCCAGCAGTTTTTATGCAAGCTGCACAA

TCTGTGTGCCAGGTTGAAATGGTAGGTGAAAGACCTTACATTGTCTTGCTGAAAGATAAA

GACAAAGTGAAGGAGAAAGAAAAGGATAAGCATAAATCATTGGAGAAAGAAAAAGTACAG

AATGGTGATGGTAAGGTTGGTGTTGGGCATACAAACACAGCAGGTTCTGGCAATGGCCAT

GGCAAAATTCACGATTCAAATACCAAGAGTGTCAAAGGTCATAGAAAACCTAGTCAAAGT

TTTATTGATGTGATAGAACTGCTGCTTGAATCTATATGCACGTTTATTCCCCCGTTGAAG

GATGATGTTGACCCAAATGTTCTTCCTGGCACCATAGCATCAAGTGACATGGACATTGAT

GTCTCTACGAGTAAGGGAAAAGGAAAAGCGGTCGCTACTGTGTCTGACGGAAATGAAACC

AGTAGTCAAGAAGCTTCTGCATCACTTGCGAAAATTGTCTTTATTTTGAAGCTTTTGACA

GAGATATTATTGTTTTATTCATCATCAGTTTATGTTCTACTTCGACGAGATGCTGAATTG

AGCAGCAGTAGGGTCACTTATCAGAAGAGTCCTGTTGGTATAAGCATCGGTGGAATATTC

TACCACATTCTTCATAATTTTCTTCCATATTCTCGAAACTCCAAAAAGGACAAGAAAGTT

GACGGTGATTGGAGGCAGAAACTAGCAACCAGGGCTAACCAGTTTATGGTGGCTGCTTGT

GTCCGTTCCACAGAGGCTAGGAAGAGGATTTTTAGTGAGATTAGTTCCATCATCAATGAA

TTTGTTGATTGTCATGGTGTTAAGCATCCAGGTAATGAAATTCTGGTTTTTGTTGATCTG

ATTAATGATGTTCTGGCTGCTCGCACACCTTCTGGTTCATGCATCTCAGCTGAGGCCTCT

GCCACTTTTATTGATGCTGGTTTGGTTAAATCATTTACTCGTACTCTTCAAGTTTTGGAC

CTGGATCATGCTGATTCATCTAAAGTTGCAACTGGTATTATTAAAGCTCTTGAGTTGGTT

AGCAAGGAGCATATCCATTCAGCTGATTCTAATGCAGGGAAAGCAAAACCTGATCTACAA

CAACCTGGACGAATAGATAATATTGGTGATATGTCTCAGTCCATGGAAACGACTTCTCAA

GCCAATCATGGTTCTCGTCAGGCTGATCAAGTAGGACCTTACACAGGTCAGACTTATGGC

GGGTCTGAAGCTGTTACCGATGACATGGAACATGATCAAGATCTTGATGGGAACTTTGCT

CCTTCTAACGAGGATGATTACATGCATGAAAATTCTGAGGATGCACGAGATGTTGAAAAT

GGAATGGAAAGTGTAGGTTTACAATTTGAAATCCAACCTCATGGCCAAGAGAATCTTGAC

GAAGATGACGATGAGGATGATGATATGTCTGGAGATGAAGGTGAAGATGTAGATGAAGAC

GAGGATGATGAGGAACATAATGACTTGGAACACGAAGTCCATCACTTGCCACATCCTGAC

ACAGATCAAGATGACCATGAGATTGATGATGATGAGTTTGATGATGAAGTGATGGAAGAA

GATGATGAGGAAGACGAGGAAGATGAGGATGGTGTTATACTGCGACTTGAGGAGGGCATT

AATGGAATTAATGTTCTCGACCACATTGAGGTTCTTGGCAGAGATAATAACTTTCCAAAT

GAAGCTTTTCATGTAATGCCAGTTGAAGTTTTTGGCTCTAGACGCCCTGGGAGGACAACA

TCTATTTATAATCTTCTGGGAAGAACTGGTGATACTGCTACGCCTTCCCGCCACCCACTC

TTGGTTGATCCTTCTTCCTCATTCCCCCCATCTACGGGGCAGTCAGATAGTCTTATGGAG

AACAACACATCAGGTTTGGATAATATATTTCGATCATTGAGAAGTGGACGCCATGGAAAT

CGTATGAACTTGTGGACTGATAATACCCAACAGAGTGGTGGATCAAACACCAGTGTTGTA

CCACAAGGCCTCGAGGAGTTGCTTGTCTCTCAATTAAGGCAACAAACCCCTGAAAACTCG

CCTAATCAGGATGTAGCAGAAGCAGGTTCTCATGGTAATGTTGAAACCAGCCAGGCACAA

GATTCAGGCAGTGCAATGCCAGAAATCCCTGTTGAGAGTAATGCTATCCAAGGAGTTAGT

ATAACTACTCCTTCAATAATTGATAACAGTAATGATGCTGGTATCAGACCTGCAGGGACA

GGAGAGCAAACAAATGTATCAAACAGTCACTCACCGGCTGCTGAGATTCAATTCGAGCAT

AATGATGGTGCATTGAGGGATGTTGAAGCTGTGAGCCAGGAGAGTGGTGGTAGCGGAGCA

ACTTTTGGGGAAAGCCTCCGGAGCCTAGATGTTGAGATTGGAAGTGCTGATGGCCATGAT

GATGGTGGGGAAAGACAGGTTTCTGCAGACAGAATAGCTGGTGATTCACAGGCTGCACGC

TCAAGAAGAGCAAATATGCCTCCTGGTCATTTTCCCCCTGTTATTGGAAGAGATACACCC

CTTCACAGTGTTGCTGAAGTTTCTGAGAATTCCAGTCGTGATGCAGATCAAGTGAGTCCA

ACAGCAGAGCAGCAAGTGAATAGTGATGCTGGATCAGGAGCAATTGATCCTGCTTTTCTA

GATGCTCTTCCTGAGGAGCTGCGTGCTGAAGTCCTCTCCGCACAGCAAGGTCAAGTGGCT

CAGCCACCAAATGTCGAATCTCAGAACTCTGGGGATATTGACCCAGAGTTCCTTGCTGCC

CTCCCTGCAGATATTCGAGCAGAAGTTCTAGCTCAACAGCAAGCACAGAGGTTAAATCAG

TCTCAGGAGTTGGAAGGTCAACCTGTAGAAATGGATACAGTCTCAATAATTGCAACGTTT

CCTTCAGATTTACGAGAAGAGGTTCTGTTAACTTCATCTGATAACATCCTTGCCAATCTT

ACACCTGCCCTTGTCGCCGAGGCAAATATGTTGCGGGAGAGGTATGCACACCGTTACAGT

CGTACCCTCTTTGGTATGTATCCAAGAAGCCGTAGAGGTGAGACATCAAGACGTGATGGC

ATTGGTTCTGGCCTAGATGCAGTAGGGGGGCCTATTTCTTCACGCCGGTCCAGTGGAACT

AAGGTTGTTGAAGCTGATGGAGCTCCACTAGTTGATACGGAAGCCTTGCATGGCATGGTT

CGGTTATTTCGCATGGTGCAGCCACTCTACAAAGGCCAATTGCAGAGGCTTCTTTTAAAT

CTTTGTGCTCATAGTGAAACAAGAATATCTTTGGTGAAAATTCTGATGGACTTGCTGATG

CTTGATGTGAGAAGATCTGTCAGTTCATTTGGCACTGTTGAGCCACCGTATAGATTATAT

GGTTGTCAGAGCAATGTAATGTATTCACGCCCGCAGTCTTTTGATGGAGTACCCCCACTG

CTGTCTCGACGAGTTCTTGAAACCCTCACCTATCTTGCTCGTAATCATCTATATGTGGCA

AAGAGTTTGCTTCAGTCTAGGCTTCCTCATCCTGAGATTAAAGAACCAAATAACACTAGT

GATGCACGTGGCAAAGCCGTGATGGTTGTTGAAGATGAAGTCAATATAGGTGAAAGTAAT

GAAGGGTACATCTCTATTGCAATGCTTTTGGCTCTTTTGAACCAGCCACTTTATTTAAGG

AGCATAGCCCATCTTGAACAGCTGCTAAATTTACTGGATGTAATCATTGACAGTGCTGGA

AGCAAGTCCAGTTCATCTGATAAATCCTTGATTTCAACTCCAAAGCCATCGTCAGGTCCT

CAAATTTCTGCTGTCGAGGCAGAAACGAATGCAGGTTCTGGTGATGCATCTAATACAGTT

AACGATTCATCCAAACCCACACCCGTTGATGATATTATAGAATCTGAGTCTCAGAGAGTA

TTGAGTAACTTGCCACAATCTGAACTCCGGCTCCTGTGCTCATTGCTTGCACATGAAGGT

TTGTCAGATAATGCATATACTCTTGTTGCGGAGGTAGTGAAGAAATTGGTGGCCATTGCT

CCAACTCATTGTCAGCTTTTTGTCACTGTGCTGGCTGAAGCAGTTCAAAACTTGACATCT

TCTGCAATGGCTGAGTTACGTGTCTTCAGTGAAGCTATGAAAGCTCTTCTGAGTACTACA

TCTACAGATGGAGCTGCAATTTTGAGAGTTTTGCAAGCCTTGAGTTCCCTTGTCACCTCA

CTAACTGAGGACCATGGTGGCAGAGTTAATCCTGCTGCTCTTTCTGAGGTTTGGCAAATC

AATTCAGCATTAGAACCATTGTGGCAGGAGCTGAGCTGTTGCATAAGCAAGATAGAGTCC

TACTCCGAGTCTACATCTGAGTTTGTCACCCCATCTAGTTCCTCTACTTCTCAACCAGCT

GGTACCATGCCTCCACTTCCAGCTGGCTCTCAAAATATCTTGCCATTCATAGAATCTTTC

TTTGTGGTTTGTGAGAAATTGCATCCTGCACAGCCAGGTGCTAGTCATGACCCAAGTATT

CCTGTTATTTCTGACGTTGAGAATGCCAGTACATCCGAATCTCCGCAGAAAGTATCCGGA

CCTGCTGTGAAAGTAGATGAGAAAAATATGGCTTTTGTCAAGTTCTCAGAGAAACATAGG

AAGCTTCTAAATGCTTTTATAAGGCAAAATCCTGGTTTGCTTGAGAAATCCTTCTCACTC

ATGCTGAAGGTTCCAAGATTTATCGATTTTGATAACAAGCGCGCTCACTTCCGATCAAAA

ATTAAGCATCAGCATGACCATCACCATAGCCCGTTAAGAATATCTGTAAGAAGGGCATAT

GTTCTAGAAGATTCTTATAACCAACTTCGCATGAGACCAACTCAAGATTTGAAGGGAAGG

TTGACTGTTCACTTTCAAGGGGAGGAAGGTATCGATGCAGGAGGACTTACAAGGGAGTGG

TATCAATTGTTGTCCAGAGTTATTTTTGACAAGGGAGCACTGCTTTTTACTACAGTGGGC

AACGAGTCAACATTTCAACCAAACCCTAACTCTGTTTATCAAACAGAACATTTATCTTAT

TTCAAGTTTATTGGTAGAGTGGTTGGTAAAGCACTATTTGATGGTCAACTTTTGGATGTT

CATTTTACTCGCTCATTCTACAAGCACATACTTGGGGTGAAGGTTACATATCATGATATT

GAAGCCATTGACCCTGACTATTTCAAAAATTTGAAATGGATGCTTGAGAATGATATCAGT

GATGTTCTGCATCTTACTTTTAGCATTGATGCAGATGAGGAAAAATTGATCTTATATGAA

CGGACAGAAGTGACTGATTATGAGTTGATTCCTGGCGGACGGAATATCAAAGTTACTGAA

GAGAACAAGCATCAGTATGTTGATTTGGTTGCTGAGCATCGGTTGACAACTGCTATTCGA

CCTCAAATAAATGCTTTCTTGGAAGGGTTCAGTGAATTAATTCCCAGAGAGTTGATATCC

ATATTCAATGACAAAGAGCTTGAATTATTGATCAGTGGGCTTCCTGATATTGATTTGGAT

GACTTGAGAGCAAATACAGAATATTCTGGATATAGTGCTGCATCGCCAGTTATTCAATGG

TTTTGGGAGGTTGTTCAAGGCTTAAGCAAAGAAGACAAGGCTCGCCTTTTGCAATTTGTA

ACTGGCACATCCAAGGTGCCTTTGGAAGGTTTTAGTGCTCTTCAAGGAATTTCAGGCTCC

CAGAAATTTCAGATACATAAAGCATATGGAAGTCCTGATCACTTGCCTTCTGCTCATACT

TGGAAGATGAACAAGATTTACACATATTTGTATTCTAATCATCTTTATTCTAGACTCTGA

>MS.gene049959.t1

ATGGAACTTTTTGCCGCTAATTTTCTCTTTCTCTTCTTGTTTTTATGTCTTGCAGTTGCG

CTCACATTCATATTTTTTCACGCATTGCTGTCTTGGATTTTGAGCCGTATTTTGTCTGCA

TCTGTTGGATTTTGTGTTGGTGGATGCAACAGTTTACGAGATGTGGTTGTGAAATTTAAA

AAGGGTCCCACTGAATCTGTATCTATTGGTGAAATTAAACTCAGCTTATGCCTTTCCTCT

GTCGAACCTGGTGTGGATTCTCGTTCTTGGAATCCAAAGTTACAACTATTAATATGCGAC

TTAGAAGTTGTTACGAGGCCTACAAATAAAAGCCCTGCAAATAAAAGCCATGCGAAGAAG

AAAACTCAGAAATCCAAAACTCGTTCTTTAGGAAAGGGAAAATGGAAGACTATTGTCAAT

ATTGCAAGATATTTTTCTTTTTCTTTGACAGGGCTAGTTGTAAAGACACCTAAAAGTAGT

GTTGGGATCGGGAAGTTGAATGTTGATATATCTAAAGGTGGTGGATCAGAGTCAAATTTG

CTTGTTAGTGTACAGATATTACCCATTGTCGTTCACATTGGTGATCCTCAAGTTAGTCGT

GATCTATTATCAAAATTCAGTGTTTCTAGCCAGGCATCTGTTGCTCCTAGAGAAAAGTCT

TCTGCTCCTTTTATCTGTGAAAAGTTCTCCGTTTCATGTGAATTTGGTCATTATAGGGAA

GTGGGTATTGTTATTAAGAATGTAGATATATCCTGTGGCGAAGTTACTGTGAATTTGAAT

GAGCGGTTGCTTGTCAAAAGAAAGAGGTCATCAGAATCTCCATCTGTCTCTGATAGAAGT

ATAGGGTCAAATGTTGATCATACTAGCACAAAACCGTCTTTAACGAAGGAAGAAAAACTA

GCACGCTACAGTTCTTTGTTTCCTGAGAAGGTTGGCTTCAATCTACCAAAGCTGAATGTG

AGTTTTGAGCATTGCGAATATGGTCTTTCTGTTGAAAATTACATCACTGGTATCCAATTT

AATAGCATCAAATCACGCTCCAACAAGGATATTGGGGAGAGTGCACGACTTCATATTAAA

CTGGAGTTCAGAGAAATTCATCTTCTTAGAGAAGCTGACGCTTCCATCCTGGAGATAACA

AAGGTGAATCTGGTCTCCTTTGTGTATGTCCCAGTTCAGTCAATCTCACTTCTTAGAGCT

GAAATCGAAATCAAGCTTGGACGTTCACAATGCAACATAATTTTGAGCAGATTGAAGCCT

TGGTTGCTCATCCACTCCTCCAAAAAGAAAAAAGTTGTACTTCGAGAGGAAGCTTCGGTT

GCAAAACCGAAATCAAATGATAACAAGACAATCACATGGACATGTAAATTTTCAACTCCT

GAGATGACAATCATGCTTTATAATATGGCTGGTTTTCCGGTGTATCGTGGTTGCTTACAA

TCACCACATCTCTTTGCAAAAAACATTTCAAATATGGGGACCTCTGTACACTTTGAGCTT

GGTGAATTCAATCTCCAGCTGGCTGATGAAAATAAAGAATGCTTAAAAGAAACCATTTTT

GGTGTGGAATCAGATTTTGGCTCCATTATTTACATTACAAAGGTTAGTTTGGATTGGGGC

AAAAAGGACATGAAATCGTCTGAAGAAGATGGTCCAAGGTGTATGCTAGGTTTATCAGTT

GATGTTACTAGCATGGGAATTTATCTAACTTTCAAGCGTTTAGAATCATTAATATCAGCA

GCGATATCCTTTCAAGCTTTAATGAAAAGCATATCTTCAAAGAAAAAATCAACTCAAAGT

CGAGGGCGTTCATCAAAAACATCGGGTAAGGGAACTCAAATGTTGAAATGCAGTCTTGTA

CAATGTTCAGTATATATATTGGGGGAGACTGGATTGGAAAATACAGTTGTTCTAGATCCC

AAACGAGTTAATTACGGTTCACAGGGTGGTAGAGTTATAATAGATGTGTCGGCAGACGGT

ACCCCACGCAGTGCAAAGATAGTGTCCACTGTTTCTGATGACTACCAAAAACTAAAGTAC

TGTATCTCTCTTGAAATCATTCGAATCACATTATCTGTAAACAAGGTGAAACAGTCTACA

CAGATAGAACTTGAAACAGCCAGATCTATCTATCAGGAATATGTGGAAGAAAATAGGCCA

ATGACAAAGGTGGCATTATTTGATATGCAAAATACTAAATTTGTGAAACGCTTAGGGGGC

GTCAAAGAGAATGCAGCTTGTTCTCTTTTCAGTGCTACTGACATTACAATGAGGTGGGAG

CCTGATGTTCATCTATCACTAATTGAACTTGTTCTCCAGATGAAACTAATTGTACACAAT

AAGAAGCTTGAGGAGTGTGGTAATGAACATGTGGAAGATGCATCTAATGTAAGAAATACC

AGTTCAAAAAATGAAGCTACCACGGAATCACGGAATCTTGAAAAGAAGAAAGGTTCTATT

TTTGCTGTTGATGTTGAAATGTTGAATATATCTGCTGGGCTAGGAGATGGAGTTGAAGCT

ATGGTTCAAGTGCAGTCAATTTTCTCTGAGAATGCTAGTATAGGAGTGCTATTTGAAGGA

CTAATGATCAATTTTAATGGGGCCAGAATCTTGAAGAGTAGTAGGATGCAAATTTCACGA

ATTCCTAGCATATCTGCTAGTGCATCTGATGCAAAGGGACCTGCAGCCACAACATGGGAC

TGGGTAATTCAAGGTCTCTATGTTCACATTTGTTTGCCATACAGATTGGAATTGCGTGCT

ATTGATGATGCTCTTGAGGATATGTTGCGAGCGTTAAAGCTTATTGTGGCAGCAAAAACT

AATTTGATTTTTCCTGTGAAAAAGGACAGTTCTAAGGTCAAAAAACCTAGTTCATCAAAA

TTTGGATGCATAAAGTTTTTCTTACGCAAGTTAACTGCCGACATTGAAGAGGAACCAATC

CAAGGATGGCTTGATGAACACTACAAGATGTTGAAGAAGGAAGCTGGTGAATTAGTTGTC

CGGTTAAACTTTCTAGATGAATTTATATCGAAGGCCAAGCAAGATCCAAAAACTTCTGAT

GACTTAAATAATTCTTCTGAAGAGGGAAAAATTTATTTTAATGATGTTGAGGTTGATGTA

AATAATCCCTCAATTATTGAATCCATGCGAGAAGAAATTTATAAACGGTCATTCCGTTCA

TATTACGAGGCATGCCAGAAGATAGTGTTTTCTGAAGGCTCAGGTGCTTGTAAGGATGGC

TTTCAAGCTGGTTTCAAACCTAGTGCTTCAAGGTCTTCTCTTTTTTCGATATCTGTATCA

GACTTGGATCTAAGCTTGACAAAAATTGATGGTGGAGATGCTGGGATGATTGAGTTTCTG

AGGAAACTTGATCCTGTTTGTCTTGAATGTGATATACCATTTTCCCGATTGTATGGGGCA

AATATTCTCTTAAATATGAGTTCTCTTGTGGTTCAGCTTCGAAATTATACATTTCCTCTT

TTTGCTGGAAGTTCCGGCAAATGTAAAGGCTGTCTTGTGTTGGCTCAGCAGGCAACTAGC

TTTCAACCTCAAATACTTCAAGATGTCTATGTTGGGCAATGGAGAAAGGTGTGCATGCTT

CGATCAGCTACTGGTACGACACCACCAATGAAGACATTCTTAGATTTACCAATACATTTT

CAGAGAGGCGAAGTGTCATTTGGGGTGGGTTATGAGCCAGTTTTAGCGGATCTTAGCTAT

GCTTTCACCGTGGTGATGCGGAGGGCTAATCTAAGTATTAGGAATCCTGGCCCACTTATT

CTGCCACCGAAAAAGGAGAAGAGTTTGCCATGGTGGGATGATATGAGAAATTACATTCAT

GGAAGAACTTCCTTATTATTTTCTGAAACTAGATGGAATATTTTGGCTACTTCAGATCCA

TATGAAAATCTTGATAAACTTCAAATTGTGACTAGCTCTATGGAAATTCACCAGTCAGAT

GGTCGTGTTTTACTTTCTTCCAAAGATTTCAAGATTTTTTTGAGTAGTTTGGAAAGTTTG

GCAAACAAACGTGGTTCTAAAATTCCAGCTGGCGTCTCTGGTTCATTTTTGGAAGCCCCA

GTTTTTACAGTTGAAGTTACAATGGACTGGGATTGTGACTCTGGAAAACCCTTGAACCAT

TATTTATTTGCACTTCCAATTGAAGGGAAAGCTCGTGAAATAGTATTTGATCCCTTCAGA

TCCACTTCTCTTTCCCTTCGATGGAACATATCTTTTGGATCCGGTCTTCCTTTGTCAGAA

AAAAAACATCCATCCTCCACAGCAAGAGATAGTATAGAAGGAGATGTTAATGTACCTCAT

CCTCTTAAAATTTGTCAGAATGATTCACCTGCTTCTCCAACATTGAAATTCGGTGCTTAT

GATCTAGCATGGATTCTAAGATTCTGGAACTTGAACTTTCTTCCTCCACATAAACTGCGC

TCCTTCTCACGATGGCCTCGTTTTGGAGTTCCAAGAATTGTCAGATCAGGCAATCTTTCA

CTTGATAGAGTGATGACCGAATTTATGTTGCGTCTTGATTGCACACCAATCTGTATTAAG

AACATGCCTTTACATGATGATGATCCAGCTAAAGGACTGACCTTAATGATGACAAAGTTG

AAGCTTGAACTATGTTTTAGTAGGGGCAGCCAACATTATACTTTTGAAAGCAAACGTGAC

CTTCTTGATCTTGTTTACCAAGGTATAGACCTTTATATGCCCAAGGGTTTCCTAATTAAA

GAAGAGTACGGAAATGTTGCTAAATCAATTAATGTGATGCCAAAAAACTCACAATCTGCA

TCCGAGGAGAAAACTTTCTCTGAAAAGGGTTATTTTACACATAAAAATAACGATGATGGA

TTTCTGTTTTCTTGCGATTACTTTACCATACGAAAACAGTCTCCAAAGGCTGATCCTGAT

CGGTTATTAGCCTGGCATGAAGCCGGAAGAAGAAATCTTGAGAAGACGAATGTACAGTCT

AACTGTGAAAAAGAGAGTGAAACTGATGAGCATATGGAACCAGACCCAAGCGGTGATGAT

GGGTACAATGTGGTGATAGAGGATGATGGGTACAATGTGGTAATAGCTGACAGTTGTCAG

CGTGTGTTTGTCTATGGTCTAAAGCTTTTGTGGACTATTGAAAACAGAAATGCAATTTGT

TTCTGGGTTGGCGGTCTATCCAAAGCTTTTGCACCTGCCAAGCCTTCTCCTTCCCGGCAG

TATGCACAGAGAAAATTATATGAGAACAACAAGAAGCAAGATGGAACTGAAACTAGTCAA

GATGAGGCATGTGAAACCCATCAAGATGATGAAACTGAAACGCATAGAGATGATGGAGCT

GAAACCCATAAAGATGAAGGAGTTGAAACCCATCAAGATGAAGTTAGTAAGTTCCTTCCT

ACTGGTGACATCTCAGATTCCCCCTCTTCTCTGGCTGCCAGTACCTCAGAGATCCCTTCA

TTCCCATCACATTCACTTAAGCTGGACAGCTTACCATCTGCTAAATATGAAAGTACAGAC

GATTCGAAGGAGGGGACTCGACACTTCATGGTTAATGTTATTGAACCACAATTTAATCTT

CACTCAGAGGGCGCAAATGGTAGGTTTTTGCTTGCTGCTGTAAGTGGACGCGTTTTAGCC

CAATCGTTTCATTCAGTTCTTCGTGTTGGCCATGACATGATTGAGCAAGCACTTGGTACA

AAAGATGAAAATACTAGTCAATACCAGCCCGAAATTGCATGGAAAAGAATGGAAATATCT

GTTATGTTGGAGCATGTGCAGGCACATGTAGCACCAACAGATGTTGATCTAGGGGCTGGG

GTTCAGTGGCTACCAAAAATTATTAGAGGCTCTCCAAAAGTAATGCGTACAGGTGCACTT

CTTGAGAGAGTTTTTATGCCCTGTGACATGTACTTTCAGTTCACGAGGCACAAAGGGGGC

ACTCCAGAAGTGAAGGTGAAGCCCCTGAAGGAGCTCAAGTTCAATTCTCATAATATTATA

GCAACAATGACATCTCGCCAGTTTCAAGTCATGTTGGATGTGTTAAACAATCTTCTATTT

GCACGGCTTCCAAAGCCTCCAAAAAGTAGCCTGACACTTTCTGTTGAAGATGATGAAAAT

GTCGAAGAGGAAGCCGATGAGGTGGTTCCTGATGGTGTTGAAGAGGTGGAACTTGCAAAA

ATTAACCTCGAAAAGAAAGAGAGGGAACAAAATTTGCTTCTTGATGATATGAAAAAGTTG

TCTTTTTGGTGTGACACTTCCAACAGAAAGGAACCAGAAAAGGAACGAGACTTGTGGATG

ATATATGGTGGAATAGCCATGCTGGTCCAAGGACTGAAGAGAGAACTTGTAAGTGCACAG

AAATCCAGGAAGGAAGCATATTCCGAGTTAAGAACGGCTATGCAAAAAGCTGCACAGGTG

CGGCTAATGGAGAAGGAGAAAAACAAAAGTCCTTCCTATGCTATGCGCGTATTTTTGCAA

ATTAACAAAGTTGTTTGGAGCATGATTCTTGATGGTAAATCCTTTGCTGAAGCCGAAATC

AATGACATGATCTATGACTTCGACCGGGATTATAAGGATGTGGGTATTTCTCAGTTTACA

ACAAAATATATTGTTTTCAAAAACTGTCTTCCCAATGCCAAATCCGATACAATTGTTTCA

GCGTGGAAGCCTCCAGATGAATGGGGAAATAAAGTCATGCTTCAAGTTGATGCGAGACAG

GGAGCTCCAAAGGATGGAAGTTCTCCCTTTGAACTTTTCCAAGTGGAGATATATCCACTT

AAGATCCATTTAACAGAGACAATGTACAGAATGATGTGGGTATATTTCTTCCCAGAGGAA

GAACGAGATTCACAACGCCGGCAGGAAGTTTGGAAGGTCTCAACCACAGGTGGTGCAAGG

CGTGTAAAGAAAGGTTCATCAGCCCATGAAGCTTCCACTTCAAGTAATCAGTCGACAAAA

GAGTCTGAGGCCTCATCCAAATCTGGCATTTCTGCATTGCTTTTTCCTGCAACCAATCAG

CCTTCTGGTTCTGCACAAACTTCCAAAGCACAAAATGTCAAAGTAGTTCCTGGTGCTGGT

TCAACCCCCGAATGTGAAGTGACTGTGGCAGAATCTGTAGCTGATGAAAATGGCCCATTT

GGCTCAATTGAGAAGAAAGATGAAGTGTCTAAGAATAAATCAAAAGATGTGAAAGGTGTA

AAAGCTGGTCAATCATCTCTTGAAGAAAAGAAGGTGGCTAAGCCACAAGACGATAAGAGA

TCTAGGCCAAAAAAGATAATGGAATTTCACAACATCAAAATTAGTCAGGTGGAATTGTGT

ATTACGTATGAAGGGCAAAGGTTTGTTGTAAACGATATGAAATTATTGATGGATCAATTT

GTCCGGGCCGAGTTTACCGGGACCTGGCAAAGACTCTTCTCACGAGTTAAGAAGCACATC

ATTTGGGGAGTCCTAAAGTCTGTGACTGGCATGCAGGGGAAGAAATTTAAAGACAAAGTT

CAGAATCAGCCAAGTGGAGCTGGTGCTCCGGAAGGTGAAGCTAATTTTAGTGACACTGAA

GGCCATGCTGGAAAACCTGATAAGTTTCCACCATCTTGGCCTAAGCGTCCTACTGATGGA

GCAGGCGATGGATTTGTAACATCCGTTAGAGGAATATTTAACTCTCAACGCCGTAAGGCA

AAGAAATTGCTTAAGCCAAAGAAAGATGAAGCAGAAAATGAAGGTCATGGAGATTTGAGT

GAGAATGAGGTAGAGACCACTCATTTTGCTAGGCAGCTCACAATAATAAAAGCTAAAAAG

CTTTTTAAGCGGGACCCTAAGAAGCCCCCCCACTCTAAAGGACAAAAAGGTTCATCCTCA

ATACAAAACGAAGAACTACCATCATCTCCAAAAGAGGCAATTGCATATGACAGTGATTCA

TCAAGTGGATCCTCATCCTATGAACTTATTCTTGAATAG

>MS.gene049953.t1

ATGGTGGAGAAGTTCCTGACTGTGCCACCGTTTGAGTGCGCGTGGCGAGAGGATTTAAAA

TTCCAGGAAGCAGGTAGGGGATGCGTGGCTTTCGATGCATTTGCCTGCAACGATGTCACA

TTGGTTTTTAGGGAGAATGTTGGAAGCCAAGGCTACCACTACAAAAGAGATAGTAGTCCA

CACTACACAATCATATTGGGGAGTCATAGGAATCGGCGTCTCAAAATTGAGGTCAACGGT

GAAACTGTTGTTGACGTGGTAGGGGTTGGACTCTGCTGTTCCAATTCGTTTCAGAGTTAT

TGGATCAGTATCTACGATGGCTTCATCAGCATTGGTAATGGGAAATACCCTTTTCAGGAT

GTTGTCTTCCAGTGGTCTGATTCTCGTCCCAATCGTAATGTTCAGTACATTGGCCTCAGT

AGTTGGGATAAACATGTTAAGTACAGAAACGTTAATGTCCTCTCATTAAAACACACTCTT

ATGCCCTTATCTAAGCATATGCTTTTTGGTGATTATCAAGTTGAGGATGATGTTACTGCT

GCTGACAAACAGTTGCATATGGACTATGACAAATGGGGTCTCGACAATTTTCTGGAGAGC

TGGGACTTGTCTGATATGTTATTCATCATTGGTCCTGAGGAAAGACCTGTTCCTGCTCAC

AAGCCTATTTTAGCTGCTTCTGGAAACTTCCCTTTATGTTCCTCCTTTGCCATCACACTA

CCCACTGTTTCTTATCCGCTTTTCCGTGCACTACTTCACTACATCTACACTGGCTGGACA

CAGATTCCACAAGAACACCTTGATTCTTTGAGGGCTTTAAGTCTACAGTTTCAAGTGATG

CCACTGGTGAAGCAATGTGAAGAGGTTATGGAACGAATTAAGGTAGATAACAAGTTGTTT

GACACCGGAAAGAATGTGGAGTTAACATATCCATGTATTGGGCCTCATTGTTCAACGTTA

CCCTCACTTCCTGTCAGCATTCAGAGACTGGTTCAATTAAAACTGTCGGGCCAGTACAGC

GATGTGAACATCTACATTGAGAGTTATGGATTTGTTGCACGAGTACATAGAATTGTTCTC

AGCTTATGGAGTATCCCCTTTGCCAAGATGTTTACAAATGGAATGAGTGAAAGCATGTCA

TCAGAGGTTACTTTACGGGATGTGCCGCCAGAAGCTTTCAAGGCTATGATTGACTTTTTA

TATGGTGGACAATTGAATGAGAAAATTATTGATTCTGGTTCTTTGTTACTCCAACTTCTT

CTATTGGCTGATCGATTCGGAGTGACGTTTCTTCACCAAGAATGCTGCAAAATGCTTTTA

GAATGCCTCTCACAGGACTCTGTATGTCCACTCCTCCATGTGGTTTCTTCAATCCCATCA

TGCAGACTTCTTAAAGAAACATTACAGAAGAGAATTGCCATAAACTTTGACTATTTTATC

AGTGCCAGTACTGACTTTGTTTTGTTAGATGATACAACTTTCTTCAATATCATTAAGCAT

CCAGATCTGACGGTAACATCTGAAGAGAAAGTTCTTAATGCAATCCTAATGTTTGGCATG

AATGCAAATGAGTTATTTGGATGGGAAGTGGTGGATCAGTTAAATTCAAAACCTGAACTC

CTTTTTGGGGAGAGGCTTCAATTAGTCTATGACTTGCTGTCATTTGTGCGATTTCCACTG

CTACAACACTCCTTACTTGACAAGTTGCAGAATAGCAACATTGTCAGGCATATTCCTGTT

CTACAAAGTCTCGTTCAGGAGGCAATAAATTTTGCTAAACATGGGCTGGGAAGGCCAGAA

AATGAAAATAATGTTAGATTCCAACATAGACGGTCTAGTTACAAGGAGCTCCAGTATATA

TGTGATGGGGATGACCATGGAGTTCTTTACTTTGCAGGCACATCATATGGTGAACACCAG

TGGGTTAATCCTCTTTTGGCTGAGGCAAAGAAAATTACCATCACAGCCAGCAGTCCCCAC

TCAAGATACACTGATCCCAAGGTTTTGGTCTCAAGAACATATCAGGGAACATGTTTCGCT

GGACCTCGTTTGGAAAATGGACATAACTGTTCCTGGTGGATGGTTGATCTTGGACAAGAT

CATCAGCTTATGTGCAACTACTATACCATGAGGCAGGATGGCTCCAAGGCCTTCCCGAGA

TGTTGGAATATTCAGGGATCAGCGGACGGAAAGAGCTGGCGGGACTTGAGGGTCCATGAA

AACGACAGGACAGTATGCAAACCTGGTCAATTTGCATCTTGGCCCGTAGTTGGTCCCAAT

GCCCTGCTTCCCTTTAGGTATTTCCGGGTTGTTCTCACAGGACCGACCATTGACGCTACT

AATCCTTGGAACTTCTGTATTTGCTACTTGGAACTTTATGGCTACTTCCTCTAA

>MS.gene049949.t1

ATGGATTCTTTGTTGGATCAAGCTACTAGTACATGTTCTCACTGTGGTCGAGCTATTCCT

GTTGCGAATGTTGACTTGCATTATGCTCATTGCTCAAGGAAGCTTCAAAAATGCAAAGTT

TGTGGTGATATGGTTCCTAGAAAAAATGCTGAGGATCACTATTTAACCACTCATGCGCCA

GTTTCCTGTTCATTGTGCAGTGAGACAGTGGATCGTGATATCATAGATATCCATACAGGT

GAAAACTGCCCTAAAAGGATTGTCACTTGTGACTTCTGTGAGTTCCCATTGCCAGCAATT

GATCTAGCTGAGCATCAGTATGCGGGAATCGAACAGAACTCTGTCAACTTTGTAACAAAT

ATGTTAGACTTCGTGAAAGATACAATCATGAAGCCAGGTGCAATGGCATTCAAGATAGCA

CTGTGGGCTCTTCAAGGTAAGAAATATCACTATTCATGTGAATAA

>MS.gene049957.t1

ATGATGCCGGGAGAGTTACTGAATCACTCGGGAACCCGCAAGATTGAGTGTTTAGAGGAG

ATAGGTGATGGGGTTTGGGAGTTGGATAGGGCATCCCAAACTCTGACTTTCACAGGGTTG

GTTAGGACAACCGAAGGAATAAGAGGTTTAGTGGTGTCATCTGTCATTCATCATGTTACC

TTGGAGGTAGTGTTACCATTAGAGAGGATGATTAGTACATTAGGTCTTGATAGAGCTAGG

GAGAATTCGACCTCTGAAAGGGCTAGAGGACCAAGGCCTCGTTTTCGTCGTACCGCTCGC

ATTAGTGTGCGTCCCCCCGTTGTTTATCATTTTGAGAATGATCCCGAGGAGGATGGAGCA

GACATGACCGTGACTGATGTGGAGGTGAGTGGACGTGCTGGAGATGAAGTGGATCCCTCT

GAAGACCTTGGTGTTGGTGGTGCTAATGAAGATCTCCCGAATGGTGTTGTAAAATATCTA

ACTAACCCTGACAAGCCTGAGTATGCTGAAAGGGATATTCGAAAGCAACAAAGGGAAGAA

AGAAATGCTGAAAATAAAGGATTGGTAGATTTCAGACGTCACGATCCTCCACAATTTCTT

GGAGAAACCAAACCAGAGAAAGCTGACTTATGGCTCCAAGAGATAGAAAAGATATTCGCT

GTGTTGAGGTGTCCTGACGAAGTAAAGGTAACATATGCATCTTACTTACTATTGGGAGAT

GCCGAATACTGGTGGAAAGGAACTCGCAAGATGATTGAAGCTAACAATCAAGATATTACT

TGGGAAATATTTCGTACGAAATTTCTCGACAAGTATTTTCCTAGAAGTGCTAGGACTGCA

AAGGAACAAGAATTTCTGACCCTGAAACAAGGAGGAATGACGATTGGAGAATATGCAGCA

ACGTTTGAGTCGTTGGCAAAATATTTTCGTTTCTTTCAAGACCAGGTGGATGAAGATTGG

CTATGTGAACGATTTGAAGGTGGACTGAAGCATAGCATCAAAGAATCTGTCTTGCCACTA

GAAATTCGTCAATTCCAACCACTTGTATAA

>MS.gene049954.t1

ATGTCTGATTCCATGTCTGATAGTGAGTCCCAGCATTCACCCAAACATTCTTCACCCAAA

CCTGAATCACCCCAACCTGTCAGAACATACCATGAAAATCACCATGTTCAGATCACAACA

ATTCGGCTGAACGACAAGAAACAACCAGACAAGGCAGATGCAGATTATGATACATGGGAT

GCTGAAAATTCCATGATCATGACATGGTTAGTCAATTCTATGACTGAAGAGATAGGTGTG

AACTATCTCTGTTATGATACCGCAAAAGATCTTTGGGAGAGTGTCTCTCAAATGTACTCT

GATTTGGGAAACCAGTCCCAAATTTACGAATTAACTCTTCAGCTTGGAGAAATTCAGCAA

CCTAAAGATTCAGTCACCAAATACTTCAATTGTCTCAAACGTATTTGGCAAGATCTGGAT

CTCTTCAATGAATATGAGTGGAAGTCACCTGAGGACTGCAAGCACAACAAGAAGATGGTG

GATGTTAGCCGTGTCTTCAAATTTCTTGCAGGTTTAAATGTTGAGTTTGATGAGGTTCGT

GGCCGAATCCTTGGTAGGAATCTTATCCCTTCAATTGGTGACGTCTTTGCGGAAGTGCGT

CGCGAAGAGAGTCGTAGGCAAGTGATGCTCGGAAAAAAGATAGTCGTTGTCCCGCCACTG

GTTGAAGGATTTGCCCTGGCCGTTCCTCAAGTCAATCGCAAATCTTTCCCTAATCCACGA

GGTGGTGACAAGACCCATTTAGTTTGCGACTACTATGGCCGCAATCGTCACACTCGAGAA

ACTTGTTTCAAGCTACATGGTAGGCCAAACAATAGCAAGGCTGGTAAGTTTGGTGACCGA

CCTATGCCTACAACCAACGATGTTGCTTCATCTCCATTTACTAAGAAGCAGATGGATCAC

CTTCTTAAGCTTCTAAGGTTCAATTCATCCCCTAATACTCTTGTTGGTATTATGGCACAA

ACATGTAAGGATTCTTGGGCACTATCTGTCCAAAACCATTCCAACCCCTGGATAATAGAT

TCAGGGGCATCTGAATACATGACTAATTACTCTCATCTATTCAATTCATATTTTCCTAGT

TCAGGTTCTGAACAAGTTAAGATAGCTGATGGGGGAGAAGAGGGAGAAACATTGATTAAG

AATCCTGAACTTAAAGTGTATGTTCGGAAAAAATTTCACAAAGATGGTACAGACCCCCTT

GTCTTTCTAGTAGAAGTTCAATCGGATTCTCCAAGTGAAGGTCCTACTGATAATCCATCT

TCCAGTTCCTCTGGTAATTCTTCTCACTCATCCAATGATTTACCTGATTTGTCTTTTCCT

GATATTAATCTTCCAATTGCAGTGAGAAAAAATATTCCAGACCTCAATATCCCTACTGCT

GAAAGAAAATGTGCCCGCGAAGTTGAAGTTGTCTGTACCAAACCACCGATCAAGTCTCCT

CCTTCGATGACCTCCATTTCTGACGACTCACCACAACGACATCATTTCCTTGCAGTGGCT

ACAAGTTTGACCTTCATCTCCAAGATGGCCCCTCCTGTCACCGCACTGTTGTGA

>MS.gene049946.t1

ATGCGAGGCCAGCCCTCAGCTGAACTTACCTTTATTCCAGAAGTTGAAAAGTACTGCCGA

GCAATCCGGAAAGAAACAAAACTCAGAAAACTTCGAGAAAAGGAAGGGACTAGCGGGGTA

GAGGTTGACACGGACGAGCAGTTAGACGAAGAGATGGCGGAGGGACAACAGCCACCACCG

CCACCACCTGCAGAAAGACTTCTCGGTGACTATGGGGCTCGTGACAGAAACCGAAACAGG

TTGACCATTACTAATCAGCCTGTGACGGTGAACAAGTTTGAAATTAATCCCGGTCTATTG

CGGGAGTTGAAGGAGAATCAGTATGCGGGGAAGTACAATGAAGATGCCAACAAGCACTTG

AAGAGTTTCTTGATACTTTGTGAGACAGCTAAGGTACAAGGGCACTCTGAGGAAGCAAAA

AGATTGAGACTGTTCCCGTTCACTTTAACTGATGATGCATATGAGTGGTTTGATTCCTTA

CCGGCTGGTAGCATAACAACTTGGAACGAGATGGAAGACAAGTTCTTGGAGCAATTCTTT

CCTACAGCTCTATTTGTGAGGAGGCGACAAGACATTTTCAGCTTCCAACAGAAAGAGGGA

GAGTCTTTGGGAGAAGCGTATAAAAGGTACAAGAAGCTACTCTCATCATGTCCCGAGCAC

AACTTTGATACTACAGTTCAAATGCAGATTTTCTGTAATGGTCTACGTCTGGCTACTAGA

CAAGTCCTAGACACAGCTTCCGGTGGTTCCATAAATTTCAAAACCGCCTCTCAAATAATC

AAAATCATTGAAGCTGTTGCACTTAATGAACAAATGGAGATGTATGATAGAACTGGTGGC

ACAAGGGGAGGACTCATAGATCTGAATCAACTTGAGTTCAAAAATGCTCAAAACATATTA

ACTGGTAAGCAGATTCAAGATGCAGTAGCTGCTGAAGTGTCAAAGAAAATGGCAGCACTT

AACTTAACTCAACCCCAAGTTGCTCCGGTGAATCAAATGAACACTGTCAAATGTGATTGG

TGTGCTGGCCCTCATTTCACTATGCATTGTGATGTGCCTATGGATGCAACCCAAGTGGAG

ATGGTGAACTACCTCAGCAGTCAAGGGAACCAGGTTAGAAATAACCCTTTTGCAAATACA

TATAATCCAGGCTGGAGGAACCATCCAAATTTCTCATGGAAAAATCAACAAGGTGGTTCT

CAAAATCAACAACAAGGGGGTTATCAAGGAGGTCCATCAAACCAAGCTCCAAAGAAGGCT

GATTGGGAGTTAGCTATAGAAGCAATGGCAACCAACATGAATTCCTTAGCTCAAGAAACC

AGAGAGGCTCAAAAGAATACAAGGGCTTCAATCAAAAATCTTGAAACTCAAGTTGGTCAA

ATTGCTCAACAACTCTCACAACGAGCTCCAGGAACCCTTCCAAGCAACACTGTCGTCAAT

CCAAGGGATCATGAAAATGTGAACATGGTTACCACAAGAAGCATGAAGAGGGATGAATCA

CTATCATCTCAAGCTAAACATGATGAAGGTGACTCATCTCCTGAACTAATTGAAGTGGAG

GTTGAAGTGCGTGATGTTGTTGATACTCAGAGGTTGCTGAAGAAAGTGAAGACCCCCATG

AATGTGAAGTTCTAG

>MS.gene049951.t1

ATGGGTTCTTCATCGACAACACCTTTGCCAAATCACAACAAAACTTCTTTTGGTACTCGT

TTGAAGATCAGTTGCTTATCTTTTGCCGTTTCTATACAAGAATCTTTTAGATATGCAAAA

GCCTTTTTTGTCGGTCAGGCAAAGACTATAACTGCAAAAAATGAGAAGGAAGCAAGTGAA

GCTGAACTTGAGGCTTCAAAGAAACAAGTTGAGGCTGCTGATGCTGCTGAACATATCAAG

AATAGGCTCAATGATCCTCATAGTTAA

>MS.gene049948.t1

ATGTATCGTAAAATGAGTGTGAAGCATTTGATAATGAAGATCACTTTGCATAAGTCTGAG

TTTATGGGAATCTCATTCATGCTAAAGAGTTGCCCCAACTTAGAACGTCTCACAATTGAA

ATTGTTGATGAAATACATTTGTTGGATTATGATCCAATATACAATGTTATCCCAGAGAGA

TACGGGAAGGACATTGCAAATGATTATAAATGTGTGAAGTTTAATCTAAAAGAGATGGAG

ATTAATGGCTTTAAGGGAACTAAAAATGAGCTTAATGTGATTAAGTACTTCCTCATTCAT

GGGAAGGCCTTGAGAAAGGTGAGTATCAATTTACTTAAAGATGATGTGGAGCGTGAGGAT

GGACTGAATGTCGCTGTCCGTCGTGAAGCAGAGGAACTTTTGTTGAATCTTCCAAAAGCT

TCCACTAATTTGAAGATTTCAATCTATTAG
